# Sodium channels Na_v_1.7, Na_v_1.8 and pain; two distinct mechanisms for Na_v_1.7 null analgesia

**DOI:** 10.1016/j.ynpai.2024.100168

**Published:** 2024-10-11

**Authors:** Federico Iseppon, Alexandros H. Kanellopoulos, Naxi Tian, Jun Zhou, Gozde Caan, Riccardo Chiozzi, Konstantinos Thalassinos, Cankut Çubuk, Myles J. Lewis, James J. Cox, Jing Zhao, Christopher G. Woods, John N. Wood

**Affiliations:** a Molecular Nociception Group, Wolfson Institute for Biomedical Research, UCL, Gower Street, London WC1E 6BT, UK; b Institute of Structural and Molecular Biology, Division of Biosciences, University College London, London WC1E 6BT, UK; c Cambridge Institute for Medical Research, Keith Peters Building, Biomedical Campus, Hills Rd, Cambridge CB2 0XY, UK; d University College London Mass Spectrometry Science Technology Platform, Division of Biosciences, University College London, London, UK; e Centre for Experimental Medicine and Rheumatology, William Harvey Research Institute, Barts and The London School of Medicine and Dentistry, Queen Mary University of London, London EC1M 6BQ, UK

**Keywords:** Pain, Sodium channels, Nav1.7, Nav1.8, Side effects, Genetic deletion, Drugs

## Abstract

Genetic deletion and pharmacological inhibition are distinct approaches to unravelling pain mechanisms, identifying targets and developing new analgesics. Both approaches have been applied to the voltage-gated sodium channels Na_v_1.7 and Na_v_1.8. Genetic deletion of Na_v_1.8 in mice leads to a loss of pain and antagonists are effective analgesics. The situation with Nav1.7 is more complex. Complete embryonic loss of Na_v_1.7 in humans or in mouse sensory neurons leads to anosmia as well as profound analgesia as a result of diminished neurotransmitter release. This is mediated by enhanced endogenous opioid signaling in humans and mice. In contrast, anosmia is opioid-independent. Sensory neuron excitability and autonomic function appear to be normal.

Adult deletion of Na_v_1.7 in sensory neurons also leads to analgesia, but through diminished sensory and autonomic neuron excitability. There is no opioid component of analgesia or anosmia as shown by a lack of effect of naloxone. Pharmacological inhibition of Na_v_1.7 in mice and humans leads both to analgesia and dramatic side-effects on the autonomic nervous system with no therapeutic window. These data demonstrate that specific Na_v_1.7 channel blockers will fail as analgesic drugs. The viability of embryonic null mutants suggests that there are compensatory changes to replace the lost Na_v_1.7 channel. Here we show that sensory neuron sodium channels Na_v_1.1, Na_v_1.2 and β4 subunits detected by Mass Spectrometry are upregulated in Na_v_1.7 embryonic null neurons and, together with other proteome changes, potentially compensate for the loss of Na_v_1.7. Interestingly, many of the upregulated proteins are known to interact with Nav1.7.

## Highlights

• Distinct mechanisms of analgesia occur in embryonic and adult SCN9A Nav1.7 null mutants.
• Embryonic null mutants lose nociceptor neurotransmitter release through an endogenous opioid regulatory system.
• Adult gene deletion results in loss of excitability with no role for the opioid system.
• Embryonic null mice are viable because of compensatory expression of Nav1.1 and Nav1.2 in sensory neurons.
• Nav1.7 targeted drugs act on both pain and the autonomic nervous system to produce unacceptable side effects.

## Introduction

The peripheral nervous system is the driver of conscious pain sensing in the brain (Emery and Wood, 2019). Inflammatory mediators and interactions with the immune system control pain thresholds and the gain of activity of damage-sensing neurons (Coaccioli et al., 2024). These neurons release glutamate and neuropeptides within the dorsal horn of the spinal cord in response to tissue damage (Mcmahon et al., 2014). Importantly, all analgesic drugs in clinical use also work well in mice, supporting this animal as a useful model system to explore pain mechanisms (Wood, 2021). The easy availability of DNA sequencing has allowed us to identify human pain genes (Lischka et al., 2022). In principle, mechanistic studies of mouse models should provide us with a direct route to analgesic drugs, desperately needed in these days of the opioid overdose crisis.

Unfortunately, there is remarkable redundancy in signal transduction in the peripheral pain system. For example, there are at least four major heat sensors in mice and humans (Vandewauw et al., 2018). Thus attacking nociceptor transduction is generally unappealing for analgesic drug development. Moving away from transduction, the ion channels involved in electrical transmission are less redundant and the sodium channels Na_v_1.7 and Na_v_1.8 have been unambiguously linked to human pain and damage sensing through detection of functional variants of the *SCN9A* and *SCN10A* genes (Lischka et al., 2022). The encoded channels are the targets of the potent anesthetic and analgesic lidocaine. Its long lasting effects as an anasthetic and analgesic and superiority to procaine and cocaine were established in human trials in 1944 (Gordh, 2010). Low dose systemic lidocaine is an effective pain treatment, but high doses can cause death (Gordh, 2010). Therefore Na_v_1.7 and Na_v_ 1.8 selective blockers are of considerable interest, especially as sensory neuron Na_v_1.7 and Na_v_1.8 null mice are healthy but have diminished pain sensing (Lischka et al., 2022).

The tetrodotoxin-resistant sodium channel Na_v_ 1.8, cloned in 1996 (Akopian et al., 1996), is selectively expressed in sensory neurons and has been shown to play an important role particularly in inflammatory and mechanical pain (Akopian et al., 1999). Antagonists are potent analgesics in preclinical models of neuropathic and inflammatory pain (Ekberg et al., 2006). Gene therapy with antisense oligonucleotides (Khasar et al., 1998) produces analgesia, but these studies have been overtaken by FDA approved orally active antagonists that work in humans (Jones et al., 2023).

Many Na_v_1.8-focused drug development programs were halted after the discovery of a genetic link with cardiovascular problems and Brugada sudden death syndrome and Na_v_1.8 (Chambers et al., 2010). This issue has been resolved by Christoffels et al. who showed that a cryptic intronic promoter drives the production of a C-terminal fragment named SCN10A-*short* comprising the last 8 transmembrane segments of Na_v_1.8 in the heart (Man et al., 2021). There, this inactive protein promotes the activity of the heart channel Na_v_1.5, explaining why the loss of SCN10A-*short* can result in cardiac dysfunction and Brugada sudden death syndrome. The role of this short Nav1.8 protein may explain the absence of human bi-allelic loss of function Na_v_1.8 mutants with diminished pain. The loss of Na_v_1.8 is likely to lead to cardiovascular dysfunction during development that may be lethal. An orally active Na_v_1.8 antagonist, Suzetrigine, that is analgesic, has succeeded in phase 3 trials and has FDA fast track and breakthrough therapy approval (Jones et al., 2023).

A major reason for a change of focus from Na_v_1.8 to Na_v_1.7 was the remarkable demonstration that Na_v_1.7 was required for pain in both humans and mice (Lischka et al., 2022, Nassar et al., 2004, Cox et al., 2006). A genetic link with three human pain disorders was also found for dominant activating variants of this channel; a subset of severe Primary Erythermalgia cases, a similar but lesser phenotype in some cases of small fiber neuropathy, and the distinct phenotype Paroxysmal Extreme Pain disorder, (OMIM 133020 and 167400). Given the role of Na_v_1.7 in sympathetic neurons in heat pain in mice (Minett et al., 2012), it is possible that hyperactive Na_v_1.7 channels in sympathetic neurons play a significant role in the very rare cases of Primary Erythermalgia linked to Na_v_1.7 mutations (OMIM 133020). However, mouse models of gain-of-function human Na_v_1.7 mutants do not show an erythermalgia phenotype, whilst a human mutation linked to painful small fiber neuropathy does exhibit enhanced pain, interestingly in a sex dependent manner (Xue et al., 2022). The inactivation-defective Na_v_1.7 mutant sensory neurons characteristic of paroxysmal extreme pain disorder (PEPD) that are linked to mechanical pain are more plausibly linked to mechanosensitive sensory neuron dysfunction (Fertleman et al., 2006). These findings, taken together, make Na_v_1.7 the best-validated human pain target to have been discovered.

Embryonic deletion of Na_v_1.7 in sensory neurons leads to analgesia, but does not alter sensory neuron excitability in mice (MacDonald et al., 2021). As Na_v_1.7 plays an important role in sensory neuron action potential generation (Deng et al., 2023), this requires that compensatory effects rescue the excitability of sensory neurons in embryonic nulls. Na_v_1.7 is the principal human parasympathetic sodium channel, and plays an important role in sympathetic neurons as well as throughout the CNS (Branco et al., 2016, Kocmalova et al., 2017) and potentially in insulin release (Zhang et al., 2014). Therefore there must be compensatory mechanisms, perhaps involving upregulation of other channels in the embryonic nulls to rescue central, autonomic and sensory function. Importantly, if Na_v_1.7 is deleted in adult mice with tamoxifen-inducible Cre recombinase, analgesia is also obtained (Deng et al., 2023). However, in these experiments, in contrast to embryonic nulls, there is a dramatic loss of electrical excitability in sensory neurons and no apparent role detected for the opioid system. This difference with the findings in embryonic nulls (MacDonald et al., 2021) supports the view that there are two distinct types of analgesia associated with embryonic or adult loss of Nav1.7 expression. As Na_v_1.7 is the voltage-gated channel that responds first with action potentials to sensory neuron depolarization, the adult data make sense. The lack of therapeutic window and autonomic side effects of potent selective Nav1.7 channel blockers contrasts with the apparent normality of embryonic Nav1.7 null mice and humans (Regan et al., 2024). We therefore examined the hypothesis that expression of other ion channels could compensate for the loss of Nav1.7 in embryonic null sensory neurons. We also explored the potential utility of gene therapy in targeting Nav1.8 in sensory neurons.

## Results

### Nav1.8 as an analgesic target

We examined the potential for gene therapy in blocking Nav1.8 expression in transgenic mice. Early studies by Levine had shown that antisense oligonucleotide block of Nav1.8 expression could produce useful analgesia (Khasar et al., 1998). Our results, using AAV delivery of a dead Cas9 to diminish Nav1.8 transcription also results in some mechanical analgesia in recipient mice.

However, the recent development of potent highly selective Nav1.8 channel blockers which are orally active makes a gene therapy approach unappealing in terms of both cost and feasibility (Nichols 2024, Jones et al., 2023). The range of pain conditions that can be treated with Nav1.8 blockers that appear to have no serious side effects is vast based on rodent data (Ekberg et al., 2006). This finding, and the possibility of using other analgesic drugs in with Nav1.8 antagonists in combination therapies promises to advance and improve pain treatment.

### Nav1.7 as a drug target

Given the compelling human genetic data pinpointing Na_v_1.7 as a pain target, and the failure of Na_v_1.7 channel blockers we wanted to explore differences in mechanisms of analgesia after embryonic or adult deletion of Nav1.7. Although sensory neurons appear completely normal in embryonic mouse Na_v_1.7 nulls, they nonetheless show a dramatic loss of Substance P and glutamate release on depolarization (MacDonald et al., 2021). Interestingly synaptotagmins are physically associated with Na_v_1.7 (Kanellopoulos et al., 2018). This inhibition of neurotransmitter release can be partially reversed by naloxone, as can analgesia in both mice and humans, suggesting that the opioid-dependent block of neurotransmitter release (MacDonald et al., 2021) plays a major role in Na_v_1.7 embryonic null analgesia. Importantly, the anosmia associated with loss of Na_v_1.7 seems to only depend on diminished electrical activity rather than induction of the opioid system (Weiss et al., 2011, MacDonald et al., 2021), a quite different mechanism from that which occurs in embryonic null somatosensory neuron. The apparent normality of sensory neurons in embryonic null mutants contrasts with the loss of excitability found in adult gene deletion studies. We therefore examined if SCN9a gene deletion might alter expression of other ion channels at the level of transcription in sensory ganglia. We could find no evidence for this with microarray analysis(Table 1) Analysis of mRNA transcripts in Na_v_1.7 null sensory ganglia does not reveal enhanced transcription of other sodium channels (Minett et al., 2015). We also used a qPCR analysis of one relevant sodium channel Na_v_1.1 and found little change in mRNA on embryonic gene deletion of Na_v_1.7.

**Table 1:** Micro array analysis of sodium channel transcripts in embryonic Na_v_1.7 null sensory neurons Relative expression levels in DRG somata of mRNA encoding voltage-gated sodium channels. Na_v_1.7 mRNA is present in SCN9A null DRG as only a functional RNA region is deleted (Minett et al., 2015).

	Fold change		p-value
Na_v_1.1	−1.14		0.01
Na_v_1.2	–1.12		0.07
Na_v_1.3	–1.16		0.09
Na_v_1.5	−1.21		0.01
Na_v_1.7	–1.34		0.009
Na_v_1.8	–1.34		0.009
Na_v_1.9	–1.2		0.009
Nax	−1.09		0.001

We also checked the expression of Na_v_1.1 using qPCR. The SCN1A gene encoding Na_v_1.1 is adjacent to SCN9A that encodes Na_v_1.7. Thes experiments showed a small increase in Na_v_1.1 mRNA at a non-significant level rather than a small decrease, also not significant, found in microarray experiments (Fig. 2).

**Fig. 1:**
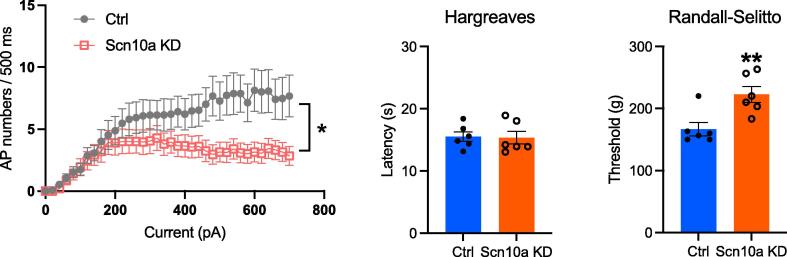
**Epigenetic inhibition of Nav1.8 expression results in mechanical analgesia***. AAV mediated delivery of targeted dead CMV-dSaCas9-ZIM3-pA results in lowered TTX-resistant sodium channel activity in sensory neurons (left panel) with normal heat sensing (Hargreaves test) and concomitant loss of noxious mechanosensation (Randall-Selitto test) in behavioral assays. N = 6 for behavioral assays n > 10 for electrophysiological studies. qPCR showed a 50 % drop in mRNA encoding Nav1.8 (data not shown).*

**Fig. 2:**
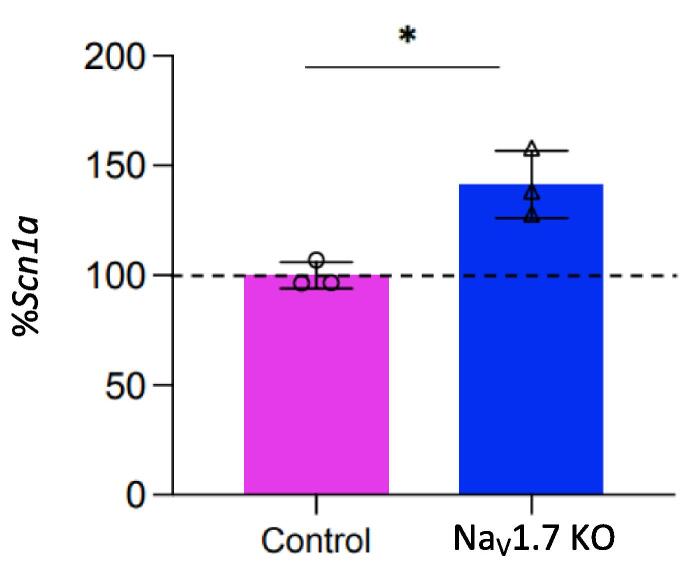
mRNA Analysis of Scn1a Expression in the DRG.

We next used mass spectrometry (MS) to interrogate changes in protein level in the sensory neurons that normally express Na_v_1.7. We isolated both DRG cell bodies and axons, as well as the dorsal horn of the spinal cord containing sensory neuron terminals from wild type and embryonic null Nav1.7 mice. We used 2 male and 2 female mice (n = 4) for control and embryonic null comparison. Out of more than 6000 proteins that we could identify, 85 % showed no significant change in expression in the Nav1.7 null mutant mouse compared with wild type animals. The complete data for both sensory neuron somata and terminals within the dorsal horn of the spinal cord are listed in Supplementary Tables 1 and 2. There were some interesting alterations in the expression of ion channels and proteins associated with neurotransmitter release. In particular, we found the voltage gated sodium channels Na_v_1.1and Na_v_1.2 as well as the β-4 subunit of sodium channels to be upregulated at the protein level in embryonic Na_v_1.7 nulls (Table 2, Supplementary Tables 1 and 2). Sodium channels exist as dimers, and mass spectrometry of sensory neuron epitope-tagged Na_v_1.7 shows that B_2_ and B_3_ subunits, as well as Na_v_1.1 may associate with Na_v_1.7 (Kannelopoulos et al., 2018). Na_v_1.1 (13 hits 6.7 % coverage on mass spectrometry of immunoprecipitates) is thus a candidate to replace Na_v_1.7 in peripheral neurons of embryonic null mice.

**Table 2:** **Proteome changes in sensory neurons of Nav1.7 embryonic null mice** The complete mass spectrometric analysis is presented in Supplementary Tables 1 and 2*.*

DRG somata and axons proteome (n = 4)			
		Fold change	p value
**Scn2a**	Sodium channel Nav1.2	1.7	0.35
**Scn2a**	Sodium channel Nav1.2 (males)	2.84	0.05
**HCN2**	K/Na hyperpolarisation-activated channel	1.52	0.1
**TRPV1**	Transient receptor Potential channel V1	1.30	0.1
**KCNA2**	Kv1.2 Potassium channel	1.23	0.08
**SYTL2**	Synaptotagmin-like protein 2	1.3	0.05
**Syt1**	Synaptotagmin 1	1.18	0.03
***KCND1*** Potassium channel Kv4.1		**0.6**	**0.03**

Dorsal horn axons and terminals proteome			

		Fold change	p value

**SCN1A**	Sodium channel Nav1.1	3.06	0.09
**SCN4b**	Sodium channel β 4 subunit	1.43	0.04
**TRPV1**	Transient Receptor Potential channel V1	1.35	0.0006
**Snap2**3	Synaptosome associated protein 23	1.17	0.04
**Tac 3**	Tachykinin 3	**0.**58	0.0008

The MS data provide plausible candidates for compensation in terms of sensory neuron excitability. The complete data are presented in Supplementary Figs. 1 and 2 for DRG and spinal cord dorsal horn tissue respectively. There are some notable changes in pain related proteins that are summarized in Table 2. For example, TAC3 (tachykinin precursor 3 protein) was almost halved in the dorsal horn terminals of Na_v_1.7 nulls, whilst TRPV1 protein expression was upregulated in sensory neurons. TAC3 gives rise to the peptide Neuromedin K. The values in Supplementary Tables 1 and 2 are in some case complicated by differences in expression between males and females. We used n = 4 for the experiments, but if we focus on the induction of Nav1.2, this is 2.8 fold in the two males with a small p value, but adding the female data produces a combined value for induction of 1.7 fold.

**Fig. 3:**
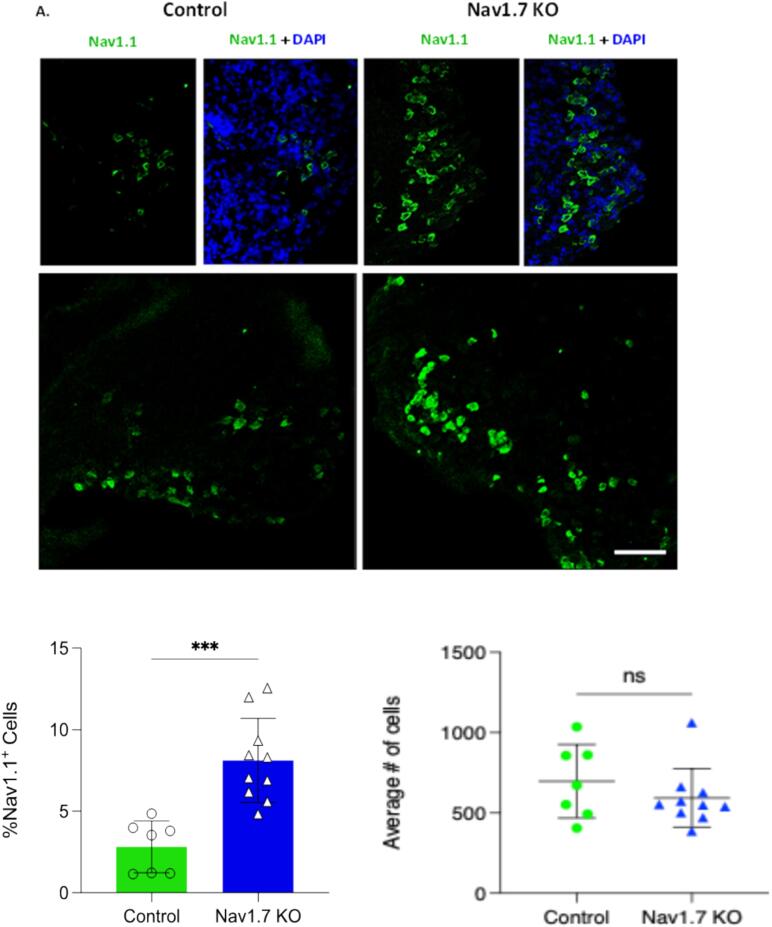
Increased Nav1.1 Expression in the Dorsal Root Ganglia of Nav1.7 KO Mice.

We also used immunocytochemical analysis to examine changes in Nav1.1 expression in sensory neurons (Fig. 3). We found that the MS analysis was confirmed with a 3-fold increase in the number of positive neurons expressing Na_v_1.1. We expressed positive cells as a percentage of all the cells in a section, and the total number of cells was similar in control and Na_v_1.7 embryonic null mice.

**Fig. 4:**
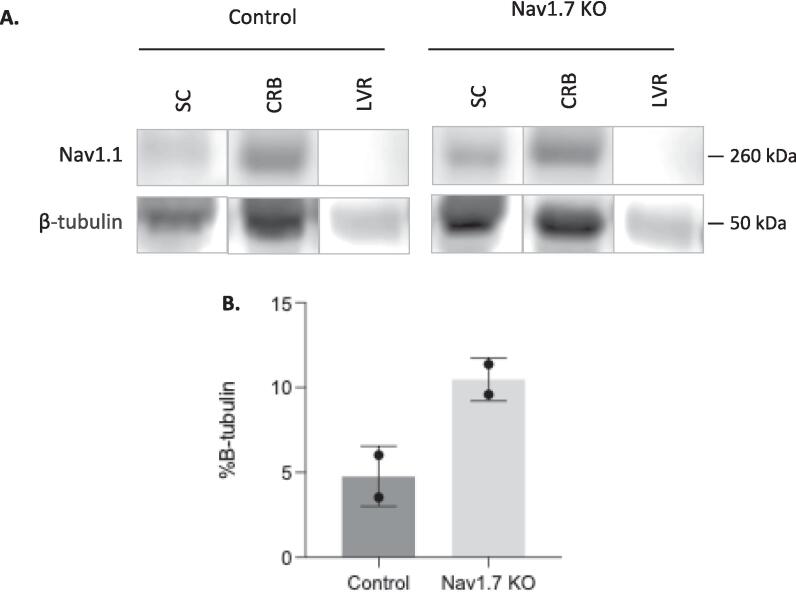
**Western Blot Analysis Using β-Tubulin as a Loading Control Confirms Nav1.1 Upregulation in the Spinal Cord of Na_v_1.7 KO Mice.** (**A**) Representative Western blot images showing Nav1.1 expression in the spinal cord, cerebellum and liver of control and Nav1.7 KO mice. The cerebellum served as a positive control, and the liver was used as a negative control. (**B**) Nav1.1 band density in both control and Nav1.7 KO spinal cord samples is presented as a percentage relative to β-Tubulin. Data are presented as means. Abbreviations: LVR, liver; CRB, cerebellum; SC, spinal cord.

Western blots (Fig. 4) were again consistent with the MS data showing a 3-fold increase in immunoreactivity protein in the null DRG samples.

We conclude that Nav1.1, as well as Nav1.2 together with a range of other ion channels (Table 1, Table 2) are upregulated in the embryonic null mutant mice, and may play a role in the apparently normal excitability of peripheral neurons in the embryonic Na_v_1.7 null mouse.

### Compensatory mechanisms

**Fig. 5:**
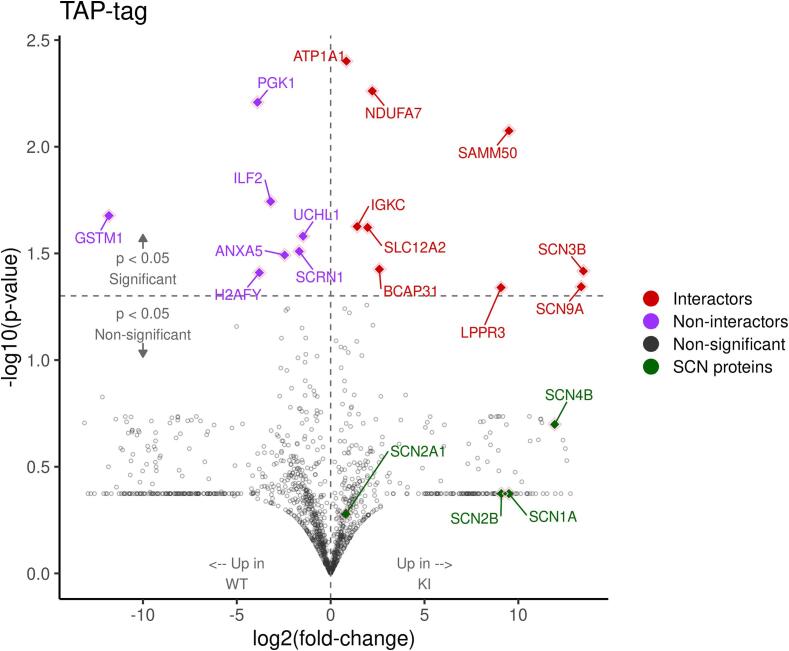
Volcano plot of differentially abundant proteins in embryonic null knock‐in mice – link to interacting proteins. *Student’s t-test was used for statistical comparisons, and ratio of group means was used for fold-change calculation. The x-axis and y-axis show log2 fold-change and log10 p-value, respectively. Proteins on the right side of the plot interact with Nav1.7 and non-interacting proteins are shown on the left side. Non-significant proteins are shown in gray, significant non-interactors (p < 0.05) with log2 fold-change < 0 with purple, and significant interactors with log2 fold-change > 0 are represented with red color. The sodium channel proteins are shown in green.*

**Fig. 6:**
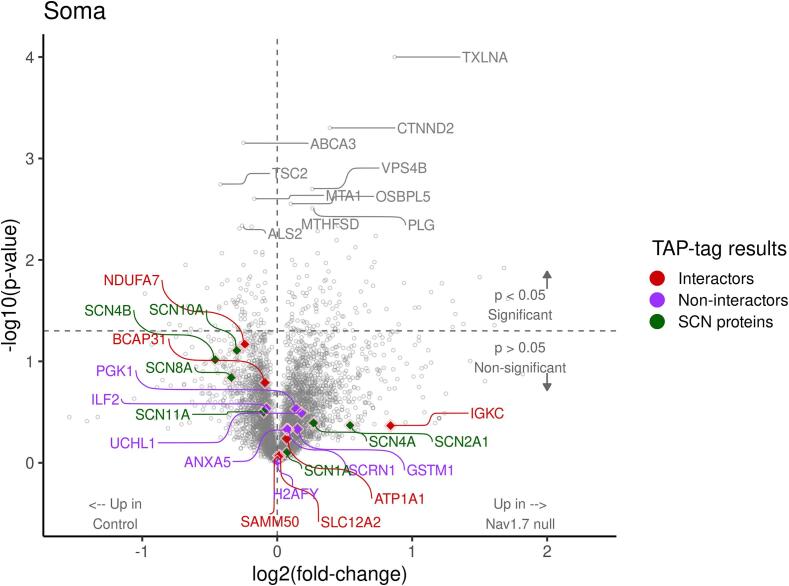
Volcano plot of differentially expressed DRG soma proteins between Nav1.7 null and control mice – links to interactors. *Student’s t-test was used for statistical comparisons, and ratio of group means was used for fold-change calculation. The x-axis and y-axis show log2 fold-change and log10 p-value, respectively. Proteins on the right side of the plot are upregulated in Nav1.7 null somata and upregulated control proteins are shown on the left side. The top 10 most significant proteins are labelled on this plot. Interactor (red) and non-interactor (purple) proteins identified by TAP-tag analysis and sodium channel proteins are highlighted on this volcano plot*.

**Fig. 7:**
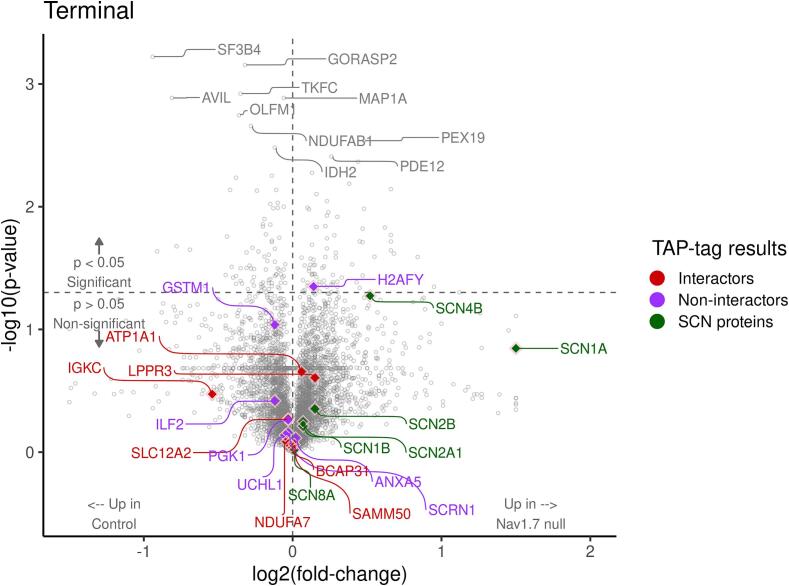
Volcano plot of differentially terminal-expressed proteins in embryonic Nav1.7 null mice. *Student’s t-test was used for statistical comparisons, and ratio of group means was used for fold-change calculation. The x-axis and y-axis show log2 fold-change and log10 p-value, respectively. Proteins on the right side of the plot are upregulated in Nav1.7 null terminals and upregulated control proteins are shown on the left side. The top 10 most significant proteins are labelled in grey on this plot. Interactor (red) and non-interactor (purple) proteins identified by TAP-tag analysis and sodium channel proteins are highlighted on this volcano plot.* These plots summarise more comprehensive data presented in Supplementary Tables 1-3.

What mechanisms are at play to alter ion channel proteins that may replace Nav1.7 to increase sensory neuron electrical excitability, but not the control of neurotransmitter release? There is no major change in sodium channel transcript levels in the embryonic knock-out mouse, so why should we see upregulation of Nav1.1 and Nav1.2 proteins? A simple view might be that proteins that interact with Nav1.7 are dysregulated in their expression and this contributes to the compensation. There is some experimental evidence that supports this simple mechanism. Sodium channels have been shown to exist as dimers (Clatot et al., 2017), and both Nav1.1 and Nav1.2 have been co-immunoprecipitated with Nav1.7 in epitope tagged Nav1.7 mouse experiments (Supp. Table 3). Other proteins that interact with Nav1.7 are also up regulated – for example the accessory β 3 and β 4 subunits, as well as the potassium channel KCNA2 (but not other potassium channels) and Synaptotagmin 1, all known to interact with Nav1.7 (Kanellopoulos et al., 2018). This does not hold true for all dysregulated proteins, but it is a potential explanation for some of the protein upregulation. Epitope tagged Nav1.7 interacting proteins are highlighted in Fig. 5, which shows proteins upregulated in Nav1.7 embryony null mice that interact with Nav1.7 (top right red quartile). Interestingly, only Nav1.1 and Nav1.2 which are upregulated in the Nav1.7 null show any potential channel interaction, whilst other sodium channels are not detectable as interactors (Supp. Table 3). The identity of proteins that are dysregulated in Nav1.7 embryonic null mice are shown in three volcano plots. Fig. 5 shows the total cohort of dysregulated proteins and Fig. 6 and Fig. 7 show the changes in soma and spinal cord terminals respectively. The complete data set are shown in Supplementary Figs. 1 and 2.

### Endogenous opioid mediated analgesia

How does activation of the opioid system occur in embryonic nulls? Is this related to altered intracellular sodium levels linked to the loss of Nav1.7? Na_v_1.7 has unique biophysical properties resulting in a persistent sodium current that has been observed in sensory neurons (Cummins et al., 1998, Baker and Bostock, 1997). This current can occur over many minutes at voltages around the resting potential. The concept that this current could act as an amplifier for small depolarizing currents has been experimentally demonstrated in hypothalamic neurons (Branco et al., 2016). This unusual channel may play a role in defining the intracellular sodium concentration. The channel is potentially expressed in a complex with μ-opioid receptors based on studies of interacting proteins (Kanellopoulos et al., 2018). Immunoprecipitation of TAP-tagged Na_v_1.7 shows the presence of GNAO1, a μ-opioid receptor binding partner. Class-A G-protein coupled receptors such as opioid receptors have a transmembrane sodium binding pocket that, when occupied, inhibits downstream signaling (Agasid et al., 2021, Jessell and Iversen, 1977). Is it possible that sodium entry through Na_v_1.7 acts as a second messenger? In the presence of Na_v_1.7, sodium normally inhibits opioid GPCR regulation of neurotransmitter release (Katritch et al., 2014, Shang et al., 2014). With the loss of Na_v_1.7, enhanced opioid signaling could occur if sodium block of GPCR activity was reduced (Pereira et al., 2018). Intriguingly both PENK and NFAT5 mRNA expression are enhanced by lowering intracellular sodium (Pereira et al., 2018). Sodium as a second messenger controlling potassium currents is a well-established phenomenon, and a broader role is plausible (Lu et al., 2015, Rose and Konnerth, 2001).

We therefore examined the effect of changing intracellular sodium concentrations on the activity of μ-opioid receptors in individual mouse sensory neurons using a sensitive electrophysiological assay. Protein Kinase A (PKA) is known to phosphorylate five serine residues in the first intracellular loop of Na_v_1.8, the tetrodotoxin-insensitive voltage-gated sodium channel that is uniquely expressed in sensory neurons (Akopian et al., 1999, Fitzgerald et al., 1999). This results in a large increase in TTX-resistant sodium channel activity that can be quantitated by electrophysiological recording. Fentanyl, acting through μ-opioid receptors and Gi proteins can suppress the activity of PKA, and diminish the level of tetrodotoxin-resistant current (Isensee et al., 2017). These observations provide us with a simple assay system for measuring opioid action in intact cells, that allows us to vary the level of intracellular sodium and examine the consequences.

**Fig. 8:**
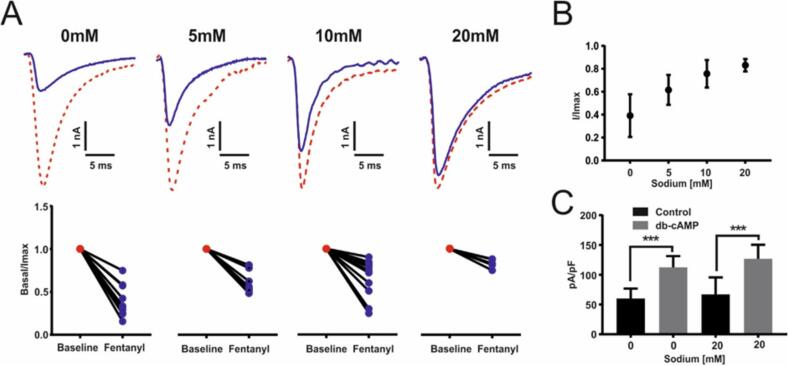
Effect of varying intracellular sodium on opioid inhibition of Nav1.8 currents. A) Electrophysiological example traces of TTXr Nav1.8 currents from dorsal root ganglia neurons following the exposure to 100 nM fentanyl at different intracellular concentrations of sodium (0 mM, 5 mM, 10 mM and 20 mM). Corresponding dot plot of TTXr Nav1.8 current following the exposure to 100 nM fentanyl at different intracellular concentrations of sodium 0 mM n = 14, 5 mM n = 10, 10 mM n = 14 and 20 mM n = 6. All currents are normalised and compared to baseline. Student’s t test, 0 mM p < 0.001, 5 mM p < 0.001, 10 mM p < 0.001 and 20 mM p < 0.05. (B) Data from (A) plotted as normalised peak currents compared to sodium concentration. Data represents + SEM. (C) Electrophysiological recordings of TTXr Nav1.8 currents in dorsal root ganglia neurons following exposure to db-cAMP in 0 mM and 20 mM intracellular concentrations of sodium. WT 0 mM vs db-cAMP 0 mM, ***p < 0.0002, change from baseline Δ = 52.3pA/pF. WT 20 mM vs db-cAMP 20 mM, ***p < 0.0006, change from baseline Δ = 59.8pA/pF. No significant change between db-cAMP 0 mM vs db-cAMP 20 mM.

We tested this concept by examining the functional expression of the TTXr sodium channel Nav1.8 and effects of external opioids with altered intracellular sodium (Fig. 8A, B). Here it can be seen that the inhibition of Na_v_1.8 functional expression by fentanyl can be potentiated in conditions of low sodium within sensory neurons in culture. The activation of PKA by dbcAMP (Fig. 8C) is independent of altered sodium concentrations, suggesting that the opioid signaling mechanism itself is regulated by sodium. The proposed mechanism thus appears to be feasible. Partial loss of Na_v_1.7 activity effected by drugs may not lower the levels of intracellular sodium to an adequate level to activate the opioid system to the level found with embryonic gene deletion

## Discussion

### Comparing Na_v_1.7 null analgesic mechanisms

This paper presents evidence that are two routes to analgesia linked to the loss of expression of Na_v_1.7 in sensory neurons. Adut partial channel deletion with inducible Cre-recombinase results in some analgesia with a clear loss of excitability of peripheral sensory neurons (Deng et al., 2023) in marked contrast to the embryonic gene deletion mouse that has normal sensory neuron excitability but a loss of neurotransmitter release in the spinal cord (MacDonald et al., 2021). Adult channel inhibition with specific drugs causes both analgesia and dramatic side effects that are not present in the embryonic nulls (Regan et al., 2024). There is no therapeutic window that avoids such effects, precluding ion channel blockers from being effective analgesic drugs (Regan et al., 2024).

However, another route to Nav1.7-mediated analgesia is possible through inhibition of trafficking. Khanna et al have focussed on the role of collapsin response mediator protein CRMP2 that is regulated by SUMOylating and phosphorylation (Brustovetsky et al., 2014). This protein has been shown to bind to and control Nav1.7 functional expression. However Khanna and others have shown that CRMP2 may also regulate many other ion channels implicated in somatosensation (Ji et al., 2019, Chi et al., 2009, Hestehave et al., 2024, Braden et al., 2022), and the fact that there is no serious loss of sympathetic function with the drugs targeting this interaction (Khanna personal communication) suggests that these useful analgesics are not targeting Nav1.7 alone, but also acting on other channels involved in peripheral pain pathways. The channel repertoires within sympathetic and sensory neuron are likely to be distinct. Hence this approach to pain control remains potentially important, as side effect issues that mitigate against channel blockers may be less significant for broad spectrum channel trafficking blockers.

The embryonic form of analgesia necessarily involves compensatory mechanisms that support the survival of null humans and mice. In the present study we have presented evidence that sodium channels Na_v_1.1 and Na_v_1.2 may compensate for embryonic loss of Na_v_1.7. How might this compensation occur? There is strong evidence that voltage-gated sodium channels exist as dimers linked by 14–3-3 proteins that interact with the first intracellular loop of the channels (Clatot et al., 2017). Indeed, the original work of Hodgkin and Huxley on the ionic basis of action potential generation (Hodgkin and Huxley, 1952a, Hodgkin and Huxley, 1952b) fits well with a model that invokes co-operative activation of closely associated channels (Huang et al., 2012, Kumar et al., 2024) rather than individual channels acting non-cooperatively. Analysis of Nav1.7 interacting proteins in a physiological setting has been carried out with an epitope tagged Nav1.7 knock-in mouse. Intriguingly Nav1.1 and Nav1.2, but not other voltage gated sodium channels are candidates to bind to Nav1.7. These are the only two isoforms that are upregulated in the embryonic null mutant mouse.

**Fig. 9:**
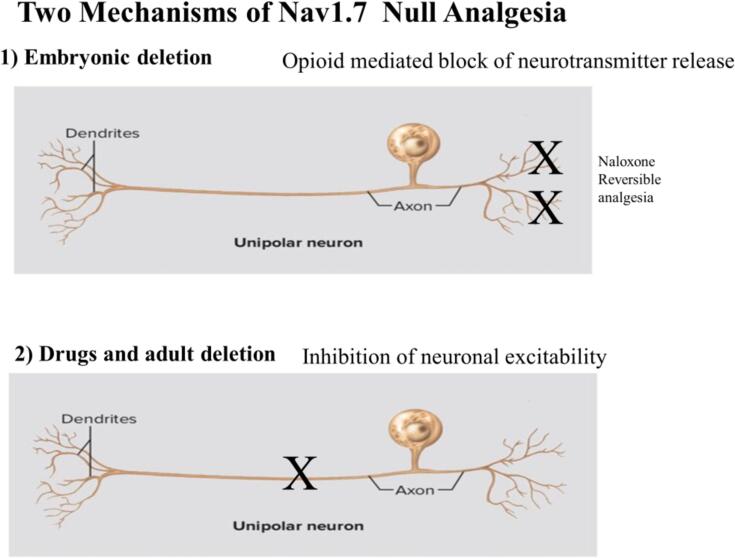
Distinct embryonic and adult mechanisms of analgesia associated with loss of expression of Na_v_1.7. *See references 16,18, 21, 22. Anosmia in both embryonic and adult nulls is a consequence of loss of sodium channel activity and electrical excitability.(1) Embryonic sensory neurons from Na_v_1.7 null mice are apparently normal in all respects. However, release of substance P and glutamate is severely compromised because of activation of the endogenous opioid system. (2) Adult sensory neuron nulls show some analgesia and anosmia linked to lowered excitability of sensory neurons and olfactory neurons respectively. The opioid signaling system seems to play no role in adult null Na_v_1.7 pain or olfactory deficits. Drug treatment blocks pain but also blocks the autonomic nervous system so there is no therapeutic potential for these agents.*

If we step away from the focus on Na_v_1.7 electrical activity, the induction of the opioid system in embryonic null sensory neurons suggests a new route to develop analgesia (Isensee et al., 2017) (Fig. 9). There is increased PENK mRNA expression in embryonic Na_v_1.7 null sensory neurons, but increasing enkephalin levels to a similar level by deleting transcription factor NFAT5 does not cause analgesia (Pereira et al., 2018). As well as increased opioid peptide expression, the opioid signaling pathway in sensory neurons was found to be massively potentiated in embryonic Na_v_1.7 null mice by the Hucho group (Isensee et al., 2017), and this seems to be the critical element in opioid signaling that leads to analgesia in Na_v_1.7 nulls. An intimate association between Na_v_1.7 and μ–opioid receptors in the membrane is consistent with the role for this receptor demonstrated by Pereira et al. (Kanellopoulos et al., 2018) in Na_v_1.7 null analgesia (see below) and the findings of the Hucho group (Isensee et al., 2017), who showed that opioid signaling was massively potentiated in Na_v_1.7 null DRG neurons. One of the G-proteins involved in μ-opioid signaling, GNAO1 is linked to Na_v_1.7 in studies of epitope tagged Na_v_1.7 interacting proteins, consistent with a close physical relationship between Na_v_1.7 and μ-opioid receptor expression (Kanellopoulos et al., 2018).

The experimental evidence suggests that lowering intracellular sodium levels can activate the endogenous opioid system with subsequent effects on neurotransmitter release. The role of sodium in G-protein mediated signaling has been a topic of interest for some time. Pert and Snyder showed the influence of sodium on opioid receptor activity in 1974, demonstrating that increased sodium concentrations caused diminished agonist binding (Pert et al., 1973, Katritch et al., 2014). Forty years later the binding site for sodium on the δ-opioid receptor was identified by Fenalti et al. (2014). Opioid receptors are members of the Class-A GPCR family that comprises about 700 members.

Human embryonic Na_v_1.7 nulls have a lifetime of endogenous opioid-induced analgesia without respiratory depression, euphoria or constipation (although they all have Charcot's joints by teenage-hood and other risks inherent on having no pain to guide their behaviours). The opioid system is massively potentiated in Na_v_1.7 null sensory neurons (Isensee et al., 2017, MacDonald et al., 2021). Does the induction of the opioid system require complete (genetic deletion) rather than partial (pharmacological) loss of Na_v_1.7 activity to induce opioid-mediated analgesia via lowered sodium levels? Expensive sensory neuron specific gene therapy (ASOs, siRNA, CRISPR) that profoundly lowers Na_v_1.7 levels only in sensory neurons may be considered as a potential route to analgesia, but Na_v_1.7 in sympathetic neurons is clearly also important for pain. Defining the precise mechanisms through which embryonic Na_v_1.7 channel loss leads to opioid signaling potentiation, neurotransmitter release loss and profound analgesia promises to be the most useful translational aspect of Na_v_1.7 −related pain studies.

The history of Na_v_1.7-focused analgesic development raises some interesting issues (MacDonald et al., 2021, Regan et al., 2024). Firstly, mechanistic studies of the events linked to Na_v_1.7 loss *in utero* and in adults have clearly been important in terms of drug development strategies, where misinformation has played a significant role in Pharma failure (Waxman et al., 2023). Secondly, mice and humans seem to be very similar in terms of mechanisms. Thirdly, with Na_v_1.8 antagonists on the horizon, the future for pain treatment is looking brighter than it has for some time. It is ironic that genetic studies of an embryonic deletion of the pain-related Na_v_1.7 sodium channel in humans and mice should lead us back to the sensory neuron opioid system, a remarkable endogenous analgesic system that, could we manipulate it effectively, would save much human suffering. Finally, many of these studies have built on classic insights from Jessell, Iversen, Pasternak, Pert and Snyder. With the historically well-known role of a tetrodotoxin-resistant sodium channel in pain (Elliott and Elliott, 1993), it is very much “back to the future” for contemporary pain research.

## Materials and methods

### Animals

All animal experiments were approved by the United Kingdom Home Office Animals Scientific Procedures Act 1986. Experiments were conducted using both female and male mice. For experiments using transgenic mice, wild-type littermate animals were used as controls. All strains of mice used for procedures were of C57Bl/6 background. All mice used for experimentation were at least 6 weeks old.

### ADENO-associated viruses

Three different AAV plasmids were assembled using Gibson assembly and sent for packaging into AAV9 (VectorBuilder): (1) CMV-dSaCas9-ZIM3-pA; (2) U6-sgRNA A, U6-sgRNA B, CMV-tdTomato-WPRE-pA, U6-sgRNA C, U6-sgRNA D; and (3) CMV-tdTomato-WPRE-pA. Guide RNA sequences were designed to map to the promoter of *Scn10a* (sgRNA A: GCCCGTCCTTAGCAGGATGGG; sgRNA B: GGGGGACAAAACACGCTTTG; sgRNA C: CTACAAGGAATCACGCCTTC and sgRNA D: GGCGTGATTCCTTGTAGATCC). C57BL6/J (9 weeks old, n = 6 per group) were injected by the *retro*-orbital route with either AAVs 1 and 3 (control mice) or AAVs 1 and 2 (test mice). Pain behavioural tests were carried out from 14 days post injection.

### Electrophysiology

All electrophysiological recordings were performed using an Axopatch 200B amplifier and a Digidata 1440A digitizer (Axon Instruments), controlled by Clampex software (version 10, Molecular Devices). Filamented borosilicate microelectrodes (GC150TF-7.5, Harvard Apparatus) were coated with beeswax and fire-polished using a microforge (Narishige) to give resistances of 2 to 3 megohms. For voltage-clamp experiments, the following solutions were used. The extracellular solution contains 70 mM NaCl, 70 mM choline chloride, 3 mM KCl, 1 mM MgCl2, 1 mM CaCl2, 20 mM tetraethylammonium chloride, 0.1 mM CdCl2, 300 nM TTX, 10 mM Hepes, and 10 mM glucose (pH 7.3) with NaOH. The intracellular solution contains 140 mM CsF, 1 mM EGTA, 10 mM NaCl, and 10 mM Hepes (pH 7.3) with CsOH. Unless otherwise stated, standard whole-cell currents were acquired at 25 kHz and filtered at 10 kHz (low-pass Bessel filter). After achieving whole-cell configuration, the cell was left for 5 min to dialyze the intracellular solution. A holding potential of −100 mV was applied, and series resistance was compensated by ≥70 %. All currents were leak-subtracted using a p/4 protocol. To record TTXr sodium currents, we applied a depolarizing voltage-pulse protocol to the cell; the cell was held at −100 mV and then stepped to −15 mV for 50 ms before returning back to −100 mV. This step was applied every 5 s for the duration of the experiment. The cells were continuously perfused using a gravity-fed perfusion system. All electrophysiological data were extracted using Clampfit (version 10, Molecular Devices) and analyzed using GraphPad Prism software (version 6, GraphPad).

Statistical analysis were performed with either Student’s *t* test with respective post hoc tests. *P* < 0.05 was considered statistically significant. Voltage-clamp experiments were analysed using cCLAMP software and Origin (OriginLab Corp., Northampton, MA) software programs. Current density–voltage (pA/pF) analysis by measuring peak currents at different applied voltage steps and normalised to cell capacitance. Voltage dependent activation data was fitted to a Boltzman equation y = (A2 + (A1−A2)/(1 + exp((Vh−x)/k)))*(x−Vrev), where A1 is the maximal amplitude, Vh is the potential of half-maximal activation, x is the clamped membrane potential, Vrev is the reversal potential, and k is a constant. All Boltzmann equations were fitted using ORIGIN software. ±SEM data were assumed to be normally distributed. Unpaired Student’s *t* test was used for statistical comparisons. Significance was determined at p < 0.05. Individual p values are given for each comparison made. Fentanyl dose response curve and IC50 calculations were fitted and measured using ORIGIN software.

### Proteomics

The mass spectrometry proteomics data have been deposited to the ProteomeXchange Consortium via the PRIDE partner repository with the dataset identifier PXD052513.

The sensory ganglia from all spinal levels, or superficial dorsal horn tissue were collected and boiled in lysis buffer (5 % sodium dodecyl sulphate (SDS), 5 mm tris(2-carboxyethyl)phosphine (TCEP), 10 mm chloroacetamide (CAA), 100 mm Tris, pH 8.5) for 10 min followed by micro tip probe sonication (Q705 Sonicator from Fisherbrand) for 2 min with pulses of 1 s on and 1 s off at 80 % amplitude. Protein concentration was estimated by NanoDrop (Thermo Fisher Scientific). Protein digestion was automated on a KingFisher APEX robot (Thermo Fisher Scientific) in 96-well format using a protocol from Bekker-Jensen et al. (2020) with some modifications. The 96-well comb is stored in plate #1, the sample in plate #2 in a final concentration of 70 % acetonitrile and with magnetic MagReSyn Hydroxyl beads (ReSyn Biosciences) in a protein/bead ratio of 1:2. Washing solutions are in plates #3–5 (95 % Acetonitrile (ACN)) and plates #6–7 (70 % Ethanol). Plate #8 contains 300 μL digestion solution of 100 mm Tris pH 8.5 and trypsin (Promega) in an enzyme:protein ratio of 1:100. The protein aggregation was carried out in two steps of 1 min mixing at medium mixing speed, followed by a 10 min pause each. The sequential washes were performed in 2.5 min and slow speed, without releasing the beads from the magnet. The digestion was set to 12 h at 37 degrees with slow speed. Protease activity was quenched by acidification with trifluoroacetic acid (TFA) to a final ph of 2, and the resulting peptide mixture was purified on OASIS HLB 96 wellplate (Waters). Peptides were eluted twice with 100 µL of 50 % ACN and dried in a Savant DNA120 (Thermo Fisher Scientific).

Peptides were then dissolved in 1 % TFA before liquid chromatography–tandem mass spectrometry (MS/MS) analysis. The mixture of tryptic peptides was analysed using an Ultimate3000 high-performance liquid chromatography system coupled online to an Eclipse mass spectrometer (Thermo Fisher Scientific). Buffer A consisted of water acidified with 0.1 % formic acid, while buffer B was 80 % acetonitrile and 20 % water with 0.1 % formic acid. The peptides were first trapped for 1 min at 30 μl/min with 100 % buffer A on a trap (0.3 mm by 5 mm with PepMap C18, 5 μm, 100 Å; Thermo Fisher Scientific); after trapping, the peptides were separated by a 50 cm μPAC Neo HPLC Column (Thermo Fisher Scientific). The gradient was 7 to 35 % B in 43 min at 750 nl/min. Buffer B was then raised to 55 % in 3 min and increased to 99 % for the cleaning step. Peptides were ionized using a spray voltage of 2.1 kV and a capillary heated at 280 °C. The mass spectrometer was set to acquire full-scan MS spectra (350 to 1400 mass/charge ratio) for a maximum injection time set to Auto at a mass resolution of 60,000 and an automated gain control (AGC) target value of 100 %. For MSMS fragmentation we chose the DIA approach: AGC target value for fragment spectra was set at 200 %. 60 windows of 10 Da were used with an overlap of 1 Da (*m*/*z* range from 380 to 980). Resolution was set to 15,000 and IT to 40 ms. Normalized collision energy was set at 30 %. All raw files were analysed by Spectronaut v18.7, searching against library generated automatically using mouse proteome (downloaded from UniProt) and automatic settings.

### Bioinformatics

The normalized spectral index quantity of proteins was used as input for the data analysis. Prior to conducting statistical tests, the missing values for each variable were imputed using the arithmetic mean of the observed values for that variable. Data distribution and homoscedasticity were assessed using Shapiro-Wilk (stats v3.6.2) and Breusch-Pagan tests (lmtest v0.9.40, https://doi.org/10.32614/CRAN.package.lmtest), respectively. P-value distribution of normality and homoscedasticity tests suggested that our proteomics data was normally distributed and appropriate for *t*-test assumptions. Therefore, imputed values with unpaired Student’s *t*-test were used for statistical comparisons. P < 0.05 was considered statistically significant. The mean value of each variable was used to calculate fold-change and define the direction of the regulation (up/down).Volcano plots were used to illustrate differentially abundant proteins using R package easylabel v0.2.4 (https://github.com/myles-lewis/easylabel) Additionally, these plots were supplemented with sodium channel proteins (with SCN- prefix) and significantly upregulated proteins in the NaV1.7knock-in group that were reported through the TAP-tag assay (Kanellopoulos et al., 2018).

### Neuronal cultures

Experiments were conducted using both female and male mice. For experiments using transgenic mice, wild-type littermate animals were used as controls. All strains of mice used for procedures were of C57Bl/6 background. All mice used for experimentation were at least 6 weeks old. Sensory neurons were isolated as described (Macdonald et al., 2021).

### Co-immunoprecipitation

Epitope-tagged Nav1.7 mice were generated, characterised and used for immunoprecipitation and mass spectrometric studies as described (Kanellopoulos et al., 2018). Nav1.7 complexes were purified with M2 Magnetic FLAG coupled beads (Sigma-Aldrich).

### Western blot

DRG, spinal cord, liver, and cerebellum from Nav1.7 KO and control mice were dissected and homogenised using a Precellys Minilys homogeniser in 1x RIPA buffer with protease inhibitor. Supernatants were collected and protein concentrations were measured using the Pierce BCA Protein Assay Kit. Then, 75 μg of each sample was separated on an SDS-PAGE gel using the Bio-Rad Mini-PROTEAN Vertical Electrophoresis Cell System. The proteins were transferred to an Immobilin-P membrane (IPVH00010, Millipore) in transfer buffer (25 mM Tris-HCl, pH 8.3, 192 mM glycine, 0.1 % SDS, and 20 % methanol) for 1 h at 100 V with a Bio-Rad transfer cell system. The membrane was blocked with PBS containing 0.1 % Tween 20 and 5 % semi-skimmed milk powder for 90 mins at RT and incubated overnight at 4 °C with primary antibodies: rabbit polyclonal anti-Nav1.1 (1:200, Millipore) and rabbit anti-β-Actin (Invitrogen, 1:1000), diluted in blocking buffer. Membranes were washed 4x10 mins in PBS- Tween 20 and incubated with secondary antibody, goat anti-rabbit anti-IgG-HRP (1:25000), in blocking buffer at RT for 1 h. After secondary antibody incubation, membranes were washed 4x10 mins in PBS-Tween 20 and 1x10 mins with PBS. The membrane was incubated with SuperSignal™ West Atto Ultimate Sensitivity Substrate for 5 mins, as per the manufacturer’s instructions. Images were acquired using a LI-COR C-DiGit® Blot Scanner and analysed with Image Studio 5 software. Each band density was normalised to the corresponding β-tubulin band to ensure accurate quantification.

### Quantitative PCR

DRGs were dissected, immersed in TRIzol, and stored at −80 °C until use. RNA was extracted using TRIzol® Reagent (Ambion Life Technologies) according to the manufacturer’s protocol, and then purified with the PureLink™ RNA Micro Kit (Invitrogen). Reverse transcription was carried out using iScript™ Reverse Transcription Supermix (Bio-Rad) for RT-qPCR. Complementary DNA (cDNA) amplification was performed in duplicates using PowerUp™ SYBR™ Green Master Mix with specific primers (Table 1). Amplification was conducted with the following cycling programme: 95 °C for 2 min, followed by 40 cycles at 60 °C for 10 s, 72 °C for 10 s, and 95 °C for 10 s. DNA amplification was quantified using a Bio-Rad CFX Connect™ Real-Time PCR Detection System. Target gene expression levels were normalised to the housekeeping gene mRNA (Actin). Fold changes were calculated using the 2−ΔΔCt method (Livak and Schmittgen, 2001), with wild-type littermate DRG cDNA samples as the calibrator. Data are presented as the mean relative fold gene expression ± standard deviation (SD). Immunohistochemistry L3-L6 DRGs were dissected and cryoprotected overnight at 4 °C in phosphate-buffered saline (PBS) with 30 % sucrose. Cryoprotected tissues were embedded in OCT compound and snap-frozen on dry ice. Sections of DRG (11 μm) were mounted on Menzel Gläser SuperFrost® Plus slides (Thermo Scientific) and dried overnight at room temperature (RT). Slides were washed once with PBS containing 0.3 % Triton X-100 (PBST) to remove OCT compound, then post-fixed with pre-cooled (−20 °C) acetone for 10 mins, dried at RT for 20 mins, and washed 2x5 mins in PBS. Slides were incubated with blocking buffer (10 % goat serum in PBST) for 90 mins, then with mouse monoclonal anti-Nav1.1 (1:250, NeuroMab, clone K74/71) and rabbit anti-NF200 (1:250, Merck) overnight at 4 °C in a sealed humidified chamber. After washing 3x10 mins in PBS, slides were incubated with goat anti-mouse Alexa Fluor 488 (1:1000), goat anti-rabbit Alexa Flour 594 (1:1000) and DAPI (1:2000) for 1 h at RT. All antibodies were diluted in blocking buffer. After washing 3x10 mins in PBS and once for 2 mins in distilled water, slides were dried, mounted with mounting medium (Abcam), and covered with Menzel Gläser coverslips. Images were acquired using a Leica SP8 confocal microscope. Total cells and Nav1.1-positive cells were quantified in triplicate using ImageJ software (Schneider, Rasband and Eliceiri, 2012), and data were normalised to show the percentage of Nav1.1- positive cells in each DRG section.

## Funding

We acknowledge with gratitude the following sources of funding: Versus Arthritis UK (21950), Medical Research Council (MR/V012509/1; 571476), the Wellcome Trust and Cancer Research UK (185341) and Cambridge NIHR BRC. The mass spectrometer was funded by a Wellcome multiuser equipment grant to KT (221521/Z/20/Z).

## CRediT authorship contribution statement

**Federico Iseppon:** Writing – review & editing, Writing – original draft, Investigation. **Alexandros H. Kanellopoulos:** Investigation. **Naxi Tian:** Investigation. **Jun Zhou:** Investigation. **Gozde Caan:** Investigation. **Riccardo Chiozzi:** Methodology, Funding acquisition. **Konstantinos Thalassinos:** Methodology, Investigation, Formal analysis. **Cankut Çubuk:** Formal analysis, Data curation. **Myles J. Lewis:** Methodology, Formal analysis, Data curation. **James J. Cox:** Writing – original draft, Supervision. **Jing Zhao:** Writing – original draft, Supervision, Conceptualization. **Christopher G. Woods:** Writing – review & editing, Writing – original draft, Conceptualization. **John N. Wood:** Writing – review & editing, Writing – original draft, Supervision, Methodology, Investigation, Funding acquisition, Conceptualization.

## Declaration of competing interest

The authors declare that they have no known competing financial interests or personal relationships that could have appeared to influence the work reported in this paper.

## Data Availability

Data will be made available on request.
