# Supplementary material for: Sodium channels Nav1.7, Nav1.8 and pain; two distinct mechanisms for Nav1.7 null analgesia
Source: Neurobiol Pain. 2024 Oct 11;16:100168. doi: 10.1016/j.ynpai.2024.100168 (PMC11570969; doi:10.1016/j.ynpai.2024.100168)
Supplement: Supplementary Data 1 [file mmc1.pdf]

Supplementary Figure 1 - DRG somata

Individual mouse values for defined proteins (vertical columns) were averaged and null mutant data compared with normal mouse data. The fold change and p value are shown in the right hand side 2 columns

Dorsal root ganglia protein analysed by Spectronaut v18.7

Male samples Black text

Female samples Red text

Control mice Blue cells

Nav1.7 null mice Yellow cells

PG.ProteinGroups	PG.Genes	PG.ProteinDescriptions	[1] 080320	[4] 080320	[5] 080320	[8] 080320	AVERAGES	[2] 080320	[3] 080320	[6] 080320	[7] 080320	AVERAGES	Fold-Change (KO/WT)	TTESTS KO-WT
Q8VC30	Tkfc	Triokinase/FMN cyclase	16168.14	11027.43	14275.37	15308.44	**14195**	11319.49	12515.37	9483.986	10449.85	**10942**	**1.2973**	**0.04593**
Q8VCI5	Pex19	Peroxisomal biogenesis factor 19	15303.42	16372.68	16137.3	13693.46	**15377**	13029.26	15321.31	11699.05	8925.848	**12244**	**1.2559**	**0.07652**
Q8QZY9	Sf3b4	Splicing factor 3B subunit 4	8515.416	11132.98	11409.23	9516.26	**10143**	6790.313	7675.7	6348.563	9140.807	**7489**	**1.3545**	**0.02796**
Q99JX3	Gorasp2	Golgi reassembly-stacking protein 2	57337.19	59234.34	62075.81	50034.45	**57170**	54065.29	53349.02	58591.86	62736.21	**57186**	**0.9997**	**0.99656**
Q704Y3	Trpv1	Transient receptor potential cation channel subfamil	22428.97	30826.87	36081.84	34350.38	**30922**	20291.66	29548.31	24896.3	19895.76	**23658**	**1.3070**	**0.10364**
O88998	Olfm1	Noelin	10339.59	8098.277	9610.639	9010.023	**9265**	5316.213	5122.29		7590.54	**6010**	**1.5416**	**0.01333**
P54071	Idh2	Isocitrate dehydrogenase [NADP], mitochondrial	206784.2	195754.8	187107.6	213408.8	**200764**	199246.2	207317.7	177421.8	185059.9	**192261**	**1.0442**	**0.37741**
O88398	Avil	Advillin	429562.7	232004.5	202504.9	222278.4	**271588**	427507.6	229983.9	438048.8	388336.8	**370969**	**0.7321**	**0.21475**
Q9QYR6	Map1a	Microtubule-associated protein 1A	416582.8	430135.3	375091.3	485487.2	**426824**	422089.4	440671.4	421293.3	424001.9	**427014**	**0.9996**	**0.99375**
Q9CR21	Ndufab1	Acyl carrier protein, mitochondrial	22156.81	25548.42	16541.62	19874.91	**21030**	18018.92	25902	13976.02	14080.6	**17994**	**1.1687**	**0.40377**
Q7M759	Abhd17b	Alpha/beta hydrolase domain-containing protein 17E	18566.93	17905.1	14615.14	18786.51	**17468**	17036.47	16578.14	28072.32	23209.84	**21224**	**0.8230**	**0.24354**
Q3UEB3	Puf60	Poly(U)-binding-splicing factor PUF60	93834.4	90546.91	102957.8	98329.84	**96417**	98947.19	96859.33	114140.4	110199.3	**105037**	**0.9179**	**0.13606**
Q3TIU4	Pde12	2',5'-phosphodiesterase 12	17564.62	18281.58	18694.58	13925.95	**17117**	15113.22	18839.39	15864.29	15298.87	**16279**	**1.0515**	**0.56951**
Q8BP92	Rcn2	Reticulocalbin-2	8923.335	10900.86	2193.029	5456.339	**6868**	7282.04	15949.5	6026.395		**9753**	**0.7043**	**0.44273**
P56379	Atp5mpl	ATP synthase subunit ATP5MPL, mitochondrial	118077.5	86127.64	115993.1	101445.5	**105411**	102628.4	79110.47	55044.27	82732.97	**79879**	**1.3196**	**0.08240**
P61027	Rab10	Ras-related protein Rab-10	341715.1	367267.7	356842.1	382025.9	**361963**	378843.8	362213.9	343212.9	392600.8	**369218**	**0.9803**	**0.61382**
Q61753	Phgdh	D-3-phosphoglycerate dehydrogenase	311953.1	323688.7	356968.3	308674.8	**325321**	297212.7	328649.8	382927.7	355947.8	**341184**	**0.9535**	**0.48711**
Q6ZWR6	Syne1	Nesprin-1	12842.98	11798.9	12809.02	12207.32	**12415**	12511.84	12041.84	10571.75	11926.13	**11763**	**1.0554**	**0.22929**
P47867	Scg3	Secretogranin-3	29862.84	23947.89	26853.8	25794.01	**26615**	25746.64	26160.29	27559.04	33085.12	**28138**	**0.9459**	**0.49523**
P47708	Rph3a	Rabphilin-3A	10398.03	8938.403	9114.167	9575.832	**9507**	10073.85	14622.04	9463.82	9836.013	**10999**	**0.8643**	**0.28010**
Q9D6R2	Idh3a	Isocitrate dehydrogenase [NAD] subunit alpha, mitoc	286478.8	291085.7	285094.3	330564.3	**298306**	293834.3	321049.3	333400.1	334789.2	**320768**	**0.9300**	**0.16984**
Q06890	Clu	Clusterin	73356.31	70620.03	65916.34	76024.85	**71479**	55597.97	79658.98	50950.2	56857.32	**60766**	**1.1763**	**0.16501**
P50516	Atp6v1a	V-type proton ATPase catalytic subunit A	235100.4	239442.9	203127.8	206173.4	**220961**	230935	229985.7	202257.5	194102.2	**214320**	**1.0310**	**0.63774**
Q6W8Q3	Pcp4l1	Purkinje cell protein 4-like protein 1	21497.35	26413.13	21679.13	25600.95	**23798**	25441.77	24322.9	23339.41	20499.89	**23401**	**1.0170**	**0.81974**
Q61885	Mog	Myelin-oligodendrocyte glycoprotein	1114183	88127.71	401122.4		**534478**	31542.89	96029.01			**63786**	**8.3792**	**0.31672**
P26638	Sars1	Serine--tRNA ligase, cytoplasmic	267350.8	280410.8	259251.1	256494.9	**265877**	256561.2	265626.4	271236.9	244895.1	**259580**	**1.0243**	**0.45394**
Q8BH95	Echs1	Enoyl-CoA hydratase, mitochondrial	167813.8	154811.3	171509.9	174908.4	**167261**	147801.1	161043	153057.8	168556.5	**157615**	**1.0612**	**0.17820**
P49312	Hnrnpa1	Heterogeneous nuclear ribonucleoprotein A1	237565.3	246963.5	267403.4	287537.7	**259867**	263918.2	260809.6	307798.5	307093.7	**284905**	**0.9121**	**0.19430**
Q6ZQ82	Arhgap26	Rho GTPase-activating protein 26	18905.91	15263.4	13194.23	16166.28	**15882**	13888.92	12406.15	13568.14	14533.99	**13599**	**1.1679**	**0.12122**
P55099	Tac3	Tachykinin-3												
Q6NVE9	Pptc7	Protein phosphatase PTC7 homolog	12834.2	8985.343	12734.55	12145.99	**11675**	12012.4	11941.93	13215.89	11247.93	**12105**	**0.9645**	**0.68163**
Q8R3D1	Tbc1d13	TBC1 domain family member 13	17471.24	15479.36	12976.78	11876.61	**14451**	14557.81	15973.81	17083.59	15296.88	**15728**	**0.9188**	**0.38636**
P0C673	Igsf11	Immunoglobulin superfamily member 11	31278.94	38600.62	25592.82	28654.53	**31032**	27809.29	31426.77	19462.71	30188.71	**27222**	**1.1400**	**0.36277**
Q9D7P6	Iscu	Iron-sulfur cluster assembly enzyme ISCU	38908.94	42365.48	46837.35	48903.45	**44254**	44061.04	44566.58	50269.82	48924.39	**46955**	**0.9425**	**0.36042**
Q63844	Mapk3	Mitogen-activated protein kinase 3	562903.2	577989.1	544357.1	515623.9	**550218**	560633.1	551167.2	565172.6	535447.7	**553105**	**0.9948**	**0.85323**
Q9R0K7	Atp2b2	Plasma membrane calcium-transporting ATPase 2	75328.81	75945.88	58673.11	80023.64	**72493**	77531.42	72945.21	60026.14	72369.76	**70718**	**1.0251**	**0.77837**
Q9ER99	Rsc1a1	Regulatory solute carrier protein family 1 member 1	37951.09	38149.18	21714.38	34974.21	**33197**	28544.39	35483.04	17871.63	28262.4	**27540**	**1.2054**	**0.32894**
Q80ZW2	Them6	Protein THEM6	137363.9	159916.2	161617.9	192646.7	**162886**	145801.4	163761.8	180279.8	176135.3	**166495**	**0.9783**	**0.80166**
Q9Z2X1	Hnrnpf	Heterogeneous nuclear ribonucleoprotein F	56370.86	59738.87	58235.13	62153.62	**59125**	60709.49	60924.1	69464.88	65717.43	**64204**	**0.9209**	**0.08158**
P97493	Txn2	Thioredoxin, mitochondrial	23831.56	32091.75	21695.02	19479.96	**24275**	19530.69	28474.67	19894.03	19856.56	**21939**	**1.1065**	**0.53071**
Q9QZD8	Slc25a10	Mitochondrial dicarboxylate carrier	131997.9	136552.2	175485.8	158107.4	**150536**	142748.1	140814.2	165398.4	188735.8	**159424**	**0.9442**	**0.57773**
Q8BGR8	Gskip	GSK3B-interacting protein	8940.687	9755.482	8050.327	8332.655	**8770**	10607.59	10904.86	11539.41	4537.297	**9397**	**0.9332**	**0.72077**
Q60931	Vdac3	Voltage-dependent anion-selective channel protein 3	347207.8	325340	385758	409636.2	**366986**	366983.8	374821	401449.3	452701.6	**398989**	**0.9198**	**0.28191**
Q9R0X4	Acot9	Acyl-coenzyme A thioesterase 9, mitochondrial	137037.6	122568.7	132744.3	156469.1	**137205**	149592.1	150158.4	151424.5	152410.3	**150896**	**0.9093**	**0.10324**
Q8BMF3	Me3	NADP-dependent malic enzyme, mitochondrial	8448.739	12080.21	8461.885	10691.06	**9920**	8407.8	10525.32	7910.924	9142.457	**8997**	**1.1027**	**0.41618**
Q8BTV1	Tusc3	Tumor suppressor candidate 3	59702.79	64021.82	59337.37	49914.49	**58244**	56801.73	58596.11	46064.75	58272.26	**54934**	**1.0603**	**0.46170**
Q91WM1	Strbp	Spermatid perinuclear RNA-binding protein	27708.79	25705.68	25654.52	21229.51	**25075**	26215.13	18998.63	21263.52	25938.72	**23104**	**1.0853**	**0.41359**
Q8BXA5	Clptm1l	Lipid scramblase CLPTM1L	17325.74	14772.34	15133.52	18289.58	**16380**	15056.8	17336.47	15440.4	18056.14	**16472**	**0.9944**	**0.93699**

P47962	Rpl5	Large ribosomal subunit protein uL18	202906.1	202922.8	233989.1	224413.8	216058	205781.4	215166.8	251342	244518.4	229202	0.9427	0.36988
Q9CQA3	Sdhb	Succinate dehydrogenase [ubiquinone] iron-sulfur su	211764.3	207190.8	220689.9	233137.6	218196	208461.7	223450.6	218573.7	216613.4	216775	1.0066	0.83452
Q8JZX9	Cdc42ep2	Cdc42 effector protein 2	51835.52	20414.36	29245.45	24829.5	31581	19135.02	19299.04	44687.68	29624.1	28186	1.1204	0.72552
Q9CQH7	Btf3l4	Transcription factor BTF3 homolog 4	58597.66	64377.64	67487.45	57756.97	62055	62884.53	60329.9	63591.43	62724.4	62383	0.9947	0.89753
P61290	Psme3	Proteasome activator complex subunit 3	26492.98	24563.13	28325.68	18674.33	24514	27400.04	22045.02	26578.62	21260.97	24321	1.0079	0.94347
O54829	Rgs7	Regulator of G-protein signaling 7	12146.11	12463.97	10676.01	14400.71	12422	13781.24	16665.86	12763.02	13725.14	14234	0.8727	0.16290
P54823	Ddx6	Probable ATP-dependent RNA helicase DDX6	198875.8	186278.3	196416	202006.3	195894	168149.7	197200.3	185386.7	208478.6	189804	1.0321	0.53550
A6X935	Itih4	Inter alpha-trypsin inhibitor, heavy chain 4	25076.14	16997.5	14441.11	20960.65	19369	13928.05	12298.24	18066.05	10498.16	13698	1.4140	0.09223
P50153	Gng4	Guanine nucleotide-binding protein G(I)/G(S)/G(O) s	12215.54	26067.78	7388.535	9303.096	13744		17074.99			17075	0.8049	
Q8K4B0	Mta1	Metastasis-associated protein MTA1	16423.05	16381.02	17042.86	15518.13	16341	17982.96	18770.38	18240.05	18739.64	18433	0.8865	0.00128
Q3THS6	Mat2a	S-adenosylmethionine synthase isoform type-2	92316.72	85581.26	82053.46	73306.52	83314	80692.31	82519.08	82907.66	75842.44	80490	1.0351	0.53359
Q99PU8	Dhx30	ATP-dependent RNA helicase DHX30	47993.09	42029.79	44945.13	51908.27	46719	45609.33	47547.48	46207.52	52173.09	47884	0.9757	0.66797
Q9QXZ0	Macf1	Microtubule-actin cross-linking factor 1, isoforms 1/:	103167.3	96646.76	92041.71	97917.42	97443	94408.49	101064.3	87727.16	97654.92	95214	1.0234	0.56350
Q5SSZ5	Tns3	Tensin-3	61606.77	61748.83	68069.55	56712.09	62034	64257.71	56898.52	61247.3	61520.12	60981	1.0173	0.71785
Q8BG67	Efr3a	Protein EFR3 homolog A	17828.1	11178.95	18322.71	11148.09	14619	16217.77	16361.37	10194.93	7559.602	12583	1.1618	0.51949
Q8K273	Mmgt1	ER membrane protein complex subunit 5	47207.79	43646.07	59126.81	57495.35	51869	42278.57	45017.67	52962.38	55927.39	49047	1.0575	0.59190
Q9CYK1	Wars2	Tryptophan--tRNA ligase, mitochondrial												
Q8BZB2	Ppcdc	Phosphopantothenoylcysteine decarboxylase	11628.33	11336.34	12189.93	9251.418	11102	10594.51	7734.138		7418.013	8582	1.2935	0.07738
Q9CQ26	Stambp	STAM-binding protein	23386.9	25930.46	21977.98	21986.77	23321	24874.47	22224.87	22911.01	24583.75	23649	0.9861	0.78154
A1L3P4	Slc9a6	Sodium/hydrogen exchanger 6	13056.17	12863.92	12294.14	14943.39	13289	13433.62	15878.64	13868.17	13801.06	14245	0.9329	0.27571
Q7TSV4	Pgm2	Phosphopentomutase	16554.37	18104.96	19578.66	15858.86	17524	20186.68	15952.55	20847.56	16007.98	18249	0.9603	0.65804
Q60759	Gcdh	Glutaryl-CoA dehydrogenase, mitochondrial	33745.69	30706.65	34175.69	36192.59	33705	31305.17	33754.8	28205.38	30535.88	30950	1.0890	0.13788
Q8BG05	Hnrnpa3	Heterogeneous nuclear ribonucleoprotein A3	530130.9	516842.4	556963.8	615706	554911	546708.9	518198.8	604389.1	596773.4	566518	0.9795	0.71269
P35279	Rab6a	Ras-related protein Rab-6A	248046.6	252180.9	276293.6	284102.7	265156	274070.1	262228.3	279919.3	300370.8	279147	0.9499	0.28524
Q6ZPR5	Smpd4	Sphingomyelin phosphodiesterase 4	10529.69	9587.873	10384.09	11916.97	10605	10544.94	11023.56	11223.29	11004.18	10949	0.9686	0.52058
Q8CBH5	Mfsd6	Major facilitator superfamily domain-containing protein 6												
Q6EDY6	Carmil1	F-actin-uncapping protein LRRC16A	1860.042	1711.88		2802.789	2125			7732.647	4787.707	6260	0.3394	0.03959
Q8BXZ1	Tmx3	Protein disulfide-isomerase TMX3	164793.5	162216.1	163291.4	186391	169173	167450.5	182576.5	162531.6	181512.2	173518	0.9750	0.59064
Q8BG92	Clvs2	Clavesin-2	9443.44	8728.424	7527.889	6831.406	8133	9667.415	8033.606	5980.699	6944.291	7657	1.0622	0.64573
Q8K183	Pdxk	Pyridoxal kinase	29619.19	28443.39	24850.1	22511.15	26356	28349.36	25384.82	19405.4	21741.72	23720	1.1111	0.34334
Q61735	Cd47	Leukocyte surface antigen CD47	247110.2	217918.9	182474.6	250313.8	224454	248136.4	260767	189790.2	194728.9	223356	1.0049	0.96506
O35900	Lsm2	U6 snRNA-associated Sm-like protein LSm2	18296.68	19426.25	20846.13	17382.36	18988	19724.13	18141.98	20806.61	18717.51	19348	0.9814	0.71789
Q922K7	Nop2	Probable 28S rRNA (cytosine-C(5))-methyltransferas	10780.82	9853.734	10205.32	6590.114	9357	12084.86	7563.071	8003.328	13353.27	10251	0.9128	0.62394
Q8BFY6	Pef1	Peflin	29908.23	38483.3	30258.68	31280.51	32483	34337.46	35294.8	30031.02	29582.2	32311	1.0053	0.94748
Q3TC72	Fahd2	Fumarylacetoacetate hydrolase domain-containing p	16593.82	23067.07	22678.98	17579.38	19980	16784.66	16838.52	15605.41	22973.13	18050	1.1069	0.44645
Q62420	Sh3gl2	Endophilin-A1	79989	91389.44	72498.23	84179.23	82014	87247.84	77253.38	76500.28	63899.86	76225	1.0759	0.38676
Q78WH7	Camk2n2	Calcium/calmodulin-dependent protein kinase II inhibitor 2												
P62627	Dynlrb1	Dynein light chain roadblock-type 1	261845.5	281659.3	299247.4	318401.1	290288	282960.9	282382.2	322189.8	337394.7	306232	0.9479	0.42099
Q9DBL1	Acadsb	Short/branched chain specific acyl-CoA dehydrogen	60211.61	56988.53	69281.79	66574.81	63264	61907.25	58644.66	65783.16	73436.27	64943	0.9742	0.70706
Q9QXY6	Ehd3	EH domain-containing protein 3	152597.8	169308.4	157272.2	158373.5	159388	157349.8	166753.1	148069.7	147704.5	154969	1.0285	0.47039
Q8N7N5	Dcaf8	DDB1- and CUL4-associated factor 8	15645.69	16058.43	17220.47	15333.22	16064	17077.05	14447	18461.35	19929.55	17479	0.9191	0.29657
P98197	Atp11a	Phospholipid-transporting ATPase IH	14176.52	13032.88	14586.54	13329.93	13781	14499.8	10840.48	12019.18	17864.47	13806	0.9982	0.98823
P70398	Usp9x	Probable ubiquitin carboxyl-terminal hydrolase FAF-:	134630.5	129198.6	130059.1	127156.1	130261	128755.3	136958.3	126835.8	131643.6	131048	0.9940	0.78131
P62254	Ube2g1	Ubiquitin-conjugating enzyme E2 G1	10470.46	14104.51	13683.95	8617.19	11719	14073.64	10805.7	17911.58	12203.55	13749	0.8524	0.35495
P61294	Rab6b	Ras-related protein Rab-6B	72010.45	68403.12	74245.29	85018.74	74919	86410.89	72074.77	77293.55	83038.9	79705	0.9400	0.35488
P18581	Slc7a2	Cationic amino acid transporter 2	43915.45	51376.65	58277.56	61121.49	53673	49030.91	54819.72	50003.68	51592.54	51362	1.0450	0.58861
Q60714	Slc27a1	Long-chain fatty acid transport protein 1	116815.1	114153.8	117354.4	138946	121817	118448.6	131024.1	117727.9	134270.8	125368	0.9717	0.63745
Q501J2	Antkmt	Adenine nucleotide translocase lysine N-methyltrans	4718.905	3014.246	3978.543	4116.478	3957		6277.113			6277	0.6304	
Q9R0P5	Dstn	Destrin	369060.7	414317.9	392177.1	345780.1	380334	425178.1	358432.6	385013.8	363334.2	382990	0.9931	0.90437
Q9CPZ8	Cmc1	COX assembly mitochondrial protein homolog	30707.51	35666.25	30632.73	45275.18	35570	38091.52	40307.62	46199.51	49869.68	43617	0.8155	0.11536
Q8BX10	Pgam5	Serine/threonine-protein phosphatase PGAM5, mito	12280.38	12053.54	15627.75	10479.7	12610	7441.755	2992.311	9917.173	15745.05	9024	1.3974	0.25815
P56565	S100a1	Protein S100-A1	11103.71	15279.04	6784.985	6910.588	10020	8030.241	12065.11	5658.269	4458.969	7553	1.3265	0.38377
P08032	Spta1	Spectrin alpha chain, erythrocytic 1	89817.26	99919.1	82333.59	59116.35	82797	39828.09	57860.35	87536.24	65113.11	62584	1.3230	0.17498
Q6ZWV7	Rpl35	Large ribosomal subunit protein uL29	528870.8	530706.4	605692.3	610174.4	568861	613462.4	538741.3	693442.1	692458.6	634526	0.8965	0.18072
Q9DAI2	Ift22	Intraflagellar transport protein 22 homolog	18771.78	24465.32	15406.73	15280.13	18481	20596.59	14455.17	11559.18	16707.67	15830	1.1675	0.39206
Q6PAM0	Prkab2	5'-AMP-activated protein kinase subunit beta-2	25523.03	24189.48	20809.19	23371.64	23473	22811.85	26163.32	20586.87	19790.91	22338	1.0508	0.53782
Q9JLI8	Sart3	Squamous cell carcinoma antigen recognized by T-ce	41206.85	41839.69	42391.6	40512.79	41488	40539.07	44075.57	41067.2	37708.71	40848	1.0157	0.65586
Q99KI3	Emc3	ER membrane protein complex subunit 3	77273.13	73537.79	76444.11	79195.88	76613	75591	78868.83	80291.64	85199.6	79988	0.9578	0.19550
Q99LP6	Grpel1	GrpE protein homolog 1, mitochondrial	31214.8	29382.05	35068.79	34120.71	32447	31566.27	31225.13	33051.98	39969.02	33953	0.9556	0.55773

Q3V3Q7	Pacs2	Phosphofurin acidic cluster sorting protein 2	57759.93	58325.6	62682.65	57014.04	**58946**	62340.07	59986.59	73710.17	68736.48	**66193**	**0.8905**	**0.07467**
Q91WD5	Ndufs2	NADH dehydrogenase [ubiquinone] iron-sulfur prote	130619.9	137448.5	144752.5	163737.6	**144140**	136248.4	151882.7	138075.1	135515.5	**140430**	**1.0264**	**0.66372**
Q9JJK2	Lancl2	LanC-like protein 2	20841.68	22612.09	22345.45	26029.14	**22957**	23645.34	26354.76	20447	20548.23	**22749**	**1.0092**	**0.91107**
Q5SV85	Synrg	Synergin gamma	13025.64	12484.25	11595.54	13148.37	**12563**	12800.15	12846.12	10540.57	10990.27	**11794**	**1.0652**	**0.31228**
Q9R0N0	Galk1	Galactokinase	24752.59	23119.92	31250.98	20408.46	**24883**	22308.36	22721.26	24247.14	22708.58	**22996**	**1.0820**	**0.45147**
Q99JN2	Klhl22	Kelch-like protein 22	13196.64	14631.54	13109.45	12739.48	**13419**	12124.98	12855.43	10995.76	10091.19	**11517**	**1.1652**	**0.04197**
Q8R5L3	Vps39	Vam6/Vps39-like protein	13575.79	14388.04	15634.39	16438.72	**15009**	13903.74	16078.04	16434.25	17851.1	**16067**	**0.9342**	**0.34661**
P62897	Cycs	Cytochrome c, somatic	390020.8	428077.4	416098.5	415021.8	**412305**	352505.3	397658.2	372279.7	421905.4	**386087**	**1.0679**	**0.17578**
P80318	Cct3	T-complex protein 1 subunit gamma	448602.2	465886.3	477349.7	480342.4	**468045**	477507.5	484502.5	467110.5	487568.3	**479172**	**0.9768**	**0.23856**
Q9JHE3	Asah2	Neutral ceramidase		13758.85			**13759**	5050.095				**5050**	**2.7245**	
Q66X03	Nlrp9a	NACHT, LRR and PYD domains-containing protein 9A	9103.986				**9104**		5711.063			**5711**	**1.5941**	
Q920R0	Als2	Alsin	5568.826	5334.47	6242.545	5703.199	**5712**	6766.133	6848.751	6603.905	7116.083	**6834**	**0.8359**	**0.00224**
Q9WTK3	Gpaa1	Glycosylphosphatidylinositol anchor attachment 1 p	36778.04	35695.86	32084.76	44317.7	**37219**	34813.43	37967.19	28552.9	39723.98	**35264**	**1.0554**	**0.60233**
P34884	Mif	Macrophage migration inhibitory factor	235377.1	274223	174908.4	140305.5	**206203**	211963.2	220455.6	185967.5	164196.6	**195646**	**1.0540**	**0.75714**
P48758	Cbr1	Carbonyl reductase [NADPH] 1	128319.4	142408.2	127555.6	113830.8	**128029**	130569.1	131295.4	115453.4	101181.5	**119625**	**1.0703**	**0.39765**
Q8BJF9	Chmp2b	Charged multivesicular body protein 2b	19091.97	19834.04	20494.79	15362.93	**18696**	20753.73	15853.12	13427.11	9798.279	**14958**	**1.2499**	**0.19581**
P39053	Dnm1	Dynamin-1	364779.1	368879.6	317836.6	342818.8	**348579**	373832.7	360545.1	349532.3	340593.3	**356126**	**0.9788**	**0.60304**
Q8K2B3	Sdha	Succinate dehydrogenase [ubiquinone] flavoprotein	248690.4	244917.8	261493.5	274757.8	**257465**	225915.2	260255.9	256570.5	246516.4	**247314**	**1.0410**	**0.36039**
Q9WVR4	Fxr2	RNA-binding protein FXR2	100323.7	101432.3	109564	118321	**107410**	112282.4	106445.9	120125.1	127510.7	**116591**	**0.9213**	**0.18982**
P70206	Plxna1	Plexin-A1	13807.59	20781.67	29399.3	15143.72	**19783**	13463.49	22579.41	9578.167	13646.88	**14817**	**1.3352**	**0.31078**
Q8C5L3	Cnot2	CCR4-NOT transcription complex subunit 2	26408.31	18894.56	29331.07	16108.33	**22686**	17794.61	16437.87	3800.805	19052.46	**14271**	**1.5896**	**0.12371**
O88630	Gosr1	Golgi SNAP receptor complex member 1	16503.92	22010.45	17153.17	21660.67	**19332**	23845.42	20358.72	12801.26	18011.82	**18754**	**1.0308**	**0.83975**
Q7TNV1	Tlcd3b	Ceramide synthase	45177.93	46173.56	69381.44	61172.82	**55476**	59050.54	46442.34	48187.1	68091.38	**55443**	**1.0006**	**0.99669**
Q8BGH4	Reep1	Receptor expression-enhancing protein 1	50102.08	59151.71	58697.84	65976.18	**58482**	53775.54	61153.17	64386.82	66803.95	**61530**	**0.9505**	**0.50618**
Q9CR56	Nkiras2	NF-kappa-B inhibitor-interacting Ras-like protein 2	16811.41	13140.35	17242.25	13161.01	**15089**	13217.29	13594.43	15383.13	16233.34	**14607**	**1.0330**	**0.73020**
Q9DAM7	Tmem263	Transmembrane protein 263	32408.39	27434.69	29292.78	25274.93	**28603**	25157.02	23535.56	26736.94	25455.66	**25221**	**1.1341**	**0.08604**
Q9D855	Uqcrb	Cytochrome b-c1 complex subunit 7	75826.87	78109.3	77083.43	96341.72	**81840**	74095.59	80641.59	93200.27	82232.59	**82543**	**0.9915**	**0.91447**
Q812A2	Srgap3	SLIT-ROBO Rho GTPase-activating protein 3	14541.92	13141.82	12474.24	10105.89	**12566**	15516.12	15661.51	10274.37	12676.72	**13532**	**0.9286**	**0.56426**
Q8C996	Tmem163	Transmembrane protein 163												
Q61548	Snap91	Clathrin coat assembly protein AP180	239242.7	262046.3	234761.9	242655.7	**244677**	264606.9	258299.9	259556	231553.6	**253504**	**0.9652**	**0.39176**
Q9DBS2	Tprg1l	Tumor protein p63-regulated gene 1-like protein	47459.66	49627.44	47261.34	59755.15	**51026**	52631.05	56744.8	50804.72	53077.29	**53314**	**0.9571**	**0.50263**
Q3V3N7	Bbs1	Bardet-Biedl syndrome 1 protein homolog	3923.627	2185.49	1511.103	5178.15	**3200**	914.0998	4035.438	2621.686		**2524**	**1.2678**	**0.60947**
Q921M3	Sf3b3	Splicing factor 3B subunit 3	99693.59	98539.17	111866.8	104652.4	**103688**	104790.4	103376.4	118953.9	120452.8	**111893**	**0.9267**	**0.18287**
Q9DBG5	Plin3	Perilipin-3	79248.44	79450.43	89549.16	69005.42	**79313**	75393.57	72847.45	90948.63	82192.77	**80346**	**0.9872**	**0.86525**
P47199	Cryz	Quinone oxidoreductase	32566.4	34009.8	29341.1	24589.76	**30127**	32982.54	30765.31	21700.05	21785.51	**26808**	**1.1238**	**0.39488**
Q6PB66	Lrpprc	Leucine-rich PPR motif-containing protein, mitochon	60065.14	65736.84	69595.45	67165.48	**65641**	65380.95	71158.57	73115.33	75579.46	**71309**	**0.9205**	**0.10478**
P26041	Msn	Moesin	420750.2	410040.4	400762.4	416553.3	**412027**	405895.9	396988.8	386394	375400.8	**391170**	**1.0533**	**0.03860**
Q8CIB5	Fermt2	Fermitin family homolog 2	116763.3	121581.1	133293.6	111586.9	**120806**	122523.1	115561.4	131865.1	121858.9	**122952**	**0.9825**	**0.72068**
P80560	Ptprn2	Receptor-type tyrosine-protein phosphatase N2			1775.601		**1776**							
Q8BYB9	Poglut1	Protein O-glucosyltransferase 1	61541.18	60679.36	67578.16	70948.21	**65187**	69102.35	65816.97	81630.07	80350.51	**74225**	**0.8782**	**0.10114**
Q64010	Crk	Adapter molecule crk	77839.07	83636.93	83375.8	73425.68	**79569**	81510.8	78415.22	77707.01	71499.57	**77283**	**1.0296**	**0.50457**
Q9CYR0	Ssbp1	Single-stranded DNA-binding protein, mitochondrial	23154.03	19179.42	20973.23	25532.54	**22210**	19696.55	20318.69	20006.69	23395.52	**20854**	**1.0650**	**0.43451**
Q8BYH7	Tbc1d17	TBC1 domain family member 17	29154.77	29639.58	29234.9	28769.53	**29200**	32903.87	28707	26825.05	28469	**29226**	**0.9991**	**0.98447**
P47856	Gfpt1	Glutamine--fructose-6-phosphate aminotransferase	22052.24	21492.63	19643.29	22510	**21425**	22106.74	18256.79	21943.59	22367.1	**21169**	**1.0121**	**0.83265**
P80314	Cct2	T-complex protein 1 subunit beta	660697.2	692821.3	692883.5	713155.3	**689889**	668571.9	736656.8	729401.1	694455.5	**707271**	**0.9754**	**0.40031**
Q69ZS8	Kazn	Kazrin	10738.7	11039.8	9001.979	13766.98	**11137**	7979.423	9139.737	10858.14	13839.13	**10454**	**1.0653**	**0.68635**
P26443	Glud1	Glutamate dehydrogenase 1, mitochondrial	388764.5	393741.3	392305.6	446090.9	**405226**	390158.4	430723.6	374389.5	366280	**390388**	**1.0380**	**0.48194**
Q8JZW4	Cpne5	Copine-5												
Q9D379	Ephx1	Epoxide hydrolase 1	229779.5	244242.5	223960.8	264471.2	**240613**	230802.8	237731.7	197036.2	213284.2	**219714**	**1.0951**	**0.15488**
Q14C51	Ptcd3	Small ribosomal subunit protein mS39	30591.23	27057.46	33239.94	31555.11	**30611**	32487.26	29830.06	30508.48	36867.65	**32423**	**0.9441**	**0.41131**
P60755	Mdga2	MAM domain-containing glycosylphosphatidylinositol anchor protein 2												
E9Q3C1	C2cd2	C2 domain-containing protein 2	25791.46	22061.24	25588.95	31819.87	**26315**	27477.28	31819.41	29818.24		**29705**	**0.8859**	**0.25162**
Q8VDS8	Stx18	Syntaxin-18	26893.68	28454.9	32551.62	28408.59	**29077**	28971.51	30573.09	30360.61	32101.67	**30502**	**0.9533**	**0.33927**
Q9D3B1	Hacd2	Very-long-chain (3R)-3-hydroxyacyl-CoA dehydratase 2		7848.753	6335.695	8320.696	**7502**	11688.86	13989.73	9036.597	12545.54	**11815**	**0.6349**	**0.02283**
Q9DCS2	Mettl26	Methyltransferase-like 26	23466.96	23557.29	19737.33	19235.7	**21499**	20958.6	21667.36	14899.58	16272.81	**18450**	**1.1653**	**0.18701**
Q3UVL4	Vps51	Vacuolar protein sorting-associated protein 51 homo	50193.42	50740.27	51354.52	48167.82	**50114**	49860.99	51120.66	54530.21	50025.7	**51384**	**0.9753**	**0.36150**
Q9CYI0		Protein Njmu-R1	119679.2	114549.5	117742	128185.7	**120039**	115146.8	112271.3	116551.2	126007.2	**117494**	**1.0217**	**0.56349**
Q9WUC3	Ly6h	Lymphocyte antigen 6H	6298.812	3562.156			**4930**		6340.917			**6341**	**0.7776**	
Q9WVL3	Slc12a7	Solute carrier family 12 member 7	11578.92	9202.417	8315.646	8172.98	**9317**	9195.396	7775.096	5634.557	10381.48	**8247**	**1.1299**	**0.43797**

Q8VCM5	Mul1	Mitochondrial ubiquitin ligase activator of NFKB 1	11667.55	13391.51	13565.39	12460.56	12771	13904.25	12111.81	14285.01	13691.12	13498	0.9462	0.30641
O88895	Hdac3	Histone deacetylase 3	91600.25	96877.25	73450.67	91038.61	88242	95825.32	94807.39	77076.87	88330.59	89010	0.9914	0.91217
Q80YE4	Aatk	Serine/threonine-protein kinase LMTK1	9667.729	11072.16	16018.14	11707.45	12116	10558.96	14750.79	10921.03	14295.78	12632	0.9592	0.77895
P56380	Nudt2	Bis(5'-nucleosyl)-tetraphosphatase [asymmetrical]	36145.1	39039.11	35727.73	33298.68	36053	36291.43	33509.07	30770.58	32061.61	33158	1.0873	0.13373
P97364	Sephs2	Selenide, water dikinase 2	3396.611	1815.514	2805.875	1002.167	2255	2482.806	1235.317	2008.364	1927.506	1913	1.1785	0.58309
Q8BY89	Slc44a2	Choline transporter-like protein 2	202858.2	188852.4	137720.6	204227	183415	198071.6	229634.4	155224.4	174503.8	189359	0.9686	0.79948
Q9QZQ8	Macroh2a1	Core histone macro-H2A.1	554033	556647	538612.1	585787.4	558770	540802.6	598630.9	528683.4	570028.2	559536	0.9986	0.96829
Q61151	Ppp2r5e	Serine/threonine-protein phosphatase 2A 56 kDa reg	32961.58	33851.93	35826.55	36317.75	34739	35743.17	36655.23	36302.52	35724.17	36106	0.9621	0.15013
Q9JJ61	Galnt16	Polypeptide N-acetylgalactosaminyltransferase 16				3201.987	3202							
E9Q236	Abcc4	ATP-binding cassette sub-family C member 4	20359.92	17034.18	17482.3	23346.36	19556	17184.48	16413.32	19481.68	19045.59	18031	1.0845	0.38755
Q99LD4	Gps1	COP9 signalosome complex subunit 1	80573.95	85581.07	69980.02	63955.28	75023	72124.66	82358.69	71440.65	64237.37	72540	1.0342	0.70143
O09044	Snap23	Synaptosomal-associated protein 23	54807.38	54183.8	50444.47	54776.02	53553	51731.65	54089.97	57204.18	59493.59	55630	0.9627	0.33956
Q569Z5	Ddx46	Probable ATP-dependent RNA helicase DDX46	35019.68	30554.89	34930.44	31227.99	32933	32020.68	27735.43	30839.14	33862.6	31114	1.0585	0.33886
P20108	Prdx3	Thioredoxin-dependent peroxide reductase, mitocho	378251.6	372386.9	351999.9	411465.7	378526	379438.9	419874.8	373752.2	400032.1	393275	0.9625	0.39791
Q60963	Pla2g7	Platelet-activating factor acetylhydrolase	24336.65	21871.28	21476.39	17134.51	21205	20679.37	19539.86	23350.68	26327.88	22474	0.9435	0.57254
Q68FD5	Cltc	Clathrin heavy chain 1	1102044	1136424	1148062	1191644	1144544	1126158	1148049	1112634	1202098	1147235	0.9977	0.92389
Q3V132	Slc25a31	ADP/ATP translocase 4	23256.61	17653.47	21110.91	30923.71	23236	20466.86	20072.72	7279.052	22195.67	17504	1.3275	0.24431
Q9D826	Pipox	Peroxisomal sarcosine oxidase	11912.12	8748.502	7323.6	8954.658	9235	7623.264	8356.149	10812.48	9547.002	9085	1.0165	0.90384
Q99J83	Atg5	Autophagy protein 5	12642.32	15078.1	15979.34	8609.913	13077	15179.3	17896.45	8779.84	9960.482	12954	1.0095	0.96520
P23780	Glb1	Beta-galactosidase	52504.68	49133.27	65580.05	46516.97	53434	57744.41	48598.04	72083.69	75328.3	63439	0.8423	0.23319
Q8K2Q7	Brox	BRO1 domain-containing protein BROX	77018.34	86197.09	86969.84	66993.65	79295	86302.38	80102.27	81911.41	78317.3	81658	0.9711	0.65219
P70388	Rad50	DNA repair protein RAD50	13309.35	9998.634	12549.64	14225.41	12521	11114.47	14420.25	9712.481	9709.93	11239	1.1140	0.40609
Q920N7	Syt12	Synaptotagmin-12	58287.11		26280.23		42284	28436.69				28437	1.4869	
Q3TGW2	Eepd1	Endonuclease/exonuclease/phosphatase family dor	34944.59	36819.35	36897.84	43292.67	37989	39263.67	36196.8	42392.42	42682.18	40134	0.9465	0.40161
Q6PDY2	Ado	2-aminoethanethiol dioxygenase	28977.59	32103.57	33960.59	27597.4	30660	25518.61	25952.52	28521.08	34373.59	28591	1.0723	0.43980
O64324	Stxbp2	Syntaxin-binding protein 2	9107.833	6892.064	10559.14	7884.015	8611	11590.18	6251.286	7264.224	10224.77	8833	0.9749	0.88555
Q91YR5	Mettl13	eEF1A lysine and N-terminal methyltransferase	12528.71	14485.36	12779.67	9800.146	12398	13786.12	11720.94	5638.961	8754.86	9975	1.2429	0.27624
Q9JHU9	Isyna1	Inositol-3-phosphate synthase 1	66989.74	70067.05	66973.08	59441.11	65868	62168.63	64537.31	52702.46	53156.33	58141	1.1329	0.08804
Q99M31	Hspa14	Heat shock 70 kDa protein 14	40505.02	39368.88	38878.11	36754.08	38877	38007.92	41535.47	33927.16	38803.94	38069	1.0212	0.66213
P37040	Por	NADPH--cytochrome P450 reductase	208631.3	199081	254517.7	240840.5	225768	215021.3	206667.5	230524.1	258411.9	227656	0.9917	0.91689
Q8R016	Blmh	Bleomycin hydrolase	77574.02	79302.96	77252.35	57841.3	72993	64533.91	66852.39	57739.55	70989.55	65029	1.1225	0.21737
Q8C4Q6	Aida	Axin interactor, dorsalization-associated protein	30997.38	23483.56	30522.96	27592.97	28149	29074.17	30613.1	23002.88	28903.44	27898	1.0090	0.92042
Q91Z40	Gbp7	Guanylate-binding protein 7	12828.5	7565.926	5345.302	4036.782	7444	5946.574	5697.083	8588.429	6236.068	6617	1.1250	0.70037
Q8K4I3	Arhgef6	Rho guanine nucleotide exchange factor 6	37125.94	34064.84	33865.86	32465.82	34381	33260.1	41458.56	24572.29	31908.36	32800	1.0482	0.67568
Q80Y14	Glrx5	Glutaredoxin-related protein 5, mitochondrial	21776.33	23016.77	26104.53	23089	23497	24249.9	23093.74	28340.81	29135.3	26205	0.8967	0.17297
P61022	Chp1	Calcineurin B homologous protein 1	54690.91	56178.49	48757.53	55748.27	53844	52148.63	60120.23	48767.65	51174.33	53053	1.0149	0.80113
Q8BK64	Ahsa1	Activator of 90 kDa heat shock protein ATPase homo	225627.5	239897.4	203624.5	176269.5	211355	233349.7	187584.8	204909.7	193117.3	204740	1.0323	0.71409
Q3U3R4	Lmf1	Lipase maturation factor 1	24173.53	25230.49	29523.09	26185.3	26278	28499.31	25445.32	27059.17	32650.18	28413	0.9248	0.31076
Q8R3B1	Plcd1	1-phosphatidylinositol 4,5-bisphosphate phosphodi	37612.68	37132.66	42097.68	33572.72	37604	32762.29	36498.52	41367.4	38657.77	37321	1.0076	0.91446
Q99PL6	Ubxn6	UBX domain-containing protein 6	50786.25	51320.84	43785.72	46849.62	48186	50314.71	48230.46	46550.65	45558.52	47664	1.0110	0.80811
Q99J77	s	Sialic acid synthase	96985.34	103325.7	96682.45	76526.42	93380	87553.54	83978.73	84093.92	73716.04	82336	1.1341	0.14253
P05480	Src	Proto-oncogene tyrosine-protein kinase Src	26124.91	23460.83	25778.76	24896.44	25065	21781.44	25356.24	22207.17	25503.54	23712	1.0571	0.28757
Q811S7	Ubp1	Upstream-binding protein 1	16088.67	15436.2	16894.34	15602.26	16005	20455.89	14554.36	14058.18	15270.71	16085	0.9951	0.95986
O35127	Grcc10	Protein C10	29881.15	30934.3	29762.08	28236.34	29703	33659.29	28682.1	35327.73	31160.36	32207	0.9223	0.15880
Q61133	Gstt2	Glutathione S-transferase theta-2	32037.17	34804.62	23125.98	23453	28355	22639.23	28322.67	19380.21	9920.572	20066	1.4131	0.13969
P14094	Atp1b1	Sodium/potassium-transporting ATPase subunit beta	1807186	1702234	1477988	1804717	1698031	1658055	1698725	1461951	1572298	1597757	1.0628	0.32421
Q8VD65	Pik3r4	Phosphoinositide 3-kinase regulatory subunit 4	30602.71	29930.84	32344.44	30970.65	30962	28264.16	30397.9	26533.25	30594.9	28948	1.0696	0.11365
Q3URQ0	Tex10	Testis-expressed protein 10	6899.39	7194.468	9320.617	8302.756	7929	8417.082	7272.982	8474.612	5834.796	7500	1.0573	0.62389
Q61738	Itga7	Integrin alpha-7	67188.84	68233.21	65166	72613.42	68300	65904.2	72106.7	63682.06	68545.32	67560	1.0110	0.76796
P18872	Gnao1	Guanine nucleotide-binding protein G(o) subunit alp	256590	274247.5	220716.8	251441.8	250749	276507.2	308091	259130.5	238484.2	270553	0.9268	0.32475
P23818	Gria1	Glutamate receptor 1												
Q6P069	Sri	Sorcin	128824.2	138766.2	117977.9	107894.5	123366	139317.8	131177.4	107811.3	113076.3	122846	1.0042	0.96018
Q9CQ10	Chmp3	Charged multivesicular body protein 3	55472.84	53312.14	46834.89	49880.1	51375	39772.36	54981.7	46620.01	42242.12	45904	1.1192	0.20452
Q99M04	Lias	Lipoyl synthase, mitochondrial	10837.55	7213.499	10367.11	8179.999	9150	7919.195	8239.504	9560.453	7550.82	8317	1.1000	0.42447
Q7M729	Scn4b	Sodium channel subunit beta-4	27233.56	18268.92	24865.18	39026.32	27348	40088.69	41531.24	29941.33	39379	37735	0.7248	0.08638
O35286	Dhx15	ATP-dependent RNA helicase DHX15	84341.22	80113.5	88191.27	83721.85	84092	80799.55	82669.31	95398.44	91347.8	87554	0.9605	0.40366
P61327	Magoh	Protein mago nashi homolog	14287.49	16930.85	18415.97	13691.73	15832	14305.15	14789.23	16989.17	20886.79	16743	0.9456	0.64293
Q7TMB8	Cyfip1	Cytoplasmic FMR1-interacting protein 1	111338.1	115735	112278.9	110491.2	112461	109073.4	106384.8	104652.2	111803.6	107978	1.0415	0.06058
Q69ZP3	Pnkd	Probable hydrolase PNKD	23686.1	18241.67	27785.64	31504.64	25305	16319.67	21968.22	24060.57	31971.16	23580	1.0731	0.70300

Q9DAS9	Gng12	Guanine nucleotide-binding protein G(I)/G(S)/G(O) s	62465.23	56868.77	47827.84	70144.05	59326	60942.22	61970.06	56793.09	55842.73	58887	1.0075	0.93197
Q8BYA0	Tbcd	Tubulin-specific chaperone D	52883.29	55417.32	49316.8	44983.32	50650	54162.19	50831.98	46831.97	47517.3	49836	1.0163	0.78279
Q8K1X1	Wdr11	WD repeat-containing protein 11	19636.62	20083.48	19252.98	16847.76	18955	18548.22	20250.13	13349.25	15931.18	17020	1.1137	0.29199
P10518	Alad	Delta-aminolevulinic acid dehydratase	116745.2	160628.1	89668.26	108821.3	118966	78557.96	113901.9	83745.32	123419.6	99906	1.1908	0.34589
Q8K2Y7	Mrpl47	Large ribosomal subunit protein uL29m	19687.48	20986.02	20400.54	22964.73	21010	19557.62	22331.67	20398.15	22760.05	21262	0.9881	0.81650
Q3UIU2	Ndufb6	NADH dehydrogenase [ubiquinone] 1 beta subcomp	89349.97	105277.9	89090.14	97620.26	95335	93365.84	101367.2	94710.56	97923.69	96842	0.9844	0.73523
Q8BG51	Rhot1	Mitochondrial Rho GTPase 1	49923.55	58184.51	57258.88	59076.11	56111	54626.76	62347.12	53190.61	62740.47	58226	0.9637	0.54170
Q9JHS4	Clpx	ATP-dependent Clp protease ATP-binding subunit clp	10141.46	7001.624	10857.93	9996.301	9499	9071.112	8826.431	7130.063	13689.02	9679	0.9814	0.91644
Q9D708	S100a16	Protein S100-A16	11314.41	24068.31	8607.479	19023.52	15753	19353.02	12526.77	11999.37	8403.181	13071	1.2053	0.54804
P62500	Tsc22d1	TSC22 domain family protein 1	11148.35	15435.36	14578.63	12348.04	13378	9954.602	11788.18	12340.38	10960.34	11261	1.1880	0.10667
Q6GQW0	Abtb3	Ankyrin repeat and BTB/POZ domain-containing prot	14811.97				14812							
Q9CQU0	Txndc12	Thioredoxin domain-containing protein 12	53767.25	47948.11	61194.81	54759.84	54418	54095.06	49128.04	67599.57	69962.89	60196	0.9040	0.35452
Q9DAF3	Ddi1	Protein DDI1 homolog 1	17037.91	14295.49	13764.47		15033	15536.7	14854.1			15195	0.9893	0.91059
Q3TVI8	Pbxip1	Pre-B-cell leukemia transcription factor-interacting protein 1												
P97461	Rps5	Small ribosomal subunit protein uS7	255578.8	256710.3	272401	307190.5	272970	290633.8	293403.9	343338.3	342094	317368	0.8601	0.05770
Q62383	Supt6h	Transcription elongation factor SPT6	20896.2	20945.17	21261.73	19758.38	20715	19071.52	20162.7	16244.23	20781.83	19065	1.0866	0.16950
Q9CR51	Atp6v1g1	V-type proton ATPase subunit G 1	107119.8	121466.5	103478	102770.4	108709	96595.03	119355.8	99729.02	94922.61	102651	1.0590	0.42877
Q9JKK7	Tmod2	Tropomodulin-2	146450.6	132410.8	113220.3	138397.8	132620	128000	123572.7	133950.7	112626.7	124538	1.0649	0.37252
P01867	Ighg2b	Immunoglobulin heavy constant gamma 2B	757375.2	462185.7	169609.6	238148.7	406830	704199.1	437481.8	83838.09	117843.8	335841	1.2114	0.73148
Q9EPR5	Sorcs2	VPS10 domain-containing receptor SorCS2	14087.25	15222.11	17620.7	14567.23	15374	13571.42	16235.16	17695.33	19185.13	16672	0.9222	0.39927
Q64337	Sqstm1	Sequestosome-1	55092.59	57901.95	56064.11	64548.95	58402	59517.68	61947.33	59637.75	56064.05	59292	0.9850	0.72903
Q9WTQ8	Timm23	Mitochondrial import inner membrane translocase s	24446.82	18552.3	25650.02	28568.25	24304	26660.39	25291.85	34141.48	25879.08	27993	0.8682	0.25771
Q62245	Sos1	Son of sevenless homolog 1	21032.34	23976.87	19682.7	20559.88	21313	21253.83	21122.89	14611.79	15654.65	18161	1.1736	0.16465
Q8C5H8	Nadk2	NAD kinase 2, mitochondrial	7029.718	6940.112	9812.075	13693.07	9369	10176.43	9497.305	8058.778	5621.841	8339	1.1235	0.60363
Q60972	Rbbp4	Histone-binding protein RBBP4	80757.4	84027.67	93964.51	91382.18	87533	82721.63	81914.73	85071.07	86493.93	84050	1.0414	0.32680
P97447	Fhl1	Four and a half LIM domains protein 1	66357.09	72537.33	74267.53	57728.82	67723	65833.11	54704.3	57743.12	50243.9	57131	1.1854	0.07739
P05622	Pdgfrb	Platelet-derived growth factor receptor beta	9726.107	9736.284	3582.799	8624.548	7917	8553.651	9404.769	9361.293	9940.12	9315	0.8500	0.38621
Q5XJY5	Arcn1	Coatomer subunit delta	144078.8	142524.9	158354.2	153461.8	149605	155655.6	150578.1	169712.4	177595.8	163385	0.9157	0.10762
P24369	Ppib	Peptidyl-prolyl cis-trans isomerase B	653357.4	627693.7	766618.6	719535	691801	660577.7	675860.1	718958.5	806931.6	715582	0.9668	0.62043
Q9D6J6	Ndufv2	NADH dehydrogenase [ubiquinone] flavoprotein 2, m	193996.1	193702.6	216427.7	234636.4	209691	210528.6	215399.8	216810.4	231968.3	218677	0.9589	0.44142
Q9QX60	Dguok	Deoxyguanosine kinase, mitochondrial	45784.55	40424.28	39973.6	42689.57	42218	41607.2	43367.33	44014.21	42449.95	42860	0.9850	0.66930
Q8BIW9	Chtf18	Chromosome transmission fidelity protein 18 homol	26546.3	22392.9	35808.78	30729.69	28869	34528.53	22130.89	31837.63	35322.65	30955	0.9326	0.63542
P31648	Slc6a1	Sodium- and chloride-dependent GABA transporter 1	6658.65	7585.873	1592.004	2826.972	4666	8061.71	12095.64	4022.101	8087.162	8067	0.5784	0.17260
Q9D898	Arpc5l	Actin-related protein 2/3 complex subunit 5-like prot	67631.61	94693.8	72359.59	75412.23	77524	84509.17	91783.4	68334.61	64802.06	77357	1.0022	0.98542
Q9DB77	Uqcrc2	Cytochrome b-c1 complex subunit 2, mitochondrial	353328.4	362972.3	390510.8	436800.3	385903	384078.3	401636.4	415819.8	427384.1	407230	0.9476	0.34708
Q6PAJ1	Bcr	Breakpoint cluster region protein	22136.74	21795.1	19141.55	24499.07	21893	24912.43	21012.88	19496.97	20673.35	21524	1.0172	0.82597
Q922R1	Phaf1	Phagosome assembly factor 1	6444.538	5801.442	6548.761	6510.831	6326	7419.622	5675.721	4362.826	3610.801	5267	1.2011	0.26077
Q6PER3	Mapre3	Microtubule-associated protein RP/EB family membe	329134.9	322935.8	283490.9	305213.1	310194	288973.6	298824.2	272136.4	283886.8	285955	1.0848	0.08269
Q8K209	Adgrg1	Adhesion G-protein coupled receptor G1	29573.94	32708.35	63489.2	43726.82	42375	60352.08	37540.07	33166.74	31340.88	40600	1.0437	0.86744
Q9D0R8	Lsm12	Protein LSM12	114354.7	122439.2	126673.3	120665.1	121033	136554.2	129591.6	141982.2	150229	139589	0.8671	0.01044
Q80TL4	Phf24	PHD finger protein 24	131860	98512.48	78325.99	101642	102585	134951.3	102723.6	161424.8	144006.9	135777	0.7555	0.09152
P56212	Arpp19	cAMP-regulated phosphoprotein 19	31972.65	33800.69	29272.74	29812.06	31215	32936.95	35441.17	32463.68	31973.56	33204	0.9401	0.17554
Q9CZA6	Nde1	Nuclear distribution protein nudE homolog 1	46742.68	42720.26	53707.89	52340.29	48878	45061.48	48907.51	62747.38	60715.89	54358	0.8992	0.31855
A2AG50	Map7d2	MAP7 domain-containing protein 2	97998.66	108909.3	98741.43	123560.1	107302	101142.2	108279.1	113769.6	116979	110042	0.9751	0.70496
Q50H33	Kctd8	BTB/POZ domain-containing protein KCTD8	1492.888	496.6164	1221.694	509.9109	930	1057.714	681.1494		777.6217	839	1.1090	0.78227
Q8R555	Crtac1	Cartilage acidic protein 1	28781.35	28620.82	27423.9		28275	25292.05	30998.74		24867.03	27053	1.0452	0.57812
P28474	Adh5	Alcohol dehydrogenase class-3	80669.84	89665.86	89261.58	76982.11	84145	85687.65	80910.9	70038.85	74335.95	77743	1.0823	0.22140
Q9D1H7	Get4	Golgi to ER traffic protein 4 homolog	16233.26	16820.21	15761.21	16300.37	16279	16788.02	22899.49	14030.67	13805.51	16881	0.9643	0.78678
Q9DBL7	Coasy	Bifunctional coenzyme A synthase	54748.04	54591.39	57820.18	52336.15	54874	55695.33	51501.95	53049.98	53293.04	53385	1.0279	0.33502
Q99P30	Nudt7	Peroxisomal coenzyme A diphosphatase NUDT7	42132.83	65283.43	82548.91	71614.41	65395	69705.6	67354.91	84569.07	94080.02	78927	0.8285	0.24987
Q99J09	Wdr77	Methylosome protein WDR77	33141.94	38020.93	30990.34	27431.8	32396	37140.53	38331.19	33075.97	36651.63	36300	0.8925	0.16743
Q91WR3	Ascc2	Activating signal cointegrator 1 complex subunit 2	11180.09	11097.58	12670.45	11032.91	11495	12131.19	12148.31	7685.004	14154.05	11530	0.9970	0.98150
Q3UMY5	Eml4	Echinoderm microtubule-associated protein-like 4	18012.6	19255.32	20598.7	18906.66	19193	18723.98	20112.56	16888.71	20738.05	19116	1.0041	0.94122
Q80XH1	Kxd1	KxDL motif-containing protein 1	9683.837	6066.31	9001.087	8743.163	8374	8703.149	9223.482	4415.215	7116.816	7365	1.1370	0.48028
Q921G8	Tubgcp2	Gamma-tubulin complex component 2	10608.65	9454.231	10608.02	10536.37	10302	10666.44	10277.74	12778.52	12298.72	11505	0.8954	0.12359
Q14CH7	Aars2	Alanine--tRNA ligase, mitochondrial	3327.903	3682.9	3072.62	3079.358	3291	3724.881	2974.551	4567.15	7494.524	4690	0.7016	0.21120
Q91V64	Isoc1	Isochorismatase domain-containing protein 1	36504.76	39946.48	35095.9	35708.39	36814	44677.89	37839.7	32722.83	31311.75	36638	1.0048	0.95813
P61922	Abat	4-aminobutyrate aminotransferase, mitochondrial	116822.9	107029.7	100143	121153.7	111287	109122.8	125686.4	111738.8	118244.9	116198	0.9577	0.44560
P14131	Rps16	Small ribosomal subunit protein uS9	1010355	970349.2	1157752	1110480	1062234	1071367	1071226	1290389	1274501	1176871	0.9026	0.17669

P17225	Ptbp1	Polypyrimidine tract-binding protein 1	105513.1	101065.1	134690.2	114693.1	113990	106777.5	97256.84	128176.6	133696.2	116477	0.9787	0.83485
Q91WG5	Prkag2	5'-AMP-activated protein kinase subunit gamma-2	40609.07	37186.7	33791.77	35203.47	36698	33845.73	41600.34	34550.2	38225.87	37056	0.9903	0.88270
P47857	Pfkm	ATP-dependent 6-phosphofructokinase, muscle type	143484	153281.7	131274.7	127293.9	138834	133604.4	143541.1	124196.4	117866.8	129802	1.0696	0.31042
Q7TQF7	Amph	Amphiphysin	132386.6	134468.3	99234.37	117328.1	120854	114649.7	132884.4	113553.3	103580.7	116167	1.0403	0.66162
Q9JHG6	Rcan1	Calcipressin-1				27333.64	27334	7696.384				7696	3.5515	
P27600	Gna12	Guanine nucleotide-binding protein subunit alpha-12	27915.64	32980.16	26460.63	31880.91	29809	27397.41	32053.05	29387.18	27187.29	29006	1.0277	0.69106
O35071	Kif1c	Kinesin-like protein KIF1C	37857.08	38231.25	47100.84	35158.68	39587	49421.23	39562.54	51018.45	49764.04	47442	0.8344	0.07853
Q8K1A5	Tmem41b	Transmembrane protein 41B	8765.813	9508.922	8979.442	13381.79	10159	9618.066	13854.8	10569.69	11224.93	11317	0.8977	0.44454
P13595	Ncam1	Neural cell adhesion molecule 1	374335.3	354781.1	354452.9	357074.3	360161	362767.3	400540.3	396064.3	401848.8	390305	0.9228	0.02753
Q04736	Yes1	Tyrosine-protein kinase Yes	74032.2	75998.38	80843.38	86605.91	79370	75831.41	72977.78	81772.35	85434.18	79004	1.0046	0.92966
O35954	Pitpnm1	Membrane-associated phosphatidylinositol transfer	36212.89	37839.43	28680.81	34676.12	34352	29081.58	38163.07	29726.61	31596.02	32142	1.0688	0.47212
Q8BHS3	Rbm22	Pre-mRNA-splicing factor RBM22	11620.44	13320.11	12701.78	16453.17	13524	13164.34	13338.31	11875.46	14482.18	13215	1.0234	0.80014
Q02053	Uba1	Ubiquitin-like modifier-activating enzyme 1	581406.3	567939.6	534951.6	459432.1	535932	546070.3	548869.3	516309.3	459160.3	517602	1.0354	0.61268
O35655	Ppef1	Serine/threonine-protein phosphatase with EF-hand	9454.298	7871.293	7735.434	9098.729	8540	9729.406	10579.13	10694.63	10538.46	10385	0.8223	0.00896
Q9JK53	Prelp	Prolargin	583207.2	514596.9	397119.6	515412.8	502584	473849.2	530813.3	343714.1	391871.4	435062	1.1552	0.28000
P40237	Cd82	CD82 antigen	36252.26	32657.89	26205.61	36456.41	32893	30179.88	36887.64	27979.76	24719.42	29942	1.0986	0.43316
P70377	Fgf13	Fibroblast growth factor 13	22555.95	18557.03	22731.42	23381.65	21807	26527.93	26830.07	19932.23	18397.48	22922	0.9513	0.66517
Q0GA42	Cnnm1	Metal transporter CNNM1												
Q7TQK5	Ccdc93	Coiled-coil domain-containing protein 93	17022.61	15700.58	17755.92	15075.06	16389	17310.18	17419.59	13612.88	16032.4	16094	1.0183	0.79311
Q8K4F5	Abhd11	Protein ABHD11	19677.74	19946.25	20881.82	19112.52	19905	23026.56	20660.02	20173.6	20591.61	21113	0.9428	0.15585
P03975	Iap	IgE-binding protein	13565.14	14866.66	29519.99	20188.35	19535	13870.96	28531.07	13813.51	15506.95	17931	1.0895	0.76267
Q9R1P3	Psmb2	Proteasome subunit beta type-2	173078.5	175976.5	160843.3	144576.9	163619	168882.4	171966.9	171424.6	168816.6	170273	0.9609	0.39058
Q9DCX2	Atp5pd	ATP synthase subunit d, mitochondrial	236904.2	225742.4	227822.7	248052.1	234630	219865.3	243921	228962.9	222596.8	228837	1.0253	0.46359
Q9D6S7	Mrrf	Ribosome-recycling factor, mitochondrial	25186.36	31283.69	34268.73	36623.73	31841	28735.88	26905.19	24703.88	31418.13	27941	1.1396	0.22057
Q9CWJ9	Atic	Bifunctional purine biosynthesis protein ATIC	132458.3	136566.2	128389	114811.6	128056	126301.9	124626.9	122259.1	109157.7	120586	1.0619	0.26817
A2ALS5	Rap1gap	Rap1 GTPase-activating protein 1	23113.31	21660.98	20879.83	19392.4	21262	19115.38	21858.58	18868.3	20683.46	20131	1.0561	0.32167
Q7TSQ8	Pdpr	Pyruvate dehydrogenase phosphatase regulatory sul	28689.78	28299.96	29752.46	33309.91	30013	26745.83	30336.92	26681.67	28510.39	28069	1.0693	0.22362
P55302	Lrpap1	Alpha-2-macroglobulin receptor-associated protein	72217.07	62341.5	63454.21	68655.21	66667	53609.2	72024.23	67848.99	60752.43	63559	1.0489	0.52967
Q91W53	Golga7	Golgin subfamily A member 7	33249.87	28945.6	25157.63	31391.91	29686	30067.62	37000.2	25526.67	32196.91	31198	0.9515	0.62723
Q9D1P4	Chordc1	Cysteine and histidine-rich domain-containing prote	29288.03	33554.19	28495.31	25833.43	29293	31885.2	26256.88	27236.84	29972.76	28838	1.0158	0.83173
Q9D6J5	Ndufb8	NADH dehydrogenase [ubiquinone] 1 beta subcomp	98734.28	65934.34	67723.9	70275.12	75667	60936.06	84611.79	79039.37	73249.55	74459	1.0162	0.90042
P15864	H1-2	Histone H1.2	207699.6	144120.6	215769.3	227831.1	198855	150951.4	148135.1	223059.6	213971.4	184029	1.0806	0.60780
Q8VHH5	Agap3	Arf-GAP with GTPase, ANK repeat and PH domain-co	53003.97	55836.39	51149.23	53996.59	53497	52550.57	49619.67	48161.59	51202.75	50384	1.0618	0.06278
Q8VI63	Mob2	MOB kinase activator 2	42471.3	43264.44	34947.8	36691.1	39344	44010.95	40782.87	41984.53	40400.41	41795	0.9414	0.31294
Q8JZV7	Amdhd2	N-acetylglucosamine-6-phosphate deacetylase	37913.43	33287.07	36780.07	32585.1	35141	36167.88	36223.42	32408.42	33609.88	34602	1.0156	0.74961
P31254	Uba1y	Ubiquitin-like modifier-activating enzyme 1 Y	36412.16	38365.65	36203.06	23887.1	34217	32817.08	34291.17	23664.67	25171.44	28986	1.1805	0.27754
O54984	Get3	ATPase GET3	79244.56	84841.88	82059.95	83595.51	82435	78280.57	84645.23	75463.65	82854.79	80311	1.0265	0.41397
P10649	Gstm1	Glutathione S-transferase Mu 1	194912.3	210639.5	156166.4	177041.4	184690	208656.5	190771.5	138385.6	128981.6	166699	1.1079	0.45928
Q9CY73	Mrpl44	Large ribosomal subunit protein mL44	17987.04	19666.52	16484.82	15407.71	17387	15311.56	18125.81	13070.37	15129.85	15409	1.1283	0.20510
O08573	Lgals9	Galectin-9	57499.25	61904.54	60936.94	69741.42	62521	56642.94	69712.2	59088.65	57647.71	60773	1.0288	0.67574
Q62415	Ppp1r13b	Apoptosis-stimulating of p53 protein 1	8306.711	10768.59	8448.683	6937.006	8615	5577.772	9042.475	9580.435	2955.273	6789	1.2690	0.33605
Q8BT60	Cpne3	Copine-3	962296	971689.8	881784.3	963662	944858	1016225	941425.9	996466.6	925290.4	969852	0.9742	0.44082
P58871	Tnks1bp1	182 kDa tankyrase-1-binding protein	35862.96	37482.97	41383.85	38085.68	38204	36597.21	37398.63	40492.79	42738.8	39307	0.9719	0.56926
Q91VZ6	Smap1	Stromal membrane-associated protein 1	68702.45	63971.38	68514.97	56707.03	64474	61591.08	66440.05	62265.55	57920.14	62054	1.0390	0.49214
Q9R0N8	Syt6	Synaptotagmin-6	16380.93	11376.89	9455.002	5694.094	10727	9635.89	9098.193	9763.242	8184.417	9170	1.1697	0.51544
Q9EPE9	Atp13a1	Endoplasmic reticulum transmembrane helix translo	34912.64	31958.57	35782.3	37141.38	34949	32682.2	34941.97	38994.56	39937.86	36639	0.9539	0.43672
P17047	Lamp2	Lysosome-associated membrane glycoprotein 2	166839.9	178255.9	161855.2	166201.2	168288	156953.9	198238	192217.8	161400.8	177203	0.9497	0.45200
Q7TQI3	Otub1	Ubiquitin thioesterase OTUB1	100799	108549.2	73074.86	75776.28	89550	92247.52	93638.88	78852.54	68649.48	83347	1.0744	0.58270
Q9Z0F7	Sncg	Gamma-synuclein	1985348	1938463	1809421	1571614	1826212	2047704	1793413	1853016	1740902	1858759	0.9825	0.78550
O54991	Cntnap1	Contactin-associated protein 1	153682.9	145380.7	105874.3	154601.9	139885	135424.2	166567.9	93204.62	106468.9	125416	1.1154	0.49595
P60202	Plp1	Myelin proteolipid protein	851806.8	849052.1	651283.7	811561.9	790926	1003891	1345764	616389.6	627078.8	898281	0.8805	0.57395
Q9QXE0	Hacl1	2-hydroxyacyl-CoA lyase 1	4197.889	4299.561	7483.169	7625.243	5901	6493.931	5540.519	11158.33	8431.443	7906	0.7464	0.24747
P61979	Hnrnpk	Heterogeneous nuclear ribonucleoprotein K	351808.3	378729.1	416751.5	398313.4	386401	388387.2	377132.3	428658.5	437867.5	408011	0.9470	0.32936
Q6PHS9	Cacna2d2	Voltage-dependent calcium channel subunit alpha-2	5169.456	3282.256	5227.958	4590.809	4568	4390.176	10213.27	3609.036	4137.201	5587	0.8175	0.55103
O43790;P97861;Q1453	Krt86;;Krt81	Keratin, type II cuticular Hb6;;Keratin, type II cuticula	2801851	3193397	1422920	2796978	2553786	2304571	3357489	1416102	1445451	2130903	1.1985	0.50755
Q8K3W0	Babam2	BRISC and BRCA1-A complex member 2	26018.01	26616.18	24664.51	21220.07	24630	26217.13	25875.02	26519.19	25473.47	26021	0.9465	0.30054
Q9CQL5	Mrpl18	Large ribosomal subunit protein uL18m	16395.26	15049.29	17008.08	21572.06	17506	15107.68	16092.4	22629.49	22839.47	19167	0.9133	0.53231
Q80W54	Zmpste24	CAAX prenyl protease 1 homolog	48591.84	43562.94	56367.41	55467.12	50997	50334.37	49206.87	58396.86	56052.04	53498	0.9533	0.52982
Q2YDW2	Msto1	Protein misato homolog 1	11815.13	17275.65	15425.32	5668.452	12546	14281.67	9757.969	12512.79	13336.73	12472	1.0059	0.97935

O35945	Aldh1a7	Aldehyde dehydrogenase, cytosolic 1	96712.11	97370.13	66510.19	89347.88	87485	98414.78	95783	73117.12	76395.53	85928	1.0181	0.87799
Q6IRU5	Cltb	Clathrin light chain B	213230.4	187344.1	219362.1	198634.6	204643	207247.5	232002.5	191327.5	235828.9	216602	0.9448	0.38540
Q9WUD1	Stub1	E3 ubiquitin-protein ligase CHIP	105045.9	106752	101928.6	104047.9	104444	101550.6	112133.2	104949.7	106213.7	106212	0.9834	0.49335
P97450	Atp5pf	ATP synthase-coupling factor 6, mitochondrial	199217.3	180559.4	183527.2	192928	189058	195092.3	182764.8	187825.8	185891.6	187894	1.0062	0.82450
O54946	Dnajb6	DnaJ homolog subfamily B member 6	26267.74	23798.28	22427.95	17594.01	22522	24287	23149.16	21984.61	22704.62	23031	0.9779	0.79630
Q69ZK0	Prex1	Phosphatidylinositol 3,4,5-trisphosphate-dependent	13949.5	9577.837	8543.592	5037.162	9277	8281.799	4839.375	6888.651	9489.032	7375	1.2580	0.39771
Q8K2A8	Alg3	Dol-P-Man:Man(5)GlcNAc(2)-PP-Dol alpha-1,3-man	6669.094	6275.189	6583.238	4599.925	6032	8519.223	7621.461	5101.827	7653.821	7224	0.8350	0.22538
O88741	Gdap1	Ganglioside-induced differentiation-associated prot	27779.73	33581.81	29000.45	34667.2	31257	29364.4	31774.05	32233.96	30712.81	31021	1.0076	0.90025
Q80YT7	Pde4dip	Myomegalin	32952.92	36544.18	33168.5	36027.66	34673	36305.05	33662.89	42153.23	43663.32	38946	0.8903	0.14473
Q9QYI3	Dnajc7	DnaJ homolog subfamily C member 7	32233.45	30652.77	28753.21	29891.33	30383	31946.21	30709.22	29719.21	29897.61	30568	0.9939	0.84172
P25799	Nfkb1	Nuclear factor NF-kappa-B p105 subunit	13575.85	13926.62	15767.55	10077.87	13337	14333.11	12519.85	13619.04	14202.74	13669	0.9757	0.80081
Q5U3K5	Rabl6	Rab-like protein 6	34458.61	33769.23	29130.28	30695.41	32013	34434.56	35905.28	26303.3	24924.54	30392	1.0534	0.61536
Q61490	Alcam	CD166 antigen	56746.81	56282.66	61383.31	67889.27	60576	61575.11	55920.59	53515.47	56038.66	56762	1.0672	0.27716
Q8VE62	Paip1	Polyadenylate-binding protein-interacting protein 1	19026.18	19628.61	22455.6	21180.73	20573	23180.06	18713.63	14656.22	11261.14	16953	1.2135	0.22681
Q8C078	Camkk2	Calcium/calmodulin-dependent protein kinase kinas	373443.3				373443							
Q5EBJ4	Ermn	Ermin							12543.59			12544	0.0000	
Q7TPH6	Mycbp2	E3 ubiquitin-protein ligase MYCBP2	4097.45	4721.396	4773.727	3359.159	4238	3769.551	3199.115	3353.544	3729.507	3513	1.2064	0.09019
P15327	Bpgm	Bisphosphoglycerate mutase	15899.68	11685.17	21285.27	16286.67	16289	12928.43	8805.207	14738.56	11206.96	11920	1.3666	0.11060
Q8R035	Mrpl58	Large ribosomal subunit protein mL62	9313.829	11442.84	11959.59	9112.967	10457	11657.34	10551.88	13354.3	9660.898	11306	0.9249	0.46090
Q8VBT0	Tmx1	Thioredoxin-related transmembrane protein 1	27782.1	25569.19	31770.49	33801.29	29731	26950.32	27934.2	31824.39	30584.09	29323	1.0139	0.85814
Q61074	Ppm1g	Protein phosphatase 1G	46925.55	45808.85	52236.12	42490.38	46865	49464.44	46620.95	47849.45	48476.72	48103	0.9743	0.57865
Q91X52	Dcxr	L-xylulose reductase	73110.88	101168	86257.04	19283.69	69955	24257.89	104103		18877.28	49079	1.4253	0.53378
P26043	Rdx	Radixin	811014.1	761432.7	793045.1	731269.3	774190	774323.9	750615.8	710094	724551.6	739896	1.0463	0.18029
P62075	Timm13	Mitochondrial import inner membrane translocase s	81923.3	102256.8	86186.18	97618.83	91996	93091.2	95103.88	113885.7	109883.5	102991	0.8932	0.17052
Q61411	Hras	GTPase HRas	48167.18	75171.16	76818.13	105996.3	76538	58817.38	85142.33	85223.91	86632.21	78954	0.9694	0.86477
Q9CT10	Ranbp3	Ran-binding protein 3	25755.2	26993.45	27420.97	25242.53	26353	21661.21	17957.11	25778.22	25844.89	22810	1.1553	0.12047
Q9DB32	Haghl	Hydroxyacylglutathione hydrolase-like protein	18231.49	16042.88	12349.46	12970.25	14899	14948.87	15664.82	13353.39	13066.99	14259	1.0449	0.68627
Q9Z1B3	Plcb1	1-phosphatidylinositol 4,5-bisphosphate phosphodi	36958.82	36940.86	29424.79	37756.23	35270	34734.49	35037.45	32173.71	31334.17	33320	1.0585	0.40222
Q8K221	Arfip2	Arfaptin-2	7209.964	7365.336	9659.693		8078	5790.225	8788.744	7955.317	8610.45	7786	1.0375	0.79208
Q6A4J8	Usp7	Ubiquitin carboxyl-terminal hydrolase 7	54278.41	49842.13	50701.59	47121.57	50486	52337.66	50682.79	52881.63	50837.18	51685	0.9768	0.47521
P63325	Rps10	Small ribosomal subunit protein eS10	373884.9	361508.7	433105.1	422549.8	397762	343958.3	409421.5	514784.8	510751.9	444729	0.8944	0.33790
Q9QZD9	Eif3i	Eukaryotic translation initiation factor 3 subunit I	48976.71	49693.32	50946.43	55173.87	51198	54697.36	54800.02	64345.47	61737.88	58895	0.8693	0.03408
P60060	Sec61g	Protein transport protein Sec61 subunit gamma	136452.9	124975.6	109817.1	102871.7	118529	144976.5	142944.3	99537.59	111201.3	124665	0.9508	0.66936
Q8VDP6	Cdipt	CDP-diacylglycerol--inositol 3-phosphatidyltransfera	70825.52	76633.47	76131.54	81393.81	76246	78956.01	86710.33	83371.34	96005.31	86261	0.8839	0.05495
Q8JZY2	Commd10	COMM domain-containing protein 10	28235.77	27687.05	23907.39	27655.61	26871	33493.74	34915.45	22537.96	24880.8	28957	0.9280	0.54337
O70172	Pip4k2a	Phosphatidylinositol 5-phosphate 4-kinase type-2 al	55433.88	58070.51	56652.52	57137.77	56824	55304.46	55198.22	54609.21	70731.4	58961	0.9638	0.60926
P48722	Hspa4l	Heat shock 70 kDa protein 4L	186193.2	212709.1	190606.8	199613.5	197281	212448.5	190071.2	194814.5	209068.8	201601	0.9786	0.60757
Q9QYB8	Add2	Beta-adducin	161977.5	170882	148894.1	201128.4	170720	162407.8	173820.7	155570	159691	162872	1.0482	0.52953
Q9ER88	Dap3	Small ribosomal subunit protein mS29	17007.53	19859.38	20350.58	26726.02	20986	21209.85	23179.9	19668.6	21561.47	21405	0.9804	0.85345
Q9CR88	Mrps14	Small ribosomal subunit protein uS14m	12622.04	7036.376	2869.428	7340.447	7467	6812.648	6295.908	7668.344		6926	1.0782	0.82960
Q02819	Nucb1	Nucleobindin-1	95990.39	92790.52	99418.39	104275.8	98119	91281.89	99009.23	109628.5	110261.8	102545	0.9568	0.42534
P41241	Csk	Tyrosine-protein kinase CSK	76492.1	78523.91	70109	67001.25	73032	76634.95	75915.54	66457.17	64311.77	70830	1.0311	0.61608
P60521	Gabarapl2	Gamma-aminobutyric acid receptor-associated prot	45935.3	47174.45	36057.62	47847.43	44254	49569.37	44699.29	40293.39	37545.64	43027	1.0285	0.75863
O88643	Pak1	Serine/threonine-protein kinase PAK 1	55979.93	53382.72	49376.95	42825.41	50391	53403.57	51580.84	45360.73	47911.2	49564	1.0167	0.81520
Q9JIK5	Ddx21	Nucleolar RNA helicase 2	33544.8	27379.83	31393.28	36710.03	32257	28676.58	31812.74	40952.27	36712.52	34539	0.9339	0.51975
P97300	Nptn	Neuroplastin	189093.1	171365.8	190950.3	204031.2	188860	171696.3	185404.1	182174.4	169546.6	177205	1.0658	0.18353
Q8BWM0	Ptges2	Prostaglandin E synthase 2	31571.2	27813.19	30750.08	31869.98	30501	34669.08	29325.43	34707.4	35598.25	33575	0.9084	0.12170
Q61464	Znf638	Zinc finger protein 638	26360.51	16576.36	15106.55	12866.41	17727	15392.26	21776.47	3829.094	14658.49	13914	1.2741	0.45418
Q9DC16	Ergic1	Endoplasmic reticulum-Golgi intermediate compartr	64437.92	67870.91	74730.03	75097.37	70534	71541.84	73018.75	70231.27	75843.09	72659	0.9708	0.48968
Q80Y56	Rbsn	Rabenosyn-5	18671.92	15342.5	20442.76	11015.86	16368	16505.93	14842.43	17650.06	17255	16563	0.9882	0.93112
P24668	M6pr	Cation-dependent mannose-6-phosphate receptor	280539.3	263236.8	252689.7	295837.1	273076	264735.6	285385.4	288740.3	300662	284881	0.9586	0.36684
P62814	Atp6v1b2	V-type proton ATPase subunit B, brain isoform	318037.8	337205.8	292420.2	317158.2	316206	339424.8	342128.3	313231.9	296863.7	322912	0.9792	0.65376
Q08509	Eps8	Epidermal growth factor receptor kinase substrate 8			25776.82	11026.83	18402			8675.429		8675	2.1211	
O88520	Shoc2	Leucine-rich repeat protein SHOC-2	158818.5	128669.8	129834.6	158031.9	143839	161724.6	153228.9	165958.5	163383.8	161074	0.8930	0.09988
P16125	Ldhb	L-lactate dehydrogenase B chain	1362826	1318855	1080249	898137.3	1165017	1140520	1202727	911437.4	901625.6	1039078	1.1212	0.38149
Q8VBY2	Camkk1	Calcium/calmodulin-dependent protein kinase kinas	28439.69	48864.16	84519.91	30418.21	48060	24852.19	19070.68	9633.297	17551.16	17777	2.7035	0.06406
P23506	Pcmt1	Protein-L-isoaspartate(D-aspartate) O-methyltransfe	99556.55	94720.26	84779.86	95661.88	93680	106930.4	91289.24	86810.96	88398.21	93357	1.0035	0.95586
P12382	Pfkl	ATP-dependent 6-phosphofructokinase, liver type	117340.7	130223.4	107762.7	106207.3	115384	120190.3	123828.7	94837.62	95962.66	108705	1.0614	0.50813
Q91WK0	Lrrfip2	Leucine-rich repeat flightless-interacting protein 2	14441.89	12746.24	14175.89	16424.24	14447	13914.29	14179.92	15267.27	14956.13	14579	0.9909	0.87726

Q6PDS3	Sarm1	NAD(+) hydrolase SARM1	35287.55	40790.78	34798.54	33690.84	**36142**	34790.71	36483.43	26745.21	30402.05	**32105**	1.1257	0.18701
Q60875	Arhgef2	Rho guanine nucleotide exchange factor 2	4040.731	1868.503	4572.947	3769.655	**3563**	3095.387	2324.594	2150.765	4044.483	**2904**	1.2270	0.40162
E9Q557	Dsp	Desmoplakin	13074.56	11956.11	14521.04	18174.86	**14432**	18059.27	13241.87	11968.32	30145.12	**18354**	0.7863	0.40293
Q9D1C8	Vps28	Vacuolar protein sorting-associated protein 28 homo	22598.87	26283.59	24059.35	24065.97	**24252**	22742.51	23241.88	24147.24	28136.22	**24567**	0.9872	0.83425
Q9DBC3	Cmtr1	Cap-specific mRNA (nucleoside-2'-O-)-methyltransfe	15021.16	14183.75	13243.85	13195.24	**13911**	14802.34	12145.76	13985.64	12458.94	**13348**	1.0422	0.48962
Q80ZS3	Mrps26	Small ribosomal subunit protein mS26	13053.66	17362.11	21270.95	19837.19	**17881**	23774.32	19511.58	26694.6	18001.37	**21995**	0.8129	0.17577
Q80WQ2	Vac14	Protein VAC14 homolog	17226.48	23052.51	15593.54	11606.09	**16870**	15248.32	14869.15	16374.38	24090.48	**17646**	0.9560	0.81751
Q8BP71	Rbfox2	RNA binding protein fox-1 homolog 2	44836.78	43702.3	45201	45412.02	**44788**	48103.22	45143.37	46676.92	46518.81	**46611**	0.9609	0.04355
Q6IR34	Gpsm1	G-protein-signaling modulator 1	22509.25	24323.7	23262.65	22082.97	**23045**	24739.25	22022.61	19268.37	20373.31	**21601**	1.0668	0.30475
Q67BT3	Slc13a5	Na(+)/citrate cotransporter												
Q8CHP5	Pym1	Partner of Y14 and mago	12300.07	11469.99	14827.2	17192.6	**13947**	10414.63	13400.32	12809.44	15721.7	**13087**	1.0658	0.62933
P22907	Hmbs	Porphobilinogen deaminase	35026.14	35473.45	34761.5	34170.02	**34858**	32536.46	20227.1	29965.02	24948.14	**26919**	1.2949	0.02760
Q9CRC9	Gnpda2	Glucosamine-6-phosphate isomerase 2	60287.44	53572.98	47780.73	42257.3	**50975**	49943.05	55357.04	44522.86	39867.94	**47423**	1.0749	0.51373
O35405	Pld3	5'-3' exonuclease PLD3	77533.87	75511.79	79595.66	77618.93	**77565**	72521.09	87499.42	64940.05	81010.01	**76493**	1.0140	0.83704
Q6ZQ38	Cand1	Cullin-associated NEDD8-dissociated protein 1	276647.9	279574.9	250727.4	223950.4	**257725**	268927.4	259649.8	243883.6	242716	**253794**	1.0155	0.79486
Q80XE1	Ric8b	Synembryn-B	8225.781	8201.285	6622.742		**7683**	6431.228	6415.133		6197.297	**6348**	1.2104	0.06727
Q6A098	Secisbp2l	Selenocysteine insertion sequence-binding protein 2	10254.83	5344.947	3360.609	7318.008	**6570**	5881.492	5886.354	12410.2	14213.55	**9598**	0.6845	0.29266
Q8C052	Map1s	Microtubule-associated protein 1S	43552.94	42199.24	40681.98	38741.8	**41294**	41380.08	43938.86	36523.81	42570.29	**41103**	1.0046	0.92395
Q9Z218	Dpp6	Dipeptidyl aminopeptidase-like protein 6	31993.06	29684.63	28784.89	31489.37	**30488**	29677.06	31895.07	28923.7	30338.19	**30209**	1.0093	0.78584
Q9Z2M6	Ubl3	Ubiquitin-like protein 3	95079.57	109858.3	81728.71	101734.4	**97100**	98940.24	101321.9	80654.2	81523.62	**90610**	1.0716	0.45438
Q91YN0	D6Wsu163e	Protein C12orf4 homolog	20759.81	18092.69	17275.46	18603.03	**18683**	12993.09	17367.1	15795.37	19097.97	**16313**	1.1452	0.16400
Q8BYJ6	Tbc1d4	TBC1 domain family member 4	4764.278	8473.633	4387.394	4598.169	**5556**	5953.038	5043.273	1862.197	6533.855	**4848**	1.1460	0.63756
Q9DBP5	Cmpk1	UMP-CMP kinase	204104.7	226847.1	219696.4	154366.1	**201254**	201164	184364.1	196557.8	186007.9	**192023**	1.0481	0.60327
P08556	Nras	GTPase NRas	275353.5	251872.7	286170.3	300745.8	**278536**	277479.9	275313.3	281513.6	310489.3	**286199**	0.9732	0.58163
Q8JZN7	Rhot2	Mitochondrial Rho GTPase 2	24925	19840.21	23289.93	25056.49	**23278**	23356.08	23495.87	23988.5	24897.36	**23934**	0.9726	0.62188
Q8BG32	Psmd11	26S proteasome non-ATPase regulatory subunit 11	110195.2	114924	119789.7	115092.9	**115000**	111098.4	110443.3	118714.7	121616.5	**115468**	0.9959	0.89508
P99026	Psmb4	Proteasome subunit beta type-4	130197.9	132398.6	130377.1	112003.9	**126244**	119343.6	132979.4	124903.1	120399.9	**124407**	1.0148	0.75774
P62331	Arf6	ADP-ribosylation factor 6	136651.3	124255	120518.9	130897.7	**128081**	133737.4	136126.5	119797.3	132373.4	**130509**	0.9814	0.65152
Q9QYJ0	Dnaja2	DnaJ homolog subfamily A member 2	76936.77	83705.17	64456.61	59813.49	**71228**	67503.52	87128	66291.4	61800.76	**70681**	1.0077	0.94683
Q9R0A1	Clcn2	Chloride channel protein 2	19917.94	20811.43	13425.1	19095.01	**18312**	19058.45	18858.51	20844.27	19409.04	**19543**	0.9371	0.50269
Q9Z2L7	Crlf3	Cytokine receptor-like factor 3	3772.123	5689.809	1591.54		**3684**	2546.9	3464.781	1.34914		**2004**	1.8383	0.34568
Q64152	Btf3	Transcription factor BTF3	86694.62	85844.08	96324.55	92479.02	**90336**	98161.81	89193.92	114417.2	112610.5	**103596**	0.8720	0.08798
Q91ZA3	Pcca	Propionyl-CoA carboxylase alpha chain, mitochondri	60905.37	53116.73	56982.73	64245.38	**58813**	59476.41	55729.95	52642.58	57732.3	**56395**	1.0429	0.42440
Q9JIA7	Sphk2	Sphingosine kinase 2	24188.74	24042.59	24695.07	21607.4	**23633**	24460.65	23972.3	22272.36	17453.52	**22040**	1.0723	0.39538
P49615	Cdk5	Cyclin-dependent kinase 5	91155.79	94481.91	91203.4	81336.63	**89544**	93566.56	85702.09	90648.66	81539.88	**87864**	1.0191	0.68126
Q9DBE0	Csad	Cysteine sulfinic acid decarboxylase	21047.46	19953.2	18732.41	17464.61	**19299**	16508.6	19449.7	16647.39	16565.87	**17293**	1.1160	0.10617
Q6PFD9	Nup98	Nuclear pore complex protein Nup98-Nup96	29596.77	32442.5	30536.37	34767.78	**31836**	30008.69	29679.67	29341.64	34260.91	**30823**	1.0329	0.55570
P18572	Bsg	Basigin	191537.8	169311.4	189395	184338.4	**183646**	161760.6	175835.6	180561.9	177189.5	**173837**	1.0564	0.18224
Q9WUA6	Akt3	RAC-gamma serine/threonine-protein kinase	14246.98	13235.66	13791.95	12111.51	**13347**	14194.83	15456.58	11765.03	12126.75	**13386**	0.9971	0.96957
Q8CJF9	Ago3	Protein argonaute-3	27492.41	26986.86	30952.05	27127.91	**28140**	28161.47	28220.21	29387.64	29436.43	**28801**	0.9770	0.53564
Q9ESP1	Sdf2l1	Stromal cell-derived factor 2-like protein 1	35606.05	48104.35	42705.18	42174.33	**42147**	36575.14	40584.18	36101.17	41728.5	**38747**	1.0878	0.28898
Q8BH86	Dglucy	D-glutamate cyclase, mitochondrial	13012.11	15408.54	14555.68	15944.5	**14730**	13732.8	16467.38	11371.89	15754.23	**14332**	1.0278	0.77132
Q8R4N0	Clybl	Citramalyl-CoA lyase, mitochondrial	51347.62	45015.45	43801.17	60105.87	**50068**	46249.48	49674.24	48485.42	49028.08	**48359**	1.0353	0.66933
Q9Z0R9	Fads2	Acyl-CoA 6-desaturase	23731.45	25824.97	26761.92	27537.35	**25964**	26309.95	27057.36	27384.12	28710.6	**27366**	0.9488	0.19590
Q6ZPS2	Carns1	Carnosine synthase 1	7893.546	10216.66	11075.59		**9729**	8456.908		8580.834	16082.29	**11040**	0.8812	0.65195
Q80YV4	Pank4	4'-phosphopantetheine phosphatase	17425.99	20852.03	17238.32	14536.23	**17513**	17901.37	18791.72	15433.57	14112.58	**16560**	1.0576	0.59238
Q9Z2C4	Mtmr1	Myotubularin-related protein 1	12382.69	11704.79	10008.75	11743.31	**11460**	10486.88	9295.994	10188.66	11831.8	**10451**	1.0966	0.21657
Q9EQJ9	Magi3	Membrane-associated guanylate kinase, WW and PD	21936.53	19549.75	16971.29	17859.6	**19079**	24290.53	17861.89	18310.62	23133.32	**20899**	0.9129	0.39203
Q6ZWV3	Rpl10	Large ribosomal subunit protein uL16	198328.8	200816.8	209602.2	212232.4	**205245**	192100.1	198996.6	205539.6	217363.1	**203500**	1.0086	0.79224
Q6DFW4	Nop58	Nucleolar protein 58	24900.77	25622.54	28004.25	26416.1	**26236**	25905.95	24053.66	23496.18	27124.04	**25145**	1.0434	0.34701
Q9CY16	Mrps28	Small ribosomal subunit protein bS1m	16809.63	17774.85	19232.39	19908.1	**18431**	16819.74	18021.89	24740.01	19555.87	**19784**	0.9316	0.49863
Q91VM9	Ppa2	Inorganic pyrophosphatase 2, mitochondrial	82809.34	78183.52	66385.42	72316.27	**74924**	69541.64	71144.66	64302.02	65805.27	**67698**	1.1067	0.11368
P51432	Plcb3	1-phosphatidylinositol 4,5-bisphosphate phosphodi	117455.7	120057	110427.7	120161	**117025**	128456.4	118200	120270.1	120259	**121796**	0.9608	0.18939
Q8VE99	Ccdc115	Coiled-coil domain-containing protein 115		14392.77	9144.291	11374.67	**11637**	3602.967	8606.415		2196.015	**4802**	2.4235	0.05041
Q91W34	Rusf1	RUS family member 1	9272.316	7828.451	8232.989	5614.759	**7737**	6049.903	8814.024	6029.399	6810.896	**6926**	1.1171	0.45299
Q06138	Cab39	Calcium-binding protein 39	44633.75	48030.52	34130.88	30488.5	**39321**	41365.1	43840.08	33482.09	33613.19	**38075**	1.0327	0.80971
Q8C0D5	Efl1	Elongation factor-like GTPase 1	16403.29	15706.35	17540.53	16021.67	**16418**	17766.52	15036.02	12644.99	17221.05	**15667**	1.0479	0.56525
Q64522	H2ac21	Histone H2A type 2-B	153015.8	164179.1	191224.3	209715	**179534**	146258.8	149691	239400.4	224795.8	**190037**	0.9447	0.71716
A2APX8	Scn1a	Sodium channel protein type 1 subunit alpha	36975.94	78916.7		50673.15	**55522**	54803.49	54110.16	72736.53	47768.66	**57355**	0.9680	0.88577

Q9CQ49	Ncbp2	Nuclear cap-binding protein subunit 2	47128.86	48813.61	44933.84	51595.42	48118	48967.56	47478.03		46605.18	47684	1.0091	0.81487
Q9Z2W0	Dnpep	Aspartyl aminopeptidase	103718.3	106279.5	113590.7	91925.01	103878	99338.56	101694	111953.4	107222.2	105052	0.9888	0.83262
Q3U0M1	Trappc9	Trafficking protein particle complex subunit 9	7164.926	7873.248	4845.686	6136.279	6505	5702.353	5744.271	7327.21	7313.219	6522	0.9974	0.98407
Q8CE50	Snx30	Sorting nexin-30	12099.68	10645.48	11454.37	12569.83	11692	18151.38		11012.24		14582	0.8018	0.27267
P11930	Nudt19	Acyl-coenzyme A diphosphatase NUDT19	15639.16	14224.73	16280	15401.28	15386	15262.76	22130.22	22472.74	20564.59	20108	0.7652	0.03365
P61028	Rab8b	Ras-related protein Rab-8B	78284.85	64253.77	69200.78	63242	68745	68750.23	72797.37	88319.82	88426.49	79573	0.8639	0.13075
Q9Z140	Cpne6	Copine-6	360027.3	356058.2	274581.8	319742.6	327602	335553.5	370920.5	279948.8	262658.3	312270	1.0491	0.64794
P58158	B3gat3	Galactosylgalactosylxylosylprotein 3-beta-glucuronc	68413.9	63729.04	72999.85	71203.71	69087	72970.36	69443.67	75614.63	81423.4	74863	0.9228	0.12432
Q9D6Y7	Msra	Mitochondrial peptide methionine sulfoxide reductas	24531.11	21915.99	15873.66	19002.42	20331	17293.95	16601.99	16326.74	16001.78	16556	1.2280	0.09227
Q91V01	Lpcat3	Lysophospholipid acyltransferase 5	34721.64	31926.65	29316.76	29952.12	31479	33992.7	28729.27	40548.19	39056.16	35582	0.8847	0.21279
O08915	Aip	AH receptor-interacting protein	65127.33	59570.23	64272.07	55496.78	61117	65316.09	62033.38	72331.93	61965.69	65412	0.9343	0.24164
Q920Q6	Msi2	RNA-binding protein Musashi homolog 2	17240.36	18524.86	17505.51	18601.45	17968	19114.3	18195.47	19624.44	20119.33	19263	0.9328	0.05291
P70340	Smad1	Mothers against decapentaplegic homolog 1	25185.26	29441.38	27833.44	26272.6	27183	26425.5	26362.76	23994.5	24836.07	25405	1.0700	0.15819
Q05D44	Eif5b	Eukaryotic translation initiation factor 5B	30513.53	36773.43	34852.34	30994.27	33283	31143.12	31312.2	27604.97	26244.89	29076	1.1447	0.07767
P12849	Prkar1b	cAMP-dependent protein kinase type I-beta regulato	42785.82	47098.2	42523.67	44965.93	44343	31814.71	52262.51	41993.27	31554.86	39406	1.1253	0.36523
Q8R1I1	Uqcr10	Cytochrome b-c1 complex subunit 9	287658.4	337969.8	256076.8	289268.3	292743	255259.9	326119	261749.9	254612.2	274435	1.0667	0.47776
Q9CR26	Vta1	Vacuolar protein sorting-associated protein VTA1 ho	132478.8	141510.9	108190.1	95544.2	119431	124262.9	116790.3	107313.9	94085.55	110613	1.0797	0.50571
Q9CQW2	Arl8b	ADP-ribosylation factor-like protein 8B	109119	108894.4	113763.2	114623.6	111600	106875.6	103097.4	91086.76	112234.8	103324	1.0801	0.13111
P62889	Rpl30	Large ribosomal subunit protein eL30	454084.8	442971.8	481880.3	519602.8	474635	472814	490040.1	535001.9	539070	509231	0.9321	0.19488
Q80U72	Scrib	Protein scribble homolog	16435.51	16462.95	16962.83	19353.98	17304	15487.04	14822.65	17205.75	19918.06	16858	1.0264	0.74943
Q923B0	Ggact	Gamma-glutamylaminecyclotransferase	31654.1	30563.47	33162.79	25240.21	30155	31642.46	27141.11	30199.63	31127.27	30028	1.0042	0.95113
Q80U87	Usp8	Ubiquitin carboxyl-terminal hydrolase 8	31409.46	28333.21	29602.63	31318.51	30166	25653.17	27534.93	29059.71	31817.1	28516	1.0579	0.31277
Q91ZX7	Lrp1	Prolow-density lipoprotein receptor-related protein 1	72090.61	69082.27	82336.2	75004.23	74628	74377.74	70152.83	80090.68	81180.22	76450	0.9762	0.65137
P21279	Gnaq	Guanine nucleotide-binding protein G(q) subunit alp	645384.6	667132	555340.8	760339.6	657049	691117.3	748842.1	617200.4	638589.1	673937	0.9749	0.75336
Q8R4R6	Nup35	Nucleoporin NUP35	12732.39	12547.09	14103.79	15073.09	13614	12496.34	13498.7	17738.11	17007.05	15185	0.8965	0.31095
P61021	Rab5b	Ras-related protein Rab-5B	466148	435243.2	441858.3	458839.7	450522	462114.9	440744.6	438889.1	445744.1	446873	1.0082	0.69696
Q8R1H0	Hopx	Homeodomain-only protein	8526.033	9477.158	4111.471		7372		7607.751			7608	0.9690	
Q6NTA4	Rragb	Ras-related GTP-binding protein B	82252.39	81937.97	81581.48	74211.55	79996	84194.02	81901.96	70593.01	78433.67	78781	1.0154	0.74364
Q9CW79	Golga1	Golgin subfamily A member 1	11287.58	10413.85	11842.26	11308.55	11213	9686.139	11244.51	10211.7	11860.05	10751	1.0430	0.45084
Q8C0T5	Sipa1l1	Signal-induced proliferation-associated 1-like protei	11171.75	9412.27	8783.588	12229.84	10399	8246.16	9166.618	5934.524	9139.838	8122	1.2804	0.08333
Q99KV1	Dnajb11	DnaJ homolog subfamily B member 11	57618.31	61015.71	68346.66	65507.36	63122	62835.25	65051.62	61984.48	71831.08	65426	0.9648	0.50615
Q8BG26	Rusc1	AP-4 complex accessory subunit RUSC1	8070.776	9783.502	13735.96	12737.78	11082	13775.65	9818.66	13144.71	12108.13	12212	0.9075	0.49892
Q8C5Q4	Grsf1	G-rich sequence factor 1	23724.44	25715.23	23323.06	24912.22	24419	26963.25	25965.19	25334.64	25010.76	25818	0.9458	0.09129
P0C192	Lrrc4b	Leucine-rich repeat-containing protein 4B												
Q8VHR5	Gatad2b	Transcriptional repressor p66-beta	22506.28	19321.54	25027.32	24771.14	22907	19640.06	19510.25	27440.74	25939.49	23133	0.9902	0.92983
Q9CYH2	Prxl2a	Peroxiredoxin-like 2A	187679.5	186792.6	187313.5	207012.1	192199	193589.8	197425.8	194046.9	203866.1	197232	0.9745	0.39387
Q8C8T8	Tsr2	Pre-rRNA-processing protein TSR2 homolog	22194.58	32876.86	14069.93	23215.29	23089	24389.41	29954.62	19772.42	21149.97	23817	0.9695	0.87598
P47941	Crkl	Crk-like protein	95597.2	89826.64	91695.04	86980.36	91025	96443.73	90379.8	89766.13	92980.19	92392	0.9852	0.58339
P23198	Cbx3	Chromobox protein homolog 3	51104.39	46179.26	57408.36	52006.14	51675	50160.43	48796.87	52780.95	55527.84	51817	0.9973	0.96035
Q9WU28	Pfdn5	Prefoldin subunit 5	16769.9	16355.49	13294.7	9472.385	13973	12692.64	13923.78	13029.27	11346.03	12748	1.0961	0.51496
P61087	Ube2k	Ubiquitin-conjugating enzyme E2 K	86974.8	99085.61	71171.05	67358.91	81148	84220.43	79423.81	64869.87	60958.02	72368	1.1213	0.37818
Q9QUR8	Sema7a	Semaphorin-7A	25571.88	18751.38	17450.22	22178.67	20988	21386.41	21626.53	18166.74	18840.58	20005	1.0491	0.64464
Q9Z2W9	Gria3	Glutamate receptor 3			7217.925		7218		10939.04	40402.08	9057.084	20133	0.3585	
P61205;P84078	Arf3;Arf1	ADP-ribosylation factor 3;ADP-ribosylation factor 1	756565.3	761014.8	806454.6	687697.8	752933	804328.8	744293.2	773753.4	698444.8	755205	0.9970	0.94780
Q91VM3	Wdr45	WD repeat domain phosphoinositide-interacting pro	29215.41	29723.98	29341.33	26756.67	28759	30640.47	29414.78	23092.17	30051.21	28300	1.0162	0.81494
Q9DCJ1	Mlst8	Target of rapamycin complex subunit LST8	16484.47	14975.14	15960.65	16505.06	15981	15753.66	15931.42	12790.25	16702.21	15294	1.0449	0.48861
O08582	Gtpbp1	GTP-binding protein 1	15670.04	16190.97	16291.65	19455.36	16902	17337.07	17545.75	14426.82	17065.35	16594	1.0186	0.79396
Q8BFQ8	Gatd1	Glutamine amidotransferase-like class 1 domain-co	46599.23	42527.89	41903.39	38879.93	42478	44646.84	43858.59	47833.8	43732.25	45018	0.9436	0.22008
Q91YJ2	Snx4	Sorting nexin-4	24466.92	23946.82	30697.85	21496.12	25152	24531.9	22518.97	22436.16	25777.65	23816	1.0561	0.55208
P0C605	Prkg1	cGMP-dependent protein kinase 1	37931.73	44668.48	40731	33492.58	39206	41921.33	41259.09	41490.3	35034.94	39926	0.9820	0.80990
Q8VHK5	Mlc1	Membrane protein MLC1		4458.802		802.6813	2631	19020.06				19020	0.1383	
Q8VDP2	Steep1	STING ER exit protein	9958.021	9176.246	10339.42	9451.665	9731	11186.37	6911.361	11538.82	11509.33	10286	0.9460	0.64844
Q8CFI2	Cdc34	Ubiquitin-conjugating enzyme E2 R1	10531.89	10170.89	10419.67	7919.264	9760	11321.81	8423.267	5537.97	7417.783	8175	1.1939	0.28687
P70704	Atp8a1	Phospholipid-transporting ATPase IA	52583.08	56426.55	52411.29	56207.87	54407	51380	54332.52	49310.39	52617.31	51910	1.0481	0.15346
Q9JII5	Dazap1	DAZ-associated protein 1	103540.7	99487.13	113461.8	102805.9	104824	106122.2	113377.9	126876.3	123646.4	117506	0.8921	0.06537
Q60604	Scin	Scinderin	11106.73	8741.603	4834.825	4838.947	7381	9501.591	5516.526	6835.813	8501.37	7589	0.9726	0.91067
Q8CJH3	Plxnb1	Plexin-B1	27993.15	24904.31	23716.81	29352.55	26492	23233.01	24524	20537.75	26838.9	23783	1.1139	0.19500
Q60749	Khdrbs1	KH domain-containing, RNA-binding, signal transduc	76698.3	72387.86	75121.91	74541.57	74687	72176.12	76070.95	86170.8	74390.63	77202	0.9674	0.46452
Q3U1F9	Pag1	Phosphoprotein associated with glycosphingolipid-e	18786.88	18927.48	15941	19376.54	18258	20082.44	16733.34	4612.661	19683.28	15278	1.1951	0.45317

P47802	Mtx1	Metaxin-1	43607.81	39892.44	45477.56	48587.23	44391	44336.33	44840.3	45143.22	44003.1	44581	0.9957	0.92113
Q8BJU0	Sgta	Small glutamine-rich tetratricopeptide repeat-contai	59711.32	65967.73	62487.12	53161.51	60332	67689.95	65164.56	65279.48	57090.31	63806	0.9456	0.36729
Q8VE80	Thoc3	THO complex subunit 3	18815.98	16232.42	18952.13	13049.72	16763	12257.83	13981.12	15234.37	15664.72	14285	1.1735	0.16860
Q8VEH8	Erlec1	Endoplasmic reticulum lectin 1	26590.97	23815.41	22668.01	26694.36	24942	22879.52	28681.32	29934.01	26076.48	26893	0.9275	0.33435
Q91YR7	Prpf6	Pre-mRNA-processing factor 6	12925.69	16229.6	16628.46	19984.04	16442	14517.63	15617.48	21785.53	22006.99	18482	0.8896	0.43760
P84104	Srsf3	Serine/arginine-rich splicing factor 3	371640.4	424832.3	445438.1	503827.3	436435	438167.3	441974.8	551299.9	531200.1	490661	0.8895	0.22615
Q8R238	Sdsl	Serine dehydratase-like						2771.044				2771	0.0000	
Q60865	Caprin1	Caprin-1	185280.8	178635.3	195610.8	212183.4	192928	205575.7	204769.9	228504	240669.7	219880	0.8774	0.05716
Q9Z2P8	Vamp5	Vesicle-associated membrane protein 5	64035.41	64195.04	61084.07	57236	61638	59651.21	64461.41	54803.27	63357.02	60568	1.0177	0.70810
Q60809	Cnot7	CCR4-NOT transcription complex subunit 7	9912.509	9136.704	9231.206		9427	7575.748	10061.51	10026.89	11566.77	9808	0.9612	0.71852
Q9R1V4	Adam11	Disintegrin and metalloproteinase domain-containin	39078.49	42668.11	31649.7	41258.46	38664	35763.1	44290.73	33187.05	32695.78	36484	1.0597	0.57102
O35295	Purb	Transcriptional activator protein Pur-beta	130369	140142.8	172196.8	162532.8	151310	148025.8	136008.7	208399.3	202446.9	173720	0.8710	0.32458
Q99KR7	Ppif	Peptidyl-prolyl cis-trans isomerase F, mitochondrial	17068.09	13405.52	15595.33	14564.39	15158	15044.93	14525.23	14899.95	16465.25	15234	0.9950	0.93488
Q91XU0	Wrnip1	ATPase WRNIP1	9058.312	13044.42	8880.503	9431.052	10104	11033.87		8473.871	9629.662	9712	1.0403	0.77922
P15532	Nme1	Nucleoside diphosphate kinase A	137945.1	126231.2	94400.43	92846.56	112856	105075.1	127337.4	78148.17	85315.47	98969	1.1403	0.41441
P56399	Usp5	Ubiquitin carboxyl-terminal hydrolase 5	208306.7	220214.3	192860.3	170038.2	197855	205473.3	215680.9	181251.5	169706	193028	1.0250	0.76100
Q99LM2	Cdk5rap3	CDK5 regulatory subunit-associated protein 3	24042.92	22839.47	25273.95	23640.39	23949	20581.99	21919.47	20365.64	26657.4	22381	1.0701	0.35121
Q8R001	Mapre2	Microtubule-associated protein RP/EB family membe	74000.55	79687.57	70252.71	72619.04	74140	69090.05	89368.43	71236.53	69846.72	74885	0.9900	0.89165
Q04519	Smpd1	Sphingomyelin phosphodiesterase	24642.96	26720.17	26758.73	22196.01	25079	27054.7	24813.57	22257.69	24000.97	24532	1.0223	0.72216
Q04207	Rela	Transcription factor p65	58514.86	59331.59	69882.15	43705.39	57858	51408.76	56006.38	31903.68	64218.86	50884	1.1371	0.45424
Q9D8P4	Mrpl17	Large ribosomal subunit protein bL17m	27005.98	13243.82	26303.64	25632.79	23047	19309.48	29922.32	24192.38	30682.17	26027	0.8855	0.50723
P40630	Tfam	Transcription factor A, mitochondrial	28002.29	22600.11	23232.39	32474.57	26577	25803.28	25913.87	30504.59	24561.59	26696	0.9956	0.96579
Q9JIW9	Ralb	Ras-related protein Ral-B	17124.61	19177.22	19176.36	24236.35	19929	21463.5	17907.94	22168.5	26273.53	21953	0.9078	0.41041
Q8JZN5	Acad9	Complex I assembly factor ACAD9, mitochondrial	68279	66515.52	70454.45	75266.53	70129	65294.77	79543.33	71764.48	70491.2	71773	0.9771	0.65500
Q8BZF2	Nipal4	Magnesium transporter NIPA4												
P31938	Map2k1	Dual specificity mitogen-activated protein kinase kin	84604.04	83585.49	75103.25	63929.4	76806	85131.55	72870.75	79703.47	64203.16	75477	1.0176	0.84682
Q6PAK3	Prmt8	Protein arginine N-methyltransferase 8	17291.48	18828.1	15646.04	15548.31	16828	18215	20427.84	25294.18	19697.11	20909	0.8049	0.05520
Q08288	Lyar	Cell growth-regulating nucleolar protein	16594.34	13287.62	19343.62	14828.1	16013	14982.86	14498.78	13718.74	16520.2	14930	1.0726	0.47663
Q9R0Y5	Ak1	Adenylate kinase isoenzyme 1	273684.1	339554.3	290534	226684.4	282614	271640.4	271869.8	228215.8	223397.4	248781	1.1360	0.25405
Q9EPC2	Fgf23	Fibroblast growth factor 23												
Q9Z1F9	Uba2	SUMO-activating enzyme subunit 2	21329	24253.41	21520.48	14838.97	20485	17895.38	21177.34	19557.17	17875.51	19126	1.0711	0.55013
Q9CX00	Ist1	IST1 homolog	48471.38	53847.63	45599.3	51567.79	49872	46960.51	53697.34	39655.21	42369.36	45671	1.0920	0.28263
P08207	S100a10	Protein S100-A10	507953.2	487513.9	481553.3	604426.2	520362	560997.7	454480.2	495415.9	536757.7	511913	1.0165	0.82683
Q8BWU8	Etnppl	Ethanolamine-phosphate phospho-lyase	6420.544	7670.035			7045							
Q921L5	Cog2	Conserved oligomeric Golgi complex subunit 2	23620.33	24565.43	24035.83	21883.52	23526	23732.97	23281.48	23002.55	25641.31	23915	0.9838	0.65694
Q8JZV9	Bdh2	Dehydrogenase/reductase SDR family member 6	21167.46		17627.3	21795.03	20197	17830.96	15305.73	11221.33		14786	1.3659	0.08023
Q8C0L9	Gpcpd1	Glycerophosphocholine phosphodiesterase GPCPD1	21718.36	24079.44	20150.14	18409.15	21089	20011.86	24638.66	31397.31	10541.72	21647	0.9742	0.90619
P02463	Col4a1	Collagen alpha-1(IV) chain	1697568	1621832	1132560	2103712	1638918	1674837	1858842	1313493	1514881	1590513	1.0304	0.84064
O08677	Kng1	Kininogen-1	98043.08	99864.67	96133.84	50052.81	86024	77893.99	60570.7	57819.8	44648.63	60233	1.4282	0.11133
Q9D1M4	Eef1e1	Eukaryotic translation elongation factor 1 epsilon-1	49632.64	54994.04	50948.15	55630.07	52801	58082.82	57414.88	48105.58	59120.89	55681	0.9483	0.36634
Q8BVL3	Snx17	Sorting nexin-17	22860.33	22853.43	22351.79	23610.65	22919	22887.76	23863.14	23012.75	24353.03	23529	0.9741	0.21080
Q3TDK6	Rogdi	Protein rogdi homolog	23182.41	22629.02	19339.46	19199.49	21088	17031.29	21761.11	21729.05	18629.27	19788	1.0657	0.44235
Q8VDD5	Myh9	Myosin-9	282558.3	294116.6	305431.3	286486.5	292148	275524.3	277459.5	291159.8	285367.7	282378	1.0346	0.16624
Q8BLY2	Tars3	Threonine--tRNA ligase 2, cytoplasmic	237417.3	234135.3	239913.3	261745.4	243303	239493.8	271452.7	253214	273217.6	259345	0.9381	0.16577
P19258	Mpv17	Protein Mpv17	22981.7	21986.58	26992.46	26849.88	24703	21973.63	23461.81	29432.09	26908.85	25444	0.9709	0.73914
O08688	Capn5	Calpain-5	65369.27	73051.47	67039.39	85666.41	72782	64415.86	77268.7	66362.43	64638.99	68171	1.0676	0.43619
Q3TC33	Ccdc127	Coiled-coil domain-containing protein 127	6359.252	5631.876	9286.308	10402.6	7920	7428.582	7647.24	9167.979	8876.013	8280	0.9565	0.77857
O08848	RO60	RNA-binding protein Ro60	20637.89	23835.39	22599.76	20224.75	21824	21246.31	20746.79	19932.2	22484.44	21102	1.0342	0.49811
Q9D4F2	Plpp6	Polyisoprenoid diphosphate/phosphate phosphohyd	5372.783	5358.978		3855.293	4862	893.3432	5463.546		4045.511	3467	1.4023	0.38798
Q3UHD2	Gfod1	Glucose-fructose oxidoreductase domain-containing	24823.92	24070.8	24616.15	25267.58	24695	25190.97	23831.1	22048.87	22679.24	23438	1.0536	0.13782
O54692	Zw10	Centromere/kinetochore protein zw10 homolog	16650.59	14518.56	18538.36	19286.95	17249	16488.9	15882.58	19204.56	22037.96	18403	0.9372	0.53778
Q9D8Y1	Tmem126a	Transmembrane protein 126A	18129.85	14560.41	14759.6	22171.24	17405	43737.44	20884.29	18332.09	14931.32	24471	0.7113	0.33727
Q9QXL2	Kif21a	Kinesin-like protein KIF21A	169709.1	176039.8	159406.3	159273.5	166107	180593.1	162407.2	171965.6	165653.7	170155	0.9762	0.50734
O70325	Gpx4	Phospholipid hydroperoxide glutathione peroxidase	131006.5	133935.8	95754.41	113442.6	118535	121115.5	118970.6	96540.32	83991.58	105154	1.1272	0.32915
Q6PAM1	Txlna	Alpha-taxilin	22275.79	23678.29	20639.82	21754.87	22087	11423.3	13079.33	12607.32	11391.15	12125	1.8216	0.00001
Q9DBT5	Ampd2	AMP deaminase 2	123692.7	128240.4	105927.7	107585.6	116362	109995.1	129000.2	85977.8	106730.9	107926	1.0782	0.45064
Q91WK2	Eif3h	Eukaryotic translation initiation factor 3 subunit H	61600.64	63722.54	56884.95	57438.21	59912	59794.95	69086.73	67364.18	65664.96	65478	0.9150	0.07668
Q91WK5	Gcsh	Glycine cleavage system H protein, mitochondrial	35090.43	21602.2	15657.74	18276.87	22657	17019.79	38657.58	14710.47	14602.43	21248	1.0663	0.85241
P0DN34	Ndufb1	NADH dehydrogenase [ubiquinone] 1 beta subcomp	25632.31	14095.74	15657.01	19389.04	18694	13939.59	16876.53	8630.688	18047.12	14373	1.3006	0.24036

P25444	Rps2	Small ribosomal subunit protein uS5	644824.3	612896.6	669207.1	700220.4	656787	667741.4	656966.8	752579.6	765117.1	710601	0.9243	0.16050
O89017	Lgmn	Legumain	55371.33	58673.43	65901.56	60297.15	60061	63986.73	64609.09	64739.89	64979.11	64579	0.9300	0.08696
Q91XE4	Acy3	N-acyl-aromatic-L-amino acid amidohydrolase (carb	25793.82	33600.95	22037.77	18099.02	24883	25611.26	19405.02	13375.58	16752.26	18786	1.3245	0.19648
Q9JKK1	Stx6	Syntaxin-6	11275.27	10719.31	12645.22	8962.722	10901	8439.527	10348.96	10876.3	9324.633	9747	1.1183	0.26368
Q9JI46	Nudt3	Diphosphoinositol polyphosphate phosphohydrolas	104659.8	110045.5	94468.01	74640.74	95954	102916.6	91793.54	65825.14	74548.13	83771	1.1454	0.32765
Q9CQR2	Rps21	Small ribosomal subunit protein eS21	227089.8	247715.6	266550.4	280809.3	255541	248579.3	242293.1	317126.4	311929.7	279982	0.9127	0.33194
Q5RJG7	Crppa	D-ribitol-5-phosphate cytidylyltransferase							19831.87			19832	0.0000	
Q9EQC5	Scyl1	N-terminal kinase-like protein	21179.79	19203.4	21683.08	28586.43	22663	24046.25	22995.5	19608.8	25264.04	22979	0.9863	0.89885
Q9CZU6	Cs	Citrate synthase, mitochondrial	1011277	1094538	1062657	1267038	1108878	1154576	1215322	1100725	1212268	1170723	0.9472	0.35524
Q7TSS2	Ube2q1	Ubiquitin-conjugating enzyme E2 Q1	19299.77	24839.66	17847.79		20662	29814.36	22043.13			25929	0.7969	0.27893
Q62318	Trim28	Transcription intermediary factor 1-beta	93778.15	94351.4	110669.6	99219.73	99505	101830.2	95267.67	108615.4	101835.8	101887	0.9766	0.63530
Q9CQX2	Cyb5b	Cytochrome b5 type B	86301.53	85899.66	95401.48	99579.59	91796	87716.33	91000.92	91632.51	106030.1	94095	0.9756	0.67967
Q9R112	Sqor	Sulfide:quinone oxidoreductase, mitochondrial	50756.28	46960.85	46388.02	53576.37	49420	51765.56	48903.86	41465.34	42147.83	46071	1.0727	0.31365
D3Z7P3	Gls	Glutaminase kidney isoform, mitochondrial	162355.4	178875.9	189430	189028.6	179922	184350	192254.1	192771.5	199939.4	192329	0.9355	0.13114
Q9Z1E4	Gys1	Glycogen [starch] synthase, muscle	19665.84	15019.67	19473.67	16497.54	17664	12875.11	16330.3	13240.74	14369.98	14204	1.2436	0.04615
Q6P8I4	Pcnp	PEST proteolytic signal-containing nuclear protein	38623.13	19398.58	38437.3	15404.93	27966	18271.1	16389.8	31922.78	32595.63	24795	1.1279	0.68808
P67984	Rpl22	Large ribosomal subunit protein eL22	1092288	1046451	1352061	1436488	1231822	1260996	1000405	1436637	1459796	1289459	0.9553	0.70078
E9PXF8	Sbf2	Myotubularin-related protein 13	32204.98	29186.51	29417.48	29513.34	30081	29926.01	25119.23	31705.12	26541.69	28323	1.0621	0.33348
O35864	Cops5	COP9 signalosome complex subunit 5	94911.6	104581.7	92019.16	84288.76	93950	82259.46	90222.83	81791.51	84692.04	84741	1.1087	0.09319
Q9CQQ8	Lsm7	U6 snRNA-associated Sm-like protein LSm7	38847.95	31725.17	37275.39	38590.31	36610	42732.72	38309.99	35003.9	39620.81	38917	0.9407	0.35625
B0F2B4	Nlgn4l	Neuroligin 4-like												
Q6P9S0	Mtss2	Protein MTSS 2	17138.52	18102.45	18961.38	21220.87	18856	19053.42	20662.19	17649.7	22072.52	19859	0.9495	0.46852
Q6GQT1	A2m	Alpha-2-macroglobulin-P	44820.64	44441.37	43747.78	31416.08	41106	44311.19	41467.93	40197.77	41827.93	41951	0.9799	0.80935
Q91YI0	Asl	Argininosuccinate lyase	43198.11	49871.11	45033.75	39488.42	44398	41567.52	42143.95	37516.61	36584.05	39453	1.1253	0.10327
P43277	H1-3	Histone H1.3	3688931	2912975	3620687	3590256	3453212	2986926	3009350	3855753	3682643	3383668	1.0206	0.81800
O89053	Coro1a	Coronin-1A	166799.9	153670.5	131977	135306	146938	146743.9	146640	136874.3	129853.6	140028	1.0494	0.47798
Q6P6M7	Sepsecs	O-phosphoseryl-tRNA(Sec) selenium transferase	19780.1	15106.45	19093.64	22807.53	19197	13548.96	17544.68	29651.25	9942.903	17672	1.0863	0.74985
A2AGT5	Ckap5	Cytoskeleton-associated protein 5	130464	116526.7	115981.1	126233.8	122301	127045.7	129709.5	135609.1	142110.3	133619	0.9153	0.06090
Q8C8U0	Ppfibp1	Liprin-beta-1	23851.56	23638.71	27906.97	23964.05	24840	22072.55	16019.9	26572.22	24489.72	22289	1.1145	0.34717
P67778	Phb1	Prohibitin 1	306104	338949.1	337659.1	363058.5	336443	318166.8	336280	378628.4	353181.3	346564	0.9708	0.58132
Q6NZC7	Sec23ip	SEC23-interacting protein	56499	58158.73	58009.91	57684.32	57588	54819.45	57760.47	51704.59	57880.68	55541	1.0368	0.22391
Q9JLC8	Sacs	Sacsin	27128.8	29421.13	26534.77	29699.51	28196	27565.87	30492.27	25384.77	26714.19	27539	1.0238	0.64263
Q99M71	Epdr1	Mammalian ependymin-related protein 1	1232955	1455459	1239830	1235081	1290831	1465650	1412769	1465669	1467895	1452995	0.8884	0.02844
Q6NZK8	Ptpdc1	Protein tyrosine phosphatase domain-containing prc	23695.59	19986.65	23249.62	18875.02	21452	18661.48	18801.1	23972.51	21970.46	20851	1.0288	0.74425
P19096	Fasn	Fatty acid synthase	320529.9	347340.9	401421.8	305631.7	343731	335414	322699.7	366215.9	369789.7	348530	0.9862	0.84838
P54103	Dnajc2	DnaJ homolog subfamily C member 2	52780.12	29511.08	59636.66	37718.29	44912	42139.5	24027.34	29894.33	28129.07	31048	1.4465	0.12999
O09172	Gclm	Glutamate--cysteine ligase regulatory subunit	94515.38	107141.8	96828.12	72382.45	92717	87652.98	83665.83	98669.36	69688.57	84919	1.0918	0.44088
P63002	Tle5	TLE family member 5	26669.49	28545.11	14912.16	15316.99	21361	20351.95	27687.84	18252.8	16696.35	20747	1.0296	0.89284
P70261	Pald1	Paladin	22438.22	19147.83	19051.74	19069.91	19927	19708.61	21500.72	17176.87	18429.13	19204	1.0377	0.58304
Q3TUH1	Tamm41	Phosphatidate cytidylyltransferase, mitochondrial	29788.6	24750.27	22571.3	30569.03	26920	27583.58	30422.17	22215.78	24974.44	26299	1.0236	0.82044
Q91YT0	Ndufv1	NADH dehydrogenase [ubiquinone] flavoprotein 1, m	262386.9	259129.6	291111.3	290584.7	275803	255394.1	280709.3	264652	283667.5	271106	1.0173	0.68399
Q9D8Y0	Efhd2	EF-hand domain-containing protein D2	59866.22	56772.98	41044.83	40535.43	49555	39718.99	50053.96	37718.52	39473.06	41741	1.1872	0.22810
Q8C166	Cpne1	Copine-1	74441.48	69091.84	71561	63292.93	69597	76352.53	64277.85	71176.57	66291.32	69525	1.0010	0.98460
D3Z7Q2	Smim20	Small integral membrane protein 20	6034.808	6096.863		10934.17	7689		5659.539	3575.806	4757.021	4664	1.6485	0.15559
Q641P0	Actr3b	Actin-related protein 3B	22953.56	20969.5	36350.82	19823.43	25024	25057.47	41104.31	18427.54	21454.38	26511	0.9439	0.82236
Q80YN3	Bcas1	Breast carcinoma-amplified sequence 1 homolog	309351.5	288268.6	277428.2	467968	335754	258617	294985.3	345964.3	396802.1	324092	1.0360	0.83558
Q8VDK1	Nit1	Deaminated glutathione amidase	13982.78	16927.81	14898.34	13296.53	14776	16035.12	14872.91	14587.36	14169.11	14916	0.9906	0.87961
Q5XG73	Acbd5	Acyl-CoA-binding domain-containing protein 5	18161.84	15050.61	19271.36	17779.86	17566	16675.8	20125.38	18310.69	23192.37	19576	0.8973	0.27120
P14069	S100a6	Protein S100-A6	469457.8	512471.9	446201.8	337187.5	441330	410003.2	428400.3	304479.9	337415.7	370075	1.1925	0.18434
P84099	Rpl19	Large ribosomal subunit protein eL19	153025.7	142780.5	170690.3	182584.6	162270	149105.7	163173.8	163389.3	165611.9	160320	1.0122	0.84671
P63011	Rab3a	Ras-related protein Rab-3A	65650.93	83340.45	57180.29	47735.93	63477	55857.48	73938.36	46142.14	52515.86	57113	1.1114	0.53329
Q6PGE7	Slc6a7	Sodium-dependent proline transporter						9060.631				9061	0.0000	
Q3TIV5	Zc3h15	Zinc finger CCCH domain-containing protein 15	15779.87	15202.02	16571.58	13561.41	15279	15812.37	19413.76	15322.42	15652.48	16550	0.9232	0.31212
Q80W21	Gstm7	Glutathione S-transferase Mu 7	7335.572	14314.58	9970.828	7414.763	9759	5152.859	10207.72	9528.634	11744.62	9158	1.0656	0.79062
Q5F2E8	Taok1	Serine/threonine-protein kinase TAO1	30578.79	33755.92	28127.7	30851.54	30828	30781.63	37908.74	31404.63	29973.44	32517	0.9481	0.46304
Q8BL80	Arhgap22	Rho GTPase-activating protein 22	151120.9	180156.4	109579.9	128399.7	142314	143520.1	141256.9	125595.4	99899.05	127568	1.1156	0.44934
Q8R0G9	Nup133	Nuclear pore complex protein Nup133	17839.18	15311.88	12815.59	17333.97	15825	17627.64	15586.25	18681.37	17388.43	17321	0.9136	0.29725
Q61595	Ktn1	Kinectin	50063.73	51471.75	51977.75	59576.73	53272	56648.12	57129.51	61928.66	61238.17	59236	0.8993	0.05714
Q8VEK2	Rhbdd2	Rhomboid domain-containing protein 2	14723.95	15341.17	16975.53	17456.4	16124	14053.49	14630.8	21575.98	13896.8	16039	1.0053	0.96687

Q6PA06	Atl2	Atlastin-2	17414.95	18029.37	20839.43	18936.14	18805	15833.89	19138.23	19058.59	18977.9	18252	1.0303	0.63295
Q8BG40	Katnb1	Katanin p80 WD40 repeat-containing subunit B1	10371.74	12850.12	7759.844	8021.586	9751	9267.95	10270.54	5579.593	8944.633	8516	1.1450	0.46005
Q80U93	Nup214	Nuclear pore complex protein Nup214	29940.64	30616.71	19852.77	31081.09	27873	29918.91	17396.38	19133.5	26562.53	23253	1.1987	0.29323
Q8R3P0	Aspa	Aspartoacylase	173278.4	163371.1	119703.2	128633.6	146247	145358.7	155463.7	124293.7	113818.6	134734	1.0854	0.50256
Q9D6Y9	Gbe1	1,4-alpha-glucan-branching enzyme	32673.07	30845.65	30291.92	23262.1	29268	33204.28	28598.3	27733.67	26999.09	29134	1.0046	0.95877
P12960	Cntn1	Contactin-1	135038	121871.8	91208.76	138588	121677	115639	148921	86540.07	92929.26	111007	1.0961	0.56960
Q8K2K6	Agfg1	Arf-GAP domain and FG repeat-containing protein 1	33250.91	33193.68	31345.58	33338.29	32782	35280.9	31532.78	35227.58	33139.02	33795	0.9700	0.36053
Q69ZH9	Arhgap23	Rho GTPase-activating protein 23	19542.45	8895.928	8870.705	8167.478	11369	12705.72			33635.87	23171	0.4907	0.19565
Q91XE8	Tmem205	Transmembrane protein 205	90237.26	105401.6	132566.6	122356.6	112641	118670.5	114111.1	110097.6	106338.9	112304	1.0030	0.97350
Q61749	Eif2b4	Translation initiation factor eIF-2B subunit delta	40551.43	43538.89	45312.26	42729.48	43033	45260.99	43653.41	46772.87	46870.79	45640	0.9429	0.08113
Q9CQ06	Mrpl24	Large ribosomal subunit protein uL24m	13139.52	14303.51	15194.97	13351.6	13997	15093.94	12386.14	11859.32	15730.28	13767	1.0167	0.83753
F7BWT7	Tspan15	Tetraspanin-15	57312.61	60711.82	57050.17	69742	61204	62662.96	66677.31	57977.9	61196.54	62129	0.9851	0.79891
Q923X4	Glrx2	Glutaredoxin-2, mitochondrial	8492.237	7048.726	4703.269	7411.808	6914	7659.698	8533	5424.562	5213.379	6708	1.0308	0.86304
Q62465	Vat1	Synaptic vesicle membrane protein VAT-1 homolog	969351.5	858667.3	642919.3	916945.6	846971	907905.1	963643.9	636535.1	639922	787002	1.0762	0.61299
Q9EQQ9	Oga	Protein O-GlcNAcase	137165.7	151748	160305.5	139403.6	147156	158947.7	143492.7	154020.7	143158.1	149905	0.9817	0.69597
Q9CQW0	Emc6	ER membrane protein complex subunit 6	56403.16	35467.61	60608.95	59117.5	52899	47811.3	55425.18	77115.94	75592.73	63986	0.8267	0.28202
Q64213	Sf1	Splicing factor 1	66923.59	61222.45	67990.11	63353.56	64872	56024.36	50508.46	69550.99	64150.96	60059	1.0802	0.32669
O09111	Ndufb11	NADH dehydrogenase [ubiquinone] 1 beta subcomp	59079.93	57825.93	65252.11	62135.45	61073	53174.82	63190.54	62476.48	68175.77	61754	0.9890	0.85386
Q9QXX4	Slc25a13	Electrogenic aspartate/glutamate antiporter SLC25A	27108.18	26460.01	28683.08	33717.34	28992	26044.33	29525.24	24434.6	32952.79	28239	1.0267	0.77425
O55142	Rpl35a	Large ribosomal subunit protein eL33	203347.5	184214.1	240461.9	222053.9	212519	205133.2	177165.8	236106.1	245430.3	215959	0.9841	0.86709
Q99K01	Pdxdc1	Pyridoxal-dependent decarboxylase domain-contain	106510.9	114634.5	124045.4	90272.45	108866	115251.2	109357	117587.3	108986.2	112795	0.9652	0.61789
P19324	Serpinh1	Serpin H1	70884.45	84057.45	170757.1	89199.79	103725	87962.5	64261.89	144441.8	171162.8	116957	0.8869	0.70664
Q62470	Itga3	Integrin alpha-3	23830.01	22029.62	24614.54	26778.97	24313	22634	26011.87	22225.69	23012.02	23471	1.0359	0.54344
Q3UH68	Limch1	LIM and calponin homology domains-containing prot	89861.48	93841.7	106997.3	113431.3	101033	91238.49	93326.72	116771.5	120516.8	105463	0.9580	0.65549
Q9EPK7	Xpo7	Exportin-7	37773.53	43434.74	38822.57	39061.54	39773	37545.41	41457.48	32067.28	40024.41	37774	1.0529	0.43964
Q9CZX7	Pip4p2	Type 2 phosphatidylinositol 4,5-bisphosphate 4-pho	35310.08	33396.7	40950.36	40616.48	37568	39742.5	40061.1	47028.21	45012.75	42961	0.8745	0.08582
Q9Z2D0	Mtmr9	Myotubularin-related protein 9	40339.66	41042.33	39307.46	34648.23	38834	41368.85	39568.36	36220.76	35759.98	38229	1.0158	0.76940
Q91VC3	Eif4a3	Eukaryotic initiation factor 4A-III	77618.45	85715.35	97914.89	92118.3	88342	89744.26	87158.55	104464.4	98264.19	94908	0.9308	0.30801
Q921H8	Acaa1a	3-ketoacyl-CoA thiolase A, peroxisomal	154356.4	134764.6	165010.6	161476.4	153902	142968.7	153570.3	190868.8	186319	168432	0.9137	0.32847
Q8BGS2	Bola2	BolA-like protein 2	54550.96	46618.55	45528.4	36949.63	45912	38775.14	49514.05	38155.11	42721.35	42291	1.0856	0.44677
Q9QYB5	Add3	Gamma-adducin	122980.5	106829.2	104155.1	112822.7	111697	109744.3	103598.3	94361.55	102397.5	102525	1.0895	0.13045
P30999	Ctnnd1	Catenin delta-1	522975.8	491410.2	498513.8	579754.1	523163	497362.3	517319.6	495650.6	547348.9	514420	1.0170	0.72119
Q80X85	Mrps7	Small ribosomal subunit protein uS7m	8168.968	4679.797	9450.436	7132.57	7358	6975.904	8966.58	7197.812	9612.362	8188	0.8986	0.51565
Q9CZP7	Cdc37l1	Hsp90 co-chaperone Cdc37-like 1	11977.15	10149.01	10128.54	12002.7	11064	12031.91	11019.52	11677.66	10545.26	11319	0.9775	0.70023
O55128	Sap18	Histone deacetylase complex subunit SAP18	41998.43	41873.2	42419.33	44681.71	42743	47349.73	38840.08	24328.56	48288.61	39702	1.0766	0.60576
P30275	Ckmt1	Creatine kinase U-type, mitochondrial	396374.2	355137.4	405788.4	439957.5	399314	374571.3	388151.3	447008.1	424210.1	408485	0.9775	0.71626
Q04690	Nf1	Neurofibromin	37248.61	38509.56	36118.91	40246.16	38031	40999.66	40808.7	35149.97	38848.29	38952	0.9764	0.59047
Q8K1R7	Nek9	Serine/threonine-protein kinase Nek9	18822.87	23110.59	18710.11	19267.27	19978	21509.57	18822.09	13357.56	15952.9	17411	1.1474	0.25781
Q9JK81	Myg1	MYG1 exonuclease	61210.14	60855.91	64557.7	53447.22	60018	62859.14	51980.57	58233.3	48181.65	55314	1.0850	0.28565
Q791T5	Mtch1	Mitochondrial carrier homolog 1	120888.6	132403.7	126604.3	128821.8	127180	116343.8	128458	135115.3	137430.4	129337	0.9833	0.69865
Q61187	Tsg101	Tumor susceptibility gene 101 protein	71697.04	69803.23	72601.62	69027.92	70782	71358.48	71881.66	71727.96	72277.39	71811	0.9857	0.27029
Q9D6M3	Slc25a22	Mitochondrial glutamate carrier 1	102669.5	90941.3	94635.58	128459.9	104177	106008.4	112633.9	134318.8	127522.2	120121	0.8673	0.18621
Q8C407	Yipf4	Protein YIPF4	54610.67	53073.35	61788.9	55254.22	56182	66605.52	51853.1	42267.97	62112.03	55710	1.0085	0.93746
Q9WU84	Ccs	Copper chaperone for superoxide dismutase	8447.936	14998.85	3307.802		8918	7566.117	6187.222			6877	1.2969	0.67410
Q8BSY0	Asph	Aspartyl/asparaginyl beta-hydroxylase	51962.43	51966.6	51709.45	57507.8	53287	49832.43	47355.92	47677.37	52938.25	49451	1.0776	0.09100
Q5NCE8	Mrs2	Magnesium transporter MRS2 homolog, mitochondrial		8355.616	8034.749	18379.3	11590		8322.434		11908.02	10115	1.1458	0.76876
P97346	Nxn	Nucleoredoxin	111916.2	109760.1	123367.6	107514.6	113140	111843.6	121028.7	102492.3	104252.6	109904	1.0294	0.57811
Q3TDD9	Ppp1r21	Protein phosphatase 1 regulatory subunit 21	24787.1	24472.81	25888.48	22293.43	24360	27944.53	23887.04	26937.96	29033.07	26951	0.9039	0.10120
Q9D2N9	Vps33a	Vacuolar protein sorting-associated protein 33A	25145.27	26062.17	26319.98	25191.78	25680	24117.24	27082.78	22133.26	24220.54	24388	1.0529	0.26955
Q62219	Tgfb1i1	Transforming growth factor beta-1-induced transcrip	10799.59	8162.167	9046.979	8715.515	9181	12097.35	7897.149	11666.76	8620.926	10071	0.9117	0.48766
Q3TMH2	Scrn3	Secernin-3	41679.91	40612.74	33951.87	32551.85	37199	35891.85	38222.22	29331.26	28468.79	32979	1.1280	0.25265
Q9CPS6	Hint3	Adenosine 5'-monophosphoramidase HINT3	31174.67	26000.91	23590.81	27777.67	27136	25107.81	25149.7	25061.08	21929.94	24312	1.1162	0.16434
Q9WUD8	Faim	Fas apoptotic inhibitory molecule 1	18446.88	17858.08	16663.98	15142.29	17028	15906.47	16941.97	18521.35	14415.8	16446	1.0354	0.62565
Q9ER41	Tor1b	Torsin-1B	11385.38	11331.47	13629.84	8466.264	11203	13634.58	12418.83	14260.59	15346.96	13915	0.8051	0.06824
Q64378	Fkbp5	Peptidyl-prolyl cis-trans isomerase FKBP5	25374.46	26643.16	26389.84	29245.81	26913	23636.18	25700.48	24577.24	23798.71	24428	1.1017	0.03974
P56480	Atp5f1b	ATP synthase subunit beta, mitochondrial	2377873	2360076	2481475	2706090	2481378	2500379	2647959	2493189	2586893	2557105	0.9704	0.42122
Q6P9R2	Oxsr1	Serine/threonine-protein kinase OSR1	67132.05	63741.22	63099.72	91446.4	71355	66385.45	70092.3	63990.05	64203.83	66168	1.0784	0.48078
P10853;Q64475;Q8CGP	H2bc7;H2bc3;H2bc12;Hist1l	Histone H2B type 1-F/J/L;Histone H2B type 1-B;Histc	10034786	8353808	9642254	11003846	9758674	9822410	9895712	10043411	11184176	10236427	0.9533	0.48022
Q3TIR1	Trappc13	Trafficking protein particle complex subunit 13	12574.65	16869.16	11316.4	9103.527	12466	19568.16	22469.28	20453.02	17507.25	19999	0.6233	0.00797

Q8BZ36	Rint1	RAD50-interacting protein 1	7815.319	7211.851	6304.639	5808.172	6785	8156.33	8191.864	8145.177	9185.424	8420	0.8058	0.01957
Q9CXZ1	Ndufs4	NADH dehydrogenase [ubiquinone] iron-sulfur prote	71101.57	68724.89	72987.55	77493.55	72577	69702.7	78558.8	75078.79	69586.69	73232	0.9911	0.82713
P97813	Pld2	Phospholipase D2	11044.92	10235.79	10431.69	10153	10466	8261.212	10804.02	7899.587	6593.103	8389	1.2476	0.06126
Q80UW8	Polr2e	DNA-directed RNA polymerases I, II, and III subunit R	20989.8	16888.25	19740.3	21737.12	19839	18944.04	19045.15	19247.24	18566.31	18951	1.0469	0.44062
P61089	Ube2n	Ubiquitin-conjugating enzyme E2 N	251272.1	282671.2	193332.3	152416.9	219923	204361.9	248239.2	180500.2	157246.7	197587	1.1130	0.54713
P63323	Rps12	Small ribosomal subunit protein eS12	261598	254915.6	283211.3	264871.5	266149	263603.7	282365.4	301126.4	309722.3	289204	0.9203	0.10125
Q9JHU2	Palmd	Palmdelphin	59253.59	61243.73	68268.89	76562.58	66332	56272.19	64090.99	71755.21	64959.99	64270	1.0321	0.69661
Q80ZJ7	Snx32	Sorting nexin-32	6461.542	7709.053	5790.451	5398.134	6340	5752.054	8027.925	5529.199	5239.014	6137	1.0330	0.81192
Q61387	Cox7a2l	Cytochrome c oxidase subunit 7A-related protein, m	21785.2	28449.41	28970.6	16915.08	24030	30977.57	25728.53	32367.19	19880.49	27238	0.8822	0.45785
Q9JIH2	Nup50	Nuclear pore complex protein Nup50	8614.838	7996.491	9582.552	9522.664	8929	9133.219	8112.607	10054.7	8820.158	9030	0.9888	0.86150
Q8VBZ3	Clptm1	Putative lipid scramblase CLPTM1	53822.98	50292.92	56423.38	55870.65	54102	51416.57	57653.3	63181.93	61748.14	58500	0.9248	0.19031
P62855	Rps26	Small ribosomal subunit protein eS26	262504.5	297201.8	280516.5	307392.7	286904	294448.6	281007	372074.1	342870.3	322600	0.8893	0.17720
Q9WUB3	Pygm	Glycogen phosphorylase, muscle form	143511.2	144459	135278.8	169819	148267	148351.9	133796.3	122846.5	120788.6	131446	1.1280	0.13643
Q9EP89	Lactb	Serine beta-lactamase-like protein LACTB, mitochon	19185.52	19540.63	21030.97	17519.43	19319	18125.27	19042.63	11274	27021.4	18866	1.0240	0.89535
Q8VHI3	Pofut2	GDP-fucose protein O-fucosyltransferase 2	10912.99	10693.27	12972.14	10744.16	11331	6875.721	12227.32	8808.029	13333.35	10311	1.0989	0.54603
Q9D6K5	Synj2bp	Synaptojanin-2-binding protein	35051.04	37799.65	40150.5	42600.29	38900	39100.72	37295.26	43752.14	48303.13	42113	0.9237	0.31826
Q91WM2	Hdhd5	Haloacid dehalogenase-like hydrolase domain-conta	16225.23	13130.16	16884.81	18564.55	16201	13944.58	15609.67	15405.76	12432.14	14348	1.1292	0.22040
Q5SW19	Cluh	Clustered mitochondria protein homolog	38750.92	43676.02	44322.76	37877.42	41157	41492.75	42803.77	40421.72	38671.39	40847	1.0076	0.87419
Q8CC88	Vwa8	von Willebrand factor A domain-containing protein 8	75294.23	66820.21	76728.52	77185.15	74007	73082.88	72865.7	74071.29	75582.55	73901	1.0014	0.96751
Q8K1N1	Pnpla8	Calcium-independent phospholipase A2-gamma	14641.51	20326.39	11184.51	19551.57	16426	14199.1	13526.17	18813.83	14000.54	15135	1.0853	0.62157
Q8WTY4	Ciapin1	Anamorsin	21276.92	23761.66	22326.72	21739.77	22276	22244.09	19177.28	20019.04	23492.81	21233	1.0491	0.39164
P12367	Prkar2a	cAMP-dependent protein kinase type II-alpha regulat	32730.31	28250.36	30752.09	27117.61	29713	31552.74	29861.24	33234.84	28312.14	30740	0.9666	0.55597
Q99104	Myo5a	Unconventional myosin-Va	82449.2	82908.92	80825.96	94173.97	85090	83947.88	89077.76	78871.09	81527.25	83356	1.0208	0.66039
P97390	Vps45	Vacuolar protein sorting-associated protein 45	25316.42	25887.81	27361.42	24353.47	25730	25381.57	26997.94	30365.72	31372.98	28530	0.9019	0.11897
Q9CXW3	Cacybp	Calcyclin-binding protein	124388.8	128093.6	143661.5	117459.2	128401	132622	118454	140172.2	137456.3	132176	0.9714	0.62607
P08030	Aprt	Adenine phosphoribosyltransferase	55324.43	52949.74	56431.13	58231.1	55734	56140	52340.89	60064.95	45538.25	53521	1.0413	0.52551
Q64471	Gstt1	Glutathione S-transferase theta-1	125188.7	136453.8	97304.74	120307.9	119814	124865.1	133691.4	78228.06	78963.69	103937	1.1528	0.38333
Q5SVL6	Rap1gap2	Rap1 GTPase-activating protein 2	25721.7	24176.03	22177.13	18367.34	22611	23142.69	19633.54	25208.38	22376.93	22590	1.0009	0.99214
P20444	Prkca	Protein kinase C alpha type	87657.78	81841.15	65838.23	91626.19	81741	88608.91	84808.25	78017.34	77908.34	82336	0.9928	0.92732
P14231	Atp1b2	Sodium/potassium-transporting ATPase subunit bet	78822.38	71681.49	78808.55	75535.29	76212	69223.3	89058.78	73394.24	72977.84	76164	1.0006	0.99214
Q61990	Pcbp2	Poly(rC)-binding protein 2	109546.8	120151.4	115059.7	96947.2	110426	104071.7	117131.9	107259.3	121815.8	112570	0.9810	0.75238
Q80UY2	Kcmf1	E3 ubiquitin-protein ligase KCMF1	9017.854	7269.277	7360.786	7705.261	7838	7144.811	8025.33	5925.44	6655.857	6938	1.1298	0.18282
Q9D1F4	Akt1s1	Proline-rich AKT1 substrate 1	25002.29	29221.85	26974.27	28955.05	27538	25040.2	25616.04	29176.99	29695.36	27382	1.0057	0.92292
P70168	Kpnb1	Importin subunit beta-1	213528.3	233971.6	211795.1	220803.6	220025	221584.9	220766.2	216821	223066.3	220560	0.9976	0.92164
P59235	Nup43	Nucleoporin Nup43	15091.11	13106.02	14963.02	17267.04	15107	18364.98	11845.9	13079.05	13202.11	14123	1.0697	0.57916
O08750	Nfil3	Nuclear factor interleukin-3-regulated protein												
Q3UHG7	Dennd11	DENN domain-containing protein 11												
Q9CQV7	Dnajc19	Mitochondrial import inner membrane translocase s	12549.74	11401.48	15198.68	14011.36	13290	7813.437	9266.663	12518.85	14866.08	11116	1.1956	0.27121
Q6NZN0	Rbm26	RNA-binding protein 26	6135.858	6546.788	8030.161	4727.277	6360	6477.224	6190.864	5229.957	6092.01	5998	1.0604	0.63743
P12787	Cox5a	Cytochrome c oxidase subunit 5A, mitochondrial	434051.5	430287.9	456335.8	467779.5	447114	411196.7	461675.4	421138.1	420518	428632	1.0431	0.24628
P59325	Eif5	Eukaryotic translation initiation factor 5	129474.6	112309	118205	114113.8	118526	119380.4	117590.1	114987.3	120236.5	118049	1.0040	0.90948
P11031	Sub1	Activated RNA polymerase II transcriptional coactiva	50568.18	47695.64	54931.57	43723.38	49230	44108.32	46180.17	51883.58	46600.18	47193	1.0432	0.50668
P54923	Adprh	ADP-ribosylhydrolase ARH1	45958.73	44567.69	38931.95	28399.97	39465	39505.1	39918.36	43552.11	34827.75	39451	1.0003	0.99759
Q9EPV8	Ubl5	Ubiquitin-like protein 5	16146.34	15917.76	15849.84	8317.234	14058	10453.23	14071.66	13075.21	16187	13447	1.0454	0.79544
Q68FE2	Atg9a	Autophagy-related protein 9A	7763.778	8894.998	6976.032	5894.732	7382	8852.74	9066.5	5567.977	7332.097	7705	0.9582	0.76446
Q91WC9	Daglb	Diacylglycerol lipase-beta	16035.09	17876.63	10857.46	12546.03	14329	12794.89	15353.63	9799.344	8537.724	11621	1.2330	0.26738
P62257	Ube2h	Ubiquitin-conjugating enzyme E2 H	23396.23	22811.33	27465.39	14959.02	22158	17910.75	19260.58	17547.36	19281.13	18500	1.1977	0.21699
Q8C6B2	Rtkn	Rhotekin	33725.29	34031.54	41765.33	35972.17	36374	35687.86	33699.08	35337.87	37179.5	35476	1.0253	0.66890
Q62417	Sorbs1	Sorbin and SH3 domain-containing protein 1	243151.6	226336.3	215163.4	260381.8	236258	240694.5	246602.4	254359.9	249630.9	247822	0.9533	0.30415
Q3TEA8	Hp1bp3	Heterochromatin protein 1-binding protein 3	282791.1	266722.2	279021.4	317188.2	286431	256400.2	287306.3	300670.1	326482.8	292715	0.9785	0.74102
Q8CH18	Ccar1	Cell division cycle and apoptosis regulator protein 1	21301.49	26343.78	25158.01	27380.83	25046	18818.37	19583.13	27361.99	27061.16	23206	1.0793	0.51683
Q8K2X3	Stn1	CST complex subunit STN1		21131.46	13465.47		17298	9724.426		10692.77		10209	1.6945	0.20792
Q6PDL0	Dync1li2	Cytoplasmic dynein 1 light intermediate chain 2	124981.1	131863.4	134892.3	135251.8	131747	121871.1	138679.5	125636.5	125065.6	127813	1.0308	0.40696
Q8R5C5	Actr1b	Beta-centractin	74780.93	96394.2	76038.15	78565.3	81445	84955.95	95270.89	92695.19	83857.58	89195	0.9131	0.22856
Q7TPR4	Actn1	Alpha-actinin-1	183628.5	159220.5	166630.3	160815.5	167574	166272.7	158690.5	161174.2	156170.4	160577	1.0436	0.28674
Q8BUJ9	Lrp12	Low-density lipoprotein receptor-related protein 12	6183.506	7360.86	7182.166	8234.93	7240	8447.119	5963.612	9383.725	6642.856	7609	0.9515	0.69455
P17439	Gba1	Lysosomal acid glucosylceramidase	44236.3	42642.34	53309.14	50956.28	47786	45520.96	48315.71	61273.51	61194.68	54076	0.8837	0.24684
Q62442	Vamp1	Vesicle-associated membrane protein 1	37920.39	36368.28	45063.48	49534.37	42222	38981.08	41830.79	44097.98	45957.61	42717	0.9884	0.89004
P54285	Cacnb3	Voltage-dependent L-type calcium channel subunit b	18574.27	16153.22	18420.62	14630.22	16945	22455.13	15143.46	22010.7	15808.04	18854	0.8987	0.41380

O35633	Slc32a1	Vesicular inhibitory amino acid transporter	75209.63		59037.28		67123		228554.3	166612.9		197584	0.3397	0.05526
Q64338	Pde1c	Dual specificity calcium/calmodulin-dependent 3',5'	17906.58	18120.84	14117.17	12625.74	15693	15478.92	14508.67	15354.15	12941.54	14571	1.0770	0.48113
Q9R099	Tbl2	Transducin beta-like protein 2	19336.09	23301.35	30165	27133.13	24984	24675.56	23435.32	30308.74	34503.9	28231	0.8850	0.38707
P53026	Rpl10a	Large ribosomal subunit protein uL1	473651.1	471568.3	534101.3	582798.6	515530	527757	479533.4	600563.2	629784.7	559410	0.9216	0.35083
O54865	Gucy1b1	Guanylate cyclase soluble subunit beta-1	12105.9	15840.89	11423.53	10782.89	12538	12873.55	11349.16	12136.67	9672.856	11508	1.0895	0.46642
P54726	Rad23a	UV excision repair protein RAD23 homolog A	42485.38	47988.14	42258.47	40477.64	43302	42921.47	39064.02	37174.07	40432.15	39898	1.0853	0.14381
Q9WTK5	Nfkb2	Nuclear factor NF-kappa-B p100 subunit	15009.87	13448.36	16087.06	9318.583	13466	10967.72	10870.16	11238.29	10705.53	10945	1.2303	0.14144
Q07076	Anxa7	Annexin A7	258135.7	266816.7	235400.6	213327.1	243420	256231.9	251347.1	235979.2	212765.1	239081	1.0181	0.78883
Q9QZ06	Tollip	Toll-interacting protein	62464.39	58721.07	55485.4	60046.52	59179	57560.41	66593.36	53696.07	55354.63	58301	1.0151	0.79434
Q8BLK3	Lsamp	Limbic system-associated membrane protein	13516.45	18697.91	13685.32	17328.02	15807	17421.17	22840.19	13120.31	14947.95	17082	0.9253	0.62574
Q3V3V9	Carmil2	Capping protein, Arp2/3 and myosin-I linker protein 2	13202.69	11450.39	8977.488	11083.95	11179	22479.79	20002.48	6371.817	9463.472	14579	0.7667	0.43054
Q8BFZ9	Erlin2	Erlin-2	173185.2	174326.2	190709.3	191689.6	182478	190573.1	196550.7	207823.7	210811	201440	0.9059	0.03387
Q5SQY2	Bod1	Biorientation of chromosomes in cell division protein	5460.878	4251.261	5532.785	730.5264	3994	4229.976	2239.376	2671.078	5351.219	3623	1.1024	0.79056
Q63959	Kcnc3	Potassium voltage-gated channel subfamily C memb	6337.532	9087.542	7832.756	6647.023	7476	4414.537	9715.964	7288.501	7125.202	7136	1.0477	0.79488
P14152	Mdh1	Malate dehydrogenase, cytoplasmic	2006010	1986711	1733969	1495628	1805579	1812087	1793236	1389913	1447629	1610716	1.1210	0.28008
Q63912	Omg	Oligodendrocyte-myelin glycoprotein			21214.69		21215							
Q3TYS2	Cybc1	Cytochrome b-245 chaperone 1	56792.85	54713.46	53597.17	52398.7	54376	50698.19	54426.43	46402.8	55304.9	51708	1.0516	0.27780
P61226	Rap2b	Ras-related protein Rap-2b	52585.14	49464.7	42373.98	53116.8	49385	45792.38	55624.31	38924.54	47883.45	47056	1.0495	0.60215
P21300	Akr1b7	Aldo-keto reductase family 1 member B7												
Q9EPN1	Nbea	Neurobeachin	79721.95	45365.31	161867.3	37786.77	81185	56851.71	49309.29		21816.16	42659	1.9031	0.31819
Q3UUI3	Them4	Acyl-coenzyme A thioesterase THEM4	56492.27	58949.12	66771.81	75287.8	64375	57205.49	68624.59	76417.91	71346.59	68399	0.9412	0.51929
Q99LC8	Eif2b1	Translation initiation factor eIF-2B subunit alpha	18428.21	20532.86	17218.91	13791.81	17493	17333.81	19795.65	15340.12	16382.95	17213	1.0163	0.87483
Q7TNG5	Eml2	Echinoderm microtubule-associated protein-like 2	95768.02	87595.59	85470.74	87317.52	89038	80550.95	88408.38	89230.09	88587.9	86694	1.0270	0.47538
Q924M7	Mpi	Mannose-6-phosphate isomerase	40671.19	46623.07	35607.06	31476.35	38594	38221.76	38147.04	28838.28	29620.55	33707	1.1450	0.28580
Q9ER72	Cars1	Cysteine--tRNA ligase, cytoplasmic	81685.37	92038.55	93640.09	98933.34	91574	93835.2	95242.43	93889.73	97735.41	95176	0.9622	0.37088
Q9CQ65	Mtap	S-methyl-5'-thioadenosine phosphorylase	74758.84	59051.2	50588.64	42402.56	56700	56797.8	58245.06	48316.51	40568.33	50982	1.1122	0.50359
Q8C570	Rae1	mRNA export factor	18977	19429.2	22835.12	23220.24	21115	19322.55	16333.87	21814.01	20271.12	19435	1.0864	0.33468
Q8R0A7	Kiaa0513	Uncharacterized protein KIAA0513	23405.26	23110.14	20142.13	18659.04	21329	19539.02	19776.56	11910.04	15943.04	16792	1.2702	0.08260
P54775	Psmc4	26S proteasome regulatory subunit 6B	135692.3	129473.4	137984.9	124884.9	132009	128909.6	130431.7	140149.9	136370.8	133966	0.9854	0.63912
Q8K0Z7	Taco1	Translational activator of cytochrome c oxidase 1	36688.33	41147.66	34983.29	40898.67	38429	34923.2	41962.89	27497.57	37499.78	35471	1.0834	0.41735
Q9QWI6	Srcin1	SRC kinase signaling inhibitor 1	6951.592	11446.85	16909.22	6073.316	10345	15504.31	14798.04	13061.47	7515.037	12720	0.8133	0.46909
Q8BMA6	Srp68	Signal recognition particle subunit SRP68	95524.24	95024.94	108434.8	105877.7	101215	104551.2	99009.74	110488.4	113771.8	106955	0.9463	0.27369
Q91ZP9	Necab2	N-terminal EF-hand calcium-binding protein 2												
Q9JIY5	Htra2	Serine protease HTRA2, mitochondrial	46372.53	46091.55	55775.42	56366.13	51151	47919.6	51650.14	56963.21	55129.34	52916	0.9667	0.62974
Q3V384	Afg1l	AFG1-like ATPase	25672.66	45737.33	24895.41		32102	47819.15	44739.04	14285.45	32208.08	34763	0.9234	0.81289
Q9D1H6	Ndufaf4	NADH dehydrogenase [ubiquinone] 1 alpha subcom	19785.89	17287.2	15153.33	12593.65	16205	16369.33	19209.54	19297.51	13520.08	17099	0.9477	0.67899
Q99N87	Mrps5	Small ribosomal subunit protein uS5m	21654.73	25698.96	17884.52	27744.28	23246	28979.13	24294.81	23099.29	29467.69	26460	0.8785	0.28228
P11276	Fn1	Fibronectin	121433.5	104750.7	133033.6	119551.7	119692	116080.1	93675.98	118414.7	114604.8	110694	1.0813	0.31205
O70131	Ninj1	Ninjurin-1	9349.512	7458.527	9054.966	6533.554	8099	5915.269	9009.104		5714.5	6880	1.1773	0.35286
Q921F4	Hnrnpll	Heterogeneous nuclear ribonucleoprotein L-like	35561.38	36792.66	40228.18	38171.95	37689	38503.39	37304.79	43203.58	41683.65	40174	0.9381	0.19302
Q99PL5	Rrbp1	Ribosome-binding protein 1	103318.7	112710.4	128354.6	126707.7	117773	118855.5	117330.2	140942.8	142916.9	130011	0.9059	0.22808
O89001	Cpd	Carboxypeptidase D	46833.95	46534.32	54059.8	55225.09	50663	49211.55	51999.87	52017.26	61458.65	53672	0.9439	0.42762
O88986	Gcat	2-amino-3-ketobutyrate coenzyme A ligase, mitocho	22956.9	23510.1	23554.52	27707.53	24432	22613	24727.88	20323.85	27143.63	23702	1.0308	0.70313
Q9EPR4	Slc23a2	Solute carrier family 23 member 2	10990.5	8472.181	12229.17	15393.98	11771	9641.329	13332.38	15634.29	11713.82	12580	0.9357	0.68775
Q80VL1	Tdrkh	Tudor and KH domain-containing protein	24536.23	20300.97	24561.06	25339.75	23685	22891.38	20905.48	26510.15	29552.55	24965	0.9487	0.58739
Q925F2	Esam	Endothelial cell-selective adhesion molecule	11507.29	13292.77	14742.55	12773.13	13079	9315.016	12741.7	13166.42	14109.96	12333	1.0605	0.57016
Q8R3F5	Mcat	Malonyl-CoA-acyl carrier protein transacylase, mitoc	5730.647	4239.739	8590.729	7353.738	6479	5856.5	7529.688	8410.019	9222.919	7755	0.8354	0.32538
Q8BU88	Mrpl22	Large ribosomal subunit protein uL22m	25007.21	17907.9	17746.2	19999.05	20165	17325.18	16924.79	12207.54	19490.4	16487	1.2231	0.15858
Q6PDQ2	Chd4	Chromodomain-helicase-DNA-binding protein 4	47092.03	42298.03	46389.83	44275.08	45014	45454.99	44306.78	45778.83	48429.92	45993	0.9787	0.50826
P56393	Cox7b	Cytochrome c oxidase subunit 7B, mitochondrial	10594.31	11228.17	13600.83	11227.3	11663	9855.851	11785.45	7304.104	9226.186	9543	1.2221	0.11157
Q8VD33	Sgtb	Small glutamine-rich tetratricopeptide repeat-contai	20851.5	24561.79	24775.62	25296.09	23871	26920.19	24112.41	18462.79	23460.46	23239	1.0272	0.76636
Q3UPL0	Sec31a	Protein transport protein Sec31A	115627.4	117797.3	128932.7	100366.4	115681	115350	114331.1	125737	121761.6	119295	0.9697	0.59670
Q8CJ53	Trip10	Cdc42-interacting protein 4	14658.94	20755.83	20900.75	15685.49	18000	14854.43	19113.18	17534.42	13078.24	16145	1.1149	0.41683
P97372	Psme2	Proteasome activator complex subunit 2	21904.25	16849.14	15806.46	13274.81	16959	14210.52	15094.75	15631.63	16108.39	15261	1.1112	0.39581
Q9WV34	Mpp2	MAGUK p55 subfamily member 2	18416.33	18394.89	16097.24	19608.92	18129	14708	15105.35	12611.94	16718.95	14786	1.2261	0.02444
Q9JMH6	Txnrd1	Thioredoxin reductase 1, cytoplasmic	147527.6	172862.8	137263.2	128415.6	146517	156155.5	155403	115146.9	110717.6	134356	1.0905	0.46773
Q9CQR6	Ppp6c	Serine/threonine-protein phosphatase 6 catalytic su	44288.61	49587.73	42552.62	41506.84	44484	44426.59	41509.17	30890.89	39011.27	38959	1.1418	0.15714
P70392	Rasgrf2	Ras-specific guanine nucleotide-releasing factor 2	2724.896	6496.658		6979.566	5400	2416.816	6994.525		7377.4	5596	0.9650	0.92968
P35951	Ldlr	Low-density lipoprotein receptor	27385.01	25643.15	29439.81	31192.83	28415	31713.57	33110.52	35293.75	32750.34	33217	0.8554	0.01498

P27671	Rasgrf1	Ras-specific guanine nucleotide-releasing factor 1	38222.42			17209.95	**27716**				36068.03	**36068**	**0.7684**	
P97393	Arhgap5	Rho GTPase-activating protein 5	15736.04	13984.85	12895.71	14696.56	**14328**	16321.9	13863.66	9687.526	14951.83	**13706**	**1.0454**	0.70220
Q5RL79	Krtcap2	Keratinocyte-associated protein 2	17673.16	17251.05	19023.18	17621.49	**17892**	18365.35	20253.06	17695.01	18078.59	**18598**	**0.9621**	0.34492
Q8BKK4	Ppp1r1c	Protein phosphatase 1 regulatory subunit 1C	28108.89	31774.18	27416.52	28407.55	**28927**	28142.56	30214.69	31361.32	24609.58	**28582**	**1.0121**	0.85218
P13808	Slc4a2	Anion exchange protein 2	11516.57	8323.688	8230.151	11189.1	**9815**	11576.64	8076.289	3062.884	19985.09	**10675**	**0.9194**	0.82249
P62746	Rhob	Rho-related GTP-binding protein RhoB	46640.98	45764.87	28653.01	50377.6	**42859**	45137.64	70587.58	39973.61	43416.47	**49779**	**0.8610**	0.44800
Q3UR70	Tgfbrap1	Transforming growth factor-beta receptor-associate	8270.383	6375.33	8185.559	5821.339	**7163**	6421.729	7150.55	7479.365	7405.437	**7114**	**1.0069**	0.94423
Q8C754	Vps52	Vacuolar protein sorting-associated protein 52 homo	31129.25	34140.45	33037.8	35208.38	**33379**	31906.82	31600.94	29244.38	31641.29	**31098**	**1.0733**	0.07709
Q9CX86	Hnrnpa0	Heterogeneous nuclear ribonucleoprotein A0	43647.13	46489.15	49114.9	47788.1	**46760**	47490.76	50969.23	53288	50475.18	**50556**	**0.9249**	0.06321
Q99JY9	Actr3	Actin-related protein 3	155521.1	158119.5	159317.4	148607.5	**155391**	154093.6	155394.5	157859.4	157684.7	**156258**	**0.9945**	0.74692
Q9CZ69	Cmtm6	CKLF-like MARVEL transmembrane domain-containi	159792.1	163352.1	119508.7	185775.6	**157107**	143432.5	168762.3	131262.5	145651.6	**147277**	**1.0667**	0.55810
P63239	Pcsk1	Neuroendocrine convertase 1	13276.36		3873.446	7994.615	**8381**	16793.02		760.7349		**8777**	**0.9549**	0.95809
Q8BK72	Mrps27	Small ribosomal subunit protein mS27	16014.54	15031.14	17498.89	15833.14	**16094**	15202.52	16214.65	16920.3	15994.61	**16083**	**1.0007**	0.98602
Q920Q8	Ivns1abp	Influenza virus NS1A-binding protein homolog	19694.16	21166.77	25505.44	20628.28	**21749**	19354.99	19479.32	21253.79	24725.68	**21203**	**1.0257**	0.77176
P97355	Sms	Spermine synthase	19441.72	18057.73	18121.27	13952.6	**17393**	15804.82	15075.49	10577.63	16530.02	**14497**	**1.1998**	0.15721
P63001	Rac1	Ras-related C3 botulinum toxin substrate 1	629361.3	558967.9	589674.8	672003.9	**612502**	614151.9	586071.1	518711	568829.4	**571941**	**1.0709**	0.24757
Q7TMM9	Tubb2a	Tubulin beta-2A chain	3885457	3939096	4149350	3967575	**3985369**	4418943	3866951	3696843	3986218	**3992239**	**0.9983**	0.96803
P14576	Srp54	Signal recognition particle subunit SRP54	28721.08	29937.86	31558.13	33025.19	**30811**	30337.61	29310.8	35928.6	33395.51	**32243**	**0.9556**	0.44999
Q9D273	Mmab	Corrinoid adenosyltransferase MMAB	26298.83	17273.63	25634.5	24114.5	**23330**	21505.7	15887.68	15889.55	23082.55	**19091**	**1.2220**	0.18005
Q99P31	Hspbp1	Hsp70-binding protein 1	20428.11	20920.53	15748.21	13901.27	**17750**	14685	15872.18	15412.82	16174.28	**15536**	**1.1425**	0.25601
Q8BLR2	Cpne4	Copine-4	11531.59	10301.64	8030.966	7652.775	**9379**	9007.187	13980.39	5743.204	8795.272	**9382**	**0.9998**	0.99910
Q7TSH2	Phkb	Phosphorylase b kinase regulatory subunit beta	13214.93	17118.04	5206.862	25875.89	**15354**	8912.833	9838.246	3062.146		**7271**	**2.1116**	0.19351
O88502	Pde8a	High affinity cAMP-specific and IBMX-insensitive 3',5'-cyclic phosphodiesterase 8A												
O35857	Timm44	Mitochondrial import inner membrane translocase s	29912.22	28329.53	32095.42	37165.85	**31876**	32610.26	34123.89	33017.25	35003.14	**33689**	**0.9462**	0.39962
P05213	Tuba1b	Tubulin alpha-1B chain	8182770	8045828	7578190	7675343	**7870533**	8034980	7634482	7022460	7167327	**7464812**	**1.0544**	0.18683
Q811Q9	Pcyt1b	Choline-phosphate cytidylyltransferase B	37689.33	35752.38	43296.11	38841.43	**38895**	42904.11	37191.08	42067.57	30776.24	**38235**	**1.0173**	0.84405
Q923B6	Steap4	Metalloreductase STEAP4	171386.1	146310.5	161642.7	165763.7	**161276**	144634.3	162725.7	140072.9	125516.8	**143237**	**1.1259**	0.10236
P80313	Cct7	T-complex protein 1 subunit eta	212880.6	206914.7	232308	237073.6	**222294**	233558	236267.2	266959.3	244762.1	**245387**	**0.9059**	0.07095
Q9DBS5	Klc4	Kinesin light chain 4	156092.3	151529.3	142570.3	130211.1	**145101**	151392.7	150135.8	156945.5	145494.3	**150992**	**0.9610**	0.37652
Q62077	Plcg1	1-phosphatidylinositol 4,5-bisphosphate phosphodi	19641.29	15956.07	20019.52	15763.8	**17845**	14683.58	14940.01	16736.82	17188.72	**15887**	**1.1232**	0.18586
Q8VE33	Gdap1l1	Ganglioside-induced differentiation-associated prot	32148.39	40926.73	39987.79	31806.44	**36217**	38862.67	31292.42	32260.76	32915.62	**33833**	**1.0705**	0.45595
Q9DCD0	Pgd	6-phosphogluconate dehydrogenase, decarboxylatir	129947.5	135411.8	144105.7	117077.4	**131636**	126530	123975.1	140400.6	130001.5	**130227**	**1.0108**	0.84073
P57722	Pcbp3	Poly(rC)-binding protein 3	1197119	1531706	1259947	1322990	**1327941**	1238710	1519251	1498879	1348669	**1401377**	**0.9476**	0.48320
Q9CQQ7	Atp5pb	ATP synthase F(0) complex subunit B1, mitochondria	352918	353643.3	363313	389221.1	**364774**	344922.7	372127.8	352695.7	371627.6	**360343**	**1.0123**	0.69861
P63330	Ppp2ca	Serine/threonine-protein phosphatase 2A catalytic s	26245.66	19481.64	17674.81	22804.98	**21552**	17535.49	18346.86	24190.59	16866.77	**19235**	**1.1204**	0.39497
Q8VHP7	Serpinb1b	Leukocyte elastase inhibitor B	28432.65	17875.38	19688.68	27642.3	**23410**	19467.94	19214.74	29525.82	19105.42	**21828**	**1.0724**	0.68616
Q8C0L0	Tmx4	Thioredoxin-related transmembrane protein 4	53354.91	53152.47	51993.46	58659.22	**54290**	52030.18	63001.38	59601.79	62160.29	**59198**	**0.9171**	0.14213
P56391	Cox6b1	Cytochrome c oxidase subunit 6B1	231430.3	255396.8	243525.2	276155.9	**251627**	228946.9	257802.3	249150.7	256688.6	**248147**	**1.0140**	0.77501
P56812	Pdcd5	Programmed cell death protein 5	73024.3	77047.61	75722.54	71888.29	**74421**	79887.72	69730.57	81619.6	69292.21	**75133**	**0.9905**	0.84448
O35454	Clcn6	H(+)/Cl(-) exchange transporter 6	29782.89	35284.06	37141.5	40490.33	**35675**	35177.73	36789.73	37442.7	42661.24	**38018**	**0.9384**	0.42909
Q8VE47	Uba5	Ubiquitin-like modifier-activating enzyme 5	25127.71	25902.58	24249.56	21807.02	**24272**	28547.44	23870.72	20601.24	20710.57	**23432**	**1.0358**	0.69876
Q9CQ91	Ndufa3	NADH dehydrogenase [ubiquinone] 1 alpha subcom	25273.51	22298.78	19488.64	20883.55	**21986**	19720.57	17039.44	22463.65	17989.67	**19303**	**1.1390**	0.16917
Q8VE19	Mios	GATOR complex protein MIOS	9702.143	11759.69	12479.28	9706.56	**10912**	11964.04	9228.918	11656.86	9568.682	**10605**	**1.0290**	0.76913
Q9D125	Mrps25	Small ribosomal subunit protein mS25	8137.271	9878.558	13555.6	13119.36	**11173**	10577.5	13687.29	14644.57	13033.06	**12986**	**0.8604**	0.29080
Q61035	Hars1	Histidine--tRNA ligase, cytoplasmic	197527.1	209437.2	184813.1	173223	**191250**	199188.9	192031.4	192858.9	173521.8	**189400**	**1.0098**	0.85339
Q91VH6	Memo1	Protein MEMO1	23184.58	19275.78	21721.67	17264.29	**20362**	22630.8	19753.18	18244.3	18572.65	**19800**	**1.0284**	0.74478
Q924D0	Rtn4ip1	Reticulon-4-interacting protein 1, mitochondrial	23717.14	25312.1	24635.42	27576.2	**25310**	24921	27180.65	23858.61	25909.65	**25467**	**0.9938**	0.88958
Q8C8T7	Elfn1	Protein ELFN1												
Q9JKL4	Ndufaf3	NADH dehydrogenase [ubiquinone] 1 alpha subcom	25107.75	17928.46	16527.93	24155.89	**20930**	15112.78	24049.2	14410.46	20699.88	**18568**	**1.1272**	0.48345
Q9WUQ2	Preb	Prolactin regulatory element-binding protein	89262.63	101697.7	101958.8	116833.9	**102438**	107802	99742.03	119463	127233.1	**113560**	**0.9021**	0.22897
P46471	Psmc2	26S proteasome regulatory subunit 7	126832.6	124846.4	138265.5	125549.4	**128874**	124815.2	131493.3	133516.2	131633.9	**130365**	**0.9886**	0.70003
Q8BYI8	Fam234b	Protein FAM234B	7796.768	8166.149	5420.329	7420.749	**7201**			32391.63	6400.547	**19396**	**0.3713**	0.20259
P51830	Adcy9	Adenylate cyclase type 9	5548.539	4950.609	5116.131	10426.7	**6510**	5057.863	6387.801	8619.606	6622.072	**6672**	**0.9758**	0.91803
O08784	Tcof1	Treacle protein	10027.38	11447.88	8666.573	13171.17	**10828**	10517.24	11052.32	12616.1	15302.46	**12372**	**0.8752**	0.32609
O08579	Emd	Emerin	22691.99	21662.97	24179.47	26679.56	**23803**	24323.79	24838.69	25717.33	25593.97	**25118**	**0.9477**	0.29160
Q9R0A0	Pex14	Peroxisomal membrane protein PEX14	54922.91	38939.56	46835.14	40717.65	**45354**	41594.52	47714.64	41372.28	49581.85	**45066**	**1.0064**	0.94729
P04627	Araf	Serine/threonine-protein kinase A-Raf	20809.9	21320.42	19634.13	23737.06	**21375**	22935.27	24074.59	21986.96	22323.54	**22830**	**0.9363**	0.18716
P19157	Gstp1	Glutathione S-transferase P 1	260807.4	272338	209985	198237.3	**235342**	226812	234512	171592.5	163641.5	**199139**	**1.1818**	0.21225
Q9JM63	Kcnj10	ATP-sensitive inward rectifier potassium channel 10	77189.49	66713.07	81780.76	81876.24	**76890**	77050.32	92914.31	83375.09	79576.5	**83229**	**0.9238**	0.25027

O88543	Cops3	COP9 signalosome complex subunit 3	200985.7	212379.2	193161.3	186684.7	198303	213750.5	195681.6	179823.6	166686	188985	1.0493	0.45134
O89051	Itm2b	Integral membrane protein 2B	31364.41	40055.78	35602.19	36335.85	35840	33719.45	38556.4	28905.31	34939	34030	1.0532	0.52375
Q99L20	Gstt3	Glutathione S-transferase theta-3	18453.96	16445.16	11761.55	8797.711	13865	14489.49	11844.03	4607.403	7570.77	9628	1.4400	0.22157
O88456	Capns1	Calpain small subunit 1	364412.3	408651.9	330920.6	284774.5	347190	346158.8	334755.1	335201.9	320526.3	334161	1.0390	0.64309
Q9D0B5	Tstd3	Thiosulfate sulfurtransferase/rhodanese-like domair	11882.25	13008.08	10365	5782.25	10259		13597.34	10962.69	8662.185	11074	0.9264	0.72926
P42859	Htt	Huntingtin	23913.84	25160.3	23314.83	21798.73	23547	21651.27	24328.58	20414.1	21878.61	22068	1.0670	0.21860
Q5EG47	Prkaa1	5'-AMP-activated protein kinase catalytic subunit alp	48496.38	44917.5	42002.26	55045.32	47615	52131.05	47819.17	45935.23	50131.31	49004	0.9717	0.67160
Q6P1D5	Sez6l	Seizure 6-like protein												
Q9D8N0	Eef1g	Elongation factor 1-gamma	1492877	1552755	1649612	1533488	1557183	1500446	1545550	1586901	1613225	1561531	0.9972	0.91978
Q62073	Map3k7	Mitogen-activated protein kinase kinase kinase 7	13145.1	11244	9254.616	5406.312	9763	7923.915	9960.741	9058.709	10803.46	9437	1.0345	0.85975
Q8CC86	Naprt	Nicotinate phosphoribosyltransferase	15619.85	15161.63	16578.72	13869.84	15308	15033.38	12796.76	9527.587	11028.19	12096	1.2655	0.04997
Q9ES74	Nek7	Serine/threonine-protein kinase Nek7	104605.8	106017.9	99925.11	104823.3	103843	110964.2	101508.9	100671.1	103873.7	104254	0.9961	0.88368
Q91Y86	Mapk8	Mitogen-activated protein kinase 8	5412.187	5259.902	4967.654	3941.316	4895	5734.485	2979.247	5151.696	4669.854	4634	1.0564	0.71352
Q8BSF4	Pisd	Phosphatidylserine decarboxylase proenzyme, mitoc	28575.65	28584.33	30539.13	29606.88	29326	31439.94	30772.07	28033.82	28621.85	29717	0.9869	0.69468
Q9WTL7	Lypla2	Acyl-protein thioesterase 2	33495.63	32020.66	26330.26	22647.48	28624	28691.16	28064.9	24390.22	20468.1	25404	1.1268	0.34698
Q9CR30	Josd2	Josephin-2	32793.36	20706.95	27587.31		27029				24896.24	24896	1.0857	
Q9Z0P4	Palm	Paralemmin-1	116071.2	120841.9	96784.26	126129.6	114957	124753.9	105406.5	113157.5	111662.1	113745	1.0107	0.87796
Q6PEV3	Wipf2	WAS/WASL-interacting protein family member 2		7816.29		11709.88	9763			13640.76	3757.929	8699	1.1223	0.85978
P14869	Rplp0	Large ribosomal subunit protein uL10	522537.8	475045.3	587275.1	605791.9	547663	549501.4	522816.2	608491	651419.4	583057	0.9393	0.42920
Q8BMD8	Slc25a24	Mitochondrial adenyl nucleotide antiporter SLC25A2	26775.12	26399.6	25008.16	27477.04	26415	25085.34	24662.41	22959.05	25044.78	24438	1.0809	0.03387
Q8CGQ8	Slc24a4	Sodium/potassium/calcium exchanger 4												
P70303	Ctps2	CTP synthase 2	24601.29	28175.67	24981.4	25009.56	25692	27983.05	27709.89	24779.47	21530.35	25501	1.0075	0.91526
O35239	Ptpn9	Tyrosine-protein phosphatase non-receptor type 9	10923.5	10264.19	11680.28	6891.308	9940	10213.8	8496.438	7817.278	7526.34	8513	1.1675	0.28528
P70414	Slc8a1	Sodium/calcium exchanger 1								32654.34		32654	0.0000	
Q8BZ60	Ston2	Stonin-2	7163.676	7363.446	6009.424		6846	5630.726	4795.164	7445.011	7012.69	6221	1.1004	0.47365
Q8CCB4	Vps53	Vacuolar protein sorting-associated protein 53 homo	41035.9	40928.98	39321.34	39808.54	40274	39519.46	40260.9	39222	40635.83	39910	1.0091	0.52001
Q8K2P7	Slc38a1	Sodium-coupled neutral amino acid symporter 1												
P13634	Ca1	Carbonic anhydrase 1	19675.59	20530.45	18362.68	31578.48	22537	14561.38	15636.22	20315.06	6455.232	14242	1.5824	0.09524
A2AAY5	Sh3pxd2b	SH3 and PX domain-containing protein 2B	54759.46	53932.29	68167.47	45817.54	55669	41911.72	49545.87	56769.88	51460.62	49922	1.1151	0.34097
P33173	Kif1a	Kinesin-like protein KIF1A	137744.4	146211.7	136707.3	137949.8	139652	138243.6	137142.8	147013.3	140290.8	140673	0.9927	0.75494
Q810U4	Nrcam	Neuronal cell adhesion molecule	9205.114	6012.49	7190.196	6632.653	7260	6270.832	7876.137	6138.147	7016.405	6825	1.0637	0.60594
Q6P5D3	Dhx57	Putative ATP-dependent RNA helicase DHX57	25805.88	23828.77	20746.2	23870.36	23563	23610.25	25431.47	22751.74	23734.1	23882	0.9866	0.79705
Q64327	Mea1	Male-enhanced antigen 1	21256.11	16592.33	22377.05	18938.31	19791	24738.97	17608.89	26671.32	24865.54	23471	0.8432	0.17293
Q60900	Elavl3	ELAV-like protein 3	55094.05	60284.68	59885.52	68492.27	60939	62352.02	63986.36	66352.16	65888.66	64645	0.9427	0.25264
Q4PJX1	Odr4	Protein odr-4 homolog	34373.14	29689.99	31250.49	31650.2	31741	31479.63	33102.55	30576.38	29809.76	31242	1.0160	0.69303
Q5SWP3	Nacad	NAC-alpha domain-containing protein 1	112492.7	111922	108261.3	133546	116555	111855.7	124197.1	130882.7	131371.4	124577	0.9356	0.31532
Q9WUP7	Uchl5	Ubiquitin carboxyl-terminal hydrolase isozyme L5	27794.89	24604.87	27370.6	25979.12	26437	27048.53	26699.38	28313.1	32764.45	28706	0.9210	0.19920
Q8BYN5	Fsd1l	FSD1-like protein	40896.14	44018.39	36900.44	36779.55	39649	39612.36	41078.04	33421.57	36379.68	37623	1.0538	0.43845
O35566	Cd151	CD151 antigen	64275.7	58847.3	29705.62	53145.4	51494	48181.45	68552.42	30609.06	31133.69	44619	1.1541	0.58003
Q9R0Q9	Mpdu1	Mannose-P-dolichol utilization defect 1 protein	66433.39	60157.46	63305.21	78737.09	67158	72413.69	80458.26	78377.09	86911.87	79540	0.8443	0.04960
Q9JKC6	Cend1	Cell cycle exit and neuronal differentiation protein 1	60325.51	59037.56	65941.01	90979.94	69071	57150.93	64651.92	93897.94	96769.85	78118	0.8842	0.49754
P22005	Penk	Proenkephalin-A												
Q9EP72	Emc7	ER membrane protein complex subunit 7	100428.5	84755.57	121367.8	118239	106198	109346.3	105345.8	121184.2	115267.2	112786	0.9416	0.50010
Q80ZE5	Paqr8	Membrane progestin receptor beta		142.4233		183.3381	163	161.2978				161	1.0098	
Q61001	Lama5	Laminin subunit alpha-5	150991.3	142155.6	113718.4	179437	146576	138102.9	155727.2	118451.5	136162.4	137111	1.0690	0.56465
Q8CFE6	Slc38a2	Sodium-coupled neutral amino acid symporter 2	8238.264	8370.088	9200.752	9236.359	8761	7430.018	9027.908	9030.488	5620.659	7777	1.1265	0.29301
P62141	Ppp1cb	Serine/threonine-protein phosphatase PP1-beta cat	92977.05	119123.7	96262.33	99216.05	101895	107982.8	113793.4	112153.6	96391.14	107580	0.9472	0.45209
Q3UHK1	Slc2a13	Proton myo-inositol cotransporter												
P28738	Kif5c	Kinesin heavy chain isoform 5C	110609.9	101523.7	88727.34	102726.9	100897	95697.92	94643.85	86977.27	91854.34	92293	1.0932	0.13162
P40124	Cap1	Adenylyl cyclase-associated protein 1	277897.7	281003.4	292579	261943.8	278356	268683.3	268393.5	272008.1	263230.1	268079	1.0383	0.16890
P52479	Usp10	Ubiquitin carboxyl-terminal hydrolase 10	47266.15	46483.39	50175.33	49394.92	48330	45815	50943.81	52687.54	55300.16	51187	0.9442	0.23849
O08917	Flot1	Flotillin-1	129000.5	130573.2	112410.9	139740.1	127931	129494.6	143965.5	120833.9	125169.9	129866	0.9851	0.80727
A6H6E9	Ttc23l	Tetratricopeptide repeat protein 23-like	44943.2	45155.96	38108.72	37526.34	41434	41152.17	44767.02	47870.19	37630.11	42855	0.9668	0.65747
B2RR83	Ythdc2	3'-5' RNA helicase YTHDC2	18488.19	16003.31	14488.18	20028.78	17252	17424.64	19473.46	21479.06	17705.67	19021	0.9070	0.29835
Q8CI75	Dis3l2	DIS3-like exonuclease 2	21434.6	23053.84	23160.88	8620.15	19067	14403.17	16434.15	13836.9	16948.84	15406	1.2377	0.34659
Q9CY57	Chtop	Chromatin target of PRMT1 protein	28100.2	27036.89	29959.22	34358.99	29864	29761.12	28559.3	32721.4	36778.78	31955	0.9346	0.42452
Q8VDS4	Rprd1a	Regulation of nuclear pre-mRNA domain-containing	8308.782	9241.026	9466.771	8551.712	8892	8927.861	8334.622	9843.627	8318.008	8856	1.0041	0.93901
Q8BZW8	Nhlrc2	NHL repeat-containing protein 2	17920.94	16118.01	11806.09	11243.08	14272	13986.9	12171.39	11430.57	9759.557	11837	1.2057	0.23678
Q99MS8	Tpgs1	Tubulin polyglutamylase complex subunit 1	7653.601	6826.603	6137.657	3757.131	6094	4481.835	3986.763	5501.735	4952.023	4731	1.2882	0.18008

Q60790	Rasa3	Ras GTPase-activating protein 3	16025.32	16485.51	15323.38	16862.97	16174	15572.04	16263.94	14508.46	16850.65	15799	1.0238	0.55606
Q9Z266	Snapin	SNARE-associated protein Snapin	35129.37	39883.7	35365.71	38092.5	37118	40017.91	37873.34	42433.04	34767.86	38773	0.9573	0.43692
P56695	Wfs1	Wolframin	29133.2	24175.8	25620.08	31979.53	27727	29253.27	28820	26849.32	30280.43	28801	0.9627	0.59250
Q8BG94	Commd7	COMM domain-containing protein 7	17508.07	17351.96	8930.536	9423.995	13304	13645.06	17438.84	15384.91	15948.51	15604	0.8526	0.39473
Q6DFV3	Arhgap21	Rho GTPase-activating protein 21		8944.097	8976.366	7427.302	8449		9105.703			9106	0.9279	
Q8R4U7	Luzp1	Leucine zipper protein 1	16239.77	12041.41	14887	14830.14	14500	17129.01	14041.4	16371.71	15193.72	15684	0.9245	0.32767
Q9EQ20	Aldh6a1	Methylmalonate-semialdehyde dehydrogenase [acyl	304844	274343.5	261157.8	337404.3	294437	286852.4	307242.6	245565.9	271431.1	277773	1.0600	0.46569
P07356	Anxa2	Annexin A2	2158869	2116387	2061824	2331903	2167246	2118779	2166999	2010134	2039826	2083934	1.0400	0.26988
Q9DCR2	Ap3s1	AP-3 complex subunit sigma-1	30887.63	28853.52	28877.94	28187.67	29202	29965.75	31108.8	32978.86	30956.47	31252	0.9344	0.05408
Q99J56	Derl1	Derlin-1	63019.65	66593.66	72777.17	80076.27	70617	67404.61	68881.34	74778.66	84400.23	73866	0.9560	0.56748
Q8BQZ4	Ralgapb	Ral GTPase-activating protein subunit beta	15497.58	15686.43	18549.84	19598.89	17333	15327.81	17500.36	17863.06	16749.2	16860	1.0281	0.70036
P18760	Cfl1	Cofilin-1	2479975	2398756	2176771	2079556	2283764	2295746	2014151	2072128	1911208	2073308	1.1015	0.14023
O70566	Diaph2	Protein diaphanous homolog 2	13626.35	15778.59	17772.1	15288.72	15616	16706.57	18088.22	14961.66	17417.32	16793	0.9299	0.32034
Q3B7Z2	Osbp	Oxysterol-binding protein 1	35579.13	41118.75	40899.25	36336.43	38483	38735.35	35439.07	40487.73	38120.98	38196	1.0075	0.87844
Q8K2I1	Fntb	Protein farnesyltransferase subunit beta	50415.86	53698.45	64351.52	55355.51	55955	54403.63	54421.45	42951.72	49155.47	50233	1.1139	0.20631
Q6P9Q6	Fkbp15	FK506-binding protein 15	27343.57	27365.83	26437.18	27680.68	27207	22443.99	27937.68	26585.15	28109.88	26269	1.0357	0.51234
P09470	Ace	Angiotensin-converting enzyme	37841.48	37865.61	46020.8	35112.27	39210	38462.77	43564.85	38559.91	44027.93	41154	0.9528	0.51527
O55098	Stk10	Serine/threonine-protein kinase 10	22095.84	20199.1	16130.93	17782.77	19052	17725.07	19321.82	14509.89	18614.37	17543	1.0860	0.40616
Q924N4	Slc12a6	Solute carrier family 12 member 6	22617.53	26811.52	23368.4	25747.01	24636	23977.14	22578.89	18622.91	21217.38	21599	1.1406	0.09056
P70399	Tp53bp1	TP53-binding protein 1	20531.16	20353.99	19735.25	22544.65	20791	22863.16	23718.39	25968.49	26310.98	24715	0.8412	0.00927
Q9Z2I9	Sucla2	Succinate--CoA ligase [ADP-forming] subunit beta, m	171497.5	187421.4	168967.9	192644.2	180133	174894.4	190273.9	184071.8	184553.1	183448	0.9819	0.63569
Q922H4	Gmppa	Mannose-1-phosphate guanyltransferase alpha	27455.61	20709.58	26401.01	16098.31	22666	22166.75	15301.36	21464	22979.63	20478	1.1069	0.51606
O35691	Pnn	Pinin	23260.68	20762.22	23721.2	21741.52	22371	19178.59	20435.94	20718.38	25146.39	21370	1.0469	0.52129
Q9JMF3	Gng13	Guanine nucleotide-binding protein G(I)/G(S)/G(O) subunit gamma-13												
Q8CIP4	Mark4	MAP/microtubule affinity-regulating kinase 4								9323.652		9324	0.0000	
O88967	Yme1l1	ATP-dependent zinc metalloprotease YME1L1	27725.47	29841.9	34807.68	31987.47	31091	27368.63	28222.34	34540.36	35832.13	31491	0.9873	0.88426
P47791	Gsr	Glutathione reductase, mitochondrial	51531.8	48541.55	46375.99	46379.29	48207	51898.94	47984.97	51024.04	47366.66	49569	0.9725	0.44148
Q60960	Kpna1	Importin subunit alpha-5	16013.48	15745.45	16000.73	13524.64	15321	13064.4	15452.99	12636.18	15476.86	14158	1.0822	0.27530
Q9D832	Dnajb4	DnaJ homolog subfamily B member 4	120913.2	117893.2	102225.4	129985.4	117754	108837.6	127769.1	105566.6	101218.7	110848	1.0623	0.43325
Q76LS9	Mindy1	Ubiquitin carboxyl-terminal hydrolase MINDY-1	22776.53	23970.13	25499.36	19525.58	22943	25654.39	22658.03	26552.37	25999.5	25216	0.9099	0.19021
Q9CQJ6	Denr	Density-regulated protein	20869.1	22127.24	24402.1	21468.41	22217	36869.79	25644.23	23758.27	26459.91	28183	0.7883	0.09819
Q8R5H6	Wasf1	Actin-binding protein WASF1	19598.14	17150.04	18872.27	16353.33	17993	17705.49	17246.47	17039.21	16336.24	17082	1.0534	0.29915
Q5KU39	Vps41	Vacuolar protein sorting-associated protein 41 homo	14383.11	14385.9	15202.5	15340.88	14828	15660.72	14803.87	15354.44	15539.17	15340	0.9667	0.16081
Q8BTG3	Tcp11l1	T-complex protein 11-like protein 1	55173.19	55617.5	44331.85	44545.23	49917	51002.66	52874.43	49173.29	47152.65	50051	0.9973	0.96983
P03921	Mtnd5	NADH-ubiquinone oxidoreductase chain 5	20820.37	22532.3	23161.04	28407.66	23730	20596.38	25141.25	21127.51	25899.89	23191	1.0232	0.80829
P61971	Nutf2	Nuclear transport factor 2	96961.17	109611	82336.25	60121.45	87257	82714.5	92342.36	64327.75	58829.88	74554	1.1704	0.37284
Q8R3G1	Ppp1r8	Nuclear inhibitor of protein phosphatase 1	20701.98	19758.96	15770.75	17582.29	18453	18194.1	16847.36	16003.01	21480.69	18131	1.0178	0.85037
Q9Z321	Top3b	DNA topoisomerase 3-beta-1	14647	12196.37	12738.35	15689.28	13818	14029.17	14094.44	17315.17	14826.31	15066	0.9171	0.30858
Q80XI4	Pip4k2b	Phosphatidylinositol 5-phosphate 4-kinase type-2 be	17686.03	17460.4	14102.33	14986.26	16059	16063.89	16309.5	15630.16	15563.08	15892	1.0105	0.86059
O89079	Cope	Coatomer subunit epsilon	61883.75	58475.48	69569.99	60990.77	62730	59839.31	63638.14	73093.49	72223.82	67199	0.9335	0.31060
Q8C080	Snx16	Sorting nexin-16	26084.7	22749.68	21041.17	20325.78	22550	28823.12	21158.33	22892.65	28974.48	25462	0.8856	0.26873
P97449	Anpep	Aminopeptidase N	40303.43	38907.05	39207.58	40354.79	39693	40435.98	36117.36	34047.78	33729.19	36083	1.1001	0.06346
Q9D9E0	Slc22a17	Solute carrier family 22 member 17	14674.39	6279.132	11951.57	9140.475	10511	9920.069	10924.23	9062.848	9415.823	9831	1.0692	0.72586
P15388	Kcnc1	Potassium voltage-gated channel subfamily C member 1								19538.2		19538	0.0000	
Q61024	Asns	Asparagine synthetase [glutamine-hydrolyzing]	50466.49	57692.99	53337.54	41335.14	50708	55741.34	48627.84	39312	41574.76	46314	1.0949	0.42004
Q61609	Slc20a1	Sodium-dependent phosphate transporter 1												
Q9CQF3	Nudt21	Cleavage and polyadenylation specificity factor subu	82465.66	83033.26	100075.6	86792.3	88092	90251.94	81750.15	89183.46	98352.28	89884	0.9801	0.74811
Q7TN33	Celf6	CUGBP Elav-like family member 6	31376.89	29676.69	29608.33	36949.28	31903	31815.2	31425.36	35975.3	35385.63	33650	0.9481	0.43626
P38647	Hspa9	Stress-70 protein, mitochondrial	371167.1	377404.4	397529.9	450886.2	399247	394591.9	408980.8	424236	445699.8	418377	0.9543	0.40068
P62881	Gnb5	Guanine nucleotide-binding protein subunit beta-5	20919.96	21131.38	18364.06	22235.3	20663	19197.57	19568.23	18308.43	19401.32	19119	1.0807	0.12471
O88196	Ttc3	E3 ubiquitin-protein ligase TTC3	39840.65	37359.93	29888.12	21795.97	32221	30118.09	32629.56	14356.46	21492.27	24649	1.3072	0.24180
Q99MN1	Kars1	Lysine--tRNA ligase	151809.8	167520	168141.4	174566.3	165509	171804.8	175751.8	174798.5	177406.1	174940	0.9461	0.10693
Q9JI90	Rnf14	E3 ubiquitin-protein ligase RNF14	56844.87	59947.89	51792.26	43682.95	53067	53859.2	51801.07	49638.96	44868.26	50042	1.0605	0.48234
Q8BH55	Thnsl1	Threonine synthase-like 1	10098.64	9589.807	10308.32	14917.11	11228	12256.57	15535.04	10632.21	10492.48	12229	0.9182	0.57883
P58281	Opa1	Dynamin-like 120 kDa protein, mitochondrial	72698.79	70648.27	73544.22	79070.35	73990	74142.45	77482.56	80382.09	78431.7	77610	0.9534	0.15446
Q7TT50	Cdc42bpb	Serine/threonine-protein kinase MRCK beta	33342.69	32069.4	34876.78	35716.13	34001	32483.8	38114.54	36266.3	37322.59	36047	0.9433	0.21797
Q61423	Kcna4	Potassium voltage-gated channel subfamily A member 4								891.9319	9209.808	5051	0.0000	
Q9DC61	Pmpca	Mitochondrial-processing peptidase subunit alpha	38588.82	34552.93	37174.65	42368.82	38171	32977.35	34836	33536.66	36781.53	34533	1.1054	0.09476
Q64737	Gart	Trifunctional purine biosynthetic protein adenosine-3	51903.07	55203.03	55369.63	49681.48	53039	54379.66	48910.71	56469.82	52004.91	52941	1.0019	0.96475

P70697	Urod	Uroporphyrinogen decarboxylase	60253.3	53898.83	47965.99	36056.52	**49544**	48469.3	39667.44	43487.8	36966.79	**42148**	1.1755	0.24367
P70280	Vamp7	Vesicle-associated membrane protein 7	39881.23	48209.99	51069.67	51410.3	**47643**	45086.47	47794.74	48735.88	49044	**47665**	0.9995	0.99392
Q9JMG7	Hdgfl3	Hepatoma-derived growth factor-related protein 3	46541.88	46870.14	49619.91	52115.61	**48787**	54621.77	45405.64	49524.36	48113.98	**49416**	0.9873	0.79642
P27546	Map4	Microtubule-associated protein 4	601286.8	619678.1	658969.1	639325.8	**629815**	608906.2	593043.9	694397.3	687220.6	**645892**	0.9751	0.59912
Q99MN9	Pccb	Propionyl-CoA carboxylase beta chain, mitochondria	95011.83	95530.45	82700.92	98473.16	**92929**	89379.44	97354.45	68866.73	79513.58	**83779**	1.1092	0.24415
Q91WG2	Rabep2	Rab GTPase-binding effector protein 2	24913.19	27456.04	26929.77	28390.89	**26922**	26483.89	29769.67	24796.49	23157.86	**26052**	1.0334	0.60439
Q8BMJ3	Eif1ax	Eukaryotic translation initiation factor 1A, X-chromos	51460.58	52129.79	63515.63	57548.73	**56164**	55661.43	50590.58	50796.48	68437.44	**56371**	0.9963	0.96846
P52483;Q91W82	Ube2e3;Ube2e2	Ubiquitin-conjugating enzyme E2 E3;Ubiquitin-conju	35090.05	29583.59	30425.32	26896.53	**30499**	27840.8	29683.28	32544.52	32309.49	**30595**	0.9969	0.96417
O88653	Lamtor3	Ragulator complex protein LAMTOR3	52261.8	49872.71	39768.46	59246.91	**50287**	57764.52	55862.62	57403.54	62247.25	**58319**	0.8623	0.10822
P97927	Lama4	Laminin subunit alpha-4	475411.3	443734.2	424351	566371.2	**477467**	456134	479260.7	505927.1	492173.1	**483374**	0.9878	0.86457
Q3TC46	Patl1	Protein PAT1 homolog 1	1496.949	1935.874	1980.439	1977.246	**1848**	2603.693	1565.426	2618.391	3691.378	**2620**	0.7053	0.13672
O35551	Rabep1	Rab GTPase-binding effector protein 1	33016.59	34317.48	34842.39	33081.82	**33815**	33350.91	35248.25	38452.83	34975.75	**35507**	0.9523	0.19501
Q8VDC0	Lars2	Probable leucine--tRNA ligase, mitochondrial	15577.95	15514.72	17107.23	16535.43	**16184**	19657.42	16731.06	16595.6	17912.98	**17724**	0.9131	0.10504
Q9CRB6	Tppp3	Tubulin polymerization-promoting protein family mei	1879830	1856169	1647394	1545895	**1732322**	1706561	1643444	1660498	1531911	**1635603**	1.0591	0.31999
Q99JP7	Ggt7	Glutathione hydrolase 7	53352.59	58244.08	52809	52090.4	**54124**	47666.93	60792.5	51539.75	57575.15	**54394**	0.9950	0.93688
Q9CQY6	Uqcc2	Ubiquinol-cytochrome-c reductase complex assemb	36795.92	33260.71	24135.35	31407.55	**31400**	26210.63	35669.92	20149.59	18897.54	**25232**	1.2445	0.23433
P0DN89	Tmem254	Transmembrane protein 254	36168.89	36400.74	29851.1	26043.33	**32116**	18641.63	29699.63	28726.44	34365.86	**27858**	1.1528	0.34617
Q9CPQ3	Tomm22	Mitochondrial import receptor subunit TOM22 homo	74820.91	64821.81	65368.41	74919.32	**69983**	62833.32	72543.42	72585.16	75677.51	**70910**	0.9869	0.82309
Q9CR62	Slc25a11	Mitochondrial 2-oxoglutarate/malate carrier protein	254376.4	270111	284704.4	323582.5	**283194**	311900.9	305442.3	311826	332446.7	**315404**	0.8979	0.08985
Q9QXS1	Plec	Plectin	906270.6	855164.1	792844.2	1007742	**890505**	899660.3	928140.3	841159.3	856569.4	**881382**	1.0104	0.86015
Q8R0X7	Sgpl1	Sphingosine-1-phosphate lyase 1	52950.6	52858.11	59465.5	57706.48	**55745**	56466.86	56283.44	59896.73	64330.19	**59244**	0.9409	0.21538
Q8VCM7	Fgg	Fibrinogen gamma chain	154196	113059.2	186462.6	205036	**164688**	112254.1	90967.2	149924.8	111988.2	**116284**	1.4163	0.08614
P97434	Mprip	Myosin phosphatase Rho-interacting protein	39252.78	38940.03	36111.36	37167.27	**37868**	32920.37	36032.29	33678.64	40547.76	**35795**	1.0579	0.31041
Q9CYR6	Pgm3	Phosphoacetylglucosamine mutase	30529.6	30918.5	30478.35	31360.64	**30822**	28405.21	29865.74	21247.37	29482.62	**27250**	1.1311	0.12978
Q6PNC0	Dmxl1	DmX-like protein 1	8271.404	5699.867	6571.853	6277.376	**6705**	8970.211	8366.414	11363.66	6771.137	**8868**	0.7561	0.09716
Q80UP3	Dgkz	Diacylglycerol kinase zeta	101332	103256.2	89573.2	94665.66	**97207**	104667.7	104360.8	107770	96138.64	**103234**	0.9416	0.18313
Q9CR00	Psmd9	26S proteasome non-ATPase regulatory subunit 9	75775.58	73575.37	75238.59	72474.51	**74266**	77666.89	69195.37	73650.14	74251.34	**73691**	1.0078	0.77217
Q9WVK4	Ehd1	EH domain-containing protein 1	75983.91	73630.53	83926.44	74061.49	**76901**	71539.99	64399.38	80178.39	80334.34	**74113**	1.0376	0.56028
Q6P542	Abcf1	ATP-binding cassette sub-family F member 1	52255.73	61913.9	52804.73	53870.41	**55211**	50281.52	60991.79	54574.86	59121.74	**56242**	0.9817	0.76500
Q8BZM1	Glmn	Glomulin	8128.623	9382.006	7061.426	5516.402	**7522**	6870.383	8806.976	7375.646	7566.087	**8155**	0.9224	0.54988
Q7TN22	Txndc16	Thioredoxin domain-containing protein 16	9349.314	8948.143	9387.171	10857.51	**9636**	9394.705	9220.916	8566.594	10053.85	**9309**	1.0351	0.55233
Q9R069	Bcam	Basal cell adhesion molecule	275822.2	245853.7	195140.3	303407.9	**255056**	244795	274383.8	222455.3	217836.5	**239868**	1.0633	0.58782
P61963	Dcaf7	DDB1- and CUL4-associated factor 7	19452.09	20917.76	19250.12	21295.54	**20229**	18056.18	19925.37	22264.89	20566.34	**20203**	1.0013	0.98054
Q8BWY3	Etf1	Eukaryotic peptide chain release factor subunit 1	32456.19	34911.83	35150.65	31781.04	**33575**	30203.78	37805.41	29380.11	31075.85	**32116**	1.0454	0.51487
P62880	Gnb2	Guanine nucleotide-binding protein G(I)/G(S)/G(T) su	850009.1	768342.9	689376.8	926250.6	**808495**	789698.7	912542.3	703827	748959.6	**788757**	1.0250	0.78143
Q9DBN5	Lonp2	Lon protease homolog 2, peroxisomal	12020.14	11498.81	14664.79	12629.18	**12703**	12163.67	10816.88	12427.09	11548.48	**11739**	1.0821	0.26289
P51807	Dynlt1	Dynein light chain Tctex-type 1	18325.46	11565.81	35634.5	22105.99	**21908**	14924.2	18218.54	44738.25	33906.27	**27947**	0.7839	0.50942
Q99JB8	Pacsin3	Protein kinase C and casein kinase II substrate prote	30123.79	24535.94	27206.39	21918.95	**25946**	25997.67	29683.38	21660.34	25109.88	**25613**	1.0130	0.89459
O08992	Sdcbp	Syntenin-1	77558.52	65994.63	53752.63	59605.15	**64228**	71750.61	67415.55	70398.04	65991.09	**68889**	0.9323	0.41028
Q9D938	Tmem160	Transmembrane protein 160	20204.66	22693.53	13686.49	21447.98	**19508**	12760.08	15038.07	19203.57	14568.17	**15392**	1.2674	0.14053
Q8BHJ6	Serinc5	Serine incorporator 5	57183.29	58428.73	45247.09	72070.77	**58232**	58101.26	72483.1	43519.62	49826.79	**55983**	1.0402	0.79598
P16045	Lgals1	Galectin-1	1361321	1356814	1206073	1045498	**1242427**	1281159	1215096	957712.1	1010477	**1116111**	1.1132	0.28752
Q9D964	Gatm	Glycine amidinotransferase, mitochondrial	100422.7	95678.04	110224.1	106535.3	**103215**	101648.7	101071.4	110025.2	114988.6	**106933**	0.9652	0.45606
Q70IV5	Synm	Synemin	328061.2	290241	190551.3	326474.3	**283832**	270044.5	343826.2	274665.4	265409.8	**288486**	0.9839	0.90462
Q8VDN2	Atp1a1	Sodium/potassium-transporting ATPase subunit alpl	1688601	1642605	1381307	2003919	**1679108**	1618838	1737422	1502891	1521734	**1595221**	1.0526	0.56702
Q8CAA7	Pgm2l1	Glucose 1,6-bisphosphate synthase	150732	163626.7	140358.1	124108.4	**144706**	158563.7	145331.5	135819.6	125755.7	**141368**	1.0236	0.76955
Q62348	Tsn	Translin	45684.15	42677.94	40085.62	35045	**40873**	38632.04	43040.18	33714.77	32397.79	**36946**	1.1063	0.28134
Q9CQJ8	Ndufb9	NADH dehydrogenase [ubiquinone] 1 beta subcomp	83108.08	76104.26	85445.98	96593.08	**85313**	84846.62	87944.8	90990.19	93996.52	**89445**	0.9538	0.41179
Q9D0L8	Rnmt	mRNA cap guanine-N7 methyltransferase	34422.48	36139.37	41895.91	36688.89	**37287**	39366.19	35587.13	39109.81	39401.43	**38366**	0.9719	0.58259
Q61656	Ddx5	Probable ATP-dependent RNA helicase DDX5	183444.3	183938.8	206330.7	210864.6	**196145**	189901.3	193879.3	220093.7	214721.9	**204649**	0.9584	0.44580
P70158	Smpdl3a	Acid sphingomyelinase-like phosphodiesterase 3a	8482.024	6533.338	8956.22	6387.876	**7590**	8130.122	6570.013	7010.304	7880.893	**7398**	1.0260	0.80756
P04919	Slc4a1	Band 3 anion transport protein	74476.59	79516.96	132485.3	133624.1	**105026**	77486.11	63142.28	128052.1	66229.63	**83728**	1.2544	0.37348
Q8BTX9	Hsdl1	Inactive hydroxysteroid dehydrogenase-like protein 1	94984.09	112300.4	112265	108414.8	**106991**	106728	121964.4	116582.8	133145	**119605**	0.8945	0.11592
Q8BIP0	Dars2	Aspartate--tRNA ligase, mitochondrial	10276.06	11914.74	10890.54	20611.83	**13423**	12932.5	13292.16	10154.77	13150.11	**12382**	1.0841	0.69532
P62082	Rps7	Small ribosomal subunit protein eS7	509360.3	479171.1	528796.1	555761.3	**518272**	510690.8	485151.1	569927.7	598436.9	**541052**	0.9579	0.48589
Q61361	Bcan	Brevican core protein	15952.2	10964.9	24888.59	34348.36	**21539**	11758.63	16089.86	8086.749	10702.17	**11659**	1.8473	0.11780
Q8R5A6	Tbc1d22a	TBC1 domain family member 22A	18858.58	18582.29	19976.67	18065.3	**18871**	17636.53	20243.43	18303.7	20939.85	**19281**	0.9787	0.65758
Q8BJU2	Tspan9	Tetraspanin-9	20435.82	17203.39	15501.17	6368.408	**14877**	16879.32	22538.06	15908.56	13772.58	**17275**	0.8612	0.52444
Q68FF6	Git1	ARF GTPase-activating protein GIT1	56442.09	54545.91	62226.95	55522.95	**57184**	53193.06	56501.06	57193.36	60749.16	**56909**	1.0048	0.90935

E9Q6C8	Xkr6	XK-related protein 6												
P0C0S6;Q3THW5	H2az1;H2az2	Histone H2A.Z;Histone H2A.V	571962.6	473324.7	618015.9	717307.3	595153	520426.5	520592.1	678347.9	683104.9	600618	0.9909	0.93911
P97797	Sirpa	Tyrosine-protein phosphatase non-receptor type sub	63453.38	62442.54	48242.03	60096.65	58559	58286.66	59909.59	45465.88	50363.21	53506	1.0944	0.34079
Q3ULF4	Spg7	Paraplegin	15482.65	14753.64	15042.2	17816.12	15774	14044.3	18699.84	17592.62	13838.6	16044	0.9832	0.85522
Q8C7K6	Pcyox1l	Prenylcysteine oxidase-like	105981.8	116438.8	124807	120660.5	116972	113884.2	116914.9	125016.3	131663.4	121870	0.9598	0.42338
Q8VCD6	Reep2	Receptor expression-enhancing protein 2	46160.78	46141.87	45399.29	53120.13	47706	46077.48	47833.41	57501.09	52327.39	50935	0.9366	0.34231
Q9WTX5	Skp1	S-phase kinase-associated protein 1	107178.3	112496.6	88239.94	99532.77	101862	100842.1	120435.6	95638.06	77142.8	98515	1.0340	0.75717
Q91WK1	Spryd4	SPRY domain-containing protein 4	17229.25	15291.51	14764.65	14727.47	15503	15081.37	19280.3	15276.12	16440.51	16520	0.9385	0.40438
Q9D1J3	Sarnp	SAP domain-containing ribonucleoprotein	50651.3	56724.6	61027.55	60871.51	57319	58885.16	59677.07	77419.7	77312.27	68324	0.8389	0.10478
Q8BMI3	Gga3	ADP-ribosylation factor-binding protein GGA3	38378.52	37288.36	37267.79	32041.28	36244	39616.97	38714.83	37511.62	36319.63	38041	0.9528	0.30306
P58771	Tpm1	Tropomyosin alpha-1 chain	355621.3	296957.4	327467.8	363993.3	336010	307444.4	232925.8	253067.8	259596.4	263259	1.2763	0.01597
Q91VR5	Ddx1	ATP-dependent RNA helicase DDX1	195620.2	212165.8	216945.7	220040.9	211193	213864.3	220098.2	245584.3	249023.8	232143	0.9098	0.09077
Q9DCM2	Gstk1	Glutathione S-transferase kappa 1	37853.16	35761.8	37613.23	40416.88	37911	35018.48	35532.25	36416.02	39756.52	36681	1.0335	0.42316
P97370	Atp1b3	Sodium/potassium-transporting ATPase subunit beta	570035.4	510931.3	443284	556879	520282	504341.5	580662.9	392482.4	456885.2	483593	1.0759	0.48148
Q9DB25	Alg5	Dolichyl-phosphate beta-glucosyltransferase	20532.45	17853.96	20706.94	20723.1	19954	16259.33	19002.8	16844.73	19724.97	17958	1.1112	0.11661
Q91YW3	Dnajc3	DnaJ homolog subfamily C member 3	91909.71	120298	143573.1	119563.7	118836	127555.1	135481	109634.2	108125.8	120199	0.9887	0.91694
A2A432	Cul4b	Cullin-4B	22733.29	25243.86	18707.63	21332.32	22004	23943.61	22315.37	17422.18	21136.02	21204	1.0377	0.69513
Q8BGK9		Uncharacterized protein C3orf18 homolog												
P27601	Gna13	Guanine nucleotide-binding protein subunit alpha-13	99110.86	93032.92	73690.47	93286.82	89780	89931.54	98207.33	74427.32	73275.95	83961	1.0693	0.50593
Q9EQG7	Enpp5	Ectonucleotide pyrophosphatase/phosphodiesteras	12837.83	15231.34		13741.62	13937	12174.82	19155.63		27608.83	19646	0.7094	0.27482
O55222	Ilk	Integrin-linked protein kinase	148110.6	157564.3	163435.3	149670.9	154695	151332.9	148552.7	159069.5	153145.5	153025	1.0109	0.70527
O88811	Stam2	Signal transducing adapter molecule 2	12965.02	15607.5	12239.78	11196.48	13002	14261.88	12608.04	11408.6	9843.572	12031	1.0808	0.49145
Q80VD1	Fam98b	Protein FAM98B	23314.11	22004.47	26716.7	19607.51	22911	24764.34	22361.23	32463.82	24583.21	26043	0.8797	0.28360
P21614	Gc	Vitamin D-binding protein	80053.66	70515.65	80018.63	49773.27	70090	68183.15	53772.26	69627.78	49442.74	60256	1.1632	0.30442
P84309	Adcy5	Adenylate cyclase type 5	21816.72	21635.47	9031.079	30744.82	20807	12736.21	21682.63	17117.23	14031	16392	1.2694	0.40109
P62849	Rps24	Small ribosomal subunit protein eS24	267848.3	262776.5	276307.9	283842.4	272694	271347.8	285226.1	304428.9	339050.5	300013	0.9089	0.12626
P70349	Hint1	Adenosine 5'-monophosphoramidase HINT1	82934.23	100218.9	74665.8	67375.4	81299	79901.37	91495.99	78379.91	63061.23	78210	1.0395	0.74750
P61148	Fgf1	Fibroblast growth factor 1	259369.9	290116.4	281421.5	266062.5	274243	298164.1	279289.7	309911.4	258601.2	286492	0.9572	0.39085
O70166	Stmn3	Stathmin-3	24257.03	24968.34	27171.77	35576.89	27994	25342.76	24916.38	32410.49	30345.73	28254	0.9908	0.93773
Q9CX80	Cygb	Cytoglobin	12061.29	15674.96			13868		14099.57	10728.72		12414	1.1171	0.61584
Q8BWT5	Dip2a	Disco-interacting protein 2 homolog A	26372.95	28608.07	29317.2	30196.12	28624	29122.61	33050.88	23418.91	26719.76	28078	1.0194	0.81133
Q9ER35	Fn3k	Fructosamine-3-kinase	17721.32	15820.88	12578.7	26072.9	18048	19754.74	22484.07	16147.38	18637.44	19256	0.9373	0.71585
Q91XM9	Dlg2	Disks large homolog 2	9114.464	9741.702	7719.479	9661.848	9059	10523.93	10105.85	9279.62	8807.762	9679	0.9360	0.34754
P52332	Jak1	Tyrosine-protein kinase JAK1	26320.41	26428.61	23611.92	28758.15	26280	24302.31	26786.4	27379.1	28379.3	26712	0.9838	0.76216
Q99LD9	Eif2b2	Translation initiation factor eIF-2B subunit beta	16789.59	18060.42	15539.87	14375.79	16191	13599.85	17836.2	16792.85	25638.18	18467	0.8768	0.42768
Q8BWR2	Pithd1	PITH domain-containing protein 1	50375.88	52763.34	40630.16	41726.37	46374	35251.28	43844.03	37836.32	43005.47	39984	1.1598	0.13315
P16627	Hspa1l	Heat shock 70 kDa protein 1-like	78888.61	88594.2	81903.44	85587.06	83743	95866.81	84789.38	106847	86436.1	93485	0.8958	0.12709
A2A8Z1	Osbpl9	Oxysterol-binding protein-related protein 9	562746.8	598384.7	426570.8	503027.4	522682	502732.8	545461.9	512102.7	573359.1	533414	0.9799	0.80191
O35963	Rab33b	Ras-related protein Rab-33B	21164.74	15670.63	21716.04	21297.4	19962	22795.01	19333.33	26094.62	25447.38	23418	0.8524	0.15149
P46660	Ina	Alpha-internexin	247636.7	249586.8	196675.7	318414.7	253078	240495	284950.9	199242.9	216352	235260	1.0757	0.58808
P97478	Coq7	5-demethoxyubiquinone hydroxylase, mitochondrial	20775.61	16976.06	15672.72	18668.86	18023	17531.17	19114.35	16179.79	13273.39	16525	1.0907	0.40113
Q7TN99	Cpeb3	Cytoplasmic polyadenylation element-binding protei	24388.31	22689.76	22173.99	21260.28	22628	23896.88	23603.16	23416.53	24884.39	23950	0.9448	0.12161
Q3TLH4	Prrc2c	Protein PRRC2C	20472.47	21633.09	22619.86	27984.91	23178	19349.69	25785.55	30587.09	34426.3	27537	0.8417	0.27755
Q9D328	Tmem35a	Novel acetylcholine receptor chaperone	86450.2	81747.53	78978.09	86624.63	83450	70776.95	75877.62	125355.5	120541.7	98138	0.8503	0.35069
P20357	Map2	Microtubule-associated protein 2	153443.1	149305.3	140754.7	167262.3	152691	153995.3	158853.8	143024.8	147019.5	150723	1.0131	0.77435
P26039	Tln1	Talin-1	199689.7	183222.4	207973.8	202893.9	198445	195772.3	190418.6	204905.7	195145.2	196560	1.0096	0.76963
Q9QUK3	Cln8	Protein CLN8		21343.19	21752.93	17717.71	20271	21167.59			18203.44	19686	1.0298	0.78769
Q3TL44	Nlrx1	NLR family member X1	18147.81	16675.18	20320.97	22461.74	19401	15954.2	19135.65	21976.95	21879.1	19736	0.9830	0.86609
Q8VE22	Mrps23	Small ribosomal subunit protein mS23	34538.5	34887.02	38314.07	44703.43	38111	35577.15	37395.98	39792.84	41907.98	38668	0.9856	0.84501
Q8VC42	Rmc1	Regulator of MON1-CCZ1 complex	10308.86	11364.27	10795.09	8854.802	10331	12614.71	9557.122	6190.636	10913.37	9819	1.0521	0.73854
Q8BFW6	Entpd3	Ectonucleoside triphosphate diphosphohydrolase 3	31926.46	29856.56	24709.89	35152.09	30411	28603.07	30790.13	35112.48	30436.62	31236	0.9736	0.76090
Q61016	Gng7	Guanine nucleotide-binding protein G(I)/G(S)/G(O) s	15262.73	15204.81		6336.893	12268	15150.43	16229.76	6831.252	5767.5	10995	1.1158	0.76710
Q8K394	Plcl2	Inactive phospholipase C-like protein 2												
Q8HW98	Iglon5	IgLON family member 5												
P84084	Arf5	ADP-ribosylation factor 5	58560.84	55644.28	45951.62	44419.03	51144	57242.43	48328.09	56410.79	43035.93	51254	0.9978	0.98270
Q9QXG4	Acss2	Acetyl-coenzyme A synthetase, cytoplasmic	42349.59	45825.37	44629.74	29634.23	40610	39888.75	37741.3	39178.83	39039.5	38962	1.0423	0.67623
P70336	Rock2	Rho-associated protein kinase 2	52330.39	52432.65	53840.38	50631.72	52309	48983.85	48049.64	52881.45	57684.81	51900	1.0079	0.86418
Q9CYZ2	Tpd52l2	Tumor protein D54	61092	66369.92	71449.07	57845.66	64189	62286.32	57647.3	64428.68	68357.3	63180	1.0160	0.79585
P62748	Hpcal1	Hippocalcin-like protein 1	138191.9	160369.8	109975.9	124184	133180	126192.6	158160.6	82629.21	85249.55	113058	1.1780	0.37486

P19783	Cox4i1	Cytochrome c oxidase subunit 4 isoform 1, mitochor	598231.1	623276.4	663122.9	727833.6	**653116**	631567.3	643467.2	623252.1	699528.2	**649454**	**1.0056**	**0.91547**
Q63ZW7	Patj	InaD-like protein	37102.42	34382.42	25419.55	21572.05	**29619**	35566.43	39209.12	32599.06	48032.49	**38852**	**0.7624**	**0.11208**
P35278	Rab5c	Ras-related protein Rab-5C	151941.6	126398.3	126053.1	138950.1	**135836**	139310.7	133624.2	131009	127785.4	**132932**	**1.0218**	**0.67612**
Q9D0M5	Dynll2	Dynein light chain 2, cytoplasmic	83455.8	65421.43	50398.07	72101.26	**67844**	64942.23	66509.45	43311.47	51050.1	**56453**	**1.2018**	**0.24711**
Q9Z0Z4	Heph	Hephaestin												
Q8VCA8	Scrn2	Secernin-2	12791.18	10449.07	10975.21	10224.38	**11110**	10452.61	12378.71	7578.062	8214.064	**9656**	**1.1506**	**0.28608**
P45878	Fkbp2	Peptidyl-prolyl cis-trans isomerase FKBP2	220854.7	210511.1	209149.3	243323.6	**220960**	223180.7	250947	216672.9	224284	**228771**	**0.9659**	**0.50233**
Q9CQ86	Mien1	Migration and invasion enhancer 1	40639.82	38740.38	29193.86	25229.04	**33451**	32871.71	39266.76	36690.61	29581.51	**34603**	**0.9667**	**0.79679**
Q80U40	Rimbp2	RIMS-binding protein 2												
Q9Z2Z6	Slc25a20	Mitochondrial carnitine/acylcarnitine carrier protein	59507.09	66635.84	75928.95	75508.52	**69395**	63085.57	69267.2	79444.09	74518.44	**71579**	**0.9695**	**0.69308**
Q6ZPJ3	Ube2o	(E3-independent) E2 ubiquitin-conjugating enzyme U	104315.9	105861.8	90732.36	91637.05	**98137**	99741.67	105704.2	95015.85	88795.18	**97314**	**1.0085**	**0.88379**
Q9D385	Arl2bp	ADP-ribosylation factor-like protein 2-binding proteir	11221.5	13490.23	9635.333	8307.022	**10664**	11764.47	11273.81	8854.944	10035.77	**10482**	**1.0173**	**0.89301**
Q9DB29	Iah1	Isoamyl acetate-hydrolyzing esterase 1 homolog	57304.21	46537.55	46910.79	34618.65	**46343**	41936.85	36596.04	50191.56	55091.61	**45954**	**1.0085**	**0.95213**
P97457	Myl11	Myosin regulatory light chain 11	134109.4	87327.96	77269.09	143272	**110495**	33863.18	54026.15	13510.6	36249.63	**34412**	**3.2109**	**0.00624**
Q6NZM9	Hdac4	Histone deacetylase 4	14816.79	16482.69	14421.08	6417.067	**13034**	15658.18	6921.616	10090.65		**10890**	**1.1969**	**0.55752**
Q8CH77	Nav1	Neuron navigator 1	51634.93	46066.02	42340.18	42911.4	**45738**	54487.93	44718.18	59013.45	37414.41	**48908**	**0.9352**	**0.57173**
P11835	Itgb2	Integrin beta-2	13328.58	6224.308	5322.912	8215.215	**8273**	6157.655	6888.569	5564.066	3644.029	**5564**	**1.4869**	**0.20801**
Q91YS8	Camk1	Calcium/calmodulin-dependent protein kinase type	11913.21	17853.22	16898.49	11406.29	**14518**	12635.51	16685.33	7719.848		**12347**	**1.1758**	**0.49201**
P07310	Ckm	Creatine kinase M-type	92059.84	173554.8	146833	101861.8	**128577**	74699.23	75950.59	81475.87	55219.85	**71836**	**1.7899**	**0.02965**
P06802	Enpp1	Ectonucleotide pyrophosphatase/phosphodiesteras	20401.18	16686.54	20515.38	21684.53	**19822**	15544.53	20984.91	15101.59	19198.47	**17707**	**1.1194**	**0.28275**
P14733	Lmnb1	Lamin-B1	164881.5	170782.9	205289.8	208290	**187311**	179310.9	153361	221609.4	218346.5	**193157**	**0.9697**	**0.77899**
Q9JL56	Gde1	Glycerophosphodiester phosphodiesterase 1	40768.96	36985.5	38408.09	44053.38	**40054**	39232.39	43938.27	46198.13	44673.98	**43511**	**0.9206**	**0.15972**
P24549	Aldh1a1	Aldehyde dehydrogenase 1A1	270488.5	258527.2	179703.5	229537	**234564**	280627.7	242341.1	188147	173609.4	**221181**	**1.0605**	**0.68973**
E9Q634	Myo1e	Unconventional myosin-Ie	40262.21	41944.92	39411.85	43528.86	**41287**	40255.74	43815.48	42609.98	42642.72	**42331**	**0.9753**	**0.41036**
P43275	H1-1	Histone H1.1	1752505	1472825	3057230	2402134	**2171174**	1638342	1413868	3755694	3726228	**2633533**	**0.8244**	**0.55100**
Q99M87	Dnaja3	DnaJ homolog subfamily A member 3, mitochondrial	68336.65	74567.71	70249.43	75152.42	**72077**	68799.61	70709.73	56096.9	72643.83	**67063**	**1.0748**	**0.26612**
P51954	Nek1	Serine/threonine-protein kinase Nek1	15555.77	14068.57	15360.85	15363.59	**15087**	13555.17	17417.49	16036.99	15277.29	**15572**	**0.9689**	**0.59971**
Q9D5V5	Cul5	Cullin-5	24425.96	25105.58	27764.14	21611.61	**24727**	24169.41	23641.4	19633.2	23693.88	**22784**	**1.0852**	**0.28306**
O35075	Vps26c	Vacuolar protein sorting-associated protein 26C	21367.88	21547.08	22371.65	19533.17	**21205**	22412.96	20875.71	18741.22	27596.96	**22407**	**0.9464**	**0.56602**
A6H5Z3	Exoc6b	Exocyst complex component 6B	12735.42	13164.51	13603.86	10834.75	**12585**	11747.18	14191.33	14069.63	11711.29	**12930**	**0.9733**	**0.72139**
P55288	Cdh11	Cadherin-11	29596.59	29160.97	27383.95	26660.37	**28200**	29622.8	26455.92	24912.73	29204.82	**27549**	**1.0236**	**0.64068**
Q3UGR5	Hdhd2	Haloacid dehalogenase-like hydrolase domain-conta	68980.31	65866.66	63029.2	46088.52	**60991**	59214.41	58274.72	59164.2	51176.73	**56958**	**1.0708**	**0.48864**
P61202	Cops2	COP9 signalosome complex subunit 2	39645.66	34762.36	37715.03	36290.55	**37103**	35003.71	34675.63	33856.88	32179.17	**33929**	**1.0936**	**0.04014**
P58137	Acot8	Acyl-coenzyme A thioesterase 8	15005.35	13742.12	14450.27	16394.16	**14898**	15602.98	15045.18	15411.49	16844.63	**15726**	**0.9473**	**0.27159**
Q80W47	Wipi2	WD repeat domain phosphoinositide-interacting pro	36525.33	35968.3	35903.7	34494.19	**35723**	37544.72	36668.26	40772.9	37154.68	**38035**	**0.9392**	**0.06506**
Q9JKZ2	Slc5a3	Sodium/myo-inositol cotransporter	5649.458	5300.694	3493.492	3020.207	**4366**	5073.473	5689.859	2350.152	3641.889	**4189**	**1.0423**	**0.86417**
Q99LG2	Tnpo2	Transportin-2	29819.51	32305.71	25315.43	24129.82	**27893**	26546.14	32025.24	19434.17	23726.39	**25433**	**1.0967**	**0.47920**
Q61553	Fscn1	Fascin	263853.9	291537.9	365993.1	274717.3	**299026**	286505.8	268270.2	335383.8	362023.9	**313046**	**0.9552**	**0.67274**
Q9CQ02	Commd4	COMM domain-containing protein 4	9367.188	12700.08	13790.52	7639.125	**10874**	11368.54	8301.06	10794.67	11149.7	**10403**	**1.0452**	**0.77822**
Q8BX17	Gemin5	Gem-associated protein 5	11938.29	11047.86	12307.7	11365.62	**11665**	12980.9	12410.57	9975.229	12249.06	**11904**	**0.9799**	**0.75102**
P18052	Ptpra	Receptor-type tyrosine-protein phosphatase alpha	37352.09	33759.9	32215.1	36866.82	**35048**	33086.32	32781.57	25190.62	36247.74	**31827**	**1.1012**	**0.27000**
Q8C854	Myef2	Myelin expression factor 2	189527.5	202017	224201	225826.2	**210393**	204880.3	211488	227167.1	236596.3	**220033**	**0.9562**	**0.43057**
Q148V7	Relch	RAB11-binding protein RELCH	30344.92	31357.35	29832.12	27640.42	**29794**	28520.18	31811.77	28506.63	26421.59	**28815**	**1.0340**	**0.49960**
Q9CQ85	Timm22	Mitochondrial import inner membrane translocase s	29127.32	28400.05	35827.79	31504.73	**31215**	31134.07	26151.99	34563.17	35805.84	**31914**	**0.9781**	**0.80672**
Q0GNC1	Inf2	Inverted formin-2	61778.6	62929.91	62211.13	59280.2	**61550**	54539.37	61386.07	56210.59	68190.19	**60082**	**1.0244**	**0.65968**
P62322	Lsm5	U6 snRNA-associated Sm-like protein LSm5	22755.61	25194.14	26589.36	21588.44	**24032**	25463.02	24774.64	27977.62	25333.29	**25887**	**0.9283**	**0.21584**
P10833	Rras	Ras-related protein R-Ras	227448.1	213006.5	187302	232726.1	**215121**	217621.4	219767.4	192419.7	192983.4	**205698**	**1.0458**	**0.48428**
P09925	Surf1	Surfeit locus protein 1	33540.58	34128.95	33755.37	38399.59	**34956**	33165.5	39510.15	47388.24	43852.77	**40979**	**0.8530**	**0.11532**
P15379	Cd44	CD44 antigen	182131.8	170192.3	169315.3	185822.6	**176866**	170056.5	175578.8	168638.3	181756.3	**174007**	**1.0164**	**0.59796**
Q6X893	Slc44a1	Choline transporter-like protein 1	252670	228649.4	160804.3	266028.6	**227038**	213354.2	261714.4	184076.3	199237.3	**214596**	**1.0580**	**0.68085**
Q8C3F2	Fam120c	Constitutive coactivator of PPAR-gamma-like proteir	78609.65	70777.61	81527.1	82762.39	**78419**	77332.5	80545.64	79234.45	87073.27	**81046**	**0.9676**	**0.47188**
Q9Z0S1	Bpnt1	3'(2'),5'-bisphosphate nucleotidase 1	69786.45	74873.1	75578.32	63434.64	**70918**	75382.55	63457.2	73032.91	67645.98	**69880**	**1.0149**	**0.79818**
Q9CPT4	Mydgf	Myeloid-derived growth factor	48477.39	48993.25	56348.76	59956.84	**53444**	53451	49618.87	52108.7	65011.62	**55048**	**0.9709**	**0.72966**
Q9ES97	Rtn3	Reticulon-3	240392.8	229365.2	213579.1	266916	**237563**	216109.8	250806.1	236367.5	248033.2	**237829**	**0.9989**	**0.98517**
A2APT9	Klhdc7a	Kelch domain-containing protein 7A												
P54227	Stmn1	Stathmin	397938.3	450996.8	522528.7	401830.1	**443323**	371254.4	331121.6	402828.8	437957.3	**385791**	**1.1491**	**0.16982**
P24547	Impdh2	Inosine-5'-monophosphate dehydrogenase 2	39635.95	38199.04	36216.7	37803.77	**37964**	33942.07	36427.94	32159.37	34439	**34242**	**1.1087**	**0.01621**
P24452	Capg	Macrophage-capping protein	207360.3	198817.3	193290.7	179525.2	**194748**	202758.3	190307.3	173508.8	174200.3	**185194**	**1.0516**	**0.33592**
Q80VJ2	Sra1	Steroid receptor RNA activator 1	13095.1	11832.06		6994.484	**10641**	9562.063				**9562**	**1.1128**	

Q5XJV6	Lmtk3	Serine/threonine-protein kinase LMTK3												
O35488	Slc27a2	Long-chain fatty acid transport protein 2											
Q9JI39	Abcb10	ATP-binding cassette sub-family B member 10, mitoc	6955.481	7133.539	8486.596	12956.84	8883	11992.25	8848.322	13986.11	13519.22	12086	0.7350	0.12863
P11627	L1cam	Neural cell adhesion molecule L1	181424.4	171528.4	167638	207925.2	182129	181100.8	179644.8	175173.2	180379.1	179074	1.0171	0.75046
O70152	Dpm1	Dolichol-phosphate mannosyltransferase subunit 1	39951.56	43986.5	48082.01	49923.39	45486	40044.51	46226.62	40307.75	49024.98	43901	1.0361	0.63236
Q9WTX6	Cul1	Cullin-1	27238.54	28327.56	26155.37	24948.38	26667	25551.75	25355.49	24046.71	25791.91	25186	1.0588	0.12202
Q8C0Y0	Ppp4r4	Serine/threonine-protein phosphatase 4 regulatory s	38098.77	36801.45	11921.43	30188.9	29253	32201.1	37593.9	33748.41	31567.13	33778	0.8660	0.49170
Q9JM76	Arpc3	Actin-related protein 2/3 complex subunit 3	176237.8	194659.2	170851.1	169057	177701	172941.8	154238.2	196268.9	176838.6	175072	1.0150	0.80916
Q8CCT4	Tceal5	Transcription elongation factor A protein-like 5	13334.62	15629.24	23658.41	18821.3	17861	13913.79	14255.07	16000.03	15879.59	15012	1.1898	0.26185
Q91V76		Ester hydrolase C11orf54 homolog	44471.55	49006.33	47573.57	37608.69	44665	40837.04	49715.07	46231.61	39612.3	44099	1.0128	0.87557
Q8BMK4	Ckap4	Cytoskeleton-associated protein 4	81924.96	84760.54	124590.6	96948.01	97056	97572.19	84955.56	120737.2	135925.7	109798	0.8840	0.42880
P61329	Fgf12	Fibroblast growth factor 12	31639.94	35098.09	30730.12	36520.29	33497	26722.2	36381.51	28316.63	31091.26	30628	1.0937	0.29978
Q61586	Gpam	Glycerol-3-phosphate acyltransferase 1, mitochondi	20772.65	14752.49	17815.13	19280.97	18155	16866.38	18022.98	22032.44	20208.97	19283	0.9415	0.53735
Q3UHH1	Zswim8	Zinc finger SWIM domain-containing protein 8	12116.21	12297.57	8268.696	9063.611	10437	6984.222	8831.075		8594.581	8137	1.2827	0.14143
Q7TQG1	Plekha6	Pleckstrin homology domain-containing family A mer	22739.66	24912.86	23810.6	25487.01	24238	21677.15	24677.06	21929.29	29981.55	24566	0.9866	0.87621
P70318	Tial1	Nucleolysin TIAR	29328.03	31483.03	30771.29	33356.73	31235	32831.34	31957.75	37317.88	32640.23	33687	0.9272	0.14949
Q9WV98	Timm9	Mitochondrial import inner membrane translocase s	24739.5	28562.89	26354.57	24151.96	25952	27593.23	29705.15	25950.15	34518.87	29442	0.8815	0.14835
Q9EQH2	Erap1	Endoplasmic reticulum aminopeptidase 1	40060.93	34528.56	38446.13	36194.36	37307	36811.23	36329.59	34798.84	36546.43	36122	1.0328	0.39689
Q8R3L2	Tcf25	Ribosome quality control complex subunit TCF25	16591.39	16482.72	16321.07	15497.16	16223	17145.02	16884.19	15812.67	17630.55	16868	0.9618	0.20826
P62862	Fau	Ubiquitin-like FUBI-ribosomal protein eS30 fusion pr	370833.4	336534.5	472916.5	646219.1	456626	368195.5	345555.5	502587.3	601381.8	454430	1.0048	0.98170
Q9CRA7	Dmac2l	ATP synthase subunit s, mitochondrial	21269.91	24073.84	23422.1	24286.78	23263	24260.11	27422.86	23619.01	24485.48	24947	0.9325	0.17369
Q7TNM2	Trim46	Tripartite motif-containing protein 46	14139.67	15526.38	19570.78	20739.88	17494	16449.13	17044.52	15995.99	21155.53	17661	0.9905	0.93532
Q9D7H3	RtcA	RNA 3'-terminal phosphate cyclase	51887.95	47443.94	45508.63	44471.37	47328	48039.74	49384.94	50152.38	49566.9	49286	0.9603	0.29317
Q8CCF0	Prpf31	U4/U6 small nuclear ribonucleoprotein Prp31	20531.12	20224.25	18554.92	20510	19955	18661.93	19385.63	21088.61	22043	20295	0.9833	0.72061
Q9DCC4	Pycr3	Pyrroline-5-carboxylate reductase 3	39947.14	39480.8	42590.7	38099.84	40030	41591.53	34957.92	39462.21	39423.97	38859	1.0301	0.51252
O70481	Ubr1	E3 ubiquitin-protein ligase UBR1	14690.66	13528	11290.88	9903.723	12353	12688.22	13809.36	9301.35	13451.37	12313	1.0033	0.97910
Q99KN9	Clint1	Clathrin interactor 1	46104.23	47390.1	46751.76	46595.91	46711	41079.73	41506.47	43450.17	48576.32	43653	1.0700	0.12951
Q9DCM7	Nacc2	Nucleus accumbens-associated protein 2	19436.08	17052.78	22162.58	16952.82	18901	20590.09	18430.63	23962.99	22044.2	21257	0.8892	0.21413
P28352	Apex1	DNA-(apurinic or apyrimidinic site) endonuclease	49384.93	52068.72	55009.59	44198.68	50165	50112.16	49084.53	54423.02	50054.03	50918	0.9852	0.78085
Q8BU14	Sec62	Translocation protein SEC62	25647.59	28704.69	25367.61	27165.61	26721	23456.47	22922.71	24461.17	26818.05	24415	1.0945	0.09303
Q99N94	Mrpl9	Large ribosomal subunit protein bL9m	24226.13	21439.42	26033.36	26836.96	24634	20008.13	22640.19	23837.96	24642.72	22782	1.0813	0.28210
P20152	Vim	Vimentin	53203008	49868508	52907196	56008920	52996908	51630216	52656932	52040836	53741632	52517404	1.0091	0.73209
P62702	Rps4x	Small ribosomal subunit protein eS4	678043.6	671605.6	770075.1	763672.2	720849	745475.9	726836.8	828018.8	825383.8	781429	0.9225	0.15749
Q9JLB0	Pals2	Protein PALS2	240143.6	236430.1	225541.8	285463.3	246895	249729	250682	244824	252382.9	249404	0.9899	0.85680
Q80TS3	Adgrl3	Adhesion G protein-coupled receptor L3	4692.341	13203.89	6053.989	1300.143	6313	5495.706	8375.627	4347.97	5029.256	5812	1.0861	0.85683
Q9CZL5	Pcbd2	Pterin-4-alpha-carbinolamine dehydratase 2	26524.23	25158.19	23095.43	30595.61	26343	26608.63	26529.64	26270.01	24814.91	26056	1.0110	0.86639
Q61285	Abcd2	ATP-binding cassette sub-family D member 2	12423.49	8283.324	8035.463	6061.82	8701			11131.44	7232.458	9182	0.9476	0.84678
Q61301	Ctnna2	Catenin alpha-2	37601.39	31772.59	30182.43	24932.79	31122	22191.87	32210.97	24838.29	28006.25	26812	1.1608	0.24985
P29758	Oat	Ornithine aminotransferase, mitochondrial	232037	243029.7	276222.8	279134.8	257606	248921.1	258482	257867.9	270381	258913	0.9950	0.92085
Q9CWM4	Pfdn1	Prefoldin subunit 1	35280.49	38366.79	36845.84	26819.82	34328	33483.9	38957.49	45262.67	35099.08	38201	0.8986	0.33281
P83940	Eloc	Elongin-C	44711.29	46303.62	43335.46	37309.02	42915	43156.14	44740.58	41168.13	40039.29	42276	1.0151	0.78366
Q9DBN4	P33monox	Putative monooxygenase p33MONOX												
Q9CRT8	Xpot	Exportin-T	12194.24	9131.923	9854.025	8951.613	10033	10543.07	9844.807	10470.3	7667.769	9631	1.0417	0.70339
Q3TRM4	Pnpla6	Patatin-like phospholipase domain-containing prote	28192.63	28378.92	26879.96	30666.55	28530	26045.11	28608.85	29948.88	31852.89	29114	0.9799	0.70125
Q99MR8	Mccc1	Methylcrotonoyl-CoA carboxylase subunit alpha, mit	48829.57	52652.5	48184.56	58521.73	52047	46361.79	56839.66	50541.14	47941.25	50421	1.0323	0.64059
Q7TPB0	Plppr3	Phospholipid phosphatase-related protein type 3												
Q9Z2C9	Mtmr7	Myotubularin-related protein 7	35242.87	34374.75	36035.64	28107.16	33440	32810.21	33528.2	33495.5	32001.9	32959	1.0146	0.80298
Q9CYV5	Tmem135	Transmembrane protein 135									69467.07	69467	0.0000	
Q8K1M6	Dnm1l	Dynamin-1-like protein	217302	238559.9	222659.3	180258.5	214695	208939.2	219676.7	201146.6	192618.4	205595	1.0443	0.52866
Q8BY87	Usp47	Ubiquitin carboxyl-terminal hydrolase 47	19791.03	18282.85	15899.65	13585.62	16890	15644.8	18615.64	16522.68	17448.28	17058	0.9901	0.91463
E9Q735	Ube4a	Ubiquitin conjugation factor E4 A	54306.71	57051.41	62458.5	60364.96	58545	53071.47	58125.27	54182.89	62762.4	57036	1.0265	0.61383
P56564	Slc1a3	Excitatory amino acid transporter 1	27234.16	31570.01	21209.13	20605.63	25155	18102.29	36251.48	36161.04	24777.53	28823	0.8727	0.50545
P62827	Ran	GTP-binding nuclear protein Ran	405186.7	427548.8	450604.9	357307.7	410162	413202	371929.1	402752.7	379120.6	391751	1.0470	0.43777
Q9D2V7	Coro7	Coronin-7	45811.59	41254.71	41824.24	35649.85	41135	43008.54	38688.99	45520.36	41036.32	42064	0.9779	0.72778
O70378	Emc8	ER membrane protein complex subunit 8	34161.57	34938.44	34346.03	43770.84	36804	36949.92	38746.2	38048.68	46170.35	39979	0.9206	0.35005
P59108	Cpne2	Copine-2	58831.7	58995.08	52536.24	62798.36	58290	59043.16	56731.72	58346.48	55219.41	57335	1.0167	0.69129
Q8C129	Lnpep	Leucyl-cystinyl aminopeptidase	141178.4	135722.5	144435.1	155974	144328	144247.1	158883.1	136953	148422	147126	0.9810	0.67084
Q9JLV1	Bag3	BAG family molecular chaperone regulator 3	73481.19	81263.59	67606.15	91084.66	78359	72903.19	82991.33	89731.7	81038.77	81666	0.9595	0.61005
P17426	Ap2a1	AP-2 complex subunit alpha-1	276988.1	285346.2	295473.2	302446.8	290064	275154.2	296628.3	293041.9	284223.8	287262	1.0098	0.71715

Q3UUQ7	Pgap1	GPI inositol-deacylase	22979.54	24223.3	26032.96	27234.01	25117	26938.15	29037.49	32720.27	32254.08	30237	0.8307	0.02177
Q80U28	Madd	MAP kinase-activating death domain protein	6345.802	7950.975	5881.642	6289.175	6617	7198.748	4974.413	4923.959	5282.381	5595	1.1827	0.19870
Q8BHI4	Kbtbd3	Kelch repeat and BTB domain-containing protein 3	20516.52			11578.84	16048							
Q923G2	Polr2h	DNA-directed RNA polymerases I, II, and III subunit R	22499.05	23906.17	20856.38	19351.34	21653	19912.6	26711.4	24858.6	19669.09	22788	0.9502	0.59624
Q9Z2C5	Mtm1	Myotubularin	11246.92	16336.64	5801.728	8116.063	10375	11713.81	8841.359	8214.125	8956.861	9432	1.1001	0.70865
Q9R1L5	Mast1	Microtubule-associated serine/threonine-protein kin	17299.82	18204.44	15345.27	14236.97	16272	14968.42	18596.04	16870.35	16232.47	16667	0.9763	0.74850
Q8R1A4	Dock7	Dedicator of cytokinesis protein 7	33302.19	31966.36	32931.13	32181.7	32595	31506.14	33495	29974.29	35191.46	32542	1.0016	0.96530
P51163	Uros	Uroporphyrinogen-III synthase	23236.77	25788.16	17351.84	17245.51	20906	21404.37	19184.48	17547.82	15466.95	18401	1.1361	0.35290
Q6PD24	Ankrd13d	Ankyrin repeat domain-containing protein 13D	15973.9	13807.62	12399.53	14995.22	14294	15715.53	16089.34	10798.38	14468.47	14268	1.0018	0.98604
Q05BC3	Eml1	Echinoderm microtubule-associated protein-like 1	176921.5	174090.4	165921.8	187132.2	176017	180670	188240.6	185673	172024.8	181652	0.9690	0.35714
Q80U95	Ube3c	Ubiquitin-protein ligase E3C	41484.64	40954.85	34887.39	37572.87	38725	40674.94	42432.66	29298.46	31098.27	35876	1.0794	0.46593
Q80WG7	Trim36	E3 ubiquitin-protein ligase Trim36	55349.39	61408.67	61996.54	55019.41	58444	60166.16	60005.68	63134.73	60342.45	60912	0.9595	0.26919
B9EHT4	Clip3	CAP-Gly domain-containing linker protein 3	10986	10809.48	6988.746	9044.068	9457	10784.83	10125.54	8954.133	11543.57	10352	0.9135	0.43972
Q68FM6	Elfn2	Protein phosphatase 1 regulatory subunit 29												
Q9EPL8	Ipo7	Importin-7	13849.26	16313.87	14841.84	9657.056	13666	15772.6	15492.45	13981.92	13646.76	14723	0.9281	0.51375
Q7TNV0	Dek	Protein DEK	104373.5	104094.5	105434.3	107615.1	105379	96630.88	103645.1	91643.87	101115.5	98259	1.0725	0.04164
P49446	Ptpre	Receptor-type tyrosine-protein phosphatase epsilon	44359.38	40190.25	42836.86	41310.07	42174	36679.29	41834.73	36708.73	43401.92	39656	1.0635	0.24683
Q8R3S6	Exoc1	Exocyst complex component 1	22980.48	20619.92	20586.78	19477.03	20916	19174.45	22061.73	17339.93	18338.34	19229	1.0878	0.22756
Q91WE1	Snx15	Sorting nexin-15	4428.791	4217.091	5640.539	3202.302	4372	2238.628	5668.503	2341.51	5025.155	3818	1.1450	0.60784
P61750	Arf4	ADP-ribosylation factor 4	62570.68	79493.98	59592.85	64462.98	66530	73072.04	83404.07	74027.02	57176.68	71920	0.9251	0.47167
Q99K28	Arfgap2	ADP-ribosylation factor GTPase-activating protein 2	18502.44	16705.6	19928.88	15035.87	17543	16225.35	16438.68	20319.4	18065.6	17762	0.9877	0.88281
Q8JZQ9	Eif3b	Eukaryotic translation initiation factor 3 subunit B	107152.2	108940.7	114180.7	116505.3	111695	106575.1	112831.5	120510.2	124333.9	116063	0.9624	0.37211
Q9QX11	Cyth1	Cytohesin-1	51630.75	52977.18	36960.86	41408.41	45744	51951.76	38946.77	46145.54	37316.11	43590	1.0494	0.69120
P53986	Slc16a1	Monocarboxylate transporter 1	94485.97	110636.5	101608.2	107207.5	103485	93934.85	111134.5	94394.73	98593.56	99514	1.0399	0.48560
Q9R0M8	Slc35a2	UDP-galactose translocator	7197.084	9154.784	12660.21	11515.37	10132	9093.43	12670.54	14667.42	15387.33	12955	0.7821	0.18080
Q91YL3	Uckl1	Uridine-cytidine kinase-like 1	25779.29	25996.18	29262.23	27815.79	27213	29487.68	24425.07	23109.29	23282.05	25076	1.0852	0.25777
P53395	Dbt	Lipoamide acyltransferase component of branched-(	39067.69	38664.63	45029.22	46154.45	42229	40071.98	43922.04	48949.94	43405.72	44087	0.9578	0.51402
P22315	Fech	Ferrochelatase, mitochondrial	85114.25	80673.72	78474.24	89011.47	83318	81076.39	85447.81	75662.41	80896.63	80771	1.0315	0.44045
Q9CQA1	Trappc5	Trafficking protein particle complex subunit 5	21911.46	21375.52	22279.7	20290.62	21464	18916.62	21711.77	18596.63	19956.85	19795	1.0843	0.08940
G5E829	Atp2b1	Plasma membrane calcium-transporting ATPase 1	151443.8	151328.6	137391	153213.5	148344	151933.8	156177.7	160911.5	155501.4	156131	0.9501	0.10722
O70362	Gpld1	Phosphatidylinositol-glycan-specific phospholipase	17334.34	18493.52	19067.89	14744.45	17410	15021.63	19629.28	19069.54	17151.4	17718	0.9826	0.83521
Q9CQ92	Fis1	Mitochondrial fission 1 protein	165239.5	179261.8	172809.4	182278.2	174897	170214.5	171736.5	181688.7	203921.8	181890	0.9616	0.44924
P06151	Ldha	L-lactate dehydrogenase A chain	1004794	1007985	1017706	759854.1	947585	1039073	800050.6	994079.8	819511.9	913179	1.0377	0.70651
P10107	Anxa1	Annexin A1	53443.34	48639.03	49179.35	48272.39	49884	40337.27	41283.91	32868.49	32102.65	36648	1.3611	0.00270
P55937	Golga3	Golgin subfamily A member 3	8119.691	7235.101	9333.447	7532.337	8055	7155.429	7947.021	8198.069	8881.019	8045	1.0012	0.98723
Q9D0W5	Ppil1	Peptidyl-prolyl cis-trans isomerase-like 1	12914.03	15056.2	8879.308	11530.57	12095	14678.99	17953.15	12676	13108.57	14604	0.8282	0.20440
Q8CGK7	Gnal	Guanine nucleotide-binding protein G(olf) subunit al	57524.28	64983.23			61254	58404.38	53571.92	9101.173	29413.03	37623	1.6281	0.24356
Q9R1P4	Psma1	Proteasome subunit alpha type-1	134415.9	137730.7	142476.7	120208.9	133708	137261.7	133079.1	139097.7	130493.4	134983	0.9906	0.81371
Q8K4Z5	Sf3a1	Splicing factor 3A subunit 1	29060.67	27626.41	29668.47	25450.54	27952	27599.19	29893.15	32033.76	32513.62	30510	0.9161	0.13119
Q9JIZ9	Plscr3	Phospholipid scramblase 3	100029.3	90732.42	91669.65	98691.1	95281	98414.81	85760.18	90693.77	95880.45	92687	1.0280	0.50786
P38585	Ttl	Tubulin--tyrosine ligase	89767.05	111728.4	94982.74	89410.15	96472	97820.67	105988.4	76081.58	76370.2	89065	1.0832	0.45294
Q9Z0S9	Rabac1	Prenylated Rab acceptor protein 1	61543.5	60384.46	69863.95	43503.39	58824	69987.14	69974.48	49840.92	66698.44	64125	0.9173	0.49704
Q9CRA5	Golph3	Golgi phosphoprotein 3	39837.99	43460.22	40243.92	43355.08	41724	42332.88	44471.48	44194.48	45067.07	44016	0.9479	0.09110
Q64444	Ca4	Carbonic anhydrase 4		9657.716		3215.298	6437	5298.606	5473.216		3587.673	4786	1.3447	0.55984
Q9Z1W8	Atp12a	Potassium-transporting ATPase alpha chain 2	101704.9	93647.27	82534.44	122727.1	100153	96301.39	105507.3	90019.5	63463.14	88823	1.1276	0.39589
E9Q0S6	Tns1	Tensin-1	198932.7	207306.8	189302.3	226929	205618	204052.6	204638.6	216813.1	217809.1	210828	0.9753	0.57686
Q9Z1N5	Ddx39b	Spliceosome RNA helicase Ddx39b	315793.1	315310.9	369300.4	317162.4	329392	346386.5	315209.9	360113.2	364180.3	346472	0.9507	0.36229
O55203	Ldb2	LIM domain-binding protein 2	13983.74	14603.37	15001.89	15392.26	14745	13647	14832.35	16361.59	16625.14	15367	0.9596	0.44400
P98203	Arvcf	Splicing regulator ARVCF	22275.41	24565.65	25965.46	27415.46	25055	23017.67	22693.77	26516.88	27882.73	25028	1.0011	0.98743
Q9R1T2	Sae1	SUMO-activating enzyme subunit 1	49394.97	53521.08	53250.18	41398.74	49391	49679.45	46995.93	49776.5	45549.98	48000	1.0290	0.66046
Q6NZL0	Soga3	Protein SOGA3	65082.64	68824.85	68352.77	78666.2	70232	70499.51	72872.22	77512.71	73724.85	73652	0.9536	0.33635
Q9CRD2	Emc2	ER membrane protein complex subunit 2	37308.47	40698.38	44818.13	44212.47	41759	42273.01	44603.81	45527.05	47153.02	44889	0.9303	0.17151
P62911	Rpl32	Large ribosomal subunit protein eL32	300049.7	303696.8	292988.3	320746.2	304370	288172.9	313442.4	308076.6	338218.3	311978	0.9756	0.54513
Q91YH5	Atl3	Atlastin-3	90233.59	90399.15	96428.1	103463.4	95131	93925.77	98128.7	100032.2	104115.3	99050	0.9604	0.33944
Q9DCZ1	Gmpr	GMP reductase 1	39935.43	38119.95	28436.49	28338.75	33708	37409.77	36174.16	21120.23	20921.09	28906	1.1661	0.41708
Q9D0F3	Lman1	Protein ERGIC-53	112906.1	98543.3	115033.2	128551	113758	105377.3	106881.9	113955.5	126442	113164	1.0053	0.94174
Q03137	Epha4	Ephrin type-A receptor 4												
Q9CQX8	Mrps36	Alpha-ketoglutarate dehydrogenase component 4	43880.8	41753.8	48181.71	42363.03	44045	43637.78	42095.7	50258.36	57178.26	48293	0.9120	0.29972
Q9Z1K6	Arih2	E3 ubiquitin-protein ligase ARIH2		12194.91		11435.54	11815	14905.93				14906	0.7927	

Q91VF2	Hnmt	Histamine N-methyltransferase	8224.229	9955.564	11354.87	9007.537	9636	8651.829	10013.37	6953.407	9793.669	8853	1.0884	0.45128
Q78IK4	Apool	MICOS complex subunit Mic27	18872	19835.42	21848.56	22008.71	20641	19378.54	19852.41	21884.76	23423.11	21135	0.9766	0.69805
P70232	Chl1	Neural cell adhesion molecule L1-like protein	86788.2	86479.84	74582.51	104146.2	87999	87409.04	89434.57	83756.66	79086.98	84922	1.0362	0.65246
Q9D1G3	Hhatl	Protein-cysteine N-palmitoyltransferase HHAT-like p	12384.08	12704.96	13072.58	14908.24	13267	7929.303	14266.37	15656.22	14958.92	13203	1.0049	0.97347
Q9JIG8	Praf2	PRA1 family protein 2	79160.3	96934.02	89492.97	86360.16	87987	89870.63	89576.74	97187.03	108152.9	96197	0.9147	0.20023
Q8CE08	Acp3	Prostatic acid phosphatase	84467.91	72353.76	64118.1	88100.77	77260	97582.86	100144.8	86754.66	91370.7	93963	0.8222	0.03796
Q80U57	Rims3	Regulating synaptic membrane exocytosis protein 3	18478.7	22065.53	22246.38	21604.47	21099	19003.76	22911.07	18810.06	18148.08	19718	1.0700	0.36071
Q5SXY1	Specc1	Cytospin-B	47615.86	43883.23	32884.41	32678.1	39265	30688.24	42219.16	30414.71	25096.73	32105	1.2230	0.22196
Q9WV32	Arpc1b	Actin-related protein 2/3 complex subunit 1B	231889.6	228317.8	244786.3	198445.3	225860	218839.4	208357.2	237260.4	230950	223852	1.0090	0.86957
Q61503	Nt5e	5'-nucleotidase	90890.55	73941.25	81995.49	89164.13	83998	81309.1	74281.28	84442.33	77239.14	79318	1.0590	0.33497
Q9QXB9	Drg2	Developmentally-regulated GTP-binding protein 2	59072.53	54580.95	59387.68	52080.32	56280	59374.76	55519.13	59985.15	52304.55	56796	0.9909	0.84507
O88448	Klc2	Kinesin light chain 2	60582.88	62432.34	46262.11	51970.44	55312	51374.36	54708.09	51206.02	50308.7	51899	1.0658	0.41545
Q8K596	Slc8a2	Sodium/calcium exchanger 2		56104.34			56104		17265.7			17266	3.2495	
Q9WTI7	Myo1c	Unconventional myosin-Ic	307319.5	279864.6	305893	345989.2	309767	300805.4	289583.1	299844.2	316100.7	301583	1.0271	0.59736
B2RQC6	Cad	CAD protein	60998.21	61541.62	65758.08	54557.73	60714	58309.64	58560.95	55650.73	60017.11	58135	1.0444	0.33909
Q641K5	Nuak1	NUAK family SNF1-like kinase 1	11377.05	10152.02	11736.06	8577.588	10461	9740.863	9329.553	9035.252	9194.743	9325	1.1218	0.17043
Q6NZB0	Dnajc8	DnaJ homolog subfamily C member 8	28283.03	35123.18	34175.37	23012.78	30149	26433.61	26350.1	21453.81	20790.79	23757	1.2690	0.09326
Q9ERL9	Gucy1a1	Guanylate cyclase soluble subunit alpha-1		9205.596			9206				7106.723	7107	1.2953	
Q8VH51	Rbm39	RNA-binding protein 39	22540.47	23792.34	23306.99	25633.33	23818	22896.2	25682.44	25921.73	25697.79	25050	0.9508	0.25351
Q9Z2D6	Mecp2	Methyl-CpG-binding protein 2	81502.34	77742.38	60956.68	82868.42	75767	79206.27	86417.02	51348	58959.05	68983	1.0984	0.50999
Q91VS7	Mgst1	Microsomal glutathione S-transferase 1	54726.29	54794.54	58377.09	52796.5	55174	51382.4	54113.78	39833.82	47301.41	48158	1.1457	0.07892
Q9D2M8	Ube2v2	Ubiquitin-conjugating enzyme E2 variant 2	207331.9	239720.2	188281.4	152005.3	196835	215191.1	213475.5	207730.8	178981.5	203845	0.9656	0.74018
Q04447	Ckb	Creatine kinase B-type	1275802	1480047	1180140	1090358	1256586	1230291	1324415	1035046	1054305	1161014	1.0823	0.41425
Q9JJ28	Flii	Protein flightless-1 homolog	26312.79	27344.01	28476.89	30917.74	28263	27747.67	27280.34	26230.71	26307.88	26892	1.0510	0.24212
Q9DCM0	Ethe1	Persulfide dioxygenase ETHE1, mitochondrial	37424.66	37547.76	39519.26	42447.17	39235	35771.62	41500.2	36252.52	39382.25	38227	1.0264	0.59396
Q5SNZ0	Ccdc88a	Girdin			7559.708		7560				3812.33	3812	1.9830	
E9Q3L2	Pi4ka	Phosphatidylinositol 4-kinase alpha	19334.01	18199.73	19287.41	17695.85	18629	18534.78	18982.26	16936.13	16402.74	17714	1.0517	0.26304
Q6P2L7	Golm2	Protein GOLM2	11364.94	15354.62	12839.91	13759.51	13330	9850.762	15629.73	11147.59	11742.41	12093	1.1023	0.44057
P0C0A3	Chmp6	Charged multivesicular body protein 6	25827.99	23737.74	24430.59	25579.75	24894	22283.9	26882.34	15349.56	21283.25	21450	1.1606	0.20471
B2RY56	Rbm25	RNA-binding protein 25	54462.9	52594.9	47860.28	58607.18	53381	48384.82	57500.59	56505.9	59453.23	55461	0.9625	0.55200
Q02257	Jup	Junction plakoglobin	40176.76	40330.64	39430.38	45116.1	41263	36574.1	39000.16	37738.71	52730.17	41511	0.9940	0.95259
Q52KF3	Spire1	Protein spire homolog 1	3615.465	4536.103	3683.658	3492.347	3832	2885.829	2927.338		4310.953	3375	1.1355	0.38690
Q9QY36	Naa10	N-alpha-acetyltransferase 10	30254.41	30265.84	33120	28238.77	30470	31701.49	29353.04	30818.79	32938.92	31203	0.9765	0.58050
P47811	Mapk14	Mitogen-activated protein kinase 14	14706.03	14007.54	16324.36	11197.69	14059	12067.08	13451.65	13915.77	12318.84	12938	1.0866	0.37072
Q9D2G2	Dlst	Dihydrolipoyllysine-residue succinyltransferase com	459706.8	439865.8	517715.9	551691.2	492245	494653.9	520894.3	503645.6	530700.9	512474	0.9605	0.48291
Q8VDN4	Ccdc92	Coiled-coil domain-containing protein 92	77239.82	83371.05	86813.98	95030.27	85614	85269.52	82066.73	83953.77	90563.91	85463	1.0018	0.97218
Q6PCN3	Ttbk1	Tau-tubulin kinase 1	4106.595	5613.879	8213.356	8266.594	6550	2446.695	2090.73	4514.564	9083.6	4534	1.4447	0.33081
P62962	Pfn1	Profilin-1	572871.1	662618.8	698701.3	461975.8	599042	563359.2	607296.7	501450.8	543252.1	553840	1.0816	0.45941
Q01063	Pde4d	cAMP-specific 3',5'-cyclic phosphodiesterase 4D		674.0197			674				6587.062	6587	0.1023	
Q9R0U0	Srsf10	Serine/arginine-rich splicing factor 10	32240.48	36739.57	36174.82	43646.86	37200	37170.58	37568.8	36796.69	39536.18	37768	0.9850	0.82431
Q6P8J7	Ckmt2	Creatine kinase S-type, mitochondrial	100862.3	90234.95	110501.4	108622.4	102555	83202.14	89765.72	71413.87	91392.69	83944	1.2217	0.02810
Q9QYK7	Rnf11	RING finger protein 11	12115.95	17768.6	11546.36	16142.94	14393	10630.2	10186.74	14376.67	13255.76	12112	1.1883	0.25849
Q6ZWM4	Lsm8	U6 snRNA-associated Sm-like protein LSm8	24974.4	32648.53	27141.04	24664.93	27357	25996.04	28195.45	21608.01	22253.99	24513	1.1160	0.28444
Q3UMB5	Smcr8	Guanine nucleotide exchange protein SMCR8						3439.7			5057.781	4249	0.0000	
Q9DBG9	Tax1bp3	Tax1-binding protein 3	204096.1	181956.5	222867.2	206888.5	203952	186572.9	198139.1	205139	213698.9	200887	1.0153	0.77366
Q91VJ5	Pqbp1	Polyglutamine-binding protein 1	14522.28	13927.62	14721.84	14959.03	14533	11962.99	6271.895	19731.11	21982.63	14987	0.9697	0.90416
Q6PGL7	Washc2	WASH complex subunit 2	39762.7	41353.46	38514.63	40126.93	39939	41203.4	42663.62	42571.19	43064.99	42376	0.9425	0.01403
P68373	Tuba1c	Tubulin alpha-1C chain	1173104	1048490	1111830	3226030	1639863	6721680	984494.9	680162.3	3087494	2868458	0.5717	0.44079
Q9Z1D1	Eif3g	Eukaryotic translation initiation factor 3 subunit G	76630.27	81580.46	84935.61	89210.52	83089	82682.22	82001.45	88039.82	94121.91	86711	0.9582	0.38573
Q99LJ1	Fuca1	Tissue alpha-L-fucosidase	33684.5	25486.46	30138.86	26160.89	28868	32481.15	29580.83	30635.41	34283.82	31745	0.9094	0.23295
Q9JJF0	Nap1l5	Nucleosome assembly protein 1-like 5			6030.278		6030	4235.507		2560.523	2916.073	3237	1.8627	
Q3UM45	Ppp1r7	Protein phosphatase 1 regulatory subunit 7	99586.06	107561.8	91301.04	86072.02	96130	93467.22	87022.62	94142.94	84010.48	89661	1.0722	0.27023
Q80VP0	Tecpr1	Tectonin beta-propeller repeat-containing protein 1	18022.29	20149.38	19276.43	16239.52	18422	17668.7	17619.82	14321.4	16895.35	16626	1.1080	0.17202
Q80TJ1	Cadps	Calcium-dependent secretion activator 1	109926.6	111421.7	91936.73	87543.88	100207	95918.91	103728.1	93488.05	84052.88	94297	1.0627	0.45131
Q8BP00	Iqcb1	IQ calmodulin-binding motif-containing protein 1	11762.26	12638.13			12200							
Q9JKF1	Iqgap1	Ras GTPase-activating-like protein IQGAP1	148745.8	139466.3	159001.5	122087.9	142325	134443.4	132732.9	147005.2	134804.3	137246	1.0370	0.57189
Q8BRK9	Man2a2	Alpha-mannosidase 2x	8619.089	8005.921	11655.43	12794.52	10269	9823.767	10487.26	13351.74	14451.39	12029	0.8537	0.31557
Q9CQH3	Ndufb5	NADH dehydrogenase [ubiquinone] 1 beta subcomp	124310.9	125314.2	126375.9	140012.7	129003	112835.4	123697.7	139838.1	149158.1	131382	0.9819	0.79858
Q31125	Slc39a7	Zinc transporter SLC39A7	25772.85	26389.81	28439.86	30247.31	27712	26072.69	26549.05	26081.01	32007.55	27678	1.0013	0.98492

Q99JI6	Rap1b	Ras-related protein Rap-1b	106170	88221.58	85876.21	102594.2	95715	81908.38	98799.79	70180.12	85692.2	84145	1.1375	0.18763
Q99JG2	Gpr37l1	G-protein coupled receptor 37-like 1	7340.812	6911.933	6586.392	8906.674	7436	9066.133	8979.082	4745.596	5633.871	7106	1.0465	0.79785
Q571J5	Znf354c	Zinc finger protein 354C	31206.22	39193.18	45824.26	41880	39526	49065.5	43849.98	32631.34	41890.16	41859	0.9443	0.63118
Q9JK48	Sh3glb1	Endophilin-B1	28966.12	31104.21	32149.58	29240.9	30365	31424.23	29619.87	32042.55	35125.76	32053	0.9473	0.26579
Q3UYC0	Ppm1h	Protein phosphatase 1H	9154.065	10069.48	7536.771	8639.091	8850	9286.616	10370.58	6352.602	6426.148	8109	1.0914	0.54198
Q922X9	Prmt7	Protein arginine N-methyltransferase 7	7735.162	6928.694	5098.982	6055.627	6455	7113.487	7684.193	3862.847	6076.847	6184	1.0437	0.79904
P45376	Akr1b1	Aldo-keto reductase family 1 member B1	521642.2	485219.3	491066.9	418144.8	479018	501716.8	420226.2	335508.7	441833.6	424821	1.1276	0.23163
Q9Z2I8	Suclg2	Succinate--CoA ligase [GDP-forming] subunit beta, r	18205.92	13554.28	14312.59	14075.5	15037	14667.72	19146.42	12421.94	12396.1	14658	1.0259	0.84955
Q8K4G5	Ablim1	Actin-binding LIM protein 1	28831.17	31402.41	27910.12	36112.08	31064	29285.83	31868.01	25732.69	29441.5	29082	1.0681	0.40837
Q9CX34	Sugt1	Protein SGT1 homolog	154587.6	165702.2	149531.6	108067	144472	163070.9	134598.2	129493.9	135403.3	140642	1.0272	0.80321
Q8VHY0	Cspg4	Chondroitin sulfate proteoglycan 4	55866.13	48424.28	50342.14	55167.23	53356	52508.39	53829.57	50342.14	54347.93	52757	1.0113	0.76476
O35609	Scamp3	Secretory carrier-associated membrane protein 3	25814.73	25380.36	31891.1	28677.1	27941	26886.64	31301.08	35046.52	34763.46	31999	0.8732	0.14572
Q9DBF1	Aldh7a1	Alpha-aminoadipic semialdehyde dehydrogenase	168265.5	170625.2	164346.4	155264.6	164625	173223.5	156578.7	158906.6	157698.2	161602	1.0187	0.57938
Q9CYL5	Glipr2	Golgi-associated plant pathogenesis-related protein	26877.51	23575	27022.77	25555.37	25758	24074.71	25283.41	27530.84	25122.58	25503	1.0100	0.82140
Q921J2	Rheb	GTP-binding protein Rheb	38566.04	42734.05	41783.34	41536.47	41155	40944.82	39781.95	37949.28	41671.02	40087	1.0266	0.41225
P50544	Acadvl	Very long-chain specific acyl-CoA dehydrogenase, m	140234.9	143485.9	134720.8	162666.7	145277	132766.7	155466.3	154352.3	148678.6	147816	0.9828	0.76216
Q569Z6	Thrap3	Thyroid hormone receptor-associated protein 3	38830.8	38484.43	39429.64	45707.87	40613	36706.8	32674.18	50786.68	40240.99	40102	1.0127	0.90805
Q8CCA0	Dcun1d4	DCN1-like protein 4	8085.544	9935.853	13682.79		10568	8851.299	8272.079	6389.685	8283.603	7949	1.3295	0.14487
O35639	Anxa3	Annexin A3	168346.8	146629.4	110082.6	123011.9	137018	134577.8	136267	88941.4	75212.2	108750	1.2599	0.21285
Q91W86	Vps11	Vacuolar protein sorting-associated protein 11 homo	27837.42	24726.26	27053.46	23744.82	25840	26485.94	28214.82	28242.55	27489.41	27608	0.9360	0.14208
O70503	Hsd17b12	Very-long-chain 3-oxoacyl-CoA reductase	25305.39	28081.39	34055.41	29157.37	29150	28919.29	26782.82	29508.39	36472.54	30421	0.9582	0.66397
P15331	Prph	Peripherin	5667500	5626370	3804488	6314476	5353208	4979368	5887031	4856137	4696739	5104819	1.0487	0.69433
P12265	Gusb	Beta-glucuronidase	11654.68	12469.6	15155.91	13674.2	13239	12303.29	10792.74	12075.54	12942.26	12028	1.1006	0.22066
Q8C8N2	Scai	Protein SCAI	21521.5	16158.41	15376.51	21793.32	18712	18207.56	15551.51	16640.47	21192.11	17898	1.0455	0.71188
Q8CCJ3	Ufl1	E3 UFM1-protein ligase 1	48403.7	44704.13	52113.62	51980.31	49300	45925.2	44609.04	55992.42	62005.99	52133	0.9457	0.55344
O35841	Api5	Apoptosis inhibitor 5	38712.95	38922.91	42785.5	41023.49	40361	38921.14	38554.14	48777.86	43763.35	42504	0.9496	0.43965
Q09143	Slc7a1	High affinity cationic amino acid transporter 1	23431.22	27157.75	27400.12	23436.37	25356	21092.58	27822.33	23075.88	26980.43	24743	1.0248	0.76313
Q3UMR5	Mcu	Calcium uniporter protein, mitochondrial	71805.37	70150.63	74896.32	86261.09	75778	83029.48	74776.33	89799.09	86101.69	83427	0.9083	0.16499
Q8BH43	Wasf2	Actin-binding protein WASF2	37045.32	35081.25	40017.73	39702.61	37962	39332.64	37012.51	43417.15	39581.21	39836	0.9530	0.32992
Q8BLF1	Nceh1	Neutral cholesterol ester hydrolase 1	92906.35	109911.1	94107.77	109054	98618	94659.43	109911.1	103561.4	99968.49	102025	0.9666	0.51057
A2A5Y0;O76009;Q1452!	Krt31;Krt33b;;Krt33a;;;	Keratin, type I cuticular Ha1;Keratin, type I cuticular I	50517.22	55087.77			52802			29515.63	10140.14	19828	2.6630	0.08030
Q3UGX3	Nat8l	N-acetylaspartate synthetase	31142.21	27601.61	22673.97	28785.84	27551	22856	30842.23	24885.38	29479.22	27016	1.0198	0.84338
Q924Z4	Cers2	Ceramide synthase 2	64056.04	73274.8	85606.06	78324.55	75315	72716.34	74904.63	97061.88	106890.9	87893	0.8569	0.23502
D3YXJ0	Dgkh	Diacylglycerol kinase eta	84254.3	82200.68	75449.77	80820.76	80681	88031.7	79408.32	86493.21	79478.47	83353	0.9679	0.40080
Q8K441	Abca6	ATP-binding cassette sub-family A member 6	53349.57	53084.82	54830.23	58760.95	55006	52759.34	56412.98	52705.2	59920.77	55450	0.9920	0.84458
Q91W97	Hkdc1	Hexokinase HKDC1	73221.41	78223.45	82165.95	67128.61	75185	78667.32	96353.39	58171.81	73034.43	76557	0.9821	0.87753
Q60902	Eps15l1	Epidermal growth factor receptor substrate 15-like 1	34162.24	35541.64	35233.27	31885.13	34206	34093.15	36884.21	32063.32	35394.79	34609	0.9883	0.76954
Q3UQ44	Iqgap2	Ras GTPase-activating-like protein IQGAP2	40742.51	41960.03	39577.84	34763.81	39261	36576.56	38713.56	35486.28	39384.75	37540	1.0458	0.38082
O88737	Bsn	Protein bassoon	65086.29	25285.41		15296.8	35223	25929.38	33461.44	34721.32		31371	1.1228	0.81543
P09055	Itgb1	Integrin beta-1	188228.8	172686.1	185288.6	203989.4	187548	192704.2	198782.6	231759.4	207698.8	207736	0.9028	0.10877
P63038	Hspd1	60 kDa heat shock protein, mitochondrial	328590.5	335155.6	378700.3	411408.8	363464	359740.5	359697.3	373916.9	396370.2	372431	0.9759	0.68848
P03888	Mtnd1	NADH-ubiquinone oxidoreductase chain 1	54815.33	49363.23	56936.91	53738.87	53714	56174.24	64514.98	62544.93	61290.17	61131	0.8787	0.02104
Q8BIV3	Ranbp6	Ran-binding protein 6	28139.91	15402.71	16860.09	14697.29	18775	14560.93	17227.59	19023.02	18607.5	17355	1.0818	0.68292
Q9CPR5	Mrpl15	Large ribosomal subunit protein uL15m	9014.325	10761.69	12743.85	11781.97	11075	10673.23	15175.18	14569.49	16264.13	14171	0.7816	0.07755
Q64362	Aktip	AKT-interacting protein	17085.67	17048.65	17736.69	19270.88	17785	14872.51	14395.79	19508.72	17656.94	16608	1.0709	0.40429
Q01730	Rsu1	Ras suppressor protein 1	204005.7	208435.9	197861.5	189039.5	199836	193589.7	197960.7	230255.1	211270.7	208269	0.9595	0.39689
Q6KAR6	Exoc3	Exocyst complex component 3	32956.52	36091.76	36558.34	30717.64	34081	32637.82	33007.59	32652.08	35203.43	33375	1.0211	0.65633
P62305	Snrpe	Small nuclear ribonucleoprotein E	61220.46	55776.02	65737.98	55041.8	59444	74743.98	66576.48	68009.67	82031.88	72841	0.8161	0.02153
Q8C0M9	Asrgl1	Isoaspartyl peptidase/L-asparaginase	103453.5	120512.3	117125.8	85641.95	106683	104107.6	110897.6	96240.16	107952.4	104799	1.0180	0.83264
Q01405	Sec23a	Protein transport protein Sec23A	122966.6	128628.7	141944	132912.4	131613	136897.8	120619.4	155699.1	149309	140631	0.9359	0.34002
Q8CGA3	Slc43a2	Large neutral amino acids transporter small subunit	25758.69	21932.89	15442.92	22148.67	21321	19043.14	23900.46	15802.7	19058.77	19451	1.0961	0.51744
Q6P5F7	Ttyh3	Protein tweety homolog 3	16871.79	18852.07	5868.662	24662.84	16564	21137.78	21854.25	25969.06	24471.74	23358	0.7091	0.14759
P26516	Psmd7	26S proteasome non-ATPase regulatory subunit 7	84909.84	93231.16	97602.53	79399.34	88786	87431.87	87416.01	87898.91	99049.84	90449	0.9816	0.75045
Q9Z1X2	Ptdss2	Phosphatidylserine synthase 2	77207.96	56498.79	50973.99	38963.73	55911	51427.93	50884.87	46939.74	42001.87	47814	1.1694	0.36581
Q6PIU9		Uncharacterized protein FLJ45252 homolog	24981.8	22156.27	24568.76	21672.2	23345	23589.43	19745.63	23711.9	21386.79	22108	1.0559	0.36677
P06728	Apoa4	Apolipoprotein A-IV	239709.9	299167	332709.1	185924.8	264378	269019.6	226448.6	179837.4	168626.1	210983	1.2531	0.22834
Q8BKX1	Baiap2	Brain-specific angiogenesis inhibitor 1-associated pr	10311.37	11516.31	18072.54	18495.56	14599	19113.51	11288.87	19765.36	19483.49	17413	0.8384	0.37895
Q7TPW1	Nexn	Nexilin	18358.06	16247.17	17983.4	19264.48	17963	15692.58	21666.29	8203.659	11252.03	14204	1.2647	0.25560
Q62083	Pick1	PRKCA-binding protein	38230.89	36820.76	29322.6	17298.9	30418	30145.21	41373.4	38101.79	17880.71	31875	0.9543	0.84397

Q91YM4	Tbrg4	FAST kinase domain-containing protein 4	16658.05	15682.05	15542.27	15432.41	**15829**	14196.58	17311.08	14869.57	17848.71	**16056**	**0.9858**	**0.81659**
Q5SYD0	Myo1d	Unconventional myosin-Id	142319.5	117941.6	81595.73	138875.4	**120183**	115816.9	149714.3	77374.96	83029.98	**106484**	**1.1286**	**0.55235**
P62482	Kcnab2	Voltage-gated potassium channel subunit beta-2	145376.2	142509.4	116850.8	155906	**140161**	136096.4	148599.9	124847.8	122095.4	**132910**	**1.0546**	**0.50610**
P35290	Rab24	Ras-related protein Rab-24	13119.93	13562.54	13841.06	15461.32	**13996**	15356.5	15723.51	13037.29	16366.47	**15121**	**0.9256**	**0.25170**
Q9Z110	Aldh18a1	Delta-1-pyrroline-5-carboxylate synthase	26649.28	32535.29	31662.01	31146.13	**30498**	26165.29	33384.8	29370.54	33618.68	**30635**	**0.9955**	**0.95277**
Q9JIF7	Copb1	Coatomer subunit beta	72270.15	68452.02	77498.27	71684.8	**72476**	73137.6	75571.29	80105.79	84040.7	**78214**	**0.9266**	**0.10992**
Q8BUE4	Aifm2	Ferroptosis suppressor protein 1	10607	5748.905	11254.98	8203.656	**8954**	6776.508	5969.752	3426.343	4997.167	**5292**	**1.6918**	**0.04457**
Q9CXF4	Tbc1d15	TBC1 domain family member 15	18385.74	20677.04	21961.99	16214.58	**19310**	19847.76	17905.88	18203.6	18907.23	**18716**	**1.0317**	**0.67343**
Q00519	Xdh	Xanthine dehydrogenase/oxidase	15834.66	15210.37	7809.892	9595.26	**12113**	16804.66	17540.69	6877.71	7998.318	**12305**	**0.9843**	**0.95742**
Q61418	Clcn4	H(+)/Cl(-) exchange transporter 4	14907.45	18810.67	16923.21	16117.66	**16690**	19138.65	21571.11	18162.56	19999.78	**19718**	**0.8464**	**0.03235**
Q8BIF0	Cd99l2	CD99 antigen-like protein 2	5171.136	8957.968	6884.488	5629.689	**6661**	5320.139	2226.594	3873.379	1462.863	**3221**	**2.0681**	**0.02930**
Q91Z61	Diras1	GTP-binding protein Di-Ras1	68263.41	76739.54	68984.83	64095.27	**69521**	76128.26	70369.63	69125.84	69068.4	**71173**	**0.9768**	**0.61604**
E9Q401	Ryr2	Ryanodine receptor 2	5517.131	5669.459	7239.739	8407.131	**6708**	6030.892	6418.968	6076.889	5569.128	**6024**	**1.1136**	**0.37168**
O35658	C1qbp	Complement component 1 Q subcomponent-bindin	36060.16	35756.51	27790.04	32288.21	**32974**	31066.38	42603.68	28224.92	25635.68	**31883**	**1.0342**	**0.80414**
P42567	Eps15	Epidermal growth factor receptor substrate 15	71388.58	71712.6	65277.05	62581.9	**67740**	64110.83	73506.37	66812.45	64537.94	**67242**	**1.0074**	**0.87915**
Q9D711	Pir	Pirin	32665.55	29155.9	25003.91	29427.66	**29063**	33201.25	30762.21	29816.56	25017.04	**29699**	**0.9786**	**0.79369**
Q9D486	Cmip	C-Maf-inducing protein	19904.88	20752.97	20940.12	19460.43	**20265**	18436.93	20307.69	14900.05	16669.13	**17578**	**1.1528**	**0.06872**
Q8R180	Ero1a	ERO1-like protein alpha	28084.03	29259.12	29175.79	28373.02	**28723**	30025.57	31215.94	31897.2	33338.95	**31619**	**0.9084**	**0.00837**
Q69ZZ6	Tmcc1	Transmembrane and coiled-coil domains protein 1	1849.766			1613.475	**1732**							
Q9CR23	Tmem9	Proton-transporting V-type ATPase complex assembly regulator		11074.8			**11075**							
Q8R366	Igsf8	Immunoglobulin superfamily member 8	23906.47	22017.97	20244.91	23570.15	**22435**	26056.4	28345.59	20590.66	19670.88	**23666**	**0.9480**	**0.60607**
Q9CZR8	Tsfm	Elongation factor Ts, mitochondrial	14559.68	12683.07	12499.17	16781.58	**14131**	11998.32	14994.75	11690.69	13155.55	**12960**	**1.0904**	**0.38421**
Q0VGU4	Vgf	Neurosecretory protein VGF	11222.91	20455.25	11070.04	12679.99	**13857**	12709.89	8945.997	18023.71	16901.35	**14145**	**0.9796**	**0.92771**
Q80YX1	Tnc	Tenascin		74898.13			**74898**							
P53657	Pklr	Pyruvate kinase PKLR	73244.61	64155.43	47667.59	56622.76	**60423**	60388.77	71417.8	51522.8	35116.03	**54611**	**1.1064**	**0.55926**
Q8VC31	Ccdc9	Coiled-coil domain-containing protein 9	7850.249	10355.32	8486.544	6965.629	**8414**	8417.3	6516.535	10136.71	7619.092	**8172**	**1.0296**	**0.82488**
Q641K1	Agtpbp1	Cytosolic carboxypeptidase 1	7590.946	7082.49	7443.262	6100.975	**7054**	7640.211	8872.801	7825.692	5494.19	**7458**	**0.9459**	**0.62492**
P60867	Rps20	Small ribosomal subunit protein uS10	263985.5	250184.6	270267.2	303915.8	**272088**	274060.3	264397	284461.2	325422.5	**287085**	**0.9478**	**0.42719**
Q99KC8	Vwa5a	von Willebrand factor A domain-containing protein 5	323464.3	370378.9	336344.9	343582.3	**343443**	308729.4	359058.3	286211	268354.1	**305588**	**1.1239**	**0.13604**
Q91YQ3	Csdc2	Cold shock domain-containing protein C2	7193.771	7560.8	8064.365	6008	**7207**	6728.395	8409.328	5317.347	6480.033	**6734**	**1.0702**	**0.56324**
Q2MKA5	Chrna5	Neuronal acetylcholine receptor subunit alpha-5				92848.05	**92848**			113076.7		**113077**	**0.8211**	
O88951	Lin7b	Protein lin-7 homolog B	4011.45			3337.551	**3675**	1778.557	3955.891	3892.495	4190.639	**3454**	**1.0637**	**0.81238**
Q91W59	Rbms1	RNA-binding motif, single-stranded-interacting prote	84797.45	88549.03	91349.3	90526.53	**88806**	80061.39	88769.98	80535.26	93064.38	**85608**	**1.0374**	**0.39711**
Q80UK0	Sestd1	SEC14 domain and spectrin repeat-containing prote	16447.11	15486.38	13600.03	13081.91	**14654**	14204.7	12521.2	14198.9	14139.42	**13766**	**1.0645**	**0.35829**
P41234	Abca2	ATP-binding cassette sub-family A member 2	14051.85	15579.88	19374.65	15771.68	**16195**	14982.54	16054.21	16862.31	18500.97	**16600**	**0.9756**	**0.77397**
P29391	Ftl1	Ferritin light chain 1	307030.3	251459	158029.6	184703.8	**225306**	219323	314246.8	137032.3	118581.6	**197296**	**1.1420**	**0.63430**
Q91W90	Txndc5	Thioredoxin domain-containing protein 5	202433.5	197355.2	217325.1	224005	**210280**	205338.8	205212.6	233548.8	248443.4	**223136**	**0.9424**	**0.34081**
Q8VGR9	Or8u9	Olfactory receptor 8U9												
P97379	G3bp2	Ras GTPase-activating protein-binding protein 2	137889.1	136515.4	147594.3	165687.6	**146922**	154154.8	151593.2	165737.1	178210.6	**162424**	**0.9046**	**0.13847**
P70290	Mpp1	55 kDa erythrocyte membrane protein	115855	115727	84620.33	103578	**104945**	112084.6	115225.2	93246.37	86041.76	**101649**	**1.0324**	**0.75843**
Q80TL7	Mon2	Protein MON2 homolog	23695.52	23239.84	24290.79	18391.82	**22404**	23920.41	23072.99	21067.66	22197.84	**22565**	**0.9929**	**0.91764**
P21981	Tgm2	Protein-glutamine gamma-glutamyltransferase 2	202279	172536.4	173634.2	185470.3	**183480**	166808.9	153705.6	156199.6	168034.7	**161187**	**1.1383**	**0.02912**
Q8VEJ9	Vps4a	Vacuolar protein sorting-associated protein 4A	99404.58	99750.09	110799	79610.42	**97391**	84599.37	89786.93	92516.01	85636.33	**88135**	**1.1050**	**0.21915**
Q6Y685	Tacc1	Transforming acidic coiled-coil-containing protein 1	41117.72	34881.23	45150.93	40606.61	**40439**	36383.31	41792.91	34591.94	33276.1	**36511**	**1.1076**	**0.21351**
Q9JM14	Nt5c	5'(3')-deoxyribonucleotidase, cytosolic type	48937.37	52084.82	45390.9	43611.34	**47506**	45179.78	44112.05	40874.75	38974.98	**42285**	**1.1235**	**0.06970**
Q9JM52	Mink1	Misshapen-like kinase 1	52575.23	44555.56	47592.52	58166.95	**50723**	50324.16	48475.49	55564.36	56616.97	**52745**	**0.9617**	**0.59246**
Q8C6E0	Cfap36	Cilia- and flagella-associated protein 36	22409.82	24097.98	18635.28	21326.59	**21617**	22751.26	18088.99	21726.5	20274.94	**20710**	**1.0438**	**0.57447**
B1AVY7	Kif16b	Kinesin-like protein KIF16B	16559.82	20769.89	17772.15	20471.76	**18893**	19257.17	17544.73	15528.4	19804.32	**18034**	**1.0477**	**0.56443**
Q2UY11	Col28a1	Collagen alpha-1(XXVIII) chain	219099.7	195129.5	190917.6	266718.1	**217966**	234846.2	234837.7	186716.1	210136.7	**216634**	**1.0061**	**0.95121**
Q62095	Ddx3y	ATP-dependent RNA helicase DDX3Y		33156.63	32347.81	17573.83	**27693**	23191.81	30325.75	35757.62	30422.76	**29924**	**0.9254**	**0.68685**
P16675	Ctsa	Lysosomal protective protein	49108.64	45559.93	51854.87	51230.43	**49438**	50874.53	46729.95	49388.88	55575.65	**50642**	**0.9762**	**0.62473**
E9Q3S4	Map3k19	Mitogen-activated protein kinase kinase kinase 19	277520.3	297829.9	319440.7	428009.4	**330700**	260977.2	284693.6	451845.8	459754.1	**364318**	**0.9077**	**0.61156**
Q91VA6	Poldip2	Polymerase delta-interacting protein 2	27139.21	26187.93	28902.15	36402.28	**29658**	31408.13	30304.81	32848.18	35829.21	**32598**	**0.9098**	**0.30272**
P23927	Cryab	Alpha-crystallin B chain	794193.9	778997.2	788536	803982.7	**791427**	754789.5	736878.1	728740	792752.9	**753290**	**1.0506**	**0.04561**
Q9CRB2	Nhp2	H/ACA ribonucleoprotein complex subunit 2	34474.22	24912.71	36568.22	22891.87	**29712**	39220.78	34720.9	13853.78	21795.01	**27398**	**1.0845**	**0.74358**
O35226	Psmd4	26S proteasome non-ATPase regulatory subunit 4	144629.9	144963.2	151375.9	149738	**147677**	156290	146863	165539.3	155417.8	**156028**	**0.9465**	**0.09259**
Q8BHI7	Elovl5	Elongation of very long chain fatty acids protein 5	49224.74	46280.6	50956.41	55307.92	**50442**	53363.06	49414.06	47904.62	60313.62	**52749**	**0.9563**	**0.51726**
Q62189	Snrpa	U1 small nuclear ribonucleoprotein A	106552.9	101756.4	116835.3	116312.2	**110364**	109683.2	100860.8	113606.1	127598	**112937**	**0.9772**	**0.71397**
Q9DC07	Nebl	LIM zinc-binding domain-containing Nebulette	38136.58	43063.79	34898.28	36574.01	**38168**	45256.93	33495.23	21387.72	33024.35	**33291**	**1.1465**	**0.38292**

Q9ET30	Tm9sf3	Transmembrane 9 superfamily member 3	100271.1	94050.79	105501.9	105076.7	101225	106230	93989.61	116331.9	123357.4	109977	0.9204	0.25284
P18653	Rps6ka1	Ribosomal protein S6 kinase alpha-1	15745.95	19886.2	4922.516	9092.374	12412	13106.45	11776.48	10049.92	12723.06	11914	1.0418	0.88877
Q9ESL4	Map3k20	Mitogen-activated protein kinase kinase kinase 20	18204.37	18115.78	16809.01	16529.72	17415	22007.84	17242.02	15649.77	14584.34	17371	1.0025	0.98027
O08749	Dld	Dihydrolipoyl dehydrogenase, mitochondrial	254472.9	230878.7	258006.2	282041.6	256350	255256.7	261621.1	271264.1	278831.8	266743	0.9610	0.40818
Q64514	Tpp2	Tripeptidyl-peptidase 2	164707	159729	158280.1	135606.6	154581	164094.3	163011.2	160476.9	151378.9	159740	0.9677	0.49406
Q8C8R3	Ank2	Ankyrin-2	61804.96	65852.61	65380.36	73853.51	66723	60492.07	68194.65	62519.86	63750.07	63739	1.0468	0.36137
Q8CD19	Lancl3	LanC-like protein 3	24247.23	26628.82	21487.75	21794.5	23540	23907.21	23310.43	14901.33	15998.14	19529	1.2053	0.18181
Q9CR67	Tmem33	Transmembrane protein 33	124000.2	138501	164286.2	166415.6	148301	152535.1	144121	172724.2	193412.6	165698	0.8950	0.29232
Q9DCV4	Rmdn1	Regulator of microtubule dynamics protein 1	41216.7	41471.46	41316.68	43709.64	41929	40582.7	43409.13	47600.68	47396.39	44747	0.9370	0.16691
B9EJ86	Osbpl8	Oxysterol-binding protein-related protein 8	43087.68	41555.48	38655.41	45701.71	42250	41369.05	44120.74	42663.78	47775.85	43982	0.9606	0.42420
Q99PG2	Ogfr	Opioid growth factor receptor	628361	556978.1	519438.2	674485	594816	379058	513509.9	593228.9	458504.1	486075	1.2237	0.10520
Q99L27	Gmpr2	GMP reductase 2	27821.49	33581.12	36254.05	23509.46	30292	27804.29	26594.77	25500.53	23923.8	25956	1.1670	0.19605
Q8BGR2	Lrrc8d	Volume-regulated anion channel subunit LRRC8D	17591.55	17116.83	15129.03	13188.82	15757	13264.92	17367.11	21135.46	16178.42	16986	0.9276	0.54471
Q9DB27	Mcts1	Maligt T-cell-amplified sequence 1	59261.11	55095.87	54903.38	49148.62	54602	58957.92	58689.46	60937.46	56520.23	58776	0.9290	0.11497
Q8CCK0	Macroh2a2	Core histone macro-H2A.2	148400.9	149655.1	162239.5	192130.5	163107	162380.9	151061.9	161820.9	166938.4	160551	1.0159	0.81930
Q8CIE6	Copa	Coatomer subunit alpha	281152	282773	320305.8	291725.8	293989	284136.5	284333	329589.1	330613.6	307168	0.9571	0.44307
Q9CQD1	Rab5a	Ras-related protein Rab-5A	140384.6	137507.1	116082.9	127450.2	130356	129732.4	139977.7	146166.2	134187.9	137516	0.9479	0.31696
P97371	Psme1	Proteasome activator complex subunit 1	100197.8	79470.83	80349.18	64743.23	81190	75819.07	69248.04	68059.38	63526.86	69163	1.1739	0.16965
Q8VEK0	Tmem30a	Cell cycle control protein 50A	25546.86	26602.01	28613.53	30580.87	27836	30861.87	36745.47	30365.75	30708.74	32170	0.8653	0.06182
Q9CX56	Psmd8	26S proteasome non-ATPase regulatory subunit 8	81372.73	84116.63	87795.77	77466.11	82688	88907.23	87635.87	86736.66	91868.45	88787	0.9313	0.04735
P97382	Kcnab3	Voltage-gated potassium channel subunit beta-3								1953.21		1953	0.0000	
O09174	Amacr	Alpha-methylacyl-CoA racemase	13620.01	13822.27	15362.52	15585.83	14598	14834.12	13409.53	16289.06	16282.43	15204	0.9601	0.50598
Q8VD26	Tmem143	Transmembrane protein 143	11077.46	9621.617	2488.181	6318.437	7376	6690.106	10081.67	4703.719	7394.738	7218	1.0220	0.94502
Q80ZU0	Arl5a	ADP-ribosylation factor-like protein 5A	17842.92	16585.33	19792.57	17362.42	17896	19083.85	19719.14	19254.04	15859.78	18479	0.9684	0.62013
P63280	Ube2i	SUMO-conjugating enzyme UBC9	84555.92	78042.56	89607.17	65311.28	79379	70867.28	81226.04	72433.31	70441.73	73742	1.0764	0.37099
Q9D7J4	Cox20	Cytochrome c oxidase assembly protein COX20, mit	7602.653	10056.12	12400.46	6520.569	9145	11687.02	8871.265	10434.08	7230.938	9556	0.9570	0.80940
Q9JKL5	Tesc	Calcineurin B homologous protein 3	8253.191	9446.693	9558.52	10031.38	9322	11105.92	9114.247	11608.49	10620.37	10612	0.8785	0.09771
O35449	Prrt1	Proline-rich transmembrane protein 1												
P16254	Srp14	Signal recognition particle 14 kDa protein	57103.85	61209.25	70573.92	70350.7	64809	69532	59769.02	74220.48	77248.63	70193	0.9233	0.33129
P47934	Crat	Carnitine O-acetyltransferase	39642.23	40561.76	38506.14	43173.34	40471	36734.5	45755.81	36179.89	44234.74	40726	0.9937	0.92714
Q9Z2Y3	Homer1	Homer protein homolog 1		3949.281			3949		10684.73	3052.093	3521.63	5753	0.6865	
Q922J9	Far1	Fatty acyl-CoA reductase 1	26178.69	30803.61	35020.63	31835.98	30960	30708.34	32676.37	37558.89	42123.55	35767	0.8656	0.17758
Q8BGD9	Eif4b	Eukaryotic translation initiation factor 4B	120527.9	118145.3	129281.2	129263.3	124304	120564.3	114729.5	132501.4	141387.3	127296	0.9765	0.66858
Q9QUI0	Rhoa	Transforming protein RhoA	280938.7	285839.8	284079.7	303424.6	288571	311717.2	314145.7	261376.8	296883	296031	0.9748	0.59172
P68404	Prkcb	Protein kinase C beta type	24518.52	22781.41	10360.32	14983.45	18161	20383.87	17090.19	11266.89	12465.96	15302	1.1869	0.49511
P58404	Strn4	Striatin-4	23733.36	20091.89	21461.78	19583.89	21218	21148.49	24865.47	26632.37	25228.88	24469	0.8671	0.07237
Q8C0P5	Coro2a	Coronin-2A	75323.34	77106.33	71582.48	83717.37	76932	78212.38	76260.48	76104.56	79477.49	77514	0.9925	0.83450
P62878	Rbx1	E3 ubiquitin-protein ligase RBX1	28312.72	27128.96	27104.58	24616.14	26791	26329.89	22327.92	28350.13	27630.41	26160	1.0241	0.69855
O08914	Faah	Fatty-acid amide hydrolase 1	10808.55	11933.89	14550.72	16676	13492	12031.54	10275.7	9114.196	16126.83	11887	1.1350	0.45797
O35526	Stx1a	Syntaxin-1A	3585.618	7855.3	3498.226	5220.428	5040	4131.94	5117.631	4006.707	4696.643	4488	1.1229	0.61840
Q91WU5	As3mt	Arsenite methyltransferase	22383.4	24582.44	25270.47	18920.68	22789	23014.22	20885.42	27473.81	25041.21	24104	0.9455	0.53659
Q9WTP6	Ak2	Adenylate kinase 2, mitochondrial	73861.34	74263.93	79814.52	86956.96	78724	74918.06	79427.33	77737.7	84643.35	79182	0.9942	0.90518
P28230	Gjb1	Gap junction beta-1 protein	42800.09	41294.45	37305.31	52259.76	43415	38613.71	34060	31287.34	41996.77	36489	1.1898	0.13095
Q68EF6	Begain	Brain-enriched guanylate kinase-associated protein												
Q8CI94	Pygb	Glycogen phosphorylase, brain form	417604.6	402102.3	357457.8	348375.9	381385	372021.3	371457.3	332538.1	321783.3	349450	1.0914	0.18460
P28661	Septin4	Septin-4	15239.68	18206.4	13420.99	10661.92	14382	18297.9	17530.18	9834.08	11500	14291	1.0064	0.97353
Q80WS3	Fbll1	rRNA/tRNA 2'-O-methyltransferase fibrillarin-like pro	80073.09	64308.32	59153.48	30689.95	58556	30301.2	80468.07	67073.88	55018.7	58215	1.0059	0.98240
Q8R395	Commd5	COMM domain-containing protein 5	7191.881	9655.369	10655	7086.026	8647	7107.364	8850.297	11241.12	10314.91	9378	0.9220	0.58594
P10493	Nid1	Nidogen-1	1296197	1209328	1105275	1516038	1281710	1234435	1284103	1278115	1254352	1262751	1.0150	0.83667
P63254	Crip1	Cysteine-rich protein 1	36731.13	38265.18	37098.17	33024.54	36280	31857.6	33190.7	41268.97	36989.11	35827	1.0126	0.85642
O88441	Mtx2	Metaxin-2	26457.24	27319.76	25559.57	31789.22	27781	25863.96	28569.43	29016.39	25145.53	27149	1.0233	0.72050
P83870	Phf5a	PHD finger-like domain-containing protein 5A	50653.38	45879.83	51714.73	46481.46	48682	39527.84	43750.6	42553.28	50938.18	44192	1.1016	0.16338
Q05920	Pc	Pyruvate carboxylase, mitochondrial	169880.5	180308.9	169724.6	176012	173981	173917.3	197950.6	161990.4	173460.3	176830	0.9839	0.73355
Q99LD8	Ddah2	N(G),N(G)-dimethylarginine dimethylaminohydrolas	525760.9	438027.7	348793.1	439410.5	437998	402452.5	495939.7	329877.5	339741.1	392003	1.1173	0.41527
Q8K1R3	Pnpt1	Polyribonucleotide nucleotidyltransferase 1, mitoch	20870.48	22981.01	27014.81	24931.16	23949	23767.63	22411.23	23175.59	23536.13	23223	1.0313	0.60945
Q9QX47	Son	Protein SON	24465.86	22648.95	22578.49	25044.78	23685	22727.13	22914.18	27496.11	27811.95	25237	0.9385	0.35006
Q8BGZ1	Hpcal4	Hippocalcin-like protein 4	2695.785				2696							
Q8CI43	Myl6b	Myosin light chain 6B	17905.66	20068.87	9882.464	12497.92	15089	16620.2		13056.8		14839	1.0169	0.94929
Q99L88	Sntb1	Beta-1-syntrophin	232472.1	234477.5	195323.7	277561.8	234959	245311	258564.3	211391.6	216563.4	232958	1.0086	0.92460

P18654	Rps6ka3	Ribosomal protein S6 kinase alpha-3	27281.48	29872.73	27160.85	25955.28	**27568**	26832.1	26877.72	25348.51	24022	**25770**	**1.0698**	**0.14410**
Q8R570	Snap47	Synaptosomal-associated protein 47	80284.56	87735.51	80638.56	83243.25	**82975**	81967.37	83273.3	75739.5	81995.47	**80744**	**1.0276**	**0.39095**
Q5RJI5	Brsk1	Serine/threonine-protein kinase BRSK1	12781.06	12522.85	11414.61	11557.7	**12069**	13026.01	11656.67	11414.15	10590.26	**11672**	**1.0340**	**0.53933**
Q8C7R4	Uba6	Ubiquitin-like modifier-activating enzyme 6	38203.08	46404.75	38073.05	31530.36	**38553**	41729.28	40590.91	37075.4	33173.39	**38142**	**1.0108**	**0.91306**
Q9DC11	Plxdc2	Plexin domain-containing protein 2	76378.64	78305.64	57734.69	77283.52	**72426**	69426.7	72407.63	65039.03	59205.25	**66520**	**1.0888**	**0.33925**
Q91ZP3	Lpin1	Phosphatidate phosphatase LPIN1	26430.54	22169.52	25366.09	20497.91	**23616**	24020.24	24582.59	28911.52	20373.04	**24472**	**0.9650**	**0.71404**
P70698	Ctps1	CTP synthase 1	49032.46	52008.25	57698.28	46189.88	**51232**	42725.78	43362.5	50509.93	46790.68	**45847**	**1.1175**	**0.12734**
Q9JKR6	Hyou1	Hypoxia up-regulated protein 1	166562.7	174721.4	189482.8	186169.9	**179234**	174636.1	189749.4	196944.3	192060.5	**188348**	**0.9516**	**0.24899**
Q80WM4	Hapln4	Hyaluro and proteoglycan link protein 4									35512.95	**35513**	**0.0000**	
Q9R1K9	Cetn2	Centrin-2	5238.388	5459.417	6080.781	3085.984	**4966**	3815.756	3651.146	3094.895	5291.454	**3963**	**1.2530**	**0.25808**
Q62226	Shh	Sonic hedgehog protein	16518.05	13845.33		5632.184	**11999**	10705.13	16006.46	3369.949		**10027**	**1.1966**	**0.70882**
P14873	Map1b	Microtubule-associated protein 1B	1171420	1213099	1078682	1376472	**1209918**	1178203	1291141	1137948	1174921	**1195553**	**1.0120**	**0.84527**
P08414	Camk4	Calcium/calmodulin-dependent protein kinase type	11572.18	12145.5	8813.041	9116.359	**10412**	7328.783	11333.49		10910.15	**9857**	**1.0562**	**0.71977**
Q4VAE3	Tmem65	Transmembrane protein 65	89094.76	106804.7	106501.4	119333.3	**105434**	103882.7	113201.4	110789.7	109823.3	**109424**	**0.9635**	**0.56297**
Q61166	Mapre1	Microtubule-associated protein RP/EB family membe	95841.94	105827.4	103320.9	97645.33	**100659**	93332.3	105056.5	106765.4	100329.5	**101371**	**0.9930**	**0.85802**
Q61739	Itga6	Integrin alpha-6	583498.1	541008	529238.3	633759.4	**571876**	541867.6	570902.1	578478.4	606349.6	**574399**	**0.9956**	**0.92896**
Q91WQ3	Yars1	Tyrosine--tRNA ligase, cytoplasmic	122565.9	130314.3	121687.8	124044.4	**124653**	124232.8	121755.8	118770.6	118666.6	**120856**	**1.0314**	**0.15903**
Q8QZY1	Eif3l	Eukaryotic translation initiation factor 3 subunit L	71182.57	73964.46	78209.77	75723.95	**74770**	76367.12	76635.78	73515.41	75526.25	**75511**	**0.9902**	**0.66712**
Q6PIC6	Atp1a3	Sodium/potassium-transporting ATPase subunit alpl	468935.7	444444.4	398112	507093.4	**454646**	441874.4	479288.8	422325.9	456307.2	**449949**	**1.0104**	**0.86150**
Q99K48	Nono	Non-POU domain-containing octamer-binding protei	70364.79	69765.23	80420.16	79999.95	**75138**	68614.24	69380.6	81124.85	80339.19	**74865**	**1.0036**	**0.95348**
O88384	Vti1b	Vesicle transport through interaction with t-SNAREs	75578.86	83109.23	67348.84	73225.8	**74816**	71258.1	87063.76	78479.52	77592	**78598**	**0.9519**	**0.44259**
Q69ZK9	Nlgn2	Neuroligin-2	48505.01	87631.59	19661.16	58864.2	**53665**	54553.7	14696.94	138804.2	51585.33	**64910**	**0.8268**	**0.71858**
Q9ESD7	Dysf	Dysferlin	37371.57	33024.64	31094.01	40384.41	**35469**	33478.84	38199.63	37669.93	37254.51	**36651**	**0.9677**	**0.63411**
O35350	Capn1	Calpain-1 catalytic subunit	163737	162733	132598.5	136127.1	**148799**	150604.2	153106.2	139330.3	130094.5	**143284**	**1.0385**	**0.59821**
Q8BGT5	Gpt2	Alanine aminotransferase 2	26377.66	28048.81	28135.25	30547.81	**28277**	25672.66	28963.59	30637.76	35670.26	**30236**	**0.9352**	**0.41828**
Q5SUF2	Luc7l3	Luc7-like protein 3	11470.19	10069.22	10385.73	12244.79	**11042**	5765.372	7615.479	6430.908	12280.64	**8023**	**1.3763**	**0.09981**
O08788	Dctn1	Dynactin subunit 1	177881.9	176998.6	182376.2	185485.8	**180686**	181281.2	182378.9	181189.4	185035.3	**182471**	**0.9902**	**0.44393**
P62900	Rpl31	Large ribosomal subunit protein eL31	472477.5	442735.8	577921.3	601149.5	**523571**	576794	480754.4	683123.8	676911.5	**604396**	**0.8663**	**0.23785**
O35326	Srsf5	Serine/arginine-rich splicing factor 5	39039.92	40257.55	40466.07	46948.02	**41678**	42675.25	42245.69	51441.91	48874.76	**46309**	**0.9000**	**0.16126**
Q8CIF4	Btd	Biotinidase	54418.45	44645.59	49600.22	50283.08	**49737**	56758.17	47262.64	41833.79	49055.65	**48728**	**1.0207**	**0.79303**
Q8R1Q8	Dync1li1	Cytoplasmic dynein 1 light intermediate chain 1	163209.9	165922.6	166531.5	166499.3	**165541**	166450.9	165748.4	184211.6	179330.4	**173935**	**0.9517**	**0.12441**
P39061	Col18a1	Collagen alpha-1(XVIII) chain	172653.4	170718.1	158212.1	188682.9	**172567**	182105.1	179227.1	170703.8	175617.5	**176913**	**0.9754**	**0.54162**
P56387	Dynlt3	Dynein light chain Tctex-type 3	90144.31	80006.27	78332.94	83569.94	**83013**	86429.06	99571.45	71536.64	81965.41	**84876**	**0.9781**	**0.77986**
O70305	Atxn2	Ataxin-2	23488.74	21882.36	23056.57	27395.58	**23956**	28744.44	23527.87	31253.08	30921.33	**28612**	**0.8373**	**0.07324**
Q8BVF2	Pdcl3	Phosducin-like protein 3	20076.58	17999.48	23692.64	14707.29	**19119**	25909.31	19525.86	19861.63	19692.15	**21247**	**0.8998**	**0.41706**
Q9R190	Mta2	Metastasis-associated protein MTA2	26449.7	25204.43	29620.82	25169.1	**26611**	23117.09	21326.61	26731.86	29414.61	**25148**	**1.0582**	**0.51066**
Q9WVF8	Tusc2	Tumor suppressor candidate 2	5514.827	5650.91	5623.618	4846.385	**5409**	4964.108	5363.029	3514.738	4038.816	**4470**	**1.2100**	**0.08896**
Q4KUS2	Unc13a	Protein unc-13 homolog A							4417.91			**4418**	**0.0000**	
Q9JM13	Rabgef1	Rab5 GDP/GTP exchange factor	19110.26	19230.97	18152.4	14433.12	**17732**	18539.35	14420.8	15197.46	14091.45	**15562**	**1.1394**	**0.20301**
Q9JK42	Pdk2	[Pyruvate dehydrogenase (acetyl-transferring)] kinas	26970.32	27584.08	25633.29	29340.78	**27382**	26600.57	26774.73	22593.31	33527.54	**27374**	**1.0003**	**0.99742**
P51150	Rab7a	Ras-related protein Rab-7a	627788.9	597379	604308.3	642520.4	**617999**	632972.6	625510.2	651613.6	624819.2	**633729**	**0.9752**	**0.24370**
Q9CWQ0	Dph5	Diphthine methyl ester synthase	1574.436	1357.272	1137.41		**1356**	725.6973	929.3866	557.1901	737.8401	**738**	**1.8391**	**0.00664**
Q8BZZ3	Wwp1	NEDD4-like E3 ubiquitin-protein ligase WWP1	31948.36	27600.1	28014.69	31713.46	**29819**	36807.83	25394.18	32250.04	45703.93	**35039**	**0.8510**	**0.28187**
Q9JJE4	Paqr4	Progestin and adipoQ receptor family member 4	29346.73	29347.98	31858.52	36168.66	**31680**	29881.54	32473.16	33859.97	37339.05	**33388**	**0.9488**	**0.47403**
Q8CJ19	Mical3	[F-actin]-monooxygenase MICAL3	5686.496	4955.417	12762.93		**7802**		3788.942	6726.675	8496.267	**6337**	**1.2311**	**0.63367**
Q3TYD4	Arsg	Arylsulfatase G				13022.67	**13023**							
Q6P3D0	Nudt16	U8 snoRNA-decapping enzyme	29524.94	28820.09	25295.48	25305.97	**27237**	27373.42	30853.15	29922.99	23989.71	**28035**	**0.9715**	**0.68980**
P57759	Erp29	Endoplasmic reticulum resident protein 29	269198.9	284787.1	329474.3	319619	**300770**	288328.9	294028.3	328578.1	355881.2	**316704**	**0.9497**	**0.48201**
Q3U2P1	Sec24a	Protein transport protein Sec24A	13749.24	13566.73	19764.21	10807	**14472**	16044.83	15335.5	16853.41	18898.71	**16783**	**0.8623**	**0.30029**
P63017	Hspa8	Heat shock cognate 71 kDa protein	1638881	1751690	1828817	1644645	**1716008**	1802990	1687710	1634508	1728776	**1713496**	**1.0015**	**0.96679**
Q9Z2Q5	Mrpl40	Large ribosomal subunit protein mL40	18033.64	19802.69	21338.06	25319	**21123**	22986.42	24476.39	29158.56	22994.81	**24904**	**0.8482**	**0.12655**
Q8BI84	Mia3	Transport and Golgi organization protein 1 homolog	26358.3	27911.46	32239.16	33038.51	**29887**	32089.03	30412.31	32722.06	34225.3	**32362**	**0.9235**	**0.22037**
Q8CGF5	Tlcd4	TLC domain-containing protein 4	42242.23	50566.13	27683.94	54292.47	**43696**	45733.82	50345.19	56934.61	56276.34	**52322**	**0.8351**	**0.23078**
Q8CDN6	Txnl1	Thioredoxin-like protein 1	442640.9	411724.6	379473	392955.7	**406699**	422534.5	395700.2	426294.6	368758.3	**403322**	**1.0084**	**0.86578**
Q9DBC0	Selenoo	Protein adenylyltransferase SelO, mitochondrial	29359.61	27689.28	31025.29	28520.98	**29149**	28735.38	26705.31	24290.77	26622.36	**26588**	**1.0963**	**0.06841**
O08795	Prkcsh	Glucosidase 2 subunit beta	102788.4	102865.5	107407.4	115059.6	**107030**	102719.9	102413.6	123178	114413.4	**110681**	**0.9670**	**0.55133**
Q91VD9	Ndufs1	NADH-ubiquinone oxidoreductase 75 kDa subunit, n	288658.9	293448.4	316184.4	336485.4	**308694**	296710.9	323666.1	315748.5	324694.7	**315205**	**0.9793**	**0.62917**
Q9WVQ5	Apip	Methylthioribulose-1-phosphate dehydratase	27741.13	31949.48	34497.45	20465.45	**28663**	27715.76	25304	37468.97	23071.07	**28390**	**1.0096**	**0.95260**
Q9QXS6	Dbn1	Drebrin	110888.3	114874.7	100740.7	111224.6	**109432**	104301.1	114480.2	108725.2	104633.1	**108035**	**1.0129**	**0.72924**

Q8BUY5	Timmdc1	Complex I assembly factor TIMMDC1, mitochondrial	10286.82	9134.337	11224.38	10514.58	10290	12034	11088.97	14649.76	12602.37	12594	0.8171	0.03799
Q8CI59	Steap3	Metalloreductase STEAP3	59925.93	61950.15	70858.65	56293.51	62257	59521.92	70099.93	59772.37	69250.42	64661	0.9628	0.59153
Q8C398	Pigw	Phosphatidylinositol-glycan biosynthesis class W protein						25679.85				25680	0.0000	
Q9JII6	Akr1a1	Aldo-keto reductase family 1 member A1	294164.9	282248.1	244403.9	216570.9	259347	264429.8	247182.8	209315.7	207800.8	232182	1.1170	0.27617
Q6A0A2	Larp4b	La-related protein 4B	23500.54	24082.71	28413.27	30668.7	26666	26930.52	26538.87	27119.99	35139.11	28932	0.9217	0.43310
Q80X73	Pelo	Protein pelota homolog	10009.92	10282.56	11159.01	8868.182	10080	8557.845	9881.385	4002.644	8009.729	7613	1.3241	0.11767
Q9DBJ1	Pgam1	Phosphoglycerate mutase 1	689309.4	671861.6	582968.8	544114.8	622064	681587.2	650273.7	544211.3	468013.3	586021	1.0615	0.57149
Q6ZQB6	Ppip5k2	Inositol hexakisphosphate and diphosphoinositol-pe	20083.55	19063.53	17856.35	17179.91	18546	18296.14	18656.34	19797.98	18318.13	18767	0.9882	0.77331
Q8BG81	Poldip3	Polymerase delta-interacting protein 3	10911.43	11806.41	15457.76	12597.97	12693	10945.24	12595.61	16258.34	15555.67	13839	0.9172	0.49838
P15209	Ntrk2	BDNF/NT-3 growth factors receptor	14049.23	11063.71	12319.32	9938.231	11843	8914.993	9780.054	13298.13	8915.854	10227	1.1579	0.28182
Q8C4Y3	Nelfb	Negative elongation factor B	7681.968	10759.06	10203.01	5632.877	8569	8807.461	3072.583	11330.95	8424.396	7909	1.0835	0.76409
Q9CQF9	Pcyox1	Prenylcysteine oxidase 1	100957.6	96484.64	104042.2	117376.5	104715	103438.9	108926.9	111323.9	115073.2	109691	0.9546	0.36824
Q9CZS1	Aldh1b1	Aldehyde dehydrogenase X, mitochondrial	115397.1	119153.5	114319.6	124935.1	118451	91992.27	107050.7	129527.2	123124.8	112924	1.0489	0.55139
Q9D0L4	Adck1	AarF domain-containing protein kinase 1	16351.92	18157.46	22450.37	24041.67	20250	19496.21	19284.8	18718.62	21764.78	19816	1.0219	0.82846
Q9D517	Agpat3	1-acyl-sn-glycerol-3-phosphate acyltransferase gam	57410.57	56285.47	56376.89	62187.15	58065	63268.42	58722.23	63051.32	76491.34	65383	0.8881	0.12405
Q8BUN9	Slc24a2	Sodium/potassium/calcium exchanger 2		34266.77	21495.23	30284.12	28682		23961.95		23602.31	23782	1.2060	0.38869
P51410	Rpl9	Large ribosomal subunit protein uL6	388313.1	366602.7	407094.8	436479.8	399623	418201.9	409186.2	423290.2	475596.8	431569	0.9260	0.17997
O08664;Q921K9;Q9CXE	Bcl7c;Bcl7b;Bcl7a	B-cell CLL/lymphoma 7 protein family member C;B-c	30140.44	39457.98	34602.86	41881.93	36521	31668.6	29101.03	32753.82	37387.6	32728	1.1159	0.27146
P82349	Sgcb	Beta-sarcoglycan	81209.83	73006.67	67567.86	93324.86	78777	80164.32	70999.89	88231.39	80273.83	79917	0.9857	0.86886
Q920P5	Ak5	Adenylate kinase isoenzyme 5	85603.43	85599.81	90020.27	78755.05	84995	89558.12	84679.43	92542.77	92025.79	89702	0.9475	0.16036
P00416	mt-Co3	Cytochrome c oxidase subunit 3	6399.983	5445.646	5126.717	5560.579	5633	4802.231	6901.799	4542.774	5678.811	5481	1.0277	0.80789
P25785	Timp2	Metalloproteinase inhibitor 2	9022.004	9502.188	22200.13	21098.05	15456	23442.01	10477.62	11500.69	9529.363	13737	1.1251	0.73499
Q3UJD6	Usp19	Ubiquitin carboxyl-terminal hydrolase 19	25356.66	25666.11	29781.85	27893.72	27175	27701.34	26003.84	29148.56	30150.37	28251	0.9619	0.46322
O88958	Gnpda1	Glucosamine-6-phosphate isomerase 1	64933.47	61264.36	54262.37	45509.6	56492	56975.38	51865.69	45688.26	48391.87	50730	1.1136	0.28617
Q6P9K8	Caskin1	Caskin-1	31487.98	32568.43	31217.36	34053.38	32332	35107.97	38133.11	42532.54	43582.65	39839	0.8116	0.01106
Q8K157	Galm	Galactose mutarotase	41826.34	38862.34	33204.71	37656.85	37888	40959.97	33163.42	27903.46	32238.96	33566	1.1287	0.23255
P97855	G3bp1	Ras GTPase-activating protein-binding protein 1	50213.25	54023.7	52262.96	58697.41	53799	49345.21	52700.54	52492.36	54292.2	52208	1.0305	0.47398
Q9D061	Acbd6	Acyl-CoA-binding domain-containing protein 6	10514.67	8402.844	9351.397	1432.733	7425	6597.422	8017.524	1364.42	3330.291	4827	1.5382	0.34655
P34152	Ptk2	Focal adhesion kinase 1	33898.54	32993.58	34253.8	26552.5	31925	29087.35	30838.66	31769.22	29263.18	30240	1.0557	0.41424
A2AJI0	Map7d1	MAP7 domain-containing protein 1	64886.97	72493.39	66509.05	79116.84	70752	60243.17	62281.54	78287.14	80207.13	70255	1.0071	0.93817
A0A1W2P872	Nova2	RNA-binding protein Nova-2	1346.193	2320.777	3732.365		2466	4012.987	4118.195	1018.909	2304.229	2864	0.8613	0.72134
Q8K0D0	Cdk17	Cyclin-dependent kinase 17	12656.54	15080.67	13947.49	29188.28	17718	15745.48	14102.24	22423.21		17424	1.0169	0.95560
Q8CBC4	Cnst	Consortin	13444.24	13089.3	14164.22	14636.77	13834	16563.7	14830.83	18203.95	17182.56	16695	0.8286	0.01100
P70255	Nfic	Nuclear factor 1 C-type	23847.86	24668.24	31902.48	27639.94	27015	28570.92	22937.37	12442.91	29232.45	23296	1.1596	0.41929
P14106	C1qb	Complement C1q subcomponent subunit B	8791.67	7578.311	13661.29	7502.675	9383	6432.01	3993.492	9614.058	10058.06	7524	1.2471	0.39706
Q9DCC8	Tomm20	Mitochondrial import receptor subunit TOM20 homolog												
P29699	Ahsg	Alpha-2-HS-glycoprotein	127217.3	140751.6	164268.6	72097.71	126084	113409.1	82783.82	97032.53	90434	95915	1.3145	0.19364
Q91V57	Chn1	N-chimaerin	20752.8	36014.79	22298.01	29359.88	27106	28414.95	34848.5	15164.37	18550.89	24245	1.1180	0.63469
Q9ERG2	Strn3	Striatin-3	26683.01	27779.18	31181.78	29241.56	28721	28571.99	26045.78	29571.06	32771.83	29240	0.9823	0.77029
Q6P1B1	Xpnpep1	Xaa-Pro aminopeptidase 1	36369.51	41327.03	41262.17	25859.77	36205	35235.43	35245.37	37265.58	31957.14	34926	1.0366	0.74799
Q9DB15	Mrpl12	Large ribosomal subunit protein bL12m	54964.3	53657.65	64157.82	63046.09	58956	57656.54	58899.33	66062.06	63137.45	61439	0.9596	0.48376
Q9JKB3	Ybx3	Y-box-binding protein 3	27375.31	31421.9	33569.04	25571.84	29485	25519.97	27053.98	26949.01	32252.53	27944	1.0551	0.53683
P97772	Grm1	Metabotropic glutamate receptor 1												
Q8CI04	Cog3	Conserved oligomeric Golgi complex subunit 3	8575.435	10540.63	8521.909	7808.627	8862	7964.453	9291.675	6029.708	8236.566	7881	1.1245	0.31651
P24270	Cat	Catalase	92535.33	92190.48	108836.5	102359.9	98981	89138.47	97835.88	116041.1	118105.8	105280	0.9402	0.46765
P62918	Rpl8	Large ribosomal subunit protein uL2	704344.6	704095.6	821081.4	856653.2	771544	776664.6	696812.1	816540.7	931806.5	805456	0.9579	0.60921
Q8R2Z5	Vwa1	von Willebrand factor A domain-containing protein 1	272051.8	271290.5	232356.3	307839.8	270885	257513.3	274110.2	270072.3	270641.6	268084	1.0104	0.86548
O08709	Prdx6	Peroxiredoxin-6	459681.3	496584.9	404313.3	376695.8	434319	438578.2	397058.3	378685.7	379928.5	398563	1.0897	0.28401
Q60738	Slc30a1	Proton-coupled zinc antiporter SLC30A1	13921.62	16786.84	17250.17	20634.52	17148	13633.85	18443.24	17954.95	18613.48	17161	0.9992	0.99448
B2RSH2	Gnai1	Guanine nucleotide-binding protein G(i) subunit alph	182459.7	166408.4	145356.8	209551.6	175944	192637.9	184723.9	166361	170753.1	178619	0.9850	0.86294
Q6P4S6	Sik3	Serine/threonine-protein kinase SIK3	18610.03	5606.741	22295.49	12417.24	14732	8753.106	25848.73	5733.538	9345.503	12420	1.1862	0.70571
P68368	Tuba4a	Tubulin alpha-4A chain	1338233	1224451	939842.9	1226431	1182239	1146611	1162710	800609.3	910723.9	1005163	1.1762	0.20096
Q61036	Pak3	Serine/threonine-protein kinase PAK 3	7802.759	17185.2	6812.277	4933.969	9184	11068.39	10419.14	19071.44	18616.86	14794	0.6208	0.17017
A2ADY9	Ddi2	Protein DDI1 homolog 2	36001.8	35386.02	34988.7	26451.91	33207	32436.98	30431.43	28236.16	25803.24	29227	1.1362	0.18724
Q3TCN2	Plbd2	Putative phospholipase B-like 2	50839.78	49043.27	43296.96	46660.24	47460	45131.93	53678.72	43592.88	44846.92	46813	1.0138	0.82663
O35206	Col15a1	Collagen alpha-1(XV) chain	1417625	1478645	1438466	1591365	1481525	1476378	1325935	1498996	1520028	1455334	1.0180	0.67090
Q5FWK3	Arhgap1	Rho GTPase-activating protein 1	71577.29	66302.68	65196.46	59656.39	65683	62556.86	62594.33	63048.55	66800.64	63750	1.0303	0.49312
P51863	Atp6v0d1	V-type proton ATPase subunit d 1	99310.02	102844.9	107357.2	110502.5	105004	107911	106606.4	114681	113477.8	110669	0.9488	0.12460
Q9CQ40	Mrpl49	Large ribosomal subunit protein mL49	12677.81	11724.22	16218.04	4600.135	11305	12263.31	16111.34	13918.61	16246.4	14635	0.7725	0.25002

Q9Z172	Sumo3	Small ubiquitin-related modifier 3	393496.9	379460.4	370933.9	327764.1	**367914**	206260.4	349684	226284.3	294884.5	**269278**	**1.3663**	**0.03295**
Q61686	Cbx5	Chromobox protein homolog 5	18297.66	14385.79	19670.95	17994.32	**17587**	14178.33	17913.78	14785.22	16257.57	**15784**	**1.1143**	**0.24582**
Q8CHK3	Mboat7	Lysophospholipid acyltransferase 7	17378.71	15259.25	19327.96	20027.7	**17998**	17858.24	17608.24	15770.16	21119.93	**18089**	**0.9950**	**0.95506**
Q68EF4	Grm4	Metabotropic glutamate receptor 4												
Q91WG8	Gne	Bifunctional UDP-N-acetylglucosamine 2-epimerase	11298.01	13695.41	15638.61	11548.29	**13045**	13271.71		13651.95	14744.36	**13889**	**0.9392**	**0.53256**
Q9D0T1	Snu13	NHP2-like protein 1	73878.66	71967.63	83604.35	83232.3	**78171**	78089.43	85136.05	79487.91	88059.98	**82693**	**0.9453**	**0.28512**
P01837	Igkc	Immunoglobulin kappa constant	660874.6	313517.8	85213.7	123609.8	**295804**	276842.7	311957.5	28863.24	43811.13	**165369**	**1.7888**	**0.42192**
P27573	Mpz	Myelin protein P0	35309144	29403818	22721470	29268286	**29175680**	25832234	27617134	30481684	25461664	**27348179**	**1.0668**	**0.54024**
P11087	Col1a1	Collagen alpha-1(I) chain	2706371	2567789	2171851	3743901	**2797478**	2206241	2488754	3734626	3693683	**3030826**	**0.9230**	**0.66993**
Q8CHW4	Eif2b5	Translation initiation factor eIF-2B subunit epsilon	38237.42	44978.93	40341.78	37345.7	**40226**	42471.37	41504.54	47567.39	42124.71	**43417**	**0.9265**	**0.19785**
Q8CEC5	Nkiras1	NF-kappa-B inhibitor-interacting Ras-like protein 1	20457.96	12521.54	20600.82	19326.39	**18227**	24972.64	16775.43	23747.88	19659.01	**21289**	**0.8562**	**0.29886**
O35855	Bcat2	Branched-chain-amino-acid aminotransferase, mito	66202.31	69552.84	68677.67	73682.43	**69529**	70250.45	75090.98	69877.69	68386.99	**70902**	**0.9806**	**0.54292**
P47758	Srprb	Signal recognition particle receptor subunit beta	32916.38	33765.84	39281.59	38489.05	**36113**	38063.86	37815.45	38974.85	46293.93	**40287**	**0.8964**	**0.15770**
Q99N89	Mrpl43	Large ribosomal subunit protein mL43		37596.63	20792.52		**29195**	13460.33	39521.64	35547.02	33368.53	**30474**	**0.9580**	**0.90550**
Q8K297	Colgalt1	Procollagen galactosyltransferase 1	37908.01	42033.21	39306.38	39369.55	**39654**	38817.02	39388.78	40943.01	41196.55	**40086**	**0.9892**	**0.69223**
Q8R3R8	Gabarapl1	Gamma-aminobutyric acid receptor-associated prot	41874.7	48151.39	42432.48	49446.95	**45476**	39233.18	43565.82	41393.21	46308.65	**42625**	**1.0669**	**0.29054**
O08807	Prdx4	Peroxiredoxin-4	55304	58935.72	57741.69	63976.89	**58990**	52399.16	62032.4	61024.76	59853.6	**58827**	**1.0028**	**0.95650**
Q9Z2D3	Gsdme	Gasdermin-E	18261.38	18044.63	13922.48	14706.71	**16234**	13469.15	19783.87	10018.48	6219.534	**12373**	**1.3121**	**0.25804**
Q9ESJ4	Nckipsd	NCK-interacting protein with SH3 domain	47518	47094.79	40169.51	42544.78	**44332**	44496.02	43392.88	35073.11	37312.45	**40069**	**1.1064**	**0.19306**
P10605	Ctsb	Cathepsin B	264596.1	268077.7	252474.8	266821.9	**262993**	274485.5	280450.3	281614.1	261376.3	**274482**	**0.9581**	**0.09758**
Q62188	Dpysl3	Dihydropyrimidinase-related protein 3	945323.8	1074772	1056045	1102840	**1044745**	1013311	1054016	1000587	956347.6	**1006065**	**1.0384**	**0.37022**
O88569	Hnrnpa2b1	Heterogeneous nuclear ribonucleoproteins A2/B1	543210.3	574270.6	655565.4	702946.8	**618998**	602214.7	590928.8	780364.9	716327.2	**672459**	**0.9205**	**0.39717**
Q8VCM8	Ncln	BOS complex subunit NCLN	54751.93	50924.77	58922.52	59891.95	**56123**	54171.76	52886.27	65134.86	63412.01	**58901**	**0.9528**	**0.48665**
Q99L04	Dhrs1	Dehydrogenase/reductase SDR family member 1	277293.1	280041.1	293114.9	295772.8	**286555**	284034.1	291628	321406.7	335488.8	**308139**	**0.9300**	**0.14841**
Q9D1G1	Rab1b	Ras-related protein Rab-1B	51111.86	42160.55	51566.72	49473.71	**48578**	49076.71	49594.39	54151.01	54336.85	**51790**	**0.9380**	**0.26414**
A2AWA9	Rabgap1	Rab GTPase-activating protein 1	39004.13	39791.41	37892.2	35438.89	**38032**	36829.31	40617.17	28929.22	33836.5	**35053**	**1.0850**	**0.30294**
Q8BYI6	Lpcat2	Lysophosphatidylcholine acyltransferase 2								15344.84		**15345**	**0.0000**	
E9Q5F9	Setd2	Histone-lysine N-methyltransferase SETD2	4983.792	3445.827	4900.656	8456.252	**5447**	5627.128	6411.865		1878.067	**4639**	**1.1741**	**0.65819**
P17156	Hspa2	Heat shock-related 70 kDa protein 2	2193024	2102501	2074523	2186161	**2139052**	2304319	2172601	2190906	2124944	**2198192**	**0.9731**	**0.26648**
Q80U78	Pum1	Pumilio homolog 1	14750.2	14435.47	15454.61	16364.55	**15251**	14783.36	15916.63	14802.14	16180.47	**15421**	**0.9890**	**0.77376**
Q9CQI3	Gmfb	Glia maturation factor beta	93288.55	97550.06	88915.32	71291.46	**87761**	88800.55	89925.91	68139.53	71892.37	**79690**	**1.1013**	**0.35566**
Q9CTY5	Micu3	Calcium uptake protein 3, mitochondrial	23773.43	23495.02	22350.23	26759.48	**24095**	22994.13	26504.2	23512.33	27720.19	**25183**	**0.9568**	**0.49061**
Q9CRC0	Vkorc1	Vitamin K epoxide reductase complex subunit 1	45318.39	51445.9	57983.49	50865.36	**51403**	57633.11	55489.7	54986.81	53335.29	**55361**	**0.9285**	**0.19860**
Q8BIK4	Dock9	Dedicator of cytokinesis protein 9	17801.5	17861.45	17023.46	17578.18	**17566**	16492.15	18845.96	15420.79	16352.44	**16778**	**1.0470**	**0.33595**
Q8R404	Micos13	MICOS complex subunit MIC13	36169.1	37070.36	40194.68	47645.51	**40270**	40053.94	38459.45	48699.37	44955.04	**43042**	**0.9356**	**0.45859**
Q8BFQ4	Wdr82	WD repeat-containing protein 82	12156.97	12230.13	15320.13	14391.04	**13525**	15590.54	15744.16	16352.45	16207.18	**15974**	**0.8467**	**0.02355**
P58742	Aaas	Aladin	6130.525	5826.563	6999.966	6283.236	**6310**	6380.053	6241.16	4097.96	8724.62	**6361**	**0.9920**	**0.96018**
P62960	Ybx1	Y-box-binding protein 1	92608.18	94893.38	128767.5	126680.7	**110737**	93309.15	109944.4	143184	146391.7	**123207**	**0.8988**	**0.47174**
P63024	Vamp3	Vesicle-associated membrane protein 3	87796.76	86990.73	104941.5	112929.7	**98165**	101279.1	77830.32	116135.8	117523.3	**103192**	**0.9513**	**0.67037**
Q9QZA0	Ca5b	Carbonic anhydrase 5B, mitochondrial	21845.11	14790.43	10506.72	16926.51	**16017**		21908.64	15714.59	11504.35	**16376**	**0.9781**	**0.92779**
Q8R4V2	Dusp15	Dual specificity protein phosphatase 15	223162.2	222137.1	160405.5	236027.3	**210433**	201288.2	220803	153955	171176.5	**186806**	**1.1265**	**0.33670**
Q00558	F8a1	40-kDa huntingtin-associated protein	10793.88	10354.37	7973.68	7509.577	**9158**	7383.334	6980.883	6584.25	7367.602	**7079**	**1.2937**	**0.04996**
P97770	Thumpd3	tRNA (guanine(6)-N2)-methyltransferase THUMP3	10519.68	10589.4	10256.64	10529.13	**10474**	11552.1	12655.87	7094.229	11617.68	**10730**	**0.9761**	**0.84314**
O09159	Man2b1	Lysosomal alpha-mannosidase	19839.69	19150.52	24674	22172.63	**21459**	20579.43	17859.59	15991.5	20048.66	**18620**	**1.1525**	**0.13355**
Q8VCE6	Nt5m	5'(3')-deoxyribonucleotidase, mitochondrial	9289.15	7161.216	8121.347	7632.767	**8051**	5593.727	8789.526	4548.207	5988.838	**6230**	**1.2923**	**0.12276**
P01887	B2m	Beta-2-microglobulin	158282.1	91958.88	91094.85	98150.75	**109872**	82405.28	100202.8	75837.72	99431	**89469**	**1.2280**	**0.28370**
Q99JW4	Lims1	LIM and senescent cell antigen-like-containing doma	116903.5	104488	117039.7	110462	**112223**	106264.1	102298.5	109694.2	108889.6	**106787**	**1.0509**	**0.16424**
Q9CQP2	Trappc2	Trafficking protein particle complex subunit 2								5756.778		**5757**	**0.0000**	
Q3TBW2	Mrpl10	Large ribosomal subunit protein uL10m	11042.64	10211.92	13170.86	11062.43	**11372**	12455.33	12729.76	12882.75	14799.43	**13217**	**0.8604**	**0.06735**
Q8R2Y2	Mcam	Cell surface glycoprotein MUC18	118279.6	110022.4	127478.9	138931.1	**123678**	141036.5	125753	150389.3	139513.6	**139173**	**0.8887**	**0.10162**
Q8VIJ6	Sfpq	Splicing factor, proline- and glutamine-rich	239415.8	233846.4	268124.1	297652.4	**259760**	236582.9	247763.2	288157.5	285479.9	**264496**	**0.9821**	**0.81789**
Q8BGF9	Slc25a44	Solute carrier family 25 member 44		9014.501	6817.909	8489.033	**8107**	9592.068		7028.935	12502.65	**9708**	**0.8351**	**0.40328**
Q9D8X2	Ccdc124	Coiled-coil domain-containing protein 124	33913.34	31164.15	34194.87	39680.25	**34738**	34208.05	37053.81	33478.91	38192.1	**35733**	**0.9722**	**0.65372**
A2AR02	Ppig	Peptidyl-prolyl cis-trans isomerase G	24943.55	24717.32	27064.07	23424.88	**25037**	27113.76	25876.29	26628.67	28316.68	**26984**	**0.9279**	**0.07658**
Q8BWG8	Arrb1	Beta-arrestin-1	49405.56	55086.04	52303.98	52820.02	**52404**	50110.59	49234.28	49021.14	46958.11	**48831**	**1.0732**	**0.03771**
Q922Q8	Lrrc59	Leucine-rich repeat-containing protein 59	267376.3	260421.7	281067.6	304617.2	**278371**	294990.6	278782	382723.2	384024	**335130**	**0.8306**	**0.10450**
O54818	Tpd52l1	Tumor protein D53												
Q78YZ6	Scoc	Short coiled-coil protein	43076.87	44171.92	48123.83	34276.68	**42412**	49868.34	43415.67	50103.5	45843.31	**47308**	**0.8965**	**0.19330**
Q3U7U3	Fbxo7	F-box only protein 7	24986.81	24045.08	23100.73	16997.79	**22283**	22479.26	18439.36	21454.42	21368.19	**20935**	**1.0644**	**0.52599**

P25976	Ubtf	Nucleolar transcription factor 1	18522.93	16013.06	19069.63	19823.14	18357	17334.53	17143.13	19387.72	20245.86	18528	0.9908	0.88451
Q91X97	Ncald	Neurocalcin-delta	26781.69	15790.4	8298.056	10863.41	15433	18679.55	35645.49	5058.273	10275.32	17415	0.8862	0.80902
P21619	Lmnb2	Lamin-B2	119144	117143.5	88216.61	128631.1	113284	119029.7	129201.4	100387	98440.62	111765	1.0136	0.89891
P97429	Anxa4	Annexin A4	90577.82	92345.59	73275.62	84266.41	85116	90896.16	85042.12	69520.53	66616.69	78019	1.0910	0.36879
Q9JMD3	Stard10	START domain-containing protein 10	57118.57	58418.61	49198.43	41389.31	51531	59541.46	53199.81	40374.21	36722.03	47459	1.0858	0.56306
Q8R502	Lrrc8c	Volume-regulated anion channel subunit LRRC8C	13629.81	14036.63	11612.02	14488.81	13442	12812.63	15251.22	10497.08	14987.59	13387	1.0041	0.96722
Q8BZ98	Dnm3	Dynamin-3	416982.9	408524.9	367813.9	378241.4	392891	409007.5	395175.9	387362.3	355117.2	386666	1.0161	0.71767
Q5U430	Ubr3	E3 ubiquitin-protein ligase UBR3	17339.89	16061.55	17145.58	14333.54	16220	14992.19	17009.4	15391.04	16827.87	16055	1.0103	0.85334
Q9D023	Mpc2	Mitochondrial pyruvate carrier 2	70044.75	77204.85	78734.18	82397.09	77095	74497.05	83169.92	80236.96	86203.43	81027	0.9515	0.31618
P59708	Sf3b6	Splicing factor 3B subunit 6	17323.35	15291.82	17257.59	18710.23	17146	15491.92	17257.45	18258.8	16305.83	16829	1.0189	0.74273
Q9JK92	Hspb8	Heat shock protein beta-8	71024.05	84052.52	71052.56	111522.4	84413	92039.73	88509.27	86305.26	97290	91036	0.9272	0.52592
O70443	Gnaz	Guanine nucleotide-binding protein G(z) subunit alpl	55558.72	52600.11	45501.08	55614.54	52319	54005.5	56345.02	36219.22	43106.93	47419	1.1033	0.38956
Q80TB8	Vat1l	Synaptic vesicle membrane protein VAT-1 homolog-l	891305.9	958756.8	1055882	1002471	977104	947404.1	842845.1	946924.9	1020609	939446	1.0401	0.48391
Q9CZ28	Snf8	Vacuolar-sorting protein SNF8	26608.87	25969.65	26780.03	25278.66	26159	24005.28	25835.09	25265.31	23957.69	24766	1.0563	0.05282
Q80X19	Col14a1	Collagen alpha-1(XIV) chain	30447.31	28821.83	37735.62	32218.58	32306	26977.49	27369.66	37276.37	43500.22	33781	0.9563	0.75231
Q8BTM8	Flna	Filamin-A	313037.2	298771.5	374371.1	313183.8	324841	306416.9	296378.2	360963.5	350687	328611	0.9885	0.87637
O70370	Ctss	Cathepsin S	32663.47	17386.15	14138.09	13601.85	19447	19605.99	17981.51	17736	14380.12	17426	1.1160	0.67677
Q9CQE3	Mrps17	Small ribosomal subunit protein uS17m	18991.19	19128.1	16835.17	15863.21	17704	18447.88	16034.7	19548.15	18738.89	18192	0.9732	0.67458
Q60854	Serpinb6	Serpin B6	380600.7	377843.1	342496	318316.5	354814	379247	367313.5	331993.3	308717.6	346818	1.0231	0.72904
Q9Z1L5	Cacna2d3	Voltage-dependent calcium channel subunit alpha-2	5972.677	7364.905	4464.481	7459.71	6315		7036.493			7036	0.8975	
P56394	Cox17	Cytochrome c oxidase copper chaperone	22140.75	23509	20990.75	30105.12	24186	20253.52	22381.04	44127.16	36656.54	30855	0.7839	0.31496
P46935	Nedd4	E3 ubiquitin-protein ligase NEDD4	82006.95	86744.14	87007.5	79706.55	83866	77354.01	81499.57	84630.7	77397.88	80221	1.0454	0.19806
P62717	Rpl18a	Large ribosomal subunit protein eL20	392219.5	365911.3	447848.4	429702.6	408920	424244.9	377561.5	469329.7	510656.5	445448	0.9180	0.32531
Q9DB34	Chmp2a	Charged multivesicular body protein 2a	69293.64	67686.79	61453.21	57132.73	63892	59964.31	60825.52	52790.74	53842.2	56856	1.1238	0.09047
Q9QZM0	Ubqln2	Ubiquilin-2	92927.91	92306.34	84360.79	79918.78	87378	91250.34	85875.79	98734.2	93412.02	92318	0.9465	0.27697
Q66GT5	Ptpmt1	Phosphatidylglycerophosphatase and protein-tyrosi	16334.72	17602.92	16361.01	18083.44	17096	18105.35	19160.23	16565.36	20273.26	18526	0.9228	0.16503
P51881	Slc25a5	ADP/ATP translocase 2	1379115	1363485	1686005	1591392	1504999	1409838	1523551	1447809	1598091	1494822	1.0068	0.91358
O09126	Sema4d	Semaphorin-4D								2264.448		2264	0.0000	
O88307	Sorl1	Sortilin-related receptor	34178.79	29701.47	29657.57	38609.17	33037	32728.34	34111.02	37952.49	38092.47	35721	0.9249	0.33021
Q04899	Cdk18	Cyclin-dependent kinase 18		10394.35	2023.602		6209							
P61620	Sec61a1	Protein transport protein Sec61 subunit alpha isofor	32926.67	35894.31	40002.58	40500.99	37331	36239.23	37232.53	39153.58	45866.13	39623	0.9422	0.44649
Q9Z130	Hnrnpdl	Heterogeneous nuclear ribonucleoprotein D-like	49348.71	48361.12	52177.39	50317.91	50051	47679.87	49159.76	46187.76	58523.98	50388	0.9933	0.91126
Q8BTY8	Scfd2	Sec1 family domain-containing protein 2	27917.51	28504.36	29353.21	30461.58	29059	28139.5	31405.23	31430.46	29710.29	30171	0.9631	0.29173
Q8BFW7	Lpp	Lipoma-preferred partner homolog	32108.09	33988.04	35210	31185.43	33123	29092.58	30243.85	31130.93	35244.82	31428	1.0539	0.33512
Q8CIN4	Pak2	Serine/threonine-protein kinase PAK 2	44837.37	43958.86	47600.11	39307.57	43926	42171.12	40811.53	40499.11	47239.16	42680	1.0292	0.61159
P62313	Lsm6	U6 snRNA-associated Sm-like protein LSm6	20535.15	19559.29	26047.88	18340.23	21121	22052.76	16629.8	27492.74	23552.89	22432	0.9415	0.65836
Q9Z1Q5	Clic1	Chloride intracellular channel protein 1	167015.2	191058.8	185172.2	137755.7	170250	163485.4	163121.3	167826.7	144428.3	159715	1.0660	0.45070
P48962	Slc25a4	ADP/ATP translocase 1	1571534	1560299	1821714	1921100	1718661	1779908	1764627	1867501	1815143	1806795	0.9512	0.38158
Q62425	Ndufa4	Cytochrome c oxidase subunit NDUFA4	471819.9	514952.7	452055.7	490472.3	482325	454249.8	488276.1	425696.4	444501.4	453181	1.0643	0.17120
P55258	Rab8a	Ras-related protein Rab-8A	41329.86	44007.25	50637.04	49735.88	46428	54681.46	51644.63	54765.87	57548.73	54660	0.8494	0.01791
Q9R1P1	Psmb3	Proteasome subunit beta type-3	122993.1	113008.9	114112.7	111819.9	115484	120479.3	125228	117065.3	113653	119106	0.9696	0.34665
Q8VCX5	Micu1	Calcium uptake protein 1, mitochondrial	25092.51	22117.44	26121.58	26455.61	24947	25435.24	22971.28	27210.27	25215.09	25208	0.9896	0.84910
Q99MK8	Grk2	Beta-adrenergic receptor kinase 1	44928.26	43833.99	36784.25	34477.74	40006	47904.96	40786.38	43634.32	43481.11	43952	0.9102	0.23219
Q9CQ75	Ndufa2	NADH dehydrogenase [ubiquinone] 1 alpha subcom	162166.4	159454.3	174698.8	188958.8	171320	165855.5	190517.6	190066.5	246003.8	198111	0.8648	0.19281
O88738	Birc6	Baculoviral IAP repeat-containing protein 6	38674.34	40717.23	40619.82	37342.53	39338	40063.72	38115.03	38228.82	40421.49	39207	1.0033	0.90122
E9Q5C9	Nolc1	Nucleolar and coiled-body phosphoprotein 1	13353.49	14438.04	13133.44	20467.61	15348	13967.69	15434.24	15299.83	18702.79	15851	0.9683	0.80997
Q9DBG7	Srpra	Signal recognition particle receptor subunit alpha	30277.59	31384.6	35645.77	37081.14	33597	34167.98	33869.95	35931.57	41367.63	36334	0.9247	0.29564
Q9DCI3	Stard3nl	STARD3 N-terminal-like protein	54597.7	88657.56	49272.44	49293.48	60455	80003.42	80683.41	56673.52	65951.85	70828	0.8536	0.38706
Q8K3G9	Appl2	DCC-interacting protein 13-beta	10557.55	10961.72	9409.997	9850.721	10195	10538.88	11116.63	7399.147	10212.52	9817	1.0385	0.68815
P42932	Cct8	T-complex protein 1 subunit theta	525250.3	562220.4	581476.1	586795.6	563936	569863.8	591056.9	608851.5	600188.2	592490	0.9518	0.12949
P35283	Rab12	Ras-related protein Rab-12	22974.59	18156.82	20625.83	19981.46	20435	18980.81	17181.91	15907.87	19049.45	17780	1.1493	0.07817
Q9WUK2	Eif4h	Eukaryotic translation initiation factor 4H	70011.75	68443.94	72916.27	71204.86	70644	61234.27	68250.26	78795.7	70298.4	69645	1.0144	0.79805
Q64373	Bcl2l1	Bcl-2-like protein 1	39165.02	32993.06	42131.84	37482.95	37943	38143.98	37512.41	36162.85	35799.6	36905	1.0281	0.62013
Q5H8C4	Vps13a	Intermembrane lipid transfer protein VPS13A	29943.75	28425.32	27544.4	28313.58	28557	27506.12	27604.14	26240.9	26361.35	26928	1.0605	0.03918
Q78T54	Vma21	Vacuolar ATPase assembly integral membrane prote	101756.5	87625.26	41250.46	51442.43	70519	58279.18	115374.5	38991.92	72665.87	71328	0.9887	0.97145
P20918	Plg	Plasminogen	58297.43	55946.73	57937.67	53957.27	56535	50216.95	47939.03	47978.7	43250.64	47346	1.1941	0.00206
Q80TY0	Fnbp1	Formin-binding protein 1	60951.37	54388.98	49224.59	50470.75	53759	54260.85	52832.95	53366.8	52852.8	53328	1.0081	0.87668
Q8BH59	Slc25a12	Electrogenic aspartate/glutamate antiporter SLC25A	307965.7	307671.7	310529.9	361319.1	321872	311693.1	350149.4	314917.5	334351.8	327778	0.9820	0.72365
Q9DCT1	Akr1e2	1,5-anhydro-D-fructose reductase	22943.64	23333.82	22848.51	14801.74	20982	23363.07	18367.64	22945.17	20521.76	21299	0.9851	0.89768

P14602	Hspb1	Heat shock protein beta-1	441300.1	516445.8	499352.9	551854.1	502238	470672	462481.5	609276	523697.5	516532	0.9723	0.73860
Q8BGC0	Htatsf1	HIV Tat-specific factor 1 homolog	17610.4	17532.31	25752.77	23115.78	21003	15329.66	17632.65	23269.11	26225.36	20614	1.0189	0.90843
P28656	Nap1l1	Nucleosome assembly protein 1-like 1	197463.4	214130.2	174566.4	171690.3	189463	182683.9	195075.2	173644.3	179230.7	182659	1.0373	0.55966
P01863;P01865	Ighg;Igh-1a	Ig gamma-2A chain C region, A allele;Ig gamma-2A c	42088.14	23730.54	5131.841	11482.86	20608	41301.32	25878.65			33590	0.6135	0.37723
Q69Z99	Znf512	Zinc finger protein 512	18877.99	18183.57	14616.09	15765.84	16861	16451.62	17044.43	11222.09	18485.07	15801	1.0671	0.59236
Q61334	Bcap29	B-cell receptor-associated protein 29	10696.07	9665.585	7934.76	9347.603	9411	7075.186	10143.26	10793.12	9120.035	9283	1.0138	0.90152
Q9CQF0	Mrpl11	Large ribosomal subunit protein uL11m	33189.68	27274.04	38342.67	34557	33341	35379.02	26342.73	38891.64	39175.52	34947	0.9540	0.68527
P19246	Nefh	Neurofilament heavy polypeptide	5175982	4615601	3566892	5838133	4799152	4624851	4918131	3455948	3746885	4186454	1.1464	0.34177
Q9CZ04	Cops7a	COP9 signalosome complex subunit 7a	96115.1	100610.5	93432.23	88301.28	94615	102005	109215.7	98269.84	92227.74	100430	0.9421	0.23336
Q61233	Lcp1	Plastin-2	275893.3	255271.9	193089.8	198110.8	230591	231573.2	211947.3	182746.1	174768.3	200259	1.1515	0.26174
Q3UHD6	Snx27	Sorting nexin-27	48888.93	51391.43	44539.61	51186.39	49002	51603.45	48612.45	44266.74	49231.75	48429	1.0118	0.80389
Q9R257	Hebp1	Heme-binding protein 1		12256.99			12257		20460.54			20461	0.5991	
Q6P5H2	Nes	Nestin	57014.61	73008.8	77645.81	61203.8	67218	59577.96	63106.65	56249.78	64715.32	60912	1.1035	0.27141
Q8BFU3	Rnf214	RING finger protein 214	17429.39	15769.76	19820.68	16590.78	17403	15303.04	14518.3	18366.24	17083.11	16318	1.0665	0.41249
Q61598	Gdi2	Rab GDP dissociation inhibitor beta	555633.9	583462.9	555139.6	461801.2	539009	555631.1	528586.2	520834.3	498850.1	525975	1.0248	0.66935
O35459	Ech1	Delta(3,5)-Delta(2,4)-dienoyl-CoA isomerase, mitoc	58528.82	60728.86	62656.82	75482.54	64349	65940.66	65866.45	75170.45	70233.97	69303	0.9285	0.30314
Q71M36	Cspg5	Chondroitin sulfate proteoglycan 5												
Q9DCF9	Ssr3	Translocon-associated protein subunit gamma	20026.8	17837.33	24136.95	19382.96	20346	17610.38	21560.07	26701.71	22085.58	21989	0.9253	0.50106
P55088	Aqp4	Aquaporin-4	16002.39	35219.59	30176.79		27133	10066.82			8275.693	9171	2.9585	0.09511
Q3U1J4	Ddb1	DNA damage-binding protein 1	102995	103831.3	104006.3	98257.8	102273	108916.9	102712.1	111762.6	109267.8	108165	0.9455	0.04638
O70589	Cask	Peripheral plasma membrane protein CASK	26553.16	29136.36	33998.47	28824.08	29628	29337.41	27127.75	26809.86	31395.96	28668	1.0335	0.63067
Q9D1I5	Mcee	Methylmalonyl-CoA epimerase, mitochondrial	17048.51	20295.77	18567.42	20687.17	19150	18531.31	20622.17	19026.5	21898.82	20020	0.9565	0.47338
P02468	Lamc1	Laminin subunit gamma-1	786097.4	738081.1	637378.2	880909.2	760616	755802	783119.8	720615.1	731727.9	747816	1.0171	0.81563
Q8CE90	Map2k7	Dual specificity mitogen-activated protein kinase kin	22151.6	28158.49	17737.91	19974.63	22006	19541.51	22307.23	19365.25	21876.64	20773	1.0594	0.62123
P97315	Csrp1	Cysteine and glycine-rich protein 1	328394.5	292615.7	266033.8	283992.1	292759	296978.9	310722.4	277508.3	250085.3	283824	1.0315	0.64735
Q5SX39	Myh4	Myosin-4	368463.2	264782.3	286617.4	543441.4	365826	161193.8	172545.4	79890.72	173616.5	146812	2.4918	0.01721
Q60668	Hnrnpd	Heterogeneous nuclear ribonucleoprotein D0	261134.3	244429.6	254280.8	282861.3	260677	259825.4	266147.3	291253.9	301485.1	279678	0.9321	0.18994
Q9CR09	Ufc1	Ubiquitin-fold modifier-conjugating enzyme 1	75484.7	63063.95	81656.91	55922.64	69032	69047.98	57732.71	57161.71	62177.93	61530	1.1219	0.28882
Q9D967	Mdp1	Magnesium-dependent phosphatase 1	39591.03	38301.2	38456.88	29225.6	36394	34437.3	36489.63	39492.16	34927.04	36337	1.0016	0.98357
Q9CQ54	Ndufc2	NADH dehydrogenase [ubiquinone] 1 subunit C2	67199.72	71808.11	67166.74	76326.97	70625	70651.08	75281.3	75437.56	77309.88	74670	0.9458	0.17207
P70404	Idh3g	Isocitrate dehydrogenase [NAD] subunit gamma 1, m	105367.1	99854.13	101530.8	110409	104290	104468.1	109793.5	109073.7	113933.3	109317	0.9540	0.14945
Q8K1H1	Tdrd7	Tudor domain-containing protein 7	9428.679	10146.87	10390.49	11459.42	10356	9631.826	12136.63	8730.088	9586.579	10021	1.0334	0.70601
P58544	Btbd1	BTB/POZ domain-containing protein 1	90446.77	66754.82	111228.5	108133.9	94141	72547.02	63961.9	86444.05	70375.7	73332	1.2838	0.11401
Q9CQR4	Acot13	Acyl-coenzyme A thioesterase 13	96776.98	98062.38	98014.24	126789.7	104911	96900.6	93586.92	110199.6	120253.5	105235	0.9969	0.97401
Q9CZX9	Emc4	ER membrane protein complex subunit 4	40629.32	38518.66	46740.34	41948.43	41959	41889.02	45814.05	45373.41	53236.45	46578	0.9008	0.16912
Q5SQX6	Cyfip2	Cytoplasmic FMR1-interacting protein 2	37450.38	41201.41	36077.83	37174.32	37976	42436.13	41157.73	36671.33	36306.88	39143	0.9702	0.56449
Q6P2B1	Tnpo3	Transportin-3	38806.23	46810.53	40579.36	42300.48	42124	35863.63	41828.07	35587.32	34311.8	36898	1.1416	0.07237
Q6P4T2	Snrnp200	U5 small nuclear ribonucleoprotein 200 kDa helicase	49116.9	48652.36	52939.71	56941.52	51913	51716.41	51039.97	54080.95	59085.77	53981	0.9617	0.46573
Q9ERR7	Selenof	Selenoprotein F	37546.21	28054.95	30533.39	40113.25	34062	34697.7	37214.42	31505.07	43887.3	36826	0.9249	0.50238
P62264	Rps14	Small ribosomal subunit protein uS11	438821.5	413146.2	490413	507878.3	462565	468201.8	451006.7	500749	553034.9	493248	0.9378	0.36708
Q91WJ8	Fubp1	Far upstream element-binding protein 1	58933.21	64899.82	68886.56	61205.73	63481	66880.21	60879.8	72836.2	76694.53	69323	0.9157	0.20344
Q8BYM5	Nlgn3	Neuroligin-3	14821.52	22948.48	15699.35	15950.26	17355	12966	20143.84	12782.6	15507.62	15350	1.1306	0.46074
P62874	Gnb1	Guanine nucleotide-binding protein G(I)/G(S)/G(T) su	555595.4	478002.8	465716.4	612722.1	528009	565005.9	572346.5	510757.8	525804.4	543479	0.9715	0.69533
Q6DFW0	C9orf72	Guanine nucleotide exchange factor C9orf72 homolog												
P50114	S100b	Protein S100-B	488873.9	527816.2	375882.4	394828.9	446850	444869.7	486017.5	253975.9	308944.9	373452	1.1965	0.30866
Q61702	Itih1	Inter-alpha-trypsin inhibitor heavy chain H1	40221.82	29937.23	30773.69	38842.45	34944	31215.06	29895.59	24322.73	25505.32	27735	1.2599	0.06193
Q9R118	Htra1	Serine protease HTRA1	19785.85	17216.2	10636.89	13498.74	15284	13001.72	18631.42	8751.037	12195.64	13145	1.1628	0.48473
Q05793	Hspg2	Basement membrane-specific heparan sulfate prote	1268075	1202933	1255953	1514149	1310277	1237806	1206974	1378954	1411044	1308694	1.0012	0.98590
Q8R2Y0	Abhd6	Monoacylglycerol lipase ABHD6	23341.89	23239.06	29550.7	26291.2	25606	20051.5	21656.47	21588.23	23357.05	21663	1.1820	0.05289
P63115	Slc7a10	Asc-type amino acid transporter 1												
P56382	Atp5f1e	ATP synthase subunit epsilon, mitochondrial	164098.6	150807.2	180376.7	170829.1	166528	154311.9	161930.5	176404.4	158953.3	162900	1.0223	0.65952
Q01339	Apoh	Beta-2-glycoprotein 1	83782.71	63072.16	62117.39	81116.16	72522	62024.06	73035.27	63059.48	67066.41	66296	1.0939	0.35964
Q8K3E5	Ahi1	Jouberin	57682.38	24188.4		44838.97	42237	29234.35	42904.64			36069	1.1710	0.68071
Q3V0K9	Pls1	Plastin-1	16007.46	15855.77	14186.04	13429.44	14870	21399.81	15201.91	13966.4	17105.81	16918	0.8789	0.28512
Q8BYI9	Tnr	Tenascin-R	19940.74	21104.04	24385.08	14623.34	20013	18887.57	20319.33	224750		87986	0.2275	0.28854
Q922Q9	Chid1	Chitinase domain-containing protein 1	67057.79	66214.51	80382.2	79524.83	73295	72490.88	78626.2	63377.8	87963.31	75615	0.9693	0.73141
O08638	Myh11	Myosin-11	327922.4	301946.3	326368.8	319829.1	319017	308021	308679.6	341674.2	319876.4	319563	0.9983	0.95762
Q9EPQ7	Stard5	StAR-related lipid transfer protein 5	13069.52	12504.46	13768.79	10820.18	12541	10293.62	9011.361	9230.031	12379.17	10229	1.2261	0.05896
Q9D0E3	Lysmd1	LysM and putative peptidoglycan-binding domain-co	2423.742				2424							

O55131	Septin7	Septin-7	152760.1	156843.2	166102.9	153374.8	157270	154185.7	161374.1	155640.5	155467.1	156667	1.0039	0.86768
O08530	S1pr1	Sphingosine 1-phosphate receptor 1												
O70252	Hmox2	Heme oxygenase 2	83067.46	76155.6	75079.89	82309.2	79153	76696.23	86364.28	75317.27	76043.13	78605	1.0070	0.87427
Q8R2R3	Aagab	Alpha- and gamma-adaptin-binding protein p34	13876.95	14169.65	11803	9149.918	12250	13795.31	11598.65	10976.61	6926.398	10824	1.1317	0.46871
P26040	Ezr	Ezrin	46254.97	46912.11	54718.39	44998.04	48221	45176.15	42236.75	48699.89	49718.36	46458	1.0380	0.55051
Q61189	Clns1a	Methylosome subunit pICln	32428.27	27212.17	31618.37	22960.37	28555	23916.66	23289.38	22012.16	26140.29	23840	1.1978	0.09191
P63158	Hmgb1	High mobility group protein B1	296237.7	278280.4	346399.5	294182.5	303775	314675.8	274510.7	318200.5	309471.8	304215	0.9986	0.98117
P31650	Slc6a11	Sodium- and chloride-dependent GABA transporter 3	18624.29	23855.04	8642.604		17041	25184.61	10290.2	9654.577	11398.07	14132	1.2058	0.63475
Q9CQV6	Map1lc3b	Microtubule-associated proteins 1A/1B light chain 3B	225737.1	208771.7	157205.6	203942.1	198914	193394.1	221575.1	158646.3	110228.5	170961	1.1635	0.35852
Q9CWG8	Ndufaf7	Protein arginine methyltransferase NDUFAF7, mitocl	12295.13	10816.97	12701.34	13451.37	12316	12178.77	11589.81	11360.74	14065.58	12299	1.0014	0.98382
Q60676	Ppp5c	Serine/threonine-protein phosphatase 5	51076.48	54378.43	42460.7	40955.32	47218	48458.61	48684.77	47417.09	45626.44	47547	0.9931	0.92474
O35083	Agpat1	1-acyl-sn-glycerol-3-phosphate acyltransferase alph	59519.47	56737.91	63127.09	60014.49	59850	63905.84	56435.94	64138.16	66481.29	62740	0.9539	0.29906
Q9JHR9	Nrip2	Nuclear receptor-interacting protein 2	16689.09	12484.92	12398.85	17216.69	14697	16014.95	11740.07	10870.7	12702.04	12832	1.1454	0.32087
Q68FL4	Ahcyl2	Putative adenosylhomocysteinase 3	112240.2	119573.7	99889.24	117819	112381	108460.6	133260.9	104067.9	110828.1	114154	0.9845	0.82965
Q8CFI7	Polr2b	DNA-directed RNA polymerase II subunit RPB2	14674.22	14931.1	17431.26	18410.37	16362	17572.04	19622.32	15200.18	12629.8	16256	1.0065	0.95432
Q9JL15	Lgals8	Galectin-8	16588.46	17643.36	17498.4	18658.69	17597	16601.43	18277.88	19531.01	16476.7	17722	0.9930	0.88751
P48320	Gad2	Glutamate decarboxylase 2			78522.38		78522							
A6H584	Col6a5	Collagen alpha-5(VI) chain	62963.77	70233.49	46186.83	65588.85	61243	46456.37	35311.4	46195.79	47149.19	43778	1.3989	0.02618
Q9EQ06	Hsd17b11	Estradiol 17-beta-dehydrogenase 11	380812.6	363740.1	373742.5	421334.2	384907	350061.5	425855.5	384534.2	431455.3	397977	0.9672	0.58886
Q9DBR3	Armc8	Armadillo repeat-containing protein 8	30571.24	28216.41	28682.37	37169.78	31160	30886.73	32095.13	30792.44	33568.43	31836	0.9788	0.76567
Q99KI0	Aco2	Aconitate hydratase, mitochondrial	684879	653403.4	736976.4	755740.8	707750	688977.8	753881.6	721431	709659.1	718487	0.9851	0.70614
P70670;Q60817	Naca	Nascent polypeptide-associated complex subunit al	97505.81	111590.3	119355.7	109661.6	109528	119366.5	110515.7	125214.9	116256.2	117838	0.9295	0.17911
Q8BHC4	Dcakd	Dephospho-CoA kinase domain-containing protein	31119.28	35907.47	37787.75	32667.63	34371	31671.95	32935.8	35789.98	40603.69	35250	0.9750	0.73629
Q80X90	Flnb	Filamin-B	272318.4	263651.9	313943.9	278376.2	282073	270870.4	252051.3	311869.7	321559.5	289088	0.9757	0.73621
P35762	Cd81	CD81 antigen	345581.2	250208.2	228100.8	395284.6	304794	340034.8	315379.8	265048	297706.3	304542	1.0008	0.99547
Q8BIJ7	Rufy1	RUN and FYVE domain-containing protein 1	9521.453	12138.09	12265.96	10364.43	11072	10128.72	12607.97	9631.063	10392.11	10690	1.0358	0.69907
O55201	Supt5h	Transcription elongation factor SPT5	23325.29	25357.11	28645.62	26173.86	25875	23174.09	21520.91	23711.09	24766.9	23293	1.1109	0.09264
Q60675	Lama2	Laminin subunit alpha-2	632800.3	615814.1	532055.6	757163.6	634458	623347	655197.8	594111.5	601258.4	618479	1.0258	0.75269
Q9EPU4	Cpsf1	Cleavage and polyadenylation specificity factor subu	17397.18	16975.38	17550.6	19853.28	17944	16886.48	16915.54	15711.48	18815.73	17082	1.0504	0.38131
Q9CYI4	Luc7l	Putative RNA-binding protein Luc7-like 1	16330.76	16931.73	18196.54	18391.42	17463	15723.88	15313.01	17395.96	19377.16	16953	1.0301	0.64442
Q61214	Dyrk1a	Dual specificity tyrosine-phosphorylation-regulated I	6662.477	6887.046	10179	13663.31	9348	6545.325	11273.69	10246.37	9575.845	9410	0.9934	0.97536
Q9CPQ1	Cox6c	Cytochrome c oxidase subunit 6C	175691.3	181375.8	209708.4	211870.2	194661	182904.8	199540.7	206314	205912.7	198668	0.9798	0.72520
Q61029	Tmpo	Lamina-associated polypeptide 2, isoforms beta/del	181511.7	165914.8	189895.1	210336.8	186915	189602.6	173160.8	203477.2	203294.2	192384	0.9716	0.65709
Q9JI75	Nqo2	Ribosyldihydronicotinamide dehydrogenase [quinone	30863.36	34474.23	24665.48	27255.95	29315	30204.57	27415.96	25014.57	22008.33	26161	1.1206	0.29657
Q8BP40	Acp6	Lysophosphatidic acid phosphatase type 6	20147.47	25651.89	19833.08	25109.07	22685	21646.84	22442.83	20269.62	24427.38	22197	1.0220	0.79361
Q9JJV2	Pfn2	Profilin-2	306764.3	359332.3	347822.6	273142.9	321766	342411.8	322432.4	275927.2	258301.7	299768	1.0734	0.45950
E9PV24	Fga	Fibrinogen alpha chain	161690.2	126301	182271.8	227335.2	174400	103632.4	100924.5	155049.9	110342.2	117487	1.4844	0.06005
Q8K0T4	Katnal1	Katanin p60 ATPase-containing subunit A-like 1	22671.63	23643.59	18490.08	16267.34	20268	24566.84	23440.92	15060.68	20180.53	20812	0.9739	0.84976
Q99NB1	Acss1	Acetyl-coenzyme A synthetase 2-like, mitochondrial	14366.06	13557.7	15734.32	14669.93	14582	13363.48	13769.18	11949.98	17566.54	14162	1.0296	0.75441
Q9WUP4	Srd5a3	Polyprenol reductase	7469.851	5167.354	4026.943	6792.813	5864	6296.629	4455.652	6562.125	4271.779	5397	1.0867	0.65140
Q9QY81	Nup210	Nuclear pore membrane glycoprotein 210	10301.31	7453.735	9531.482	9051.174	9084	7969.337	6479.022	9347.299	6566.818	7591	1.1968	0.15036
Q9CWD8	Nubpl	Iron-sulfur protein NUBPL	14569.25	12629.67	12647.09	15011.94	13714	14179.27	12923.41	12218.41	18090.66	14353	0.9555	0.67573
Q9R1Z7	Pts	6-pyruvoyl tetrahydrobiopterin synthase	13366.62	20604.3	21133.87		18368	15672.08		25482.75		20577	0.8926	0.68160
Q9ES89	Extl2	Exostosin-like 2	21807.5	23063.48	22466.76	21465.42	22201	20302.92	28957.75	27400.04	27438.63	26025	0.8531	0.10078
Q9JLB2	Pals1	Protein PALS1	20530.85	21772.42	25071.46	24165.9	22885	25331.34	22810.08	24302.6	25059.6	24376	0.9388	0.25748
Q9JMG1	Edf1	Endothelial differentiation-related factor 1	36576.89	35914.28	39192.84	39765.92	37862	35313.69	32561.37	40796.52	40174.17	37211	1.0175	0.77654
Q9R0M6	Rab9a	Ras-related protein Rab-9A	24127.34	26797.04	23304.96	20649.56	23720	25788.17	27868.57	27595.51	26394.74	26912	0.8814	0.05709
P08226	Apoe	Apolipoprotein E	154083.5	160885.3	174386.1	160949.8	162576	138393.3	158725	185330.2	157404.5	159963	1.0163	0.81253
Q9Z1W9	Stk39	STE20/SPS1-related proline-alanine-rich protein kina	100375.8	100944.6	87363.03	90157.95	94710	93011.31	102576.1	83588.41	78351.71	89382	1.0596	0.43547
Q6PEE2	Ctif	CBP80/20-dependent translation initiation factor	43453.55	37248.34	37119.19	33567.93	37847	38547.11	44023.95	40710.41	43234.82	41629	0.9092	0.16659
Q91ZU6	Dst	Dystonin	100088.6	99812.27	97118.91	109662.6	101671	102552.7	107125.6	107968.9	106531.3	106045	0.9588	0.19484
Q9D1K2	Atp6v1f	V-type proton ATPase subunit F	54938.52	46810.76	39962.37	46921.86	47158	44288.93	41004.33	39500.63	52340.71	44284	1.0649	0.51862
Q9ESN6	Trim2	Tripartite motif-containing protein 2	81863.96	85598.75	86409.05	93809.14	86920	88825.51	90296.99	88894.16	93207.96	90306	0.9625	0.25685
P60904	Dnajc5	DnaJ homolog subfamily C member 5	25892.12	33665.46	23102.82	32277.28	28734	29151.35	30358.1	24694.8	27358.92	27891	1.0302	0.77423
Q61165	Slc9a1	Sodium/hydrogen exchanger 1	19894.98	15440.19	15034.96	17868.99	17060	17380.6	19287.47	12708.03	12917.33	15573	1.0954	0.48430
P52196	Tst	Thiosulfate sulfurtransferase	33617.38	35907.4	40046.88	42304.89	37969	33143.44	36922.38	37696.66	37434.22	36299	1.0460	0.48302
P51642	Cntf	Ciliary neurotrophic factor	267737.5	293196.1	252760.6	252066.8	266440	267168.4	282501.3	244784.2	253566.9	262005	1.0169	0.73823
O88487	Dync1i2	Cytoplasmic dynein 1 intermediate chain 2	137074.8	150475	138116	152979.8	144661	135441	144898.9	147198.5	134911.7	140613	1.0288	0.46573
P62274	Rps29	Small ribosomal subunit protein uS14	97927.3	81884.33	109168.9	106558.9	98885	101089.8	86167.51	118710.4	121283.5	106813	0.9258	0.46925

Q9WUN2	Tbk1	Serine/threonine-protein kinase TBK1	8586.496	9488.396	11168.25	7728.982	**9243**	10507.52	9124.348	7578.176	8737.555	**8987**	1.0285	0.79684
O54724	Cavin1	Caveolae-associated protein 1	365361.7	326836.3	337898.8	362188.3	**348071**	335812.8	329544	346934.6	342684.7	**338744**	1.0275	0.39217
Q8BH66	Atl1	Atlastin-1	162449.1	172884.3	163675.4	198452.7	**174365**	177530.1	194982.3	195204.7	206369.8	**193522**	0.9010	0.11127
Q9CZY3	Ube2v1	Ubiquitin-conjugating enzyme E2 variant 1	67847.23	64112.33	57871.89	49421.2	**59813**	62295.74	65449.71	55273.02	60618.85	**60909**	0.9820	0.81791
Q8BHL3	Tbc1d10b	TBC1 domain family member 10B	12498.6	10527.57	12089.16	14438.94	**12389**	12294.67	11485.84	17234.27	13596.12	**13653**	0.9074	0.43279
Q60872	Eif1a	Eukaryotic translation initiation factor 1A	35077.99	34353.86	41946.79	37429.07	**37202**	37761.05	37328.29	50600.32	45661.92	**42838**	0.8684	0.17314
Q9JKD3	Scamp5	Secretory carrier-associated membrane protein 5	41620.76	48978.84	56200.52	58185.96	**51247**	43545.59	46413.41	52248.93	54273.66	**49120**	1.0433	0.65475
Q9CZ42	Naxd	ATP-dependent (S)-NAD(P)H-hydrate dehydratase	42617.25	42405.77	46123.88	45221.41	**44092**	42258.72	43699.97	46012.23	45161.76	**44283**	0.9957	0.88311
Q6ZWQ7	Spcs3	Signal peptidase complex subunit 3	28207.85	26619.08	25244.43	29545.32	**27404**	26522.33	25789.03	23917.33	27865.71	**26024**	1.0531	0.31046
Q8C1E7	Tmem120a	Ion channel TACAN								17328.79		**17329**	0.0000	
Q9QYE9	Plekhb1	Pleckstrin homology domain-containing family B me	61582.31	58282.82	55551.48	54242.75	**57415**	51229	53846.4	47803.1	43054.52	**48983**	1.1721	0.02505
Q9JHW2	Nit2	Omega-amidase NIT2	23652.58	26691.76	28314.53	22375.53	**25259**	19721.01	24317.53	23945.4	24231.88	**23054**	1.0956	0.25695
P49722	Psma2	Proteasome subunit alpha type-2	145801.9	145184.2	166252.3	141892.7	**149783**	160945.3	151586.8	174053	157550.4	**161034**	0.9301	0.17474
P70345	Bcl2l2	Bcl-2-like protein 2	45107.41	56412.6	35745.74	32426.76	**42423**	31755.75	24339.65	32348.25	30476.11	**29730**	1.4269	0.06706
Q8BL86	Mblac2	Acyl-coenzyme A thioesterase MBLAC2	30478.45	25645.45	27369.42	32733.82	**29057**	25002.58	31076.65	23405.93	30262.25	**27437**	1.0590	0.53684
Q8R1B0	Stac2	SH3 and cysteine-rich domain-containing protein 2	20039.5	22168.12	20966.3	24803.99	**21994**	25742.46	24625.69	24531.74	21979.88	**24220**	0.9081	0.13872
Q5DTL9	Slc4a10	Sodium-driven chloride bicarbonate exchanger	5018.458	4412.877	4720.139	3464.623	**4404**	5512.87	4574.098	12743.29	8824.859	**7914**	0.5565	0.11122
Q8BVA5	Ldah	Lipid droplet-associated hydrolase	22311.1	28717.59	23018.36	17778.82	**22956**	23165.94	29754.59	13607.11	22680.63	**22302**	1.0293	0.87556
Q8BLK9	Rps6kc1	Ribosomal protein S6 kinase delta-1	2803.05				**2803**	2452.977	1745.51			**2099**	1.3353	
Q8BH58	Tiprl	TIP41-like protein	29219.59	34185.18	26790.98	27159.78	**29339**	28980.77	32821.82	27199.63	28208.92	**29303**	1.0012	0.98683
Q8K4R4	Pitpnc1	Cytoplasmic phosphatidylinositol transfer protein 1	48984.03	49013.29	42519.3	44169.99	**46172**	45455.17	43704.08	41405.55	39719.83	**42571**	1.0846	0.13572
Q3UTJ2	Sorbs2	Sorbin and SH3 domain-containing protein 2	235487.8	211042.3	229772.2	245672.5	**230494**	211932.6	238253.8	253178.6	238069.4	**235359**	0.9793	0.68029
Q0VGY8	Tanc1	Protein TANC1	16545.27	16391.42	18914.68	17542.12	**17348**	15890.14	16107.2	15930.58	17879.1	**16452**	1.0545	0.27843
O35143	Atp5if1	ATPase inhibitor, mitochondrial	94709.2	106496.6	116121.2	147662.6	**116247**	112864.2	100765.9	140333	135812.7	**122444**	0.9494	0.68879
Q14CH0	Fam171b	Protein FAM171B												
P29387	Gnb4	Guanine nucleotide-binding protein subunit beta-4	31801.57	33261.09	31020.74	37318.43	**33350**	31944.76	31426.38	25785.44	33515.82	**30668**	1.0875	0.26722
Q922D8	Mthfd1	C-1-tetrahydrofolate synthase, cytoplasmic	101976.3	106920.9	117603.6	100845.4	**106837**	105420.8	100860.5	117775	111281.1	**108834**	0.9816	0.71901
Q69ZR2	Hectd1	E3 ubiquitin-protein ligase HECTD1	15850.9	16603.65	17412.94	16035.69	**16476**	17588.92	13961.15	8651.771	15339.64	**13885**	1.1866	0.22813
Q99J47	Dhrs7b	Dehydrogenase/reductase SDR family member 7B	37518.45	39599.09	44456.71	42084.84	**40915**	41277.91	39610.85	46223.28	47130.48	**43561**	0.9393	0.30834
Q9CQV4	Retreg3	Reticulophagy regulator 3	20713.03	21087.6	23121.38	23184.21	**22027**	32786.89	22595.1	20576.55	23596.08	**24889**	0.8850	0.34368
P24638	Acp2	Lysosomal acid phosphatase	30338.49	20345.47	28395.42	28027.47	**26777**	24512.03	29946.34	28658.27	28268.3	**27846**	0.9616	0.68292
P63321	Rala	Ras-related protein Ral-A	206006.3	195530.2	177464.6	229167.9	**202042**	205775.2	200253.5	162732.6	174880.4	**185910**	1.0868	0.31996
Q04857	Col6a1	Collagen alpha-1(VI) chain	1082049	1084768	966469.2	1294673	**1106990**	1033439	1271257	862788.2	965017.3	**1033125**	1.0715	0.52860
Q9JMA1	Usp14	Ubiquitin carboxyl-terminal hydrolase 14	158830.1	155701.8	142087.1	129465.3	**146521**	150568.1	150131.1	130100.8	121023.6	**137956**	1.0621	0.42497
P57724	Pcbp4	Poly(rC)-binding protein 4	315721	341428.5	362647.5	279466.6	**324816**	376345.2	381925.1	427335.7	387562.3	**393292**	0.8259	0.01833
Q8R3T5	Stxbp6	Syntaxin-binding protein 6	480114.8	422855.7	294607.9	484466.3	**420511**	435887.7	486718.9	302029.6	320149.7	**386196**	1.0889	0.60521
Q8K1J6	Trnt1	CCA tRNA nucleotidyltransferase 1, mitochondrial	17037.51	17400.98	16068.93	16567.94	**16769**	17247.36	15500.97	14784.89	16213.37	**15937**	1.0522	0.21444
Q9CR58	Slc25a30	Kidney mitochondrial carrier protein 1	8722.407	10369.37	11281.29	11704.42	**10519**	12094.44	11287.03	7929.273	10698.37	**10502**	1.0016	0.98831
Q3UGC7	Eif3j1	Eukaryotic translation initiation factor 3 subunit J-A	51072.59	54611.71	49984.13	46140.32	**50452**	51645.31	52064.11	49665.93	51896.67	**51318**	0.9831	0.65300
P53996	Cnbp	CCHC-type zinc finger nucleic acid binding protein	58412.56	60867.3	65121.73	63621.53	**62006**	56296.17	58453.42	72654.41	60586.29	**61998**	1.0001	0.99841
P17427	Ap2a2	AP-2 complex subunit alpha-2	134093.5	148738.4	157314.9	153189.7	**148334**	145862.9	148447	147925.5	160366.2	**150650**	0.9846	0.71427
Q9CXY6	Ilf2	Interleukin enhancer-binding factor 2	91660.08	98839.71	102600.9	100048.8	**98287**	94197.22	100029.9	112906.6	109049	**104046**	0.9447	0.28024
Q9Z0W3	Nup160	Nuclear pore complex protein Nup160	12443.47	13397.53	13041.23	15081.52	**13491**	12628.59	15050.18	11612.95	15202.44	**13624**	0.9903	0.90423
Q80T41	Gabbr2	Gamma-aminobutyric acid type B receptor subunit 2			20286.15		**20286**							
Q9JIX8	Acin1	Apoptotic chromatin condensation inducer in the nu	43775.17	41662.27	49687.33	46356.92	**45370**	43152.99	45160.8	52874.63	52187.62	**48344**	0.9385	0.36045
Q6P9K9	Nrxn3	Neurexin-3	74525.11		89907.7	63378.45	**75937**	304044.6	25128.35	26009.41	299665.1	**163712**	0.4638	0.39604
Q8BGN3	Enpp6	Glycerophosphocholine cholinephosphodiesterase ENPP6			2611.552	309854.6	**156233**	295583.1	34730.7			**165157**	0.9460	0.96870
Q60866	Pter	Phosphotriesterase-related protein	23771.03	23914.6	27112.52	20372.92	**23793**	22233.76	17437.01	19675.92	21697.83	**20261**	1.1743	0.09101
Q8CAL5	Gpc5	Glypican-5	242629.6	232383.4	243390.2	290639	**252261**	251661.9	244011.6	326487.7	300815.1	**280744**	0.8985	0.27437
Q8R1N4	Nudcd3	NudC domain-containing protein 3	24057.85	23380.3	25680.41	22110.77	**23807**	26024.63	25667.31	22035.93	25270.97	**24750**	0.9619	0.45529
Q9DCD6	Gabarap	Gamma-aminobutyric acid receptor-associated prot	13790.78	12812.47	9822.655	11662.83	**12022**	11648.28	10951.47	16826.47	10175.76	**12400**	0.9695	0.83417
Q8BG02	Ppp2r2c	Serine/threonine-protein phosphatase 2A 55 kDa reg	6150.45	7269.853	4194.498	6651.1	**6066**	7726.546	6433.664	4152.886	6168.565	**6120**	0.9912	0.95848
Q9CQ80	Vps25	Vacuolar protein-sorting-associated protein 25	37866.7	37814.84	33803.94	35451.71	**36234**	33370.8	35208.43	33043.11	35360.3	**34246**	1.0581	0.13644
Q9D1N9	Mrpl21	Large ribosomal subunit protein bL21m	20652.43	21661.8	21704.25	20049.02	**21017**	22583.24	25152.53	23010.61	21967.63	**23179**	0.9067	0.03567
Q80TL0	Ppm1e	Protein phosphatase 1E	24665.41	24127.85	21091.08	18526.53	**22103**	27651.03	21745.77	18916.16	18897.58	**21803**	1.0138	0.90865
P35282	Rab21	Ras-related protein Rab-21	79197.87	62674.73	71298.26	74386.49	**71889**	73468.78	69642.91	63357.2	64444.11	**67728**	1.0614	0.35980
Q8K440	Abca8b	ABC-type organic anion transporter ABCA8B	25591.89	21709.58	22783.88	26596.62	**24170**	24781.3	22196.51	27550.21	28115.66	**25661**	0.9419	0.43584
Q91Z67	Srgap2	SLIT-ROBO Rho GTPase-activating protein 2	12234.57	10793.47	12268.66	9952.738	**11312**	11040.31	10316.29	8439.751	10457.35	**10063**	1.1241	0.16980
Q8CAB8	Castor2	Cytosolic arginine sensor for mTORC1 subunit 2	19229.6	24493.51			**21862**							

Q9JK23	Psmg1	Proteasome assembly chaperone 1	13562.45	12399.62	10282.55	9408.56	11413	14437.18	13142.03	13966.32	12643.77	13547	0.8425	0.08467
Q9R111	Gda	Guanine deaminase	45889.22	45755.06	39849.03	40652.76	43037	41719.21	40337.98	43449.81	36146.53	40413	1.0649	0.28705
Q02614	Sap30bp	SAP30-binding protein								4341.31		4341	0.0000	
Q91XU3	Pip4k2c	Phosphatidylinositol 5-phosphate 4-kinase type-2 ga	40858.97	40834.01	31130.47	38840.95	37916	32593.62	35591.64	32782.88	29159.81	32532	1.1655	0.08934
Q61206	Pafah1b2	Platelet-activating factor acetylhydrolase IB subunit	388664.4	476741.4	339361.1	281782.1	371637	367117.8	455182.5	323771.8	295881.9	360489	1.0309	0.84325
Q8BGR9	Ublcp1	Ubiquitin-like domain-containing CTD phosphatase 1	26226.83	22475.21	21380.26	15582.41	21416	22583.76	20799.58	21183.88	17228.14	20449	1.0473	0.71013
O88735	Map7	Ensconsin	22251.85	17956.28	19322.35	23619.78	20788	10405.9	24590.06	10197.01	18899.65	16023	1.2973	0.24936
A3KMP2	Ttc38	Tetratricopeptide repeat protein 38	18554.66	24422.95	19577.78	19510.81	20517	16631.08	19549.22	17683	14159.38	17006	1.2065	0.08963
Q8BUR4	Dock1	Dedicator of cytokinesis protein 1	9819.91	9015.298	9885.453	10024.27	9686	8395.9	8808.241	10004.59	9922.498	9283	1.0435	0.41635
Q8K021	Scamp1	Secretory carrier-associated membrane protein 1	48743.79	46811.06	49886.26	53523.35	49741	49562.11	52984.6	61427.28	52300.48	54069	0.9200	0.18950
Q91YX5	Lpgat1	Acyl-CoA:lysophosphatidylglycerol acyltransferase 1	18845.35	14612.32	17356.65	15084.53	16475	15481.7	10859.89	12954.24	21519.37	15204	1.0836	0.63088
Q8JZZ7	Adgrl2	Adhesion G protein-coupled receptor L2						16340.97		16783.27		16562	0.0000	
Q9R1C6	Dgke	Diacylglycerol kinase epsilon	34811.64	32368.28	35190.43	44991.33	36840	36456.3	34585.99	34265.59	39671.63	36245	1.0164	0.85170
Q8BL66	Eea1	Early endosome antigen 1	56093.71	55154.28	59302.52	53626.52	56044	58783.88	59116.32	61209.68	58740.67	59463	0.9425	0.04296
Q3UUF8	Ankrd34b	Ankyrin repeat domain-containing protein 34B												
Q9Z277	Baz1b	Tyrosine-protein kinase BAZ1B	24124.86	21235.15	23117.97	22649.5	22782	24310.73	23615.76	10330.66	20790.52	19762	1.1528	0.39404
Q62159	Rhoc	Rho-related GTP-binding protein RhoC	137215.9	134953.1	128155	127222.8	131887	130368.8	147812.6	125713.7	120566.6	131115	1.0059	0.90818
Q91ZS8	Adarb1	Double-stranded RNA-specific editase 1			12415.85	10706.11	11561	10951.6	7926.56	10297.61	10038.98	9804	1.1792	0.18939
O88712	Ctbp1	C-terminal-binding protein 1	53759.45	51621.75	52724.44	50865.88	52243	50538.73	52037.68	47804.66	51709.8	50523	1.0340	0.18577
Q8VI75	Ipo4	Importin-4	34178.3	37465.16	36386.86	32290.16	35080	37079.74	35880.17	36602.56	34957.26	36130	0.9709	0.43091
Q78JW9	Ubfd1	Ubiquitin domain-containing protein UBFD1	21541.12	18007.92	25130.69	17953.9	20658	14642.98	24163.46	11753.77	19037.89	17400	1.1873	0.34805
Q8BJY1	Psmd5	26S proteasome non-ATPase regulatory subunit 5	108650.3	122335.9	113432.4	99154.62	110893	111575.1	117250.8	98926.04	99229.66	106745	1.0389	0.55606
O35683	Ndufa1	NADH dehydrogenase [ubiquinone] 1 alpha subcom	61971.89	82594.54	76998.09	54167.47	68933	66435.88	76592.77	50404.71	64701.08	64534	1.0682	0.62325
Q8BVI5	Stx16	Syntaxin-16	10471.18	7563.909	9984.295	9613.349	9408	7097.516	9850.683	559.2338	13069.06	7644	1.2308	0.54265
Q19LI2	A1bg	Alpha-1B-glycoprotein	18440.2	116258.1	8764.591		47821	120256.1	95251.7			107754	0.4438	0.27756
O88910	Mpp3	MAGUK p55 subfamily member 3												
B2RPU2	Plekhd1	Pleckstrin homology domain-containing family D me	36277.14	38777.48	36921.12	42146.58	38531	39245.34	40282.13	35658.46	36608.41	37949	1.0153	0.74479
P20029	Hspa5	Endoplasmic reticulum chaperone BiP	737609.5	785877.1	948749.6	917384.4	847405	821031.2	763366.6	897324.8	939942.4	855416	0.9906	0.90487
P53810	Pitpna	Phosphatidylinositol transfer protein alpha isoform	89436.73	99877.81	90264.2	84104.65	90921	96997.22	82596.48	92503.05	80282.26	88095	1.0321	0.60354
Q9CZW4	Acsl3	Fatty acid CoA ligase Acsl3	38460.93	41177.18	49682.52	45681.53	43751	41428.55	44939.83	44565.07	49118.75	45013	0.9720	0.68219
O55125	Nipsnap1	Protein NipSnap homolog 1	237788.1	196326.6	204941	251201.8	222564	210605.5	205559	247491.3	227443.1	222775	0.9991	0.99003
Q9D8N2	Dennd10	DENN domain-containing protein 10	17160.41	17978.54	14541.09	8480.605	14540	16353.49	13200.04	14477.84	16381.26	15103	0.9627	0.81354
Q3UDW8	Hgsnat	Heparan-alpha-glucosaminide N-acetyltransferase	38875.13	44640.27	42770.52	49977.49	44066	39786.07	45651.53	41070.02	46481.09	43247	1.0189	0.78292
Q8K2L8	Trappc12	Trafficking protein particle complex subunit 12	10628.82	12262.46	12072.12	11459.96	11606	13330.84	15317.22	11487.29	16459.96	14149	0.8203	0.07044
Q9D892	Itpa	Inosine triphosphate pyrophosphatase	33162.47	34130.19	25713.38	18841.14	27962	27154.18	33018.42	22539.31	25299.88	27003	1.0355	0.82727
O35598	Adam10	Disintegrin and metalloproteinase domain-containin	111524.8	105286.6	110155.1	131314.2	114606	115787.3	114347	117809.7	125293.3	118309	0.9687	0.57368
Q8K3H0	Appl1	DCC-interacting protein 13-alpha	52271.68	52956.5	54181.8	49219.62	52157	50665.85	50703.48	52446.56	49587.19	50851	1.0257	0.32182
Q6PE15	Abhd10	Palmitoyl-protein thioesterase ABHD10, mitochondr	56435.29	55119.48	49205.49	60540.71	55325	55208.64	56294.65	52945.23	57827.49	55569	0.9956	0.92721
Q9D880	Timm50	Mitochondrial import inner membrane translocase s	49060.53	54364.04	43586.13	43846.1	47714	45464.77	58027.13	52817.69	45799.18	50527	0.9443	0.50342
Q64442	Sord	Sorbitol dehydrogenase	82253.36	88396.2	74658.2	71724.94	79258	84367.42	71456.35	78227.04	65620.89	74918	1.0579	0.46359
P51125	Cast	Calpastatin	77747.15	88506.84	82596.42	83278.28	83032	86307.95	82204.96	85866.84	78158.58	83135	0.9988	0.97303
Q8BMF4	Dlat	Dihydrolipoyllysine-residue acetyltransferase compo	445479.5	457893.9	444108.9	516411.9	465974	482541.7	502868.6	501809.5	501063.4	497071	0.9374	0.13074
Q6ZWX6	Eif2s1	Eukaryotic translation initiation factor 2 subunit 1	229304.4	232574.8	249779.7	224352.9	234003	228014.4	232103.4	230727.4	253219.4	236016	0.9915	0.80988
Q9DBH5	Lman2	Vesicular integral-membrane protein VIP36	110180.1	109420.8	109596.7	110420.2	109904	113893.8	124244.3	117353.8	119870.4	118841	0.9248	0.00651
Q61941	Nnt	NAD(P) transhydrogenase, mitochondrial	96249.45	48953.38	53435.55	101089.1	74932	51116.28	97805.93	46032.31	50459.25	61353	1.2213	0.48838
P50637	Tspo	Translocator protein	42419.35	38019.71	31075.95	35188.38	36676	36815.5	35343.77	24473.64	29545.13	31545	1.1627	0.21518
Q8C6G1	Cfap410	Cilia- and flagella-associated protein 410	13387.78	11902.12	18806.37	9907.312	13501	12775.7	12158.66	14125.63	9910.996	12243	1.1028	0.57095
P35821	Ptpn1	Tyrosine-protein phosphatase non-receptor type 1	17135.59	22221.08	22160.27	20531.51	20512	14723.01	25390.53	18506.4	25927.83	21137	0.9704	0.84055
P19137	Lama1	Laminin subunit alpha-1		2985.859		5915.339	4451	3360.51				3361	1.3244	
Q9ERD7	Tubb3	Tubulin beta-3 chain	1046489	1173939	1017176	1130117	1091930	1175755	1150827	1031504	993762.2	1087962	1.0036	0.94715
P20060	Hexb	Beta-hexosaminidase subunit beta	87713.85	82581.21	76714.26	84044.49	82763	89234.59	88573.26	88759.1	89444.41	89003	0.9299	0.03476
Q91ZH7	Abhd3	Phospholipase ABHD3	13672.18	12233.88	10843.72	11747.01	12124	16952.7	11635.48	10521.3	14814.44	13481	0.8994	0.42514
Q8BHN3	Ganab	Neutral alpha-glucosidase AB	151821.1	157282.7	168480.3	175211.1	163199	154833.5	171526.1	168005.3	176152.2	167629	0.9736	0.55024
P49817	Cav1	Caveolin-1	177476.1	151366.3	148539.6	168772.6	161539	148483.5	159542.1	159648.7	154600.4	155569	1.0384	0.45231
O08585	Clta	Clathrin light chain A	67089.57	91053.66	85762.86	86978.41	82721	85446.09	90503.63	66042.09	81658.35	80913	1.0224	0.81752
Q3UVR3	Ttbk2	Tau-tubulin kinase 2	13887.29	12388.09	12609.26	15970.07	13714	12155.91	13027.04	12514.73	14664.98	13091	1.0476	0.55275
Q62419	Sh3gl1	Endophilin-A2	14994.66	15640.45	17069.65	12825.87	15133	13779.58	13875.2	14997.06	15427.29	14520	1.0422	0.55206
Q8VE91	Retreg1	Reticulophagy regulator 1	20298.73	22556.59	14948.51	14376.41	18045	17038.77	24913.34	18921.45	23152.3	21006	0.8590	0.31719
O88322	Nid2	Nidogen-2	685832.4	648108.4	582772.4	779525.4	674060	624329.4	645301.5	644847.8	652607.7	641772	1.0503	0.46658

Q6NVF0	Ocrl	Inositol polyphosphate 5-phosphatase OCRL	11271.76	9286.812	10938.35	8890.854	**10097**	10002.86	10718.34	8285.856	9034.252	**9510**	**1.0617**	**0.48960**
Q9CY58	Serbp1	SERPINE1 mRNA-binding protein 1	81346.16	84209.88	88299.33	94553.84	**87102**	73245.48	85580.89	89351.29	83610.84	**82947**	**1.0501**	**0.38957**
Q9CZN8	Qrsl1	Glutamyl-tRNA(Gln) amidotransferase subunit A, mi	14088.87	15565.95	15321.61	13868.65	**14711**	16843.02	15677.73	18869.67	20357.09	**17937**	**0.8202**	**0.02867**
P62761	Vsnl1	Visinin-like protein 1	241182.5	231003.1	174691.9	221732	**217152**	192591.6	246486	160346.4	157771	**189299**	**1.1471**	**0.31386**
Q9QUH0	Glrx	Glutaredoxin-1	39603.7	35036.05	20874.28	22484.86	**29500**	28017.02	25469.09	21399.46	19805.9	**23673**	**1.2461**	**0.28705**
Q5F285	Tmem256	Transmembrane protein 256	24680.42	20140.31	12903.49	16045.36	**18442**	14765.38	15829.7	15049.93	12139.73	**14446**	**1.2766**	**0.18593**
Q8CGA0	Ppm1f	Protein phosphatase 1F	47499.81	42162.35	30440.67	33279.75	**38346**	37162.48	41420.23	26129.93	27716.31	**33107**	**1.1582**	**0.36941**
Q02105	C1qc	Complement C1q subcomponent subunit C				241847.4	**241847**				8112.742	**8113**	**29.8108**	
Q99KK7	Dpp3	Dipeptidyl peptidase 3	56263.87	57464.41	52649.32	43717.68	**52524**	53104.7	49583.16	45732.14	46563.31	**48746**	**1.0775**	**0.32562**
Q8BNY6	Ncs1	Neuronal calcium sensor 1	16201.23	19635.07	15005.04	17173.56	**17004**	20039.88	22709.71	19135.82	15701.28	**19397**	**0.8766**	**0.22026**
Q9WV18	Gabbr1	Gamma-aminobutyric acid type B receptor subunit 1	32833.53	34959.2	33320.87	43400.17	**36128**	31834.37	39067.95	36532.21	36397.87	**35958**	**1.0047**	**0.95490**
Q3TDN2	Faf2	FAS-associated factor 2	70874.93	70537.93	71088.1	83405.69	**73977**	69644.92	77581.25	82851.34	83510.05	**78397**	**0.9436**	**0.36293**
Q9DAK9	Phpt1	14 kDa phosphohistidine phosphatase	17127.86	18461.46	16688.25	11883.95	**16040**	14924.47	18538.02	14126.22	13572.51	**15290**	**1.0491**	**0.69449**
Q64727	Vcl	Vinculin	373661.6	380397.2	385773.3	370328.8	**377540**	385992.2	376689.3	387268.3	365877.3	**378957**	**0.9963**	**0.82238**
Q9Z0Y1	Dctn3	Dynactin subunit 3	101454.5	99436.68	94179.91	99584.92	**98664**	96452.02	100715.9	99303.79	99463.13	**98984**	**0.9968**	**0.86522**
Q9DBG6	Rpn2	Dolichyl-diphosphooligosaccharide--protein glycosy	211977.5	215366.5	226264.4	233196.2	**221701**	218511.9	230577.6	248783.7	248800.2	**236668**	**0.9368**	**0.14324**
Q2NL51	Gsk3a	Glycogen synthase kinase-3 alpha	29977.14	25705.52	26998.54	23705.28	**26597**	26715.06	25501.14	21684.18	24385.54	**24571**	**1.0824**	**0.27787**
P49442	Inpp1	Inositol polyphosphate 1-phosphatase	81127.3	83690.84	70822.2	58738.52	**73595**	68397.44	70667.2	67904.03	66612.65	**68395**	**1.0760**	**0.40010**
Q8BW41	Pomgnt2	Protein O-linked-mannose beta-1,4-N-acetylglucosa	66944.87	60885.8	63608.64	65829.59	**64317**	62257.13	71959.98	65610.85	69596.8	**67356**	**0.9549**	**0.27472**
Q9JMB0	Gkap1	G kinase-anchoring protein 1	21135.31	21275.69	21343.53	25566.29	**22330**	23380.72	23984.74	28860.74	24467.67	**25173**	**0.8871**	**0.13581**
Q7SIG6	Asap2	Arf-GAP with SH3 domain, ANK repeat and PH domai	11692.11	12833.14	10393.14	10716.05	**11409**	10729.35	11582.42	11398.69	15005.62	**12179**	**0.9367**	**0.51211**
Q921W0	Chmp1a	Charged multivesicular body protein 1a	41333.93	43611.98	38537.23	32917.05	**39100**	36116.64	39784.79	29126.17	37652.24	**35670**	**1.0962**	**0.33366**
Q8R138	Tmem119	Transmembrane protein 119	16028.45	16862.07	17733.86	16241.63	**16717**	15517.02	12659.38	17612.03	18133.47	**15980**	**1.0461**	**0.59196**
O88983	Stx8	Syntaxin-8	27573.77	25628.17	25708.62	28868.55	**26945**	28128.21	28624.83	27384.83	30536.36	**28669**	**0.9399**	**0.14600**
Q9D2P8	Mobp	Myelin-associated oligodendrocyte basic protein							30461.77			**30462**	**0.0000**	
Q9CQI7	Snrpb2	U2 small nuclear ribonucleoprotein B''	27240.29	24829.61	29922.33	26386.6	**27095**	27500.42	26476.35	27085.21	32577.67	**28410**	**0.9537**	**0.48409**
Q9R1Z8	Sorbs3	Vinexin	41945.2	38374.7	45992.18	47212.27	**43381**	38165.07	37158.59	54210.98	52123.13	**45414**	**0.9552**	**0.69437**
Q9D2Z4	Senp8	Sentrin-specific protease 8	9346.579	10818.3	7581.825	5664.393	**8353**	8091.844	6840.47	4190.043	5952.013	**6269**	**1.3325**	**0.18259**
Q9ERS2	Ndufa13	NADH dehydrogenase [ubiquinone] 1 alpha subcom	270836.8	288320.6	344123.5	355489	**314692**	308752.4	297707.7	321411.9	348836.9	**319177**	**0.9859**	**0.85469**
O09131	Gsto1	Glutathione S-transferase omega-1	50565.29	47204.19	43486.63	42121.42	**45844**	40452.62	42045.66	39286.18	39639.54	**40356**	**1.1360**	**0.03365**
Q91W92	Cdc42ep1	Cdc42 effector protein 1	27922.74	29351.09	32677.62	41105.7	**32764**	29500.03	28598.38	39159.48	34590.62	**32962**	**0.9940**	**0.96056**
P43006	Slc1a2	Excitatory amino acid transporter 2	41878.46	52006.71	9786.113	2678.166	**26587**	9358.593	17190.01	11212.75		**12587**	**2.1123**	**0.37435**
A2A6T1	Cdr2l	Cerebellar degeneration-related protein 2-like	34261.83	39372.45	36005.47	37546.54	**36797**	32887.45	36546.93	28680.23	36101.89	**33554**	**1.0966**	**0.17689**
Q9Z0E0	Ncdn	Neurochondrin	200467.7	236953.2	207250.4	180843.1	**206379**	187192	214817.3	187798.5	169373.4	**189795**	**1.0874**	**0.30933**
Q8K4Q7	Cerk	Ceramide kinase	6747.658	4864.964	4713.951	3906.135	**5058**	6949.462	6552.787	149.039	5096.768	**4687**	**1.0792**	**0.83206**
Q91VH2	Snx9	Sorting nexin-9	48772.25	45981.24	41148.63	41578.41	**44370**	46130.28	48103.49	44535.63	43849.17	**45655**	**0.9719**	**0.55571**
Q8K358	Pigu	Phosphatidylinositol glycan anchor biosynthesis clas	20232.25	20986.2	20190.8	19052.91	**20116**	22379.37	21225.71	16063.5	19709.25	**19844**	**1.0137**	**0.85594**
O35684	Serpini1	Neuroserpin	15419.61	16237.95	14479.29	16710.52	**15712**	13423.97	17113.6	13248.51	15201.15	**14747**	**1.0654**	**0.38410**
Q9CPP0	Npm3	Nucleoplasmin-3	15028.57	12262.17	14182.01	13186.32	**13665**	11761.33	11841.31	14565.55	15659.45	**13457**	**1.0154**	**0.86260**
Q91W18	Tdrd3	Tudor domain-containing protein 3	20152.59	24327.67	23741.3	24076.41	**23074**	21284.44	23837.57	22462.41	23999.01	**22896**	**1.0078**	**0.88372**
P84096	Rhog	Rho-related GTP-binding protein RhoG	100903.8	79489.38	85488.97	94721.22	**90151**	77099.75	93642.64	66248.2	83972.7	**80241**	**1.1235**	**0.23334**
Q6DIC0	Smarca2	Probable global transcription activator SNF2L2	14496.07	7708.531	15664.73	13114.65	**12746**	13829.89	15492.23	13412.98	14453.6	**14297**	**0.8915**	**0.42564**
Q8CH25	Sltm	SAFB-like transcription modulator	49393.26	43715.05	53311.13	64554.64	**52744**	50938.97	56948.48	64728.23	49271.12	**55472**	**0.9508**	**0.64475**
Q64343	Abcg1	ATP-binding cassette sub-family G member 1	12723.47	11063.51	8814.266	10848.57	**10862**	12113.41	11286.05	8498.342	14681.37	**11645**	**0.9328**	**0.62172**
Q8BL65	Ablim2	Actin-binding LIM protein 2	33968.67	32919.71	27212.75	39243.75	**33336**	36646.65	35017.27	28680.68	31787.96	**33033**	**1.0092**	**0.92368**
Q8CBE3	Wdr37	WD repeat-containing protein 37	27360.09	26690.2	24666.26	25572.07	**26072**	24558.26	24452.87	23446.29	25584.16	**24510**	**1.0637**	**0.07909**
P63046	Sult4a1	Sulfotransferase 4A1	53772.75	52227.98	41416.43	40539.66	**46989**	47765.04	47863.68	45900.26	40433.09	**45491**	**1.0329**	**0.71413**
Q9CPR4	Rpl17	Large ribosomal subunit protein uL22	306637.4	313831	341683.5	364663.3	**331704**	328171.6	345045.7	384990.9	393417.6	**362906**	**0.9140**	**0.18010**
Q9Z1X4	Ilf3	Interleukin enhancer-binding factor 3	70797.42	72286.93	77087.38	80163.27	**75084**	68000.77	75323.18	79163.38	80610.95	**75775**	**0.9909**	**0.85227**
Q9JJ59	Abcb9	ABC-type oligopeptide transporter ABCB9	21172.79	21235.29	17240.19	21915.38	**20391**	21265.58	24360.63	15295.35	17758.89	**19670**	**1.0366**	**0.75987**
P63044	Vamp2	Vesicle-associated membrane protein 2	62155.8	61255.4	63629.81	80338.73	**66845**	73871.13	67102.81	66386.23	77818.83	**71295**	**0.9376**	**0.43295**
Q8K0E8	Fgb	Fibrinogen beta chain	203687.2	167928.3	241198	289218.6	**225508**	148447.3	125266.1	190590	143537.1	**151960**	**1.4840**	**0.04652**
Q9EQP2	Ehd4	EH domain-containing protein 4	148809.4	146584.4	135704.6	143892.3	**143748**	131522.8	142902.7	144963.6	141849.6	**140310**	**1.0245**	**0.43879**
Q61292	Lamb2	Laminin subunit beta-2	850572.1	697006.9	447354	836758.9	**707923**	705539.4	842674.1	524888.4	522899.9	**649000**	**1.0908**	**0.64474**
P45952	Acadm	Medium-chain specific acyl-CoA dehydrogenase, mi	354142.4	333414.9	364316.2	404816.6	**364173**	347570.2	332185.8	351575.9	384227.1	**353890**	**1.0291**	**0.59965**
Q3UYG8	Macrod2	ADP-ribose glycohydrolase MACROD2	7892.561	9037.335	5523.111	4287.239	**6685**	6624.929	2500.805	435.9595	2752.833	**3079**	**2.1714**	**0.07618**
O54824	Il16	Pro-interleukin-16	21409.1	26720.24	17341.16	24180.69	**22413**	16237.97	30794.52	29252.95	22564.04	**24712**	**0.9069**	**0.57688**
Q9Z2M7	Pmm2	Phosphomannomutase 2	45608.1	45363.31	42553.93	36779.32	**42576**	40801.12	36240.25	33811.74	30125.46	**35245**	**1.2080**	**0.05229**
Q08943	Ssrp1	FACT complex subunit SSRP1	19434.13	22807.11	21677.5	23130.07	**21762**	22433.1	21168.7	21157.52	28249.48	**23252**	**0.9359**	**0.45998**

P50136	Bckdha	2-oxoisovalerate dehydrogenase subunit alpha, mitc	26537.3	27158.32	28973.14	30465.87	28284	27594.87	29753.28	30074.8	30011.58	29359	0.9634	0.35418
Q9D1M0	Sec13	Protein SEC13 homolog	45039.64	50941.04	46161.49	41333.54	45869	47705.48	51222.77	44251.63	38309.83	45372	1.0109	0.88834
Q99PU5	Acsbg1	Long-chain-fatty-acid--CoA ligase ACSBG1	92856.23	98526.51	87367.3	100345.7	94774	87395.05	117186.7	75506.13	87231.73	91830	1.0321	0.76405
Q9QZE7	Tsnax	Translin-associated protein X	38150.04	38530.36	32787.21	31257.47	35181	35999.66	36072.06	26753.85	25615.73	31110	1.1309	0.27653
P97820	Map4k4	Mitogen-activated protein kinase kinase kinase kinas	84585.52	99578.83	97553.19	105580.3	96824	97185.89	92339.53	113807.5	122616.3	106487	0.9093	0.29072
Q3THE2	Myl12b	Myosin regulatory light chain 12B	230782	269097.7	203010.6	198896.3	225447	213129.2	228587.9	213418.2	199440.3	213644	1.0552	0.51920
Q921M7	Cyrib	CYFIP-related Rac1 interactor B	81445.71	71650.97	70531.18	63552.01	71795	67977.66	68280.66	62478.61	65522.99	66065	1.0867	0.19416
P50543	S100a11	Protein S100-A11	163826.1	181838.8	190890.1	148932.3	171372	185431.3	144769	141490.5	154344.4	156509	1.0950	0.31995
Q9QZB7	Actr10	Actin-related protein 10	42294.94	44021.22	41968.1	41780.58	42516	40373.61	44100.7	44040.64	45928.45	43611	0.9749	0.42270
O08989	Mras	Ras-related protein M-Ras	47682.83	44465.59	40574.29	59051.03	47943	49819.1	53105.24	44821.98	49927.48	49418	0.9702	0.74498
Q8K0D7	Get1	Guided entry of tail-anchored proteins factor 1	32918.18	36918.63	39790.55	43335.86	38241	35817.07	43432.62	37693.71	40620.11	39391	0.9708	0.69223
Q3TC93	Hs1bp3	HCLS1-binding protein 3	17225.86	18555.59	15080.84	11180.63	15511	15503.05	12483.86	11177.04	13234.79	13100	1.1841	0.23999
Q91VM5	Rbmxl1	RNA binding motif protein, X-linked-like-1	196254	213857.6	240817.4	273674.4	231151	265420	243519.6	336120	289492.4	283638	0.8150	0.09063
B1AVZ0	Uprt	Uracil phosphoribosyltransferase homolog	26771	28803.65	28360.01	28927.37	28216	30422.72	31246.89	22733.29	26581.82	27746	1.0169	0.82382
P56213	Gfer	FAD-linked sulfhydryl oxidase ALR	9026.983	8387.75	9949.544	11820.15	9796	10403.96	14606.43	12654.46	16718.75	13596	0.7205	0.04887
Q99LH2	Ptdss1	Phosphatidylserine synthase 1	65433.04	67066.13	63891.73	66937.45	65832	66396.54	78521.42	67692.95	66750.17	69840	0.9426	0.23006
Q3UDP0	Wdr41	WD repeat-containing protein 41	9248.402	12553.45	12126.68	10273.78	11051	14201.14	19540.14	10344.5	13158.11	14311	0.7722	0.16724
A2ASQ1	Agrn	Agrin	26836.95	26641.66	23108.41	33768.52	27589	27883.06	25545.53	24473.98	27980.85	26471	1.0422	0.65712
Q80XQ2	Tbc1d5	TBC1 domain family member 5	11249.53	16016.73	10389.33	10517.31	12043	16542.44	10656.75	8586.559	14996.92	12696	0.9486	0.78478
Q9WVG6	Carm1	Histone-arginine methyltransferase CARM1	36056.89	36056.66	39924.06	32522.93	36140	40662.59	31603.4	19166.71	30680.66	30528	1.1838	0.27367
Q8BPE4	Tmem177	Transmembrane protein 177	12713.22	11814.51	13632.2	12301.94	12615	12431.73	14876.97	15126.67	16299.85	14684	0.8591	0.06106
P60824	Cirbp	Cold-inducible RNA-binding protein	54645.47	47466.56	40546.32	50941.08	48400	36123.05	66909.61	47872.48	38680.41	47396	1.0212	0.89920
Q6NVG1	Lpcat4	Lysophospholipid acyltransferase LPCAT4	32557.96	33344.23	35068.04	32298.88	33317	36032.8	31510.94	39795.82	39390.84	36683	0.9083	0.14645
P62301	Rps13	Small ribosomal subunit protein uS15	981090.8	934076.4	1092541	1100591	1027075	1016204	1030595	1196913	1215829	1114885	0.9212	0.23926
Q80Y98	Ddhd2	Phospholipase DDHD2	9502.584	8525.334	9381.081	6226.737	8409	7978.293	6492.348	3827.465	6725.298	6256	1.3442	0.11202
Q8C6I2	Sdhaf2	Succinate dehydrogenase assembly factor 2, mitoch	5727.206	6576.294	5818.498	6006.89	6032	6888.104	6323.639	6361.079	6775.599	6587	0.9158	0.05884
Q8BG39	Sv2b	Synaptic vesicle glycoprotein 2B		16165.34		11781.99	13974		25503.11	12533.18		19018	0.7348	0.53789
Q8BG95	Ppp1r12b	Protein phosphatase 1 regulatory subunit 12B	106266	91325.02	88806.04	97841.68	96060	84988.69	95589.55	76392.63	90160.73	86783	1.1069	0.15147
Q9CR29	Ccdc43	Coiled-coil domain-containing protein 43				77590.75	77591	63697.32	44870.15	71822.4	22949.91	50835	1.5263	
P61358	Rpl27	Large ribosomal subunit protein eL27	842593	745876.5	932708.3	956676.7	869464	843833.2	798006.1	1008446	993635.8	910980	0.9544	0.58219
Q64523;Q6GSS7	H2ac20;Hist2h2aa1	Histone H2A type 2-C;Histone H2A type 2-A	142745.1	92755.7	159858.5	276101.3	167865	171658.8	57632.29	182519.8	194408.7	151555	1.1076	0.75563
P51910	Apod	Apolipoprotein D	254977.7	234814.2	210011.7	207103.2	226727	222642.9	251177.6	217091.8	209559.8	225118	1.0071	0.91522
Q8VD57	Sft2d2	Vesicle transport protein SFT2B	23804.95	24587.98	15357.03	34336.86	24522	27739.11	29750	16985.92	19118.73	23398	1.0480	0.82956
Q8BVE3	Atp6v1h	V-type proton ATPase subunit H	106569.2	109317.6	93032.02	95331.66	101063	106054.2	114568.2	104441.2	97575.2	105660	0.9565	0.42235
Q9R062	Gyg1	Glycogenin-1	144385.7	165410.5	150084.8	115099.4	143745	145745	124798.7	132528	126179.7	132313	1.0864	0.36109
Q9CZX8	Rps19	Small ribosomal subunit protein eS19	243035.7	241770.2	266467.1	283192.4	258616	246691.3	254155.4	285977.1	286593	268354	0.9637	0.52552
Q9CQ19	Myl9	Myosin regulatory light polypeptide 9	85272.21	65204.65	98495.22	79649.95	82156	70111.19	66027.39	75202.59	82921.87	73566	1.1168	0.31275
Q80XI3	Eif4g3	Eukaryotic translation initiation factor 4 gamma 3	65605.36	70503.98	59799.64	62202.11	64528	57425.36	67461.6	64145.32	61600.12	62658	1.0298	0.57348
Q8BK67	Rcc2	Protein RCC2	58782.2	61330.56	61686.67	55334.87	59284	62799.84	51794.04	66374.48	60309.11	60319	0.9828	0.77296
Q99MR0	Actl6b	Actin-like protein 6B	18154.49	20176			19165		20727.75	20152.37		20440	0.9376	0.34894
Q8BH24	Tm9sf4	Transmembrane 9 superfamily member 4	53516.32	48716.09	57863.46	53314.7	53353	60595.3	58934.29	64165.95	74116.4	64453	0.8278	0.02877
Q63739	Ptp4a1	Protein tyrosine phosphatase type IVA 1	25898.07	24305.94	15434.26	23944.79	22396	19333.97	24471.23	15722.23	20663.42	20048	1.1171	0.45947
Q923D5	Wbp11	WW domain-binding protein 11	22824.96	21268.4	25358.96	24972.4	23606	25723.38	20993.95	24192	25283.57	24048	0.9816	0.76839
Q8C008	Dzank1	Double zinc ribbon and ankyrin repeat-containing pro	21391.11	22071.9	15050.94	20416.09	19733	18805.37	18886.49	20992.12	19592.02	19569	1.0084	0.92543
Q3UBX0	Tmem109	Transmembrane protein 109	29133.18	30097.16	36644.63	36393.11	33067	31383.95	28126.34	23542.08	27549.45	27650	1.1959	0.07957
Q7TT37	Elp1	Elongator complex protein 1	28338.07	26811.3	31375.79	27981.23	28627	30190.7	29430.85	32382.73	27696.55	29925	0.9566	0.38127
Q9CQ89	Cuta	Protein CutA	88574.18	95792.34	81223	55616.82	80302	83331.03	76684.55	71481.7	73213.05	76178	1.0541	0.66744
P09803	Cdh1	Cadherin-1	89962.77	89362.69	78560.52	90002.34	86972	62197.58	90848.08	90785.81	97213.74	85261	1.0201	0.84393
P21995	Emb	Embigin	35679.81	38674.29	39391.06	45658.38	39851	42260.57	42106.38	45518.9	41599.63	42871	0.9295	0.23322
Q9QZF2	Gpc1	Glypican-1	22052.2	20163.99	16070.88	19042.85	19332	20049.86	20380.93	15951.29	18816.81	18800	1.0283	0.75151
Q91WV0	Dr1	Protein Dr1	25145.16	26435.18	35600.37	20956.21	27034	28941.92	25590.97	39850.73	29156.64	30885	0.8753	0.41236
Q5PR73	Diras2	GTP-binding protein Di-Ras2	56683.31	49888.16	38799.79	41736.55	46777	51016.82	45787	42376.5	40640.84	44955	1.0405	0.70882
Q8VCN9	Tbcc	Tubulin-specific chaperone C	29438.11	28022.83	26755.54	22138.16	26589	28623.97	27800.97	27565.62	25270.71	27315	0.9734	0.69024
P0C7M9	Clec2l	C-type lectin domain family 2 member L	48524.34	53930.97	32185.64	54141.89	47196	40444.08	53340.33	39949.77	35204.94	42235	1.1175	0.47211
Q8R464	Cadm4	Cell adhesion molecule 4	1327235	1196409	1011133	1323366	1214536	1194778	1275403	1055761	1070651	1149148	1.0569	0.49898
A2AKG8	Focad	Focadhesin		5907.126	7748.744		6828	6373.315	6152.027	9776.18	4940.891	6811	1.0025	0.99215
Q9JL35	Hmgn5	High mobility group nucleosome-binding domain-co	14718.72	10369.86	16770.91	22852.83	16178	11550.62	10337.79	16175.66	18658.55	14181	1.1409	0.56099
Q8CGK3	Lonp1	Lon protease homolog, mitochondrial	121249.5	119207.6	136484.4	151234.1	132044	134200.8	129727.6	132630.7	137999.9	133640	0.9881	0.84195
Q505B7	Zbtb8os	Protein archease		21223.9	21174.41	18031.29	20143		14378.19	15788.31	20580.84	16916	1.1908	0.20838

O35381	Anp32a	Acidic leucine-rich nuclear phosphoprotein 32 family	46396.8	48403.02	57889.48	46174.28	49716	47373.69	46476.26	52129.52	51436.77	49354	1.0073	0.91128
A2RSJ4	Bltp3b	Bridge-like lipid transfer protein family member 3B	10765.99	13762.68	8584.091	26093.39	14802	10238.81	34877.49	6185.959	8246.131	14887	0.9943	0.99157
O70309	Itgb5	Integrin beta-5	20288.02	19217.37	18689.44	20213.26	19602	17092.62	18435.66	15097.43	20105.56	17683	1.1085	0.14004
Q9D1J1	Necap2	Adaptin ear-binding coat-associated protein 2	18859.58	18261.49	22029.79	13451.44	18151	14282.1	17966.71	13811.24	15420.78	15370	1.1809	0.21387
Q8R1X6	Spart	Spartin	36589.92	39483.37	38282.7	31823.68	36545	35678	36248.05	33273.4	37485.57	35671	1.0245	0.66184
Q99KP6	Prpf19	Pre-mRNA-processing factor 19	111981.8	106767.1	118253.4	115595.3	113149	109249	108705.4	136667.7	130068.3	121173	0.9338	0.33106
Q9D7B6	Acad8	Isobutyryl-CoA dehydrogenase, mitochondrial	54609.96	51481.36	54041.31	64632.96	56191	55013.45	59364.09	53377.5	50178.41	54483	1.0313	0.63997
Q00493	Cpe	Carboxypeptidase E	32246	35480.06	33880.02	38581.51	35047	32220.79	41017.65	39125.42	36923.16	37322	0.9390	0.36603
Q9QZX7	Srr	Serine racemase	1093.813	7720.681	4394.693		4403	3190.01	5526.483	5.938489	974.6715	2424	1.8162	0.40233
P05202	Got2	Aspartate aminotransferase, mitochondrial	797312.5	760902.2	795999.1	868555.5	805692	789172.6	856174.9	792305	857961	823903	0.9779	0.56125
Q9WU60	Atrn	Attractin	7166.887	9877.483	7576.71	9730.073	8588	10785.39	8469.82	10114.63	9277.128	9662	0.8888	0.26232
Q9Z1J3	Nfs1	Cysteine desulfurase	45915.33	42149.34	45997.59	50893.73	46239	44466.88	49676.67	50831.85	51533.06	49127	0.9412	0.27462
Q8BFY9	Tnpo1	Transportin-1	52167.61	55377.01	49299.47	49762.74	51652	47654.97	52987.43	50097.95	53986.93	51182	1.0092	0.82204
P47753	Capza1	F-actin-capping protein subunit alpha-1	88890.87	86418.08	87786.19	80011.98	85777	79807.65	86762.04	82388.45	68621.04	79395	1.0804	0.19254
Q6ZQI3	Mlec	Malectin	172012.2	171161.4	189111.6	184301.2	179147	179296.8	181264.9	209281.6	212827.7	195668	0.9156	0.14900
Q3UHJ0	Aak1	AP2-associated protein kinase 1	80797.52	81641.03	71533.23	76200.27	77543	71660.55	76203.66	68697.13	74255.55	72704	1.0666	0.13984
Q8BRU6	Slc18a2	Synaptic vesicular amine transporter												
Q8BH64	Ehd2	EH domain-containing protein 2	217968.9	190675.1	208104.4	183225	199993	182605.5	168245.2	192393.4	181935.4	181295	1.1031	0.09287
Q62000	Ogn	Mimecan	1045741	1024625	824280.4	1057760	988102	948553.9	962838.6	601658.7	776604.9	822414	1.2015	0.15257
O54734	Ddost	Dolichyl-diphosphooligosaccharide--protein glycosy	318081.8	286098.2	309445.7	322775.3	309100	309396.6	333985.1	332650	365442.1	335368	0.9217	0.11172
O55003	Bnip3	BCL2/adenovirus E1B 19 kDa protein-interacting pro	15740.76	15830.76	11387.95	18470.71	15358	17159.04	16951.83	14359.04	12410.7	15220	1.0090	0.94331
P62192	Psmc1	26S proteasome regulatory subunit 4	141725.7	140574.3	137911.4	123831.2	136011	132970	132244.1	137572.4	124445.7	131808	1.0319	0.42873
Q8K2Y9	Ccm2	Cerebral cavernous malformations protein 2 homolc	10633.5	10219.64	12023.22	6514.549	9848	9154.077	11143.09	11367.87	10579.49	10561	0.9324	0.59661
P14206	Rpsa	Small ribosomal subunit protein uS2	888619.3	871746.3	956326.6	972777.3	922367	929834.3	943746.9	1046078	1100822	1005120	0.9177	0.13558
P36993	Ppm1b	Protein phosphatase 1B	17123.66	13808.85	14670.22	8535.35	13535	11209.45	12121.2	11257.41	10990.76	11395	1.1878	0.28552
Q922B1	Macrod1	ADP-ribose glycohydrolase MACROD1	42585.49	40540.64	39758.85	46988.25	42468	45402.63	43417.24	35041.53	39517.54	40845	1.0397	0.58361
Q9DBR1	Xrn2	5'-3' exoribonuclease 2	15893.49	15548.82	18246.3	18428.83	17029	18769.65	22746.66	19073.66	20907.53	20374	0.8358	0.03108
Q924L1	Letmd1	LETM1 domain-containing protein 1	21339.6	18098.15	23288.3	27325.75	22513	20667.22	24822.34	21052.36	21003.96	21886	1.0286	0.78197
Q9CWW6	Pin4	Peptidyl-prolyl cis-trans isomerase NIMA-interacting	16554.29	18305.98	13113.08	18138.74	16528	21044.01	13800.15	15903.46	13265.77	16003	1.0328	0.81487
Q8R3I3	Cog6	Conserved oligomeric Golgi complex subunit 6	25174.46	22650.28	23426.69	22654.86	23477	22897.53	22815.92	26892.24	22941.16	23887	0.9828	0.73690
O55026	Entpd2	Ectonucleoside triphosphate diphosphohydrolase 2	256054.1	233290.4	215089.4	277598.2	245508	270384.2	255635.7	261451.8	267156.2	263657	0.9312	0.24161
P63101	Ywhaz	14-3-3 protein zeta/delta	550734.3	586743.8	567678.9	544734.6	562473	632620.7	550873.8	592598.8	568221.4	586079	0.9597	0.28416
Q9Z1Z0	Uso1	General vesicular transport factor p115	157849.5	159744.3	155082.3	139087.4	152941	160695.2	159584.1	176230.9	166559.9	165768	0.9226	0.07872
Q8BXV2	Bri3bp	BRI3-binding protein	19877.03	20555.4	24166.7	21407.68	21502	20089.28	22965.87	24404.35	26957.43	23604	0.9109	0.26613
Q8BZN6	Dock10	Dedicator of cytokinesis protein 10	19914.37	17968.64	16956.08	18824.64	18416	18427.33	16426.68	19212.66	21206.1	18818	0.9786	0.74306
Q99P72	Rtn4	Reticulon-4	548397.4	587193.2	596173.2	681619.1	603346	582540.8	598272	661071.2	635400.5	619321	0.9742	0.64778
Q9QYS2	Grm3	Metabotropic glutamate receptor 3												
Q60857	Slc6a4	Sodium-dependent serotonin transporter			930.6943		931							
Q9EQN3	Tsc22d4	TSC22 domain family protein 4	23407.74	16504.31	19658.86	15115.57	18672	20318.87	17730.69	16345.59	21613.53	19002	0.9826	0.88539
P35700	Prdx1	Peroxiredoxin-1	1095681	1028603	1165393	956601.3	1061570	1001371	965604.7	1100149	1009716	1019210	1.0416	0.45572
Q80TR4	Slit1	Slit homolog 1 protein	19871.98	22876.86	18731.73	18312.14	19948	17961.79	20081.86	16987.39	19139.92	18543	1.0758	0.29753
Q9Z1G3	Atp6v1c1	V-type proton ATPase subunit C 1	85793.12	84060.98	69104.98	67499.33	76615	73694.99	82809.47	62481.61	64542.93	70882	1.0809	0.42565
O55103	Prx	Periaxin	5281937	4361246	3685928	6160236	4872337	4461244	5075771	4085274	4451117	4518352	1.0783	0.56236
Q924C1	Xpo5	Exportin-5	14500.04	12887.89	14223.89	8916.077	12632	13836.55	12594.53	11826.37	12024.01	12570	1.0049	0.96546
Q8C3X8	Lmf2	Lipase maturation factor 2	24832.53	26180.64	36286.95	31070.63	29593	29338.28	28802.13	31754.3	37993.23	31972	0.9256	0.50408
P56873	Znrd2	Protein ZNRD2	23480.77	28594.72	30622.22	25558.55	27064	27051.67	23832.84	38771.6	30991.07	30162	0.8973	0.42135
Q9WVD5	Slc25a15	Mitochondrial ornithine transporter 1	12945.95	9075.767	9948.886	13334.53	11326	13174.42	14697.03		8048.995	11973	0.9459	0.77060
O35435	Dhodh	Dihydroorotate dehydrogenase (quinone), mitochon	59369.14	55935.55	56952.59	61654.93	58478	51500.28	57400.29	48758.34	61223.06	54720	1.0687	0.27054
Q62059	Vcan	Versican core protein	29967.1	33866.3	32520.36	30677.12	31758	30877.79	32944.1	22255.8	22212.93	27073	1.1731	0.16462
Q9D8S4	Rexo2	Oligoribonuclease, mitochondrial	46552.93	45346.68	39140.95	48802.89	44961	50385.2	49768.62	59206.33	45407.88	51192	0.8783	0.13021
Q8CH09	Sugp2	SURP and G-patch domain-containing protein 2	9773.75	9584.907	10617.28	8446.66	9606	9549.864	8864.2	7244.156	10894.54	9138	1.0512	0.61472
Q8VHJ5	Mark1	Serine/threonine-protein kinase MARK1	17493.08	22690	14763.99	21898.12	19211	17989.94	18405.42	14097.92	17869.6	17091	1.1241	0.35670
P52503	Ndufs6	NADH dehydrogenase [ubiquinone] iron-sulfur prote	59462.38	56899.54	65069.8	75187.23	64155	60242.53	66386.34	73872.29	75161.38	68916	0.9309	0.40710
Q9DC50	Crot	Peroxisomal carnitine O-octanoyltransferase	39894.92	42065.93	51384.64	36621.95	42492	42373.21	43605.77	54824.89	48199.6	47251	0.8993	0.30466
Q8BGC4	Ptgr3	Prostaglandin reductase-3	66390.59	72363.08	66138.09	76214.37	70277	71330.15	77467.26	71618.99	76425.12	74210	0.9470	0.22665
Q64018	Glra1	Glycine receptor subunit alpha-1												
P56389	Cda	Cytidine deaminase	73606.51	86247.63	77847.07	71476.13	77294	84379.75	65772.02	73415.69	67629.74	72799	1.0617	0.42983
Q9CXV1	Sdhd	Succinate dehydrogenase [ubiquinone] cytochrome	20015.84	21484.54	14150.6	17920.99	18393	18710.09	26660.3	13730.74	14555.67	18414	0.9988	0.99517
Q9D8B6	Fam210b	Protein FAM210B, mitochondrial	6389.839	10756.56	8338.372	9181.048	8666	4936.964	13956.79		7581.281	8825	0.9820	0.95159

Q6P9J9	Ano6	Anoctamin-6	16715.6	15092.89	15579.03	16653.18	16010	16301.08	16398.39	13047.48	14156.83	14976	1.0691	0.30283
Q62165	Dag1	Dystroglycan 1	498195.2	484994.6	418358.8	542121.8	485918	479072.8	514003.9	467125.7	455633.6	478959	1.0145	0.81561
Q9EPL2	Clstn1	Calsyntenin-1	9220.714	12313.11	12055.59	12206.89	11449	10672.56	10812.56	10649.74	11028.35	10791	1.0610	0.41371
P60840	Ensa	Alpha-endosulfine	18419.29	19540.04	20787.07	16341.07	18772	15348.87	16886.97	17070.88	15974.67	16320	1.1502	0.05407
Q9CW03	Smc3	Structural maintece of chromosomes protein 3	34758.42	33572.85	38463.08	40061.55	36714	37269.53	33373.48	40844.07	40969.84	38114	0.9633	0.57440
Q9D1E8	Agpat5	1-acyl-sn-glycerol-3-phosphate acyltransferase epsi	12641.77	16879.93	15050.3	15694.54	15067	16090.2	15941.87	15026.75	16084.45	15786	0.9544	0.46800
Q6SKR2	N6amt1	Methyltransferase N6AMT1	13235.79	15241.96	13749.74	10259.32	13122	9026.79	14183.2	7375.376	8288.817	9719	1.3502	0.11535
Q91ZV0	Mia2	Melanoma inhibitory activity protein 2	26363.26	26787.49	27204.2	28757.69	27278	26448.02	31095.88	32828.21	31998.51	30593	0.8917	0.07182
Q03141	Mark3	MAP/microtubule affinity-regulating kinase 3	24570.34	25767.44	26980.49	27736.59	26264	28011	23218.95	24029.31	27169.58	25607	1.0256	0.64650
Q99KY4	Gak	Cyclin-G-associated kinase	44358.88	39962.79	45030.69	46291.64	43911	42834.18	42238	45378.79	45214.91	43916	0.9999	0.99737
Q91VX2	Ubap2	Ubiquitin-associated protein 2	56444.73	54677	58893	67938.95	59488	61784	62529.15	71504.43	70326.23	66536	0.8941	0.12023
Q61316	Hspa4	Heat shock 70 kDa protein 4	344110.6	349632.5	341823.4	330939.7	341627	370819.9	343347.1	354149.1	350591.7	354727	0.9631	0.11104
Q9JIG7	Ccdc22	Coiled-coil domain-containing protein 22	54205.97	43139.79	52000.99	45811.95	48790	46348.68	48844.05	55417.38	48354.51	49741	0.9809	0.77968
G5E8K5	Ank3	Ankyrin-3	180221.4	161614.4	168479	202611.8	178232	185007.7	167829.9	199877.5	208119.4	190209	0.9370	0.37931
Q78PY7	Snd1	Staphylococcal nuclease domain-containing protein	198028.2	192477	211016.5	212764.8	203572	193724.6	202768.6	221803	227275.4	211393	0.9630	0.43322
Q61097	Ksr1	Kinase suppressor of Ras 1	17576.74	19885.22	20694.78	10328.19	17121	18313.46	16586.63	17893.66	15359.99	17038	1.0049	0.97416
Q2TBE6	Pi4k2a	Phosphatidylinositol 4-kinase type 2-alpha	57993.56	63204.22	59893.46	62505.4	60899	60339.73	67300.83	64095.52	67818.24	64889	0.9385	0.10661
Q60770	Stxbp3	Syntaxin-binding protein 3	37157.61	38178.01	34036.19	40057.51	37357	38478.92	40858.1	37305.45	34673.77	37829	0.9875	0.80199
Q8BK03	Miga2	Mitoguardin 2	16697.9	15422.14	17052.35	16235.93	16352	16674.99	18759.02	19338.64	18056.25	18207	0.8981	0.03310
Q8BWQ6	Vps35l	VPS35 endosomal protein-sorting factor-like	13426.82	12626.34	12679.22	15128.67	13465	12701.3	13742.43	11303.04	12359.22	12526	1.0749	0.26881
P43276	H1-5	Histone H1.5	247294.9	253865.2	431144.9	410472.8	335694	246924.2	218151.5	505421.7	490981.4	365370	0.9188	0.75652
P28028	Braf	Serine/threonine-protein kinase B-raf	36544.26	33227.43	42582.5	41887.68	38560	46948.3	33439.21	28040.72	31721.96	35038	1.1005	0.48111
Q62448	Eif4g2	Eukaryotic translation initiation factor 4 gamma 2	57952.39	53818	63472.7	60411.95	58914	56651.76	60274.39	79251.2	65981.38	65540	0.8989	0.26264
Q60648	Gm2a	Ganglioside GM2 activator	27867.9	28865.47	33875.46	27548.03	29539	29366.43	28042.64	31595.73	37474.13	31620	0.9342	0.44610
P62196	Psmc5	26S proteasome regulatory subunit 8	125124.1	128527.7	131986.5	120954.2	126648	124501.8	123233.5	125189.8	126229	124789	1.0149	0.47506
Q9D6U8	Fam162a	Protein FAM162A	49266.69	49739.93	57706.77	60727.41	54360	53083.71	56326.04	46982.83	48970.32	51341	1.0588	0.42809
Q924T2	Mrps2	Small ribosomal subunit protein uS2m	12227.31	12929.06	15236.52	15565.25	13990	13664.76	15828.87	16381.16	17735.51	15903	0.8797	0.15777
Q61545	Ewsr1	RNA-binding protein EWS	58575.77	56381.17	74608.45	69299.48	64716	69482.95	53600.67	85943	62465.37	67873	0.9535	0.71033
Q62261	Sptbn1	Spectrin beta chain, non-erythrocytic 1	1180943	1177231	1031395	1249886	1159864	1141021	1186615	1033759	1049212	1102652	1.0519	0.36807
Q8R0A5	Tceal3	Transcription elongation factor A protein-like 3	39004.65	69618.22	61820.88	50930.46	55344	68154.9	29699.59	71279.37	78468.98	61901	0.8941	0.62715
Q9JMC3	Dnaja4	DnaJ homolog subfamily A member 4	46176.46	58259.45	52420.2	44762.13	50405	45694.34	49402.63	45214.59	47749.06	47015	1.0721	0.33710
P11531	Dmd	Dystrophin	95256.53	88581.02	96820.33	102763.6	95855	94510.57	96824.98	102251.9	107983.5	100393	0.9548	0.32008
Q61937	Npm1	Nucleophosmin	225403.5	208662.8	237839.5	217458.4	222341	205395	208951.4	223561.7	204632.6	210635	1.0556	0.17465
Q8K2H2	Otud6b	Deubiquitinase OTUD6B	31176.62	25487.04	24503.04	23865.95	26258	27326.93	25695.61	22287.99	24719.08	25007	1.0500	0.55037
Q922J6	Tspan2	Tetraspanin-2							174877.1			174877	0.0000	
Q91VR7	Map1lc3a	Microtubule-associated proteins 1A/1B light chain 3	509507.8	528327.8	468519.6	561179.8	516884	510250.9	610073.5	533668	521975.9	543992	0.9502	0.39653
Q5HZI9	Slc25a51	Mitochondrial nicotinamide adenine dinucleotide tra	39168.19	35454.26	38220.78	41425.45	38567	39498.88	36044.49	43322.3	46268.59	41284	0.9342	0.32760
Q99MR6	Srrt	Serrate RNA effector molecule homolog	29550.3	29041.42	35020.28	32207.42	31455	31534.41	33973.11	35633.05	33206.18	33587	0.9365	0.23578
P58802	Tbc1d10a	TBC1 domain family member 10A	25810.56	26838.81	30017.31	37364.93	30008	26784.35	26888.27	25371.57	30470.84	27379	1.0960	0.38841
Q80VQ0	Aldh3b1	Aldehyde dehydrogenase family 3 member B1	61786.27	57978.15	50498.02	66629.81	59223	54291.86	56094.51	56302.71	57847.64	56134	1.0550	0.40913
P28658	Atxn10	Ataxin-10	73157.62	80447.18	67203.75	57386.47	69549	66828.32	75462.27	63144.97	65459.04	67724	1.0269	0.75420
Q7M6Y3	Picalm	Phosphatidylinositol-binding clathrin assembly prote	28640.32	31018.05	31493.38	30983.47	30534	30005.78	30035.67	30855.22	32689.48	30897	0.9883	0.70051
Q9Z0H4	Celf2	CUGBP Elav-like family member 2	48281.79	42801.49	52888.22	45167.62	47285	45828.89	48520.07	51637.07	56906.62	50723	0.9322	0.32744
P46414	Cdkn1b	Cyclin-dependent kinase inhibitor 1B	35697.66	38404.92	36285.53	32274.49	35666	35184.79	28614.48	38427.13	32461.36	33672	1.0592	0.44476
Q3U2V3	Nudt18	8-oxo-dGDP phosphatase NUDT18	24512.91	19051.93	21268.49	19788.73	21156	25386.65	24568.75	34678.77	23205.52	26960	0.7847	0.09036
Q8R0Y8	Slc25a42	Mitochondrial coenzyme A transporter SLC25A42	9104.258	7263.605	9882.729	8605.569	8714	9097.579	9002.697	7697.727	8565.13	8591	1.0143	0.85279
Q9CPV4	Glod4	Glyoxalase domain-containing protein 4	165257.7	165856.5	163248.1	137872.2	158059	152292.1	156390.1	154635.3	143954.6	151818	1.0411	0.42491
Q8R1F1	Niban2	Protein Niban 2	70649.41	71851.77	64786.95	70591.23	69470	68343.91	72314.77	63940.48	63170.25	66942	1.0378	0.37716
Q8CFI0	Nedd4l	E3 ubiquitin-protein ligase NEDD4-like	40691.92	40288.89	40232.54	43470.96	41171	45175.12	40078.86	43850.29	43264.1	43092	0.9554	0.19852
P54754	Ephb3	Ephrin type-B receptor 3	37017.5	55672.45	43676.95	29751.92	41530	72826.17	47255.28	25183.09	32535.62	44450	0.9343	0.81382
Q9JLV6	Pnkp	Bifunctional polynucleotide phosphatase/kinase	23162.7	20072.2	25904.62	23491.31	23158	22116.34	21270.22	27311.52	27704.48	24601	0.9413	0.51187
Q9JI59	Jam2	Junctional adhesion molecule B	27420.7	23356.78	22239.12	26956.64	24993	24641.94	25051.31	22153.64	22859.82	23677	1.0556	0.40397
G5E897	Poglut3	Protein O-glucosyltransferase 3	27206.19	25826.5	30063.06	29809.07	28226	30004.58	26678.04	27018.96	33411.97	29278	0.9641	0.59489
Q00560	Il6st	Interleukin-6 receptor subunit beta	34913.28	39188.43	42304.9	44535.99	40236	38534.61	41989.91	41263.44	41777.2	40891	0.9840	0.77903
Q9DAU1	Cnpy3	Protein canopy homolog 3	30340.58	25738.84	38710.98	44599.48	34847	40390.36	35910.27	30887.51	44726.25	37979	0.9176	0.56604
Q8CJG1	Ago1	Protein argonaute-1	18020.49	16841.74	17967.7	17715.34	17636	18873.49	15128.59	16925.86	14847.98	16444	1.0725	0.26532
Q3U9G9	Lbr	Delta(14)-sterol reductase LBR	30595.9	24749.58	30581.15	28780.09	28677	24202.06	23423	23912.82	28699.57	25059	1.1444	0.09720
Q80UC6	Gpr62	G-protein coupled receptor 62												
Q9D7M1	Gid8	Glucose-induced degradation protein 8 homolog	34147.95	34566.04	29190.97	37183.75	33772	31812.52	34334.45	28894.29	32324.27	31841	1.0606	0.37409

O70176	Adcyap1	Pituitary adenylate cyclase-activating polypeptide												
P55014	Slc12a1	Solute carrier family 12 member 1	93544.84	83126.71	97129.04	92647.08	91612	101630.7	93506.35	59552.61	86196.88	85222	1.0750	0.53021
Q8BV13	Cops7b	COP9 signalosome complex subunit 7b	9156.633	10618.27	6278.028	3585.051	7409	9833.461	8445.971	3510.619	6134.507	6981	1.0614	0.84421
Q8BFT9	Svop	Synaptic vesicle 2-related protein							7839.39			7839	0.0000	
Q06335	Aplp2	Amyloid beta precursor like protein 2	25621.61	24416.22	22807.51	24084.72	24233	23609.42	27534.37	23773.88	28046.24	25741	0.9414	0.29720
P49025	Cit	Citron Rho-interacting kinase		12696.73		116711.5	64704	8540.499	16068.85		135891.3	53500	1.2094	0.87576
Q8BVG4	Dpp9	Dipeptidyl peptidase 9	42900.6	39957.95	43129.73	42338.26	42082	40180.62	43301.83	42546.46	44877.29	42727	0.9849	0.61561
P56375	Acyp2	Acylphosphatase-2	77550.88	70775.51	71267.85	50357.59	67488	70656.13	70075.34	62924.37	57069.08	65181	1.0354	0.74373
P49070	Camlg	Guided entry of tail-anchored proteins factor CAMLG	22291.46	22807.16	24323.68	26996.44	24105	24343.27	23620.1	21789.54	27304.83	24264	0.9934	0.92173
Q6PEB6	Mob4	MOB-like protein phocein	28548.55	29394.8	33762.77	30769.72	30619	37157.1	31741.76	39602.76	35876.09	36094	0.8483	0.03399
Q6ZQ18	Efr3b	Protein EFR3 homolog B	17697.73	14059.29	20037.84	19339.36	17784	17702.45	22062.58	17618.73	16077.1	18365	0.9683	0.76444
Q922R8	Pdia6	Protein disulfide-isomerase A6	462284.1	521885.6	573562.3	617827.7	543890	517307.8	481665.9	570948	627002.3	549231	0.9903	0.91170
P28740	Kif2a	Kinesin-like protein KIF2A	73460.1	76622.64	65771.64	68412.91	71067	71472.27	69003.92	70061.08	77475.58	72003	0.9870	0.77219
Q62193	Rpa2	Replication protein A 32 kDa subunit	16980.68	18844.37	16029.78	14126.46	16495	16550.25	16281.82	14607.45	16432.72	15968	1.0330	0.64377
P63082	Atp6v0c	V-type proton ATPase 16 kDa proteolipid subunit c	179261.4	115616.2	87918.65	208465.3	147815	181272.3	221936	120543.5	169832	173396	0.8525	0.48978
Q9D8W5	Psmd12	26S proteasome non-ATPase regulatory subunit 12	115476.6	121869.3	121163.6	108021.6	116633	120458.6	115734.8	118549.8	118989.9	118433	0.9848	0.61090
O08600	Endog	Endonuclease G, mitochondrial	14356.7	14676.66	17465.49	14846.22	15336	13598.89	11843.62	15685.28	17412.2	14635	1.0479	0.63658
Q8VEG4	Exd2	Exonuclease 3'-5' domain-containing protein 2	14674.49	14224.33	14258.52	10236.63	13348		14139.6	10994.44	9783.255	11639	1.1469	0.34593
Q9D0K0	Tbc1d7	TBC1 domain family member 7				2293.758	2294		6054.81	2752.173		4403	0.5209	
Q9D8L5	Ccdc91	Coiled-coil domain-containing protein 91	20624.74	10892.78	18438	14334.03	16072	15213.07	13229.79	8988.352	10474.74	11976	1.3420	0.16242
Q9D0J8	Ptms	Parathymosin	295933.6	351087.1	411199.5	355841.9	353516	442380.5	336150.3	571533.7	396100.3	436541	0.8098	0.18357
Q9JHR7	Ide	Insulin-degrading enzyme	64045.04	73003.61	94356.67	43102.92	68627	64713.37	68206.35	70828.34	80759.84	71127	0.9649	0.83028
P52480	Pkm	Pyruvate kinase PKM	3106195	3162758	2631076	2651867	2887974	2889555	2988108	2221425	2291234	2597581	1.1118	0.28002
Q9ERI6	Rdh14	Retinol dehydrogenase 14	43428.18	39831.99	41317.08	47977.97	43139	41714.95	39514.97	43284.71	50930.57	43861	0.9835	0.82057
Q56A07	Scn2b	Sodium channel subunit beta-2												
P39098	Man1a2	Mannosyl-oligosaccharide 1,2-alpha-mannosidase I	31296.5	32011.65	33272.71	30903.34	31871	27268.9	35548.63	32030.68	34354.59	32301	0.9867	0.82878
Q9WU63	Hebp2	Heme-binding protein 2	52750.69	48454.16	31001.53	31136.7	40836	44931	43657.79	30350.34	27698.41	36659	1.1139	0.58481
Q9D071	Mms19	MMS19 nucleotide excision repair protein homolog	21131.42	22224.45	19391.18	18984.2	20433	19509.61	25317.77	18436.47	16170.74	19859	1.0289	0.79277
P42232	Stat5b	Signal transducer and activator of transcription 5B	22620.21	26991.93	24834.87	18117.67	23141	22923.55	26645.84	22540.23	21758.55	23467	0.9861	0.88642
Q8K0C4	Cyp51a1	Lanosterol 14-alpha demethylase	139655.1	127409.3	195037	163453.6	156389	161729.7	160232.3	214953.7	237041	193489	0.8083	0.17897
Q9D8T7	Slirp	SRA stem-loop-interacting RNA-binding protein, mitc	14844.38	11101.4	10389.04	15047.99	12846	21343.67	11682.13	14039.94	18037.4	16276	0.7893	0.21318
P00158	Mt-Cyb	Cytochrome b	12400.28	14695.46	17831.77	14742.05	14917	13289.91	16115.62	11795.93	14153.59	13839	1.0779	0.48032
Q8VDH1	Fbxo21	F-box only protein 21	6559.658	5136.466	6825.952	3902.528	5606	6327.923	7260.702	6317.163	6101.71	6502	0.8622	0.26326
Q9EST5	Anp32b	Acidic leucine-rich nuclear phosphoprotein 32 family	130625.5	118763.3	144543.3	118681.9	128153	126747.7	124923.9	147728.8	120823.8	130056	0.9854	0.83225
Q9DC23	Dnajc10	DnaJ homolog subfamily C member 10	22818.45	27862.2	28349.29	27620.17	26663	26230.44	27512.31	26269.37	26785.33	26699	0.9986	0.97871
P42227	Stat3	Signal transducer and activator of transcription 3	99053.38	99572.45	91667.32	84771.1	93766	92729.98	95349.29	83935.32	82372.59	88597	1.0583	0.31774
P00397	Mtco1	Cytochrome c oxidase subunit 1	40088.97	42856.11	40585.91	44482.51	42003	32958.86	51186.41	33769.5	45436.64	40838	1.0285	0.80803
P62983	Rps27a	Ubiquitin-ribosomal protein eS31 fusion protein	2242458	2694029	2464694	2348045	2437306	2594436	2129454	2539986	2553352	2454307	0.9931	0.91095
Q9CR98	Fam136a	Protein FAM136A	43938.95	44794.15	50616.21	50231.61	47395	46428.55	45346.61	50562.31	53074.59	48853	0.9702	0.58360
Q570Y9	Deptor	DEP domain-containing mTOR-interacting protein	42029.77	41112.91	38651.97	39944.77	40435	40568.94	40059.23	41887.46	38376.47	40223	1.0053	0.84397
P24529	Th	Tyrosine 3-monooxygenase	18038.34	14445.89	15798.59	11174.44	14864	14084.25	10593.05	15034.05	14124.79	13459	1.1044	0.44974
Q8BNU0	Armc6	Armadillo repeat-containing protein 6	22575.04	27891.05	26616.94	18781.19	23966	25669.84	23401.36	21732.93	20670.88	22869	1.0480	0.65517
P16546	Sptan1	Spectrin alpha chain, non-erythrocytic 1	915336.8	884303	809359.3	967077.7	894019	882182.5	895524.2	805226.9	824268.1	851800	1.0496	0.32735
Q9D1B9	Mrpl28	Large ribosomal subunit protein bL28m	11397.96	13542.55	16144.15	15077.37	14041	14572.92	15412.35	16172.8	18416.73	16144	0.8697	0.16213
Q99P88	Nup155	Nuclear pore complex protein Nup155	15218.09	14360.27	14169.07	16643.58	15098	14226.67	15459.75	13691.97	15739.56	14779	1.0215	0.68464
Q8BZJ7	Dcun1d2	DCN1-like protein 2	17382.02	18689.77	11126.15	15156.62	15589	23397.31	17731.77	8661.978	9459.636	14813	1.0524	0.84850
Q8BL97	Srsf7	Serine/arginine-rich splicing factor 7	122379.2	128832	136043.7	148966.7	134055	128192.9	128430.7	162016.3	146708.5	141337	0.9485	0.49146
Q61191	Hcfc1	Host cell factor 1	28921.55	28602.04	31706.91	30976.39	30052	27959.08	28468.33	31330.83	29481.8	29310	1.0253	0.51236
Q8CCN5	Bcas3	BCAS3 microtubule associated cell migration factor	11742.49	12296.02	11776.34	11534.65	11837	11467.24	10556.38	10291.66	11058.79	10844	1.0917	0.01794
Q8C7M3	Trim9	E3 ubiquitin-protein ligase TRIM9	16021.91	15549.58	15136.57	15187.42	15474	18906.21	17627.08	13146.69	17075.24	16689	0.9272	0.37151
Q8C025	Chpt1	Cholinephosphotransferase 1	47145.7	45743.73	37555.47	54180.18	46156	46113.86	47227.97	45603.85	45786.33	46183	0.9994	0.99403
Q9R1Q9	Atp6ap1	V-type proton ATPase subunit S1	42026.47	43085.77	40086.19	63648.54	47212	45226.2	53822.84	49984.71	26115.46	43787	1.0782	0.69279
A6H611	Mipep	Mitochondrial intermediate peptidase	5778.774	6165.645	7665.319	7559.828	6792	5787.021	6692.127	4169.164	5076.097	5431	1.2506	0.10723
P18826	Phka1	Phosphorylase b kinase regulatory subunit alpha, sk	13723.25	3741.073		10436.36	9300		7725.733	10664.23	10135.06	9508	0.9781	0.94926
P57776	Eef1d	Elongation factor 1-delta	696903.9	751398.4	787617.4	765194.7	750279	714274.5	759303.9	904721.1	912971.4	822818	0.9118	0.22848
Q9Z0M3	Cdh20	Cadherin-20												
Q8QZZ7	Tprkb	EKC/KEOPS complex subunit Tprkb	6787.365		8357.401	8041.242	7729	6144.13			5675.705	5910	1.3077	0.06629
O88447	Klc1	Kinesin light chain 1	110781.1	112249.6	105659.1	99587.27	107069	113307.4	108619.4	105606.5	101663.8	107299	0.9979	0.95340
Q80U23	Snph	Syntaphilin	14620.6	14063.58	9794.318	10836.9	12329	12378.8	12598.09	9320.114	12712.99	11752	1.0490	0.70269

Q9CR20	Ier3ip1	Immediate early response 3-interacting protein 1	57796.33	56484.52	55861.59	56514.17	**56664**	48270.86	60729.73	53059.86	51597.15	**53414**	1.0608	0.26883
P18242	Ctsd	Cathepsin D	1045539	994648.9	947282.1	974974.1	**990611**	1033711	984541.9	952831.4	1037207	**1002073**	0.9886	0.70676
Q9Z306	Slc22a4	Solute carrier family 22 member 4												
P26369	U2af2	Splicing factor U2AF 65 kDa subunit	78521.45	81598.37	91445	86831.01	**84599**	86484.84	91454.11	103763.2	100458.7	**95540**	0.8855	0.06706
Q9CWE0	Mtfr1l	Mitochondrial fission regulator 1-like	16766.57	18056.4	18391.49	20418.57	**18408**	15928.73	19060.08	20950.56	24624.44	**20141**	0.9140	0.41275
Q64339	Isg15	Ubiquitin-like protein ISG15	61222.99	42412.28	46634.92	41030.98	**47825**	47431.53	35267.03	51064.44	34804.95	**42142**	1.1349	0.39648
Q7TNG8	Ldhd	Probable D-lactate dehydrogenase, mitochondrial	8524.07	9482.058	11853.58	10834.91	**10174**	9589.325	10293.97	8071.841	11116.71	**9768**	1.0415	0.69252
P62242	Rps8	Small ribosomal subunit protein eS8	510159.2	523539.8	591336.1	572053.9	**549272**	551592.9	595555.9	659855.8	645225.6	**613058**	0.8960	0.08805
P61211	Arl1	ADP-ribosylation factor-like protein 1	166366.5	184471.2	180727.2	147915.2	**169870**	164789.5	178494.8	187025.3	183677.5	**178497**	0.9517	0.40485
P83741	Wnk1	Serine/threonine-protein kinase WNK1	28054.7	22825.21	25690.67	29920.22	**26623**	22536.9	26607.5	29174.4	25271.45	**25898**	1.0280	0.73740
P01864		Ig gamma-2A chain C region secreted form	536094.9	305153.2	51074.72	84768.24	**244273**	257428.5	197699.1	25948.09	23355.85	**126108**	1.9370	0.38925
Q8C0E2	Vps26b	Vacuolar protein sorting-associated protein 26B	82369.3	80357.89	84303.38	69828.02	**79215**	75988.05	82436.45	71398.9	83139.98	**78241**	1.0124	0.82716
P54818	Galc	Galactocerebrosidase	42052.04	37717.23	40052.2	32656.13	**38119**	36310.76	39962.34	36697.5	35974.28	**37236**	1.0237	0.70509
Q8BRF7	Scfd1	Sec1 family domain-containing protein 1	55833.65	56601.02	59487.9	56804.38	**57182**	56049.54	57253.76	63058.84	62044.18	**59602**	0.9594	0.25158
P10630	Eif4a2	Eukaryotic initiation factor 4A-II	176019.6	180700.8	184550.4	152306	**173394**	172480.3	166345.1	176175.3	167818.9	**170705**	1.0158	0.73496
P99024	Tubb5	Tubulin beta-5 chain	2819192	3209772	2994828	2765427	**2947305**	2914321	2984969	2888824	3004486	**2948150**	0.9997	0.99378
P46638	Rab11b	Ras-related protein Rab-11B	394190.4	372067.2	372334.3	403749.1	**385585**	378091.1	379928.5	442826.6	418086.5	**404733**	0.9527	0.31836
P43406	Itgav	Integrin alpha-V	71973.97	69932.35	93246.2	81423.3	**79144**	78501.85	73939.87	88521.17	91551.13	**83129**	0.9521	0.57634
Q60996	Ppp2r5c	Serine/threonine-protein phosphatase 2A 56 kDa reg	62329.3	58830.71	60027.55	47161.61	**57087**	53930.2	71856.63	35921.96	60418.35	**55532**	1.0280	0.85653
P63216	Gng3	Guanine nucleotide-binding protein G(I)/G(S)/G(O) s	21551.49	25871.49	21172.7	27326.39	**23981**	22424.45	27963.51	22015.09	21800.16	**23551**	1.0182	0.84716
Q3U5Q7	Cmpk2	UMP-CMP kinase 2, mitochondrial	56177.06	43236.31	38404.07	43852.9	**45418**	45920.16	46466.09	40136.23	40824.54	**43337**	1.0480	0.63275
P24457	Cyp2d11	Cytochrome P450 2D11	34794.36	33305.15	23250.67	38612.01	**32491**	26653	37050.02	25172.36	21708.79	**27646**	1.1752	0.33776
Q99NB8	Ubqln4	Ubiquilin-4	25751.75	28056.64	27637.87	24084.21	**26383**	25620.76	23413.85	20933.05	26602.7	**24143**	1.0928	0.20054
Q9DCZ4	Apoo	MICOS complex subunit Mic26	139169.6	146741.3	134147.1	154646.1	**143676**	129664.6	158564.6	134767.3	124816.9	**136953**	1.0491	0.47014
Q9CY18	Snx7	Sorting nexin-7	13257.01	11149.99	15958.04	10112.06	**12619**	11559.24	9236.939	9976.804	14064.77	**11209**	1.1258	0.43233
P02469	Lamb1	Laminin subunit beta-1	398614.3	398920.3	454569.8	488318.1	**435106**	390606.7	366707.3	475225.1	550942.3	**445870**	0.9759	0.82824
Q8VBW6	Nae1	NEDD8-activating enzyme E1 regulatory subunit	59788.87	64210.46	57332.4	55542.37	**59219**	61483.2	62281.89	49189.05	54712.57	**56917**	1.0404	0.54744
Q3UFF7	Lyplal1	Lysophospholipase-like protein 1	24219.12	21079.97	20090.55	17898.47	**20822**	18486.7	17136.32	12012.54	13515.89	**15288**	1.3620	0.03280
Q80XU3	Nucks1	Nuclear ubiquitous casein and cyclin-dependent kin	11359.09	9074.552	13350.83	8280.509	**10516**	6353.959	8507.306	9687.712	11507.39	**9014**	1.1666	0.37747
F8VPZ3	Usp32	Ubiquitin carboxyl-terminal hydrolase 32	3362.625	7434.224	2849.575	2931.987	**4145**	4456.345	4613.919		5525.383	**4865**	0.8519	0.61217
P46460	Nsf	Vesicle-fusing ATPase	399696.5	376082.1	325745.7	390409.9	**372984**	407010.8	376824.6	363673.6	369877.6	**379347**	0.9832	0.75002
P05063	Aldoc	Fructose-bisphosphate aldolase C	286542.8	277974.5	241481.7	249350.8	**263837**	253515.3	266146.6	256826.8	235413.1	**252975**	1.0429	0.42371
Q9CV28	Mindy3	Ubiquitin carboxyl-terminal hydrolase MINDY-3	24480.47	20654.92	13347.58	17815.67	**19075**	16766.27	19211.26	14099.17	16945.41	**16756**	1.1384	0.40154
P20065	Tmsb4x	Thymosin beta-4	139653.8	118187	144271.9	140410.6	**135631**	149988	73606.44	172205.4	151721.1	**136880**	0.9909	0.95747
Q8R4X3	Rbm12	RNA-binding protein 12	21954.15	17369.68	20041.28	13810.75	**18294**	15545.13	16316.85	15000.65	14651.64	**15379**	1.1896	0.15689
Q8VEH3	Arl8a	ADP-ribosylation factor-like protein 8A	84703.99	79520.77	81976.97	86050.18	**83063**	91242.65	95859.27	72465.91	90503.1	**87518**	0.9491	0.43742
P28571	Slc6a9	Sodium- and chloride-dependent glycine transporter 1							9387.356			**9387**	0.0000	
P60766	Cdc42	Cell division control protein 42 homolog	802650.3	808568.8	772485.3	899822.1	**820882**	829366.6	895268.8	757656.9	841556.3	**830962**	0.9879	0.80688
Q4VAA2	Cdv3	Protein CDV3	56159.63	61517.83	71252.52	65228.44	**63540**	64729.72	57384.37	76601.38	77308.81	**69006**	0.9208	0.38077
Q8BHE8	Maip1	m-AAA protease-interacting protein 1, mitochondrial	12462.18	9102.472	12462.8	16076.78	**12526**	11176.03	12511.66	10851.75	11359.86	**11475**	1.0916	0.50117
Q8K0D5	Gfm1	Elongation factor G, mitochondrial	44646.96	47254.55	46899.56	48062.57	**46716**	45277.16	49690.45	46923.94	49724.49	**47904**	0.9752	0.40144
Q91V09	Wdr13	WD repeat-containing protein 13	20444.24	19201.45	24040.36	17640.68	**20332**	19651.82	19909.14	19561.47	18700.85	**19456**	1.0450	0.55123
Q9WTU6	Mapk9	Mitogen-activated protein kinase 9	14121.67	11861.52	12129.07	9738.285	**11963**	12649.07	12950.23	10225.42	7073.604	**10725**	1.1154	0.47630
Q6ZQ58	Larp1	La-related protein 1	127459.9	121010.8	119239.8	146504.6	**128554**	117994.5	133202	134394.8	141529.5	**131780**	0.9755	0.69942
Q9CZB0	Sdhc	Succinate dehydrogenase cytochrome b560 subunit	50163.59	44973.3	51660.08	43094.17	**47473**	41356.57	46687.66	39831.55	56888.4	**46191**	1.0277	0.77895
P22599	Serpina1b	Alpha-1-antitrypsin 1-2	156298.8	90440.48	239179.9	120319	**151560**	156734.5	123023.1	122003.8	132725.3	**133622**	1.1342	0.60801
P17182	Eno1	Alpha-enolase	968363.8	933378.6	984789.3	770780.9	**914328**	993939	840148.1	910338.1	833097.2	**894381**	1.0223	0.75753
Q9CRA9	Fgfr1op2	FGFR1 oncogene partner 2 homolog	27954.78	24468.41	24520.46	26121.11	**25766**	26571.79	28093.88	25482.76	27940.26	**27022**	0.9535	0.26820
Q9CXI0	Coq5	2-methoxy-6-polyprenyl-1,4-benzoquinol methylase	7860.289	10562.61	9650.772	7354.497	**8857**	10662.52	10859.16	8500.55	8678.291	**9675**	0.9154	0.43610
P70671	Irf3	Interferon regulatory factor 3	30695.18	27550.88	25930.01	13315.01	**24373**	22289.91	20470.11	6490.302	8201.948	**14363**	1.6969	0.12349
Q11136	Pepd	Xaa-Pro dipeptidase	75886.92	88465.07	80752.44	69152.59	**78564**	79732.43	74305.13	79445.64	74180.29	**76916**	1.0214	0.71785
Q3URS9	Ccdc51	Mitochondrial potassium channel	11018.93	10525.76	10636.85	14662.51	**11711**	13100.85	14791.81	9982.36	11504.25	**12345**	0.9487	0.67349
Q01065	Pde1b	Dual specificity calcium/calmodulin-dependent 3',5'	29539.88	26142.73	30668.96	19854.14	**26551**	22601.36	32386.83	15186.74	32000.2	**25544**	1.0394	0.84035
Q8BPA8	Dpcd	Protein DPCD	12066.14	8872.503	9428.123	6946.708	**9328**	5542.614	11817.9	9096.366	8695.291	**8788**	1.0615	0.75633
O70133	Dhx9	ATP-dependent RNA helicase A	147946.8	146582.9	164779.8	170953.5	**157566**	155269.4	154240.8	177964.7	181117.5	**167148**	0.9427	0.34817
Q03719	Kcnd1	Potassium voltage-gated channel subfamily D memb	8314.389	4670.947	8057.418	4901.166	**6486**	14012.07	8029.109	9510.058	11079.16	**10658**	0.6086	0.04154
Q924K8	Mta3	Metastasis-associated protein MTA3	23166.88	21841.12	26991.04	22813.14	**23703**	25139.39	20851.26	21429.2	22334.92	**22439**	1.0563	0.42503
P08122	Col4a2	Collagen alpha-2(IV) chain	2067127	1772452	1207273	2329578	**1844107**	1785873	1988294	1581022	1653440	**1752157**	1.0525	0.73267
P55065	Pltp	Phospholipid transfer protein	28547.37	24843.85	20604.38	17381.3	**22844**	29085.14	23809.12	21597.05	18239.72	**23183**	0.9854	0.92248

Q9CZT8	Rab3b	Ras-related protein Rab-3B	9885.352	8541.201	5882.987	8272.7	**8146**	9184.922	8317.234	4435.217	13415.36	**8838**	0.9216	0.74359
Q9DCL9	Paics	Bifunctional phosphoribosylaminoimidazole carboxy	165816	169762.6	167016.2	159529.8	**165531**	154936.3	156491	146246.7	156011.3	**153421**	1.0789	0.00967
Q8CES0	Naa30	N-alpha-acetyltransferase 30	70526.38	67104.47	54551.7	70492.73	**65669**	62522.8	73004.84	52258.37	65575.92	**63340**	1.0368	0.69873
Q9CQS4	Slc25a46	Mitochondrial outer membrane protein SLC25A46	17448.34	18503.99	19113.04	23505.38	**19643**	22221.92	25179.29	18986.71	18919.61	**21327**	0.9210	0.43304
Q61239	Fnta	Protein farnesyltransferase/geranylgeranyltransfera	31320.38	33908.54	33997.37	28517.55	**31936**	32213.15	29015.69	30790.83	26533.07	**29638**	1.0775	0.24520
Q9D020	Nt5c3a	Cytosolic 5'-nucleotidase 3A	9786.226	9365.017	9684.597	11625.91	**10115**	8972.802	10890.29	7964.583	9653.841	**9370**	1.0795	0.38715
P83887	Tubg1	Tubulin gamma-1 chain	54028.9	54775.16	65124.13	51381.48	**56327**	60163.47	52454.21	47879.29	64983.16	**56370**	0.9992	0.99331
Q9JHL1	Nherf2	Na(+)/H(+) exchange regulatory cofactor NHE-RF2	52085.34	47725.7	46575.16	53004.82	**49848**	53963	44042.9	57183.51	52881.91	**52018**	0.9583	0.52638
Q8CI78	Rmnd1	Required for meiotic nuclear division protein 1 homolog												
Q0VBD0	Itgb8	Integrin beta-8	17310.98	15090.2	15715.69	19998.01	**17029**	15113.12	16195.97	15007.3	22394.59	**17178**	0.9913	0.94501
P63037	Dnaja1	DnaJ homolog subfamily A member 1	75051.96	82873.84	81901.76	81885.61	**80428**	80597.12	79470.82	84059.64	81063.95	**81298**	0.9893	0.68697
Q9JKN6	Nova1	RNA-binding protein Nova-1		3710.378		7592.198	**5651**	6374.93		6081.348	6958.288	**6472**	0.8733	0.61907
Q3UVG3	Fam91a1	Protein FAM91A1	16230.04	16817.09	14182.68	14293.75	**15381**	16392.38	16114.88	16042.27	16113.6	**16166**	0.9514	0.28929
P48193	Epb41	Protein 4.1	35224.12	31486.25	27385.19	42099.55	**34049**	30471.17	37610.82	26010.15	28191.42	**30571**	1.1138	0.41938
Q9DC51	Gnai3	Guanine nucleotide-binding protein G(i) subunit alph	126419.8	122241	123362	161584.1	**133402**	132596.2	126641.1	110705.6	134757.9	**126175**	1.0573	0.53156
P23116	Eif3a	Eukaryotic translation initiation factor 3 subunit A	94328.54	96578.8	99985.13	110972.9	**100466**	95358.9	101563.3	115728.8	113344.7	**106499**	0.9434	0.35958
Q8R059	Gale	UDP-glucose 4-epimerase	48003.5	45910.72	37640.49	32746.44	**41075**	41985.37	43770.22	35548.73	38546.86	**39963**	1.0278	0.79058
P55772	Entpd1	Ectonucleoside triphosphate diphosphohydrolase 1	22958.57	24852.01	27968.44	25693.21	**25368**	23255.37	21667.06	25305.24	25373.61	**23900**	1.0614	0.32486
Q61578	Fdxr	NADPH:adrenodoxin oxidoreductase, mitochondrial	26954.64	24460.16	26206.88	28396.74	**26505**	25989.55	22602.22	25345.86	27238.37	**25294**	1.0479	0.37967
Q99N92	Mrpl27	Large ribosomal subunit protein bL27m	13407.58	11204.98	12395.67	13223.76	**12558**	12679.71	21824.53	10768.83	15596.87	**15217**	0.8252	0.32249
P50427	Sts	Steryl-sulfatase	43385.04	40013.23	43897.37	48553.06	**43962**	38058.95	37594.74	46555.21	54266.09	**44119**	0.9965	0.97234
Q99JR1	Sfxn1	Sideroflexin-1	36654.74	42184.13	45497.22	45512.43	**42462**	40050.68	44009.01	39978.86	39048.04	**40772**	1.0415	0.50098
Q9JLM8	Dclk1	Serine/threonine-protein kinase DCLK1	100828	103683.6	97604.87	102429.9	**101137**	103090.7	110259.6	97910.16	95057.9	**101580**	0.9956	0.90573
Q8VE11	Mtmr6	Myotubularin-related protein 6	34811.8	34033	33389.13	27969.48	**32551**	30463.19	30055.42	29038.15	28922.05	**29620**	1.0990	0.11670
Q3TKT4	Smarca4	Transcription activator BRG1	18163.47	18908.29	17906.79	19077.21	**18514**	17259.31	19158.03	17409.63	16680.75	**17627**	1.0503	0.19270
Q8CGC6	Rbm28	RNA-binding protein 28	16264.44				**16264**				9814.054	**9814**	1.6573	
Q80X95	Rraga	Ras-related GTP-binding protein A	13569.52	13921.94	9410.092	10820.93	**11931**	9980.507	13269.97	10101.93	10175.2	**10882**	1.0964	0.46666
Q9CXI5	Manf	Mesencephalic astrocyte-derived neurotrophic facto	73619.28	77993.78	79717.45	77146.41	**77119**	78274.76	77711.34	77209.53	73872.23	**76767**	1.0046	0.83510
P35550	Fbl	rRNA 2'-O-methyltransferase fibrillarin	61849.38	62066.18	61771.79	66835.53	**63131**	62314.48	63651.08	68290.39	66004.34	**65065**	0.9703	0.32565
O88668	Creg1	Protein CREG1	23728.12	18146.99	15548.12	18626.32	**19012**	15712.95	19816.55	11550.39	14344.74	**15356**	1.2381	0.18261
Q99KJ8	Dctn2	Dynactin subunit 2	264885.1	260747.1	259643	251630.6	**259226**	259565.6	281914.3	283592.8	275893.5	**275242**	0.9418	0.04025
Q8BRJ3	Rell2	RELT-like protein 2												
P43274	H1-4	Histone H1.4	2827172	2505344	2654198	3269862	**2814144**	2395895	2607538	3671973	2755489	**2857724**	0.9848	0.89814
Q69ZS7	Hbs1l	HBS1-like protein	17376.95	18203.52	18492.9	16294.69	**17592**	17366.34	17695.46	17183.55	18337.11	**17646**	0.9970	0.92612
P62342	Selenot	Thioredoxin reductase-like selenoprotein T	33659.61	34548.74	32438.29	39606.95	**35063**	35341.48	35505.16	44740.73	41838.08	**39356**	0.8909	0.17961
Q8BGT8	Phyhipl	Phytanoyl-CoA hydroxylase-interacting protein-like	66293.82	61278.61	49966.71	43274.61	**55203**	57297.16	60056.02	49586.38	38242.94	**51296**	1.0762	0.60504
Q8K3A0	Hscb	Iron-sulfur cluster co-chaperone protein HscB	10150.67	7627.53	9028.845	8364.905	**8793**	7895.312	7730.956	6807.699	5720.27	**7039**	1.2493	0.05374
Q61550	Rad21	Double-strand-break repair protein rad21 homolog	39324.61	35692.28	48908.04	53470.48	**44349**	48932	34035.78	55231.13	44977.27	**45794**	0.9684	0.81971
Q9Z0P5	Twf2	Twinfilin-2	50585.87	46310.82	42921.52	36081.82	**43975**	41912.3	41584.69	36369.01	35107.85	**38743**	1.1350	0.18886
Q8VE88	Fam114a2	Protein FAM114A2	11436.89	13186.44	12351.83	10146.22	**11780**	9763.482	10515.01	8501.95	8409.429	**9297**	1.2670	0.02397
P35505	Fah	Fumarylacetoacetase	58057.8	60938	56595.9	45821.77	**55353**	55864.11	53979.53	40561.34	43741.9	**48537**	1.1404	0.22226
Q69Z98	Brsk2	Serine/threonine-protein kinase BRSK2	8081.281	12022.66	7300.007	10846.89	**9563**	10575.51	8411.39	11604.71	8850.174	**9860**	0.9698	0.83207
Q91V12	Acot7	Cytosolic acyl coenzyme A thioester hydrolase	1195265	1346680	1236061	1089836	**1216960**	1317982	1175450	1201672	1102984	**1199522**	1.0145	0.80993
Q62203	Sf3a2	Splicing factor 3A subunit 2	27722.14	21221.85	17093.27	26297.77	**23084**	22426.85	24430.32	24604	23336.21	**23699**	0.9740	0.81285
Q8BTF8	Ralyl	RNA-binding Raly-like protein	9066.038	4993.446	13905.8	11018.22	**9746**	15486.09	9331.151	9495.999	10179.53	**11123**	0.8762	0.58326
P10639	Txn	Thioredoxin	442961.8	494611.5	362457.5	316065.2	**404024**	482975.7	452212.3	413472.9	382228.6	**432722**	0.9337	0.55277
P62073	Timm10	Mitochondrial import inner membrane translocase s	9543.811	10566.2	11364.65	12753.12	**11057**	8537.913	9825.824	8994.702	11013.31	**9593**	1.1526	0.14271
P57716	Ncstn	Nicastrin	32759.01	33881.97	37101.3	30969.28	**33678**	30402.09	34648.48	32506.16	32582.25	**32535**	1.0351	0.48958
Q91ZJ5	Ugp2	UTP--glucose-1-phosphate uridylyltransferase	149266.2	163798.8	141114.1	129632.6	**145953**	156289.7	150127.1	143208.4	130703.1	**145082**	1.0060	0.92639
O09117	Sypl1	Synaptophysin-like protein 1	42781.39	60959.69	36943.35	61280.27	**50491**	46800.71	58007.55	41580.92	44493.7	**47721**	1.0581	0.71403
P41216	Acsl1	Long-chain-fatty-acid--CoA ligase 1	76111.23	72203.89	77794.54	78730.03	**76210**	67757.37	76157.41	69424.43	76571.12	**72478**	1.0515	0.21460
O35166	Gosr2	Golgi SNAP receptor complex member 2	23907.14	20868.5	26757.91	24086.82	**23905**	24678.71	24632.95	26639.82	29578.35	**26382**	0.9061	0.18944
Q9WVE8	Pacsin2	Protein kinase C and casein kinase substrate in neur	78928.05	77263.21	73149.31	73276.94	**75654**	75697.94	76964.98	71770.98	73686.64	**74530**	1.0151	0.56465
P60122	Ruvbl1	RuvB-like 1	91197.88	87295.53	91468.86	87588.55	**89388**	91600.99	88596.59	94661.41	98708.07	**93392**	0.9571	0.15156
O54931	Akap2	A-kinase anchor protein 2	23658.35	19939.57	23369.35	22777.36	**22436**	22771.04	21547.05	26364.42	23561.81	**23561**	0.9523	0.43037
Q922Y1	Ubxn1	UBX domain-containing protein 1	46910.53	40791.07	46668.47	41801.52	**44043**	45608.76	40523.33	42361.21	42177.74	**42668**	1.0322	0.50105
P35438	Grin1	Glutamate receptor ionotropic, NMDA 1	87995.09	93826.74		93934.99	**91919**	56326.6				**56327**	1.6319	
Q9DCA2	Mrps11	Small ribosomal subunit protein uS11m	4844.732	3781.08	5759.845	4980.924	**4842**	5730.897	4662.469	3764.804	6890.755	**5262**	0.9201	0.61293
Q99LI2	Clcc1	Chloride channel CLIC-like protein 1	12126.7	10626.4	10366.57	10665.36	**10946**	9959.133	12244.38	8002.867	11987.35	**10548**	1.0377	0.72230

Q8R420	Abca3	Phospholipid-transporting ATPase ABCA3	8763.732	8873.769	9435.415	9366.064	9110	10890.09	10346.23	11279.17	10777.92	10823	0.8417	0.00054
Q9R0P3	Esd	S-formylglutathione hydrolase	172677.1	193227.7	154808.6	198643.6	179839	198840.6	144326.5	154794.1	188549.6	171628	1.0478	0.63639
Q9D289	Trappc6b	Trafficking protein particle complex subunit 6B	21554.63	18312.06	19653.13	20895.19	20104	18856.47	20921.72	17046.17	19551.29	19094	1.0529	0.38502
P26231	Ctnna1	Catenin alpha-1	80564	80532.14	80124.07	85431.66	81663	83546.02	85961.19	86167.52	83963.54	84910	0.9618	0.06348
P70175	Dlg3	Disks large homolog 3	12494.88	9644.493	7296.174	9176.699	9653	9363.136	11637.67	5712.264	6469.838	8296	1.1636	0.46407
Q8BP67	Rpl24	Large ribosomal subunit protein eL24	686602.6	676764.2	777571.7	743348.1	721072	684639.8	674714.4	726317.4	815579.1	725313	0.9942	0.91902
Q9DBX6	Cyp2s1	Cytochrome P450 2S1	18011.01	17720.36	19210.44	14660.81	17401	15996.82	14400.86	10698.42	12530.82	13407	1.2979	0.03753
Q922B2	Dars1	Aspartate--tRNA ligase, cytoplasmic	548311.4	566833.4	546425.3	545426.6	551749	566782	593746.8	604217.1	583831.1	587144	0.9397	0.00950
Q91YN9	Bag2	BAG family molecular chaperone regulator 2	18527.41	35153.89	34408.59	34472.13	30641	30308.57	28436.36	30312.56	32905.88	30491	1.0049	0.97236
P60229	Eif3e	Eukaryotic translation initiation factor 3 subunit E	72222.53	76361.07	75877.68	71998.07	74115	70672.68	79808.82	77319.02	81473.35	77318	0.9586	0.27108
Q99J36	Thumpd1	THUMP domain-containing protein 1	2934.267	4715.936	5623.175	3650.499	4231	5150.139	4394.87	5926.216	5387.062	5215	0.8114	0.19308
Q61140	Bcar1	Breast cancer anti-estrogen resistance protein 1			14767.86	19640.96	17204							
P55264	Adk	Adenosine kinase	153397.2	167327.7	144267.6	131007.4	149000	152453.5	152877.7	135835.6	126989.5	142039	1.0490	0.51111
Q91WG4	Elp2	Elongator complex protein 2	18616.05	18274.25	17306.89	14958.19	17289	10573.74	16836.21	18089.54	16866.83	15592	1.1089	0.40322
P46097	Syt2	Synaptotagmin-2	28393.64	40708.8	41947.3	37214.75	37066	35791.37	39686.75	33420.48	34593.89	35873	1.0333	0.73381
O70258	Sgce	Epsilon-sarcoglycan	23100.7	21776.17	16648.81	24083.01	21402	22048.77	20183.02	22545.41	18743.9	20880	1.0250	0.78962
E9Q7X7	Nrxn2	Neurexin-2		54991.48	19149.55	65339.79	46494				8353.238	8353	5.5659	
Q3TVA9	Ccdc136	Coiled-coil domain-containing protein 136	36653.54	33193.77		38586.04	36144		34162.1	30481.85		32322	1.1183	0.21735
Q6ZPU9	Kifbp	KIF-binding protein	14901.43	14067.76	14626.14	10006.55	13400	13895.6	12775.68	11683.64	11824.61	12545	1.0682	0.52038
Q9DBC7	Prkar1a	cAMP-dependent protein kinase type I-alpha regulat	111374.6	103623.6	122663.4	117135	113699	114700.8	109020.2	122439.1	111186.6	114337	0.9944	0.90320
O08583	Alyref	THO complex subunit 4	40487.16	39326.86	44265.73	46020.57	42525	37674.58	42590.61	52269.59	46472.51	44752	0.9502	0.54394
Q8QZS1	Hibch	3-hydroxyisobutyryl-CoA hydrolase, mitochondrial	91677.38	92946.58	89945.61	102918.6	94372	94160.8	94070.34	83918.5	93287.1	91359	1.0330	0.46164
P56371	Rab4a	Ras-related protein Rab-4A	40428.49	40632.24	40774.63	39959.91	40449	45179.63	38287.63	48388.46	47005.12	44715	0.9046	0.10649
Q61315	Apc	Adenomatous polyposis coli protein			13238.4		13238							
P97807	Fh	Fumarate hydratase, mitochondrial	251932.5	239857.4	258113.7	269211.3	254779	252152	248978.7	237053.1	243906.9	245523	1.0377	0.23156
Q64133	Maoa	Amine oxidase [flavin-containing] A	251192.7	229233.2	225029.7	267275.1	243183	237679	245416	286977.9	262083.4	258039	0.9424	0.35148
Q8CBY8	Dctn4	Dynactin subunit 4	80539.84	75286.4	83066.83	83777	80668	80756.14	79907.41	77497.86	81779.23	79985	1.0085	0.75948
O35393	Efnb3	Ephrin-B3						853187.1				853187	0.0000	
O88696	Clpp	ATP-dependent Clp protease proteolytic subunit, mit	43311.13	40067.16	38671.2	44089.98	41535	38910.73	43981.84	32596.63	38520.77	38502	1.0788	0.29826
Q99K46	Usp11	Ubiquitin carboxyl-terminal hydrolase 11	3298.556				3299	24877.39			24257.65	24568	0.1343	
P46467	Vps4b	Vacuolar protein sorting-associated protein 4B	65154.35	61394.14	58122.09	61156.62	61457	51705.49	52392.09	48712.56	52220.82	51258	1.1990	0.00090
Q9CQC6	Bzw1	eIF5-mimic protein 2	78276.77	76929.62	80281.2	67963.31	75863	73794.55	82274.09	73105.98	78138.69	76828	0.9874	0.78932
Q3UV17	Krt76	Keratin, type II cytoskeletal 2 oral	161497.7	140054.8	213394.6	203377.7	179581	248254.6	150884.8	319084	437534.8	288940	0.6215	0.13218
Q80X82	Sympk	Symplekin	10068.92	8125.096	11351.5	9251.776	9699	11276.11	10466.51	11193.66	10524.4	10865	0.8927	0.15303
P84086	Cplx2	Complexin-2	50828.07	48680.78	41523.68	39608.29	45160	47829.45	40128.08	50641.45	32006.64	42651	1.0588	0.63310
Q9ERB0	Snap29	Synaptosomal-associated protein 29	21678.63	19955.55	28057.35	22784.07	23119	20700.36	30517.03	17099.23	19921.01	22059	1.0480	0.76621
P17095	Hmga1	High mobility group protein HMG-I/HMG-Y	34879.29	33729.18	29810.53	37841.23	34065	26656.06	40584.34	24626.31	26910.34	29694	1.1472	0.31919
O88533	Ddc	Aromatic-L-amino-acid decarboxylase	9477.909	7587.72	6450.628	7614.213	7783	7251.469	7591.755	6836.369	8734.299	7603	1.0236	0.81858
Q9DAM5	Slc25a19	Mitochondrial thiamine pyrophosphate carrier	10548.59	11456.23	11393.02	9119.589	10629	13304.07	9822.503	8278.896	9542.695	10237	1.0383	0.75596
Q9DCL8	Ppp1r2	Protein phosphatase inhibitor 2	72640.16	65478.2	59489.9	51919.89	62382	66801.7	54580.79	69467.48	58465.45	62329	1.0009	0.99275
Q8BH79	Ano10	Anoctamin-10	38869.06	44151.5	41751.57	49376.41	43537	42600.84	42085.79	42043.1	44453.49	42796	1.0173	0.75784
Q08879	Fbln1	Fibulin-1	33638.38	31586.1	41100.2	22927.73	32313	41043.48	31043.49	27679.13	25636.46	31351	1.0307	0.85554
Q9Z1Q9	Vars1	Valine--tRNA ligase	171839	169625	175222.7	180615.9	174326	182071.5	187826.2	195328.4	187762.6	188247	0.9260	0.00851
Q3TZZ7	Esyt2	Extended synaptotagmin-2	134133.6	129596.1	140366.4	155568	139916	138025.6	137806.7	141025	147168.7	141007	0.9923	0.86335
Q6ZQ88	Kdm1a	Lysine-specific histone demethylase 1A	14110.37	13948.7	16539.09	12407.54	14251	13799.97	13431.41	12615.61	17158.39	14251	1.0000	0.99995
Q9WTK7	Stk11	Serine/threonine-protein kinase STK11		10271.44	8989.39		9630							
Q9D0S9	Hint2	Adenosine 5'-monophosphoramidase HINT2	95980.31	83897.02	81589.15	111296.7	93191	90256.09	93042.4	102475.6	95223.79	95249	0.9784	0.78727
O35681	Syt3	Synaptotagmin-3		38084.57		25922.68	32004							
P68037	Ube2l3	Ubiquitin-conjugating enzyme E2 L3	49849.66	46694.4	43399.21	34002.21	43486	41467.68	41168.24	34976.52	35286.74	38225	1.1376	0.22213
Q9WV55	Vapa	Vesicle-associated membrane protein-associated pr	311437.4	297957.3	333899.3	374265.7	329390	324983.7	311409	366815.8	369154.1	343091	0.9601	0.55994
Q8BVQ5	Ppme1	Protein phosphatase methylesterase 1	64176.27	80483.07	67388.99	60383.81	68108	71192.98	67463.9	63331.75	68088.59	67519	1.0087	0.90351
P49813	Tmod1	Tropomodulin-1	278718.7	253248	215884.9	290845.1	259674	245489.3	265912.1	261861	245101.8	254591	1.0200	0.78043
Q91Z53	Grhpr	Glyoxylate reductase/hydroxypyruvate reductase	97774.6	83433.42	79304.05	70030.23	82636	84000.91	82735.94	56803.36	56296.4	69959	1.1812	0.23743
P37804	Tagln	Transgelin	141852.8	129235.7	115460.9	98120.62	121168	109633.4	117295.5	95395.95	92320.9	103661	1.1689	0.16543
Q9Z1R2	Bag6	Large proline-rich protein BAG6	45158.58	50276.61	46726.68	49887.84	48012	45995	46487.5	46228.47	46036.85	46187	1.0395	0.19286
P45377	Akr1b8	Aldose reductase-related protein 2	44077.55	39441.47	29648.3	32311.82	36370	43981.12	42584.32	34807.41	27823.76	37299	0.9751	0.85845
Q8R349	Cdc16	Cell division cycle protein 16 homolog	19032.66	21280.2	16989.68	19292.3	19149	17940.91	14495.21	18988.35	16551.68	16994	1.1268	0.15078
Q8R0S4	Cacnb4	Voltage-dependent L-type calcium channel subunit beta-4				5490.055	5490	6016.153	9140.793	6135.366	7552.784	7211	0.7613	
Q3UQN2	Fcho2	F-BAR domain only protein 2	10178.63	12358.33	8016.617	6710.167	9316	9170.819	8652.979	2987.198	5473.868	6571	1.4177	0.20004

A2APV2	Fmnl2	Formin-like protein 2	111142.6	108755.8	93409.33	123065	109093	109006.5	118161.3	97261.42	102725.9	106789	1.0216	0.77100
Q9D8W7	Ociad2	OCIA domain-containing protein 2	6877.587	6268.407	4598.881	7877.576	6406	2770.006	6336.015	6414.375	5506.353	5257	1.2186	0.33502
P53994	Rab2a	Ras-related protein Rab-2A	306405.9	281834	296946.8	308706.8	298473	287734.2	288216.5	296455.8	316167.9	297144	1.0045	0.88769
Q61771	Kif3b	Kinesin-like protein KIF3B	12139.8	12801.13	12037.31	10673.51	11913	12656.02	12860.94	9598.889	12251.88	11842	1.0060	0.93831
Q8CIM7	Cyp2d26	Cytochrome P450 2D26	21074.83	23080.1		18512.54	20889	14915.93	25064.5	9285.513		16422	1.2720	0.40492
Q64331	Myo6	Unconventional myosin-VI	23551.8	22398.01	22892.19	25324.89	23542	21684.97	25196.33	22989.69	25070.24	23735	0.9918	0.86163
Q8BHA3	Dtd2	D-aminoacyl-tRNA deacylase 2	27034.49	23629.92	22981.6	25277.64	24731	23053.34	24051.8	25302.8	21367.86	23444	1.0549	0.33585
E9Q7G0	Numa1	Nuclear mitotic apparatus protein 1	31285.71	29231.95	31604.49	33819.18	31485	29216.74	27697.59	31643.37	32582.64	30285	1.0396	0.44212
Q99NE5	Rims1	Regulating synaptic membrane exocytosis protein 1	24296.69	18944.89	28632.58	24229.94	24026	23892.18	19590.85	19280.34	22408.4	21293	1.1284	0.27486
A2A5R2	Arfgef2	Brefeldin A-inhibited guanine nucleotide-exchange p	55145.41	51607.91	52130.39	44328.16	50803	55381.06	52008.47	53304.99	53880.76	53644	0.9470	0.28109
Q9D819	Ppa1	Inorganic pyrophosphatase	115872.8	137442.4	121132.2	101199.6	118912	122586.1	117543.7	109623.9	104625.2	113595	1.0468	0.55397
P39447	Tjp1	Tight junction protein ZO-1	97792.34	103872.6	104360.6	119919.5	106486	99626.79	104501.7	109752.1	105313.1	104798	1.0161	0.75453
O88544	Cops4	COP9 signalosome complex subunit 4	89120.28	89478.77	80501.23	73809.13	83227	85423.77	90787.23	87274.05	78812.85	85574	0.9726	0.62256
Q9CSN1	Snw1	SNW domain-containing protein 1	18097.64	19665.69	20765.26	25360.4	20972	19196.96	20344.27	20755.81	22172.96	20617	1.0172	0.83959
O08756	Hsd17b10	3-hydroxyacyl-CoA dehydrogenase type-2	193850.3	194671.4	204658.2	231856.2	206259	206876.2	213767.8	223707.4	240604.6	221239	0.9323	0.24077
Q9CR64	Tmem167a	Protein kish-A	72967.91	72543.08	78228.15	74126.52	74466	76170.55	81429.77	77628.15	83373.21	79650	0.9349	0.04934
Q9QYY8	Spast	Spastin	33435.22	30139.62	34701.5	27873.45	31537	29350.55	28410.61	26971.92	30009.69	28686	1.0994	0.14213
Q6PGF7	Exoc8	Exocyst complex component 8	25942.41	30432.89	26205.56	28041.62	27656	24202.08	27907.06	26745.86	27829.46	26671	1.0369	0.49332
Q9JKW0	Arl6ip1	ADP-ribosylation factor-like protein 6-interacting pro	15866.71	14650.3	14587.1	18159.91	15816	15669.65	18755.09	17564.64	15828.11	16954	0.9329	0.34638
Q61207	Psap	Prosaposin	138036.5	139780.9	113250.6	124147.9	128804	134722.3	154067.9	142556	134540.2	141472	0.9105	0.15374
P16054	Prkce	Protein kinase C epsilon type	51790.42	48282.21	46974.98	46328.97	48344	45063.24	47077.64	49463.5	45146.9	46688	1.0355	0.34024
P22892	Ap1g1	AP-1 complex subunit gamma-1	87383.11	88123.53	85698.15	85548.42	86688	86374.9	93316.5	88345.88	85043.13	88270	0.9821	0.44184
Q8CGF6	Wdr47	WD repeat-containing protein 47	33666.41	42302.25	32656.75	31278.66	34976	34783.92	30058.68	27281.62	28281.01	30101	1.1619	0.15472
Q3U487	Hectd3	E3 ubiquitin-protein ligase HECTD3	35189.85	39067.94	36854.6	36492.22	36901	37193.27	38091.36	40899.51	38261.55	38611	0.9557	0.18225
Q6NVE8	Wdr44	WD repeat-containing protein 44	30707.81	32002.84	28270.3	28791.33	29943	28474.69	31734.05	28474.83	29560.27	29561	1.0129	0.75221
Q99PW4	Tp53rkb	EKC/KEOPS complex subunit Tp53rkb	17280.5	6524.625	13401.52	5205.839	10603	5308.413	8447.328	3547.264	7675.565	6245	1.6980	0.20574
P46662	Nf2	Merlin	39796.38	40003.67	33179.18	30364.13	35836	35636.34	40605.02	31095.26	7489.132	28706	1.2484	0.39148
Q8R3U1	Plaat3	Phospholipase A and acyltransferase 3	14113.11	18220.41	15944.65	16923.22	16300	16926.88	18519.5	20065.44	16265.26	17944	0.9084	0.22428
P97822	Anp32e	Acidic leucine-rich nuclear phosphoprotein 32 family	46004.27	44104.64	42627.2	36984.88	42430	38359.21	44181.36	40847.36	32587.01	38994	1.0881	0.31339
Q9CZV8	Fbxl20	F-box/LRR-repeat protein 20	19032.96	18384.69	18264.11	21705.31	19347	18624.99	19060.03	14026.59	19283.73	17749	1.0900	0.32321
Q9EPC1	Parva	Alpha-parvin	88662.75	87466.13	85454.92	72672.09	83564	87012.85	89470.41	98004.12	90353.38	91210	0.9162	0.13199
B2RXS4	Plxnb2	Plexin-B2	26456.87	25490.64	27757.81	27204.05	26727	24715.91	26623.26	20698.01	23348.17	23846	1.1208	0.07496
Q3TW96	Uap1l1	UDP-N-acetylhexosamine pyrophosphorylase-like pr	61768.02	62861.64	44403.25	47464.64	54124	53832.98	53917.68	47904.52	49273.39	51232	1.0565	0.58552
O88485	Dync1i1	Cytoplasmic dynein 1 intermediate chain 1	75675.21	70492.45	66780.86	72311.8	71315	71088.63	68323.3	76107.7	79321.13	73710	0.9675	0.46731
Q8R105	Vps37c	Vacuolar protein sorting-associated protein 37C	42234.73	51351.44	49519.72	55739.12	49711	46780.32	51555.66	54118.18	53009.37	51366	0.9678	0.62825
Q8BTU1	Cfap20	Cilia- and flagella-associated protein 20	8546.252	5662.181	10466.46	9421.037	8524	4088.401	3965.8	10687.38	7921.542	6666	1.2788	0.37159
Q8R1V4	Tmed4	Transmembrane emp24 domain-containing protein 4	48342.68	48603.4	56869.64	59707.37	53381	49306.69	51460.98	64118.55	68530.97	58354	0.9148	0.40286
Q9R008	Mvk	Mevalonate kinase	44953.72	35347.32	48569.23	37964.27	41709	37243.26	34205.2	41420.47	43041.13	38978	1.0701	0.48322
Q9D4H1	Exoc2	Exocyst complex component 2	27981.74	31727.06	28613.88	25320.82	28411	26447.24	29037.6	30407.84	30860.17	29188	0.9734	0.65381
P70425	Rit2	GTP-binding protein Rit2	29428.92	30402.84	29971.13		29934	21094.32	23310.79	27516.86	27674.93	24899	1.2022	0.04799
Q3UJU9	Rmdn3	Regulator of microtubule dynamics protein 3	14978.77	13854.47	12312.25	14482.28	13907	11719.8	11768.52	8035.934	11060.54	10646	1.3063	0.02158
Q9ET22	Dpp7	Dipeptidyl peptidase 2	64545.31	69196.73	70625.37	63171.1	66885	75166.35	53294.07	83535.54	80053.13	73012	0.9161	0.41666
Q8BHG1	Nrdc	Nardilysin	40600.6	42442.11	45916.79	39627.32	42147	42285.31	44486.29	42582.71	46846.8	44050	0.9568	0.31583
Q9R1Q8	Tagln3	Transgelin-3	176543	184772.6	156109.7	153477.5	167726	173475.3	159156.8	141994.8	147349	155494	1.0787	0.28303
P48168	Glrb	Glycine receptor subunit beta			1609.756	5946.419	3778	3403.887	6194.35	5397.716		4999	0.7558	0.57372
Q32NY4	Cnnm3	Metal transporter CNNM3	9200.717	6347.493	3899.776	9974.433	7356	7886.436	6972.463		10367.52	8409	0.8748	0.59413
A2ALU4	Shroom2	Protein Shroom2	8814.651	7466.676	7363.491		7882	4085.509	6585.95	5519.92	5254.615	5361	1.4700	0.01751
Q9JIX0	Eny2	Transcription and mRNA export factor ENY2	27871.8	26050.8	30843.59	30657	28856	24342.82	27548.09	34268.3	28646.12	28701	1.0054	0.95013
O54879	Hmgb3	High mobility group protein B3	32243.04	32340.56	38126.67	22175.04	31221	28679.89	26737.75	32121.65	31970.84	29878	1.0450	0.71920
Q80TM9	Nisch	Nischarin	47467.42	46568.64	50897.61	42742.73	46919	49218.09	46811.73	48272.8	47541.82	47961	0.9783	0.57391
Q78JE5	Fbxo22	F-box only protein 22	19937.15	25035.68	20429.59	11775.06	19294	23157.77	20175.5	20131.11	19385.52	20712	0.9315	0.64000
P50172	Hsd11b1	11-beta-hydroxysteroid dehydrogenase 1				20426.1	20426							
Q810U3	Nfasc	Neurofascin	46352.35	44613.56	49194.62	51495.02	47914	45302.76	50036.05	45038.68	48295.68	47168	1.0158	0.71441
Q9CY64	Blvra	Biliverdin reductase A	69162.92	76123.34	67289.5	54136.94	66678	66804.34	61865.34	58880.88	55963.48	60879	1.0953	0.30243
P54869	Hmgcs2	Hydroxymethylglutaryl-CoA synthase, mitochondrial	730100.6	468683.7	668954.7	728004.2	648936	437992	498305.3	644404.4	650634.3	557834	1.1633	0.30650
Q8C5W3	Tbcel	Tubulin-specific chaperone cofactor E-like protein	25757.85	26287.45	27706.75	20636.79	25097	23124.19	23175.11	27362.8	23425.16	24272	1.0340	0.67216
O35215	Ddt	D-dopachrome decarboxylase	152775.2	188976.8	144905.9	112419.7	149769	147016.1	164952	118569.7	118487.4	137256	1.0912	0.54332
P59999	Arpc4	Actin-related protein 2/3 complex subunit 4	481525.2	493663.8	514742.7	442281.5	483053	480598.1	445198.4	495896.3	456836.2	469632	1.0286	0.50740
Q9JKV5	Scamp4	Secretory carrier-associated membrane protein 4	38728.47	35824.54	40918.95	47477.1	40737	35421.21	39833.51	43640.31	49218.25	42028	0.9693	0.74778

Q3TDQ1	Stt3b	Dolichyl-diphosphooligosaccharide--protein glycosy	77426.16	83405.08	89356.02	97612.23	86950	86007.73	86310.07	98577.76	96643.88	91885	0.9463	0.39971
Q8VE38	Oxnad1	Oxidoreductase NAD-binding domain-containing protein 1						28474.27			26945.75	27710	0.0000	
Q8K1A6	Cc2d1a	Coiled-coil and C2 domain-containing protein 1A	26155.65	26964.11	29711.2	21474.67	26076	28882.51	24601.21	24176.29	26883.46	26136	0.9977	0.97759
Q5XKE0	Mybpc2	Myosin-binding protein C, fast-type	39359.85	60124.41	29492.58	48600.65	44394	50274.6	52448.04	47482.8	54034.05	51060	0.8695	0.35741
O08648	Map3k4	Mitogen-activated protein kinase kinase kinase 4	34291.91	34841.05	35543.69	30061.75	33685	33018.76	35497.77	22921.96	24616.04	29014	1.1610	0.20991
Q6RI63	Fam120b	Constitutive coactivator of peroxisome proliferator-a	10878.54	9940.181	8519.928	8069.105	9352	6774.697	10416.76	3842.365	6862.735	6974	1.3409	0.16220
Q6PDI5	Ecpas	Proteasome adapter and scaffold protein ECM29	56336.31	55631.16	53113.4	49002.22	53521	54850.27	55952.08	53063.77	54396.53	54566	0.9809	0.57481
Q9CWP6	Mospd2	Motile sperm domain-containing protein 2	32088.3	31548.56	35935.07	30086.38	32415	30661.13	30773.72	22149.38	37410.08	30249	1.0716	0.54376
Q8BI72	Cdkn2aip	CDKN2A-interacting protein	14111.12	12382.5	18083.59	13093.04	14418	18561.84	10705.26	21249.57	17825.04	17085	0.8439	0.34189
Q99LN9	Dohh	Deoxyhypusine hydroxylase	37280.92	29108.77	33408.26	24076.63	30969	26492.96	27688.49	21741.16	22993.26	24729	1.2523	0.09655
Q9CQZ6	Ndufb3	NADH dehydrogenase [ubiquinone] 1 beta subcomp	115453.1	126894.4	113545.6	134006.5	122475	116892.1	136569.8	132814.6	131798.6	129519	0.9456	0.32002
Q6ZWY8	Tmsb10	Thymosin beta-10	156308.2	175596.5	188507.5	141351.4	165441	163749.4	160183.8	181976.3	148019.5	163482	1.0120	0.88113
Q9D0R2	Tars1	Threonine--tRNA ligase 1, cytoplasmic	126144.8	124868.8	124764.5	124870	125162	120151.7	132784.2	117787.7	127971.6	124674	1.0039	0.89320
Q8JZR0	Acsl5	Long-chain-fatty-acid--CoA ligase 5	27144.81	28287.31	28019.42	26536.05	27497	25092.66	27691.64	24739.11	30071.19	26899	1.0222	0.66375
O08739	Ampd3	AMP deaminase 3	55317.95	59548.68	54008.37	57692.79	56642	48780.1	54403.66	45749.05	49442.6	49594	1.1421	0.01772
Q9DCJ9	Npl	N-acetylneuraminate lyase	32569.89	33408.38	28838.8	27059.24	30469	25973.54	29238.26	27576.38	22893.88	26421	1.1532	0.09263
Q9R1V7	Adam23	Disintegrin and metalloproteinase domain-containin	56581.32	44979.39	43627.53	55043.87	50058	41539.33	51497.51	41554.1	46515.72	45277	1.1056	0.28876
P70297	Stam	Signal transducing adapter molecule 1	37944.08	42634.66	39036.87	36844.48	39115	43160.17	41389.77	41535.75	41203.57	41822	0.9353	0.08878
Q9D662	Sec23b	Protein transport protein Sec23B	31779.35	29797.49	33801.29	31737.7	31779	31559.7	28944.43	33768.78	32373.38	31662	1.0037	0.93112
O35668	Hap1	Huntingtin-associated protein 1	8155.882				8156							
P27612	Plaa	Phospholipase A-2-activating protein	73690.45	73829.88	72344.98	65561.74	71357	75848.39	75213.91	75649.35	70769.07	74370	0.9595	0.23852
Q8R1B4	Eif3c	Eukaryotic translation initiation factor 3 subunit C	140769.6	139751	158399.2	155254.7	148544	153896.5	146200.6	150992.3	169829.1	155230	0.9569	0.37877
Q9DCG9	Trmt112	Multifunctional methyltransferase subunit TRM112-l	46784.67	57350.62	56485.33	50899.52	52880	58404.11	49763.8	49009.05	52351.97	52382	1.0095	0.88410
P62281	Rps11	Small ribosomal subunit protein uS17	390334.5	379969.2	442231	437490.1	412506	408930	412410.2	446036.7	470588.7	434491	0.9494	0.34948
Q8VCF0	Mavs	Mitochondrial antiviral-signaling protein	15561.69	13613.47	17247.44	13270.69	14923	13192.55	15216.53	13336.12	15250.02	14249	1.0473	0.55725
Q6PDG5	Smarcc2	SWI/SNF complex subunit SMARCC2	34447.82	37452.52	41943.01	42812.48	39164	39971.33	36839.18	42461.3	41669.6	40235	0.9734	0.66105
Q8CAQ8	Immt	MICOS complex subunit Mic60	211680	208843.4	219487.5	244751.4	221191	216740.2	230321.2	231434.1	230351.3	227212	0.9735	0.52337
P11798	Camk2a	Calcium/calmodulin-dependent protein kinase type	72394.68	100857.9	82649.02	93396.41	87324	94020.48	104500.3	78629.77	83288.35	90110	0.9691	0.75408
Q8C033	Arhgef10	Rho guanine nucleotide exchange factor 10	30615.03	32411.68	36322.23	27196.37	31636	30424.27	29272.31	34105.48	34382.24	32046	0.9872	0.86432
Q9WVJ2	Psmd13	26S proteasome non-ATPase regulatory subunit 13	98498	95052.2	96215.97	89167.22	94733	93955.63	93448.59	93941.05	91665.13	93253	1.0159	0.49952
Q9CY27	Tecr	Very-long-chain enoyl-CoA reductase	183895.5	171352.9	191189.8	196392.9	185708	167215.4	181550	225294.6	216170.6	197558	0.9400	0.45530
O89023	Tpp1	Tripeptidyl-peptidase 1	136114.3	105211.4	112353.1	105237.5	114729	94188.17	125461.3	105832.6	113349.4	109708	1.0458	0.62794
Q8R3Q2	Ppp6r2	Serine/threonine-protein phosphatase 6 regulatory s	18872.14	17277.81	18833.88	16851.58	17959	16953.59	17019.49	14901.52	13286.6	15540	1.1556	0.05888
Q8K019	Bclaf1	Bcl-2-associated transcription factor 1	12413.11	19152.42	20851.74	21876.94	18574	16073.62	21177.52	27222.83	22311.3	21696	0.8561	0.35625
Q8BHC1	Rab39b	Ras-related protein Rab-39B	46604.5	43164.14	43666.73	45676.22	44778	40935.29	43592.12	41715.34	44092.09	42584	1.0515	0.09510
Q8BTS4	Nup54	Nuclear pore complex protein Nup54	14646.46	14483.24	17310.76	15023.35	15366	18179.93	14167.15	16322.53	16617.49	16322	0.9414	0.40024
Q64516	Gk	Glycerol kinase	61082.8	57208.55	54672.82	61473.79	58609	58531.79	65053.38	57375.02	60008.93	60242	0.9729	0.51274
P83917	Cbx1	Chromobox protein homolog 1	13380.74	15356.03	20663.45	15467.04	16217	16930	15122.63	19194.14	19766.98	17753	0.9134	0.44717
Q8CAK1	Iba57	Putative transferase CAF17 homolog, mitochondrial	21742.94	18527.41	23552.02	17175.61	20249	12872.95	14743	7717.171	13948.51	12320	1.6436	0.01027
Q9Z2G6	Sel1l	Protein sel-1 homolog 1	76149.06	66189.85	65013.63	74351.41	70426	69744.7	84166.91	79524.76	74618.98	77014	0.9145	0.16761
Q91VC7	Ppp1r14a	Protein phosphatase 1 regulatory subunit 14A	6766.518	5632.474	12584.77		8328							
O88532	Zfr	Zinc finger RNA-binding protein	22127.38	24429.43	26783.41	26947.43	25072	26675.54	26818.93	29573.35	30185.73	28313	0.8855	0.06802
Q99LB7	Sardh	Sarcosine dehydrogenase, mitochondrial	18411.03	21218.49	18370.92	20475.35	19619	20332.81	17016.84	16745.19	23406.54	19375	1.0126	0.89269
Q9D024	Ccdc47	PAT complex subunit CCDC47	43583.16	38855.37	45488.32	48630	44139	44263.79	43424.41	43302.67	47879.47	44718	0.9871	0.81074
Q9CQ20	Mid1ip1	Mid1-interacting protein 1	13689.98	17015.79	19425.19	9034.078	14791	19115.7	23574.36	35346.2	24989.9	25757	0.5743	0.03696
A2AQ25	Skt	Sickle tail protein	21286.18	25615.61	20526.59	19957.28	21846	21883.39	20980.7	12511.25	15449.75	17706	1.2338	0.16013
Q80UM3	Naa15	N-alpha-acetyltransferase 15, NatA auxiliary subunit	50116.76	55899.36	52443.42	53600.52	53015	56544.14	54941.69	59730.91	51933.18	55787	0.9503	0.21949
P62715	Ppp2cb	Serine/threonine-protein phosphatase 2A catalytic s	283468.6	306742.1	286707.1	270944.7	286966	293287.9	282807.1	287957.8	284227.5	287070	0.9996	0.98972
O35874	Slc1a4	Neutral amino acid transporter A	24183.53	26582.94	25429.99	30530.18	26682	27792.49	35075.25	22674.94	25909.05	27863	0.9576	0.70397
P23492	Pnp	Purine nucleoside phosphorylase	81975.48	75009.74	84646.38	64610.26	76560	75802.99	64782.93	61995.41	68551.53	67783	1.1295	0.15391
Q91WT9	Cbs	Cystathionine beta-synthase		4298.279			4298							
Q62421	Sh3gl3	Endophilin-A3												
Q6DVA0	Lemd2	LEM domain-containing protein 2	18314.96	17588.11	24053.1	29805.46	22440	16270.79	25269.44	26230.61	27260.76	23758	0.9445	0.74125
Q6P5D8	Smchd1	Structural maintece of chromosomes flexible hinge d	32957.03	30818.13	28702.54	26643.4	29780	30311.63	27148.43	27261.65	26291.82	27753	1.0730	0.25722
Q68EF0	Rab3ip	Rab-3A-interacting protein	4978.971	2535.36	4450.393	3752.988	3929	2246.882	1340.577	3689.425	1540.94	2204	1.7825	0.06097
P11404	Fabp3	Fatty acid-binding protein, heart	384714.8	426358.1	342126.4	266306.8	354877	379850	290368.3	286762.3	266044.3	305756	1.1607	0.29168
Q8CIV8	Tbce	Tubulin-specific chaperone E	11903.26	12891.2	11655.27	8046.624	11124	9611.881	9758.723	8476.058	10312.3	9540	1.1661	0.20969
Q8BRT1	Clasp2	CLIP-associating protein 2	23107.13	22985.18	24796.08	24281.03	23792	21741.56	23698.5	24700.75	26901.95	24261	0.9807	0.70085
Q9R1J0	Nsdhl	Sterol-4-alpha-carboxylate 3-dehydrogenase, decarl	42396.62	40534.46	55351.84	52080.82	47591	44416.48	47631.21	57874.52	67512.13	54359	0.8755	0.32880

P04925	Prnp	Major prion protein	60527.84	54112.03	36233.75	53096.3	**50992**	46556.94	58865.66	47908.16	37877.48	**47802**	**1.0667**	**0.65276**
Q8K2T1	Nmral1	NmrA-like family domain-containing protein 1	32567.34	32322.98	28269.26	99039.55	**48050**	31224.09	13805.07	10301.79		**18444**	**2.6052**	**0.21502**
Q8BNW9	Kbtbd11	Kelch repeat and BTB domain-containing protein 11	26462.4	22456.53	22596.22	23349.27	**23716**	22943.84	23843.91	19991.55	20728.79	**21877**	**1.0841**	**0.20805**
Q9D6Z1	Nop56	Nucleolar protein 56	49014.15	46159.97	55428.7	51011.84	**50404**	50666.41	50095.42	54394.33	59153.99	**53578**	**0.9408**	**0.30907**
Q9CZ13	Uqcrc1	Cytochrome b-c1 complex subunit 1, mitochondrial	368452.5	339353.8	340289.3	355359	**350864**	343574.6	372743.4	360368.2	362216.7	**359726**	**0.9754**	**0.37154**
Q3UFY7	Nt5c3b	7-methylguanosine phosphate-specific 5'-nucleotida	32067.38	25177.4	30431.47	25776.52	**28363**	36223.02	26408.62	16894.27	24885.04	**26103**	**1.0866**	**0.61931**
Q6NS82	Retreg2	Reticulophagy regulator 2	20775.73	27221.87	23804.9	21152.4	**23239**	21575.61	20398.43	27691.37	15887.42	**21388**	**1.0865**	**0.54046**
Q9CYN9	Atp6ap2	Renin receptor	10225.95	9529.526	12432.89	7984.187	**10043**	17455.38	11525.57	10226.76	15782.35	**13748**	**0.7305**	**0.10572**
Q91W69	Epn3	Epsin-3	33889.93	33913.87	36079.97	29096.48	**33245**	32408.49	34572.17	30526.19	30097.88	**31901**	**1.0421**	**0.48225**
Q9D1G5	Lrrc57	Leucine-rich repeat-containing protein 57	116354.7	106250.6	94578.67	125351.9	**110634**	112465.7	116289	104104.1	104970.8	**109457**	**1.0107**	**0.87642**
Q61210	Arhgef1	Rho guanine nucleotide exchange factor 1	45691.35	42761.65	42098.16	35849.86	**41600**	46277.13	41266.01	37291.53	41484.67	**41580**	**1.0005**	**0.99435**
P48678	Lmna	Prelamin-A/C	716270.6	654668.2	711818.4	779297.6	**715514**	723124.5	704397.2	729393	703067.4	**714996**	**1.0007**	**0.98493**
Q8CF89	Tab1	TGF-beta-activated kinase 1 and MAP3K7-binding protein 1												
Q8BYY4	Ttc39b	Tetratricopeptide repeat protein 39B	21483.33	19158.91	18383.77	15341.24	**18592**	14075.48	17661.63	10904.19	12638.9	**13820**	**1.3453**	**0.04706**
P13542	Myh8	Myosin-8	31311.12	39720.83	20199.59	50882.37	**35528**	15649	30057.82	7800.484	16391.51	**17475**	**2.0331**	**0.06412**
Q9EQK5	Mvp	Major vault protein	151599.1	164201.8	166043.7	185279	**166781**	162145.6	181182.9	166925.3	169639.3	**169973**	**0.9812**	**0.70515**
P01027	C3	Complement C3	299107.3	275362.9	315600.3	190693	**270191**	240060.9	205584.8	197590.5	156867	**200026**	**1.3508**	**0.07482**
Q8BH57	Wdr48	WD repeat-containing protein 48	10760.06	16356.29	11309.81	11285.41	**12428**	14703.21	16906.3	12497.43	9439.979	**13387**	**0.9284**	**0.65905**
Q59J78	Ndufaf2	NADH dehydrogenase [ubiquinone] 1 alpha subcom	32474.34	34508.72	37613.52	35796.93	**35098**	35794.75	38549.44	33454.19	41376.33	**37294**	**0.9411**	**0.32028**
P47740	Aldh3a2	Aldehyde dehydrogenase family 3 member A2	150913.4	145329	156761.5	171549.3	**156138**	148947.4	150823	158205.4	166362.2	**156084**	**1.0003**	**0.99403**
Q60605	Myl6	Myosin light polypeptide 6	409995.9	407242.8	441432.2	452082.6	**427688**	420026.4	439935.5	512205.6	463681.1	**458962**	**0.9319**	**0.21964**
P11103	Parp1	Poly [ADP-ribose] polymerase 1	52556.2	51914.31	58690.6	53841.01	**54251**	60388.59	55336.11	54858.89	61295.53	**57970**	**0.9358**	**0.15217**
Q8CFA2	Amt	Aminomethyltransferase, mitochondrial							696.9954			**697**	**0.0000**	
Q8VC88	Gca	Grancalcin	24265.6				**24266**		10962.74	7660.318		**9312**	**2.6060**	
Q9R078	Prkab1	5'-AMP-activated protein kinase subunit beta-1	9543.866	15827.62	14852.74	13362.93	**13397**	13046.25	16522.26	6443.567	9121.429	**11283**	**1.1873**	**0.44843**
Q3URJ8	Nkain3	Sodium/potassium-transporting ATPase subunit beta	4552.417	4701.459	7401.53	4372.675	**5257**	53427.59	5659.324		5165.407	**21417**	**0.2455**	**0.28235**
Q7TNF0	Doc2a	Double C2-like domain-containing protein alpha												
Q922G0	Slc25a36	Solute carrier family 25 member 36	15258.09	21299.75	24721.31	17020.53	**19575**	19542.9	20345.69	22188.94	25117.24	**21799**	**0.8980**	**0.40196**
P33175	Kif5a	Kinesin heavy chain isoform 5A	427756	372560.4	342894.6	372473.2	**378921**	393217.1	353772.9	382017.8	351473.7	**370120**	**1.0238**	**0.68303**
Q60631	Grb2	Growth factor receptor-bound protein 2	113812.8	120537.3	101084.4	113385.2	**112205**	111341.9	118154.8	102982.3	99814.99	**108073**	**1.0382**	**0.50293**
Q8BFR4	Gns	N-acetylglucosamine-6-sulfatase	20280.05	24150.23	25159.43	25119.71	**23677**	22409.92	23041.63	26577.17	26558.98	**24647**	**0.9607**	**0.56847**
P28659	Celf1	CUGBP Elav-like family member 1	35137.14	30845.01	34872.12	31384.08	**33060**	32368.86	32728.55	30618.56	34346.05	**32516**	**1.0167**	**0.70381**
O35465	Fkbp8	Peptidyl-prolyl cis-trans isomerase FKBP8	131453	131061.8	146853.6	145797.4	**138791**	139648.1	149995.2	150169.2	144074.8	**145972**	**0.9508**	**0.20429**
O08759	Ube3a	Ubiquitin-protein ligase E3A	28721.05	31895.55	31605.42	24498.62	**29180**	30716.24	29465.09	30064.27	28509.14	**29689**	**0.9829**	**0.78472**
Q8K0P3	Meak7	MTOR-associated protein MEAK7	9162.857	6714.691	9609.863	9180.315	**8667**	10564.62	9900.825	8280.592	10648.04	**9849**	**0.8800**	**0.21739**
Q9DBB8	Dhdh	Trans-1,2-dihydrobenzene-1,2-diol dehydrogenase	53592.25	53485.34	59592.61	50179.84	**54213**	52621.07	43453.58	49479.39	45272.91	**47707**	**1.1364**	**0.06252**
Q8BU33	Ilvbl	2-hydroxyacyl-CoA lyase 2	46666.49	40032.11	46540.63	52415.61	**46414**	42290.88	47418.84	50333.64	47722.89	**46942**	**0.9888**	**0.86780**
O35988	Sdc4	Syndecan-4	12785.36	12672.63	8854.331	6769.592	**10270**	7490.585	10517.62	6260.252	6174.021	**7611**	**1.3495**	**0.18912**
Q9D706	Rpap3	RNA polymerase II-associated protein 3	6307.342	5709.708	5548.396	4922.339	**5622**	5427.835	5890.508	6143.669	5120.708	**5646**	**0.9958**	**0.95034**
O35409	Folh1	Glutamate carboxypeptidase 2	55287.19	51434.65	47795.18	46868.98	**50346**	39699.39	57644.78	53790.04	46350.21	**49371**	**1.0198**	**0.83280**
P70460	Vasp	Vasodilator-stimulated phosphoprotein	39152.81	39235.29	39617.15	36722.88	**38682**	35050.76	35343.25	45060.44	38864.86	**38580**	**1.0026**	**0.96768**
Q80ZI6	Lrsam1	E3 ubiquitin-protein ligase LRSAM1	156360.4	151964.1	144561.8	144321.5	**149302**	150860	146178.4	140998.9	128558.7	**141649**	**1.0540**	**0.22343**
P58044	Idi1	Isopentenyl-diphosphate Delta-isomerase 1	107838.2	120006.6	171968.5	96925.7	**124185**	116353.4	108219.7	134558.1	161270.3	**130100**	**0.9545**	**0.78109**
P61164	Actr1a	Alpha-centractin	262566.5	269994.7	282618.2	299232.5	**278603**	268113.4	270059.1	281292.5	294952.7	**278604**	**1.0000**	**0.99989**
Q8BQM8	Eml5	Echinoderm microtubule-associated protein-like 5			8970.964		**8971**				5321.6	**5322**	**1.6858**	
Q9D5T0	Atad1	Outer mitochondrial transmembrane helix transloca	64924.32	70466.11	64266.62	72100	**67939**	59211.73	71737.35	52127.14	57424.48	**60125**	**1.1300**	**0.13983**
P10711	Tcea1	Transcription elongation factor A protein 1	74602.66	82508.86	81157.17	61715.7	**74996**	70788.2	64844.22	76580.25	72939.47	**71288**	**1.0520**	**0.51415**
Q78IK2	Atp5mk	ATP synthase membrane subunit K, mitochondrial	319139.7	277345.2	327078	341548.2	**316278**	314357.4	303295.7	320447.3	333625.3	**317931**	**0.9948**	**0.91671**
Q91VC9	Ghitm	Growth hormone-inducible transmembrane protein	73167.86	74124.24	70052.25	78195.94	**73885**	68025.3	81953.58	76895.91	85731.72	**78152**	**0.9454**	**0.34699**
Q9EP69	Sacm1l	Phosphatidylinositol-3-phosphatase SAC1	186358.8	185636	201283.8	215000.9	**197070**	205730.3	192801.2	212635.5	216230	**206849**	**0.9527**	**0.30309**
Q6ZQK5	Acap2	Arf-GAP with coiled-coil, ANK repeat and PH domain-	29819.88	54204.53	21397.09	30006.85	**33857**	23366.9	25583.14	12068.57	13562.24	**18645**	**1.8159**	**0.10083**
P32848	Pvalb	Parvalbumin alpha	88368.56	176315.6	100096.4	102525.5	**116827**	101297	128694.4	58523.5	56482.86	**86249**	**1.3545**	**0.29475**
P08553	Nefm	Neurofilament medium polypeptide	8747365	7939258	6022434	9462533	**8042898**	7795517	8333133	5761233	6323922	**7053451**	**1.1403**	**0.34114**
Q91W52	Tmem19	Transmembrane protein 19	32391.38	32198.41	40724.74	38897.21	**36053**	34281.33	36688.85	44473.86	45127.95	**40143**	**0.8981**	**0.28849**
P62270	Rps18	Small ribosomal subunit protein uS13	742635.1	736381.7	897948.4	887648.6	**816153**	850227.9	824274.2	1030121	1009039	**928416**	**0.8791**	**0.15560**
O35495	Cdk14	Cyclin-dependent kinase 14	12372.25	15027.85	15681.77	14908.1	**14497**	17874.25	16978.36	13962.21	11704.62	**15130**	**0.9582**	**0.70495**
Q61171	Prdx2	Peroxiredoxin-2	666730.9	689863.6	646653.5	610824.8	**653518**	643963.4	654049.5	618043.8	582121.7	**624545**	**1.0464**	**0.25810**
O70318	Epb41l2	Band 4.1-like protein 2	1287463	1192002	1072795	1410805	**1240766**	1161031	1230380	1062510	1161679	**1153900**	**1.0753**	**0.31692**
Q8CG76	Akr7a2	Aflatoxin B1 aldehyde reductase member 2	66049.86	63651.07	68474.03	57006.63	**63795**	62376	68810.47	47641.11	54933.86	**58440**	**1.0916**	**0.34319**

Q6A068	Cdc5l	Cell division cycle 5-like protein	25218.38	24439	28560.2	26509.74	**26182**	24830.35	25351.45	32813.71	32271.04	**28817**	**0.9086**	**0.30261**
P01029	C4b	Complement C4-B	18227.4	11538.18	17177.63	10889.82	**14458**	13589.89	10926.12	10434.68	10266.6	**11304**	**1.2790**	**0.17350**
Q8R550	Sh3kbp1	SH3 domain-containing kinase-binding protein 1	16596.56	14248.94	13878.73	13190.38	**14479**	12548.66	15102.52	13339	14623.02	**13903**	**1.0414**	**0.56409**
Q8K386	Rab15	Ras-related protein Rab-15	1010309	2136826	1002987	1090438	**1310140**	2127439	1010162	1204055	1057091	**1349687**	**0.9707**	**0.92074**
Q9WVL0	Gstz1	Maleylacetoacetate isomerase	48270.18	51107.66	48391.39	50356.14	**49531**	46216.77	46790.48	42551.94	44972.46	**45133**	**1.0975**	**0.00971**
Q5M8N0	Cnrip1	**CB1 cannabinoid receptor-interacting protein 1**	65534.22	70619.3	68557.35	72200.48	**69228**	62475.85	62748.07	52869.16	59043.84	**59284**	**1.1677**	**0.01050**
P68254	Ywhaq	14-3-3 protein theta	98144.83	112460.2	101868.7	93012.38	**101372**	106524.6	105438.4	101542.2	99563.84	**103267**	**0.9816**	**0.68364**
Q8VE09	Ttc39c	Tetratricopeptide repeat protein 39C	39702.5	25540.5	18053.69	13324.47	**24155**	13748.72	18244.96	13223.19	32723.33	**19485**	**1.2397**	**0.54826**
Q9CQT1	Mri1	Methylthioribose-1-phosphate isomerase	24731.02	24686.8	23203.04	19665.54	**23072**	24053.53	28909.59	20308.38	20324.1	**23399**	**0.9860**	**0.89420**
P51855	Gss	Glutathione synthetase	33905.54	35463.12	29158.15	25663.75	**31048**	32114.06	31988.28	25668.09	25050.69	**28705**	**1.0816**	**0.45900**
Q9D142	Nudt14	Uridine diphosphate glucose pyrophosphatase NUD	23580.86	24115.88	24200.18	16323.45	**22055**	21105.71	22034.33	24727.15	18954.24	**21705**	**1.0161**	**0.88200**
O88851	Rbbp9	Putative hydrolase RBBP9	36323.65	36570.8	30239.6	32831.44	**33991**	35324.42	27105.53	28404.02	29998.59	**30208**	**1.1252**	**0.15946**
Q91WF7	Fig4	Polyphosphoinositide phosphatase		448.3788			**448**				2352.647	**2353**	**0.1906**	
Q8C7H1	Mmaa	Methylmalonic aciduria type A homolog, mitochondr	10380.06	570.7798		6917.422	**5956**							
Q9Z0V8	Timm17a	Mitochondrial import inner membrane translocase s	10224.61	9276.604	11662.78	8932.879	**10024**	11707.67	9465.01	8947.911	11048.47	**10292**	**0.9740**	**0.77386**
O88342	Wdr1	WD repeat-containing protein 1	245071.1	243886	228479.4	206333.3	**230942**	234109.8	217093.6	225967.6	230131.3	**226826**	**1.0181**	**0.68724**
P84075	Hpca	Neuron-specific calcium-binding protein hippocalcir	124328.6	141005.1	127236.5	99486.32	**123014**	105162.8	134089.8	70722.18	88275.19	**99563**	**1.2355**	**0.19354**
O54962	Banf1	Barrier-to-autointegration factor	80118.47	79750.98	85544.5	93433.28	**84712**	87287.22	89931.93	81649.71	93713.76	**88146**	**0.9610**	**0.43208**
Q921H9	Coa7	Cytochrome c oxidase assembly factor 7	9686.301	18373.9	11299.83		**13120**		14014.09			**14014**	**0.9362**	
Q8VDM4	Psmd2	26S proteasome non-ATPase regulatory subunit 2	104805.8	107611.1	113340.6	103630.9	**107347**	108956.8	102597.5	116306.9	119320.8	**111795**	**0.9602**	**0.34479**
G5E870	Trip12	E3 ubiquitin-protein ligase TRIP12	16688.53	17489.65	15717.09	17392.33	**16822**	12827.53	15322.86	18024.24	15987.69	**15541**	**1.0825**	**0.30666**
Q9D154	Serpinb1a	Leukocyte elastase inhibitor A	84124.64	81645.73	68587.99	60643.44	**73750**	70949.07	84316.85	57136.34	52087.66	**66122**	**1.1154**	**0.43548**
Q9Z2A5	Ate1	Arginyl-tRNA--protein transferase 1	17635	17687.8	17057.62	16400.07	**17195**	18164.28	15710.75	18655.8	15290.05	**16955**	**1.0141**	**0.79916**
E9Q912	Rap1gds1	Rap1 GTPase-GDP dissociation stimulator 1	513446.5	555322.4	468219.2	377014.4	**478501**	484821.5	470069.5	464231.2	433660.4	**463196**	**1.0330**	**0.71318**
Q8BXR1	Slc7a14	Probable cationic amino acid transporter	340685.7				**340686**							
Q8BFV2	Pcid2	PCI domain-containing protein 2	3359.47	2774.824	3152.871	2488.212	**2944**	3395.124	4086.41	772.9711	2755.933	**2753**	**1.0695**	**0.80460**
O55028	Bckdk	[3-methyl-2-oxobutanoate dehydrogenase [lipoamid	8149.396	8880.854	11694.49	9538.102	**9566**	8833.423	8966.197	11785.13	7914.044	**9375**	**1.0204**	**0.87169**
Q9JIA1	Lgi1	Leucine-rich glioma-inactivated protein 1	44553.14	48555.12	49117.3	56926.64	**49788**	52833.98	47203.73	49776.79	51639.35	**50363**	**0.9886**	**0.84738**
Q99J23	Ghdc	GH3 domain-containing protein	12667.99	11513.84	12949.96	16206.85	**13335**	14390.88	12342.08	10567.08	10297.96	**11899**	**1.1206**	**0.33890**
Q9Z0J4	Nos1	Nitric oxide synthase 1		16006.12			**16006**							
Q9JJU8	Sh3bgrl	Adapter Sh3bgrl	43057.81	46909.55	40098.82	28795.9	**39716**	36799.48	40014.77	36892.01	31321.45	**36257**	**1.0954**	**0.45151**
P08752	Gnai2	Guanine nucleotide-binding protein G(i) subunit alph	559848.3	491860.1	468182.2	588044.1	**526984**	525670.4	593732.8	480461.3	457175.7	**514260**	**1.0247**	**0.76777**
Q9Z2A0	Pdpk1	3-phosphoinositide-dependent protein kinase 1	25319.57	25922.35	22626.38	26305.71	**25043**	24014.88	27691.94	21702.28	25456.36	**24716**	**1.0132**	**0.83537**
P50428	Arsa	Arylsulfatase A	35951.69	37396.09	36447.97	29759.66	**34889**	37401.13	33801.79	35657.01	36497.14	**35839**	**0.9735**	**0.63432**
P70689	Gjb6	Gap junction beta-6 protein						1857.725				**1858**	**0.0000**	
P09411	Pgk1	Phosphoglycerate kinase 1	597989.1	577886.3	558260.1	457651.6	**547947**	567629.1	527598.9	462401.2	434470.5	**498025**	**1.1002**	**0.29463**
P35123	Usp4	Ubiquitin carboxyl-terminal hydrolase 4	34412.84	37983.23	32260.39	26309.38	**32741**	32149.17	33458.27	28824.73	25128.56	**29890**	**1.0954**	**0.38967**
Q91YD9	Wasl	Actin nucleation-promoting factor WASL	37504.86	34099.29	31823.54	35994.58	**34856**	32689.59	36094.12	34601.14	35550.11	**34734**	**1.0035**	**0.93522**
Q05CL8	Larp7	La-related protein 7	16562.97	17421.97	15189.62	18043.41	**16804**	17811.42	16270.04	14860.13	17998.76	**16735**	**1.0041**	**0.94474**
P26645	Marcks	Myristoylated alanine-rich C-kinase substrate	174442	207187.1	173394.8	185336.5	**185090**	160342	228920	174153.8	184859.8	**187069**	**0.9894**	**0.90994**
Q922Q1	Mtarc2	Mitochondrial amidoxime reducing component 2	152398.1	129097.9	139786.8	155386.8	**144167**	137720	138520.7	138468.8	157125.1	**142959**	**1.0085**	**0.88011**
Q9CRA4	Msmo1	Methylsterol monooxygenase 1	67828.1	64850.96	85986.15	76890.93	**73889**	81453.11	76880.99	84113.42	107156.8	**87401**	**0.8454**	**0.15345**
Q8QZV4	Stk32c	Serine/threonine-protein kinase 32C		2478.825		2835.884	**2657**		3595.374		3948.661	**3772**	**0.7045**	**0.04720**
Q61644	Pacsin1	Protein kinase C and casein kinase substrate in neur	151572	138755	111401.6	126970.4	**132175**	134289.4	139655	123472.6	116624.2	**128510**	**1.0285**	**0.72687**
Q6PDM2	Srsf1	Serine/arginine-rich splicing factor 1	134017	146449.2	158678.7	194362.7	**158377**	161004.1	162676.7	209704.7	200290.3	**183419**	**0.8635**	**0.21617**
Q9D164	Fxyd6	FXYD domain-containing ion transport regulator 6	11247.5	9254.578	7854.903	7103.121	**8865**	10958.48	7814.02	11190.61	4136.107	**8525**	**1.0399**	**0.86288**
Q9CQ62	Decr1	2,4-dienoyl-CoA reductase [(3E)-enoyl-CoA-producii	224094.7	201312.6	208372.9	254131.2	**221978**	220333.4	218332.3	224405.5	213737.1	**219202**	**1.0127**	**0.82383**
Q62418	Dbnl	Drebrin-like protein	27196.6	26234.67	25571.9	21839.02	**25211**	25971.93	23975.61	24005.75	21683.95	**23909**	**1.0544**	**0.40825**
Q8BHF7	Pgs1	CDP-diacylglycerol--glycerol-3-phosphate 3-phosph	32726.96	35353.99	38956	40072.63	**36777**	33610.46	30051.7	33818.64	37691.19	**33793**	**1.0883**	**0.24138**
Q8R3I2	Mboat2	Lysophospholipid acyltransferase 2	15277.19	18226.97	19300.93	21664.15	**18617**	17133.44	20129.65	21116.92	20253.96	**19658**	**0.9470**	**0.53556**
Q922J3	Clip1	CAP-Gly domain-containing linker protein 1	13992.22	13777.32	15151.56	16725.57	**14912**	15686.67	13857.91	13731.28	16227.05	**14876**	**1.0024**	**0.97033**
Q03517	Scg2	Secretogranin-2	33586.5	36160.47	47007.48	43184.73	**39985**	34777.44	41063.26	43822.5	49975.15	**42410**	**0.9428**	**0.60307**
P06745	Gpi	Glucose-6-phosphate isomerase	334233.2	347681.1	309181.5	283033.6	**318532**	344048.6	324565.9	295235.7	271100.8	**308738**	**1.0317**	**0.66453**
Q8BM72	Hspa13	Heat shock 70 kDa protein 13		13671.45	7493.066		**10582**	4234.81	12863.66	8211.542	6259.575	**7892**	**1.3408**	**0.46732**
Q9DD18	Dtd1	D-aminoacyl-tRNA deacylase 1	35086.75	30450.06	24904	24031.35	**28618**	26807.85	30812.77	28472.95	25151.25	**27811**	**1.0290**	**0.78671**
Q3UZV7	Elapor2	Endosome/lysosome-associated apoptosis and autc	31760.77	30701.05	26116.13	35543.61	**31030**	27114.63	31335.62	25733.49	25806.49	**27498**	**1.1285**	**0.18271**
P54116	Stom	Stomatin	36484.82	30680.3	38916.78	37833.43	**35979**	34794.67	34068.88	33826.44	31509.33	**33550**	**1.0724**	**0.26318**
Q8CCJ4	Amer2	APC membrane recruitment protein 2	5688.55	6089.642	4939.033	7241.266	**5990**	14589.34	13046.53	8122.419	8226.411	**10996**	**0.5447**	**0.02741**
Q8VCH6	Dhcr24	Delta(24)-sterol reductase	15242.9	25220.06	47445.52	45250.48	**33290**	37338.02	18802.11	48172.24	52851.5	**39291**	**0.8473**	**0.60117**

Q8VHI6	Wasf3	Actin-binding protein WASF3		5031.101			**5031**				10009.12	**10009**	0.5027	
Q91W89	Man2c1	Alpha-mannosidase 2C1	49554.41	44771.66	38889.79	37329.41	**42636**	43524.28	42036.37	35432.05	32027.91	**38255**	1.1145	0.30530
P55012	Slc12a2	Solute carrier family 12 member 2	265657.9	275240.7	253440.6	314305.8	**277161**	264412.6	297484.9	266927.4	268035	**274215**	1.0107	0.85360
Q9CQ60	Pgls	6-phosphogluconolactonase	106129.4	112569.8	113527.5	90389.86	**105654**	99632.83	92579.1	92959.13	84494.52	**92416**	1.1432	0.07591
Q8C167	Prepl	Prolyl endopeptidase-like	37319.67	37205.97	33807.27	34887.65	**35805**	36457.98	35822.61	32395.17	32142.74	**34205**	1.0468	0.30377
O35316	Slc6a6	Sodium- and chloride-dependent taurine transporter	14808.04	17591.88			**16200**		37900.53			**37901**	0.4274	
Q6ZWU9	Rps27	Small ribosomal subunit protein eS27	32870.15	37288.67	39363.68	37998.16	**36880**	40214.09	34462.1	48778.77	50720.59	**43544**	0.8470	0.15040
Q62446	Fkbp3	Peptidyl-prolyl cis-trans isomerase FKBP3	216326.2	213706.6	232873	229023.5	**222982**	196706.8	199398.4	237933.8	235896.2	**217484**	1.0253	0.66757
P07758	Serpina1a	Alpha-1-antitrypsin 1-1	256540.5	281433.9	438372.2	203552.9	**294975**	225058	171710.9	263762.4	217740.1	**219568**	1.3434	0.21130
Q80U63	Mfn2	Mitofusin-2	30047.86	36262.15	34221.16	39633.8	**35041**	34383.73	32407.2	32825.38	34665.34	**33570**	1.0438	0.50617
Q61699	Hsph1	Heat shock protein 105 kDa	158520.1	191633	197085.1	172805.8	**180011**	190428.4	167230.8	197836.6	191899.6	**186849**	0.9634	0.56125
Q8C6G8	Wdr26	WD repeat-containing protein 26	17284.56	16626.25	20324.11	18447.63	**18171**	17736.47	18863.86	16735.18	18936.33	**18068**	1.0057	0.91867
Q99KW9	Itfg1	T-cell immunomodulatory protein	15669.65	15278.93	18913.09	16164.38	**16507**	16378.7	21213.61	23025.22	22056.11	**20668**	0.7986	0.04899
P62334	Psmc6	26S proteasome regulatory subunit 10B	77396.04	88573.59	88251.98	78640.9	**83216**	83813.16	81691.13	90297.15	91294.63	**86774**	0.9590	0.38906
Q80VP1	Epn1	Epsin-1	38048.99	37550.89	37233.66	35926.21	**37190**	35082.62	37091.73	39021.07	34541.31	**36434**	1.0207	0.52426
Q9Z1G4	Atp6v0a1	V-type proton ATPase 116 kDa subunit a 1	92153.96	100835.7	110617	108150.6	**102939**	102578.4	107417.4	108043.8	116263.6	**108576**	0.9481	0.30526
P15920	Atp6v0a2	V-type proton ATPase 116 kDa subunit a 2	11139.07	10185.64	13280.47	13660.03	**12066**	13520.4	13078.92	13557.97	13351.98	**13377**	0.9020	0.17145
P70122	Sbds	Ribosome maturation protein SBDS	33455.93	34603.4	33910.43	33147.34	**33779**	32198.23	32283.44	31151.97	29562.93	**31299**	1.0792	0.01277
Q8BJW6	Eif2a	Eukaryotic translation initiation factor 2A	53073.32	48522.48	53397.56	53493.03	**52122**	47438.42	51687.68	53988.52	57592.01	**52677**	0.9895	0.82787
P62307	Snrpf	Small nuclear ribonucleoprotein F	23421.31	23586.35	18242.14	18103.38	**20838**	25160.26	22818.12	37126.38	27110.38	**28054**	0.7428	0.08523
P28741	Kif3a	Kinesin-like protein KIF3A	21270.78	22049.01	18562.72	21615.23	**20874**	23220.19	20951.21	20056.44	20532.84	**21190**	0.9851	0.77457
Q62426	Cstb	Cystatin-B	62069.42	60023.59	73140.82	53981.6	**62304**	67137.45	52826.93	53070.5	58477.1	**57878**	1.0765	0.42883
P17809	Slc2a1	Solute carrier family 2, facilitated glucose transporte	510723.7	495654.1	436465.4	558214.4	**500264**	515931.4	486463.7	463824	456926.3	**480786**	1.0405	0.51852
Q9D0L7	Armc10	Armadillo repeat-containing protein 10	22153.88	23905.74	24345.21	24691.12	**23774**	24683.38	21309.17	24795.33	26764.38	**24388**	0.9748	0.64447
Q9DC63	Fbxo3	F-box only protein 3	20055.03	18794.18	18406.38	16553.93	**18452**	19128.74	18029.95	17636.16	18227.5	**18256**	1.0108	0.81159
P62046	Lrch1	Leucine-rich repeat and calponin homology domain-	8914.336	8918.235	9717.316	8663.34	**9053**	9209.81	7296.147	8230.822	12057.52	**9199**	0.9842	0.89500
O08808	Diaph1	Protein diaphanous homolog 1	7581.011	2802.352	8427.771	3561.046	**5593**	6648.689	7606.547		2842.912	**5699**	0.9813	0.96095
P62267	Rps23	Small ribosomal subunit protein uS12	450757.3	417063.9	500452.1	494057.3	**465583**	455448.8	451682.1	532156.8	531494.9	**492696**	0.9450	0.39956
Q6Y7W8	Gigyf2	GRB10-interacting GYF protein 2	25045.46	25220.24	27564.14	25653.21	**25871**	25043.76	19921.45	24885.23	27879.12	**24432**	1.0589	0.44300
Q6WVG3	Kctd12	BTB/POZ domain-containing protein KCTD12	30187.84	30077.82	34685.96	29034.5	**30997**	29590.59	30887.89	24905.15	27055.48	**28110**	1.1027	0.16605
Q5XG69	Fam169a	Soluble lamin-associated protein of 75 kDa	27175.21	31005.53	21849.53	21998.6	**25507**	29987.2	28214.85	23902.86	25155.63	**26815**	0.9512	0.63457
Q9ESE1	Lrba	Lipopolysaccharide-responsive and beige-like ancho	8952.186	10441.19	7146.64	11315.63	**9464**	9010.961	6072.457	7299.396	8939.313	**7831**	1.2086	0.20708
Q921C1	Gjc3	Gap junction gamma-3 protein	48572.27	54904.71	62161.38	62434.87	**57018**	52716.22	58526.45	65019.84	69793.09	**61514**	0.9269	0.40231
Q4V9Z5	Sez6l2	Seizure 6-like protein 2	11476.29	12105.04	14720.85	13462.16	**12941**	11172.12	13771.19	11232.9	14599.23	**12694**	1.0195	0.83513
Q3TLP5	Echdc2	Enoyl-CoA hydratase domain-containing protein 2, m	5287.776	13399.71	14462.07	4028.912	**9295**	11941.62	8042.663			**9992**	0.9302	0.87671
P98191	Cds1	Phosphatidate cytidylyltransferase 1	14498.55	14488.87	14182.27	14264.43	**14359**	17202.19	18711.2	14240.64	15176.04	**16333**	0.8791	0.09805
B9EJA2	Cttnbp2	Cortactin-binding protein 2	49927.09	6497.562	7276.421		**21234**							
Q9JJC6	Rilpl1	RILP-like protein 1	11633.31	13616.87	16172.17	12851.78	**13569**	14839.24	12113.38	15775.7	11580.77	**13577**	0.9994	0.99523
Q6P5G6	Ubxn7	UBX domain-containing protein 7	24612.21	13481.66	14301.76	11202.3	**15899**	17973.35	15366.13	16924.36	13008.58	**15818**	1.0051	0.98033
Q60710	Samhd1	Deoxynucleoside triphosphate triphosphohydrolase	60620.33	41653	38558.72	35217	**44012**	38722	38660.2	34217.8	33630.1	**36308**	1.2122	0.23625
Q8BS40	Cptp	Ceramide-1-phosphate transfer protein	11844.78	13515.13	8547.405		**11302**	15833.2	8237.445			**12035**	0.9391	0.84318
Q8BH82	Napepld	N-acyl-phosphatidylethanolamine-hydrolyzing phosp	7710.194	10801.15	9884.325	11410.43	**9952**	9685.354	9356.603	5684.466	7941.052	**8167**	1.2185	0.19332
Q8BK63	Csnk1a1	Casein kinase I isoform alpha	42340.25	41340.35	45276.7	49266.2	**44556**	43766.05	41843.36	43237.79	47342.04	**44047**	1.0115	0.81917
Q9JI78	Ngly1	Peptide-N(4)-(N-acetyl-beta-glucosaminyl)asparagi	34835.13	28451.99	31552.17	28635.25	**30869**	34196.05	27341.36	28399.74	30133.65	**30018**	1.0283	0.70294
Q9Z2I0	Letm1	Mitochondrial proton/calcium exchanger protein	64738.67	67712.91	71383.73	77020.57	**70214**	70134.6	77607.41	72754.91	74524.66	**73755**	0.9520	0.29328
Q8BHK1	Nipa1	Magnesium transporter NIPA1	51203.91	35969.84	32531.47	35480.83	**38797**	43635.51	49196.6	49676.02	46050.91	**47140**	0.8230	0.10914
Q9CPQ8	Atp5mg	ATP synthase subunit g, mitochondrial	456954.4	422185.8	469779.3	475929.2	**456212**	444457.4	394299.7	496257.5	469584.4	**451150**	1.0112	0.84502
Q61599	Arhgdib	Rho GDP-dissociation inhibitor 2	48486.21	34452.32	35878.46	36568.94	**38846**	31021.17	25717.99	26898.47	17439.66	**25269**	1.5373	0.01991
O08547	Sec22b	Vesicle-trafficking protein SEC22b	189086.8	178891.6	203684.3	215455.1	**196779**	196371.7	194039.5	191433	226946.7	**202198**	0.9732	0.65591
Q5XPI3	Rnf123	E3 ubiquitin-protein ligase RNF123	16377.86	11712.46	10461.12	13325.96	**12969**	13260.89	14858.61	10427.55	12821.55	**12842**	1.0099	0.93818
O88545	Cops6	COP9 signalosome complex subunit 6	66398.15	80468.94	67133.63	66833.7	**70209**	66746.02	72257.78	70804.61	67194.31	**69251**	1.0138	0.80338
Q07417	Acads	Short-chain specific acyl-CoA dehydrogenase, mitoc	75109.89	74028.88	76404.3	83667.77	**77303**	69635.69	75221.71	77266.69	76684.81	**74702**	1.0348	0.38699
Q9WTQ5	Akap12	A-kinase anchor protein 12	211815.5	227140.1	238591.9	242041.8	**229897**	235391.8	217119.5	246636.2	236968.8	**234029**	0.9823	0.66872
Q3TJZ6	Fam98a	Protein FAM98A	60638.29	60422.05	66840.96	70362.83	**64566**	60298.88	64427.18	59599.74	70880.64	**63802**	1.0120	0.83695
Q7TSC1	Prrc2a	Protein PRRC2A	21740.18	20806.11	21941.24	28052.12	**23135**	22380.74	22653.67	25609.76	33665.36	**26077**	0.8872	0.38078
Q61704	Itih3	Inter-alpha-trypsin inhibitor heavy chain H3	83222.2	55954.37	48197.36	79252.2	**66657**	61609.07	55768.39	47214.1	50631.97	**53806**	1.2388	0.21017
Q9Z0J0	Npc2	NPC intracellular cholesterol transporter 2	53924.7	56383.3	67778.64	54176.73	**58066**	56337.57	52485.13	72658.12	77317.73	**64700**	0.8975	0.37339
P48024	Eif1	Eukaryotic translation initiation factor 1	17174.94	24973.92	15883.34	15293.46	**18331**	23318.28	21063.96	21859.88	18197.93	**21110**	0.8684	0.30775
Q9CQM9	Glrx3	Glutaredoxin-3	200187.3	213902.5	174260.3	152644.1	**185249**	195091.2	183470.6	162888.8	146620.1	**172018**	1.0769	0.47506

A2AT37	Upf2	Regulator of nonsense transcripts 2	10865.5	10951.64	8582.336	8428.585	9707	10266.5	9895.525	3868.815	4172.259	7051	1.3767	0.20843
Q9R0H0	Acox1	Peroxisomal acyl-coenzyme A oxidase 1	48813.41	49913.47	61161.88	50918.38	52702	48871.05	55511.06	59685.87	59606.44	55919	0.9425	0.43222
P70663	Sparcl1	SPARC-like protein 1	9188.635	10064.48	7600.405	10229.06	9271	8236.602	6653.86		7941.08	7611	1.2181	0.09875
Q78HU3	Mvb12a	Multivesicular body subunit 12A	16482.11	15156.07	9927.28	9300.875	12717	17060.28	13049.54	14917.75	11179.62	14052	0.9050	0.56793
O70435	Psma3	Proteasome subunit alpha type-3	101738.4	102398.5	94445.51	84654	95809	99648.47	112674.2	100069.8	91413.49	100951	0.9491	0.42620
Q8BU03	Pwp2	Periodic tryptophan protein 2 homolog	1096341	1180418	1385601	1377394	1259939	1109920	1023298	1272515	913375.7	1079777	1.1669	0.13617
O55013	Trappc3	Trafficking protein particle complex subunit 3	57068.36	42477.93	29722.71	35453.14	41181	30931.4	40458.09	32530.18	36721.45	35160	1.1712	0.37487
Q9CR57	Rpl14	Large ribosomal subunit protein eL14	774326.4	749296.4	840680.7	902995.8	816825	831370.8	830644.2	916577	972878.3	887868	0.9200	0.19773
Q80XK6	Atg2b	Autophagy-related protein 2 homolog B	10260.76	9681.704	8584.29	9694.544	9555	12046.15	9911.409	6559.161	7420.09	8984	1.0636	0.67400
A2A699	Fam171a2	Protein FAM171A2		63581.7			63582	29814.11				29814	2.1326	
Q3TH73	Ttyh2	Protein tweety homolog 2												
Q6PB44	Ptpn23	Tyrosine-protein phosphatase non-receptor type 23	40861.91	42232.5	40567.81	39730.63	40848	41414.93	43085.4	36532.11	41346.21	40595	1.0062	0.87177
Q61425	Hadh	Hydroxyacyl-coenzyme A dehydrogenase, mitochond	132244.1	118827.1	135221.6	152315.8	134652	140691.4	122084.3	150287.2	149531.7	140649	0.9574	0.55154
Q3UHL1	Camkv	CaM kinase-like vesicle-associated protein						33548.46				33548	0.0000	
Q9WUU7	Ctsz	Cathepsin Z	37371.07	37096.55	38978.35	27393.29	35210	35427.89	39221.89	36434.12	34255.61	36335	0.9690	0.70603
Q9DCP2	Slc38a3	Sodium-coupled neutral amino acid transporter 3												
Q8VDP3	Mical1	[F-actin]-monooxygenase MICAL1	90832.89	82280.95	89054	73606.9	83944	82799.52	82498.64	92766.45	89113.02	86794	0.9672	0.56173
P97930	Dtymk	Thymidylate kinase	19516.07	17796.71	18029.17	21814.55	19289	20572.46	22894.13	14893.49	18749.93	19278	1.0006	0.99538
Q6P1F6	Ppp2r2a	Serine/threonine-protein phosphatase 2A 55 kDa reg	82021.09	82235.69	74511.87	72035.31	77701	76797.66	82153.79	79061.62	74738.08	78188	0.9938	0.87853
P28663	Napb	Beta-soluble NSF attachment protein	100826.4	103540.3	98761.43	111169.2	103574	101946.6	100772.5	101765.7	101007.8	101373	1.0217	0.45070
Q9Z204	Hnrnpc	Heterogeneous nuclear ribonucleoproteins C1/C2	470463.3	500478.8	545643.5	589139.8	526431	516387.1	505668.2	564917.2	600891.6	546966	0.9625	0.56942
Q99LC3	Ndufa10	NADH dehydrogenase [ubiquinone] 1 alpha subcom	224048.2	233592.8	261769.5	251911.2	242830	236617.3	235822.8	257577.4	278170.1	252047	0.9634	0.51144
Q80UM7	Mogs	Mannosyl-oligosaccharide glucosidase	81609.77	79389.9	89877.41	88456.8	84833	84029.41	86070.64	94413.01	95764.76	90069	0.9419	0.22781
Q8BTV2	Cpsf7	Cleavage and polyadenylation specificity factor subu	23062.91	23205.07	26044.7	30031.68	25586	26480.11	21339.4	29633.62	32688.52	27535	0.9292	0.52953
Q9CX60	Lbh	Protein LBH	26972.41	24408.62	20105.9	16148.17	21909	23515.73	17194.06	17529.94	13917.99	18039	1.2145	0.26022
Q6PD03	Ppp2r5a	Serine/threonine-protein phosphatase 2A 56 kDa reg	20512.78	24761.19	14972.56	14918.97	18791	19472.74	16494.31	14481.78	16408.17	16714	1.1243	0.45426
Q4VAA7	Snx33	Sorting nexin-33												
Q8C1B7	Septin11	Septin-11	115659.4	116985.3	113137.7	112141.1	114481	101118.4	119010.5	108008.7	101497.2	107409	1.0658	0.15309
P57780	Actn4	Alpha-actinin-4	103861.7	98515.26	102706.7	101183.1	101567	99755.86	103429.5	100725.7	99520.38	100858	1.0070	0.64504
O54990	Prom1	Prominin-1												
Q9R020	Zranb2	Zinc finger Ran-binding domain-containing protein 2			4131.609	3575.77	3854	4237.365		6758.917	4296.717	5098	0.7560	0.33750
Q8BHE3	Atcay	Caytaxin	12230.69	14190.38	16569.31	11826.01	13704	13576.27	20347.02	7010.43	13677.66	13653	1.0038	0.98662
Q99NH2	Pard3	Partitioning defective 3 homolog	12848.62	12593.46	11203.24	12525.97	12293	13363.39	13293.59	7367.224	11126.45	11288	1.0890	0.51521
P49962	Srp9	Signal recognition particle 9 kDa protein	49285.05	56213.74	54200.4	57076.02	54194	50589.63	51271.82	52592.94	52982.98	51859	1.0450	0.24952
P51660	Hsd17b4	Peroxisomal multifunctional enzyme type 2	246602	232571.5	297445.1	278273.6	263723	266454	254683.1	308666.7	342945.2	293187	0.8995	0.28392
Q9D8S3	Arfgap3	ADP-ribosylation factor GTPase-activating protein 3	15676.28	12632.01	11701.75	11134.75	12786	12075.2	12417.67	13107.23	12851.46	12613	1.0137	0.87278
Q9DBG3	Ap2b1	AP-2 complex subunit beta	166835.9	171882.3	177196.2	175903.6	172954	173482.1	176248.7	188040.2	190293.2	182016	0.9502	0.10782
Q8C650	Septin10	Septin-10	24963.99	24761.96	25929.55	18160.12	23454	24904.18	28249.07	25171.52	24464.51	25697	0.9127	0.30060
Q8BIG7	Comtd1	Catechol O-methyltransferase domain-containing pr	21754.82	25368.08	26416.98	26815.14	25089	22856.74	30499.39	23019.71	31939.99	27079	0.9265	0.48426
P32037	Slc2a3	Solute carrier family 2, facilitated glucose transporte	16660.73	27337.05	15268.09	20499.06	19941	19838.11	20116.4	15778.91	13633.05	17342	1.1499	0.43834
Q8VDG5	Ppcs	Phosphopantothenate--cysteine ligase	9992.619	11854.81	9936.323	6415.71	9550	14987.17	13815.67	19196.55		16000	0.5969	0.02000
Q91VT4	Cbr4	3-oxoacyl-[acyl-carrier-protein] reductase	43087.56	48637.45	46023.04	49837.81	46896	43940.11	41101.88	34972.68	46695.07	41677	1.1252	0.12444
Q3U0V1	Khsrp	Far upstream element-binding protein 2	116171.6	120902.1	127199.6	120570.9	121211	116236.2	115170.4	143709.5	139665.5	128695	0.9418	0.37905
A6H630	Armt1	Damage-control phosphatase ARMT1	4033.356	8877.81		4203.911	5705	8266.94		7488.978	6997.65	7585	0.7522	0.31299
Q99LI7	Cstf3	Cleavage stimulation factor subunit 3	36125.48	34701.88	33149.26	44382.04	37090	40940.14	39564.1	58174.87	46193.31	46218	0.8025	0.11292
P99028	Uqcrh	Cytochrome b-c1 complex subunit 6, mitochondrial	10983.54	17109.13	21663.84	16079.41	16459	16111.25	11767.57	9525.695	15181.8	13147	1.2520	0.26115
Q505D7	Opa3	Optic atrophy 3 protein homolog	41959.06	42785	39614.97	44601.55	42240	38324.63	47014.35	41011.28	45639.32	42997	0.9824	0.74985
Q9DAW6	Prpf4	U4/U6 small nuclear ribonucleoprotein Prp4	17395.22	12931.99	17137.67	16503.48	15992	16782.36	16626.58	18810	18033.63	17563	0.9105	0.22469
Q8C050	Rps6ka5	Ribosomal protein S6 kinase alpha-5	13407.71	14608.08	15619.84	14628.62	14566	13471.55	16941.54	14769.1	13828.57	14753	0.9873	0.84278
Q64012	Raly	RNA-binding protein Raly	114067.8	112932.5	136714.6	142943.5	126665	129972	128820.3	147400.3	142553.8	137187	0.9233	0.28590
Q9CPU4	Mgst3	Glutathione S-transferase 3, mitochondrial	46317.28	45353.33	42492.88	46150.5	45078	43182.06	44249.98	50631.04	60217.08	49570	0.9094	0.30559
Q61137	Astn1	Astrotactin-1			652.4746	2275.365	1464	1854.916			1834.366	1845	0.7936	0.68513
Q9CXY9	Pigk	GPI-anchor transamidase	38550.16	34924.89	37370.54	39201.38	37512	34921.79	39267	39328.56	40791.02	38577	0.9724	0.52519
Q6P9R4	Arhgef18	Rho guanine nucleotide exchange factor 18	10316.74	8016.967	5988.581	5137.771	7365	7046.815	10146.08	4054.29	7302.211	7137	1.0319	0.89771
Q8BX70	Vps13c	Intermembrane lipid transfer protein VPS13C	17399.09	14669.55	15194.72	13522.09	15196	15440.62	14839.14	10603.89	14554.95	13860	1.0964	0.36642
Q61176	Arg1	Arginase-1		62033.49	46792.5	65796.98	58208	32645.79	51415.3		46115.82	43392	1.3414	0.13991
Q8BG58	P4htm	Transmembrane prolyl 4-hydroxylase	13645.98	13489.02	14031.63	15506.52	14168	11917.82	15282.07	11919.61	18937.66	14514	0.9762	0.84862
O35593	Psmd14	26S proteasome non-ATPase regulatory subunit 14	115876.7	118751.5	119176.4	109669.4	115869	108832.8	106738.4	136651	121712.5	118484	0.9779	0.73037
Q9CWF2	Tubb2b	Tubulin beta-2B chain	680048.7	724771.8	783292.1	575836.1	690987	689874.9	674037.9	683532.3	669004.5	679112	1.0175	0.79660

Q8QZR5	Gpt	Alanine aminotransferase 1	11948.61	15099.02	10091.83	11256.08	12099	10770.27	10263.06	13784.91	15205.71	12506	0.9674	0.80768
Q99M51	Nck1	Cytoplasmic protein NCK1	16254.58	18487.82	19034.94	16719.19	17624	18525.25	16942.59	16979.42	16983.97	17358	1.0153	0.74354
Q99M08		Uncharacterized protein C4orf3 homolog	31605.28	39430.8	34910.68	43456.42	37351	43308.86	30492.4	55723.44	42883.38	43102	0.8666	0.35706
Q9CRB9	Chchd3	MICOS complex subunit Mic19	101244.1	91708.86	103213.8	103513.7	99920	94972.52	92583.98	101058.7	107164.1	98945	1.0099	0.82784
O35972	Mrpl23	Large ribosomal subunit protein uL23m	7252.962	9191.549	9087.3	11353	9221	10192.06	10519.23	9142.329	11524.54	10345	0.8914	0.29158
Q08331	Calb2	Calretinin	68260.77	72107.2	39544.35	34506.22	53605	61621.82	55757.05	32558.51	33743.74	45920	1.1673	0.55239
P70296	Pebp1	Phosphatidylethanolamine-binding protein 1	676496.6	710911.5	645566.4	525656	639658	636174.6	676469.1	533768.5	527178.3	593398	1.0780	0.43155
Q9CZP5	Bcs1l	Mitochondrial chaperone BCS1	13323.43	11673	14041.41	15748.83	13697	14565.22	12447.8	10343.81	14063.75	12855	1.0655	0.53288
Q5MPP0	Fa2h	Fatty acid 2-hydroxylase	18747.8	19526.13	32016.62	24628.55	23730	17246.3	18311.43	30955.12	34208.44	25180	0.9424	0.79346
Q8BJI1	Slc6a17	Sodium-dependent neutral amino acid transporter SLC6A17		29924.74		27746.6	28836			9811.511		9812	2.9390	
Q60597	Ogdh	2-oxoglutarate dehydrogenase complex component	200445.7	216507	224592.3	232140	218421	218053.6	224618.3	241254.1	242004.6	231483	0.9436	0.19983
Q3UMC0	Afg2a	ATPase family gene 2 protein homolog A	1326891	1137787	1385210	1011988	1215469	1054748	1107656	1196901	857962.6	1054317	1.1528	0.20007
Q6P3A8	Bckdhb	2-oxoisovalerate dehydrogenase subunit beta, mitoc	29067.6	26084.34	29847.43	30127.24	28782	24570.24	27306.54	15514.85	25228.96	23155	1.2430	0.08870
P46061	Rangap1	Ran GTPase-activating protein 1	44355.78	43505.49	41820.8	39973.37	42414	39775.54	46123.06	40948.01	39785.49	41658	1.0181	0.68873
Q91X72	Hpx	Hemopexin	151052.3	99866.08	179387.5	90566.02	130218	82801.5	72297.04	116498.2	87708.16	89826	1.4497	0.13131
Q9D8E6	Rpl4	Large ribosomal subunit protein uL4	568366.5	570585	626346.6	641334.6	601658	605593.6	591345.4	698568.1	696196.2	647926	0.9286	0.22645
Q9WUT3	Rps6ka2	Ribosomal protein S6 kinase alpha-2	29118.25	29768.92	25790.19	24708.88	27347	24650.53	29638.15	30577.82	22531.02	26849	1.0185	0.83611
Q920L1	Fads1	Acyl-CoA (8-3)-desaturase	17205.09	17037.12	19276.02	20780.76	18575	16438.63	19706.51	21435.14	22910.34	20123	0.9231	0.38553
Q8CIP5	Disp2	Protein dispatched homolog 2	6562.895	7104.775	5767.869	6049.356	6371	4930.74	4517.249	3860.068	9728.953	5759	1.1063	0.67154
Q8R104	Sirt3	NAD-dependent protein deacetylase sirtuin-3							8557.673			8558	0.0000	
F8VPK0	Skic3	Superkiller complex protein 3	14756.25	12145.83	16934.37	9942.546	13445	13511.81	16499.69	14329.56	13907.36	14562	0.9233	0.52668
O88602	Cacng2	Voltage-dependent calcium channel gamma-2 subunit												
Q9DB50	Ap1s2	AP-1 complex subunit sigma-2	27430.06	33457.2	34176.25	33934.67	32250	37152.81	34242.63	37823.07	36147.11	36341	0.8874	0.06250
Q8BUV3	Gphn	Gephyrin	49546.82	55269.7	53753.93	50857.71	52357	50227.09	55946.69	48896.16	57449.51	53130	0.9855	0.76544
Q9D4V7	Rabl3	Rab-like protein 3	109527.6	90247.38	105592.5	99039.03	101102	101929	81709.95	142112.1	122007.6	111940	0.9032	0.45774
D3YZU1	Shank1	SH3 and multiple ankyrin repeat domains protein 1												
B1AWN6	Scn2a	Sodium channel protein type 2 subunit alpha	44829.69	45830.3		3944.147	31535	22975.09	9235.001	19038.54	24814.84	19016	1.6583	0.35386
Q922U1	Prpf3	U4/U6 small nuclear ribonucleoprotein Prp3	14826.09	7067.363	8093.394	18905.35	12223	6650.623	16375.85	26588.32	8672.061	14572	0.8388	0.67462
P0DP26;P0DP27;P0DP2	Calm1;Calm2;Calm3	Calmodulin-1;Calmodulin-2;Calmodulin-3	789130.1	889364.3	668775.8	521960	717308	739945.3	809883.4	559013.3	568266.9	669277	1.0718	0.65120
P47757	Capzb	F-actin-capping protein subunit beta	216586.2	217253.3	224081.9	212149	217518	215797.6	205762.9	238386.2		224068	0.9708	0.46129
P56546	Ctbp2	C-terminal-binding protein 2	60770.18	67482.55	73737.59	70936.59	68232	71393.02	57400.02	55208.97	74549.35	64638	1.0556	0.54614
Q9ES00	Ube4b	Ubiquitin conjugation factor E4 B	29479.29	31397.81	28408.41	22984.19	28067	27682	29730.22	30408.18	26475.83	28574	0.9823	0.81028
Q61282	Acan	Aggrecan core protein	20454.03	9124.551	10762.61	7360.833	11926	16957.02	21259.73	8579.423	6871.907	13417	0.8888	0.75160
O88809	Dcx	Neuronal migration protein doublecortin	65026.17	63566.88	57426.11	63028.73	62262	60381.49	67429.91	64181.52	59859.49	62963	0.9889	0.78293
Q8BGQ1	Vipas39	Spermatogenesis-defective protein 39 homolog	15496.68	7139.515	12479.28	4655.815	9943	10819.6	8830.521	6257.187	8199.997	8527	1.1661	0.61113
P70205	Adcyap1r1	Pituitary adenylate cyclase-activating polypeptide type I receptor												
Q3TAS6	Emc10	ER membrane protein complex subunit 10	29639.1	29377.23	38614.86	38293.32	33981	36038.19	34529.46	43299.26	39208.73	38269	0.8880	0.23273
Q01147	Creb1	Cyclic AMP-responsive element-binding protein 1	32453.08	20272.51	31432.4	32051.63	29052	36144.63	28806.83	37162.79	39091.08	35301	0.8230	0.14195
O55074;Q7TN79	Akap7	A-kinase anchor protein 7 isoform alpha;A-kinase anchor protein 7 isoform gamma												
P12815	Pdcd6	Programmed cell death protein 6	69336.55	79320.07	57183.39	60111.57	66488	65754.46	69989.41	60540.45	56679.36	63241	1.0513	0.59521
P28650	Adss1	Adenylosuccinate synthetase isozyme 1	25506.56	19943.83	18618.64	17472.74	20385	14155.81	15482.19	25399.45	3504.479	14635	1.3929	0.27788
Q8K4P8	Hecw1	E3 ubiquitin-protein ligase HECW1	17012.29	15688.17	14628.19	16453.59	15946	16733.33	16920.55	14639.4	19177.09	16868	0.9453	0.41838
Q9CZD3	Gars1	Glycine--tRNA ligase	330019	354874.4	301780.3	276002.2	315669	318458.2	326946.3	308352.8	276683.2	307610	1.0262	0.70547
P08551	Nefl	Neurofilament light polypeptide	16707021	14971325	10886747	16680189	14811321	14770547	16116381	9713696	10856062	12864172	1.1514	0.37999
Q61831	Mapk10	Mitogen-activated protein kinase 10	43740.88	46620.2	44476.48	41283.79	44030	46040.59	43701.08	41461.51	42008.16	43303	1.0168	0.64639
Q3U7R1	Esyt1	Extended synaptotagmin-1	350094.2	381092.8	387149	443543.2	390470	368128.8	398570	390356.6	395564.3	388155	1.0060	0.91438
Q9CQM5	Txndc17	Thioredoxin domain-containing protein 17	132076.8	141910.8	151931.8	95499.3	130355	120909.5	118799.2	110472.5	103096.4	113319	1.1503	0.23688
Q8C2E7	Washc5	WASH complex subunit 5	34324.89	37626.1	35096.93	32206.38	34814	41029.51	38085.62	31184.3	34868.35	36292	0.9593	0.55965
Q8VDR9	Dock6	Dedicator of cytokinesis protein 6	7692.039	5502.642	4578.366	4898.503	5668	7027.337	4667.097	4115.473	5009.429	5205	1.0890	0.64188
P60335	Pcbp1	Poly(rC)-binding protein 1	398353.5	440456.2	447554.8	372799.2	414791	436123.4	404024.4	395835.5	377215.5	403300	1.0285	0.61325
Q9DC70	Ndufs7	NADH dehydrogenase [ubiquinone] iron-sulfur prote	52076.99	52581.31	63934.38	57757.53	56588	51663.57	50640.5	76503.62	53936.4	58186	0.9725	0.82037
Q6NXM2	Rcbtb1	RCC1 and BTB domain-containing protein 1	7175.101	7650.787	6506.026	4026.457	6340		8102.703			8103	0.7824	
Q8C0Z1	Fam234a	Protein FAM234A	61512.74	52149.43	48743.11	45425.84	51958	43263.82	36790.52	52586.33	49808.8	45612	1.1391	0.24710
Q8R480	Nup85	Nuclear pore complex protein Nup85	63932.79	75058.9	72923.48	63450.63	68841	67191.92	74962.02	57330.57	70169.84	67414	1.0212	0.77546
Q91XI1	Dus3l	tRNA-dihydrouridine(47) synthase [NAD(P)(+)]-like	12412.57	14278.2	16807.84	15615.11	14778	11893.44	15267.49	15354.58	14939.21	14364	1.0289	0.75226
P09671	Sod2	Superoxide dismutase [Mn], mitochondrial	261059.9	251373.2	250350.7	254665.4	254362	240317.1	272916.8	238950.3	258814.2	252750	1.0064	0.85507
Q9DCH4	Eif3f	Eukaryotic translation initiation factor 3 subunit F	78706.09	75138.04	80794.76	77837.79	78119	78616.74	79120.52	90809.66	87071.77	83905	0.9310	0.12325
P52623	Uck1	Uridine-cytidine kinase 1	28333.36	29229.05	26894.37	26220.38	27669	26379.26	24399.86	17207.41	23873.92	22965	1.2048	0.06701
Q3V4B5	Commd6	COMM domain-containing protein 6	11975.69	7892.265	9526.101	8271.547	9416	13342.52	12196.58	13377.47	11939.22	12714	0.7406	0.01617

Q60737	Csnk2a1	Casein kinase II subunit alpha	54014.42	49581.73	49132.8	49030.61	50440	49280.97	50648.35	55916.87	47571.32	50854	0.9918	0.85434
Q9WTX2	Prkra	Interferon-inducible double-stranded RNA-depender	83935.71	74511.91	78229.16	92980.69	82414	74832.72	87666.35	91384.26	94535.13	87105	0.9462	0.45726
Q80X71	Tmem106b	Transmembrane protein 106B	37003.76	41392.59	37365.64	44904.8	40167	34514.43	38652.98	35406.97	47915.86	39123	1.0267	0.78078
Q60936	Coq8a	Atypical kinase COQ8A, mitochondrial	20312.02	20148.98	16892.48	23451.4	20201	24084.66	21834.33	14946.3	22183.31	20762	0.9730	0.82352
Q3UUJ4	Strada	STE20-related kinase adapter protein alpha	8961.082	10409.7	11896.99	7659.1	9732	15810.3	5164.813	10241.32	7065.672	9571	1.0168	0.95072
Q9Z2D8	Mbd3	Methyl-CpG-binding domain protein 3	7949.528	9253.627	8879.291	7658.207	8435	5689.642	7038.349	5824.535	7320.723	6468	1.3041	0.01275
Q8C0I1	Agps	Alkyldihydroxyacetonephosphate synthase, peroxisc	44749.28	39424.05	50367.3	51449.49	46498	43458.3	45754.75	48514.44	56298.96	48507	0.9586	0.62839
Q8R4G0	Ntng1	Netrin-G1	10367.67	7875.359	10189.19	10660.47	9773	11455.27	11319.54	5925.189	9246.033	9487	1.0302	0.84881
Q9JKV1	Adrm1	Proteasomal ubiquitin receptor ADRM1	80251.63	89788.45	82037.41	78426.86	82626	74502.97	85217.29	68757.7	70454.79	74733	1.1056	0.12730
Q8C522	Endod1	Endonuclease domain-containing 1 protein	272428.4	286571.8	282232.1	324544.8	291444	248126.5	285049	268618.8	297466.8	274815	1.0605	0.32851
Q6ZQ29	Taok2	Serine/threonine-protein kinase TAO2	11320.6	15203.56	10523.66	16801.3	13462	12630.14	15680.55	16372.69	20770.71	16364	0.8227	0.24637
P40240	Cd9	CD9 antigen	3429733	2721325	1884545	3001840	2759361	2608981	3266401	1628296	2008897	2378144	1.1603	0.46124
Q91XD6	Vps36	Vacuolar protein-sorting-associated protein 36	18769.77	21006.43	20823.01	18030.02	19657	19339.09	18328.12	21119.67	21558.72	20086	0.9786	0.69983
Q91XL9	Osbpl1a	Oxysterol-binding protein-related protein 1	71547.05	77547.88	66585.73	57246.66	68232	71613.12	64286.49	61093.59	69941.73	66734	1.0224	0.77202
Q9CPU0	Glo1	Lactoylglutathione lyase	169883.1	187719.7	197207.5	145900.7	175178	181364.4	170497.9	147665.3	141908.9	160359	1.0924	0.35063
Q3UPH1	Prrc1	Protein PRRC1	56762.37	58528.16	48178.39	39443.45	50728	43668.02	41755.59	53400.95	45955.04	46195	1.0981	0.40620
P51569	Gla	Alpha-galactosidase A			9015.587		9016	12905.63		10874.53	5797.293	9859	0.9144	
P62743	Ap2s1	AP-2 complex subunit sigma	81049.67	79481.88	88807.16	85221.98	83640	80993.67	94511.56	80646.92	79734.84	83972	0.9961	0.93824
Q0KL02	Trio	Triple functional domain protein	16319.81	17974.43	17946.89	16627.26	17217	18056.79	17150.87	14333.06	17443.84	16746	1.0281	0.63179
Q99ME2	Wdr6	tRNA (34-2'-O)-methyltransferase regulator WDR6	23024.99	23212.45	37623.39	23230.33	26773	38042.56	23101.61	30080.91	30402.6	30407	0.8805	0.47169
P17710	Hk1	Hexokinase-1	715925.9	726885.6	743650.6	705100.8	722891	748904.4	756493	731311.8	714509	737805	0.9798	0.27719
Q8K4Z0	Lgi2	Leucine-rich repeat LGI family member 2	12412.44	11398.19	8903.334	12365.86	11270	9631.133	10865.24	8359.183	7940.683	9199	1.2251	0.09749
Q9QYF9	Ndrg3	Protein NDRG3	48133.08	50794.91	54520.33	42202.8	48913	49803.54	50054.12	57332.12	50759.92	51987	0.9409	0.36696
Q99MI1	Erc1	ELKS/Rab6-interacting/CAST family member 1	27919.54	24153.85	31844.93	30017.48	28484	24073.3	25993.19	28749.47	26200.65	26254	1.0849	0.28729
Q99K23	Ufsp2	Ufm1-specific protease 2	39862.96	37062.43	39273.91	39862.62	39015	37792.63	42725.25	40428.35	40228.91	40294	0.9683	0.33064
Q9D2R0	Aacs	Acetoacetyl-CoA synthetase	64652.62	62306.77	124123.3	58152.79	77309	71913.66	57763.41	109464.8	113478.9	88155	0.8770	0.62187
Q9DCE9	Igtp	Immunity-related GTPase family M protein 3	31689.7	15979.89	14466.72	14862.75	19250	15853.23	16263.67	9794.943	14422.59	14084	1.3668	0.28637
Q8BHZ0	Cyria	CYFIP-related Rac1 interactor A	14613.12	15584	13907.34	8060.808	13041	11991.13	14359.5	8470.088	8324.693	10786	1.2091	0.35266
Q7TPM9	Mtmr10	Myotubularin-related protein 10	20857.38	16104.04	21562.64	21424.58	19987	22007.34	21738.87	23215.71	22739.64	22425	0.8913	0.12012
Q99LC5	Etfa	Electron transfer flavoprotein subunit alpha, mitoch	522873.1	499524.6	552670.3	594691.8	542440	534547	531751.9	562820.1	548149.9	544317	0.9966	0.93399
Q8BRN9	Cc2d1b	Coiled-coil and C2 domain-containing protein 1B	22582.06	20977.66	24867.58	22667.17	22774	22417.53	20382.79	20597.2	25856.43	22313	1.0206	0.76896
Q9CWX2	Ndufaf1	Complex I intermediate-associated protein 30, mitoc	29190.58	26918.27	25952.55	33354.95	28854	19217.4	35187.48	15525.32	21499.24	22857	1.2624	0.23973
Q9JLV5	Cul3	Cullin-3	53280.95	54491.47	54203.75	49970.21	52987	53403.37	48824.91	44581.13	52209.56	49755	1.0650	0.19817
Q63932	Map2k2	Dual specificity mitogen-activated protein kinase kin	41283.53	37198.05	29937.2	20685.16	32276	31331.57	32467.28	25131.11	34610.14	30885	1.0450	0.78844
Q9D394	Rufy3	Protein RUFY3	559493.1	574298.4	503799.2	491762.9	532338	543705	553656.2	502844.8	478499.7	519676	1.0244	0.65423
Q8VD04	Gripap1	GRIP1-associated protein 1	55277.78	51390.21	51328.16	46628.97	51156	51108.98	57097.18	47866.08	48072.2	51036	1.0024	0.96700
Q62086	Pon2	Serum paraoxonase/arylesterase 2	20382.45	19864.4	24982.96	23474.94	22176	18378.94	20390.96	16159.02	22377.5	19327	1.1474	0.16732
O55106	Strn	Striatin	48983.82	50248.01	50784.62	54355.02	51093	48713.55	47121.94	57550.28	54221.49	51902	0.9844	0.77297
Q9CYT6	Cap2	Adenylyl cyclase-associated protein 2	21127.31	22267.66	16860.3	24947.99	21301	20314.79	21059.89	13768.01	15151.4	17574	1.2121	0.18405
Q9JKP5	Mbnl1	Muscleblind-like protein 1	111218.1	110476	115298.6	108164	111289	116125.5	115233.6	119276.2	123628.6	118566	0.9386	0.02342
P70362	Ufd1	Ubiquitin recognition factor in ER-associated degrad	140594.9	133810.6	140450.6	142091.4	139237	136170.2	138069.4	141745.5	147987.2	140993	0.9875	0.60196
Q8QZY6	Tspan14	Tetraspanin-14	9323.895	11263.31	9287.47	7844.97	9430	9706.941	11482.75	10764.93	7277.245	9808	0.9615	0.75478
Q61147	Cp	Ceruloplasmin	358665.2	309550.2	277350.3	299679.6	311311	305934.2	325328.1	246972.5	260697.8	284733	1.0933	0.33260
Q61554	Fbn1	Fibrillin-1	179603.5	168776.5	135723.4	196397.2	170125	160534.4	205051.3	145335.9	144470	163848	1.0383	0.75398
Q9DCJ5	Ndufa8	NADH dehydrogenase [ubiquinone] 1 alpha subcomp	150219.6	147645.5	169904.2	188715.1	164121	145041.8	168587.3	173805.9	184901.2	168084	0.9764	0.76639
Q9ESW4	Agk	Acylglycerol kinase, mitochondrial	90217.48	93286.33	113653.8	108185.7	101336	94469.55	100615.3	109558	118128.5	105693	0.9588	0.59123
Q80VC9	Camsap3	Calmodulin-regulated spectrin-associated protein 3	6851.76	6567.051	5141.131	4180.842	5685	6337.787	5237.614	4904.881	6149.841	5658	1.0049	0.97042
P81117	Nucb2	Nucleobindin-2	50404.79	40260.45	56913.08	44278.75	47964	39270.05	44714.7	35774.52	40471.21	40058	1.1974	0.10076
Q8CH72	Trim32	E3 ubiquitin-protein ligase TRIM32	11569.56	13159.58	15527.9	11748.8	13001	12530.81	12774.86	13185.57	13044.77	12884	1.0091	0.90317
P14685	Psmd3	26S proteasome non-ATPase regulatory subunit 3	106446.7	109191	112516.3	107176.9	108833	108646.6	108998.6	120895.8	113488.9	113007	0.9631	0.23435
Q9WVT6	Ca14	Carbonic anhydrase 14	17831.94	11452.59	12957.33	17507.17	14937	11005.75	17990.51	16670.96	14532.43	15050	0.9925	0.96111
P83510	Tnik	Traf2 and NCK-interacting protein kinase	13424.49	12022.01	9179.626	15348.05	12494	11700.52	12746.18	10171.54	14276.66	12224	1.0221	0.86834
Q9EQF6	Dpysl5	Dihydropyrimidinase-related protein 5	196754.2	187475.9	160727.9	189645.3	183651	197385.3	179890.4	184713.2	176437.6	184607	0.9948	0.92002
Q9CXJ4	Abcb8	Mitochondrial potassium channel ATP-binding subur	12237.75	15173.27	15972.83	16858.75	15061	12320.15	14201.17	11503.22	15760.34	13446	1.1201	0.28796
P62869	Elob	Elongin-B	99614.37	100488.2	96182.7	91530.51	96954	102945.7	92406.19	67540.63	89348.71	88060	1.1010	0.29239
Q3U2A8	Vars2	Valine--tRNA ligase, mitochondrial	16252.41			10901.79	13577		5219.021	11634.36	10913.52	9256	1.4669	0.28068
Q60715	P4ha1	Prolyl 4-hydroxylase subunit alpha-1	16306.84	19085.58	31411.03	22280.3	22271	19509.16	17176.29	29723.03	31471.32	24470	0.9101	0.66696
P63080	Gabrb3	Gamma-aminobutyric acid receptor subunit beta-3												
P46978	Stt3a	Dolichyl-diphosphooligosaccharide--protein glycosy	54830.05	59539.64	59047.61	57061.47	57620	56564.8	72596.8	70579.35	70833.03	67643	0.8518	0.04127

Q63810	Ppp3r1	Calcineurin subunit B type 1	7798.588	8945.993	7923.974	6191.333	7715	7486.709	9041.531	5228.67	5783.563	6885	1.1205	0.45329
Q8JZL3	Thtpa	Thiamine-triphosphatase	19024.99	22481.46	19980.37	11743.7	18308	14704.47	18569.32	20599.86	13776.25	16912	1.0825	0.63741
O35682	Myadm	Myeloid-associated differentiation marker	83805.06	75089.76	72385.04	97079.48	82090	79536.43	93387.55	70500.86	66758.5	77546	1.0586	0.59612
Q9JIF0	Prmt1	Protein arginine N-methyltransferase 1	89312.69	94424.99	106066.8	89442.44	94812	100187.2	93581.24	81834.63	88935.43	91135	1.0403	0.52982
O54774	Ap3d1	AP-3 complex subunit delta-1	77742.66	85158.38	78593.88	70215.75	77928	83465.64	82152.2	88480.56	81469.88	83892	0.9289	0.13405
O54941	Smarce1	SWI/SNF-related matrix-associated actin-dependent	23421.44	26166.15	27764.29	27608.63	26240	25585.63	26332.86	24070.65	24788.59	25194	1.0415	0.38607
C0HK79;C0HK80	Arxes1;Arxes2	Adipocyte-related X-chromosome expressed sequer	25661.52	12702.78	28298.8	14177.43	20210	28118.16	29462.94	24999.74	16243.63	24706	0.8180	0.39862
Q9QZ23	Nfu1	NFU1 iron-sulfur cluster scaffold homolog, mitochor	49205.91	41808.33	49846.51	57129.75	49498	47547.08	52606.77	41753.98	55931.02	49460	1.0008	0.99341
P61079	Ube2d3	Ubiquitin-conjugating enzyme E2 D3	102252.9	118980.1	83088.88	101717.9	101510	104722.2	116492.6	104814.9	101813.1	106961	0.9490	0.52214
Q3UH60	Dip2b	Disco-interacting protein 2 homolog B	34052.18	34648.58	35052.46	33853.4	34402	35652.54	34358.89	34681.35	38263.28	35739	0.9626	0.19917
Q8K0T0	Rtn1	Reticulon-1	197894.4	183682.6	181203	193160	188985	175949.1	195201.6	168932	175487.2	178892	1.0564	0.19375
Q4KML4	Abracl	Costars family protein ABRACL	44418.63	32940.95	41997.39	26336.4	36423	27493.54	28648.41	34782.87	30472.45	30349	1.2001	0.22290
Q80TA1	Selenoi	Ethanolaminephosphotransferase 1	24932.44	26375.8	28843.13	20514.44	25166	29939.61	31606.02	34831.16	33776.68	32538	0.7734	0.01174
P99027	Rplp2	Large ribosomal subunit protein P2	515624	502446.8	441966.3	446564.3	476650	467458.7	553854.5	489329.4	446153.5	489199	0.9743	0.69027
Q9QXK7	Cpsf3	Cleavage and polyadenylation specificity factor subu	48125.16	33635.6	47197.13	38650.76	41902	40816.61	39557.7	46971.67	40272.63	41905	0.9999	0.99951
Q60829	Ppp1r1b	Protein phosphatase 1 regulatory subunit 1B	26100.23	24448.22	19969.37	14991.26	21377	19884.91			20225.68	20055	1.0659	0.74151
O88587	Comt	Catechol O-methyltransferase	58704.71	58599.67	69083.31	65704.33	63023	61020.98	61265.95	67684.59	67901.12	64468	0.9776	0.67173
Q9CQB5	Cisd2	CDGSH iron-sulfur domain-containing protein 2	48622.23	51109.86	53447.45	49999.69	50795	50769.87	61154.52	58011.87	58332.13	57067	0.8901	0.04220
P84089	Erh	Enhancer of rudimentary homolog	152332.3	167937.5	153689.1	181143	163775	142875.3	149435.8	145921.2	170230.3	152116	1.0767	0.25095
Q9Z280	Pld1	Phospholipase D1	3732.663		4375.497	7084.135	5064			9558.114	1792.854	5675	0.8923	0.85995
Q8BKZ9	Pdhx	Pyruvate dehydrogenase protein X component, mitoc	71144.73	87396.41	80152.09	89004.72	81924	87422.33	99089.65	89607.62	95488.93	92902	0.8818	0.06531
P63087	Ppp1cc	Serine/threonine-protein phosphatase PP1-gamma (	113285.6	126355.2	119071.1	110667.1	117345	121142.2	118841.6	123965.6	109414.3	118341	0.9916	0.83904
P63328	Ppp3ca	Protein phosphatase 3 catalytic subunit alpha	288436.3	290690.3	212499.4	261230.4	263214	273139	278019.2	198823.9	229713.5	244924	1.0747	0.51070
Q920I9	Wdr7	WD repeat-containing protein 7	46595.43	42195.29	40500	41649.88	42735	41419.15	43408.39	39022.55	41890.05	41435	1.0314	0.45145
P57746	Atp6v1d	V-type proton ATPase subunit D	85681.16	88311.03	74952.79	80817.66	82441	86432.99	84669.2	79202.3	81807.52	83028	0.9929	0.86630
Q9D1K7	Adissp	Adipose-secreted signaling protein	55788.01	54764.93	51169.81	39080.82	50201	55058.46	46289.34	51233.62	31758.3	46085	1.0893	0.54291
Q9DBX3	Susd2	Sushi domain-containing protein 2	230231	216663.4	127607.1	210258.3	196190	179518.5	254105.2	161237	138586.8	183362	1.0700	0.72009
Q8VCR7	Abhd14b	Putative protein-lysine deacylase ABHD14B	42198.67	37199.7	36440.91	35607.38	37862	33537.46	40236.21	33587.63	28292.8	33914	1.1164	0.21669
A2AHC3	Camsap1	Calmodulin-regulated spectrin-associated protein 1	10790.3	8202.248	9377.035	12364.98	10184	9297.346	11192.66	11799.05	11102.74	10848	0.9388	0.54977
Q8BWZ3	Naa25	N-alpha-acetyltransferase 25, NatB auxiliary subunit	37891.35	36766.64	36461.78	32728.75	35962	35543.56	36312.88	39037.89	38478.8	37343	0.9630	0.36211
Q91VW3	Sh3bgrl3	SH3 domain-binding glutamic acid-rich-like protein 3	344795.2	392615.1	337151.4	293971.6	342133	365233.3	378698	298874.9	296802.8	334902	1.0216	0.81492
O35927	Ctnnd2	Catenin delta-2	12748.47	12596.28	13239.26	11744.72	12582	9446.714	10425.1	9335.041	9241.094	9612	1.3090	0.00037
Q9QYJ3	Dnajb1	DnaJ homolog subfamily B member 1	28770.38	26395.25	28574.97	32660.86	29100	32907.63	34824.61	32644.85	31346.8	32931	0.8837	0.04206
O70400	Pdlim1	PDZ and LIM domain protein 1	31178.18	21502.84	23720.4	23588.03	24997	27374.95	21949.96	30168.29	29418.99	27228	0.9181	0.45889
Q9QYR9	Acot2	Acyl-coenzyme A thioesterase 2, mitochondrial	18266.11	15784.16	16063.57	17746.48	16965	17608.37	14772.9	16170.48	18279	16708	1.0154	0.80406
P40142	Tkt	Transketolase	792957.9	803504.8	1022546	742255.6	840316	816372.3	724199.3	881032.6	870741.9	823087	1.0209	0.81836
Q9CQN1	Trap1	Heat shock protein 75 kDa, mitochondrial	68707.48	75173.91	84326.2	80259.69	77117	82269.7	77757.66	88302.66	85881.98	83553	0.9230	0.16558
Q8R4C2	Rufy2	RUN and FYVE domain-containing protein 2	87512.28	97759.78	64579.29	74323.59	81044	81467.41	93710.22	72309.82	64894.65	78096	1.0378	0.76859
Q8BKH7	Mapkap1	Target of rapamycin complex 2 subunit MAPKAP1	8681.497	7367.193	9869.146	11035.37	9238	8724.562	9785.921	13932.71	9179.8	10406	0.8878	0.44593
Q99JH7	Clstn3	Calsyntenin-3		4521.046			4521		4517.6			4518	1.0008	
Q3THF9	Coq10b	Coenzyme Q-binding protein COQ10 homolog B, mit	4181.184	5041.217	5765.095	8264.792	5813	5229.638	6065.815	4215.449	7076.918	5647	1.0294	0.88162
Q3UFK8	Frmd8	FERM domain-containing protein 8	46132.96	40240.71	45034.93	51754.98	45791	40300.2	45155.34	47412.43	40738.47	43402	1.0551	0.44579
Q61136	Prpf4b	Serine/threonine-protein kinase PRP4 homolog	14341.55	15450.55	16819.46	16544.96	15789	15805.4	15005.35	15195.25	18012.69	16005	0.9865	0.81730
O35623	Bet1	BET1 homolog	10500.16	15383.67	14225.48	13436.16	13386	16216.15	13081.12	9754.076	17658.46	14177	0.9442	0.71191
Q9DB60	Prxl2b	Prostamide/prostaglandin F synthase	26044.85	28115.26	25991.54	21651.96	25451	22155.79	21675.44	23038.85	22841.38	22428	1.1348	0.07340
Q9D6K8	Fundc2	FUN14 domain-containing protein 2	13135.53	12878.31	11299.25	10568.77	11970	10501.6	12545.75	6059.447	8291.385	9350	1.2803	0.13751
P16460	Ass1	Argininosuccinate synthase	120505.9	126828.7	111228.3	97792.5	114089	113235	120517.1	75315.52	82163.45	97808	1.1665	0.25212
Q8C729	Hycc2	Hyccin 2		1286.174			1286	17541.55	9171.063	19604.02		15439	0.0833	
Q8K411	Pitrm1	Presequence protease, mitochondrial	53467.04	50610.02	50585.11	54721.82	52346	53025.88	58546.71	55147.24	52782.69	54876	0.9539	0.18560
Q8JZR6	Slc4a8	Electroneutral sodium bicarbonate exchanger 1	1966.219	1905.125		1814.392	1895		2768.485			2768	0.6846	
Q9D187	Ciao2b	Cytosolic iron-sulfur assembly component 2B	18175.44	20596.05	19189.62	18983.16	19236	17757.68	19002.65	14627.33	14872.04	16565	1.1613	0.06612
Q9QXJ4	Arl10	ADP-ribosylation factor-like protein 10	51078.25	52978.13	41807.4	62979.97	52211	45952.51	55103.55	68489.05	67745.57	59323	0.8801	0.34487
P58021	Tm9sf2	Transmembrane 9 superfamily member 2	131770.3	142718.5	136545.6	122204.8	133310	120853.8	142026.9	127364.8	134952	131299	1.0153	0.76080
Q3UTZ3	Trappc14	Trafficking protein particle complex subunit 14	1988.12	631.2315			1310	3686.868	3454.92	3240.436	1994.885	3094	0.4233	0.06395
Q8K0X8	Fez1	Fasciculation and elongation protein zeta-1				4124.072	4124	7806.531			7295.433	7551	0.5462	
Q8BKY8	Mterf2	Transcription termination factor 2, mitochondrial						2352.7	3763.893	2056.569		2724	0.0000	
Q9CQF4	Mtres1	Mitochondrial transcription rescue factor 1	10176.36	12240.78	11732.19	13740	11972	9415.907	11928.4	9715.948	14618.8	11420	1.0484	0.70887
Q9DB41	Slc25a18	Mitochondrial glutamate carrier 2	135857.4	108776.3	132357.3	120928.6	124480	108312.5	116643.9	134157.7	126799.8	121478	1.0247	0.73154
Q9D7A8	Armc1	Armadillo repeat-containing protein 1	32328.53	32270.38	34213.87	35330.02	33536	29625.56	25829.12	36714.71	38238.23	32602	1.0286	0.76834

Q8BM55	Tmem214	Transmembrane protein 214	7192.479	5422.666	7922.725	6494.521	6758	6171.015	7500.041	8430.522	10217.83	8080	0.8364	0.23564
P45591	Cfl2	Cofilin-2	211340.1	200605.3	212365.3	197842.6	205538	201938.5	184653.5	183471.7	191573.9	190409	1.0795	0.03602
Q3UKJ7	Smu1	WD40 repeat-containing protein SMU1	22735.5	21606.05	21277.92	21603.21	21806	20687.08	21501.89	19971.92	23469.69	21408	1.0186	0.64454
Q99JF8	Psip1	PC4 and SFRS1-interacting protein	47739.94	50293.89	31012.71	32326.42	40343	47400.23	27415.41	27003.86	56137.85	39489	1.0216	0.92653
Q8R086	Suox	Sulfite oxidase, mitochondrial	22750.14	32534.55	29167.39	24386.88	27210	26101.2	24178.79	25733.1	27691.27	25926	1.0495	0.60463
Q7TPV4	Mybbp1a	Myb-binding protein 1A	22499.94	23028.13	24893.98	23539.28	23490	22503.77	22277.12	25694.22	25833.33	24077	0.9756	0.61364
Q8VHQ9	Acot11	Acyl-coenzyme A thioesterase 11	53741.5	47197.12	45627.11	42933.15	47375	45751.08	46139.12	44430.85	46112.87	45608	1.0387	0.47760
Q62288	Spock1	Testican-1	8123.405	10594.71	9694.592	10910.49	9831		10448.63	15486.24	11166.34	12367	0.7949	0.15378
Q80UW2	Fbxo2	F-box only protein 2	326361.2	278646.5	225630.7	295909.9	281637	270566.3	333782.1	197864.3	192605.7	248705	1.1324	0.43722
O55022	Pgrmc1	Membrane-associated progesterone receptor comp	240004.4	218938.2	253730.2	296513.4	252297	251627.2	237620.2	243725.9	271134.6	251027	1.0051	0.94586
Q5NCF2	Trappc1	Trafficking protein particle complex subunit 1	38880.89	35149.13	39617.71	35543.15	37298	39230.83	28607.69	40190.47	45027.75	38264	0.9747	0.79964
Q8BGE6	Atg4b	Cysteine protease ATG4B	25293.88	26743.55	20137.71	20768.02	23236	21313.28	22432.85	14535.03	17136.19	18854	1.2324	0.12534
Q9QZE5	Copg1	Coatomer subunit gamma-1	43790.39	43251.24	46226.12	43104.98	44093	43543.07	43455.73	45176.91	47735.91	44978	0.9803	0.50119
Q8C0L6	Paox	Peroxisomal N(1)-acetyl-spermine/spermidine oxida	23105.81	26601.04	31589.95	13651.68	23737	13343.84	19818.34	19398.31	21738	18575	1.2779	0.26492
Q99JY0	Hadhb	Trifunctional enzyme subunit beta, mitochondrial	752813.2	721852.3	792807.9	894577.6	790513	785837.9	856522.9	787266	843772.6	818350	0.9660	0.53142
Q80SY6	Adal	Adenosine deaminase-like protein				2251.825	2252							
Q00897	Serpina1d	Alpha-1-antitrypsin 1-4	144151.3	179942	181666.2	91071.41	149208	140770.9	109503.4	114985.7	91256.44	114129	1.3074	0.18702
O88271	Cfdp1	Craniofacial development protein 1	16906.44	13831.02	18948.44	17952.87	16910	18308.58	12839.25	15727.21	16689.02	15891	1.0641	0.54673
Q6PGC1	Dhx29	ATP-dependent RNA helicase DHX29	12463.08	21922.57	13659.28	20472.7	17129	20704.27	12901.24	18054.61	13200.88	16215	1.0564	0.77444
Q8BJD1	Itih5	Inter-alpha-trypsin inhibitor heavy chain H5	94367.82	105261.7	146165.5	117815.1	115903	111610.9	114497.8	156923.4	165873.2	137226	0.8446	0.28038
Q8C181	Mbnl2	Muscleblind-like protein 2	63368.12	69215.88	60354.56	63974.64	64228	70032.66	74267.25	96550.69	85923.79	81694	0.7862	0.03160
Q91YN5	Uap1	UDP-N-acetylhexosamine pyrophosphorylase	22954.27	23353.14	22145.88	18092.83	21637	18081.88	19252.42	16773.07	17432.2	17885	1.2098	0.02933
Q02248	Ctnnb1	Catenin beta-1	145864.2	136290.1	138949.7	157942.7	144762	143624.3	147130.8	141946.9	150007.2	145677	0.9937	0.86499
B9EJ80	Pdzd8	PDZ domain-containing protein 8	31483.56	34021.39	34535.39	37213.27	34313	33185.81	36365.6	34394.27	44840.87	37197	0.9225	0.35561
Q9Z2I2	Fkbp1b	Peptidyl-prolyl cis-trans isomerase FKBP1B	54737.24	73213.09	67641.63	58181.33	63443	63927.58	65797.85	65035.11	55355.8	62529	1.0146	0.85785
O88848	Arl6	ADP-ribosylation factor-like protein 6	23492.53	18996.41	21826.37	19527.15	20961	21635.54	21267.7	16361.35	16214.79	18870	1.1108	0.29473
O35685	Nudc	Nuclear migration protein nudC	91378.09	103756.7	94785.34	75790.59	91428	94738.2	87439.07	81122.7	83388.38	86672	1.0549	0.49523
O54901	Cd200	OX-2 membrane glycoprotein	111095.3	113646.4	89446.29	108929.7	105779	98747.54	122452.9	76441.27	96559.05	98550	1.0734	0.53255
P21460	Cst3	Cystatin-C	52461.39	47318.48	49978.02	56209.18	51492	44566.67	51581.29	53637.56	47062.49	49212	1.0463	0.44714
P55096	Abcd3	ATP-binding cassette sub-family D member 3	72985.68	69618.9	87031.34	80011.59	77412	72106.88	80031.88	84224.37	93995.32	82590	0.9373	0.41968
Q99KH8	Stk24	Serine/threonine-protein kinase 24	45427.16	42676.89	44823.26	39244.24	43043	45833.62	41315.63	40557.55	46171.81	43470	0.9902	0.84041
Q9Z0M5	Lipa	Lysosomal acid lipase/cholesteryl ester hydrolase	36670.98	34042.39	33920.38	34017.13	34663	37684.94	38985.4	31837.88	23315.41	32956	1.0518	0.65499
O08553	Dpysl2	Dihydropyrimidinase-related protein 2	2294954	2251740	2171624	2177018	2223834	2293780	2029018	2172592	2136579	2157992	1.0305	0.33090
Q9D883	U2af1	Splicing factor U2AF 35 kDa subunit	73992.02	63460.58	81194.07	75295.17	73485	75519.55	74672.34	73140.05	85090.3	77106	0.9531	0.45908
Q8K4X7	Agpat4	1-acyl-sn-glycerol-3-phosphate acyltransferase delt	23388.44	34563.15	35419.13	37881.46	32813	32804.29	34096.41	32799.98	36796.97	34124	0.9616	0.70933
Q9CQ56	Use1	Vesicle transport protein USE1	20570.41	20747.82	20994.15	24563.81	21719	21406.39	21371.38	24149.43	25035.36	22991	0.9447	0.37917
Q8VDM6	Hnrnpul1	Heterogeneous nuclear ribonucleoprotein U-like pro	31513.51	31250.73	34988.48	32552.55	32576	32400.34	31322.06	34028.43	35537.82	33322	0.9776	0.57465
Q922F4	Tubb6	Tubulin beta-6 chain	3456696	3551404	3355746	3552437	3479071	3675903	3458145	3337028	3372414	3460872	1.0053	0.84529
P54310	Lipe	Hormone-sensitive lipase	15625.04	13696.34	13640.08	9182.781	13036	9920.943	13219.93	4034.709	10469.74	9411	1.3851	0.17632
Q99N85	Mrps18a	Large ribosomal subunit protein mL66	12041.43	10628.91	14899.36	8978.922	11637	6481.718	14964.55	11906.38	12617.95	11493	1.0126	0.94951
Q3TFD2	Lpcat1	Lysophosphatidylcholine acyltransferase 1	28289.01	29575.38	36607.05	35379.83	32463	34241.27	33238.03	42771.34	40195.46	37612	0.8631	0.14767
P70441	Nherf1	Na(+)/H(+) exchange regulatory cofactor NHE-RF1	99944.16	111914.5	106231.3	112703.1	107698	105025.7	111327.6	109856.2	111785.9	109499	0.9836	0.60916
P61166	Tmem258	Transmembrane protein 258	58596.4	60913.66	72221.63	72556.77	66072	73387.41	66889.73	84646.28	70137.44	73765	0.8957	0.19927
O88597	Becn1	Beclin-1	14074.65	14940.96	18505.13		15840	17694.87	14528.84	19003.49	26054.29	19320	0.8199	0.31181
Q9R0Q3	Tmed2	Transmembrane emp24 domain-containing protein 2	65133.35	75113.84	62403.99	65503.16	67039	54038.93	72899.71	36006.93	51578.96	53631	1.2500	0.14715
Q9CQC7	Ndufb4	NADH dehydrogenase [ubiquinone] 1 beta subcomp	123626.5	118655.5	148155.3	165423.7	138965	145691	128862.8	173664.4	166665.2	153721	0.9040	0.36159
A2A690	Tanc2	Protein TANC2	214145.8	212488.9	240155.4	227694.8	223621	216379	214381.5	230909.4	248145.3	227454	0.9832	0.71884
Q9D0J4	Arl2	ADP-ribosylation factor-like protein 2	64267.22	52713.26	52581.71	45928.63	53873	51524.05	50628.36	60503.21	46203.83	52215	1.0318	0.74402
E9Q137	Tex264	Testis-expressed protein 264 homolog	40711.63	41540.93	45029.05	42203.85	42371	40509.3	41897.81	40592.46	44780.16	41945	1.0102	0.76585
Q9CZ30	Ola1	Obg-like ATPase 1	185019.3	181490.3	168636.8	134559.9	167427	182222.5	168211.4	175285.3	164439.3	172540	0.9704	0.68880
O35136	Ncam2	Neural cell adhesion molecule 2	21300.4	22377.66	23585.92	26739.81	23501	20203.37	28878.12	25780.49	27829.27	25673	0.9154	0.37425
P08228	Sod1	Superoxide dismutase [Cu-Zn]	763722.8	783878.9	648567.7	617466.2	703409	741893.9	766800.9	649460.3	549282.7	676859	1.0392	0.69472
Q921G7	Etfdh	Electron transfer flavoprotein-ubiquinone oxidoredu	68604.23	69413.76	68789.88	82256.33	72266	69058.56	77573.37	72374.06	69203.66	72052	1.0030	0.95793
Q9Z0R4	Itsn1	Intersectin-1	13940.82	15734.89	18393.86	15808.65	15970	14204.66	15970.74	15306.3	17864.53	15837	1.0084	0.91504
P47911	Rpl6	Large ribosomal subunit protein eL6	896331	895552.4	972989.4	1016518	945348	953536.6	989231.8	1119139	1107753	1042415	0.9069	0.10740
Q9EPU5	Tnfrsf21	Tumor necrosis factor receptor superfamily member 21						19438.9				19439	0.0000	
P61082	Ube2m	NEDD8-conjugating enzyme Ubc12	126501.4	148274.6	118755.6	113079.1	126653	140937	139935.7	127602.2	125591	133516	0.9486	0.46016
Q02788	Col6a2	Collagen alpha-2(VI) chain	1896968	1847949	1374024	1650714	1692414	1692897	1806750	1139622	1258199	1474367	1.1479	0.32028
Q8BMG7	Rab3gap2	Rab3 GTPase-activating protein non-catalytic subuni	43571.42	41002.61	38634	43161.46	41592	41002.09	40414.07	40512.56	41402.54	40833	1.0186	0.53642

Q8VD75	Hip1	Huntingtin-interacting protein 1	20513.06	22337.12	23417.27	24454.58	**22681**	22710.77	22432.14	23011.21	25833.29	**23497**	**0.9653**	**0.50549**
Q7TN29	Smap2	Stromal membrane-associated protein 2	54655.19	53839.29	53782.22	49946.29	**53056**	53559.02	47366.29	55518.28	49765.09	**51552**	**1.0292**	**0.50444**
Q9EQ80	Nif3l1	NIF3-like protein 1	66753.17	74151.51	65195.9	59419.87	**66380**	71502.25	64090.57	64214.94	66962.69	**66693**	**0.9953**	**0.93164**
O70493	Snx12	Sorting nexin-12	129784.3	138374.6	127059.1	111343.4	**126640**	137777.6	123175.3	95805.89	110464.3	**116806**	**1.0842**	**0.38849**
P60764	Rac3	Ras-related C3 botulinum toxin substrate 3	41835.71	29990.88	46916.26	28083.66	**36707**	40061.91	40503.89	23122.58	36185.51	**34968**	**1.0497**	**0.78570**
Q7TSY6	Celf4	CUGBP Elav-like family member 4	5005.477	4474.439	4305.354	7274.116	**5265**	5181.978	4000.052	3148.304	4772.238	**4276**	**1.2314**	**0.27298**
Q9D4F8	Tubgcp4	Gamma-tubulin complex component 4	40108.71	44930.77	41223.66	44809	**42768**	51881.09	41021.38	57313.01	46012.08	**49057**	**0.8718**	**0.14408**
Q91YE6	Ipo9	Importin-9	23323.26	19697.69	20041.99	16470.2	**19883**	20228.01	16901.25	17379.68	17985.99	**18124**	**1.0971**	**0.30864**
Q8VIM9	Irgq	Immunity-related GTPase family Q protein	67495.01	62196.38	65112.64	58456.72	**63315**	64693.92	61150.88	70347.49	68796.66	**66247**	**0.9557**	**0.34276**
P35803	Gpm6b	Neuronal membrane glycoprotein M6-b	135453.6	161539.9	91047.31	143970.4	**133003**	154570.8	172957.6	134662	128691	**147720**	**0.9004**	**0.44644**
Q60870	Reep5	Receptor expression-enhancing protein 5	212568.6	189222.8	242299.7	302961.9	**236788**	239350	229450.9	266036.8	256498.3	**247834**	**0.9554**	**0.68492**
P16406	Enpep	Glutamyl aminopeptidase	18373.82	20825.17	17265.85	18071.5	**18634**	13636.36	20889.61	15446.66	17014.97	**16747**	**1.1127**	**0.31560**
Q3TWL2	Pip4p1	Type 1 phosphatidylinositol 4,5-bisphosphate 4-pho	48641.7	50685.89	56466.51	60859.54	**54163**	57761.23	56375.98	51379.57	53287.55	**54701**	**0.9902**	**0.86946**
P80315	Cct4	T-complex protein 1 subunit delta	254698.4	261277.1	274443.3	276766.8	**266796**	272300.8	288148.6	297057.3	278652.4	**284040**	**0.9393**	**0.06301**
Q61792	Lasp1	LIM and SH3 domain protein 1	135559.7	142547.3	166397.1	135882.8	**145097**	148048.2	134771.2	155881.7	157122.8	**148956**	**0.9741**	**0.68007**
P60469	Ppfia3	Liprin-alpha-3	31606.37	30508.68	33238.14	31256.72	**31652**	33713.46	29212.2	27755.51	32123.56	**30701**	**1.0310**	**0.54181**
O54916	Reps1	RalBP1-associated Eps domain-containing protein 1	13014.08	12308.38	12765.49	13516.61	**12901**	13239.09	13499.17	11941.46	13574.22	**13063**	**0.9876**	**0.73434**
Q6NSR8	Npepl1	Probable aminopeptidase NPEPL1	26727.82	29690.42	31809.47	24675.49	**28226**	27544.08	27627.67	30266.56	32167.05	**29401**	**0.9600**	**0.56535**
Q9D4V0	Etnk1	Ethanolamine kinase 1	24456.54	22502.37	20039.05	21983.57	**22245**	23539	24758.67	25856.39	21120.13	**23819**	**0.9340**	**0.29226**
Q4KMM3	Oxr1	Oxidation resistance protein 1	65926.89	73829.6	61444.23	58555.47	**64939**	68178.11	69623.75	60859.25	58841.26	**64376**	**1.0088**	**0.89914**
P97952	Scn1b	Sodium channel subunit beta-1				7985.193	**7985**	24323.41				**24323**	**0.3283**	
P61600	Naa20	N-alpha-acetyltransferase 20	14912.22	14648.35	14808.62	15566.3	**14984**	15276.64	17895.01	14717.53	16840.85	**16183**	**0.9259**	**0.16296**
P35285	Rab22a	Ras-related protein Rab-22A	42118.84	36814.68	41241.66	41510.54	**40421**	32083.59	42597.98	38608.14	42367.68	**38914**	**1.0387**	**0.60197**
Q03963	Eif2ak2	Interferon-induced, double-stranded RNA-activated	21130.63	17841.66	17771.38	17962.09	**18676**	17717.37	19680.72	14817.92	15596.4	**16953**	**1.1017**	**0.25469**
P42337	Pik3ca	Phosphatidylinositol 4,5-bisphosphate 3-kinase cata	13490.12	7600.805	11839.9	9124.533	**10514**		15216.35	13969.95	9608.563	**12932**	**0.8130**	**0.30491**
Q8R146	Apeh	Acylamino-acid-releasing enzyme	66251.87	68019.23	62307.1	55203.63	**62945**	66897.77	67633.02	63738.71	57512.05	**63945**	**0.9844**	**0.79387**
Q811I0	Atpaf1	ATP synthase mitochondrial F1 complex assembly fa	15598.26	17758.52	17289.44	13158.65	**15951**	9592.132	13962.43	16087.34	13527.62	**13292**	**1.2000**	**0.17045**
Q9D8B7	Jam3	Junctional adhesion molecule C	253776.4	210696.9	155507.6	237738.7	**214430**	234304.7	247396.8	150345.6	157770.3	**197454**	**1.0860**	**0.62735**
P97742	Cpt1a	Carnitine O-palmitoyltransferase 1, liver isoform	95437.09	92596.96	103358.7	101509.4	**98226**	94451.1	100511.2	97724.05	106103	**99697**	**0.9852**	**0.69139**
Q8R0F8	Fahd1	Acylpyruvase FAHD1, mitochondrial	18166.69	20208.71	22670.34	24431.12	**21369**	15943.13	19967.79	17498.45	19382.55	**18198**	**1.1743**	**0.10345**
Q80U56	Avl9	Late secretory pathway protein AVL9 homolog	12777.17	10524.87	10152.53	11528.6	**11246**	10002.72	13274.07	10812.57	10459.06	**11137**	**1.0098**	**0.91154**
Q01853	Vcp	Transitional endoplasmic reticulum ATPase	1029484	1043058	1078656	1077142	**1057085**	1080670	1054573	1128255	1111250	**1093687**	**0.9665**	**0.12400**
Q9DB26	Phyhd1	Phytanoyl-CoA dioxygenase domain-containing protein 1		13381.79		26191.42	**19787**							
A2BDX3	Mocs3	Adenylyltransferase and sulfurtransferase MOCS3	85156.13	71964.73	93132.3	91187.97	**85360**	88749.94	77934.25	54587.7	59182.14	**70114**	**1.2175**	**0.15306**
Q9D1L0	Chchd2	Coiled-coil-helix-coiled-coil-helix domain-containing	86721	84131.63	88061.12	91431.98	**87586**	87864.33	86524.49	111435.7	108881	**98676**	**0.8876**	**0.15538**
Q91ZR2	Snx18	Sorting nexin-18	12774.1	14154.99	14426.08	12615.5	**13493**	15655.8	13478.32	15222.97	14799.78	**14789**	**0.9123**	**0.09775**
Q8C419	Gpr158	Metabotropic glycine receptor		12659.58		13736.99	**13198**		7932.036			**7932**	**1.6639**	
Q8C3W1		Uncharacterized protein C1orf198 homolog	23362.24	27889.02	27737.71	24860.04	**25962**	21045.17	27716.73	17830.17	24419.89	**22753**	**1.1410**	**0.23047**
Q9WVJ3	Cpq	Carboxypeptidase Q	108331.5	91465.7	88512.88	78967.89	**91819**	92124.37	88793.32	76334.02	83188.3	**85110**	**1.0788**	**0.37647**
Q925E7	Ppp2r2d	Serine/threonine-protein phosphatase 2A 55 kDa reg	42962.09	49073.77	41009.71	41730.99	**43694**	42460.97	42432.35	37514.71	42027.86	**41109**	**1.0629**	**0.28372**
Q7TMC8	Fcsk	L-fucose kinase	12188.92	9445.75	13202.32	2708.594	**9386**	11479.26	10651.61		15778.91	**12637**	**0.7428**	**0.34190**
Q8K009	Aldh1l2	Mitochondrial 10-formyltetrahydrofolate dehydrogenase				1706.569	**1707**							
Q8VHE0	Sec63	Translocation protein SEC63 homolog	29844.28	31973.43	36596.79	32472.47	**32722**	30759.96	31351.08	37020.12	38299.66	**34358**	**0.9524**	**0.51916**
Q7M750	Opalin	Opalin												
Q9D7G0	Prps1	Ribose-phosphate pyrophosphokinase 1	202745.7	206809.4	199817.3	200236.1	**202402**	206147.9	212936.5	167215.9	189234.1	**193884**	**1.0439**	**0.44055**
Q8CF66	Lamtor4	Ragulator complex protein LAMTOR4	32189.64	30192.81	32263.4	32311.08	**31739**	29413.38	31354.65	32387.3	32383.11	**31385**	**1.0113**	**0.69774**
Q8BHD7	Ptbp3	Polypyrimidine tract-binding protein 3	36630.77	34706.39	34626.52	39618.6	**36396**	40133.89	35643.95	41301.14	42569.43	**39912**	**0.9119**	**0.11488**
Q8R3Z5	Cacnb1	Voltage-dependent L-type calcium channel subunit beta-1												
Q8CFV9	Rfk	Riboflavin kinase	33856.59	36582.91	39910.1	22835.09	**33296**	32502.13	35894.48	23745.42	27223.64	**29841**	**1.1158**	**0.47947**
Q9QZ73	Dcun1d1	DCN1-like protein 1	9652.531	8060.943	10162.87	9684.977	**9390**	10436.94	7510.285	7937.258	8690.68	**8644**	**1.0864**	**0.38210**
Q9QXK3	Copg2	Coatomer subunit gamma-2	123519.9	123664.5	133624.2	124050.4	**126215**	129968.2	128172.2	139112.7	147973.9	**136307**	**0.9260**	**0.10001**
Q80V91	Dtx3	Probable E3 ubiquitin-protein ligase DTX3	10912.5	12966.92	11587.18	8475.69	**10986**		11929.22	4486.914	7415.974	**7944**	**1.3829**	**0.21175**
Q7TQD2	Tppp	Tubulin polymerization-promoting protein	11649.39	16185.93	18822.83	11453.77	**14528**	16227.82	12812.54	14128.7	17164.17	**15083**	**0.9632**	**0.79596**
O08529	Capn2	Calpain-2 catalytic subunit	410705.9	485408.4	413275.3	361226.1	**417654**	377495.5	399303.5	354359.3	352992.6	**371038**	**1.1256**	**0.14481**
Q07235	Serpine2	Glia-derived nexin	23043.59	20483.96	25571.94	24061.76	**23290**	25042.73	21151.58	26610.55	27224.97	**25007**	**0.9313**	**0.36035**
Q8VD66	Abhd4	(Lyso)-N-acylphosphatidylethanolamine lipase	43213.28	46081.58	46795.91	36413.04	**43126**	37405.46	38059.94	34706.68	39568.09	**37435**	**1.1520**	**0.06926**
Q9DB10	Smdt1	Essential MCU regulator, mitochondrial												
Q8K215	Lyrm4	LYR motif-containing protein 4	33915.87	27949.79	27230.54	36146.43	**31311**	34393.36	39449.55	35789.98	35167.04	**36200**	**0.8649**	**0.09503**
P85094	Isoc2a	Isochorismatase domain-containing protein 2A	86776.16	68474.09	73093.26	94647.13	**80748**	88297.18	78350.17	69401.52	77966.91	**78504**	**1.0286**	**0.76510**

Q8BH93	Mapk1ip1l	MAPK-interacting and spindle-stabilizing protein-like	24052.6	20473.83	18908.8	14220.38	19414	23759.38	14288.29	28678.52	21670.06	22099	0.8785	0.48605
Q8CAY6	Acat2	Acetyl-CoA acetyltransferase, cytosolic	89307.3	93081.16	109927.9	82986.32	93826	93636.41	91002.17	113062.1	106320.7	101005	0.9289	0.39150
Q9QZN4	Fbxo6	F-box only protein 6	16795.23	19552.68	20051.86	19212.84	18903	18754.34	18418.48	15446.87	15793.26	17103	1.1052	0.16083
Q5U5Q9	Uimc1	BRCA1-A complex subunit RAP80	59789.05	78978.84	74675.77	78634.72	73020	89099.5	83384.58	103646.3	89432.72	91391	0.7990	0.02591
Q91YP0	L2hgdh	L-2-hydroxyglutarate dehydrogenase, mitochondrial	13689.64	11209.91	8584.749	11954.49	11360	10386.84	11089.64	7430.054	9173.204	9520	1.1933	0.21568
Q99JR5	Tinagl1	Tubulointerstitial nephritis antigen-like	71592.13	62405.17	42632.2	73519.88	62537	63087.7	72602.46	44757.85	45618.45	56517	1.1065	0.56241
Q9JJL8	Sars2	Serine--tRNA ligase, mitochondrial	14522.63	13008.42	16106.77	16775.48	15103	13102.04	14320.71	16052.52	17826.04	15325	0.9855	0.87302
Q99LI8	Hgs	Hepatocyte growth factor-regulated tyrosine kinase s	83537.33	87453.78	85197.44	80198.21	84097	80861.41	86942.58	91348.49	85498.29	86163	0.9760	0.46466
Q68ED7	Crtc1	CREB-regulated transcription coactivator 1	70544.92	86847.36	86747.77	115146	89822	96363.84	72843.68	77119.9	75498.66	80457	1.1164	0.41575
P62965	Crabp1	Cellular retinoic acid-binding protein 1	9942.103	13832.76	7892.318	6385.423	9513	17134.71	13618.79	21578.04	11668.15	16000	0.5946	0.05372
Q9Z0V2	Kcnd2	Potassium voltage-gated channel subfamily D member 2		1606.482			1606	1569.685			1642.366	1606	1.0003	
O88531	Ppt1	Palmitoyl-protein thioesterase 1	87128.6	78108.2	73628.97	94446.45	83328	91497.24	92662.75	70622.58	80148.13	83733	0.9952	0.95564
Q7TNS2	Micos10	MICOS complex subunit Mic10	3299.635	2498.282	4439.009	3867.173	3526	4842.297	3305.457	2921.179	6049.371	4280	0.8239	0.39983
Q6PGB6	Naa50	N-alpha-acetyltransferase 50	38877.45	40265.59	37016.11	36500.19	38165	39060.89	42477.39	36451.18	41263.92	39813	0.9586	0.33792
Q99JB2	Stoml2	Stomatin-like protein 2, mitochondrial	47294.3	43147.43	49249.84	57774.59	49367	47044.73	51328.29	55112.36	57389.09	52719	0.9364	0.41427
Q8R2U4	Ntmt1	N-terminal Xaa-Pro-Lys N-methyltransferase 1	21621.1	20908.53	21153.99	21665.49	21337	20370.94	20001.25	18611.96	20883.99	19967	1.0686	0.03884
P62311	Lsm3	U6 snRNA-associated Sm-like protein LSm3	114623.6	110550.9	111136.4	100884.7	109299	114966.8	99928.88	128408.1	121100	116101	0.9414	0.35111
Q99KF1	Tmed9	Transmembrane emp24 domain-containing protein 9	316307.7	314096.4	337112.9	334547.5	325516	302470.5	314886.5	390952.1	369549.5	344465	0.9450	0.42411
P62754	Rps6	Small ribosomal subunit protein eS6	789891.1	863741.2	876413.4	962251.7	873074	919497.1	886065.6	940159.1	918973.8	916174	0.9530	0.28886
Q8CHX7	Rftn2	Raftlin-2			8304.357		8304	12310.15	17012.98	6911.95	22853.68	14772	0.5622	
Q05186	Rcn1	Reticulocalbin-1	35995.23	40130.32	34837.72	35123.89	36522	31316.32	33521.59	37279.6	31753.65	33468	1.0913	0.14620
P46735	Myo1b	Unconventional myosin-Ib	290551.9	257565.2	256695	301509.7	276580	257057.7	293039.6	257863.2	273406.8	270342	1.0231	0.67646
Q62087	Pon3	Serum paraoxonase/lactonase 3	41480.32	34432.39	36011.54	32968.33	36223	30723.2	41842.26	33638.68	31973.38	34544	1.0486	0.60984
Q9JKX6	Nudt5	ADP-sugar pyrophosphatase	31660.53	32444.79	31696.98	25051.12	30213	32610.79	27850.72	27537.85	28497.25	29124	1.0374	0.62156
P07091	S100a4	Protein S100-A4	169372.1	139834.4	159672.6	112238.9	145280	114364	131416.3	119595.8	104386.5	117441	1.2370	0.09043
Q80UG2	Plxna4	Plexin-A4	13466.51	12834.81	13898.18	18931.37	14783	9987.646	16794.78	11883.04	12235.35	12725	1.1617	0.34570
Q9EQX4	Aif1l	Allograft inflammatory factor 1-like	23242.07	26892.37	23022.8	14984.55	22035	20054.33	25739.26	18633.88	16001.49	20107	1.0959	0.57426
O70251	Eef1b	Elongation factor 1-beta	232131.3	227944.3	227774.2	196172	221005	189308.8	222216.3	221343.5	203493.1	209090	1.0570	0.33905
Q8CIG8	Prmt5	Protein arginine N-methyltransferase 5	32870.32	34474.45	27650.78	25732.61	30182	26332.69	32303.02	31874.71	27843.23	29588	1.0201	0.82373
Q9Z2S7	Tsc22d3	TSC22 domain family protein 3	34493.95	38000.06		11984.83	28160			24713.54		24714	1.1394	
Q99PT1	Arhgdia	Rho GDP-dissociation inhibitor 1	132247.9	124104.3	122363.3	97812.87	119132	116712.5	109376.1	109481.7	101694.4	109316	1.0898	0.26760
P14148	Rpl7	Large ribosomal subunit protein uL30	570673.9	546935.6	630191.4	672176.9	604994	567419.9	566780.2	712462.8	726605.3	643317	0.9404	0.49266
P59279	Rab2b	Ras-related protein Rab-2B	24215.56	21791.56	21750.61	23403.09	22790	24937.89	26130.19	22723.56	24566.11	24589	0.9268	0.10228
Q9QXY9	Pex3	Peroxisomal biogenesis factor 3	6086.355	4880.476	5909.551	6685.698	5891	5113.479	6700.864	5809.485	6220.02	5961	0.9882	0.89340
Q9JIK9	Mrps34	Small ribosomal subunit protein mS34	34909.2	33452.79	40780.92	38242.57	36846	44043.95	34180.45	34154.68	48911.48	40323	0.9138	0.42283
Q9QXV0	Pcsk1n	ProSAAS	78448.82	75031.29	100752.5	84838.29	84768	87136.75	90324.65	87459.8	100158.6	91270	0.9288	0.35344
Q99K70	Rragc	Ras-related GTP-binding protein C	51398.45	57554.66	55069.25	47968.71	52998	61912.53	55729.3	51955.87	55121.66	56180	0.9434	0.32325
Q80ZJ6	Zer1	Protein zer-1 homolog	9350.438	10637.6	11248.52	9536.381	10193	8367.633	8965.899	6764.343	11120.25	8805	1.1577	0.21751
Q91VN4	Chchd6	MICOS complex subunit Mic25	11276.87	15670.39	14686.33	13501.91	13784	15531.59	18216.8	18228.59	17351.71	17332	0.7953	0.02071
Q61205	Pafah1b3	Platelet-activating factor acetylhydrolase IB subunit	61903.09	65235.36	49985.85	49792.18	56729	69421.02	58264.54	42259.99	49455.24	54850	1.0343	0.80005
P46664	Adss2	Adenylosuccinate synthetase isozyme 2	85009.54	78756.51	66680.75	60595.75	72761	82900.42	71595.79	71919.37	69251.05	73917	0.9844	0.86138
Q99KP3	Cryl1	Lambda-crystallin homolog	13853.66	12601.27	10746.76	10885.58	12022	13785.78	10598.9	10605.45	9794.465	11196	1.0737	0.50124
Q3UNZ8	Cryzl2	Quinone oxidoreductase-like protein 2	42419.63	38712.48	34821.87	61775.83	44432	43038.86	55336.28	29380.44	35288.26	40761	1.0901	0.67008
B1AZP2	Dlgap4	Disks large-associated protein 4	10742.2	13304.08	14726.52	11313.92	12522	11273.09	12270.98	13743.26	11451.59	12185	1.0277	0.76485
D3YWQ0	Dgki	Diacylglycerol kinase iota	94679.2	112036.8	108336	117976.6	108257	109948.4	114428.5	92835.39	99572.84	104196	1.0390	0.58086
Q921E2	Rab31	Ras-related protein Rab-31	12412.89	11442.69	15345.78	13397.39	13150	12769.55	13104.57	12936.66	14234.58	13261	0.9916	0.90503
Q61879	Myh10	Myosin-10	150966.7	169801.8	174089.7	158317.8	163294	156962.5	153836.7	149570	168663.1	157258	1.0384	0.40150
B2RUJ5	Apba1	Amyloid-beta A4 precursor protein-binding family A r	20352.66	17539.41	16766.05	14505.23	17291	19808.67	17734.33	14789.39	18490.52	17706	0.9766	0.80498
P56135	Atp5mf	ATP synthase subunit f, mitochondrial	698222.1	684925.9	779564.9	835703.8	749604	802858.2	833938.3	721734.6	813365.8	792974	0.9453	0.35422
Q64669	Nqo1	NAD(P)H dehydrogenase [quinone] 1	23853.71	22059.21	17846.49	18304.68	20516	23016.58	20357.78	13984.06	16302	18415	1.1141	0.43170
Q8K406	Lgi3	Leucine-rich repeat LGI family member 3	76105.04	75188.73	52648.45	76324.35	70067	73713.63	74995.51	52357.68	58750.07	64954	1.0787	0.54939
P38060	Hmgcl	Hydroxymethylglutaryl-CoA lyase, mitochondrial	23655.72	23641.63	27475.68	24106.44	24720	24844.12	23301.1	25119.88	27069.44	25084	0.9855	0.77307
Q9QY76	Vapb	Vesicle-associated membrane protein-associated pr	468806.6	450002.2	464129.3	543082.5	481505	446450.2	483299.5	527196	535643.3	498147	0.9666	0.59219
P28667	Marcksl1	MARCKS-related protein	27514.22	35923.44	32150.19	40663.08	34063	30680.25	30648.57	36287.46	34604.31	33055	1.0305	0.75872
P39087	Grik2	Glutamate receptor ionotropic, kainate 2												
Q9QYC0	Add1	Alpha-adducin	362798.6	352336.7	330728.2	403552.1	362354	354392	358157.9	329113.8	337802.9	344867	1.0507	0.33649
Q00898	Serpina1e	Alpha-1-antitrypsin 1-5	14881.44	7463.425	520582.6	164929.8	176964	56229.47	2368.902	322705.8	269807.9	162778	1.0872	0.92451
P54822	Adsl	Adenylosuccinate lyase	65178.34	69963.9	71902.3	56952.37	65999	67801.07	57800.83	59078.43	61160.06	61460	1.0739	0.30027
Q9CQN6	Tmem14c	Transmembrane protein 14C	27104.11	29708.18	34850.43	31115.27	30694	30097.65	34662.66	31688.53	33638.8	32522	0.9438	0.37531

Q9WV60	Gsk3b	Glycogen synthase kinase-3 beta	82855.09	91320.91	85682.91	85171.03	86257	89711.07	99457.18	95001.69	88904.52	93269	0.9248	0.06129
P63168	Dynll1	Dynein light chain 1, cytoplasmic	364835.7	342255.7	339511.1	359468.5	351518	343751.2	320673.7	316419.4	333314.6	328540	1.0699	0.04038
O88343	Slc4a4	Electrogenic sodium bicarbonate cotransporter 1	52262.38	51164.89	42165.75	49349.9	48736	47946.61	61978.08	34793.91	42026.19	46686	1.0439	0.75199
P97798	Neo1	Neogenin	39673.06		11535.46		25604				20459.41	20459	1.2515	
O89106	Fhit	Bis(5'-adenosyl)-triphosphatase	56931.25	41404.74	51349.6	50651.86	50084	37445.16	28131.79	21685.02	50830.05	34523	1.4508	0.07079
Q9ESX5	Dkc1	H/ACA ribonucleoprotein complex subunit DKC1	34818.02	35209	34845.6	38192.28	35766	36089.3	35588.72	34364.78	36515.88	35640	1.0036	0.89700
O08997	Atox1	Copper transport protein ATOX1	62073.89	69556.71	69293.13	45045.16	61492	59307.59	59444.18	57219.96	61264.27	59309	1.0368	0.71997
Q923M0	Ppp1r16a	Protein phosphatase 1 regulatory subunit 16A												
Q8BGY7	Fam210a	Protein FAM210A	22267.17	34538.98	28192.49	28864.4	28466	23061.88	25899.06	19595.04	25288.37	23461	1.2133	0.13356
P62737	Acta2	Actin, aortic smooth muscle	1474139	1149462	1146542	1445656	1303950	736127.5	1026974	642087.8	747185.6	788094	1.6546	0.00564
O70591	Pfdn2	Prefoldin subunit 2	75475.77	79660.38	78219.67	71075.83	76108	76356.45	72888.95	89580.16	72137.19	77741	0.9790	0.72747
Q8VEB6	Elac1	Zinc phosphodiesterase ELAC protein 1	7493.121	5992.64	4338.486	3169.194	5248	3878.594	5628.156	4485.299	4074.241	4517	1.1620	0.50168
Q60673	Ptprn	Receptor-type tyrosine-protein phosphatase-like N	11685.55	19649.65	7834.052	26209.25	16345	10380.24	9838.062		12768.9	10996	1.4865	0.32631
Q99NB9	Sf3b1	Splicing factor 3B subunit 1	108384.3	109657.6	120892.8	119826.9	114690	111225.6	107197.7	117910.2	126234.3	115642	0.9918	0.86363
P24527	Lta4h	Leukotriene A-4 hydrolase	110440.8	112962.3	108245.4	93172.77	106205	106481.9	100992.8	101655.3	91262.4	100098	1.0610	0.30729
O09114	Ptgds	Prostaglandin-H2 D-isomerase	25540.18	17876.24	26960.94	23191.28	23392	25588.42	19520.15	12691.25	9287.511	16772	1.3947	0.16099
P63040	Cplx1	Complexin-1	77766.75	90385.03	55279.8	48073.03	67876	80250.75	87717.06	65516.3	55220.27	72176	0.9404	0.73709
Q3UMU9	Hdgfl2	Hepatoma-derived growth factor-related protein 2	72631.54	76323.89	63943.02	68943.7	70461	66459.91	63283.05	64972.57	78013.09	68182	1.0334	0.61201
Q9CQY5	Magt1	Magnesium transporter protein 1	128029.4	117457.4	140313.6	143094	132224	132428.2	125656.5	134203.8	142970.7	133815	0.9881	0.82534
Q9WTR5	Cdh13	Cadherin-13	40563.43	24422.29	50711.27	43421.38	39780	42278.8	39845.34	34124.25	42349.84	39650	1.0033	0.98305
O70161	Pip5k1c	Phosphatidylinositol 4-phosphate 5-kinase type-1 ga	42861.79	41321.18	41761.04	43103.97	42262	43650.24	40684.34	38471.89	40959.12	40941	1.0323	0.29228
P05214	Tuba3a	Tubulin alpha-3 chain	1884790	2258754	1928808	1529275	1900407	1859298	2138968	1601644	1936890	1884200	1.0086	0.93342
Q9R0N3	Syt11	Synaptotagmin-11	11816.51	14091.05	11628.99	14226.63	12941	16317.95	13452.08	13666.51	13981.44	14354	0.9015	0.19445
P48453	Ppp3cb	Serine/threonine-protein phosphatase 2B catalytic s	201300.8	202036.6	180389.3	205335.3	197266	202767.4	200643.9	179355.2	187826.3	192648	1.0240	0.58175
Q78ZA7	Nap1l4	Nucleosome assembly protein 1-like 4	90382.56	87665.73	89679.38	78030.94	86440	76120.47	82725.38	86076.05	79161.57	81021	1.0669	0.18136
B1AR13	Cisd3	CDGSH iron-sulfur domain-containing protein 3, mit	28231.35	33744.27	36546.37	36025.09	33637	32563.62	36380.66	32484.88	40438.92	35467	0.9484	0.52034
B2RXC1	Trappc11	Trafficking protein particle complex subunit 11	22267.11	20258.71	21930.73	21260.53	21429	20948.99	21314.75	20213.09	23267.96	21436	0.9997	0.99327
Q8VD37	Sgip1	SH3-containing GRB2-like protein 3-interacting prote	5091.694	5976.015	6700.735	5653.972	5856	5027.465	7339.933	4018.943	5628.851	5504	1.0639	0.66506
Q9DC71	Mrps15	Small ribosomal subunit protein uS15m	14595.3	12041.3	15690.64	14137.82	14116	12677.78	13742.95	16182.33	17725.11	15082	0.9360	0.50963
Q6NS60	Fbxo41	F-box only protein 41	11600.06	9377.871	11869.74		10949							
Q8R127	Sccpdh	Saccharopine dehydrogenase-like oxidoreductase	141166.5	128452.6	139094.1	162427.3	142785	137833.5	133641.5	148497.8	161650.8	145406	0.9820	0.79131
Q8CFV4	Nrn1	Neuritin	18208.29	11903.66		14815.48	14976	10740.84	67800.97	13557.03		30700	0.4878	0.44681
P13439	Umps	Uridine 5'-monophosphate synthase	16698.61	18205.97	22714.79	14397.36	18004	15666.85	15823.4	14298.09	16299.66	15522	1.1599	0.21854
Q80ZJ1	Rap2a	Ras-related protein Rap-2a	90796.47	82990.35	81234.52	96805.23	87957	90713.53	94427.72	84100.2	88228.34	89367	0.9842	0.74894
Q3UW53	Niban1	Protein Niban 1	75365.24	69811.1	73144.09	72271.56	72648	65414.91	73466.19	68381.07	66355.55	68404	1.0620	0.09374
Q9D115	Znf706	Zinc finger protein 706	70326.38	46823.88	55627.7	62997.98	58944	42086.41	54307.23	59679.44	65140.93	55304	1.0658	0.62379
E9Q4P1	Wdfy1	WD repeat and FYVE domain-containing protein 1	13620.55	14856.18	14695.38	25747.01	17230	23689.51		12992.3	14965.46	17216	1.0008	0.99756
P63141	Kcna2	Potassium voltage-gated channel subfamily A memb	44231.07	39635.98	37258.1	50286.05	42853	42860.64	30791.52	32876.91	32326.41	34714	1.2345	0.08655
O35737	Hnrnph1	Heterogeneous nuclear ribonucleoprotein H	258233	256102.2	282020	276836.5	268298	273991.2	277218.9	316312.8	302282.8	292451	0.9174	0.09242
P18894	Dao	D-amino-acid oxidase	5923.063				5923							
Q9JI91	Actn2	Alpha-actinin-2	14535.06	19054.13	14489.92	22719.17	17700	12448.77	16480.27	15208	13793.34	14483	1.2221	0.18863
Q3V1U8	Elmod1	ELMO domain-containing protein 1												
Q91W39	Ncoa5	Nuclear receptor coactivator 5	26246.54	24915.45	28640.55	32068.45	27968	26178.21	28366.98	24298.36	30226.08	27267	1.0257	0.74202
Q8BK26	Fbxo44	F-box only protein 44	9508.544	7886.337			8697		10458.14			10458	0.8316	
Q99LU0	Chmp1b1	Charged multivesicular body protein 1B1	8578.243	10166.63			9372	8316.812	7912.816	10030.41	5409.462	7917	1.1838	0.39007
Q61733	Mrps31	Small ribosomal subunit protein mS31	5062.533	4952.426	4410.445	5914.506	5085	4884.723	5091.435	5826.913	4646.563	5112	0.9946	0.94784
P26339	Chga	Chromogranin-A												
Q923D2	Blvrb	Flavin reductase (NADPH)	131111.7	140478	138371.5	123652.2	133403	124989.3	116810.4	106659.9	102632.4	112773	1.1829	0.01727
Q6ZWN5	Rps9	Small ribosomal subunit protein uS4	917060.1	872304.6	1003281	1017716	952590	941486.9	952917.6	1179119	1143509	1054258	0.9036	0.20397
O35621	Pmm1	Phosphomannomutase 1	23243.13	21365.24	16835.24	16790.45	19559	21645.31	20246.08	18125.31	17143.91	19290	1.0139	0.89354
P97351	Rps3a	Small ribosomal subunit protein eS1	341979.7	348141.8	373702.7	389072.8	363224	351872.4	380614.1	455798.1	431214.4	404875	0.8971	0.16093
Q8K2D3	Edc3	Enhancer of mRNA-decapping protein 3	28316.58	25791.87	25305.1	25958.64	26343	27182.31	23323.67	24514.48	27696.27	25679	1.0259	0.61357
O54833	Csnk2a2	Casein kinase II subunit alpha'	11398.84	11357.13	10314.49	10000.27	10768	9260.953	9171.703	7990.656	8503.032	8732	1.2332	0.00476
Q3UV70	Pdp1	[Pyruvate dehydrogenase [acetyl-transferring]]-phos	24748.76	24349.2	24400.69	25278.45	24694	23760.48	25914.3	22496.61	25388.09	24390	1.0125	0.71961
Q9R049	Amfr	E3 ubiquitin-protein ligase AMFR	36552.74	41219.43	44653.31	49225.01	42913	41116.72	37449.82	42748.77	43014.23	41082	1.0446	0.56045
Q9Z2H5	Epb41l1	Band 4.1-like protein 1	33995.75	40343.87	33491.15	39162.93	36748	38668.85	41329.57	37437.35	32118.85	37389	0.9829	0.81456
Q3UZ39	Lrrfip1	Leucine-rich repeat flightless-interacting protein 1												
Q63829	Commd3	COMM domain-containing protein 3	17204.13	13610.98	16269.94	12674.98	14940	11494.6	13567.26	11214.5	12609.1	12221	1.2225	0.06415
P61759	Vbp1	Prefoldin subunit 3	49584.17	45161.05	47849.17	42863.49	46364	50361.41	52888.03	45301.23	43712.09	48066	0.9646	0.53784

Q3TIX9	Usp39	Ubiquitin carboxyl-terminal hydrolase 39	14385.73	15601.2	16667.08	14710.84	**15341**	15385.94	17089.02	15130.08	14837.95	**15611**	0.9827	0.72057
F6SEU4	Syngap1	Ras/Rap GTPase-activating protein SynGAP	2372.275				**2372**			22285.71		**22286**	0.1064	
P02088	Hbb-b1	Hemoglobin subunit beta-1	5597459	7012330	7992487	6305443	**6726930**	5360074	5421555	6333118	3945830	**5265144**	1.2776	0.08523
Q14BB9	Map6d1	MAP6 domain-containing protein 1				394.9603	**395**							
Q8BH69	Sephs1	Selenide, water dikinase 1	46892.98	46721.72	59664.89	45934.46	**49804**	49339.6	37478.8	48889.43	54073.8	**47445**	1.0497	0.64224
Q6VNS1	Ntrk3	NT-3 growth factor receptor	20323.89	17853.96	23879.71	23605.59	**21416**	21524.02	21590.81	23791.92	29712.15	**24155**	0.8866	0.29770
P62774	Mtpn	Myotrophin	88252.61	91962.13	84550.13	67236.83	**83000**	84001.52	81589.84	80941.05	69192.71	**78931**	1.0516	0.54795
Q9D9V3	Echdc1	Ethylmalonyl-CoA decarboxylase	69033.91	78979.15	87766.87	63829.23	**74902**	77150.38	69969.84	69522.24	76113.12	**73189**	1.0234	0.77312
O89112	Lancl1	Glutathione S-transferase LANCL1	123831.7	117413.4	95766.49	106017.4	**110757**	110040.3	115899.3	84113.23	101532.7	**102896**	1.0764	0.43024
Q7TMQ7	Wdr91	WD repeat-containing protein 91	26407.42	28677.9	26779.49	34351.04	**29054**	26430.49	32516.32	25794.09	26589.12	**27833**	1.0439	0.63104
Q8BG73	Sh3bgrl2	SH3 domain-binding glutamic acid-rich-like protein 2	105338.7	120528	88172.19	83465.14	**99376**	123031.6	98479.99	89551.7	78573.23	**97409**	1.0202	0.88201
P42225	Stat1	Signal transducer and activator of transcription 1	153740.6	83790.84	67502.99	68878.99	**93478**	88031.63	83262.98	63905.41	69865.69	**76266**	1.2257	0.44759
E9QAT4	Sec16a	Protein transport protein Sec16A	18835.96	15683.91	16737.55	16548.3	**16951**	19211.31	17419.5	20121.57	20907.74	**19415**	0.8731	0.04968
Q8K3K8	Optn	Optineurin	35701.17	38803.24	37984.51	27232.99	**34930**	34115.95	33050.25	25634.88	30471.81	**30818**	1.1334	0.25312
Q9D0E1	Hnrnpm	Heterogeneous nuclear ribonucleoprotein M	204697.2	207333.5	238468.2	227973	**219618**	210158.1	208426.3	252234.4	256643.5	**231866**	0.9472	0.45691
Q6IEE6	Tmem132e	Transmembrane protein 132E		25390.34	20348.32	23216.66	**22985**	26234.18				**26234**	0.8762	
Q8BYZ1	Abi3	ABI gene family member 3	6957.214	4734.601	5508.643	5474.962	**5669**	4444.856	4325.415	7164.227	6650.16	**5646**	1.0040	0.98005
Q9D0I9	Rars1	Arginine--tRNA ligase, cytoplasmic	377900.2	396332.7	390064.8	410018.5	**393579**	392379.4	408716.6	424864.8	428716.9	**413669**	0.9514	0.10866
Q62432	Smad2	Mothers against decapentaplegic homolog 2	39941.15	37152.41	41243.88	39131.63	**39367**	38829.77	37851.72	42305.92	43374.64	**40591**	0.9699	0.46927
Q6NZR5	Skic2	Superkiller complex protein 2	27782.48	26576.32	30542.15	32474.56	**29344**	32337.14	31496.78	33992.49	33862.6	**32922**	0.8913	0.05024
Q9QZB9	Dctn5	Dynactin subunit 5	34159.46	34734.4	35284.1	36260.99	**35110**	32563.99	37358.86	33204.16	33457.41	**34146**	1.0282	0.44375
Q8BJH1	Zc2hc1a	Zinc finger C2HC domain-containing protein 1A	26769.24	19889.38	19121	32229.26	**24502**	25255.93	24767.99	23926.36	23564.25	**24379**	1.0051	0.96970
O35382	Exoc4	Exocyst complex component 4	59525.83	55467.29	56952.56	58300.72	**57562**	55551.61	59289.43	57676.69	58708.72	**57807**	0.9958	0.84495
Q9QUR6	Prep	Prolyl endopeptidase	65807.23	68943.51	70131.43	55041.88	**64981**	65698.57	62259.19	61742.71	61635.56	**62834**	1.0342	0.56949
Q2PFD7	Psd3	PH and SEC7 domain-containing protein 3				10146.18	**10146**	9791.572		4486.691	5288.292	**6522**	1.5556	
Q8C165	Pm20d1	N-fatty-acyl-amino acid synthase/hydrolase PM20D1												
Q9Z2X2	Psmd10	26S proteasome non-ATPase regulatory subunit 10	15358.66	14635.77	14153.67	11474.55	**13906**	15061.88	13589.11	7649.245	9993.023	**11573**	1.2015	0.26273
Q6PIE5	Atp1a2	Sodium/potassium-transporting ATPase subunit alph	2203746	2062278	2013185	2444972	**2181045**	2105630	2371022	1867554	1982496	**2081676**	1.0477	0.51884
Q9D1C3	Pyurf	Protein preY, mitochondrial	13550.74	14586.86	14308.49	13340.33	**13947**	14637.13	13163.95	8921.903	11997.53	**12180**	1.1450	0.20700
O35129	Phb2	Prohibitin-2	269512.5	246915	291158.1	304106.4	**277923**	275588.9	288087.4	306495	336168.9	**301585**	0.9215	0.24107
P11983	Tcp1	T-complex protein 1 subunit alpha	409772.9	410687.2	438530	438426.9	**424354**	447599.3	446121.5	457016.6	470975.3	**455428**	0.9318	0.02059
P70333	Hnrnph2	Heterogeneous nuclear ribonucleoprotein H2	148925.6	152767.8	170590.3	175838.3	**162030**	151379.3	167102.6	168803.6	167046.5	**163583**	0.9905	0.84798
Q9CSU0	Rprd1b	Regulation of nuclear pre-mRNA domain-containing	17671.11	14828.9	18631.05	19725.4	**17714**	17719.84	15256.25	19229.55	17637.87	**17461**	1.0145	0.85551
Q8VCL2	Sco2	Protein SCO2 homolog, mitochondrial		121335.6	105603	50690.97	**92543**	10719.28	20193.76	70562.36	107672.6	**52287**	1.7699	0.26710
Q9DC04	Rgs3	Regulator of G-protein signaling 3	81033.79	66815.98	55573.98	52962.77	**64097**	75342.73	54236.7	70912.06	72171.88	**68166**	0.9403	0.62739
P97441	Slc30a3	Probable proton-coupled zinc antiporter SLC30A3		23389.6			**23390**			19103.6		**19104**	1.2244	
Q9WUZ9	Entpd5	Ectonucleoside triphosphate diphosphohydrolase 5	14498.6	16920.1	17508.02	17449.94	**16594**	17557.56	16679.36	15653.38	19058.39	**17237**	0.9627	0.54886
Q9D868	Ppih	Peptidyl-prolyl cis-trans isomerase H	15937.38	15287.75	14893.62	14356.49	**15119**	14783.08	14547.49	10140.05	14825.88	**13574**	1.1138	0.24323
P35293	Rab18	Ras-related protein Rab-18	229984.8	227415.7	204291.5	225760.6	**221863**	217236.6	227303.4	218082.7	217480.8	**220026**	1.0084	0.78376
Q9D2N4	Dtna	Dystrobrevin alpha	60544	54333.79	48316.08	56574.82	**54942**	55471.69	54096.89	42966.13	45616.44	**49538**	1.1091	0.22632
Q9ESZ8	Gtf2i	General transcription factor II-I	37054	36004.49	40310.13	38351.88	**37930**	38138.98	36304.39	38665.6	44095.66	**39301**	0.9651	0.50108
Q5SWU9	Acaca	Acetyl-CoA carboxylase 1	114059.6	114573.1	132350.6	107869.4	**117213**	117539.8	114553	123332.4	131643.1	**121767**	0.9626	0.50828
Q9JJZ2	Tuba8	Tubulin alpha-8 chain	24869.41	30081.59	28324.82	32045.54	**28830**	31873.97	28667.68	29383.43	30516.58	**30110**	0.9575	0.47411
Q8K4Q0	Rptor	Regulatory-associated protein of mTOR	21876.25	22954.39	21602.29	21763.46	**22049**	22043.69	21397.63	22066.08	22621.21	**22032**	1.0008	0.96725
P35436	Grin2a	Glutamate receptor ionotropic, NMDA 2A												
Q9CQE5	Rgs10	Regulator of G-protein signaling 10	28792.01	26400.86	26255.65	24189.56	**26410**	35191.8	23103.45	36729.97	30873.21	**31475**	0.8391	0.16400
Q80UU9	Pgrmc2	Membrane-associated progesterone receptor comp	59194.02	59433.1	61398.07	57264.89	**59323**	56412.41	62005.04	58531.36	67126.02	**61019**	0.9722	0.52074
P34022	Ranbp1	Ran-specific GTPase-activating protein	62106.23	65820.52	71865.41	57294.64	**64272**	66905.24	57564.1	64757.94	59364.14	**62148**	1.0342	0.59474
Q8CE96	Trmt6	tRNA (adenine(58)-N(1))-methyltransferase non-catalytic subun		7707.148	10238.14	10023.99	**9323**		7984.473	11412.12		**9698**	0.9613	0.83484
Q6ZQ08	Cnot1	CCR4-NOT transcription complex subunit 1	30536.09	30092.26	29956.37	29143.65	**29932**	28258.33	29909.6	27541.76	32903.36	**29653**	1.0094	0.82768
Q8CHH9	Septin8	Septin-8	99642.36	108902.4	111089.9	112662	**108074**	111001	118248.6	116309.4	110702.7	**114065**	0.9475	0.13573
Q9WTR1	Trpv2	Transient receptor potential cation channel subfamil	19497.22	15511.14	19948	24175.21	**19783**	21158.6	21379.32	16299.5	18841.02	**19420**	1.0187	0.87034
Q6PD10	Ip6k1	Inositol hexakisphosphate kinase 1	24435.17	23240.66	22793.83	20167.38	**22659**	22338.51	22442.38	20471.51	24698.24	**22488**	1.0076	0.89515
A2AJA9	Ajm1	Apical junction component 1 homolog												
Q8BVI4	Qdpr	Dihydropteridine reductase	34305.14	37692.61	32762.81	30349.44	**33777**	34151.31	34696.68	32165.26	29223.65	**32559**	1.0374	0.55985
P06797	Ctsl	Procathepsin L	44225.38	43488.4	43758.12	37201.8	**42168**	41624.55	43478.09	39970.72	44153.54	**42307**	0.9967	0.94469
Q810A3	Ttc9c	Tetratricopeptide repeat protein 9C	12864.96	10037.01	8514.182	5391.316	**9202**	9650.674	9144.241	11229.65	10583.03	**10152**	0.9064	0.58030
Q69ZS6	Sv2c	Synaptic vesicle glycoprotein 2C	5077.503	6940.164	5542.996	4946.401	**5627**	16019.46	10742.56	8477.947	7712.095	**10738**	0.5240	0.03805
S4R1M9	Osbpl10	Oxysterol-binding protein-related protein 10	20579.69	22115.87	22198.49	22209.49	**21776**	20047.32	24650.17	21623.84	22757.06	**22270**	0.9778	0.65413

Q9D906	Atg7	Ubiquitin-like modifier-activating enzyme ATG7	25477.24	22632.59	20964.82	21912.89	**22747**	24187.78	23173.51	21178.78	23244.51	**22946**	0.9913	0.86925
Q3TPX4	Exoc5	Exocyst complex component 5	15451.87	16600.98	13097.55	14402.44	**14888**	14532.41	15553.66	10793.48	14359.76	**13810**	1.0781	0.43177
Q921W4	Cryzl1	Quinone oxidoreductase-like protein 1	23280.84	24293.15	24035.25	24723.08	**24083**	22109.15	21593.17	18452.06	21223.37	**20844**	1.1554	0.00993
Q8BGP6	Slc25a40	Probable mitochondrial glutathione transporter SLC2	11586.67	8382.656	15892.19	17192.71	**13264**	15765.99	14449.58	19252.92	19810.32	**17320**	0.7658	0.14306
O88507	Cntfr	Ciliary neurotrophic factor receptor subunit alpha	35025.91	35027.86	21812.54	27765.65	**29908**	23709.59	31911.47	19668.56	19694.43	**23746**	1.2595	0.20216
Q64455	Ptprj	Receptor-type tyrosine-protein phosphatase eta												
Q8CG72	Adprs	ADP-ribosylhydrolase ARH3	24883.37	23305.03	23674.02	22623.28	**23621**	24141.25	19789.97	24587.89	24584.5	**23276**	1.0148	0.79298
Q8BGT6	Micall1	MICAL-like protein 1	6124.116	3723.793	4392.473		**4747**	2076.403			4095.57	**3086**	1.5382	0.25754
Q3UN02	Lclat1	Lysocardiolipin acyltransferase 1	46039.73	44220.52	47209.3	54957.63	**48107**	52802.75	33446.48	36423.18	51911.72	**43646**	1.1022	0.45558
Q3UMT1	Ppp1r12c	Protein phosphatase 1 regulatory subunit 12C	31605.03	28099.65	23202.03	27796.14	**27676**	26335.87	26913.36	21147.51	20251.16	**23662**	1.1696	0.15079
Q9WTP7	Ak3	GTP:AMP phosphotransferase AK3, mitochondrial	169413.6	182079.9	185390.3	211855.6	**187185**	182004.1	185235.1	199952.8	203277	**192617**	0.9718	0.61887
Q9ERU9	Ranbp2	E3 SUMO-protein ligase RanBP2	64111.39	67839.36	66366.45	66494.5	**66203**	65911.61	73019.31	68960.84	72542.3	**70109**	0.9443	0.07762
P15626	Gstm2	Glutathione S-transferase Mu 2	237643.5	244909.8	234216.8	206960.3	**230933**	223181.4	205758.5	175660.1	179351	**195988**	1.1783	0.04674
Q8BR92	Palm2	Paralemmin-2	15818.3	13302.29			**14560**	9260.089	10622.96			**9942**	1.4646	0.08403
Q8BTG7	Ndrg4	Protein NDRG4	93429.02	105350.9	99506.81	78604.03	**94223**	92045.08	94984.52	92267.14	91336.65	**92658**	1.0169	0.79650
P12023	App	Amyloid-beta precursor protein	45414.91	43241.51	42589.17	57013.23	**47065**	47561.42	48208.96	54510.75	52760.95	**50761**	0.9272	0.36557
Q8C561	Lmbrd2	G-protein coupled receptor-associated protein LMBRD2									592.8536	**593**	0.0000	
Q4ACU6	Shank3	SH3 and multiple ankyrin repeat domains protein 3	4739.972		5091.613		**4916**							
Q58A65	Spag9	C-Jun-amino-terminal kinase-interacting protein 4	46901.7	44993.78	44737.64	45393.29	**45507**	46117.77	44342.44	53664.19	50606.47	**48683**	0.9348	0.19444
Q9D338	Mrpl19	Large ribosomal subunit protein bL19m	15686.16	18102.77	28428.13	15354.34	**19393**	21102.08	19081.5	15822.11	14307.26	**17578**	1.1032	0.61654
P16330	Cnp	2',3'-cyclic-nucleotide 3'-phosphodiesterase	1738652	1513268	1528673	1811235	**1647957**	1611005	1690786	1530277	1595115	**1606796**	1.0256	0.63281
O88455	Dhcr7	7-dehydrocholesterol reductase	72692.61	76866.7	103052.9	85537.51	**84537**	87230.12	87733.73	108173.4	121969.1	**101277**	0.8347	0.17210
Q9CQK7	Rwdd1	RWD domain-containing protein 1	16946.71	17724.24	19263.16	18247.44	**18045**	16540.35	17202.92	16295.97	16465.63	**16626**	1.0854	0.03545
P58059	Mrps21	Small ribosomal subunit protein bS21m	26353.73	23084.32	26784.7	25293.07	**25379**	28315.64	28283.03	25266.53	26735.03	**27150**	0.9348	0.15900
Q6PF93	Pik3c3	Phosphatidylinositol 3-kinase catalytic subunit type :	22150.15	24086.78	30240.7	24322.99	**25200**	28112.31	22658.56	31731.99	27459.46	**27491**	0.9167	0.40485
Q7M764	Usp17le	Ubiquitin carboxyl-terminal hydrolase 17-like protein E						5333.324	4286.438			**4810**	0.0000	
D3YXK2	Safb	Scaffold attachment factor B1	58821.86	64829.64	73065.48	69743.13	**66615**	63211.98	63739.31	70572.3	72832.98	**67589**	0.9856	0.81268
Q9CR60	Golt1b	Vesicle transport protein GOT1B	37965.79	35394.83	36572.13	45244.13	**38794**	42819.19	39950.28	50201.69	46743.43	**44929**	0.8635	0.09946
Q5PR69	Cracd	Capping protein-inhibiting regulator of actin dynamic	16726.04	18344.47			**17535**	23043.65	18008.53		21415.17	**20822**	0.8421	0.20011
Q9CR68	Uqcrfs1	Cytochrome b-c1 complex subunit Rieske, mitochon	198716.5	207912.3	208690.6	247167	**215622**	199690.9	238603.7	229873.4	200391.3	**217140**	0.9930	0.92115
Q61696	Hspa1a	Heat shock 70 kDa protein 1A	50545.91	52213.62	54292.96	61825.24	**54719**	39197.45	59187.5	65184.75	64511.81	**57020**	0.9596	0.73851
P98192	Gnpat	Dihydroxyacetone phosphate acyltransferase	21871.65	21251.59	22165.65	22375.97	**21916**	21838.24	21964.43	20072.43	28136.02	**23003**	0.9528	0.56430
O88413	Tulp3	Tubby-related protein 3	19994.43	19673.84	21120.96	15526.89	**19079**	22073.01	19606.18	14044.2	13446.21	**17292**	1.1033	0.49189
Q5DQR4	Stxbp5l	Syntaxin-binding protein 5-like	11871.67	12093.41	10307.07	13249.2	**11880**	12071.04	14683.08	7794.056	11173.9	**11431**	1.0394	0.78085
O54950	Prkag1	5'-AMP-activated protein kinase subunit gamma-1	23600.87	23701.22	26852.04	26413.89	**25142**	25612.73	25162.86	18662.41	27551.28	**24247**	1.0369	0.68736
Q6A0A9	FAM120A	Constitutive coactivator of PPAR-gamma-like protein	58047.23	54768.13	59842.08	63891.58	**59137**	59003.68	58846	69877.69	70561.68	**64572**	0.9158	0.20022
Q06185	Atp5me	ATP synthase subunit e, mitochondrial	534163.8	455513.7	556510.5	599581.2	**536442**	562493.3	585373.6	568613.6	570001.8	**571621**	0.9385	0.29389
Q9D7A6	Srp19	Signal recognition particle 19 kDa protein	38557.16	41284.25	47712.13	42227.05	**42445**	50637.08	37891.76	50959.9	42448.41	**45484**	0.9332	0.44722
Q922P9	Glyr1	Cytokine-like nuclear factor N-PAC	20591.9	18996.96	18149.25	21946.46	**19921**	15066.03	18046.7	21118.04	21510.53	**18935**	1.0521	0.58839
Q8BMS1	Hadha	Trifunctional enzyme subunit alpha, mitochondrial	409981.1	387756.1	406587.3	465516.7	**417460**	406627.5	432669.4	417594.8	434006.4	**422725**	0.9875	0.77950
Q8CHT0	Aldh4a1	Delta-1-pyrroline-5-carboxylate dehydrogenase, mit	82413.09	78215.4	84816.34	97687.23	**85783**	83497.48	86769.54	78354.19	78241.33	**81716**	1.0498	0.41864
Q8R2U6	Nudt4	Diphosphoinositol polyphosphate phosphohydrolase	98187.13	43332.36	34616.86	60169.73	**59077**	28988.2	30072.53	17369.56	30641.47	**26768**	2.2070	0.06634
Q9JKS5	Habp4	Intracellular hyaluro-binding protein 4	31185.07	31820.38	29986.82	36340.31	**32333**	32730.25	35890.14	38165.93	36873.77	**35915**	0.9003	0.09503
A2AN08	Ubr4	E3 ubiquitin-protein ligase UBR4	77777.1	82585.9	83277.67	83450.69	**81773**	81070.24	83129.09	81100.4	86153.3	**82863**	0.9868	0.56706
O08908	Pik3r2	Phosphatidylinositol 3-kinase regulatory subunit bet	5144.739		3677.758	1994.074	**3606**	5991.58	3201.866	4146.292	3787.625	**4282**	0.8420	0.54528
Q922S4	Pde2a	cGMP-dependent 3',5'-cyclic phosphodiesterase							5545.229			**5545**	0.0000	
Q8BTJ4	Enpp4	Bis(5'-adenosyl)-triphosphatase enpp4	42861.46	47065.35	48846.14	48950.92	**46931**	50910.03	42001.57	59149.13	57631.48	**52423**	0.8952	0.23482
Q91V14	Slc12a5	Solute carrier family 12 member 5	24355.09	21686.3	14642.29	38603.51	**24822**	14445.96	14402.07	25197.19	26832.54	**20219**	1.2276	0.47568
Q4QQM4	Trp53i11	Tumor protein p53-inducible protein 11	24524.38	19442.43	21149.83	22420.47	**21884**	20388.91	22966.74	13469.82	16617.96	**18361**	1.1919	0.18381
Q8R1S0	Coq6	Ubiquinone biosynthesis monooxygenase COQ6, mi	8535.271	7141.936	10387.94	10599.71	**9166**	7194.367	8761.46	10724.01	6507.447	**8297**	1.1048	0.51077
Q923S9	Rab30	Ras-related protein Rab-30	8686.562		21211.28	18462.56	**16120**	24378.72	22589.02	7903.403	17418.42	**18072**	0.8920	0.73285
A2A863	Itgb4	Integrin beta-4	54714.09	53946.02	49850.2	63083.74	**55399**	52579.83	53522.88	54086.48	53564.02	**53438**	1.0367	0.50907
P09103	P4hb	Protein disulfide-isomerase	394832.8	386879.5	419215.3	407056.4	**401996**	395028.3	418062.1	420297.2	451437.8	**421206**	0.9544	0.20682
Q80V26	Bpnt2	Golgi-resident adenosine 3',5'-bisphosphate 3'-phos	51326.8	38360.97	37631.15	40459.27	**41945**	35254.08	42299.73	41019.7	38489.69	**39266**	1.0682	0.47826
P61793	Lpar1	Lysophosphatidic acid receptor 1	65864.94	66428.13	85821.44	78299.9	**74104**	91559.2	86401.04	104281.3	77749.77	**89998**	0.8234	0.07431
P35922	Fmr1	Fragile X messenger ribonucleoprotein 1	41719.36	40203.46	46597.45	52338.35	**45215**	40544.91	44888.58	50335.5	48709.3	**46120**	0.9804	0.80467
P24788	Cdk11b	Cyclin-dependent kinase 11B	19735.9	10326.72	11066.8	12741.15	**13468**	12029.92	16822.57	10582.61	6946.179	**11595**	1.1615	0.55121
Q8VHN8	Nudt16l1	Tudor-interacting repair regulator protein	68650.34	62347.02	57194.58	68432.34	**64156**	64076.43	71627.05	66967.53	68952.7	**67906**	0.9448	0.28180
Q99J39	Mlycd	Malonyl-CoA decarboxylase, mitochondrial	13098.56	12382.78	15711.25	14828.72	**14005**	11492.21	7357.899	12180.77	14611.95	**11411**	1.2274	0.17585

Q8K0U4	Hspa12a	Heat shock 70 kDa protein 12A	1709443	1712533	1328471	1722637	**1618271**	1637050	1656706	1416429	1441552	**1537934**	1.0522	0.51270
O55100	Syngr1	Synaptogyrin-1	111124.9	135303.6	90557.16	115291.4	**113069**	68632.48	80816.97	138737.2	159590.4	**111944**	1.0100	0.96395
Q8K341	Atat1	Alpha-tubulin N-acetyltransferase 1	3581.717	9963.675	18716.72	9300.841	**10391**		9585.145		8208.835	**8897**	1.1679	0.76670
Q8BGB7	Enoph1	Enolase-phosphatase E1	18670.25	18440.25	21676.97	19684.68	**19618**	26801.08	21478.4	28880.42	24614.75	**25444**	0.7710	0.01569
P62835	Rap1a	Ras-related protein Rap-1A	526593.1	485724.3	439544	640968.6	**523208**	525386.6	515913.8	470191.9	455521.1	**491753**	1.0640	0.52265
Q9JIS5	Sv2a	Synaptic vesicle glycoprotein 2A	17281.7	2157.164	3197.425		**7545**		1025.327			**1025**	7.3590	
E9Q6B2	Ccdc85c	Coiled-coil domain-containing protein 85C			487.1393		**487**							
O35604	Npc1	NPC intracellular cholesterol transporter 1	33476.46	39552.97	40641.96	35361.55	**37258**	38786.39	37785.51	35008.18	41760.97	**38335**	0.9719	0.64139
O08539	Bin1	Myc box-dependent-interacting protein 1	76560.94	79583.38	64527.92	63792.91	**71116**	66631.3	75065.9	65388.24	62171.61	**67314**	1.0565	0.46798
P27659	Rpl3	Large ribosomal subunit protein uL3	403740.6	394688	446931.1	453671.8	**424758**	423966	434815.5	480162.8	489647.4	**457148**	0.9291	0.19305
Q924T7	Rnf31	E3 ubiquitin-protein ligase RNF31	904.3571				**904**	1448.276		1761.911		**1605**	0.5634	
Q9DCS9	Ndufb10	NADH dehydrogenase [ubiquinone] 1 beta subcomp	186884.7	192817.5	197496.1	214155.6	**197838**	185564.8	181969	208766.5	192010.1	**192078**	1.0300	0.51555
Q9D358	Acp1	Low molecular weight phosphotyrosine protein phos	171635.3	177546.9	146459.5	138662.1	**158576**	163295.5	159868.7	119039.4	117743.1	**139987**	1.1328	0.28030
Q9EQH3	Vps35	Vacuolar protein sorting-associated protein 35	195160.3	206407.3	197715.8	183187.3	**195618**	186765.5	199563.2	177159.2	178192.6	**185420**	1.0550	0.19865
Q5FWH7	Slc39a12	Zinc transporter ZIP12												
P63166	Sumo1	Small ubiquitin-related modifier 1	71304.31	58725.14	55873.57	43331.09	**57309**	47897.35	68257.78	70850.67	68293.33	**63825**	0.8979	0.43782
Q9Z2B2	Slc25a14	Brain mitochondrial carrier protein 1	1454.309	1961.681	3250.157	2512.172	**2295**		2394.033	2656.614	4628.249	**3226**	0.7112	0.26652
Q8CCP0	Nemf	Ribosome quality control complex subunit NEMF	19752	19176.93	20651.42	22668.89	**20562**	20312.9	18780.95	23572.79	20874.35	**20885**	0.9845	0.80603
Q9CZM2	Rpl15	Large ribosomal subunit protein eL15	240176.9	243969.7	252111.9	269236.1	**251374**	253519.7	248890.5	293847.2	284253.2	**270128**	0.9306	0.19544
P01868;P01869	Ighg1	Ig gamma-1 chain C region secreted form;Ig gamma-	171194.8	77845.37	53711.68	79926.15	**95669**	50368.02	111234.5	39926.01	32442.55	**58493**	1.6356	0.28247
P59648	Fxyd7	FXYD domain-containing ion transport regulator 7	92709.37	115372	101612.2	137729.7	**111856**	102419	129934	169360.6	146304	**137004**	0.8164	0.19309
Q8R2Q4	Gfm2	Ribosome-releasing factor 2, mitochondrial	12473.46	13802.35	12414.77	14781.46	**13368**	12597.29	14633.01	11993.84	14472.64	**13424**	0.9958	0.95088
Q9JI10	Stk3	Serine/threonine-protein kinase 3	37740.57	31706.25	36243.79	28173.98	**33466**	30553.14	27579.8	27531.92	31402.24	**29267**	1.1435	0.13083
Q8BXQ2	Pigt	GPI transamidase component PIG-T	38052.71	32619.37	35752.71	38433.36	**36215**	35869.21	36848.41	35213.23	37932.78	**36466**	0.9931	0.86918
Q7TQ95	Lnpk	Endoplasmic reticulum junction formation protein lu	82924.06	87756.24	95457.48	104667.9	**92701**	92501.53	88632.44	87960.74	105447	**93635**	0.9900	0.88612
A2APY7	Ndufaf5	Arginine-hydroxylase NDUFAF5, mitochondrial	15004.11	13135.66	11797.82	13481.46	**13355**	15499.41	14646.37	15301.18	13206.01	**14663**	0.9108	0.16969
P01872	Ighm	Immunoglobulin heavy constant mu	171377.3	54433.32	21123.6	14081.52	**65254**	40140.98	60805.41	8274.479	7042.658	**29066**	2.2450	0.38608
Q99L13	Hibadh	3-hydroxyisobutyrate dehydrogenase, mitochondrial	70913.55	76101.02	80223.36	83716.41	**77739**	63471.17	77230.22	70512.09	64618.46	**68958**	1.1273	0.08112
Q8R574	Prpsap2	Phosphoribosyl pyrophosphate synthase-associatec	59927.57	55540.74	57679.61	52262.78	**56353**	54942.09	51338.71	47746.29	48885	**50728**	1.1109	0.04861
Q91W67	Ubl7	Ubiquitin-like protein 7	11807.45	11658.58	11181.29	10740.09	**11347**	9542.592	11923.15	11933.58	11575.65	**11244**	1.0092	0.87383
P59672	Anks1a	Ankyrin repeat and SAM domain-containing protein 1	22092.44	20953.45	27979.37	20486.66	**22878**	24874.27	21803.49	25312.54	24959.69	**24237**	0.9439	0.50469
P60843	Eif4a1	Eukaryotic initiation factor 4A-I	1116887	1140618	1202508	1037266	**1124320**	1117147	1139183	1211826	1126678	**1148708**	0.9788	0.56800
Q3UHX2	Pdap1	28 kDa heat- and acid-stable phosphoprotein	53746.48	50701.08	55734.55	55898.82	**54020**	51889.89	49593.23	56597.93	57124.6	**53801**	1.0041	0.92382
Q8C547	Heatr5b	HEAT repeat-containing protein 5B	9953.681	9974.816	8815.059	6735.955	**8870**	9378.134	9612.434	7213.992	8059.974	**8566**	1.0355	0.75959
Q8K298	Anln	Anillin	15696.21	22281.93	28170.52	18071.19	**21055**	21029.37	15256.86	11356.61	26930.51	**18643**	1.1294	0.60061
Q91YJ5	Mtif2	Translation initiation factor IF-2, mitochondrial	11672.98	10459.81	14743.33	11763.32	**12160**	10468.81	7899.751	13928.76	11663.29	**10990**	1.1064	0.47931
Q64332	Syn2	Synapsin-2	37675.19	37895.6	46531.78	38255.41	**40089**	43096.88	43405.37	35663.33	37147	**39828**	1.0066	0.93199
Q3USB7	Plcl1	Inactive phospholipase C-like protein 1	6302.531	11397.21	11324.52	9268.2	**9573**	7708.628	10350.55	8210.709	8460.246	**8683**	1.1026	0.52765
Q9D8U2	Tmem41a	Transmembrane protein 41A	14374.29	17909	15612.33	20268.88	**17041**	14885.77	17535.96	14794.28		**15739**	1.0828	0.48154
Q8R3V5	Sh3glb2	Endophilin-B2	87319.05	96884.96	88508.92	84782.57	**89374**	87788.91	85478.48	99113.42	91240.27	**90905**	0.9832	0.71298
P26450	Pik3r1	Phosphatidylinositol 3-kinase regulatory subunit alpl	13029.58	17071.32	13502.07	11823.84	**13857**	18469.67	13366.54	16063.8	15052.19	**15738**	0.8805	0.27125
P32020	Scp2	Sterol carrier protein 2	139258.9	132592.9	149485.5	148712.2	**142512**	134546.1	152104	179427	160667.7	**156686**	0.9095	0.21269
Q920N2	Hlcs	Biotin--protein ligase	49926.9	47347.87	39538.75	42825.98	**44910**	48845.19	34317.84	21047.11	22630.55	**31710**	1.4163	0.10174
Q62108	Dlg4	Disks large homolog 4	10814.19	12631.59	9532.65	11793.93	**11193**	12091.81	12476.53	10990.33	12204.53	**11941**	0.9374	0.35273
Q9D0K2	Oxct1	Succinyl-CoA:3-ketoacid coenzyme A transferase 1,	125206	115197.1	118378.2	143172.2	**125488**	123646.4	130821.3	128217.9	143367	**131513**	0.9542	0.45497
Q91Y63	Slc13a3	Na(+)/dicarboxylate cotransporter 3	20723.15	16837.79	5161.837	11935.59	**13665**	16178.19	1302.317			**8740**	1.5634	0.50841
Q3UJP5	Cfap418	Cilia- and flagella-associated protein 418												
Q8BHG2	Czib	CXXC motif containing zinc binding protein	62241.63	56295.08	58929.98	41972.91	**54860**	46972.36	54089.36	65325.34	52415.91	**54701**	1.0029	0.97935
P62823	Rab3c	Ras-related protein Rab-3C								27765.22		**27765**	0.0000	
Q8VD62	Bles03	UPF0696 protein C11orf68 homolog	26904.74	29205.96	25776.3	20015.12	**25476**	28222.03	31549.16	26476.28	29317.15	**28891**	0.8818	0.17561
Q5SSH8	Cyb5d2	Neuferricin	26935.13	24088.11	26521.17	24788.49	**25583**	26078.01	28908.49	23777.58	29883.5	**27162**	0.9419	0.34642
Q91Z38	Ttc1	Tetratricopeptide repeat protein 1	62587.35	76735.42	63542.27	61352.49	**66054**	67574.48	64527.67	60123.84	68156.65	**65096**	1.0147	0.81996
P47738	Aldh2	Aldehyde dehydrogenase, mitochondrial	634590.4	596448.6	749170.9	813059.9	**698317**	689782.3	622452.9	775369.8	757783.3	**711347**	0.9817	0.83822
Q6A065	Cep170	Centrosomal protein of 170 kDa	31609.98	33615.32	33950.29	39848.77	**34756**	34311.36	33093.41	35486.89	36659.28	**34888**	0.9962	0.94791
P56959	Fus	RNA-binding protein FUS	187875	166412.1	219428.8	214508.8	**197056**	187307.8	175208.8	229180.3	221972	**203417**	0.9687	0.73600
P61264	Stx1b	Syntaxin-1B	99048.73	96757.02	83732.63	93817.46	**93339**	92742.38	103561.4	83038.81	81447.22	**90197**	1.0348	0.62616
Q8BWF0	Aldh5a1	Succinate-semialdehyde dehydrogenase, mitochonc	30358.24	29811.54	33110.68	32394.08	**31419**	26656.44	34565.7	25164.07	25248.92	**27909**	1.1258	0.19084
Q8CGF7	Tcerg1	Transcription elongation regulator 1	444463.7	416460.7	449423.8	495227.5	**451394**	445544.3	439219.7	508234.3	483267.8	**469067**	0.9623	0.47227
Q6Q477	Atp2b4	Plasma membrane calcium-transporting ATPase 4	111917.3	109565.9	107849.3	117820.1	**111788**	101138.6	89158.16	109872.6	102474.4	**100661**	1.1105	0.05996

O35118	Gfra3	GDNF family receptor alpha-3	4769.732	9252.739	10780.64	7648.377	8113	7426.474	7670.122	7592.498	5528.299	7054	1.1501	0.47300
Q149F3	Gspt2	Eukaryotic peptide chain release factor GTP-binding	53210.26	51240.95	56651.35	54522.43	53906	52646.13	52672.84	52868.74	52681.31	52717	1.0226	0.33628
Q8BTZ4	Anapc5	Anaphase-promoting complex subunit 5	9027.461	9440.158	12428.62	7038.941	9484	10918.35	9432.721	6233.311	9660.419	9061	1.0466	0.78686
Q62179	Sema4b	Semaphorin-4B	12183.53	10530.73	9149.895	9720.183	10396	10429.48	13186.61	9579.869	13279.5	11619	0.8948	0.33052
Q71RI9	Kyat3	Kynurenine--oxoglutarate transaminase 3	35232.02	41191.4	36862.46	41275.77	38640	35648.08	40118.58	26147.49	30787.29	33175	1.1647	0.15774
Q8C437	Pex5l	PEX5-related protein	33323	32188.12	26220.91	27834.94	29892	29659.56	32854.47	33490.88	32254.68	32065	0.9322	0.29584
Q9WV85	Nme3	Nucleoside diphosphate kinase 3	28320.01	30673.51	42139	28197.24	32332	27550.54	31191.23	29353.87	33656.52	30438	1.0622	0.61429
O88746	Tom1	Target of Myb1 membrane trafficking protein	73569.08	72394.25	72245.91	74292.66	73125	78127.64	80301.63	75946.94	74968.42	77336	0.9456	0.01691
Q8BSS9	Ppfia2	Liprin-alpha-2	24182.35	24013.47	25708.91	23336.36	24310	21699.95	20425.81	22251.16	22945.76	21831	1.1136	0.01468
Q8BZA9	Tigar	Fructose-2,6-bisphosphatase TIGAR	40472.9	42502.82	37372.27	28884.13	37308	42357.67	35312.53	33031.01	34381.77	36271	1.0286	0.78590
Q9DBR4	Apbb2	Amyloid beta precursor protein binding family B member 2									1339.329	1339	0.0000	
O35943	Fxn	Frataxin, mitochondrial	3790.502	76773.2	70479.42	26256.74	44325			37156.29	18274.71	27715	1.5993	0.57156
Q8VHK9	Dhx36	ATP-dependent DNA/RNA helicase DHX36	18235.69	17166.82	15476.47	16254.68	16783	14405.18	16765.56	13634.06	18306.93	15778	1.0637	0.44420
Q9D0G0	Mrps30	Large ribosomal subunit protein mL65	13141.58	10030.75	11634.68	13857.29	12166	13388.47	12291.67	10724.01	12848.95	12313	0.9880	0.89058
Q9CPY7	Lap3	Cytosol aminopeptidase	200329.4	176620.1	185558.5	175720.8	184557	177240.1	161674.6	191809.4	189826.1	180138	1.0245	0.64052
Q6PDI6	Mindy2	Ubiquitin carboxyl-terminal hydrolase MINDY-2	12650.47	9941.016	15177.46	9144.133	11728	10277.08	11687.57	10285.12	10279.07	10632	1.1031	0.46868
Q9DA03	Lyrm7	Complex III assembly factor LYRM7		8262.144			8262		10835.5			10835	0.7625	
Q8CGZ0	Cherp	Calcium homeostasis endoplasmic reticulum protei	18466.78	16504	16545.94	16925.98	17111	17289.62	17451.67	18159.05	20876.21	18444	0.9277	0.21082
Q8R326	Pspc1	Paraspeckle component 1	55956.95	54756.48	53826.94	58137.32	55669	61142.91	56871.85	56322.05	52949.32	56822	0.9797	0.57078
Q9R226	Khdrbs3	KH domain-containing, RNA-binding, signal transduc	19650.84	26487.04	24658.94	23061.4	23465	22722.22	28786.54	31534.35	24281.33	26831	0.8745	0.22564
Q91XD7	Creld1	Protein disulfide isomerase Creld1	64880.84	84219.78	83001.38	85285.66	79347	77461.14	92587.34	102393.6	89554.27	90499	0.8768	0.16534
Q9Z2G9	Htatip2	Oxidoreductase HTATIP2	48411.65	45492.72	50654.8	54776.11	49834	46652.89	50609.09	55108.91	55395.54	51942	0.9594	0.48791
Q9CRC8	Lrrc40	Leucine-rich repeat-containing protein 40	11694.02	12522.4	12187.68	4819.104	10306	8845.852	11883.33	6911.947	7149.593	8698	1.1849	0.48565
Q6GQS1	Slc25a23	Mitochondrial adenyl nucleotide antiporter SLC25A2	26831.99	23577.43	24773.61	24413.5	24899	27653.69	17464.82	22783.07	24117.14	23005	1.0824	0.42689
Q3V3R1	Mthfd1l	Monofunctional C1-tetrahydrofolate synthase, mitoc	81821.96	92952.68	96267.45	105662.9	94176	93976.56	99267.17	94572.11	102456.6	97568	0.9652	0.54700
Q3TPE9	Ankmy2	Ankyrin repeat and MYND domain-containing protein	13186.88	9998.219	13057.06	9047.28	11322	10282.61	8525.891	11423.53	8700.829	9733	1.1633	0.25456
O88207	Col5a1	Collagen alpha-1(V) chain	100081.2	106872	98211.41	99365.16	101132	91576.46	108362.3	175557.3	112073.6	121892	0.8297	0.30560
Q62186	Ssr4	Translocon-associated protein subunit delta	103247.4	101666.8	116050.3	120809.7	110444	110463.9	114461.6	145806.2	130018	125187	0.8822	0.16571
Q64105	Spr	Sepiapterin reductase	142910.5	162179	144114.8	137105.9	146578	165772.3	140723.9	133677.1	131131	142826	1.0263	0.70923
Q9WVA2	Timm8a1	Mitochondrial import inner membrane translocase s	44721.81	45761.81	44593.71	44768.33	44711	43673.32	56898.62	50002.63	47829.37	49601	0.9014	0.13129
O88703	Hcn2	Potassium/sodium hyperpolarization-activated cycli	36610.91	37051.58	20203.69	28687.47	30638	31495.21	16788.94	20257.42	12153.89	20174	1.5187	0.11739
Q9DAW9	Cnn3	Calponin-3	241561.5	237782.8	254233.4	194237.6	231954	225140.2	209944	231553.4	235010.7	225412	1.0290	0.66091
Q8K3J1	Ndufs8	NADH dehydrogenase [ubiquinone] iron-sulfur prote	36482.55	38059.17	30392.04	31274.49	34052	28953.59	43513.14	27583.03	33022.88	33268	1.0236	0.85371
Q8JZX4	Rbm17	Splicing factor 45	7317.691	7649.163	6269.841	7613.522	7213	6541.646	9768.56	10652.9	9166.803	9032	0.7985	0.10147
Q9CX30	Yif1b	Protein YIF1B	10184.62	10746.94	12781.44	14353.22	12017	10843.01	17734.48	15794.77	16751.51	15281	0.7864	0.12075
Q3UU96	Cdc42bpa	Serine/threonine-protein kinase MRCK alpha	29027.81	29448.65	30189.35	34433.16	31025	33010.17	33622.04	30975.44	33541.93	32787	0.9462	0.24746
Q8K370	Acad10	Acyl-CoA dehydrogenase family member 10	11608.88	13136.9	14340.12	13635.31	13180	13156.47	13711.23	11741.31	11873.47	12621	1.0443	0.48623
P01942	Hba	Hemoglobin subunit alpha	2894541	3361334	4170758	3119862	3386624	2429844	2297345	3675608	2286542	2672335	1.2673	0.15266
Q9CRY7	Gdpd1	Lysophospholipase D GDPD1	39436.52	40743.93	43582.81	45772.5	42384	41529.29	44428.67	50154.37	47016.82	45782	0.9258	0.19411
Q8BFP9	Pdk1	[Pyruvate dehydrogenase (acetyl-transferring)] kinas	20746.07	26029.69	26833.71	28668.07	25569	29059.86	23005.31	19782.79	20805.6	23163	1.1039	0.40459
Q8BMB3	Eif4e2	Eukaryotic translation initiation factor 4E type 2	16980.63	18122.95	22124.31	23519.77	20187	19481.07	19065.49	21469.63	23533.09	20887	0.9665	0.72111
Q8BIQ5	Cstf2	Cleavage stimulation factor subunit 2	25786.89	26421.54	24770.53	26331.66	25828	27778.59	21272.13	22544.41	26497.05	24523	1.0532	0.44590
O88704	Hcn1	Potassium/sodium hyperpolarization-activated cycli	8863.465	13437.68	7727	11834.14	10466	15846.54	17885.64	7384.133	10503.76	12905	0.8110	0.40853
Q8VEE4	Rpa1	Replication protein A 70 kDa DNA-binding subunit	33726.63	37136.54	44728.62	32684.15	37069	41514.97	26283.53	37635.98	44329.13	37441	0.9901	0.94088
Q9Z1M0	P2rx7	P2X purinoceptor 7	143085.3	123591.2	102669.6	149091.8	129609	138014	151375.3	116509.4	115985.3	130471	0.9934	0.95157
P31786	Dbi	Acyl-CoA-binding protein	611228.7	569152.5	705501.6	628622.3	628626	587610.3	464133.6	695090.1	635860.1	595673	1.0553	0.58246
P00329	Adh1	Alcohol dehydrogenase 1	17771.86	20441.64	23247.88	23002.91	21116	16364.1	13543.24	15707.32	17374.77	15747	1.3409	0.01225
Q9CWS0	Ddah1	N(G),N(G)-dimethylarginine dimethylaminohydrolas	666139	534858.1	373397	530654.3	526262	490878	634286.3	333549.1	385220.7	460984	1.1416	0.49284
P61967	Ap1s1	AP-1 complex subunit sigma-1A	25330.9	25430.17	28544.47	27974.46	26820	26033.27	23528.04	31468.71	27761.64	27198	0.9861	0.84629
P62908	Rps3	Small ribosomal subunit protein uS3	1202401	1148065	1333238	1346666	1257592	1234260	1258413	1453597	1491021	1359323	0.9252	0.26123
Q9DCN2	Cyb5r3	NADH-cytochrome b5 reductase 3	605829.3	586346.6	623168.8	670398.6	621436	571826.7	645464.2	621579.9	705497.8	636092	0.9770	0.67313
Q8BLV3	Slc9a7	Sodium/hydrogen exchanger 7	8267.725	6787.935		9501.96	8186	8792.133	8122.139	4098.89		7004	1.1687	0.51649
P17918	Pcna	Proliferating cell nuclear antigen	28506.72	22105.8	22647.91	21561.26	23705	23148.83	23935.26	21898.72	21263.14	22561	1.0507	0.53174
Q9CZC8	Scrn1	Secernin-1	103020.7	111034	94011.28	86439.99	98627	97344.05	103761	86098.64	85842.05	93261	1.0575	0.46823
P0DP60	Lynx1	Ly-6/neurotoxin-like protein 1	36136.43	31104.65	17998.46	38275.43	30879	28657.66	35987.69	15267.86	21696.84	25403	1.2156	0.42318
Q99LS3	Psph	Phosphoserine phosphatase	59585.64	67834.57	67229.56	54312.9	62241	57365.62	61865.98	57533.54	59850.64	59154	1.0522	0.40057
Q62093	Srsf2	Serine/arginine-rich splicing factor 2	116027.3	115034.2	128419.1	116188	118917	113759.1	110268.8	127733.8	119749.2	117878	1.0088	0.84133
Q80UG5	Septin9	Septin-9	324231.3	341247.7	324262.6	310898.5	325160	326778.8	330996.8	314405.5	319022.7	322801	1.0073	0.75609
Q8BM13	Olfm2	Noelin-2				1301.116	1301							

Q9WV69	Dmtn	Dematin	94867.73	90152.55	70235.17	111092.4	91587	81726.14	93999.63	83637.5	78038.1	84350	1.0858	0.45587
Q3ULD5	Mccc2	Methylcrotonoyl-CoA carboxylase beta chain, mitocl	32467	32045.05	34787.55	39438.52	34685	33100.24	34219.66	34898.81	34822.27	34260	1.0124	0.81606
Q9CVB6	Arpc2	Actin-related protein 2/3 complex subunit 2	123955.8	123503.6	120035.9	110953.7	119612	119305.5	126946.9	111870.3	112772.5	117724	1.0160	0.69656
Q9EPW0	Inpp4a	Inositol polyphosphate-4-phosphatase type I A	16220.66	15249.15	13988.53	14153.94	14903	15476.3	14693.16	13633.27	14501.98	14576	1.0224	0.62968
E9PVA8	Gcn1	Stalled ribosome sensor GCN1	44306.27	46305.47	54181.21	43346.41	47035	45237.61	44497.17	49272.84	53416.75	48106	0.9777	0.74977
Q7TNR6	Igsf21	Immunoglobulin superfamily member 21				4171.368	4171							
D3YZG8	Mthfd2l	Bifunctional methylenetetrahydrofolate dehydrogena	5945.056	6408.453	7040.181	7302.198	6674	6829.678	5544.639	4259.995	6990.495	5906	1.1300	0.31925
P08003	Pdia4	Protein disulfide-isomerase A4	123672.5	131285.6	150864.4	140859.7	136671	129235.1	135723.2	147462.8	157392.4	142453	0.9594	0.52584
P07724	Alb	Albumin	9325728	12210253	11052071	3977403	9141364	9390596	7554980	6232379	4632392	6952587	1.3148	0.33345
P12970	Rpl7a	Large ribosomal subunit protein eL8	391007.8	397876.3	438686.8	475899.2	425868	424514.2	412915.7	505842.6	514008.3	464320	0.9172	0.28845
Q3UPL5	Ag2	Uncharacterized protein C11orf96 homolog	18301.65	21126.96	17686.13	17013.67	18532	23104.87	21803.47	19879.68	13052.26	19460	0.9523	0.71372
Q8CJG0	Ago2	Protein argonaute-2	14354	14541.75	15113.23	14500.49	14627	14849.4	14375.59	12552.63	13883.66	13915	1.0512	0.22191
Q9D1I2	Card19	Caspase recruitment domain-containing protein 19	28763.83	31073.49	31547.97	35321.26	31677	31612.41	34166.69	37800.14	35456.27	34759	0.9113	0.15109
Q8BSL7	Arf2	ADP-ribosylation factor 2	29431.88	33370.48	14288.56	12375.03	22366	20324.68	33809.95	12637.19	15602.59	20594	1.0861	0.81025
Q91XV3	Basp1	Brain acid soluble protein 1	60729.93	86333.22	71293.9	85082.05	75860	76082.05	90208.25	81148.87	71284.02	79681	0.9520	0.61983
Q9CXS4	Cenpv	Centromere protein V	61378.82	59047.79	61563.18	72838.73	63707	62151.35	61680.75	51970.57	62277.4	59520	1.0703	0.33472
P28271	Aco1	Cytoplasmic aconitate hydratase	109362.4	113658.3	141373.3	92054.53	114112	109151.9	94966.21	125227.1	123901.4	113312	1.0071	0.95083
Q8BX94	Osbpl2	Oxysterol-binding protein-related protein 2	37741.65	50639.41	36974.12	46702.66	43014	47904.08	45450.76	38392.46	29003.82	40188	1.0703	0.62022
Q8BGZ2	Fam168a	Protein FAM168A												
Q91VR2	Atp5f1c	ATP synthase subunit gamma, mitochondrial	439600.4	419947.8	452671.8	504907.7	454282	449871.8	479738.5	486744.9	493979.8	477584	0.9512	0.30086
Q3U4G3	Xxylt1	Xyloside xylosyltransferase 1	55770.91	48546.83	48075.55	42765.5	48790	53481.7	62087.87	48926.34	9236.619	43433	1.1233	0.67152
Q9D0F9	Pgm1	Phosphoglucomutase-1	221801.7	234985.9	217306.8	175450.4	212386	212674.5	197758	196603.6	180300.5	196834	1.0790	0.32383
Q80XR2	Atp2c1	Calcium-transporting ATPase type 2C member 1	37553.21	37811.78	35941.41	40185.51	37873	38163.66	39241.81	39019.64	47742.37	41042	0.9228	0.23656
Q8CHP8	Pgp	Glycerol-3-phosphate phosphatase	137497.8	150108.9	139358.2	109211.9	134044	128847.3	149074	126591.4	125395.2	132477	1.0118	0.88473
Q5SWT3	Slc25a35	Solute carrier family 25 member 35	16763.7	12953.83	20986.95	19271.7	17494	14335.04	16207.67	14857.23	20853.45	16563	1.0562	0.69850
P32067	Ssb	Lupus La protein homolog	138881.3	125932.5	151071.9	147354.1	140810	144665.2	137638.6	159616	152636.5	148639	0.9473	0.32715
Q8R0F6	Ilkap	Integrin-linked kinase-associated serine/threonine p	15999.81	15599.25	20606.01	13750.28	16489	16811.25	14551.6	15908.87	16402.11	15918	1.0358	0.72346
Q9DCT5	Sdf2	Stromal cell-derived factor 2	23386.53	23008.21	28434.46	27268.75	25524	24899.78	21463.41	32807.81	36125.14	28824	0.8855	0.40263
Q99JW2	Acy1	Aminoacylase-1	42530.48	32347.69	30368.66	29982.48	33807	29365.93	39627.59	27132.54	26787.93	30728	1.1002	0.49361
P63030	Mpc1	Mitochondrial pyruvate carrier 1	32101.38	41861.4	44403.97	41124.38	39873	35478.86	39037.75	53124.1	52725.62	45092	0.8843	0.36361
P97411	Ica1	Islet cell autoantigen 1	8823.533	12327.83	8927.129	14323	11100	12286.43		25491.17	11810.03	16529	0.6716	0.24035
Q3UFS0	Zyg11b	Protein zyg-11 homolog B	19006.25	21367.9	19543.84	25672.36	21398	31037.2	20705.74	18254.48	23644.71	23411	0.9140	0.54719
P12658	Calb1	Calbindin	135496.4	162238.8	127122.8	41759.46	116654	70614.45	114877.6	98787.28	99513.64	95948	1.2158	0.48222
Q8BML9	Qars1	Glutamine--tRNA ligase	129842.4	122875.4	140142.4	147598.4	135115	133714.2	143468.9	155978.1	150647.2	145952	0.9257	0.18752
Q9R0P6	Sec11a	Signal peptidase complex catalytic subunit SEC11A	119543.1	105032.2	126301.5	117766.1	117161	106553.1	112833.3	121472.4	135417.5	119069	0.9840	0.81172
Q9EPU0	Upf1	Regulator of nonsense transcripts 1	83591.07	89723.77	92154.98	95526.57	90249	92208.63	89766.8	103277.7	102277.9	96883	0.9315	0.17110
Q8K4K6	Pank1	Pantothenate kinase 1	45364.86	46815.88	57111.73		49764	40235.88	22874.73		41601.35	34904	1.4257	0.10348
C0HKE1;C0HKE2;C0HKE	H2ac4;H2ac6;H2ac7;H2ac8;	Histone H2A type 1-B;Histone H2A type 1-C;Histone	10462302	9158762	12089513	14891458	11650509	10846148	7833311	13623696	14626861	11732504	0.9930	0.96804
Q9DB16	Cab39l	Calcium-binding protein 39-like	132514	132063.6	118189.2	104604	121843	123781.5	119580.1	115248	112714.5	117831	1.0340	0.59112
P50518	Atp6v1e1	V-type proton ATPase subunit E 1	185583.4	178117.4	161825.7	172496.7	174506	176858.3	185348.5	143736.2	154709.1	165163	1.0566	0.42235
Q03173	Enah	Protein enabled homolog	26730.8	28509.01	29204.57	30005.98	28613	23482.43	24128.68	26116.85	27580.81	25327	1.1297	0.03069
Q9D920	Borcs5	BLOC-1-related complex subunit 5	45599.57	52357.13	45798.54	45310.77	47267	45048.59	54534.57	48751.55	52968.53	50326	0.9392	0.30594
Q8VE70	Pdcd10	Programmed cell death protein 10	48830.01	52668.02	49337.55	42903.4	48435	48512.68	48236.78	48431.99	49289.65	48618	0.9962	0.93156
Q922H1	Prmt3	Protein arginine N-methyltransferase 3	16436.88	17530.81	16448.29	15276.16	16423	17100.43	15529.27	12705.88	17594.24	15732	1.0439	0.58378
Q5M8N4	Sdr39u1	Epimerase family protein SDR39U1	43179.49	36574.69	37921.48	47855.45	41383	37939.15	47451.74	38155.64	38280.41	40457	1.0229	0.79920
Q8K2C9	Hacd3	Very-long-chain (3R)-3-hydroxyacyl-CoA dehydratas	164354.9	187118	206835.4	225949.9	196065	183051.8	208001.7	209771.2	212809.8	203409	0.9639	0.63929
Q9D924	Isca1	Iron-sulfur cluster assembly 1 homolog, mitochondr	8670.727	7962.019	8494.185	10023.03	8787	8253.688	10043.34	12752.46	8791.862	9960	0.8822	0.32531
Q62148	Aldh1a2	Retinal dehydrogenase 2	64719.75	74920.08	76029.1	68697.15	71092	74746.12	69391.84	56985.21	65667.91	66698	1.0659	0.37539
Q8BFZ2	Plppr1	Phospholipid phosphatase-related protein type 1												
Q505F5	Lrrc47	Leucine-rich repeat-containing protein 47	37919.03	35038.51	37243.3	38700.93	37225	37616.31	37680.63	37229.38	38942.94	37867	0.9830	0.48895
Q9R0Q6	Arpc1a	Actin-related protein 2/3 complex subunit 1A	52556.84	53286.98	43014.91	44643.81	48376	47150.6	49933.25	29731.83	40228.81	41761	1.1584	0.25217
Q8BGS7	Cept1	Choline/ethanolaminephosphotransferase 1	61332.05	61641.02	70872.54	78936.03	68195	73055.83	66157.38	61017.1	88761.05	72248	0.9439	0.60155
Q9CPX8	Uqcr11	Cytochrome b-c1 complex subunit 10	7982.545	14481.86	10686.15	9666.035	10704		10862.6		8835.927	9849	1.0868	0.71207
Q9WTN0	Ggps1	Geranylgeranyl pyrophosphate synthase	38296.89	43289.72	31870.65	26911.56	35092	34859.42	45452.21	29593.58	34822.3	36182	0.9699	0.83128
Q61655	Ddx19a	ATP-dependent RNA helicase DDX19A	61100.46	64145.04	65935.66	60225.8	62852	62338.2	60693.31	61629.24	59502.43	61041	1.0297	0.26184
P52760	Rida	2-iminobutanoate/2-iminopropanoate deaminase	144146	164321.5	120403.8	118961.6	136958	134671.2	149255.7	120139.8	119674.5	130935	1.0460	0.65660
Q8WUR0		Protein C19orf12 homolog	18982.01	25230.62	25301.2	20401.75	22479	22428.7	9248.782	14512.04	20769.95	16740	1.3428	0.14601
Q99JX7	Nxf1	Nuclear RNA export factor 1	26081.71	28028.76	28246.16	24639.75	26749	26525.62	28747.07	31306.71	33455.96	30009	0.8914	0.10911
P00405	Mtco2	Cytochrome c oxidase subunit 2	691451.2	751397.8	809125.3	890019	785498	792901.3	897958.6	694602.2	842864	807082	0.9733	0.73337

P62631	Eef1a2	Elongation factor 1-alpha 2	1999985	2056409	2232088	1929539	**2054505**	2197384	2155009	2359447	2280659	**2248125**	**0.9139**	**0.04966**
Q9DAZ9	Zfyve19	Abscission/NoCut checkpoint regulator	23256.21	16560.87	22361.78	19445.68	**20406**	16551.65	19990.36	21723.88	18956.83	**19306**	**1.0570**	**0.57642**
P15508	Sptb	Spectrin beta chain, erythrocytic	191155.8	205522.1	213984.5	235854.2	**211629**	185926.9	200406.5	190434	186691.4	**190865**	**1.1088**	**0.08134**
Q9R1P0	Psma4	Proteasome subunit alpha type-4	218576.9	210183.8	206553	182446.6	**204440**	203959.8	195986.1	214027.7	198316.7	**203073**	**1.0067**	**0.88067**
Q9CXK8	Nip7	60S ribosome subunit biogenesis protein NIP7 homolog			26149.87		**26150**			29899.75		**29900**	**0.8746**	
Q6NV83	U2surp	U2 snRNP-associated SURP motif-containing protei	21182.69	20059.17	27771.65	24316.61	**23333**	23639.53	20767.71	23586.94	24893.24	**23222**	**1.0048**	**0.95634**
P47754	Capza2	F-actin-capping protein subunit alpha-2	369169.4	368340.3	336874.5	330234.6	**351155**	352089.4	399421.7	373726.5	368819.8	**373514**	**0.9401**	**0.16597**
Q9QYG0	Ndrg2	Protein NDRG2	123680	152042.3	132045.5	123869.6	**132909**	115330.5	134246.2	125362.8	115532.9	**122618**	**1.0839**	**0.24890**
Q62048	Pea15	Astrocytic phosphoprotein PEA-15	146021.7	167717	122263.5	111944.1	**136987**	127196.1	159955.6	107110.4	108556.2	**125705**	**1.0898**	**0.54351**
Q8C0N2	Gpat3	Glycerol-3-phosphate acyltransferase 3			7173.157	6684.043	**6929**	7114.681	8180.847			**7648**	**0.9060**	**0.34491**
Q9D1P0	Mrpl13	Large ribosomal subunit protein uL13m	7575.868	11522.31	12555.89	11200.63	**10714**	11948.78	11787.99	13968.45	14671.38	**13094**	**0.8182**	**0.11769**
Q9ER58	Spock2	Testican-2	32482.2	30298.29	25527.83	32856.27	**30291**	35546.8	25906.86	31414.69	31520.6	**31097**	**0.9741**	**0.76698**
Q6A0D4	Rftn1	Raftlin	10625.42				**10625**				900.995	**901**	**11.7930**	
Q9CRB8	Mtfp1	Mitochondrial fission process protein 1		6514.929	17333.08	16319.94	**13389**	3261.49	18860.91	3455.801	13827.37	**9851**	**1.3591**	**0.54343**
Q6PE01	Snrnp40	U5 small nuclear ribonucleoprotein 40 kDa protein	29347.21	29191.86	31396.51	30148.91	**30021**	29449.92	27687.04	34981.42	32298	**31104**	**0.9652**	**0.54332**
Q8BSZ2	Ap3s2	AP-3 complex subunit sigma-2	26053.63	18486.62	25934.62	12338.7	**20703**	17251.17	17779.89	13253.05	17007.79	**16323**	**1.2684**	**0.25257**
Q8K211	Slc31a1	High affinity copper uptake protein 1			21090.53	13394.51	**17243**	22225.57	17963.38	21496.81		**20562**	**0.8386**	**0.39278**
E9Q8I9	Fry	Protein furry homolog	11581.64	11497.58	11600.85	11195.07	**11469**	10411.97	12277.36	10377.69	12556.07	**11406**	**1.0055**	**0.91897**
Q3UIA2	Arhgap17	Rho GTPase-activating protein 17	29563.56	29495.51	30310.29	27006.7	**29094**	31030.16	27790.49	32132.89	29935.54	**30222**	**0.9627**	**0.37334**
Q8CDM8	Fhip2a	FHF complex subunit HOOK interacting protein 2A	28946.87	27908.45	26804.84	20696.64	**26089**	19177.89	22117.21	17951.54	21592.53	**20210**	**1.2909**	**0.03103**
Q9R0P9	Uchl1	Ubiquitin carboxyl-terminal hydrolase isozyme L1	2718854	2744131	2254421	1923799	**2410301**	2478930	2370126	1839717	1815023	**2125949**	**1.1338**	**0.32126**
Q3UTH8	Arhgef9	Rho guanine nucleotide exchange factor 9	14416.5	18121.42	17259.18		**16599**	12420.18	16098.13	8077.197	15549	**13036**	**1.2733**	**0.19320**
O70194	Eif3d	Eukaryotic translation initiation factor 3 subunit D	94103.07	97209.65	102212.1	111405.2	**101233**	100063.1	106325.5	113654.8	116569	**109153**	**0.9274**	**0.18582**
Q8VED9	Lgalsl	Galectin-related protein	23187.94	27058.89	18563.55	11587.94	**20100**	24317.58	22620.42	20718.29	19065.36	**21680**	**0.9271**	**0.66881**
Q8VEF1	Gramd1a	Protein Aster-A		31455.69	30151.43	27507.82	**29705**	22499.8		26153.18	29914.48	**26189**	**1.1342**	**0.22231**
Q8BIW1	Prune1	Exopolyphosphatase PRUNE1	34809.45	35772.58	29092.31	25157.49	**31208**	32901.29	29593.37	28702.91	25587.98	**29196**	**1.0689**	**0.51608**
P70288	Hdac2	Histone deacetylase 2	48068.33	48249.18	54281.32	54408.35	**51252**	45890.95	50411.1	58051.11	52724.58	**51769**	**0.9900**	**0.87273**
Q60634	Flot2	Flotillin-2	126952.7	142698	123834.8	140045.7	**133383**	137999.3	148478.6	126709	126896.9	**135021**	**0.9879**	**0.82286**
P08103	Hck	Tyrosine-protein kinase HCK												
Q8BLQ9	Cadm2	Cell adhesion molecule 2	21013.57	17364.22	16641.8	17601.96	**18155**	17640.98	15636.15	16951.05	17153.14	**16845**	**1.0778**	**0.26443**
P52194	Clgn	Calmegin	42219.33	39623.15	34902.23	39770.75	**39129**	42883.54	44849.07	44201.63	42542.25	**43619**	**0.8971**	**0.03260**
Q8R1T1	Chmp7	Charged multivesicular body protein 7	8203.138	8621.986	8817.514	8799.78	**8611**	9491.019	8739.378	9230.031	8837.433	**9074**	**0.9489**	**0.08555**
Q9Z2U1	Psma5	Proteasome subunit alpha type-5	201649.8	224862.4	209170.1	176340.9	**203006**	211807.7	216141.5	201210.7	207430.3	**209148**	**0.9706**	**0.58369**
Q9D1R9	Rpl34	Large ribosomal subunit protein eL34	560661.7	519641.8	542535.6	571853.4	**548673**	560578.5	577818.3	622360	625512.8	**596567**	**0.9197**	**0.05207**
P27773	Pdia3	Protein disulfide-isomerase A3	1166740	1170360	1282860	1265314	**1221319**	1128714	1201047	1308542	1333393	**1242924**	**0.9826**	**0.71633**
P11352	Gpx1	Glutathione peroxidase 1	162856.6	169164.1	154630.6	140379.7	**156758**	153021	158995.5	149154.2	133820.4	**148748**	**1.0538**	**0.36728**
Q6PD19	Armh3	Armadillo-like helical domain-containing protein 3	11013.89	12295.09	10163.93	9400.908	**10718**	8542.799	10212.01	10719.49	9621.14	**9774**	**1.0966**	**0.26966**
Q3TES0	Iqsec3	IQ motif and SEC7 domain-containing protein 3	9307.105	5865.355	4694.688	3306.655	**5793**	3158.933	8872.39	20264.5	5489.571	**9446**	**0.6133**	**0.39667**
Q66L44	Cbarp	Voltage-dependent calcium channel beta subunit-associated regulatory protein												
Q64676	Ugt8	2-hydroxyacylsphingosine 1-beta-galactosyltransfer	58864.91	71072.49	162225.4	80728.22	**93223**	70374.52	56722.21	145629.9	174404.3	**111783**	**0.8340**	**0.63354**
O35114	Scarb2	Lysosome membrane protein 2	89074.9	85415.58	75051.74	87260.29	**84201**	98367.04	92873.2	86801.34	90752.73	**92199**	**0.9133**	**0.08984**
Q6P8J2	Sat2	Thialysine N-epsilon-acetyltransferase												
O55057	Pde6d	Retinal rod rhodopsin-sensitive cGMP 3',5'-cyclic ph	28178.36	21847.71	21837.13	18929.28	**22698**	23551.71	20402.69	24826.02	21457.49	**22559**	**1.0061**	**0.95163**
Q9QZB0	Rgs17	Regulator of G-protein signaling 17								41763.12		**41763**	**0.0000**	
Q8BVY0	Rsl1d1	Ribosomal L1 domain-containing protein 1	16678.55	17682.61	16858.71	20289.33	**17877**	10573.8	14542.67	16326.75	20543.36	**15497**	**1.1536**	**0.32662**
Q8R5H1	Usp15	Ubiquitin carboxyl-terminal hydrolase 15	13682.62	14473.68	13009.14	12614.36	**13445**	14611	14557.73	12404.75	13487.81	**13765**	**0.9767**	**0.64579**
Q810A7	Ddx42	ATP-dependent RNA helicase DDX42	55982.87	51763	60142.29	63234.96	**57781**	58774.25	54727.35	64433.42	72836.02	**62693**	**0.9216**	**0.33149**
Q9QUR7	Pin1	Peptidyl-prolyl cis-trans isomerase NIMA-interacting	110349.5	107561.1	75386.92	88536.63	**95459**	93305.36	99158.59	71935.95	92595.62	**89249**	**1.0696**	**0.56440**
P09242	Alpl	Alkaline phosphatase, tissue-nonspecific isozyme	17335.42	16974.08	17481.42	18521.77	**17578**	15808.23	17344.86	13394.79	16354.87	**15726**	**1.1178**	**0.08602**
Q99KB8	Hagh	Hydroxyacylglutathione hydrolase, mitochondrial	39193.21	38797.8	33580.28	32772.47	**36086**	36569.89	35525.35	36165.8	30151.85	**34603**	**1.0428**	**0.53595**
Q68ED2	Grm7	Metabotropic glutamate receptor 7		19691.85	4362.224	4646.454	**9567**	3365.103	3363.748	5102.314	10207.38	**5510**	**1.7364**	**0.42274**
Q6PGH2	Jpt2	Jupiter microtubule associated homolog 2	7907.504	7921.948	11744.94	10745.59	**9580**	6434.869	8036.139	13218.06	13915.69	**10401**	**0.9210**	**0.71000**
P56818	Bace1	Beta-secretase 1												
P42703	Lifr	Leukemia inhibitory factor receptor	8324.458	10803.21	11184.73	8690.399	**9751**	10686.5	9150.692	8674.853	10056.93	**9642**	**1.0112**	**0.90315**
P97823	Lypla1	Acyl-protein thioesterase 1	54825.16	60781.2	51132.48	48036.63	**53694**	54550.83	52799.17	48117.7	46678.11	**50536**	**1.0625**	**0.37794**
Q924Y0	Bbox1	Gamma-butyrobetaine dioxygenase												
Q9Z0N2	Eif2s3y	Eukaryotic translation initiation factor 2 subunit 3, Y-linked			14694.83	14254.66	**14475**			17832.81	18063.56	**17948**	**0.8065**	**0.00508**
Q6R891	Ppp1r9b	Neurabin-2	19795.99	20799.41	20877.63	20884.18	**20589**	21642.96	21847.4	19118.15	17791.96	**20100**	**1.0243**	**0.64957**
Q9JME7	Trappc2l	Trafficking protein particle complex subunit 2-like pr	41745.01	47580.48	44576.54	43493.14	**44349**	46679.52	46534.91	45119.3	51540.39	**47469**	**0.9343**	**0.14484**

Q68FH0	Pkp4	Plakophilin-4	14793.51	11336.23	14827.02	11482.2	13110	9863.947	17282.24	5954.443	16324.67	12356	1.0610	0.80165
Q3UHH2	Slc22a23	Solute carrier family 22 member 23												
Q6PHU5	Sort1	Sortilin	29608.27	31376.25	31278.2	36411.91	32169	31402.44	35509.88	36493.18	37207.94	35153	0.9151	0.17902
Q9EQG9	Cert1	Ceramide transfer protein	27586.85	22336.79	22964.7	18377.46	22816	22517.36	21785.94	24589.48	24984.46	23469	0.9722	0.75995
Q8K2F8	Lsm14a	Protein LSM14 homolog A	9605.178	10483.83	11697.71	11000.46	10697	13307.09	12269.14	10993.63	14905.93	12869	0.8312	0.05971
Q9WV96	Timm10b	Mitochondrial import inner membrane translocase s	12943.45	11624.62	6831.576	10099.97	10375	9886.724	8073.83	9657.986	7420.631	8760	1.1844	0.30708
Q64511	Top2b	DNA topoisomerase 2-beta	44209.52	44031.45	48245.41	45923.17	45602	44579.31	44589.13	41837.41	47941	44737	1.0194	0.60506
Q8R2K1	Fuom	Fucose mutarotase	19158.89	24460.47	16408.78	7895.206	16981	18738.3	16030.28	13273.16	11814.22	14964	1.1348	0.61309
O08599	Stxbp1	Syntaxin-binding protein 1	194991	204204.1	169766.3	200441.9	192351	191978.8	191817	169744.5	175721.4	182315	1.0550	0.33659
P84091	Ap2m1	AP-2 complex subunit mu	182013.9	184349.6	205551.7	207416.8	194833	198824	198918.7	199692.7	215027	203116	0.9592	0.33128
Q91ZW3	Smarca5	SWI/SNF-related matrix-associated actin-dependent	21747.58	18627.79	18033.89	23603.57	20503	17571.52	16423.5	20682.23	21872.84	19138	1.0714	0.48513
Q9DC29	Abcb6	ATP-binding cassette sub-family B member 6	27245.22	31226.78	36150.26	35599.29	32555	27644.33	37064.29	41141.86	40413.89	36566	0.8903	0.32463
P56376	Acyp1	Acylphosphatase-1	83893.76	85045.38	72099.35	64400.95	76360	82260.32	74265.27	76981.77	54533.13	72010	1.0604	0.59813
Q80U49	Cep170b	Centrosomal protein of 170 kDa protein B	27018.84	27172	27283.73	22577.28	26013	23684.1	23585.22	16579.56	22205.31	21514	1.2091	0.06885
Q9D009	Lipt2	Putative lipoyltransferase 2, mitochondrial	2805.336		1763.124		2284				2532.962	2533	0.9018	
Q3TYD6	Lmtk2	Serine/threonine-protein kinase LMTK2												
P34914	Ephx2	Bifunctional epoxide hydrolase 2	55639.11	51126.97	40437.02	46947.89	48538	62353.65	44981.83	34976.8	39155.67	45367	1.0699	0.65905
Q8BQP9	Rgs7bp	Regulator of G-protein signaling 7-binding protein	13678.04	14854.66	13525.13	15049.77	14277	16500.32	12495.2	10239.19	15525.58	13690	1.0429	0.70636
Q8BGQ7	Aars1	Alanine--tRNA ligase, cytoplasmic	64894.77	67766.12	58052.21	55198.84	61478	62692.55	61921.12	52799.5	53592.66	57751	1.0645	0.38041
Q64521	Gpd2	Glycerol-3-phosphate dehydrogenase, mitochondria	138903.3	138512.1	129221.6	172268.9	144726	137950.2	168732.1	134650.5	140865	145549	0.9943	0.94872
Q8CBG9	Rnf170	E3 ubiquitin-protein ligase RNF170	19278.68	15709.29	17981.45	18459.81	17857	14703.96	17523.08	19677.16	16495.18	17100	1.0443	0.57822
Q9JKB1	Uchl3	Ubiquitin carboxyl-terminal hydrolase isozyme L3	29918.8	30501.56	32080.78	22677.28	28795	27096.03	25843.98	24275.89	19935.03	24288	1.1856	0.13477
P43024	Cox6a1	Cytochrome c oxidase subunit 6A1, mitochondrial	112933.5	129683.8	120477.8	124889	121996	107323.1	131539.4	112513.8	115915.5	116823	1.0443	0.44381
Q8BLN5	Lss	Lanosterol synthase	20399.56	18672.16	33267.61	20538.27	23219	17182.54	20461.5	33496.29	40262.85	27851	0.8337	0.49634
Q9D958	Spcs1	Signal peptidase complex subunit 1	43544	39455.68	46220.79	41679.21	42725	39377.75	37830.46	48836.28	49561.44	43901	0.9732	0.74082
Q9JHJ0	Tmod3	Tropomodulin-3	50565.67	50295.67	54238.03	48174.8	50819	48681.31	46141.13	50914.18	48757.63	48624	1.0451	0.21741
Q91V24	Abca7	ATP-binding cassette sub-family A member 7		6131.531	4335.846	6877.748	5782	5201.463	6555.379	5866.939	5777.421	5850	0.9883	0.92693
Q9Z0R6	Itsn2	Intersectin-2	21315.44	23251.55	18756.19	24422.35	21936	19901.52	22323.45	22864.84	21375.25	21616	1.0148	0.82654
Q99J99	Mpst	3-mercaptopyruvate sulfurtransferase	30547.76	26203.46	27701.22	28555.99	28252	25444.65	27445.51	23898.44	25549.37	25584	1.1043	0.06138
Q8VDP4	Ccar2	Cell cycle and apoptosis regulator protein 2	62606.72	63678.72	68744.63	70917.2	66487	64207.42	65501.77	70099.45	72804.52	68153	0.9755	0.57663
P60879	Snap25	Synaptosomal-associated protein 25	70309.38	68134.85	67339.13	74935.91	70180	63432.93	73274.14	66424.65	68048.72	67795	1.0352	0.40703
Q9CQI6	Cotl1	Coactosin-like protein	142553.4	137825	135455	98278.28	128528	110445.8	132188.8	99494.91	96583.19	109678	1.1719	0.19731
Q5BKP2	Usp13	Ubiquitin carboxyl-terminal hydrolase 13	115034.4	99163.97	87557.59	67250.85	92252	85930.02	94408.88	57798.25	84756.88	80724	1.1428	0.40294
Q6I6G8	Hecw2	E3 ubiquitin-protein ligase HECW2	34495.31	38737.71	34706.82	40727.07	37167	34924.6	34909.37	31770.88	33696.01	33825	1.0988	0.09798
Q9WV92	Epb41l3	Band 4.1-like protein 3	561437.9	544132.1	401570.7	574485	520406	517949.2	566805.6	390681.6	414276.6	472428	1.1016	0.43960
Q80WG5	Lrrc8a	Volume-regulated anion channel subunit LRRC8A	28150.86	25958.1	30627.61	29922.92	28665	29911.07	29570.66	27063.21	32784.88	29832	0.9609	0.48451
Q9JLJ1	Selenok	Selenoprotein K	11389.28	12656.47	16399.79	10260.27	12676	10705.5	11050.24	13453.52	16341.19	12888	0.9836	0.91355
Q00612	G6pdx	Glucose-6-phosphate 1-dehydrogenase X	58061.7	54546.18	52088.25	45866.14	52641	48919.39	50268.77	44159.54	44414.77	46941	1.1214	0.10659
Q8CAE9	Podxl2	Podocalyxin-like protein 2												
Q7TSJ2	Map6	Microtubule-associated protein 6	231130.3	227771.1	174689.4	264098.9	224422	187873.7	235707.5	211027.7	221936	214136	1.0480	0.64280
Q3TKY6	Cwc27	Spliceosome-associated protein CWC27 homolog	7893.428	5755.417	5455.342		6368	6789.628	4666.099	4376.004	4396.904	5057	1.2592	0.22234
Q9R1T4	Septin6	Septin-6	31339.93	41818.89	36856.51	39422	37359	41784.06	35506.1	47802.23	34700.36	39948	0.9352	0.52073
P57774	Npy	Pro-neuropeptide Y												
Q80TR1	Adgrl1	Adhesion G protein-coupled receptor L1												
Q9JJI8	Rpl38	Large ribosomal subunit protein eL38	154146.3	150069.7	160321.5	166066.5	157651	161091	149024.3	180027.5	179649.6	167448	0.9415	0.28462
P80316	Cct5	T-complex protein 1 subunit epsilon	260165.7	256179.6	273263.4	280186.9	267449	269737.8	261078.9	286294.1	288327.9	276360	0.9678	0.34205
Q9D1I6	Mrpl14	Large ribosomal subunit protein uL14m	7937.005	6904.145	11546.42	12235.72	9656	11099.38	10997.34	14056.95	14942.96	12774	0.7559	0.10938
Q9ET01	Pygl	Glycogen phosphorylase, liver form	658894.4	622977.4	607586.9	644107	633391	572958.8	582213	459352.8	572442.9	546742	1.1585	0.03263
Q9ES28	Arhgef7	Rho guanine nucleotide exchange factor 7	33273.07	27746.58	32240.38	26516.43	29944	21627.26	27240.51	27661.25	31554.75	27021	1.1082	0.30900
Q6NS52	Dgkb	Diacylglycerol kinase beta								3675.015		3675	0.0000	
Q9CQE8	RTRAF	RNA transcription, translation and transport factor p	85290.61	99344.14	86816.08	84700.02	89038	78396.62	102540.7	92046.86	93984.13	91742	0.9705	0.67215
Q8VHX6	Flnc	Filamin-C	52944.95	54101.39	66970.7	48011.63	55507	47274.11	47740.07	48030.34	55942.21	49747	1.1158	0.25173
Q5DU25	Iqsec2	IQ motif and SEC7 domain-containing protein 2	11432.55	11243.67	13406.2	9987.865	11518	7847.201	9385.609		11243.61	9492	1.2134	0.14454
Q9D287	Bcas2	Pre-mRNA-splicing factor SPF27	30113.57	26536.17	30374.41	33565.74	30147	29673.36	27670.98	33447.01	30947.88	30435	0.9906	0.88343
P63073	Eif4e	Eukaryotic translation initiation factor 4E	72753.12	74878.83	80244.41	76772.41	76162	78983.91	76315.83	90218.11	84234.31	82438	0.9239	0.11945
P59764	Dock4	Dedicator of cytokinesis protein 4	18115.8	17773.79	20513.52	14856.37	17815	18164.21	16762.14		19131.65	18019	0.9887	0.89604
P61924	Copz1	Coatomer subunit zeta-1	124679.8	136640	148480.8	143638.8	138360	142676.5	125549	161293.5	166276.1	148949	0.9289	0.35830
Q8BGD8	Coa6	Cytochrome c oxidase assembly factor 6 homolog	19522.89	16398.53	19806.9	26973.51	20675	19394.29	22301.89	26164.4	24844.26	23176	0.8921	0.38839
Q5SVR0	Tbc1d9b	TBC1 domain family member 9B	28622.69	35414.25	39004.48	31239.18	33570	32557.59	33967.77	31301.21	35740.34	33392	1.0053	0.94497

Q7TPM1	Prrc2b	Protein PRRC2B	9532.506	10398.63	11265.69	13571.76	11192	8104.169	10095.01	11823.42	15133.31	11289	0.9914	0.95704
Q810B6	Ankfy1	Rabankyrin-5	40383.32	41554.46	42383.18	38961.5	40821	41078.68	43048.1	39391.71	40224.11	40936	0.9972	0.91866
Q99JY8	Plpp3	Phospholipid phosphatase 3	74493.48	77296.62	73701.03	89767.01	78815	70988.39	92072.98	81090.34	80935.63	81272	0.9698	0.68136
Q9WVQ1	Magi2	Membrane-associated guanylate kinase, WW and PD	15206.95	17106.34	15527.87	18540.56	16595	16465.01	15872.02	16321.61	17037.38	16424	1.0104	0.83871
P82198	Tgfbi	Transforming growth factor-beta-induced protein ig-l	1065558	949733.9	916735.2	1167330	1024839	988129.4	910030	812616.1	863562.8	893585	1.1469	0.10296
P70245	Ebp	3-beta-hydroxysteroid-Delta(8),Delta(7)-isomerase	89104.44	78845.09	101434.8	96375.45	91440	83597.15	86380.4	108703.4	102083.6	95191	0.9606	0.64769
Q6PHQ8	Naa35	N-alpha-acetyltransferase 35, NatC auxiliary subunit	17645.98	15677.15	16346.75	16927.24	16649	17169.11	16601.47	15420	18571.08	16940	0.9828	0.72078
Q8BZH4	Pogz	Pogo transposable element with ZNF domain	1388.262			698.4702	1043	677.3072	556.7471	2990.303		1408	0.7410	0.75217
Q02111	Prkcq	Protein kinase C theta type	22333.88	17608.88	16414.77	18773.02	18783	19856.88	17464.54	21816.66	22908.38	20512	0.9157	0.36135
Q62384	Zpr1	Zinc finger protein ZPR1	14008.1	16786.07	14438.82	12337.25	14393	14159.15	10656.29	12046.4	13112.39	12494	1.1520	0.16000
Q9D7S9	Chmp5	Charged multivesicular body protein 5	43176.04	39928.47	38205.45	36889.15	39550	40424.51	39780.43	42901.88	44731.88	41960	0.9426	0.22367
Q9Z2A9	Ggt5	Glutathione hydrolase 5 proenzyme	5286.021		7593.81	7394.233	6758	4272.958	5923.485	7840.841	9051.011	6772	0.9979	0.99233
Q99PV0	Prpf8	Pre-mRNA-processing-splicing factor 8	42967.84	43919.36	48728.18	47950.09	45891	43051.62	45086.28	46547.07	48953.05	45910	0.9996	0.99269
Q99MU3	Adar	Double-stranded RNA-specific adenosine deaminase	8429.442	5301.832		8575.678	7436		3888.66	7825.454	4318.01	5344	1.3914	0.27161
Q65CL1	Ctnna3	Catenin alpha-3	35979.02	33843.16	30792.95	38759.51	34844	31339.8	34250.06	31447.29	34462.61	32875	1.0599	0.33758
Q9CYN2	Spcs2	Signal peptidase complex subunit 2	58151.43	47183.19	59588.23	59354.3	56069	46033.29	62804.93	59935.55	63704.57	58120	0.9647	0.70019
A6H6A9	Rabgap1l	Rab GTPase-activating protein 1-like	7769.309	12747.63	11584.37	9532.824	10409	12476.22	6886.724	11177.56	6029.716	9143	1.1385	0.53584
Q9WTM5	Ruvbl2	RuvB-like 2	65416.3	67443.25	70639.8	69350.42	68212	67162.58	67009.47	73802.92	69576.04	69388	0.9831	0.56919
P50396	Gdi1	Rab GDP dissociation inhibitor alpha	354699.4	366059.9	344183.6	314023.5	344742	372511.5	351231.4	292399.5	305892.4	330509	1.0431	0.53963
A2A8U2	Tmem201	Transmembrane protein 201	24196.36	25573.44	21496.97	39744.32	27753	30402.85	25977.71	30497.32	34319.71	30299	0.9160	0.58606
Q8R5M8	Cadm1	Cell adhesion molecule 1	67153.77	59043.77	51358.41	72643.71	62550	67655.01	71522.69	64531.28	68933.32	68161	0.9177	0.29415
P55821	Stmn2	Stathmin-2	565930	536504.3	547731.3	515208.4	541344	518460.3	538803.6	621231.1	510180.9	547169	0.9894	0.83946
Q61838	Pzp	Pregcy zone protein	145705.2	121118.4	141189.5	75063.61	120769	104239.9	84621.48	82413.55	66617.25	84473	1.4297	0.08884
Q9EP53	Tsc1	Hamartin	16176.17	5214.517		16986.61	12792		13985.1	13225.09	15632.51	14281	0.8958	0.71956
Q8BTZ7	Gmppb	Mannose-1-phosphate guanyltransferase beta	39244.78	41452.77	51504.9	31626	40957	45345.54	36898.82	43970.99	44493.07	42677	0.9597	0.71765
P35276	Rab3d	Ras-related protein Rab-3D	63711.54	72527.64	69827.38	74043.53	70028	61098.98	60755.6	68766.98	64373.16	63749	1.0985	0.07676
Q9CZ62	Cep97	Centrosomal protein of 97 kDa	9440.426	8924.563	6457.054	11642.74	9116	9365.121	11122.11	12464.51	5755.387	9677	0.9421	0.76615
Q8BU31	Rap2c	Ras-related protein Rap-2c	23317.6	21813.15	16457.77	24340.02	21482	18587.3	22600.15	16271.13	18261.18	18930	1.1348	0.28976
Q8R0H9	Gga1	ADP-ribosylation factor-binding protein GGA1	16784.26	17702.08	17940.75	12716.23	16286	18467.89	17737.19	18929.03	15182.78	17579	0.9264	0.41434
Q60649	Clpb	Mitochondrial disaggregase	13307.44	12655.1	12775.39	15077.81	13454	14711.15	15534.46	13723.47	15121.41	14773	0.9107	0.10091
P97470	Ppp4c	Serine/threonine-protein phosphatase 4 catalytic su	21672.92	28014.91	20736.25	22880.78	23326	20132.8	28163.72	29119.01	17288.84	23676	0.9852	0.92024
Q8C2Q3	Rbm14	RNA-binding protein 14	43144.67	49156.22	50917.46	54259.8	49370	44313.39	47348.95	51531.93	55517.46	49678	0.9938	0.93021
Q3UVX5	Grm5	Metabotropic glutamate receptor 5												
Q9Z2E1	Mbd2	Methyl-CpG-binding domain protein 2	21899.94	12698.91	16383.69	15748.55	16683	16475.48	20834.07	17941.34	18928.45	18545	0.8996	0.41418
P63213	Gng2	Guanine nucleotide-binding protein G(I)/G(S)/G(O) s	91997.55	80178	55031.01	75944.77	75788	75150.79	100914.8	56371.52	49846.53	70571	1.0739	0.71844
O55137	Acot1	Acyl-coenzyme A thioesterase 1	265012.4	252190.8	306475.6	285113.5	277198	278280.1	248832.8	320675.1	293038.6	285207	0.9719	0.68981
B9EKR1	Ptprz1	Receptor-type tyrosine-protein phosphatase zeta		89083.41			89083		2837.745		5326.68	4082	21.8223	
Q8VE85	Prelid3a	PRELI domain containing protein 3A												
Q61037	Tsc2	Tuberin	3696.628	4316.217	4210.31	4489.038	4178	5596.463	5498.053	6116.188	5167.795	5595	0.7468	0.00159
Q3TZX3	Slc25a33	Solute carrier family 25 member 33												
P22437	Ptgs1	Prostaglandin G/H synthase 1	2882.144	1500.521	1043.803	2331.215	1939	2545.527	1940.328	944.3133	814.1362	1561	1.2424	0.54085
P07214	Sparc	SPARC	49310	50524.98	78765.09	47666.77	56567	50433.61	50775.15	70767.18	71259.1	60809	0.9302	0.67015
P15105	Glul	Glutamine synthetase	167429.6	185374.3	186646.5	163297.5	175687	156790	181133.7	151850.3	162324.9	163025	1.0777	0.19992
Q9D6W8	Borcs6	BLOC-1-related complex subunit 6	24527.37	32357.52	31120.1	35116.18	30780	22108.71	32519.83	25553.7	32740.47	28231	1.0903	0.48932
Q3UPH7	Arhgef40	Rho guanine nucleotide exchange factor 40	7865.136	7640.84	9388.802	8994.802	8472	7896.905	8461.924	11612.25	11078.95	9763	0.8679	0.25309
O88291	Znf326	DBIRD complex subunit ZNF326	9184.317	9494.062	11837.63	10482.73	10250	6627.866	9893.429	12916.3	12674.83	10528	0.9736	0.86647
Q9Z1M8	Ik	Protein Red	15838.9	17614.95	16312.09	19928.56	17424	15119.39	15987.82	19191.12	20348.74	17662	0.9865	0.88304
Q99L45	Eif2s2	Eukaryotic translation initiation factor 2 subunit 2	57633.01	59099.88	64583.83	64196.05	61378	61884.99	58758.04	73268.37	71884.32	66449	0.9237	0.25349
O55135	Eif6	Eukaryotic translation initiation factor 6	66705.52	54993.42	60505.82	50800.77	58251	55161.95	51394.93	67026.05	60273.84	58464	0.9964	0.96630
A2A8L5	Ptprf	Receptor-type tyrosine-protein phosphatase F	19217.97	20592.06	22738.24	22450.21	21250	23313.6	23152.38	22396.68	26228.58	23773	0.8939	0.07653
Q99LT0	Dpy30	Protein dpy-30 homolog	20684.69	33214.01	21487.59	16117.33	22876	17211.23	22003.92	17172.7	21463.77	19463	1.1754	0.41217
Q9WUM3	Coro1b	Coronin-1B	61649.5	65152.98	61197.15	60459.45	62115	63731.27	62214.07	69971.49	65434.73	65338	0.9507	0.15396
Q99020	Hnrnpab	Heterogeneous nuclear ribonucleoprotein A/B	137621.3	121235.8	139080.7	135351.5	133322	132276.6	121589	140559.2	156117	137635	0.9687	0.62426
Q8BFS6	Cpped1	Serine/threonine-protein phosphatase CPPED1	19300.31	18582.42	18237.84	16665.05	18196	13403.15	18812.09	11950.38	13935.01	14525	1.2528	0.06033
P62488	Polr2g	DNA-directed RNA polymerase II subunit RPB7	10340.03	9950.008	13075.39	12742.47	11527	11163.42	10415.29	10501.91	12444.4	11131	1.0356	0.68567
P50608	Fmod	Fibromodulin	47258.98	62037.33	50989.16	50124.01	52602	55064.26	50658.56	38579.58	36390.38	45173	1.1645	0.23208
A2ASZ8	Slc25a25	Mitochondrial adenyl nucleotide antiporter SLC25A2	38903.66	40473.54	41360.11	38680.25	39854	42027.83	41468.67	41239.95	45134.19	42468	0.9385	0.05646
Q61161	Map4k2	Mitogen-activated protein kinase kinase kinase kinas	3713.194	2982.099	4606.759		3767	3210.964	2252.005	8301.799		4588	0.8211	0.69324
O70228	Atp9a	Probable phospholipid-transporting ATPase IIA	35829.21	42293.38	44793.09	44568.77	41871	40231.81	41572.54	42000.61	42879.39	41671	1.0048	0.92933

O08663	Metap2	Methionine aminopeptidase 2	95886.73	95021.79	100896.3	86719.75	**94631**	97971.53	98726.77	100544.5	95123.8	**98092**	**0.9647**	**0.31359**
Q3U186	Rars2	Probable arginine--tRNA ligase, mitochondrial	12625.83	11676.27	12062.19	12466.5	**12208**	10959.49	11125.45	9793.184	13699.96	**11395**	**1.0714**	**0.37609**
Q6PEE3	Rrm2b	Ribonucleoside-diphosphate reductase subunit M2 I	12515.74	10790.63	8674.174	20778.49	**13190**	11080.78	26141.02	12136.25	6777.226	**14034**	**0.9399**	**0.87058**
Q7TSI3	Ppp6r1	Serine/threonine-protein phosphatase 6 regulatory s	36524.18	29523.24	31264.49	26651.88	**30991**	33745.24	28191.93	34908.46	34679.41	**32881**	**0.9425**	**0.49618**
Q8BXC6	Commd2	COMM domain-containing protein 2	49979.09	42069.05	46373.63	39807.54	**44557**	44858.98	43071.79	42030.46	50030.11	**44998**	**0.9902**	**0.88332**
P52825	Cpt2	Carnitine O-palmitoyltransferase 2, mitochondrial	72742.76	77960.95	77272.89	97556.75	**81383**	81261.41	82200.45	82346.16	86343.34	**83038**	**0.9801**	**0.77868**
P05532	Kit	Mast/stem cell growth factor receptor Kit	8717.638	9738.231	5084.047	7395.267	**7734**	9003.034	10530.6	6271.569	6458.705	**8066**	**0.9588**	**0.82521**
Q9R0P4	Smap	Small acidic protein	13318.26	11979.05	12077.16	7339.318	**11178**	11391.48	12878.23	11003.4	6292.202	**10391**	**1.0757**	**0.69891**
Q91VE0	Slc27a4	Long-chain fatty acid transport protein 4	117036.8	122927.6	127907.7	125289.9	**123291**	119314.4	133732.7	124925.4	143119.4	**130273**	**0.9464**	**0.26668**
Q9D882	Fam241b	Protein FAM241B	27082.5	25650.65	27451.7	32104.42	**28072**	23478.62	23607.97	30735.39	33791.14	**27903**	**1.0061**	**0.95612**
Q9CZR3	Tomm40l	Mitochondrial import receptor subunit TOM40B	10137.69	7908.07	4782.778	9026.458	**7964**	9888.131	14714.53	11952.84	10717.03	**11818**	**0.6739**	**0.04874**
Q7TPS5	C2cd5	C2 domain-containing protein 5	13354.2	14762.3	13324.19	13212.03	**13663**	10943.59	11161.78	9226.908	12492.47	**10956**	**1.2471**	**0.01221**
P00375	Dhfr	Dihydrofolate reductase	17166.17	20841.09	17376.22	12211.97	**16899**	11753.67	16572.35	5727.833	9481.942	**10884**	**1.5526**	**0.08170**
P61804	Dad1	Dolichyl-diphosphooligosaccharide--protein glycosy	124900.8	114197.6	112508.8	102150.9	**113440**	111950.8	124880.3	134309.3	127216.3	**124589**	**0.9105**	**0.14171**
P40336	Vps26a	Vacuolar protein sorting-associated protein 26A	47188.68	49177.9	50275.25	43672.14	**47578**	48699.08	48018.37	39598.67	45010.41	**45332**	**1.0496**	**0.40857**
O54781	Srpk2	SRSF protein kinase 2	19350.97	21634.92	22521.8	23698.62	**21802**	22346.13	23257.04	22094.26	22534.92	**22558**	**0.9665**	**0.45756**
Q00PI9	Hnrnpul2	Heterogeneous nuclear ribonucleoprotein U-like pro	54977.06	55957.28	65271.46	63470.84	**59919**	59813.96	61941.85	68924.3	68099.77	**64695**	**0.9262**	**0.21476**
Q9Z210	Pex11b	Peroxisomal membrane protein 11B	13626.91	14520.38	11265.57	13150.4	**13141**	14535.62	20568.57	12189.25	11597.92	**14723**	**0.8925**	**0.49175**
Q60864	Stip1	Stress-induced-phosphoprotein 1	429055.7	449322.5	461557.8	439909.1	**444961**	471452.1	411584.2	448753.8	472619.3	**451102**	**0.9864**	**0.71191**
Q3UH66	Wnk2	Serine/threonine-protein kinase WNK2	58188.98	56640.53		12828.9	**42553**			9943.966	12521.7	**11233**	**3.7883**	**0.20168**
Q7TMK9	Syncrip	Heterogeneous nuclear ribonucleoprotein Q	278745.7	308309	358732.4	329406.1	**318798**	297724.1	280826.7	379370.9	351470.4	**327348**	**0.9739**	**0.77436**
Q6PD26	Pigs	GPI transamidase component PIG-S	45325.22	38014.11	44419.15	47994.38	**43938**	42814.81	45749.68	47112.86	49738.71	**46354**	**0.9479**	**0.38172**
P98084	Apba2	Amyloid-beta A4 precursor protein-binding family A r	11006.1	7006.062	6736.188	7869.405	**8154**	9728.637	7590.147	12657.1	11665.81	**10410**	**0.7833**	**0.18039**
Q9WUL7	Arl3	ADP-ribosylation factor-like protein 3	142976.6	162666.2	144105.7	107366.1	**139279**	146893.5	132300.8	131996.9	128323.7	**134879**	**1.0326**	**0.73204**
P57784	Snrpa1	U2 small nuclear ribonucleoprotein A'	60524.62	54975.39	63769.25	64686.18	**60989**	54926.93	58754.01	69095.38	67386.52	**62541**	**0.9752**	**0.71460**
D3Z4S3	Ptrhd1	Putative peptidyl-tRNA hydrolase PTRHD1	14744.97	12652.85	9926.016	7426.006	**11187**	13841.64	11460.46	6291.611	6261.075	**9464**	**1.1821**	**0.51360**
Q7TQA1	Igsf1	Immunoglobulin superfamily member 1												
Q9CR95	Necap1	Adaptin ear-binding coat-associated protein 1	63397.57	64926.22	53103.86	59404.46	**60208**	63419.85	67463.52	49179.96	55642.56	**58926**	**1.0217**	**0.80048**
Q925N0	Sfxn5	Sideroflexin-5	32359.25	32665.88	26184.17	32368.02	**30894**	28501.29	35586.91	23463.27	31062.53	**29654**	**1.0418**	**0.69150**
Q8BH04	Pck2	Phosphoenolpyruvate carboxykinase [GTP], mitocho	74357.64	70986.78	75102.63	80035.94	**75121**	70653.26	76084.22	74228.38	76090.63	**74264**	**1.0115**	**0.71821**
Q9D6D0	Slc25a27	Mitochondrial uncoupling protein 4	19447.57	23698.5	23254.96	22921.46	**22331**	24660.51	19882.73	20518.48	22667.13	**21932**	**1.0182**	**0.79405**
Q9D695	Serpinb7	Serpin B7	39110.73	43568.11	46854.75	43663.33	**43299**	39551.7	38046.66	64715.77	52583.17	**48724**	**0.8887**	**0.43250**
O55143	Atp2a2	Sarcoplasmic/endoplasmic reticulum calcium ATPas	666152.3	662297.5	718886.6	777867.6	**706301**	725478.3	718484.1	784513.4	812114.7	**760148**	**0.9292**	**0.17933**
Q9DBL9	Abhd5	1-acylglycerol-3-phosphate O-acyltransferase ABHD	8033.366	10055.49	11995.7	8690.964	**9694**	10699.93	10951.64	9492.245	11161.3	**10576**	**0.9166**	**0.38955**
Q8C0C7	Farsa	Phenylalanine--tRNA ligase alpha subunit	291829.2	292565	284022.4	308275.8	**294173**	298476.6	301947	332823.5	314531.6	**311945**	**0.9430**	**0.10405**
Q6GQT9	Nomo1	BOS complex subunit NOMO1	154257.3	156135.8	163191.8	172349.3	**161484**	160173.1	176892	174005.5	183504.9	**173644**	**0.9300**	**0.10607**
P97765	Wbp2	WW domain-binding protein 2	145348.7	138126.5	132684.5	126217.5	**135594**	138192.3	125985	136538.4	122503.9	**130805**	**1.0366**	**0.42595**
Q9CQC9	Sar1b	GTP-binding protein SAR1b	214057.1	228415.1	242477.6	184046.6	**217249**	201222.5	192197.6	201490.1	229366.2	**206069**	**1.0543**	**0.48058**
P32921	Wars1	Tryptophan--tRNA ligase, cytoplasmic	205119.3	171242.4	171807.7	143589.6	**172940**	176144	156004.1	159967.3	163736.6	**163963**	**1.0547**	**0.52543**
Q64735	Cr1l	Complement component receptor 1-like protein	63621.17	48754.11	51423.64	63247.84	**56762**	58113.45	63551.13	58889.59	62948.23	**60876**	**0.9324**	**0.35775**
Q99N28	Cadm3	Cell adhesion molecule 3	484929.6	419568.4	349747.2	544023.3	**449567**	441573	426507.8	345333.9	377404.8	**397705**	**1.1304**	**0.31573**
Q9CQ22	Lamtor1	Ragulator complex protein LAMTOR1	76218.14	69906.09	81130.69	86162.36	**78354**	79538.55	74159.68	96248.16	96344.54	**86573**	**0.9051**	**0.26535**
Q8R2U0	Seh1l	Nucleoporin SEH1	30423.58	28703.35	32299.08	28191.87	**29904**	33905.97	32488.17	34138.58	33527.66	**33515**	**0.8923**	**0.01117**
Q9EQZ6	Rapgef4	Rap guanine nucleotide exchange factor 4								5014.376		**5014**	**0.0000**	
Q64433	Hspe1	10 kDa heat shock protein, mitochondrial	726225.9	816167	890465.6	940103.6	**843241**	874238.7	835127.9	1008905	983054.7	**925332**	**0.9113**	**0.23802**
A6H5Y3	Mtr	Methionine synthase	21630.23	22522.51	26029.51	18262.77	**22111**	21867.41	20983.78	18467.86	23551.98	**21218**	**1.0421**	**0.65742**
Q6KAU4	Mvb12b	Multivesicular body subunit 12B	15207.73	17738.29	16837.53	19924.46	**17427**	15737.93	14668.55	18685.04	19132.35	**17056**	**1.0218**	**0.80943**
P63276	Rps17	Small ribosomal subunit protein eS17	242626.4	242212.8	291164.7	292529.7	**267133**	266147.2	251388.3	305619.8	312858.2	**284003**	**0.9406**	**0.44558**
O88952	Lin7c	Protein lin-7 homolog C	127951.4	111095.7	131938.4	154747.7	**131433**	122662.2	114412.7	140591.6	138130.4	**128949**	**1.0193**	**0.82815**
Q9QYX7	Pclo	Protein piccolo	49942.85	49731.89		34532.7	**44736**	11083.92	26248.09			**18666**	**2.3966**	**0.05749**
Q3TYX3	Smyd5	Histone-lysine N-trimethyltransferase SMYD5	30712.55	32701.48	31132.86	25959.78	**30127**	21007.86	27711.29	29785.24	18829.88	**24334**	**1.2381**	**0.10148**
Q99LR1	Abhd12	Lysophosphatidylserine lipase ABHD12	182099.5	183191.9	193327	202191.1	**190202**	174667.1	196600.3	206186.6	224546.9	**200500**	**0.9486**	**0.40144**
Q8C0J2	Atg16l1	Autophagy-related protein 16-1	18698.11	20481.94	19726.27	17992.24	**19225**	19075.97	17398.89	13756.37	15463.82	**16424**	**1.1705**	**0.07119**
O08919	Numbl	Numb-like protein	36022.21	44305.21	47331.93	45329.31	**43247**	36275.73	40740.56	42189.7	41406.77	**40153**	**1.0771**	**0.31467**
P41242	Matk	Megakaryocyte-associated tyrosine-protein kinase												
Q8BP47	NARS1	Asparagine--tRNA ligase, cytoplasmic	214559.2	211452.6	191038.5	175255	**198076**	182160.6	205960	166262	163490.8	**179468**	**1.1037**	**0.21476**
P07901	Hsp90aa1	Heat shock protein HSP 90-alpha	1432832	1679990	1677409	1380802	**1542758**	1663132	1404850	1572485	1481525	**1530498**	**1.0080**	**0.90351**
P61514	Rpl37a	Large ribosomal subunit protein eL43	85847.02	87441.2	94171.93	92815.59	**90069**	87736.71	89102.34	115846.5	99583.12	**98067**	**0.9184**	**0.28395**
Q8BGU5	Ccny	Cyclin-Y	18971.8	14339.71	15302	14635.95	**15812**	13317.22	15644.67	15133.03	11329.82	**13856**	**1.1412**	**0.22654**

Q91Z31	Ptbp2	Polypyrimidine tract-binding protein 2	110165.5	111513.9	119197	127669.8	117137	127665.1	122826.4	142371.5	139887.7	133188	0.8795	0.04146
Q80TT2	Baiap3	BAI1-associated protein 3												
Q9DCW4	Etfb	Electron transfer flavoprotein subunit beta	478161.4	458962.2	508923	554922.3	500242	506332.3	485527.5	490193.5	532966.9	503755	0.9930	0.88612
Q6ZPS6	Ankib1	Ankyrin repeat and IBR domain-containing protein 1	11512.19		9728.846	9182.228	10141	8299.108	9813.14	7008.402	10922.59	9011	1.1254	0.37933
Q8BXR9	Osbpl6	Oxysterol-binding protein-related protein 6	12821.42	13500.92	11483.77	11104.5	12228	12808.91	11358.09	14924.17	12756.69	12962	0.9433	0.45772
P54797	Tango2	Transport and Golgi organization 2 homolog	24513.55	26283.09	27668.01	18125.77	24148	23491.38	23556.74	21815.66	24539	23351	1.0341	0.72758
Q62523	Zyx	Zyxin	48464.85	45008.43	45721.5	54977	48543	37114.42	48107.74	51925.15	49201.75	46587	1.0420	0.63988
Q922D4	Ppp6r3	Serine/threonine-protein phosphatase 6 regulatory s	10632.3	7831.496	6887.595	6537.058	7972	7548.772	7354.041	5091.353	6721.045	6679	1.1936	0.27734
P32233	Drg1	Developmentally-regulated GTP-binding protein 1	57004.55	60874	66918.55	60595.61	61348	67469.08	59835.84	70880.86	70935.01	67280	0.9118	0.12444
Q9ER00	Stx12	Syntaxin-12	62076.07	60529.61	56230.16	76505.52	63835	62516.14	63830.8	81693.34	77359.27	71350	0.8947	0.29292
Q8K400	Stxbp5	Syntaxin-binding protein 5	56581.68	47805.23	42276.26	38425.45	46272	42827.21	36156.32	41333.21	41855.33	40543	1.1413	0.22274
Q9CXR1	Dhrs7	Dehydrogenase/reductase SDR family member 7	53389.93	53400.9	50354.78	58104.41	53813	55135.27	56033.07	54754.49	60120.2	56511	0.9523	0.23003
P32883	Kras	GTPase KRas	39369.75	32165.85	28809.42	34989.97	33834	30914.59	38852.13	32347.56	30518.6	33158	1.0204	0.82701
Q8BIF2	Rbfox3	RNA binding protein fox-1 homolog 3	38203.98	36782.29	34619.84	40024.58	37408	39555.04	31741.57	39556.32	41063.63	37979	0.9850	0.81961
P17563	Selenbp1	Methanethiol oxidase	82173.32	79094.46	71512.88	70299.82	75770	76495.86	65650.57	56243.74	52386.7	62694	1.2086	0.07594
Q8BMG8	Slc25a32	Mitochondrial folate transporter/carrier	17170.36	16166.77	16439.12	20181.21	17489	14628.09	13049.92	13163.96	14291.19	13783	1.2689	0.01019
Q3TCH7	Cul4a	Cullin-4A	17901.01	24472.94	21207.28	19208.38	20697	23020.28	22647.67	16975.19	24723.45	21842	0.9476	0.62306
O35066	Kif3c	Kinesin-like protein KIF3C	8614.392	11136.41	11561.52	12234.49	10887	15527.46	18081.84	11891.49	10527.95	14007	0.7772	0.15033
P28867	Prkcd	Protein kinase C delta type	108276.6	101659.7	89107.36	100660.3	99926	108416	110082.8	98605.7	104956.7	105515	0.9470	0.28138
Q6PCP5	Mff	Mitochondrial fission factor	51825.88	51188.06	54144.1	61679.81	54709	47180.32	54271.32	52025.73	64309.39	54447	1.0048	0.95364
O88685	Psmc3	26S proteasome regulatory subunit 6A	140181.1	143355.9	152805.7	133932.5	142569	144534.3	145588.9	154541.9	153129.3	149449	0.9540	0.19297
P00920	Ca2	Carbonic anhydrase 2	66052.91	87277.84	79985.95	76521.8	77460	73565.6	72370.4	72128.15	51268.09	67333	1.1504	0.19517
P48036	Anxa5	Annexin A5	3994375	4104367	3882329	3363042	3836028	4067801	3739217	3554057	3244748	3651456	1.0505	0.46723
O88736	Hsd17b7	3-keto-steroid reductase/17-beta-hydroxysteroid de	31388.92	30185.58	48160.57	40135.41	37468	32468.33	34590.34	45573.68	49058.56	40423	0.9269	0.63113
Q5IRJ6	Slc30a9	Proton-coupled zinc antiporter SLC30A9, mitochond	36481.34	35709.9	37427.04	41143.74	37691	36503.71	39327.93	37441.66	38683.27	37989	0.9921	0.83334
Q9CPU2	Ndufb2	NADH dehydrogenase [ubiquinone] 1 beta subcomp	62832.31	63317.81	84636.59	101345.6	78033	75416.79	65696.55	107437.4	96251.69	86201	0.9052	0.56176
P98086	C1qa	Complement C1q subcomponent subunit A	10941.99	10947.04	14170.33	11864.4	11981	9861.379	8946.612	9484.045	16326.27	11155	1.0741	0.67785
Q99N93	Mrpl16	Large ribosomal subunit protein uL16m	9405.318	9707.406	9773.436	12406.79	10323		10064.77	11607.56	16086.86	12586	0.8202	0.24597
Q91YM2	Arhgap35	Rho GTPase-activating protein 35	25016.59	25853.36	23167.48	25791.62	24957	21379.86	25323.58	19581.21	20449	21683	1.1510	0.05983
Q9CZN7	Shmt2	Serine hydroxymethyltransferase, mitochondrial	52668.07	52177.71	65959.34	61758.26	58141	51210.6	54855.29	64165.16	68172.54	59601	0.9755	0.78909
Q9D172	Gatd3	Glutamine amidotransferase-like class 1 domain-co	261917.2	261018	238757.9	287349.8	262261	262231.3	275221.8	267868.3	265015.9	267584	0.9801	0.62429
Q7TQH0	Atxn2l	Ataxin-2-like protein	66420.98	67711.57	70449.37	68405.49	68247	70768.59	67224.73	78692.67	80084	74192	0.9199	0.11354
Q3SXD3	Hddc2	5'-deoxynucleotidase HDDC2	39346.95	47802.96	31273.15	33122.4	37886	23991.67	36232.4	29088.25	27735.23	29262	1.2947	0.10525
Q99JT9	Adi1	Acireductone dioxygenase	7559.184	5552.869	8225.787		7113							
Q6P5F9	Xpo1	Exportin-1	45732.04	47293.87	48839.39	42974.33	46210	49326.55	50125.88	48184.25	50239.43	49469	0.9341	0.05076
Q9JKY5	Hip1r	Huntingtin-interacting protein 1-related protein	12537.88	16104.69	13409.56	13543.17	13899	12288.15	15863.9	12597.94	13708.72	13615	1.0209	0.80752
Q9CPW0	Cntnap2	Contactin-associated protein-like 2	39495.38	41157.11	31011.22	39764.26	37857	40871.29	40834.04	30315.26	32692.84	36178	1.0464	0.65622
Q8BYL4	Yars2	Tyrosine--tRNA ligase, mitochondrial	11412.71	9184.072	10277.73	12600.9	10869	9047.039	14587.81	11018.68	11745.91	11600	0.9370	0.61098
O08532	Cacna2d1	Voltage-dependent calcium channel subunit alpha-2	80577.05	88228.73	80615.29	102165	87897	81307.07	94340.45	89047.45	86562.71	87814	1.0009	0.98909
Q9WUA2	Farsb	Phenylalanine--tRNA ligase beta subunit	254697.2	258558.7	273863.7	263844.8	262741	254176	281839	313982.6	293180.8	285795	0.9193	0.12987
Q8CHG3	Gcc2	GRIP and coiled-coil domain-containing protein 2	9509.036	15573.51	17227.18	13621.96	13983	17171.32	13197.38	13759.8	20098.52	16057	0.8708	0.40458
Q9JI19	Fibp	Acidic fibroblast growth factor intracellular-binding p	18027.14	17985.82	15370.62	16888.86	17068	12735.44	17721.98	11409.87	14304.59	14043	1.2154	0.08994
A2AWP8	Arhgef10l	Rho guanine nucleotide exchange factor 10-like prot	10377.69	6251.591	12713.85	5852.874	8799	12783.09	6291.398	8582.506	11202.82	9715	0.9057	0.69049
O09106	Hdac1	Histone deacetylase 1	22543.62	24445.17	27755.15	19838.59	23646	20124.29	21252.4	18660.09	24619.82	21164	1.1172	0.28047
Q9D7X8	Ggct	Gamma-glutamylcyclotransferase	26012.45	29033.8	32782.66	22870.7	27675	24451.14	28038.63	23082.44	36237.51	27952	0.9901	0.94160
Q8BWP8	B4gat1	Beta-1,4-glucuronyltransferase 1	9517.33	12723.81	17650.15	7803.853	11924	9081.178		12231.54	12766.37	11360	1.0497	0.84474
Q8R164	Bphl	Valacyclovir hydrolase	42325.59	37900.41	49577.3	48771.07	44644	45069.75	44845.07	41475.78	47617.53	44752	0.9976	0.97275
P63085	Mapk1	Mitogen-activated protein kinase 1	233375.4	228688.6	223891.4	210900	224214	215167	219739.7	209874.6	199797.5	211145	1.0619	0.08976
Q8K1E0	Stx5	Syntaxin-5	52414.9	54454.04	53723.43	54960.15	53888	55103.73	55158.64	47852.46	54349.55	53116	1.0145	0.69078
Q9WUR2	Eci2	Enoyl-CoA delta isomerase 2	62439.88	63279.97	67991	79422.11	68283	62870	71188.38	81184.56	80075.76	73830	0.9249	0.37578
Q91WS0	Cisd1	CDGSH iron-sulfur domain-containing protein 1	196537.7	170873.2	193598.4	207603.1	192153	193431.3	188543.4	217578.6	209443.9	202249	0.9501	0.36345
Q9JHG2	Rcan2	Calcipressin-2												
Q99LX0	Park7	Parkinson disease protein 7 homolog	246741.9	256064.8	220426.8	192963.6	229049	240915.4	233765.6	189574.1	177853.5	210527	1.0880	0.41573
Q8BGH7	Cdc42se2	CDC42 small effector protein 2	15669.84	14755.19	17259.58	22208.48	17473	15913.22	21610.77	17424.82	19168.51	18529	0.9430	0.62702
Q8CIQ7	Dock3	Dedicator of cytokinesis protein 3	4798.686	4276.131	4544.512	5455.272	4769	5468.542	5131.198	3916.816	3958.98	4619	1.0324	0.76196
Q9DCU2	Pllp	Plasmolipin	1703351	1628258	1065348	1843303	1560065	1508990	2036412	1370875	1459412	1593922	0.9788	0.88656
Q9CWU6	Uqcc1	Ubiquinol-cytochrome-c reductase complex assemb	35536.4	36648.99	38698.26	34270.75	36289	34684.06	42279.71	26557.14	29916.59	33359	1.0878	0.43912
Q9D3P8	Plgrkt	Plasminogen receptor (KT)	11940.55	17716.02	19261.74	21220.24	17535	14589.31	18751.49	29681.92	25937.51	22240	0.7884	0.27902
P61222	Abce1	ATP-binding cassette sub-family E member 1	84560.31	91656.41	95642.29	76299.95	87040	86212.49	89274.26	84972.83	86315.8	86694	1.0040	0.93917

Q3UN90	Lyrm9	LYR motif-containing protein 9												
P62315	Snrpd1	Small nuclear ribonucleoprotein Sm D1	162569.9	161412.9	172520.3	177674.1	168544	155678.9	155601.1	151760.1	159768.7	155702	1.0825	0.02357
Q99L47	St13	Hsc70-interacting protein	472726.9	486258.5	470627.6	423493.8	463277	474800.4	489410.2	451775	439692.3	463919	0.9986	0.97220
Q9ERA0	Tfcp2	Alpha-globin transcription factor CP2	18220.21	19931.78	25908.02	24720.91	22195	19983.29	20304.58	14136	15196.36	17405	1.2752	0.09775
Q9QWY8	Asap1	Arf-GAP with SH3 domain, ANK repeat and PH domai	14604.7	17796.86	16857.44	16528.22	16447	21795.36	17955.93	17283.25	18091.31	18781	0.8757	0.10431
Q8VC85	Lsm1	U6 snRNA-associated Sm-like protein LSm1	11387.4	10212.08	13000.3	9871.522	11118	10739.67	12146.99	11452.41	11713.03	11513	0.9657	0.62418
Q9D387	Lamp5	Lysosome-associated membrane glycoprotein 5	14233.33	21859.91	9896.565	15905.93	15474	10151.74	20315.95		15190.4	15219	1.0167	0.94949
Q6PAR5	Gapvd1	GTPase-activating protein and VPS9 domain-contain	34807.12	36274.34	38792.76	34694.89	36142	39016.85	36178.15	36998.21	41135.24	38332	0.9429	0.18494
P47809	Map2k4	Dual specificity mitogen-activated protein kinase kin	44279.58	49171.55	41582.14	36617.84	42913	47676.01	40250.06	39112.62	34206.24	40311	1.0645	0.52157
Q8CCM6	Timm21	Mitochondrial import inner membrane translocase subunit Tim21												
Q00623	Apoa1	Apolipoprotein A-I	662531.2	515767.9	879395.3	341819.4	599878	551466.1	388161.1	616136.3	473537	507325	1.1824	0.48402
Q9DB43	Zfpl1	Zinc finger protein-like 1	8519.747	7107.959	8815.916	5971.637	7604	12269.32	11593.21	4196.633	12301.93	10090	0.7536	0.27675
Q9CZX0	Elp3	Elongator complex protein 3	23573.7	21624.59	20715.01	19378	21323	18484.42	22611.2	18900.06	17186.42	19296	1.1051	0.21421
Q8VD63	Tspyl4	Testis-specific Y-encoded-like protein 4	25427.67	25581.58	31004.25	29151.35	27791	28094.96	22048.91	22094.55	27634.53	24968	1.1131	0.24028
Q64191	Aga	N(4)-(beta-N-acetylglucosaminyl)-L-asparaginase	45172.64	38051.21	44690.14	44534.88	43112	44954.19	43235.95	52198.54	46212.12	46650	0.9242	0.21937
Q91V61	Sfxn3	Sideroflexin-3	413083	361807.6	369652.3	450301.5	398711	449348.5	387306.3	469594.1	469542.3	443948	0.8981	0.16131
Q920B9	Supt16h	FACT complex subunit SPT16	16364.06	18277.49	18044.5	16502.76	17297	18043.54	19735.21	21313.08	25934.07	21256	0.8137	0.06649
Q91YT8	Tmem63a	CSC1-like protein 1						5207.914				5208	0.0000	
Q9DBS1	Tmem43	Transmembrane protein 43	68401.13	64760.73	70717.05	72987.44	69217	67789.84	67201.78	74415.95	74788.02	71049	0.9742	0.52324
Q9JM96	Cdc42ep4	Cdc42 effector protein 4	49714.02	44278.69	42712.87	39860.05	44141	46473.01	43554.8	46649.91	43713.61	45098	0.9788	0.68388
Q9CZU3	Mtrex	Exosome RNA helicase MTR4	64163.42	67505.73	86395.61	74144.94	73052	64710.47	77325.84	59731.23	67851.59	67405	1.0838	0.39383
Q9CXW4	Rpl11	Large ribosomal subunit protein uL5	537401.6	532874.8	602043.2	558612.6	557733	567808.3	546339.2	681325	668888.8	616090	0.9053	0.17452
Q923Z3	Mto1	Protein MTO1 homolog, mitochondrial												
Q8CI51	Pdlim5	PDZ and LIM domain protein 5	64460.79	67826.95	66481.28	61745.39	65129	61622.55	59931.72	59767.66	58762.07	60021	1.0851	0.01248
P20352	F3	Tissue factor	21055.96	20906.37	18346.18	21421.63	20433	19716.51	19893.65	19903.32	19227.51	19685	1.0380	0.34022
Q810D6	Grwd1	Glutamate-rich WD repeat-containing protein 1	5869.58	8160.729	7077.066	6311.085	6855	9377.69	7245.417		8168.419	8264	0.8295	0.13333
P19253	Rpl13a	Large ribosomal subunit protein uL13	403162	411535.3	471111.5	471865.2	439418	448349.2	441571.3	490691.9	522704.4	475829	0.9235	0.22022
P70452	Stx4	Syntaxin-4	31505.47	32794.43	28484.65	35995.71	32195	31148.13	31345.99	30894.43	28848.24	30559	1.0535	0.36235
Q9QXA5	Lsm4	U6 snRNA-associated Sm-like protein LSm4	37668.64	33278.56	43717.23	38743.02	38352	34681.68	32082.54	43576.71	43520.16	38465	0.9971	0.97637
P62320	Snrpd3	Small nuclear ribonucleoprotein Sm D3	277336.2	268316.6	289427.6	329166.4	291062	301141.5	288425	331743.3	336608.1	314479	0.9255	0.23649
Q80YD1	Supv3l1	ATP-dependent RNA helicase SUPV3L1, mitochondri	23225.82	15380.49	16305.95	19942.19	18714	13968.32	15813.49	11537.6	16772.23	14523	1.2886	0.09736
Q6URW6	Myh14	Myosin-14	84155.99	84875.73	91269.37	100462.6	90191	84021.85	82952.48	91631.3	97303.65	88977	1.0136	0.81882
D3YZP9	Ccdc6	Coiled-coil domain-containing protein 6	38854.73	38638.09	36276.61	34719.88	37122	35288.84	42637.34	27430.43	32880.35	34559	1.0742	0.46771
Q3UJB9	Edc4	Enhancer of mRNA-decapping protein 4	35363.5	35713.29	35204.67	37925.07	36052	35187.64	33566.14	38887.81	39127.38	36692	0.9825	0.68746
P27048;P63163	Snrpb;Snrpn	Small nuclear ribonucleoprotein-associated protein	248248.3	238238.7	262867.2	255738.5	251273	254026.5	242264.7	259585.9	293264.4	262285	0.9580	0.39943
P97427	Crmp1	Dihydropyrimidinase-related protein 1	800222.9	808687.3	741740.9	776391.7	781761	789653.4	802639.8	712420.9	708060.6	753194	1.0379	0.36435
P21278	Gna11	Guanine nucleotide-binding protein subunit alpha-1:	95795.45	91633.38	86967.27	104972.4	94842	92724.76	105360.6	87408.77	90285.41	93945	1.0096	0.87590
Q8VHL1	Setd7	Histone-lysine N-methyltransferase SETD7	40750.31	41690.92	46237.15	34441.7	40780	43103.15	40208.13	41018.91	41913.36	41561	0.9812	0.76596
Q8VDZ4	Zdhhc5	Palmitoyltransferase ZDHHC5			15672.66		15673	21988.38				21988	0.7128	
Q8C7X2	Emc1	ER membrane protein complex subunit 1	75822.98	71906.02	82029.89	83870.16	78407	77594.88	81592.11	87373.84	92978.52	84885	0.9237	0.18748
P06801	Me1	NADP-dependent malic enzyme	184693.7	193611.9	172697	156740.9	176936	191303.8	199504.9	174165.8	153807.3	179695	0.9846	0.83750
Q9CQW1	Ykt6	Synaptobrevin homolog YKT6	78193.82	78340.45	83078.09	73169.23	78195	72955.16	71069.34	77220.77	73985.2	73808	1.0594	0.11708
P70268	Pkn1	Serine/threonine-protein kinase N1	20651.6	18315.98	18687.34	10849.05	17126	16489.02	16328.98	15921.74	16672.53	16353	1.0473	0.73273
Q5SUC9	Sco1	Protein SCO1 homolog, mitochondrial	24668.71	25705.05	27617.67	32407.75	27600	25996.37	24641.59	24895.07	26842.1	25594	1.0784	0.30506
Q9CQZ5	Ndufa6	NADH dehydrogenase [ubiquinone] 1 alpha subcom	106999.6	103680.9	115001.5	108535.2	108554	107131.1	113546.1	127743.5	121211.9	117408	0.9246	0.13192
Q8BZF8	Pgm5	Phosphoglucomutase-like protein 5	12107.8	10847.97	13508.82	10578.15	11761	10479.04	9181.08	8146.422	10335.66	9536	1.2334	0.04232
Q5U4D9	Thoc6	THO complex subunit 6 homolog	52960.71	37703.14	52421.76	49868.95	48239	43308.54	48770.29	36108.21	47421.25	43902	1.0988	0.37931
Q9Z0X1	Aifm1	Apoptosis-inducing factor 1, mitochondrial	110541.2	113117.3	126637	133864	121040	120099.9	123312.9	134100.8	131856.6	127343	0.9505	0.36814
Q9Z0S6	Cldn10	Claudin-10												
O55102	Bloc1s1	Biogenesis of lysosome-related organelles complex	12073.81	12457.29	12918.93	10565.28	12004	13457.71	16213.71	13280.88	10505.6	13364	0.8982	0.32599
Q80TV8	Clasp1	CLIP-associating protein 1	124452.5	137787.1	122267.1	147585.2	133023	141257.3	146345.6	135792	133202.2	139149	0.9560	0.39082
Q8R310	Tmcc3	Transmembrane and coiled-coil domain protein 3	5321.271	5378.757	8057.836	10171.77	7232	2788.725	5407.844	11217.11	6456.879	6468	1.1182	0.72992
P59017	Bcl2l13	Bcl-2-like protein 13	20740.24	20527.21	21789.02	21291	21087	20295.79	22632.79	24778.89	26327.5	23509	0.8970	0.12120
Q9WVK8	Cyp46a1	Cholesterol 24-hydroxylase	6558.298	4141.589	10711.15	5667.137	6770	6268.743	5385.928	3169.559	10490.49	6329	1.0697	0.83913
O89086	Rbm3	RNA-binding protein 3	247976.7	189027.4	194645	272960.7	226152	171199.2	235615.9	233878.3	210206.5	212725	1.0631	0.61600
Q93092	Taldo1	Transaldolase	264130.5	259504.5	266978.6	221741.8	253089	256468.7	250701.8	251262.3	232473	247726	1.0216	0.66532
Q8BQU6	Gjc2	Gap junction gamma-2 protein												
P27545	Cers1	Ceramide synthase 1	15303.88	18939.84	15426.24	20769.64	17610	13663.51	16410.58	26006.65	23485.35	19892	0.8853	0.50291
Q80X80	C2cd2l	Phospholipid transfer protein C2CD2L	19858.37	23790.99	21534.07	28553.4	23434	26518.4	23696.21	23457.08	25917.41	24897	0.9412	0.50012

P68510	Ywhah	14-3-3 protein eta	334043.5	330429.7	332653.1	306202.8	325832	366620.2	279319.5	341678.2	326634.8	328563	0.9917	0.89328
Q9Z1Z2	Strap	Serine-threonine kinase receptor-associated protein	70367.22	73124.38	64748.63	63797.28	68009	65769.89	71381.89	60937.6	64861.23	65738	1.0346	0.49210
Q9CWZ7	Napg	Gamma-soluble NSF attachment protein	162602.4	156059.2	149295.2	175898.4	160964	159866.7	157360.6	147949.6	150160.5	153834	1.0463	0.30400
P61458	Pcbd1	Pterin-4-alpha-carbinolamine dehydratase	30996.96	27159.75	26609.4	24256.23	27256	31313.26	24913.54	29520.24	24065.83	27453	0.9928	0.93274
P06837	Gap43	Neuromodulin	24022.83	16226.69	23341	23058.44	21662	14559.52	18373.29	15641.37	15701.38	16069	1.3481	0.03105
Q61234	Snta1	Alpha-1-syntrophin	206064.7	211748.7	208628.8	233304.5	214937	195850.8	205662.8	244288.4	229893	218924	0.9818	0.76434
Q9DB70	Fundc1	FUN14 domain-containing protein 1	7605.185	8947.18	7780.062	10463.66	8699		6792.923	7158.102	7774.964	7242	1.2012	0.13428
Q8BLE7	Slc17a6	Vesicular glutamate transporter 2		25756.88	42990.54	52846.2	40531		54048.79	56698.28		55374	0.7320	0.24406
Q6PHZ2	Camk2d	Calcium/calmodulin-dependent protein kinase type	102811.8	105048.6	91917.28	97782.89	99390	84595.24	112059.1	88347.16	89588.23	93647	1.0613	0.43572
Q9CQ88	Tspan31	Tetraspanin-31	5279.119	19460.49	13686.89	22120.64	15137	23491.49	16377.97	33133.12	14762.43	21941	0.6899	0.27030
Q3UHC7	Dab2ip	Disabled homolog 2-interacting protein	15246.18	13711.47	17230.08	13146.88	14834	14924.94	14520.45	12398.79	16033.47	14469	1.0252	0.76967
Q9D0D3	Mtpap	Poly(A) RNA polymerase, mitochondrial	5905.354	4227.918	2753.857		4296	9611.986	4512.586	5646.283	6076.565	6462	0.6648	0.21054
P05201	Got1	Aspartate aminotransferase, cytoplasmic	574732.4	574642.1	456398.3	436548.2	510580	575058.7	509813.8	453579.1	428835.3	491822	1.0381	0.71738
Q6PFR5	Tra2a	Transformer-2 protein homolog alpha	29710.1	31066.98	32183.98	37580.51	32635	33284.5	33896.84	33693.53	42093.16	35742	0.9131	0.29906
Q8R010	Aimp2	Aminoacyl tRNA synthase complex-interacting multi	201253.2	215207.1	184893.5	204482.7	201459	194082.8	232612.2	204729.2	204400.9	208956	0.9641	0.49716
Q9Z2N8	Actl6a	Actin-like protein 6A	39180.36	38703.64	35968.08	38983.64	38209	36095.22	40097.45	45042.22	41572.56	40702	0.9388	0.25902
Q91YP2	Nln	Neurolysin, mitochondrial	14200.2	11996.64	16704.05	11874.21	13694	11199.65	12942.3	9670.227	12985.39	11699	1.1705	0.20041
Q921I1	Tf	Serotransferrin	1058124	1118040	1178929	503182.7	964569	960265.4	759056.9	774505.4	583006.4	769209	1.2540	0.30392
Q0KK55	Kndc1	Kinase non-catalytic C-lobe domain-containing prote	11696.11	9608.162		17779.85	13028	12961.65	9133.208			11047	1.1793	0.60822
Q8BGZ4	Cdc23	Cell division cycle protein 23 homolog	13011.07	13057.8	18028.69	15170.69	14817	15736	13393.47	17883.47	21660.11	17168	0.8630	0.30926
Q8K3C3	Lzic	Protein LZIC	40240.82	48525.39	51898.98	37609.34	44569	45284.92	45843.13	39388.72	38723.34	42310	1.0534	0.58029
Q80X50	Ubap2l	Ubiquitin-associated protein 2-like	83373.48	82935.18	90202.21	92557.85	87267	89556.79	83285.02	97950.34	101044.4	92959	0.9388	0.27214
Q9CX13	Cnih4	Protein cornichon homolog 4	47125.31	45495.82	52054.61	49876.04	48638	41812.82	51726.19	36817.01	45952.35	44077	1.1035	0.23779
Q60766	Irgm1	Immunity-related GTPase family M protein 1	32112.32	18988.13	8387.896	16658.14	19037	12895.13	14760	12636.94	7982.92	12069	1.5773	0.22269
P35235	Ptpn11	Tyrosine-protein phosphatase non-receptor type 11	45209.57	50724.46	44853.2	39569.95	45089	46393.61	47510.32	41359.23	43264.68	44632	1.0102	0.87016
Q80UJ7	Rab3gap1	Rab3 GTPase-activating protein catalytic subunit	19531.25	19758.91	17404.76	17394.63	18522	15446.34	15645.61	16418.03	17381.34	16223	1.1417	0.02624
Q99LB2	Dhrs4	Dehydrogenase/reductase SDR family member 4	55765.24	62454.5	69638.52	74377.99	65559	67026.66	62319.11	71011.84	73910.55	68567	0.9561	0.55357
Q6P1I6	Psd2	PH and SEC7 domain-containing protein 2												
Q9QYY0	Gab1	GRB2-associated-binding protein 1	33568.12	31879.95	31819.13	29712.11	31745	30137.13	29127.33	31234.24	29463.56	29991	1.0585	0.10400
Q8CI71	Vps50	Syndetin	12388.76	15297.51	13986.01	13974.03	13912	12589.25	14473.87	10254.41	12535.04	12463	1.1162	0.21638
Q9JLN9	Mtor	Serine/threonine-protein kinase mTOR	51201.99	53793.17	48455.67	47967.34	50355	55216.01	54629.56	48868.97	53074.22	52947	0.9510	0.23576
Q91ZM2	Sh2b1	SH2B adapter protein 1	25323.18	33202.11	28013.68	32548.81	29772	29822.59	28435.36	21376.6	30544.84	27545	1.0809	0.45965
Q8CHC4	Synj1	Synaptojanin-1	120374.2	124830.4	105031.3	114819	116264	121992.9	120474.4	104843.8	102943.5	112564	1.0329	0.59517
Q9D710	Tmx2	Thioredoxin-related transmembrane protein 2	160414.8	149591.3	153430.7	179204	160660	159340.4	191892.8	180251.1	195115.3	181650	0.8844	0.09077
P58389	Ptpa	Serine/threonine-protein phosphatase 2A activator	78374.61	91450.06	86232.86	71036.48	81774	77833.86	73293.28	74271.16	69570.31	73742	1.1089	0.14441
Q8VEM8	Slc25a3	Solute carrier family 25 member 3	336068	346666.3	363457.1	402548.5	362185	351618.4	372287.5	348287.8	365185.9	359345	1.0079	0.86193
E9Q6P5	Ttc7b	Tetratricopeptide repeat protein 7B	14495.61	6810.756	8746.985	6768.318	9205	10027.8	11075.15	6945.292	8524.122	9143	1.0068	0.97654
Q9DCB8	Isca2	Iron-sulfur cluster assembly 2 homolog, mitochondr	45080.34	43619.84	41469.12	47595.07	44441	52131.34	52850.02	46758.64	53093.97	51208	0.8678	0.01400
Q8BGG7	Ubash3b	Ubiquitin-associated and SH3 domain-containing pr	40239.84	34212.01	32238.37	35249.66	35485	35554.27	31605.09	38331.65	36190.27	35420	1.0018	0.97758
P09602	Hmgn2	Non-histone chromosomal protein HMG-17	132021.9	127903.7	139461.9	556656.4	239011	107820.4	139930	238437.5	215118.8	175327	1.3632	0.58466
Q8VCH8	Ubxn4	UBX domain-containing protein 4	45141.23	43429.57	48088.22	49578.97	46559	49290.16	53700.64	40044.01	55183.52	49555	0.9396	0.44700
Q9D4H8	Cul2	Cullin-2	38852.07	42264.29	34576.73	34911.66	37651	36521.6	39303.59	34156.95	34251.93	36059	1.0442	0.49356
Q9CXT8	Pmpcb	Mitochondrial-processing peptidase subunit beta	65856.84	57464.59	66529.47	79116.41	67242	65354.05	69083.68	67754.26	65912.67	67026	1.0032	0.96369
Q91V41	Rab14	Ras-related protein Rab-14	191349.5	188586.4	190837.6	195199.2	191493	196843.3	196469.5	185119.4	197529.2	193990	0.9871	0.47372
Q9Z2Q6	Septin5	Septin-5	54850.8	68251.78	64134.8	51413.64	59663	50172.55	66559.55	55862.16	60801.53	58349	1.0225	0.81094
Q8R307	Vps18	Vacuolar protein sorting-associated protein 18 homo	15095.55	16581.97	19882.98	14816.29	16594	17300.81	16515.85	19112.61	22942.29	18968	0.8749	0.24557
Q9D6F9	Tubb4a	Tubulin beta-4A chain	360328.7	365678.2	327034.4	373230.5	356568	341675.4	332464	342861.9	338243.6	338811	1.0524	0.14042
Q9Z2W8	Gria4	Glutamate receptor 4	3145.464	4970.828		46786.03	18301		10496.43	11132.6		10815	1.6922	0.71142
O35344	Kpna3	Importin subunit alpha-4	50598.85	58420.82	55289.98	64033.12	57086	60768.42	56872.55	54414.17	60579.71	58159	0.9816	0.74960
Q8R1I2	Grp	Gastrin-releasing peptide												
P52019	Sqle	Squalene monooxygenase	32930.01	32643.95	48747.62	39728.29	38512	38780.33	40265.6	46732.91	57205.75	45746	0.8419	0.24750
Q501J6	Ddx17	Probable ATP-dependent RNA helicase DDX17	476935.8	515390.4	518307.3	560770.6	517851	506940.7	510966.5	586706.5	582149.6	546691	0.9472	0.33865
Q3THK7	Gmps	GMP synthase [glutamine-hydrolyzing]	54932.58	57449.85	55001.24	50417.46	54450	58955.18	52477.25	51049.99	45890.25	52093	1.0452	0.47079
Q8CC35	Synpo	Synaptopodin	19577.83	22083.25	21534.23	22890.23	21521	19735.06	18259.26	21292.54	23452.74	20685	1.0404	0.54849
Q9CQ48	Nudcd2	NudC domain-containing protein 2	23699.03	23404.06	30758.58	20123.19	24496	22079.14	21747.81	22391.23	24510.81	22682	1.0800	0.46482
P22723	Gabrg2	Gamma-aminobutyric acid receptor subunit gamma-2		8772.009			8772	28754.38		483.4007		14619	0.6000	
Q9DCC7	Isoc2b	Isochorismatase domain-containing protein 2B	3780.614	5076.712	4476.147	2938.964	4068	3973.61	4830.51	6745.727	5117.384	5167	0.7874	0.18822
Q8C460	Eri3	ERI1 exoribonuclease 3	14962.99	18843.88	15994.7	10441.25	15061	15168.27	17498.09	13898.82	15555.54	15530	0.9698	0.81282
Q9CR86	Carhsp1	Calcium-regulated heat stable protein 1	43271	42209.3	45530.16	44242.84	43813	46160.9	45982.59	58438.46	52094.11	50669	0.8647	0.06478

P10637	Mapt	Microtubule-associated protein tau	128219.9	139940.6	139777.9	151635.5	139893	141451	138367.3	161472.6	148870.5	147540	0.9482	0.31779
Q9D0A3	Arpin	Arpin	41017.23	47574.52	43729.96	38292.2	42653	49263.1	43083.29	42267.21	40288.09	43725	0.9755	0.71215
P16332	Mmut	Methylmalonyl-CoA mutase, mitochondrial	53755.02	49959.8	55548.07	61226.14	55122	53657.38	60207.38	61821.22	63673.44	59840	0.9212	0.19095
P63094;Q6R0H7	Gnas	Guanine nucleotide-binding protein G(s) subunit alpl	212350.3	193332	160083	218646.5	196103	181852.1	224536.9	158540.9	160277.9	181302	1.0816	0.49179
Q8CGY8	Ogt	UDP-N-acetylglucosamine--peptide N-acetylglucosa	45055.32	48241.63	46242.32	48086.39	46906	49239.07	49969.02	45980.88	44111.91	47325	0.9912	0.79941
Q8K268	Abcf3	ATP-binding cassette sub-family F member 3	51189.16	44520.97	44547.86	41719.52	45494	39091.13	43306.92	39844.41	42872.9	41279	1.1021	0.11312
Q8BTI8	Srrm2	Serine/arginine repetitive matrix protein 2	22313.56	25342.03	25194.61	29693.97	25636	23497.57	25483.48	26714.98	28652.95	26087	0.9827	0.81709
Q9CQV1	Pam16	Mitochondrial import inner membrane translocase s	7989.062	11165.17	10477.86	7832.607	9366	9069.133	11048.51	12485.11	12221	11206	0.8358	0.16196
O88844	Idh1	Isocitrate dehydrogenase [NADP] cytoplasmic	436319.2	430665.7	456995.6	363515	421874	418816.7	405769.7	441312.8	399353.4	416313	1.0134	0.81121
P53995	Anapc1	Anaphase-promoting complex subunit 1	7519.728	8715.936	11449.79	9579.659	9316	9133.195	8994.201	8656.377	8383.271	8792	1.0597	0.55724
Q9DCU6	Mrpl4	Large ribosomal subunit protein uL4m	12231.82	11013.02	16273.81	12514.11	13008	18300.66	17675.95	14476.53	19532.22	17496	0.7435	0.02859
Q8C0L8	Cog5	Conserved oligomeric Golgi complex subunit 5	23030.44	23158.35	19276.41	12462.17	19482	23332.06	19593.59	20483.07	22932.3	21585	0.9026	0.46062
Q9EST4	Psmg2	Proteasome assembly chaperone 2	98075.08	327575.3	94957.83	227751.9	187090	88908.89	73529.21	77559.78	256917.5	124229	1.5060	0.41333
Q99KX1	Mlf2	Myeloid leukemia factor 2	20530.62	20267.89	20876.93	19967.45	20411	16670.19	14836.49	19981.57	16568.24	17014	1.1996	0.02085
P28660	Nckap1	Nck-associated protein 1	41648.86	40922.95	40688.78	39634.42	40724	42911.12	44469.85	40795.66	42429.08	42651	0.9548	0.06708
Q9Z1K5	Arih1	E3 ubiquitin-protein ligase ARIH1	24227.96	27615.43	29671.67	29020.97	27634	16446.84	29975.43	14337.56	29911.04	22668	1.2191	0.30147
P16388	Kcna1	Potassium voltage-gated channel subfamily A memb	76676.2	76079.9	61768.8	83021.41	74387	73292.54	82738.38	61150.82	71935.52	72279	1.0292	0.74939
Q62351	Tfrc	Transferrin receptor protein 1	44904.95	38561.6	46134.02	46794.43	44099	39383.52	42215.26	48774.23	49675.2	45012	0.9797	0.78078
Q8QZT1	Acat1	Acetyl-CoA acetyltransferase, mitochondrial	455468.1	446879.7	499037.3	529073.1	482615	473424.4	507179.3	472889.6	515053.3	492137	0.9807	0.68295
Q3ULJ0	Gpd1l	Glycerol-3-phosphate dehydrogenase 1-like protein	127650.3	114315.1	115995.3	100415.2	114594	126557.9	105034.1	135263.9	127247	123526	0.9277	0.33620
Q3UGS4	Mcrip1	Mapk-regulated corepressor-interacting protein 1	48333.93	48904.1	45221.83	39649.73	45527	42591.69	43889.99	42086.57	48107.09	44169	1.0308	0.60950
Q9R013	Ctsf	Cathepsin F	24662.15	19680.86	19133.72	19892.84	20842	22004.87	25883.14	16070.61	16506.3	20116	1.0361	0.79527
Q8R0P4	Aamdc	Mth938 domain-containing protein	46565.15	50372.66	44171.84	41879.64	45747	56501.3	49279.1	40326.68	37904.96	46003	0.9944	0.95783
Q9D0M1	Prpsap1	Phosphoribosyl pyrophosphate synthase-associatec	48496.14	48834.18	42195.79	47339.53	46716	48707.79	48270.26	46793.64	43343.7	46779	0.9987	0.97565
O35343	Kpna4	Importin subunit alpha-3	17842.01	18286.16	16557.39	14891.51	16894	16002.14	20662.01	12118.19	16566.65	16337	1.0341	0.78003
Q8R0Y6	Aldh1l1	Cytosolic 10-formyltetrahydrofolate dehydrogenase	42622.95	42267.67	41853.53	33828.13	40143	33583.53	34670.05	42918.72	40712.38	37971	1.0572	0.51030
Q62277	Syp	Synaptophysin	54774.81	61207.2	52989.89	58038.61	56753	53411.24	64899.99	54339.97	61212.52	58466	0.9707	0.62274
P19536	Cox5b	Cytochrome c oxidase subunit 5B, mitochondrial	311869.2	292412.8	350704.1	374274.4	332315	333672.6	275273.2	314746.9	368765.6	323115	1.0285	0.74371
P48771	Cox7a2	Cytochrome c oxidase subunit 7A2, mitochondrial	18567.24	15304.44	16897.85	14186.26	16239	15565.19	15821.1	10534.52	8273.74	12549	1.2941	0.12986
P39054	Dnm2	Dynamin-2	164313	152087.3	175491.7	147347.8	159810	152609.8	160503.2	181496.4	171239.1	166462	0.9600	0.48456
Q9QXT0	Cnpy2	Protein canopy homolog 2	63639.95	59344.84	68701.8	65913.34	64400	52656.51	63711.87	52463.91	65368.16	58550	1.0999	0.19377
P61982	Ywhag	14-3-3 protein gamma	743314.4	804680.3	831285	846500.3	806445	797623.6	874665.1	690555.6	794615.3	789365	1.0216	0.71194
Q61330	Cntn2	Contactin-2	46587.56	42859.6	39498.82	57428.94	46594	49149.81	49969.25	36124.09	40884.13	44032	1.0582	0.63516
Q9QY42	Gpr37	Prosaposin receptor GPR37												
Q9D3D9	Atp5f1d	ATP synthase subunit delta, mitochondrial	182881.1	204572.2	179770.5	191427.7	189663	170293.8	190972.9	159444.2	192868	178395	1.0632	0.29572
Q8R2H9	Phospho1	Phosphoethanolamine/phosphocholine phosphatas	19758.88	20931.66	16882.98	16984.86	18640	19000.57	16475.36	13856.47	15192.79	16131	1.1555	0.14388
Q8R3Q6	Mix23	Protein MIX23								15089.19		15089	0.0000	
Q3UQ84	Tars2	Threonine--tRNA ligase, mitochondrial				27596.26	27596							
Q9CY50	Ssr1	Translocon-associated protein subunit alpha	169254.8	181142.7	150703.4	166336.8	166859	155743.5	192953.3	169038.8	165655	170848	0.9767	0.70591
Q8K2Q5	Chchd7	Coiled-coil-helix-coiled-coil-helix domain-containing	12877.24	13585.95	17021.87	19074.46	15640	13760.45	14990.55	20292.5	14636.29	15920	0.9824	0.89723
Q7TN98	Cpeb4	Cytoplasmic polyadenylation element-binding protei	13272.45	17140.66	17078.12	17755.02	16312	14344.06	15276.74	15957.84	16598.62	15544	1.0494	0.52334
P29533	Vcam1	Vascular cell adhesion protein 1	23455.29			15359.44	19407	16893.23	16316.64		9128.473	14113	1.3752	0.31661
Q8BMS4	Coq3	Ubiquinone biosynthesis O-methyltransferase, mitoc	15663.21	13061.79	14858.49	14186.23	14442	12212.06	19125	13458.08	15944.4	15185	0.9511	0.66318
O55229	Chkb	Choline/ethanolamine kinase	30040.43	38726.85	29218.29	28325.61	31578	30044.36	28338.91	22807.82	29809.96	27750	1.1379	0.24105
Q9CR89	Ergic2	Endoplasmic reticulum-Golgi intermediate compartr	10642.54	11062.76	12352.08	12812.08	11717	8884.756	12976.77	13356.39	13458.75	12169	0.9629	0.72265
Q62178	Sema4a	Semaphorin-4A		15305.29			15305		11471.77			11472	1.3342	
Q791V5	Mtch2	Mitochondrial carrier homolog 2	78962.76	83747.71	84300.8	99299.02	86578	83018.28	91889.91	100166.7	99749.09	93706	0.9239	0.27808
P48318	Gad1	Glutamate decarboxylase 1	16040.75	12965.1	8402.019	16517.35	13481			15380.55	10097.38	12739	1.0583	0.82972
Q80TH2	Erbin	Erbin	20727.17	19133.71	17909.9	20690.59	19615	18492.66	19032.65	13213.77	19167.52	17477	1.1224	0.22504
Q99KE1	Me2	NAD-dependent malic enzyme, mitochondrial	81331.41	74199.82	75209.54	83256.34	78499	76915.02	83872.41	76971.59	83937.47	80424	0.9761	0.54561
P17742	Ppia	Peptidyl-prolyl cis-trans isomerase A	2498242	2406505	2373724	2468161	2436658	2588509	2335674	2154670	2279550	2339601	1.0415	0.34868
B0V2N1	Ptprs	Receptor-type tyrosine-protein phosphatase S	8725.145	7474.508	8420.899	7534.3	8039	6341.002	8286.18	7427.962	7997.928	7513	1.0699	0.36194
Q99MP8	Brap	BRCA1-associated protein	35658.98	36443.8	32069.63	29001.41	33293	36449.93	37187.13	38186.61	34172.98	36499	0.9122	0.14579
Q9CQY2	Ramac	RNA guanine-N7 methyltransferase activating subun	50277.54	39267.47	59895.04	49935.32	49844	48246.25	34829.81	41806.71	39264.5	41037	1.2146	0.13246
P68134	Acta1	Actin, alpha skeletal muscle	152681.2	121099.9	109485	178666.2	140483	62327.07	70410.52	90707.15	68507.91	72988	1.9247	0.00703
Q8K1Z0	Coq9	Ubiquinone biosynthesis protein COQ9, mitochondri	18898.46	20439.87	19281.72	21498.04	20030	18005.3	21425.53	18842.11	21629.74	19976	1.0027	0.96208
Q9D554	Sf3a3	Splicing factor 3A subunit 3	27946.23	27105.01	27210.58	28768.22	27758	25447.59	25530.18	28221.19	28007.92	26802	1.0357	0.30460
Q9EPJ9	Arfgap1	ADP-ribosylation factor GTPase-activating protein 1				25943.56	25944	10056.87			12330.59	11194	2.3177	
Q5SUR0	Pfas	Phosphoribosylformylglycinamidine synthase	27322.67	22660.54	23004.86	20040.22	23257	22372.69	19388.81	20017.27	18679.46	20115	1.1562	0.11533

Q8BHS6	Armcx3	Armadillo repeat-containing X-linked protein 3	12829.96	12386.75	13505.75	11926.1	12662	12435.88	13132.62	12823.86	14600.34	13248	0.9558	0.35142
Q8BWU5	Osgep	tRNA N6-adenosine threonylcarbamoyltransferase		10567.64	10556.52	6969.087	9364	8262.519	7735.01	7771.496	10002.1	8443	1.1092	0.47165
P70202	Lxn	Latexin	461696.1	429232.9	405104.8	339072.6	408777	481022.3	345024.8	421203.6	461547	427199	0.9569	0.65933
P51859	Hdgf	Hepatoma-derived growth factor	112733.2	115726.7	148545	109643.7	121662	121964.9	102052.1	131221.4	124080.7	119830	1.0153	0.87311
Q922H2	Pdk3	[Pyruvate dehydrogenase (acetyl-transferring)] kinas	60682.77	61056.68	71710.53	73492.74	66736	71425.04	69100.93	79919.88	73294.36	73435	0.9088	0.15549
Q9DD02	Hikeshi	Protein Hikeshi	9777.463	9869.354			9823	7688.16	9761.245	6410.369		7953	1.2351	0.23467
Q9QWR8	Naga	Alpha-N-acetylgalactosaminidase	76808.22	64479.08	81095.52	69505.05	72972	72654.34	69695.66	77759.72	86108.06	76554	0.9532	0.51369
Q61235	Sntb2	Beta-2-syntrophin	238667	220509.4	183021.5	238019.4	220054	230577.2	241745.8	172874.6	197594.2	210698	1.0444	0.66289
P22682	Cbl	E3 ubiquitin-protein ligase CBL	35843	37766.43	38115.43	40053.05	37944	37162.89	38954.14	37312.02	38328.82	37939	1.0001	0.99602
Q7TNE3	Spag7	Sperm-associated antigen 7	19370.9	17993.9	19520.04	16259.79	18286	19008.37	16898.09	14779.12	15888.71	16644	1.0987	0.21194
Q9R060	Nubp1	Cytosolic Fe-S cluster assembly factor NUBP1	10597.32	13424.25	10764.05		11595	8034.01	10346.89		10033.93	9472	1.2242	0.14311
Q6ZPQ6	Pitpnm2	Membrane-associated phosphatidylinositol transfer	38973.15	34430.34	35375.43	41038.93	37454	36772.81	38475.14	38602.96	40341.74	38548	0.9716	0.54557
Q9CQU3	Rer1	Protein RER1	92244.63	94035.45	98254.23	102090.5	96656	98932.82	95597.3	105869	105621.6	101505	0.9522	0.19972
Q4LDD4	Arap1	Arf-GAP with Rho-GAP domain, ANK repeat and PH d	38302.13	34947.09	36803.32	29951.47	35001	38709.79	34426.32	35873.92	33778.05	35697	0.9805	0.75409
Q8BKC8	Pi4kb	Phosphatidylinositol 4-kinase beta	17515.45	16283.53	23667.95	12451.28	17480	11819.15	12909.52	8769.021	10718.39	11054	1.5813	0.04171
P49443	Ppm1a	Protein phosphatase 1A	90245.66	88354.61	80912.72	77716.8	84307	84097.96	75007.02	72027.08	63680.74	73703	1.1439	0.08568
Q9DC69	Ndufa9	NADH dehydrogenase [ubiquinone] 1 alpha subcom	181315.8	186442.7	208734.5	203042.8	194884	190670.1	197066.1	186050.8	201147.4	193734	1.0059	0.88076
Q80WJ7	Mtdh	Protein LYRIC	97267.53	89849.95	99187.44	117729.7	101009	96629.09	101532	109090.5	124167.7	107855	0.9365	0.44831
Q91Z69	Srgap1	SLIT-ROBO Rho GTPase-activating protein 1	29089.87	25704.64	28663.31	17846.98	25326	24379.04	28748.28	22546.9	25764.22	25360	0.9987	0.99122
Q9WUM4	Coro1c	Coronin-1C	58725.96	52707.93	62643.07	58785	58215	58962.96	58032.95	58917.02	61262.45	59294	0.9818	0.63613
Q6GYP7	Ralgapa1	Ral GTPase-activating protein subunit alpha-1	13324.17	13497.98	16603.15	13533.4	14240	14598.26	14772.43	14762.87	16539.68	15168	0.9388	0.34827
P17897	Lyz1	Lysozyme C-1	56751.74	37614.54	68961.44	23369.7	46674	52597.77	40675.39	26366.83	35327.84	38742	1.2047	0.51573
Q99N57	Raf1	RAF proto-oncogene serine/threonine-protein kinase	20125.13	10553.71	9482.246	8713.598	12219	17052.56	10231.71	5753.833	7216.445	10064	1.2141	0.57731
Q6PD28	Ppp2r5b	Serine/threonine-protein phosphatase 2A 56 kDa reg	74425.71	72520.81	71484.84	64807.62	70810	66319.63	64022.44	66574.65	64782.09	65425	1.0823	0.04838
Q9D1L9	Lamtor5	Ragulator complex protein LAMTOR5	11646.8	9598.775	11525.76	10391.94	10791	11254.33	10959.68	10406.31	10341.26	10740	1.0047	0.92799
Q99JI4	Psmd6	26S proteasome non-ATPase regulatory subunit 6	83678.69	81456.46	81594.88	78695.46	81356	77525.99	84200.19	74863.7	81573.58	79541	1.0228	0.46275
Q9QYI4	Dnajb12	DnaJ homolog subfamily B member 12	93905.22	60437.27	80202.19	66368.95	75228	52333.63	59460.05	46113.09	64002.46	55477	1.3560	0.05806
Q62376	Snrnp70	U1 small nuclear ribonucleoprotein 70 kDa	106263.4	113732	131787.8	151032.2	125704	135665.4	123348.4	138609.4	151071.1	137174	0.9164	0.35729
P07934	Phkg1	Phosphorylase b kinase gamma catalytic chain, skeletal muscle/heart isoform												
O35218	Cpsf2	Cleavage and polyadenylation specificity factor subu	7397.139	7212.003	8339.707	8556.374	7876	6815.12	8658.208	7414.556	6989.723	7469	1.0545	0.47498
Q8R050	Gspt1	Eukaryotic peptide chain release factor GTP-binding	37068.52	38126.37	35785.53	27947	34732	35813.66	33225.81	29287.5	34169.69	33124	1.0485	0.57267
Q8C4B4	Unc119b	Protein unc-119 homolog B	28858.51	27148.62	33760.93	33813.88	30895	31760.23	29997.14	30321.24	33333.41	31353	0.9854	0.81475
Q9Z1P6	Ndufa7	NADH dehydrogenase [ubiquinone] 1 alpha subcom	33113	34759.48	36088.46	38828.2	35697	34976.93	44377.49	44553.38	44487.18	42099	0.8479	0.05307
Q9WVM1	Racgap1	Rac GTPase-activating protein 1	30842.88	29823.74	28838.4	31882.12	30347	35369.03	29740.77	31282.74	32244.61	32159	0.9436	0.22992
P30416	Fkbp4	Peptidyl-prolyl cis-trans isomerase FKBP4	167239	204216.3	214372.6	175504.5	190333	205862.7	168917.7	202147.4	192670.1	192399	0.9893	0.88748
Q9D7I5	Lhpp	Phospholysine phosphohistidine inorganic pyrophos	1504.538	2639.588	5598.345	2339.179	3020	3075.714	2319.867		2159.55	2518	1.1993	0.66211
P59016	Vps33b	Vacuolar protein sorting-associated protein 33B	24869.78	24723.56	26145.69	23463.29	24801	22074.75	25614.14	26178.51	22920.83	24197	1.0249	0.61642
Q71LX4	Tln2	Talin-2	138469.2	133126.6	132340.3	132175.4	134028	136339.7	138463	138497.1	138564.4	137966	0.9715	0.04804
P51880	Fabp7	Fatty acid-binding protein, brain	1806591	2389774	2320708	2311404	2207119	1949763	2097596	2178324	2008708	2058598	1.0721	0.34115
Q149L6	Dnajb14	DnaJ homolog subfamily B member 14	9316.944	10314.24	11195.25	10339.88	10292	10906.63	10882.82	9249.418	11632.16	10668	0.9647	0.57428
Q9CS42	Prps2	Ribose-phosphate pyrophosphokinase 2	30617.25	28483.58	27768.55	24605.81	27869	28050.68	28118.88	23703.55	25436.23	26327	1.0585	0.38472
Q9CRD0	Ociad1	OCIA domain-containing protein 1	97379.65	96737.34	101765.7	107475.9	100840	100428.1	106356.3	114263.8	118298.8	109837	0.9181	0.10433
Q9CZ44	Nsfl1c	NSFL1 cofactor p47	103827.9	111794.6	108211.3	113815.7	109412	116153.5	107681.7	123775.9	113867	115370	0.9484	0.18534
P62245	Rps15a	Small ribosomal subunit protein uS8	411314	414666.2	441406.3	451581.7	429742	425523.1	434792.3	485217.9	503215.7	462187	0.9298	0.18003
Q80YA7	Dpp8	Dipeptidyl peptidase 8	23497.3	27548.27	21437.78	21401.87	23471	21446.4	23517.27	22070.02	19861.21	21724	1.0804	0.32516
Q640R3	Hepacam	Hepatic and glial cell adhesion molecule	60394.04	65718.68	68985.44	78558.75	68414	60927.66	75073.22	80502.59	86634.85	75785	0.9027	0.31239
P36916	Gnl1	Guanine nucleotide-binding protein-like 1	30279.66	30850.82	29102.72	25257.41	28873	28237.57	26346.31	27470.19	28158.14	27553	1.0479	0.36033
Q8C1B1	Camsap2	Calmodulin-regulated spectrin-associated protein 2	12324.79	12414.81	11496.21	10562.25	11700	11422.54	12413.11	10587.93	13120.47	11886	0.9843	0.79980
Q03157	Aplp1	Amyloid beta precursor like protein 1		72190.24	5540.223		38865			41492.9		41493	0.9367	
Q9WV02	Rbmx	RNA-binding motif protein, X chromosome	29947.89	17901.9	22453.99	21468.07	22943	25913.01	27393.19	22489.31	21472.13	24317	0.9435	0.65149
Q6TEK5	Vkorc1l1	Vitamin K epoxide reductase complex subunit 1-like	39695.02	39241.2	45590.54	40862.1	41347	38380.77	42996.48	43543.96	47978.85	43225	0.9566	0.47129
P67871	Csnk2b	Casein kinase II subunit beta	33604.85	35517.1	36559.13	37041.05	35681	35698.87	37586.39	36282.72	40922.65	37623	0.9484	0.21321
Q80ZX8	Spag1	Sperm-associated antigen 1												
Q8VDU5	Snrk	SNF-related serine/threonine-protein kinase		4354.692			4355		10312.97	4031.666		7172	0.6072	
P23591	Gfus	GDP-L-fucose synthase	28460.2	25477.23	23921.85	23642.35	25375	27449.92	25281.26	23245.42	24669.05	25161	1.0085	0.88422
P62843	Rps15	Small ribosomal subunit protein uS19	210105.5	204209.4	236730.2	254942	226497	241803.2	203173.9	260863.3	259119.2	241240	0.9389	0.44099
P97384	Anxa11	Annexin A11	79008.66	80268.22	77758.65	76361.36	78349	81839.69	76186.02	75862.44	72558.59	76612	1.0227	0.43967
P20934	Evi2a	Protein EVI2A												
O08810	Eftud2	116 kDa U5 small nuclear ribonucleoprotein compo	438769.4	438922.1	474835.9	480245.8	458193	439819.5	457878	473891.2	506134	469431	0.9761	0.55547

O54983	Crym	Ketimine reductase mu-crystallin	40584.26	37138.54	31322.3	21306.07	32588	30875.3	38921.24	25200.81	21613.46	29153	1.1178	0.56613
Q9QXG2	Chm	Rab proteins geranylgeranyltransferase component /	19741.21	21172.6	17820.59	13709.24	18111	18158.01	19898.29	14393.67	12588.46	16260	1.1139	0.45763
Q80XN0	Bdh1	D-beta-hydroxybutyrate dehydrogenase, mitochondr	65822.89	68201.17	82996.16	79464.23	74121	71609.87	74161.63	93550.39	94701.83	83506	0.8876	0.25464
Q9JHS3	Lamtor2	Ragulator complex protein LAMTOR2	29446.49	26164.61	23423.14	24580.53	25904	25079.91	27350.94	27154.6	29968.44	27388	0.9458	0.40215
O35387	Hax1	HCLS1-associated protein X-1	15798.83	8275.488	18194.35	24319.17	16647	21017.59	15970.52	25928.31	24785.17	21925	0.7593	0.23567
Q91VK4	Itm2c	Integral membrane protein 2C	17571.49	18929.36	19733.69	17063.01	18324	16178.39	19727.47	16303.79	17220.12	17357	1.0557	0.38260
Q61768	Kif5b	Kinesin-1 heavy chain	95633.87	103808.3	99506.05	93506.27	98114	97145.24	96199.58	101450	97073.2	97967	1.0015	0.95614
Q8CI32	Bag5	BAG family molecular chaperone regulator 5	27437.25	28635.43	26245.87	25056.11	26844	26457.59	25736.69	24294.23	24578.52	25267	1.0624	0.13763
Q8VCB3	Gys2	Glycogen [starch] synthase, liver	7723.89	9881.567	8866.724	6268.801	8185	7119.111	8586.872	8611.14	9411.36	8432	0.9707	0.79555
Q80W37	Snupn	Snurportin-1	2300.502	1671.995	3980.439	1388.407	2335	3067.535	2373.858	3541.99	1977.36	2740	0.8523	0.57206
Q3UVK0	Ermp1	Endoplasmic reticulum metallopeptidase 1	54916.09	58514.81	60790.73	63118.95	59335	59866.21	64769.5	63407.23	65534.67	63394	0.9360	0.10817
P97825	Jpt1	Jupiter microtubule associated homolog 1	55973.95	64049.74	68802.19	78308.34	66784	54736.26	62049.25	81016.95	94617.14	73105	0.9135	0.55787
Q6ZPJ0	Tex2	Testis-expressed protein 2	24879.2	18731.67	26286.61	28974.91	24718	25933.58	22887.61	29561.51	26020.79	26101	0.9470	0.60885
Q01768	Nme2	Nucleoside diphosphate kinase B	653550.3	617004.9	659576.4	543056.3	618297	609234	629550.4	498843.3	518765.8	564098	1.0961	0.24514
Q9Z108	Stau1	Double-stranded RNA-binding protein Staufen homo	13959.45	14955.43	17035.26	17305.44	15814	15147.58	15109.27	16917.83	17230.7	16101	0.9821	0.78102
Q9WTX8	Mad1l1	Mitotic spindle assembly checkpoint protein MAD1	13993.61	13995.55	12404.85	15645.06	14010	12421.11	16195.38	3228.728	13052.01	11224	1.2482	0.36885
Q810C0	Slitrk2	SLIT and NTRK-like protein 2			15213.91		15214							
Q6PHN9	Rab35	Ras-related protein Rab-35	68713.44	58323.72	54344.01	62370.43	60938	54015.16	69333.68	60352.38	51217.29	58730	1.0376	0.67747
Q8CHG7	Rapgef2	Rap guanine nucleotide exchange factor 2	15988.02	16684.18	12884.35	17201.26	15689	20065.61	15771.32	13793.06	15355.23	16246	0.9657	0.74794
Q9QYI5	Dnajb2	DnaJ homolog subfamily B member 2	26571.47	25367.05	26036.24	25236.29	25803	28097.47	27801.73	22020.57	24718.5	25660	1.0056	0.92541
O55091	Impact	Protein IMPACT	15558.56	17261.86	19503.98	19480.4	17951	16598.4	15546.61	14932.52	15081.22	15540	1.1552	0.05715
Q8BK75	Elp6	Elongator complex protein 6	10413.85	9366.494	9083.681	12641.02	10376	6769.612	8824.215		10446	8680	1.1954	0.25052
Q3URD3	Slmap	Sarcolemmal membrane-associated protein	38364.09	32840.45	38386.61	50097.8	39922	41581.99	41413.46	41543.59	38833.63	40843	0.9775	0.81151
Q9WUB4	Dctn6	Dynactin subunit 6	56541.97	53355.82	56712.91	56546.08	55789	46465.69	52918.65	70243.36	69611.68	59810	0.9328	0.53065
Q9WUA3	Pfkp	ATP-dependent 6-phosphofructokinase, platelet type	112772.6	109969.4	94301.14	99485.91	104132	111658.9	102045.7	102310.3	97649.37	103416	1.0069	0.89603
Q6GQT5	Tmem151a	Transmembrane protein 151A												
P10922	H1-0	Histone H1.0	1074044	1015652	784726.4	1232853	1026819	1176196	1233945	1034127	1003926	1112048	0.9234	0.46033
Q6ZQA6	Igsf3	Immunoglobulin superfamily member 3	7812.304	6836.137	10329.62	8834.258	8453	7365.614	7596.074	9181.227	9976.721	8530	0.9910	0.93983
Q3TZM9	Alg11	GDP-Man:Man(3)GlcNAc(2)-PP-Dol alpha-1,2-mann	10931.29	11122.84	11763.91	12567.42	11596	11997.23	11904.08	13563.38	12530.44	12499	0.9278	0.13983
Q9JHQ5	Lztfl1	Leucine zipper transcription factor-like protein 1	34605.74	35279.52	33891.98	25703.04	32370	32562.12	32344.59	34207.2	30403.06	32379	0.9997	0.99704
Q8K212	Pacs1	Phosphofurin acidic cluster sorting protein 1	96415.09	85538.08	78847.02	83279.58	86020	111406.4	85230.54	70183.51	99168.39	91497	0.9401	0.59067
P58774	Tpm2	Tropomyosin beta chain	768110.8	730368.6	704636.4	959695.3	790703	598338.8	589074.8	503103.6	606777.4	574324	1.3768	0.01353
Q8CGV2	Tph2	Tryptophan 5-hydroxylase 2	2783.259	7359.094			5071			16319.66		16320	0.3107	
A2ARP1	Ppip5k1	Inositol hexakisphosphate and diphosphoinositol-pentakisphos|		24246.49	18169.02		21208	23123.24				23123	0.9172	
Q8K4Z3	Naxe	NAD(P)H-hydrate epimerase	259718.3	237667.1	223710.1	229025.9	237530	236003.7	230551.1	207090.3	204866.5	219628	1.0815	0.16258
P32261	Serpinc1	Antithrombin-III	69069.48	68596.56	59036.91	52441.12	62286	61649.89	63958.13	51653.87	46967.59	56057	1.1111	0.31594
Q8BR90	Rimoc1	RAB7A-interacting MON1-CCZ1 complex subunit 1	15314.92	19292.29	19166.69	15155.21	17232	19428.81	18050.87	17042.39	10777.25	16325	1.0556	0.69867
Q64524	H2bc21	Histone H2B type 2-E	231457.3	166509.3	169529.1	224457.5	197988	211416.9	214228.7	185986.2	172560.6	196048	1.0099	0.92619
Q8K245	Uvrag	UV radiation resistance-associated protein	9243.738	11379.03	9129.694	6605.562	9090	8288.604	8253.213	8319.301	7384.862	8061	1.1275	0.34452
Q6PGN3	Dclk2	Serine/threonine-protein kinase DCLK2	74766.27	69226.87	61737.41	74491.8	70056	66656.42	74272.55	62144.63	68286.8	67840	1.0327	0.59512
P09405	Ncl	Nucleolin	457617.4	414264.6	446026.8	490921.4	452208	458932.8	430634.3	483625.5	469560.2	460688	0.9816	0.67732
Q8BHW2	Oscp1	Protein OSCP1	5608.434	10113.24	6413.704	4256.076	6598	6183.597	10341.07	3699.072	11535.36	7940	0.8310	0.56600
Q60847	Col12a1	Collagen alpha-1(XII) chain	287608.1	262512.5	220401.2	291573.8	265524	272014.1	280218.8	175060.7	228969	239066	1.1107	0.39888
Q9D1X0	Nol3	Nucleolar protein 3	111568.4	126135.6	113674.3	97612.29	112248	115951.8	114169.8	100576.3	84846.76	103886	1.0805	0.40249
P62259	Ywhae	14-3-3 protein epsilon	538753.6	550682.4	547211.1	501416.9	534516	526000.5	521792.8	544554.1	507833.3	525045	1.0180	0.51269
Q9Z2E9	Bscl2	Seipin	26953.73	27999.75	28143.58	30648.35	28436	27044.12	30505.69	23522.99	29571.53	27661	1.0280	0.67272
Q9EPK2	Rp2	Protein XRP2	33933.07	31612.44	16778.95	42441.59	31192	30961.12	19590.16	25429.56	16538.08	23130	1.3485	0.24270
Q7TNC9	Inpp5a	Inositol polyphosphate-5-phosphatase A	13861.17	14240.29	11706.03	10186.95	12499	15321.77	11752.56	10766.94	10988.49	12207	1.0239	0.84473
O54786	Dffa	DNA fragmentation factor subunit alpha	67595.16	69601.31	52460.07	48610.84	59567	64328.14	58100.15	44690.34	42362.81	52370	1.1374	0.37290
O35887	Calu	Calumenin	167898.9	187562	213009.8	194556.1	190757	165363.8	170597.6	188524.3	198935	180855	1.0547	0.44645
Q6IRU2	Tpm4	Tropomyosin alpha-4 chain	119336.2	124596.4	124120.4	115579.8	120908	130174.5	107825.2	124835.4	120252.4	120772	1.0011	0.98003
O35250	Exoc7	Exocyst complex component 7	30053.02	29143.38	29879	31836.29	30228	29966.11	30228.01	30227.15	31751.8	30543	0.9897	0.66892
Q9JLR9	Higd1a	HIG1 domain family member 1A, mitochondrial	31528.61	34325.49	37903.72	15775.19	29883	37243.6	29093.78	25722.12	51500.06	35890	0.8326	0.45560
P46412	Gpx3	Glutathione peroxidase 3	100473.6	105774.1	59624.62	88045.78	88480	103640.4	102602.1	50012.69	48492.6	76187	1.1613	0.53454
Q60771	Cldn11	Claudin-11	6524.866	11411.16	8741.46	11839.62	9629	7304.153	13416.4	6376.409	9904.563	9250	1.0410	0.85646
O35474	Edil3	EGF-like repeat and discoidin I-like domain-containi	17099.87	20940.51	20463.69	20313.16	19704	23225.75	21171.96	17184.5	20061.19	20411	0.9654	0.66162
Q8BFR6	Zfand1	AN1-type zinc finger protein 1												
Q920M5	Coro6	Coronin-6	26373.47	24952.08	21044.36	28393.03	25191	33377.8	22913.85	21272.86	23414.62	25245	0.9979	0.98690
P42208	Septin2	Septin-2	359115.6	384817	364640.7	345308	363470	342990.1	341643.6	339423.5	352572.8	344157	1.0561	0.06804

Q5U458	Dnajc11	DnaJ homolog subfamily C member 11	47166.7	46785.29	50040.9	58026.95	**50505**	49584.45	48456.43	51470.37	59051.2	**52141**	**0.9686**	**0.66002**
Q8BHL8	Psmf1	Proteasome inhibitor PI31 subunit	78738.75	82160.18	79518.22	62759.93	**75794**	73515.57	68032.43	65385.75	64671.16	**67901**	**1.1162**	**0.15415**
Q8K2C6	Sirt5	NAD-dependent protein deacylase sirtuin-5, mitocho	8843.99	8237.866	8198.471	8334.74	**8404**	9701.303	8786.345	5724.04	4523.125	**7184**	**1.1698**	**0.36234**
Q9D5R2	Wdr20	WD repeat-containing protein 20	4659.76	10306.14	13776.67	10027.58	**9693**	5385.811	3687.839	11524.51	12565.21	**8291**	**1.1691**	**0.64587**
Q99L43	Cds2	Phosphatidate cytidylyltransferase 2	212635.2	220468.9	185363.9	218701.3	**209292**	219907.5	226527.1	214761.2	217127.8	**219581**	**0.9531**	**0.27356**
Q76MZ3	Ppp2r1a	Serine/threonine-protein phosphatase 2A 65 kDa reg	458511.6	490152.4	470203.4	427546.4	**461603**	480799.7	481901.4	461651.3	459077.8	**470858**	**0.9803**	**0.54534**
Q99N96	Mrpl1	Large ribosomal subunit protein uL1m	14888.06	12448.54	17254.45	7641.546	**13058**	11747.64	12550.04	3289.004	12846.57	**10108**	**1.2918**	**0.37416**
Q61474	Msi1	RNA-binding protein Musashi homolog 1												
Q9D8B3	Chmp4b	Charged multivesicular body protein 4b	74872.36	76296.85	76604.67	65800.27	**73394**	77274.72	65954.7	75551.99	70390.71	**72293**	**1.0152**	**0.77178**
P23819	Gria2	Glutamate receptor 2	1711.275	3946.621	6361.927		**4007**		5435.772	1588.75	3120.117	**3382**	**1.1848**	**0.73865**
Q91W61	Fbxl15	F-box/LRR-repeat protein 15	45393.9	18057.69	24547.28	15263.07	**25815**	13237.45	19083.35	13218.26	10978.85	**14129**	**1.8271**	**0.14737**
Q6VNB8	Wdfy3	WD repeat and FYVE domain-containing protein 3	12649.78	12338.4	10822.97	9251.893	**11266**	10134.56	9715.142	9062.042	10110.66	**9756**	**1.1548**	**0.11514**
O09061	Psmb1	Proteasome subunit beta type-1	149483.2	136568.7	127306.2	131315.5	**136168**	132256.2	131824.5	134911	132517.3	**132877**	**1.0248**	**0.52483**
Q9CWL8	Ctnnbl1	Beta-catenin-like protein 1	34326.96	33398.49	39295.22	36546.15	**35892**	37430.79	33539.61	36537.77	41119.07	**37157**	**0.9660**	**0.55788**
Q9JME5	Ap3b2	AP-3 complex subunit beta-2	96996.56	98522.51	93140.29	91320.72	**94995**	105470.1	99291.82	97503.81	105334.8	**101900**	**0.9322**	**0.04018**
Q9JKC8	Ap3m1	AP-3 complex subunit mu-1	85484.14	84594.21	84650.85	74671.09	**82350**	92103.74	81741.53	89865.28	96186.87	**89974**	**0.9153**	**0.10386**
Q9Z0L0	Tpbg	Trophoblast glycoprotein												
Q6ZPE2	Sbf1	Myotubularin-related protein 5	27150.9	30326.11	31078.29	32554.87	**30278**	27523.52	27154.04	27165.05	28795.84	**27660**	**1.0946**	**0.07275**
Q9QZ88	Vps29	Vacuolar protein sorting-associated protein 29	95734.09	94503.41	95825.23	90744.02	**94202**	95979.01	95613.65	94463.71	106075.1	**98033**	**0.9609**	**0.24190**
Q761V0	Slc6a5	Sodium- and chloride-dependent glycine transporter	21059.09		41273.63	24064.13	**28799**		25485.53			**25486**	**1.1300**	
Q9D428	GOLGA7B	Golgin subfamily A member 7B	28207.46	36355.27	22208.23	35543.63	**30579**	32839.29	26154.17		25693.76	**28229**	**1.0832**	**0.61597**
Q8K310	Matr3	Matrin-3	123025	121387.5	137225.3	138907.4	**130136**	132830.5	137056.8	148055.2	146127.9	**141018**	**0.9228**	**0.11291**
Q69ZF3	Gba2	Non-lysosomal glucosylceramidase	5924.895	7003.99	7788.925	7779.927	**7124**	7801.035	7138.526	7683.23	8757.404	**7845**	**0.9081**	**0.24114**
Q3TFQ1	Spryd7	SPRY domain-containing protein 7	22781.21	33112.89	25981.27	24739.52	**26654**	30718.36	28046.93	22373.82	29275.77	**27604**	**0.9656**	**0.75431**
Q923T9	Camk2g	Calcium/calmodulin-dependent protein kinase type	50750.01	50261.46	50215.83	57627.2	**52214**	45552.91	57731.64	48635.65	46122.98	**49511**	**1.0546**	**0.45068**
Q8VDL4	Adpgk	ADP-dependent glucokinase	54912.64	55832.89	64557.89	62388.95	**59423**	53019.13	54536.43	57197.87	56127.64	**55220**	**1.0761**	**0.15132**
P35288	Rab23	Ras-related protein Rab-23	22921.59	21553.5	19974.71	21841.3	**21573**	19818.61	28964.17	19608.27	19251.31	**21911**	**0.9846**	**0.89405**
Q9D7N9	Apmap	Adipocyte plasma membrane-associated protein	96245.13	97765.82	97229.5	113953.4	**101298**	100652.1	107659.4	107287.8	110910.1	**106627**	**0.9500**	**0.30440**
Q9JLQ0	Cd2ap	CD2-associated protein	42669	43045.03	37347.55	41445.34	**41127**	40337.32	45017.4	42739.6	41535.8	**42408**	**0.9698**	**0.46539**
G3X8U3	QNG1	Queuosine 5'-phosphate N-glycosylase/hydrolase	35833.46	38503.77	35342.52	27709.51	**34347**	30255.96	29552.8	24124.87	27392.57	**27832**	**1.2341**	**0.05217**
Q6PFX8	Rimkla	N-acetylaspartylglutamate synthase A	139669.8	153019.4	118692.4	125445	**134207**	147328.2	140973.3	129695.8	128717.6	**136679**	**0.9819**	**0.78995**
P03995	Gfap	Glial fibrillary acidic protein	71075.86	94179.82	100026.9	136949.3	**100558**	107851	136498.8	85875.98	90991.22	**105304**	**0.9549**	**0.79852**
Q9JHU4	Dync1h1	Cytoplasmic dynein 1 heavy chain 1	737372.1	766518.7	764519.3	769773.3	**759546**	754012.3	787776.3	778494.3	768107.4	**772098**	**0.9837**	**0.27313**
Q80W04	Tmcc2	Transmembrane and coiled-coil domains protein 2												
Q9QZQ1	Afdn	Afadin	12543.92	11509.44	11760.2	11508.4	**11830**	10998.45	9948.193	8799.843	10951.55	**10175**	**1.1628**	**0.02775**
O55029	Copb2	Coatomer subunit beta'	209191.5	203837.5	214234.1	205655.9	**208230**	199390	216084.6	215504	224262.2	**213810**	**0.9739**	**0.36437**
P48428	Tbca	Tubulin-specific chaperone A	64386.68	69744.42	60431.99	57772.89	**63084**	59168.17	60120.37	60584.9	74642.57	**63629**	**0.9914**	**0.90776**
Q8K274	Fn3krp	Ketosamine-3-kinase	33437.12	30191.3	29163.62	28597.15	**30347**	32326.98	28382.31	20760.34	28319.49	**27447**	**1.1057**	**0.31559**
O35678	Mgll	Monoglyceride lipase	40405.14	43885.04	67270.03	47247.32	**49702**	45964.85	44124.85	67455.57	75417.02	**58241**	**0.8534**	**0.41943**
Q8CIH9	Ppat	Amidophosphoribosyltransferase	29391.25	26020.68	27870.32	27024.14	**27577**	30351.65	26888.64	17616.97	23160	**24504**	**1.1254**	**0.31728**
Q60805	Mertk	Tyrosine-protein kinase Mer	11290.73	15756.32	12733.7	9715.305	**12374**	14725.02	14210.07	1526.803	9095.636	**9889**	**1.2512**	**0.48277**
Q62443	Nptx1	Neuronal pentraxin-1	14060.27	12110.17	14467.27	14915.06	**13888**	12256.89	15955.96	6083.82	7303.458	**10400**	**1.3354**	**0.19067**
Q8C1A5	Thop1	Thimet oligopeptidase	79184.77	84843.8	73274.2	58245.06	**73887**	76185.96	71563.63	75449.6	64123.6	**71831**	**1.0286**	**0.75726**
O70492	Snx3	Sorting nexin-3	166514.3	154628.6	174112.8	148582.8	**160960**	179032.3	164932.3	179921.4	187264.6	**177788**	**0.9053**	**0.06349**
P60710	Actb	Actin, cytoplasmic 1	6617027	6520792	6368234	6281988	**6447010**	6313191	5961140	5950627	6184801	**6102440**	**1.0565**	**0.02510**
Q9CQA9	Ntpcr	Cancer-related nucleoside-triphosphatase homolog	23231.04	22768.92	24891.39	18645.11	**22384**	21512.38	22191.19	17724.33	22550.63	**20995**	**1.0662**	**0.45271**
Q80Y81	Elac2	Zinc phosphodiesterase ELAC protein 2	10663.89	11551.46	15256.23	13422.67	**12724**	11641.42	8884.888	12386.87	13658.77	**11643**	**1.0928**	**0.48021**
P60670	Nploc4	Nuclear protein localization protein 4 homolog	37575.51	37474.02	39513.75	38824.74	**38347**	39060.86	38009.09	36375.52	34949.13	**37099**	**1.0336**	**0.27172**
Q8K2Q0	Commd9	COMM domain-containing protein 9	19267.57	20693.43	21174.66	21524.04	**20665**	18209.02	20788.9	21397.99	18711.42	**19777**	**1.0449**	**0.37254**
Q8BPQ7	Sgsm1	Small G protein signaling modulator 1	14876.83	11543.19	15801.36	13058.45	**13820**	15643.29	15769.92	14110.91	8955.628	**13620**	**1.0147**	**0.91787**
Q9CR27	Washc3	WASH complex subunit 3	45241.91	46614.95	49197.13	45146.47	**46550**	46655.84	47208.21	50550.32	55368.79	**49946**	**0.9320**	**0.17588**
Q5SSH7	Zzef1	Zinc finger ZZ-type and EF-hand domain-containing p	34563.96	30185.15	30361.15	31907.18	**31754**	31843.53	33064.83	29483.1	34092.47	**32121**	**0.9886**	**0.80464**
Q8BGW1	Fto	Alpha-ketoglutarate-dependent dioxygenase FTO	7579.078	6758.793	9170.397	4980.465	**7122**	7170.333	6535.604	6691.669	5774.844	**6543**	**1.0885**	**0.55173**
A2TJV2	Palm3	Paralemmin-3			9075.553	14243.64	**11660**			2648.62	7039.373	**4844**	**2.4070**	**0.18214**
Q6NXK8	Asic1	Acid-sensing ion channel 1												
Q9EQU5	Set	Protein SET	50097.44	60416.59	56777.4	56382.43	**55918**	58433.3	52425.69	62558.42	52176.07	**56398**	**0.9915**	**0.88917**
O35379	Abcc1	Multidrug resistance-associated protein 1	16167.15	12838.08	11822.68	14054.3	**13721**	12386.5	17181.13	11490.67	12888.97	**13487**	**1.0173**	**0.88672**
Q922V4	Plrg1	Pleiotropic regulator 1	32274.52	32484.02	35726.73	36399.27	**34221**	34138.93	35448.48	37908.66	41066.37	**37141**	**0.9214**	**0.16833**
Q3TY60	Fam131b	Protein FAM131B												

Q923Z0	Gprc5b	G-protein coupled receptor family C group 5 membe	18329.49	14057.72	20020.37	21289.65	18424	18265.96	18205.4	17914.93	16764.15	17788	1.0358	0.70704
Q8C163	Exog	Nuclease EXOG, mitochondrial	21277.61	23406.74	24093.62	26415.61	23798	20445.79	26058.15	22780.39	22953.55	23059	1.0320	0.65332
O88845	Akap10	A-kinase anchor protein 10, mitochondrial		9019.811	10200.67		9610	12158.53	12127.87	12467.93	11677.64	12108	0.7937	0.00461
Q9Z2H2	Rgs6	Regulator of G-protein signaling 6	19028.39	20008.99	20671.6	23876.6	20896	26555.3	20440.58	18588.12	21718.79	21826	0.9574	0.65853
Q91VR8	Brk1	Protein BRICK1	26461.57				26462				17624.74	17625	1.5014	
P14211	Calr	Calreticulin	885640.7	897829.1	1066408	965050.1	953732	894486.8	949833.6	968359.6	1013030	956428	0.9972	0.95716
Q8CHU3	Epn2	Epsin-2	35091.75	39677.51	42364.79	43502.71	40159	38194.3	36772.18	40229.5	41908.03	39276	1.0225	0.69993
Q8BI08	Mal2	Protein MAL2	42874.46	41464.75	38606.44	60136.59	45771	45883.98	49146.61	47877.19	50844.99	48438	0.9449	0.61155
Q8BMP6	Acbd3	Golgi resident protein GCP60	44872.65	38985.91	38734.63	46543.33	42284	40440.58	42518.66	43756.76	41455.37	42043	1.0057	0.91347
Q6NXK7	Dpp10	Inactive dipeptidyl peptidase 10	17052.95	10180.7	10504.68	9746.462	11871	9503.535		11356.91	11833.1	10898	1.0893	0.66777
Q9WU78	Pdcd6ip	Programmed cell death 6-interacting protein	125679.6	131926.1	115563.5	111595.8	121191	118709.4	120388.9	113340.7	106073.5	114628	1.0573	0.28993
Q8VBV7	Cops8	COP9 signalosome complex subunit 8	103460.4	111240.7	98603.06	101555.8	103715	103245.4	113074.6	88639.69	95910.28	100217	1.0349	0.57355
P31750	Akt1	RAC-alpha serine/threonine-protein kinase	19561.11	20866.47	17428.53	12143.98	17500	16818.47	17063.26	14295.48	15078.4	15814	1.1066	0.43904
P70302	Stim1	Stromal interaction molecule 1	26185.4	25496.11	28396.08	32208.58	28072	26788.26	25229.97	23383.01	28624.74	26006	1.0794	0.31387
Q5SS80	Dhrs13	Dehydrogenase/reductase SDR family member 13	8443.915	8027.7	10466.45	8966.445	8976	7601.428	13238.55	8759.364	3625.356	8306	1.0807	0.75471
P55066	Ncan	Neurocan core protein						30444.9	227122.1			128783	0.0000	
P62077	Timm8b	Mitochondrial import inner membrane translocase subunit Tim8 B			11151.91	11312.34	11232	10870.5	12043.04	15295.96	13043.45	12813	0.8766	0.32478
Q9Z0G0	Gipc1	PDZ domain-containing protein GIPC1	20389.93	18835.62	17611.1	14871.58	17927	16771.79	14614.98	13984.24	13656.7	14757	1.2148	0.05864
O35345	Kpna6	Importin subunit alpha-7	56263.77	57669.55	56327.38	54357.96	56155	54888.35	51958.79	49469.99	52240.19	52139	1.0770	0.02144
Q80V42	Cpm	Carboxypeptidase M								28920.2		28920	0.0000	
Q8BKC5	Ipo5	Importin-5	90613.6	102760.9	91006.73	78674.43	90764	89933.38	100110.3	83144.76	80443.91	88408	1.0266	0.73283
Q61923	Kcna6	Potassium voltage-gated channel subfamily A memb	7897.682	8814.831	6007.807	7369.898	7523	7413.571	9002.288	3762.377	6885.838	6766	1.1118	0.56553
P62309	Snrpg	Small nuclear ribonucleoprotein G	82721.71	62773	81870.99	84672.37	78010	70185.91	78626.15	79257.18	95492.18	80890	0.9644	0.70885
P17879	Hspa1b	Heat shock 70 kDa protein 1B	349564	419370.5	473953.5	436581.1	419867	430329	385914.8	505789.1	472522.3	448639	0.9359	0.46407
Q61730	Il1rap	Interleukin-1 receptor accessory protein	27143.29	26157.61	24669.32	24869.49	25710	15691.8	22442.59	22751.83	30149.23	22759	1.1297	0.36471
P62484	Abi2	Abl interactor 2	34250.43	31545.71	36763.48	37295.13	34964	34388.52	35490.54	38898.66	40719.61	37374	0.9355	0.26820
P24472	Gsta4	Glutathione S-transferase A4	104988.6	98940.2	81383.4	54687.32	85000	75847.66	88198.7	76797.56	47521.84	72091	1.1791	0.39887
Q62241	Snrpc	U1 small nuclear ribonucleoprotein C	22104.16	18978.62	22062.02	22176.53	21330	19848.46	23143.22	24720.82	20667.45	22095	0.9654	0.59644
Q8VCT3	Rnpep	Aminopeptidase B	28318.92	34629.91	36219.54	23710.57	30720	33942.46	29952.93	29144.33	29100.97	30535	1.0060	0.95466
Q61543	Glg1	Golgi apparatus protein 1	79277.23	79521.08	82786.02	86141.96	81932	81057.24	87143.43	82689.29	91737.27	85657	0.9565	0.24534
Q3TY86	Aifm3	Apoptosis-inducing factor 3			727.0399		727				52841.1	52841	0.0138	
P70188	Kifap3	Kinesin-associated protein 3	29100.57	32826.3	33759.01	25327.31	30253	33340.13	35004.5	38115.84	30911.59	34343	0.8809	0.14593
Q60967	Papss1	Bifunctional 3'-phosphoadenosine 5'-phosphosulfat	20594.48	21392.21	31944.46	14851.72	22196	19015.29	16314	14053.66	23930.24	18328	1.2110	0.38702
Q924S8	Spred1	Sprouty-related, EVH1 domain-containing protein 1						15208.32		37787.16		26498	0.0000	
P11859	Agt	Angiotensinogen	5917.954	7349.733	7216.78		6828	6391.201	5804.014		7426.746	6541	1.0440	0.68493
Q8CHY3	Dym	Dymeclin	38683.41	35495.32	29557.86	31000.56	33684	41855.72	42505.01	29767.31	28897.31	35756	0.9421	0.64423
Q9QYF1	Rdh11	Retinol dehydrogenase 11	46862.08	49898.64	66198.26	57499.61	55115	50862.59	48180.77	62458.71	72267.93	58443	0.9431	0.65290
Q8K354	Cbr3	Carbonyl reductase [NADPH] 3	195891.5	214393.9	186044.3	191358.4	196922	200094.5	204310.6	131243.8	139857.3	168877	1.1661	0.21631
Q9DAR7	Dcps	m7GpppX diphosphatase	43725.42	46319.5	49476.29	43239.83	45690	53123.7	43612.46	54728.44	42853.61	48580	0.9405	0.43084
P62069	Usp46	Ubiquitin carboxyl-terminal hydrolase 46	43088.2	40028.32	30927.59	30772.44	36204	43419.05	22631.78	24234.7	23571.41	28464	1.2719	0.23809
P35802	Gpm6a	Neuronal membrane glycoprotein M6-a	34288.18	31978.13	28947.79	36744.07	32990	36767.17	43139.32	52657.3	30718.21	40821	0.8082	0.16660
P62892	Rpl39	Large ribosomal subunit protein eL39	70392.66	73418.24	74520.7	85081.85	75853	72034.15	76487.57	82146.12	93547.98	81054	0.9358	0.39233
Q8VEH5	Epm2aip1	EPM2A-interacting protein 1	80532.38	80698.3	69797.72	63286.77	73579	77877.12	77809.01	76311.91	73652.09	76413	0.9629	0.54225
Q9CQV8	Ywhab	14-3-3 protein beta/alpha	1221161	1359241	1300183	1137967	1254638	1249126	1199674	1103311	1158687	1177700	1.0653	0.22704
Q9JLZ3	Auh	Methylglutaconyl-CoA hydratase, mitochondrial	285207.8	249768.8	251509.8	286290.1	268194	275382.9	272361	284223.1	301195.2	283291	0.9467	0.25637
Q9QZ08	Nagk	N-acetyl-D-glucosamine kinase	42198.57	38576.91	25657.34	32503.75	34734	37396.04	37475.94	32318.91	32750.35	34985	0.9928	0.95067
Q9CQS8	Sec61b	Protein transport protein Sec61 subunit beta	34701.13	54504.99	55221.07	77607.31	55509	43488.69	55025.73	49268.07	54983.09	50691	1.0950	0.61895
Q6P5E4	Uggt1	UDP-glucose:glycoprotein glucosyltransferase 1	87952.75	82760.38	87248.67	91160.61	87281	90270.27	89770.23	96969.93	95893.51	93226	0.9362	0.05818
Q9JHZ2	Ankh	Progressive ankylosis protein	6207.688	6173.952	7050.424	6184.065	6404	6128.735	7115.222	7307.682	9145.667	7424	0.8626	0.17592
Q9JJV5	Cacng3	Voltage-dependent calcium channel gamma-3 subunit												
P70236	Map2k6	Dual specificity mitogen-activated protein kinase kin	28743.65	27928.07	34660.91	26003.45	29334	29469.85	29389.96	27370.46	26928.33	28290	1.0369	0.61699
Q9JJZ4	Ube2j1	Ubiquitin-conjugating enzyme E2 J1	57629.8	53617.33	48734.06	54423.06	53601	48869.6	58927.48	38203.21	48683.7	48671	1.1013	0.32633
Q3UHE1	Pitpnm3	Membrane-associated phosphatidylinositol transfer protein 3												
Q811P8	Arhgap32	Rho GTPase-activating protein 32		9677.975	6132.85	5226.301	7012	4552.886	5832.955			5193	1.3504	0.39050
P53612	Rabggtb	Geranylgeranyl transferase type-2 subunit beta	8694.492	9463.431	6546.273	5760.004	7616	8563.656	8592.461	6126.943	5539.057	7206	1.0570	0.74101
Q62422	Ostf1	Osteoclast-stimulating factor 1	255124.7	246136.8	233844.3	192157	231816	230676.6	229931.7	263161.8	237049.1	240205	0.9651	0.61811
E0CYV9		Uncharacterized protein C4orf54 homolog	41304.29	44988.56	40657.21	46854.07	43451	45410.38	45123.24	51331.34	45165.12	46758	0.9293	0.17109
Q99NH0	Ankrd17	Ankyrin repeat domain-containing protein 17	18819.85	16426.46	18916.47	16088.31	17563	18661	15621.11	22266.56	18394.56	18736	0.9374	0.48027
Q9QUJ7	Acsl4	Long-chain-fatty-acid--CoA ligase 4	76024.7	80227.41	92849.31	96552.58	86414	80292.66	86251.79	91940.93	94480.59	88241	0.9793	0.76512

Q7TMS5	Abcg2	Broad substrate specificity ATP-binding cassette trar	49430.66	51058.96	41526.2	58217.13	50058	47754.35	52406.04	48728.55	49256.14	49536	1.0105	0.88853
Q8R527	Rhoq	Rho-related GTP-binding protein RhoQ												
Q3UGY8	Arfgef3	Brefeldin A-inhibited guanine nucleotide-exchange p	35393.82	34924.61	36333.16	33590.48	35061	33555.2	33905.56	34290.01	35237.16	34247	1.0238	0.27428
Q8BGH2	Samm50	Sorting and assembly machinery component 50 hom	164459.9	164076.7	169715.4	199536.8	174447	168829.4	173821.9	173099.5	174935	172671	1.0103	0.84263
Q9D7V2	Lysmd2	LysM and putative peptidoglycan-binding domain-co	2563.006	5335.682		1837.89	3246	2253.618				2254	1.4401	
Q9QUP5	Hapln1	Hyaluro and proteoglycan link protein 1	11706.52	17299.55			14503	17769.02	13123.47	44439.45	20378	23927	0.6061	0.42573
P35564	Canx	Calnexin	655766.3	684374.6	732525.7	780971.6	713410	722315.4	670976	787865.3	790602.3	742940	0.9603	0.48592
P21550	Eno3	Beta-enolase	73639.88	86162.09	59445.93	42404.61	65413	49135.94	59635.76	29701.27	52319.48	47698	1.3714	0.17043
O70311	Nmt2	Glycylpeptide N-tetradecanoyltransferase 2	25222.3	26575.85	27905.35	25936.66	26410	20783.44	28919.49	20782.36	24224.92	23678	1.1154	0.22268
Q7TPM6	Fsd1	Fibronectin type III and SPRY domain-containing prot	42655.83	44597.47	34127.16	32545.75	38482	38697.71	36990.93	29483.34	34861.79	35008	1.0992	0.37420
Q9QYS9	Qki	KH domain-containing RNA-binding protein QKI	51165.05	59267.88	75736.95	51860.24	59508	54591.49	51990.29	83593.16	79041.15	67304	0.8842	0.46358
O70250	Pgam2	Phosphoglycerate mutase 2	212908.5	242463	224272.5	177066	214178	219913.9	180470.7	173215.1	166361	184990	1.1578	0.16131
Q9Z0U1	Tjp2	Tight junction protein ZO-2	102010.3	97765.63	89797.36	108252.3	99456	94587.71	100144.5	87179.41	95596.98	94377	1.0538	0.32266
Q9ERE7	Mesd	LRP chaperone MESD	31992.47	31672.2	32486.93	35058.11	32802	35163.14	35039.92	37149.67	31276.08	34657	0.9465	0.24756
Q6PDH0	Phldb1	Pleckstrin homology-like domain family B member 1	39590.33	39729.27	50378.44	42484.89	43046	43593.81	26423.32	51456.14	57632.55	44776	0.9613	0.81848
Q8BVU5	Nudt9	ADP-ribose pyrophosphatase, mitochondrial	26647.79	20906.63	23940.48	22098.87	23398	19385.73	25198.99	25359.65	20920.38	22716	1.0300	0.73998
Q64520	Guk1	Guanylate kinase	40582.22	49818.9	37324.71	40675.41	42100	44429.13	44682.75	39006.59	40022.42	42035	1.0015	0.98374
P05977	Myl1	Myosin light chain 1/3, skeletal muscle isoform	157824.9	111775.5	103667.8	204149.3	144354	64399.03	92226.81	26300.97	51184.21	58528	2.4664	0.01905
Q8BN59	Larp6	La-related protein 6	15338.37	12387.61	10094.87	10715.2	12134	9639.482	14584.69	9199.688	10898.26	11081	1.0951	0.55686
Q8R2R9	Ap3m2	AP-3 complex subunit mu-2	23218.3	29424.76	25782.63	26954.6	26345	25281.88	23127.82	27210.49	29976.27	26399	0.9980	0.97872
P46096	Syt1	Synaptotagmin-1	18874.51	21044.82	18527.51	20075.22	19631	17653.88	17451.11	13819.37	17720.31	16661	1.1782	0.03681
Q8CCS6	Pabpn1	Polyadenylate-binding protein 2	52687.68	51973.79	56608.4	61850.3	55780	53488.11	56998.84	63287.23	60952.23	58682	0.9506	0.39005
Q61249	Igbp1	Immunoglobulin-binding protein 1	16513.7	16135.87	16478.28	14114.57	15811	14086.08	14635.99	13610.96	12368.49	13675	1.1561	0.02910
P62996	Tra2b	Transformer-2 protein homolog beta	61189.46	60769.58	60990.25	76227.3	64794	68545.34	69488.34	73790.23	77351.34	72294	0.8963	0.13336
Q9D3A9	Ttyh1	Protein tweety homolog 1	22182.17	21571.5	17684.01	21415.65	20713	18233.36	22728.11	15637.43	15744.65	18086	1.1453	0.22642
Q3UMB9	Washc4	WASH complex subunit 4	26353.42	26693.48	25528.16	22289.81	25216	25132.34	25457.36	23826.54	26012.63	25107	1.0043	0.92481
Q91VI7	Rnh1	Ribonuclease inhibitor	84300.38	88114.66	85888.55	71922.96	82557	78317.34	81931.77	76891.98	69702.31	76711	1.0762	0.23649
B9EJI9	Tmem229a	Transmembrane protein 229A	6003.97	5676.687	6756.98	8295.318	6683	4431.33	7922.036	3440.01	8676.77	6118	1.0925	0.70240
Q91X56	S1pr5	Sphingosine 1-phosphate receptor 5												
Q6PAL8	Dennd5a	DENN domain-containing protein 5A												
P61255	Rpl26	Large ribosomal subunit protein uL24	537951.2	493765	599388.6	592970.1	556019	552550.5	550202.8	648169.1	681665.5	608147	0.9143	0.25819
P83882	Rpl36a	Large ribosomal subunit protein eL42	91864.62	100135.5	95575.04	106336.8	98478	91184.88	106601.2	107938.2	114860.8	105146	0.9366	0.30056
Q9JLI6	Scly	Selenocysteine lyase	9589.546	10505.17	10805.29	6634.313	9384	10523.41	8967.021	10300.8	9563.726	9839	0.9537	0.67001
Q91VU0	Fam3c	Protein FAM3C	27761.5	25371.46	27134.44	26517.46	26696	29466.05	28276.34	26911.82	32576.7	29308	0.9109	0.09349
P41731	Cd63	CD63 antigen	26010.32	29046.87	24929.04	25132.28	26280	27041.88	32309.97	24093.6	27025.14	27618	0.9516	0.51982
Q62167	Ddx3x	ATP-dependent RNA helicase DDX3X	209795.2	205144.5	201272.3	208687.4	206225	213158.8	225132.1	224920.5	228390.7	222901	0.9252	0.00497
Q80XA6	Reps2	RalBP1-associated Eps domain-containing protein 2	30126.02	33176.43	29100.59	32901.99	31326	31409.04	36394.37	25021.18	30349.49	30794	1.0173	0.84096
Q6P9J5	Kank4	KN motif and ankyrin repeat domain-containing prot	54142.75	55639.46	61095	55851.27	56682	51155.47	51035.66	63843.63	63852.43	57472	0.9863	0.84937
Q922E4	Pcyt2	Ethanolamine-phosphate cytidylyltransferase	49875.14	56930.38	67232.27	47460.11	55374	54474.05	46964.01	52235.46	57635.98	52827	1.0482	0.62665
P62812	Gabra1	Gamma-aminobutyric acid receptor subunit alpha-1												
Q9WV27	Atp1a4	Sodium/potassium-transporting ATPase subunit alpl	235070.6	242738.4	245422.4	280261.5	250873	171096	255003.2	185002.9	208058.8	204790	1.2250	0.07016
Q07813	Bax	Apoptosis regulator BAX	22304.08	20458.83	19424.3	15575.95	19441	19268.84	18669.47	19373.44	14877.86	18047	1.0772	0.46258
Q99PJ0	Ntm	Neurotrimin		8533.811	9710.195		9122	11470.98				11471	0.7952	
Q8R4H2	Arhgef12	Rho guanine nucleotide exchange factor 12	30787.96	31909.9	32535.3	30887.27	31530	29271.59	30312.57	32613.3	33206.98	31351	1.0057	0.86686
P47915	Rpl29	Large ribosomal subunit protein eL29	82737.07	79374.27	91155.66	123221.4	94122	72521.16	84526.77	133314.6	133675.3	106009	0.8879	0.55301
Q9D1E6	Tbcb	Tubulin-folding cofactor B	87444.71	92037.58	86049.63	73496.22	84757	89289.66	87204.88	75005.58	68343.06	79961	1.0600	0.48033
Q9Z160	Cog1	Conserved oligomeric Golgi complex subunit 1	32160.85	33449.2	35784.84	34702.48	34024	36434.63	31396.07	40526.23	39376.45	36933	0.9212	0.23092
Q8R4E6	Purg	Purine-rich element-binding protein gamma	111987.7	100867	115762.3	113385.9	110501	109880.2	113680	135102.3	130553.2	122304	0.9035	0.14375
S4R2P9	Slc8a3	Sodium/calcium exchanger 3												
P03893	mt-Nd2	NADH-ubiquinone oxidoreductase chain 2	18891.46	13834.33	11488.65	10076.56	13573	13540.37	24150.42	2754.693	9275.302	12430	1.0919	0.82307
P50429	Arsb	Arylsulfatase B	35329.46	27327.87	30375.93	24659.44	29423	38340.58	26997.05	31198.58	31794.48	32083	0.9171	0.44786
Q9Z0V7	Timm17b	Mitochondrial import inner membrane translocase s	29097.38	31950.88	31242.54	33806.89	31524	30806.59	30707.55	24517.59	28493.46	28631	1.1010	0.15212
Q8BLJ3	Plcxd3	PI-PLC X domain-containing protein 3	20510.13	18174.97	21868.25	11052.86	17902	20836.51	14324.76	24861.3	22494.12	20629	0.8678	0.44014
Q8JZK9	Hmgcs1	Hydroxymethylglutaryl-CoA synthase, cytoplasmic	101712.6	107553.7	157399.2	92882.61	114887	112230.9	97798.12	151372.7	150373.6	127944	0.8979	0.53501
Q91WC3	Acsl6	Long-chain-fatty-acid--CoA ligase 6	78112.91	74265.43	75480.55	80286	77036	72734.45	85418.1	73100.63	80616.87	77968	0.9881	0.79091
Q8BH44	Coro2b	Coronin-2B	36462.97	35699.29	32811.38	38758.09	35933	34788.29	35931.02	33332.41	36179.97	35058	1.0250	0.55178
P10852	Slc3a2	Amino acid transporter heavy chain SLC3A2	188701.7	206882.5	156555.8	218662.3	192701	191784.2	203500	154023.8	189073.3	184595	1.0439	0.65462
Q64518	Atp2a3	Sarcoplasmic/endoplasmic reticulum calcium ATPas	292825.3	276371.2	293234.9	333564.1	298999	277148.6	268819.9	267404.5	317147.9	282630	1.0579	0.36984
Q9JLJ5	Elovl1	Elongation of very long chain fatty acids protein 1	30916.49	30987.5	44346.68	35684.21	35484	26110.77	29374.7	30995.84	56226.44	35677	0.9946	0.98057

O55042	Snca	Alpha-synuclein	50024.32	66546.14	66508.21	54949.38	59507	62752.51	53352.48	78158.46	72369.36	66658	0.8927	0.33805
Q3V038	Ttc9	Tetratricopeptide repeat protein 9A	61832.79	67308.89	53159.19	47758.54	57515	81078.3	40123.83	38243.7	50655.16	52525	1.0950	0.66098
A2AF47	Dock11	Dedicator of cytokinesis protein 11	67514.57	51241.41	59075.5	66117.31	60987	72447.9	65679.51	75207.42	72162.57	71374	0.8545	0.05008
P31324	Prkar2b	cAMP-dependent protein kinase type II-beta regulatc	116064.4	108809.1	98046.75	94472.85	104348	111238.9	103671.6	126084.7	118099.1	114774	0.9092	0.18085
Q8BGD5	Cpt1c	Carnitine O-palmitoyltransferase 1, brain isoform	26623.4	31187.61	32250.39	41023.58	32771	32865.08	27781.67	23830.6	38155.13	30658	1.0689	0.64260
Q9JHK4	Rabggta	Geranylgeranyl transferase type-2 subunit alpha	53099.22	52542.42	49046.52	44231.04	49730	48245.84	44498.21	35438.55	44511.35	43173	1.1519	0.10242
Q8R191	Syngr3	Synaptogyrin-3	20941	33027.2	28310.93	12657.51	23734	10862.09	13018.97	42639.95	34986.53	25377	0.9353	0.86254
Q8C863	Itch	E3 ubiquitin-protein ligase Itchy	9419.639	8374.437	10582.47	9300.056	9419	7162.58	10001.26	5727.797	8812.577	7926	1.1884	0.20092
Q9CR16	Ppid	Peptidyl-prolyl cis-trans isomerase D	60253.78	71399.05	64876.19	53173.2	62426	63923.04	57384.88	53876	53563.3	57187	1.0916	0.29155
Q810J8	Zfyve1	Zinc finger FYVE domain-containing protein 1	7907.486	6542.195	10093.38	4085.673	7157	9099.85	7833.475	5983.345	8768.915	7921	0.9035	0.61457
Q9D9H8		Mitochondrial protein C2orf69 homolog	8105.326				8105	4301.501	2256.231	4911.543	8092.549	4890	1.6574	
Q80TZ3	Dnajc6	Putative tyrosine-protein phosphatase auxilin	37714.03	40019.57	37084.49	37679.89	38124	41515	40588.27	36932.82	38742.79	39445	0.9665	0.31540
Q8BPM0	Daam1	Disheveled-associated activator of morphogenesis 1	26505.48		30364.63	39324.21	32065		26467.73	16665.04	20808.77	21314	1.5044	0.08600
Q9CZG9	Pdzd11	PDZ domain-containing protein 11	13509.74	14014.48	14804.13	13266.72	13899	12924.41	10138.27	8524.887	13772.34	11340	1.2256	0.08941
Q52KR3	Prune2	Protein prune homolog 2	57277.35	54937.78	49991.81	52972.45	53795	56460.34	59891.88	57558.99	58841.7	58188	0.9245	0.04279
P11499	Hsp90ab1	Heat shock protein HSP 90-beta	5820076	6216382	6602778	5509668	6037226	6395490	5582985	6289229	5911966	6044917	0.9987	0.98048
P70408	Cdh10	Cadherin-10												
Q8BZX4	Srek1	Splicing regulatory glutamine/lysine-rich protein 1	7543.235	7179.698	8320.189	8858.629	7975	8803.321	8293.924	11763.8	10804.51	9916	0.8043	0.07524
Q8CEE7	Rdh13	Retinol dehydrogenase 13	9178.116	9651.418	10567.92	6591.903	8997	8941.139	6739.719	6741.575	10164.67	8147	1.1044	0.50611
Q9CXE7	Tmed5	Transmembrane emp24 domain-containing protein 5	29069.71	39073.81	28277.26	22511.04	29733	24819.73	37736.94	28723.98	19066.67	27587	1.0778	0.69506
Q9D1D4	Tmed10	Transmembrane emp24 domain-containing protein 1	176028.6	166996.3	189505.5	194148.7	181670	179825.7	178637.8	199674.4	213939.2	193019	0.9412	0.32194
P62806	H4c1	Histone H4	8660734	7859414	8768753	9283190	8643023	8379554	8120358	9054772	9737624	8823077	0.9796	0.71331
Q9DB73	Cyb5r1	NADH-cytochrome b5 reductase 1	90488.19	101210.8	100629.2	124092.7	104105	102193.4	108856.5	112242.5	110516	108452	0.9599	0.58008
F8VPU2	Farp1	FERM, ARHGEF and pleckstrin domain-containing pr	17821.05	17244.65	18921.44	16543.4	17633	17302.25	19646.85	17395.75	20840.73	18796	0.9381	0.29106
Q1HFZ0	Nsun2	RNA cytosine C(5)-methyltransferase NSUN2	11905.76	11793.3	11121	11036.75	11464	10840.31	11117.47	10825.26	7371.986	10039	1.1420	0.17196
Q9CPX6	Atg3	Ubiquitin-like-conjugating enzyme ATG3	43183.6	44852.12	28202.16	30463.96	36675	33540.27	39138.77	29537.29	25938.86	32039	1.1447	0.40086
Q8BU30	Iars1	Isoleucine--tRNA ligase, cytoplasmic	280747.3	303395.8	293257.2	314383.4	297946	285536.2	331613.1	331559.4	335207.8	320979	0.9282	0.14733
B2RUR8	Otud7b	OTU domain-containing protein 7B	19202.08	17732.85	16311.41	14337.46	16896	15516.45	16877.45	13328.1	13853.74	14894	1.1344	0.17882
Q9CZW5	Tomm70	Mitochondrial import receptor subunit TOM70	96469.49	99142.47	104300.5	109250.7	102291	100096	95051.38	101032.3	104989.4	100292	1.0199	0.58798
B7ZMP1	Xpnpep3	Xaa-Pro aminopeptidase 3	16981.37	16482.52	15155.01	9779.067	14599	14333.68	11242.48		14641.85	13406	1.0890	0.60367
P16283	Slc4a3	Anion exchange protein 3												
Q3KNM2	Marchf5	E3 ubiquitin-protein ligase MARCHF5	40941.1	22502.01	30002.27	24760.12	29551	21480.93	24030.31	29009	30924.95	26361	1.1210	0.51846
Q9D2R6	Coa3	Cytochrome c oxidase assembly factor 3 homolog, n	45332.88	40326.34	43570.1	51276.37	45126	38297.08	38460.14	48064.3	53543.61	44591	1.0120	0.90725
P26049	Gabra3	Gamma-aminobutyric acid receptor subunit alpha-3												
Q9D4J1	Efhd1	EF-hand domain-containing protein D1	81879.3	84173.54	76892.05	71974.27	78730	69652.02	73107.35	51728.98	98422.13	73228	1.0751	0.60179
P46737	Brcc3	Lys-63-specific deubiquitinase BRCC36	21604.31	26278.36	25729.53	24329.75	24485	24931.58	20774.51	23439.46	26851.29	23999	1.0203	0.77858
Q8BW96	Camk1d	Calcium/calmodulin-dependent protein kinase type	45485.52	43181.71	30703.56	27304.52	36669	42426.31	41386.24	24428.14	22670.9	32728	1.1204	0.59214
P16014	Chgb	Secretogranin-1												
Q99J45	Nrbp1	Nuclear receptor-binding protein	35884.28	35984.1	32279.68	38566.44	35679	29943.93	35778.07	30430.31	35607.48	32940	1.0831	0.23018
Q8K448	Abca5	Cholesterol transporter ABCA5	11887.17	8728.062	6641.201	12052.64	9827	12014.32	6732.963	8551.702	11777.73	9769	1.0059	0.97575
Q9ERN0	Scamp2	Secretory carrier-associated membrane protein 2	9970.275	7583.642	9870.268	8341.361	8941	6640.768	8522.426	8294.351	9283.529	8185	1.0924	0.38572
P97445	Cacna1a	Voltage-dependent P/Q-type calcium channel subunit alpha-1A		11362.09	10827.89	10596.94	10929		11145.34	9928.683	12046.45	11040	0.9899	0.87328
Q811U4	Mfn1	Mitofusin-1	11459.28	10154.99	13066.33	13618.89	12075	11240.65	12045.89	12097.01	13424.63	12202	0.9896	0.89316
Q8R2Y8	Ptrh2	Peptidyl-tRNA hydrolase 2, mitochondrial	51046.59	43556.73	55113.74	60761.78	52620	51801.71	45632.91	72015.09	58810.8	57065	0.9221	0.53291
Q3UE37	Ube2z	Ubiquitin-conjugating enzyme E2 Z	54329.09	55018.69	49668.5	41197.41	50053	44772.49	44383.95	42886.34	37047.31	42273	1.1841	0.07704
Q61102	Abcb7	Iron-sulfur clusters transporter ABCB7, mitochondri	60670.56	52091.26	57999.47	59903.22	57666	53905.83	55472.63	56053.99	61671.89	56776	1.0157	0.74153
Q80U19	Daam2	Disheveled-associated activator of morphogenesis 2	18211.79	22344.33	15375.76	12611.37	17136	11554.26	18880.16	10608	11280.01	13081	1.3100	0.20402
P04370	Mbp	Myelin basic protein	38451332	27477262	19114220	42156296	31799778	25646008	34253344	24321452	25869518	27522581	1.1554	0.48303
Q8JZU2	Slc25a1	Tricarboxylate transport protein, mitochondrial	421761.6	418276.4	471467	497293	452199	464067.6	432500.5	516732.3	625702.5	509751	0.8871	0.26276
P70295	Aup1	Lipid droplet-regulating VLDL assembly factor AUP1	15846.96	14304.04	17351.12	18717.48	16555	16836.54	15946.6	16570.36	18941.13	17074	0.9696	0.66849
Q8BW75	Maob	Amine oxidase [flavin-containing] B	35379.02	32040.67	29159.24	33972.19	32638	35891.45	31219.08	27239.95	26004.57	30089	1.0847	0.36579
Q9DCT8	Crip2	Cysteine-rich protein 2	111335.4	128726.2	122493.2	110232.3	118197	111870.6	110015.3	119558.5	121898.5	115836	1.0204	0.67293
E9PUL5	Prrt2	Proline-rich transmembrane protein 2	19781.44		15012.76	22506.55	19100	20957.78	26942.89	27870.18		25257	0.7562	0.11628
Q99N84	Mrps18b	Small ribosomal subunit protein mS40												
P24288	Bcat1	Branched-chain-amino-acid aminotransferase, cyto:	56558.24	62697.02	49018.49	43660.77	52984	54114.76	47255.55	42511.75	45703.24	47396	1.1179	0.29266
Q61335	Bcap31	B-cell receptor-associated protein 31	41160.45	41627.44	43206.82	46791.35	43197	42778.94	46224.69	47543.44	46871.51	45855	0.9420	0.16015
Q99K85	Psat1	Phosphoserine aminotransferase	109046.6	111096	124144.1	92323.76	109153	104885.9	109140.1	115711.1	114644.7	111095	0.9825	0.79068
Q9CQE1	Nipsnap3b	Protein NipSnap homolog 3B	51192.27	59203.1	58433.59	61815.22	57661	57080.78	60247.98	59672.57	61511.65	59628	0.9670	0.45403
P17183	Eno2	Gamma-enolase	296394.9	313938.7	214176.2	211625.1	259034	267736.9	283288	188639.3	185855.3	231380	1.1195	0.48507

P30677	Gna14	Guanine nucleotide-binding protein subunit alpha-14	19264.58	24852.15	17532.45	19619.03	**20317**	20628.94	24273.11	16089.21	21865.89	**20714**	**0.9808**	**0.87039**
P42669	Pura	Transcriptional activator protein Pur-alpha	809959.9	701803.4	771881.9	753740.8	**759347**	718714.4	748926.6	684427.8	757028.6	**727274**	**1.0441**	**0.29370**
P80317	Cct6a	T-complex protein 1 subunit zeta	317856.9	310714.5	335569.7	343501.8	**326911**	343389.5	336592.4	392891.6	360737.7	**358403**	**0.9121**	**0.07580**
Q9R1V6	Adam22	Disintegrin and metalloproteinase domain-containin	36621.37	42968.93	33259.48	45035.72	**39471**	42619.68	49489.31	35709.07	36688.07	**41127**	**0.9598**	**0.70678**
P63028	Tpt1	Translationally-controlled tumor protein	52564.95	54816.8	46938.3	37824.87	**48036**	48424.03	50898.05	45740.33	43644.52	**47177**	**1.0182**	**0.84096**
Q4VBD2	Tapt1	Transmembrane anterior posterior transformation pr	14937.48	27835.93	17292.29	17034.21	**19275**	16482.96	17591.06	15957.82	19125.95	**17289**	**1.1148**	**0.53070**
Q2KN98	Specc1l	Cytospin-A	16044.15	15603.64	16484.03	14509.84	**15660**	14931.01	15408.83	12697.33	17014.98	**15013**	**1.0431**	**0.53615**
Q8BFZ3	Actbl2	Beta-actin-like protein 2	38706.41	62667.68	52645.57	35496.54	**47379**	47979.78	58184.95	73409.91	48935.37	**57128**	**0.8294**	**0.30206**
Q9CQ79	Txndc9	Thioredoxin domain-containing protein 9	34936.63	39079.6	40826.18	39147.57	**38497**	42564.61	37037.09	51300.14	40850.62	**42938**	**0.8966**	**0.22298**
P05132	Prkaca	cAMP-dependent protein kinase catalytic subunit alp	106376.8	112416.3	116141.6	113866.9	**112200**	118735.1	119448.8	106639.2	109762.6	**113646**	**0.9873**	**0.71876**
Q8C4X2	Csnk1g3	Casein kinase I isoform gamma-3	12168.96	9817.331	10725.67	11883.09	**11149**	11590.65	12735.63	5981.186	9747.814	**10014**	**1.1133**	**0.49820**
P01899	H2-D1	H-2 class I histocompatibility antigen, D-B alpha cha	138654	69802.37	58717.5	68310.24	**83871**	68647.47	73043.05	57345.05	74994.43	**68507**	**1.2243**	**0.44605**
Q8K2M0	Mrpl38	Large ribosomal subunit protein mL38	11683.56	9430.396	14769.52	15274.06	**12789**	14585.55	11664.08	11404.83	11418.55	**12268**	**1.0425**	**0.75213**
Q9CY28	Gtpbp8	GTP-binding protein 8	17870.8	14931.01	13997.89	16844.15	**15911**	13935.22	17039.86	14405.53	9876.307	**13814**	**1.1518**	**0.26923**
O54988	Slk	STE20-like serine/threonine-protein kinase	39079.18	43143.54	41551.11	34450.93	**39556**	37947.27	40482.01	35966.95	26037.09	**35108**	**1.1267**	**0.27305**
Q8BK08	Tmem11	Transmembrane protein 11, mitochondrial	36482.77	30430.29	35000.03	37761.01	**34919**	32908.86	34706.09	38196.66	37671.86	**35871**	**0.9735**	**0.65557**
Q9D6G9	Cmtm5	CKLF-like MARVEL transmembrane domain-containi	68347.07	72349.3	68157.3	75784.23	**71159**	59860.1	64683.86	79243.15	68607.05	**68099**	**1.0449**	**0.52233**
Q9Z224	Mocs2	Molybdopterin synthase sulfur carrier subunit	20677.86	25690.21	17772.49		**21380**	21884.82	29080.11	28183.85	15753.43	**23726**	**0.9011**	**0.59633**
P35980	Rpl18	Large ribosomal subunit protein eL18	532343.1	520319.5	559409.6	581313.9	**548347**	565594.9	550423.7	666490.4	669382.1	**612973**	**0.8946**	**0.11190**
Q3UZP4	Svip	Small VCP/p97-interacting protein	181516.7	151001.1	90486.08	195657.1	**154665**	146295.6	209029.3	116185.7	114210.7	**146430**	**1.0562**	**0.80641**
P48774	Gstm5	Glutathione S-transferase Mu 5	43656.46	45388.21	36164.33	31896.54	**39276**	32390.05	37395.75	28927.07	25880.78	**31148**	**1.2609**	**0.08968**
P08113	Hsp90b1	Endoplasmin	1622663	1731581	1979880	1865701	**1799956**	1813711	1738068	1854151	1993483	**1849853**	**0.9730**	**0.61659**
P21107	Tpm3	Tropomyosin alpha-3 chain	316711	313494.8	317585.4	327270.3	**318765**	310631	266738.9	277441.7	293146.3	**286989**	**1.1107**	**0.01926**
Q91ZR1	Rab4b	Ras-related protein Rab-4B	105306.1	104007.9	107464.3	107606.6	**106096**	105077	99928.02	112900.5	114720.9	**108157**	**0.9810**	**0.58363**
Q61390	Cct6b	T-complex protein 1 subunit zeta-2	44351.98	42548.43	44898.63	44435.77	**44059**	41457.27	48136.7	50302.08	43230.35	**45782**	**0.9624**	**0.44931**
P97352	S100a13	Protein S100-A13	167338.2	158806.6	151287.3	170392.4	**161956**	154856.3	157989.3	143365.8	128277.9	**146122**	**1.1084**	**0.09496**
Q9QZH6	Ecsit	Evolutionarily conserved signaling intermediate in To	10162.97	10328.02	9315.741	14077.94	**10971**	8984.818	9957.984	5838.704	11739.77	**9130**	**1.2016**	**0.30136**
Q2TPA8	Hsdl2	Hydroxysteroid dehydrogenase-like protein 2	85157.73	91774.93	94944.62	93891.3	**91442**	93790.63	99414.49	99802.51	103368	**99094**	**0.9228**	**0.04130**
P98200	Atp8a2	Phospholipid-transporting ATPase IB	25493.94	19340.41	18425.16	21029.71	**21072**	20305.89	24010.2	18326.34	22722.23	**21341**	**0.9874**	**0.89825**
Q80ZK0	Mrps10	Small ribosomal subunit protein uS10m	16841.61	26138.72	12236.1	26936.86	**20538**	26625.48	12253.43	24567.98	23409.98	**21714**	**0.9458**	**0.81563**
O54754	Aox1	Aldehyde oxidase 1	11632.66	16685.87	12864.66	14152.8	**13834**	14147.99	13177.88	13546.61	11180.16	**13013**	**1.0631**	**0.53815**
Q14B80	Kcnc2	Potassium voltage-gated channel subfamily C member 2								5767.296		**5767**	**0.0000**	
Q8BYN3	Itpk1	Inositol-tetrakisphosphate 1-kinase	35852.51	35121.52	31592.74	27475.79	**32511**	34214.17	32836.32	31830.47	31008.52	**32472**	**1.0012**	**0.98564**
Q9QYA2	Tomm40	Mitochondrial import receptor subunit TOM40 homo	71416.13	68088.67	76848.63	70043.82	**71599**	67214.92	80423.48	73326.49	81001.95	**75492**	**0.9484**	**0.34121**
Q8BUK6	Hook3	Protein Hook homolog 3	34426.16	38758.78	40121.73	35671.41	**37245**	36583.84	37657.03	38470.56	39313.36	**38006**	**0.9800**	**0.61704**
P28652	Camk2b	Calcium/calmodulin-dependent protein kinase type	229727.1	234680.4	248320.5	291246.7	**250994**	244281.6	243333.5	240332.1	252658.8	**245152**	**1.0238**	**0.69563**
Q91UZ5	Impa2	Inositol monophosphatase 2												
Q99LG4	Ttc5	Tetratricopeptide repeat protein 5	20942.12	22034.62	21968.94	21793.66	**21685**	19452.17	23536.14	21148.53	19662.37	**20950**	**1.0351**	**0.47923**
P63260	Actg1	Actin, cytoplasmic 2	2367425	1862794	1724338	1611327	**1891471**	1737766	1660944	3941174	1682535	**2255605**	**0.8386**	**0.55740**
Q05AA6	Drp2	Dystrophin-related protein 2	108685.7	94981.86	75477.98	112853.3	**98000**	97829.03	106590.2	80672.41	81628	**91680**	**1.0689**	**0.57076**
Q9R1E6	Enpp2	Ectonucleotide pyrophosphatase/phosphodiesteras	25047.1	14618.04	13856.41	19555.95	**18269**	15041.35	23199.43	19587.25	12338.84	**17542**	**1.0415**	**0.84370**
P29341	Pabpc1	Polyadenylate-binding protein 1	624692.4	603055.1	697018.1	688982.9	**653437**	681035.4	643523.1	750557.4	771760.7	**711719**	**0.9181**	**0.17498**
B2RY04	Dock5	Dedicator of cytokinesis protein 5	28701.06	26431.33	28552.65	29549.31	**28309**	26367.81	28103.35	21949.74	23263.05	**24921**	**1.1359**	**0.07243**
Q68FL6	Mars1	Methionine--tRNA ligase, cytoplasmic	167930.1	184905.3	198706.7	210980.2	**190631**	187697.8	200499.2	209680.4	202074	**199988**	**0.9532**	**0.39933**
Q9R0D8	Wdr54	WD repeat-containing protein 54	31672.36	42564.13	24032.11	27512.65	**31445**	42454.51	48966.23	28614.25	29546.27	**37395**	**0.8409**	**0.38876**
Q8BGY2	Eif5a2	Eukaryotic translation initiation factor 5A-2	13517.93	25204.16	19530.11	18943.33	**19299**	25123.8	24589.19	23721.68	18973.29	**23102**	**0.8354**	**0.21914**
Q61081	Cdc37	Hsp90 co-chaperone Cdc37	116398.2	119025.2	117037.4	111511.5	**115993**	117587.3	100666.1	123507.8	113148.3	**113727**	**1.0199**	**0.67238**
Q9CY66	Gar1	H/ACA ribonucleoprotein complex subunit 1	17900.8	19042.62	19327.12	22593.66	**19716**	21698.3	20778.82	20984.49	20841.93	**21076**	**0.9355**	**0.23470**
Q922Q4	Pycr2	Pyrroline-5-carboxylate reductase 2	45814.09	44809.36	54852.86	52044.79	**49380**	38751.11	46919.29	48993.52	52766.22	**46858**	**1.0538**	**0.53435**
Q9DCS3	Mecr	Enoyl-[acyl-carrier-protein] reductase, mitochondria	48664.84	48591.43	49217.8	58017.68	**51123**	49414.06	51877	55838.47	55738.38	**53217**	**0.9607**	**0.48062**
Q9Z1Q2	Abhd16a	Phosphatidylserine lipase ABHD16A	86937.66	77051.4	85506.94	83504.73	**83250**	77320.4	85657.27	86777.46	88376.02	**84533**	**0.9848**	**0.71051**
Q9WV95	Phlda3	Pleckstrin homology-like domain family A member 3	24172.15	21275.7	21791.78	25267.72	**23127**	20777.97	21514.83	20600.33	22323.79	**21304**	**1.0856**	**0.12734**
Q11011	Npepps	Puromycin-sensitive aminopeptidase	162707.4	172997.4	149586.5	138728.7	**156005**	162656.9	157247.2	140280.3	142106.5	**150573**	**1.0361**	**0.58107**
P57080	Usp25	Ubiquitin carboxyl-terminal hydrolase 25	23642.91	43694.68		14722.48	**27353**	57390.27	15907.94	12529.46		**28609**	**0.9561**	**0.94392**
Q61701	Elavl4	ELAV-like protein 4	335079.2	344338.1	374473.8	426358.6	**370062**	369857.5	374429.8	440296.2	409491.2	**398519**	**0.9286**	**0.32192**
Q68FH4	Galk2	N-acetylgalactosamine kinase	32123.58	33923.34	30485.26	20478.98	**29253**	31087.86	24647.5	26101.32	21498.19	**25834**	**1.1323**	**0.38022**
Q9R1X5	Abcc5	ATP-binding cassette sub-family C member 5	20979.97	10851.06	17566.7	21440.96	**17710**	20715.17	18018.66	21384.55	19891.06	**20002**	**0.8854**	**0.40328**
Q8R081	Hnrnpl	Heterogeneous nuclear ribonucleoprotein L	228577.5	233454.9	261044.4	275691.2	**249692**	254457.6	247537.2	264842.2	285562.4	**263100**	**0.9490**	**0.37394**
P51791	Clcn3	H(+)/Cl(-) exchange transporter 3		3106.352	2183.902	3642.621	**2978**							

Q4VA53	Pds5b	Sister chromatid cohesion protein PDS5 homolog B	13347.36	13700.61	15177.02	12477.07	13676	12470.15	11871.99	12302.29	12353.51	12249	1.1164	0.04854
Q62433	Ndrg1	Protein NDRG1	215419.8	215909.3	201396.7	203891.8	209154	214685.8	212217.3	182296	194370.5	200892	1.0411	0.37181
Q9R0Q7	Ptges3	Prostaglandin E synthase 3	261326.2	248484.6	256187	242704.5	252176	244376.9	248705.1	296590.3	219729.2	252350	0.9993	0.99193
Q99LF4	Rtcb	RNA-splicing ligase RtcB homolog	145426.6	143952.5	151968.1	149502.4	147712	136398.2	146876.5	157853.7	159848	150244	0.9831	0.67404
P05064	Aldoa	Fructose-bisphosphate aldolase A	1403224	1356234	1456745	1272227	1372108	1448680	1237603	1318923	1287452	1323164	1.0370	0.44345
Q9JMG3	Tmub1	Transmembrane and ubiquitin-like domain-containir	16958.85	20075.02	16284.03	18861.36	18045	16017.03	24282.77	13962.72	19310.42	18393	0.9811	0.88992
Q9DBD5	Pelp1	Proline-, glutamic acid- and leucine-rich protein 1	16536.97	22620.88	20059.93	14771.25	18497	18683.12	18187.84	16788.81	23268.05	19232	0.9618	0.75525
P62137	Ppp1ca	Serine/threonine-protein phosphatase PP1-alpha ca	88622.68	84085.17	82884.1	73479.13	82268	80684.41	86650	87586.43	83613.99	84634	0.9720	0.52923
Q921I0;Q9CQZ0	Ormdl1;Ormdl2	ORM1-like protein 1;ORM1-like protein 2	5168.969	7325.364	3757.232	5484.629	5434	4941.764	5809.112	3817.01	4930.654	4875	1.1148	0.53004
Q91WP6	Serpina3n	Serine protease inhibitor A3N	21931.42	16767.94	21687.56	47442.99	26957	12917.39	14588.85	18559.71	14488.8	15139	1.7807	0.14395
Q8BHN0	Ppm1l	Protein phosphatase 1L	38882.71	35352.47	50314.45	43896.75	42112	44617.17	39620.64	48512.41	60193.34	48236	0.8730	0.30440
Q5SSM3	Arhgap44	Rho GTPase-activating protein 44	13123.14	13311.16	12104.68	11958.01	12624	11815.08	14319.64	10694.36	12678.19	12377	1.0200	0.77799
Q8BX57	Pxk	PX domain-containing protein kinase-like protein	10732.78	6852.694	9236.569	10749.9	9393	10120.51	10132.85	8314.024	9578.124	9536	0.9850	0.89204
Q9CXW2	Mrps22	Small ribosomal subunit protein mS22	25832.9	20056.05	22845.54	27672.07	24102	23709.94	23607.24	23335.62	27569.14	24555	0.9815	0.82416
Q8VHC3	Selenom	Selenoprotein M	31551.34	38805.62	35910.52	38575.23	36211	31900.18	45983.36	31052.34	24644.55	33395	1.0843	0.57914
P54731	Faf1	FAS-associated factor 1	52792.12	54560.92	52533.31	56100.95	53997	58399.54	56378.62	47287.46	55022.17	54272	0.9949	0.91819
Q8C079	Strip1	Striatin-interacting protein 1	22247.24	18946.56	23734.41	22313.01	21810	23051.35	21097.13	25664.38	25997.59	23953	0.9106	0.21335
Q9D8Z6	Atg101	Autophagy-related protein 101	18950.99	14435.11	15034.84		16140							
Q6ZPZ3	Zc3h4	Zinc finger CCCH domain-containing protein 4	2458.851	1401.166	2681.381	1622.238	2041	1314.623	657.8726	7086.216	6135.322	3799	0.5373	0.33316
P62751	Rpl23a	Large ribosomal subunit protein uL23	495181.2	447774.7	526048.9	539730.6	502184	480575	495920.8	537780.7	599773.9	528513	0.9502	0.46254
P97467	Pam	Peptidyl-glycine alpha-amidating monooxygenase	5453.705	11703.36	8933.453	10054.8	9036	25098.9	5308.892	6622.285	11217.62	12062	0.7492	0.54486
Q6P8X1	Snx6	Sorting nexin-6	72909.47	77108.34	83117.76	65445.16	74645	77402.45	68907.1	73561.11	73917.27	73447	1.0163	0.78009
Q9CR41	Hypk	Huntingtin-interacting protein K	37302.79	42399.22	39293.54	36856.88	38963	36756.57	42016.04	38451.36	39220.85	39111	0.9962	0.93229
Q6NZJ6	Eif4g1	Eukaryotic translation initiation factor 4 gamma 1	112172.5	118082.2	127824.8	123304.7	120346	115023.7	120882.6	126457.8	130540.5	123226	0.9766	0.56830
Q9R087	Gpc6	Glypican-6	20735.93	15777.82	20314.74	20660.3	19372	17303.65	16828.72	11660.17	20213.62	16502	1.1740	0.22959
Q80UP5	Ankrd13a	Ankyrin repeat domain-containing protein 13A	10983.27	7510.342	11057.57	9359.133	9728	9993.772	8583.973	8713.081	11008.77	9575	1.0159	0.88532
P54728	Rad23b	UV excision repair protein RAD23 homolog B	104869.7	115103.6	95938.2	80070.66	98996	112401	109096.2	98210.34	92006.54	102929	0.9618	0.67085
O35098	Dpysl4	Dihydropyrimidinase-related protein 4	46982.14	47505.91	48498.75	46695.05	47420	39925.85	45573	48847.36	48750	45774	1.0360	0.46885
Q9JHW4	Eefsec	Selenocysteine-specific elongation factor	16846.03	23385.07	12952.72	15595.55	17195	18297.23	14144.34	6909.22	16539.26	13973	1.2306	0.37248
Q91YR1	Twf1	Twinfilin-1	65962.4	70192.41	72597.27	65942.71	68674	76631.36	65780.55	79939.62	72669.17	73755	0.9311	0.19251
Q7TMF3	Ndufa12	NADH dehydrogenase [ubiquinone] 1 alpha subcom	187940.7	181718.5	211761	224132.8	201388	192600.6	186745.1	213444.2	205997.1	199697	1.0085	0.88967
Q6P5E8	Dgkq	Diacylglycerol kinase theta	15862.48	13052.32	14335.96	17491.27	15186	11812.75	9899.184	10856.65	8146.727	10179	1.4919	0.00676
P14824	Anxa6	Annexin A6	1719252	1733555	1401282	1377613	1557925	1589962	1566037	1272431	1237564	1416498	1.0998	0.33563
O70293	Grk6	G protein-coupled receptor kinase 6	10156.41	12446.68	13014.02	10564.14	11545	13443.85	14135.82	12622.99	12857.12	13265	0.8704	0.06863
Q05816	Fabp5	Fatty acid-binding protein 5	338864	332310.7	339756.3	305712.6	329161	305258.4	316685.8	342488.8	291628.4	314015	1.0482	0.30225
Q8CHT1	Ngef	Ephexin-1												
P17751	Tpi1	Triosephosphate isomerase	576356.4	595698.6	561951.4	485749.6	554939	576246.1	522306.8	518575.5	479413.5	524135	1.0588	0.36210
P70271	Pdlim4	PDZ and LIM domain protein 4	41004.7	40200.77	42201	37937.97	40336	36626.05	33381.63	41396.48	36619.38	37006	1.0900	0.12683
Q80SW1	Ahcyl1	S-adenosylhomocysteine hydrolase-like protein 1	131575.6	121015.1	111624.9	120688.9	121226	112716.1	128070.4	108385.1	98665.91	111959	1.0828	0.25452
P47963	Rpl13	Large ribosomal subunit protein eL13	557062.8	592522.6	633151.2	692680.8	618854	618276.1	596403.1	729888.4	705197.4	662441	0.9342	0.35648
Q812E0	Cpeb2	Cytoplasmic polyadenylation element-binding protei	18702.35	16187.33	16247.26	18490.7	17407	15459.8	16235.18	21965.28	17049.28	17677	0.9847	0.87283
Q9D1H8	Mrpl53	Large ribosomal subunit protein mL53	33732.76	29840.25	29679.81	29659.42	30728	27513.67	27263.73	23515.29	27346.29	26410	1.1635	0.02107
P50171	Hsd17b8	(3R)-3-hydroxyacyl-CoA dehydrogenase	53803.5	55078.82	61326.43	72661.91	60718	60481.35	55857.73	59813.52	60646.88	59200	1.0256	0.74482
P61161	Actr2	Actin-related protein 2	157475.5	151286.4	153785.9	132358	148726	142106.3	145249.1	132624.3	140578	140139	1.0613	0.21621
Q8CGC7	Eprs1	Bifunctional glutamate/proline--tRNA ligase	213164	227694.5	235580.7	248371.8	231203	230225.8	250577.4	267999.5	258815.3	251905	0.9178	0.10672
Q91X96	Rabif	Guanine nucleotide exchange factor MSS4	26338.97	25908.84	25949.03	27579.52	26444	25376.61	24220.75	25331.46	24633.63	24891	1.0624	0.01793
Q8BXK8	Agap1	Arf-GAP with GTPase, ANK repeat and PH domain-co	16558.72	14317.14	15536.38	13988.7	15100	13512.64	14268.03	12178.53	12500.89	13115	1.1514	0.03977
P09528	Fth1	Ferritin heavy chain	247967.7	218006	125674.1	172764.2	191103	183562.9	255701.1	106658.2	106460	163096	1.1717	0.55390
Q9WTT4	Atp6v1g2	V-type proton ATPase subunit G 2	23505.02	19069.38	23400.49	25291.66	22817	24430.74	19024.07	26620.39	20379.89	22614	1.0090	0.92961
Q9ESN9	Mapk8ip3	C-Jun-amino-terminal kinase-interacting protein 3	32018.66	31303.17	34257.68	32508.24	32522	31890.61	29488.16	29210	31937.24	30631	1.0617	0.10015
Q8C1Y8	Ccz1	Vacuolar fusion protein CCZ1 homolog	22878.8	31640.12	21921.68	21064.71	24376	34049.85	30634.64	23858.63	26453.85	28749	0.8479	0.23674
Q5DTY9	Kctd16	BTB/POZ domain-containing protein KCTD16	8589.086	12276.81	9472.378	6728.591	9267	4671.702	8150.577	8177.63	6891.279	6973	1.3290	0.15697
Q8R1F6	Hid1	Protein HID1	15743.48	16093.1	17250.25	14061.05	15787	16178.54	17918.33	15120.73	13118.64	15584	1.0130	0.87139
Q8BR63	Fam177a1	Protein FAM177A1	18362.6	20605.19	22053.63	19016.74	20010	18198.22	21602.24	28743.94	22226.68	22693	0.8818	0.29766
Q8VDQ8	Sirt2	NAD-dependent protein deacetylase sirtuin-2	225428	223550.5	202962.3	222973.1	218728	220296.5	242391.4	208746.9	213893.4	221332	0.9882	0.78435
P63242	Eif5a	Eukaryotic translation initiation factor 5A-1	310791.6	324056.3	302547.9	268349.3	301436	296426.8	308779.5	285442.1	270116.4	290191	1.0388	0.46598
O55023	Impa1	Inositol monophosphatase 1	148378.2	145681.6	136602.8	105943.2	134151	137485	129182.9	121608.3	114400.1	125669	1.0675	0.46703
Q9D4C9	Clvs1	Clavesin-1	36109.59	36911.63	32040.57	34333.51	34849	35759.12	34141.98	28032.63	27527.76	31365	1.1111	0.19042
Q9D1A2	Cndp2	Cytosolic non-specific dipeptidase	244460.5	232659.9	225330.1	196829.6	224820	228712.5	227375.8	211702.4	200627.2	217104	1.0355	0.54889

Q3UUG6	Tbc1d24	TBC1 domain family member 24	9090.696	7758.667	19663.79	7416.763	10982	9502.002	7063.584	4204.585	6568.623	6835	1.6069	0.23095
P84228	H3c2	Histone H3.2	6954139	5672553	7255846	6613330	6623967	5425754	5396070	5335948	6290379	5612038	1.1803	0.04915
O09167	Rpl21	Large ribosomal subunit protein eL21	147359	137558.6	164326.8	162359.6	152901	149325.1	139771.4	181659.6	174962.8	161430	0.9472	0.49986
Q8K0S0	Phyhip	Phytanoyl-CoA hydroxylase-interacting protein	24215.2	25214.61	20816.9	19450.86	22424	25197.73	21967.3	18321.32	19988.64	21369	1.0494	0.61880
P51175	Ppox	Protoporphyrinogen oxidase	17831.88	13176.7	15529.18	16721.44	15815	16594.35	17523.15	15450.74	16851.61	16605	0.9524	0.49449
Q8R429	Atp2a1	Sarcoplasmic/endoplasmic reticulum calcium ATPas	357324.3	342525.1	317575.9	489098.6	376631	253011.5	303596	238499.9	302316.8	274356	1.3728	0.05034
Q99J08	Sec14l2	SEC14-like protein 2	13337.49	19942.1	23608.45	17265.37	18538	14385.89	13435.25	16784.05	15851.98	15114	1.2265	0.18578
Q8K0V4	Cnot3	CCR4-NOT transcription complex subunit 3	3837.48	5322.714	3800.19	6205.666	4792	5802.521	2020.733	887.9252	3582.732	3073	1.5590	0.20767
Q64310	Surf4	Surfeit locus protein 4	38440.44	41971.63	44971.61	47650.96	43259	46274.19	40299.4	39121.39	47966.78	43415	0.9964	0.95928
P56501	Ucp3	Putative mitochondrial transporter UCP3												
Q3UMF0	Cobll1	Cordon-bleu protein-like 1	17532.16	15387.07	15131.09	13726.19	15444	14775.45	15483.19	15784.25	15183.8	15307	1.0090	0.87160
P28665	Mug1	Murinoglobulin-1	50738.02	38264.28	114007.6	60729.05	65935	30217.65	29984.26	85581.8	67049.27	53208	1.2392	0.57866
Q9QZS3	Numb	Protein numb homolog	32918.25	32033.55	33528.84	32179.27	32665	32702.42	34030.11	36154.99	38361.08	35312	0.9250	0.08561
Q9D753	Exosc8	Exosome complex component RRP43	24065.53	24850.27	27540.08	23601.12	25014	23462.86	25475.65	22246.59	23259.29	23611	1.0594	0.25312
Q8BPU7	Elmo1	Engulfment and cell motility protein 1	28746.96	29173.96	27624.28	27345.81	28223	27485.96	26797.45	25516.69	27180.05	26745	1.0553	0.05343
Q6DFV7	Ncoa7	Nuclear receptor coactivator 7	10389.22	7672.539	8910.959	9567.402	9135	8015.03	10449.25	9375.528	8904.09	9186	0.9945	0.94910
B1AY13	Usp24	Ubiquitin carboxyl-terminal hydrolase 24	4140.666	4976.592	4434.904	5185.445	4684	7434.419	5436.94	7583.05	6408.713	6716	0.6975	0.01055
Q64487	Ptprd	Receptor-type tyrosine-protein phosphatase delta	19133.62	26158.52	21546.61		22280	40000.87	28703.08	33727.32	27108.2	32385	0.6880	0.04662
P63248	Pkia	cAMP-dependent protein kinase inhibitor alpha	15901.02	15764.44	11491.63		14386	12005.53	15109.26	15276.53	9602.005	12998	1.1067	0.52141
P21447	Abcb1a	ATP-dependent translocase ABCB1	82452.18	78772.36	75598.8	94378.42	82800	86714.17	88654.52	67931.81	76098.6	79850	1.0370	0.65827
O70439	Stx7	Syntaxin-7	135547.3	144272.5	152691.8	158773.2	147821	151094.4	155008.9	171998.2	168691.4	161698	0.9142	0.10156
Q6PE13	Prrt3	Proline-rich transmembrane protein 3												
Q8K442	Abca8a	ABC-type organic anion transporter ABCA8A	30492.45	27130.36	30854.93	31960.24	30109	29959.63	29817.4	27476.54	34857.7	30528	0.9863	0.83026
Q9ERF3	Skic8	Superkiller complex protein 8	17516.79	19230.56	17199.39	18531.04	18119	14966.65	18441.85	9291.279	17925.22	15156	1.1955	0.21747
P00493	Hprt1	Hypoxanthine-guanine phosphoribosyltransferase	246791.6	240493.9	185077.5	193006.7	216342	209635.1	228990.1	189346.9	163412.3	197846	1.0935	0.41674
Q9ERK4	Cse1l	Exportin-2	8579784	8683241	8352466	6864042	8119883	9265909	7576992	9090919	7984232	8479513	0.9576	0.56609
Q9D8U8	Snx5	Sorting nexin-5	32115.21	32534.41	37284.89	34667.56	34151	32057.44	28961.53	33892.66	31000.01	31478	1.0849	0.13957
Q8VDD8	Washc1	WASH complex subunit 1	29584.32	27908.43	26536.66	24040.18	27017	29607.21	28205.81	29331.37	27984.88	28782	0.9387	0.20425
Q9D1Q6	Erp44	Endoplasmic reticulum resident protein 44	79672.49	76182	83401.27	86273.56	81382	78163.5	78405.99	85251.6	86125.11	81987	0.9926	0.85053
Q9CWD3	Nudt17	Nucleoside diphosphate-linked moiety X motif 17												
Q91X21	Kiaa2013	Uncharacterized protein KIAA2013	9562.797	8897.929	17440.48	12099.85	12000	10506.61	7109.855	9495.489	9384.942	9124	1.3152	0.21380
P70372	Elavl1	ELAV-like protein 1	157367	154593	182674.2	180226.6	168715	170794.7	167402.8	188732.3	202558.3	182372	0.9251	0.26215
Q3TLS3	Gdpgp1	GDP-D-glucose phosphorylase 1	6290.201	8112.29	5735.146	5480.548	6405	6815.663	6065.754	9391.761	4960.602	6808	0.9407	0.72918
Q6ZWZ2	Ube2r2	Ubiquitin-conjugating enzyme E2 R2	30789.63	30507.41	25097.97	20915.48	26828	25818.19	26961.24	22252.96	22599.75	24408	1.0991	0.39466
P61961	Ufm1	Ubiquitin-fold modifier 1	76338.27	86887.3	67548.07	56142.44	71729	84087.64	77693.49	77436.35	71384.22	77650	0.9237	0.43159
Q9D8S9	Bola1	BolA-like protein 1	16351.05	20538.51	16449.5	13928.53	16817	15640.91	17216.88	11966.46	17539.94	15591	1.0786	0.53720
Q61584	Fxr1	RNA-binding protein FXR1	68342.13	64310.25	65732.34	73679.58	68016	68327.54	72632.76	71304.36	78862.27	72782	0.9345	0.16678
Q921M4	Golga2	Golgin subfamily A member 2	73539.34	61359.66	67429.52	76071.63	69600	68067.39	67771.7	77075.64	74767.22	71920	0.9677	0.58742
P23242	Gja1	Gap junction alpha-1 protein	19470.28	11567.96	10773.87	7689.587	12375	18135.24	20406.63	7160.302	13754.73	14864	0.8326	0.54152
P68372	Tubb4b	Tubulin beta-4B chain	6292058	6920842	6504030	6810979	6631977	7149863	6328213	6316757	6650589	6611355	1.0031	0.93501
Q99P58	Rab27b	Ras-related protein Rab-27B	23617.74	20699.62	21890.9	21370.77	21895	21010.63	23614.49	10856.56	8982.35	16116	1.3586	0.16839
Q9Z1S5	Septin3	Neuronal-specific septin-3	35635.3	43445.07	60943.19	44036.82	46015	47182.98	37773.13	27316.03	45910.71	39546	1.1636	0.39279
P23953	Ces1c	Carboxylesterase 1C	219708.9	218386.9	155467.4	61272.99	163709	171071.4	133147.4	81185.55	53506.59	109728	1.4920	0.28145
Q9DBE8	Alg2	Alpha-1,3/1,6-mannosyltransferase ALG2	144585.9	140398.2	143803.5	161645.3	147608	145962.1	158946.1	145961.6	157571.7	152110	0.9704	0.47788
Q8BXJ9	Tmem62	Transmembrane protein 62	7957.153	8895.931	10734.09	7414.814	8750	9101.402	9204.174	11681.14	13089.84	10769	0.8126	0.14867
Q99P47	Cntnap4	Contactin-associated protein-like 4												
Q80WC7	Agfg2	Arf-GAP domain and FG repeat-containing protein 2	31743.27	27156.8	21289.36	27006.15	26799	23707.55	22488.02	37426.14	21732.69	26339	1.0175	0.91806
P08249	Mdh2	Malate dehydrogenase, mitochondrial	1800360	1713518	1892668	2117766	1881078	1848008	1908191	2026844	2052586	1958907	0.9603	0.46421
Q9EPL9	Acox3	Peroxisomal acyl-coenzyme A oxidase 3	31236.04	29455.01	27741.62	30628.61	29765	28319.71	29174.12	29587.56	36328.95	30853	0.9648	0.60601
Q8VEK3	Hnrnpu	Heterogeneous nuclear ribonucleoprotein U	374022.8	380831.1	445036	449474.7	412341	403673.6	363575.3	439037.3	473800.2	420022	0.9817	0.81325
P62852	Rps25	Small ribosomal subunit protein eS25	720196.2	678633.2	810699.3	780956.9	747621	731452.1	728761.3	908743.3	921748	822676	0.9088	0.26610
Q9CPT3	p	N-acylneuraminate-9-phosphatase												
Q3TXS7	Psmd1	26S proteasome non-ATPase regulatory subunit 1	126522.1	123645.3	134448.9	126853.6	127867	131842	125107.7	140291.1	132865	132526	0.9648	0.27418
Q921T2	Tor1aip1	Torsin-1A-interacting protein 1	65359.35	59236.88	54323.11	69750.81	62168	60871.15	66550.59	54440.8	63640.63	61376	1.0129	0.85878
Q3UIL6	Plekha7	Pleckstrin homology domain-containing family A mei	24949.46	23123.03	23858.77	27720.39	24913	21847.42	23671.36	21708.38	23517.3	22686	1.0982	0.09795
P62071	Rras2	Ras-related protein R-Ras2	19281.04	17495.86	16059.1	18342.81	17795	18598.22	16812.19	14940.75	16295.69	16662	1.0680	0.30909
Q78XF5	Ostc	Oligosaccharyltransferase complex subunit OSTC	166356	168434.2	199030.6	183286	179277	162032.2	175459.5	177811.5	215209.8	182628	0.9816	0.81484
P53668	Limk1	LIM domain kinase 1	26553.86	30481.23	27626.89	23350.33	27003	25265.58	25646.29	23218.72	22857.97	24247	1.1137	0.14251
Q1RLL3	Cpne9	Copine-9	116172.4	99547.81	93290.36	131407.7	110105	137489.6	21419.9	97499.1	109568	91494	1.2034	0.50506

Q9D0M3	Cyc1	Cytochrome c1, heme protein, mitochondrial	227006.8	225169.8	208319.2	219426.5	219981	203357.4	221639.3	198919.1	194351	204567	1.0753	0.07963
Q6A058	Armcx2	Armadillo repeat-containing X-linked protein 2	8675.044	7611.932	6562.867	5377.754	7057	7383.095	10593.8	6427.218	5980.189	7596	0.9290	0.68324
Q03059	Chat	Choline O-acetyltransferase	109033	96314.45	71334.77	71394.59	87019	98375.52	89033.2	52145.93	52288.79	72961	1.1927	0.39494
Q9DB72	Btbd17	BTB/POZ domain-containing protein 17									25047.46	25047	0.0000	
Q8BJZ4	Mrps35	Small ribosomal subunit protein mS35	12611.43	8863.186	10920.13	17777.57	12543	9226.712	13154	15391.98	12071.12	12461	1.0066	0.97263
Q3URE1	Acsf3	Malonate--CoA ligase ACSF3, mitochondrial	15021.47	14833.85	16135.56	18409	16100	15760.07	16558.94	15597	16635.4	16138	0.9977	0.96645
Q8BGN8	Synpr	Synaptoporin	9782.703				9783	5295.771	5565.054	6757.121		5873	1.6658	
Q9Z0H8	Clip2	CAP-Gly domain-containing linker protein 2	90253.38	97840.92	93765.03	100551.3	95603	87906.25	90442.98	91046.91	93471.01	90717	1.0539	0.10230
Q920E5	Fdps	Farnesyl pyrophosphate synthase	176335.4	193603.4	214301.2	150386.6	183657	184240.9	174206.4	201321.8	183968.6	185934	0.9877	0.88164
Q8VDJ3	Hdlbp	Vigilin	285273.6	285635.5	339353.1	332795	310764	334153.1	310627.9	362773.7	383039.1	347648	0.8939	0.13913
Q9D8B4	Ndufa11	NADH dehydrogenase [ubiquinone] 1 alpha subcomp	61929.85	57256.46	81747.55	86323.2	71814	69990.41	68617.58	83968.56	81995.2	76143	0.9432	0.61693
Q9WUM5	Suclg1	Succinate--CoA ligase [ADP/GDP-forming] subunit al	110539.3	106451	108308.3	130631.9	113983	109943.8	112103.7	106348.4	110696.9	109773	1.0383	0.49133
Q8R1G6	Pdlim2	PDZ and LIM domain protein 2	4042.615	5750.13	5315.498	5817.47	5231	3389.528	3688.366	4386.284	4046.359	3878	1.3491	0.02692
P59481	Lman2l	VIP36-like protein	98941.54	92616.62	102413.1	99060.59	98258	92393.3	127088.8	99051.65	104402.1	105734	0.9293	0.37501
Q9D7S7	Rpl22l1	Ribosomal protein eL22-like 1	21581.78	23181.33	37360.2	28305.18	27607	32672.84	23457.46	35171.33	35168.57	31618	0.8732	0.40844
Q05512	Mark2	Serine/threonine-protein kinase MARK2	15649.92	14715.83	17216.83	18270.21	16463	15310.94	17385.78	15418.78	18265.29	16595	0.9920	0.90671
Q60692	Psmb6	Proteasome subunit beta type-6	117794.9	120308.6	119947.2	105841.9	115973	115816.9	117944.9	110463.1	122032.7	116564	0.9949	0.89227
P97490	Adcy8	Adenylate cyclase type 8	20616.49				20616	16027.3	12933.69	12397.95		13786	1.4954	
Q9D051	Pdhb	Pyruvate dehydrogenase E1 component subunit beta	432429.3	433238.8	405703.2	474007.4	436345	426450.3	471965.9	409472	433630.7	435380	1.0022	0.96177
Q921S7	Mrpl37	Large ribosomal subunit protein mL37	13276.16	15674.52	15260.74	14044.3	14564	12511.05	16124.65	15408.3	15280.01	14831	0.9820	0.79182
Q9CZJ2	Hspa12b	Heat shock 70 kDa protein 12B	491853.7	474432.3	487677.2	455250.2	477303	540931.2	462336.7	413891	409933.9	456773	1.0449	0.53959
Q02357	Ank1	Ankyrin-1	40210.77	40873.46	42551.44	49010.97	43162	40093.64	41973.31	43882.97	39816.18	41442	1.0415	0.46819
Q9CWK8	Snx2	Sorting nexin-2	84720.48	78657.24	86953.27	71368.35	80425	81288.99	74920.63	81241.88	77438.73	78723	1.0216	0.67162
Q9DBZ5	Eif3k	Eukaryotic translation initiation factor 3 subunit K	127236.5	170684.7	124243.9	164520.7	146671	133293	126794.5	155272.8	143725.8	139772	1.0494	0.63172
Q9JLR1	Sec61a2	Protein transport protein Sec61 subunit alpha isofor	66648.63	68039.82	73946.5	70477	69778	64127.3	79682.71	72823.48	81001.88	74409	0.9378	0.31089
P20917	Mag	Myelin-associated glycoprotein	616231.8	577340	620438.6	699326.1	628334	607260.8	592568.9	569815.2	633051.4	600674	1.0460	0.37405
Q3TIR3	Ric8a	Synembryn-A	38474.21	45997.37	41202.98	37164.27	40710	40812.99	43601.04	40164.04	40604.34	41296	0.9858	0.78992
Q99KQ4	Nampt	Nicotinamide phosphoribosyltransferase	101647.1	89644.61	99498.35	81518.09	93077	83778.63	78516.52	83448.99	83440.97	82296	1.1310	0.06680
Q0VBF8	Stum	Protein stum homolog												
P62317	Snrpd2	Small nuclear ribonucleoprotein Sm D2	106522.6	104124	119544	117372.6	111891	109198.3	111553.6	121617.5	118963.7	115333	0.9702	0.50453
P53798	Fdft1	Squalene synthase	74580.61	79223.2	103680.5	86646.6	86033	79341.94	86814.1	109077.3	121548.5	99195	0.8673	0.30241
Q80Y17	Llgl1	Lethal(2) giant larvae protein homolog 1	25584.76	27833.48	23823.34	31014.95	27064	27971.95	25648.16	23305.53	28255.74	26295	1.0292	0.70481
Q9ER73	Elp4	Elongator complex protein 4	15505.58	12559.01	11847.39	13816.46	13432	13213.63	9192.008	10106.55	12480.39	11248	1.1942	0.13021
Q8R1G2	Cmbl	Carboxymethylenebutenolidase homolog	50907.02	53029.18	43123.5	41141.5	47050	46218.6	48249.57	42776.54	44946.59	45548	1.0330	0.64708
O08576	Rundc3a	RUN domain-containing protein 3A	42573.87	39269.59	38711.22	38056.95	39653	36927.89	40787.42	35897.22	39739.61	38338	1.0343	0.42259
P41105	Rpl28	Large ribosomal subunit protein eL28	305680.9	269192.3	333870.1	347159.9	313958	319481.5	295081.9	332403	362368	327334	0.9591	0.56920
P63005	Pafah1b1	Platelet-activating factor acetylhydrolase IB subunit	52745.4	51441.21	52537.38	42721.47	49861	54120.62	50100.71	51647.82	51675.22	51886	0.9610	0.45524
Q3TCJ1	Abraxas2	BRISC complex subunit Abraxas 2	23909.87	19455.11	19266.35	20922.79	20889	18868.19	22411.82	20970.96	21254.75	20876	1.0006	0.99289
Q3UDE2	Ttll12	Tubulin--tyrosine ligase-like protein 12	39743.41	38514.46	36283.12	32298.65	36710	40186.94	34974.16	32777.26	34414.01	35588	1.0315	0.64155
Q99JA0	Calca	Calcitonin gene-related peptide 1	48622.13	71578.17	43004.21	43508.74	51678	31302.21	65709.05	46734.52	41970.14	46429	1.1131	0.61377
Q8CFE4	Scyl2	SCY1-like protein 2	16953.91	17207.79	15522.52	12766.43	15613	16427.21	16412.59	15926.25	13514.13	15570	1.0027	0.97356
P97819	Pla2g6	85/88 kDa calcium-independent phospholipase A2	8526.213	9557.678	11507.61	10669.96	10065	11581.44	10581.93	10607.78	12504.99	11319	0.8892	0.16618
O70310	Nmt1	Glycylpeptide N-tetradecanoyltransferase 1	283595.6	302913.4	292744.4	293045.3	293075	295585.7	316310	286430.9	300705.8	299758	0.9777	0.40108
P31001	Des	Desmin	263501.4	221863.6	191008.1	251101.2	231869	168649.3	227699.4	112208.6	215663.5	181055	1.2807	0.15044
Q8K0C9	Gmds	GDP-mannose 4,6 dehydratase	24437.3	30169.17	22532.28	21712.35	24713	27564.25	27462.61	24626.67	23421.04	25769	0.9590	0.64384
P21126	Ubl4a	Ubiquitin-like protein 4A	30989.06	33932.81	30637.02	26851.43	30603	31402.8	30235.92	32993.98	28269.26	30725	0.9960	0.94661
Q03958	Pfdn6	Prefoldin subunit 6	58918.76	58627.48	54188.04	47841.8	54894	59114.63	50872.63	46997.66	49297.82	51571	1.0644	0.40310
Q9WU79	Prodh	Proline dehydrogenase 1, mitochondrial	14242.61	11521.69	10875.45	14762.27	12851	13075.85	13688.61	9890.726	11679.59	12084	1.0635	0.57171
Q3UHQ6	Dop1b	Protein dopey-2	19247.47	18647.45	19080.1	16766.58	18435	19166.11	18501.16	17707.63	19694.22	18767	0.9823	0.65844
P31230	Aimp1	Aminoacyl tRNA synthase complex-interacting multi	166169.9	168227.8	166160.8	171417.2	167994	162592	173959.1	179396.6	178689.1	173659	0.9674	0.21382
P63054	Pcp4	Calmodulin regulator protein PCP4	939397.9	996665.7	663698.4	436045.2	758952	479366.1	614139	408625.8	414271	479100	1.5841	0.08962
Q9D824	Fip1l1	Pre-mRNA 3'-end-processing factor FIP1	22611.94	17266.35	28837.54	28186.25	24226	24695.38	19552.96	25971.2	23295.35	23379	1.0362	0.79011
Q64096	Mcf2l	Guanine nucleotide exchange factor DBS	3647.471	4116.674	3349.602		3705		4131.188			4131	0.8967	
O88935	Syn1	Synapsin-1	45260.49	42293.36	42696.66	39537.75	42447	40665.01	39763.73	50176.86	48369.26	44744	0.9487	0.45779
P61953	Gng11	Guanine nucleotide-binding protein G(I)/G(S)/G(O) s	4721.186			1841.318	3281	6778.155			4558.713	5668	0.5789	0.31957
Q9WV80	Snx1	Sorting nexin-1	54061.35	53943.25	63160.13	48302.94	54867	50399.61	51517.66	50979.36	53560.79	51614	1.0630	0.34157
P03930	Mtatp8	ATP synthase protein 8	40822.1	15988.72	23083.51	27249.92	26786	17649.42	36061.68	17855.79	27248.85	24704	1.0843	0.77075
Q6PH08	Erc2	ERC protein 2	17820.75	16611.1	22811.15	20094.82	19334	19881.49	19902.2	26236.48	29571.92	23898	0.8090	0.15066
P06909	Cfh	Complement factor H	84242.79	58687.02	56226.76	61821.39	65244	53730.3	48465.31	38783.7	38624.65	44901	1.4531	0.03401

Q9WV54	Asah1	Acid ceramidase	121589.8	118422.6	99428.18	116315.2	113939	123998.9	127531.1	110945.7	111833.1	118577	0.9609	0.50275
O35643	Ap1b1	AP-1 complex subunit beta-1	125384.5	124797.4	126117.5	125562	125465	122074.2	139162.7	128485.8	133099.5	130706	0.9599	0.19829
Q8CDA1	Inpp5f	Phosphatidylinositide phosphatase SAC2	8969.676	8212.71	7374.147	5817.81	7594	8740.405	7525.421	6508.048	6473.586	7312	1.0385	0.75487
Q8BIJ6	Iars2	Isoleucine--tRNA ligase, mitochondrial	67686.45	67933.19	68382.92	81146.67	71287	74623.21	74124.45	75703.5	76696.93	75287	0.9469	0.27617
Q8C7D2	Crbn	Protein cereblon	28788.71	28465.09	28071.63	25349.65	27669	31293.21	28971.79	28355.49	26909.08	28882	0.9580	0.35269
P70227	Itpr3	Inositol 1,4,5-trisphosphate receptor type 3	36664.86	62452.82	41380.38	39686.43	45046	32224.62	35512.74	38735.09	43105.62	37395	1.2046	0.27188
P62821	Rab1A	Ras-related protein Rab-1A	201534.5	185057.2	206905.2	222979.3	204119	210532.7	207682.8	232870.3	227557.3	219661	0.9292	0.17071
Q9CQN7	Mrpl41	Large ribosomal subunit protein mL41	14781.74	15426.49	17028.8	15955.71	15798	14476.31	17271.87	14638.23	17075.95	15866	0.9958	0.94235
Q7M753	Pank2	Pantothenate kinase 2, mitochondrial	3939.515	4153.564	9450.399	2275.927	4955	4550.432	4230.219	2969.897		3917	1.2650	0.60556
Q9QYK9	Pnck	Calcium/calmodulin-dependent protein kinase type 1B				19793.15	19793							
O35969	Gamt	Guanidinoacetate N-methyltransferase	23574.32	24175.39	23814.2	23847.77	23853	24517.66	24991.57	25311.91	23991.81	24703	0.9656	0.03489
P56528	Cd38	ADP-ribosyl cyclase/cyclic ADP-ribose hydrolase 1	25142.87	22300.77	18889.81	23473.21	22452	17701.59	22756.04	23219.7	18813.89	20623	1.0887	0.37690
P62858	Rps28	Small ribosomal subunit protein eS28	519268.8	534809	661461.2	587643.9	575796	566886.9	646614.8	637568.1	757319.3	652097	0.8830	0.18354
Q9QYE6	Golga5	Golgin subfamily A member 5	22531.56	20647.79	24716.19	27199.29	23774	24993.87	21661.63	27132.4	30378.46	26042	0.9129	0.36477
Q9EQP6	Arr3	Arrestin-C	22509.62	28846.34	23082.16	18756.83	23299	20709.29	27862.69	13831.01	17828.55	20058	1.1616	0.40499
Q8BFR5	Tufm	Elongation factor Tu, mitochondrial	452302.3	449972	457946.6	476012.8	459058	451586.5	466765.5	452713.7	469159.7	460056	0.9978	0.89815
Q3UHB1	Nt5dc3	5'-nucleotidase domain-containing protein 3	39257.17	47495.98	51628.61	52948.74	47833	49808.46	54065.77	43793.35	51959.54	49907	0.9584	0.60472
Q921L3	Tmco1	Calcium load-activated calcium channel	35715.71	33014.48	39463.97	39765.32	36990	35595.33	38137.84	51509.76	50767.69	44003	0.8406	0.16674
P63318	Prkcg	Protein kinase C gamma type												
P49769	Psen1	Presenilin-1	3713.456	3686.662	2690.315	4010.065	3525	4088.845	4171.807		3301.184	3854	0.9147	0.46108
Q9D0B6	Pbdc1	Protein PBDC1	27295.67	34712.88	20742.62	21288.71	26010	29463.79	32523.59	12099.48	20855.06	23735	1.0958	0.70052
Q9ES56	Trappc4	Trafficking protein particle complex subunit 4	23980.07	21959.78	27470.17	25940.13	24838	25636.24	26244.43	27653.75	26328.56	26466	0.9385	0.24687
Q99KK2	Cmas	N-acylneuraminate cytidylyltransferase	95991.89	95729.79	96852.3	96221.88	96199	97701.23	96322.29	111270.7	111839.6	104283	0.9225	0.10363
Q925I1	Atad3	ATPase family AAA domain-containing protein 3	35885.11	32043.72	34861.85	40060.48	35713	35405.02	36999.33	33377.97	37794.61	35894	0.9949	0.92802
P29416	Hexa	Beta-hexosaminidase subunit alpha	67170.17	63862.1	61630.67	64440.34	64276	65277.38	70868.12	58991.14	62724.74	64465	0.9971	0.94714
Q8R123	Flad1	FAD synthase	25784.53	29873.34	25559.58	31031.05	28062	27239.87	25904.58	19503.68	22877.26	23881	1.1751	0.10855
P26048	Gabra2	Gamma-aminobutyric acid receptor subunit alpha-2												
Q8C7B6	Fbxl22	F-box and leucine-rich protein 22	34205.36	29967.7	31971.36	35172.55	32829	32506.83	34925.87	37035.63	38261.71	35683	0.9200	0.14797
G3X9K3	Arfgef1	Brefeldin A-inhibited guanine nucleotide-exchange p	19745.56	21188.95	22422.65	15712.02	19767	19980.8	18534.93	15856.93	22250.88	19156	1.0319	0.76792
P08775	Polr2a	DNA-directed RNA polymerase II subunit RPB1	5013.29	5102.814	5742.581	3242.186	4775	3111.927	4782.146	3370.775	3485.586	3688	1.2949	0.14698
Q8R0S2	Iqsec1	IQ motif and SEC7 domain-containing protein 1	50065.1	44190.02	44260.22	54718.86	48309	50890.99	53724.72	43887.85	44132.87	48159	1.0031	0.96770
P16858	Gapdh	Glyceraldehyde-3-phosphate dehydrogenase	1437663	1504245	1363917	1337711	1410884	1592315	1377938	1201286	1269772	1360328	1.0372	0.60769
P17665	Cox7c	Cytochrome c oxidase subunit 7C, mitochondrial	16122.48			14934.27	15528			13473.42		13473	1.1525	
Q78PG9	Ccdc25	Coiled-coil domain-containing protein 25	10893.35	11406.29	12959.82	12953.18	12053	11192.43	9890.541	11707.3	11262.25	11013	1.0944	0.16630
Q5XKN4	Jagn1	Protein jagunal homolog 1	29630.18	25105.18	29570.32	24595.55	27225	22632.94	37503.48	23317	19030.78	25621	1.0626	0.72172
Q3U1V6	Uevld	Ubiquitin-conjugating enzyme E2 variant 3	9756.284	9614.839	7913.052	8349.867	8909	7936.435	6653.56	6513.733	6699.962	6951	1.2816	0.01342
Q91XY4	Pcdhga4	Protocadherin gamma-A4	22532.74	18224.73	20346.91	21357.23	20615	18436.86	20926.51	14376.32	22311.63	19013	1.0843	0.44616
Q9ESM3	Hapln2	Hyaluro and proteoglycan link protein 2	21253.34	36243.39	6305.616	29616.54	23355	37545.83	44361.11	31448.54		37785	0.6181	0.14081
Q9CR61	Ndufb7	NADH dehydrogenase [ubiquinone] 1 beta subcomp	39338.23	44405.11	52341.33	59214.64	48825	41711.79	59831.79	62269.97	63167.73	56745	0.8604	0.28124
P63143	Kcnab1	Voltage-gated potassium channel subunit beta-1	6769.856	6973.484	5936.998	6311.399	6498	5628.803	6739.467	6176.897	5055.082	5900	1.1013	0.21375
P70265	Pfkfb2	6-phosphofructo-2-kinase/fructose-2,6-bisphospha	14068.23	14836.46	12814.66	10830.15	13137	13859.94	16460.62	12115.21		14145	0.9287	0.52580
P69566	Ranbp9	Ran-binding protein 9	13246.73	16498.17	14316.05	17459.55	15380	12103.25	12773.11	14255.67	15337.55	13617	1.1294	0.19609
P47964	Rpl36	Large ribosomal subunit protein eL36	236519.6	233925.7	261368	273463.9	251319	221083.1	235563.4	291100.2	276773.8	256130	0.9812	0.81029
Q8BTY1	Kyat1	Kynurenine--oxoglutarate transaminase 1	15706.89	16200.15	10603.17	14550.37	14265	19631.58	17663.19	10158.08		15818	0.9019	0.60853
P68040	Rack1	Small ribosomal subunit protein RACK1	824206.9	785348.8	905233.4	936933.4	862931	869837.8	853372.8	1011609	973233.6	927013	0.9309	0.26602
Q8BP48	Metap1	Methionine aminopeptidase 1	40923.22	36306.4	39297.1	37822.22	38587	36314.97	39807.88	40396.02	41569.27	39522	0.9763	0.55658
Q8JZP2	Syn3	Synapsin-3	18755.64	32884.78		12070.58	21237		13354.04			13354	1.5903	
P60603	Romo1	Reactive oxygen species modulator 1	13654.92	13652.63	15615.79	13793.49	14179	13410.16	15709.58	12770.99	12787.53	13670	1.0373	0.56874
Q9Z307	Kcnj16	Inward rectifier potassium channel 16												
P29595	Nedd8	NEDD8	58070.09	57445.71	55165.67	39493.28	52544	59428.1	54637.16	55808.45	52011.57	55471	0.9472	0.55270
Q7TSE6	Stk38l	Serine/threonine-protein kinase 38-like	15195.16	18221.61	15265.26	11709.99	15098	15760.77	13416.13	10940.67	11336.24	12863	1.1737	0.24451
Q9ERR1	Ndel1	Nuclear distribution protein nudE-like 1	55826.71	35878.63	49768.33	37452.52	44732	50715.05	39834.96	29561.91	46250.5	41591	1.0755	0.65396
P53811	Pitpnb	Phosphatidylinositol transfer protein beta isoform	20581.43	20255.2	21357.59	14068.43	19066	18473.17	17200.83	12392.31	14920.08	15747	1.2108	0.17343
Q8BK30	Ndufv3	NADH dehydrogenase [ubiquinone] flavoprotein 3, m	8957.184	22372.63	14186.18	11982.37	14375	15480.8	13471.69	13504.6	17443.79	14975	0.9599	0.84920
Q9D404	Oxsm	3-oxoacyl-[acyl-carrier-protein] synthase, mitochonc	43124.61	46808.53	43480.83	49661.73	45769	42869.59	44141.37	44344.7	45079.87	44109	1.0376	0.34149
P52432	Polr1c	DNA-directed RNA polymerases I and III subunit RPA	35245.91	32449.33	32137.62	28117.54	31988	36201.35	36452.56	30512.16	33126.21	34073	0.9388	0.34455
Q8R317	Ubqln1	Ubiquilin-1	123683.6	135828	119910.3	89860.05	117321	111460.5	121987.7	110631.5	111352.7	113858	1.0304	0.74425
A6X919	Dpy19l1	Probable C-mannosyltransferase DPY19L1	17303.3		26161.2	22084.56	21850	28287.95	27582.26	24780.06	27479.33	27032	0.8083	0.07655
Q9CPP6	Ndufa5	NADH dehydrogenase [ubiquinone] 1 alpha subcom	88039.8	83836.84	93444.87	104547.7	92467	83114.45	86372.43	96069.57	102533.9	92023	1.0048	0.94618

P62830	Rpl23	Large ribosomal subunit protein uL14	568424.7	562387.4	665918.4	657294.5	613506	664263.9	557926.2	729614.4	765278.7	679271	0.9032	0.26402
Q8BKE6	Cyp20a1	Cytochrome P450 20A1	10960.08	10500.23	12528.64	12325.33	11579	10990.42	12984.75	12739.02	16365.12	13270	0.8725	0.21816
Q60780	Gas7	Growth arrest-specific protein 7	44567.32	41767.33	39700.55	45382.4	42854	43065.43	43221.42	44747.84	43244.09	43570	0.9836	0.61877
P42125	Eci1	Enoyl-CoA delta isomerase 1, mitochondrial	121101.1	120767.1	113511.5	133932.5	122328	109610.4	120992.1	107974.1	113498	113019	1.0824	0.12016
Q5F2E7	Nufip2	FMR1-interacting protein NUFIP2	40375.63	42084.66	43836.84	46429.06	43182	38947.95	42460.61	50672.84	49224.63	45327	0.9527	0.51024
Q9JHI5	Ivd	Isovaleryl-CoA dehydrogenase, mitochondrial	80096.61	68267.34	70244.82	83612.55	75555	79015.39	82885.23	76744.23	75782.63	78607	0.9612	0.47962
Q3TLI0	Trappc10	Trafficking protein particle complex subunit 10	17113.18	16737.13	19517.52	12792.66	16540	16008.82	14157.07	14386.6	16415.79	15242	1.0852	0.42130
Q9QYB1	Clic4	Chloride intracellular channel protein 4	52461.02	51927.29	48967.52	41025.07	48595	48105.29	51422	49308.61	46502.02	48834	0.9951	0.93547
Q8BMJ2	Lars1	Leucine--tRNA ligase, cytoplasmic	197771.2	208569.3	217996	238335	215668	207121.4	226731.5	238421.5	233789.3	226516	0.9521	0.36346
Q8VBT9	Aspscr1	Tether containing UBX domain for GLUT4	18450.9	16674.86	18934.27	21958.01	19005	18352	17418.84	16708.65	18622.74	17776	1.0691	0.33870
Q9CPW4	Arpc5	Actin-related protein 2/3 complex subunit 5	205692.7	193309.7	217989.6	202468.3	204865	195170.6	201518.1	222443.8	188834.1	201992	1.0142	0.75774
Q5SSL4	Abr	Active breakpoint cluster region-related protein	34290.93	34613.68	32725.01	27371.64	32250	31182.8	33655.21	28151.95	27041.4	30008	1.0747	0.35725
P50571	Gabrb1	Gamma-aminobutyric acid receptor subunit beta-1			3011.117	10323.94	6668							
F6ZDS4	Tpr	Nucleoprotein TPR	22867.44	20879.89	22524.4	22733.75	22251	20849.41	22488.71	22433	22376.14	22037	1.0097	0.73675
P15116	Cdh2	Cadherin-2	50673.78	49208.61	53040.53	52488.94	51353	46949.54	48086.73	50524.21	54652.14	50053	1.0260	0.52289
Q80TN5	Zdhhc17	Palmitoyltransferase ZDHHC17	52177.26	45888	49510.41	50199.61	49444	47488.48	49889.46	54433.43	57798.78	52403	0.9435	0.30725
Q99LY9	Ndufs5	NADH dehydrogenase [ubiquinone] iron-sulfur prote	70542.14	74104.41	79472.66	89082.72	78300	73709.89	82045.64	74646.41	85891.46	79073	0.9902	0.88205
Q3UX10	Tubal3	Tubulin alpha chain-like 3	12681.16	13601.22	7940.553	7164.763	10347	10185.87	13426.8	5017.327	8255.578	9221	1.1221	0.65578
P26883	Fkbp1a	Peptidyl-prolyl cis-trans isomerase FKBP1A	371643.3	406313.2	451148	333990.5	390774	405887.6	377200.2	367487.3	388002.3	384644	1.0159	0.82335
Q9D7E4		UPF0449 protein C19orf25 homolog	40267.14	39558.32	42894.79	43023.63	41436	46673.7	40248.67	44219.35	48051.21	44798	0.9249	0.13204
Q8C878	Uba3	NEDD8-activating enzyme E1 catalytic subunit	51343.84	53072.38	45742.43	44096.71	48564	43948.54	45152.19	44881.39	41747.7	43932	1.1054	0.09000
Q920Q4	Vps16	Vacuolar protein sorting-associated protein 16 homo	42000.3	40075.58	42275.77	34528.03	39720	37692.14	39853.82	34422.81	42368.89	38584	1.0294	0.66116
P11438	Lamp1	Lysosome-associated membrane glycoprotein 1	88401.66	99475.62	102408.6	94855.81	96285	103540.8	103613.8	84731.16	100101.9	97997	0.9825	0.76353
Q9JMH9	Myo18a	Unconventional myosin-XVIIIa	61785.87	56230.51	55022.85	60068.75	58277	58591.77	56080.5	58335.06	60547.39	58389	0.9981	0.95340
P07309	Ttr	Transthyretin	203633.6	188025.3	165790.6	78665.55	159029	127653.1	127582.9	134288.3	97571.51	121774	1.3059	0.24738
Q80YA3	Ddhd1	Phospholipase DDHD1	19264.22	19556.33	19449.23	16597.04	18717	17502.37	20154.64	17585.15	20381.45	18906	0.9900	0.86422
Q8VDQ1	Ptgr2	Prostaglandin reductase 2	29789.23	34069	34152.01	23099.73	30277	27598.23	29794.75	24302.21	27069.15	27191	1.1135	0.31809
Q9Z0N1	Eif2s3x	Eukaryotic translation initiation factor 2 subunit 3, X-	140417.6	141600.7	154796.5	148343.4	146290	151559.1	146951	163056.7	171429.2	158249	0.9244	0.11402
Q80UP8	Slc20a2	Sodium-dependent phosphate transporter 2	10031.44	4817.809	10634.4	9447.611	8733	8836.671	10677.29	10167.84	11873.89	10389	0.8406	0.30257
Q3THG9	Aarsd1	Alanyl-tRNA editing protein Aarsd1	29553.1	32442.78	27236.75	23245.1	28119	25714.4	28157.07	21612.97	23109.1	24648	1.1408	0.20159
Q9R0N7	Syt7	Synaptotagmin-7							47544.39	6141.417		26843	0.0000	
Q8R0N6	Adhfe1	Hydroxyacid-oxoacid transhydrogenase, mitochondrial				2707.629	2708							
P53702	Hccs	Holocytochrome c-type synthase	22617.32	27202.96	41174.62	30145.1	30285	22950.26	35507.46	38060.43	39400.68	33980	0.8913	0.52336
P09813	Apoa2	Apolipoprotein A-II	113056.1	86707.16	107890.9	54632.61	90572	79104.7	84121.28	76591.33	54361.4	73545	1.2315	0.29403
Q6PB70	Ano8	Anoctamin-8	15237.89	15261.59	14536.2	16834.06	15467	12136.36	15100.42	11210.59	13476.65	12981	1.1915	0.04353
Q8R361	Rab11fip5	Rab11 family-interacting protein 5	27262.34	23461.42	23312.13	26750.63	25197	25398.7	26871.29	24952.77	25920.49	25786	0.9772	0.62034
P35486	Pdha1	Pyruvate dehydrogenase E1 component subunit alph	552027.1	570105.6	557816.7	616523.8	574118	571824.9	614224.4	567922.3	588708.2	585670	0.9803	0.54532
Q3UYV9	Ncbp1	Nuclear cap-binding protein subunit 1	13206.28	13812.77	12486.76	12131.09	12909	10465.67	12854.47	10892.71	12733.53	11737	1.0999	0.15558
Q9Z1T1	Ap3b1	AP-3 complex subunit beta-1	13638.91	19209.44	24319.26	14298.06	17866	17940.47	17665.38	19487.01	21511.82	19151	0.9329	0.64333
Q60899	Elavl2	ELAV-like protein 2	214552.5	213308.6	201784.5	261392	222759	230922.7	249571	250222.4	265864.8	249145	0.8941	0.12918
Q9DB20	Atp5po	ATP synthase subunit O, mitochondrial	1020876	1007008	1122338	1199556	1087444	1069531	1083876	1195881	1211104	1140098	0.9538	0.40231
P35585	Ap1m1	AP-1 complex subunit mu-1	92379.95	98909.1	99061.06	95101.38	96363	104161.1	95546.86	105099	114567.2	104844	0.9191	0.09062
O70480	Vamp4	Vesicle-associated membrane protein 4	44583.06	43251.99	35478.06	50329.71	43411	44087.11	46578.72	53569	47699.2	47984	0.9047	0.25790
A2RT62	Fbxl16	F-box/LRR-repeat protein 16	13328.17	13441.19	8675.935	8019.019	10866	13323.31	11080.36	9655.104	8571.914	10658	1.0196	0.91087
P70335	Rock1	Rho-associated protein kinase 1	27751.47	28800.68	27648.85	24933.87	27284	22287.17	24475.85	23605.65	23420.75	23447	1.1636	0.00650
Q9D8V0	Hm13	Minor histocompatibility antigen H13	50752.88	55245.16	56950.41	52107.08	53764	60649.25	54996.25	63646.96	62754.12	60512	0.8885	0.03097
Q9CYW4	Hdhd3	Haloacid dehalogenase-like hydrolase domain-conta	15317.61	19819.5	11772.75	17716.14	16157	8384.229	13701.49	10750.44	18414.61	12813	1.2610	0.27217
Q99LB6	Mat2b	Methionine adenosyltransferase 2 subunit beta	50139.7	55554.71	57963.06	48014.14	52918	54803.19	48663.5	57932.43	52375.53	53444	0.9902	0.86794
Q91XF0	Pnpo	Pyridoxine-5'-phosphate oxidase	11490.55	13711.2	16615.37	13642.22	13865	17296.54	9307.313	16200.37	12906.88	13928	0.9955	0.97690
Q5SRX1	Tom1l2	TOM1-like protein 2	158260.7	162861	155669.1	156958.1	158437	162299.5	165218.8	159158	151088.3	159441	0.9937	0.77936
Q9QUM9	Psma6	Proteasome subunit alpha type-6	189687.5	189224.2	185725.7	174057.9	184674	192366.6	183545.9	213180.1	196407.4	196375	0.9404	0.15543
Q8K353	Cystm1	Cysteine-rich and transmembrane domain-containir	54949.78	51390.77	45684.18	74457.6	56621	60534.57	73643.84	45639.29		59939	0.9446	0.75392
P10126	Eef1a1	Elongation factor 1-alpha 1	713771.3	798099.1	975979.2	711126.4	799744	771009.9	771892.4	846868.6	853931.9	810926	0.9862	0.87139
Q9WTU3	Scn8a	Sodium channel protein type 8 subunit alpha	13689.17	15882.27		7902.208	12491	15947.61	17881.25		17593.99	17141	0.7287	0.13118
Q9JL62	Gltp	Glycolipid transfer protein	98492.86	88723.05	81430	95885.83	91133	94990.7	96165.73	112383.3	90283.72	98456	0.9256	0.27914
Q3TYP4	Tmem88b	Transmembrane protein 88B												
P47955	Rplp1	Large ribosomal subunit protein P1	581643.2	581810.1	714591.3	602318.5	620091	615276	629931.1	627298.1	744688.8	654299	0.9477	0.46619
O55186	Cd59a	CD59A glycoprotein	56461.75	32676.42	24237.29	28456.51	35458	39370.37	43290.48	23953.86	27015.09	33407	1.0614	0.81945
Q80Z24	Negr1	Neuronal growth regulator 1	20249.97	55185.09	22605.3	42245.69	35072	32129.48	19925.2	32544.45	39741.81	31085	1.1282	0.68257

Q61263	Soat1	Sterol O-acyltransferase 1	15501.05	11803.95	10940.15	7712.818	**11489**	10427.08	11935.12	9147.022	11010.44	**10630**	**1.0809**	**0.63194**
Q91YT7	Ythdf2	YTH domain-containing family protein 2	10443.64	7431.318	8128.646	7143.563	**8287**	7783.852	7266.947	8232.37	6478.754	**7440**	**1.1137**	**0.35122**
Q8JZQ2	Afg3l2	AFG3-like protein 2	54952.8	54118.97	54788.16	63144.21	**56751**	53710.02	56108.75	62671.92	63412.09	**58976**	**0.9623**	**0.51507**
Q61627	Grid1	Glutamate receptor ionotropic, delta-1		1554.974			**1555**			2313.378		**2313**	**0.6722**	
Q8R151	Znfx1	NFX1-type zinc finger-containing protein 1	9787.478	9463.449	10621.79	9788.095	**9915**	10315.84	10458.27	5822.972	8991.833	**8897**	**1.1144**	**0.39235**
Q9JIS8	Slc12a4	Solute carrier family 12 member 4	38326.25	37175.62	34869.67	39095.32	**37367**	35424.45	33903.55	54683.37	53554.43	**44391**	**0.8418**	**0.26422**
Q63918	Cavin2	Caveolae-associated protein 2	81286.93	66245.63	60219.88	72967.98	**70180**	66626.68	72121.96	69847.22	60635.09	**67308**	**1.0427**	**0.59841**
Q6NSQ9	G6pc3	Glucose-6-phosphatase 3	37013.11	32445.73	35107.76	36953.43	**35380**	38654.98	37580.87	41547	46677.55	**41115**	**0.8605**	**0.04696**
Q8K010	Oplah	5-oxoprolinase	37624.95	36263.58	34879.2	30673.21	**34860**	32747.8	36132.91	31017.97	29251.55	**32288**	**1.0797**	**0.26666**
Q8VCW8	Acsf2	Medium-chain acyl-CoA ligase ACSF2, mitochondria	81639.08	65812.07	67739.71	78124.85	**73329**	67101.77	82917.74	60567.87	72081.67	**70667**	**1.0377**	**0.67788**
Q78RX3	Smim12	Small integral membrane protein 12	12953.55	15601.12	16233.45	19614.7	**16101**	11089.05	11132.18	17068.18	16512.62	**13951**	**1.1541**	**0.35371**
Q9JKF6	Nectin1	Nectin-1	26046.96	15390.93	11662.81	14656.75	**16939**	11766.46	15134.43	23253.07	11402.22	**15389**	**1.1007**	**0.72325**
Q9D5V6	Syap1	Synapse-associated protein 1	10246.25	19344.06	11803.27	9400.579	**12699**	17383.12	15865.47	17164.29	12130.62	**15636**	**0.8121**	**0.29751**
Q9WUR9	Ak4	Adenylate kinase 4, mitochondrial	11265.63	12910.68	15386.71	12188.89	**12938**	8160.681	10755.58	10176.58	5217.459	**8578**	**1.5083**	**0.02924**
Q99K51	Pls3	Plastin-3	125292.9	125386.2	97588.08	90703.8	**109743**	115691.7	113602.8	91340.22	90466.47	**102775**	**1.0678**	**0.56393**
Q9JKF7	Mrpl39	Large ribosomal subunit protein mL39	11634.36	11615.49	13724.47	12562.61	**12384**	12016.55	12038.13	15104.98	14329.19	**13372**	**0.9261**	**0.33190**
Q9D7X3	Dusp3	Dual specificity protein phosphatase 3	76575.69	74205.36	79609.73	62560.16	**73238**	70529.83	65827.48	58684.49	66989.43	**65508**	**1.1180**	**0.13515**
O55234	Psmb5	Proteasome subunit beta type-5	126222.7	132214.9	140873.2	125620.2	**131233**	133814	137697	142771.7	139325.6	**138402**	**0.9482**	**0.12325**
Q9Z0H3	Smarcb1	SWI/SNF-related matrix-associated actin-dependent	63979.27	63068.35	79435.38	66032.02	**68129**	70675.34	64343.13	65587.11	80902.3	**70377**	**0.9681**	**0.68972**
P36552	Cpox	Oxygen-dependent coproporphyrinogen-III oxidase, r	21207.85	20927.25	20836.79	20875.89	**20962**	20052.56	21476.64	19239.55	21530.28	**20575**	**1.0188**	**0.52063**
Q6GV12	Kdsr	3-ketodihydrosphingosine reductase	38326.94	38806.04	38275.32	41835.16	**39311**	37345.78	43284.46	33900.5	35680.84	**37553**	**1.0468**	**0.45591**
Q8BPN8	Dmxl2	DmX-like protein 2	44229.29	41773.99	39879.39	43617.55	**42375**	43489.79	45012.86	37946.21	41557.96	**42002**	**1.0089**	**0.84376**
P70699	Gaa	Lysosomal alpha-glucosidase	55479.34	55660.62	65987.77	64608.16	**60434**	60997.32	56603.45	65539.34	70064.86	**63301**	**0.9547**	**0.50521**
Q60930	Vdac2	Voltage-dependent anion-selective channel protein 2	663681.8	676596	767544.7	736943.3	**711191**	669997.5	777409.1	704065.6	789261.3	**735183**	**0.9674**	**0.54985**
Q9DCT2	Ndufs3	NADH dehydrogenase [ubiquinone] iron-sulfur prote	398634.3	338137.7	390033.5	416907.6	**385928**	385701.6	384038.8	418389.8	421258.6	**402347**	**0.9592**	**0.43614**
Q9R1R2	Trim3	Tripartite motif-containing protein 3	39246.5	35983.71	38921.53	39206.71	**38340**	40250.95	38446.61	32403.29	38159.18	**37315**	**1.0275**	**0.60452**
Q8JZS0	Lin7a	Protein lin-7 homolog A	78457.27	84873.3	87416.62	105839.9	**89147**	91775.38	87515.73	105986.8	101363.2	**96660**	**0.9223**	**0.34000**
Q8BGN2	D3Ertd751e	UPF0462 protein C4orf33 homolog	20809.27	19903.51	18826.75		**19847**	18518.57	15164.22	11308.47		**14997**	**1.3234**	**0.08817**
Q6NVF9	Cpsf6	Cleavage and polyadenylation specificity factor subu	27196.75	26805.96	33011.12	31488.57	**29626**	27747.89	28327.49	27526.6	31170.97	**28693**	**1.0325**	**0.61595**
Q9CYG7	Tomm34	Mitochondrial import receptor subunit TOM34	64664.87	68325.27	59973.96	60980.06	**63486**	74296.38	67654.62	70238.46	64086.96	**69088**	**0.9189**	**0.09788**
Q9Z2D1	Mtmr2	Myotubularin-related protein 2	50364.5	49279.12	49316.49	54444.38	**50851**	47203.33	47599.77	43915.45	52333.22	**47763**	**1.0647**	**0.19572**
Q8BHL5	Elmo2	Engulfment and cell motility protein 2	36774.4	30841.14	35315.31	31400.09	**33583**	32290.56	31805.82	30913.91	33914.92	**32231**	**1.0419**	**0.42713**
A2ASS6	Ttn	Titin	264825.3	252386.8	266786.7	362336.3	**286584**	179915.4	220920.3	282114.5	235069.3	**229505**	**1.2487**	**0.13487**
Q02013	Aqp1	Aquaporin-1	95102.63	96757.42	92987.82	101567.7	**96604**	99338.15	93105.3	102889.7	100542	**98969**	**0.9761**	**0.42668**
Q8VDT9	Mrpl50	Large ribosomal subunit protein mL50	23553.04	21774.38	26542.81	30345.48	**25554**	23561.41	27083.81	31736.78	37547.95	**29982**	**0.8523**	**0.26003**
Q9EQI8	Mrpl46	Large ribosomal subunit protein mL46	17126.15	16639.87	18531.84	19717.88	**18004**	17233.76	18186.03	19160.36	18543.94	**18281**	**0.9848**	**0.74274**
Q3V1L4	Nt5c2	Cytosolic purine 5'-nucleotidase	33667.96	33180.02	30782.95	29395.94	**31757**	30532.26	21830.33	21125.09	22164.13	**23913**	**1.3280**	**0.01813**
Q8K0G5	Eipr1	EARP and GARP complex-interacting protein 1	45721.61	43566.52	40763.65	44503.75	**43639**	51061.34	51209.21	44477.95	43091.31	**47460**	**0.9195**	**0.16051**
Q8VE95		UPF0598 protein C8orf82 homolog	11808.6	9471.633	9587.149	10889.72	**10439**	13196.9	12274.95	10930.18	9420.253	**11456**	**0.9113**	**0.34613**
Q08642	Padi2	Protein-arginine deiminase type-2	44412.43	53830.86	34555.07	41733.92	**43633**	40026.96	48624.92	30666.66	36943.71	**39066**	**1.1169**	**0.43504**
Q9CR39	Wdr45b	WD repeat domain phosphoinositide-interacting pro	39918.73	37934.65	38582.97	30530.58	**36742**	36827.72	43951.88	28518.43	38476.55	**36944**	**0.9945**	**0.95966**
Q9CS84	Nrxn1	Neurexin-1	9215.402	19376.53	7404.479		**11999**	12448.22		16273.47	9223.51	**12648**	**0.9486**	**0.88583**
Q6P6J9	Txndc15	Thioredoxin domain-containing protein 15	15513.62	20058.03	19517.57	22787.16	**19469**	19563	17614.07	22718.97	23530.88	**20857**	**0.9335**	**0.52122**
P03899	mt-Nd3	NADH-ubiquinone oxidoreductase chain 3	112743.1	134719	111548.8	138000.5	**124253**	119687.5	157355.3	138016.2	142577.7	**139409**	**0.8913**	**0.19788**
Q99LE6	Abcf2	ATP-binding cassette sub-family F member 2	67485.55	65384.5	64595.77	69075.29	**66635**	68759.43	71002.71	70134.97	66896.05	**69198**	**0.9630**	**0.10739**
Q8BGX2	Timm29	Mitochondrial import inner membrane translocase s	16405.3	14440.69	10917.61	11378.42	**13286**	15239.57	9108.498	10335.54	13618.83	**12076**	**1.1002**	**0.55312**
Q6ZWY3	Rps27l	Small ribosomal subunit protein eS27-like	185126.9	163080.9	210331.8	227242	**196445**	200487.7	196262	213128.3	244809.5	**213672**	**0.9194**	**0.37212**
Q60803	Traf3	TNF receptor-associated factor 3												
Q60932	Vdac1	Voltage-dependent anion-selective channel protein 1	826189	842872.1	886101.8	981507.3	**884168**	836194.3	929602.3	950700.4	981512.1	**924502**	**0.9564**	**0.42207**
P60487	Pdxp	Chronophin	17582.27	25228.12	24301.33	11894.73	**19752**	23305.44	21647.99	17944.9	20133.55	**20758**	**0.9515**	**0.77240**
P56395	Cyb5a	Cytochrome b5	121380.8	127000.9	132164.6	130618.6	**127791**	102975.2	127188.9	111433.9	114617.4	**114054**	**1.1204**	**0.04848**
Q9CQ69	Uqcrq	Cytochrome b-c1 complex subunit 8	293798.5	307953.3	320870.6	374431	**324263**	322413.1	338404.2	378878.5	354501.2	**348549**	**0.9303**	**0.29845**
Q60598	Cttn	Src substrate cortactin	122818.3	131858.1	125528.1	129373.8	**127395**	131930.5	132380.6	148327.3	142619.4	**138814**	**0.9177**	**0.04391**
Q9R0E1	Plod3	Multifunctional procollagen lysine hydroxylase and g	16714.72	19284.52	20912.21	12497.47	**17352**	15917.36	18662.32	17354.45	19126.29	**17765**	**0.9768**	**0.84102**
Q05909	Ptprg	Receptor-type tyrosine-protein phosphatase gamma		23500.4		19251.46	**21376**	12558.12	14825.17	15191.33	12116.19	**13673**	**1.5634**	**0.01167**
Q3TWN3	Cnnm2	Metal transporter CNNM2												
P68181	Prkacb	cAMP-dependent protein kinase catalytic subunit be	51587.57	41754.3	36524.13	36673.66	**41635**	40961.36	46073.52	40422.09	36830.85	**41072**	**1.0137**	**0.89302**
Q91Z49	Fyttd1	UAP56-interacting factor	35076.11	35256.53	35623.56	42728.63	**37171**	38852.85	32698.14	43535.91	45980.23	**40267**	**0.9231**	**0.40586**
Q8CDG3	Vcpip1	Deubiquitinating protein VCPIP1	24023.24	23433.59	24393.09	20583.09	**23108**	22588.97	25244.48	21190.9	23559.13	**23146**	**0.9984**	**0.97628**

Q91WC0	Setd3	Actin-histidine N-methyltransferase	45010.84	44824.75	46541.3	42484.01	44715	44805.81	41323.89	44975.3	43694.84	43700	1.0232	0.42520
Q7TNC4	Luc7l2	Putative RNA-binding protein Luc7-like 2	22857.85	22396.14	25275.91	24970.49	23875	24641.58	25241.65	27717.38	31642.31	27311	0.8742	0.09714
Q3UNH4	Gprin1	G protein-regulated inducer of neurite outgrowth 1	22292.84		23615.8		22954							
Q6QD59	Bnip1	Vesicle transport protein SEC20	22465.51	18175.9	25438.31	27096.44	23294	22361.25	22934.72	26495.63	31763.38	25889	0.8998	0.40772
O08746	Matn2	Matrilin-2	56590.95	52256.79	75343.03	56821.43	60253	49474.45	45650.41	75727.3	78091.65	62236	0.9681	0.84863
Q99JX4	Eif3m	Eukaryotic translation initiation factor 3 subunit M	65538.52	65711.17	61186.67	64439.1	64219	63145.15	66030.95	62134.55	68558.92	64967	0.9885	0.69089
Q9WVA3	Bub3	Mitotic checkpoint protein BUB3	48492.8	47704.25	56134.55	49755.9	50522	51568.16	49236.98	56192.82	56800.16	53450	0.9452	0.31135
Q9JLJ0	Litaf	Lipopolysaccharide-induced tumor necrosis factor-a	46385.19	53022.63	43873.87	62431.04	51428	48725.19	46122.43	43300.09	57465.13	48903	1.0516	0.64150
Q9DC28	Csnk1d	Casein kinase I isoform delta	47907.97	54006.9	47802.06	55691.24	51352	54674.49	54003.88	57435.2	54793.95	55227	0.9298	0.12630
Q8CGU1	Calcoco1	Calcium-binding and coiled-coil domain-containing p	25149.08	27175.01	22660.14	27172.29	25539	21602.33	28129.28	18050.08	21083.94	22216	1.1496	0.21154
Q8BYK6	Ythdf3	YTH domain-containing family protein 3	65513.75	65872.96	62863.49	88670.42	70730	51508.49	69339.33	72687.02	74084.61	66905	1.0572	0.64830
P03911	Mtnd4	NADH-ubiquinone oxidoreductase chain 4	27012.63	21053.75	19996.66	23496.49	22890	19893.39	23066.78	32848.49	22386.37	24549	0.9324	0.62770
Q91V92	Acly	ATP-citrate synthase	1021003	1001633	884691.7	927420.3	958687	1066978	981055.7	800349.1	829957.3	919585	1.0425	0.60006
Q91YQ5	Rpn1	Dolichyl-diphosphooligosaccharide--protein glycosy	288526	299278.6	313913.7	318124.2	304961	295856.8	318183.5	336544.7	334959	321386	0.9489	0.20862
P51174	Acadl	Long-chain specific acyl-CoA dehydrogenase, mitocl	276161.2	284188.3	269131.7	311449.9	285233	267205.1	322966.3	274722.6	273885.6	284695	1.0019	0.97404
Q8BWT1	Acaa2	3-ketoacyl-CoA thiolase, mitochondrial	395306.2	393330.2	406330.7	477261.6	418057	408207.5	424438.5	417469.5	405636.7	413938	1.0100	0.84669
Q80W22	Thnsl2	Threonine synthase-like 2	10527.18	7666.094	8681.899	7412.998	8572	6875.226	7868.468		4672.28	6472	1.3245	0.12759
P59644	Inpp5j	Phosphatidylinositol 4,5-bisphosphate 5-phosphata	4706.497	4958.988	7807.05	9156.514	6657	6643.675	6611.198	5245.085	6067.102	6142	1.0839	0.66647
P50247	Ahcy	Adenosylhomocysteinase	524173.7	541249.8	639810.4	481368.8	546651	516911.3	503759.1	605416.1	576719.1	550701	0.9926	0.92510
Q91WA3	Hdac11	Histone deacetylase 11	22068.65	24854.35	22313.84	22228.53	22866	20792.46	22616.84	27723.12	15696.12	21707	1.0534	0.66785
P70195	Psmb7	Proteasome subunit beta type-7	47110.53	48981.53	53636.41	48753.84	49621	59252.92	50622.76	62718.38	51538.55	56033	0.8856	0.09735
P01831	Thy1	Thy-1 membrane glycoprotein	551202.7	496958.8	516261.8	678316.4	560685	586196.9	619066.8	556088.6	573508.1	583715	0.9605	0.61069
Q9WU40	Lemd3	Inner nuclear membrane protein Man1	8618.763	13157.02	9504.562	11502.22	10696	12104.26	9764.684	6726.265	11144.34	9935	1.0766	0.64158
P07759	Serpina3k	Serine protease inhibitor A3K	89954.52	163851.5	412062.1	145784.5	202913	105997.3	76134.14	211814.7	204912.7	149715	1.3553	0.52744
Q3TWW8	Srsf6	Serine/arginine-rich splicing factor 6	87038.88	94879.84	105360.7	109990.9	99318	96075.24	91521.84	96928.56	101838	96591	1.0282	0.64281
Q8K097	Faim2	Protein lifeguard 2												
Q64519	Sdc3	Syndecan-3												
Q04750	Top1	DNA topoisomerase 1	33283.22	31919.2	39375.87	35826.55	35101	31634.79	33827.53	38178.86	41625.24	36317	0.9665	0.67605
Q91WL8	Wwox	WW domain-containing oxidoreductase		6943.559	5099.807	7960.59	6668	8366.192	6957.591	8375.42	6588.725	7572	0.8806	0.35756
Q99MR3	Slc12a9	Solute carrier family 12 member 9	13860.37	14108.91	17637.64	17584.64	15798	13350.9	13252.54	18568.64	20065.23	16309	0.9686	0.81142
P99029	Prdx5	Peroxiredoxin-5, mitochondrial	714912.4	751772.3	747725.6	732324.9	736684	762702.7	705198	775372.6	751569.3	748711	0.9839	0.51626
Q3TWI9	Tmem63b	CSC1-like protein 2	61764.27	59725.08	62829.7	65256.38	62394	62268.02	66441.53	76050.34	79593.97	71088	0.8777	0.08420
P50580	Pa2g4	Proliferation-associated protein 2G4	132119.2	135113.6	151496.2	138618.7	139337	138306	147680.7	157676	162250.6	151478	0.9198	0.12603
Q9JL26	Fmnl1	Formin-like protein 1	80621.38	76892.28	43588.45	51134	63059	51609.54	74979.38	60766.14	46896.77	58563	1.0768	0.69962
Q9CY34	Ube2f	NEDD8-conjugating enzyme UBE2F	18181.62	14766.23	15531.44		16160	10229.25	11189.5		15722.74	12380	1.3053	0.12965
Q9QXJ1	Apbb1	Amyloid beta precursor protein binding family B men	14864.17	12345.61	13505.62	11196.28	12978	13809.72	16053.22	11006.2	11799.34	13167	0.9856	0.89507
Q8CBW3	Abi1	Abl interactor 1	34674.59	31829.59	33179.24	33210.09	33223	30196.13	31764.4	38966.73	35509.43	34109	0.9740	0.68073
O55242	Sigmar1	Sigma non-opioid intracellular receptor 1	35426.21	37358.71	41701.41	39361.53	38462	40255.61	45235.56	49861.86	50477.9	46458	0.8279	0.02633
Q9D8V7	Sec11c	Signal peptidase complex catalytic subunit SEC11C	19313.83	20049.43	21268.48	21031	20416	18572.82	21607.95	18028.45	18851.59	19265	1.0597	0.25695
Q9R1C7	Prpf40a	Pre-mRNA-processing factor 40 homolog A	42549.68	44267.97	34771.38	39835.59	40356	36836.38	42022.48	37671.78	38657.67	38797	1.0402	0.53418
Q9DBR7	Ppp1r12a	Protein phosphatase 1 regulatory subunit 12A	35682.82	34201.49	38555.03	36725.69	36291	34423.82	33488.52	43443.54	38161.24	37379	0.9709	0.67102
Q9JLT4	Txnrd2	Thioredoxin reductase 2, mitochondrial	24746.44	24822.78	21597.04	31869.1	25759	26022.38	28018.83	26004.08	25583.38	26407	0.9754	0.78187
Q8VE37	Rcc1	Regulator of chromosome condensation	58554.43	58250.32	63476.5	65476.5	61439	61607.22	60112.95	61775.02	66747.89	62561	0.9821	0.64449
Q64674	Srm	Spermidine synthase	86300.8	95975.8	104124.5	69410.6	88953	89504.41	79458.54	92738.41	82341.52	86011	1.0342	0.72806
O08586	Pten	Phosphatidylinositol 3,4,5-trisphosphate 3-phospha	34870.49	42750.68	34150.4	39483.07	37814	39608.98	40305.74	33642.67	34440.7	37000	1.0220	0.76975
Q6PGB8	Smarca1	Probable global transcription activator SNF2L1	34283.17	33391.46	29640.42	38826.3	34035	27747.92	32282.72	28405.35	32478.62	30229	1.1259	0.14367
P13707	Gpd1	Glycerol-3-phosphate dehydrogenase [NAD(+)], cytc	163466.5	194502.4	225376.8	162494.6	186460	164918.6	184415.3	192351.8	207379	187266	0.9957	0.96449
A2A935	Prdm16	Histone-lysine N-methyltransferase PRDM16	363260.8	399796.8	505197.3	329747.2	399501	392746.9	377124.2	414058.9	427543.5	402868	0.9916	0.93505
Q8CCX5	Krt222	Keratin-like protein KRT222	51447.5	42899.81	30832.39	52916.41	44524	46434.13	49554.45	25704.92	34395.59	39022	1.1410	0.49028
Q8VBX6	Mpdz	Multiple PDZ domain protein	20848	20032.04	22541.71	21478.75	21225	21257.78	17252.12	18934.41	24884.91	20582	1.0312	0.72379
Q9Z2U0	Psma7	Proteasome subunit alpha type-7	180605.3	180069.4	178962.8	164658.4	176074	172195.4	175387.7	175203	174841.5	174407	1.0096	0.68340
Q9Z2Y8	Plpbp	Pyridoxal phosphate homeostasis protein	55151.32	53707.34	49202.37	43422.96	50371	51440.48	49953.29	51016.05	44823.3	49308	1.0216	0.73940
Q6RHR9	Magi1	Membrane-associated guanylate kinase, WW and PD	23668.09	20225.69	18407.73	18912.56	20304	22078.09	21558.1	21697.32	19463.37	21199	0.9577	0.52375
Q99JF5	Mvd	Diphosphomevalonate decarboxylase	30181.06	30380.41	47866.6	25002.74	33358	34091.02	25341.12	43418.9	42474.27	36331	0.9182	0.66526
Q9CU62	Smc1a	Structural maintece of chromosomes protein 1A	47244.07	47521.42	48045.33	51451.11	48565	46849.9	45442.68	47955.65	56745.54	49248	0.9861	0.81092
Q9CYH5	Gfod2	Glucose-fructose oxidoreductase domain-containing	23843.46	23145.17	25434.24	28910.37	25333	24489.26	24764.53	21050.14	21479.93	22946	1.1040	0.18950
Q920A5	Scpep1	Retinoid-inducible serine carboxypeptidase	31674.65	36345.51	36188.31	30811.99	33755	31715.23	33282.45	31987.56	32393.22	32345	1.0436	0.38356
O55126	Nipsnap2	Protein NipSnap homolog 2	177533.8	174423	175924.9	199435.7	181829	173034	176539.3	155755	165988.6	167829	1.0834	0.11018
O54784	Dapk3	Death-associated protein kinase 3	9439.653	6673.387	10475.38	13725.32	10078	9452.592	12022.38	4465.6	6945.115	8221	1.2259	0.42748

Q62393	Tpd52	Tumor protein D52	96880.77	97726.72	96015.39	85186.2	93952	89641.71	98587.85	87618.43	93225.52	92268	1.0182	0.67322
P70429	Evl	Ena/VASP-like protein	17169.45	13813.53	13529.16	13075.21	14397	11444.45	14256.42	17239.55	9428.605	13092	1.0996	0.52657
Q8BIZ1	Anks1b	Ankyrin repeat and sterile alpha motif domain-containing protein 1B												
Q8K003	Tma7	Translation machinery-associated protein 7	10486.5	11512.28	10279.64	11276.24	10889	9160.937		11914.29	6729.297	9268	1.1748	0.26844
Q9Z127	Slc7a5	Large neutral amino acids transporter small subunit	88645.22	110720.5	66032.11	110526	93981	75008.03	92014.73	84535.02	79846.83	82851	1.1343	0.36103
P61965	Wdr5	WD repeat-containing protein 5	34833.88	33552.43	37709.11	39634.97	36433	34832.57	31361.24	35268.36	37795.15	34814	1.0465	0.42922
Q69ZW3	Ehbp1	EH domain-binding protein 1	35016.09	30582.28	36390.48	36254.02	34561	27726.4	29778.54	33132.3	36831.75	31867	1.0845	0.30745
Q91W50	Csde1	Cold shock domain-containing protein E1	87495.63	87666.13	90130.35	91251.98	89136	92934.5	93597.59	100544.3	97339.89	96104	0.9275	0.01301
Q9DB05	Napa	Alpha-soluble NSF attachment protein	163312.6	159392.8	133924.6	150606.8	151809	154594.3	156868.6	144627.5	138767.7	148715	1.0208	0.70485
Q9D7N6	Mrpl30	Large ribosomal subunit protein uL30m	6738.61	9311.116	11561.26	8476.869	9022	7459.155	5326.408	10190.23	9309.221	8071	1.1178	0.54206
Q811D0	Dlg1	Disks large homolog 1	59945.51	61055.26	69840.3	72251.43	65773	62570.39	59005.24	70959.91	75204.99	66935	0.9826	0.81830
Q8R5J9	Arl6ip5	PRA1 family protein 3	130383.6	130271	141272.8	149872.5	137950	119734.9	129233.4	160656.7	155522.9	141287	0.9764	0.77224
Q9JLJ2	Aldh9a1	4-trimethylaminobutyraldehyde dehydrogenase	138533.1	141503.9	134000.8	119889.6	133482	134483.1	127025.6	117736.7	120421.6	124917	1.0686	0.20813
Q8CA72	Gan	Gigaxonin	19013.96	16267.29	15882.51	14766.96	16483	15806.12	17101.5	15268.86	16406.04	16146	1.0209	0.74368
P58252	Eef2	Elongation factor 2	734847	803730.5	929462.4	694046.3	790522	782545.4	728841.4	843493.1	825916.4	795199	0.9941	0.93785
A3KFX0	Nt5c1a	Cytosolic 5'-nucleotidase 1A							2350.876			2351	0.0000	
Q9D8X1	Cutc	Copper homeostasis protein cutC homolog				1520.155	1520							
P35979	Rpl12	Large ribosomal subunit protein uL11	494875.8	491615.1	509743.9	553474.4	512427	490942	533380.3	546460.2	564413.8	533799	0.9600	0.35126
Q99M28	Rnps1	RNA-binding protein with serine-rich domain 1	60982.26	62269.1	55783.71	67750.1	61696	48813.37	54659.19	54591.05	59174.17	54309	1.1360	0.06324
Q8K199	Cmc2	COX assembly mitochondrial protein 2 homolog				522.1578	522							
P21836	Ache	Acetylcholinesterase	22185.38	13324.48	16692.83	18263.12	17616	15558.59	15305.1	14168.43	14897.44	14982	1.1758	0.20724
Q921F2	Tardbp	TAR DNA-binding protein 43	259490	252407.2	275293	291508.3	269675	264476.1	276940.4	284106.3	299702	281306	0.9587	0.34683
Q99KR3	Lactb2	Endoribonuclease LACTB2	55994.74	52612.48	54590.17	46641.71	52460	57426.03	52617.49	52028.34	54322.02	54098	0.9697	0.51843
P28063	Psmb8	Proteasome subunit beta type-8	89043.68	69511.34	64663.24	59684.54	70726	68615.23	70496.78	68952.04	69016.11	69270	1.0210	0.82869
Q8K2J7	Rell1	RELT-like protein 1	18034.46	14576.81	12382.24	15490.35	15121	12707.07	15243.2	17254.3	18567.44	15943	0.9484	0.65181
P97494	Gclc	Glutamate--cysteine ligase catalytic subunit	34479.59	39234.5	28641.89	22742.48	31275	31053.39	30979.25	20298.95	24723.39	26764	1.1685	0.34784
Q78J03	Msrb2	Methionine-R-sulfoxide reductase B2, mitochondrial	31966.85	33489.91	29901.46	41537.37	34224	32348.95	38724.23	44264.84	41282.12	39155	0.8741	0.21906
P36536	Sar1a	GTP-binding protein SAR1a	101856	99985.7	121305	87140.32	102572	101465.8	89313.79	95706.21	105672.8	98040	1.0462	0.58677
Q7TMY8	Huwe1	E3 ubiquitin-protein ligase HUWE1	109952.5	103891.1	104895.5	101697.3	105109	107542.2	107356.8	110522.9	103768.4	107298	0.9796	0.36384
Q9WVA4	Tagln2	Transgelin-2	181888.3	207697.9	219846.2	182055.9	197872	140240.8	224486.8	158907.6	141791	166357	1.1894	0.20187
Q91ZZ3	Sncb	Beta-synuclein	56681.09	51745.22	53767.06	35035.05	49307	52456.63	48891.42	55721.29	46153.52	50806	0.9705	0.78656
G5E8V9	Arfip1	Arfaptin-1	24435.75	21826.59	25807.75	18692.56	22691	19920.31	23469.75	25073.45	27420.46	23971	0.9466	0.58548
Q8VE97	Srsf4	Serine/arginine-rich splicing factor 4	218841	198972.3	216373.5	243069.8	219314	258166.1	240413	296240.8	254733.2	262388	0.8358	0.02822
Q8BHH2	Rab9b	Ras-related protein Rab-9B	39416.48	55765.28	47933.23	55165.68	49570	51071.3	48660.59	45535.15	49453.53	48680	1.0183	0.83116
Q3TB82	Plekhf1	Pleckstrin homology domain-containing family F mem	8327.676	14802.68	18259.24	9831.52	12805	15713.64	13613.74	7041.285	10411.68	11695	1.0949	0.72129
P13020	Gsn	Gelsolin	500996.9	553179.3	576563.9	474111.4	526213	532749.6	482980	547486.8	508300.9	517879	1.0161	0.77143
P39688	Fyn	Tyrosine-protein kinase Fyn	34385.03	32042.63	32623.65	36738.7	33948	30177.61	36510.71	31282.41	32994.84	32741	1.0368	0.51416
P14115	Rpl27a	Large ribosomal subunit protein uL15	1004235	903572.5	985498.3	1128251	1005389	1003067	1019628	1213382	1258343	1123605	0.8948	0.19148
Q8BJ71	Nup93	Nuclear pore complex protein Nup93	38612.21	37067.94	37241.96	38770.43	37923	40214.78	34731.42	38266.46	38604.58	37954	0.9992	0.98073
Q03265	Atp5f1a	ATP synthase subunit alpha, mitochondrial	2185989	2096856	2460482	2601441	2336192	2235627	2292099	2492022	2549607	2392339	0.9765	0.70211
Q9CRC6	Borcs7	BLOC-1-related complex subunit 7	17324.6	16489.29	17897.81	15980.33	16923	18756.34	16195.88	19598.69	16716.78	17817	0.9498	0.36714
Q91V36	Nrbp2	Nuclear receptor-binding protein 2	34312.64	40521.09	39212.01	33252.16	36824	35896.52	36860.71	34042.43	35227.14	35507	1.0371	0.51070
Q91VU6	Dcaf11	DDB1- and CUL4-associated factor 11	884.9177	555.568	996.9413	827.1721	816			2462.355	2268.538	2365	0.3450	0.00053
P28798	Grn	Progranulin	20579.91	20303.91	20056.97	19822.73	20191	22039.91	24138.6	22687.95	23223.09	23022	0.8770	0.00097
Q9ER64	Osbpl5	Oxysterol-binding protein-related protein 5	25057.01	24554.47	24411.82	25553.63	24894	23027.1	23400.06	22852.46	23442.66	23181	1.0739	0.00118
Q8VEB4	Pla2g15	Phospholipase A2 group XV	24535.34	24980.76	24664.8	23867.57	24512	27825.56	25701.83	27699.62	27972.51	27300	0.8979	0.00310
Q9D8C4	Ifi35	Interferon-induced 35 kDa protein homolog	7733.51	5930.327	7183.008	5523.21	6593	4285.681	4414.139	4077.857	3912.16	4172	1.5800	0.00385
Q8C838	Trarg1	Trafficking regulator of GLUT4 1	19079.99	20324.85	21287.79	21813.05	20626	24889.88	23074.27	25607.02	26863.62	25109	0.8215	0.00405
Q3URQ7	Mthfsd	Methenyltetrahydrofolate synthase domain-containi	6680.892	7855.286	6612.337	7436.504	7146	6020.035	4523.695	4980.521	5333.921	5215	1.3704	0.00443
Q80WV3	Chst2	Carbohydrate sulfotransferase 2	1309.797	2107.951	3930.87	1902.092	2313			7655.739	6865.889	7261	0.3185	0.00495
Q9D032	Ssbp3	Single-stranded DNA-binding protein 3	19343.35	18230.08	13495.16	16178.8	16812	20995.04	23234.43	22411.29	22701.48	22336	0.7527	0.00690
Q9JKS4	Ldb3	LIM domain-binding protein 3	12507.14	9544.081	8482.963	11657.75	10548		3737.75		2858.704	3298	3.1981	0.00693
Q8R015	Bloc1s5	Biogenesis of lysosome-related organelles complex	13576.89	11639.54	12288.19	11577.82	12271	9765.729	10730.83	9149.139	10228.6	9969	1.2309	0.00700
Q8BXN9	Tmem87a	Transmembrane protein 87A	8174.777	10092.19	8671.219	7440.893	8595	11549.46	12359.9	10147.33	11661.3	11429	0.7520	0.00797
Q91WJ7	Spats2l	SPATS2-like protein	18720.72	17483.39	14642.72	18688.69	17384	10017.34	14106.29	10540.5	13566.8	12058	1.4417	0.00929
P35441	Thbs1	**Thrombospondin-1**	30365.57	30262.72	34620.01	29752.43	31250	23468.45	28460.15	24006.58	21720.71	24414	1.2800	0.00960
O88990	Actn3	Alpha-actinin-3	27567.52	17603.87	20284.19	38508.96	25991	8287.189	10170.08	2846.127	10377.28	7920	3.2816	0.01110
P29788	Vtn	Vitronectin	48462.8	43642.03	45344.77	54483.1	47983	37911.11	40763.4	38848.63	38942.3	39116	1.2267	0.01128
Q05117	Acp5	Tartrate-resistant acid phosphatase type 5	15293.16	12147.43	14404.48	11168.2	13253	5750.318	10386.56	7139.833	9336.658	8153	1.6255	0.01153

P20801	Tnnc2	Troponin C, skeletal muscle	33198.07	25183.38	16266.35	27314.63	25491	7532.04	17003.86	9576.116	9716.879	10957	2.3264	0.01188
P31266	Rbpj	Recombining binding protein suppressor of hairless	8693.597	6740.44	7455.459	8330.962	7805	4762.391		2402.939		3583	2.1786	0.01249
E9Q1P8	Irf2bp2	Interferon regulatory factor 2-binding protein 2	9027.035		10305.79	10229.67	9854	6524.506		7060.183		6792	1.4508	0.01268
P13541	Myh3	Myosin-3	21318.47	13555.13	14266.59	13329	15617	7004.251	10705.33	3.95024	4308.641	5506	2.8366	0.01412
Q8VC65	Nrm	Nurim	14551.67	15137.8	15329.28	11226.87	14061	8862.724	6356.588	12239.17	6999.898	8615	1.6323	0.01564
P59900	Emilin3	EMILIN-3	10196.06	9486.093		8840.59	9508	7414.654	7358.476	4353.877	5614.646	6185	1.5371	0.01631
Q5SX40	Myh1	Myosin-1	60326.13	74436.97	53113.46	98230.42	71527	31852.62	50153.1	19580.79	28463.66	32513	2.2000	0.01649
Q62147	Sspn	Sarcospan	25470.9	22580.56	22120.21	25319.92	23873	29809.34	27787.59	27089.41	26185.48	27718	0.8613	0.01687
Q9Z1T2	Thbs4	**Thrombospondin-4**	29093.18	27976.2	25518.59	22888.65	26369	21911.38	22807.91	19210.73	16791.27	20180	1.3067	0.01884
Q8K2Y0	Obi1	ORC ubiquitin ligase 1		895.9513	883.6301		890	243.7205		401.8645		323	2.7565	0.01901
Q8K013	Gtpbp10	GTP-binding protein 10		7250.704	9455.685	7235.935	7981		11246.55	13687.76	15750.06	13561	0.5885	0.02029
Q99JP6	Homer3	Homer protein homolog 3		6811.695	7314.308	6825.831	6984			5254.344	3959.06	4607	1.5160	0.02044
Q3TBT3	Sting1	Stimulator of interferon genes protein	4732.305	7130.71	6999.318	4568.56	5858		2216.346	1581.712		1899	3.0846	0.02055
A2BH40	Arid1a	AT-rich interactive domain-containing protein 1A	10879.44	8412.732	8451.164	8725.883	9117	5786.128	6329.873	6088.706	8255.719	6615	1.3783	0.02175
Q8R4H9	Slc30a5	Proton-coupled zinc antiporter SLC30A5	10654.52	9940.839	9735.128	9644.09	9994	11942.16	11184.15	14691.08	11991.9	12452	0.8026	0.02208
Q9QZ47	Tnnt3	Troponin T, fast skeletal muscle	73273.5	50986.34	45194.06	110170.5	69906	24509.69	31035.94	9352.508	26945.06	22961	3.0446	0.02293
P27005	S100a8	Protein S100-A8	44475.11	25503.73	26467.45	41315.46	34440	12280.17	25340.91	17639.87	12576.08	16959	2.0308	0.02353
Q9JK37	Myoz1	Myozenin-1	17273.1	8666.511	10369.84	18870.89	13795	4321.586	5278.33		2973.613	4191	3.2915	0.02476
Q8K3H5	Myo3a	Myosin-IIIa	17277.46	16386.32	15988.57	17107.82	16690	15556.45	14334.22	10184.61	12072.51	13037	1.2802	0.02508
O88693	Ugcg	Ceramide glucosyltransferase	5677.363	5084.253	3856.456	6824.982	5361	6997.549	10396.3	9452.508	7083.987	8483	0.6320	0.02528
Q64261	Cdk6	Cyclin-dependent kinase 6	5485.256	6792.658	8583.881	9651.847	7628	2909.642	4669.79	5878.736	3939.509	4349	1.7539	0.02613
P13412	Tnni2	Troponin I, fast skeletal muscle	65415.51	34398.37	38579.94	78051.48	54111	23202.45	25281.31	7595.809	26850.81	20733	2.6100	0.02662
Q9JKN1	Slc30a7	Zinc transporter 7	17630.89	15396.67	15024.46	13599.06	15413	17444.02	19445.48	17430.86	18593.27	18228	0.8455	0.02694
Q9JMF7	Dolpp1	Dolichyldiphosphatase 1	24200.65	30939.7	25894.74	27932.24	27242	29330.86	34193.48	33201.31	37149.59	33469	0.8139	0.02842
P97314	Csrp2	Cysteine and glycine-rich protein 2	21996.41	22308.05	21214.62		21840	29442.96	23033.72	27162.64	28846.23	27121	0.8053	0.02844
P42925	Pxmp2	Peroxisomal membrane protein 2	1439.632	590.0884	1402.523	1193.324	1156	1664.063	2010.861		2211.648	1962	0.5893	0.02993
Q8C0X8		Sperm motility kinase X	940.0978	2443.648	2398.588	2438.023	2055	887.1137	990.7928	921.313	1157.648	989	2.0775	0.02996
P55284	Cdh5	Cadherin-5	24722.65	20394.29	22694.85	22592.37	22601	20418.35	20107.84	18018.73	15435.31	18495	1.2220	0.02998
Q8BNE1	Tcaf1	TRPM8 channel-associated factor 1	23928.51	24758.79	23682.36	23650.6	24005	21260.85	23414.26	20994.68	22941.7	22153	1.0836	0.03011
Q8BUH8	Senp7	Sentrin-specific protease 7		23427.37	16154.77	22582.36	20722			7597.06	10158.09	8878	2.3341	0.03194
P31725	S100a9	Protein S100-A9	100042.3	66773.11	81588.74	135348.3	95938	41001.66	64046.95	60330.37	41392.07	51693	1.8559	0.03266
Q64512	Ptpn13	Tyrosine-protein phosphatase non-receptor type 13	15283.32	13919.5	15344.71	16542.17	15272	13085	13590.37	13728.49	14250.39	13664	1.1177	0.03373
Q62234	Myom1	Myomesin-1	24253.85	21741.71	17935.27	31868.58	23950	15228.56	14208.46		11714.37	13717	1.7460	0.03576
Q60590	Orm1	Alpha-1-acid glycoprotein 1	18338.97	15530.13	20637.62	14677.51	17296	15613.75	9894.143	11083.69	12749.16	12335	1.4022	0.03583
Q8BFY0	Pirt	Phosphoinositide-interacting protein	25146.45	31150.46	31017.27	33408.95	30181	32887.9	36318.61	35664.72	36785.77	35414	0.8522	0.03767
Q9CXG3	Ppil4	Peptidyl-prolyl cis-trans isomerase-like 4	12183.7	13954.29	8728.932		11622	8500.805	6009.386	3239.462	7662.386	6353	1.8294	0.03781
Q61247	Serpinf2	Alpha-2-antiplasmin	27345.17	26296.18	29987.72	20382.16	26003	23073.85	18772.97	19742.44	16928.2	19629	1.3247	0.03789
Q3UA37	Qrich1	Transcriptional regulator QRICH1	6261.66	6254.092	8212.54		6909	2766.587			4100.962	3434	2.0122	0.03790
O88878	Zfand5	AN1-type zinc finger protein 5		2390.83	2779.823	2459.222	2543			6904.293	4539.466	5722	0.4445	0.03803
Q80TE4	Sipa1l2	Signal-induced proliferation-associated 1-like protein 2		9116.472	6389.859	9747.314	8418		5284.329	4094.326	5591.537	4990	1.6869	0.03833
Q8CIV2	Tmem259	Membralin	18393.11	18491.01	18212.87	18992.6	18522	19344.3	20471.45	18820.12	21001.88	19909	0.9303	0.03931
Q9WUH1	Tmem115	Transmembrane protein 115	12473.65	12327.19	12630.52	12217.99	12412	12502.72	17370.35	16255.77	14494.16	15156	0.8190	0.04241
Q8K1L5	Ppp1r11	E3 ubiquitin-protein ligase PPP1R11	4234.441	4688.647	3148.421	2549.549	3655	2870.787	2408.879	820.5505	1731.179	1958	1.8670	0.04275
P35546	Ret	Proto-oncogene tyrosine-protein kinase receptor Ret	23115.76	22932.79	20860.67	20924.91	21959	22973.34	23697.62	27551.07	26494.81	25179	0.8721	0.04286
Q07113	Igf2r	Cation-independent mannose-6-phosphate recepto	19363.05	16625.51	18027.42	19707.91	18431	15943.1	17597.9	12403.15	14585.53	15132	1.2180	0.04471
Q8C3P7	Mettl3	N6-adenosine-methyltransferase subunit METTL3	7618.896	7637.593	8013.983	7246.028	7629	7031.403	6098.579			6565	1.1621	0.04519
Q80TY4	St18	Suppression of tumorigenicity 18 protein	8410.724	6608.181	8760.69	10421.98	8550	5616.708	7913.555	5401.667	5335.822	6067	1.4093	0.04711
Q9D0B0	Srsf9	Serine/arginine-rich splicing factor 9	46371.03	39309.72	38173.31	28304.91	38040	29244.04	30445.16	24465.65	28906.06	28265	1.3458	0.04779
Q80XM9	Slc66a2	Solute carrier family 66 member 2	8687.358	8867.172	6372.546	5836.838	7441	9262.573	9661.61	8913.521	9981.898	9455	0.7870	0.04821
Q52KI8	Srrm1	Serine/arginine repetitive matrix protein 1	15789.71	13317.28	14156.3	14283.18	14387	17433.77	18556.91	18998.51	14237.13	17307	0.8313	0.04976
P54729	Nub1	NEDD8 ultimate buster 1	22812.98	22120.76	22627.4	20780.82	22085	17795.42	19431.24	11708.01	20119.52	17264	1.2793	0.04987
Q3UP87	Elane	Neutrophil elastase	35679.51	23549.06	19418.19	31948.27	27649	11119.91	24871.3	15428.74	9750.657	15293	1.8080	0.05039
P41245	Mmp9	Matrix metalloproteinase-9	10852.84	5599.964	6223.299	10860.76	8384	2814.743	5640.34		1542.541	3333	2.5159	0.05081
P11672	Lcn2	Neutrophil gelatinase-associated lipocalin	43449.54	21292.32	28121.45	39767.47	33158	19231.52	26085.38	17575.21	14252.76	19286	1.7192	0.05099
Q8BHD4	Frmd3	FERM domain-containing protein 3	3856.706	6000.623	7029.975	6285.484	5793	8560.329	7075.743		10382.94	8673	0.6680	0.05215
O70255	Mpzl2	Myelin protein zero-like protein 2	7201.334	8950.245		7874.503	8009	5985.388	4172.909			5079	1.5768	0.05297
Q9D1H9	Mfap4	Microfibril-associated glycoprotein 4	40124.26	51666.38	54478.73	40912.72	46796	41228.89	38855.26	26538.61	33820.83	35111	1.3328	0.05453
Q7TT15	Galnt17	Polypeptide N-acetylgalactosaminyltransferase 17	46727.07	47379.33	47428.16	53452.5	48747	48508.2	57320.68	57264.38	58163.84	55314	0.8813	0.05548
Q91XL3	Uxs1	UDP-glucuronic acid decarboxylase 1	17289.73	17905.69	16509.16	17253.03	17239	14041.7	15638.46	11390.87	16743.54	14454	1.1927	0.05877

Q99N50	Sytl2	Synaptotagmin-like protein 2	9902.259	9259.159	9269.574	12342.81	10193	9625.131	8298.873	6740.105	6506.541	7793	1.3081	0.05913
Q08093	Cnn2	Calponin-2	11759.83	12604.5	10537.57	8667.86	10892	9489.299	8603.136	9104.884	7183.951	8595	1.2673	0.05981
Q9QXD8	Limd1	LIM domain-containing protein 1	9177.382	9077.351	8306.132	10112.41	9168	8213.244	8123.135	5838.645	8129.825	7576	1.2101	0.05982
Q6P9P0	Slf2	SMC5-SMC6 complex localization factor protein 2	2371.939	1758.747	2469.403	1055.074	1914	1001.968	1190.749		371.4256	855	2.2391	0.06048
Q99LC9	Pex6	Peroxisomal ATPase PEX6	6289.166	5492.595	6491.559	7601.121	6469	8610.25		11465.27	7456.079	9177	0.7049	0.06053
P08071	Ltf	Lactotransferrin	28269.42	16652.51	23003.29	36456.53	26095	13186.5	18418.21	18430.34	13293.16	15832	1.6483	0.06059
Q4FZC9	Syne3	Nesprin-3	14315.04	13987.82	11831.4	15762.78	13974	12296.26	11486.61	12315.39	12224.55	12081	1.1567	0.06405
Q6P1H6	Ankle2	Ankyrin repeat and LEM domain-containing protein 2	2975.003		2999.703	2235.096	2737	2156.597	1657.211	1969.234	2446.676	2057	1.3301	0.06435
O08692	Ngp	Neutrophilic granule protein	118781.7	63015.4	68179.83	140838	97704	36126.02	63078.25	63109.34	45762.82	52019	1.8782	0.06482
Q921Y4	Mfsd5	Molybdate-anion transporter	13079.91	10663.86		11461.62	11735		15006.23	14068.26		14537	0.8072	0.06491
P04117	Fabp4	Fatty acid-binding protein, adipocyte	25919.54	41221.34	49311.29	41756.62	39552	33725.2	30177.01		14693.62	24712	1.6005	0.06507
P59326	Ythdf1	YTH domain-containing family protein 1	9955.848	9197.572	8363.044	15605.96	10781	6075.01	8631.716	6176.785	6479.892	6841	1.5759	0.06513
Q9Z2W1	Stk25	Serine/threonine-protein kinase 25	9003.521	7213.62	10163.07	7755.64	8534	11525.29	8539.775	11228.95	11930.08	10806	0.7897	0.06612
Q00915	Rbp1	Retinol-binding protein 1	31819.54	42624.05	47780.24	39661.32	40471	42050.51	17847.52	21358.59	23707.82	26241	1.5423	0.06633
Q9ERY9	Erg28	Ergosterol biosynthetic protein 28 homolog	20204.08	20832.07	25177.97	23115.59	22332	17223.35	15758.92	14049.95	22711.4	17436	1.2808	0.06689
Q80Z19	Muc2	Mucin-2	14213.56	13817.05	12712.19	17813.08	14639	9035.33	15168.27	8394.049	7835.338	10108	1.4482	0.06720
Q8K2V1	Ppp4r1	Serine/threonine-protein phosphatase 4 regulatory s	4048.197	4083.073	3498.304	3428.195	3764	3201.324	3608.991	2021.138	2837.963	2917	1.2904	0.06744
Q6NWW9	Fndc3b	Fibronectin type III domain-containing protein 3B	13238.03		16049.82		14644	9940.687	10998.19		12027.56	10989	1.3326	0.06780
Q8K1B8	Fermt3	Fermitin family homolog 3	16563.94	13075.65	21619.46	19008.02	17567	12499.81	16270.73	11879.34	9377.735	12507	1.4046	0.07103
P11247	Mpo	Myeloperoxidase	83377.13	43320.9	50088.66	86837.71	65906	28270.98	58427.34	33423.27	25376.72	36375	1.8119	0.07123
Q8CJ67	Stau2	Double-stranded RNA-binding protein Staufen homo	14971.57	15551.45	17162.18	11696.64	14845	16194.31	19507.95	16719.83	20303.05	18181	0.8165	0.07223
P51912	Slc1a5	Neutral amino acid transporter B(0)	30427.35	29510.32	26754.84	25846.76	28135	22892.27	26720.52	26203.15	24629.59	25111	1.1204	0.07273
O70622	Rtn2	Reticulon-2	45465.98	39277.32	29100.1	34746.57	37147	26594.24	36086.41	10070.46	19375.68	23032	1.6129	0.07327
Q80XL6	Acad11	Acyl-CoA dehydrogenase family member 11	17806.52	11920.93	17235.9	14738.9	15426	11540.22	13216.33	8538.953	13286.25	11645	1.3246	0.07338
Q925J9	Med1	Mediator of RNA polymerase II transcription subunit	1379.881	734.2912	400.9521	544.1782	765		1660.037	1093.973	1519.225	1424	0.5369	0.07391
P49586	Pcyt1a	Choline-phosphate cytidylyltransferase A	11915.5	12092.02	12292.14	8888.931	11297	10163.66	10258.9	7175.245	7772.389	8843	1.2776	0.07396
Q8R242	Ctbs	Di-N-acetylchitobiase	12176.29	12697.58	13215.9	17605.06	13924	18232.84	15617.94	16093.56	17735.58	16920	0.8229	0.07545
Q6NSU3	Glt8d1	Glycosyltransferase 8 domain-containing protein 1	3301.696	3409.704	5955.233	2522.692	3797	5328.402	4908.68	5583.333	6148.826	5492	0.6914	0.07549
P61372	Isl1	Insulin gene enhancer protein ISL-1	11834.89	9352.623	9427.311	10804.89	10355	12940.56	9948.134	15907.13	15283.08	13520	0.7659	0.07578
Q8BQS4	Eeig2	EEIG family member 2	7059.499	7354.744	5917.874	5440.792	6443	6372.709	4348.676	4190.544	5088.706	5000	1.2886	0.07628
P04247	Mb	Myoglobin	14656.27	30179.39	17087.56	19035.56	20240	14955.59	15545.05	8508.3	7705.956	11679	1.7330	0.07664
Q9CQU5	Zwint	ZW10 interactor	10928.62	11954.59	10087.14	9751.83	10681	10075.9	8595.509	9188.744	5608.293	8367	1.2765	0.07722
Q6QIY3	Scn10a	Sodium channel protein type 10 subunit alpha	15976.25	11892.03	12174.11	15087.87	13783	17726.74	14104.61	16747.35	19398.51	16994	0.8110	0.07772
Q7TSG2	Ctdp1	RNA polymerase II subunit A C-terminal domain pho:	4069.193	4248.553	5266.803	2965.036	4137	2965.567	3616.265	2723.193	2934.393	3060	1.3522	0.07882
O35309	Nmi	N-myc-interactor	14302.17	13969.67	14349.41	15168.47	14447	14627.85	16288.38	17723.16		16213	0.8911	0.08021
Q6A028	Swap70	Switch-associated protein 70	18394.49	19294.18	18052.08	14613.4	17589	16980	15204.99	13861.68	13456.69	14876	1.1824	0.08153
O55033	Nck2	Cytoplasmic protein NCK2	11037.49	11005.08	10512.28	8056.644	10153	9199.18	9343.606	6296.787	5075.919	7479	1.3575	0.08158
Q8K1C9	Lrrc41	Leucine-rich repeat-containing protein 41	7460.393	6064.439	6756.227	6452.97	6684		5836.691	2576.966		4207	1.5887	0.08589
P54320	Eln	Elastin	52807.28	42887.44	50929.95	53076.14	49925	52415.41	56401.63	86489.21	97657.96	73241	0.6817	0.08667
O54965	Rnf13	E3 ubiquitin-protein ligase RNF13	11678.05	11083.86	9503.331	10176.35	10610	8274.943	10626.19	2254.545	6241.83	6849	1.5491	0.08674
Q61646	Hp	Haptoglobin	62812.53	12969.25	15855.42	50152.32	35447	10622.1	9444.662	8988.884	11068.86	10031	3.5337	0.08707
P13864	Dnmt1	DNA (cytosine-5)-methyltransferase 1	10865.01	7610.745	9093.203	7390.489	8740	8053.987	7299.432	3835.775	5757.92	6237	1.4013	0.08810
Q6DTY7	Pfkfb4	6-phosphofructo-2-kinase/fructose-2,6-bisphospha	8447.646	5243.511	6887.959	6740.614	6830	10453.5	7222.63		10121.62	9266	0.7371	0.08902
Q8CIZ8	Vwf	von Willebrand factor	14313.32	15376.61	16171.52	16726.79	15647	14635.85	12037.87	15509.13	11694.37	13469	1.1617	0.09050
O89020	Afm	Afamin	23929.92	27852.33	17356.19		23046	21580.84	15197.75	8483.164	5606.559	12717	1.8122	0.09096
Q5S006	Lrrk2	Leucine-rich repeat serine/threonine-protein kinase	4291.475	4487.621	6086.063	5163.987	5007	6351.047	5806.108	8518.296	5568.01	6561	0.7632	0.09527
Q61508	Ecm1	Extracellular matrix protein 1	23965.83	22581.74	20809.42	12966.93	20081	17840.67	13229.56	15498.94	11622.25	14548	1.3803	0.09616
P19221	F2	Prothrombin	21401.22	19560.24	21172.26	12990.83	18781	17716.68	14137.08	13063.57	11797.44	14179	1.3246	0.09766
O88796	Rpp30	Ribonuclease P protein subunit p30	23377.51	23785.46	11011.28	8691.975	16717	11712.42	9660.313	1585.972	8307.857	7817	2.1386	0.09850
P97313	Prkdc	DNA-dependent protein kinase catalytic subunit	16768.69	10740.23	13418.74	10198.36	12782	9388.528	8807.261	8603.414	11514.98	9579	1.3344	0.09947
Q99LC2	Cstf1	Cleavage stimulation factor subunit 1	8816.133	9251.16	11383.08	8988.54	9610	8356.713	7555.125	7837.719	9230.094	8245	1.1655	0.09986
Q9ESB3	Hrg	Histidine-rich glycoprotein	22213.69	16770.55	15574.97	15790.94	17588	14512.75	17396.87	9377.671	9866.915	12789	1.3753	0.10099
Q8BRK8	Prkaa2	5'-AMP-activated protein kinase catalytic subunit alp	7406.404	7624.649	8338.165	6836.859	7552	9668.43	10295.31	8505.702	7333.2	8951	0.8437	0.10156
A6H6A4	Lrriq4	Leucine-rich repeat and IQ domain-containing protei	437521.9	455398.4	499597.3	452288.8	461202	434238.1	359726.3			396982	1.1618	0.10209
Q99JP0	Map4k3	Mitogen-activated protein kinase kinase kinase kinas	14003.49	9154.638	12282.73	9295.556	11184	10397.7	9160.177	4517.06	7213.336	7822	1.4298	0.10239
Q61129	Cfi	Complement factor I	31956.38	27714.95	33871.42	22832.23	29094	26365.92	26675.98	18017.04	19793.45	22713	1.2809	0.10246
O09012	Pex5	Peroxisomal targeting signal 1 receptor	16017.54	17424.59	17230.54		16891		15701.53	15120.31	16315.96	15713	1.0750	0.10294
Q9R1Q7	Plp2	Proteolipid protein 2	18762.56	26928.94	17352.55	28278.86	22831	27578.46	30209.34	30789.27	25890.54	28617	0.7978	0.10304
Q99JR8	Smarcd2	SWI/SNF-related matrix-associated actin-dependent	22961.74	20123.15	18711.76	18278.67	20019	22815.63	23110.81	22483.41	20726.8	22284	0.8983	0.10437

O09005	Degs1	Sphingolipid delta(4)-desaturase DES1	5169.839	5884.473	4485.351	4672.175	**5053**	4705.414	4285.717	3884.133	4572.86	**4362**	1.1584	0.10458
O35744	Chil3	Chitinase-like protein 3	68917.3	58614.49	28191.07	77677.37	**58350**	30688.76	58583.61	15801.54	15275	**30087**	1.9394	0.10488
Q99LQ7	PEDS1	Plasmanylethanolamine desaturase 1	6822.089	9982.443	19612.94	11726.71	**12036**	18751.03	13225.17	19379.74	21585.39	**18235**	0.6600	0.10514
Q9CZU4	Eral1	GTPase Era, mitochondrial	28513.46		33627.34	38167.2	**33436**	36714.15	40733.52	38764.68	37646.6	**38465**	0.8693	0.10538
P28481	Col2a1	Collagen alpha-1(II) chain	33448.95	33103.57	24030.42	39682.61	**32566**	35867.62	36640.65	42420.68	47828.25	**40689**	0.8004	0.10543
Q9JJG0	Tacc2	Transforming acidic coiled-coil-containing protein 2	7823.444	5679.405	7049.325	5744.832	**6574**	9467.361	6564.818	7619.241	10607.63	**8565**	0.7676	0.10602
Q99J95	Cdk9	Cyclin-dependent kinase 9	8327.179	9438.432	7204.401	7646.824	**8154**	6587.881	7027.642	7222.845	7696.608	**7134**	1.1430	0.10662
Q60994	Adipoq	Adiponectin	43185.04	51178.82	48531.37	35778.39	**44668**	45144.31	32863.13	32959.12	33261.53	**36057**	1.2388	0.10743
Q9Z2L6	Minpp1	Multiple inositol polyphosphate phosphatase 1		6871.962	16429.96	14544.09	**12615**	28984.23	19268.43	15193.35	20013.82	**20865**	0.6046	0.10797
P39876	Timp3	Metalloproteinase inhibitor 3	18488.16	16644.59	16899.36	17993.09	**17506**	14783.09	18262.17	12673.54	15013.73	**15183**	1.1530	0.10881
Q61510	Trim25	E3 ubiquitin/ISG15 ligase TRIM25	10886.96	11343.6	11286.03	8124.304	**10410**	13477.59	11443.99	11302.8	12319.04	**12136**	0.8578	0.10897
Q8BHJ5	Tbl1xr1	F-box-like/WD repeat-containing protein TBL1XR1	11513.86	12900.69	11853.47	11251.13	**11880**	10877.22	12170.57	7995.144	8901.007	**9986**	1.1896	0.11035
Q5U4C1	Gprasp1	G-protein coupled receptor-associated sorting prote	6296.318	4562.896	5281.693	5795.789	**5484**	3834.947	5671.791	4257.587	4009.645	**4443**	1.2342	0.11190
P21812	Mcpt4	Mast cell protease 4	242983.2	120727.8	122335.5	159892	**161485**	67841	160727.2	58525.01	85895.84	**93247**	1.7318	0.11353
Q05144	Rac2	Ras-related C3 botulinum toxin substrate 2	45139.34	17068.79	20781.47	26538.93	**27382**	14139.91	18508.5	14436.69	15800.28	**15721**	1.7417	0.11414
Q61878	Prg2	Bone marrow proteoglycan	106069.4	58048.4	28914.9	52644.52	**61419**	28763.32	49623.42	19601.56	18728.91	**29179**	2.1049	0.11830
Q8CEC0	Nup88	Nuclear pore complex protein Nup88	15000.88	16659.47	17421.5	16090.97	**16293**	15800.5	12861.6	14301.69	15846.98	**14703**	1.1082	0.11892
Q8VC03	Eml3	Echinoderm microtubule-associated protein-like 3	9440.949	9201.815	9908.611	7243.363	**8949**	7581.951	7708.525	4871.356	8493.164	**7164**	1.2492	0.11976
P49290	Epx	Eosinophil peroxidase	57089.84	30984.58	15969.44	31576.54	**33905**	12879.52	29415.17	13235.08	11866.97	**16849**	2.0123	0.12298
Q78KK3	Slc22a18	Solute carrier family 22 member 18		4488.422	4167.703	4539.123	**4398**	6398.062	4380.898	10434.48	7464.9	**7170**	0.6135	0.12330
P51437	Camp	Cathelicidin antimicrobial peptide	19290.42	7555.733	12540.79	18987.63	**14594**	5457.402	13619.46	7973.851	7596.667	**8662**	1.6848	0.12341
Q91YV9	Pgap4	Post-GPI attachment to proteins factor 4	6896.48	6017.307	7457.626	4133.8	**6126**	6839.661	7945.01	8602.012	7053.444	**7610**	0.8050	0.12555
Q8C1D8	Iws1	Protein IWS1 homolog	1590.378	1144.655			**1368**	2289.101	1694.022		2685.183	**2223**	0.6152	0.12657
Q9D6L8	Ppil3	Peptidyl-prolyl cis-trans isomerase-like 3	18935.44	19368.42	18353.38	15732.05	**18097**	14101.58	18010.31	16953.46	14725.54	**15948**	1.1348	0.13104
Q9DBX2	Pdcl	Phosducin-like protein	5729.371	6150.127	6425.489	6883.647	**6297**	5596.552	8850.785	8615.023	7577.082	**7660**	0.8221	0.13119
P32507	Nectin2	Nectin-2	19135	20445.62	20611.45	21901.22	**20523**	17483.04	21534.87	14575.27	17819.46	**17853**	1.1496	0.13261
Q9Z2F7	Bnip3l	BCL2/adenovirus E1B 19 kDa protein-interacting pro	11183.73	13994.76	13386.53	19991.83	**14639**	23920.4	13263.72	19291.39	30046.89	**21631**	0.6768	0.13283
Q80U38	Khnyn	Protein KHNYN	16156.76	16896	12954	18222.21	**16057**	18116.33	19981.57	16126.78	20097.35	**18581**	0.8642	0.13437
P16015	Ca3	Carbonic anhydrase 3	95724.08	168649.6	114690.1	94878.2	**118485**	110197.7	98169.83	61781.12	39807.98	**77489**	1.5291	0.13511
Q9JLF6	Tgm1	Protein-glutamine gamma-glutamyltransferase K	6485.246	8657.78	6816.06	8025.756	**7496**	22496.98	8232.966	7312.706	17563.41	**13902**	0.5392	0.13577
Q60973	Rbbp7	Histone-binding protein RBBP7	10989.68	9262.466	10408.51	7457.751	**9530**	8683.802	8521.89	6496.21	8092.222	**7949**	1.1989	0.13826
Q8BUB4	Wdfy2	WD repeat and FYVE domain-containing protein 2	35502.44	33235.42	25656.55	23938.62	**29583**	23642.14	31377.93	13961.1	16621.76	**21401**	1.3823	0.14028
Q61112	Sdf4	45 kDa calcium-binding protein	16495.09	13815.54	13262.64	14615.78	**14547**	15454.79	12686.38	10022.71	9871.019	**12009**	1.2114	0.14050
Q3ULW8	Parp3	Protein mono-ADP-ribosyltransferase PARP3	25546.27	21566.54	26223.14	22791.38	**24032**	18544.8	24299.17	20475.05	21724.38	**21261**	1.1303	0.14143
Q6J756	Capn11	Calpain-11	47592.68	66486.88	49675.13	53520.56	**54319**	65343.9	59152.68	76536.26	57286.01	**64580**	0.8411	0.14174
Q9QXC1	Fetub	Fetuin-B	283173	213608.5	305801.2	152961.6	**238886**	182189.6	184172.5	204957.2	113555.3	**171219**	1.3952	0.14176
Q8BH60	Gopc	Golgi-associated PDZ and coiled-coil motif-containir	18619.45	21003.59	20657.79	16914.55	**19299**	20686.71	20931.87	20331.69	22317.34	**21067**	0.9161	0.14239
Q8CFX1	H6pd	GDH/6PGL endoplasmic bifunctional protein	15476.99	12923.56	13071.15	15028.29	**14125**	12683.03	13403.21	9249.188	13223.94	**12140**	1.1635	0.14261
Q8CD91	Smoc2	SPARC-related modular calcium-binding protein 2	20618.85	17203.55	21766.16	19448.67	**19759**	16996.96	16807	18005.3	19475.19	**17821**	1.1088	0.14292
Q9EPX2	Papln	Papilin	46180.75	43808.11	36809.43	52634.41	**44858**	40973.61	41853.2	31938.71	38027.62	**38198**	1.1744	0.14378
Q8BVW0	Ganc	Neutral alpha-glucosidase C	4644.025	4231.693	4591.39	4546.001	**4503**	4041.991	4700.533	2624.14	3668.997	**3759**	1.1980	0.14466
Q8R0A0	Gtf2f2	General transcription factor IIF subunit 2	5747.403	5263.745	7956.802	3892.967	**5715**	6077.951	3316.187	3167.622	2606.609	**3792**	1.5071	0.14473
Q9QZD4	Ercc4	DNA repair endonuclease XPF	2178.386	1638.209	901.7452	1186.902	**1476**	559.0518	847.4116			**703**	2.0993	0.14480
Q6Q899	Rigi	Antiviral innate immune response receptor RIG-I	12544.07	12695.82	13596.48	9062.672	**11975**	9477.012	12151.77	7476.866	9532.953	**9660**	1.2397	0.14535
Q6VVW5	Npr2	Atrial natriuretic peptide receptor 2	11099.37	9814.754	10515.94	9939.243	**10342**	10562.37	11647.62	10499.34	11467.96	**11044**	0.9364	0.14567
P15306	Thbd	Thrombomodulin	4122.083	3032.739	4023.805	2890.156	**3517**	3083.595	2428.116	2892.811	3217.817	**2906**	1.2105	0.14577
Q6P2K6	Ppp4r3a	Serine/threonine-protein phosphatase 4 regulatory subunit 3A		5413.033	6333.808	5388.965	**5712**		5775.239	2654.575	3291.67	**3907**	1.4619	0.14588
Q925T6	Grip1	Glutamate receptor-interacting protein 1	11627.19			10004.24	**10816**	8958.173			7320.451	**8139**	1.3288	0.14598
O35516	Notch2	Neurogenic locus notch homolog protein 2	11994.17	5808.716	6513.782	1438.894	**6439**	676.9709	5850.734	48.22984	2310.914	**2222**	2.8982	0.14602
Q9D3E6	Stag1	Cohesin subunit SA-1	7751.087	6430.737	7350.302	6635.501	**7042**	6854.274	6117.178	3091.101	6144.355	**5552**	1.2684	0.14603
P31649	Slc6a13	Sodium- and chloride-dependent GABA transporter	18036.38	14430.58	13484.03	15679.83	**15408**	14985.76	13947.74	3556.376	10698.24	**10797**	1.4270	0.14610
Q3UKC1	Tax1bp1	Tax1-binding protein 1 homolog	53213.66	64330.73	47705.8	51388.32	**54160**	35097.41	54084.31	36046.58	50837.22	**44016**	1.2304	0.14669
O35074	Ptgis	Prostacyclin synthase	22748.85	19333.38	18400.85	15609	**19023**	19695.84	16208.74	9606.669	13310.66	**14705**	1.2936	0.14788
O54782	Man2b2	Epididymis-specific alpha-mannosidase	17162.18	14720.95	17680.65	16129.77	**16423**	16649.16	13876.67	13752.54	15184.5	**14866**	1.1048	0.14870
Q9Z126	Pf4	Platelet factor 4	14551.84	14804.44	23992.69	19252.71	**18150**	13060.76	15903.52	12264.73	15509.74	**14185**	1.2796	0.14962
Q91WD0	Gpr108	Protein GPR108	16984.47	21271.82	26544.68	28826.47	**23407**	26087.07	27046.38	32426.15	27927.91	**28372**	0.8250	0.15000
P49300	Clec10a	C-type lectin domain family 10 member A	16538.89	12478.43	11878.38	6597.232	**11873**	7888.78	12635.67	2595.955	4668.008	**6947**	1.7091	0.15065
Q5SW46	Lpo	Lactoperoxidase	13318.92		5715.891	11450.47	**10162**	5895.7	7590.194		2512.002	**5333**	1.9056	0.15179
Q9CYA6	Zcchc8	Zinc finger CCHC domain-containing protein 8	802.3248	1148.241	765.8395		**905**	1774.226	2413.375	611.3356	1975.724	**1694**	0.5346	0.15183

O89114	Dnajb5	DnaJ homolog subfamily B member 5	13227.49	11946.12	10457.14	15977.46	12902	9719.909	12531.37	9426.932	10977.19	10664	1.2099	0.15297
Q9DCE5	Pak1ip1	p21-activated protein kinase-interacting protein 1	4434.382	5848.817	5923.898	4301.303	5127	4657.273	5307.797	2440.014	2501.961	3727	1.3758	0.15388
P18155	Mthfd2	Bifunctional methylenetetrahydrofolate dehydrogena	5635.406	7452.018	10004.66	7807.424	7725	5976.843	7473.172	6014.006	3094.304	5640	1.3698	0.15509
P97290	Serping1	Plasma protease C1 inhibitor	45996.51	34937.23	37701.5	24887.51	35881	34401.92	26429.42	24756.54	26288.82	27969	1.2829	0.15519
Q99PN3	Trim26	Tripartite motif-containing protein 26	14674.43	14025.48	16077.47	12178.6	14239	11465.07	11881.39	11174.39	14852.86	12343	1.1536	0.15720
Q8BYU6	Tor1aip2	Torsin-1A-interacting protein 2	1524.631	1598.409	1321.133		1481	1615.777	1872.51	3584.617	2306.393	2345	0.6318	0.15893
O54749;O54750	Cyp2j5;Cyp2j6	Cytochrome P450 2J5;Cytochrome P450 2J6	69616.16	53905.31	82534.45	74865.91	70230	64249.1	62949.63	49825.91	59795.75	59205	1.1862	0.16001
P49138	Mapkapk2	MAP kinase-activated protein kinase 2	12628.23	13524.51	11700.82	10664.79	12130	11073.56	11821.75	11073.18	8833.97	10701	1.1335	0.16019
P52875	Tmem165	Transmembrane protein 165	40225.88	36097.71	43026.28	38438.6	39447	41608.21	38971.68	47882.43	44847.57	43327	0.9104	0.16086
P49935	Ctsh	Pro-cathepsin H	16767.18	14670.84	17102.66	11985.96	15132	14504.09	10771.42	12114.54	13861.38	12813	1.1810	0.16127
Q9JIM1	Slc29a1	Equilibrative nucleoside transporter 1	22877.6	24278.71	25714.67	32693.27	26391	30441.65	27602.8	36380.46	29547.09	30993	0.8515	0.16180
Q8BMD6	Tmem260	Protein O-mannosyl-transferase TMEM260	1923.297	2594.306	4580.458	2424.682	2881	4000.301		5492.25	3397.32	4297	0.6705	0.16280
P04186	Cfb	Complement factor B	33638.14	38868.8	45767.88	23049.49	35331	32805.41	23814.7	23870.76	27529.78	27005	1.3083	0.16295
O09164	Sod3	Extracellular superoxide dismutase [Cu-Zn]	37707.5	37861.16	31984.32	30333.19	34472	31657.41	32893.01	19104.82	30531.1	28547	1.2076	0.16325
Q9D0E5	Efcab11	EF-hand calcium-binding domain-containing protein	6995.976		4430.607	4938.044	5455	3772.238	4394.844	5111.521	3274.659	4138	1.3181	0.16379
Q3UWW6	Gas2l3	GAS2-like protein 3	34295.63	28632.23	30307.41	25943.51	29795	28511.6	27520.2	24420.18	26316.4	26692	1.1162	0.16415
Q8K0G8	Esrp2	Epithelial splicing regulatory protein 2	9178.283	2383.596	6628.264	1583.06	4943	1599.184	2800.559	2175.586	1715.608	2073	2.3849	0.16474
P97401	Frzb	Secreted frizzled-related protein 3	19097.44	14052.79	13783.47	14400.04	15333	13182.17	11112.42	12535.87	15018.61	12962	1.1829	0.16476
Q01279	Egfr	Epidermal growth factor receptor	17599.69	17648.12	26583.07	15230.35	19265	16659.93	12787.59	15053.67	16022.24	15131	1.2732	0.16883
Q91VS8	Farp2	FERM, ARHGEF and pleckstrin domain-containing pr	2865.788	3001.604	1806.178	2600.171	2568	1651.538	1261.98	2573.129	2289.994	1944	1.3211	0.17002
P28843	Dpp4	Dipeptidyl peptidase 4	37975.45	28278.52	29188.16	38694.94	33534	33807.09	30111.2	17045.77	24662.38	26407	1.2699	0.17087
Q99JV5	Stard4	StAR-related lipid transfer protein 4	1343.766	537.1747	1349.94		1077	908.3626	845.3805	2.076787	394.0734	537	2.0037	0.17089
O70423	Aoc3	Membrane primary amine oxidase	36074.6	32300.47	39452.05	35916.13	35936	31097.22	35879.39	31034.2	34096.4	33027	1.0881	0.17344
Q8K449	Abca9	ATP-binding cassette sub-family A member 9	64483.78	59021.28	67266.41	71255.55	65507	68071.34	65380.68	75091.05	76114.6	71164	0.9205	0.17475
C0HKG5;C0HKG6	Rnaset2a;Rnaset2b	Ribonuclease T2-A;Ribonuclease T2-B	9532.104	7054.494	11958.16	5254.047	8450	7059.184	7609.259	3041.808	5051.238	5690	1.4849	0.17493
Q8K2C8	Gpat4	Glycerol-3-phosphate acyltransferase 4	9476.976	9064.659	14169.97	14001.7	11678		12186.22	16794.79	15579.85	14854	0.7862	0.17533
Q9JHW9	Aldh1a3	Retinaldehyde dehydrogenase 3	1012.778	4987.978	3110.033	2328.474	2860	880.4179		198.4052	2278.23	1119	2.5556	0.17714
Q9WVC3	Cav2	Caveolin-2	61966.53	71736.41	55860.54	66476.76	64010	75643.57	80225.09	65722.75	65169.29	71690	0.8929	0.17726
Q8BR07	Bicd1	Protein bicaudal D homolog 1	20570.79	15786.52	21941.09	13404.7	17926	18513.23	14339.01	8659.213	12794.97	13577	1.3203	0.17863
Q8C0D4	Arhgap12	Rho GTPase-activating protein 12	3186.119	3384.029	6797.355	3866.442	4308	4013.985	5889.037	7213.742	6825.616	5986	0.7198	0.17940
Q8VBV3	Exosc2	Exosome complex component RRP4	6654.725	6257.845	6511.989	5469.095	6223	5704.474	6550.608	4892.532	4755.039	5476	1.1366	0.17949
Q9D6J1	Cers4	Ceramide synthase 4	16135.31	14441.42	16278.16	15721.26	15644	15979.48	15034.46	19572.13	20179.33	17691	0.8843	0.17968
Q8CGB6	Tns2	Tensin-2	20015.93	17216.69	16218.5	18808.86	18065	17936.96	17817.25	13077.41	14405.73	15809	1.1427	0.17970
Q8CDJ8	Ston1	Stonin-1	11991.4	14071.44	10463.27	10011.92	11635	8662.351	10110.07	8266.488	11978.28	9754	1.1928	0.18122
Q8K363	Ddx18	ATP-dependent RNA helicase DDX18	17084	11463.93	8422.75	9366.104	11584	8890.946	10072.13	2403.368	9226.058	7648	1.5146	0.18421
P97366	Evi5	Ecotropic viral integration site 5 protein	12878.39	5812.721	7813.843	4194.405	7675	4700.147	4030.442	4751.718	5730.736	4803	1.5978	0.18501
Q91X91	Qprt	Nicotinate-nucleotide pyrophosphorylase [carboxyla	11066.1	13768	9702.889	6464.304	10250	7672.489	4036.226	8991.925		6900	1.4855	0.18546
Q8BW10	Nob1	RNA-binding protein NOB1	4270.89	3412.899			3842	3242.926	3421.712	2744.78		3136	1.2249	0.18620
Q8BHB4	Wdr3	WD repeat-containing protein 3	15110.57	15951.83	10347.57	12165.22	13394	14880.02	11417.87	3759.228	7015.787	9268	1.4451	0.18643
O88968	Tcn2	Transcobalamin-2	8522.383	7131.844	7640.554	6164.755	7365	6597.215	7934.479	4482.544	4827.414	5960	1.2356	0.18714
Q3UPF5	Zc3hav1	Zinc finger CCCH-type antiviral protein 1	12273.01	9671.129	14379.04	11214.36	11884	14263.59	14601.59	17676.72	11014.83	14389	0.8259	0.18717
Q03145	Epha2	Ephrin type-A receptor 2	4293.694	3817.398	5381.774	4664.435	4539	5032.076	4956.471		5558.534	5182	0.8759	0.18753
Q9ES46	Parvb	Beta-parvin	1240.362	1870.739	1888.514	1234.054	1558	1370.058	1095.399		1163.212	1210	1.2884	0.18993
Q8R0G7	Spns1	Protein spinster homolog 1	13508.59	16473.76	17969.38	13913.25	15466	21019.5	14882.83	18305.56	17415.41	17906	0.8638	0.19026
Q9CQL4	Mrpl20	Large ribosomal subunit protein bL20m	12074.92	11042.52	13819.4	11869.78	12202	5995.059	13155.07	3240.967	12065.18	8614	1.4165	0.19429
Q4PZA2	Ece1	Endothelin-converting enzyme 1	16360.99	13409.24	19254.38	8753.162	14444	14110.75	19170.13	20647.49	19418.88	18337	0.7877	0.19468
Q9D0Q7	Mrpl45	Large ribosomal subunit protein mL45	3151.411	6211.808	5114.453	3725.724	4551	4380.325	2695.006	1361.108	4074.772	3128	1.4550	0.19610
P21843	Mcpt3	Mast cell protease 3 (Fragment)	5901.396	6595.244	4281.242	7817.553	6149	5320.813	6190.709	4239.082	2846.437	4649	1.3225	0.19639
O08800	Serpinb8	Serpin B8	10288.76	14067.45	13245.16	10035.93	11909	11244.88	11326.43	9387.515	8500.67	10115	1.1774	0.19807
Q8VI36	Pxn	Paxillin	30179.91	31716.24	19900.4	27801.56	27400	29667.52	31729.66	34084.91	30249.04	31433	0.8717	0.20048
Q8BPB5	Efemp1	EGF-containing fibulin-like extracellular matrix prote	23106.23	27838.66	26249.66	22873.71	25017	25415.56	22042.4	21619.2	22421	22875	1.0937	0.20061
P05555	Itgam	Integrin alpha-M	128300.8	66724.45	54292.22	76586.98	81476	41525.5	68887		43749.84	51387	1.5855	0.20382
Q60778	Nfkbib	NF-kappa-B inhibitor beta	19477.24	19699.32	23402.29	15515.13	19523	21286.65	18603.51	32981.84	25297.57	24542	0.7955	0.20397
Q8BG30	Nelfa	Negative elongation factor A	9681.848	9653.898		10033.17	9790	10866.3	9147.918	15979.1	13561.18	12389	0.7902	0.20421
Q3UY34	Custos	Protein CUSTOS	4646.855	3931.792	5562.912	6413.804	5139	3875.516	5887.188	2234.582	3219.641	3804	1.3508	0.20641
B1AZI6	Thoc2	THO complex subunit 2	23520.79	15063.54	16949.66	15775.65	17827	16451.28	14660.77	9397.798	16386.17	14224	1.2533	0.20765
Q80TP3	Ubr5	E3 ubiquitin-protein ligase UBR5	3301.008		642.6887	2200.117	2048		3930.727	3383.692		3657	0.5600	0.21145
Q9CQW9	Ifitm3	Interferon-induced transmembrane protein 3	53476	43758.09	52915.84	53963.75	51028	47835.31	42997.96	50539.48	46581.83	46989	1.0860	0.21209
P06537	Nr3c1	Glucocorticoid receptor	31627.33	29942.38	34128.73	24534.31	30058	25789.62	25066.07	21347.2	31619.06	25955	1.1581	0.21227

B2RXR6	Ankrd44	Serine/threonine-protein phosphatase 6 regulatory a	13993.29	13222.84	16118.61	10265.62	13400	15888.99	14431.32	14454.94	15963.96	15185	0.8825	0.21410
P13516	Scd1	Acyl-CoA desaturase 1	15567.04	12896.36	12257.42	15860.72	14145	14059.91	11043.18	10033.77	13875.89	12253	1.1544	0.21514
O70475	Ugdh	UDP-glucose 6-dehydrogenase	4747.886	4350.127	7036.019	3980.813	5029	4993.138	3946.817	3631.771	3250.896	3956	1.2713	0.21927
Q9CWU2	Zdhhc13	Palmitoyltransferase ZDHHC13	16854.13	14697.33	14168.66	12937.1	14664	12322.7	14779.03	8021.085	14085.72	12302	1.1920	0.21971
O08715	Akap1	A-kinase anchor protein 1, mitochondrial	1872.167	2833.683	1001.327	2235.698	1986	1647.231	3361.828		4187.83	3066	0.6477	0.22127
Q71KT5	Tm7sf2	Delta(14)-sterol reductase TM7SF2	23716.12	27906.25	33649.44	26052.09	27831	28653.2	28244.31	33703.62	36803.27	31851	0.8738	0.22328
P11881	Itpr1	Inositol 1,4,5-trisphosphate receptor type 1	14480.38	26856.43	16265.03	9866.08	16867	7893.315	16964.53	12547.7	6903.476	11077	1.5227	0.22435
Q99J31	Ophn1	Oligophrenin-1	14651.69	12484.11	15035.49	9869.255	13010	9869.026	13319.48	11743.57	8488.604	10855	1.1985	0.22436
Q99PM9	Uck2	Uridine-cytidine kinase 2	11309.67	11407.73	12019.82	10980.61	11429	14761.74	12878.62	11358.6	11280.05	12570	0.9093	0.22652
Q9EQW7	Kif13a	Kinesin-like protein KIF13A	6522.844	5722.86	5786.586	4804.561	5709	7783.498	5286.256	5942.899	7712.248	6681	0.8545	0.22685
P49222	Epb42	Protein 4.2	10166.59	10467.93	13241.27	14510.47	12097		6420.407	12754.91	8898.118	9358	1.2927	0.22689
Q9DBS9	Osbpl3	Oxysterol-binding protein-related protein 3	73227.42	93502.44	84223.19	95532.13	86621	71280.58	93303.19	69012.28	71892.45	76372	1.1342	0.22779
Q00262	Stx2	Syntaxin-2	24200.86	22402.57	21630.38	26464.58	23675	22094.52	24156.75	33140.33	30330.53	27431	0.8631	0.22829
D0QMC3;P0DOV2	Mndal;Ifi204	Myeloid cell nuclear differentiation antigen-like prote	13320.19	10623.76	9396.793	6470.936	9953	7387.393	9061.071	3753.275	9344.975	7387	1.3474	0.22900
Q8BND5	Qsox1	Sulfhydryl oxidase 1	14415.75	11835.59	16569.92	9605.063	13107	10850.29	11779.49	11115.72	10432.46	11044	1.1867	0.22966
P02301;P84244	H3-5;H3-3a	Histone H3.3C;Histone H3.3	17357.06	21197.97	15781.82	16416.97	17688	14000.27	20536.74	6963.001	13068.04	13642	1.2966	0.23036
Q99PP7	Trim33	E3 ubiquitin-protein ligase TRIM33	1869.434	1303.884	1006.543	2186.796	1592	2787.288	3101.646	705.1578	3237.295	2458	0.6476	0.23059
Q9DBD0	Ica	Inhibitor of carbonic anhydrase	30237.5	27456.73	32012.53	11089.84	25199	23327.47	16063.21	20067.64	13367.36	18206	1.3841	0.23305
P21956	Mfge8	Lactadherin	30191.54	23747.98	20469.89	20136.32	23636	19096.85	24250.16	19579.13	16679.34	19901	1.1877	0.23328
P58196	Plscr4	Phospholipid scramblase 4	27720.74	24901.58	21563.01	24174.14	24590	27843.74	20543.75	18591.42	17118.09	21024	1.1696	0.23398
Q9QZ85	Iigp1	Interferon-inducible GTPase 1	71539.07	14254.73			42897	11283.02	13791.19		8479.625	11185	3.8353	0.23566
P58466	Ctdsp1	Carboxy-terminal domain RNA polymerase II polypep	12155.68	14449.99	13577.4	12101.02	13071	9288.753	14301.02		9408.058	10999	1.1884	0.23600
Q9ER69	Wtap	Pre-mRNA-splicing regulator WTAP	5995.72	5439.222	5989.897	8217.771	6411	8442.989	5006.014	10522.6	8492.34	8116	0.7899	0.23745
Q9EPB4	Pycard	Apoptosis-associated speck-like protein containing a	12348.49	4684.466	10195.74	10030.52	9315	4706.377	7398.922		7495.515	6534	1.4257	0.23803
Q80TT8	Cul9	Cullin-9	7395.3		4603.244	4926.116	5642	4923.164	4897.369	3419.094	4770.469	4503	1.2530	0.23957
Q03734	Serpina3m	Serine protease inhibitor A3M	14864.9	13064.53	23815.68	11331.92	15769	12133.67	10069.53	15755.73	8506.17	11616	1.3575	0.24057
Q9DC53	Cpne8	Copine-8	7445.107	7491.655	6813.646	9218.624	7742	6070.717	6753.941	6435.79	8146.496	6852	1.1300	0.24245
P51885	Lum	Lumican	1249189	1309673	991028.5	1160008	1177475	1138116	1211229	903368.1	917435.4	1042537	1.1294	0.24293
Q8VDI9	Alg9	Alpha-1,2-mannosyltransferase ALG9	68730.22		20584.13	25026.59	38114	20733.06	19094.61	19091.59	25201.81	21030	1.8123	0.24478
P01796;P01797;P01799;P01801		Ig heavy chain V-III region A4;Ig heavy chain V-III regi	14646.9	10984.25	8849.571		11494	17053.6	13370.09			15212	0.7556	0.24530
Q9Z223	Mocs2	Molybdopterin synthase catalytic subunit	23579.4	22092.5	23024.25	21604.18	22575	23471.27	22200.21	24519.76	23431.63	23406	0.9645	0.24913
Q9JHJ3	Glmp	Glycosylated lysosomal membrane protein	17797.47	15176.89	14922.65	17540.4	16359	15501.66	14519.74	9064.056	16848.67	13984	1.1699	0.25077
P28184	Mt3	Metallothionein-3	6666.617	5971.514	7126.113	7455.497	6805	6347.21	4639.688	5946.374	7157.41	6023	1.1299	0.25105
A2AQ07	Tubb1	Tubulin beta-1 chain	34165.32	79629.69	82864.43	66469.02	65782	26549.34	26557.8	46645.49	78915.71	44667	1.4727	0.25109
Q8BGT7	Smndc1	Survival of motor neuron-related-splicing factor 30	16313.27	11676.73	13739.94	11674.48	13351	8571.245	4568.516	12876.5	14635.31	10163	1.3137	0.25142
O70551	Srpk1	SRSF protein kinase 1	9155.701	8183.706	10103.53	9903.26	9337	10802.87	9246.472	9500.924	10886.03	10109	0.9236	0.25253
Q99MJ9	Ddx50	ATP-dependent RNA helicase DDX50	27619.05	24686.16	32833.68	30600.8	28935	31929.16	27926.84	31773.24	37132.44	32190	0.8989	0.25577
Q60596	Xrcc1	DNA repair protein XRCC1	12128.8	12324.81	17107.77	9202.992	12691	14032.2		15854.48	15872.15	15253	0.8320	0.25686
P18528		Ig heavy chain V region 6.96	29091.38	21871.27		21770.61	24244	25086.73	17597.54	5375.195		16020	1.5134	0.25758
Q9CR25	Dph2	2-(3-amino-3-carboxypropyl)histidine synthase subu	8161.791	7587.877	7414.183	2890.651	6514	5894.909	3204.101	4381.935		4494	1.4495	0.25768
Q80XC3	Usp6nl	USP6 N-terminal-like protein	11877.59	11471.93	12241.18	13595.19	12296	12003.37	12664.83	7144.916	10799.74	10653	1.1543	0.25790
Q6PB93	Galnt2	Polypeptide N-acetylgalactosaminyltransferase 2	16546.67	12010.22	16098.68	12181.81	14209	12147.51	10171.97	8112.904	16031.64	11616	1.2233	0.25976
Q8BZQ7	Anapc2	Anaphase-promoting complex subunit 2	13673.98	11647.54	12029.4	7677.489	11257	8989.676	10205.92	10546.92	8572.25	9579	1.1752	0.26233
Q8K4P0	Wdr33	pre-mRNA 3' end processing protein WDR33	4566.247	6094.612	7677.88	8653.551	6748	5048.185	12125.59	9112.871	9142.06	8857	0.7619	0.26313
Q8R0W6	Ndfip1	NEDD4 family-interacting protein 1	8077.16	10904.52	11359.51	11323.72	10416	11762.5	9881.123	15666.29	11537.32	12212	0.8530	0.26366
D3YU32	Tex13c1	Testis-expressed protein 13C-1	9281.081	7402.394	6726.084	8710.522	8030	8585.613	6937.648	4216.177	7073.031	6703	1.1980	0.26592
Q8R409	Hexim1	Protein HEXIM1	10239.62	9653.444	6483.624	9371.406	8937	11293.17	9414.826	10609.47	9178.172	10124	0.8828	0.26938
Q8BVU0	Lrch3	DISP complex protein LRCH3	19454.98	19523.58	20621.61	18789.31	19597	19902.76	18899.31	21314.52	22609.72	20682	0.9476	0.27165
Q80YQ8	Rmnd5a	E3 ubiquitin-protein ligase RMND5A	6001.08	6238.842	6773.25	6169.344	6296	7789.619	6515.884	5699.774	7776.576	6945	0.9064	0.27256
Q80UK8	Ints2	Integrator complex subunit 2	26168.06	32346.93	24553.88	23718.54	26697	29178.56	36024.91	28384.71	26796.94	30096	0.8870	0.27350
Q9D8T4	Tvp23b	Golgi apparatus membrane protein TVP23 homolog I	14409.21	19199.83	19759.84	21452.43	18705	15466.27	18337.07	13297.96	18345.38	16362	1.1432	0.27353
Q08274	Dmwd	Dystrophia myotonica WD repeat-containing protein	8687.884		10885.3	15751.84	11775	11666.59		18819.53	16048.55	15512	0.7591	0.27378
Q3TEW6	Mpzl1	Myelin protein zero-like protein 1	10783.62	13820.71	21548.79	16460.91	15654	16670.58	16515.71	20210.56	21907.46	18826	0.8315	0.27537
Q8R2T8	Gtf3c5	General transcription factor 3C polypeptide 5	6258.856	6207.515	9194.686	8418.418	7520	6309.613	4137.246	6442.408	7952.296	6210	1.2109	0.27584
Q7TS72	Itpkc	Inositol-trisphosphate 3-kinase C	16401.91	17263.64	17072.08	14687.9	16356	17287.22	13595.85	13982.21	15645.42	15128	1.0812	0.27760
O35153	Bet1l	BET1-like protein	37253.5	39267.36	22889.55	25518.64	31232	34110.75	24815.72	16634.2	23377.27	24734	1.2627	0.27939
P22935	Crabp2	Cellular retinoic acid-binding protein 2	17905.06	14277.66	14874.97	13484.75	15136	14931.06	11763.08	15220.11	12467.02	13595	1.1133	0.28060
Q62009	Postn	Periostin	36055.16	43911.66	44007.59	33515.56	39372	35781.89	33243.57	37597.29	37276.56	35975	1.0944	0.28207
P37889	Fbln2	Fibulin-2	16112.84	20063.63	24880.82	18754.88	19953	20322.38	16485.84	9579.611	19152.05	16385	1.2178	0.28314

Q8CI70	Lrrc20	Leucine-rich repeat-containing protein 20	12509.75	12538.05	12089.38	9291.685	11607	13482.13	9171.957	7733.047	9145.504	9883	1.1744	0.28506
P61957	Sumo2	Small ubiquitin-related modifier 2	7068.194	3314.049	5200.769		5194	3319.71	4634.288	2709.84	4872.999	3884	1.3373	0.28531
P0C7L0	Wipf3	WAS/WASL-interacting protein family member 3		8196.068		10737.08	9467			26645.19	12720.91	19683	0.4810	0.28566
Q6PCZ4	Magee1	Melanoma-associated antigen E1	27295.49	28371.86	23399.09	21879.59	25237	23325.22	26064.82	22056.94	20301.51	22937	1.1002	0.28647
Q99MQ4	Aspn	Asporin	142735.8	153424.8	93533.96	131866.6	130390	131383.5	135452.3	72728.94	86002.88	106392	1.2256	0.28693
Q9Z0E6	Gbp2	Guanylate-binding protein 2	36740.25	13913.2	6321.996	7464.786	16110	10979.28	10575.86	4169.942	4537.813	7566	2.1293	0.28719
P01644;P01645		Ig kappa chain V-V region HP R16.7;Ig kappa chain V-	38952.72	23738.28			18623	45327.66	24987.05			35157	0.5297	0.28772
P28293	Ctsg	Cathepsin G	18700.27	4448.183	7217.005	20179.29	12636	3838.042	10073.46	6413.218		6775	1.8651	0.28928
Q3UPR9	Sbspon	Somatomedin-B and thrombospondin type-1 domain	230212.6	216362.3	212954.5	263508.1	230759	200987.6	193299.6	216672.1	240177.5	212784	1.0845	0.29011
Q8BG18	Necab1	N-terminal EF-hand calcium-binding protein 1	43501.37	50677.13	53287.8	34489.93	45489	43986.72	49449.8	30792.43	24925.84	37289	1.2199	0.29040
Q9D2U5	Naa38	N-alpha-acetyltransferase 38, NatC auxiliary subunit	11089.52	14691.36	12048.72	13346.44	12794	11470.51	12267.42	4473.363	13105.47	10329	1.2386	0.29110
Q8VE28	Nkd2	Protein naked cuticle homolog 2	35029.71	35434.32	31617.62	31073.06	33289	33201.99	34297.27	28713.45	27936.5	31037	1.0725	0.29234
Q8K2I3	Fmo2	Dimethylaniline monooxygenase [N-oxide-forming] 2	32667.45	28823.44	24673.24	33379.12	29886	27760.21	28992.3	25086.69	27704.15	27386	1.0913	0.29251
Q9CXI3	Moxd1	DBH-like monooxygenase protein 1	6894.627	7137.183	7570.724	8690.075	7573	7780.502	7924.434	9228.162	7795.443	8182	0.9256	0.29419
P50285	Fmo1	Flavin-containing monooxygenase 1	72484.91	71267.23	74480.41	85661.37	75973	69890.2	77123.84	63279.27	73358.77	70913	1.0714	0.29571
P27641	Xrcc5	X-ray repair cross-complementing protein 5	11009.37	10107.13	13337.54	10412.68	11217	10682.38	10501.14	10192.83	10092.09	10367	1.0819	0.29699
Q8K2A1	Gulp1	PTB domain-containing engulfment adapter protein 1	30625.55	28819.27	35476.57	33561.22	32121	32030.48	32142.36	25675.52	28892.87	29685	1.0820	0.29755
Q80WW9	Ddrgk1	DDRGK domain-containing protein 1	9445.52	7952.618	7445.594	9267.015	8528	7830.421	8464.954	11673.17	10956.86	9731	0.8763	0.29779
Q9CQJ2	Pih1d1	PIH1 domain-containing protein 1	19709.23	19655.21	18359.27	6825.091	16137	12053.98	17942.63	5919.95	10474.07	11598	1.3914	0.29829
Q64435	Ugt1a6	UDP-glucuronosyltransferase 1-6	71058.48	72103.08	68539.04	70250.59	70488	63169.87	74687.42	65427.27	66779.88	67516	1.0440	0.29900
O89090	Sp1	Transcription factor Sp1	4188.126		2194.832		3191	2479.306		2423.518	734.9922	1879	1.6983	0.29918
Q00724	Rbp4	Retinol-binding protein 4	26423.78	24734.51	26246.19	15443.24	23212	19811.43	21565.4	18295.3	20867.91	20135	1.1528	0.29986
Q6GUQ1	Egfl8	Epidermal growth factor-like protein 8	17302.09	17157.81	15459.77	16776.86	16674	16771.89	17830.89	12143.76	13681.64	15107	1.1037	0.30199
Q3UH53	Sdk1	Protein sidekick-1			6290.983	6007.126	6149	5703.85	3550.577	5569.141	5890.88	5179	1.1874	0.30450
Q9D8T2	Gsdmd	Gasdermin-D	5859.524	3289.153			4574	3231.721		2047.676		2640	1.7329	0.30493
Q62074	Prkci	Protein kinase C iota type	7823.114	7899.483	8036.139	7329.552	7772	6679.346	8019.979	4085.762	8067.047	6713	1.1578	0.30554
Q5SVQ0	Kat7	Histone acetyltransferase KAT7	12072.55	14424.52	15780.88	14396.37	14169	16597.69	13101.14	7817.344	9916.847	11858	1.1948	0.30620
Q8C7E7	Stbd1	Starch-binding domain-containing protein 1	47306.23	44955.2	45703.91	49754.93	46930	45825.11	48336.92	42627.18	43486.71	45069	1.0413	0.30625
Q9DBF7	Cwc25	Pre-mRNA-splicing factor CWC25 homolog	11414.84		7367.556	10173.45	9652	6726.627	8279.787	6577.54	10409.56	7998	1.2067	0.30705
Q8BS95	Gpr89	Golgi pH regulator	25822.84	22702.33	21645.55	23274.18	23361	19723.13	23696.82	14883.87	24535.63	20710	1.1280	0.30772
Q9CZR2	Naalad2	N-acetylated-alpha-linked acidic dipeptidase 2	14367.67	12252.52	14354.04	15386.89	14090	14645.6	13690.94	11192.77	12364.45	12973	1.0861	0.30784
Q62356	Fstl1	Follistatin-related protein 1	23339.19	27987.68	24812.77	29034.6	26294	25942.97	34052.51	26619.13	28674.79	28822	0.9123	0.30787
Q9EPS3	Glce	D-glucuronyl C5-epimerase	6543.843	5859.019	4189.53	4084.92	5169	5963.596	5312.542	6610.856	5766.854	5913	0.8742	0.30864
Q3UM29	Cog7	Conserved oligomeric Golgi complex subunit 7	16244.4	15006.06	15096.85	13694.95	15011	14665.39	14154.4	14125.43	14688.41	14408	1.0418	0.31063
Q62084	Ppp1r14b	Protein phosphatase 1 regulatory subunit 14B	20393.46	17177.87	18764.01	8598.615	16233	17639.99	12200.33	11722.69	9505.578	12767	1.2715	0.31261
Q9R053	Scn11a	Sodium channel protein type 11 subunit alpha	32537.73	29613.17	26222	29457.51	29458	34537.52	29275.74	28875.17	33775.79	31616	0.9317	0.31333
Q6NXN1	Szrd1	SUZ domain-containing protein 1	3949.137	4474.894		4747.807	4391	3767.614	4568.306	7015.408	6260.124	5403	0.8126	0.31466
Q3TUA9	Pomk	Protein O-mannose kinase	17207.15	18062.88	17223.04	20744.82	18309	16915.38	24397.86	121770.3	20982.7	46017	0.3979	0.31565
Q3U319	Rnf40	E3 ubiquitin-protein ligase BRE1B	12575.22	11687.79	7985.068	8202.919	10113	9080.312	9460.709	8181.455	8408.146	8783	1.1514	0.31630
P16110	Lgals3	Galectin-3	47604.85	36433.9	31267.6	39136.34	38611	37441.79	38706.43	29395.19	30878.81	34106	1.1321	0.31725
Q6NXJ0	Wwc2	Protein WWC2	12628.26	13061.04	10310.65	12963.68	12241	10790.01	14637.01	18858.72	12716.13	14250	0.8590	0.31753
Q9DBB4	Naa16	N-alpha-acetyltransferase 16, NatA auxiliary subunit	2022.263	894.5889	1369.977		1429	1951.535			1909.639	1931	0.7402	0.32032
A2RSY6	Trmt1l	TRMT1-like protein	5246.108	7341.23	7103.545	7921.365	6903	6901.989	8037.469	8612.6	7106.158	7665	0.9006	0.32089
P36371	Tap2	Antigen peptide transporter 2	34619.29	15101.79	12006.44	15188.95	19229	14836.6	15722.9	9859.362	13429.73	13462	1.4284	0.32176
Q8C6U2	Slc66a3	Solute carrier family 66 member 3	10415.03	13260.88	13516.66	10524.92	11929		11922.07	9342.795	10646.03	10637	1.1215	0.32241
Q07646	Mest	Mesoderm-specific transcript protein	6610.936	6457.099	8396.877	6764.847	7057	6773.796	6156.452	9460.465	11514.54	8476	0.8326	0.32384
P06800	Ptprc	Receptor-type tyrosine-protein phosphatase C	21722.01	12459.32	11691.03	12584.38	14614	11684.85	14389.64	8484.93	12407.76	11742	1.2446	0.32426
O70624	Myoc	Myocilin	1610.733	1726.233		2161.781	1833	809.4711	1334.183	2034.791		1393	1.3160	0.32503
Q8CFB4	Gbp5	Guanylate-binding protein 5	39962.32	24521.4	22767.92	24013.89	27816	23052.84	26827.37	20706.82	22415.51	23251	1.1964	0.32559
Q8BW00	Ptrh1	Probable peptidyl-tRNA hydrolase	8519.111	11914.61	11904.36	10791.25	10782	12569.05	9982.846	16856.91	11037.9	12612	0.8549	0.32576
Q8VCB1	Ndc1	Nucleoporin NDC1	3488.622	3459.138	2927.56	3962.275	3459	2421.195	3252.702	2680.625	3852.343	3052	1.1336	0.32719
Q80W00	Ppp1r10	Serine/threonine-protein phosphatase 1 regulatory s	12970.25	9835.565	11415.1	10937.17	11290	10963.76	10957.79	6780.477	11152.67	9964	1.1331	0.32803
Q8BX09	Rbbp5	Retinoblastoma-binding protein 5	3875.724	3600.515	4876.41	3578.075	3983	3714.469	3988.714	3574.863	3158.103	3609	1.1035	0.32825
P43135	Nr2f2	COUP transcription factor 2	7370.028	10074.16	8303.245	4640.537	7597	6572.275	5989.565	6132.798	6817.161	6378	1.1911	0.32991
Q8BQ30	Ppp1r18	Phostensin	20234.44	16506.61	18494.15	16158.61	17848	16326.89	15717.3	18291.58	16397.92	16683	1.0698	0.32991
Q9DBU0	Tm9sf1	Transmembrane 9 superfamily member 1	19606.59	17402.22	20683.01	10899.13	17148	15126.84	16673.99	10001.64	15600.28	14351	1.1949	0.33147
P97298	Serpinf1	Pigment epithelium-derived factor	28145.57	29624.08	35084.46	11099.22	25988	21237.55	21859.61	19705.93	19097.56	20475	1.2693	0.33176
P01878		Ig alpha chain C region	17041.08	6287.502			11664	6291.846	2684.754			4488	2.5988	0.33321
Q3U3W5	Prmt9	Protein arginine N-methyltransferase 9	7216.138	3199.45		4712.199	5043	5412.836	8042.315		6295.892	6584	0.7659	0.33372

Q8BH97	Rcn3	Reticulocalbin-3	31999.41	35106.21	40083.39	21151.36	32085	24174.81	31743.26	25915.24	28330.38	27541	1.1650	0.33431
Q99KK1	Reep3	Receptor expression-enhancing protein 3	16908.59	17033.52	20822.54	20067.29	18708	19846.7	20178.41	18046.52	22413.58	20121	0.9298	0.33678
Q1EG27	Myo3b	Myosin-IIIb	35020.07	34236.54	27917.9	20830.78	29501	21883.01	32963.17	22507.52	23036.11	25097	1.1755	0.33692
Q91WT4	Dnajc17	DnaJ homolog subfamily C member 17	782.2939	1017.034	1320.826	2925.844	1511	1830.399			2939.712	2385	0.6337	0.33715
Q8BH61	F13a1	Coagulation factor XIII A chain	45004.59	45253.31	56899.8	36310.04	45867	40697.13	42500.36	43060.6	39213.78	41368	1.1088	0.33728
Q8BG07	Pld4	5'-3' exonuclease PLD4	10244.17	6453.109	4010.6		6903	7786.727	6572.837	713.629	1712.452	4196	1.6449	0.34037
Q9JHI7	Exosc9	Exosome complex component RRP45	8846.908	9391.517	11004.08	9518.952	9690	9678.641	9740.097	7887.97	8841.186	9037	1.0723	0.34234
Q9CTN4	Rhobtb3	Rho-related BTB domain-containing protein 3	5550.986	7132.294	10968.51	8345.664	7999	9240.509	7819.858	9500.354	10762.85	9331	0.8573	0.34271
Q9Z255	Ube2a	Ubiquitin-conjugating enzyme E2 A	24840.14	26036.44	26292.25	17451.03	23655	24302.86	24309.17	14177.22	18526.55	20329	1.1636	0.34294
P52293	Kpna2	Importin subunit alpha-1	9012.753	6729.308	5074.103	5358.825	6544	7643.869	7400.951	8151.904	6829.643	7507	0.8717	0.34512
Q9QY40	Plxnb3	Plexin-B3	8599.968	4887.384	6014.847	5375.608	6219	6293.594	3887.619	5329.198	5417.106	5232	1.1888	0.34557
Q640N1	Aebp1	Adipocyte enhancer-binding protein 1	45677.39	38995.92	40425.86	40698.92	41450	41393.46	41931.21	33627.5	39054.27	39002	1.0628	0.34572
Q3UR32	P2rx3	P2X purinoceptor 3	19386.19	22325.15	19963.55	16719.55	19599	20147.87	22316.52	23906.81	18689.7	21265	0.9216	0.34589
O35730;Q9CQJ4	Ring1;Rnf2	E3 ubiquitin-protein ligase RING1;E3 ubiquitin-protei	13162.92	7220.681	10771.88	9497.174	10163	7034.408	8935.132	8049.223	10663.56	8671	1.1721	0.34604
P21958	Tap1	Antigen peptide transporter 1	30368.2	18724.33	16061.62	13186.53	19585	16915.19	18885.02	10504.36	14977.64	15321	1.2784	0.34619
Q7TQ48	Srl	Sarcalumenin	19982.03	19793.11	13300.39	29240.53	20579		19459.31		9330.675	14395	1.4296	0.34743
Q9D6N1	Ca13	Carbonic anhydrase 13	5431.745	4217.846	3038.725	1877.767	3642	3705.85	2883.733	1029.288	3060.448	2670	1.3640	0.34880
Q8K2J0	Plcd3	1-phosphatidylinositol 4,5-bisphosphate phosphodi	18889.6	13456.48	12394.15	17033.29	15443	15336.63	13336.88	11885.65	14371.56	13733	1.1246	0.35000
P53808	Pctp	Phosphatidylcholine transfer protein	5505.013	9296.917	2041.345		5614	7855.324	9472.284			8664	0.6480	0.35154
Q61555	Fbn2	Fibrillin-2	25481.06	24141.66	27558.05	33865.16	27761	28991.49	22319.74	37507.65	41736.61	32639	0.8506	0.35308
Q68FE6	Ripor1	Rho family-interacting cell polarization regulator 1	3164.003		4390.439	4024.203	3860	2766.378	2507.066	2787.422	4665.081	3181	1.2131	0.35384
Q9EPM5	Sync	Syncoilin	25034.35	19489.06	16320.48	20707.3	20388	18720.42	21133.45	14115.16	18264.99	18059	1.1290	0.35393
P58022	Loxl2	Lysyl oxidase homolog 2	3459.479	3175.364	7080.185	5432.331	4787	5580.916	3784.67	6664.564	8559.82	6147	0.7787	0.35399
Q9CSH3	Dis3	Exosome complex exonuclease RRP44	9436.755	7196.311	7115.107	8884.534	8158	6722.812	7556.031	8131.79	7592.246	7501	1.0877	0.35606
P28076	Psmb9	Proteasome subunit beta type-9	12432.4	8780.756	4524.169	5701.204	7860	5072.149	5126.926			5100	1.5412	0.35695
Q6NVG7	Colgalt2	Procollagen galactosyltransferase 2	21615.74	17455.05	17868.16	16822.64	18440	15286.28	14812.46	19875.77	17504.73	16870	1.0931	0.36022
Q9CQT5	Pomp	Proteasome maturation protein	8135.513	8602.954	8136.854	6178.376	7763	5693.955	8725.63	5620.995	7373.195	6853	1.1328	0.36030
Q8CFQ3	Aqr	RNA helicase aquarius	8044.993	7417.316	8875.757	4403.228	7185	7298.33	7093.568	3239.443	5791.935	5856	1.2270	0.36257
E9PYK3	Parp4	Protein mono-ADP-ribosyltransferase PARP4	9109	8234.103	7031.021	8951.507	8331	8203.903	10504.85	8094.67	9424.427	9057	0.9199	0.36484
Q8BQM4	Heatr3	HEAT repeat-containing protein 3	24930.46	24715.22	25251.97	19781.72	23670	27348.07	28633.77	21716.76	24839.9	25635	0.9234	0.36484
P10854;Q64478;Q6452	H2bc14;H2bc9;Hist2h2bb;H	Histone H2B type 1-M;Histone H2B type 1-H;Histone	2036.86	1864.15		13492.95	5798	3291.493	907.928		1205.229	1802	3.2183	0.36563
Q91X88	Pomgnt1	Protein O-linked-mannose beta-1,2-N-acetylglucosa	85528.41	62746.04	72309.84	59359.91	69986	88581.52	66297.45	75310.66	78917.54	77277	0.9057	0.36595
Q61830	Mrc1	Macrophage mannose receptor 1	31431.62	26349.93	33767.77	28558.33	30027	27096.36	28034.56	28186.9	30008.27	28332	1.0598	0.36602
Q8VCT4	Ces1d	Carboxylesterase 1D	12187.15	7779.899	7310.175	7287.727	8641	8474.651	11126.4	3075.785	2468.113	6286	1.3746	0.36725
Q8CIB6	Tmem230	Transmembrane protein 230	9660.792	8398.245	8862.381	11177.7	9525	8061.055	10609.75	5642.739	9085.031	8350	1.1407	0.36831
Q64314	Cd34	Hematopoietic progenitor cell antigen CD34	25111.11	26051.12	27114	21525.9	24951	31306.46	24051.93	28542.34	24215.09	27029	0.9231	0.36918
Q80VI1	Trim56	E3 ubiquitin-protein ligase TRIM56	26762.75	21866.72	23103.09	22856.34	23647	23277.29	23990.72	17136.85	22795.73	21800	1.0847	0.36953
Q8R422	Cd109	CD109 antigen	3866.203	2196.225	2537.24		2867	2883.923	2246.636	1514.603		2215	1.2941	0.36959
Q5RKZ7	Mocs1	Molybdenum cofactor biosynthesis protein 1	3822.097	3795.138	5361.5	5005.131	4496	4653.108			5728.765	5191	0.8661	0.37022
P47759	Nsg2	Neuronal vesicle trafficking-associated protein 2	4620.999	2750.251	1476.966		2949	3981.946	2178.56	5416.734	4614.006	4048	0.7286	0.37060
O08811	Ercc2	General transcription and DNA repair factor IIH helic	8571.131	8750.293	9787.974	9867.056	9244	10168.22	9492.647	8756.45	10594.3	9753	0.9478	0.37086
Q80XL7	Mppe1	Metallophosphoesterase 1	5936.093	3578.308	5091.15	5903.55	5127	6181.76	3640.549	6937.699	7827.419	6147	0.8341	0.37171
Q8BZ00	Slc9a9	Sodium/hydrogen exchanger 9	17951.07	19172.9	15838.69	17291.77	17564	16635.01	19844.13	13693.68	13923.76	16024	1.0961	0.37223
Q61475	Cd55	Complement decay-accelerating factor, GPI-anchor	36598.02	33605.54	24611.59	35527.09	32586	38142.08	37582.1	32713.87	33644.85	35521	0.9174	0.37357
P28653	Bgn	Biglycan	310175.9	314793.7	290373.9	284684	300007	327951.2	304195.8	193147.2	257877.5	270793	1.1079	0.37643
Q6PIX5	Rhbdf1	Inactive rhomboid protein 1	3585.126	3702.987	3789.716	2499.684	3394	1666.472	2597.747	3890.65	3272.961	2857	1.1881	0.37729
Q9D945	Llph	Protein LLP homolog	3368.674	1817.865	5511.712	2246.265	3236	1642.048	1320.026	1019.314	4545.651	2132	1.5181	0.37788
Q8K4L3	Svil	Supervillin	14490.27	5686.739	8763.05	6995.579	8984	7945.618	5197.453	2278.538	10518.41	6485	1.3853	0.37857
P01654		Ig kappa chain V-III region PC 2880/PC 1229	73791.27	47119.42	9370.862	6562.12	34211	15522.5	23959.92	6646.779		15376	2.2249	0.37883
Q9QYH6	Maged1	Melanoma-associated antigen D1	10933.81	11723.87	10328.32	7403.932	10097	9814.702	11267.16	5259.184	7969.676	8578	1.1772	0.37942
Q62440;Q62441	Tle1;Tle4	Transducin-like enhancer protein 1;Transducin-like e	20309.75	21028.63	22873.8	19640.79	20963	22648.9	27997.29	26242.61	16743.92	23408	0.8956	0.38000
Q9Z2R6	Unc119	Protein unc-119 homolog A	12951.56	12297.7		3847.852	9699	7878.958		7776.149	4024.255	6560	1.4786	0.38135
P97801	Smn1	Survival motor neuron protein	32890.71	34154.54	29392.11	43502.44	34985	40497.91	35171.02	36016.21	40933.74	38155	0.9169	0.38209
P11688	Itga5	Integrin alpha-5	5986.274			6241.595	6114	6361.304	6201.321			6281	0.9734	0.38223
F6W8I0	Yjefn3	YjeF N-terminal domain-containing protein 3	10252.79	6988.477	14497.86	12206.34	10986	10401.25	11821.94	11826.51	20335.56	13596	0.8080	0.38287
Q8VDB2	Alg12	Dol-P-Man:Man(7)GlcNAc(2)-PP-Dol alpha-1,6-man	19655.6	11635.57	15246.29	15968.55	15627	17788.35	19524.08	15206.95	17012.09	17383	0.8990	0.38430
Q9Z2B9	Rps6ka4	Ribosomal protein S6 kinase alpha-4	12530.14	8778.033	5965.895		9091	12636.56	3326.99	3364.996	5417.045	6186	1.4696	0.38540
Q99K41	Emilin1	EMILIN-1	48494.75	43761.06	44552.07	42908.89	44929	44625.46	39634.42	40393.2	47043.63	42924	1.0467	0.38673
P97792	Cxadr	Coxsackievirus and adenovirus receptor homolog	18284.86	12217.61	11340.46	15160.91	14251	12774.69	13051.65	12618.39	12678.14	12781	1.1150	0.38696

Q8K377	Lrrtm1	Leucine-rich repeat transmembrane neuronal protei	5051.27	6899.977	5518.841	7710.127	**6295**	6609.59	7769.45	6445.71	6893.226	**6929**	**0.9084**	**0.38732**
D3KU66	Asmt	Acetylserotonin O-methyltransferase	98629.31	73370.96	118375.7	124484.5	**103715**	101650.6	48817.74	99945.76	100193.4	**87652**	**1.1833**	**0.38983**
Q02780	Nfia	Nuclear factor 1 A-type	10225.67	10337.21	10994.52	10916.27	**10618**	10066.63	10765.54	8595.203	10943.33	**10093**	**1.0521**	**0.39102**
Q9DD23	Lypd2	Ly6/PLAUR domain-containing protein 2	8389.617	8877.148	7510.262	10195.4	**8743**	9213.734	8746.322		3760.988	**7240**	**1.2076**	**0.39170**
Q9ER60	Scn4a	Sodium channel protein type 4 subunit alpha	79793.91	79522.09	54450.79	74017.63	**71946**	57833.29	80912.97	27205.88	72946.34	**59725**	**1.2046**	**0.39269**
Q9DB76	Emc9	ER membrane protein complex subunit 9	8335.188	12319.87	10675.5	6419.4	**9437**	8512.159	11059.26	4869.545	6485.454	**7732**	**1.2206**	**0.39485**
Q91XC8	Dap	Death-associated protein 1	7953.593	10901.22	10507.04	1787.537	**7787**	8640.808	5150.044	869.4241		**4887**	**1.5936**	**0.39516**
O70433	Fhl2	Four and a half LIM domains protein 2	9289.18	8164.236	8837.054	5836.409	**8032**	8361.824	7420.242		4737.579	**6840**	**1.1742**	**0.39602**
Q2HXL6	Edem3	ER degradation-enhancing alpha-mannosidase-like p	14928.41	14340.62	12075.36	9372.83	**12679**	14350.7	13642.56	7099.672	6934.408	**10507**	**1.2068**	**0.39694**
Q9CZE3	Rab32	Ras-related protein Rab-32	21736.29	17903.68	19363.56	19570.36	**19643**	23056.46	16063.15	15763.6	16792.45	**17919**	**1.0962**	**0.39865**
Q64345	Ifit3	Interferon-induced protein with tetratricopeptide rep	17636.82	7597.104	2727.314	5008.742	**8242**	6647.157	8226.613	3106.477	2012.964	**4998**	**1.6491**	**0.40173**
Q9CW46	Raver1	Ribonucleoprotein PTB-binding 1	11274.58	11121.6	12412.01	12465.57	**11818**	10571.29	9605.252	11537.54	12792.42	**11127**	**1.0622**	**0.40369**
Q3ZK22	Vezt	Vezatin	10228.85	6314.882	7380.174	4574.24	**7125**	9040.156	4620.583	5140.426	3753.756	**5639**	**1.2635**	**0.40657**
P40936	Inmt	Indolethylamine N-methyltransferase	3400.804	5003.237	4694.743	4957.943	**4514**	3312.246	4480.225			**3896**	**1.1586**	**0.40823**
Q8BVD5	Mpp7	MAGUK p55 subfamily member 7	6660.964	9836.301	8682.715		**8393**	8229.332	7852.042	5787.688		**7290**	**1.1514**	**0.40933**
Q9CQ71	Rpa3	Replication protein A 14 kDa subunit	22812.15	26046.22	27291.03	23339.1	**24872**	26308.81	23538.92	25337.09	31245.11	**26607**	**0.9348**	**0.41169**
Q61142	Spin1	Spindlin-1	8460.333	11814.29	11790.13	8170.634	**10059**	10911.88	7131.142	8833.215	8881.485	**8939**	**1.1252**	**0.41222**
P10417	Bcl2	Apoptosis regulator Bcl-2	23783.82	23324.83	26646.09	16851.09	**22651**	18399.26	22659.46	22327.16	18977.12	**20591**	**1.1001**	**0.41350**
P04945		Ig kappa chain V-VI region NQ2-6.1	45916.75	11423.97			**28670**	12572.27	9498.708			**11035**	**2.5980**	**0.41560**
P70665	Siae	Sialate O-acetylesterase		6839.722	4735.259	5637.547	**5738**		6346.854	1264.629		**3806**	**1.5076**	**0.41575**
Q8R1S4	Mtss1	Protein MTSS 1	30176.88	28351.36	22553	29043.98	**27531**	23885.23	27629.57	19220.67	29493.93	**25057**	**1.0987**	**0.41649**
P26262	Klkb1	Plasma kallikrein	15955.57	12821.4	11722.47	5709.732	**11552**	10237.11	12254.83	3929.562	10031.04	**9113**	**1.2677**	**0.41701**
P06683	C9	Complement component C9	8739.506	4351.776	21689.09	10179.79	**11240**	4313.577	2461.384	12330.94	10549.29	**7414**	**1.5161**	**0.41779**
Q9JKY0	Cnot9	CCR4-NOT transcription complex subunit 9	8986.717	8122.268	9303.223	6960.404	**8343**	7738.442	8359.522	9073.694	11755.05	**9232**	**0.9038**	**0.42058**
P24526	Pmp2	Myelin P2 protein	1349573	1589805	1867650	1809215	**1654060**	1550273	1181228	1442298	1827492	**1500323**	**1.1025**	**0.42154**
Q8C170	Myo9a	Unconventional myosin-IXa	14087.29	13511.62	13419.57	14207.28	**13806**	14011.01	14682.26	12282.01	11772.61	**13187**	**1.0470**	**0.42226**
Q8R2Q8	Bst2	Bone marrow stromal antigen 2	78794.38	41811	42985.35	38372.68	**50491**	34929.53	60661.33	21230.82	42224.93	**39762**	**1.2698**	**0.42532**
Q91X78	Erlin1	Erlin-1	19853.47	20765.95	18397.33	20998.96	**20004**	18746.8	21825.97	14879.86	19266.47	**18680**	**1.0709**	**0.42597**
Q9D0Y8	Mrpl52	Large ribosomal subunit protein mL52	5449.255	4325.034	2571.226	8674.867	**5255**	4395.704	5264.63	2157.756	4276.415	**4024**	**1.3061**	**0.42651**
Q69ZN7	Myof	Myoferlin	60511.13	57111.14	62178.56	55069.5	**58718**	63786.46	55474.57	60679.38	55924.25	**55466**	**1.0586**	**0.43058**
Q9QZK2	Bcar3	Breast cancer anti-estrogen resistance protein 3 hor	8502.698	8520.153	11656.04	9666.255	**9586**	8634.19	10617.6	8445.671	7178.077	**8719**	**1.0995**	**0.43081**
Q9DBG1	Cyp27a1	Sterol 26-hydroxylase, mitochondrial	12768.08	12664.18	13574.44	17250.24	**14064**	14806.21	11179.4		12206.08	**12731**	**1.1048**	**0.43330**
Q9D666	Sun1	SUN domain-containing protein 1	19777.71	18562.35	15098.98	16152.62	**17398**	18273.63	19816.57	17308.96	18191.75	**18398**	**0.9457**	**0.43452**
Q9JLC4	Sorcs1	VPS10 domain-containing receptor SorCS1	18272.64	19114.51	11394.09	15260.73	**16010**	12316.25	19725.82	8428.82	13783.81	**13564**	**1.1804**	**0.43470**
P63271;Q9Z199	Supt4h1a;Supt4h1b	Transcription elongation factor SPT4-A;Transcription	17193.36	18954.77	19040.28	11451.53	**16660**	19453.52	18428.89	18842.74	16313.71	**18260**	**0.9124**	**0.43510**
Q8BRH0	Tmtc3	Protein O-mannosyl-transferase TMTC3	15761.78	14960.92	11310.86	14684.56	**14180**	13014.41	14840.85	9401.863	14236.47	**12873**	**1.1015**	**0.43597**
Q3V0C5	Usp48	Ubiquitin carboxyl-terminal hydrolase 48	25470.38	22078.49	17871.61	24374.46	**22449**	21400.77	20794.24		20030.67	**20742**	**1.0823**	**0.43603**
Q8BX02	Kank2	KN motif and ankyrin repeat domain-containing prote	42109.04	39774.2	42543.04	42687.23	**41778**	42774.12	41136.38	41459.97	46254.95	**42906**	**0.9737**	**0.43663**
P97386	Lig3	DNA ligase 3	1718.27		968.583	1975.969	**1554**	2300.261	1635.036			**1968**	**0.7899**	**0.43702**
Q8BQ47	Cnpy4	Protein canopy homolog 4	35233.45	31044.51	35631.34	30984.99	**33224**	36057.56	31066.41	32245.25	44470.38	**35960**	**0.9239**	**0.43723**
Q8BGT0	Ostm1	Osteopetrosis-associated transmembrane protein 1		14346.69	10568.78	8302.04	**11073**	12264.61	9981.523	8359.263	6947.642	**9388**	**1.1794**	**0.43816**
P0DOV1;Q8CGE8	Mnda;Ifi205a	Interferon-activable protein 205-B;Interferon-activab	13529.36	11884.16	7011.949	8745.395	**10293**	11926.49		6168.166	6973.974	**8356**	**1.2317**	**0.43948**
Q9QXT5	Egfl7	Epidermal growth factor-like protein 7		7767.681	10764.28	14310.47	**10947**	9124.509	7790.576	8039.662	12183.84	**9285**	**1.1791**	**0.43972**
A6H8H5;Q03717	Kcnb2;Kcnb1	Potassium voltage-gated channel subfamily B memb	38258.79	37838.55	40564.74	39020.24	**38921**	34760.74	39367.52	41105.29	34756.25	**37497**	**1.0380**	**0.44173**
Q9Z247	Fkbp9	Peptidyl-prolyl cis-trans isomerase FKBP9	149184.4	183659.4	219964.7	186010.8	**184705**	182266.2	176287.1	221753.1	220698.7	**200251**	**0.9224**	**0.44218**
Q91ZP6	Ndfip2	NEDD4 family-interacting protein 2 (Fragment)	12939.79	16651.81	15590.06	16308.67	**15373**	14844.66	15560.9	17104.18	17391.5	**16225**	**0.9474**	**0.44325**
P62984	Uba52	Ubiquitin-ribosomal protein eL40 fusion protein	2536.404	3436.585	2878.58	4167.594	**3255**	2486.604	3821.007	2551.781	2589.482	**2862**	**1.1372**	**0.44393**
Q9CWU9	Nup37	Nucleoporin Nup37	18342.97	17397.01	17170.82	11277.43	**16047**	18708.64	13397.57	8820.113	14774.21	**13925**	**1.1524**	**0.44542**
P01843		Ig lambda-1 chain C region	21850.47	11881.42			**16866**	5747.389	15152.97			**10450**	**1.6139**	**0.44799**
Q9D975	Srxn1	Sulfiredoxin-1	14281.33	13558.59	14305.55	13405.92	**13888**	12758.88	15954.09	12851.36	9954.4	**12880**	**1.0783**	**0.45008**
P22227;Q00899;Q3TTC2	Zfp42;Yy1;Yy2	Zinc finger protein 42;Transcriptional repressor prote	6738.386	9001.63	11357.93	10894.03	**9498**	15566	9463.232	6840.227	13243.19	**11278**	**0.8422**	**0.45088**
Q9DCB1	Hmgn3	High mobility group nucleosome-binding domain-coi	14106.83	24472	19972.38	18244.11	**19199**		25563.37	18786.64	20710.99	**21687**	**0.8853**	**0.45145**
Q03311	Bche	Cholinesterase	9068.518	7182.871	8006.756	5619.093	**7469**	8839.011	6523.769	2670.536	7073.066	**6277**	**1.1900**	**0.45376**
Q9Z0F8	Adam17	Disintegrin and metalloproteinase domain-containin	11331.89	14716.17	13412.17	13964.54	**13356**	11266.22	15720.87	16289.85	14409.15	**14422**	**0.9261**	**0.45597**
Q8BMZ5	Tsen34	tRNA-splicing endonuclease subunit Sen34	9719.264	6812.439	11898.53	7422.658	**8963**	8136.976	6330.491	9480.907	7665.607	**7903**	**1.1341**	**0.45628**
Q6P9Z1	Smarcd3	SWI/SNF-related matrix-associated actin-dependent	8455.907	7586.649	8648.151	9448.337	**8535**	10148.79	9009.049	6426.132	4057.893	**7410**	**1.1517**	**0.45704**
P16092	Fgfr1	Fibroblast growth factor receptor 1			8691.725	6669.321	**7681**		9561.563	8086.913		**8824**	**0.8704**	**0.45724**
P70444	Bid	BH3-interacting domain death agonist	6491.875	9591.98	7223.985	7839.112	**7787**	11598.24	4663.226	1457.998	6416.689	**6034**	**1.2905**	**0.46002**
Q00422	Gabpa	GA-binding protein alpha chain	5319.938	3742.946	3259.116	1508.803	**3458**	2716.323	2574.848	4280.274	1028.17	**2650**	**1.3048**	**0.46194**

Q9JJR9	Nrip3	Nuclear receptor-interacting protein 3	6108.573	8565.965	10275.36	7326.461	8069	7732.318	10860.08		8720.824	9104	0.8863	0.46390
Q8CGC4	Lsm14b	Protein LSM14 homolog B	17152.48	17558.45	16445.2	16341.48	16874	18309.9	16989.5	14391.33	14757.31	16112	1.0473	0.46403
Q921I9	Exosc4	Exosome complex component RRP41	10860.07	10507.99	4631.761	5282.717	7821	7355.009	12710.17	7456.82	10293.82	9454	0.8272	0.46566
Q3TJD7	Pdlim7	PDZ and LIM domain protein 7	16176.42	11856.03	16773.01	16495.5	15325	12537.64	11499.66	16601.69	15458.35	14024	1.0928	0.46586
Q9D9I4	Tbc1d20	TBC1 domain family member 20	6784.313		10569.84	6544.001	7966	7765.239	7003.741	5566.417	7620.715	6989	1.1398	0.46715
Q03347	Runx1	Runt-related transcription factor 1	3706.677	2349.467		3779.286	3278	5287.769	5734.859	1919.322		4314	0.7600	0.46742
Q99PP9	Trim16	Tripartite motif-containing protein 16	24536.4	33816.99	28348		28900	30753.49	42435.8	27291.03	29570.42	32513	0.8889	0.46744
Q8CD26	Slc35e1	Solute carrier family 35 member E1	13585.6	12103.89	11919.18	12312.25	12480	8911.089	10151.67	10629.52	15558.34	11313	1.1032	0.46838
Q9JJY4	Ddx20	Probable ATP-dependent RNA helicase DDX20	25375.25	11993.69	22761.1	35030.19	23790	19834.53	25017.04	11927.26	21433.27	19553	1.2167	0.46896
O35704	Sptlc1	Serine palmitoyltransferase 1	45996.59	37157.63	41538.42	30011.53	38676	28914.97	42239.4	21064.83	42935.98	33789	1.1446	0.46897
Q9R233	Tapbp	Tapasin	66661.83	27041.81	22715.54	28533.28	36238	31758.85	33716.82	21534.09	25175.08	28046	1.2921	0.46907
Q8CBY0	Gatc	Glutamyl-tRNA(Gln) amidotransferase subunit C, mi	3831.667		5656.364		4744		4817.152		7626.863	6222	0.7625	0.47067
Q8K0L9	Zbtb20	Zinc finger and BTB domain-containing protein 20	7583.277	6079.604	8584.766	9008.386	7814	8078.092	7955.193	7940.404	9667.179	8410	0.9291	0.47068
P06330		Ig heavy chain V region AC38 205.12	46122.07	21792.13	10870.65	13971	23189	32168.48	18602.54	4486.299	6131.404	15347	1.5110	0.47330
Q9CQG2	Mettl16	RNA N6-adenosine-methyltransferase METTL16	9207.142	9069.538	7098.322	5742.07	7779	9146.637	5240.236	6538.354	6636.278	6890	1.1290	0.47481
Q9QY06	Myo9b	Unconventional myosin-IXb		4055.351	5714.11	4250.272	4673	4775.466		4730.583	6177.654	5228	0.8939	0.47653
Q9DCN1	Nudt12	NAD-capped RNA hydrolase NUDT12	9226.576	9090.119	7526.563	8040.432	8471	7321.12	8449.546	7051.753	9128.178	7988	1.0605	0.47664
Q9WVL1	Ap4s1	AP-4 complex subunit sigma-1	5190.663	7623.642	10354.1	6671.661	7460	7982.473	7290.013	8555.875	9634.723	8366	0.8917	0.47704
Q9JLV2	Trpc4ap	Short transient receptor potential channel 4-associa	4982.946	5761.563	7077.28	5177.968	5750	6499.453	5415.078	7108.383	5804.988	6207	0.9264	0.47723
P35330	Icam2	Intercellular adhesion molecule 2	12383.83	9320.412	10692.1	5393.537	9447	11241.3	12646.42	8275.877	10847.94	10753	0.8786	0.48297
Q9EQS3	Mycbp	c-Myc-binding protein	30612.45	30372.49	30155.66	34829.79	31493	26838.75	35924.53	24351.45	30636.44	29438	1.0698	0.48398
Q3U962	Col5a2	Collagen alpha-2(V) chain	67818.02	69389.65	52337.51	75477.87	66256	58023.44	65863.06	59956.54	65406.77	62312	1.0633	0.48502
Q8VC98	Plekha4	Pleckstrin homology domain-containing family A mer	23555.55	28671.48	32528.34	40808.04	31391	26193.21	27607.29	47034.61	44203.16	36260	0.8657	0.48509
Q60953	Pml	Protein PML	10607.8	7133.534	6109.113	4659.577	7128	7370.977	7244.328	4994.991	4542.774	6038	1.1804	0.48559
Q91WG7	Dgkg	Diacylglycerol kinase gamma	24875.21	24453.45	17681.47	22595.3	22401	23842.77	25275.37	25629.66	20754.84	23876	0.9383	0.48632
P28654	Dcn	Decorin	669485.5	606598.3	526544.8	634014.4	609161	603472.7	665206	486083	534707.1	572367	1.0643	0.48633
Q8K114	Ints9	Integrator complex subunit 9	11499.09	2745.734	7398.929	3685.04	6332	8164.7	6967.262	72.18777	1847.866	4263	1.4854	0.48681
P11589	Mup2	Major urinary protein 2		14614.69	30841.3	14324.32	19927	10216.22	4967.093	19211.51	24570.36	14741	1.3518	0.48783
Q571I9	Aldh16a1	Aldehyde dehydrogenase family 16 member A1	9924.729	9994.693	13641.62	3289.676	9213	10837.88	10317.24	3898.484	2959.074	7003	1.3155	0.48824
Q2QI47	Ush2A	Usherin	10345.43	12540.27	11481.96	9422.246	10947		12790.89	5496.375	10169.18	9485	1.1541	0.48859
Q9ET54	Palld	Palladin	14952.87	9145.735	15740.98	10806.69	12662	8199.455	13480.4	11202.34		10961	1.1552	0.48928
Q6PAV2	Herc4	Probable E3 ubiquitin-protein ligase HERC4	5049.73	5488.042	7598.495	5971.54	6027	4045.36	7716.421	5313.605	3984.194	5265	1.1447	0.48938
Q8BK62	Olfml3	Olfactomedin-like protein 3	35061.61	30194.16	31470.08	28408.69	31284	33330.05	30605.5	28356.24	27081.14	29843	1.0483	0.49120
P14483	H2-Ab1	H-2 class II histocompatibility antigen, A beta chain	66340.32	21781.29	28860.79	20746.79	34432	28891.97	29122.79	18891.88	28374.7	26320	1.3082	0.49129
Q9CTG6	Atp13a2	Polyamine-transporting ATPase 13A2	5135.787	5883.359		5598.06	5539	8156.219	9280.386	4621.646	4449.642	6627	0.8358	0.49145
Q01149	Col1a2	Collagen alpha-2(I) chain	1032062	1131050	910416.4	1740564	1203523	1010879	1124283	1685478	1776570	1399302	0.8601	0.49208
Q9EPK6	Sil1	Nucleotide exchange factor SIL1	5660.354		5450.923	7861.221	6324		7898.051		6498.181	7198	0.8786	0.49341
Q9JJH1	Rnase4	Ribonuclease 4	16185.75	10606.69	11019.37	9327.709	11785	12707.55	12269.9	8278.29	8205.326	10365	1.1370	0.49350
O55226	Chad	Chondroadherin	12216.22	9817.294	12295.94	4248.262	9644	9212.838	21756.78	7603.149		12858	0.7501	0.49418
Q8BJ64	Chdh	Choline dehydrogenase, mitochondrial	36878.82	32808.58	31390.47	40001.88	35270	35612.28	34723.09	30569.3	33654.71	33640	1.0485	0.49546
P08074	Cbr2	Carbonyl reductase [NADPH] 2	50125.55	50257.45	76794.59	46461.98	55910	54088.45	59649.32	63957.11	68069.94	61441	0.9100	0.49571
Q9JI48	Plac8	Placenta-specific gene 8 protein	13784.79			1497.266	7641	524.7816		4268.733		2397	3.1881	0.49998
Q9R061	Nubp2	Cytosolic Fe-S cluster assembly factor NUBP2	12154.34	9020.754	10664	8358.769	10049	10954.97	8955.178	7749.601	9440.538	9275	1.0835	0.50040
P97287	Mcl1	Induced myeloid leukemia cell differentiation proteir	2109.457	1661.712	1678.218		1816	1747.065	1454.746	4008.152	2066.795	2319	0.7832	0.50087
O88327	Ctnnal1	Alpha-catulin	33543.46	35941.74	29817.23	37762.87	34266	35713.48	32999.13	26813.25	34134.09	32415	1.0571	0.50262
Q61703	Itih2	Inter-alpha-trypsin inhibitor heavy chain H2	28224.81	27664.57	24019.18	25967.06	26469	29889.02	24616.37	22165.17	23721.09	25098	1.0546	0.50299
P51655	Gpc4	Glypican-4	60977.86	49769.18	39184.66	50297.9	50057	58313.44	56369	50272.16	49370.64	53581	0.9342	0.50494
Q9JJA2	Cog8	Conserved oligomeric Golgi complex subunit 8	9360.598	9384.135	9979.488	5842.296	8642	8653.217	10335	2447.115	7618.013	7263	1.1898	0.50508
Q62313	Tgoln1	Trans-Golgi network integral membrane protein 1	27169.96	20974.15	21981.5	21746.37	22968	26945.25	27434	21563.85	21910.39	24463	0.9389	0.50731
Q60575	Kif1b	Kinesin-like protein KIF1B	12877.23	12356.19	11109.27	9320.453	11416	10557.34	11123.47	10633.65	11082.42	10849	1.0522	0.50766
Q9D2P4	Urm1	Ubiquitin-related modifier 1	8695.553	5086.576	5457.958	4115.523	5839	4106.692	934.0078	5420.486	7936.133	4599	1.2695	0.50850
P01901	H2-K1	H-2 class I histocompatibility antigen, K-B alpha chai	53248.75	27054.88	21642.41	26365.77	32078	29209.48	30287.3	22163.3	25942.71	26901	1.1925	0.50974
P98078	Dab2	Disabled homolog 2	7307.166	7777.327	10838.11	7911.44	8459	6791.185	8100.092	8396.545	8081.421	7842	1.0786	0.50989
Q9DBU3	Riok3	Serine/threonine-protein kinase RIO3	12780.58	10507.88	11323.53	6648.425	10315	10944.45	8566.194	6766.759	10450.73	9182	1.1234	0.51056
P49182	Serpind1	Heparin cofactor 2	26787.28	12747.53	13163.89	7466.041	15041	12179.03	8859.702			10519	1.4299	0.51068
P58058	Nadk	NAD kinase	4205.096	4922.001	4530.559	1290.217	3737	3584.632	2937.902		2537.274	3020	1.2374	0.51073
P21844	Cma1	Chymase	79138.17	52585.56	35805.25	71369.58	59725	63522.86	69396.95	35775.88	32248.87	50236	1.1889	0.51079
P98083	Shc1	SHC-transforming protein 1	3273.843		2885.892	2751.324	2970	2841.683	2797.844			2820	1.0534	0.51176
Q8BH65	Dennd6a	Protein DENND6A	219.9784	975.4883	1028.897		741	784.6534	379.9377		435.2523	533	1.3904	0.51296

Q9D2V8	Mfsd10	Major facilitator superfamily domain-containing prot	23686.82	18234.48	17608.33	15785.62	18829	17469.05	21163.41	14124.45	16322.58	17270	1.0903	0.51412
P70403	Cux1	Protein CASP	9761.626	16647.02	14490.01	19522.83	15105	18782.28	15766.46	13691.45	18860.89	16775	0.9005	0.51438
Q9CY97	Ssu72	RNA polymerase II subunit A C-terminal domain pho	11108.28	13966.21	12401.27	12343.77	12455		11232.28	10809.08	13327.57	11790	1.0564	0.51538
P86046	Kcnj13	Inward rectifier potassium channel 13	11343.74	6722.1	7019.496	8797.761	8471	5389.44	10976.04	8491.696	3661.884	7130	1.1881	0.51555
Q99JY3	Gimap4	GTPase IMAP family member 4	24874.8	17782.99	16484.76	16591.23	18933	19394.08	17914.71	14586.69	17669.68	17391	1.0887	0.51729
Q9CXJ1	Ears2	Probable glutamate--tRNA ligase, mitochondrial	13023.34	7431.174	3793.209	5808.447	7514	5005.889	10142.91	4454.549	3683.494	5822	1.2907	0.51795
Q61823	Pdcd4	Programmed cell death protein 4	19338.41	17062.23	13853.05	16746.08	16750	15134.61	18488.9	13749.35	15499.55	15718	1.0656	0.51819
Q9JJE7	Fads3	Fatty acid desaturase 3	16530.05	15286.86	15233.61	20607.43	16914	16331.74	13950.14	28130.93	18454.75	19217	0.8802	0.51861
Q9ER04	Tmprss5	Transmembrane protease serine 5	9619.198	6579.335	9345.297	9518.08	8765	7688.562	6126.563	4676.308	11873.03	7591	1.1547	0.51963
Q8CG19	Ltbp1	Latent-transforming growth factor beta-binding prote	28928.52	32404.98	24258.02	35856.55	30362	30863.02	30240.17	23285.78	28807.27	28299	1.0729	0.51997
P47880	Igfbp6	Insulin-like growth factor-binding protein 6	26044.49	19462.03	10917.77	18940.03	18841	18991.96	19355.97	14024.88	13578.33	16488	1.1427	0.52238
Q9JMD0	Znf207	BUB3-interacting and GLEBS motif-containing protei	15118.75	15302.1	11923.69	10083.44	13107	14811.84	17638.05	12535.54	12273.03	14315	0.9156	0.52285
Q91X51	Gorasp1	Golgi reassembly-stacking protein 1	7988.461	6801.727	6455.295	4557.933	6451	5117.418	8247.604	4288.142	5105.688	5690	1.1338	0.52466
Q497V5	Srbd1	S1 RNA-binding domain-containing protein 1	7899.486	7984.862	6622.139	21282.79	10947	5134.459	8260.703	2981.17	15518.56	7974	1.3729	0.52542
Q78HU7	Gypc	Glycophorin-C	31213.14	29739.13	26915.1	36041.07	30977	29290.75	33907.67	30687.07	21033.91	28730	1.0782	0.52618
P23475	Xrcc6	X-ray repair cross-complementing protein 6	9170.006	7524.534	7263.209	3749.875	6927	4907.504	6101.775	6036.49	7321.223	6092	1.1371	0.52636
P01898	H2-Q10	H-2 class I histocompatibility antigen, Q10 alpha cha	32271.35	15369.88	16206.06	13793.34	19410	19268.83	14239.23	18275.61	13656.53	16360	1.1864	0.52682
Q4VBE8	Wdr18	WD repeat-containing protein 18	12339.8	8415.989	13374.58	13943.51	12018	12263.51	9008.663	16903.47	15748.52	13481	0.8915	0.52703
P06684	C5	Complement C5	17326.93	7206.361	21568.85	10975.41	14269	12735.26	8538.337	11160.75	15249.58	11921	1.1970	0.52738
P97402	Gcnt2	N-acetyllactosaminide beta-1,6-N-acetylglucosamir	15740.51	9943.208	8715.748	10082.78	11121	7577.938	12068.44	10968.5	8869.921	9871	1.1266	0.52854
Q9D853	Eef1akmt2	EEF1A lysine methyltransferase 2	144412.4	167391.3	52313.09	204149	142066	124282.8	124967.1	104355	127026.3	120158	1.1823	0.52876
P03953	Cfd	Complement factor D	15112.21	28081.88	32665.45	8239.982	21025	27392.86	13425.34	16184.14	8763.97	16442	1.2788	0.53137
Q9JJ80	Rpf2	Ribosome production factor 2 homolog	7305.273	12796.01	9963.938	10048.54	10028	9894.735		6440.082	10359.54	8898	1.1270	0.53138
Q9BCZ4	Selenos	Selenoprotein S	20301.67	19752.83	21297.75	27223.65	22144	18525.57		18077.1	24460.3	20354	1.0879	0.53211
Q8BHR8	Cstpp1	Centriolar satellite-associated tubulin polyglutamyla	7022.56	5305.796	5535.249	3547.912	5353	5692.036	5336.288	1859.975	5459.7	4587	1.1670	0.53256
A2AJ15	Man1b1	Endoplasmic reticulum mannosyl-oligosaccharide 1	56135.81	56525.03	59981.31	58289.69	57733	49562.61	54945.48	52710.32	64739.67	55490	1.0404	0.53295
E9Q7T7	Chadl	Chondroadherin-like protein	11051.23	8854.925	10700.51	10088.29	10174	8606.385	10893.35	14822.67	10085.53	11102	0.9164	0.53546
P28301	Lox	Protein-lysine 6-oxidase	15520.01	17385.07	19535.84	20777.25	18305	18569.5	2820.611	18536.29	21688.55	15404	1.1883	0.53553
P19788	Mgp	Matrix Gla protein	18217.42	16268.13		12596.98	15694	15263.26	48495.68	8547.144		24102	0.6512	0.53678
P01631		Ig kappa chain V-II region 26-10	73816.09	54529.82	16371.12	27491.29	43052	59862.43	47808.61	5694.809	9895.312	30815	1.3971	0.53886
Q99KG3	Rbm10	RNA-binding protein 10		9302.471	12860.58	11095.11	11086			14138.23	10603.02	12371	0.8962	0.54131
Q8JZM0	Tfb1m	Dimethyladenosine transferase 1, mitochondrial	3369.545	2125.112	2409.075	2565.879	2617	2341.924	5947.246	2566.663	2069.351	3231	0.8100	0.54176
Q9EQK7	Icmt	Protein-S-isoprenylcysteine O-methyltransferase	8450.696		3948.101	4626.099	5675	7601.808	8174.246		4753.706	6843	0.8293	0.54224
Q7TQI7	Abtb2	Ankyrin repeat and BTB/POZ domain-containing prot	13372.07	25164.44	10886.18	9434.832	14714	14318.66	11039.42	12198.07	11908.74	12366	1.1899	0.54316
Q06770	Serpina6	Corticosteroid-binding globulin	14943.88	11602.32	5891.271	11970.56	11102	14262.58	9870.718	12510.43	13190.13	12458	0.8911	0.54401
Q9R0M4	Podxl	Podocalyxin	18530.47	14293.1	15463.38	13583.26	15468	14183.76	17807.62	12592.29	24803.4	17347	0.8917	0.54445
Q9WUI1	Mapk11	Mitogen-activated protein kinase 11	13475.16	14470.69	12009.16	12879.97	13209	13484.5	15219	10837.4	9896.82	12359	1.0687	0.54491
Q9WVL2	Stat2	Signal transducer and activator of transcription 2	15250.98	16102.83	15278.61	12763.91	14849	15241.35	16673.7	10302.69	13183.91	13850	1.0721	0.54571
Q8VDD9	Phip	PH-interacting protein	8927.188	6925.089	8894.012	10304.89	8763	10827.31	7530.697	9917.455	9283.597	9390	0.9332	0.54742
Q6WKZ8	Ubr2	E3 ubiquitin-protein ligase UBR2	7731.696	7592.974	5264.31	3452.552	6010	7176.515	6205.96	2909.888	4160.773	5113	1.1754	0.54748
Q99P91	Gpnmb	Transmembrane glycoprotein NMB	21249.35	17705.14	12033.23	20180.89	17792	20769.71	17962.18	10919.73	13924.34	15894	1.1194	0.54936
Q923D4	Sf3b5	Splicing factor 3B subunit 5	21921.36	19196.29	24387.94	23322.87	22207	18028.71	24819.49	20333.63	21125.99	21077	1.0536	0.55382
P50096	Impdh1	Inosine-5'-monophosphate dehydrogenase 1	38124.03	29072.13	22656.84	28612.68	29616	29004.01	33368.61	25779.51	19461.04	26903	1.1008	0.55400
Q99MS7	Ehbp1l1	EH domain-binding protein 1-like protein 1	7791.803	7741.649	10714.23	9182.794	8858	10801.33	6974.955	6171.733	8387.92	8084	1.0957	0.55405
Q61488	Dhh	Desert hedgehog protein	9847.047	5692.641	2005.144	5245.652	5698		6003.127		1888.084	3946	1.4440	0.55488
Q8VEA4	Chchd4	Mitochondrial intermembrane space import and assembly prote		14665.48	11463.15		13064		12890.5	955.742	14285.79	9377	1.3932	0.55663
Q61576	Fkbp10	Peptidyl-prolyl cis-trans isomerase FKBP10	13061.24	11893.43	18160.04	7878.868	12748	13791.78	10433.98	13016.32	21829.52	14768	0.8633	0.55682
Q6P5E6	Gga2	ADP-ribosylation factor-binding protein GGA2	15009.45	13316.65	11597.25	12155.88	13020	17014.14	15143.9	11913.48	11717.33	13947	0.9335	0.55744
P31428	Dpep1	Dipeptidase 1	16843.89	18932.2	19060.56	22967.12	19451	16953.12	20378.54	16929.04	19670.54	18483	1.0524	0.55858
O70404	Vamp8	Vesicle-associated membrane protein 8	62787.36	32678.43	33773.45	33395.98	40659	45286.26	30589.54	33824.42	33005.85	35677	1.1397	0.55986
Q8C4J7	Tbl3	Transducin beta-like protein 3	9769.021	8319.45	8195.226	8311.265	8649	7518.076	10155.42	6273.686	8431.455	8095	1.0685	0.56018
Q7TSH8	Tmem94	Transmembrane protein 94	10339.76	11415.79	10125.52	3344.895	8806	12077.25	11536.83	25.56265	2790.693	6608	1.3328	0.56043
Q8BWL5	Rbms3	RNA-binding motif, single-stranded-interacting prote	63764.58	61699.12	71543.05	74929.51	67984	68597.31	62561.21	72854.37	79705.71	70930	0.9585	0.56053
Q08857	Cd36	Platelet glycoprotein 4	28906.11	20957.88	21162.07	21615.58	23160	22709.66	31228.74	11375.22	15619.36	20233	1.1447	0.56057
Q80WT5	Aftph	Aftiphilin	11220.51	9036.315	8365.589	6952.713	8894	9582.057	10956.64	8147.241	9487.814	9543	0.9319	0.56172
Q9WVH9	Fbln5	Fibulin-5	171889.8	210393.1	260319.6	244820.5	221856	230554.8	198110.5	256474.8	262322.4	236866	0.9366	0.56276
Q6S5L9	Shc4	SHC-transforming protein 4	8019.493	7571.737	11499.26	9078.593	9042	8231.13	7418.123	8877.213	9288.447	8454	1.0696	0.56548
Q921G6	Lrch4	Leucine-rich repeat and calponin homology domain-	13232.75	10925	10437.03	12358	11738	11540.86	9609.542	10798.33	12762.87	11178	1.0501	0.56601
P12242	Ucp1	Mitochondrial brown fat uncoupling protein 1	10817.11	19117.52	7909.793	32302.62	17537	9872.115	14796.32			12334	1.4218	0.56633

Q8R1U1	Cog4	Conserved oligomeric Golgi complex subunit 4	22540.28	23633.68	22536.61	21593.69	22576	21989.97	21125.83	19301.35	24900.32	21829	1.0342	0.56887
P03987		Ig gamma-3 chain C region	141860.1	56642.33	30669.96	24224.11	63349	39222.74	101070.8	12076.18	19932.7	43076	1.4706	0.57021
Q9CQG6	Tmem147	BOS complex subunit TMEM147	10614.52	14049.99	15600.97	8794.04	12265	17599.23	9139.153	6433.123	28086.86	15315	0.8009	0.57311
Q9R0G7	Zeb2	Zinc finger E-box-binding homeobox 2	24414.51	24057.66	33373.22	31923.41	28442	25421.39	18450.6	29328.99	31571.37	26193	1.0859	0.57328
Q8K4G1	Ltbp4	Latent-transforming growth factor beta-binding prote	39624.11	39000.26	61039.93	46864.39	46632	44980.08	38190.5	61070.43	60421.22	51166	0.9114	0.57591
Q3V009	Tmed1	Transmembrane emp24 domain-containing protein 1	5433.999	6576.908	10979.63	7138.855	7532	4680.999	7816.333	9557.086	13090.83	8786	0.8573	0.57694
Q9JLQ2	Git2	ARF GTPase-activating protein GIT2		8174.053	7846.831	8708.134	8243	5935.104	7220.942	11538.93	13103.54	9450	0.8723	0.57860
Q91YI4	Arrb2	Beta-arrestin-2	22008.25	20027.78	16622.49	12340.08	17750	26713.04	24098.2	12595.8	16698.53	20026	0.8863	0.57954
Q9WVJ5	Crybb1	Beta-crystallin B1	7527.664	10098.04	8091.392	4855.576	7643	9515.525	10192.17	5921.594	8292.073	8480	0.9013	0.58002
Q8BTI7	Ankrd52	Serine/threonine-protein phosphatase 6 regulatory a	9903.26	7747.711	9767.69	8921.964	9085	10145.99	9085.009	8555.574	10016.03	9451	0.9613	0.58004
Q8BTW3	Exosc6	Exosome complex component MTR3	8607.634	6028.727	6004.732	7557.266	7050	5148.577	6119.18	5758.362	8785.581	6453	1.0925	0.58093
Q9DBY1	Syvn1	E3 ubiquitin-protein ligase synoviolin	14357.98	20715.2	11188.54	16790.59	15763	14828.95	21587.77	17373.16	15172.7	17241	0.9143	0.58210
Q6GU68	Islr	Immunoglobulin superfamily containing leucine-rich	169817.9	137809	158183.4	143259.4	152267	159539.5	154270.4	161615.4	151336.2	156690	0.9718	0.58362
Q8CGN5	Plin1	Perilipin-1	12482.75	10236.61	5937.406	5448.515	8526	6403.777	12919.79	2265.838	6070.969	6915	1.2330	0.58459
Q91YL2	Rnf126	E3 ubiquitin-protein ligase RNF126	9244.523	11152.57	11311.97	8579.837	10072	9924.372	12563.8	6411.115	7946.454	9211	1.0934	0.58559
P97426	Ear1	Eosinophil cationic protein 1	30681.32	19754.43	14711.51	12852.04	19500	14116.83	17741.34			15929	1.2242	0.59003
Q8BH35	C8b	Complement component C8 beta chain			13695.27	5286.357	9491			7105.083	6529.744	6817	1.3921	0.59072
Q61466	Smarcd1	SWI/SNF-related matrix-associated actin-dependent	8186.907	9190.493	10108.7	6405.2	8473	7960.915	9859.621	7509.595	11216.92	9137	0.9273	0.59099
Q5ND34	Wdr81	WD repeat-containing protein 81	2510.101	2456.549	2633.991	2269.97	2468	2196.626	3298.394		900.6128	2132	1.1575	0.59183
Q9CZH3	Psmg3	Proteasome assembly chaperone 3	9782.88	10149.21	9610.308	6391.667	8984	9832.765	8421.894	9310.095	10596.2	9540	0.9416	0.59196
P0CW02	Ly6c1	Lymphocyte antigen 6C1	24536.63	20490.47	30197.25	31765.84	26748	24947.54	25075.58	32400.17	31982.86	28602	0.9352	0.59743
Q6ZPF4	Fmnl3	Formin-like protein 3	20973.79	33578.57	19365.17	23241.53	24290	22196.34	37115.57	27639.28	20925.19	26969	0.9007	0.60252
Q08481	Pecam1	Platelet endothelial cell adhesion molecule	7608.911	4341.445	7305.348	4808.211	6016	4361.63	7861.293	2673.711	6104.543	5250	1.1458	0.60355
Q9WV91	Ptgfrn	Prostaglandin F2 receptor negative regulator	33016.05	22467.09	35147.48	34929.84	31390	22937.35	24116.39	30220.92	38208.68	28871	1.0873	0.60494
Q9D1G2	Pmvk	Phosphomevalonate kinase	15836.64	14941.91	15778.83	11146.01	14426	16741.49	12311.25	17611.3	14567.19	15308	0.9424	0.60712
P46656	Fdx1	Adrenodoxin, mitochondrial	24267.16	25243.46	22433.14	19693.98	22909	21218.07	18227.16	22652.93	25367.22	21866	1.0477	0.60715
Q08024	Cbfb	Core-binding factor subunit beta	12818.18	20910.46	18855.21	15897.91	17120	19751.95	15470.61	11792.67	16255.88	15818	1.0824	0.60747
Q6P1J0	Maneal	Glycoprotein endo-alpha-1,2-mannosidase-like prot	11490.35	9546.317	8871.865	9387.31	9824	9392.785	10309.11	6683.109	10616.1	9250	1.0620	0.60870
Q9Z239	Fxyd1	Phospholemman	62858.19	88227.9	48997.41	76903.43	69247	64339.66	86107.38	57779.42	41714.41	62485	1.1082	0.60895
Q923Q2	Stard13	StAR-related lipid transfer protein 13	14464.08	13777.09	14141.69	13001.04	13846	12434.49	15056.37	12871.49	13611.84	13494	1.0261	0.61006
P63089	Ptn	Pleiotrophin	5620.454	6820.292	6524.88	7622.468	6647	6529.207	6929.832	6108.102	6033.867	6400	1.0386	0.61283
Q9CY52	Thg1l	Probable tRNA(His) guanylyltransferase	16392.1	12198.29		18876.09	15822	15646.91	12974.9		15355.77	14659	1.0793	0.61321
Q9ERA6	Tfip11	Tuftelin-interacting protein 11	4965.553	3229.348	5193.178	3550.364	4235	2437.174	5896.924	2455.178	4115.024	3726	1.1365	0.61552
Q7TSZ8	Nacc1	Nucleus accumbens-associated protein 1	11443.63	16016.51	11161.91	11107.76	12432	10724.44	7928.213	12519.34	14631.44	11451	1.0857	0.61608
Q7TSF4	Lrrc75a	Leucine-rich repeat-containing protein 75A	6695.129	2666.937	3297.533	3176.799	3959	2897.695	4772.721	2785.74	3210.518	3417	1.1588	0.61765
Q3U284	Tmem231	Transmembrane protein 231	2747.543		6410.257	3556.923	4238	2927.118	3909.709			3418	1.2398	0.61826
Q61559	Fcgrt	IgG receptor FcRn large subunit p51	9906.569	7591.845	7753.027	9398.696	8663	7511.408	6361.726	7395.887	10961.47	8058	1.0751	0.62030
Q9D8Z2	Triap1	TP53-regulated inhibitor of apoptosis 1	8341.83	7014.165	4079.665		6479	6092.778	6572.432	4216.439	6440.11	5830	1.1112	0.62263
Q9CQS2	Nop10	H/ACA ribonucleoprotein complex subunit 3	8235.085	8509.889	6704.367	8694.024	8036	7315.965	9930.67	5362.95	7395.361	7501	1.0713	0.62574
O70422	Gtf2h4	General transcription factor IIH subunit 4	2682.02	1229.695	1367.792	3146.848	2107	937.0774	2845.292		3978.519	2587	0.8143	0.62841
Q8BHL7	Cdc42se1	CDC42 small effector protein 1	14108.91	17354.86	17477.83	19365.1	17077	17105.98	15638.18	15490.7	17606.65	16460	1.0374	0.62928
Q8BIE6	Frmd4a	FERM domain-containing protein 4A	4625.822	4429.842	5900.397	4515.504	4868	5176.612	4597.469	4874.531	5664.617	5078	0.9586	0.63024
Q61033	Tmpo	Lamina-associated polypeptide 2, isoforms alpha/ze	64212.14	58104.87	62457.7	71077.57	63963	58127	61765	67540.88	61676.8	62277	1.0271	0.63046
Q3KNJ2	Nhej1	Non-homologous end-joining factor 1	5654.054	5065.894	8113.172	3795.934	5657	4839.49	5726.263	1243.92	7567.727	4844	1.1678	0.63108
Q8R1Z9	Rnf121	E3 ubiquitin ligase Rnf121	2807.084	1800.896	4590.686	6267.544	3867	3149.166	4496.246	3287.255	7323.676	4564	0.8472	0.63183
P08905	Lyz2	Lysozyme C-2	36983.55	23530.77	25525.45	25561.88	27900	29057.21	28961.51	23785.01	22783.01	26147	1.0671	0.63303
Q61398	Pcolce	Procollagen C-endopeptidase enhancer 1	16895.3	20456.29	24806.69	12444.2	18651	17846.05	15942.34	15604.09	19616.06	17252	1.0811	0.63356
Q505D9	Trim67	Tripartite motif-containing protein 67	22217.01	17110.66	20490.95	21677.94	20374	25888.29	16555.98	25256.19	18933.49	21658	0.9407	0.63670
P08121	Col3a1	Collagen alpha-1(III) chain	50529.06	53286.87	58026.77	56018.12	54465	43524.45	47529.48	69918.48	71809.47	58195	0.9359	0.63875
P50481	Lhx3	LIM/homeobox protein Lhx3	10023.64	17050.98	15456.01	20805.32	15834	14382.04	13770.95	11881.55	18177.35	14553	1.0880	0.63952
Q8BPG6	Sumf2	Inactive C-alpha-formylglycine-generating enzyme 2	4525.193	5667.859	7824.158	7129.124	6287	5643.003	4662.32	11429.8	6691.758	7107	0.8846	0.64115
P21845	Tpsb2	Tryptase beta-2	26364.67	18535.57	19716.78	23622.43	22060	21700.85	29838.15	14510.26	14113.7	20041	1.1008	0.64120
E9PY46	Ift140	Intraflagellar transport protein 140 homolog	8668.236	9652.747	13751.26	2657.076	8682	12297.18	7512.599		10615.14	10142	0.8561	0.64207
Q9CQ45	Nenf	Neudesin	18652.91	11214.73	14222.15	15286.88	14844	14011.91	18422.66		15237.91	15891	0.9341	0.64288
Q91YR9	Ptgr1	Prostaglandin reductase 1	16769.93	17401.2	22942.75	13341.9	17614	16975.89	13740.41	17141.53	18287.61	16536	1.0652	0.64380
P19973	Lsp1	Lymphocyte-specific protein 1	3796.742	3211.156	3781.773		3597		3634.762	3799.739	3653.637	3696	0.9731	0.64446
Q80TN4	Dnajc16	DnaJ homolog subfamily C member 16	10068.33	11201.67	10522.4	15346.56	11785	11437.34	10985.85	9844.352	12320.21	11147	1.0572	0.64485
Q3UDF0	Slc2a6	Solute carrier family 2, facilitated glucose transporte	12927.85	14501.03	11393.1	12861.88	12921	13921.28	10470.29	13560.9	11739.14	12423	1.0401	0.64487
Q9D8T0	Fam3a	Protein FAM3A	2418.047	2473.897	3197.915	1558.584	2412	1780.818	2110.047	4151.526	2811.988	2714	0.8889	0.64573

Q9WUE4	Nprl2	GATOR1 complex protein NPRL2	2299.8		1190.017		**1745**	594.7851	2823.487	1.400102	1604.589	**1256**	1.3892	0.64668
Q6TCG5	Paqr6	Membrane progestin receptor delta	9942.415	11513.96	14388.81	10654.98	**11625**	9912.458	9987.969	13258.13	17125.37	**12571**	0.9248	0.64744
Q9D081	Alg14	UDP-N-acetylglucosamine transferase subunit ALG14 homolog		3455.424		6080.215	**4768**	5500.539	1557.694	4498.387		**3852**	1.2377	0.64845
P18531	Ighv3-6	Ig heavy chain V region 3-6	3088.207	14124.69	1385.061		**6199**	5925.093	944.1046			**3435**	1.8050	0.64857
Q8VDG7	Pafah2	Platelet-activating factor acetylhydrolase 2, cytoplas	5524.886	5463.959	6069.743	2862.408	**4980**	5420.602	3990.654		4188.187	**4533**	1.0986	0.65078
Q0VBL3	Rbm15	RNA-binding protein 15	7581.68	7946.88	9056.462	11731.5	**9079**	7775.607	8626.809	8174.896	9773.551	**8588**	1.0572	0.65101
Q3UV71	Tmtc1	Protein O-mannosyl-transferase TMTC1	2673.177	3235.758	2831.337	2157.167	**2724**	3123.787	4575.475	3854.71	910.066	**3116**	0.8743	0.65121
P70166	Cpeb1	Cytoplasmic polyadenylation element-binding protei	12006.49	7753.122	8844.571	11436.08	**10010**	9908.373	8868.834	10768.93	13072.91	**10655**	0.9395	0.65139
A2AI05	Ndor1	NADPH-dependent diflavin oxidoreductase 1	3857.59	5227.049		2567.729	**3884**	3090.627	2021.093	5396.905	2988.63	**3374**	1.1511	0.65181
P03958	Ada	Adenosine deaminase	13029.75	8527.674	8715.776	8928.153	**9800**	8412.63	9573.958			**8993**	1.0897	0.65189
P01633	Igkv6-17	Immunoglobulin kappa chain variable 6-17	50835.67	21421.76	10433.56	11940.22	**23658**	15219.13	18552.39			**16886**	1.4011	0.65626
A2A761	Zfp69	Zinc finger protein 69	2378.337	940.2076		4846.162	**2722**	662.8698	1330.391	6997.846	5859.937	**3713**	0.7330	0.65840
Q6DID3	Scaf8	SR-related and CTD-associated factor 8	12799.37	12223.83	13413.77	9532.752	**11992**	10819.1	13416.24	11422.29	14580.45	**12560**	0.9548	0.65893
O35638	Stag2	Cohesin subunit SA-2	5416.53	5614.012	6702.018	3780.837	**5378**	5899.809	4475.697	4348.105	5468.458	**5048**	1.0654	0.65898
Q3TV70	Nr2c2ap	Nuclear receptor 2C2-associated protein	17295.35	13541.35		17131.13	**15989**	14405.33	21009.53	6218.613		**13878**	1.1521	0.65988
Q91WD2	Trpv6	Transient receptor potential cation channel subfamil	7160.086	5949.555	6559.305	10996.52	**7666**	8372.581		7734.288	4570.73	**6893**	1.1123	0.66194
Q8BGR6	Arl15	ADP-ribosylation factor-like protein 15	6587.917	2964.994	3500.751	5731.281	**4696**	7101.284	491.5069		3852.164	**3815**	1.2310	0.66217
Q91VJ2	Cavin3	Caveolae-associated protein 3	84937.82	82005.91	81586.95	95989.18	**86130**	79771.84	78869.68	104037.3	94549.71	**89307**	0.9644	0.66391
Q9CYA0	Creld2	Protein disulfide isomerase Creld2	13928.1	14403.62	15361.11	15540.72	**14808**	15418.39	17482	8657.981	14188.16	**13937**	1.0626	0.66658
Q7TQ62	Podn	Podocan	13002.1	16674.29	11320.69	10592.05	**12897**	10441.25	15818.97	12251.36	9599.229	**12028**	1.0723	0.66887
O89032	Sh3pxd2a	SH3 and PX domain-containing protein 2A	15606.53	20031.64	23897.33	19461.06	**19749**	20854	18298.18	18666.74	25389.82	**20802**	0.9494	0.67013
Q9CQJ0	Them5	Acyl-coenzyme A thioesterase THEM5	28327.58	35797.05	40277.51	32849.13	**34313**	32179.4	29703.22	27128	41163.59	**32544**	1.0544	0.67022
Q61329	Zfhx3	Zinc finger homeobox protein 3		9389.648	7155.789	9019.898	**8522**	9663.912		5405.828	8507.24	**7859**	1.0843	0.67071
Q7TMR0	Prcp	Lysosomal Pro-X carboxypeptidase	21970.68	8970.029	13811.3	11122.47	**13969**	12629	15017.84	10434.97	12438.79	**12630**	1.1060	0.67071
Q5FW52	Mlip	Muscular LMNA-interacting protein	41628.61	45370.31	52710.49	49495.96	**47301**	45032.44	38221.16	57702.67	57958.65	**49729**	0.9512	0.67138
P01753		Ig heavy chain V region 186-1	59314.8	34178.64	19325.49		**37606**	31872.91	29394.79			**30634**	1.2276	0.67558
Q8BRH3	Arhgap19	Rho GTPase-activating protein 19	14713.55	13643.65	12356.91	14802.87	**13879**	14673.74	16783.43	12758.68	13186.89	**14351**	0.9671	0.67597
Q6AW69	Cgnl1	Cingulin-like protein 1	9739.351	16517.82	9987.129	11973.3	**12054**	9727.794	15168.79	9587.534	10107.6	**11148**	1.0813	0.67632
Q9ERG0	Lima1	LIM domain and actin-binding protein 1	16414.68	14268.41	20065.79	14934.09	**16421**	16919.78	16119.11	15196.94	15052.37	**15822**	1.0378	0.67662
Q8VEL9	Rem2	GTP-binding protein REM 2	18860.07	11545.92	12660.58	5764.033	**12208**	14518.8	9880.109	15978.13	13671.71	**13512**	0.9035	0.67711
Q6A026	Pds5a	Sister chromatid cohesion protein PDS5 homolog A	17418.61	14263.39	11937.32	12878.05	**14124**	14821.52	14022.99	13065.43	12292.31	**13551**	1.0423	0.67868
O35486	Crygs	Gamma-crystallin S	13760.92	27400.32	9403.475	14880.11	**16361**	21170.47	13116.65		7365.716	**13884**	1.1784	0.68035
Q99J93	Ifitm2	Interferon-induced transmembrane protein 2	5440.551	3527.512	5112.56		**4694**	6668.344	4749.733	5045.469	3806.268	**5067**	0.9262	0.68205
Q60961	Laptm4a	Lysosomal-associated transmembrane protein 4A	17221.34	12167.17	16816.99	16834.36	**15760**	13074.26	15773.4	20770.9	16855.63	**16619**	0.9483	0.68236
Q61526	Erbb3	Receptor tyrosine-protein kinase erbB-3	6802.162	5003.552	3211.654	5856.599	**5218**	8033.695	3800.062	2443.002	4161.802	**4610**	1.1321	0.68346
Q8R3G9	Tspan8	Tetraspanin-8	22522.56	18627.99	16852.76	22438.11	**20110**	22514.19	22364.37	19790.49	18684.9	**20838**	0.9651	0.68439
P82343	Renbp	N-acylglucosamine 2-epimerase	7854.5	8416.59	6895.506	4632.083	**6950**	8695.316	6688.615	5037.095	5379.25	**6450**	1.0775	0.68569
Q921C5	Bicd2	Protein bicaudal D homolog 2	15758.36	14333.42	15366.98	12082.3	**14385**	14916.54	12835.8	11695.64	15939.96	**13847**	1.0389	0.68627
Q8BX90	Fndc3a	Fibronectin type-III domain-containing protein 3A	3407.563	2132.682	3200.867	4799.385	**3385**	2977.752	2127.413	2998.168	4250.702	**3089**	1.0960	0.68698
Q9WU42	Ncor2	Nuclear receptor corepressor 2	16786.84	12014.22	14067.7	17899.37	**15192**	13967.83	18579.48	9293.477	15002.69	**14211**	1.0690	0.68844
Q91V81	Rbm42	RNA-binding protein 42	4800.278	2680.763	7110.693	5553.12	**5036**	6524.506	5448.348	4245.938	5640.778	**5465**	0.9216	0.69269
Q7TSH6	Scaf4	SR-related and CTD-associated factor 4	1779.71	3219.354	3565.343	3706.771	**3068**	1526.372	3903.078		2798.678	**2743**	1.1185	0.69281
Q3UV16	Itpripl2	Inositol 1,4,5-trisphosphate receptor-interacting pro	6093.374	8588.881	7579.896	9682.799	**7986**	4919.358	15729.02	7143.011	8239.672	**9008**	0.8866	0.69304
Q9QZJ6	Mfap5	Microfibrillar-associated protein 5	50939.3	45020.69	43864.2	42898.39	**45681**	52632.83	52664.59	39408.02	44193.73	**47225**	0.9673	0.69439
Q8BJZ3	Tmbim1	Protein lifeguard 3	21106.75	30193.7	19522.61	29551.6	**25094**	20666.04	28168.45	21764.13	24426.98	**23756**	1.0563	0.69446
Q9QXM0	Abhd2	Monoacylglycerol lipase ABHD2	5535.634	2684.576	4130.482	4232.693	**4146**	4096.597	4160.583	2463.032	4633.55	**3838**	1.0801	0.69653
Q6TCG2	Paqr9	Membrane progesterone receptor epsilon	10892.81	7895.249	7193.377	5107.716	**7772**	6608.199	8057.224	8032.159	6296.357	**7248**	1.0723	0.69739
P55002	Mfap2	Microfibrillar-associated protein 2	20417.46	18350.3	15106.94	21266.25	**18785**	20095.91	20253.44	15446.67	16319.53	**18029**	1.0420	0.69781
O35718	Socs3	Suppressor of cytokine signaling 3	28822.37	26792.94	25807.19	26562.84	**26996**	29072.69	24729.28	28104.56	23734.1	**26410**	1.0222	0.69832
Q9CRA0	Art4	Ecto-ADP-ribosyltransferase 4	9447.251	8398.173	8062.824	10913.46	**9205**	5153.035	14709.38	6351.342	6935.858	**8287**	1.1108	0.69933
Q3UHU5	Mtcl1	Microtubule cross-linking factor 1	12274.33	13449.68	11368.37	14442.52	**12884**	12705.88	12859.5	11483.49	13239.23	**12572**	1.0248	0.70032
Q70FJ1	Akap9	A-kinase anchor protein 9	4640.066		6250.424	5024.358	**5305**	3597.514	5100.015		9471.833	**6056**	0.8759	0.70200
Q62177	Sema3b	Semaphorin-3B	20280.12	17945.18	19356.12	19673.79	**19314**	19633.47	18700.78	19720.31	20124.54	**19545**	0.9882	0.70386
Q99LJ6	Gpx7	Glutathione peroxidase 7	17453.99	18547.03	30789.2	22307.37	**22274**	20075.27	15511.64	28994.89	32377.84	**24240**	0.9189	0.70409
Q3V0G7	Garnl3	GTPase-activating Rap/Ran-GAP domain-like protein	9308.796	5160.477	4698.002	1554.054	**5180**	6193.37	8887.348	4491.472	4175.338	**5937**	0.8726	0.70765
Q920A7	Afg3l1	AFG3-like protein 1	7140.65	6008.728	4105.419	7688.639	**6236**	4763.919	5804.508		6872.837	**5814**	1.0726	0.70851
P82347	Sgcd	Delta-sarcoglycan	276779.4	277731.3	230140.7	333853.2	**279626**	289209.4	298028.5	285143.3	279873.8	**288064**	0.9707	0.70904
P52795	Efnb1	Ephrin-B1	39407.04	34951.57	36555.77	40030.22	**37736**	39314.51	34240.81	37429.39	43521.33	**38627**	0.9769	0.70948
Q9DBZ1	Ikbip	Inhibitor of nuclear factor kappa-B kinase-interacting	8423.606	11075.79	13068.72	8968.126	**10384**	9422.793	8436.188	11433.27	15045.26	**11084**	0.9368	0.71149

Q00780	Col8a1	Collagen alpha-1(VIII) chain	8171.14	7104.84	3953.354	6299.042	**6382**	7018.74	8682.204	2397.539	4913.498	**5753**	**1.1094**	**0.71239**
Q4PNJ2	Nkain2	Sodium/potassium-transporting ATPase subunit beta	19460.37	14577.72	9429.759	20225.71	**15923**	15267.9	13623.82			**14446**	**1.1023**	**0.71607**
Q6ZQH8	Nup188	Nucleoporin NUP188	2105.203	2523.414	8380.239	2591.918	**3900**	4023.821	3348.197	9045.646	2382.224	**4700**	**0.8298**	**0.71775**
Q8BH15	Cnot10	CCR4-NOT transcription complex subunit 10	18595.05	14386.02	14519.01	15034.09	**15634**	16182.86	15805.31	18241.96	8910.266	**14785**	**1.0574**	**0.72047**
Q78IS1	Tmed3	Transmembrane emp24 domain-containing protein 3	15547.13	13018.08	17112.56	16573	**15563**	15083.5	15646.01	15329.61	17853.1	**15978**	**0.9740**	**0.72076**
Q8R100	Calhm5	Calcium homeostasis modulator protein 5	24020.75	20848.55	13558.24	18603.12	**19258**	19629.47	26714.12	10550.46	13889.18	**17696**	**1.0883**	**0.72093**
Q9JJ43	Rbfox1	RNA binding protein fox-1 homolog 1	11831.17	13886.43	15014.31	14658.09	**13847**	15617.51	14995.38	10163.68	12465	**13310**	**1.0404**	**0.72187**
Q8K3X4	Irf2bpl	Probable E3 ubiquitin-protein ligase IRF2BPL		12465.99	7243.472	6196.235	**8635**	11268.03		8381.125	8708.476	**9453**	**0.9135**	**0.72232**
Q6DFX2	Antxr2	Anthrax toxin receptor 2	6724.656	7423.907	6243.702	4471.683	**6216**	6730.093	5654.021	6645.983	6855.542	**6471**	**0.9605**	**0.72307**
O08734	Bak1	Bcl-2 homologous antagonist/killer		5628.027	12702.2	6089.654	**8140**		8583.245	10043.99	8453.031	**9027**	**0.9018**	**0.72410**
Q9D7B7	Gpx8	Probable glutathione peroxidase 8	25449.56	21963.22	28117.81	23069.46	**24650**	21921		24410.09	30641.03	**25657**	**0.9607**	**0.72489**
Q3V3R4	Itga1	Integrin alpha-1	13412.74	10348.35	9942.334	11338.5	**11260**	10813.65	13152.63	7265.508	11641.94	**10718**	**1.0506**	**0.72492**
P14434;P14438	H2-Aa	H-2 class II histocompatibility antigen, A-B alpha cha	32450.17		12184.42	25629.35	**23421**	20270.56	21037.05	16636.85	27355.34	**21325**	**1.0983**	**0.72514**
Q921V5	Mgat2	Alpha-1,6-mannosyl-glycoprotein 2-beta-N-acetylglu	8263.206	7027.458	6743.311	6416.759	**7113**	8356.762	7087.387		6608.144	**7351**	**0.9676**	**0.72783**
P97760	Polr2c	DNA-directed RNA polymerase II subunit RPB3	22450.28	25053.25	21300.6	22215.32	**22755**	23772.67	24379.5	19746.3	21146.14	**22261**	**1.0222**	**0.72860**
Q62469	Itga2	Integrin alpha-2	30758.3	28185.05	33073.55	32895.14	**31228**	27680.16	31898.32	31094.04	32014.3	**30672**	**1.0181**	**0.72867**
Q8C6M1	Usp20	Ubiquitin carboxyl-terminal hydrolase 20	3848.985	5090.121	4295.506	5961.228	**4799**	4946.698	4110.965	3825.782	5449.312	**4583**	**1.0471**	**0.72998**
Q80U58	Pum2	Pumilio homolog 2	7715.878	4275.591	5328.516	5738.282	**5765**	5622.571	5736.814	5587.886	5051.488	**5500**	**1.0482**	**0.73116**
P13597	Icam1	Intercellular adhesion molecule 1	22114.84	11182.12	5587.427	10219.06	**12276**	12951.39	12610.06	6350.989		**10637**	**1.1540**	**0.73143**
Q91ZU1	Asb6	Ankyrin repeat and SOCS box protein 6	15959.67	17679.2	15117.25	12789.76	**15386**	14121.46	16521.17	17033.97	11543.7	**14805**	**1.0393**	**0.73167**
E9Q394	Akap13	A-kinase anchor protein 13	34593.15	26633.88	26556.59	31119.24	**29726**	33777.41	28652.28	30240.09	29449.31	**30530**	**0.9737**	**0.73266**
E9Q555	Rnf213	E3 ubiquitin-protein ligase RNF213	7991.805	5440.071	7716.867	6784.383	**6983**	6426.888	6180.724	7979.583	8498.352	**7271**	**0.9604**	**0.73445**
Q9JHA8	Vwa7	von Willebrand factor A domain-containing protein 7	2044.679	4441.889	5802.025	6698.851	**4747**		4519.109		6129.846	**5324**	**0.8915**	**0.73601**
P97473	Tarbp2	RISC-loading complex subunit TARBP2	17640.11	15256.52	12942.26	23083.3	**17231**	17501.31	17211.39	16252.21	14796.13	**16440**	**1.0481**	**0.73827**
P70182	Pip5k1a	Phosphatidylinositol 4-phosphate 5-kinase type-1 al	8166.582	10112.67	13201.77	10242.21	**10431**	10465.82	10666.36	12025.94	10120.25	**10820**	**0.9641**	**0.74020**
P15089	Cpa3	Mast cell carboxypeptidase A	147412.6	105715.3	75461.13	145088.7	**118419**	128414.8	152120	83145.95	75526.54	**109802**	**1.0785**	**0.74340**
Q9WVJ9	Efemp2	EGF-containing fibulin-like extracellular matrix prote	9032.261	9992.372	13798.77	6960.596	**9946**	9753.617	6806.861	7438.843	13037.32	**9259**	**1.0742**	**0.74411**
Q9JKJ9	Cyp39a1	24-hydroxycholesterol 7-alpha-hydroxylase	14488.82	11482.29	17510.23	16011.89	**14873**	12296.43	12003.79	14364.09	18204.79	**14217**	**1.0461**	**0.74471**
Q8BGD6	Slc38a9	Neutral amino acid transporter 9	14469.8	10614.93	8774.074	8480.218	**10585**	14454.59	12680.21	7767.661	10138.63	**11260**	**0.9400**	**0.74825**
P97363	Sptlc2	Serine palmitoyltransferase 2	41076.6	33906.42	42360	43272.51	**40154**	43390.96	41786.7	36499.8	42475.88	**41038**	**0.9784**	**0.74844**
Q91YE7	Rbm5	RNA-binding protein 5	844.5074	2547.144	5217.826	5191.424	**3450**	3212.247	4045.983		4415.636	**3891**	**0.8867**	**0.74848**
Q9D281	Fam114a1	Protein Noxp20	6583.834	6818.707	10845.83	3478.819	**6932**	7191.074	4741.089	6908.056	6731.57	**6393**	**1.0843**	**0.74932**
O70572	Smpd2	Sphingomyelin phosphodiesterase 2	13234.92	11680.16	15653.36	14364.66	**13733**	12642.19	14801.2	11831.57	14203.2	**13370**	**1.0272**	**0.74938**
Q8BTP0	Pigz	GPI mannosyltransferase 4	4140.392	4288.745	3381.824	4287.609	**4025**	3798.054	5330.111	1943.108	4050.497	**3780**	**1.0646**	**0.74977**
Q78T81	Eeig1	Early estrogen-induced gene 1 protein	6805.005	6673.743	4738.266	5423.735	**5910**	5795.961	6197.721	5236.011	5700.854	**5733**	**1.0310**	**0.75221**
Q61809	Lrrn1	Leucine-rich repeat neuronal protein 1		7760.354	4174.25	7287.955	**6408**	4672.684	9022.081	3923.985	10967.98	**7147**	**0.8966**	**0.75261**
Q9R0B6	Lamc3	Laminin subunit gamma-3	26822.42	26363.61	24786.02	45543.95	**30879**	25845.44	31054.18	26197.75	33555.75	**29163**	**1.0588**	**0.75525**
Q9DBV4	Mxra8	Matrix remodeling-associated protein 8	13925.61	9758.086	10441.64	11207.88	**11333**	9671.314	11443.14	12269.88	10570.09	**10989**	**1.0314**	**0.75854**
Q5DU57	Spata13	Spermatogenesis-associated protein 13	12971.22	11120.28	14739.48	15982.37	**13703**	10041.6	10342.58	17159.14	14679.14	**13056**	**1.0496**	**0.76036**
Q9QXP7	C1qtnf1	Complement C1q tumor necrosis factor-related protein 1			14809.78	14079.89	**14445**	16481.02		13480.73		**14981**	**0.9642**	**0.76157**
Q8BVG5	Galnt14	Polypeptide N-acetylgalactosaminyltransferase 14	29231.65	29690.47	17828.26	31752.52	**27126**	22245.39	35155.75	24446.48	20920.93	**25692**	**1.0558**	**0.76161**
Q8R3H7	Hs2st1	Heparan sulfate 2-O-sulfotransferase 1	16691.25	15939.71	20139.18	20187.54	**18239**	19117.96	17412.76	14672.06	19737.87	**17735**	**1.0284**	**0.76249**
Q04888	Sox10	Transcription factor SOX-10	7325.854	7754.147	11740.52	9051.189	**8968**	7608.523	6552.915	12684.96	11257.5	**9526**	**0.9414**	**0.76250**
Q61070	Ei24	Etoposide-induced protein 2.4	40312.27	39522.27	45220.83	47309.03	**43091**	36501.04	36478.73	39103.04	54355.25	**41610**	**1.0356**	**0.76277**
Q9D6Y4	Borcs8	BLOC-1-related complex subunit 8	10544.62	9099.151	12297.05	14460.95	**11600**	10532.29	8727.173	13053.18	12195	**11127**	**1.0426**	**0.76294**
O35607	Bmpr2	Bone morphogenetic protein receptor type-2	8025.621	6100.295	5922.452	6022.55	**6518**	6295.114	6555.785	6175.181	6401.623	**6357**	**1.0253**	**0.76338**
B1AVD1	Xpnpep2	Xaa-Pro aminopeptidase 2	36014.36	29185.82	23113.89	33360.05	**30419**	30241.56	33613.89	25882.89	27833.54	**29393**	**1.0349**	**0.76426**
Q61164	Ctcf	Transcriptional repressor CTCF	10568.97	12154.37	16126.15	15418.27	**13567**	15304.37	9755.665	16259.65	10270.77	**12898**	**1.0519**	**0.76478**
P97858	Slc35b1	Solute carrier family 35 member B1	3072.915	4092.894	4123.389	1860.626	**3287**	4840.119	1816.484	2388.841	5398.969	**3611**	**0.9104**	**0.76506**
Q91ZW2	Pofut1	GDP-fucose protein O-fucosyltransferase 1	23254.75	22722.02	23238.87	24269.39	**23371**	21517.54	22474.72	22444.95	25815.49	**23063**	**1.0134**	**0.76799**
Q9JJR8	Tmem9b	Transmembrane protein 9B	22976.26	24973.52	17564.61	25284.35	**22700**	18341.89	28880.23	21367.43	25830.65	**23605**	**0.9616**	**0.76860**
Q8BZ94	Zmat4	Zinc finger matrin-type protein 4		7239.678	5034.903	9032.751	**7102**	5470.905	5796.039	8602.479		**6623**	**1.0724**	**0.76897**
Q8BL03	Slc25a29	Mitochondrial basic amino acids transporter	1596.977		2529.111	1172.814	**1766**	3191.93	2294.402	1415.479	988.1813	**1972**	**0.8955**	**0.77024**
O54890	Itgb3	Integrin beta-3	39178.69	44101.36	66440.66	54734.7	**51114**	48335.81	44098.23	59102.55	61950.35	**53372**	**0.9577**	**0.77064**
Q9ERT9	Ppp1r1a	Protein phosphatase 1 regulatory subunit 1A	13824.29	12958.67	10041.53	9858.271	**11671**	13197.35	8937.044	12430.89	10425.34	**11248**	**1.0376**	**0.77241**
Q8R3Q0	Saraf	Store-operated calcium entry-associated regulatory	19728.75	20151.18	14635.58	12882.02	**16849**	18100.2	22501.87	11637.53	18648	**17722**	**0.9508**	**0.77343**
P62915	Gtf2b	Transcription initiation factor IIB	14288.23	11236.99	7782.016	15174.56	**12120**	14331.86	11360.44		12590.87	**12761**	**0.9498**	**0.77345**
Q3UGP8	Alg10b	Putative Dol-P-Glc:Glc(2)Man(9)GlcNAc(2)-PP-Dol a	29132.49	26966.51	27469.78	32682.19	**29063**	32409.42	26779.45	28853.52	30316.41	**29590**	**0.9822**	**0.77410**
Q9Z1Y4	Trip6	Thyroid receptor-interacting protein 6	1944.467	1082.27	636.5962	2264.84	**1482**	611.6817	2064.457		1255.261	**1310**	**1.1309**	**0.77459**

Q9D1R1	Tmem126b	Complex I assembly factor TMEM126B, mitochondri	5224.614	8784.234	6898.023		6969	6584.584	8267.86	9920.928	4950.151	7431	0.9378	0.77514
Q9EPB5	Serhl	Serine hydrolase-like protein	15486.66	15637.42	26163.03	18071.86	18840	14582.75	15135.42	22950.48	27336.53	20001	0.9419	0.78089
Q3UH93	Plxnd1	Plexin-D1	14513.3	14743.36	17298.04	17737.02	16073	15860.88	21594.88	13332.61	15749.08	16634	0.9662	0.78248
Q91XB0	Trex1	Three-prime repair exonuclease 1	3039.408		3927.194	2569.046	3179	1756.795	3033.392	3147.97	4024.678	2991	1.0628	0.78268
Q8BWW9	Pkn2	Serine/threonine-protein kinase N2	5451.358	4621.038	5521.695	3852.278	4862	4103.621	4938.472	4098.854	5678.166	4705	1.0333	0.78401
Q9CQ43	Dut	Deoxyuridine 5'-triphosphate nucleotidohydrolase		7122.161	7818.428	8068.378	7670	6867.235	11144.14	7526.651	6607.933	8036	0.9544	0.78415
Q91ZN5	Slc35b2	Adenosine 3'-phospho 5'-phosphosulfate transporte	11261.59	11327.02	15900.88	11938.83	12607	8512.162	14351.9	13469.2	16387.69	13180	0.9565	0.78468
Q08890	Ids	Iduronate 2-sulfatase	16426.71	14143.44	14112.32	14258.06	14735	14508.72	17089.7	15382.88	13089.72	15018	0.9812	0.78891
Q8CI11	Gnl3	Guanine nucleotide-binding protein-like 3	9028.266	10464.29		11769.22	10421	13128.83		6869.702	9578.77	9859	1.0570	0.79056
Q80U35	Arhgef17	Rho guanine nucleotide exchange factor 17	24193.77	24702.04	30884.49	23092.41	25718	25263.66	28970.28	22064.64	24046.55	25086	1.0252	0.79079
Q8BKT7	Thoc5	THO complex subunit 5 homolog	4076.122	4581.2	6290.002	3012.767	4490	3234.828	3711.17	5439.573	4646.912	4258	1.0545	0.79209
Q8VHK1	Caskin2	Caskin-2	7801.879	7309.374	8959.583	7498.66	7892	6538.377	8023.974	9078.684	8663.081	8076	0.9773	0.79267
Q6ZWQ0	Syne2	Nesprin-2	6135.284	3010.309	4969.293	2726.419	4210	4186.647	6261.729	2790.66	2288.315	3882	1.0846	0.79422
Q91X84	Crtc3	CREB-regulated transcription coactivator 3	8153.764	9853.672	10281.42	8909.612	9300	7102.1	8243.91	9821.669	10967.19	9034	1.0294	0.79459
Q9D270	Zdhhc21	Palmitoyltransferase ZDHHC21	15115.99	15095.59	12860.34	11314.27	13597	10549.74	17341.99	13099.88	11484.36	13119	1.0364	0.79587
Q8BG16	Slc6a15	Sodium-dependent neutral amino acid transporter B	10505.46	8397.218	9241.7	11827.57	9993	7533.583	8425.679	12138.58	13649.82	10437	0.9575	0.79618
Q99KD5	Unc45a	Protein unc-45 homolog A	25896.98	22412.84	24365.53	19580.98	23064	25346.03	20516.57	22596.16	21967.47	22607	1.0202	0.79649
Q6GQT6	Scap	Sterol regulatory element-binding protein cleavage-a	2949.151	3808.839	4927.905	5449.065	4284	3837.206	4216.266	4727.936	5019.766	4450	0.9626	0.79718
O89116	Vti1a	Vesicle transport through interaction with t-SNAREs	23498.97	20823.04	20459.16	27359.54	23035	27680.34	35405.02	11731.28	22901.49	24430	0.9429	0.79774
Q9DBJ3	Baiap2l1	Brain-specific angiogenesis inhibitor 1-associated pr	18797.73	17147.6	19578.68	20148.07	18918	19216.63	15355.74	19228.43	20493.38	18574	1.0185	0.79844
Q64449	Mrc2	C-type mannose receptor 2	43705.57	36998.74	53223.03	32121.17	41512	41970.61	37194.01	36389.07	45154.37	40177	1.0332	0.79896
Q8BHD0	Rab39a	Ras-related protein Rab-39A	9229.025	10016.78	10060.98	7776.855	9271	10676.25	8602.393	9065.637	9474.931	9455	0.9806	0.79998
P14901	Hmox1	Heme oxygenase 1	16608.37	14487.29	15670.68	16607.04	15843	16026.32	12519.3	17508.56	18856.94	16228	0.9763	0.80037
Q8R088	Golph3l	Golgi phosphoprotein 3-like		1865.277		2997.559	2431	2712.261	2775.198	850.1847	2554.989	2223	1.0937	0.80073
O88792	F11r	Junctional adhesion molecule A	56177.39	48540	40049.27	53168.67	49484	53063	52177.74	44361.39	43796.85	48350	1.0235	0.80076
Q8CHT3	Ints5	Integrator complex subunit 5	5537.428	3341.46	3509.688	3003.942	3848	4458.59	3842.05	2583.112	5372.834	4064	0.9468	0.80079
Q8R4Y4	Stab1	Stabilin-1	2985.921	7001.787	12140.68	11723.37	8463	7598.841		6984.475	8740.432	7775	1.0885	0.80166
Q9WVL6	Extl3	Exostosin-like 3	10342.42	7749.251	8996.799	6287.367	8344	13344.48	7656.929	2878.762	7080.39	7740	1.0780	0.80321
Q8R173	Zdhhc3	Palmitoyltransferase ZDHHC3	7341.96	6945.476	7157.178	8019.48	7366	8316.441	6157.061	8229.674	6072.734	7194	1.0239	0.80461
Q9Z2X8	Keap1	Kelch-like ECH-associated protein 1	19928.67	18738.98	23336.46		20668		18374.22	21782.85		20079	1.0294	0.80487
Q8K2C7	Os9	Protein OS-9	19559.11	18942.64	23122.69	19495.13	20280	18263.83	20348.41	21896.49	21943.44	20613	0.9838	0.80504
P30681	Hmgb2	High mobility group protein B2	26215.44	21582.75	28190.34	23287.8	24819	18811.1	20451.06	30415.77	26455.97	24033	1.0327	0.80636
Q8R2G4	Art3	Ecto-ADP-ribosyltransferase 3	129944.3	130256.7	107081.5	170307	134397	144051.5	131314.9	142173.8	133826.8	137842	0.9750	0.80721
Q8K1N4	Spats2	Spermatogenesis-associated serine-rich protein 2	7052.453	7538.371	8122.134		7571		8849.114	5415.053	9455.943	7907	0.9575	0.80832
Q80W68	Kirrel1	Kin of IRRE-like protein 1	7244.173	5849.368	5457.848	7503.963	6514	7652.931	5192.146	6937.367	7012.343	6699	0.9724	0.80871
Q8C3X2	Ccdc90b	Coiled-coil domain-containing protein 90B, mitochor	16634.52	14973.64	11901.62	12290.31	13950	15744.73	17450.69	12790.9	11574.35	14390	0.9694	0.81014
Q9EQJ0	Tpcn1	Two pore calcium channel protein 1	14786.38	14340.9	16061.82	14099.83	14822	17015.13	14933.39	12602.72	15763.32	15079	0.9830	0.81121
Q8VCG4	C8g	Complement component C8 gamma chain		5240.536	29641.65	11515.95	15466			18736.58	17094.71	17916	0.8633	0.81251
P52633	Stat6	Signal transducer and transcription activator 6	18826.79	23329.42	22966.37	16309.23	20358	20616.88	21096.53	19910.64	18015.12	19910	1.0225	0.81398
Q64689	St8sia3	Sia-alpha-2,3-Gal-beta-1,4-GlcNAc-R:alpha 2,8-sialy	15003.02	16132	16801.76	18098.69	16509	14954.99	18219.9	17787.6	13844.86	16202	1.0190	0.81401
Q9JMC8	Epb41l4b	Band 4.1-like protein 4B	12106.8	9297.381	12023.06	8543.862	10493	11246.01	10240.17	9935	9579.627	10250	1.0237	0.81424
E9PXT9	Fam170b	Protein FAM170B	3273.753	2302.838	2187.981		2588	1075.911	5014.557	3069.945	2196.723	2839	0.9116	0.81635
Q9QZZ6	Dpt	Dermatopontin	93116.77	89280.72	81368.5	85168.23	87234	94299.51	89551.66	86020.81	82521.23	88098	0.9902	0.81697
Q8BJ05	Zc3h14	Zinc finger CCCH domain-containing protein 14	14915.99	9394.826	9301.206	8234.013	10462	14442.32	13953.1	2983.431	7455.949	9709	1.0775	0.81828
Q9ES34	Ube3b	Ubiquitin-protein ligase E3B	14357.18	14018.16	9286.899	10211.84	11969		12185.54	10784.13		11485	1.0421	0.82010
Q6P5C5	Smug1	Single-strand selective monofunctional uracil DNA g	7821.486	7919.962	10617.98	6778.608	8285	8418.464	5830.75	10564.37	9556.292	8592	0.9642	0.82171
Q9DCA5	Brix1	Ribosome biogenesis protein BRX1 homolog	6876.183	4621.588	4797.222	3863.789	5040	5915.966	3719.618	1531.885	7617.191	4696	1.0732	0.82314
Q0P678	Zc3h18	Zinc finger CCCH domain-containing protein 18	15676.95	16437.19	15482.86	17918.07	16379	13726.99	15783.09	18933.83	15946.57	16098	1.0175	0.82342
Q80ZK9	Wdtc1	WD and tetratricopeptide repeats protein 1	18958.33	16940.58	24122.07	14976.36	18749	13732.44	15403.97	21867.88	21348.49	18088	1.0366	0.82430
Q5FWI3	Cemip2	Cell surface hyaluronidase	26205.57	27353.27	21358.77	24859.32	24944	25342.59	19276.23	24284.76	28702.16	24401	1.0222	0.82455
A6X8Z5	Arhgap31	Rho GTPase-activating protein 31	20246.11	18190.77	20228.66	20750.8	19854	19195.1	18816.28	18536.2	21968.42	19629	1.0115	0.82489
P25318	Col8a2	Collagen alpha-2(VIII) chain	8598.677	10269.74	8431.911	15392.48	10673	6285.495	10357.91	12640.37	15787.69	11268	0.9472	0.82521
O54998	Fkbp7	Peptidyl-prolyl cis-trans isomerase FKBP7	3991.597	2660.948	4452.379	4683.473	3947	2819.521	3114.63	3579.524	5587.996	3775	1.0455	0.83111
Q9D2C6	Polr3h	DNA-directed RNA polymerase III subunit RPC8	9737.835	8443.2	14786.48		10989	12659.41			7903.293	10281	1.0688	0.83212
P18608	Hmgn1	Non-histone chromosomal protein HMG-14	9212.736	2994.572	7134.915	6555.745	6474	3877.227	4643.603	10059.81	5653.986	6059	1.0686	0.83338
Q8BJL0	Smarcal1	SWI/SNF-related matrix-associated actin-dependent	7556.901	5151.408	7841.893	7359.739	6977	7799.249	6774.417	5478.162	8663.275	7179	0.9720	0.83447
Q8K1E6	Alkbh3	Alpha-ketoglutarate-dependent dioxygenase alkB ho	8173.295	7599.913	6443.364	5239.922	6864	7473.044	9946.161	4035.534	4715.947	6543	1.0491	0.83783
Q9ERU3	Znf22	Zinc finger protein 22	14315.16	6132.86	10634.8	9065.698	10037	8862.06	12681.42	8866.477	8045.061	9614	1.0440	0.83910
Q9DCK3	Tspan4	Tetraspanin-4	4929.432	5645.667	4201.188	7163.598	5485	5834.091	6172.764		4962.729	5657	0.9697	0.84001

Q6A051	Atrnl1	Attractin-like protein 1	17945.12	15402.31	17538.5	20840.31	17932	17946.55	18370.01	17923.95	16488.35	17682	1.0141	0.84118
Q9R207	Nbn	Nibrin	9440.134	11329.08	14064.77	14471.76	12326	13297.47	12047.51	13838.51	11227.8	12603	0.9781	0.84200
Q8BL99	Dop1a	Protein dopey-1	2733.006	2720.052	3377.066		2943	2778.677	2250.227		3529.501	2853	1.0317	0.84342
Q8C2S7	Amigo3	Amphoterin-induced protein 3	6744.107	6339.846	5543.048	2270.522	5224	3233.924	7586.064	4251.298	4690.563	4940	1.0575	0.84372
Q8CGB3	Uaca	Uveal autoantigen with coiled-coil domains and anky	87914.05	79743.38	104747.4	89325.3	90433	80921.64	75720.52	100561.9	112474.4	92420	0.9785	0.84944
Q9WTU0	Phf2	Lysine-specific demethylase PHF2	16209.86	18479.81	22376.08	24212.63	20320	19905.22	16215.24	18321.3	24796.31	19810	1.0257	0.84961
P01642	Gm10881	Ig kappa chain V-V region L7 (Fragment)	71518.55	24402.91			47961	32089.02	52805.79			42447	1.1299	0.85022
A4Q9F0	Ttll7	Tubulin polyglutamylase TTLL7	5697.691	8458.236	5883.099	5073.288	6278	4484.561	7481.928	5784.14	8239.656	6498	0.9662	0.85211
Q80UN1	Kctd9	BTB/POZ domain-containing protein KCTD9	25751.21	16969.38	24887.17	20826.01	22108	17129.85	24772.6	27286.88	21556.45	22686	0.9745	0.85271
P11679	Krt8	Keratin, type II cytoskeletal 8	15694.24	14384.22	25231.77	13044.48	17089	16547.71	16077.93	22782.57	10005.09	16353	1.0450	0.85311
Q5EBG8		Uncharacterized protein C1orf50 homolog	8346.181	8363.302	10939.77	3591.674	7810	8476.428	8260.086	9028.642	6713.726	8120	0.9619	0.85397
Q9R0E2	Plod1	Procollagen-lysine,2-oxoglutarate 5-dioxygenase 1	13625.41	15385.14	21663.09	13465.66	16035	13580.9	11508.32	17811.54	19218.26	15530	1.0325	0.85429
Q8BHK3	Slc36a2	Proton-coupled amino acid transporter 2	7869.915	6551.607	5476.986	7319.323	6804	8854.717	7345.374	5720.867	5976.655	6974	0.9756	0.85457
Q80WR1	Tspan18	Tetraspanin-18	27140.5	23752.25	22806.79	25657.53	24839	21520.77	29215.81	20930.1	26040.96	24427	1.0169	0.85678
P01638		Ig kappa chain V-V region L6 (Fragment)	28061.16	9065.163		6483.727	14537	10733.57	9904.91	28159.02		16266	0.8937	0.85761
Q9WTS2	Fut8	Alpha-(1,6)-fucosyltransferase	20294.62	22652.45	29307	23528.11	23946	19061.97	24571.32	23792.23	30630.22	24514	0.9768	0.85827
Q76N33	Stambpl1	AMSH-like protease	3171.695	3168.555	3824.109	3120.241	3321	4146.889	2897.112	2624.323	3340.023	3252	1.0212	0.85912
G3X982	Aox3	Aldehyde oxidase 3	352010.7	313695.8	365890.3	408499.6	360024	351039.8	308204	393296.7	409575.3	365529	0.9849	0.86047
Q5DTU0	Afap1l2	Actin filament-associated protein 1-like 2	13918.2	15234.22	16113.81	11877.99	14286	13851.93	11910.25	14709.64	15792.47	14066	1.0156	0.86434
Q07797	Lgals3bp	Galectin-3-binding protein	24255.39	12347.93	15416.65	17186.03	17301	20131.01	17357.18	15153.8	18500.79	17786	0.9728	0.86517
Q61469	Plpp1	Phospholipid phosphatase 1	6959.266	6776.304	5492.524	6646.156	6469	6805.497	8392.563	4675.915	5372.718	6312	1.0249	0.86549
O35099	Map3k5	Mitogen-activated protein kinase kinase kinase 5	8886.621	10074.1	9565.282	11639.01	10041	9915.846	10707.96	8417.894	10578.3	9905	1.0138	0.86816
Q80UG1	Fads6	Fatty acid desaturase 6	40173.7	35930.32	34860.49	35392.57	36589	32998.8	35844.24	35946.13	40251.67	36260	1.0091	0.87000
Q8BH74	Nup107	Nuclear pore complex protein Nup107	11390.47	13078.7	11001.99	9450.378	11230	13185.39	12204.5	6978.009	11494.49	10966	1.0241	0.87102
Q5HZJ5	Entrep3	Protein ENTREP3			5280.405	8242.486	6761	6883.444	6789.385	4283.157	8059.91	6504	1.0396	0.87187
P16879	Fes	Tyrosine-protein kinase Fes/Fps	12760.08	13854.57	16647.25	15128.13	14598	14047.41	11269.83	16925.46	17227.48	14868	0.9818	0.87376
Q8R3Y8	Irf2bp1	Interferon regulatory factor 2-binding protein 1	13979.64	13764.39	12664.74	11904.05	13078	14405.93	11205.29	12124.95	13979.41	12929	1.0115	0.87378
Q9Z2F2	Oasl2	2'-5'-oligoadenylate synthase-like protein 2	8093.338		2748.139	1312.957	4051	3664.469	4484.355	2887.704	3987.634	3756	1.0787	0.87404
Q8BXA1	Golim4	Golgi integral membrane protein 4	8105.44	11432.34	7687.533	9359.114	9146	10729.72	7743.373	7280.284	10072.84	8957	1.0212	0.87924
Q8BHC9	Fut11	Alpha-(1,3)-fucosyltransferase 11	11610.9	6329.35	10783.58	11700.51	10452	8973.228	10956.64	10783.58	12045.51	10690	0.9778	0.88087
P26350	Ptma	Prothymosin alpha	117832.8	148313.3	115792	120848.1	125697	144565.2	108115.9	147761.1	92630.18	123268	1.0197	0.88133
Q9CYD3	Crtap	Cartilage-associated protein	18055.76	11938.18	14159.94	6435.222	12647	16191.79	8469.01	12427.96	15333.02	13105	0.9650	0.88313
P97479	Myo7a	Unconventional myosin-VIIa	3722.466	4845.032	8294.733	6222.039	5771	4674.831	3679.084	4515.535	9240.284	5527	1.0441	0.88370
Q6A009	Ltn1	E3 ubiquitin-protein ligase listerin	21816.57	26659.38	21861.04	15231.42	21392	20936.79	26208.81	11168.3	24762.2	20769	1.0300	0.88479
Q8BJL1	Fbxo30	F-box only protein 30	947.7571	1489.904	579.2813	698.7369	929	1595.697	914.1907	654.6826	729.1126	973	0.9543	0.88493
Q8K296	Mtmr3	Myotubularin-related protein 3	11926.94	11070.5	10455.89	12560.03	11503	11087.19	14137.26	8285.641	11727.38	11309	1.0172	0.88533
Q9D2X5	Mau2	MAU2 chromatid cohesion factor homolog	5534.143	6043.896	5979.904	3763.685	5330	4722.529	4295.746		7436.619	5485	0.9718	0.88743
Q9CRA8	Exosc5	Exosome complex component RRP46	14017.11	12501.75	6160.511	10903.38	10896	13506.81	12004.31	7980.415	11306.44	11199	0.9729	0.88777
Q91W98	Slc15a4	Solute carrier family 15 member 4	22327.74	18943.76	18533.46	8510.892	17079	14293.98	23262.46	15763.74	17091.17	17603	0.9702	0.88823
Q9EP71	Rai14	Ankycorbin	19203.02	18544.69	20164.24	18714.07	19157	16887.46	18033.84	20620.79	20501.88	19011	1.0077	0.88845
Q5SZV5	Kiaa0319	Dyslexia-associated protein KIAA0319 homolog	14369.22	8554.056	10643.57	8568.98	10534	11316.56	10780.49	7589.4	11510.59	10299	1.0228	0.89140
Q8BUV8	Gpr107	Protein GPR107	6742.859	7207.341	4635.926	6086.987	6168	7568.84	9050.757	2051.309	5103.499	5944	1.0378	0.89493
O88551	Cldn1	Claudin-1	37555.75	35854.94	34435.7	42248.15	37524	39754.76	33618.7	35295.7	40139.23	37202	1.0086	0.89564
O70579	Slc25a17	Peroxisomal membrane protein PMP34	6299.039	4865.954	9379.381	5544.654	6522	5851.806	6555.923	4564.388	8418.578	6348	1.0275	0.89600
P0DW87	Zftraf1	Zinc finger TRAF-type-containing protein 1	3161.003	5585.751	6114.4	2042.876	4226	4156.42	6249.155	2838.193	2987.017	4058	1.0415	0.89732
Q8BGV0	Nars2	Probable asparagine--tRNA ligase, mitochondrial	4224.981	5688.809	3356.992	3752.252	4256	3346.439	5885.073		3895.031	4376	0.9726	0.89742
Q9WU66	Sfrp5	Secreted frizzled-related protein 5	12624.05	12623.66	12486.58	15899.51	13408	14169.52	10992.06	12987.34	14849.8	13250	1.0120	0.89782
B2RRE7	Otud4	OTU domain-containing protein 4	6350.501	8762.12	8154.295	10926.55	8548	5880.785	8988.041	6898.326	11613.27	8345	1.0244	0.90183
Q8BG19	Tmtc4	Protein O-mannosyl-transferase TMTC4	9106.804	8219.806	8480.356	6226.289	8008	8390.115	8436.682	4956.717	9641.599	7856	1.0194	0.90215
Q80V94	Ap4e1	AP-4 complex subunit epsilon-1	4209	2179.908	3561.36	1948.034	2975	4236.943	2361.869	1864.47	3052.278	2879	1.0332	0.90246
Q921X9	Pdia5	Protein disulfide-isomerase A5	19186	21972.22	25607.33	25641.45	23102	21278.51	22097.62	23383.37	24785.82	22886	1.0094	0.90560
Q91VW5	Golga4	Golgin subfamily A member 4	12287.82	9074.885	9658.715	6538.262	9390	8794.678	10526.82	6638.494	10847.73	9202	1.0204	0.90591
Q99JH8	Kdelr1	ER lumen protein-retaining receptor 1	16123.59	18903.81	14087.42	13762.72	15719	15877.75	18039.94	11944.2	16170.93	15508	1.0136	0.90755
Q9ERE9	Mapk8ip2	C-Jun-amino-terminal kinase-interacting protein 2		21591.82	19845.14	37901.75	26446	25796.97	38803.55	17028.43	27637.06	27317	0.9681	0.90790
Q3UX61	Naa11	N-alpha-acetyltransferase 11	29572.82	32346.66	29522.59	29959.66	30350	32399.67	27898.33	28188.87	32250.85	30184	1.0055	0.91004
P62700	Ypel5	Protein yippee-like 5		15289.45	12364.21	13144.02	13599	12932.56	11677.63	14930.51	15454.78	13749	0.9891	0.91088
Q8BXL7	Arfrp1	ADP-ribosylation factor-related protein 1	623.5802	4206.26	3356.02	2749.897	2734	1783.333	3432.153	1750.262	3551.698	2629	1.0398	0.91250
Q9Z103	Adnp	Activity-dependent neuroprotector homeobox protei	8187.119	9066.904	6927.073	9178.562	8340	10780.49	8204.167	6992.658	7825.108	8451	0.9869	0.91274
Q3UQ28	Pxdn	Peroxidasin homolog	4286.224	3527.476	7170.009	6242.833	5307	2714.791	2507.484	7087	8148.582	5114	1.0376	0.91309

Q6QI06	Rictor	Rapamycin-insensitive companion of mTOR	6769.934	4335.167	4524.55	2903.934	4633	5110.611	5053.541	3710.342	4271.615	4537	1.0214	0.91462
Q922B9	Itprid2	Protein ITPRID2	73036.05	64885.16	87454.61	82219.02	76899	70202.48	71699.85	70912.99	91591.2	76102	1.0105	0.91532
Q3TMP8	Tmem38a	Trimeric intracellular cation channel type A	40356.72	38930.64	34611.32	32571.44	36618	39294.37	33708.81	35122.18	39332.16	36864	0.9933	0.91881
Q99KW3	Triobp	TRIO and F-actin-binding protein	18243.26	20349.25	24902.72	19371.29	20717	19778.01	19212.88	19887.94	23254.43	20533	1.0089	0.91885
Q8CH40	Nudt6	Nucleoside diphosphate-linked moiety X motif 6	17891.12	21124.14	21063.49	18203.45	19571	19299.93	15791.57	20464.57	23498.01	19764	0.9902	0.91903
Q9WV70	Noc2l	Nucleolar complex protein 2 homolog	14756.76	13551.91	7669.337	12520.46	12125	11375.19	13612.4		10758.94	11916	1.0175	0.91985
Q8VCS3	Fam20b	Glycosaminoglycan xylosylkinase	15350.23	12491.5	11478.12	11478.13	12699	12753.15	13308.36	10010.56	15302.72	12844	0.9888	0.92267
Q8BMF8	Gldn	Gliomedin	9561.683	7429.4	11320.95	9106.449	9355	9637.646	9438.221	6866.225	11002.83	9236	1.0128	0.92314
P30412	Ppic	Peptidyl-prolyl cis-trans isomerase C	21492.25	20112.82	42243.48	20845.66	26174	20910.18	16415.13	31843.18	38473.6	26911	0.9726	0.92347
P97872	Fmo5	Flavin-containing monooxygenase 5	16993.89	18854	14096.68	16220.7	16541	13670.64	15837.96	18672.02	17400.13	16395	1.0089	0.92351
Q91VK1	Bzw2	eIF5-mimic protein 1	11611.32	11564.8	18382.13	12266.47	13456	11672.09	11038.37	17969.22	14050.49	13683	0.9835	0.92404
Q8BJA3	Hmbox1	Homeobox-containing protein 1	25572.6	25047.01	24901.55	26317.39	25460	24711.22	26113.83	23316.16	28123.99	25566	0.9958	0.92419
P70279	Surf6	Surfeit locus protein 6		7155.583	8551.431	7376.502	7695	5898.545	5820.12	7744.217	10753.35	7554	1.0186	0.92475
Q04646	Fxyd2	Sodium/potassium-transporting ATPase subunit gam	34086.83	28722.68	18593.63	29925.57	27832	31165.55	32901.34	20913.14	27950.26	28233	0.9858	0.92750
Q3UHI0	Ccser2	Serine-rich coiled-coil domain-containing protein 2	25428.1	22560.81	19357.21	17855.68	21300	21884.39	25047.54	16478	20865.53	21069	1.0110	0.92770
P01664;P01665;P01666;P01668		Ig kappa chain V-III region CBPC 101;Ig kappa chain V	4327.951	27025.17			15677	21201.92	7449.12			14326	1.0943	0.92819
P59281	Arhgap39	Rho GTPase-activating protein 39	35715.95	30893.81	29459.34	35419.71	32872	30386.01	29042.07	33427.52	37706.2	32640	1.0071	0.92890
Q9CQE7	Ergic3	Endoplasmic reticulum-Golgi intermediate compartm	54418.63	59290.35	53291.27	57666.92	56167	50715.36	51888.96	66216.8	54429.2	55813	1.0063	0.92909
Q61391	Mme	Neprilysin	19481.76	19121.15	24218.95	25244.42	22017	20520.88	18398.62	21918.79	26410.03	21812	1.0094	0.93261
P60605	Ube2g2	Ubiquitin-conjugating enzyme E2 G2	14488.21	7971.575	6844.434	8230.114	9384	11227.28	13282.97	4497.389	7618.677	9157	1.0248	0.93331
Q5ND29	Rilp	Rab-interacting lysosomal protein	20140.17	24647.3	23920.38	25718.36	23607	23334.09	23456.41	21721.74	26451.44	23741	0.9943	0.93430
P01635	Igkv12-41	Immunoglobulin kappa chain variable 12-41 (Fragme	10997.41	5726.794		5005.775	7243	10680.52	3147.027			6914	1.0477	0.93496
Q9D7V9	Naaa	N-acylethanolamine-hydrolyzing acid amidase	18872.19	16866.84	11927.42	15563.41	15807	16941.69	17152.32	15114.86	14561.78	15943	0.9915	0.93537
Q9Z0J1	Reck	Reversion-inducing cysteine-rich protein with Kazal r	12954.11	11529.57	14867.32	14898.06	13562	12778.92	11780.31	14700.36	14635.12	13474	1.0066	0.93773
Q4QQM5	Miga1	Mitoguardin 1	2991.104	2267.478	2161.39	2234.632	2414		2254.486		2624.435	2439	0.9894	0.93803
Q8K4D3	Slc36a1	Proton-coupled amino acid transporter 1	7796.683	7344.561	4728.347	6368.878	6560	6673.501	9040.185	4164.548	5968.088	6462	1.0152	0.93838
Q9Z329	Itpr2	Inositol 1,4,5-trisphosphate receptor type 2	5961.765	4209.385	5552.987		5241	4354.492	6285.104			5320	0.9853	0.94199
Q61107	Gbp3	Guanylate-binding protein 3	28247.88	15310.94	16113.1	10849.2	17630	18245.56	14690.18	16410.25	20023.8	17342	1.0166	0.94353
Q64282	Ifit1	Interferon-induced protein with tetratricopeptide rep	11584.71	4341.622	3926.538	4662.375	6129	7805.18	5429.247	5707.133	5005.941	5987	1.0237	0.94372
Q9ET38	Cldn19	Claudin-19	30310.8	29239.75	22008.46	26535.18	27024	30410.38	33261.56	24253.22	19090.04	26754	1.0101	0.94383
Q9CWG9	Bloc1s2	Biogenesis of lysosome-related organelles complex	12871.12	14600.3	12075.97	7858.929	11852		20068.07	5739.03	8862.401	11557	1.0255	0.94425
Q99N16;Q9EP75	Cyp4f3;Cyp4f14	Cytochrome P450 4F3;Leukotriene-B4 omega-hydro	6554.542	6107.378	9819.353	12743.35	8806	6368.925	7936.237	9076.46	12418.92	8950	0.9839	0.94530
Q3V209	Tmub2	Transmembrane and ubiquitin-like domain-containin	19189.4	15804.12	21589.78	28912.27	21374	23128.3	21700.23	18055.79	23479.11	21591	0.9900	0.94547
Q9Z315	Sart1	U4/U6.U5 tri-snRNP-associated protein 1	19246.78	20625.43	16450.23	16992.45	18329	20567.49	19382.98	17361.63	15605.97	18230	1.0054	0.94832
Q9WV68	Decr2	Peroxisomal 2,4-dienoyl-CoA reductase [(3E)-enoyl-	28745.92	29717.21	24855.31	26912.49	27558	30159.4	30586.31	24071.65	24890.79	27427	1.0048	0.95050
P52840	Sult1a1	Sulfotransferase 1A1	37773.29	32755.51	39762.47	32942.28	35308	31184.43	32062.95	44258.15	32855.28	35090	1.0062	0.95072
Q91XD2	Lims2	LIM and senescent cell antigen-like-containing doma	15881.88	16855.68	9458.419	11389.37	13396	16978.54	16729.06	7028.998	12139.76	13219	1.0134	0.95386
Q8C6K9	Col6a6	Collagen alpha-6(VI) chain	75941.96	22957.04	14869.15	28092.01	35465	16901.48	88537.74	18726.34	22969.78	36784	0.9641	0.95437
Q9Z0W1	Ngfr	Tumor necrosis factor receptor superfamily member	9934.946	10985.74	8321.83	9012.016	9564	6943.473	14231.31	9949.134	7548.208	9668	0.9892	0.95441
Q61103	Dpf2	Zinc finger protein ubi-d4		60825.97	64396.68	73513.08	66245	94892.82	55454.68	55551.99	61842.34	66935	0.9897	0.95488
Q8CIF6	Sidt2	SID1 transmembrane family member 2			3176.375	1795.006	2486	1854.571	2088.727		3669.743	2538	0.9795	0.95748
Q5D525	Syce1l	Synaptonemal complex central element protein 1-like		4234.505		13297.05	8766	6877.862	10075.57			8477	1.0341	0.95750
Q8BWW4	Larp4	La-related protein 4	18373.89	21327.39	22554.85	23943.62	21550	19481.81	20607.34	23010.03	23438.02	21634	0.9961	0.95753
Q8BVL9	Jakmip1	Janus kinase and microtubule-interacting protein 1	19513.3	23830.37	19582.94	21947.85	21219	22155.03	19119.7	19298.5	23965.08	21135	1.0040	0.95894
Q8BH47	Sec22a	Vesicle-trafficking protein SEC22a	16307.51	11111.42	15366.75	9591.757	13094	13165.77	14650.34	11726.69	13152.72	13174	0.9940	0.96485
P28828	Ptprm	Receptor-type tyrosine-protein phosphatase mu	11511.8	10100.56	8806.486	13912.25	11083	10267.09	10779.19		12381.43	11143	0.9946	0.96755
Q8BTZ5	Ankrd46	Ankyrin repeat domain-containing protein 46	27613.33	27991.34	29912.45	30000.61	28879	29645.63	30780.36	26279.79	29002.73	28927	0.9984	0.96807
Q9JLH8	Tmod4	Tropomodulin-4	28745.42	29190.67	20345.69	24607.47	25722	22982.68	31315.21	29276.74	19850.1	25856	0.9948	0.96970
A0A0G2JDV3	Gbp6	Guanylate-binding protein 6	9021.579	2622.899	3977.457	3016.348	4660	5847.317	4536.771	3730.793	4744.729	4715	0.9883	0.97258
Q9Z211	Pex11a	Peroxisomal membrane protein 11A		5589.954	7359.511	8475.139	7142	5769.613	8122.64	7625.622	6918.759	7109	1.0046	0.97348
Q8C7V8	Ccdc134	Coiled-coil domain-containing protein 134	12477.44	15971.12	13457.09	13844.12	13937	12975.01	15976.08	12426.85	14242.46	13905	1.0023	0.97705
P15655	Fgf2	Fibroblast growth factor 2	81806.1	72233.27	59290.05	105720.4	79762	82697.8	85940.68	69785.16	81837.55	80065	0.9962	0.97777
Q921Q3	Alg1	Chitobiosyldiphosphodolichol beta-mannosyltransfe	8520.864	10084.62	10735.81	11446.13	10197	8623.68	9855.775	9516.278	12672.76	10167	1.0029	0.97882
Q9JM51	Ptges	Prostaglandin E synthase	18152.54	16011.3	16548.42	20504.46	17804	18236.78	16026.49	16103.76	21010.52	17844	0.9977	0.98011
Q8BJS4	Sun2	SUN domain-containing protein 2	29200.76	23410.17	26183.84	30682.33	27369	28879.4	28573.59	26949.68	24893.62	27324	1.0017	0.98138
Q61245	Col11a1	Collagen alpha-1(XI) chain	23739.96	33473.39	29597.3	32281.27	29773	24107.66	39380.13	26164.96	29052.23	29676	1.0033	0.98160
Q921R8	Slc41a3	Solute carrier family 41 member 3		5122.69	3932.201	3745.343	4267	3384.703	5114.184	3312.235	5188.391	4250	1.0040	0.98207
P42867	Dpagt1	UDP-N-acetylglucosamine--dolichyl-phosphate N-ac	10145.23	13165.86	12805.48	12859.7	12244	12057.55	12022.32	9722.427	15075.31	12219	1.0020	0.98552
Q9CXY1	Tmem175	Endosomal/lysosomal proton channel TMEM175	5366.796	5689.251	4272.777	5195.156	5131	7315.302	6116.965		1882.013	5105	1.0051	0.98602

Q921U8	Smtn	Smoothelin	27504.84	24528.69	30109.33	29298.5	27860	23961.28	24825.38	27942.28	34908.99	27909	0.9982	0.98645
Q3UFY8	Trmt10c	tRNA methyltransferase 10 homolog C		12519.62	9409.073	9401.715	10443	10209.16	19081.6	4233.772	8416.244	10485	0.9960	0.99167
Q99LL5	Pwp1	Periodic tryptophan protein 1 homolog	4714.013	4960.929	2849.843	1570.703	3524	3850.113	4368.602	1787.854	4128.544	3534	0.9972	0.99240
O88329	Myo1a	Unconventional myosin-Ia	17092.9	11584.42	14015.64	11465.5	13540	13392.82	13608.98	11422.34	15679.51	13526	1.0010	0.99337
P46938	Yap1	Transcriptional coactivator YAP1	18664.7	19262.01	18258.89	23621.75	19952	19376.94	18016.75	19186.4	23285.26	19966	0.9993	0.99343
Q5SSW2	Psme4	Proteasome activator complex subunit 4	3657.678	3546.877	3078.281	2598.025	3220	3636.15	3512.573	3016.844	2721.539	3222	0.9995	0.99630
Q9D4H2	Gcc1	GRIP and coiled-coil domain-containing protein 1	3475.598	2348.511	5751.435	1771.598	3337	3835.059		2293.526	3866.745	3332	1.0015	0.99663
P04441	Cd74	H-2 class II histocompatibility antigen gamma chain	17563.47	4862.204	6435.843	3883.791	8186	8642.733	9659.399		6208.663	8170	1.0020	0.99683
Q9DCT6	Bap18	Chromatin complexes subunit BAP18	37049.13	19182.72	24925.04	18772.16	24982	43479.7	17357.21	18525.91	20685.77	25012	0.9988	0.99696
Q8K124	Plekho2	Pleckstrin homology domain-containing family O me	15821.52	15666.95	14628.11	10715.09	14208	13465.82	20817.83	11293.05	11280.58	14214	0.9995	0.99808
Q8CAK3	Shfl	Shiftless antiviral inhibitor of ribosomal frameshifting	1602.516	607.0366	2089.451	718.9722	1254	945.4989	1565.076			1255	0.9994	0.99895
P01630		Ig kappa chain V-II region 7S34.1	142197.9	13386.2			77792		8403.692			8404	9.2569	
Q80TL1	Adcy2	Adenylate cyclase type 2		8745.616	6801.373	6813.119	7453			2290.029		2290	3.2547	
Q9D9K3	Aven	Cell death regulator Aven		580.4714	588.9896		585		189.1237			189	3.0918	
P01674		Ig kappa chain V-III region PC 2154	14684.48				14684		4781.441			4781	3.0711	
O88593	Pglyrp1	Peptidoglycan recognition protein 1	6205.964	2194.016	4812.684	9180.948	5598		1898.016			1898	2.9496	
Q3UU94	Mansc4	MANSC domain-containing protein 4		12307.69			12308			5176.276		5176	2.3777	
P01844	Iglc2	Ig lambda-2 chain C region	11848.04	7499.514			9674		4206.231			4206	2.2999	
P18525		Ig heavy chain V region 5-84	7355.87	7299.916			7328	3207.965				3208	2.2843	
Q3T9E4;Q62293	Tgtp2;Tgtp1	T-cell-specific guanine nucleotide triphosphate-bind	49019.84				49020	25393.16			22781.42	24087	2.0351	
Q3U1C6	Tatdn3	Putative deoxyribonuclease TATDN3	17470.42				17470		9464.852			9465	1.8458	
P01670;P01671;P01672		Ig kappa chain V-III region PC 6684;Ig kappa chain V-	30447.17	20424.68			25436		17036.22			17036	1.4930	
P59913	Pcmtd1	Protein-L-isoaspartate O-methyltransferase domain-	6486.374	3710.509	4630.468		4942		3564.271			3564	1.3867	
P97425	Ear2	Eosinophil cationic protein 2	50375.28	29095.74	21267.89	19007.79	29937		21627.81			21628	1.3842	
Q9DBR0	Akap8	A-kinase anchor protein 8		3977.828			3978	1947.296	4117.735			3033	1.3117	
Q8BYM8	Cars2	Probable cysteine--tRNA ligase, mitochondrial	7877.583	7652.395	7028.517	7894.516	7613		5869.648			5870	1.2971	
P01639	Igkv9-120	Immunoglobulin kappa chain variable 9-120	38068.18	23597.69			30833		24110.01			24110	1.2788	
Q8BT42	Smim5	Small integral membrane protein 5				7014.97	7015		5486.062			5486	1.2787	
Q8CFD4	Snx8	Sorting nexin-8			1.265205		1	1				1	1.2652	
Q8BMS9;Q8CB96	Rassf2;Rassf4	Ras association domain-containing protein 2;Ras as	18581.5	18143.53	30689.63		22472				17938.23	17938	1.2527	
Q62273	Slc26a2	Sulfate transporter	4919.826	2520.741	2072.204		3171		2539.393			2539	1.2487	
Q9JMA2	Qtrt1	Queuine tRNA-ribosyltransferase catalytic subunit 1	5540.191	5635.842	8440.955		6539		5262.301			5262	1.2426	
Q8BPB0;Q921Y0	Mob1b;Mob1a	MOB kinase activator 1B;MOB kinase activator 1A	3752.642	5831.537	3678.037	1664.877	3732				3133.532	3134	1.1909	
Q9CVD2	Atxn3	Ataxin-3			4408.305		4408		2689.869		5077.431	3884	1.1351	
P70662	Ldb1	LIM domain-binding protein 1		19528.04			19528	16203.51	17537.81	20090.7	15997.77	17457	1.1186	
Q9DC33	Hmg20a	High mobility group protein 20A				5857.06	5857		4022.154		6785.056	5404	1.0839	
O35654	Pold2	DNA polymerase delta subunit 2		3243.005	4672.791	6281.673	4732	4823.622				4824	0.9811	
P04223	H2-K1	H-2 class I histocompatibility antigen, K-K alpha chai	19106.55	14264.04			16685	18365.9				18366	0.9085	
P29452	Casp1	Caspase-1	9250.047		4449.99	5690.896	6464				7227.557	7228	0.8943	
Q9Z2V5	Hdac6	Histone deacetylase 6	19200.23	14286.32	16629.54		16705		19619.62			19620	0.8515	
Q2VPA6	Helq	Helicase POLQ-like			1054.672		1055	1487.079			996.703	1242	0.8492	
O35701	Matn3	Matrilin-3		9132.628	12150.08		10641		12601.89			12602	0.8444	
Q5PSV9	Mdc1	Mediator of DNA damage checkpoint protein 1	2290.132	2854.915	754.9807		1967				2340.526	2341	0.8403	
Q9CQX0	Otub2	Ubiquitin thioesterase OTUB2	898.7935	2544.098	1110.451		1518	1836.775				1837	0.8263	
O08912	Galnt1	Polypeptide N-acetylgalactosaminyltransferase 1		3643.179			3643				4520.006	4520	0.8060	
Q9QWV9	Ccnt1	Cyclin-T1	1279.451	785.9174	452.3528	1019.78	884				1098.548	1099	0.8050	
Q9Z1S3	Rasgrp1	RAS guanyl-releasing protein 1	5920.674				5921	7657.582		8456.951	7528.511	7881	0.7513	
P09542	Myl3	Myosin light chain 3		7504.127		7945.544	7725		10345.88			10346	0.7467	
Q91YJ3	Thyn1	Thymocyte nuclear protein 1	8870.955	7774.252		7557.741	8068		11004.76			11005	0.7331	
Q91VB4	Hps3	BLOC-2 complex member HPS3	965.2304				965	818.8712	2120.822	1059.932		1333	0.7240	
P01728		Ig lambda-2 chain V region	6971.251				6971		16191.87			16192	0.4305	
Q5DTN8	Jakmip3	Janus kinase and microtubule-interacting protein 3							27540.34			27540	0.0000	
P11862	Gas2	Growth arrest-specific protein 2							3694.932			3695	0.0000	
P54763	Ephb2	Ephrin type-B receptor 2								5473.63	4323.21	4898	0.0000	
Q9DAT5	Trmu	Mitochondrial tRNA-specific 2-thiouridylase 1							1650.563			1651	0.0000	
A2AHL1	Ano3	Anoctamin-3									4561.369	4561	0.0000	
Q61781	Krt14	Keratin, type I cytoskeletal 14									10093.86	10094	0.0000	
Q7TMD7	Dsg4	Desmoglein-4									61781.94	61782	0.0000	
P01629		Ig kappa chain V-II region 2S1.3						21571.88				21572	0.0000	

P19426	Nelfe	Negative elongation factor E							6222.071		4490.431			**5356**	**0.0000**	[redacted]
Q7M6Z0	Rtn4rl2	Reticulon-4 receptor-like 2									4379.829	2973.807		**3677**	**0.0000**	[redacted]
Q9Z1P7	Kank3	KN motif and ankyrin repeat domain-containing protein 3									2421.785			**2422**	**0.0000**	[redacted]
Q3TXX3	Zfyve27	Protrudin							1316.003		486.9966	519.9932		**774**	**0.0000**	[redacted]
Q8VHG2	Amot	Angiomotin														[redacted]
Q3UBG2	Pid1	PTB-containing, cubilin and LRP1-interacting protein														[redacted]
P59808	Sash1	SAM and SH3 domain-containing protein 1														[redacted]
Q91YK0	Lrrc49	Leucine-rich repeat-containing protein 49														[redacted]
P63042	Stmn4	Stathmin-4														[redacted]
Q9D0I6	Wdsub1	WD repeat, SAM and U-box domain-containing protein 1			1541.248			**1541**								[redacted]
Q3TXX4	Slc17a7	Vesicular glutamate transporter 1														[redacted]
Q99J27	Slc33a1	Acetyl-coenzyme A transporter 1														[redacted]
Q8BTR5	Dusp28	Dual specificity phosphatase 28														[redacted]
Q6WQJ1	Dagla	Diacylglycerol lipase-alpha														[redacted]
Q8R4K2	Irak4	Interleukin-1 receptor-associated kinase 4	2886.841	866.4799	2106.724			**1953**								[redacted]
Q3U0D9	Hace1	E3 ubiquitin-protein ligase HACE1														[redacted]
Q6PAN7	Prr18	Proline-rich protein 18														[redacted]
Q9R1W5	Calcrl	Calcitonin gene-related peptide type 1 receptor														[redacted]
D3YVF0	Akap5	A-kinase anchor protein 5														[redacted]
Q9QXW9	Slc7a8	Large neutral amino acids transporter small subunit 2														[redacted]
Q8BL06	Usp54	Inactive ubiquitin carboxyl-terminal hydrolase 54														[redacted]
Q9ES07	Slc15a2	Solute carrier family 15 member 2														[redacted]
Q64387	Pnoc	Prepronociceptin														[redacted]
P21661	Pcsk2	Neuroendocrine convertase 2														[redacted]
Q8VCE1	Dnajc28	DnaJ homolog subfamily C member 28														[redacted]
Q2TA57	Asphd1	Aspartate beta-hydroxylase domain-containing prote	7275.182	7799.266	11859.82	11450.82		**9596**								[redacted]
Q0GUM3	Gm12250	Interferon-gamma-inducible GTPase 10	8003.65					**8004**								[redacted]
P29351	Ptpn6	Tyrosine-protein phosphatase non-receptor type 6	7518.788	4323.894	7111.07			**6318**								[redacted]
Q62230	Siglec1	Sialoadhesin	3506.175	3551.454				**3529**								[redacted]
P35991	Btk	Tyrosine-protein kinase BTK	2519.724	2618.352	3111.071			**2750**								[redacted]
Q9Z0T9	Itgb6	Integrin beta-6	1481.883		2941.724			**2212**								[redacted]
Q3U2J5	Camkmt	Calmodulin-lysine N-methyltransferase			1312.712	1463.626		**1388**								[redacted]
