# Supplementary material for: Sodium channels Nav1.7, Nav1.8 and pain; two distinct mechanisms for Nav1.7 null analgesia
Source: Neurobiol Pain. 2024 Oct 11;16:100168. doi: 10.1016/j.ynpai.2024.100168 (PMC11570969; doi:10.1016/j.ynpai.2024.100168)
Supplement: Supplementary Data 2 [file mmc2.pdf]

Supplementary figure 2 - DRG central terminals

Individual mouse values for defined proteins (vertical columns) were averaged and null mutant data compared with normal mouse data. The fold change and p value are shown in the right hand side 2 columns

Central terminals analysed by Spectronaut v18.7
Male samples Black text
Female samples Red text
Control mice Blue cells
Nav1.7 null mice Yellow cells

PG.ProteinGroups	PG.Genes	PG.ProteinDescriptions	[9] 10042024_P12	[10] 10042	[13] 10042	[14] 10042	AVERAGES	[11] 10042	[12] 10042	[15] 10042	[16] 10042	AVERAGES	Fold-Change (KO/WT)	TTESTS KO-WT
Q3UPR9	Sbspon	Somatomedin-B and thrombospondin type-1 domain-containing protein			32527.85	3165.66	**17846.75403**				91.18294	**91.18293762**	**195.7247**	
O88327	Ctnnal1	Alpha-catulin	22646.16602	8943.888	3.710267		**10531.25466**				115.5549	**115.554924**	**91.1364**	
P27573	Mpz	Myelin protein P0	206325.5625	143871	10114712	408048.3	**2718239.207**	44227.76	23592.02	110653.9	110108.7	**72145.58008**	**37.6771**	**0.3245**
P24526	Pmp2	Myelin P2 protein		5203.419	271686.3	23296.13	**100061.9437**				4022.95	**4022.950195**	**24.8728**	
P13542	Myh8	Myosin-8		742812.5	397.4811	519.6915	**247909.8909**			24150.75	2702.523	**13426.63562**	**18.4640**	**0.5162**
P11087	Col1a1	Collagen alpha-1(I) chain	10351.82129	5390.848	444597.6	28046.7	**122096.7318**	3822.116	9191.69	6844.219	7282.125	**6785.037598**	**17.9950**	**0.3251**
Q8BVA5	Ldah	Lipid droplet-associated hydrolase	13040.79004	10579.76		424362	**149327.5173**	11651.41		12492.96	10616.63	**11587.00065**	**12.8875**	**0.3732**
P59281	Arhgap39	Rho GTPase-activating protein 39		21356.73		13146.09	**17251.40771**			1437.938		**1437.937744**	**11.9973**	
Q80X19	Col14a1	Collagen alpha-1(XIV) chain	5197.958496	34952.99	7014.25		**15721.7321**	1.552736		3038.712		**1520.132203**	**10.3423**	**0.3379**
O08784	Tcof1	Treacle protein		32757.67	1802.598		**17280.13568**	1477.057	1716.148	2699.558	1500.085	**1848.212067**	**9.3496**	**0.1792**
Q8R420	Abca3	Phospholipid-transporting ATPase ABCA3	5271.810059		3430.352	70326.75	**26342.9707**	4231.675	1949.065			**3090.369873**	**8.5242**	**0.4731**
P05555	Itgam	Integrin alpha-M			123967.1		**123967.0547**		15032.45			**15032.45313**	**8.2466**	
P11589	Mup2	Major urinary protein 2				36613.73	**36613.73047**			2777.125	8396.19	**5586.657593**	**6.5538**	
Q9CYI0		Protein Njmu-R1	3519.254639	18566.18	26667.1	34146.47	**20724.75067**	2840.721	3424.589	3798.481	3513.651	**3394.360352**	**6.1056**	**0.0385**
Q99KD5	Unc45a	Protein unc-45 homolog A			23107.8		**23107.79688**			3447.418	4261.765	**3854.591553**	**5.9949**	
Q03734	Serpina3m	Serine protease inhibitor A3M	5580.84082				**5580.84082**				1002.881	**1002.880615**	**5.5648**	
Q8BHJ5	Tbl1xr1	F-box-like/WD repeat-containing protein TBL1XR1	5373.089355	5118.077			**5245.583252**				1045.395	**1045.394897**	**5.0178**	
Q9ER64	Osbpl5	Oxysterol-binding protein-related protein 5			5214.033		**5214.033203**			1051.407		**1051.407227**	**4.9591**	
O55103	Prx	Periaxin	11795.67773	4734.108	837851.9	50086.03	**226116.9387**	9168.492	4062.193	170965.2	7585.511	**47945.35779**	**4.7161**	**0.4250**
Q04857	Col6a1	Collagen alpha-1(VI) chain	2101.284912	7534.817	92858.64	4848.563	**26835.82635**	3701.584	3230.231	6118.102	9986.401	**5759.079712**	**4.6597**	**0.3769**
P97298	Serpinf1	Pigment epithelium-derived factor		2259.607	3668.856	22347.12	**9425.193929**				2048.353	**2048.353271**	**4.6014**	
Q01149	Col1a2	Collagen alpha-2(I) chain		179130.8	113118.9	1420.342	**97889.98645**				21558.91	**21558.91406**	**4.5406**	
Q0VBL3	Rbm15	RNA-binding protein 15		1212.646	1910.797	12157.67	**5093.705729**				1155.562	**1155.56189**	**4.4080**	
Q8CJ67	Stau2	Double-stranded RNA-binding protein Staufen homolog 2		1800.88		32959.08	**17379.98126**			4041.613		**4041.612793**	**4.3003**	
Q8BH61	F13a1	Coagulation factor XIII A chain			9440.162		**9440.162109**				2297.264	**2297.264404**	**4.1093**	
P08071	Ltf	Lactotransferrin			19853.29		**19853.29492**				4900.5	**4900.5**	**4.0513**	
P51885	Lum	Lumican	578165.5625		59230.26		**318697.9121**		86269.84			**86269.84375**	**3.6942**	
Q8BH47	Sec22a	Vesicle-trafficking protein SEC22a		12653.37			**12653.37207**		3433.57			**3433.570313**	**3.6852**	
Q60675	Lama2	Laminin subunit alpha-2	6410.359375	7828.83	94842.9	9906.84	**29747.23181**	7031.427	7636.724	10950.58	7946.934	**8391.417236**	**3.5450**	**0.3636**
E9PXF8	Sbf2	Myotubularin-related protein 13	17782.08594		8515.059	5156.089	**10484.41113**	1767.131		4160.785	3209.344	**3045.753011**	**3.4423**	**0.1247**
P51954	Nek1	Serine/threonine-protein kinase Nek1	22162.50586	13417.22	5807.191		**13795.64046**		812.5701		7223.271	**4017.920776**	**3.4335**	**0.2324**
Q9D1G2	Pmvk	Phosphomevalonate kinase		9692.16			**9692.160156**		3552.422	2319.11		**2935.765747**	**3.3014**	
Q61475	Cd55	Complement decay-accelerating factor, GPI-anchored			7307.871		**7307.870605**			2265.601		**2265.60083**	**3.2256**	
O35566	Cd151	CD151 antigen	2329.104248	2009.793	6640.584	1922.576	**3225.514435**	115.7373	970.7565	2049.304		**1045.26608**	**3.0858**	**0.1878**
A2APX8	Scn1a	Sodium channel protein type 1 subunit alpha	10944.14648	9856.062	38178.87	42351	**25332.5188**	8829.823	6892.962	11851.4	5495.668	**8267.46228**	**3.0641**	**0.0998**
P06728	Apoa4	Apolipoprotein A-IV		91260.41	38039.08	26665.4	**51988.29688**			18413.89	17826.3	**18120.09961**	**2.8691**	**0.2792**
P51642	Cntf	Ciliary neurotrophic factor	5511.419922	8387	43497.72	7791.678	**16296.95422**	5194.436	6354.582		5606.242	**5718.419759**	**2.8499**	**0.3706**
P11835	Itgb2	Integrin beta-2			4446.872	1311.91	**2879.391235**	1635.795		564.6363	859.9952	**1020.142069**	**2.8225**	**0.2304**
Q8BT42	Smim5	Small integral membrane protein 5				18113.33	**18113.32813**	5111.042	8033.651			**6572.346436**	**2.7560**	
Q9CZA6	Nde1	Nuclear distribution protein nudE homolog 1	24713.68359	49147.86	31606.35	81960.33	**46857.05566**	14102.69	17810.86	19796.71	16762.72	**17118.24487**	**2.7373**	**0.0597**
Q9WU60	Atrn	Attractin	4606.280273	4698.731	3838.112	31219.03	**11090.53925**		4115.857	4626.856	3490.881	**4077.865072**	**2.7197**	**0.4179**
Q9R233	Tapbp	Tapasin			13865.25		**13865.24609**		5239.946		5033.083	**5136.514404**	**2.6993**	
Q497V5	Srbd1	S1 RNA-binding domain-containing protein 1		831.6463		38.95887	**435.3025875**		161.5006			**161.5005646**	**2.6954**	
P02469	Lamb1	Laminin subunit beta-1	5752.035645	5329.41	72637.92	4530.13	**22062.37451**	4145.612	9702.458	13307.01	6725.813	**8470.222656**	**2.6047**	**0.4538**
Q8CI04	Cog3	Conserved oligomeric Golgi complex subunit 3		2566.186		7105.172	**4835.678833**	2019.134		1694.789		**1856.961548**	**2.6041**	**0.3207**
Q08879	Fbln1	Fibulin-1	11590.56738		1932.508		**6761.537598**	2175.23		3048.691		**2611.960449**	**2.5887**	**0.4823**
Q3URQ0	Tex10	Testis-expressed protein 10			20140.75	10741.2	**15440.97217**	6954.774	8713.108	4132.552	4093.108	**5973.385681**	**2.5850**	**0.0472**
Q01339	Apoh	Beta-2-glycoprotein 1	4681.777832	3135.679	12370.71		**6729.388428**	1634.808	3656.425			**2645.616516**	**2.5436**	**0.3572**
Q8BMD8	Slc25a24	Mitochondrial adenyl nucleotide antiporter SLC25A24	27182.76367	16329.72	5794.827		**16435.76888**	5052.144		7771.848	6563.648	**6462.546712**	**2.5432**	**0.1843**
P49817	Cav1	Caveolin-1	8444.133789	5714.254	15324.33	55325.45	**21202.04102**	6965.601		8403.853	9798.854	**8389.43571**	**2.5272**	**0.3922**
Q8BX02	Kank2	KN motif and ankyrin repeat domain-containing protein 2			5492.87		**5492.870117**			2187.114		**2187.114258**	**2.5115**	
Q9DCN1	Nudt12	NAD-capped RNA hydrolase NUDT12		14470.67		3950.837	**9210.752563**			3677.474		**3677.473633**	**2.5046**	
P82349	Sgcb	Beta-sarcoglycan			11593.83	46993.98	**29293.90527**		4892.867	10915.23	20212.2	**12006.76774**	**2.4398**	**0.3159**
Q9CW79	Golga1	Golgin subfamily A member 1	6282.258301	5089.666	3222.118	3141.7	**4433.935608**	1014.078		2646.879		**1830.478607**	**2.4223**	**0.1055**

O35206	Col15a1	Collagen alpha-1(XV) chain	25279.42578	26470.3	157756.9	30688.91	**60048.87793**	30742.93	21246.4	21332.34	26294.3	**24903.99414**	2.4112	0.3234
Q61703	Itih2	Inter-alpha-trypsin inhibitor heavy chain H2	1924.805542	3848.652			**2886.728943**				1200.805	**1200.805298**	2.4040	
P15864	H1-2	Histone H1.2	36054.08203	35031.73	61107.18	22594.21	**38696.79932**	1220.57	32082.99	13222.97	18138.21	**16166.18466**	2.3937	0.0712
P10493	Nid1	Nidogen-1	42500.15234	41290.11	231377.2	54997.73	**92541.30273**	35480.11	40528.13	41166.38	42051.23	**39806.46387**	2.3248	0.2991
P61620	Sec61a1	Protein transport protein Sec61 subunit alpha isoform 1	26216.18359	15128.04	4012.241	9169.831	**13631.57452**			4750.453	6982.227	**5866.339844**	2.3237	0.3408
Q80UG1	Fads6	Fatty acid desaturase 6	10906.44824				**10906.44824**			4717.719		**4717.719238**	2.3118	
P30681	Hmgb2	High mobility group protein B2	6438.521484		9296.725	6540.595	**7425.280273**	3276.583				**3276.583496**	2.2662	
Q571I9	Aldh16a1	Aldehyde dehydrogenase family 16 member A1	3783.277588	2812.122			**3297.699585**				1455.776	**1455.776123**	2.2653	
Q9CPR5	Mrpl15	Large ribosomal subunit protein uL15m	6242.305176	27626.22	5098.215	7772.663	**11684.85095**	3917.145	5326.516	4639.944	7038.359	**5230.490967**	2.2340	0.2758
Q03311	Bche	Cholinesterase			7091.169		**7091.169434**			3196.653		**3196.653076**	2.2183	
Q6NZN0	Rbm26	RNA-binding protein 26	7004.42627		8374.624		**7689.525146**	3081.972		6554.721	782.947	**3473.213277**	2.2140	0.1544
Q3V132	Slc25a31	ADP/ATP translocase 4	33265.82031	39741.34	20412.41	19130.59	**28137.54004**	7658.741	18103.53	18676.09	7339.778	**12944.53467**	2.1737	0.0425
Q91WR3	Ascc2	Activating signal cointegrator 1 complex subunit 2			4831.466	7693.641	**6262.553467**	3551.818		2737.874	2403.236	**2897.642741**	2.1613	0.0617
P82347	Sgcd	Delta-sarcoglycan			32031.92		**32031.92188**				14847.01	**14847.00781**	2.1575	
Q8BJS4	Sun2	SUN domain-containing protein 2			17120.7		**17120.69531**			7399.319	8553.21	**7976.264648**	2.1465	
P98197	Atp11a	Phospholipid-transporting ATPase IH	7528.022949	4073.853		7640.681	**6414.185384**	2152.713	2768.137	3485.106	3611.05	**3004.251587**	2.1350	0.0234
Q8R3L2	Tcf25	Ribosome quality control complex subunit TCF25	4113.888184		7995.956		**6054.922119**		2982.623		2704.563	**2843.593018**	2.1293	0.2407
P24452	Capg	Macrophage-capping protein	12155.55273	12451.31	49551.66	16309.02	**22616.88452**	11022.65	11814.34	8263.841	11777.84	**10719.66968**	2.1098	0.2374
Q8CI59	Steap3	Metalloreductase STEAP3	8637.262695	4686.704	27029.9	9791.569	**12536.35815**		6284.497	5009.891	6533.703	**5942.696777**	2.1095	0.3126
Q60648	Gm2a	Ganglioside GM2 activator	23764.2832	3425.837			**13595.06018**		3431.327	6621.255	9337.16	**6463.247477**	2.1034	0.4329
P16015	Ca3	Carbonic anhydrase 3	3008.494385	4302.837	86944.06	7893.986	**25537.34503**		12416.45			**12416.45215**	2.0567	
P06797	Ctsl	Procathepsin L		11794.04	1853.876	14.25254	**4554.056594**	1770.791		2663.872		**2217.331238**	2.0538	0.6555
Q8VHG2	Amot	Angiomotin				5007.926	**5007.925781**	1578.483	2666.737		3133.332	**2459.517578**	2.0361	
Q9Z2L6	Minpp1	Multiple inositol polyphosphate phosphatase 1		16616.31		2745.046	**9680.677124**			4760.475		**4760.474609**	2.0336	
Q8JZZ7	Adgrl2	Adhesion G protein-coupled receptor L2	6189.51709	33666.79	2227.723	10383.3	**13116.83112**	6232.429	8207.09	5668.983	5900.15	**6502.162842**	2.0173	0.3858
P02468	Lamc1	Laminin subunit gamma-1	24414.26563	26531.77	134611	30759.73	**54079.18604**	24964.05	25816.21	27552.8	29177.29	**26877.58496**	2.0121	0.3508
P05622	Pdgfrb	Platelet-derived growth factor receptor beta	69792.04688	88199.41	77047.34	84360.12	**79849.72852**	67674.44	69016.59	10831.69	12503.62	**40006.58276**	1.9959	0.0561
O70258	Sgce	Epsilon-sarcoglycan		3447.162	13083.42	3808.468	**6779.682048**			2420.908	4430.154	**3425.531006**	1.9792	0.4771
Q9D385	Arl2bp	ADP-ribosylation factor-like protein 2-binding protein		17835.57		28882.53	**23359.05078**		15709.41	7983.167		**11846.28735**	1.9718	0.2298
Q9CQW0	Emc6	ER membrane protein complex subunit 6		10769.85	7863.97		**9316.908447**			3619.911	5973.445	**4796.678345**	1.9424	0.1368
Q8BJZ3	Tmbim1	Protein lifeguard 3			2668.399		**2668.399414**			1626.395	1124.049	**1375.221558**	1.9403	
Q7TNV1	Tlcd3b	Ceramide synthase	26491.47461	18380.87	22185.57	20156.27	**21803.5459**	17519.93	4722.813	6161.55	16724.5	**11282.19812**	1.9326	0.0328
Q31125	Slc39a7	Zinc transporter SLC39A7			13359.02	10556.2	**11957.61182**	9.091944		9808.682	8966.818	**6261.530648**	1.9097	0.2663
Q8CCF0	Prpf31	U4/U6 small nuclear ribonucleoprotein Prp31	11157.84961	20680.45	5486.773	4706.37	**10507.86133**	4611.296	3297.689	5484.834	8622.953	**5504.192993**	1.9091	0.2418
Q8VEH8	Erlec1	Endoplasmic reticulum lectin 1	9795.051758	5747.473	18086.47	13564.82	**11798.45288**	8943	2892.705	8204.429	4871.251	**6227.84613**	1.8945	0.1121
Q9CR29	Ccdc43	Coiled-coil domain-containing protein 43	23170.29297	8295.903	87866.98	17796.38	**34282.39136**	14977.02	15961.81	16583.74	24862.05	**18096.15503**	1.8945	0.4097
P22907	Hmbs	Porphobilinogen deaminase	14270.48047	26950.7	39795.2	42498.61	**30878.74902**	15387.05	12815.03	25268.27	11976.71	**16361.76733**	1.8873	0.0895
Q80X73	Pelo	Protein pelota homolog	5720.568848	46420.4	47515.71	65196.54	**41213.30627**	24854.63		2509.446	38252.55	**21872.20915**	1.8843	0.3134
Q9JJ80	Rpf2	Ribosome production factor 2 homolog			2111.601		**2111.600586**			1125.053		**1125.053467**	1.8769	
Q61739	Itga6	Integrin alpha-6	28820.70508	22553.59	97075.35	25615.73	**43516.3457**	27730.69	21768.73	22509.55	21069.39	**23269.58984**	1.8701	0.3027
P97927	Lama4	Laminin subunit alpha-4	25570.37891	25549.64	82973.63	36085.18	**42544.70654**	22484.88	26253.67	23291.76	19736.77	**22941.771**	1.8545	0.2044
Q64324	Stxbp2	Syntaxin-binding protein 2	26530.97266	23926.16	17366.8	22828.76	**22663.17285**	22504.67	11012.55	5917.815	9835.585	**12317.65601**	1.8399	0.0434
Q5XKE0	Mybpc2	Myosin-binding protein C, fast-type			18300.18	5416.212	**11858.19409**	5530.89			7493.537	**6512.213135**	1.8209	0.4982
Q62383	Supt6h	Transcription elongation factor SPT6		11910.58	15744.67	16350.1	**14668.45052**	4344.318		11283.67	8558.588	**8062.192708**	1.8194	0.0543
Q8K441	Abca6	ATP-binding cassette sub-family A member 6		2532.978	5566.585	1991.892	**3363.818278**	1389.781		2725.68	1438.319	**1851.259969**	1.8170	0.2744
P11276	Fn1	Fibronectin	4834.510742	3234.358	11366.21	5704.011	**6284.773438**	3194.314	3750.72	3746.588	3174.255	**3466.469421**	1.8130	0.1638
P08207	S100a10	Protein S100-A10	35917.38672	27721.7	71886.6	38175.57	**43425.31543**	32573.69	12924.63	22915.03	27633.76	**24011.77881**	1.8085	0.1171
Q3ZK22	Vezt	Vezatin			1590.186		**1590.186157**			880.8694		**880.8694458**	1.8052	
Q80X82	Sympk	Symplekin	1231.808716	2118.905	4165.678	6173.795	**3422.546844**	217.4668			3595.009	**1906.237671**	1.7954	0.4807
Q8BG67	Efr3a	Protein EFR3 homolog A	8756.112305	9110.801	10113.57	14199.6	**10545.02124**	7238.132	5643.548	4322.411	6432.727	**5909.20459**	1.7845	0.0160
P39061	Col18a1	Collagen alpha-1(XVIII) chain	2461.167236	8129.546	17405.09	6086.418	**8520.554993**	6493.332	3508.998	6506.178	2610.341	**4779.71228**	1.7827	0.3057
Q9QZX7	Srr	Serine racemase		31928.55	31544.15		**31736.35059**			31539.28	4103.576	**17821.42554**	1.7808	0.4172
Q8R2Z5	Vwa1	von Willebrand factor A domain-containing protein 1	9211.322266	10031.61	33999.42	8666.916	**15477.31543**	8296.505	8797.092	8746.217	9331.354	**8792.791748**	1.7602	0.3212
Q9WVF8	Tusc2	Tumor suppressor candidate 2	7691.202148	15473.05	8033.507	27754.63	**14738.09497**	7994.405	9030.38		8187.911	**8404.231771**	1.7537	0.3064
Q61738	Itga7	Integrin alpha-7	7431.351074	6662.258	10907.29	5536.968	**7634.46814**	4074.811	4861.956	2784.662	5737.777	**4364.801575**	1.7491	0.0476
Q9WTK5	Nfkb2	Nuclear factor NF-kappa-B p100 subunit	3979.992432		5324.898		**4652.445435**			3269.711	2057.885	**2663.798096**	1.7465	0.1591
Q8BZ00	Slc9a9	Sodium/hydrogen exchanger 9			18545.54		**18545.54297**			10677.1		**10677.10449**	1.7369	
Q9DBN5	Lonp2	Lon protease homolog 2, peroxisomal	3623.308838	2441.532	2329.343	6917.85	**3828.008484**	1752.136	1464.743	3459.818	2139.853	**2204.137299**	1.7367	0.2104
Q8R0G9	Nup133	Nuclear pore complex protein Nup133		8592.811	13135.93		**10864.36816**	8327.674	4438.6		6205.722	**6323.998535**	1.7180	0.1346
Q8BTZ5	Ankrd46	Ankyrin repeat domain-containing protein 46			8388.097		**8388.09668**	4658.571		4944.267	5115.86	**4906.23291**	1.7097	
P02463	Col4a1	Collagen alpha-1(IV) chain	58972.48828	49182.8	100708	43039.79	**62975.76855**	45562.8	45529.48	35830.12	21556.87	**37119.81885**	1.6966	0.1181
Q9D666	Sun1	SUN domain-containing protein 1		25224.22			**25224.2168**	26389.71		3440.299		**14915.00708**	1.6912	
Q6P8I4	Pcnp	PEST proteolytic signal-containing nuclear protein	24860.30469	23817.8	13439.23	25950.14	**22016.87085**	2607.01	22603.13	14342.09	12635.49	**13046.92926**	1.6875	0.1243
Q8N7N5	Dcaf8	DDB1- and CUL4-associated factor 8	8293.310547	8139.01		6007.168	**7479.829753**	4794.06	3543.704	3462.922	5985.129	**4446.453857**	1.6822	0.0231
Q8BG94	Commd7	COMM domain-containing protein 7	7042.184082	13197.6	4744.386	4753.622	**7434.448975**	3459.511	3903.031	4751.414	5591.952	**4426.476929**	1.6795	0.1928

P54103	Dnajc2	DnaJ homolog subfamily C member 2		2890.317	3633.652	4408.148	**3644.038737**	1860.97		1270.024	3385.424	**2172.139648**	1.6776	0.1276
Q9CRA8	Exosc5	Exosome complex component RRP46		3032.449			**3032.449219**				1808.7	**1808.700439**	1.6766	
Q9QX47	Son	Protein SON		27014.24	13150.97	8122.312	**16095.8387**	11248.89	2655.328	14651.35	10011.17	**9641.684326**	1.6694	0.3006
B2RR83	Ythdc2	3'-5' RNA helicase YTHDC2	15737.40625	14740.44	19783.78	18438.01	**17174.90625**	18732.12	1241.422	3919.744	17322.54	**10303.95584**	1.6668	0.1901
Q8K4B0	Mta1	Metastasis-associated protein MTA1	6511.180176	6716.673	8523.288		**7250.380371**	4261.256	3897.722		4892.631	**4350.536377**	1.6665	0.0145
P25799	Nfkb1	Nuclear factor NF-kappa-B p105 subunit	6065.305176		4826.791		**5446.047852**		3461.213		3085.03	**3273.12146**	1.6639	0.0784
Q8BZ36	Rint1	RAD50-interacting protein 1		3461.406	7568.457	5532.166	**5520.676188**	3312.487	2844.641		3825.523	**3327.550456**	1.6591	0.1464
P32507	Nectin2	Nectin-2	5252.013672		1.882493		**2626.948082**				1594.462	**1594.462036**	1.6475	
Q9WTI7	Myo1c	Unconventional myosin-Ic	19337.66016	18398.32	55716.96	22037.25	**28872.5459**	16268.71	17904.14	18941.31	17457.8	**17642.99023**	1.6365	0.2585
Q09143	Slc7a1	High affinity cationic amino acid transporter 1	3207.723633		9543.833	9079.358	**7276.97168**	5078.407	2791.687	5539.447		**4469.846761**	1.6280	0.2727
P0C605	Prkg1	cGMP-dependent protein kinase 1	9868.420898	5192.523	6490.28	5752.793	**6826.004272**	4647.991		5014.116	2922.336	**4194.814046**	1.6272	0.1094
Q9R190	Mta2	Metastasis-associated protein MTA2		3794.005	6193.85		**4993.927368**	2708.225		1565	4942.244	**3071.823161**	1.6257	0.3061
Q8CH77	Nav1	Neuron navigator 1	109064.7188	124586.1	86767.61	102776.9	**105798.8359**	69743.58	121289.5	4530.18		**65187.7526**	1.6230	0.2303
Q6P9J9	Ano6	Anoctamin-6	1683.149414		2284.958	4308.633	**2758.913574**		984.874		2422.049	**1703.461395**	1.6196	0.4288
E9Q557	Dsp	Desmoplakin	10196.97754	9081.383	15148.58	8505.05	**10732.99683**	7532.045	9736.19	3491.369	5804.093	**6640.9245**	1.6162	0.0879
Q9CQ02	Commd4	COMM domain-containing protein 4	4591.528809	2724.281	10214.18	6064.174	**5898.540588**	3265.229	5404.348	3082.081	2857.176	**3652.208557**	1.6151	0.2341
Q9ERT9	Ppp1r1a	Protein phosphatase 1 regulatory subunit 1A		11054.8			**11054.80078**	6588.597	9013.171	4933.797		**6845.188314**	1.6150	
P03975	Iap	IgE-binding protein	4594.778809	4521.004	7309.366	3373.6	**4949.687012**	3242.733	3199.183	2586.888	3235.195	**3065.999573**	1.6144	0.0686
Q63739	Ptp4a1	Protein tyrosine phosphatase type IVA 1	3279.883789	11176.92	2312.456	2839.999	**4902.313782**	3358.413	3327.789	2858.645	2631.771	**3044.154602**	1.6104	0.4121
Q60809	Cnot7	CCR4-NOT transcription complex subunit 7	6405.36377	6139.906	4646.114	10305.09	**6874.119019**	5379.407	3671.928	2684.433	5487.333	**4305.775208**	1.5965	0.1134
P21614	Gc	Vitamin D-binding protein	5796.100586	12661.74	15876.72	5296.253	**9907.7052**	7429.906	5373.997	7293.49	4756.94	**6213.583252**	1.5945	0.2188
Q9QXC1	Fetub	Fetuin-B		74082.41	128363.7	100036.8	**100827.6406**				63241.8	**63241.79688**	1.5943	
Q8VE91	Retreg1	Reticulophagy regulator 1	10632.97656		9058.198		**9845.587402**			2885.123	9476.568	**6180.845459**	1.5929	0.3925
Q8VE80	Thoc3	THO complex subunit 3		10323.81	15720.32	8337.182	**11460.4401**	6815.909	6341.261	5469.299	10164.45	**7197.728882**	1.5922	0.1117
Q9WUP4	Srd5a3	Polyprenol reductase	2523.338135	3526.656	1344.543		**2464.845947**	2766.676		622.782	1270.259	**1553.239014**	1.5869	0.3659
Q8BKK4	Ppp1r1c	Protein phosphatase 1 regulatory subunit 1C	5788.10791	4881.289	4810.289	5195.304	**5168.747437**	5188.396	4015.961		574.0085	**3259.45516**	1.5858	0.1690
Q61001	Lama5	Laminin subunit alpha-5	13169.45215	12667.51	33743.27	20075.7	**19913.98218**	13125.53	12090.97	10875.71	14166.01	**12564.55273**	1.5849	0.1889
Q9CYV5	Tmem135	Transmembrane protein 135	11487.08691	18198.01			**14842.54736**	9638.382		9103.598		**9370.989746**	1.5839	0.2456
O35900	Lsm2	U6 snRNA-associated Sm-like protein LSm2	9699.830078	8142.277	6134.198	7576.736	**7888.260254**	4941.287	6560.589	3852.211	4608.625	**4990.678162**	1.5806	0.0209
Q8BXA5	Clptm1l	Lipid scramblase CLPTM1L	10565.89941	15591.14	15680.75	15728.78	**14391.6416**	8737.924	11503.38	7958.508	8244.219	**9111.007935**	1.5796	0.0130
Q8CBH5	Mfsd6	Major facilitator superfamily domain-containing protein 6	16912.58984	17896.15	12922.27	19071.14	**16700.53784**	8712.31	12209.26	7863.688	13626.21	**10602.86719**	1.5751	0.0191
Q7M759	Abhd17b	Alpha/beta hydrolase domain-containing protein 17B	26174.80078	35042.31	35536.17	31096.08	**31962.33984**	21618.23	21005.12	17338.34	21219.79	**20295.37012**	1.5749	0.0027
Q62219	Tgfb1i1	Transforming growth factor beta-1-induced transcript 1 protein	4388.277344		7723.066		**6055.671875**	4268.977	2690.957	3718.886	4725.663	**3851.120667**	1.5724	0.1436
Q8BWW9	Pkn2	Serine/threonine-protein kinase N2		3416.003	5503.001		**4459.502197**		2836.077			**2836.07666**	1.5724	
Q8VCS3	Fam20b	Glycosaminoglycan xylosylkinase		10682.91			**10682.91406**				6795.691	**6795.691406**	1.5720	
Q8CIV2	Tmem259	Membralin		4845.553			**4845.552734**			3083.336		**3083.335693**	1.5715	
Q91YR7	Prpf6	Pre-mRNA-processing factor 6	24072.45117	11315.54	12536.65	14030.27	**15488.72827**	9915.876	8062.99	11882.7	9637.238	**9874.70105**	1.5685	0.1122
Q05793	Hspg2	Basement membrane-specific heparan sulfate proteoglycan core protein	47602.37891	46797.2	159120.6	52427.57	**76486.94629**	42100.71	46445.6	50847.65	55838.27	**48808.05762**	1.5671	0.3567
Q6DFV3	Arhgap21	Rho GTPase-activating protein 21	40406.82422	14117.57	36827.3	34486.49	**31459.54883**	27984.45	13512.17	29347.82	9458.363	**20075.69922**	1.5670	0.1929
Q6ZPR5	Smpd4	Sphingomyelin phosphodiesterase 4	9779.436523			8504.707	**9142.071777**		5098.199	6461.482	5981.299	**5846.99349**	1.5636	0.0182
Q8CE08	Acp3	Prostatic acid phosphatase	5080.56543	6103.537	2833.845	10189.55	**6051.874817**	5179.47	2291.429	2796.465	5283.979	**3887.835876**	1.5566	0.2567
Q922U1	Prpf3	U4/U6 small nuclear ribonucleoprotein Prp3	13448.27344	6662.777	6979.39	620.7813	**6927.805313**	4705.794			4200.647	**4453.220703**	1.5557	0.5634
O70309	Itgb5	Integrin beta-5			6124.98	3584.53	**4854.755371**	1986.545		4257.293		**3121.918823**	1.5551	0.4161
P40240	Cd9	CD9 antigen	52386.07031	54556.98	240076.1	4546.489	**87891.41626**	52405.73	39339.16	74441.13	60281.78	**56616.9502**	1.5524	0.5734
B9EHT4	Clip3	CAP-Gly domain-containing linker protein 3	6842.59375	15667.84	15109.96	6531.696	**11038.02209**	3730.653	7122.49	6155.069	11704.05	**7178.064758**	1.5377	0.2482
O35071	Kif1c	Kinesin-like protein KIF1C	42055.86719	27659.38	34885.96	21818.03	**31604.80908**	26678.94	17155.98	18779.87	19628.67	**20560.86475**	1.5371	0.0638
Q8BGR8	Gskip	GSK3B-interacting protein	4429.281738	4788.267	5436.713	5672.919	**5081.795288**	3367.849	2177.722	4133.474	3552.689	**3307.933472**	1.5362	0.0122
P39098	Man1a2	Mannosyl-oligosaccharide 1,2-alpha-mannosidase IB	27305.69336	9282.821			**18294.25732**	15262.1		8110.474	12358.26	**11910.2793**	1.5360	0.4424
Q9QY36	Naa10	N-alpha-acetyltransferase 10	27306.48438	14960.58	13476.61	12263.14	**17001.70264**		9559.073	7409.047	16282.07	**11083.39616**	1.5340	0.2621
Q62000	Ogn	Mimecan	550562.875	541862.8	59897.66	417440.1	**392440.8379**	573298	125514.1	132183.8	194915	**256477.6953**	1.5301	0.4194
O08808	Diaph1	Protein diaphanous homolog 1			4695.132	6437.317	**5566.224854**	1149.848			6132.105	**3640.976624**	1.5288	0.5416
Q8BG92	Clvs2	Clavesin-2	15864.23535	21670.93		23648.51	**20394.55827**	14159.94	13512.36	11521.77	14183.24	**13344.33057**	1.5283	0.0198
Q8K157	Galm	Galactose mutarotase	10440.28516	14725.22	19872.77	38349.48	**20846.93799**	7108.544	14322.19	14523.66	18668.39	**13655.69495**	1.5266	0.3175
Q62165	Dag1	Dystroglycan 1	23190.83984	26135.21	92194.27	25008.24	**41632.1416**	27782.39	25250.71	27496.25	28811.63	**27335.24463**	1.5230	0.4295
Q9Z223	Mocs2	Molybdopterin synthase catalytic subunit			32064.64		**32064.63867**	24437.1			17860.92	**21149.00977**	1.5161	
Q61292	Lamb2	Laminin subunit beta-2	33847.69141	39653.96	136063.3	46983.42	**64137.08887**	39260.65	40092.2	46571.95	43565.38	**42372.54395**	1.5136	0.4028
O89017	Lgmn	Legumain	14608.64941	8191.51	18643.16	19702.07	**15286.34753**	9350.544	10035.98	7609.551	13427.6	**10105.9165**	1.5126	0.1220
P13634	Ca1	Carbonic anhydrase 1	10553.59961	11549.55	25340.72	14084.14	**15382.00098**	10436.03	9388.429	8727.19	12142.33	**10173.49487**	1.5120	0.1854
Q9DAI2	Ift22	Intraflagellar transport protein 22 homolog	10322.99902	10716.69	10060.72	11015.86	**10529.06812**	4338.026	6484.056	6990.709	10136.12	**6987.228027**	1.5069	0.0268
Q62073	Map3k7	Mitogen-activated protein kinase kinase kinase 7	10418.69336	5572.433	8563.576	10920.17	**8868.718262**	6444.46			5332.367	**5888.413818**	1.5061	0.1819
A2RSJ4	Bltp3b	Bridge-like lipid transfer protein family member 3B	3027.166016	3182.488	9638.391		**5282.681641**	5494.354	2773.808	2605.197	3211.525	**3521.220825**	1.5002	0.4160
P07356	Anxa2	Annexin A2	157430.375	155026.7	369444.2	208806	**222676.8047**	145554.3	150986	144467.2	153641.3	**148662.1914**	1.4979	0.1932
Q6P5F7	Ttyh3	Protein tweety homolog 3	15368.05371	13306.24	15977.97	14088.43	**14685.1731**	3391.057	20914.91	10637.05	4349.362	**9823.095093**	1.4950	0.2780
P83870	Phf5a	PHD finger-like domain-containing protein 5A	13195.36914	3215.6	17895.85	18058.84	**13091.41461**	11077.25	9858.557	9847.294	4365.487	**8787.145996**	1.4898	0.2994
P08122	Col4a2	Collagen alpha-2(IV) chain	75822.79688	74751.97	282345	70864.11	**125945.9688**	72085.02	79788.14	90021.3	96541.84	**84609.07617**	1.4886	0.4604

P97372	Psme2	Proteasome activator complex subunit 2	6071.290527	3619.296	6410.247	5950.437	5512.817749	4850.659	5680.726	1463.209	2830.342	3706.233795	1.4874	0.1676
Q0GA42	Cnnm1	Metal transporter CNNM1	11091.00684	5702.203	7733.907	7485.234	8003.087646	4771.507	6368.005	5112.557	5313.917	5391.496338	1.4844	0.0681
O08573	Lgals9	Galectin-9	13090.94141	11515.77	9861.05	16318.81	12696.6416	9166.77	11708.98	5396.187	7976.99	8562.231567	1.4829	0.0725
P63254	Crip1	Cysteine-rich protein 1		5580.016	16670.91	12013.87	11421.60075	10931.84	9391.991	5797.549	4756.001	7719.345093	1.4796	0.2992
Q91V57	Chn1	N-chimaerin	30021.58789	25995.82	18356.94	62773.72	34287.0166	17269.57	31308.44	22717.32	21413.06	23177.09912	1.4793	0.3193
Q99JN2	Klhl22	Kelch-like protein 22	8525.625977	7406.235	7882.471	8242.898	8014.307739		6566.882		4305.414	5436.147705	1.4743	0.0300
Q8BVW0	Ganc	Neutral alpha-glucosidase C		2660.146			2660.145752	1851.97		1758.541		1805.255737	1.4736	
Q8C8U0	Ppfibp1	Liprin-beta-1	7033.820313	9441.321	8305.494	15151.89	9983.131592	6888.509	7281.936	6105.697	6836.15	6778.07312	1.4729	0.1267
Q9DBG9	Tax1bp3	Tax1-binding protein 3	12371.42285	10039.52	29092.78	15145.33	16662.26465	9494.898	11212.29	11902.08	12705.79	11328.76318	1.4708	0.2638
P14069	S100a6	Protein S100-A6	28715.48047	28552.16	51946.46	29276.48	34622.64746	29612.45	19500.76	18883.17	26230.07	23556.61182	1.4698	0.1316
Q8K2P7	Slc38a1	Sodium-coupled neutral amino acid symporter 1	8296.926758	5914.331	5844.225	9932.872	7497.088745	5351.771		4859.83		5105.800293	1.4683	0.1851
Q5SWT3	Slc25a35	Solute carrier family 25 member 35			3363.483	13953.97	8658.726685	2498.834	9296.185			5897.509399	1.4682	0.7036
Q3TVA9	Ccdc136	Coiled-coil domain-containing protein 136	17883.375	13724.31	49357.72	8329.042	22323.61084	13860.77	14308.29	14264.13	18690.16	15280.83716	1.4609	0.4772
P97770	Thumpd3	tRNA (guanine(6)-N2)-methyltransferase THUMP3	5585.432617	9453.538	12230.38	5929.752	8299.775269	9659.6	8275.297	2289.37	2519.489	5685.939026	1.4597	0.3325
P08121	Col3a1	Collagen alpha-1(III) chain				37814.26	37814.25781		26044.99			26044.98633	1.4519	
Q9WU84	Ccs	Copper chaperone for superoxide dismutase	13150.14258	10467.69	15659.33	18881.95	14539.7793	7502.162	9135.243	13455.25		10030.88639	1.4495	0.1423
Q3UM29	Cog7	Conserved oligomeric Golgi complex subunit 7		1419.923		5164.082	3292.00238	2276.368				2276.368164	1.4462	
P15331	Prph	Peripherin	370800.8125	361802	895490.2	410892.5	509746.3828	324572	361940.5	367572.5	357541.8	352906.6953	1.4444	0.2710
P21981	Tgm2	Protein-glutamine gamma-glutamyltransferase 2	25841.46289	29200.8	71119.09	34252.84	40103.54785	29216.65	29856.45	24600.49	27456.28	27782.46875	1.4435	0.2871
Q9ES34	Ube3b	Ubiquitin-protein ligase E3B				7274.205	7274.205078			5046.319		5046.319336	1.4415	
Q99L88	Sntb1	Beta-1-syntrophin	11547.50586	17300.04	35638.17	15351.62	19959.33301	12712.15	14647.76	12760.62	15279.87	13850.10132	1.4411	0.3012
Q7M729	Scn4b	Sodium channel subunit beta-4	203259.2969	209221.3	183841.6	149938.6	186565.1953	92598.99	108717.7	177343.8	140776.7	129859.3027	1.4367	0.0488
Q9DC11	Plxdc2	Plexin domain-containing protein 2	8348.461914	5991.71	18706.79	9260.372	10576.83386	7427.002	6067.059	7323.416	8634.675	7363.037842	1.4365	0.3017
Q68EF0	Rab3ip	Rab-3A-interacting protein	1679.387573	1961.384	4191.501	5391.513	3305.946503		1708.308		2901.493	2304.900391	1.4343	0.5112
Q9Z0S9	Rabac1	Prenylated Rab acceptor protein 1	25222.375	17553.54	20874.59	17491.16	20285.41553	23842.91	2167.702	14277.41	16346.57	14158.64703	1.4327	0.2534
P00329	Adh1	Alcohol dehydrogenase 1	7862.208984		13888.81		10875.50977	14196.34		997.2568		7596.796356	1.4316	0.6956
A6H6E9	Ttc23l	Tetratricopeptide repeat protein 23-like	31195.65234	70869.51	71139.51	39092.13	53074.19922	44788.61	35830.74	33098.69	34761.43	37119.86621	1.4298	0.1900
O54724	Cavin1	Caveolae-associated protein 1	16921.05859	19057.79	51256.64	19974.59	26802.51904	19640.66	15209.72	22423.8	17784.46	18764.66016	1.4284	0.3711
Q00519	Xdh	Xanthine dehydrogenase/oxidase	4451.200195	4013.633	4247.989	1441.423	3538.561188		1879.526	3017.312	2536.925	2477.92098	1.4280	0.2812
Q9JIK5	Ddx21	Nucleolar RNA helicase 2	8037.330566	13837.41	14987.23	14686.65	12887.15662	7082.552	8266.652	9581.001	11234	9041.051758	1.4254	0.0844
Q4FZC9	Syne3	Nesprin-3		3573.586			3573.586182	2508.798				2508.798096	1.4244	
P98083	Shc1	SHC-transforming protein 1		224.385			224.3849945		197.1115	118.3412		157.7263451	1.4226	
P01867	Ighg2b	Immunoglobulin heavy constant gamma 2B	70381.48438	50126.29	69244.85	46251.95	59001.14355	36092.54	52498.18	40327.59	37060.9	41494.80566	1.4219	0.0545
Q78YZ6	Scoc	Short coiled-coil protein	16452.18555	41625.92	27455.88	50249.24	33945.80518	13848.87	19933.93	41408.67	20313.35	23876.20581	1.4217	0.3353
Q9ESP1	Sdf2l1	Stromal cell-derived factor 2-like protein 1	9514.310547	9208.627	8584.252	12970.2	10069.34766	10439.48	6460.896	5553.451	5910.187	7091.00293	1.4200	0.0945
Q6NZK8	Ptpdc1	Protein tyrosine phosphatase domain-containing protein 1	5233.17627	7185.193	7842.203		6753.523926	3086.04	6629.471	4958.979	4387.139	4765.40686	1.4172	0.1274
Q8R395	Commd5	COMM domain-containing protein 5	26698.91406	12635.53	9183.604	13377.74	15473.94727	9740.38	14190	10234.9	9519	10921.07007	1.4169	0.2990
O35655	Ppef1	Serine/threonine-protein phosphatase with EF-hands 1	5754.289063	4690.123	7419.044	5731.587	5898.76062	4328.747	2704.18	4464.055	5204.522	4175.37616	1.4127	0.0670
P19324	Serpinh1	Serpin H1	6264.427246	5892.352	10893.08	6527.084	7394.236328	6294.905	5701.555	4454.635	4548.543	5249.909546	1.4085	0.1388
P97813	Pld2	Phospholipase D2	7151.841309	9923.273	6289.647	5104.275	7117.259399	3077.131	5577.969	5247.703	6311.176	5053.494812	1.4084	0.1467
P50543	S100a11	Protein S100-A11	12176.23438	7026.143	23702.87	7580.056	12621.32471	7570.643	8798.432	6796.833	12706.22	8968.032227	1.4074	0.4058
O55128	Sap18	Histone deacetylase complex subunit SAP18	16215.08496	18766.72	13657.14	26617.06	18814.00342	14636.52	13416.69	9054.53	16376.19	13370.98364	1.4071	0.1407
P13808	Slc4a2	Anion exchange protein 2	5844.742676		8802.934	8735.403	7794.359863	4045.264	5068.347	4682.183	8368.707	5541.125305	1.4066	0.1694
Q8BHI4	Kbtbd3	Kelch repeat and BTB domain-containing protein 3	4461.638672	4910.102			4685.870117	2525.478		4137.861		3331.669434	1.4065	0.2470
Q3UBG2	Pid1	PTB-containing, cubilin and LRP1-interacting protein		3687.116			3687.115723	3799.124		1448.68		2623.901733	1.4052	
P97429	Anxa4	Annexin A4	11619.00391	10743.44	31133.61	16297.11	17448.28931	10121.23	12390.78	13713.76	13508.18	12433.48682	1.4033	0.3358
P46662	Nf2	Merlin	9928.94043	863.4063	10267.45	7112.015	7042.953613	2010.986	9530.822	5647.521	2893.997	5020.831543	1.4027	0.4910
P63089	Ptn	Pleiotrophin	1424.359619				1424.359619	23.0166	720.293	1935.377	1390.931	1017.404449	1.4000	
Q8BTF8	Ralyl	RNA-binding Raly-like protein	3511.269287	5424.692	12038.69	14001.58	8744.058533	5813.659	4165.367	8758.503		6245.843099	1.4000	0.4702
Q923B6	Steap4	Metalloreductase STEAP4	419317.25	418367.6	520303.7	594716.3	488176.1875	390591	501474.5	376136.3	126720	348730.4473	1.3999	0.1721
Q8R3D1	Tbc1d13	TBC1 domain family member 13	13688.20703	15856.33	16568.78	12814.22	14731.88525	10760.49	12272.3	9742.028	9367.38	10535.54834	1.3983	0.0087
Q8BJA3	Hmbox1	Homeobox-containing protein 1	10287.44434				10287.44434		7363.31			7363.310059	1.3971	
Q3TC46	Patl1	Protein PAT1 homolog 1			3537.298	4327.685	3932.491333		2360.612	3269.733		2815.172729	1.3969	0.2048
Q8VGR9	Or8u9	Olfactory receptor 8U9			20652.43	23173.21	21912.82227	12566.1	11598.35	22981.54		15715.33333	1.3944	0.2869
Q8VCI5	Pex19	Peroxisomal biogenesis factor 19	7148.355469	7582.261	7328.322	7416.195	7368.783325	5273.406	5658.647	4520.04	5700.568	5288.165405	1.3934	0.0004
Q9Z2Q5	Mrpl40	Large ribosomal subunit protein mL40	22352.22852	20355.98	23369.67	31351.2	24357.26953	24870.65	24733.53	796.4752	19613.09	17503.43422	1.3916	0.3107
Q91WM1	Strbp	Spermatid perinuclear RNA-binding protein	27274.85742	22845.47	27726.33	28325.16	26542.95459	21007.21	20071.83	21331.87	13986.07	19099.24487	1.3897	0.0129
Q9WV91	Ptgfrn	Prostaglandin F2 receptor negative regulator			2834.298		2834.298096	3500.925		1363.98	1258.248	2041.050781	1.3886	
Q8VEE4	Rpa1	Replication protein A 70 kDa DNA-binding subunit	61277.94922	14730.87			38004.40723			24260.95	30480.97	27370.95996	1.3885	0.6950
Q3V3N7	Bbs1	Bardet-Biedl syndrome 1 protein homolog	8207.980469	7196.042	8874.982	9808.238	8521.810913	6079.496	5834.962	7967.562	4671.5	6138.380005	1.3883	0.0347
Q00915	Rbp1	Retinol-binding protein 1		7045.522	6566.565		6806.043945			4906.898		4906.898438	1.3870	
Q00898	Serpina1e	Alpha-1-antitrypsin 1-5	1696.630127		32756.71	25897.79	20117.04077	4012.239	2842.684	23636.41	27593.02	14521.08826	1.3854	0.6320
Q6EDY6	Carmil1	F-actin-uncapping protein LRRC16A	18010.66992	13636.93	14672.94	16010.7	15582.80859	10402.88	13038.46	8870.782	12707.21	11254.83398	1.3845	0.0194
Q8BSY0	Asph	Aspartyl/asparaginyl beta-hydroxylase	12119.33984	11164.8	20049.93	21442.79	16194.21729	11396.73	12234.94	11999.98	11175.62	11701.81689	1.3839	0.1425
Q70IV5	Synm	Synemin	25011.88477	26152.3	49573.02	23980.37	31179.39453	22126.96	24194.76	20797.08	23033	22537.94873	1.3834	0.2121

Q99N92	Mrpl27	Large ribosomal subunit protein bL27m	9827.375977	12105.72	9346.192	665.9657	**7986.312714**	9556.737	5345.526	3763.154	4455.366	**5780.195801**	**1.3817**	**0.4652**
Q8R2R3	Aagab	Alpha- and gamma-adaptin-binding protein p34	3125.422363	2738.495	6082.347	2665.37	**3652.908508**	2735.317	1262.314	4204.963	2375.762	**2644.589294**	**1.3813**	**0.3599**
Q8C0L6	Paox	Peroxisomal N(1)-acetyl-spermine/spermidine oxidase	14328.74609	6781.097	1500.01	17283.07	**9973.230042**	2622.369	3860.144	5455.548	16974.51	**7228.141724**	**1.3798**	**0.5937**
O88322	Nid2	Nidogen-2	42103.19141	39276.22	114098.8	45866.63	**60336.21973**	41907.55	48004.45	42796.07	42216.62	**43731.17285**	**1.3797**	**0.3926**
Q922Q9	Chid1	Chitinase domain-containing protein 1	14332.16016	7802.92	18879.71	29288.48	**17575.81885**	10172	16332.66	14976.29	9650.633	**12782.89722**	**1.3749**	**0.3586**
Q9CZL5	Pcbd2	Pterin-4-alpha-carbinolamine dehydratase 2	23825.08398	24390.64	26154.49	23699.5	**24517.43115**	23823.73	25795.08	18627.07	3080.625	**17831.62592**	**1.3749**	**0.2439**
P58137	Acot8	Acyl-coenzyme A thioesterase 8	9935.467773	8748.864	9739.025		**9474.452474**		3487.478	9602.872	7604.604	**6898.318034**	**1.3734**	**0.2335**
Q8VD26	Tmem143	Transmembrane protein 143	13905.44043	10836.93	15197.55	12523.48	**13115.84863**	4223.091	13586.26	15650.77	4755.742	**9553.965332**	**1.3728**	**0.2944**
Q8K019	Bclaf1	Bcl-2-associated transcription factor 1			11252.48	14684.15	**12968.31543**	3733.916		8850.809	15761.45	**9448.725586**	**1.3725**	**0.5064**
Q7TNG8	Ldhd	Probable D-lactate dehydrogenase, mitochondrial		1662.223	2700.919	3999.939	**2787.693888**	2002.499	2062.883			**2032.690552**	**1.3714**	**0.4509**
P59913	Pcmtd1	Protein-L-isoaspartate O-methyltransferase domain-containing protein 1			14144.84		**14144.8418**	10326.67				**10326.66895**	**1.3697**	
E9Q236	Abcc4	ATP-binding cassette sub-family C member 4	12715.66211	10723.55	15369.86	11127.21	**12484.06982**	9360.192	11148.01	8427.315	7597.192	**9133.177856**	**1.3669**	**0.0419**
Q64522	H2ac21	Histone H2A type 2-B	237739.9688	252746.9	246858	317986.1	**263832.75**	162823.8	287227.6	159536	162925.7	**193128.2617**	**1.3661**	**0.0996**
Q7TSV4	Pgm2	Phosphopentomutase	22405.69141	22035.15	18054.72	19753.06	**20562.15723**	18769.44	12425.13	14987.44	14056.57	**15059.64307**	**1.3654**	**0.0173**
Q9DAF3	Ddi1	Protein DDI1 homolog 1		12329.54	10872.11	12065.89	**11755.84603**	8054.594	7101.001	7724.938	11564.08	**8611.154419**	**1.3652**	**0.0529**
P17095	Hmga1	High mobility group protein HMG-I/HMG-Y	15746.62207	16891.22	16438.48	43381.14	**23114.36694**	14694.87	16122.18	20014.98		**16944.01042**	**1.3642**	**0.4810**
Q9DBX6	Cyp2s1	Cytochrome P450 2S1	3917.08252	3662.494	7576.217	14088.21	**7311.001404**	5095.162	3295.166	6558.721	6520.498	**5367.386841**	**1.3621**	**0.4746**
Q9R0Q3	Tmed2	Transmembrane emp24 domain-containing protein 2	9092.351563	11029.7	163.1407	1951.345	**5559.135109**	3158.082	4123.979	4101.68	4945.051	**4082.197876**	**1.3618**	**0.6014**
Q9JIZ9	Plscr3	Phospholipid scramblase 3	10267.88379	14174.6	15484.56	12580.59	**13126.90942**	8827.335	6831.196	16968.42	5948.826	**9643.944214**	**1.3612**	**0.2529**
P62307	Snrpf	Small nuclear ribonucleoprotein F		33641.98	24614.45	5324.707	**21193.71094**	13521.65	19238.57	4986.661	24543.51	**15572.59802**	**1.3610**	**0.5406**
Q7TN33	Celf6	CUGBP Elav-like family member 6	105194.9297	97175.91	104831	114117	**105329.6953**	102639	102305.7	21940.99	83379.45	**77566.2666**	**1.3579**	**0.2022**
Q9R0M8	Slc35a2	UDP-galactose translocator	11283.92578	11310		6363.677	**9652.53597**	8739.448	9473.75	4732.957	5487.786	**7108.485229**	**1.3579**	**0.2498**
Q80U72	Scrib	Protein scribble homolog	5930.755859	4849.815	6916.028	8226.382	**6480.745239**	5685.059	5283.446	4846.543	3288.389	**4775.859314**	**1.3570**	**0.1038**
Q9Z2P8	Vamp5	Vesicle-associated membrane protein 5	15808.32715	16026.56	27640.23	22683.66	**20539.69482**	16148.55	15301.19	15638.94	13484.79	**15143.36816**	**1.3563**	**0.1134**
Q5DTN8	Jakmip3	Janus kinase and microtubule-interacting protein 3			14267.83		**14267.82813**	9400.031		12334.31	9848.836	**10527.72461**	**1.3553**	
Q920R0	Als2	Alsin	3843.027832	3844.901		4786.766	**4158.231445**	2866.685	2797.736	2903.328	3716.931	**3071.1698**	**1.3540**	**0.0314**
Q8CF66	Lamtor4	Ragulator complex protein LAMTOR4	20968.29688	5702.114	4565.047	18642.07	**12469.38196**	5860.428		17196.41	4572.082	**9209.641276**	**1.3539**	**0.6139**
A2APT9	Klhdc7a	Kelch domain-containing protein 7A	5977.986816	6000.779			**5989.383057**	5869.701	3021.706		4382.058	**4424.488118**	**1.3537**	**0.2370**
Q704Y3	Trpv1	Transient receptor potential cation channel subfamily V member 1	10805.56543	10583.81	12188.09	12205.03	**11445.62256**	8297.5	8850.673	8337.045	8345.988	**8457.801514**	**1.3533**	**0.0006**
Q6P5D3	Dhx57	Putative ATP-dependent RNA helicase DHX57	8739.337891	6447.608	10157.45		**8448.13151**	7966.595	5478.005	5296.54		**6247.046712**	**1.3523**	**0.1865**
Q8BJF9	Chmp2b	Charged multivesicular body protein 2b	15439.43555	12662.01	16226.46	11416.41	**13936.07935**	9155.559	10001.67	9948.863	12132.99	**10309.77124**	**1.3517**	**0.0318**
Q8C6K9	Col6a6	Collagen alpha-6(VI) chain			9835.174	15480.85	**12658.01025**	9367.385				**9367.384766**	**1.3513**	
O54692	Zw10	Centromere/kinetochore protein zw10 homolog	15924.14746	11122.39	17944.31	16389.21	**15345.01392**	10763.03	11561.67	15541.09	7562.07	**11356.96729**	**1.3512**	**0.1204**
Q80X90	Flnb	Filamin-B	20321.81836	20782.64	52192.53	23817.38	**29278.59229**	20935.15	22504.99	22031.68	21417.77	**21722.39893**	**1.3479**	**0.3635**
O35945	Aldh1a7	Aldehyde dehydrogenase, cytosolic 1	77539.71094	80295.09	74706.75	57454.54	**72499.02344**	65865.16	33617.71	57240.1	58976.79	**53924.93848**	**1.3444**	**0.0768**
Q8JZV9	Bdh2	Dehydrogenase/reductase SDR family member 6	13223.05957	16608.24	11615.14		**13815.48275**	12088.74	8910.032	12490.37	7623.71	**10278.2146**	**1.3442**	**0.1176**
Q9CZ69	Cmtm6	CKLF-like MARVEL transmembrane domain-containing protein 6	28146.45508	25622.71	48664.3	30383.6	**33204.26465**	23508.74	29014.02	23564.47	22814.82	**24725.51221**	**1.3429**	**0.1700**
Q8R555	Crtac1	Cartilage acidic protein 1	56519.80078	61065.53	63109.69	56277.96	**59243.24414**	57825.05	51995.89	32902.64	34026.52	**44187.52637**	**1.3407**	**0.0607**
Q5SNZ0	Ccdc88a	Girdin			5323.843	3954.039	**4638.94104**		4485.976	2730.781	3172.635	**3463.130697**	**1.3395**	**0.2608**
P19258	Mpv17	Protein Mpv17	21530.46094	18609.12	29867.66	30055.8	**25015.76074**	16592.58	15329.51	19179.72	23781.37	**18720.79517**	**1.3363**	**0.1191**
Q9DBS9	Osbpl3	Oxysterol-binding protein-related protein 3	11154.31934				**11154.31934**	11078.34	5637.804			**8358.07251**	**1.3346**	
Q9D8L5	Ccdc91	Coiled-coil domain-containing protein 91	16669.35352	11213.6	21733.77	3150.981	**13191.92615**	8679.902	9270.76	10594.61	10999.3	**9886.142578**	**1.3344**	**0.4417**
Q9Z172	Sumo3	Small ubiquitin-related modifier 3	137018.1563	148014.9	144615.2	152320.4	**145492.1836**	7846.447	146636.1	140610.1	141056.5	**109037.2876**	**1.3343**	**0.3237**
Q6GQW0	Abtb3	Ankyrin repeat and BTB/POZ domain-containing protein 3	8530.768555	13320.44	13203.8	10655.19	**11427.54907**	8752.202	8693.997	7708.151	9141.789	**8574.03479**	**1.3328**	**0.0527**
Q9DCM7	Nacc2	Nucleus accumbens-associated protein 2	12228.62402	12431.27	22275.04	21038.34	**16993.31982**	11239.91	16635.87	14726.74	8477.629	**12770.0376**	**1.3307**	**0.2424**
P16045	Lgals1	Galectin-1	141537.0156	151287.8	283068.9	177009.3	**188225.7539**	130640.2	137420.2	136143.5	162656.6	**141715.125**	**1.3282**	**0.2115**
Q64471	Gstt1	Glutathione S-transferase theta-1	72400.41406	70591.98	89079.13	77247.73	**77329.8125**	64654.97	68265.74	26080.1	74009.72	**58252.63281**	**1.3275**	**0.1530**
Q6GQT1	A2m	Alpha-2-macroglobulin-P	49410.69531	43229.93	36483.63	49460.74	**44646.24707**	38439.64	45535.04	20747.06	29810.71	**33633.11279**	**1.3274**	**0.1256**
Q8BYJ6	Tbc1d4	TBC1 domain family member 4	9492.913086	9373.973	13286.04	15080.08	**11808.25366**	9708.654	8541.506	8766.728	8585.74	**8900.656982**	**1.3267**	**0.0910**
Q5XJV6	Lmtk3	Serine/threonine-protein kinase LMTK3	3847.718506			4495.165	**4171.441772**		2622.95	3670.012		**3146.481323**	**1.3257**	**0.2378**
Q3UQN2	Fcho2	F-BAR domain only protein 2	2231.683105	2540.473	3594.564	5487.257	**3463.494263**		2741.892	2487.659		**2614.775391**	**1.3246**	**0.4853**
Q5SX39	Myh4	Myosin-4	60909	64418.28	69323.77	34743.11	**57348.53711**	19258.36	55294	69356.38	29474.8	**43345.88623**	**1.3230**	**0.3517**
P84099	Rpl19	Large ribosomal subunit protein eL19	76416.20313	57073.13	59606.07	84283.45	**69344.71191**	46359.41	65790.48	35001.54	62544.03	**52423.86523**	**1.3228**	**0.1332**
Q8BSL7	Arf2	ADP-ribosylation factor 2	5574.638184	24512.46			**15043.54761**	20195.08	18175.35	3689.593	3460.292	**11380.0791**	**1.3219**	**0.7026**
Q501J2	Antkmt	Adenine nucleotide translocase lysine N-methyltransferase	8669.28418	8178.693	9779.053	9429.24	**9014.067627**	6396.493		8097.952	5964.075	**6819.50651**	**1.3218**	**0.0249**
Q9D1H7	Get4	Golgi to ER traffic protein 4 homolog	25454.4707	25324.43	27946.63	24669.23	**25848.68945**	17948.89	27356.78	17099.58	15873.47	**19569.67896**	**1.3209**	**0.0609**
P0C0S6;Q3THW5	H2az1;H2az2	Histone H2A.Z;Histone H2A.V	336882.4063	371870.8	411712.9	387501.5	**376991.9141**	359462	344594.1	93599.97	344625.9	**285570.4844**	**1.3201**	**0.2152**
O70566	Diaph2	Protein diaphanous homolog 2			14532.05	11267.71	**12899.88232**	11192.67	8419.4	7279.242	12220.11	**9777.85376**	**1.3193**	**0.1940**
Q9Z2V5	Hdac6	Histone deacetylase 6			12820.59	18272.28	**15546.43701**			11790.36		**11790.36426**	**1.3186**	
Q9JJG0	Tacc2	Transforming acidic coiled-coil-containing protein 2			2991.688		**2991.6875**	2526.194			2012.021	**2269.107117**	**1.3184**	
P15920	Atp6v0a2	V-type proton ATPase 116 kDa subunit a 2	1733.252197	3800.182	1806.732		**2446.722046**	783.0139	2090.087		2698.391	**1857.164124**	**1.3175**	**0.5404**
Q80W54	Zmpste24	CAAX prenyl protease 1 homolog	29154.17969	30033.13	24851.81	32942.37	**29245.37207**	28737.01	15738.79	24527.58	19811.26	**22203.66064**	**1.3171**	**0.0755**
Q61147	Cp	Ceruloplasmin	12719.52344	12338.38	39417.64	8014.416	**18122.48901**	11016.06	11313.67	18751.66	13959.13	**13760.13013**	**1.3170**	**0.5769**
Q8VDG7	Pafah2	Platelet-activating factor acetylhydrolase 2, cytoplasmic			2862.903		**2862.903076**	2905.088		1750.332	1869.059	**2174.826131**	**1.3164**	
Q8VC31	Ccdc9	Coiled-coil domain-containing protein 9		5913.946	8484.143	7161.413	**7186.500488**		7144.677	6061.156	3172.958	**5459.596842**	**1.3163**	**0.2845**

Q9D486	Cmip	C-Maf-inducing protein		7330.899	11350.28	7800.678	**8827.284505**	8752.73		6560.246	4825.575	**6712.850423**	1.3150	0.2823
P14106	C1qb	Complement C1q subcomponent subunit B	28416.69531	16914.12	10022.73	19779.93	**18783.36865**	18158.12	14429.77	10241.05	14317.03	**14286.4939**	1.3148	0.3188
Q7TSH6	Scaf4	SR-related and CTD-associated factor 4	2334.955078	2072.359			**2203.657104**		1676.506			**1676.505615**	1.3144	
Q80TS3	Adgrl3	Adhesion G protein-coupled receptor L3	18986.71094	9811.704	8963.806	12318.86	**12520.271**	8477.97	9232.941	10644.53	9752.646	**9527.022949**	1.3142	0.2436
P70377	Fgf13	Fibroblast growth factor 13	5845.480469	6407.841	7680.285	6385.938	**6579.886353**			5401.065	4619.67	**5010.367676**	1.3133	0.0676
Q9D0J8	Ptms	Parathymosin	106615.5625	50747.27	153101.3	163407.3	**118467.8652**	48889.54	54390.29	118577	139045.1	**90225.47852**	1.3130	0.4419
Q8BTZ4	Anapc5	Anaphase-promoting complex subunit 5	4663.711426		15651.89	6579.847	**8965.148112**		4181.686		9479.513	**6830.599365**	1.3125	0.6867
Q9D8T7	Slirp	SRA stem-loop-interacting RNA-binding protein, mitochondrial	14779.33594	14298.45	27931.46	9285.998	**16573.81055**	16159.18	13040.35	5091.488	16338.86	**12657.46814**	1.3094	0.4436
Q9CQL5	Mrpl18	Large ribosomal subunit protein uL18m	11482.01563	11231.37	13034.86	9220.937	**11242.29468**	8336.531	11288.7	7922.538	6873.201	**8605.24353**	1.3064	0.0754
Q91Z40	Gbp7	Guanylate-binding protein 7	3530.58252	3256.706	3095.394	3024.393	**3226.76886**		2142.473	2799.405		**2470.939087**	1.3059	0.0450
Q64689	St8sia3	Sia-alpha-2,3-Gal-beta-1,4-GlcNAc-R:alpha 2,8-sialyltransferase			5351.1		**5351.100098**	4099.866				**4099.866211**	1.3052	
Q60872	Eif1a	Eukaryotic translation initiation factor 1A	7760.437012	12939.34	13321.71	5147.228	**9792.179199**	4417.643	10487.45	8168.419	6947.738	**7505.312256**	1.3047	0.3713
Q99P88	Nup155	Nuclear pore complex protein Nup155	10461.49512	24491.58	10833.92	8260.6	**13511.89941**	8960.141	13223.12	10995.7	8261.302	**10360.06421**	1.3042	0.4463
P47867	Scg3	Secretogranin-3	10637.56348	10875	9522.651	10583.75	**10404.74121**	6821.689	8868.204	7686.065	8538.586	**7978.63623**	1.3041	0.0045
O70131	Ninj1	Ninjurin-1	11526.27539	9506.521	10094.27	7207.936	**9583.750854**	10094.2	6791.002	4910.812	7655.613	**7362.905518**	1.3016	0.1641
Q80YX1	Tnc	Tenascin	10738.44824	9116.173	14635.64	8272.716	**10690.74414**	6747.16	9791.186	11668.97	4660.015	**8216.832275**	1.3011	0.2841
Q60875	Arhgef2	Rho guanine nucleotide exchange factor 2	7522.556641	6382.34	8586.644	6373.145	**7216.171265**	4394.558	6556.688	4544.849	6693.688	**5547.445801**	1.3008	0.0877
P15388	Kcnc1	Potassium voltage-gated channel subfamily C member 1	85966.99219	78875.68	79884.19	80942.6	**81417.36523**	83026.24	77350.86	64762.01	25220.99	**62590.02441**	1.3008	0.2014
O35691	Pnn	Pinin	14795.66602	13163.79	14587.39		**14182.28223**	8821.235	14996.14	8895.613		**10904.32813**	1.3006	0.1951
Q9D8N2	Dennd10	DENN domain-containing protein 10	18493.27344	7817.52	13820.36	22819.58	**15737.68506**	6717.623	13827.55	10899.4	17032.24	**12119.20044**	1.2986	0.3885
Q9D2V8	Mfsd10	Major facilitator superfamily domain-containing protein 10			4825.615		**4825.614746**				3724.24	**3724.240479**	1.2957	
Q9QZA0	Ca5b	Carbonic anhydrase 5B, mitochondrial	8755.612305	9803.786	8923.382	17445.65	**11232.10815**	11013.96	10321.15	5753.977	7635.234	**8681.07959**	1.2939	0.3312
Q3U3R4	Lmf1	Lipase maturation factor 1	8912.685547	12583.14	8709.714	10897.48	**10275.75513**	7669.845	8146.013	7662.969	8304.612	**7945.859741**	1.2932	0.0460
O54990	Prom1	Prominin-1	13962.79102	22116.27	5276.957	7181.265	**12134.32117**	11978.97	12681.89	9638.516	3235.578	**9383.739807**	1.2931	0.5530
Q8VDP6	Cdipt	CDP-diacylglycerol--inositol 3-phosphatidyltransferase	36498.16797	31518.71	25566.71	39327.66	**33227.81055**	23237.58	30670.23	21873.43	27021.2	**25700.60938**	1.2929	0.0824
Q9R099	Tbl2	Transducin beta-like protein 2	5192.029297		6978.494	8014.041	**6728.187988**	5538.864	6506.152	4382.926	4396.52	**5206.115479**	1.2924	0.1579
Q6ZQK5	Acap2	Arf-GAP with coiled-coil, ANK repeat and PH domain-containing protein 2	10344.78418	7360.727	6171.828	2749.309	**6656.662048**	6467.796	6621.744		2363.917	**5151.152018**	1.2923	0.5231
P53996	Cnbp	CCHC-type zinc finger nucleic acid binding protein	25668.2832	16356.04	9672.981	19894.94	**17898.06055**	21679.88	10903.9	10639.64	12194.22	**13854.40747**	1.2919	0.3788
Q99K46	Usp11	Ubiquitin carboxyl-terminal hydrolase 11	3700.372803	6437.904			**5069.13855**	2696.582	5335.01		3741.297	**3924.296143**	1.2917	0.4796
Q9JJ61	Galnt16	Polypeptide N-acetylgalactosaminyltransferase 16	16315.69727	16485.21		12979.49	**15260.13379**	12670.25	13319.3	10408.71	10917.58	**11828.96021**	1.2901	0.0415
P68373	Tuba1c	Tubulin alpha-1C chain	8955400	8391475	9152751	8590283	**8772477.25**	8405972	9224602	7422862	2150327	**6800940.688**	1.2899	0.2647
Q71M36	Cspg5	Chondroitin sulfate proteoglycan 5	20865.00391	11378.63	9970.05	19263.36	**15369.2627**	12891.6	15354.76	6083.954	13338	**11917.07849**	1.2897	0.3500
Q3TGW2	Eepd1	Endonuclease/exonuclease/phosphatase family domain-containing protein	6775.719238	7582.854	7061.118	7597.675	**7254.341675**	6606.97	6371.512	3943.13	5605.615	**5631.806885**	1.2881	0.0432
P70280	Vamp7	Vesicle-associated membrane protein 7	24882.02148	18176.01	13313.5	16210.75	**18145.57031**	11932.71	11420.04	16054.58	16977.8	**14096.28076**	1.2873	0.2031
P97449	Anpep	Aminopeptidase N	7313.714844	6240.857	9136.419	4478.112	**6792.275879**	6312.842	4616.951	5494.659	4685.483	**5277.483765**	1.2870	0.2007
Q8JZY2	Commd10	COMM domain-containing protein 10	28944.61523	17405.58	22952.82	26688.46	**23997.87207**	18186.48	19322.64	17596.19	19539.34	**18661.16113**	1.2860	0.0825
Q9R069	Bcam	Basal cell adhesion molecule	25228.9375	22244.87	42776.4	29657.25	**29976.86426**	22475.08	20013.36	25356.4	25492.3	**23334.28467**	1.2847	0.2085
Q8CAK3	Shfl	Shiftless antiviral inhibitor of ribosomal frameshifting protein homolog			712.1596		**712.1596069**		561.309		547.8284	**554.5686646**	1.2842	
O09159	Man2b1	Lysosomal alpha-mannosidase		7196.459	4412.658	3425.669	**5011.595215**	3814.997	4884.883	3648.211	3287.494	**3908.896484**	1.2821	0.3326
Q6PCN3	Ttbk1	Tau-tubulin kinase 1	9494.950195	4865.782		6546.282	**6969.00472**	5560.528	4657.43	5168.299	6379.763	**5441.505005**	1.2807	0.2628
O88207	Col5a1	Collagen alpha-1(V) chain	31319.83594	37210.11	13908.42	4.548251	**20610.72739**	24418.9	556.2251	9729.818	29699.2	**16101.0343**	1.2801	0.6904
P97478	Coq7	5-demethoxyubiquinone hydroxylase, mitochondrial	22639.58008	28160.04	27249.57	29350.67	**26849.96582**	12133.53	21886.25	18405.46	31537.03	**20990.56714**	1.2791	0.2230
Q91YR5	Mettl13	eEF1A lysine and N-terminal methyltransferase	8525.974609	7579.862	8446.621	9112.124	**8416.145508**	4831.86	7948.582	6754.113	6795.488	**6582.510742**	1.2786	0.0435
P59808	Sash1	SAM and SH3 domain-containing protein 1	887.369812	104.8368		2327.989	**1106.731712**			865.7449		**865.744873**	1.2784	
Q8K124	Plekho2	Pleckstrin homology domain-containing family O member 2		1719.253			**1719.25293**		1345.296			**1345.296143**	1.2780	
Q68EF4	Grm4	Metabotropic glutamate receptor 4	8141.243652	13413.76	6202.719	8330.641	**9022.091431**	8524.879	4414.959	6868.046	8469.139	**7069.255859**	1.2762	0.3240
Q922J9	Far1	Fatty acyl-CoA reductase 1	10816.57129	7459.347	9488.618	8201.521	**8991.514404**	7262.016	11108.84	3833.67	5996.563	**7050.271667**	1.2753	0.2960
Q91YN9	Bag2	BAG family molecular chaperone regulator 2	6009.973145	11262.37	3746.802	14268.69	**8821.959534**	8880.237	6231.007	5956.709	6616.406	**6921.089722**	1.2746	0.4748
Q6NZM9	Hdac4	Histone deacetylase 4	4195.233398	2805.662	2963.23		**3321.375244**	3457.327	2091.083	2167.458	2708.868	**2606.183838**	1.2744	0.2303
Q8BFW7	Lpp	Lipoma-preferred partner homolog	5631.514648	4099.427	6495.316	4811.172	**5259.357422**	3556.016	1696.102	5165.023	6091.055	**4127.049194**	1.2744	0.3409
Q99LI2	Clcc1	Chloride channel CLIC-like protein 1	11479.86133	16413.12	28582.58	15051.51	**17881.76782**	14765.23		8990.636	18351.19	**14035.68522**	1.2740	0.4726
Q9CPP0	Npm3	Nucleoplasmin-3	8789.523438	7080.04	8044.659		**7971.407389**	6752.574	10310.45	3208.975	4758.729	**6257.680847**	1.2739	0.3993
Q8BPE4	Tmem177	Transmembrane protein 177	5809.402832	7956.888	8367.353	10794.65	**8232.072754**		4613.176		8326.897	**6470.036865**	1.2723	0.4081
P55065	Pltp	Phospholipid transfer protein	12263.64648	11242.07	28592.14	29474.33	**20393.04834**	14490.63	12215.46	14426	23006.24	**16034.58081**	1.2718	0.4610
Q8CIP4	Mark4	MAP/microtubule affinity-regulating kinase 4	10731.87793	13228.03	8968.449	8322.931	**10312.82251**	8563.139	7961.813	5429.748	10490.3	**8111.250488**	1.2714	0.1962
P12265	Gusb	Beta-glucuronidase	7536.964355	7163.647	6951.772		**7217.4611**	8483.106	5578.386	5214.158	3444.495	**5680.036499**	1.2707	0.2713
Q9QXK7	Cpsf3	Cleavage and polyadenylation specificity factor subunit 3	7480.512695	17689.84	15931.53	5668.926	**11692.70166**	5734.545	18136.64	7962.982	5023.553	**9214.430786**	1.2690	0.5828
Q8BYI8	Fam234b	Protein FAM234B	14906.80762	12711.66	10584.97	17898.34	**14025.44507**	8135.313	13829.17	11338.6	10915.57	**11054.66284**	1.2687	0.1786
Q8C0Y0	Ppp4r4	Serine/threonine-protein phosphatase 4 regulatory subunit 4	11439.74023	9653.656	11427.81	16102.57	**12155.9436**	7411.313	12573.2	11365.28	7002.971	**9588.192139**	1.2678	0.2393
P43275	H1-1	Histone H1.1	326234.3125	333792.2	454989.3	263462	**344619.4531**	333046.2	339110.6	196804.5	218569.5	**271882.7031**	1.2675	0.2323
Q8BG02	Ppp2r2c	Serine/threonine-protein phosphatase 2A 55 kDa regulatory subunit B gamm	13534.39844	8573.082	14585.84	17672.11	**13591.35815**	7968.655	17367.54	10817.57	6745.828	**10724.89819**	1.2673	0.3811
Q8BP71	Rbfox2	RNA binding protein fox-1 homolog 2	33692.08984	28064.5	24472.41	38563.36	**31198.08887**	25673.51	25799.07	21743.3	25265.43	**24620.32764**	1.2672	0.0894
Q99N87	Mrps5	Small ribosomal subunit protein uS5m	32268.10742	39161.66	42880.3	25784.93	**35023.75049**	31737.31	27140.47	31516.55	20230.48	**27656.20215**	1.2664	0.1637
Q8CJ53	Trip10	Cdc42-interacting protein 4	2015.092407	1845.716		2415.2	**2092.002726**	1511.695	1793.695			**1652.694885**	1.2658	0.1671
Q9CY57	Chtop	Chromatin target of PRMT1 protein	8420.114258	7495.091	7542.123	8478.032	**7983.84021**	9390.818	4185.483	6121.726	5537.552	**6308.894775**	1.2655	0.1911

P54285	Cacnb3	Voltage-dependent L-type calcium channel subunit beta-3	27421.94727	24317.33	24155.21	34409.49	27575.99316	23895.8	28268.33	16567.29	18444.73	21794.0376	1.2653	0.1576
Q8JZV7	Amdhd2	N-acetylglucosamine-6-phosphate deacetylase	18091.18945	16980.21	21168.05	23038.05	19819.37744	15531.19	12364.75	18655.6	16139.29	15672.70898	1.2646	0.0718
Q9D8S3	Arfgap3	ADP-ribosylation factor GTPase-activating protein 3	2722.628174	7753.571	2823.42	3486.244	4196.465698	3181.323	4901.567	1447.359	3749.384	3319.908325	1.2640	0.5535
Q7TN22	Txndc16	Thioredoxin domain-containing protein 16	7059.277344	8760.492	11268.75	13619.8	10177.07959	8116.808	8048.728	6893.245	9146.455	8051.30896	1.2640	0.2085
Q6PCZ4	Magee1	Melanoma-associated antigen E1		18088.25	24388.71		21238.47949	16803.65				16803.65039	1.2639	
P14602	Hspb1	Heat shock protein beta-1	88802.38281	56866.5	142743.4	108260.6	99168.20801	69226.38	64527.04	74727.35	105682.7	78540.86035	1.2626	0.3473
Q9ER58	Spock2	Testican-2	2678.842041			11318.66	6998.749146	5280.668	2300.485	4223.878	10373.2	5544.557373	1.2623	0.7143
Q3TLH4	Prrc2c	Protein PRRC2C	10461.58398	11640.35	18509.26	11330.11	12985.32593	11683.57	10738.51	8676.895	10187.86	10321.70654	1.2581	0.2234
Q91WK0	Lrrfip2	Leucine-rich repeat flightless-interacting protein 2	12265.87598	10973.76	15182.79	12028.75	12612.79565	9187.758	11666.13	7920.239	11330.18	10026.07581	1.2580	0.0874
Q8BUY5	Timmdc1	Complex I assembly factor TIMMDC1, mitochondrial	18374.17578	12425.88	20284	29718.2	20200.56201	18036.55	13056.47	16268.2	16937.28	16074.625	1.2567	0.3126
Q8K2A8	Alg3	Dol-P-Man:Man(5)GlcNAc(2)-PP-Dol alpha-1,3-mannosyltransferase	2301.613281	2264.263			2282.937988	1954.468		1679.531		1816.999817	1.2564	0.0784
P19137	Lama1	Laminin subunit alpha-1	10744.79883	8224.968	19051.46	9336.644	11839.46875	9053.765	11611.17	9746.859	7288.829	9425.154663	1.2562	0.3917
Q9QYK7	Rnf11	RING finger protein 11	11431.58008	10491.6	13393.63	20654.37	13992.79395	11670.95	11032.8	10850.11	11020.62	11143.62061	1.2557	0.2633
P58802	Tbc1d10a	TBC1 domain family member 10A	6745.227539	2936.87	3144.301	5115.26	4485.414856	5340.86	3495.837	2749.039	2713.745	3574.870422	1.2547	0.4354
O35143	Atp5if1	ATPase inhibitor, mitochondrial	452456.4688	272474.2	332349.7	403315.1	365148.875	220097.5	452495.6	152911.5	339420.1	291231.1836	1.2538	0.3745
Q91V01	Lpcat3	Lysophospholipid acyltransferase 5	12654.83496	9418.196	9867.244	8844.347	10196.15552	8582.531	7606.526	8222.867		8137.308268	1.2530	0.1018
Q8BG39	Sv2b	Synaptic vesicle glycoprotein 2B	102750.3438	52125.48	85047.2	91437.59	82840.1543	53026.63	49440.08	112111.7	50103.76	66170.53223	1.2519	0.4093
P50608	Fmod	Fibromodulin	12331.28711		45410.59	4329.86	20690.5804	25266.38	16076.68	23613.53	1164.211	16530.19812	1.2517	0.7503
Q9Z0R9	Fads2	Acyl-CoA 6-desaturase	4870.789551	5567.97	4076.845	4021.421	4634.256409	3829.607	3393.087		3888.318	3703.670736	1.2513	0.0952
Q8CI75	Dis3l2	DIS3-like exonuclease 2	17331.49219	15033.77	12481.43	22707.8	16888.62305	13187.49	15498.33	13278.4	12056.31	13505.13208	1.2505	0.1907
P58544	Btbd1	BTB/POZ domain-containing protein 1	44443	26585.74	43126.26	33932.92	37021.97998	35035.18	34569.82	11742.91	37284.05	29657.98999	1.2483	0.3532
Q9QXE0	Hacl1	2-hydroxyacyl-CoA lyase 1	11079.17676	10701.12		10669.23	10816.50879	10888.15	7312.077	7710.899	8774.207	8671.334106	1.2474	0.0744
Q8BT60	Cpne3	Copine-3	202447.7969	231178.4	244984.7	170192.3	212200.793	169572.9	194617.1	170610.8	146617.5	170354.5703	1.2456	0.0727
Q99J23	Ghdc	GH3 domain-containing protein	7128.780762	9909.248	9035.341	2780.761	7213.532776	5762.459	9562.357	2730.698	5114.945	5792.61499	1.2453	0.5290
P20152	Vim	Vimentin	4676875	4563992	7694403	4320653	5313980.625	4391218	4389033	3929412	4379797	4272364.75	1.2438	0.2433
Q03719	Kcnd1	Potassium voltage-gated channel subfamily D member 1	8794.217773	9225.802	5138.408	7583.85	7685.569214	7918.271	9992.754	2977.125	3847.216	6183.84137	1.2428	0.4596
Q8BG81	Poldip3	Polymerase delta-interacting protein 3	10051.24414	10846.45	11379.82	5032.661	9327.544922	5883.29	9475.497	6656.028	8007.472	7505.571899	1.2427	0.3139
P32261	Serpinc1	Antithrombin-III	6590.831543	5535.132	24176.14	269.6693	9142.943459	9117.651		5598.474		7358.062744	1.2426	0.8317
Q8K596	Slc8a2	Sodium/calcium exchanger 2	14907.41016	13843.8	20668.43	16975.79	16598.85449	16224.33	11721.93	17324.18	8219.64	13372.51953	1.2413	0.2585
Q9QY81	Nup210	Nuclear pore membrane glycoprotein 210	5387.289551	5565.615	7681.932	9628.991	7065.956787		7361.386	5127.252	4595.596	5694.744466	1.2408	0.3661
Q9Z329	Itpr2	Inositol 1,4,5-trisphosphate receptor type 2				5279.882	5279.882324	4256.631				4256.631348	1.2404	
Q8CES0	Naa30	N-alpha-acetyltransferase 30	8546.650391	11467.79	12891.03	20619.03	13381.125	10041.6	15055.52	5454.95	12607.34	10789.85266	1.2402	0.4614
Q9EPR5	Sorcs2	VPS10 domain-containing receptor SorCS2	12787.3291	11543.11	10150.52	10129.59	11152.64038	8626.394	9618.152	10349.82	7381.402	8993.940796	1.2400	0.0545
Q8R4G0	Ntng1	Netrin-G1	3894.985352	4466.753			4180.869141	1609.596	4564.69	2177.161	5140.535	3372.995514	1.2395	0.5727
Q61423	Kcna4	Potassium voltage-gated channel subfamily A member 4	9647.381836	8928.193	10409.55	14583.64	10892.19092	9574.709	7724.453	10490.27	7375.317	8791.188232	1.2390	0.2027
O35684	Serpini1	Neuroserpin	10210.9834		15978.71	12962.1	13050.59668	14476.5	12420.67	9984.54	5278.521	10540.05933	1.2382	0.3992
Q9JKY0	Cnot9	CCR4-NOT transcription complex subunit 9		15497.98	11696.28	3011.946	10068.7373		8145.515			8145.514648	1.2361	
O88196	Ttc3	E3 ubiquitin-protein ligase TTC3	13477.12598	12465.46	14540.97	9788.602	12568.03931	11025.28	13398.91	7182.996	9062.759	10167.48718	1.2361	0.2023
P01864		Ig gamma-2A chain C region secreted form	17306.47852	22496.46	15428.26	21250.91	19120.52881	7022.198	10552.68	26007.15	18291.7	15468.43225	1.2361	0.4519
Q5F2E8	Taok1	Serine/threonine-protein kinase TAO1	33229.35547	33805.22	35201.02	25746	31995.39893	26736.04	26146.39	18574.16	32189.18	25911.44336	1.2348	0.1340
Q8BH43	Wasf2	Actin-binding protein WASF2	14786.28418	14778.53	24655.43	13873.09	17023.33325	11971.69	15931.39	13774.88	13475.48	13788.35864	1.2346	0.2729
O55222	Ilk	Integrin-linked protein kinase	32295.46289	29790.58	49131.66	30308.36	35381.51709	31698.93	28828.93	29951.25	24171.57	28662.66895	1.2344	0.2184
Q6ZQ82	Arhgap26	Rho GTPase-activating protein 26	12480.17383	10393.78	11308.6	12332.6	11628.78833	8962.551	10242.62	9451.584	9031.297	9422.013428	1.2342	0.0082
Q61133	Gstt2	Glutathione S-transferase theta-2	10664.89844	8570.666	9412.734	11241.62	9972.479248	7793.525	8316.265	7018.077	9196.514	8081.095215	1.2341	0.0467
Q8CIF4	Btd	Biotinidase	19755.52734	18514.39	13215.22	20729.97	18053.77734	17061.46	10876.35	20639.66	9974.473	14637.98486	1.2334	0.3053
P25976	Ubtf	Nucleolar transcription factor 1	9912.828125	6527.994	5037.577	4869.705	6587.026001	5434.239	4734.927	5431.27	5764.874	5341.327515	1.2332	0.3353
Q9ESD7	Dysf	Dysferlin	6762.681641		8492.264	4814.475	6689.806641	5280.477	5262.149		5732.465	5425.030273	1.2331	0.3040
E9Q5C9	Nolc1	Nucleolar and coiled-body phosphoprotein 1	10483.51758	13747.87	11963.62	9332.48	11381.87183	11271.6	12356.22	3991.029	9307.6	9231.610474	1.2329	0.3429
Q01730	Rsu1	Ras suppressor protein 1	62940.53125	85275.25	116562.1	70997.3	83943.79883	84742.29	61754.62	57940.54	68121.21	68139.66406	1.2319	0.2768
Q6P6M7	Sepsecs	O-phosphoseryl-tRNA(Sec) selenium transferase	46696.4375	37158.11	52354.9	40069.05	44069.62402	34674.59	32792.71	30588.84	45042.55	35774.67188	1.2319	0.1263
Q3UHG7	Dennd11	DENN domain-containing protein 11	11765.14844	9915.364	11313.44	12812.53	11451.62036	7623.281	11010.32	11612.97	6939.394	9296.491333	1.2318	0.1543
O70551	Srpk1	SRSF protein kinase 1	2771.881348				2771.881348	1027.001		4429.029	1294.898	2250.309408	1.2318	
Q91Y86	Mapk8	Mitogen-activated protein kinase 8	9637.589844	10964.44	7629.369	9592.811	9456.053467	10050.14	7972.726	7304.167	5402.866	7682.475098	1.2309	0.1833
P10518	Alad	Delta-aminolevulinic acid dehydratase	82342.94531	72402.78	83676.14	70469.23	77222.77539	54636.75	55834.03	64761.45	75782.43	62753.66406	1.2306	0.0509
O35488	Slc27a2	Long-chain fatty acid transport protein 2	8982.353516	6089.122	8831.413	11326.33	8807.305176	8484.035	8044.421	6390.357	5717.069	7158.970581	1.2302	0.2378
Q91WC9	Daglb	Diacylglycerol lipase-beta	9978.423828	9275.433	14096.74	14246.5	11899.27393	9752.991	10258.27	8696.623	10016.87	9681.1875	1.2291	0.1551
Q9QX11	Cyth1	Cytohesin-1	38553.02734	42053.1	31814.18	22443.81	33716.02979	29811.61	27485.96	20823	31653.01	27443.39355	1.2286	0.2497
Q69Z99	Znf512	Zinc finger protein 512	6585.868652	10448.94	7487.185	5434.34	7489.084229	5347.942	7609.945	7376.529	4053.099	6096.87854	1.2283	0.3482
Q8BTM8	Flna	Filamin-A	34192.18359	36100.35	67467.08	38487.59	44061.80078	35691.99	36927.73	36326.78	34550.36	35874.21582	1.2282	0.3381
Q6ZWV7	Rpl35	Large ribosomal subunit protein uL29	217428.9688	218651.9	228243.1	243057.2	226845.3125	218901.7	187403.7	176570.1	156143.3	184754.7188	1.2278	0.0263
Q9CQX0	Otub2	Ubiquitin thioesterase OTUB2				1257.652	1257.651978		1283.858	765.259		1024.558594	1.2275	
P37804	Tagln	Transgelin	13093.99219	11580.36	27549.68	11982.86	16051.7229	10285.92	15611.99	13314.21	13094.68	13076.70044	1.2275	0.4849
P18581	Slc7a2	Cationic amino acid transporter 2	34192.51172	32149.87	25839.32	29896.91	30519.65137	24025.53	25519.94	23725.33	26184.34	24863.78369	1.2275	0.0240
Q921W0	Chmp1a	Charged multivesicular body protein 1a	29493.42188	49221.06	26406.86	34582.88	34926.05664	24777.96	30489.43	17555.22	41042.04	28466.1626	1.2269	0.3967
Q9CQH7	Btf3l4	Transcription factor BTF3 homolog 4	25828.82813	27081.08	27405.67	24981.67	26324.31152	18196.37	24536.41	21343	21825.57	21475.33789	1.2258	0.0141

Q91WG2	Rabep2	Rab GTPase-binding effector protein 2	19980.65039	20755.43	24111.3	25095.26	**22485.66016**	25170.18	16815.95	18809.26	12593.42	**18347.20044**	**1.2256**	**0.2035**
P43277	H1-3	Histone H1.3	758585.875	798671.1	1161879	897726.5	**904215.7031**	739174.6	780650	759180	672522	**737881.6563**	**1.2254**	**0.1262**
Q61464	Znf638	Zinc finger protein 638		8652.018	9397.732	7497.668	**8515.80599**	6143.881		7313.301	7401.863	**6953.015137**	**1.2248**	**0.0848**
A6H611	Mipep	Mitochondrial intermediate peptidase	3709.408691	4126.648	1889.941	4245.788	**3492.94635**	3136.341	2936.326	4036.641	1300.443	**2852.437988**	**1.2245**	**0.4483**
P09470	Ace	Angiotensin-converting enzyme	15170.15527	11580.04	12707.43	11713.97	**12792.89917**	8725.007	14245.02	8194.433	10650.76	**10453.80322**	**1.2238**	**0.1946**
F7BWT7	Tspan15	Tetraspanin-15	16367.57324	14476.06	23151.32	19889.84	**18471.19629**	15025.82	16219.53	14737.98	14426.94	**15102.56763**	**1.2231**	**0.1366**
P62849	Rps24	Small ribosomal subunit protein eS24	99964.78125	94203.34	89844.82	108189.1	**98050.51172**	95357.54	103830.2	48408.96	73089.32	**80171.49316**	**1.2230**	**0.2192**
Q8BJD1	Itih5	Inter-alpha-trypsin inhibitor heavy chain H5	12993.9375	12128.28	37744.75	13739.99	**19151.73804**	15323.54	14284.67	17045.68	16000.79	**15663.66992**	**1.2227**	**0.5960**
Q61735	Cd47	Leukocyte surface antigen CD47	136429.4219	131547.6	159905.8	155083.9	**145741.6875**	122682.1	111655.1	131614.2	110974	**119231.3535**	**1.2223**	**0.0206**
Q99L27	Gmpr2	GMP reductase 2	36898.97266	28895.18	45333.03	29990.96	**35279.53271**	23149.08	23107.9	28848.44	40354.47	**28864.97412**	**1.2222**	**0.2921**
Q99KV1	Dnajb11	DnaJ homolog subfamily B member 11	37027.83203	30098.03	37732.24	34528.89	**34846.74854**	27721.27	36405.95	22826	27095.73	**28512.23584**	**1.2222**	**0.1058**
Q9EPE9	Atp13a1	Endoplasmic reticulum transmembrane helix translocase	19910.94727	16251.89	21277.23	22023.35	**19865.85303**	15655.65	14671.77	19376.69	15329.05	**16258.29028**	**1.2219**	**0.0731**
Q9WUD8	Faim	Fas apoptotic inhibitory molecule 1	25208.41797	21045.95	18351.39	18168.78	**20693.63379**	21347	16554.59	15151.21	14691.93	**16936.18164**	**1.2219**	**0.1444**
Q8BYB9	Poglut1	Protein O-glucosyltransferase 1	24561.21875	23043.45	22561.74	22491.51	**23164.47949**	15996.19	22953.15	17728.21	19203.83	**18970.34497**	**1.2211**	**0.0359**
Q64339	Isg15	Ubiquitin-like protein ISG15	7430.547852	4138.463	6190.113	2652.254	**5102.844604**	3057.802	4889.921	3971.688	4799.468	**4179.719543**	**1.2209**	**0.4508**
Q8BYZ1	Abi3	ABI gene family member 3	3078.500732		5843.992		**4461.246216**	3070.749			4240.359	**3655.554077**	**1.2204**	**0.6452**
Q8R1F1	Niban2	Protein Niban 2	7201.460938	7186.833	14545.63	7126.361	**9015.071167**	5459.86	7922.581	8236.317	7930.853	**7387.40271**	**1.2203**	**0.4367**
Q7TN98	Cpeb4	Cytoplasmic polyadenylation element-binding protein 4	2354.567627	2386.907		10082.55	**4941.340902**		3937.315		4163.008	**4050.161377**	**1.2200**	**0.8058**
Q8CFI7	Polr2b	DNA-directed RNA polymerase II subunit RPB2	5077.393555	8426.447	5525.826	9971.621	**7250.322021**	7488.802	5424.001	4611.287	6247.031	**5942.780273**	**1.2200**	**0.3612**
Q91W67	Ubl7	Ubiquitin-like protein 7			8164.426	3798.078	**5981.252197**			5359.123	4457.718	**4908.420654**	**1.2186**	**0.6778**
Q9JJ43	Rbfox1	RNA binding protein fox-1 homolog 1		4326.367			**4326.366699**		1870.541	5235.663		**3553.101807**	**1.2176**	
Q91X52	Dcxr	L-xylulose reductase	11652.97363	10330.09	13629.46	9235.392	**11211.97803**	9040.522	9321.642	8991.829	9483.313	**9209.32666**	**1.2175**	**0.0802**
Q9CQ19	Myl9	Myosin regulatory light polypeptide 9	17715.13281	15166.72	27539.3		**20140.38639**	14730.41		16667.95	18265.45	**16554.60612**	**1.2166**	**0.4108**
Q9EPV8	Ubl5	Ubiquitin-like protein 5	15455.32129	13043.87	13060.57	13125.94	**13671.4248**	13521.18	13657.43	8406.717	9375.388	**11240.18066**	**1.2163**	**0.1549**
O35166	Gosr2	Golgi SNAP receptor complex member 2	13539.14746	15782.71	11912.27	7645.765	**12219.97449**	12645.94	14170.82	4062.589	9323.302	**10050.66205**	**1.2158**	**0.4712**
O35704	Sptlc1	Serine palmitoyltransferase 1		3956.414			**3956.413574**		2023.639	2636.454	5102.452	**3254.181396**	**1.2158**	
P47802	Mtx1	Metaxin-1	54297.62109	56364.21	50467.86	63376.3	**56126.49805**	38020.28	58935.74	41234.52	46477.69	**46167.05859**	**1.2157**	**0.1113**
Q8K4Q7	Cerk	Ceramide kinase	590.6026611	528.6777	822.4048		**647.2283936**			510.3772	555.2444	**532.810791**	**1.2147**	**0.3989**
Q99JW4	Lims1	LIM and senescent cell antigen-like-containing domain protein 1	15297.7666	20582.1	22044.15	16326.54	**18562.63647**	17343.77	20905.38	8337.314	14546.62	**15283.27197**	**1.2146**	**0.3331**
Q5NCE8	Mrs2	Magnesium transporter MRS2 homolog, mitochondrial	7515.52832	9419.053	11380.79	11130.63	**9861.501465**	7172.096	8637.813	9321.852	7345.619	**8119.344971**	**1.2146**	**0.1430**
P31254	Uba1y	Ubiquitin-like modifier-activating enzyme 1 Y	52340.46094	54826.54	49520.51	53211.8	**52474.8291**	51428.12	48118.27	40110.28	33193.79	**43212.61426**	**1.2143**	**0.0719**
Q8CIV8	Tbce	Tubulin-specific chaperone E	6860.161133	8640.207	4853.474	3382.455	**5934.07428**	5457.206	3291.525	7364.424	3438.356	**4887.877747**	**1.2140**	**0.5114**
Q9R0A1	Clcn2	Chloride channel protein 2	26924.05859	18292.02	20586.04	22345.49	**22036.90283**	18324.5	16273.76	19227.63	18798.85	**18156.18408**	**1.2137**	**0.0926**
Q8VH51	Rbm39	RNA-binding protein 39	8451.105469	10796.66	12705.11	14071.45	**11506.08154**	10170.12	11826.06	6669.826	9256.089	**9480.522583**	**1.2137**	**0.2597**
O35718	Socs3	Suppressor of cytokine signaling 3			15602.38	13672.27	**14637.32373**			12064.75		**12064.74902**	**1.2132**	
P53657	Pklr	Pyruvate kinase PKLR	221468.6563	225247.6	396488	326740.3	**292486.1523**	231349.3	231469.2	273048.9	228578.5	**241111.4727**	**1.2131**	**0.2843**
Q5SSZ5	Tns3	Tensin-3	21386.42578	23048.64	24866.55	22136.04	**22859.4126**	17616.83	18612.14	21581.17	17573.55	**18845.92188**	**1.2130**	**0.0158**
O09174	Amacr	Alpha-methylacyl-CoA racemase	7274.803711	5068.551	5232.747	5226.009	**5700.527588**	5774.672	3176.549	3880.109	5971.503	**4700.708374**	**1.2127**	**0.2943**
P35821	Ptpn1	Tyrosine-protein phosphatase non-receptor type 1	11391.91602		16933.43	14179.8	**14168.38216**	14450.08	10694.97	14955.83	6637.75	**11684.6571**	**1.2126**	**0.3915**
O55028	Bckdk	[3-methyl-2-oxobutanoate dehydrogenase [lipoamide]] kinase, mitochondri	18410.08008	21402.58	7081.686	18091.94	**16246.57227**	17213.5	4937.547	16299.67	15179.9	**13407.6532**	**1.2117**	**0.5287**
O88967	Yme1l1	ATP-dependent zinc metalloprotease YME1L1	16772.34766	14358	23667.82	15626.72	**17606.22388**	14074.02	14312.25	14049.96	15684.04	**14530.06738**	**1.2117**	**0.1962**
Q99J56	Derl1	Derlin-1	36896.96094	22527.69	33006.67	33695.18	**31531.62598**	30325.83	24899.23	20682.93	28195.21	**26025.79736**	**1.2116**	**0.1935**
P45376	Akr1b1	Aldo-keto reductase family 1 member B1	180781.1563	203435.6	323739	208554.2	**229127.5078**	182059.2	191617.6	177365.2	205645.8	**189171.9297**	**1.2112**	**0.2677**
P70255	Nfic	Nuclear factor 1 C-type		10383.67		11715.15	**11049.40674**	10426.78			7823.573	**9125.175049**	**1.2109**	**0.3187**
P62855	Rps26	Small ribosomal subunit protein eS26	142779.1094	180043.9	195582.7	155299	**168426.1992**	161067.8	156598.9	104043	134730.7	**139110.0977**	**1.2107**	**0.1478**
Q8VI63	Mob2	MOB kinase activator 2	44803.60938	40982.48	46834.52	48345.29	**45241.47363**	34985.62	29881.1	39755.79	44933.31	**37388.95361**	**1.2100**	**0.0717**
Q99P30	Nudt7	Peroxisomal coenzyme A diphosphatase NUDT7	34354.22656	33768.3	34870.94	33005.77	**33999.80859**	33511.76	29739.86	27766.44	21400.84	**28104.72363**	**1.2098**	**0.0612**
Q80ZS3	Mrps26	Small ribosomal subunit protein mS26	17296.33594	15806.31	17389.53	21616.44	**18027.1543**	16176.02	14145.2	12735.68	16560.72	**14904.40576**	**1.2095**	**0.0887**
Q8R059	Gale	UDP-glucose 4-epimerase	12187.94238	15347.8	9226.063	13956.38	**12679.54517**	3826.721	11360.96	10919.09	15829.32	**10484.02252**	**1.2094**	**0.4643**
P35951	Ldlr	Low-density lipoprotein receptor	11551.0498	11550.26	11794.71	9514.307	**11102.5813**	9026.856	12280.36	8300.592	7128.164	**9183.993042**	**1.2089**	**0.1685**
Q9EQJ0	Tpcn1	Two pore calcium channel protein 1				9816.305	**9816.304688**		8120.603			**8120.602539**	**1.2088**	
Q60847	Col12a1	Collagen alpha-1(XII) chain	162.8925171	2972.025	28213.47	3942.736	**8822.780075**	12651.44	2800.658	9544.061	4199.715	**7298.967712**	**1.2088**	**0.8327**
Q9CR23	Tmem9	Proton-transporting V-type ATPase complex assembly regulator TMEM9	16816.02148	14142.84	15175.17	16067.95	**15550.49512**	11346.58	7300.888	16453.62	16362.61	**12865.9248**	**1.2087**	**0.2836**
P15327	Bpgm	Bisphosphoglycerate mutase	28354.51367	27953.49	22397.83	26040.8	**26186.65869**	25136.26	22188.21	17136.43	22217.44	**21669.58594**	**1.2085**	**0.0801**
Q08509	Eps8	Epidermal growth factor receptor kinase substrate 8	30142.60938	23651.81	26328.9	25451.35	**26393.66602**	22959.02	26276.18	18687.96	19492.63	**21853.94824**	**1.2077**	**0.0862**
Q8BYI6	Lpcat2	Lysophosphatidylcholine acyltransferase 2	20682.22656	15167.34			**17924.78564**		12696.71	17165.35	14677.94	**14846.66862**	**1.2073**	**0.3291**
P55772	Entpd1	Ectonucleoside triphosphate diphosphohydrolase 1	8347.604492		10460.54		**9404.071289**	8267.465	10925.93	7091.931	4872.017	**7789.335693**	**1.2073**	**0.4644**
Q8BY89	Slc44a2	Choline transporter-like protein 2	51225.92578	45599.82	54343.47	45310.69	**49119.97656**	37152.89	37450.19	40758.91	47404.2	**40691.54785**	**1.2071**	**0.0410**
Q8R4X3	Rbm12	RNA-binding protein 12	9097.032227	4842.347	13314.63	8009.316	**8815.832153**	6890.225	5495.813	8075.657	8775.857	**7309.387939**	**1.2061**	**0.4563**
P54310	Lipe	Hormone-sensitive lipase	12884.38672	24017.34	8520.165	7802.649	**13306.13428**	11848.87	15217.92	7069.812	9997.029	**11033.4082**	**1.2060**	**0.6006**
E9Q3C1	C2cd2	C2 domain-containing protein 2	18171.39258	16721.87	19781.08	19102.27	**18444.15186**	14096.89	13772.11	18091.97	15226.88	**15296.96143**	**1.2057**	**0.0377**
Q64338	Pde1c	Dual specificity calcium/calmodulin-dependent 3',5'-cyclic nucleotide pho	7299.177734	5310.464	6426.461	6131.667	**6291.942505**	4855.115	6117.905	6000.801	3899.537	**5218.339233**	**1.2057**	**0.1578**
Q8BHK1	Nipa1	Magnesium transporter NIPA1	14949.78223	9557.508	4439.946	10396.69	**9835.982056**	8552.237	12116.13	5190.312	6775.669	**8158.587036**	**1.2056**	**0.5453**
Q9Z0F7	Sncg	Gamma-synuclein	367155.2188	353666.9	489536.9	447244.2	**414400.8047**	339849.1	339284.5	351322.1	344756.3	**343802.9844**	**1.2053**	**0.0734**
Q9DB43	Zfpl1	Zinc finger protein-like 1	2726.954346			1021.887	**1874.420746**		1054.659		2056.494	**1555.576294**	**1.2050**	**0.7777**

Q8BZW8	Nhlrc2	NHL repeat-containing protein 2	9020.506836	8117.601	11653.22	10637.27	9857.149048	8222.437	5947.736	9809.127	8753.646	8183.236572	1.2046	0.1916
Q8VCM7	Fgg	Fibrinogen gamma chain	19182.51758	17572.1	26964.61	24030.25	21937.36963	17716.22	14675.34	18603.89	21883.89	18219.83765	1.2040	0.2065
P41242	Matk	Megakaryocyte-associated tyrosine-protein kinase	4892.921875	6065.413			5479.16748	1946.922			7154.84	4550.880859	1.2040	0.7612
Q8R0A5	Tceal3	Transcription elongation factor A protein-like 3	55260.27734	44232.14	39863.28	57434.49	49197.54688	14020.8	49119.08	48682.26	51643.18	40866.32959	1.2039	0.4335
Q6DFW4	Nop58	Nucleolar protein 58	18508.07617	16000.21	12886.42	18528.39	16480.7771	14562.65	14503.82	12886.13	12842.56	13698.79102	1.2031	0.0981
Q9DAM7	Tmem263	Transmembrane protein 263	35793.14063	34153.48	35791.51	40469.68	36551.9502	33136.72	31673.23	25153.68	31602.37	30391.5	1.2027	0.0334
Q9Z2M6	Ubl3	Ubiquitin-like protein 3	64677.44922	76659.27	79908.3	73902.71	73786.93066	71908.52	68452.38	50124.06	54930.54	61353.87305	1.2026	0.0908
O88668	Creg1	Protein CREG1		2611.288	4668.321	4627.857	3969.15568	2355.852		3712.139	3840.008	3302.666341	1.2018	0.4663
Q3TIU4	Pde12	2',5'-phosphodiesterase 12	16187.55566	17386.99	17992.72	17311.97	17219.80786	13357.4	13823.37	14626.91	15553.46	14340.28174	1.2008	0.0033
Q04736	Yes1	Tyrosine-protein kinase Yes	54454.46094	65795.84	52606.91	65331.68	59547.2207	53241.37	55007.61	43469.38	46668.17	49596.63184	1.2006	0.0657
E9Q6C8	Xkr6	XK-related protein 6	10350.38184	10408.46	9202.778	13309.35	10817.74219	11123.7	6449.458	9100.899	9375.767	9012.456909	1.2003	0.2152
Q99MJ9	Ddx50	ATP-dependent RNA helicase DDX50	1818.986694		1394.321	10298.02	4503.774414				3753.217	3753.216553	1.2000	
Q9CV28	Mindy3	Ubiquitin carboxyl-terminal hydrolase MINDY-3	3297.958496	5246.323	3337.676	5733.738	4403.923828	3034.595	4885.427	2112.677	4656.961	3672.415161	1.1992	0.4560
Q6P069	Sri	Sorcin	103410.9766	131437.2	127218.4	123091.6	121289.543	105198.3	112881	98621.98	87931.01	101158.084	1.1990	0.0484
P17439	Gba1	Lysosomal acid glucosylceramidase	19812.95117	12637.81	16034.9	18189.1	16668.69092	14181.43	14669.8	11794.42	14963.34	13902.24683	1.1990	0.1568
Q91YW3	Dnajc3	DnaJ homolog subfamily C member 3	33826.04297	30528.99	21740.28	25196.67	27822.99365	24963.05	19063.62	27874.07	20982.29	23220.75537	1.1982	0.2180
Q9D8Y1	Tmem126a	Transmembrane protein 126A	42181.08203	39425.98	36336.98	51024.24	42242.07031	38995.66	37647.79	35407.55	29102.6	35288.40088	1.1971	0.1206
Q9QYI3	Dnajc7	DnaJ homolog subfamily C member 7	15081.2627	18000.97	16744.42	14916.65	16185.82422	13777.41	13531.16	10925.52	15888.44	13530.63354	1.1962	0.0784
Q3UVX5	Grm5	Metabotropic glutamate receptor 5	6853.266113	6184.881		15239.53	9425.891927		10731.33	5033.028		7882.181396	1.1958	0.7444
P30999	Ctnnd1	Catenin delta-1	137976.3125	133299.1	189580.8	136681.3	149384.3867	139214.8	125630.2	115983.3	118907.6	124934	1.1957	0.1403
Q8BHJ6	Serinc5	Serine incorporator 5	22053.00977	25148.39	29480	31828.74	27127.53662	21681.1	27834.08	16916.18	24320.56	22687.98242	1.1957	0.2112
Q9D964	Gatm	Glycine amidinotransferase, mitochondrial	14065.18945	17557.73	24380.5	18983.56	18746.74512	15294.29	17041.62	15472.12	14927.72	15683.93774	1.1953	0.2121
P17225	Ptbp1	Polypyrimidine tract-binding protein 1	30313.05469	28212.82	37359.82	31051.91	31734.40234	29410.75	26218.42	26717.72	23864.5	26552.84668	1.1951	0.0629
P97364	Sephs2	Selenide, water dikinase 2	26653.87109	23075.96	23989.06	28607.39	25581.56982	20789.21	23635.36	18974.52	22223.26	21405.58789	1.1951	0.0409
P98203	Arvcf	Splicing regulator ARVCF	9539.675781	9585.089	9171.736	6321.557	8654.514404	9217.907	6714.966	5410.644	7624.236	7241.938232	1.1951	0.2539
P46735	Myo1b	Unconventional myosin-Ib	23833.10938	23881.76	59544.67	29021.78	34070.33203	46349.4	28907.57	19490.2	19432.79	28544.99072	1.1936	0.6230
Q8BPA8	Dpcd	Protein DPCD	13886.5	6914.852	18241.41	20126.81	14792.39417	10538.13	13929.31	13319.08	11794.54	12395.26758	1.1934	0.4591
Q9CQV6	Map1lc3b	Microtubule-associated proteins 1A/1B light chain 3B	92720.60938	84298.78	87755.79	99672.3	91111.86914	95839.84	85814.04	90519.82	33509.06	76420.68848	1.1922	0.3601
Q99M31	Hspa14	Heat shock 70 kDa protein 14	13581.96289	13516.04	14321.13	15303.72	14180.71533	10212.71	13753.39	10986.01	12627.04	11894.7854	1.1922	0.0441
Q7TSC1	Prrc2a	Protein PRRC2A	10290.78516	7263.334	4352.019	5835.962	6935.525146	7538.182	7259.361	6176.136	2299.239	5818.229187	1.1920	0.5470
Q9CQI7	Snrpb2	U2 small nuclear ribonucleoprotein B''	8246.117188	6701.773	9238.077	13568.52	9438.621826	8636.887	7926.801	5746.924	9372.643	7920.813599	1.1916	0.3977
Q91VS7	Mgst1	Microsomal glutathione S-transferase 1	26977.44336	35099.43	33479.5	21337.74	29223.52783	28892.8	25604.73	18959.05	24663.72	24530.07568	1.1913	0.2603
O88811	Stam2	Signal transducing adapter molecule 2	4590.049805	5763.545	5137.516		5163.703613	3953.012		5243.757	3809.557	4335.442383	1.1910	0.2187
P62305	Snrpe	Small nuclear ribonucleoprotein E	49717.38672	60231.55	64323.72	56709.91	57745.64258	61389.78	60013.06	38172.45	34360.27	48483.88965	1.1910	0.2770
P01872	Ighm	Immunoglobulin heavy constant mu	9115.523438	11512.93	11236.02	5342.776	9301.81189		1628.741	13656.42	8160.648	7815.270182	1.1902	0.6768
Q69ZP3	Pnkd	Probable hydrolase PNKD	7824.674316	8757.189	9125.033	9538.782	8811.4198	8167.721	6511.237	8160.265	6775.239	7403.615601	1.1902	0.0496
Q8CJG1	Ago1	Protein argonaute-1	8731.188477	10375.43	9373.848	15923.87	11101.08496	11125.51	11825.37	5873.746	8496.304	9330.231812	1.1898	0.4377
P70318	Tial1	Nucleolysin TIAR	18466.33594	19801.61	16599.53	20167.13	18758.6499	21703.61	15967.51	13521.2	11874.89	15766.80249	1.1898	0.2404
Q9D2N9	Vps33a	Vacuolar protein sorting-associated protein 33A	23868.14844	28777.01	22204.5	18422.02	23317.91943	19648.36	18156.92	20319.98	20293.6	19604.71436	1.1894	0.1433
A0A1W2P872	Nova2	RNA-binding protein Nova-2	14550.69727	16561.97	16067.56	21815.46	17248.92432	12144.73	12161.88	20334.55	13375.41	14504.14233	1.1892	0.3182
Q8VDP3	Mical1	[F-actin]-monooxygenase MICAL1	10999.64746	5100.114	13081.23	5651.869	8708.2146	7342.31	6400.7	5673.941	9911.726	7332.169067	1.1877	0.5511
Q0VGY8	Tanc1	Protein TANC1	11541.33398	14174.48	7770.065	11450.64	11234.12915	10625.61	12305.89	8627.905	6277.186	9459.148071	1.1876	0.3744
Q9DC29	Abcb6	ATP-binding cassette sub-family B member 6	4023.028564	3804.431	8642.697	6531.002	5750.289734	6104.885	176.8671	10466.22	2620.117	4842.022018	1.1876	0.7300
P70345	Bcl2l2	Bcl-2-like protein 2	12340.1123	7326.45	8890.504	10067.3	9656.091919	6258.965	8515.054	6435.455	11316.51	8131.49707	1.1875	0.3726
Q9EQH2	Erap1	Endoplasmic reticulum aminopeptidase 1	9519.898438	12683.73	10425.39	8787.931	10354.2373	11385.98	7996.967	8377.266	7122.395	8720.651733	1.1873	0.2406
Q6P2L7	Golm2	Protein GOLM2		6191.215	7835.815	6948.934	6991.987956	7096.751	5887.092		4692.482	5892.108398	1.1867	0.2611
Q8C570	Rae1	mRNA export factor	16400.70898	16622.15	14743.09	20298.81	17016.18945	13371.68	17548.23	14564.73	11881.42	14341.51489	1.1865	0.1621
P83917	Cbx1	Chromobox protein homolog 1	18309.0625	13462.9	8384.196	11639.82	12948.99292	11837.66	16189.31	7455.991	8215.049	10924.50342	1.1853	0.5083
Q9JIG8	Praf2	PRA1 family protein 2	87764.04688	74944.16	98910.46	79949.85	85392.13086	80114.52	92605.18	65954.75	49544.96	72054.85254	1.1851	0.2566
Q9EPR4	Slc23a2	Solute carrier family 23 member 2	24580.05273	24752.61	27267.96	34928.86	27882.37012	20045.85	24357.64	23556.75	26189.67	23537.47461	1.1846	0.1650
Q9D2Z4	Senp8	Sentrin-specific protease 8	8193.709961	8256.719	7927.206	3850.624	7057.064636	4984.394	5030.99	6631.43	7183.588	5957.600586	1.1845	0.3981
Q8K1X1	Wdr11	WD repeat-containing protein 11	13632.54688	12478.41	14494.87	15498.12	14025.98804	12110.79	10297.62	13329.14	11635.14	11843.1709	1.1843	0.0509
P54116	Stom	Stomatin	4270.682617	3906.639	8569.759		5582.36027	5137.229	5306.688	4390.322	4021.285	4713.880798	1.1842	0.5348
O08915	Aip	AH receptor-interacting protein	40041.49219	42879.95	54389.24	46654.3	45991.24609	42454.58	37722.55	33643.59	41568.6	38847.33105	1.1839	0.1022
O08582	Gtpbp1	GTP-binding protein 1	14568.33398	14880.41	14248.64	16739.68	15109.26733	9768.147	13234.49	15140.51	12919.4	12765.63818	1.1836	0.1089
O08688	Capn5	Calpain-5	38272.73438	32948.09	30638.77	31360.61	33305.05225	32763.5	31127.36	23329.23	25371.49	28147.89453	1.1832	0.1193
Q9CQW9	Ifitm3	Interferon-induced transmembrane protein 3			4749.876		4749.876465				4015.06	4015.060059	1.1830	
Q91VH2	Snx9	Sorting nexin-9	14896.73438	14010.94	10999.59	8840.197	12186.86548	11639.94	13064.34	5980.498	10543.94	10307.17969	1.1824	0.3989
Q80ZW2	Them6	Protein THEM6	93828.38281	105561.1	108952.1	106403.7	103686.3242	87377.04	95038.06	81771.18	86609.76	87699.00977	1.1823	0.0103
Q91X51	Gorasp1	Golgi reassembly-stacking protein 1	12470.76953				12470.76953		10552.06			10552.05762	1.1818	
O35118	Gfra3	GDNF family receptor alpha-3	2141.349121	3976.435	4310.334	3144.728	3393.211548	4433.243			1309.673	2871.458069	1.1817	0.6863
P11688	Itga5	Integrin alpha-5	14870.84375	14505.27			14688.05518		12441.2			12441.20313	1.1806	
P22437	Ptgs1	Prostaglandin G/H synthase 1			5078.493	2760.409	3919.450928	2187.876		4452.51		3320.192993	1.1805	0.7470
Q69ZK9	Nlgn2	Neuroligin-2	35376.13672	34292.55	37081.97	41208.7	36989.84082	34238.05	35686.45	38250.28	17168.57	31335.83936	1.1804	0.3039
Q91WG5	Prkag2	5'-AMP-activated protein kinase subunit gamma-2	27647.9375	28693.25	34416.74	28626.7	29846.15625	25593.22	22173.48	28398.22	24975.3	25285.05469	1.1804	0.0630

Q9JK92	Hspb8	Heat shock protein beta-8	25100.77539	18930.88	32666.49	22888.17	**24896.58057**	24948.96	23579.7	14913.59	20929.95	**21093.05054**		1.1803	0.3367
P04919	Slc4a1	Band 3 anion transport protein	28262.02148	23693	38090.41	27655.89	**29425.32813**	27336.78	24458.86	22420.64	25514.87	**24932.78857**		1.1802	0.2135
Q9JLV1	Bag3	BAG family molecular chaperone regulator 3	20559.95508	14616.49	23032.08	15421.02	**18407.38745**	15565.08	14913.76	17871.79	14060	**15602.65649**		1.1798	0.2467
Q9WTQ8	Timm23	Mitochondrial import inner membrane translocase subunit Tim23	30135.27539	25648.47	26531.8	32128.97	**28611.13135**	21632.41	23640.94	25746.8	25986.52	**24251.66992**		1.1798	0.0548
Q8CE50	Snx30	Sorting nexin-30	13304.07324	12352.74	12816.14	16340.06	**13703.25391**	10299.28	11473.72	11598.05	13126.1	**11624.28857**		1.1788	0.1002
Q8VDH1	Fbxo21	F-box only protein 21	17661.06836	16501.04		19688.42	**17950.17839**	18802.12	18581.96	7384.863	16173.37	**15235.57678**		1.1782	0.4442
P05480	Src	Proto-oncogene tyrosine-protein kinase Src	31596.13867	28583.73	27549.61	34185.52	**30478.74805**	26091.04	28778.5	24119.96	24581.34	**25892.71143**		1.1771	0.0465
Q3UPH1	Prrc1	Protein PRRC1	11814.4043	5086.946	9157.35	7657.704	**8429.101074**	6287.375	11812.27	4577.55	5983.689	**7165.220337**		1.1764	0.5738
Q8K273	Mmgt1	ER membrane protein complex subunit 5	19719.71875	19884.51	21294.5	17838.96	**19684.42334**	16745.53	17773.32	17180.08	15233.19	**16733.02979**		1.1764	0.0163
Q9Z2X1	Hnrnpf	Heterogeneous nuclear ribonucleoprotein F	24275.10742	21830.55	24372	23724.68	**23550.58398**	21889.66	20143.71	18183.44	19898	**20028.70166**		1.1758	0.0105
Q3U0M1	Trappc9	Trafficking protein particle complex subunit 9	13165.07422	11665.9	11892.22	9201.159	**11481.08813**	10127.87	8910.833	9920.949	10140.47	**9775.031006**		1.1745	0.1002
Q8K1R7	Nek9	Serine/threonine-protein kinase Nek9	13851.40039	16235.34	11273.33	13190.68	**13637.68896**	11434.3	13405.42	10852.47	10769.54	**11615.43335**		1.1741	0.1414
Q9JJF0	Nap1l5	Nucleosome assembly protein 1-like 5	16813.92578	18396.55	13071	16367.9	**16162.34595**	17242.99	15712.33	11126.98	10987.99	**13767.573**		1.1739	0.2651
O09044	Snap23	Synaptosomal-associated protein 23	33896.96094	34457.94	41305.86	41589.9	**37812.66602**	30928.37	32433.12	31895.05	33615.14	**32217.91797**		1.1737	0.0423
Q9QZD8	Slc25a10	Mitochondrial dicarboxylate carrier	104961.6797	100343.9	106460	109113	**105219.6582**	87229.7	99273.97	91963.88	80417	**89721.13672**		1.1727	0.0122
Q9D187	Ciao2b	Cytosolic iron-sulfur assembly component 2B		5232.071		8013.854	**6622.962646**	7514.401	4302.499		5128.067	**5648.322266**		1.1726	0.5896
Q9Z1X2	Ptdss2	Phosphatidylserine synthase 2	43478.57422	38131.3	38136.54	55966.32	**43928.18457**	44394.22	37381.34	28172.93	39916.56	**37466.26318**		1.1725	0.2783
Q3U2A8	Vars2	Valine--tRNA ligase, mitochondrial	8342.016602	8721.268	16822.09	6237.679	**10030.76331**	11054.95	8952.636	7379.415	6838.225	**8556.307007**		1.1723	0.5789
Q99JF8	Psip1	PC4 and SFRS1-interacting protein	34547.625	36006.32	16957.67	39263.11	**31693.6792**	16649.29	15257.07	41244.2	34999.25	**27037.45288**		1.1722	0.5921
Q06890	Clu	Clusterin	43050.38672	46696.8	49022.27	49049.28	**46954.68457**	39153.45	41782.87	38109.38	41197.3	**40060.75195**		1.1721	0.0059
Q8BG40	Katnb1	Katanin p80 WD40 repeat-containing subunit B1	16209.63281	17488.94	16603.82	18117.45	**17104.96191**	16722.13	16684.26	14124.62	10849.04	**14595.01001**		1.1720	0.1350
Q8C4Q6	Aida	Axin interactor, dorsalization-associated protein	24508.83984	22116.3	23867.02	20449.26	**22735.35498**	21787.76	17175.89	19512.91	19184.31	**19415.21729**		1.1710	0.0450
Q9Z0J0	Npc2	NPC intracellular cholesterol transporter 2	19039.31836	15496.84	21529.94	6185.34	**15562.86047**	14926.25	15206.98	12947.13	10092.48	**13293.20947**		1.1707	0.5476
Q8R4R6	Nup35	Nucleoporin NUP35	7432.029297	7507.783	7588.267	8833.685	**7840.440918**	7218.58	5824.814	5924.335	7839.579	**6701.826904**		1.1699	0.1047
Q9CR88	Mrps14	Small ribosomal subunit protein uS14m	14988.17285	15840.17	14724.63	16016.47	**15392.36035**	12619.7	10482.48	14725.96	14814.12	**13160.56616**		1.1696	0.0830
Q9D8B6	Fam210b	Protein FAM210B, mitochondrial	9465.520508	7750.732	11116.86	16216.36	**11137.36987**	11102.37	8927.303	8985.343	9077.307	**9523.080078**		1.1695	0.4285
Q6ZPU9	Kifbp	KIF-binding protein	15082.67676	11424.23	15171.88	11713.47	**13348.06226**	12637.25	17146.42	9767.444	6132.046	**11420.78992**		1.1688	0.4774
Q8C5Q4	Grsf1	G-rich sequence factor 1	10338.5332	9808.645	10803.96	11196.72	**10536.96411**	9806.546	10715.58	7776.742	7767.994	**9016.716675**		1.1686	0.1062
Q78HU3	Mvb12a	Multivesicular body subunit 12A	1951.811279		3122.551	3551.069	**2875.143799**	1380.354	2526.841	3469.605	2465.683	**2460.620758**		1.1685	0.5484
P62962	Pfn1	Profilin-1	402013.5	503218.4	649486.3	524862.7	**519895.2266**	409637.6	399930.5	427420.3	542834.8	**444955.7969**		1.1684	0.2629
P29699	Ahsg	Alpha-2-HS-glycoprotein	17562.07031	13512.36	12318.36	9858.255	**13312.7605**	12405.5	12617.17	9462.992	11097.11	**11395.69287**		1.1682	0.3189
Q6P2B1	Tnpo3	Transportin-3	47495.21875	49360.57	44495.17	30036.94	**42846.97412**	40342.59	34231.05	26007.62	46133.8	**36678.76514**		1.1682	0.3544
Q3THF9	Coq10b	Coenzyme Q-binding protein COQ10 homolog B, mitochondrial	4074.879883	4023.008	2320.844	2312.213	**3182.736328**		2383.1	3066.114		**2724.606934**		1.1681	0.5880
Q80WQ2	Vac14	Protein VAC14 homolog	21908.76758	17488.2	20946.95	21952.79	**20574.1748**	16918.26	15601.13	20386.2	17549.74	**17613.83496**		1.1681	0.0889
Q62178	Sema4a	Semaphorin-4A	24208.99219	9147.917		2911.451	**12089.45353**	14042.3	9994.933	7026.094		**10354.44173**		1.1676	0.8068
P55284	Cdh5	Cadherin-5	1362.858032	6309.686			**3836.27179**	3286.846				**3286.846191**		1.1672	
Q62245	Sos1	Son of sevenless homolog 1	18628.22656	14837.32	17577.55	18475.42	**17379.62939**	13386.82	15127.93	14890.95	16157.38	**14890.76904**		1.1671	0.0552
P23475	Xrcc6	X-ray repair cross-complementing protein 6		4042.58	3150.652		**3596.615845**				3082.326	**3082.325684**		1.1669	
Q9R0P5	Dstn	Destrin	346675.1563	408195.5	356480.9	373657.9	**371252.3672**	299586.6	350943.5	318971.8	303215.3	**318179.2891**		1.1668	0.0250
P47962	Rpl5	Large ribosomal subunit protein uL18	60214.375	64350.88	56304.3	64710.86	**61395.10352**	49759.08	56999.06	52416.96	51299.84	**52618.73535**		1.1668	0.0131
Q6PD24	Ankrd13d	Ankyrin repeat domain-containing protein 13D	15240.01563	19033.34	16984.92	20120.25	**17844.63135**	15109.09	10885.36	16275.11	18909.26	**15294.70605**		1.1667	0.2478
P97461	Rps5	Small ribosomal subunit protein uS7	128757.6406	129153.7	124829.3	143259.7	**131500.0859**	127486.9	117203.9	95375.19	110775.8	**112710.4258**		1.1667	0.0537
Q61599	Arhgdib	Rho GDP-dissociation inhibitor 2	13823.47949	12279.34	23501.99	8863.543	**14617.08887**	11928.95	15161.17	11539.01	11496.64	**12531.44336**		1.1664	0.5459
P28667	Marcksl1	MARCKS-related protein	37959.98047	24831.01	14974.19	25684.88	**25862.51636**	33402.43	7851.001	28046.29	19390.35	**22172.51941**		1.1664	0.6312
P08030	Aprt	Adenine phosphoribosyltransferase	29251.27344	29251.13	29855.63	26764.45	**28780.62158**	22459.29	26948.34	19174.28	30116.75	**24674.66504**		1.1664	0.1530
A2AT37	Upf2	Regulator of nonsense transcripts 2	7515.990234	7606.683	8256.958	5603.795	**7245.856445**	8488.266	2065.901	5835.658	8470.24	**6215.016174**		1.1659	0.5483
Q7TPW1	Nexn	Nexilin	9932.869141	9432.429	10365.01	7386.476	**9279.197021**	7701.697	6023.767	10340.63	7786.213	**7963.075439**		1.1653	0.2797
Q6PAJ1	Bcr	Breakpoint cluster region protein	19582.01953	18909.67	16726.03	18286.55	**18376.06885**	17285.23	17289.77	15038.18	13472.01	**15771.30005**		1.1652	0.0580
P97447	Fhl1	Four and a half LIM domains protein 1	32240.23633	38028.82	30054.68	31687.45	**33002.79639**	28085.35	27229.3	26943.13	31057.47	**28328.81299**		1.1650	0.0560
Q8CH25	Sltm	SAFB-like transcription modulator	18280.70117	17981.49	16699.13	17868.43	**17707.43848**	21535.42	16594.21	8272.925	14397.52	**15200.01929**		1.1650	0.4004
O35495	Cdk14	Cyclin-dependent kinase 14	35785.85938	21247.85	15541.83	18866.81	**22860.58716**	20786.19	22926.78	19843.03	14974.56	**19632.63696**		1.1644	0.5239
Q99LM2	Cdk5rap3	CDK5 regulatory subunit-associated protein 3	11468.50781	12365.54	15369.06	12853.75	**13014.21533**	10564.5	11542.62	12525.99	10088.1	**11180.3042**		1.1640	0.1151
Q61140	Bcar1	Breast cancer anti-estrogen resistance protein 1	11276.29297	10139.18	19389.27	19244.05	**15012.19824**	13507.04	15098.43	13558.88	9433.773	**12899.53125**		1.1638	0.4753
Q91VC7	Ppp1r14a	Protein phosphatase 1 regulatory subunit 14A	43069.51172	51065.86	24143.13	23325.2	**35400.9248**	26229.13	33142.68	31122.52	31207.22	**30425.38818**		1.1635	0.5090
Q9JMB0	Gkap1	G kinase-anchoring protein 1	21758.29297	24567.48	20904.71	27271.16	**23625.4126**	28049.55	23484.79	13566.98	16147.16	**20312.12036**		1.1631	0.3962
Q9JKF1	Iqgap1	Ras GTPase-activating-like protein IQGAP1	30734.26953	22468.65	34726.94	25504.45	**28358.57764**	27177.97	20273.46	22695.14	27406.05	**24388.15479**		1.1628	0.2659
Q60738	Slc30a1	Proton-coupled zinc antiporter SLC30A1	26310.63477	31123.62	22483.88	20442.66	**25090.19727**	20427.3	28109.7	17861.19	19920.17	**21579.58887**		1.1627	0.3217
Q8R0X7	Sgpl1	Sphingosine-1-phosphate lyase 1	14691.75195	13443.26	15194.04	16309.25	**14909.57349**	11249.81	15845.7	14230.63	9970.822	**12824.23975**		1.1626	0.2061
Q8CGA3	Slc43a2	Large neutral amino acids transporter small subunit 4	17670.97852	12909.67	12218.67	12700.26	**13874.89575**	13030.45	13435.22	12279.34	8997.737	**11935.68774**		1.1625	0.2776
P10853;Q64475;Q8CGP	H2bc7;H2bc3;H2bc12;Hist1	Histone H2B type 1-F/J/L;Histone H2B type 1-B;Histone H2B type 1-K;Histo	3329419	3851356	4811187	4050155	**4010529.125**	3643621	3693519	3125397	3337654	**3450047.688**		1.1625	0.1453
Q8CGC6	Rbm28	RNA-binding protein 28		9491.029	14545.87	8016.569	**10684.48828**	7752.302	8794.978	11392.22	8828.689	**9192.047363**		1.1624	0.4658
Q3UR70	Tgfbrap1	Transforming growth factor-beta receptor-associated protein 1	13571.79199	11752.59	14072.05	16459.98	**13964.10327**	12901.86	11396.78	13644.86	10117.89	**12015.3457**		1.1622	0.1696
P24668	M6pr	Cation-dependent mannose-6-phosphate receptor	52702.66406	50837	52066.82	47896.59	**50875.7666**	51534.96	46781.81	37367.38	39485.92	**43792.51758**		1.1617	0.0855
Q9R0N8	Syt6	Synaptotagmin-6	18496.3418	15181.35	15895.65	14314.85	**15972.0498**	12435.13	14163.87	13694.64	14709.56	**13750.79932**		1.1615	0.0731
O54946	Dnajb6	DnaJ homolog subfamily B member 6	23059.4375	20669.67	23981.6	25890.98	**23400.42236**	22563.07	21003.19	17462.03	19556.18	**20146.1167**		1.1615	0.0781

Q60972	Rbbp4	Histone-binding protein RBBP4	46899.92188	55758.86	46828.29	45752.44	48809.87598	41423.21	43016.88	37828.33	45830.38	42024.69824		1.1615	0.0558
P58404	Strn4	Striatin-4	20212.31055	14175.05	18547.7	19854.07	18197.28247	18482.24	18817.96	12772.69	12604.04	15669.23242		1.1613	0.2966
Q8VCH6	Dhcr24	Delta(24)-sterol reductase	16446.55859	14487.7	11050.98	8662.781	12662.0061	14342.98	7480.041		10894.93	10905.98438		1.1610	0.5356
P31648	Slc6a1	Sodium- and chloride-dependent GABA transporter 1	27276.27344	26295.08	32178.24	30714.13	29115.9292	25604.06	24709.94	22577.77	27433.71	25081.37061		1.1609	0.0574
Q9QZN4	Fbxo6	F-box only protein 6	10110.13184	6045.635	17245.1	21585.5	13746.59204	10418.31	12574.86	10059.41	14314.54	11841.78027		1.1609	0.6185
P62911	Rpl32	Large ribosomal subunit protein eL32	94948.58594	99800.27	88415.36	104491.1	96913.82813	105571.9	93529.05	59818.54	75093.37	83503.20898		1.1606	0.2547
E9Q0S6	Tns1	Tensin-1	38649.03125	37026.5	55146.09	36090.25	41727.96777	35500.9	35623.61	34565.45	38146.87	35959.20801		1.1604	0.2536
Q8R5A6	Tbc1d22a	TBC1 domain family member 22A	5429.503418	5049.509	6575.738		5684.916829		4698.039	4577.975	5431.243	4902.418783		1.1596	0.2143
O88413	Tulp3	Tubby-related protein 3			12810.79	6502.038	9656.415283	6637.1		7872.647	10473.85	8327.867025		1.1595	0.6640
Q9DCL8	Ppp1r2	Protein phosphatase inhibitor 2	22426.39453	12303.12	14459.11	18306.69	16873.82813	20663.12	11233.4	11959.23	14357.83	14553.39575		1.1594	0.4814
Q9CZX7	Pip4p2	Type 2 phosphatidylinositol 4,5-bisphosphate 4-phosphatase	21687.52734	18170.55	23560.02	22066.34	21371.10889	17931.36	19319.17	15171.25	21320.01	18435.44531		1.1592	0.1393
Q8VDS8	Stx18	Syntaxin-18	11270.34766	10020.38	11612.5	11296.91	11050.03296	8711.158	9557.085	10779.01	9089.188	9534.111328		1.1590	0.0378
Q8C6B2	Rtkn	Rhotekin	13602.50098	13809.98	13414.66	9960.018	12696.79102	11415.46	12291.43	10302.13	9813.846	10955.71533		1.1589	0.1555
Q8BIJ7	Rufy1	RUN and FYVE domain-containing protein 1	7917.006836	10304.15	8573.071	6012.246	8201.617676	7310.084	8804.925	5297.914	6899.349	7078.067993		1.1587	0.3635
Q9DCS2	Mettl26	Methyltransferase-like 26	31351.16016	26468.15	25760.66	26125.46	27426.35742	23635.33	22239.41	24214.98	24624.82	23678.63721		1.1583	0.0382
P70399	Tp53bp1	TP53-binding protein 1	12817.56445	14615.63	16335.06	11607.91	13844.0415	11492.82	10305.12	11976.8	14039.09	11953.45728		1.1582	0.1947
P26039	Tln1	Talin-1	47566.46875	46241.1	68646.29	49865.76	53079.9043	43259.7	45866.01	47422.48	46913.64	45865.45703		1.1573	0.2242
Q8R3T5	Stxbp6	Syntaxin-binding protein 6	132973.3281	100418.5	158238.4	97707.7	122334.4805	96057.91	127731.3	83881.66	115365.2	105759.0215		1.1567	0.3778
Q8BWQ6	Vps35l	VPS35 endosomal protein-sorting factor-like	8001.587402	6980.678	11071.33	12170.78	9556.094604	8371.507	5774.454	10230.9	8670.151	8261.75415		1.1567	0.4326
A6H5Z3	Exoc6b	Exocyst complex component 6B	10902.47852	10576.54	15888.88	12083.13	12362.75513	10681.91	11378.56	9815.462	10878.62	10688.63892		1.1566	0.2329
Q8BZZ3	Wwp1	NEDD4-like E3 ubiquitin-protein ligase WWP1	15476.13477	21883.2	15906.34	22731.05	18999.17969	15694.72	19301.55	13310.43	17410.11	16429.20459		1.1564	0.3073
A2AWP8	Arhgef10l	Rho guanine nucleotide exchange factor 10-like protein		3568.854	7568.279		5568.566406	2472.594	8815.587	4129.878	3852.119	4817.544434		1.1559	0.7706
Q9CRA4	Msmo1	Methylsterol monooxygenase 1	20900.11133	30359.09	15176.7	16578.01	20753.47827	22612.81	16233.4	21193.68	11801.62	17960.37598		1.1555	0.5328
Q9R0N0	Galk1	Galactokinase	28045.12891	30385.82	25657.57	29395.78	28371.07471	24599.33	23698.36	26968.59	22951.74	24554.50781		1.1554	0.0297
Q9WVG6	Carm1	Histone-arginine methyltransferase CARM1	14822.24512	18892.43	21712.25	15980.88	17851.9502	12141.19	21893.89	14702.88	13064.31	15450.56738		1.1554	0.4078
P09602	Hmgn2	Non-histone chromosomal protein HMG-17	370.1061401	3748.473	9885.924	6546.119	5137.655502	385.1281	7166.115	6449.674	3788.165	4447.27066		1.1552	0.7950
Q8BGZ1	Hpcal4	Hippocalcin-like protein 4	17698.36133	19055.01	12542.09	15522.99	16204.6145	17586.22	13549.61	13584.92	11420.71	14035.36499		1.1546	0.3011
Q9D832	Dnajb4	DnaJ homolog subfamily B member 4	47389.76563	41089.19	62438.69	50297.51	50303.78809	46001.6	45167.23	42600.3	40534.92	43576.01367		1.1544	0.1979
Q80TR4	Slit1	Slit homolog 1 protein	11280.12891	7659.648	10922.41	12035.21	10474.34924	10209.54	9246.997	5364.357	11477.43	9074.580933		1.1543	0.4247
Q8BIP0	Dars2	Aspartate--tRNA ligase, mitochondrial	17058.82422	12349.47	14675.14	16227.44	15077.71924	13702.24	13262.22	15046.39	10251.31	13065.53857		1.1540	0.2138
Q9QUK3	Cln8	Protein CLN8		10615.32	10983.74	9827.249	10475.43587	7266.676	10152.47		9816.49	9078.547038		1.1539	0.2244
E9Q634	Myo1e	Unconventional myosin-Ie	12131.95215	11013.34	13382.28	10768.06	11823.90747	10880.05	12356.39	7475.2	10285.57	10249.30286		1.1536	0.2320
Q9R257	Hebp1	Heme-binding protein 1	11846.65527	9028.874	12656.69	13433.11	11741.33203	13310	8732.292	7917.52	10760.28	10180.02283		1.1534	0.3495
Q8K2Q7	Brox	BRO1 domain-containing protein BROX	41755.41016	44074.3	47222.23	40385.82	43359.44238	38310.86	41708.13	33622.7	36760.97	37600.66602		1.1532	0.0429
Q9Z140	Cpne6	Copine-6	128946.8594	132326.2	165810.3	142398.5	142370.4648	129734.3	129732.3	108535.9	125972.2	123493.6836		1.1529	0.1007
Q8BGZ2	Fam168a	Protein FAM168A		3007.873		5595.634	4301.753174			3674.095	3789.949	3732.022339		1.1527	0.7030
Q3UMY5	Eml4	Echinoderm microtubule-associated protein-like 4	24431.62891	22224.66	21823.71	21889.23	22592.30908	20971.57	20952.3	16189.32	20291.37	19601.13989		1.1526	0.0617
Q923D5	Wbp11	WW domain-binding protein 11	15159.69531	9303.627	8556.285	13358.04	11594.41113	11512.29	9185.09	11008.3	8532.216	10059.47437		1.1526	0.4121
Q9R112	Sqor	Sulfide:quinone oxidoreductase, mitochondrial	22854.24609	24092.45	29316.32	27159.79	25855.70068	19944.84	20808.49	24979.26	24026.13	22439.68066		1.1522	0.1235
P51910	Apod	Apolipoprotein D	79144.32813	72194.43	70219.51	41268.67	65706.7334	53159.21	49976.45	72122.03	52903.43	57040.28027		1.1519	0.4100
Q9JIA7	Sphk2	Sphingosine kinase 2	14983.46973	14787.46	18329.6	18052.38	16538.22632	12874.67	14995.92	15185.53	14398.37	14363.62354		1.1514	0.0932
Q8VBV3	Exosc2	Exosome complex component RRP4	1812.986206	8290.483			5051.734802				4387.583	4387.58252		1.1514	
Q9JLJ1	Selenok	Selenoprotein K	3805.546143	2822.549			3314.047363		1386.04	2886.202	4363.264	2878.502197		1.1513	0.7341
Q9CQ56	Use1	Vesicle transport protein USE1	11873.22266	10343.49	14275.42	9051.723	11385.96411	4434.564	7623.858	11781.45	15718.64	9889.627319		1.1513	0.5997
Q91X97	Ncald	Neurocalcin-delta	20604.01367	29903.11	22506.48	30933.88	25986.87256	26302.13	22941.47	24011.67	17031.97	22571.81006		1.1513	0.3355
Q8C1E7	Tmem120a	Ion channel TACAN	17543.79297	17791.51	22672.64		19335.97852	22178.82	15759.38	13818.37	15425.51	16795.51978		1.1513	0.3718
Q00558	F8a1	40-kDa huntingtin-associated protein	11637.20898	10000.55	12003.91	15793.41	12358.76904	11877.04	9736.687	8666.866	12670.16	10737.6875		1.1510	0.3320
Q8BWR2	Pithd1	PITH domain-containing protein 1	60418.20313	39588.83	56137.16	50715.41	51714.90039	39668.53	43460.61	48255.54	48351.6	44934.07031		1.1509	0.2211
P83887	Tubg1	Tubulin gamma-1 chain	36450.43359	33588.16	26082.26	19130.26	28812.76	31672.76	20347.4	20402.54	27724.63	25036.83252		1.1508	0.4619
Q8BU14	Sec62	Translocation protein SEC62	16716.19141	12238.81	15259.29	16613.99	15207.06885	15936.56	14079.39	10828.9	12021.57	13216.60474		1.1506	0.2429
P43276	H1-5	Histone H1.5	89455.85938	114481.9	145672.5	80481.45	107522.9434	101848.4	105203.5	97305.01	69459.92	93454.19531		1.1505	0.4327
P17047	Lamp2	Lysosome-associated membrane glycoprotein 2	53763.63281	51795.98	42259.02	45678.21	48374.21289	41293.11	44200.65	39143.67	43622.71	42065.03516		1.1500	0.0732
P23927	Cryab	Alpha-crystallin B chain	238143.1563	219581.3	316623.3	202614.9	244240.6719	235201.5	225759.2	198724.1	189859.2	212385.9766		1.1500	0.2892
Q9D6S7	Mrrf	Ribosome-recycling factor, mitochondrial	41322.30078	45741.12	40191.86	39331.47	41646.68555	41588.37	33776.24	36905.4	32642.14	36228.03662		1.1496	0.0695
P10833	Rras	Ras-related protein R-Ras	65574.1875	71182.81	85926.86	74553.23	74309.27344	76656.84	73037.61	55606.41	53263.56	64641.10449		1.1496	0.2360
Q9WUN2	Tbk1	Serine/threonine-protein kinase TBK1	7562.835938	6900.083	7221.435	5780.186	6866.134766	6509.292	7996.174	4073.739	5318.537	5974.435303		1.1493	0.3710
Q9ERI6	Rdh14	Retinol dehydrogenase 14	68038.39844	68314.78	54298.01	38937.03	57397.05469	58920.43	57977.96	34056.85	48867.07	49955.57813		1.1490	0.4421
Q9JKL4	Ndufaf3	NADH dehydrogenase [ubiquinone] 1 alpha subcomplex assembly factor 3	24734.54102	24165.12	19753.99	17224.81	21469.61475	18935.67	18969.37	17834.29	19007.97	18686.82764		1.1489	0.1776
P56393	Cox7b	Cytochrome c oxidase subunit 7B, mitochondrial	23944.10938	22321.26	18548.42	26173.13	22746.72998	19983.42	22164.94	17585.44	19500.96	19808.69043		1.1483	0.1656
Q8VHK5	Mlc1	Membrane protein MLC1	41054.45703	41909.81	38935.94	42779.16	41169.84082	27754.6	38262.2	38184.09	39209.75	35852.65771		1.1483	0.1095
Q8K3A0	Hscb	Iron-sulfur cluster co-chaperone protein HscB	8353.132813	5499.912	7582.349	8857.137	7573.13269	8493.286	7223.01	6978.327	3688.311	6595.733521		1.1482	0.4685
Q9CR64	Tmem167a	Protein kish-A	33162.67188	38230.53	31684.06	38434.98	35378.06006	38170.52	43612.18	21284.85	20213.63	30820.29492		1.1479	0.4881
P38585	Ttl	Tubulin--tyrosine ligase	26984.87109	23661.15	26079.46	18065.25	23697.68359	18961.6	20662.67	18525.9	24436	20646.54199		1.1478	0.2531
Q9CR51	Atp6v1g1	V-type proton ATPase subunit G 1	102445.6094	110623	114311.1	104835	108053.666	96603.2	107733.7	85399.23	86920.95	94164.28125		1.1475	0.0544
O70166	Stmn3	Stathmin-3	18358.26172	14334.71	17041.38	20789.59	17630.98633	14298.87	13731.8	15393.84	18050.35	15368.71338		1.1472	0.2198

Q61733	Mrps31	Small ribosomal subunit protein mS31	7939.326172	3494.78		3628.782	5020.962484	4483.946	5060.816	4612.155	3349.957	4376.718506	1.1472	0.6399
Q8K386	Rab15	Ras-related protein Rab-15	1273074.5	1463732	1288964	1411376	1359286.406	463807.3	1342817	1657889	1275137	1184912.375	1.1472	0.5253
Q9ER88	Dap3	Small ribosomal subunit protein mS29	16335.52832	14717.29	16972.37	18159.58	16546.19458	14043.4	12539.61	15723.02	15394.39	14425.10327	1.1470	0.0829
Q9D4V7	Rabl3	Rab-like protein 3	123288.3906	243366.4	220935.5	248568.1	209039.6094	91240.95	230790.6	227630.8	179334	182249.0684	1.1470	0.5626
Q8K1R3	Pnpt1	Polyribonucleotide nucleotidyltransferase 1, mitochondrial	21525.19531	15629.84	23517.68	24537.64	21302.5896	19509.9	15336.61	17770.3	21698.86	18578.91895	1.1466	0.3005
Q8R3S6	Exoc1	Exocyst complex component 1	12394.99609	9210.021	8663.984	11810.52	10519.8811	8543.878	9553.122	8222.676	10389.99	9177.41748	1.1463	0.2489
P63280	Ube2i	SUMO-conjugating enzyme UBC9	68025.53125	60811.5	50408.52	80358.05	64900.90137	55814.8	66327.92	54664.04	49747.13	56638.47461	1.1459	0.2946
Q8C0Z1	Fam234a	Protein FAM234A	14324.46191	12282.54	20209.67	14235.89	15263.13989	20033.78	15358.99	9856.627	8042.297	13322.9231	1.1456	0.5688
Q922H4	Gmppa	Mannose-1-phosphate guanyltransferase alpha	13894.89941	17069.4	18466.38	18645.29	17018.99292	14818.62	17491.07	12664.64	14449.29	14855.90601	1.1456	0.1951
Q9JHE3	Asah2	Neutral ceramidase	22029.84375	19368	22781.63	21504.77	21421.06152	17760.44	20292.94	17610.99	19132.82	18699.2959	1.1456	0.0307
Q9CQ10	Chmp3	Charged multivesicular body protein 3	49210.19922	50776.02	51406.17	50803.68	50549.01855	44485.62	45829.95	37050.62	49167.07	44133.31348	1.1454	0.0486
Q9JKK1	Stx6	Syntaxin-6	10820.79004	11882.04	9943.749	10084.75	10682.83179	8741.579	10567.18	10085.18	7914.759	9327.175415	1.1453	0.1221
P54726	Rad23a	UV excision repair protein RAD23 homolog A	18917.20313	19945.38	18647.83	19104.2	19153.65381	16516.89	12681.22	19575.84	18119.37	16723.32861	1.1453	0.1589
Q9Z1M0	P2rx7	P2X purinoceptor 7	24532.32227	14350.94	50741.3	21227.4	27712.99268	18699.82	18973.04	29855.93	29262.88	24197.9209	1.1453	0.6951
P09055	Itgb1	Integrin beta-1	26691.70313	30255.8	38459.96	26321.84	30432.32617	27971.57	28444.62	27925.97	21961.25	26575.85205	1.1451	0.2753
Q8R4V2	Dusp15	Dual specificity protein phosphatase 15	68193.95313	70146.03	102317.4	63321.29	75994.67285	70131.78	61307.18	65630.59	68449.66	66379.80371	1.1448	0.3313
Q99LI7	Cstf3	Cleavage stimulation factor subunit 3	12293.85938	14415.87	9008.266	20186.97	13976.24097	16797.98	10678.99	9674.197	11681.22	12208.09741	1.1448	0.5557
Q8R502	Lrrc8c	Volume-regulated anion channel subunit LRRC8C	7716.067871	9688.276	7444.971	6262.298	7777.903198	8525.701	6417.447	5615.314	6620.313	6794.693848	1.1447	0.3363
Q3UVR3	Ttbk2	Tau-tubulin kinase 2	10418.84082	8755.919	8099.424	9272.528	9136.677979	5883.827	11146.72	6773.206	8132.334	7984.021484	1.1444	0.3924
Q9EQP2	Ehd4	EH domain-containing protein 4	24645.48633	23045.95	38185.61	23584.33	27365.34326	27231.21	23519.01	23779.73	21130.24	23915.04688	1.1443	0.4028
P62889	Rpl30	Large ribosomal subunit protein eL30	154422.6406	163223.8	156124.9	161676.2	158861.8594	161992.4	146261.9	113642.4	133496.2	138848.2129	1.1441	0.1038
Q9DB29	Iah1	Isoamyl acetate-hydrolyzing esterase 1 homolog	46328.53516	50297.29	52065.3	34122.89	45703.50293	41662.26	36665.88	38373.67	43117.82	39954.9082	1.1439	0.2301
Q9R0U0	Srsf10	Serine/arginine-rich splicing factor 10	17025.79883	12907.82	15919	12847.13	14674.93726	14563.91	14728.87	10938.51	11095.19	12831.61914	1.1437	0.2630
P29391	Ftl1	Ferritin light chain 1	37104.57813	37495.27	57336.29	45298.78	44308.72852	37844.26	38850.13	39876.23	38430.11	38750.18164	1.1434	0.2867
Q9Z0M3	Cdh20	Cadherin-20	7364.596191	8844.06	8534.16	5897.32	7660.034058	7960.566	8659.343	5754.042	4423.113	6699.265991	1.1434	0.4487
P61226	Rap2b	Ras-related protein Rap-2b	75053.28906	63373.7	67882.14	82150.25	72114.84473	62263.61	64520.32	72144.16	53411.12	63084.80273	1.1431	0.1606
O88990	Actn3	Alpha-actinin-3				1871.507	1871.50708		1009.512		2265.019	1637.265686	1.1431	
Q8BJU2	Tspan9	Tetraspanin-9	32261.91211	30996.16	28976.04	26742.79	29744.22412	30775.67	28014.07	19537.75	25768.52	26024.00391	1.1430	0.2144
Q9CR58	Slc25a30	Kidney mitochondrial carrier protein 1	12291.05664	9225.71	9915.374	11366.9	10699.7605	9327.015	12700.66	6903.436	8532.294	9365.850586	1.1424	0.3787
Q3UH68	Limch1	LIM and calponin homology domains-containing protein 1	37839.44141	35119.21	42659.11	32226.82	36961.14648	35325.21	34667.71	29236.96	30193.76	32355.9126	1.1423	0.1392
Q9Z1Q5	Clic1	Chloride intracellular channel protein 1	32048.83594	27447.33	45386.44	37344.67	35556.81885	31827.79	26374.26	30790.78	35523.85	31129.16748	1.1422	0.3414
P62983	Rps27a	Ubiquitin-ribosomal protein eS31 fusion protein	1536441.125	1068160	1687618	1781320	1518384.906	1084737	1010359	1514064	1708659	1329454.75	1.1421	0.4447
Q6PGC1	Dhx29	ATP-dependent RNA helicase DHX29	4979.42334	6412.899	8466.377	3634.617	5873.329102	5843.377	5843.718	6115.118	2770.508	5143.180176	1.1420	0.5956
Q923G2	Polr2h	DNA-directed RNA polymerases I, II, and III subunit RPABC3	14663.94629	10278.17	14025.63	12795.12	12940.71753	12577.14	12762.21	9568.912	10442.06	11337.58032	1.1414	0.2470
Q8BHI7	Elovl5	Elongation of very long chain fatty acids protein 5	20493.90625	19259.25	24344	21225.54	21330.67627	23617.87	19030.7	18249.37	13854.94	18688.21899	1.1414	0.2894
Q60866	Pter	Phosphotriesterase-related protein	13360.39063	10013.37	10146.89	8724.966	10561.40503	9501.557	11791.01	7228.424	8492.832	9253.456055	1.1413	0.3799
O35639	Anxa3	Annexin A3	38825.40234	37238.8	49662.18	33186.55	39728.23242	39016.95	36267.05	34453.81	29544.98	34820.69727	1.1409	0.2704
Q6P542	Abcf1	ATP-binding cassette sub-family F member 1	16422.92383	18985.3	20490.79	18739.56	18659.64209	16371.96	13112.28	19944.99	15993.81	16355.76099	1.1409	0.2080
Q8BZM1	Glmn	Glomulin	11358.96094	12623.57	11376.43	12835.23	12048.54834	9258.534	8573.884	12620.3	11802.9	10563.90405	1.1405	0.2082
Q9R118	Htra1	Serine protease HTRA1	15029.06348	16695.03	21292.67	16979.67	17499.10718	16308.69	10757.01	15484.34	18845.89	15348.98486	1.1401	0.3567
O89051	Itm2b	Integral membrane protein 2B	27157.9082	22686.64	28696.89	33073.68	27903.78027	24535.26	22655.57	24627.9	26099.9	24479.65674	1.1399	0.1800
Q3UJD6	Usp19	Ubiquitin carboxyl-terminal hydrolase 19	10805.92383	12313.3	8141.107	11827	10771.83362	11494.49	9251.104	9289.36	7778.761	9453.428467	1.1395	0.3162
Q924Z4	Cers2	Ceramide synthase 2	19359.71484	14083.84	16362.79	21457.48	17815.9563	16394.24	14000.18	17476.45	14688.21	15639.77075	1.1391	0.2740
E9PV24	Fga	Fibrinogen alpha chain	21734.70508	19273.41	32055.21	23539.13	24150.61426	20121.89	20233.37	19748.32	24702.5	21201.52002	1.1391	0.3655
Q9JI75	Nqo2	Ribosyldihydronicotinamide dehydrogenase [quinone]	17079.32422	13984.67	13755.25	11935.23	14188.61841	12059.52	13101.53	8957.318	15728.28	12461.66187	1.1386	0.3645
P14576	Srp54	Signal recognition particle subunit SRP54	10939.46973	12639.42	12945.81	11388.03	11978.18408	10841.11	12519.61	8673.914	10054.34	10522.24243	1.1384	0.1711
Q3UZ39	Lrrfip1	Leucine-rich repeat flightless-interacting protein 1	4021.14502		8555.383		6288.263916	5828.984	6283.568	4377.531	5606.297	5524.094971	1.1383	0.6409
Q9QWI6	Srcin1	SRC kinase signaling inhibitor 1	45211.10547	49319.02	44087.82	40416.75	44758.6748	36014.1	43217.7	32938.26	45111.07	39320.28125	1.1383	0.1633
Q8BIW9	Chtf18	Chromosome transmission fidelity protein 18 homolog	337511.0938	361805.3	359977.6	373357.9	358162.9609	322510.7	293335.1	283736.4	359051.3	314658.375	1.1383	0.0572
Q9D6Y7	Msra	Mitochondrial peptide methionine sulfoxide reductase	34444.94922	32335.82	38573.73	39752.78	36276.81934	32916.03	31301.73	28132.78	35136	31871.63477	1.1382	0.1014
Q60715	P4ha1	Prolyl 4-hydroxylase subunit alpha-1	3828.563477	4495.668	5471.237		4598.489583	3166.455			4917.356	4041.905518	1.1377	0.5790
Q9R1Z8	Sorbs3	Vinexin	12422.27539	11767.81	17789	11956.47	13483.88916	14329.67	12863.39	10492.64	9732.273	11854.49414	1.1374	0.3977
Q01063	Pde4d	cAMP-specific 3',5'-cyclic phosphodiesterase 4D	11376.6123	12502.88	8007.079	10718.2	10651.19336	10309.99	9806.669	8761.098	8581.854	9364.902344	1.1374	0.2630
P07758	Serpina1a	Alpha-1-antitrypsin 1-1	37787.41797	31285.41	66441.02	51618.89	46783.18457	35381.63	35936.02	41509.77	51706.5	41133.47852	1.1374	0.5390
Q61210	Arhgef1	Rho guanine nucleotide exchange factor 1	13539.90332	14901.1	23795.26	12511.98	16187.06226	13971.85	13907.42	11782.92	17287.36	14237.38623	1.1369	0.5155
O88735	Map7	Ensconsin	11551.25488	13439.68	16501.46	9261.337	12688.43359	10098.11	10708.5	12671.36	11167.81	11161.44385	1.1368	0.3841
P01027	C3	Complement C3	60735.11328	59990.81	64721.18	45317.56	57691.16602	68253.3	63161.59	29119.04	42520.01	50763.48535	1.1365	0.5166
Q9DCJ1	Mlst8	Target of rapamycin complex subunit LST8	12074.86035	12927	11664.65	11440.14	12026.66406	12121.87	10227.03	11125.86	8858.385	10583.28687	1.1364	0.1087
Q61411	Hras	GTPase HRas	147292.7813	155915.8	154599.5	175657.4	158366.375	150553.3	151448.6	127773.2	127670.9	139361.5254	1.1364	0.0806
P70425	Rit2	GTP-binding protein Rit2	22437.83594	20677.56	19060.64	20409.61	20646.41162	23393.37	23563.38	9752.9	15986.17	18173.95654	1.1360	0.4931
Q8BYH7	Tbc1d17	TBC1 domain family member 17	22293.37695	23109.13	23410.51	21377.96	22547.74512	21734.28	20795.77	17676.47	19188.25	19848.68848	1.1360	0.0362
O88910	Mpp3	MAGUK p55 subfamily member 3	13226.84375	12011.01	11053.02	16946.39	13309.31494	9732.664	10830.2	14898.69	11420.36	11720.47852	1.1356	0.3878
Q76LS9	Mindy1	Ubiquitin carboxyl-terminal hydrolase MINDY-1	22882.70703	19710.5	20815.58	24973.79	22095.64551	20853.2	22711.55	16783.95	17533.46	19470.5415	1.1348	0.1986
B9EJA2	Cttnbp2	Cortactin-binding protein 2	8418.808594	6211	10149.73	5441.505	7555.262085	8434.963	7137.392	6896.714	4164.035	6658.276001	1.1347	0.5445
Q8R1B0	Stac2	SH3 and cysteine-rich domain-containing protein 2	39638.00391	46943.26	30165.48	28892.92	36409.9165	35570.71	29082.79	33230.06	30469.17	32088.18457	1.1347	0.3730

O35683	Ndufa1	NADH dehydrogenase [ubiquinone] 1 alpha subcomplex subunit 1	105694.0859	59679.04	96789.37	85052.72	**86803.80371**	75323.52	66775.19	89352.43	74670.9	**76530.50977**	**1.1342**	0.3876
Q8C0L8	Cog5	Conserved oligomeric Golgi complex subunit 5		3464.287	1883.435	759.0026	**2035.57489**		965.3278	2200.473	2218.248	**1794.683167**	**1.1342**	0.7995
Q9Z2C9	Mtmr7	Myotubularin-related protein 7	33445.23828	28063.29	25126.53	26301.75	**28234.20166**	23016.53	20847.39	26692.11	29025.36	**24895.45521**	**1.1341**	0.2455
P16675	Ctsa	Lysosomal protective protein	14736.09082	15605.49	15643.79	16090.96	**15519.08032**	10057.68	17581.3	14036.43	13065.44	**13685.21436**	**1.1340**	0.2888
P15379	Cd44	CD44 antigen	93738.42188	88075.89	65336.45	84031.05	**82795.45215**	78643.67	81142.02	68976.77	63300.78	**73015.80762**	**1.1339**	0.2361
Q9CR56	Nkiras2	NF-kappa-B inhibitor-interacting Ras-like protein 2		8268.885	7435.036	8157.823	**7953.914551**	6526.311	6862.735	7419.88	7250.583	**7014.877197**	**1.1339**	0.0333
Q9WV32	Arpc1b	Actin-related protein 2/3 complex subunit 1B	46721.63672	45255.13	62161.7	41401.21	**48884.91992**	45053.97	42421.32	42851.47	42135.91	**43115.66699**	**1.1338**	0.2577
Q9JKC6	Cend1	Cell cycle exit and neuronal differentiation protein 1	168250.8594	213408	177807.7	173073.5	**183135.0195**	166934.9	188174.7	142379.8	148627.7	**161529.3008**	**1.1338**	0.1881
Q02105	C1qc	Complement C1q subcomponent subunit C	22310.75195	16196.04	15885.87	13903.76	**17074.10547**	16279.45	17789.94	12297.08	13887.5	**15063.49341**	**1.1335**	0.3942
Q99JB8	Pacsin3	Protein kinase C and casein kinase II substrate protein 3	18374.49609	18998.91	18976.73	24004.86	**20088.74902**	19923.17	15246.97	16756.36	18979.73	**17726.55786**	**1.1333**	0.2112
P28474	Adh5	Alcohol dehydrogenase class-3	131920.1875	145901.3	150216.9	147771.3	**143952.4063**	117447.8	134885.9	115856.4	139929.7	**127029.9512**	**1.1332**	0.0607
P68134	Acta1	Actin, alpha skeletal muscle	36576.37109	64918.5	88203.92	21124.28	**52705.7666**	104329.7	21638.03	29161.6	30918.19	**46511.87061**	**1.1332**	0.8085
O35598	Adam10	Disintegrin and metalloproteinase domain-containing protein 10	32381.92578	33964.19	51313.35	36165.48	**38456.23535**	29302.05	32922.68	33804.52	39744.59	**33943.46094**	**1.1329**	0.3893
Q00897	Serpina1d	Alpha-1-antitrypsin 1-4	18157.5332	13470.07	32033.46	23834.68	**21873.93628**	20760.59	15962.58	15578.05	24927.63	**19307.21338**	**1.1329**	0.5945
Q9EP72	Emc7	ER membrane protein complex subunit 7	32294.99219	36530.71	40036.67	44410.73	**38318.27441**	36476.09	34143.23	29263.41	35430.47	**33828.2998**	**1.1327**	0.1887
Q8BIF0	Cd99l2	CD99 antigen-like protein 2	42631.18359	36951.13	36302.45	34086.28	**37492.76074**	38294.8	38672.75	25568.43	29901.63	**33109.40381**	**1.1324**	0.2815
Q8VDK1	Nit1	Deaminated glutathione amidase	26166.43164	29915.82	24717.44	25935.22	**26683.72803**	25199.47	25773.25	19628.66	23666.91	**23567.07373**	**1.1322**	0.1312
Q91V64	Isoc1	Isochorismatase domain-containing protein 1	45169.25	46013.63	48401.87	41806.77	**45347.87891**	44073.2	41508.36	35217.57	39473.03	**40068.04102**	**1.1318**	0.0628
Q3UUJ4	Strada	STE20-related kinase adapter protein alpha	19305.00586	20403.6	23100.07	17108.41	**19979.271**	17898.14	25480.06	19466.9	7770.985	**17654.02173**	**1.1317**	0.5711
Q8R1A4	Dock7	Dedicator of cytokinesis protein 7	11865.60938	14121.9	16415.14	11743.24	**13536.47168**	11010.23	11532.83	13496.54	11811.39	**11962.74683**	**1.1316**	0.2474
P16627	Hspa1l	Heat shock 70 kDa protein 1-like	37389.75781	41327.82	36575.14	32495.69	**36947.1001**	40096.53	30103.78	31897.52	28534.89	**32658.18164**	**1.1313**	0.2217
Q9D142	Nudt14	Uridine diphosphate glucose pyrophosphatase NUDT14	6747.30957	7186.025	11114.59	13308.73	**9589.165161**	7869.391	8349.6	7801.927	9884.694	**8476.402954**	**1.1313**	0.5262
P00158	Mt-Cyb	Cytochrome b	91081.32031	85197.24	85779.72	96379.7	**89609.49414**	99336.64	95335.38	44832.39	77366.63	**79217.7627**	**1.1312**	0.4442
Q8BGH4	Reep1	Receptor expression-enhancing protein 1	37463.67188	37398.11	36102.22	39786.8	**37687.69922**	34469.27	31918.49	30305.96	36586.35	**33320.01758**	**1.1311**	0.0329
Q8BXZ1	Tmx3	Protein disulfide-isomerase TMX3	72886.99219	68233.75	80637.12	76198.8	**74489.16406**	65593.82	68130.51	64688.76	65022.42	**65858.87695**	**1.1310**	0.0197
Q9WVA2	Timm8a1	Mitochondrial import inner membrane translocase subunit Tim8 A	107426.8125	44837.02	43968.54	39585.13	**58954.37305**	47265.14	49236.27	56063.49	55935.24	**52125.03809**	**1.1310**	0.6908
Q8VDD5	Myh9	Myosin-9	86703.89063	76085.21	100661.1	84148.03	**86899.5625**	81021.8	79391.16	71795	75149.43	**76839.3457**	**1.1309**	0.1185
Q922X9	Prmt7	Protein arginine N-methyltransferase 7	8516.431641	8053.58	6691.982	6469.694	**7432.921875**	7268.993		5835.374	6617.626	**6573.997396**	**1.1307**	0.2676
O08795	Prkcsh	Glucosidase 2 subunit beta	88704.63281	73154.77	62416.48	71510.78	**73946.66504**	77060.64	71692.8	52854.04	60036.11	**65410.89746**	**1.1305**	0.3124
P01887	B2m	Beta-2-microglobulin	18795.21094	18406.38	26865.01	25350.74	**22354.33691**	19012.89	20067.34	17390.6	22628.33	**19774.7915**	**1.1304**	0.3331
A2A432	Cul4b	Cullin-4B	13647.74023	16702.83	17438.19	12459.19	**15061.98828**	13072.25	12456.59	13342.96	14425.27	**13324.26782**	**1.1304**	0.2180
Q9QZ85	Iigp1	Interferon-inducible GTPase 1				14188.38	**14188.37695**				12551.97	**12551.97363**	**1.1304**	
Q9QUI0	Rhoa	Transforming protein RhoA	149100.8125	202834.6	175362.5	162972.9	**172567.7148**	180810	139172.2	123252.2	167517.4	**152687.9414**	**1.1302**	0.2964
Q64327	Mea1	Male-enhanced antigen 1	20115.74023	18197.35	17467.63	21869.14	**19412.46631**	19349.2	18420.99	14237.45	16710.91	**17179.63843**	**1.1300**	0.1866
P28230	Gjb1	Gap junction beta-1 protein	22243.94531	16181.83	17932.48	14973.34	**17832.89673**	16827.72	17555.65	14071.67	14692.53	**15786.89307**	**1.1296**	0.2981
Q8C7H1	Mmaa	Methylmalonic aciduria type A homolog, mitochondrial	6325.774414	7872.313	10895.41	11729.53	**9205.757446**	7386.814	6285.802	10631.86	8303.381	**8151.9646**	**1.1293**	0.5268
P43274	H1-4	Histone H1.4	802517.375	837507.3	1211867	1089434	**985331.2188**	575268.3	1022207	864350.7	1028912	**872684.5625**	**1.1291**	0.4671
Q61490	Alcam	CD166 antigen	92764.4375	87636.54	83888.96	103968.7	**92064.66016**	80558.91	88090.11	80955.53	76585.64	**81547.54688**	**1.1290**	0.0791
P61148	Fgf1	Fibroblast growth factor 1	118135.6484	108550.4	129798.8	154650	**127783.707**	107631.2	107115.8	122621.3	115566.2	**113233.6348**	**1.1285**	0.2194
Q99JR5	Tinagl1	Tubulointerstitial nephritis antigen-like	12156.03809	10045.68	24050.99	13727.92	**14995.15674**	10637.31	15299.83	13623.39	13593.09	**13288.40649**	**1.1284**	0.6193
Q8BMA6	Srp68	Signal recognition particle subunit SRP68	30367.68164	35673.37	41970.8	33499.49	**35377.83447**	32198.79	30477.21	32659.12	30089.32	**31356.10791**	**1.1283**	0.1634
Q91ZU1	Asb6	Ankyrin repeat and SOCS box protein 6	5076.75		3349.575	2166.221	**3530.848633**				3129.929	**3129.928711**	**1.1281**	
P62046	Lrch1	Leucine-rich repeat and calponin homology domain-containing protein 1	7844.411133	7594.703	4303.326	5676.542	**6354.745605**	6053.369	6340.031		4511.082	**5634.826986**	**1.1278**	0.5415
P61028	Rab8b	Ras-related protein Rab-8B	54428.54688	57846.37	60794.82	54122.77	**56798.125**	55806.27	54653.33	43674.94	47373.41	**50376.98828**	**1.1275**	0.1005
Q8K2K6	Agfg1	Arf-GAP domain and FG repeat-containing protein 1	15729.16016	17839.68	13570.24	16148.41	**15821.87158**	14032.49	15178.33	12568.19	14378.26	**14039.31738**	**1.1270**	0.1355
E9Q7X7	Nrxn2	Neurexin-2	15571.9248	17480.53	9059.632	14349.28	**14115.34277**	9542.416	13317.06	14481.77	12779.05	**12530.07495**	**1.1265**	0.4771
P56382	Atp5f1e	ATP synthase subunit epsilon, mitochondrial	458967.75	481978.8	345663.3	433501.5	**430027.8438**	426985.6	450689.7	279098.1	370169.1	**381735.6094**	**1.1265**	0.3571
Q99JG2	Gpr37l1	G-protein coupled receptor 37-like 1	25715.68359	28990.23	28151.52	22372.39	**26307.45703**	27670.13	25033.77	18936.87	21782.2	**23355.74268**	**1.1264**	0.2671
Q924T7	Rnf31	E3 ubiquitin-protein ligase RNF31	1928.194336	2377.226	1063.032	2993.292	**2090.43634**		2345.688	1749.407	1472.583	**1855.892537**	**1.1264**	0.6747
P97798	Neo1	Neogenin	23790.25586	14680.03	12573.76	16459.77	**16875.95264**	13471.02	23113.59	9645.359	13708.82	**14984.698**	**1.1262**	0.6331
O54879	Hmgb3	High mobility group protein B3	16347.88672	16320.15	9837.276	15992.6	**14624.47998**	15381.5	14542.57	13697.81	8320.897	**12985.69629**	**1.1262**	0.4949
O70318	Epb41l2	Band 4.1-like protein 2	162674.6875	163532	281537.3	154476	**190554.9844**	151532.9	159306	188998.7	176989.9	**169206.8555**	**1.1262**	0.5239
Q80YN3	Bcas1	Breast carcinoma-amplified sequence 1 homolog	282070.7188	281390.1	312948.5	237261.7	**278417.7617**	267987.3	246353.9	249060	225925.4	**247331.6563**	**1.1257**	0.1311
Q9Z1E4	Gys1	Glycogen [starch] synthase, muscle	14941.50977	15164.54	12282.74	15072.85	**14365.40991**	14427.6	12309.06	12392.1	11918.54	**12761.82349**	**1.1257**	0.1237
Q9JHL1	Nherf2	Na(+)/H(+) exchange regulatory cofactor NHE-RF2	19704.21094	18872.84	22451.26	15371.86	**19100.04321**	21966.08	19224.02	15138.47	11543.35	**16967.98218**	**1.1257**	0.4620
P20060	Hexb	Beta-hexosaminidase subunit beta	36934.95703	31885.36	50790.04	33259.25	**38217.40332**	33261.66	34996.51	37673.48	29881.98	**33953.41016**	**1.1256**	0.3919
Q8BFW6	Entpd3	Ectonucleoside triphosphate diphosphohydrolase 3	19649.62305	14963.71	15985.28	19087.69	**17421.5752**	16284.2	15420.88	13099.44	17111.62	**15479.0352**	**1.1255**	0.2255
O54818	Tpd52l1	Tumor protein D53	15341.86328	17879.21	15031.2	12690.8	**15235.7688**	14191.9	16482.35	12834.17	10647.13	**13538.88647**	**1.1253**	0.3351
O88986	Gcat	2-amino-3-ketobutyrate coenzyme A ligase, mitochondrial	29556.98438	28329.87	34325.61	37599.67	**32453.03418**	30930.41	28471.5	28763.14	27190.81	**28838.96631**	**1.1253**	0.1647
O35405	Pld3	5'-3' exonuclease PLD3	61823.23438	71026	70781.35	76189.96	**69955.13672**	65919.76	64045.7	63705.6	55063.25	**62183.57715**	**1.1250**	0.0897
P58771	Tpm1	Tropomyosin alpha-1 chain	144130.6094	128526.4	159082.2	165574.7	**149328.4609**	130124.9	145373.8	109129	146406.3	**132758.4941**	**1.1248**	0.2167
Q8R480	Nup85	Nuclear pore complex protein Nup85	110723.9531	46524.05	87645.83	94560.15	**84863.49512**	52828.67	80983.16	85049.35	82964.88	**75456.51563**	**1.1247**	0.5693
Q80U57	Rims3	Regulating synaptic membrane exocytosis protein 3	11822.875	13319.36	15962.94	12599.07	**13426.06055**	11034.54	11134.88	14254.46	11338.38	**11940.56299**	**1.1244**	0.2571
P97371	Psme1	Proteasome activator complex subunit 1	29578.02539	29611.63	40797.62	35464.32	**33862.90039**	26581.9	29438.4	35163.76	29350.73	**30133.69922**	**1.1238**	0.2938
A2AKG8	Focad	Focadhesin	15820.2627	12529.29	13122.64	16534.11	**14501.57788**	13510.16	16959.29	10083.14	11080.65	**12908.31079**	**1.1234**	0.4151

P41241	Csk	Tyrosine-protein kinase CSK	53434.64844	56001.21	62958.79	63065.29	**58864.98242**	48176.29	51516.85	52510.31	57436.29	**52409.93457**	**1.1232**	**0.0833**
O35449	Prrt1	Proline-rich transmembrane protein 1	24405.28516	23190.84	16570.32	24866.19	**22258.16016**	20803.1	21575.62	17467.61	19424.49	**19817.70166**	**1.1231**	**0.2952**
P61329	Fgf12	Fibroblast growth factor 12	63125.19141	55007.39	48198.88	49578.35	**53977.45313**	41293.32	54175.71	44823.98	51961.39	**48063.60449**	**1.1230**	**0.2396**
P22599	Serpina1b	Alpha-1-antitrypsin 1-2	17194.66211	16336.72	23566.71	14083.14	**17795.30737**	18562.53	14730.17	17597.55	12496.69	**15846.73413**	**1.1230**	**0.4581**
P70698	Ctps1	CTP synthase 1	26584.17773	17507.83	19259.43	20175.71	**20881.78857**	18991.57	17551.22	18397.79	19453.69	**18598.56934**	**1.1228**	**0.3019**
Q6ZWV3	Rpl10	Large ribosomal subunit protein uL16	69750.82813	62965.96	58062.02	63396.93	**63543.93359**	54718.77	62689.73	50495.84	58518.12	**56605.6123**	**1.1226**	**0.0978**
Q8BHR8	Cstpp1	Centriolar satellite-associated tubulin polyglutamylase complex regulator 1		6756.103			**6756.103027**	6060.771	5872.315		6126.449	**6019.845215**	**1.1223**	
A2AG50	Map7d2	MAP7 domain-containing protein 2	79691.17188	66115.77	70763.48	80153.73	**74181.03711**	66777.63	64455.38	66203.05	66973.23	**66102.31836**	**1.1222**	**0.0602**
Q9JIW9	Ralb	Ras-related protein Ral-B	17079.27148	18135.94	19329.73	20496.57	**18760.37598**	16146.44	18616.28	14761.45	17372.3	**16724.11792**	**1.1218**	**0.1158**
Q9EPQ7	Stard5	StAR-related lipid transfer protein 5	5992.96875	5163.184	8061.099	5909.17	**6281.605469**	5709.929	6240.775	5608.607	4842.633	**5600.486084**	**1.1216**	**0.3587**
Q8C025	Chpt1	Cholinephosphotransferase 1	50426.32422	45413.66	36876.63	28763.06	**40369.9209**	41600.46	31478.13	38680.16	32266.64	**36006.3501**	**1.1212**	**0.4477**
Q8K0D7	Get1	Guided entry of tail-anchored proteins factor 1	18804.90234	16640.81	22985.85	19756.37	**19546.9834**	22102.54	13495.4	19154.4	14994.41	**17436.68921**	**1.1210**	**0.4065**
Q9Z2G6	Sel1l	Protein sel-1 homolog 1	21427.8457	17511.25	18941.35	14659.29	**18134.93604**	22173.68	10464.78	16587.23	15483.31	**16177.25146**	**1.1210**	**0.5087**
Q8C165	Pm20d1	N-fatty-acyl-amino acid synthase/hydrolase PM20D1	13077.71777	9807.24	5753.857	8611.52	**9312.58374**	7577.042	10103.64	10963.5	4600.043	**8311.055542**	**1.1205**	**0.6479**
Q9JMF3	Gng13	Guanine nucleotide-binding protein G(I)/G(S)/G(O) subunit gamma-13	12687.76465	11257.51	13622.58	13140.8	**12677.16333**	11823.25	10550.17	13248.3	9642.717	**11316.10791**	**1.1203**	**0.1959**
P61600	Naa20	N-alpha-acetyltransferase 20	1891.680664	1726.909	3003.136	2724.499	**2336.556091**	1648.06	1883.121	2726.905		**2086.028564**	**1.1201**	**0.6089**
Q8R2U6	Nudt4	Diphosphoinositol polyphosphate phosphohydrolase 2	24501.94922	9620.34	14625.22	14418.15	**15791.41553**	10452.82	16454.84	18578.81	10916.58	**14100.76343**	**1.1199**	**0.6656**
Q8BLR2	Cpne4	Copine-4	14157.53906	14906.21	15414.85	18484.71	**15740.82813**	15366.29	13060.63	13404.63	14404.79	**14059.08398**	**1.1196**	**0.1717**
Q7TNV0	Dek	Protein DEK	59034.63672	63683.39	73887.82	56638.84	**63311.17188**	63533.5	61294.57	53929.86	47457.66	**56553.89941**	**1.1195**	**0.2486**
Q99MS7	Ehbp1l1	EH domain-binding protein 1-like protein 1		6212.612			**6212.612305**	6139.274	7207.65	3302.951		**5549.958577**	**1.1194**	
Q9EQN3	Tsc22d4	TSC22 domain family protein 4	21097.99414	21755.68	17840.65	17862.82	**19639.28613**	23192.46	18771.98	13258.48	14965.67	**17547.14746**	**1.1192**	**0.4241**
Q61315	Apc	Adenomatous polyposis coli protein	7152.246582	6325.161	7021.463	6067.923	**6641.698486**	4311.988	8327.303	4829.882	6267.723	**5934.223999**	**1.1192**	**0.4786**
Q8BFY6	Pef1	Peflin	40415.30078	43189.23	36594.19	40417.73	**40154.11328**	35899.02	36560.3	35957.95	35112.66	**35882.48242**	**1.1190**	**0.0217**
Q9JI91	Actn2	Alpha-actinin-2	20754.98438		22102.09		**21428.53613**	23256.6	15047.79			**19152.19482**	**1.1189**	**0.6391**
P04627	Araf	Serine/threonine-protein kinase A-Raf	23452.14258	22946.38	23219.03	27910.18	**24381.93164**	24502.09	18694.38	21227.41	22748.13	**21793.00391**	**1.1188**	**0.1798**
Q60870	Reep5	Receptor expression-enhancing protein 5	122871.75	66734.36	77881.27	72702.3	**85047.41797**	77093.49	92419.61	88679.22	45979.37	**76042.92188**	**1.1184**	**0.6068**
Q9CQY5	Magt1	Magnesium transporter protein 1	16646.90625	17225.17	15516.65	17732.9	**16780.40723**	23378.14	17699.78	7113.704	11825.25	**15004.21802**	**1.1184**	**0.6360**
Q8C5W3	Tbcel	Tubulin-specific chaperone cofactor E-like protein	21919.28516	24013.04	15159.79	16523.65	**19403.93994**	21390.28	19071.44	16286.7	12676.99	**17356.35205**	**1.1180**	**0.4966**
Q3TUH1	Tamm41	Phosphatidate cytidylyltransferase, mitochondrial	38589.34375	33480.53	33379.45	37687.73	**35784.26172**	28278.2	34352.63	30276.72	35194.87	**32025.60596**	**1.1174**	**0.1301**
Q62189	Snrpa	U1 small nuclear ribonucleoprotein A	48809.02734	43859.89	46543.91	56089.22	**48825.51172**	43603.54	52685.32	35303.23	43199.97	**43698.01367**	**1.1173**	**0.2898**
Q64213	Sf1	Splicing factor 1	35901.98438	32907.21	34908.93	29629.93	**33337.01367**	32970.22	30045.77	25765.29	30565.54	**29836.70752**	**1.1173**	**0.1370**
Q9JJE4	Paqr4	Progestin and adipoQ receptor family member 4	21094.81055	20510.12	29249.81	23338.02	**23548.18994**	23117.58	22288.62	19026.24	19877.39	**21077.45508**	**1.1172**	**0.3081**
Q61037	Tsc2	Tuberin	5963.601563	12681.62	6311.165	14280.7	**9809.271362**	11549.06	2498.329	11640.52	9435.508	**8780.854614**	**1.1171**	**0.7468**
P63216	Gng3	Guanine nucleotide-binding protein G(I)/G(S)/G(O) subunit gamma-3	25368.91797	39125.27	29619.15	44550.52	**34665.96729**	34321.63	30292.53	30811.44	28702.73	**31032.08105**	**1.1171**	**0.4531**
Q99JX7	Nxf1	Nuclear RNA export factor 1	8466.189453	19690.05	18045.45	10977.11	**14294.69946**	8807.494	19347.12	15287.49	7754.857	**12799.24048**	**1.1168**	**0.7117**
Q9D379	Ephx1	Epoxide hydrolase 1	84873.46094	84955.93	90468.15	78474.58	**84693.0293**	81341.21	77352.84	71145.99	73529.68	**75842.42969**	**1.1167**	**0.0371**
Q9D2P8	Mobp	Myelin-associated oligodendrocyte basic protein	405169.5938	438514.7	370012.3	359959.5	**393414.0234**	418032.7	373187	232283.1	386592	**352523.6992**	**1.1160**	**0.3974**
Q04899	Cdk18	Cyclin-dependent kinase 18	15124.25	11215.76	11178.84	13365.39	**12721.05908**	10241.92	11466.75	13785.81	10105.45	**11399.98145**	**1.1159**	**0.3405**
Q80TB8	Vat1l	Synaptic vesicle membrane protein VAT-1 homolog-like	319806.9375	377653.2	497154.7	355531.3	**387536.5234**	348024.4	351867.1	352723.5	336682.5	**347324.3984**	**1.1158**	**0.3378**
Q9QYY8	Spast	Spastin	10577.63379	10568.88	8765.393	9858.179	**9942.522461**	10431.21	4932.178	9980.656	10302.74	**8911.695801**	**1.1157**	**0.4884**
Q61187	Tsg101	Tumor susceptibility gene 101 protein	40300.05078	36893.28	47000.2	40045.01	**41059.63379**	40219.81	36899.57	33715.93	36420.79	**36814.02539**	**1.1153**	**0.1417**
O35075	Vps26c	Vacuolar protein sorting-associated protein 26C	21246.91406	19997.2	24510.44	25705.21	**22864.93896**	23340.94	21358.9	19293.33	18012.82	**20501.5**	**1.1153**	**0.2328**
P60060	Sec61g	Protein transport protein Sec61 subunit gamma	125269.4766	120395.5	123204.1	137703.7	**126643.209**	123776.8	120384.3	105677.3	104429.7	**113567.0254**	**1.1151**	**0.0821**
P54823	Ddx6	Probable ATP-dependent RNA helicase DDX6	86592.30469	87316.3	92161.45	93985.47	**90013.87891**	85481.27	76499.4	78363.32	82538.03	**80720.50391**	**1.1151**	**0.0142**
Q9CYH2	Prxl2a	Peroxiredoxin-like 2A	328064.2813	298200	346141.5	328166.9	**325143.1797**	316016.5	317323.9	260962.8	272029.1	**291583.0781**	**1.1151**	**0.1069**
Q9R1V4	Adam11	Disintegrin and metalloproteinase domain-containing protein 11	37836.63672	38855.49	39825.38	40859	**39344.12695**	31267.34	32186.17	39648.46	38061.61	**35290.89404**	**1.1149**	**0.1136**
P63325	Rps10	Small ribosomal subunit protein eS10	181865.8438	177983.3	179343.8	172395.6	**177897.1289**	157586.3	182494.7	141184.6	157150	**159603.8945**	**1.1146**	**0.0819**
P63082	Atp6v0c	V-type proton ATPase 16 kDa proteolipid subunit c	164722.9219	141336	146482.6	221302.4	**168460.9531**	164887.8	160894	158220.3	120564.2	**151141.5664**	**1.1146**	**0.4412**
Q3URS9	Ccdc51	Mitochondrial potassium channel	16096.07617	15599.38	16009.32	15141.07	**15711.46094**	15255.47	17302.93	8152.9	15675.17	**14096.61572**	**1.1146**	**0.4591**
Q8BWG8	Arrb1	Beta-arrestin-1	39069.05078	44493.31	37036.82	42780.1	**40844.81934**	42671.25	43150.58	30812.14	29968.74	**36650.68018**	**1.1144**	**0.3347**
Q3TMP8	Tmem38a	Trimeric intracellular cation channel type A				2243.64	**2243.639648**				2013.673	**2013.673218**	**1.1142**	
P58742	Aaas	Aladin	6933.071777	5057.322	5818.927	6560.811	**6092.533081**	5386.382	4940.963	4868.851	6676.595	**5468.197876**	**1.1142**	**0.3307**
Q3UFY7	Nt5c3b	7-methylguanosine phosphate-specific 5'-nucleotidase	25686.47266	22965.29	15887.27	21260.87	**21449.97632**	23941.19	22738.77	14186.43	16142.8	**19252.2981**	**1.1142**	**0.5142**
Q9D071	Mms19	MMS19 nucleotide excision repair protein homolog	10928.78223	8546.305	7011.776	11053.74	**9385.151123**	6542.875	9038.724	8710.651	9403.005	**8423.813721**	**1.1141**	**0.4431**
Q9CQS4	Slc25a46	Mitochondrial outer membrane protein SLC25A46	24079.96094	21872.27	18405.21	25465.54	**22455.74707**	22729.83	24882.34	13455.6	19554.7	**20155.61401**	**1.1141**	**0.4616**
P61793	Lpar1	Lysophosphatidic acid receptor 1	15079.65918	16590.39	10763.23	18059.76	**15123.25952**	11564.36	22517.03	8668.668	11548.07	**13574.53223**	**1.1141**	**0.6683**
Q9CWW6	Pin4	Peptidyl-prolyl cis-trans isomerase NIMA-interacting 4	11966.77148	12423.63	7173.7	10500.29	**10516.09888**	10457.43	9585.111	8658.212	9059.858	**9440.152344**	**1.1140**	**0.4222**
Q61686	Cbx5	Chromobox protein homolog 5	11203.2627	11604.99	10610.79	13287.91	**11676.73926**	10056.86	9952.371	13187.83	8737.2	**10483.56543**	**1.1138**	**0.3238**
Q3UDP0	Wdr41	WD repeat-containing protein 41	17111.26172	19453.13	12284.54	17147.98	**16499.22754**	15614.89	16774.41	11526	15350.83	**14816.53271**	**1.1136**	**0.4076**
Q3UEB3	Puf60	Poly(U)-binding-splicing factor PUF60	52134.81641	52328.43	54032.88	53572.57	**53017.17285**	44762.16	47898.8	49646.59	48152.93	**47615.12305**	**1.1135**	**0.0030**
O08807	Prdx4	Peroxiredoxin-4	14942.09863	16806.91	14555.14	12143.44	**14611.89746**	12609.75	12880.99	11078.07	15926.32	**13123.78198**	**1.1134**	**0.3274**
Q8CIP5	Disp2	Protein dispatched homolog 2	7927.719727	7601.496	7680.174	8594.299	**7950.922241**	8428.344	5236.464	10160.01	4743.924	**7142.185181**	**1.1132**	**0.5612**
Q8K0E8	Fgb	Fibrinogen beta chain	31740.89063	20099.63	31659.64	24420.41	**26980.14209**	21651.22	24954.29	23815.32	26527.33	**24237.03711**	**1.1132**	**0.4020**
Q8BUE4	Aifm2	Ferroptosis suppressor protein 1	19844.09961	21835.29	16945.3	16804.29	**18857.24268**	16425.02	18759.3	14134.33	18442.24	**16940.22339**	**1.1132**	**0.2809**
P24638	Acp2	Lysosomal acid phosphatase	18417.8125	16617.21	16845.95	13989.47	**16467.61157**	14796.89	18098.63	15405.25	10903.79	**14801.13892**	**1.1126**	**0.3764**

P62900	Rpl31	Large ribosomal subunit protein eL31	236105.2344	237088	269275	321142.6	265902.7266	228322.6	278737	222182.7	226807.7	239012.4883	1.1125	0.3052
D3Z7Q2	Smim20	Small integral membrane protein 20	21308.79492	20798.79	18591.7	18173.17	19718.11279	20092.97	17422.51	16254.59	17130.04	17725.02441	1.1124	0.1307
P63323	Rps12	Small ribosomal subunit protein eS12	129465.2188	160605.4	126876.5	150064.3	141752.8691	131691.2	132696.5	122758.3	122640.1	127446.5645	1.1123	0.1473
Q9DBS2	Tprg1l	Tumor protein p63-regulated gene 1-like protein	72315.32031	79362.23	72187.96	77810.97	75419.12109	69791.52	64953.06	63776.41	72732.93	67813.47949	1.1122	0.0347
P62627	Dynlrb1	Dynein light chain roadblock-type 1	287683.8438	285768.8	257995.5	294184.2	281408.0898	239132.9	255201.7	258957.9	259019.4	253077.9805	1.1119	0.0227
Q9CZV8	Fbxl20	F-box/LRR-repeat protein 20	14064.95508	11255.83	12482.96	12529.83	12583.39575	8570.848	15136.04	12920.65	8641.875	11317.35352	1.1119	0.4913
P67984	Rpl22	Large ribosomal subunit protein eL22	313687.625	303400.1	305879.6	323718.6	311671.4766	314515.6	286663.3	233429.6	286673.9	280320.5898	1.1118	0.1244
Q9QX60	Dguok	Deoxyguanosine kinase, mitochondrial	24830.46875	27444.4	27205.45	25558.13	26259.6123	21931.45	24165.09	25992.89	22412.77	23625.55078	1.1115	0.0570
Q5RL79	Krtcap2	Keratinocyte-associated protein 2		4341.185	4445.379	4449.45	4412.00472	3973.822	3317.526	4351.762	4235.323	3969.608337	1.1114	0.1689
O09172	Gclm	Glutamate--cysteine ligase regulatory subunit	108214.7656	112839	104520.1	101682.4	106814.0586	107992.1	99109.03	81259.31	96110.48	96117.7207	1.1113	0.1280
Q9D0R8	Lsm12	Protein LSM12	32135.46484	33829.23	32546.5	32451.48	32740.66748	29705.38	25642.04	31957.35	30563.11	29466.96924	1.1111	0.0589
Q61207	Psap	Prosaposin	46457	30574.21	40775.54	44173.73	40495.11963	30967.01	27753.68	41624.23	45454.74	36449.91553	1.1110	0.4887
Q9Z2C4	Mtmr1	Myotubularin-related protein 1	17782.05078	17530.48	20074.62	17267.93	18163.76855	15358.31	15789.73	15960.45	18295.96	16351.1106	1.1109	0.0973
Q80ZU0	Arl5a	ADP-ribosylation factor-like protein 5A	15522.44336	15792.82	16124.59	16552.96	15998.2041	14798.16	13512.5	11396.89	17926.1	14408.41187	1.1103	0.2945
Q8R0Y8	Slc25a42	Mitochondrial coenzyme A transporter SLC25A42	9849.532227	14294.47	13675.06	14929.61	13187.16748	8768.385	12968.32	13628.13	12146	11877.71045	1.1102	0.4366
Q68FM6	Elfn2	Protein phosphatase 1 regulatory subunit 29	32648.43359	30532.52	23817.99	29686.75	29171.42236	26960.58	27089.15	28390.06	22676.87	26279.16455	1.1101	0.2483
Q8R0G7	Spns1	Protein spinster homolog 1	15675.38867				15675.38867	14184.96		15477.22	12704.52	14122.23307	1.1100	
O89053	Coro1a	Coronin-1A	104365.6875	118976.3	114682	124546.9	115642.7148	101079.1	117484.1	103733.5	94469.45	104191.5195	1.1099	0.1263
Q7TPB0	Plppr3	Phospholipid phosphatase-related protein type 3	20438.52539	20166.86	16177.76	21514.25	19574.34863	16709.86	20445.04	17032.66	16367.69	17638.8103	1.1097	0.2453
Q80T41	Gabbr2	Gamma-aminobutyric acid type B receptor subunit 2	14852.56934	17009.77	18178.66	20214.47	17563.86646	14258.29	17831.48	12575.54	18650.45	15828.93921	1.1096	0.3791
Q8K1H1	Tdrd7	Tudor domain-containing protein 7	4286.192871	5542.898	6398.864		5409.318359	4655.628	4868.79	4960.733	5015.438	4875.147217	1.1096	0.3529
Q9R0Q9	Mpdu1	Mannose-P-dolichol utilization defect 1 protein	58055.75781	53879	59622.94	56072.62	56907.57813	48611.87	60350.63	52750.89	43497.07	51302.61328	1.1093	0.1878
Q8VBZ3	Clptm1	Putative lipid scramblase CLPTM1	36582.88672	34980.84	40253.02	38175.09	37497.95996	36476.86	29405.21	37503.77	31845.77	33807.89893	1.1091	0.1478
Q9DB25	Alg5	Dolichyl-phosphate beta-glucosyltransferase	7443.712402	8040.48	8574.833	7292.695	7837.930176	7642.684	8106.116	6174.773	6343.63	7066.800781	1.1091	0.2179
Q0VBD0	Itgb8	Integrin beta-8	18087.91797	24842.04	15175.16	18782.17	19221.82104	16780.35	16656.37	21005.54	14891.85	17333.52856	1.1089	0.4630
Q9JLV6	Pnkp	Bifunctional polynucleotide phosphatase/kinase	7212.234863	6973.016	7375.265	7196.001	7189.129272	4816.853	8746.2	5705.332	6666.454	6483.709717	1.1088	0.4371
P14733	Lmnb1	Lamin-B1	69312.3125	60858.2	85347.58	74691.15	72552.31055	65537.36	67889.09	61287.48	67066.57	65445.125	1.1086	0.2311
Q62077	Plcg1	1-phosphatidylinositol 4,5-bisphosphate phosphodiesterase gamma-1	23866.71094	23260.18	23315.3	26745.61	24296.95117	24525.39	23724.76	19303.66	20117.19	21917.75098	1.1086	0.1727
Q91V76		Ester hydrolase C11orf54 homolog	37395.80469	46407.08	40945.14	43361.93	42027.4873	41443.39	39666.5	30481.32	40073.95	37916.29199	1.1084	0.2396
P70697	Urod	Uroporphyrinogen decarboxylase	35913.77734	36528.77	39973.03	37428	37460.89453	29644.68	38289.15	29645.41	37614.64	33798.47021	1.1084	0.2029
Q9ES74	Nek7	Serine/threonine-protein kinase Nek7	36652.57813	37380.72	45490.31	36386.31	38977.48145	32199.23	34242.22	36061.09	38176.37	35169.72949	1.1083	0.1825
Q9JIY5	Htra2	Serine protease HTRA2, mitochondrial	61197.48047	67840.67	63890.34	71723.6	66163.02246	65790.95	63588.27	58786.48	50633.41	59699.77734	1.1083	0.1634
P51432	Plcb3	1-phosphatidylinositol 4,5-bisphosphate phosphodiesterase beta-3	13403.30273	12442.89	12377.02	12143.75	12591.73975	11351.84	12917.16	10917.62	10277.67	11366.073	1.1078	0.0985
Q6PDY2	Ado	2-aminoethanethiol dioxygenase	65538.25781	63568.81	64111.94	67045.73	65066.18262	62795.17	60438.25	51946.14	59759.86	58734.85547	1.1078	0.0433
Q56A07	Scn2b	Sodium channel subunit beta-2	36832.57031	36833.54	36335.82	51446.77	40362.1748	44380.94	37534.63	30460.97	33362.56	36434.77539	1.1078	0.4421
Q9R111	Gda	Guanine deaminase	28974.00586	28845.21	28846.27	38718.9	31346.09814	32356.87	30565.38	27582.02	22682.48	28296.68945	1.1078	0.3833
G5E870	Trip12	E3 ubiquitin-protein ligase TRIP12	7418.302246	8058.708	11823.05	10129.13	9357.29895	11097.68	7083.439	8167.626	7443.741	8448.122314	1.1076	0.5277
O35350	Capn1	Calpain-1 catalytic subunit	50620.90234	46216.77	67290.02	56733.96	55215.41504	46442.15	48727.41	52139.73	52096.86	49851.53809	1.1076	0.3040
Q91Z38	Ttc1	Tetratricopeptide repeat protein 1	107474.5469	57833.3	48356.09	47178.83	65210.69238	66921.6	58547.02	49413.05	60624.25	58876.47852	1.1076	0.6824
Q62159	Rhoc	Rho-related GTP-binding protein RhoC	112957.7422	124246.6	100736.5	86798.69	106184.8848	112496	103175.1	77961.82	89850.77	95870.93164	1.1076	0.3865
Q99LD8	Ddah2	N(G),N(G)-dimethylarginine dimethylaminohydrolase 2	162926.2813	179705.9	208842.1	143042.8	173629.2852	168333.9	161036	153882.2	143812	156766.0234	1.1076	0.3002
O88441	Mtx2	Metaxin-2	48218.79297	50834	46008.93	52611.5	49418.30566	54752.75	43873.83	35373.23	44487.5	44621.82617	1.1075	0.2994
Q99PL5	Rrbp1	Ribosome-binding protein 1	34511.48438	29885.49	30194.33	29190.66	30945.49072	29612.81	31146.2	26104.03	24911.25	27943.57324	1.1074	0.1643
P35123	Usp4	Ubiquitin carboxyl-terminal hydrolase 4	20188.29688	20471.52	19677.04	21482.45	20454.82764	12935.88	22872.97	24269.15	13812.53	18472.63037	1.1073	0.5316
Q80YQ8	Rmnd5a	E3 ubiquitin-protein ligase RMND5A	2028.844727			3389.162	2709.003418				2446.559	2446.559326	1.1073	
P63115	Slc7a10	Asc-type amino acid transporter 1	80468.05469	81877.13	61590.11	67202.22	72784.37891	63020.2	80033.59	56703.56	63207.26	65741.15332	1.1071	0.3569
Q61548	Snap91	Clathrin coat assembly protein AP180	1066581.875	1094628	1029294	1112852	1075838.813	945220	1057896	899516.6	985114.1	971936.5938	1.1069	0.0346
P51569	Gla	Alpha-galactosidase A	6578.157715	5121.578	7371.751	6032.555	6276.010376	4456.886	4894.901	7657.902		5669.896322	1.1069	0.5738
Q61749	Eif2b4	Translation initiation factor eIF-2B subunit delta	22042.32031	23443.08	19019.01	22699.91	21801.08057	21214.75	18736.48	20703.64	18133.14	19697.00293	1.1068	0.1365
Q7TSY6	Celf4	CUGBP Elav-like family member 4	13789.65137	17103.71	8892.09	8766.135	12137.89526	12659.36	11500.01	9324.88	10381.8	10966.51123	1.1068	0.6055
Q9ERL9	Gucy1a1	Guanylate cyclase soluble subunit alpha-1	9121.881836	8253.183	9516.037	9708.603	9149.926025	9864.031	7963.858	8423.873	6817.183	8267.23645	1.1068	0.2591
Q9DCZ1	Gmpr	GMP reductase 1	76406.33594	62019.38	68849.21	68990.99	69066.48047	56430.65	54431.04	73506.76	65308.85	62419.3252	1.1065	0.2548
Q9JL56	Gde1	Glycerophosphodiester phosphodiesterase 1	40991.03906	36650.04	39672.36	35183.21	38124.16113	35095.49	39652.43	27997.36	35079.09	34456.09277	1.1065	0.2311
Q3UN90	Lyrm9	LYR motif-containing protein 9	7809.171387	6986.707			7397.938965	4629.867	5269.884	10159.42		6686.390788	1.1064	0.7750
Q6ZWY8	Tmsb10	Thymosin beta-10	98021.39844	100178.5	103205.2	112114.6	103379.9102	107834.4	91218.09	56430.93	118265.6	93437.25391	1.1064	0.5008
P62082	Rps7	Small ribosomal subunit protein eS7	217390.9688	213507	232344.9	244615.2	226964.5117	210834	233445.7	166551.1	209770.3	205150.2813	1.1063	0.2140
Q9EQQ9	Oga	Protein O-GlcNAcase	123378.7656	128591.6	106519.6	137468	123989.4863	116878.4	108811.2	115272.4	107519.5	112120.3906	1.1059	0.1367
Q99MR0	Actl6b	Actin-like protein 6B	13872.4248	10056.05	12274.53	14028.94	12557.9856	9876.235	11111.35		13080.16	11355.91341	1.1059	0.4117
Q80WG7	Trim36	E3 ubiquitin-protein ligase Trim36	9037.995117	7976.187	11181.64	9681.518	9469.335327	8142.369	8989.756	8190.892	8929.736	8563.18811	1.1058	0.2482
Q9DC04	Rgs3	Regulator of G-protein signaling 3	13652.46094	7987.322	14344.67	9358.139	11335.6488	9643.697	14159.69	10967.55	6236.594	10251.88269	1.1057	0.6499
P58871	Tnks1bp1	182 kDa tankyrase-1-binding protein	16146.79688	14282.72	16875.35	14963.5	15567.09082	13124.81	14457.73	13945.96	14787.74	14079.06104	1.1057	0.0729
Q8C8N2	Scai	Protein SCAI	17218.91016	17387.37	16094.43	15525.51	16556.55444	17774.71	12069.49	15987.13	14066.51	14974.46021	1.1057	0.2720
Q99N57	Raf1	RAF proto-oncogene serine/threonine-protein kinase	2322.741699	3052.23	6458.393	1335.799	3292.290771	4298.829	1413.875	2484.445	3715.479	2978.157043	1.1055	0.8151
Q9CQQ8	Lsm7	U6 snRNA-associated Sm-like protein LSm7	31892.91406	31879.72	31454.79	32581.13	31952.13965	30582.3	28167.7	24512.52	32351.83	28903.58545	1.1055	0.1253
Q6NSR8	Npepl1	Probable aminopeptidase NPEPL1	14409.60254	9908.146	12046.93	9508.98	11468.41406	15128.62	7587.908	10228.53	8554.203	10374.81714	1.1054	0.6077

Q6IR34	Gpsm1	G-protein-signaling modulator 1	34735.50391	34571.73	40203.25	33260.68	**35692.79102**	32132.82	33553.7	30472.92	33010.98	**32292.60547**	**1.1053**	**0.0894**
Q8BH57	Wdr48	WD repeat-containing protein 48	18809.82617	17687.92	18330.91	10030.81	**16214.86597**	14536.64	12322.63	15977.11	15848.44	**14671.20459**	**1.1052**	**0.5166**
Q8CFB4	Gbp5	Guanylate-binding protein 5		55024.55	45472.28	44399.55	**48298.79167**	43706.17				**43706.17188**	**1.1051**	
Q8BYA0	Tbcd	Tubulin-specific chaperone D	38542.60938	35598.07	36182.31	39896.96	**37554.98926**	35821.5	31499.44	32982.73	35643.61	**33986.81738**	**1.1050**	**0.0499**
Q9CX80	Cygb	Cytoglobin	40411.72656	43848.58	33030.37	39620.92	**39227.89844**	34207.75	31947.16	38266.21	37663	**35521.03125**	**1.1044**	**0.2199**
Q9D1P4	Chordc1	Cysteine and histidine-rich domain-containing protein 1	42675.22266	43017.8	40921.79	40456.54	**41767.83789**	39440.81	41763.72	34534.85	35544.89	**37821.06543**	**1.1044**	**0.0711**
P23818	Gria1	Glutamate receptor 1	14884.02441	13750.54	13361.76	12993.3	**13747.40723**	12573.25	13109	11535.3	12576.4	**12448.48828**	**1.1043**	**0.0483**
Q06138	Cab39	Calcium-binding protein 39	53941.74609	58326.57	54161.57	50368.54	**54199.60449**	54308.05	48362.36	44270.37	49376.59	**49079.34473**	**1.1043**	**0.0992**
Q8R104	Sirt3	NAD-dependent protein deacetylase sirtuin-3	10218.29492	12928.06	7980.602	15010.71	**11534.41602**	9321.356	11103.99	12608.87	8748.23	**10445.61035**	**1.1042**	**0.5613**
O55142	Rpl35a	Large ribosomal subunit protein eL33	79087.91406	86896.25	70901.27	74278.63	**77791.01367**	68819.02	75710.77	64238.32	73085	**70463.2793**	**1.1040**	**0.1380**
Q9QZQ8	Macroh2a1	Core histone macro-H2A.1	323180.0938	362880.4	367060.1	363582.1	**354175.6641**	333152.6	332853.3	315960.5	301414.3	**320845.1797**	**1.1039**	**0.0412**
Q91YL3	Uckl1	Uridine-cytidine kinase-like 1	19652.45898	17814.95	17422.78	18376.23	**18316.60596**	20201.99	14359.11	16245.39	15565.53	**16593.00757**	**1.1039**	**0.2504**
Q8VHY0	Cspg4	Chondroitin sulfate proteoglycan 4	10186.45801	7741.833	9502.471	8088.357	**8879.779541**	8986.622	8195.81	7725.67	7270.912	**8044.753418**	**1.1038**	**0.2684**
P14131	Rps16	Small ribosomal subunit protein uS9	350116.5625	332932.3	383212	357290.2	**355887.7813**	336820.4	341962.6	297922.9	313023	**322432.2344**	**1.1038**	**0.0629**
Q8CE90	Map2k7	Dual specificity mitogen-activated protein kinase kinase 7	24809.08398	24190.37	27447.96	27141.95	**25897.33887**	23494.29	26761.69	17072.65	26542.51	**23467.78369**	**1.1035**	**0.3509**
Q3TEA8	Hp1bp3	Heterochromatin protein 1-binding protein 3	140164.9844	159231.4	181199.5	158795.3	**159847.7969**	144027.6	155229	136458.8	143715.2	**144857.6523**	**1.1035**	**0.1559**
Q810D6	Grwd1	Glutamate-rich WD repeat-containing protein 1		4508.083		6712.926	**5610.504639**	3856.68		6312.977		**5084.828125**	**1.1034**	**0.7803**
P24547	Impdh2	Inosine-5'-monophosphate dehydrogenase 2	38674.21094	39710.43	41462.09	49466.39	**42328.28125**	40613.76	41509.61	37660.28	33689.08	**38368.18262**	**1.1032**	**0.2373**
Q9CQ26	Stambp	STAM-binding protein	24600.04883	23972.03	27055.51	25903.41	**25382.74902**	23027.02	22443.55	23536.48	23027.92	**23008.74121**	**1.1032**	**0.0167**
Q8BHL3	Tbc1d10b	TBC1 domain family member 10B	28465.30469	20093.97	31484.36	28359.89	**27100.8833**	23972.75	24215.19	27249.73	22837.3	**24568.74023**	**1.1031**	**0.3713**
P50172	Hsd11b1	11-beta-hydroxysteroid dehydrogenase 1	28022.9375	23450.67	37977.55	38274.78	**31931.48389**	23673.47	31607	29590.71	30922.22	**28948.34961**	**1.1031**	**0.4958**
Q9JMG7	Hdgfl3	Hepatoma-derived growth factor-related protein 3	38072.60156	32353.34	33881.55	37475.36	**35445.71338**	29924	37254.81	32431.64	28934.44	**32136.22266**	**1.1030**	**0.2032**
Q6P8J2	Sat2	Thialysine N-epsilon-acetyltransferase	3516.523682	4569.155	3974.593	3423.768	**3871.009766**	1934.582	2985.707		5608.492	**3509.593547**	**1.1030**	**0.7234**
Q9CQR6	Ppp6c	Serine/threonine-protein phosphatase 6 catalytic subunit	39368.35547	45037.63	38525	39321.39	**40563.09277**	41841.02	37468.96	33642.47	34180.66	**36783.27832**	**1.1028**	**0.1682**
P62488	Polr2g	DNA-directed RNA polymerase II subunit RPB7	2640.860352	4391.031			**3515.945801**		2993.257		3384.268	**3188.762817**	**1.1026**	**0.7502**
Q5XJY5	Arcn1	Coatomer subunit delta	63265.73438	60146.09	64164.99	63956.94	**62883.4375**	57296.75	62868.74	51694.78	56302.41	**57040.66992**	**1.1024**	**0.0563**
Q3TL44	Nlrx1	NLR family member X1	13038.03906	14437.08	11840.74	14143.73	**13364.89795**	12707.85	13197.91	12525.67	10082.54	**12128.49048**	**1.1019**	**0.2246**
Q6GQS1	Slc25a23	Mitochondrial adenyl nucleotide antiporter SLC25A23	30571.83594	17335.24	26055.01	33356.52	**26829.65186**	26030.44	16861.61	37190.27	17314.29	**24349.14941**	**1.1019**	**0.6898**
Q91XE8	Tmem205	Transmembrane protein 205	49297.77734	46645.21	54628.61	46310.44	**49220.50781**	48829.98	46046.16	40330.56	43479.5	**44671.55078**	**1.1018**	**0.1360**
Q9JK81	Myg1	MYG1 exonuclease	48753.26172	44115.57	51492.55	43099.84	**46865.30859**	43178.79	45686.82	43317.33	37959.11	**42535.51172**	**1.1018**	**0.1417**
P50637	Tspo	Translocator protein	16512.39844	16013.29	23385.82	17355.56	**18316.76733**	15081.23	16100.96	17289.46	18030.26	**16625.47681**	**1.1017**	**0.3914**
Q9EPL2	Clstn1	Calsyntenin-1	20109.82617	19866.17	15990.51	14777.43	**17685.98242**	15292.65	13739.6	15179.47	20019.58	**16057.8269**	**1.1014**	**0.4297**
Q05BC3	Eml1	Echinoderm microtubule-associated protein-like 1	194915.2813	198575.8	157921.6	175742.1	**181788.707**	182285	179139.4	147857.3	150949.5	**165057.8086**	**1.1014**	**0.2478**
P55302	Lrpap1	Alpha-2-macroglobulin receptor-associated protein	36825.26563	35672.38	38708.48	42169.97	**38344.02539**	33773.55	37051.52	34113.35	34328.05	**34816.61621**	**1.1013**	**0.0707**
Q8C078	Camkk2	Calcium/calmodulin-dependent protein kinase kinase 2	18666.51563	19033.94	19244.58	18572.9	**18879.48535**	19383.21	15515.16	16905.32	16769.23	**17143.23047**	**1.1013**	**0.0799**
Q80X85	Mrps7	Small ribosomal subunit protein uS7m	11489.0293	12884	10980.07	11711.5	**11766.15186**	9755.135	12074.61	10544.15	10366.31	**10685.05054**	**1.1012**	**0.1404**
P18654	Rps6ka3	Ribosomal protein S6 kinase alpha-3	6877.73291	8092.932	9035.186	9895.055	**8475.226196**	7957.219	8213.088	7203.97	7412.846	**7696.780762**	**1.1011**	**0.3013**
Q9QYE9	Plekhb1	Pleckstrin homology domain-containing family B member 1	42880.97656	36599.21	38418.6	28783.64	**36670.60547**	34873.8	31687.38	29049.02	37627.11	**33309.33008**	**1.1009**	**0.3720**
Q8JZX9	Cdc42ep2	Cdc42 effector protein 2	29414.80078	29277.3	31114.18	32519.77	**30581.5127**	27723.04	27260.08	27651.48	28481.54	**27779.03467**	**1.1009**	**0.0135**
Q6PAM0	Prkab2	5'-AMP-activated protein kinase subunit beta-2	19505.53711	20696.02	19568.79	18534.02	**19576.08984**	16690.52	17673.79	18755.04	18013.14	**17783.12354**	**1.1008**	**0.0269**
Q8VDI9	Alg9	Alpha-1,2-mannosyltransferase ALG9	9909.583984	7904.016	11365.3	9510.105	**9672.250366**			8787.909		**8787.90918**	**1.1006**	
Q8CCJ3	Ufl1	E3 UFM1-protein ligase 1	25255.16992	20749.3	26610.88	20590.06	**23301.3501**	20470.21	21998.6	23029.92	19203.99	**21175.67627**	**1.1004**	**0.2724**
P62274	Rps29	Small ribosomal subunit protein uS14	45939.19141	50900.07	36898.27	46276.25	**45003.44434**	44334.5	47086.98	33172.48	38998.48	**40898.10742**	**1.1004**	**0.3710**
Q8R3R8	Gabarapl1	Gamma-aminobutyric acid receptor-associated protein-like 1	34253.69922	26271.78	32544.2	38362.82	**32858.12598**	26516.3	32542.59	29728.23	30661.18	**29862.07617**	**1.1003**	**0.3274**
Q9JMC3	Dnaja4	DnaJ homolog subfamily A member 4	26382.16211	25917	28181.15	28937.05	**27354.34082**	31004.32	17137.19	25099.38	26223.01	**24865.97754**	**1.1001**	**0.4335**
Q8VE85	Prelid3a	PRELI domain containing protein 3A	9390.99707	6188.258	5353.438	4742.203	**6418.723877**	7113.008	6375.246	8013.57	1842.707	**5836.132751**	**1.0998**	**0.7462**
O89079	Cope	Coatomer subunit epsilon	25804.51172	30747.11	31230.76	34121.51	**30475.97119**	26990.92	29653.84	28385.64	25882.89	**27728.31982**	**1.0991**	**0.2006**
Q61595	Ktn1	Kinectin	18801.81836	19876.76	16363.04	16867.85	**17977.36621**	16363.69	17156.25	16807.83	15100.36	**16357.03198**	**1.0991**	**0.1346**
Q9CQC6	Bzw1	eIF5-mimic protein 2	57362.97266	48587.89	37851.22	36796.76	**45149.70996**	44659.5	45403.25	38347.21	35954.34	**41091.07715**	**1.0988**	**0.4803**
O70572	Smpd2	Sphingomyelin phosphodiesterase 2		4724.428			**4724.427734**		3862.152	4737.452		**4299.801636**	**1.0988**	
Q8CE96	Trmt6	tRNA (adenine(58)-N(1))-methyltransferase non-catalytic subunit TRM6	6442.808105	4905.671	7352.781		**6233.753255**	3889.573	4560.831	6887.842	7360.187	**5674.607971**	**1.0985**	**0.6538**
Q99PL6	Ubxn6	UBX domain-containing protein 6	69224.08594	71093.56	71407.34	74082.46	**71451.86133**	69816.95	65741.66	58538.76	66078.27	**65043.90918**	**1.0985**	**0.0464**
Q8JZR0	Acsl5	Long-chain-fatty-acid--CoA ligase 5	20799.80469	18292.14	12680.39	12963.56	**16183.97095**	14999.51	13932.09	15300.71	14702.29	**14733.65015**	**1.0984**	**0.5017**
Q810A3	Ttc9c	Tetratricopeptide repeat protein 9C	10769.55566	6743.308	6023.463	5957.844	**7373.542603**		7505.922	6425.251	6208.713	**6713.29541**	**1.0983**	**0.6563**
Q9DBL7	Coasy	Bifunctional coenzyme A synthase	53637.01563	52928.23	49276.33	53729.7	**52392.81738**	49252.46	51497.35	43380.84	46696.86	**47706.87598**	**1.0982**	**0.0611**
Q8BYN5	Fsd1l	FSD1-like protein	19431.35352	23136.55	21981.3	22972.91	**21880.5293**	20931.64	17937.98	22218.94	18608.12	**19924.16992**	**1.0982**	**0.1873**
Q80WS3	Fbll1	rRNA/tRNA 2'-O-methyltransferase fibrillarin-like protein 1	75731.76563	69751.66	62170.32	82289.08	**72485.7041**	73262.86	71567.38	60832.91	58390.21	**66013.33789**	**1.0980**	**0.2989**
Q6TCG5	Paqr6	Membrane progestin receptor delta		4258.935	4138.547	3360.134	**3919.205404**				3569.934	**3569.934082**	**1.0978**	
Q9CQR2	Rps21	Small ribosomal subunit protein eS21	120723.9297	109946.1	107451.9	113707.2	**112957.2773**	115447.1	105163.5	96902.34	94118.89	**102907.9648**	**1.0977**	**0.1227**
Q9D1N9	Mrpl21	Large ribosomal subunit protein bL21m	15708.51367	12109.42	14012.58	16975.28	**14701.44824**	11886.8	14330.56	11906.01	15454.93	**13394.5769**	**1.0976**	**0.3815**
Q8K0Z7	Taco1	Translational activator of cytochrome c oxidase 1	46872.81641	37952.06	48778.51	44373.36	**44494.18652**	40183.81	38988.84	42743.55	40246.36	**40540.63965**	**1.0975**	**0.1632**
Q2NL51	Gsk3a	Glycogen synthase kinase-3 alpha	38659.16406	40060.36	41052.11	48533.42	**42076.26074**	28253.06	43453.26	40283.91	41401.23	**38347.86328**	**1.0972**	**0.3958**
P81117	Nucb2	Nucleobindin-2	9685.331055	5349.772	10276.49	6748.191	**8014.945801**	7113.129	6730.384	8012.835	7363.759	**7305.026855**	**1.0972**	**0.5779**
P37040	Por	NADPH--cytochrome P450 reductase	150644.9219	138848.2	159800.8	157835.3	**151782.2813**	142437.7	134308.8	142484.5	134145.1	**138344.0313**	**1.0971**	**0.0444**
Q9WTL7	Lypla2	Acyl-protein thioesterase 2	41574.91016	45993.41	49601.17	50660.71	**46957.55078**	38679.37	45075.76	40808.76	46661.59	**42806.37012**	**1.0970**	**0.1837**

Q99M51	Nck1	Cytoplasmic protein NCK1	10441.08789	13252.76	14422.5	9163.006	**11819.83862**	12387.32	7597.643	12694.6	10420.22	**10774.94653**	1.0970	0.5592
Q91YM4	Tbrg4	FAST kinase domain-containing protein 4	18085.66992	13939.07	16415.16	18187.9	**16656.94971**	15263.98	16826.86	13221.62	15428.16	**15185.15479**	1.0969	0.2801
Q9D115	Znf706	Zinc finger protein 706		30669.85		20381.19	**25525.51855**	25116.98	24515.3		20184.41	**23272.22982**	1.0968	0.6386
P63168	Dynll1	Dynein light chain 1, cytoplasmic	392968.125	322243.3	473356.3	273374.8	**365485.6406**	376989.3	356371.7	195286.9	404328.1	**333243.9844**	1.0968	0.6328
P18572	Bsg	Basigin	143780.2344	135883.1	133075	142809.2	**138886.8672**	132579.8	131306.7	132765.3	109964.3	**126654.0195**	1.0966	0.0941
Q9D1I2	Card19	Caspase recruitment domain-containing protein 19	5916.953613	4396.7		5315.447	**5209.700195**	4252.537	3279.052		6721.662	**4751.083577**	1.0965	0.7021
Q8K4I3	Arhgef6	Rho guanine nucleotide exchange factor 6	41873.26172	40818.75	36075.32	41735.15	**40125.61914**	36493.08	37218.43	36787.06	35876.03	**36593.64746**	1.0965	0.0450
Q9DCT5	Sdf2	Stromal cell-derived factor 2	3890.132324	7332.095	5242.655	4765.989	**5307.717773**	4222.059	5696.93	2608.261	6835.739	**4840.747375**	1.0965	0.7041
Q61609	Slc20a1	Sodium-dependent phosphate transporter 1	28884.4375	33670.54	35765.12	32034.19	**32588.57275**	29339.74	32886.94	30413.23	26276.04	**29728.98926**	1.0962	0.2019
Q9D8S4	Rexo2	Oligoribonuclease, mitochondrial	30671.18945	28638.64	35646.6	22540.21	**29374.15869**	25593.83	23812.83	27906.22	29887.22	**26800.02588**	1.0960	0.4266
Q6P9Z1	Smarcd3	SWI/SNF-related matrix-associated actin-dependent regulator of chromatin	5762.768555				**5762.768555**	4710.629	6617.079	4447.448		**5258.385417**	1.0959	
Q5U3K5	Rabl6	Rab-like protein 6	50857.33594	52420.53	49747.98	58909.24	**52983.77246**	46863.38	48938.42	50242.89	47369.5	**48353.54785**	1.0958	0.0788
P61027	Rab10	Ras-related protein Rab-10	500489.4688	465652.7	483254	494440.6	**485959.1797**	443386.6	455907.5	430785.3	443988.3	**443516.9219**	1.0957	0.0037
P97470	Ppp4c	Serine/threonine-protein phosphatase 4 catalytic subunit	14414.40332	12528.05	10776.14	9114.269	**11708.21533**	13469.85	16935.53	4875.679	7477.834	**10689.724**	1.0953	0.7440
P26231	Ctnna1	Catenin alpha-1	32355.66211	31090.01	26401.98	36051.54	**31474.79688**	34735.14	32989.67	23355.35	23870.94	**28737.77734**	1.0952	0.4742
Q91WU5	As3mt	Arsenite methyltransferase	17178.97852	15962.17	16313.18	13047.28	**15625.40137**	15247.3	15922.51	12653.05	13251.77	**14268.65601**	1.0951	0.2976
Q9DC51	Gnai3	Guanine nucleotide-binding protein G(i) subunit alpha-3	42878.5625	35806.79	48594.03	54519.2	**45449.64551**	40710.11	48159.89	43667.91	33480.42	**41504.58105**	1.0951	0.4642
Q99NE5	Rims1	Regulating synaptic membrane exocytosis protein 1	17073.66602	23180.5	25398.48	21512.15	**21791.19971**	17299.58	19332	25275.08	17695.53	**19900.54639**	1.0950	0.4866
P19246	Nefh	Neurofilament heavy polypeptide	1893851.125	2018299	2615046	1920640	**2111958.719**	1830813	1820469	2017256	2047344	**1928970.531**	1.0949	0.3488
P21995	Emb	Embigin	32015.02734	37424.37	26001.69	32795.8	**32059.21924**	35536.17	26592.86	28294.62	26710.36	**29283.50488**	1.0948	0.4138
P56818	Bace1	Beta-secretase 1	19569.42969	16115.04			**17842.23486**	21895.75		15060.17	11942	**16299.30664**	1.0947	0.7259
Q9ESE1	Lrba	Lipopolysaccharide-responsive and beige-like anchor protein	15090.82422	19920.04	14095.66	15457.51	**16141.00708**	16046.68	17260.05	9598.801	16075.33	**14745.21533**	1.0947	0.5432
Q80W47	Wipi2	WD repeat domain phosphoinositide-interacting protein 2	34582.1875	34830.89	36729.21	43938.28	**37520.14355**	35809.41	31154.33	34779	35419.75	**34290.62354**	1.0942	0.2335
O88502	Pde8a	High affinity cAMP-specific and IBMX-insensitive 3',5'-cyclic phosphodiesterase	13441.25879	14768.78	12376.38	11779.09	**13091.37695**	11390.18	12502.42	12500.42	11468.38	**11965.34912**	1.0941	0.1718
P70227	Itpr3	Inositol 1,4,5-trisphosphate receptor type 3	136609	9244.893	6799.208	2016.784	**38667.47107**	975.9396	127383.9	2122.527	10892.07	**35343.60869**	1.0940	0.9434
P34152	Ptk2	Focal adhesion kinase 1	16433.97656	18136.86	14913.13	12743.26	**15556.80859**	14414.96	15338.96	13868.85	13257.29	**14220.01538**	1.0940	0.3181
Q3UN02	Lclat1	Lysocardiolipin acyltransferase 1	10042.30762	7903.139	8769.404	4720.005	**7858.714111**	7500.096	7646.947	4665.431	8925.911	**7184.596191**	1.0938	0.6578
Q99P31	Hspbp1	Hsp70-binding protein 1	16299.57129	19418.67	16787.91	18287.29	**17698.3606**	15691.87	16964.26	14534.94	17529.96	**16180.25562**	1.0938	0.1715
Q60857	Slc6a4	Sodium-dependent serotonin transporter	22468.04492	23735.74	25207.41	33251.03	**26165.55664**	21055.22	25089.41	24416.96	25130.6	**23923.05127**	1.0937	0.4239
P33175	Kif5a	Kinesin heavy chain isoform 5A	202827.3906	168991.4	212238.9	256780.4	**210209.5234**	234511	211007.7	148869.5	174449.5	**192209.4258**	1.0936	0.5184
Q7TPV4	Mybbp1a	Myb-binding protein 1A	6427.59082	6721.711	5834.226	10203.87	**7296.849731**	7770.166	5397.087	6912.771	6615.187	**6673.80249**	1.0934	0.5923
Q06335	Aplp2	Amyloid beta precursor like protein 2	33441.13281	30698.92	26130.84	21207.84	**27869.68408**	26053.11	27964.53	23142.81	24803.43	**25490.97119**	1.0933	0.4390
Q9DCD6	Gabarap	Gamma-aminobutyric acid receptor-associated protein	19307.78516	19505.37	14571.79	19275.94	**18165.22046**	18698.33	17771.84	13571.64	16419.87	**16615.42065**	1.0933	0.3808
O70503	Hsd17b12	Very-long-chain 3-oxoacyl-CoA reductase	23139.21484	23425.98	24311.95	21779.43	**23164.14404**	22335.14	24881.35	17818.77	19738.17	**21193.35742**	1.0930	0.2710
Q61016	Gng7	Guanine nucleotide-binding protein G(I)/G(S)/G(O) subunit gamma-7	73787.47656	81124.23	72818.42	85351.88	**78270.50195**	78292.96	72455.39	60459.45	75265.48	**71618.31934**	1.0929	0.2256
Q9D4F8	Tubgcp4	Gamma-tubulin complex component 4	51958.65234	30904.62	28216.08	31030.84	**35527.54785**	32361.54	33727.41	31310.79	32648.82	**32512.13623**	1.0927	0.6057
Q91W92	Cdc42ep1	Cdc42 effector protein 1	27355.91797	17922.62	24029.42	22054.5	**22840.61475**	23274.4	19714.62	19991.44	20631.21	**20902.91846**	1.0927	0.3985
P40237	Cd82	CD82 antigen	105898.3594	113026.8	112648.8	107469.2	**109760.7813**	101592.2	92443.62	97498.45	110271.4	**100451.4082**	1.0927	0.0676
Q9R1L5	Mast1	Microtubule-associated serine/threonine-protein kinase 1	13323.58301	12590.7	13740.68	11960.18	**12903.78613**	9559.524	12473.92	12373.71	12834.7	**11810.46436**	1.0926	0.2474
Q9QZD9	Eif3i	Eukaryotic translation initiation factor 3 subunit I	34371.07031	30329.14	33858.78	31268.98	**32456.99268**	31919.33	29775.03	27624.02	29510.09	**29707.11621**	1.0926	0.0820
O35454	Clcn6	H(+)/Cl(-) exchange transporter 6	19825.42188	19092.69	20176.54	23864.71	**20739.83887**	19647.88	19462.01	18978.69	17851.06	**18984.91309**	1.0924	0.1745
Q8VD57	Sft2d2	Vesicle transport protein SFT2B	9195.098633	11953.03	10643.83	8737.849	**10132.45215**	10772.83	7705.041	9039.009	9584.278	**9275.288208**	1.0924	0.4103
Q62083	Pick1	PRKCA-binding protein	30270.58594	35549.24	29304.35	26487.58	**30402.93848**	30978.84	26764.4	26824.36	26763.92	**27832.88232**	1.0923	0.2800
Q8CHK3	Mboat7	Lysophospholipid acyltransferase 7	7786.112305	8771.336	9252.271	8631.973	**8610.422852**	8290.047	9388.016	6702.333	7161.48	**7885.468994**	1.0919	0.3239
Q9CQW2	Arl8b	ADP-ribosylation factor-like protein 8B	66443.9375	62362.05	70173.55	61857.21	**65209.1875**	63220.61	61540.69	60467.27	53696.21	**59731.19531**	1.0917	0.1036
P35279	Rab6a	Ras-related protein Rab-6A	373772	348818.5	359573.8	369388.2	**362888.1172**	352903.4	329081.9	316113.4	331538.2	**332409.2344**	1.0917	0.0179
Q8BH24	Tm9sf4	Transmembrane 9 superfamily member 4	27241.67969	21822.48	28516.71	29404.64	**26746.37842**	22419.8	29754.59	21159.77	24672.76	**24501.73096**	1.0916	0.4120
Q9CPZ8	Cmc1	COX assembly mitochondrial protein homolog	95261.13281	92264.73	95619.8	103591.6	**96684.33008**	89764.84	91568.84	86281.49	86695.59	**88577.69336**	1.0915	0.0250
Q5U430	Ubr3	E3 ubiquitin-protein ligase UBR3	17208.13281	24931.62	20536.28	21625.41	**21075.36035**	20912.96	19289.81	18725.25	18305.5	**19308.37744**	1.0915	0.3365
Q8BWU5	Osgep	tRNA N6-adenosine threonylcarbamoyltransferase		3076.718	4690.789	7985.125	**5250.877116**	4292.585	5459.409	2216.918	7274.172	**4810.771118**	1.0915	0.8106
Q9ER35	Fn3k	Fructosamine-3-kinase	50074.23047	41112.37	47201.71	44216.62	**45651.2334**	41953.06	41178.84	47010.56	37166.25	**41827.17871**	1.0914	0.2201
P49962	Srp9	Signal recognition particle 9 kDa protein	22549.76953	24875.71	17393.57	17544.43	**20590.87109**	22946.85	20796.77	18138.94	13586.71	**18867.31787**	1.0914	0.5533
Q60854	Serpinb6	Serpin B6	107814.3828	109177.4	143763.9	126521.2	**121819.207**	107873.3	102865.1	109926.7	125838.7	**111625.9629**	1.0913	0.3389
Q9EQK5	Mvp	Major vault protein	22715.78125	22307.83	34693.03	22736.41	**25613.26123**	22012	22522.58	24562.91	24784.72	**23470.5542**	1.0913	0.5164
Q9WUD1	Stub1	E3 ubiquitin-protein ligase CHIP	62761.99219	57528.07	60185.3	62439.65	**60728.75195**	56735.5	60574.91	54531.93	50759.47	**55650.45313**	1.0913	0.0772
P26041	Msn	Moesin	127661.0625	121655.7	130845.2	119540.8	**124925.6797**	121520.2	109970.6	109782.9	116706.8	**114495.1328**	1.0911	0.0356
Q9D0T1	Snu13	NHP2-like protein 1	39603.70313	47754.6	42416.08	41168.94	**42735.8291**	39031.22	31431.87	41658.9	44549.67	**39167.91699**	1.0911	0.3244
Q61792	Lasp1	LIM and SH3 domain protein 1	104405.2969	125779.9	96270.98	132225.8	**114670.4941**	100607.7	120234.2	135530.2	64018.02	**105097.5176**	1.0911	0.6071
Q99K48	Nono	Non-POU domain-containing octamer-binding protein	51296.16016	43768.33	45754.87	46360.91	**46795.06836**	45592.59	49728.67	35500.77	40789.82	**42902.96289**	1.0907	0.3038
Q5KU39	Vps41	Vacuolar protein sorting-associated protein 41 homolog	17082.60547	16582.61	18747.27	16328.01	**17185.12549**	16862.62	16894.28	15899.57	13380.7	**15759.29272**	1.0905	0.1994
Q8R016	Blmh	Bleomycin hydrolase	87352.69531	99766.21	90634.03	90669.68	**92105.6543**	82058.09	88493.05	83180.22	84129.94	**84465.32422**	1.0905	0.0446
Q91VF2	Hnmt	Histamine N-methyltransferase	28798.42773	27721.62	22675.06	28261.68	**26864.1958**	26099.68	26552.31	23975.85	21936.2	**24641.01123**	1.0902	0.2554
P54227	Stmn1	Stathmin	394495.1875	412471.4	320333.9	358026.1	**371331.625**	349683.8	368681.8	321225	322882.7	**340618.3125**	1.0902	0.2372
Q8VE88	Fam114a2	Protein FAM114A2	7179.813477	8522.04	6439.819	6030.929	**7043.150269**	6861.003	5159.75	6224.306	7601.619	**6461.669556**	1.0900	0.4693
Q5DTL9	Slc4a10	Sodium-driven chloride bicarbonate exchanger	11492.53809	10923.99	10789.43	13442.13	**11662.02051**	10615.02	12201.85	11445.27	8537.571	**10699.92773**	1.0899	0.3731

P26043	Rdx	Radixin	474304.25	453530.1	511060.3	457908.6	474200.8125	442658.7	458951.5	397289.2	441563.7	435115.7656	1.0898	0.0803
Q9D1C8	Vps28	Vacuolar protein sorting-associated protein 28 homolog	24106.07813	22247.08	22473.88	20648.51	22368.88867	22033.43	19324.5	20545.57	20204.08	20526.89648	1.0897	0.0879
Q8BYI9	Tnr	Tenascin-R	412772.7813	405826.6	366580.3	524178	427339.4219	414992.8	397942.8	366075.1	389778.2	392197.2344	1.0896	0.3584
Q9DB70	Fundc1	FUN14 domain-containing protein 1	8653.860352	7708.749	2700.794	2916.724	5495.031616	4751.73	4991.018	6110.967	4319.203	5043.229614	1.0896	0.7884
B2RSH2	Gnai1	Guanine nucleotide-binding protein G(i) subunit alpha-1	229377.4531	224926.2	261369.5	229175.9	236212.2695	195398.2	184591.6	250077.8	237184.6	216813.0508	1.0895	0.3218
Q64343	Abcg1	ATP-binding cassette sub-family G member 1	10452.97754	7908.012	7262.983	9132.059	8689.00769	7007.855	7870.653	8452.449	8570.756	7975.428345	1.0895	0.4009
Q9R0P4	Smap	Small acidic protein	13623.26172	7630.538	4158.639	11463.43	9218.968384	8390.548	10489.52	8981.713	5986.458	8462.05957	1.0894	0.7525
P40124	Cap1	Adenylyl cyclase-associated protein 1	175448.9531	176514.3	213738.6	193782.8	189871.1563	163928.9	189559.8	169949.7	173757.5	174298.9688	1.0893	0.1897
P03921	Mtnd5	NADH-ubiquinone oxidoreductase chain 5	58411.56641	50675.77	48106.93	52706.18	52475.1123	43314.28	48814.61	53129.29	47455.2	48178.34277	1.0892	0.1997
Q9CQF3	Nudt21	Cleavage and polyadenylation specificity factor subunit 5	54627.375	53387.48	67112.82	58417.67	58386.33594	55887.5	54179.83	54265.07	50114.64	53611.76074	1.0891	0.2022
P62746	Rhob	Rho-related GTP-binding protein RhoB	159032.1094	127132.8	156456.7	148880.3	147875.4629	134041.5	138822	129008.8	141323.4	135798.9102	1.0889	0.1695
Q8C7K6	Pcyox1l	Prenylcysteine oxidase-like	38529.04297	40417.84	40205.21	32924.16	38019.06055	37759.34	34655.81	36080.73	31184.46	34920.0835	1.0887	0.2157
Q9D2V7	Coro7	Coronin-7	16588.63867	17181.73	15534.64	14408.12	15928.2832	15052.83	14972.81	12349.18	16145.13	14629.98999	1.0887	0.2465
Q6KAU4	Mvb12b	Multivesicular body subunit 12B	14227.52637	9361.998	10161.25	3623.904	9343.670654	11370.19	7917.392	7531.524	7514.614	8583.429688	1.0886	0.7598
P59325	Eif5	Eukaryotic translation initiation factor 5	118172.5391	123099.6	129511.9	135358.2	126535.5605	105558.2	114582	114758.7	130089.2	116247.0215	1.0885	0.1547
Q8K2D3	Edc3	Enhancer of mRNA-decapping protein 3	10753.88867	15476.58	10651.28	16969.09	13462.70972	9887.111	14579.3	9623.084	15388.81	12369.57861	1.0884	0.6404
Q3UVG3	Fam91a1	Protein FAM91A1	11160.91211	11124.31	9830.995	10581.05	10674.3186	10007.5	12207.41	7024.707	9992.615	9808.056274	1.0883	0.4641
Q921C1	Gjc3	Gap junction gamma-3 protein	20277.0293	17158.81	22733.58	15998.96	19042.09692	19067.03	21917.74	15251.97	13754.48	17497.80566	1.0883	0.5434
P27600	Gna12	Guanine nucleotide-binding protein subunit alpha-12	17915.5332	19014.77	17697.74	19575.15	18550.79883	17837.82	17141.37	15647.92	17567.95	17048.76099	1.0881	0.0638
Q8BMK4	Ckap4	Cytoskeleton-associated protein 4	20044.42969	19151.57	24042.48	20715.67	20988.53662	20422.55	20707.1	17773.73	18255.29	19289.66943	1.0881	0.2396
Q9D0S9	Hint2	Adenosine 5'-monophosphoramidase HINT2	74738.47656	70828.38	97040.91	90179.58	83196.83594	65284.95	76185.04	94851.81	69543.48	76466.31934	1.0880	0.4837
Q9QXY6	Ehd3	EH domain-containing protein 3	132881.9531	133855.6	146705.7	138344.4	137946.8906	128872.7	130940.4	122807.4	124532.1	126788.1504	1.0880	0.0228
Q9R0M6	Rab9a	Ras-related protein Rab-9A	25690.67578	26599.6	26795.24	28424.09	26877.40137	30836.55	23063.54	20826.16	24088.49	24703.68604	1.0880	0.3670
Q3TYD4	Arsg	Arylsulfatase G	19599.07617	21189.68	17858.6	18670.35	19329.42676	15527.13	19748.57	19965.59	15830.39	17767.91992	1.0879	0.3087
Q99KC8	Vwa5a	von Willebrand factor A domain-containing protein 5A	99771.19531	109463.3	131302.2	126330.1	116716.7031	103250.9	105461.4	103489.8	116991	107298.25	1.0878	0.2851
Q8BI84	Mia3	Transport and Golgi organization protein 1 homolog	6963.025879	6567.994	8078.014	8846.039	7613.768311	6478.527	7269.085	6942.211	7308.783	6999.651611	1.0877	0.3108
Q9CXI5	Manf	Mesencephalic astrocyte-derived neurotrophic factor	22341.75781	21477.12	17090.29	17816.64	19681.45166	17933.04	22269.82	14709.68	17470.24	18095.69556	1.0876	0.4659
Q91YH5	Atl3	Atlastin-3	30340.35938	31032.46	37372.03	28349.61	31773.61475	30796.75	28593.11	28224.93	29247.29	29215.52197	1.0876	0.2548
Q8BGT6	Micall1	MICAL-like protein 1	17653.19141	18009.95	13795.44	8721.685	14545.06665	14287.29	15296.37	9606.769	14309.5	13374.98267	1.0875	0.6578
Q80X95	Rraga	Ras-related GTP-binding protein A	6373.362793	6669.789	5374.386	8617.819	6758.839233	6397.068	5809.724	6422.866	6245.941	6218.900024	1.0868	0.4658
Q9D8X2	Ccdc124	Coiled-coil domain-containing protein 124	20152.01563	18234.47	25016.11	20122.32	20881.23047	19961.61	18749.82	20536.91	17607.93	19214.06592	1.0868	0.3346
O35326	Srsf5	Serine/arginine-rich splicing factor 5	22108.17773	21111.45	20632.67	23788.46	21910.19141	18930.46	21687.39	23134.4	16900.22	20163.11768	1.0866	0.3052
Q9JI46	Nudt3	Diphosphoinositol polyphosphate phosphohydrolase 1	134892.5156	148058.3	134345.7	138389.7	138921.5508	142431	123522	117736	127704.5	127848.3984	1.0866	0.1221
P62342	Selenot	Thioredoxin reductase-like selenoprotein T	23898.29102	25706.1	21708.53	21266.22	23144.78662	23960.45	24635.56	21402.19	15203.16	21300.33911	1.0866	0.4682
Q9R1J0	Nsdhl	Sterol-4-alpha-carboxylate 3-dehydrogenase, decarboxylating	10304.14844	15732.18	11557.93	10290.33	11971.14771	9838.535	11357.8	10852.63	12027.72	11019.16992	1.0864	0.5127
Q9CY58	Serbp1	SERPINE1 mRNA-binding protein 1	29773.37695	33377.06	37515.81	31399.64	33016.47363	34808.73	28914.33	24558.25	33284.61	30391.47998	1.0864	0.3928
Q9JMD0	Znf207	BUB3-interacting and GLEBS motif-containing protein ZNF207		10499.52		6573.699	8536.609863		7859.278			7859.27832	1.0862	
P03888	Mtnd1	NADH-ubiquinone oxidoreductase chain 1	225996.5313	219248.3	248719.5	284733.5	244674.4336	235158.9	219024.8	237150.4	209813.8	225286.9805	1.0861	0.2754
Q8BVG4	Dpp9	Dipeptidyl peptidase 9	25648.4043	26915.13	19056.41	25109.15	24182.271	24282.86	25340.37	19079.79	20372.45	22268.86621	1.0859	0.4391
Q6P9R2	Oxsr1	Serine/threonine-protein kinase OSR1	54857.27344	55142.07	64593.79	56788.74	57845.46973	56890.19	51169.99	54517.79	50513.68	53272.91113	1.0858	0.1452
Q80ZI6	Lrsam1	E3 ubiquitin-protein ligase LRSAM1	38357.69922	34230.36	53914.88	51561.07	44516	35942.76	41204.2	43823.32	43022.15	40998.10742	1.0858	0.5207
Q8BVY0	Rsl1d1	Ribosomal L1 domain-containing protein 1	8163.257324	6839.758	4461.24	3830.146	5823.60022	6671.237	6531.475	3883.199	4368.976	5363.721802	1.0857	0.7245
Q9ESL4	Map3k20	Mitogen-activated protein kinase kinase kinase 20	14798.30176	12421.18	12822.52	12215.63	13064.4082	12085.31	13816.05	11583.42	10648.56	12033.33423	1.0857	0.2906
Q9DCU2	Pllp	Plasmolipin	167058.75	238452.7	222118.5	330680.3	239577.5586	359070.3	127653	245949.4	150017.7	220672.5977	1.0857	0.7735
Q9Z2D0	Mtmr9	Myotubularin-related protein 9	30546.79688	27310.21	30062.5	26079.33	28499.71094	25914.03	26859.68	24328.25	27919.47	26255.35742	1.0855	0.1397
Q9JI39	Abcb10	ATP-binding cassette sub-family B member 10, mitochondrial	11290.64941	10348.56	10800.68	11289.29	10932.29346	10900.89	11194.88	8502.135	9689.622	10071.88232	1.0854	0.2379
E0CYV9		Uncharacterized protein C4orf54 homolog			67619.11	66116.83	66867.96875		19037.69	85482.35	80386.55	61635.53255	1.0849	0.8616
Q8QZZ7	Tprkb	EKC/KEOPS complex subunit Tprkb	10278.20996	12738.67	11711.7	9322.909	11012.8728	12229.56	10162.4	8798.032	9416.752	10151.68457	1.0848	0.4487
Q8CHW4	Eif2b5	Translation initiation factor eIF-2B subunit epsilon	21657.81836	24086	22695.86	17200.48	21410.04102	20068.49	18998.39	20927.33	18966.37	19740.14404	1.0846	0.3260
Q9WUQ2	Preb	Prolactin regulatory element-binding protein	69239.54688	68492.45	72614.73	77563.45	71977.54492	68141.23	73102.33	58510.95	65728.35	66370.7168	1.0845	0.1777
P28659	Celf1	CUGBP Elav-like family member 1	12624.44922	15968.63	13723.98	15346.28	14415.8335	16980.29	14128.27	10441.56	11625.83	13293.9895	1.0844	0.5188
Q8BG32	Psmd11	26S proteasome non-ATPase regulatory subunit 11	99238.17969	93242.91	100559.6	100469.1	98377.4375	90263.95	99159.04	82520.41	90977.13	90730.13086	1.0843	0.0921
O88738	Birc6	Baculoviral IAP repeat-containing protein 6	19494.06445	20581.29	16654.49	20789.84	19379.92041	15026.98	19675.21	19583.97	17220.96	17876.78027	1.0841	0.3429
Q91UZ5	Impa2	Inositol monophosphatase 2	6971.019043		13514.11		10242.5647	1737.591		12203.85	14404.15	9448.531413	1.0840	0.8963
Q9JHJ0	Tmod3	Tropomodulin-3		6233.767	10660.51		8447.140625	8574.515	7049.271		7753.461	7792.415527	1.0840	0.7325
P61294	Rab6b	Ras-related protein Rab-6B	83576.21094	84722.93	88784.84	88573.54	86414.37891	82128.27	80984.43	74394.26	81360.41	79716.8418	1.0840	0.0239
Q5SXY1	Specc1	Cytospin-B	42654.80469	41423.81	37780.25	50022.24	42970.27734	41305.31	38547.3	38465.8	40243.82	39640.55859	1.0840	0.2571
Q8VE62	Paip1	Polyadenylate-binding protein-interacting protein 1	32167.2793	34735.06	29401.14	31706.24	32002.4292	28980.92	29328.66	29016.78	30775.97	29525.58203	1.0839	0.0792
Q8BK26	Fbxo44	F-box only protein 44		5171.518	4941.144	3954.288	4688.983154		5003.687	3649.309		4326.497925	1.0838	0.6394
Q91YI0	Asl	Argininosuccinate lyase	38575.05469	36846.68	40125.82	38831.87	38594.85645	31593.72	37954.68	38062.35	34848.07	35614.70703	1.0837	0.1256
Q6PDI6	Mindy2	Ubiquitin carboxyl-terminal hydrolase MINDY-2	5324.028809		2793.795	6221.037	4779.620199	4425.507	4308.312	4693.474	4217.577	4411.217407	1.0835	0.6886
P17897	Lyz1	Lysozyme C-1	17442.51563	28386.34	59861.89	27548.11	33309.71436	25711.24	33356.12	43621.07	20285.52	30743.48975	1.0835	0.8150
Q6ZQA6	Igsf3	Immunoglobulin superfamily member 3	2041.432373	5064.972		4691.546	3932.650146	5394.105	1938.195	5015.932	2170.686	3629.729614	1.0835	0.8305
Q02111	Prkcq	Protein kinase C theta type	5401.52002		4623.968	6302.508	5442.665365	3166.837		6868.401	5037.841	5024.359782	1.0833	0.7395
Q61282	Acan	Aggrecan core protein	59903.98047	56991.4	73988.38	77529.06	67103.2041	51964.75	48699.44	74118.92	73009.45	61948.14258	1.0832	0.5640

Q9D1J3	Sarnp	SAP domain-containing ribonucleoprotein	36274.23828	33350.46	36105.24	40355.16	**36521.27539**	30921.95	33763.06	32555.52	37626.28	**33716.70117**	**1.0832**	**0.2163**
Q9D0F3	Lman1	Protein ERGIC-53	27731.97266	26405.16	26138.12	28111.66	**27096.72998**	28156.24	22743.4	21898.49	27278.8	**25019.2334**	**1.0830**	**0.2549**
Q6PIU9		Uncharacterized protein FLJ45252 homolog	17056.14453	18638.43	20929.04	16260.9	**18221.12891**	16551.67	15715.31	16635.65	18395.8	**16824.60938**	**1.0830**	**0.2789**
Q6NS60	Fbxo41	F-box only protein 41	28347.68164	26674.32	38632.01	40655.37	**33577.34375**	26453.35	22687.9	36919.28	37956.34	**31004.2168**	**1.0830**	**0.6380**
Q62086	Pon2	Serum paraoxonase/arylesterase 2	20300.35352	22163.98	24773.66	17817.21	**21263.80127**	24161.93	22393.01	13699.43	18290.68	**19636.26489**	**1.0829**	**0.5762**
Q3UYG8	Macrod2	ADP-ribose glycohydrolase MACROD2	8300.561523	7250.485	6885.642	6941.962	**7344.662598**	5385.851	6536.605	7728.891	7479.261	**6782.652222**	**1.0829**	**0.4032**
Q91ZP3	Lpin1	Phosphatidate phosphatase LPIN1	11179.6875	12151.12	14609.02	13370.06	**12827.46948**	11198.57	11399.54	11617.54	13168.57	**11846.05493**	**1.0828**	**0.3018**
P25444	Rps2	Small ribosomal subunit protein uS5	239660.0313	251034.2	228991.1	254167.7	**243463.2422**	242666.5	236316.4	208107.5	212268.3	**224839.6719**	**1.0828**	**0.1218**
Q99J47	Dhrs7b	Dehydrogenase/reductase SDR family member 7B	57155.00391	48728.4	49978.48	50501.68	**51590.8916**	54407.35	53583.57	41266.19	41329.7	**47646.70117**	**1.0828**	**0.3763**
Q61189	Clns1a	Methylosome subunit pICln	15950.45898	15901.6	15027.09	16106.19	**15746.33472**	15423.12	15820.28	10987.9	15938.88	**14542.54272**	**1.0828**	**0.3599**
Q8BLF1	Nceh1	Neutral cholesterol ester hydrolase 1	59323.92188	52029.32	62804.64	69262.17	**60855.01367**	59365.88	52736.47	57214.86	55498.07	**56203.81738**	**1.0828**	**0.2729**
Q9CQU0	Txndc12	Thioredoxin domain-containing protein 12	49987.82031	48058.25	53919.48	49824.8	**50447.58887**	49243.98	46853.46	44434.02	45862.83	**46598.57324**	**1.0826**	**0.0527**
O08585	Clta	Clathrin light chain A	210768.2969	170985.8	213565	173370.2	**192172.3164**	185055.9	183981.3	195332.4	145757.1	**177531.6914**	**1.0825**	**0.3924**
Q91XU3	Pip4k2c	Phosphatidylinositol 5-phosphate 4-kinase type-2 gamma	18093.20898	19064.41	23420.3	20333.21	**20227.78174**	17982.03	17799.22	16897.14	22083.71	**18690.52637**	**1.0822**	**0.3838**
O08638	Myh11	Myosin-11	139543.9688	135291.8	158129.4	136794.3	**142439.8672**	152082.2	141533.9	123736.9	109106.6	**131614.8945**	**1.0822**	**0.3587**
Q9CRB6	Tppp3	Tubulin polymerization-promoting protein family member 3	1144676.375	1124890	1297931	1299068	**1216641.156**	1150884	1024958	1089135	1232677	**1124413.406**	**1.0820**	**0.2052**
Q3TPE9	Ankmy2	Ankyrin repeat and MYND domain-containing protein 2	15914.37109	13189.74	6378.346	11287.96	**11692.60535**	10511.27	12524.16	9287.54	10902.53	**10806.37427**	**1.0820**	**0.6902**
Q6PDG5	Smarcc2	SWI/SNF complex subunit SMARCC2	14018.14453	19988.59	23241.43	18557	**18951.29199**	16430.1	16434.41	19044.08	18151.47	**17515.0166**	**1.0820**	**0.5042**
Q3UTZ3	Trappc14	Trafficking protein particle complex subunit 14	13606.79297	7858.809	13251.18	13460.13	**12044.22998**	9866.089	12219.53	9700.979	12748.12	**11133.6792**	**1.0818**	**0.5908**
Q9D6M3	Slc25a22	Mitochondrial glutamate carrier 1	170853.9531	181132.3	168265.8	186920.8	**176793.2227**	176876.6	171779.8	156565.7	148504.2	**163431.582**	**1.0818**	**0.1419**
Q9CQX2	Cyb5b	Cytochrome b5 type B	97915.22656	86322.9	101784.9	96769.22	**95698.04883**	87004.89	94990.2	84064.16	87806.35	**88466.40039**	**1.0817**	**0.1234**
P56387	Dynlt3	Dynein light chain Tctex-type 3	42655.30469	46868.21	39240.93	38512.62	**41819.26855**	44189.02	37920.64	34105.93	38422.1	**38659.42285**	**1.0817**	**0.3059**
Q9D517	Agpat3	1-acyl-sn-glycerol-3-phosphate acyltransferase gamma	49842.51953	50800.98	65538.5	53555.76	**54934.44043**	50267.59	47996.03	53057.5	51832.57	**50788.4209**	**1.0816**	**0.3150**
Q63844	Mapk3	Mitogen-activated protein kinase 3	347877.7188	338713.3	347462.9	329316.1	**340842.5**	320144.8	322054.5	299275.7	319094.7	**315142.4141**	**1.0816**	**0.0098**
P53395	Dbt	Lipoamide acyltransferase component of branched-chain alpha-keto acid c	53426.45313	57703.61	46319.38	60216	**54416.3623**	51323.6	52329.07	50284.7	47320.61	**50314.49609**	**1.0815**	**0.2508**
P49813	Tmod1	Tropomodulin-1	68717.85156	51941.92	67649.8	78721.43	**66757.75098**	52950.05	60539.88	71748	61675.71	**61728.40723**	**1.0815**	**0.4841**
Q91W59	Rbms1	RNA-binding motif, single-stranded-interacting protein 1	13204.68359	11169.01	13162.3	12320.32	**12464.0769**	10241.64	12568.1	12699.58	10590.99	**11525.07568**	**1.0815**	**0.2861**
Q9R1Q9	Atp6ap1	V-type proton ATPase subunit S1	44220.14063	54294	47936.03	63453.28	**52475.8623**	41734.01	48191.82	51782.32	52388.26	**48524.10156**	**1.0814**	**0.4479**
Q9R078	Prkab1	5'-AMP-activated protein kinase subunit beta-1	27829.77734	21757.93	20591.25	26255.3	**24108.56543**	16420.1	24365.58	23375.07	25015.88	**22294.15869**	**1.0814**	**0.5178**
Q9DBX3	Susd2	Sushi domain-containing protein 2	55168.83203	47349.58	83111.03	63234.6	**62216.01074**	53978.71	53414.81	64755.8	58025.96	**57543.82129**	**1.0812**	**0.5857**
Q99J09	Wdr77	Methylosome protein WDR77	33016.04688	37512.71	34342.51	35022.23	**34973.37305**	30696.92	33618.73	32017.74	33062.84	**32349.05859**	**1.0811**	**0.0612**
O88643	Pak1	Serine/threonine-protein kinase PAK 1	124242.4453	122322.6	122712.3	125184.6	**123615.4824**	102341.3	116382.8	114969.8	123669.4	**114340.8223**	**1.0811**	**0.0839**
P24369	Ppib	Peptidyl-prolyl cis-trans isomerase B	252877.5	259739.2	280857.2	269912.9	**265846.6992**	241619.7	260488.2	232869	248833.6	**245952.6328**	**1.0809**	**0.0567**
Q8K2L8	Trappc12	Trafficking protein particle complex subunit 12	9785.6875	11199.02	10107.66	10894.84	**10496.80029**	10469.57	10073.42	10873.34	7429.371	**9711.42334**	**1.0809**	**0.3886**
O89001	Cpd	Carboxypeptidase D	34964.10938	32343.92	31544.65	32533.6	**32846.56934**	27414.96	33921.26	29440.77	30783.57	**30390.14014**	**1.0808**	**0.1646**
Q8R2Y2	Mcam	Cell surface glycoprotein MUC18	60323.92969	73188.6	76753.7	67950.6	**69554.20703**	74300.83	62857.24	61622.55	58637.36	**64354.49316**	**1.0808**	**0.3340**
O88384	Vti1b	Vesicle transport through interaction with t-SNAREs homolog 1B	37302.69531	39748.91	29662.6	35683	**35599.29834**	31398.46	35282.97	33877.55	31199.84	**32939.70557**	**1.0807**	**0.3038**
P70232	Chl1	Neural cell adhesion molecule L1-like protein	38183.34375	36412.8	44378.73	35399.95	**38593.70605**	34200.29	37716.54	37488.82	33438.71	**35711.08789**	**1.0807**	**0.2558**
Q99KI3	Emc3	ER membrane protein complex subunit 3	40793.76563	40550.44	40082.64	43939.2	**41341.51172**	37241.41	39498.11	37191.73	39090.23	**38255.37012**	**1.0807**	**0.0276**
Q64442	Sord	Sorbitol dehydrogenase	63276.54688	63140.16	80208.23	77502.2	**71031.7832**	68676.89	55421.51	68268.37	70558.1	**65731.2168**	**1.0806**	**0.3902**
Q62470	Itga3	Integrin alpha-3	11011.96191	11230.53	13015.11	12550.27	**11951.96802**	11534.85	11140.94	10776.55	10792.63	**11061.24316**	**1.0805**	**0.1391**
Q61097	Ksr1	Kinase suppressor of Ras 1	15130.99609	14145.76	12718.63	17008.87	**14751.0647**	15554.97	14792.42	11381.23	12881.2	**13652.45435**	**1.0805**	**0.4319**
Q9Z0H4	Celf2	CUGBP Elav-like family member 2	18654.08594	22443.22	18869.91	23257.99	**20806.30273**	19536.87	23089.95	16490.41	17918.72	**19258.98779**	**1.0803**	**0.4361**
Q8K4X7	Agpat4	1-acyl-sn-glycerol-3-phosphate acyltransferase delta	103649.3672	109239.2	77252.12	85106.54	**93811.80664**	107268.2	100941.6	68077.02	71062.3	**86837.28125**	**1.0803**	**0.5996**
Q8K442	Abca8a	ABC-type organic anion transporter ABCA8A		24214.98	10379.83		**17297.40576**		25343.17	6683.975		**16013.57446**	**1.0802**	**0.9221**
Q8BFU3	Rnf214	RING finger protein 214	17367.83594	19195.04	19280.54	18350.13	**18548.38623**	14437.53	19770.59	15638.14	18873.15	**17179.85059**	**1.0797**	**0.3498**
Q8R180	Ero1a	ERO1-like protein alpha	12429.56738	11597.89	11004.88	10994.5	**11506.70825**	9086.163	11837.65	10187.88	11519.48	**10657.79614**	**1.0797**	**0.2823**
Q61239	Fnta	Protein farnesyltransferase/geranylgeranyltransferase type-1 subunit alpha	22290.41406	21001.13	19851.19	15193.32	**19584.01416**	20468.56	15704.68	17807.05	18588.76	**18142.26025**	**1.0795**	**0.4616**
Q02257	Jup	Junction plakoglobin	28566.38672	27295.56	33344.43	27692.21	**29224.64795**	25416.06	30004.82	26968.64	25905.73	**27073.81494**	**1.0794**	**0.2618**
P48193	Epb41	Protein 4.1	19100.64844	13770.62	20480.32	20281.2	**18408.19482**	15951.68	17214.98	16018.24	19031.78	**17054.16772**	**1.0794**	**0.4642**
Q9CZX9	Emc4	ER membrane protein complex subunit 4	18745.81445	18842.94	17181.14	21525.7	**19073.89893**	18889.63	19496.21	17599.52	14706	**17672.83838**	**1.0793**	**0.3541**
Q99LJ6	Gpx7	Glutathione peroxidase 7		2387.808			**2387.80835**	2637.631	1994.083	2239.134	1979.224	**2212.518066**	**1.0792**	
Q7M750	Opalin	Opalin	86711.17188	90218.27	60293.34	60729.64	**74488.10547**	73622.32	73675.96	78342.38	50458.77	**69024.85742**	**1.0791**	**0.6133**
Q8CGA0	Ppm1f	Protein phosphatase 1F	45103.46875	50947.55	48538.09	38882.74	**45867.96191**	36887.57	43635.81	40562.7	48956.02	**42510.52344**	**1.0790**	**0.3940**
P35550	Fbl	rRNA 2'-O-methyltransferase fibrillarin	34817.82813	36869.38	38103.43	45794.41	**38896.26172**	41334.21	40207.48	32326.39	30330.19	**36049.56592**	**1.0790**	**0.4662**
Q4ACU6	Shank3	SH3 and multiple ankyrin repeat domains protein 3	4375.986816	4439.115	2343.657	5466.397	**4156.288879**	3715.34	3876.453	3837.493	3979.674	**3852.23999**	**1.0789**	**0.6594**
Q9CQY2	Ramac	RNA guanine-N7 methyltransferase activating subunit	51832.79688	38698.12	47354.67	85109.06	**55748.66113**	76126.48	35408.77		43507.02	**51680.75651**	**1.0787**	**0.8083**
Q8C3X2	Ccdc90b	Coiled-coil domain-containing protein 90B, mitochondrial			16195.89	10852.93	**13524.40723**			12537.79		**12537.79102**	**1.0787**	
Q91VA6	Poldip2	Polymerase delta-interacting protein 2	31910.12305	37658.16	37145.74	34926.59	**35410.15479**	34710.7	28412.42	36584.29	31605.24	**32828.16113**	**1.0787**	**0.2892**
Q9CRC0	Vkorc1	Vitamin K epoxide reductase complex subunit 1	21745.21289	22286.47	18382.56	22175.37	**21147.40186**	20470.03	22379.5	17870.73	17703.23	**19605.87207**	**1.0786**	**0.3304**
O70152	Dpm1	Dolichol-phosphate mannosyltransferase subunit 1	36070.95703	33578.41	36418.25	32914.64	**34745.56348**	33583.59	34962.73	33132.18	27177.15	**32213.91455**	**1.0786**	**0.2386**
Q922Q8	Lrrc59	Leucine-rich repeat-containing protein 59	98915.33594	89054.45	89101.33	98181.84	**93813.23828**	96493.15	96688.59	82377.46	72353.26	**86978.11328**	**1.0786**	**0.3347**
O35963	Rab33b	Ras-related protein Rab-33B	35902.875	36120.24	30012.02	36476.84	**34627.99365**	32108.8	34892.88	30068.85	31379.28	**32112.45264**	**1.0783**	**0.2225**
Q6P9K9	Nrxn3	Neurexin-3	61243.67188	77085.02	79191.38	65141.77	**70665.45996**	60149.39	74605.27	63503.07	63875.46	**65533.29785**	**1.0783**	**0.3795**
Q61136	Prpf4b	Serine/threonine-protein kinase PRP4 homolog	8075.285645	7300.076	5870.256	6833.513	**7019.782593**	5815.048	5039.971	6597.736	8589.133	**6510.472046**	**1.0782**	**0.5883**

Q9D662	Sec23b	Protein transport protein Sec23B	16155.58984	19023.02	18971.82	23537.83	19422.06299	15791.25	17967.45	21537.53	16755.73	18012.98975		1.0782	0.5028
Q8JZQ9	Eif3b	Eukaryotic translation initiation factor 3 subunit B	52670.32422	43387.92	48257.66	50773.73	48772.40918	48726.13	43544.4	48028.71	40649.72	45237.24121		1.0781	0.2496
Q64727	Vcl	Vinculin	89591.49219	88550.37	117868	86782.09	95697.98633	87911.77	91515.13	90657.93	85039.84	88781.16602		1.0779	0.3953
Q9CQT1	Mri1	Methylthioribose-1-phosphate isomerase	19171.76758	12221.5	15943.38	18814.72	16537.84448	15011.82	13489.17	15803.17	17067.25	15342.85156		1.0779	0.5260
P10711	Tcea1	Transcription elongation factor A protein 1	29964.26367	26431.9	24638.92	31198.01	28058.27295	24270.41	27878.63	32005.06	19969.28	26030.84473		1.0779	0.5221
Q64337	Sqstm1	Sequestosome-1	20297.5918	20598.68	21886.08	21261.94	21011.07324	20992.01	18517.2	19157.98	19315.14	19495.58008		1.0777	0.0545
Q3V3Q7	Pacs2	Phosphofurin acidic cluster sorting protein 2	80124.46094	76935.55	72585.45	74958.55	76151.00586	70556.4	72793.77	67901.86	71426.98	70669.75391		1.0776	0.0279
P20065	Tmsb4x	Thymosin beta-4	98604.95313	83523.72	97481.91	92084.53	92923.77734	85648.15	65758.34	91414.49	102181	86250.49414		1.0774	0.4562
Q64373	Bcl2l1	Bcl-2-like protein 1	31440.83008	33773.31	27752.82	35271.47	32059.60938	26550.11	27794.6	33022.61	31670.86	29759.54346		1.0773	0.3457
A2A699	Fam171a2	Protein FAM171A2	27230.69531	18193.93	22552.15	24636.86	23153.4082	22973.04	16129.28	23045.99	23822.33	21492.6582		1.0773	0.5500
Q9D7H3	RtcA	RNA 3'-terminal phosphate cyclase	24302.50195	21266.39	25103.1	21518.94	23047.73291	19610.54	23505.76	20766.4	21695.76	21394.61816		1.0773	0.2416
P56812	Pdcd5	Programmed cell death protein 5	95375.51563	96179.75	92357.67	98941.54	95713.61914	94057.14	77128.57	88346.95	95940.99	88868.41406		1.0770	0.1745
Q68FL4	Ahcyl2	Putative adenosylhomocysteinase 3	301864.375	306627.5	246168.2	275755.3	282603.832	289493.1	286209.8	229940.7	243931.9	262393.8594		1.0770	0.3612
Q9D0E3	Lysmd1	LysM and putative peptidoglycan-binding domain-containing protein 1	23900.64453	21227.96	25342.2	25663.74	24033.63525	21609.69	24083.45	24904.32	18662.51	22314.99121		1.0770	0.3589
Q61074	Ppm1g	Protein phosphatase 1G	20360.66016	20704.54	19149.05	20117.34	20082.89941	17722.2	20391.94	18127.52	18345.73	18646.84668		1.0770	0.0802
Q9ERG2	Strn3	Striatin-3	21784.58594	21302.16	23868.83	25903.25	23214.70654	20836.56	24726.44	21071.09	19591.46	21556.38672		1.0769	0.3195
Q68EF6	Begain	Brain-enriched guanylate kinase-associated protein	14403.7998	14726.72	13116.82	13522.41	13942.43799	10575.94	13800.24	13738.51	13671.67	12946.59106		1.0769	0.2985
Q80TL7	Mon2	Protein MON2 homolog	21400.19531	23848.78	20775.98	24865.12	22722.51953	23826.28	21175.69	19279.96	20125.44	21101.84473		1.0768	0.2871
P63260	Actg1	Actin, cytoplasmic 2	9076246	8341977	2133466	2497941	5512407.375	11641537	2145130	2156105	4536359	5119782.5		1.0767	0.8971
Q3UTJ2	Sorbs2	Sorbin and SH3 domain-containing protein 2	83500.76563	95524.11	110704.9	99157.31	97221.75977	99226.74	93399.53	90803.99	77817.93	90312.04883		1.0765	0.3743
Q8VEF1	Gramd1a	Protein Aster-A	5660.493652	7577.492	6255.49		6497.825358	5736.358	6074.625	8450.396	3883.507	6036.221558		1.0765	0.7178
P27546	Map4	Microtubule-associated protein 4	254598.1719	237770.8	294264.3	243774.2	257601.8438	236932.2	238820	244170.7	237296.7	239304.9258		1.0765	0.2033
Q8K4G5	Ablim1	Actin-binding LIM protein 1	18128.20898	15128.81	17064.94	19396.97	17429.7334	16451.04	15287.17	17364.21	15671.06	16193.3667		1.0764	0.2681
Q80VQ0	Aldh3b1	Aldehyde dehydrogenase family 3 member B1	45364.17578	44780.05	58389.98	55317.92	50963.03125	44602.46	44987.39	55138.75	44665.38	47348.49414		1.0763	0.4355
P83940	Eloc	Elongin-C	27924.95898	28315.96	25068.56	25972.79	26820.56787	25132.15	26910.7	26311.38	21326.19	24920.10303		1.0763	0.2450
P26645	Marcks	Myristoylated alanine-rich C-kinase substrate	107263.9844	76071.17	85430.5	94889.53	90913.79688	79426.23	80077.95	105173.1	73211.21	84472.125		1.0763	0.5321
Q8BRN9	Cc2d1b	Coiled-coil and C2 domain-containing protein 1B	12161.79102	13336.22	13866.18	10878.3	12560.62354	14055.79	13467.11	8141.078	11020.53	11671.12512		1.0762	0.5755
Q66GT5	Ptpmt1	Phosphatidylglycerophosphatase and protein-tyrosine phosphatase 1	22748.26953	23808.88	28750.31	26566.57	25468.50928	21950.39	22899.41	26851.98	22960.37	23665.53711		1.0762	0.3399
P35803	Gpm6b	Neuronal membrane glycoprotein M6-b	275597.375	313766.5	219154.6	267771	269072.3594	261947.8	325989.9	190888.3	221277.9	250025.9727		1.0762	0.6068
Q9CQ92	Fis1	Mitochondrial fission 1 protein	243564.0625	225273.8	218067.7	224905.5	227952.75	237695.2	224618.7	192620.2	192337.4	211817.8711		1.0762	0.2514
P11531	Dmd	Dystrophin	11903.31543	12828.51	15916.68	13174.44	13455.73828	10531.51	12323.37	13236.15	13930.05	12505.271		1.0760	0.4341
Q6P3D0	Nudt16	U8 snoRNA-decapping enzyme	24602.19727	25323.21	21185.06	21420.34	23132.70117	23194.45	18858.18	20980.05	22962.7	21498.84229		1.0760	0.3090
Q9Z0N2	Eif2s3y	Eukaryotic translation initiation factor 2 subunit 3, Y-linked			4000.325	5678.458	4839.391602			4646.454	4349.164	4497.808838		1.0759	0.7273
Q99KR7	Ppif	Peptidyl-prolyl cis-trans isomerase F, mitochondrial	36097.68359	39502.35	39183.68	38456.46	38310.04395	38967.18	34908.94	33010.48	35541.96	35607.13672		1.0759	0.1138
Q9D868	Ppih	Peptidyl-prolyl cis-trans isomerase H	8843.469727	12220.95	7740.832	9891.965	9674.304565	8596.557	10676.73	10559.04	6136.921	8992.31311		1.0758	0.6505
Q9DD18	Dtd1	D-aminoacyl-tRNA deacylase 1	62392.61328	64593.36	49992.67	51716.74	57173.84668	62155.39	60879.12	46209.01	43331.63	53143.78516		1.0758	0.5345
Q9CWF2	Tubb2b	Tubulin beta-2B chain	727098.6875	729452.8	568061.2	578242.7	650713.8438	703278.8	686769.8	447328.3	582274.6	604912.8438		1.0757	0.5591
Q3TZZ7	Esyt2	Extended synaptotagmin-2	39512.78906	34007.24	42754.14	48443.73	41179.47656	39113.68	39303.21	43126.79	31613.07	38289.18896		1.0755	0.4827
Q66L44	Cbarp	Voltage-dependent calcium channel beta subunit-associated regulatory protein		14100.54	13982.42	9466.807	12516.59017	12949.73	15695.19	8759.393	9149.958	11638.56714		1.0754	0.7220
Q4V9Z5	Sez6l2	Seizure 6-like protein 2	58515.69141	47487.04	43076.9	55302.62	51095.5625	55244.23	54348.69	38114.39	42343.18	47512.62109		1.0754	0.5435
P35278	Rab5c	Ras-related protein Rab-5C	109261.3438	86164.91	93935.02	102938.8	98075.01172	92474.6	89253.77	90745.45	92331.48	91201.32617		1.0754	0.2281
P06151	Ldha	L-lactate dehydrogenase A chain	529831.25	499737.6	587239.7	617793	558650.375	510222.5	486200.5	522853.9	559022.7	519574.9063		1.0752	0.2516
Q61024	Asns	Asparagine synthetase [glutamine-hydrolyzing]	60016.30469	62471.94	53234.66	52719.79	57110.6748	56242.06	50006.71	54143.39	52073.3	53116.36816		1.0752	0.2015
Q8K1J6	Trnt1	CCA tRNA nucleotidyltransferase 1, mitochondrial	15601.64648	14177.73	13370.51	13552.26	14175.53516	15557.7	12662.07	13345.43	11171.7	13184.22876		1.0752	0.3786
P35290	Rab24	Ras-related protein Rab-24	21689.0957	22588.21	18685.84	19063.74	20506.72021	21222.73	18664.22	18395.07	18022.7	19076.17871		1.0750	0.2807
A2AWA9	Rabgap1	Rab GTPase-activating protein 1	26341.38867	30054.01	34696.41	30247.23	30334.7583	27039.47	31028.8	26516.28	28318.6	28225.78662		1.0747	0.3288
Q8R2K1	Fuom	Fucose mutarotase	15215.54688	31679.63	15068.94	22057.29	21005.35425	20752.8	18809.98	21338.95	17281.32	19545.76172		1.0747	0.7291
Q8BLN5	Lss	Lanosterol synthase	10256.83496	11648.9	9657.479	5917.52	9370.182495	12116.58	9615.251	6536.924	6608.555	8719.327026		1.0746	0.7322
P20444	Prkca	Protein kinase C alpha type	33107.62891	31238.84	37229.26	35829	34351.18164	30931.07	33344.09	31145.51	32445.11	31966.44434		1.0746	0.1535
Q8BWZ3	Naa25	N-alpha-acetyltransferase 25, NatB auxiliary subunit	21799.15039	15770.89	15535.76	25037.97	19535.94263	18131.88	17577.18	19002.49	18010.3	18180.46094		1.0746	0.5861
Q8BVI5	Stx16	Syntaxin-16	11269.4043	9743.542	9783.969	12089.05	10721.49072	10435.04	8523.049	9844.469	11111.69	9978.561523		1.0745	0.3876
Q8CAA7	Pgm2l1	Glucose 1,6-bisphosphate synthase	115706.3828	117997.4	110604.1	131344.1	118912.9883	111935.3	110021.1	100868.6	119887.4	110678.123		1.0744	0.2123
Q8BH82	Napepld	N-acyl-phosphatidylethanolamine-hydrolyzing phospholipase D	53556.74609	41465.71	40218.44	46108.45	45337.33594	49398.93	47829.28	38175.65	33403	42201.71484		1.0743	0.5447
P42227	Stat3	Signal transducer and activator of transcription 3	14180.5	19634.71	20812.35	16880.06	17876.90332	17434.28	16260.43	16626.63	16250.51	16642.96191		1.0741	0.4445
Q8BRK9	Man2a2	Alpha-mannosidase 2x			9382.654	9322.597	9352.625488	9128.771		8287.669		8708.220215		1.0740	0.2660
P60904	Dnajc5	DnaJ homolog subfamily C member 5	135442.7344	118541.3	108726.5	137546	125064.1172	126925.6	123583.2	102340.6	112954.3	116450.9395		1.0740	0.3692
O09114	Ptgds	Prostaglandin-H2 D-isomerase	49803.44922	33040.12	41791.25	35410.2	40011.25684	28893.99	33804.98	38401.47	47928.51	37257.23584		1.0739	0.6357
Q8CCK0	Macroh2a2	Core histone macro-H2A.2	77627.36719	81516.7	80118.7	75138.27	78600.26172	79116.22	77890.26	75850	59958.4	73203.71875		1.0737	0.2930
P97379	G3bp2	Ras GTPase-activating protein-binding protein 2	47874.41016	51525.92	42111.78	47241.78	47188.47266	44148.8	49013.78	39374.07	43265.46	43950.52539		1.0737	0.2869
Q7TT50	Cdc42bpb	Serine/threonine-protein kinase MRCK beta	27402.00586	24822.47	26400.48	26304.71	26232.41846	27609.99	24562.7	22881.58	22682.67	24434.23633		1.0736	0.2026
Q9JM76	Arpc3	Actin-related protein 2/3 complex subunit 3	193558.2031	172963	176028.7	195056.8	184401.6719	189036.5	179140.9	153576.1	165310.5	171766.0078		1.0736	0.2394
Q8VHE0	Sec63	Translocation protein SEC63 homolog	11359.57324	11050.64	10658.47	9236.006	10576.17261	6085.666	10559.56	11663.62	11098.84	9851.922729		1.0735	0.6132
Q9QZ73	Dcun1d1	DCN1-like protein 1	12252.11719	12143.21	10561.56	12302.44	11814.83472	13031.43	6790.176	11208.35	12995.35	11006.32471		1.0735	0.6155
Q9D1K7	Adissp	Adipose-secreted signaling protein	41629.26563	61319.72	38276.06	54203.85	48857.22461	48552.46	49263.77	39971.35	44272.3	45514.97168		1.0734	0.5854
Q921H8	Acaa1a	3-ketoacyl-CoA thiolase A, peroxisomal	100655.6484	95195.77	101926.9	106544.5	101080.6914	99148.02	100213.6	90971.7	86462.31	94198.90625		1.0731	0.1398

P26516	Psmd7	26S proteasome non-ATPase regulatory subunit 7	59596.36719	56064.54	60139.37	56459.76	**58065.00781**	45426.69	53614.73	58879.54	58556.07	**54119.25977**	**1.0729**	**0.2781**
Q921M3	Sf3b3	Splicing factor 3B subunit 3	54932.25781	54952.31	56990.39	53686.18	**55140.28613**	52124.38	52280.28	53287.51	47889.1	**51395.31738**	**1.0729**	**0.0348**
P56695	Wfs1	Wolframin	24601.82813	21389.95	26730.8	25298.34	**24505.229**	23025.75	22840.36	22864.9	22645.09	**22844.02539**	**1.0727**	**0.1925**
Q9JKL5	Tesc	Calcineurin B homologous protein 3	14887.03516	13711.42	15636.18	14052.63	**14571.81519**	14365.79	12965.37	15182.43	11830.95	**13586.13477**	**1.0726**	**0.2951**
P70414	Slc8a1	Sodium/calcium exchanger 1	41891.18359	41420.95	45418.52	43772.61	**43125.81641**	43813.73	41689.9	35768.41	39571.58	**40210.9043**	**1.0725**	**0.1847**
Q8BJU0	Sgta	Small glutamine-rich tetratricopeptide repeat-containing protein alpha	95176.60938	89746.5	84207.7	91785.66	**90229.11523**	88688.36	87362.12	78975.2	81508.36	**84133.50781**	**1.0725**	**0.1113**
Q8BH79	Ano10	Anoctamin-10	20442.13281	18565.98	20162.31	15868.54	**18759.73877**	19819.73	19448.97	16488.53	14223.07	**17495.07227**	**1.0723**	**0.4817**
Q69Z98	Brsk2	Serine/threonine-protein kinase BRSK2	29108.36523	33428.31	26421.79	35948.82	**31226.82031**	29104.37	33917.25	26889.71	26589.14	**29125.12158**	**1.0722**	**0.4698**
Q9D958	Spcs1	Signal peptidase complex subunit 1	17576.10742	13042.12	16416.73	16603.19	**15909.53638**	7933.503	16170.02	21520.96	13731.23	**14838.92773**	**1.0721**	**0.7323**
Q3TBW2	Mrpl10	Large ribosomal subunit protein uL10m	16291.82715	14728.69	13175.2	17159.02	**15338.68555**	13573.08	14407.54	15489.92	13766.34	**14309.21924**	**1.0719**	**0.3338**
Q99KP3	Cryl1	Lambda-crystallin homolog	5780.541016	6109.473	9526.8	7211.542	**7157.088989**	7244.151	5796.227	7497.8	6170.787	**6677.241211**	**1.0719**	**0.6285**
Q91W53	Golga7	Golgin subfamily A member 7	26582.11133	25300.76	25943.62	23986.54	**25453.25879**	24781.93	24575.59	22613.04	23022.78	**23748.33105**	**1.0718**	**0.0708**
Q811Q9	Pcyt1b	Choline-phosphate cytidylyltransferase B	20836.31055	22288.66	23535.31	20476.41	**21784.17285**	19312.05	21371.6	21442.92	19174.8	**20325.34375**	**1.0718**	**0.1720**
Q9QXG2	Chm	Rab proteins geranylgeranyltransferase component A 1	11801.21094	29505.44	24271.28	15459.98	**20259.47632**	30806.57	16403.43	16919.18	11489.46	**18904.66113**	**1.0717**	**0.8229**
Q9D164	Fxyd6	FXYD domain-containing ion transport regulator 6	28434.97266	26580.81	24192.56	26711.11	**26479.8623**	32092.59	21647.74	23946.22	21150.46	**24709.25293**	**1.0717**	**0.5335**
Q64010	Crk	Adapter molecule crk	110422.5469	105872.8	113147.9	109439.4	**109720.6406**	106885.7	102500.8	96140.4	104025.2	**102388.0254**	**1.0716**	**0.0360**
Q8C4B4	Unc119b	Protein unc-119 homolog B	32709.99023	36761.74		20990.89	**30154.20898**	33388.94	36895.91	10073.37	32201.04	**28139.81421**	**1.0716**	**0.8165**
Q9CQ65	Mtap	S-methyl-5'-thioadenosine phosphorylase	71604.15625	81731.23	81460.65	83867.48	**79665.88086**	68722.05	76720.26	76582.24	75374.24	**74349.69727**	**1.0715**	**0.1620**
Q8R1N4	Nudcd3	NudC domain-containing protein 3	32705.29102	28757.68	26773.83	27317.66	**28888.61768**	24922.98	31034.57	24296.97	27594	**26962.13037**	**1.0715**	**0.3807**
Q62417	Sorbs1	Sorbin and SH3 domain-containing protein 1	98226.11719	88379.57	106282.5	103046.4	**98983.66016**	93361.78	89921.38	95069.39	91197.95	**92387.625**	**1.0714**	**0.1559**
Q9CPS6	Hint3	Adenosine 5'-monophosphoramidase HINT3	45918.93359	47664.43	45534.1	42706.98	**45456.11133**	44393.21	45465.66	39296.84	40556.18	**42427.97461**	**1.0714**	**0.1442**
P70704	Atp8a1	Phospholipid-transporting ATPase IA	124701.2188	126854	125345.9	142321.4	**129805.6289**	125881.6	122328.4	119284.1	117163	**121164.2773**	**1.0713**	**0.1097**
Q91V09	Wdr13	WD repeat-containing protein 13	32508.6875	31599.54	27119.02	39766.36	**32748.40186**	31493.54	32216.36	28539.93	30026.13	**30568.99219**	**1.0713**	**0.4572**
Q6RI63	Fam120b	Constitutive coactivator of peroxisome proliferator-activated receptor gam	13395.59375	13119.66	12470.43	9044.332	**12007.50415**	10308.28	10575.42	12328.98	11622.11	**11208.69678**	**1.0713**	**0.4990**
Q9JK48	Sh3glb1	Endophilin-B1	36129.35156	34236.32	34160.15	37027.02	**35388.21094**	30731.08	37851.09	29934.16	33620.14	**33034.11719**	**1.0713**	**0.2675**
Q9CYR6	Pgm3	Phosphoacetylglucosamine mutase	35222.64453	40940.9	42514.31	38701.82	**39344.91797**	33970.35	37228.31	37295.96	38416.69	**36727.82617**	**1.0713**	**0.2067**
P70349	Hint1	Adenosine 5'-monophosphoramidase HINT1	119208.1797	116744.5	129577.3	129132.5	**123665.623**	104168.2	115165.4	114102.9	128403.2	**115459.959**	**1.0711**	**0.2193**
P11627	L1cam	Neural cell adhesion molecule L1	102752.1953	95414.25	95018.62	99912.05	**98274.27734**	102179.5	96832.2	84695.26	83315.81	**91755.68555**	**1.0710**	**0.2380**
Q8BK67	Rcc2	Protein RCC2	37231.48438	32788.09	30773.94	31488.73	**33070.56201**	31751.81	36118.99	26817.36	28825.99	**30878.53809**	**1.0710**	**0.4117**
Q61137	Astn1	Astrotactin-1	16082.18945	14853.6	12576.89	12611.82	**14031.12451**	15928.92	13855.79	12513.73	10107.59	**13101.50757**	**1.0710**	**0.5574**
P62717	Rpl18a	Large ribosomal subunit protein eL20	148430.2813	141428.6	119566.6	120498.8	**132481.0449**	124147.3	123353.4	113385.9	133963.7	**123712.5547**	**1.0709**	**0.3394**
Q9DBB8	Dhdh	Trans-1,2-dihydrobenzene-1,2-diol dehydrogenase	11201.36719	7343.076	10569.3	9763.949	**9719.423218**	10078.24	8899.683	9245.844	8082.471	**9076.559692**	**1.0708**	**0.5199**
Q9CQI3	Gmfb	Glia maturation factor beta	52541.69141	53490.37	62631.93	59619.35	**57070.83301**	53059.34	60149.78	47505.4	52472.82	**53296.83496**	**1.0708**	**0.3298**
O88271	Cfdp1	Craniofacial development protein 1	12431.8916	12586.89	10957.28	15482.3	**12864.5918**	13617.87	14337.39	10802	9301.812	**12014.7688**	**1.0707**	**0.5952**
Q6KAR6	Exoc3	Exocyst complex component 3	29893.875	32754.23	27985.47	27521.97	**29538.88721**	28077.93	29812.9	27978.35	24488.65	**27589.45654**	**1.0707**	**0.2769**
Q52KF3	Spire1	Protein spire homolog 1	17315.73438	16938.46	17964.94	16558.52	**17194.41309**	16910.78	16148.54	13613.35	17568.27	**16060.23657**	**1.0706**	**0.2619**
P61021	Rab5b	Ras-related protein Rab-5B	442765.0938	435530.3	490227.4	430851.6	**449843.5938**	424213.8	425951.9	398726.4	431898.5	**420197.6719**	**1.0706**	**0.1048**
P05214	Tuba3a	Tubulin alpha-3 chain	1780296.625	1565645	1010901	1149146	**1376497.297**	1326301	1222753	1359823	1234305	**1285795.5**	**1.0705**	**0.6362**
Q60673	Ptprn	Receptor-type tyrosine-protein phosphatase-like N	19777.14258	19811.2	15706.26	22892.15	**19546.6897**	20563.07	16556.46	13212.04	22706.07	**18259.41187**	**1.0705**	**0.6348**
Q6PD03	Ppp2r5a	Serine/threonine-protein phosphatase 2A 56 kDa regulatory subunit alpha i	25484.08984	28683.5	27791.63	26801.2	**27190.104**	30343.93	23114.26	29472.08	18672.49	**25400.69092**	**1.0704**	**0.5526**
P08556	Nras	GTPase NRas	469879.5	516836.2	491853.1	451685.8	**482563.6484**	452452.8	451188.7	432263.4	467433.3	**450834.5391**	**1.0704**	**0.0915**
P53995	Anapc1	Anaphase-promoting complex subunit 1	3770.9021		8794.418	8387.724	**6984.3479**		7016.355	5492.519	7066.539	**6525.137695**	**1.0704**	**0.7995**
Q61753	Phgdh	D-3-phosphoglycerate dehydrogenase	510366.125	526350.5	501716.2	531234.4	**517416.7969**	482257.6	482438.1	491497.3	477456	**483412.2266**	**1.0703**	**0.0039**
O35609	Scamp3	Secretory carrier-associated membrane protein 3	54973.84375	52889.81	48175.76	56309.22	**53087.15918**	53747.75	53120.99	46506.07	45020.89	**49598.92578**	**1.0703**	**0.2686**
P48678	Lmna	Prelamin-A/C	106910.6875	103593	147392.2	111626.4	**117380.5488**	109982	122546.3	101077.4	105090.1	**109673.957**	**1.0703**	**0.5156**
Q8BL86	Mblac2	Acyl-coenzyme A thioesterase MBLAC2	40070.57813	35512.41	37835.11	45179.59	**39649.41895**	34368.71	35505.45	42163.18	36150.12	**37046.86816**	**1.0703**	**0.3729**
Q62465	Vat1	Synaptic vesicle membrane protein VAT-1 homolog	568766	577415.7	642592.8	647366.5	**609035.2344**	548211.9	555435.8	582038.9	590815.4	**569125.5**	**1.0701**	**0.1367**
P70122	Sbds	Ribosome maturation protein SBDS	20934.67188	21068.43	19838.69	14493.78	**19083.89233**	20108.01	19338.21	15257.77	16631.85	**17833.9624**	**1.0701**	**0.5404**
Q9D1J1	Necap2	Adaptin ear-binding coat-associated protein 2	23403.58008	22327.25	20226.71	20011.53	**21492.26807**	21726.13	20668.89	16012.13	21937.85	**20086.25171**	**1.0700**	**0.4167**
Q8K341	Atat1	Alpha-tubulin N-acetyltransferase 1	18229.7832	18961.59	19185.02	11203.96	**16895.08887**	16369.47	18072.85	17512.87	11206.48	**15790.41724**	**1.0700**	**0.6704**
P06909	Cfh	Complement factor H			2851.48	5129.935	**3990.707153**	8545.139	2490.264		156.2244	**3730.542323**	**1.0697**	**0.9428**
Q9QXG4	Acss2	Acetyl-coenzyme A synthetase, cytoplasmic	40718.14063	38088.23	36160.16	34818.96	**37446.37207**	37321.66	37116.79	33031.7	32556.07	**35006.5542**	**1.0697**	**0.2267**
P51125	Cast	Calpastatin	29350.76367	35307.46	36753.48	37856.34	**34817.01221**	34694.99	29114.34	30793.85	35602.54	**32551.42969**	**1.0696**	**0.3904**
O35459	Ech1	Delta(3,5)-Delta(2,4)-dienoyl-CoA isomerase, mitochondrial	37761.09375	38307.55	36644.38	30566.79	**35819.95215**	33100.02	37481.46	30536.25	32838.87	**33489.14941**	**1.0696**	**0.3499**
Q6ZPS6	Ankib1	Ankyrin repeat and IBR domain-containing protein 1	10716.74219	13038.23	8136.078	9660.243	**10387.82397**	15241.49	8035.724	7083.01	8494.602	**9713.706299**	**1.0694**	**0.7625**
Q9JK42	Pdk2	[Pyruvate dehydrogenase (acetyl-transferring)] kinase isozyme 2, mitochon	43630.28125	47489.48	55405.23	54947.59	**50368.14844**	48048.29	46538.36	47742.84	46071.64	**47100.28223**	**1.0694**	**0.3067**
Q3UJP5	Cfap418	Cilia- and flagella-associated protein 418	10399.06152	21995.77	16221.81	17961.95	**16644.64746**	13920.5	15479.31	15696.36	17169.03	**15566.30005**	**1.0693**	**0.6810**
Q8C7M3	Trim9	E3 ubiquitin-protein ligase TRIM9	27799.13281	27421.85	23088.89	28012.96	**26580.71094**	29983.04	23705.99	23864.08	21888.49	**24860.40283**	**1.0692**	**0.4477**
Q8C166	Cpne1	Copine-1	68060.03906	72434.67	75561.2	76467.09	**73130.75**	70761.2	72555.73	64944.21	65355.73	**68404.2168**	**1.0691**	**0.1302**
Q9D0L4	Adck1	AarF domain-containing protein kinase 1	13013.30371	14735.7	14323.27	12948.89	**13755.29004**	13850.06	11016.63	13860.67	12740.79	**12867.04102**	**1.0690**	**0.3150**
Q3UKJ7	Smu1	WD40 repeat-containing protein SMU1	6725.987793	9447.233	7850.017	5688.479	**7427.929321**	7053.892	6371.525	6782.363	7592.369	**6950.037354**	**1.0688**	**0.5920**
P29387	Gnb4	Guanine nucleotide-binding protein subunit beta-4	29825.87695	36074.4	28235.22	28725.32	**30715.20361**	31269.17	28490.84	28659.83	26542.89	**28740.68115**	**1.0687**	**0.3748**
Q9EPN1	Nbea	Neurobeachin	52561.28906	51857.51	49842.7	50549.07	**51202.64355**	42592.18	48100.96	49057.75	51944.48	**47923.84277**	**1.0684**	**0.1610**
Q9CRB2	Nhp2	H/ACA ribonucleoprotein complex subunit 2	20297.87695	18206.52	19505.91	20512.36	**19630.66602**	19174.42	20031.81	18619.09	15672.92	**18374.56079**	**1.0684**	**0.2892**
A2A690	Tanc2	Protein TANC2	202647.625	136965.9	187554.8	134328.9	**165374.3047**	153964.8	132680.4	162843	169700.9	**154797.2617**	**1.0683**	**0.6018**

Q5SSW2	Psme4	Proteasome activator complex subunit 4		1129.122			1129.121582	1056.992				1056.992432	1.0682	
Q9DCR2	Ap3s1	AP-3 complex subunit sigma-1	41598.39844	40873.71	39113.71	42766.85	41088.16797	41945.27	37360.42	34503.07	40045.39	38463.53613	1.0682	0.1934
Q99JI6	Rap1b	Ras-related protein Rap-1b	47561.23828	47143.25	54138.94	43068.71	47978.03613	46139.77	42485.24	44130.6	46912.27	44916.96777	1.0681	0.2665
Q5EG47	Prkaa1	5'-AMP-activated protein kinase catalytic subunit alpha-1	44454.43359	44096.38	46333.12	45927.38	45202.82813	40489.41	44327.93	46272.42	38199.74	42322.37598	1.0681	0.1814
P57716	Ncstn	Nicastrin	20658.77539	16295.39	22275.43	21342.38	20142.99536	19657.1	21092.54	18359.48	16331.25	18860.09033	1.0680	0.4706
Q9Z0P4	Palm	Paralemmin-1	185518.375	181736.2	204836.8	202522.4	193653.4531	168375.9	174947.4	191375.1	190645.6	181335.9883	1.0679	0.1840
P08553	Nefm	Neurofilament medium polypeptide	4040415.75	3828935	5376247	3986125	4307930.75	3649590	3818963	4192718	4475378	4034162.188	1.0679	0.5234
Q9JMD3	Stard10	START domain-containing protein 10	33955.28906	35724.77	34758.27	39013.51	35862.95996	29165.7	33278.6	33555.6	38336.32	33584.05518	1.0679	0.3360
P70205	Adcyap1r1	Pituitary adenylate cyclase-activating polypeptide type I receptor	41908.23438	37761.57	33041.1	40103.17	38203.51953	40069.4	42794.5	27456.82	32783.76	35776.12012	1.0678	0.5642
Q61553	Fscn1	Fascin	368164.4063	381321.4	355874.1	359613.5	366243.3516	375006.5	366088.1	304310.3	326511.3	342979.0313	1.0678	0.2339
Q9CWG8	Ndufaf7	Protein arginine methyltransferase NDUFAF7, mitochondrial	17119.56055	18011.14	15353.25	17223.77	16926.93188	14577.32	16900.64	14015.09	17915.97	15852.25391	1.0678	0.3603
P11798	Camk2a	Calcium/calmodulin-dependent protein kinase type II subunit alpha	153371.7656	147387.9	111134.5	163525.5	143854.9375	145534.6	144175	127582.6	121612.1	134726.0684	1.0678	0.5050
Q91Z69	Srgap1	SLIT-ROBO Rho GTPase-activating protein 1	12504.79199	14085.75	18121.22	6913.869	12906.40918	8596.072	9149.262	11663.22	18941.81	12087.58936	1.0677	0.8137
Q8BIK4	Dock9	Dedicator of cytokinesis protein 9	18513.0293	21577.15	24045.73	21183.35	21329.81689	19332.92	18784.77	20324.95	21467.86	19977.625	1.0677	0.3305
Q61233	Lcp1	Plastin-2	104555.9609	107303.3	133468.8	106429.1	112939.2852	109162.2	107688.3	103830.7	102450.6	105782.9395	1.0677	0.3490
P97820	Map4k4	Mitogen-activated protein kinase kinase kinase kinase 4	86694.69531	70799.4	68686.31	70085.82	74066.55664	78383.36	67614.47	64354.19	67144.18	69374.0498	1.0676	0.4049
P63037	Dnaja1	DnaJ homolog subfamily A member 1	59030.83594	59942.12	51071.93	47284.94	54332.45605	56936.88	54017.33	42467.44	50147.71	50892.33984	1.0676	0.4634
Q9DCG9	Trmt112	Multifunctional methyltransferase subunit TRM112-like protein	31016.62891	26199.88	27775.84	27715.52	28176.9668	29644.78	30602.48	20801.32	24526.66	26393.81055	1.0676	0.5035
P62737	Acta2	Actin, aortic smooth muscle	170674.7031	213998.7	272812.9	242203.1	224922.3438	197841.8	240397.4	164735.9	239857.3	210708.082	1.0675	0.6343
B9EJ86	Osbpl8	Oxysterol-binding protein-related protein 8	33304.59766	32049.23	30437	35178.08	32742.22705	32267.14	34023.17	27714.66	28689.09	30673.51221	1.0674	0.2920
Q791T5	Mtch1	Mitochondrial carrier homolog 1	178844.8594	176844.4	176468.5	197106	182315.9453	182790.6	172350.6	160599.6	167449.7	170797.6328	1.0674	0.1417
Q9DCJ9	Npl	N-acetylneuraminate lyase	17492.53906	23529.37	21470.78	18778.97	20317.91602	19569.99	17352.53	22154.49	17062.01	19034.75635	1.0674	0.5019
Q9DAZ9	Zfyve19	Abscission/NoCut checkpoint regulator	5729.59082	5744.61	8304.101	3896.996	5918.82428	4951.282	5546.589	6467.307	5220.455	5546.408203	1.0671	0.7126
Q9Z2A5	Ate1	Arginyl-tRNA--protein transferase 1	18954.4707	14607.83	14075.56	18980.09	16654.48706	14719.35	14314.26	17914	15483.55	15607.78931	1.0671	0.5281
Q9ET22	Dpp7	Dipeptidyl peptidase 2	18362.60352	16043.99	18141.47	16515.28	17265.83521	15256.2	16083.87	19939.09	13451.65	16182.70239	1.0669	0.4932
Q7TPR4	Actn1	Alpha-actinin-1	91980.03125	97290.54	104702.1	91902.16	96468.70117	95664.72	91651.05	85142.31	89212.68	90417.68945	1.0669	0.1568
P35762	Cd81	CD81 antigen	205236.8281	170840.5	225237	200819	200533.3555	204676.1	176585.6	181899.5	188705.4	187966.6523	1.0669	0.3635
O09106	Hdac1	Histone deacetylase 1	9171.456055	6886.717	8827.835	8266.737	8288.186401	12627.72	5970.969	6508.615	5971.595	7769.724609	1.0667	0.7707
P21279	Gnaq	Guanine nucleotide-binding protein G(q) subunit alpha	526285.75	529622.1	581190.9	580088.3	554296.7656	536840.7	491797.2	521838.5	528303.3	519694.9063	1.0666	0.1045
P55258	Rab8a	Ras-related protein Rab-8A	55481.14453	49505.24	45742.1	52039.48	50691.99219	53287.48	48959.57	44638.69	43241.32	47531.7666	1.0665	0.3420
S4R1M9	Osbpl10	Oxysterol-binding protein-related protein 10	18558.83398	13293.21	20011.73	19598.48	17865.56396	18846.72	20744.91	13443.14	13973.89	16752.16431	1.0665	0.6565
Q8C3F2	Fam120c	Constitutive coactivator of PPAR-gamma-like protein 2	43616.62109	41108.32	40377.99	45915.71	42754.6582	40916.07	43677.78	39741.05	36038.25	40093.28809	1.0664	0.2367
Q5PR73	Diras2	GTP-binding protein Di-Ras2	55660.60547	64795.8	60964.94	56151.29	59393.15918	61598.71	46465.64	61311.8	53420.74	55699.22168	1.0663	0.4142
Q9JLI6	Scly	Selenocysteine lyase	6793.279785	4897.437	3239.813	2722.509	4413.259521	4832.126	7468.781	2761.942	1492.775	4138.905914	1.0663	0.8692
P26339	Chga	Chromogranin-A	9252.047852	9219.432		8767.844	9079.774414	9124.959	10659.67	6113.144	8166.078	8515.962769	1.0662	0.6399
Q9Z2Z6	Slc25a20	Mitochondrial carnitine/acylcarnitine carrier protein	68940.78125	69079.41	67655.66	68808.66	68621.12891	66929.73	71143.86	63158.19	56258.48	64372.56348	1.0660	0.2294
Q9CQ48	Nudcd2	NudC domain-containing protein 2	22826.1875	19244.9	10658.43	15911.48	17160.24854	19731.35	22319.43	13005.35	9337.903	16098.50708	1.0660	0.7972
Q8VE09	Ttc39c	Tetratricopeptide repeat protein 39C	32112.44141	29226.91	30835.36	31441.09	30903.9502	35251.02	30726.57	21987.13	28003.41	28992.03271	1.0659	0.5259
Q505B7	Zbtb8os	Protein archease	39201.24609	38594.79	40623.79	49328.06	41936.9707	36631.12	42300.35	41892.86	36558.89	39345.80273	1.0659	0.4155
Q60710	Samhd1	Deoxynucleoside triphosphate triphosphohydrolase SAMHD1	11354.33594	11226.68	12027.68	8193.903	10700.65039	10526.65	11411.03	9336.005	8888.644	10040.58203	1.0657	0.5446
Q8C547	Heatr5b	HEAT repeat-containing protein 5B	7227.592285	7474.495	6551.715	10234.38	7872.046265	6027.512	9042.686	6158.937	8318.744	7386.969604	1.0657	0.6782
Q9D695	Serpinb7	Serpin B7	23144.86523	26103.58	41248.75	46775.33	34318.13086	33913.36	32040.87	38900.08	23968.13	32205.61328	1.0656	0.7572
Q8BRU6	Slc18a2	Synaptic vesicular amine transporter	19776.21289	17980.89	19445.7	15159.33	18090.53418	17927.53	17568	17632.66	14779.61	16976.95068	1.0656	0.4192
Q9R062	Gyg1	Glycogenin-1	117649.25	110333.4	101221.6	135483.6	116171.9473	113999.8	109830.6	98800.7	113478.8	109027.4805	1.0655	0.4103
Q9DD02	Hikeshi	Protein Hikeshi	4108.411621	8763.92		6175.304	6349.211914	7096.746	3769.066	5266.269	7706.838	5959.729614	1.0654	0.8112
Q01405	Sec23a	Protein transport protein Sec23A	77546.82031	82760.52	82372.84	83018.09	81424.56641	82958.93	83634.11	69124.02	70040.33	76439.3457	1.0652	0.2773
P28571	Slc6a9	Sodium- and chloride-dependent glycine transporter 1	249154.0156	230497.6	254465.2	230023.8	241035.1523	174059.3	246181.5	240538.7	244357.6	226284.2539	1.0652	0.4569
Q91XE4	Acy3	N-acyl-aromatic-L-amino acid amidohydrolase (carboxylate-forming)	38847.72266	36828.09	36530.67	34082.07	36572.13867	33611.7	33034.89	36527.38	34178.58	34338.13672	1.0651	0.1220
Q60790	Rasa3	Ras GTPase-activating protein 3	24078.27734	26749.73	25939.03	23891.91	25164.73682	24820.12	25091.87	22530.62	22075.14	23629.4375	1.0650	0.1920
A6H5Y3	Mtr	Methionine synthase	18512.82422	19285.3	17001.83		18266.65104	19438.97	19577.03	20994.39	8599.379	17152.44434	1.0650	0.7595
O88543	Cops3	COP9 signalosome complex subunit 3	157896.6563	165257.9	146974.9	152226.7	155589.0547	148680.7	132641.4	155895	147183.3	146100.1016	1.0649	0.1799
Q921J2	Rheb	GTP-binding protein Rheb	32556.25391	30001.76	33566.42	36009.91	33033.5874	32734.21	32658.73	30582.88	28107.13	31020.73633	1.0649	0.2695
Q3UIA2	Arhgap17	Rho GTPase-activating protein 17	8451.102539	5860.123	7424.098	5454.282	6797.401123	5445.388	8733.708	6351.275	5002.583	6383.238525	1.0649	0.7158
Q9D0L7	Armc10	Armadillo repeat-containing protein 10	23091.57617	22796.39	22185.62	26019.5	23523.271	26004.82	24703.17	17027.69	20626.68	22090.58838	1.0649	0.5410
Q6ZQI3	Mlec	Malectin	58633.07031	57070.96	66001.05	54728.86	59108.48438	58764.39	63257.45	48535.09	51482.26	55509.7959	1.0648	0.4191
Q61029	Tmpo	Lamina-associated polypeptide 2, isoforms beta/delta/epsilon/gamma	63960.55078	65582.45	81580.43	77007.29	72032.67871	69762.46	69391.12	66634.3	64820.17	67652.01367	1.0648	0.3644
Q9QXS1	Plec	Plectin	383302.125	385336.8	457204.1	398597.8	406110.2109	381131.6	379777.8	379650	385130.4	381422.4375	1.0647	0.2060
Q9D1E8	Agpat5	1-acyl-sn-glycerol-3-phosphate acyltransferase epsilon	40570.87891	34379.23	35537.68	30569.22	35264.25195	33984.56	36646.84	32145.11	29727	33125.87842	1.0646	0.4301
Q3UDW8	Hgsnat	Heparan-alpha-glucosaminide N-acetyltransferase	100477.6719	89970.12	93671.08	107150.8	97817.41211	86085.02	81696.63	94869.55	104898.6	91887.46094	1.0645	0.3886
P08228	Sod1	Superoxide dismutase [Cu-Zn]	1127172.875	1083248	1315513	1377740	1225918.594	927752.7	1055804	1157465	1465589	1151652.547	1.0645	0.6024
B2RPU2	Plekhd1	Pleckstrin homology domain-containing family D member 1	16844.86719	13579.5	17003.08	17657.47	16271.229	14603.81	14521.41	16798.13	15230.6	15288.49023	1.0643	0.3878
Q7M6Y3	Picalm	Phosphatidylinositol-binding clathrin assembly protein	27464.25586	26788.58	27753.78	27628.83	27408.86182	26738.41	30897.06	22210.15	23170.39	25754.00293	1.0643	0.4360
P24270	Cat	Catalase	38687.61719	40774.11	37268.51	33524.02	37563.56543	37380.8	37640.64	31507.16	34671.68	35300.06982	1.0641	0.3210
P00397	Mtco1	Cytochrome c oxidase subunit 1	108294.1328	94579.2	89467.25	118055.5	102599.0098	94520.23	104776.1	86826.69	99581.43	96426.10547	1.0640	0.4447
O35465	Fkbp8	Peptidyl-prolyl cis-trans isomerase FKBP8	85992.42969	85483.35	79070.86	88911.47	84864.52734	77903.47	99216.44	77224.48	64719.25	79765.90918	1.0639	0.5193

Q9EQ20	Aldh6a1	Methylmalonate-semialdehyde dehydrogenase [acylating], mitochondrial	357369.7188	349723.5	402880.8	395550.4	376381.0938	353129.3	373333.2	352907.8	335926.9	353824.3125	1.0638	0.1932
Q8BZJ7	Dcun1d2	DCN1-like protein 2	9815.092773	10929.64	12683.73	10139.33	10891.95142	11147.71	9801.592	10908.5	9102.201	10240.00171	1.0637	0.4467
Q91WP6	Serpina3n	Serine protease inhibitor A3N	1450.875488		5414.71		3432.792969		4087.22	2556.713	3038.565	3227.499512	1.0636	0.9053
Q80Y81	Elac2	Zinc phosphodiesterase ELAC protein 2	5873.194336	3041.304	11115.21	6347.992	6594.425354	5597.009	3992.93	8824.934	6385.4	6200.068176	1.0636	0.8467
Q920P5	Ak5	Adenylate kinase isoenzyme 5	34978.98047	33585.51	28681.61	35023.41	33067.37695	28573.76	32938.28	30379.95	32474.85	31091.70654	1.0635	0.3160
Q8R3Q6	Mix23	Protein MIX23	26560.3125	34042.39	12924.08	18420.63	22986.85327	27831.17	16401.24	23860.76	18364.34	21614.37842	1.0635	0.8047
Q8BXV2	Bri3bp	BRI3-binding protein	27244.50781	27188.17	28597.14	24736.86	26941.66895	24320.58	29961.98	21895.2	25160.07	25334.46045	1.0634	0.4236
Q8C052	Map1s	Microtubule-associated protein 1S	48274.61328	45472.32	48919.7	49051.5	47929.53418	46949.35	45966.15	45632.89	41745.39	45073.44336	1.0634	0.0905
Q9QYX7	Pclo	Protein piccolo	16075.57031	14495.97	24219.91	10262.27	16263.43066	17391.65	15519.27	14465.19	13805.63	15295.43604	1.0633	0.7598
Q7SIG6	Asap2	Arf-GAP with SH3 domain, ANK repeat and PH domain-containing protein 2	15789.7041	15476.07	15978.63	12754.44	14999.71118	14699.68	12302.56	15082.09	14344.67	14107.24902	1.0633	0.3964
Q8BL97	Srsf7	Serine/arginine-rich splicing factor 7	76508.375	63078.46	76299.83	75298.42	72796.27148	68044.49	71442.45	77204.8	57187.84	68469.89355	1.0632	0.4470
Q8CAL5	Gpc5	Glypican-5	171620.4063	175463.2	152619.4	166447.3	166537.5508	167908.4	175940	136851.5	146011.3	156677.8125	1.0629	0.3806
Q8VE11	Mtmr6	Myotubularin-related protein 6	13199.44043	10890.15	15345.73	14985.27	13605.14697	13176.04	12793.19	12873.91	12357.6	12800.18408	1.0629	0.4656
Q8CAK1	Iba57	Putative transferase CAF17 homolog, mitochondrial	14405.74609	12780.62	17239.29	16414.79	15210.1084	13762.65	12862.63	14048.63	16566.83	14310.18677	1.0629	0.5085
Q8R464	Cadm4	Cell adhesion molecule 4	483875.0938	440194	565034.7	425718.5	478705.5781	461712.1	427503.2	451563.5	460781.1	450389.9844	1.0629	0.4146
Q9DBG6	Rpn2	Dolichyl-diphosphooligosaccharide--protein glycosyltransferase subunit 2	80616.96875	72229.88	73971.51	75989.23	75701.89648	77084.23	80651.84	65587.29	61574.3	71224.41602	1.0629	0.3955
Q7TMM9	Tubb2a	Tubulin beta-2A chain	5503047.5	5208185	5021979	5277282	5252623.125	4832443	4982939	4563428	5391392	4942550.25	1.0627	0.1711
Q9CZN8	Qrsl1	Glutamyl-tRNA(Gln) amidotransferase subunit A, mitochondrial	13196.13672	16134.51	16006.8	14761.87	15024.82788	14672.77	12926.95	13147.43	15807.08	14138.55762	1.0627	0.3929
Q9CQE3	Mrps17	Small ribosomal subunit protein uS17m	22595.0625	24491.54	25035.65	26791.56	24728.45459	21644.27	23232.47	26405.95	21802.79	23271.36963	1.0626	0.3387
Q61161	Map4k2	Mitogen-activated protein kinase kinase kinase kinase 2	5541.30127	2320.316	4131.963	4335.428	4082.252014	3640.884	4683.481	3424.311	3618.306	3841.745667	1.0626	0.7507
Q8CBG9	Rnf170	E3 ubiquitin-protein ligase RNF170	10119.43457	9354.719	9077.15		9517.101237	7823.414	11907.04		7139.641	8956.697266	1.0626	0.7311
O08917	Flot1	Flotillin-1	91096.97656	87704.74	96096.3	99931.86	93707.46875	86119.32	87884.23	95402.46	83366.14	88193.03906	1.0625	0.1898
P62267	Rps23	Small ribosomal subunit protein uS12	173541.7031	166426.7	158187.9	172663.8	167705.0352	184380.7	170631.4	115788	160607	157851.7832	1.0624	0.5424
Q8BFQ8	Gatd1	Glutamine amidotransferase-like class 1 domain-containing protein 1	46692.47656	48197.43	44922.71	43862.3	45918.73047	44164.13	41772.13	41180.28	45770.92	43221.8623	1.0624	0.1090
Q9JJZ4	Ube2j1	Ubiquitin-conjugating enzyme E2 J1	17354.24219	15844.56	20491.22	38271.29	22990.32959	24164.09	35194.89	12688.28	14526.15	21643.35229	1.0622	0.8601
P62702	Rps4x	Small ribosomal subunit protein eS4	288719.625	297333.9	292123.5	299674	294462.7578	297513.1	298490.5	243644.4	269300.8	277237.2109	1.0621	0.2434
Q9EPK7	Xpo7	Exportin-7	39837.62891	39805.63	34945.88	38163.25	38188.0957	35745.49	37764.79	34929.01	35396.25	35958.88477	1.0620	0.1392
Q8BFZ9	Erlin2	Erlin-2	90575.375	84240.48	92390.19	82258.3	87366.08594	85468.86	84274.38	76420.16	82953.48	82279.2207	1.0618	0.1593
Q6PGL7	Washc2	WASH complex subunit 2	16380.0752	17558.19	17302.58	16409.49	16912.58618	14976.5	14600.82	16267.49	17870.86	15928.91528	1.0618	0.2644
Q148V7	Relch	RAB11-binding protein RELCH	28801.22461	28824.75	29989.7	32655.1	30067.69287	26343.52	30704.23	27232.83	28997.09	28319.41797	1.0617	0.2353
Q99NB8	Ubqln4	Ubiquilin-4	19729.19141	15282.9	16722.03	18093.53	17456.91284	18063.54	14567.29	15521.37	17618.48	16442.67188	1.0617	0.4533
Q9DCC7	Isoc2b	Isochorismatase domain-containing protein 2B	9873.292969	5542.711	5729.994	4602.582	6437.144897	6736.055	6424.472	3862.054	7230.875	6063.364014	1.0616	0.7973
Q8C754	Vps52	Vacuolar protein sorting-associated protein 52 homolog	26776.01953	29519.85	25951.2	25416.84	26915.97607	26526.67	25237.48	25050.81	24599.94	25353.72363	1.0616	0.1697
Q99N89	Mrpl43	Large ribosomal subunit protein mL43	17195.0957	15833.5	16219.68	18120.78	16842.26343	16185.11	16684.19	13642.97	16951.62	15865.97192	1.0615	0.3272
O08739	Ampd3	AMP deaminase 3	55276.72656	51322.73	45927.33	44909.1	49358.97168	53506.64	47882.12	38183.29	46471.86	46510.97754	1.0612	0.5017
A2ASQ1	Agrn	Agrin	29435.5	28279.63	26792.98	22470.06	26744.54443	26859.11	26382.72	23797.19	23767.89	25201.72803	1.0612	0.4076
Q99LN9	Dohh	Deoxyhypusine hydroxylase	11197.40723	11131.82			11164.61279	10466.47	9425.692		11676.65	10522.93945	1.0610	0.5005
Q9R049	Amfr	E3 ubiquitin-protein ligase AMFR	7142.230469	10249.94	8029.089	9417.364	8709.655396	7332.924	8255.612	7002.913	10261.76	8213.301147	1.0604	0.6404
Q07076	Anxa7	Annexin A7	126762.8672	131619.2	145793.1	134862.9	134759.5098	121304.1	128775	125052.6	133214.3	127086.5215	1.0604	0.1591
Q80ZJ6	Zer1	Protein zer-1 homolog	17690.25977	17728.16	12436.53	14597.32	15613.06494	12048.89	15940.7	17271.51	13644.72	14726.45459	1.0602	0.6280
Q91VU0	Fam3c	Protein FAM3C	6917.824707	3837.423	3195.301		4650.182617	5696.708	4974.906	2486.864		4386.159668	1.0602	0.8693
Q5MPP0	Fa2h	Fatty acid 2-hydroxylase	11822.90723	14243.43	15449.41	10774.14	13072.47314	11038.35	13132.36	13275.36	11882.67	12332.18652	1.0600	0.5599
Q9CQ85	Timm22	Mitochondrial import inner membrane translocase subunit Tim22	75696.73438	80223.7	70222.83	74798.56	75235.45508	73170.96	76814.44	68296.37	65648.64	70982.60156	1.0599	0.2355
Q3UPL5	Ag2	Uncharacterized protein C11orf96 homolog	11722.90039	12851.68	14589.89	9866.691	12257.78931	9515.154	8787.074	13920.53	14040.84	11565.90015	1.0598	0.7010
Q99L45	Eif2s2	Eukaryotic translation initiation factor 2 subunit 2	28104.93555	23661.41	24081.77	31821.76	26917.46826	24401.44	36853.47	17467.74	22870.37	25398.25439	1.0598	0.7484
Q60714	Slc27a1	Long-chain fatty acid transport protein 1	84390.50781	87423.59	87237.27	88411.46	86865.70508	80755.25	85222.79	78841.39	83069.48	81972.22656	1.0597	0.0241
Q9CQ75	Ndufa2	NADH dehydrogenase [ubiquinone] 1 alpha subcomplex subunit 2	502462.5938	436861.2	478860.7	541456.6	489910.2656	447579.3	508386.6	450606.5	442707	462319.8359	1.0597	0.3428
Q9D706	Rpap3	RNA polymerase II-associated protein 3	7883.175781			7420.848	7652.011719		6626.671	8079.126	6958.886	7221.561198	1.0596	0.5203
Q9CWJ9	Atic	Bifunctional purine biosynthesis protein ATIC	125385.1172	128562.8	129392.1	137701.4	130260.3691	117571.9	126546.9	125662.1	121999.9	122945.2324	1.0595	0.0703
Q8BTX9	Hsdl1	Inactive hydroxysteroid dehydrogenase-like protein 1	98490.8125	85073.28	87297.67	92422.32	90821.02148	83433.03	84367.7	92036.27	83074.09	85727.77344	1.0594	0.2135
P50428	Arsa	Arylsulfatase A	13879.48633	16578.75	18678.52	18849.53	16996.56934	14491.72	16064.47	15246.03	18373.81	16044.01025	1.0594	0.5308
Q6A068	Cdc5l	Cell division cycle 5-like protein	17531.63281	17319.84	17666.73	20932.22	18362.60596	19846.79	18699.28	16666.86	14128.05	17335.24512	1.0593	0.5246
Q00493	Cpe	Carboxypeptidase E	75445.45313	64519.17	69450.95	79430.38	72211.48535	62861.07	76153.02	70906.11	62772.17	68173.0918	1.0592	0.4172
Q9CQN6	Tmem14c	Transmembrane protein 14C	24396.00586	28875.8	35946.01	30187.43	29851.31055	24193.41	34620.83	25656.07	28258.2	28182.12695	1.0592	0.6324
P45952	Acadm	Medium-chain specific acyl-CoA dehydrogenase, mitochondrial	157937.2031	151712.2	173385.8	178207.5	165310.6992	161518.8	175416.1	150068.8	137317.3	156080.2461	1.0591	0.4028
P61202	Cops2	COP9 signalosome complex subunit 2	56802.6875	49246.35	57764	55006.92	54704.98828	52072.66	55027	50548.96	48961.36	51652.49414	1.0591	0.2333
Q6PEE2	Ctif	CBP80/20-dependent translation initiation factor	13135.98438	12398.1	12259.95	11962.07	12439.02466	12512.28	11149.33	13141.67	10176.97	11745.06421	1.0591	0.3680
Q9CXE7	Tmed5	Transmembrane emp24 domain-containing protein 5	7324.383301	8329.202	5350.868	4780.834	6446.321899	5462.467	4854.063	2078.772	11953.57	6087.218628	1.0590	0.8784
Q7TMB8	Cyfip1	Cytoplasmic FMR1-interacting protein 1	105369.7734	106456.6	112450.8	106738.6	107753.9531	103534.5	103261.1	103992.9	96223.88	101753.0977	1.0590	0.0492
Q9CQX8	Mrps36	Alpha-ketoglutarate dehydrogenase component 4	60920.25781	56042.21	67521.66	65900.56	62596.17383	57222.74	61420.57	59908.32	57903.64	59113.81836	1.0589	0.2551
Q8BHE3	Atcay	Caytaxin	61843.23828	46596.26	51769.94	62696.17	55726.40137	55830.4	44036.53	57383.95	53261.26	52628.0332	1.0589	0.5531
Q8CIB5	Fermt2	Fermitin family homolog 2	52412.64844	52946.41	53523.11	52935.22	52954.34668	53026.48	49341.19	48010.95	49664.79	50010.84961	1.0589	0.0357
Q8R550	Sh3kbp1	SH3 domain-containing kinase-binding protein 1	16409.14063	16146.21	12276.06	16898.59	15432.49878	15212.37	12514.55	15498	15077.51	14575.60596	1.0588	0.5247
O55026	Entpd2	Ectonucleoside triphosphate diphosphohydrolase 2	34026.44531	33730.43	42118.48	34945.54	36205.22461	35415.11	37053.86	32107.95	32210.66	34196.89258	1.0587	0.4226
P38060	Hmgcl	Hydroxymethylglutaryl-CoA lyase, mitochondrial	31253.6875	29468.14	25139.38	26557.61	28104.70459	29216.2	30178.99	28412.35	18392.76	26550.07617	1.0586	0.6309

Q99KX1	Mlf2	Myeloid leukemia factor 2	9726.212891	11541.95	5321.666	12916.63	9876.614136	10584.92	8583.576	6255.472	11898.28	9330.563354	1.0585	0.7999
P49722	Psma2	Proteasome subunit alpha type-2	157849.4375	165541.9	180860.3	197467.1	175429.6563	154851.4	160752.8	171935.7	175618.8	165789.6836	1.0581	0.3725
Q9DAS9	Gng12	Guanine nucleotide-binding protein G(I)/G(S)/G(O) subunit gamma-12	285811.1875	283169.8	280229.5	282549.3	282939.9297	272292.2	282128.6	253467	261701.6	267397.3359	1.0581	0.0498
P14869	Rplp0	Large ribosomal subunit protein uL10	212997.5156	222164.8	193761.2	211765.4	210172.2188	207376.2	204267.3	185291.7	197576.8	198628	1.0581	0.1843
Q9CXV1	Sdhd	Succinate dehydrogenase [ubiquinone] cytochrome b small subunit, mitoc	65615.39063	62198.41	63913.22	61125.86	63213.2207	61355.74	66635.9	48332.96	62647.34	59742.98438	1.0581	0.4283
Q9CRC9	Gnpda2	Glucosamine-6-phosphate isomerase 2	46801.60547	43499.29	45536.67	48963.97	46200.38379	44411.56	43321.58	42371.88	44552.65	43664.41797	1.0581	0.0896
Q6ZQB6	Ppip5k2	Inositol hexakisphosphate and diphosphoinositol-pentakisphosphate kinas	25946.88086	24068.82	28538.49	23003	25389.30029	23043.61	23870.16	24338.18	24732.92	23996.21826	1.0581	0.3135
Q60936	Coq8a	Atypical kinase COQ8A, mitochondrial	16903.94727	14799.88	16964.43	16580.46	16312.1814	14425.69	11938.42	17134.23	18172.68	15417.75513	1.0580	0.5711
Q9DC63	Fbxo3	F-box only protein 3	14242.13281	13819.2	10979.35	14301.18	13335.46509	11050.78	13861.28	11395.84	14112.48	12605.09448	1.0579	0.5413
Q6NZL0	Soga3	Protein SOGA3	59274.67578	51599.41	56662.03	60233.57	56942.41992	54658.37	57209.66	49822.01	53641.72	53832.93945	1.0578	0.2545
Q6PGF7	Exoc8	Exocyst complex component 8	38902.64453	38118.91	37630.79	47720.41	40593.18945	34285.93	38174.54	37919.25	43131.74	38377.86328	1.0577	0.4884
Q9QYR9	Acot2	Acyl-coenzyme A thioesterase 2, mitochondrial	20303.47461	22819.71	29986.98	22820.56	23982.68164	24042.85	21051.42	24444.75	21160.1	22674.77783	1.0577	0.5866
Q9CYN9	Atp6ap2	Renin receptor	6678.352539	7502.381	7453.738	6751.435	7096.476685	6899.17	7961.402	6498.276	5481.64	6710.121948	1.0576	0.5149
P51830	Adcy9	Adenylate cyclase type 9	27924.4375	27789.11	26386.95	28633.72	27683.55273	27890.16	27328.19	24126.18	25362.27	26176.69922	1.0576	0.1792
O88487	Dync1i2	Cytoplasmic dynein 1 intermediate chain 2	74619.32031	70975.8	62708	81366.16	72417.32129	66530.58	71875.97	66943.51	68562.84	68478.22266	1.0575	0.3707
Q8VCR7	Abhd14b	Putative protein-lysine deacylase ABHD14B	21906.50977	21074.97	29132.92	22752.94	23716.83691	18616.21	23450.1	24077.39	23572.85	22429.13574	1.0574	0.5860
P46978	Stt3a	Dolichyl-diphosphooligosaccharide--protein glycosyltransferase subunit ST	17298.16602	16847.5	19597.01	15318.45	17265.27979	14545.24	19830.88	16962.16	13973.49	16327.93994	1.0574	0.5798
O35841	Api5	Apoptosis inhibitor 5	25978.36719	22588.08	27009.14	27718.16	25823.43799	24898.91	24608.14	23838.77	24351.77	24424.40039	1.0573	0.2725
Q9JKS5	Habp4	Intracellular hyaluro-binding protein 4	16833.08984	10594.09	12888.98	11629.62	12986.44702	14282.32	11559.91	12403.54	10886.33	12283.02417	1.0573	0.6660
P63044	Vamp2	Vesicle-associated membrane protein 2	192548.3438	201979.5	193566.7	225972.1	203516.6641	202086.7	207545.9	165256	195092.8	192495.3242	1.0573	0.4020
P49615	Cdk5	Cyclin-dependent kinase 5	219592.9844	199084.4	201012.5	210336.6	207506.625	191916.5	203319.8	190312.2	199558.9	196276.8711	1.0572	0.0937
Q7TNR6	Igsf21	Immunoglobulin superfamily member 21	9167.241211	8474.868	9618.143	9527.65	9196.975586	7260	10807.45	10675.18	6055.072	8699.423462	1.0572	0.7004
Q6PDQ2	Chd4	Chromodomain-helicase-DNA-binding protein 4	18712.72852	17693.66	17530.91	18357.02	18073.57959	17110.07	18649	16443.34	16186.64	17097.26392	1.0571	0.1656
Q60829	Ppp1r1b	Protein phosphatase 1 regulatory subunit 1B	39393.54688	31783.37	36701.94	29687.52	34391.59424	27474.85	31190.29	38546.28	32931.98	32535.85059	1.0570	0.5832
P97390	Vps45	Vacuolar protein sorting-associated protein 45	31796.9375	34915.29	34411.21	31618.59	33185.50586	31351.02	32249.02	32468.92	29524.71	31398.41699	1.0569	0.1523
P51410	Rpl9	Large ribosomal subunit protein uL6	111429.1953	109946.8	111482.3	121218.4	113519.1816	97044.4	108550.2	120455.3	103576	107406.4629	1.0569	0.3156
Q99P47	Cntnap4	Contactin-associated protein-like 4	9052.691406	816.1556	3063.95	3368.387	4075.296036	3276.963	8020.565	1329.517	2796.459	3855.876099	1.0569	0.9263
Q78JW9	Ubfd1	Ubiquitin domain-containing protein UBFD1	37294.60156	43840.56	38328.81	37848.06	39328.00977	35190.79	34063.95	41605.67	37996.88	37214.32227	1.0568	0.3869
Q6X893	Slc44a1	Choline transporter-like protein 1	189476	203952.2	199405	185668.3	194625.375	202862.1	180287.1	171028.9	182567.2	184186.332	1.0567	0.2366
Q62442	Vamp1	Vesicle-associated membrane protein 1	518144.0313	496088.1	525849.1	523060.7	515785.4844	502052.8	524838.2	454811.7	471094.3	488199.2422	1.0565	0.1568
P62141	Ppp1cb	Serine/threonine-protein phosphatase PP1-beta catalytic subunit	98768.19531	93863.4	90985.33	91816.27	93858.29883	90333.2	89807.75	80730.41	94508.75	88845.02539	1.0564	0.1892
P47758	Srprb	Signal recognition particle receptor subunit beta	12215.52051	11502.79	12348.15	13711.95	12444.60229	12785.86	11102.71	12150.54	11080.93	11780.01099	1.0564	0.3268
Q9JHU9	Isyna1	Inositol-3-phosphate synthase 1	89791.32031	87373.12	92930.41	95602.36	91424.30078	88247.73	86885.27	85146.38	85929.2	86552.14453	1.0563	0.0441
Q8R1B4	Eif3c	Eukaryotic translation initiation factor 3 subunit C	67125.42188	65917.59	71689.53	54969.95	64925.62402	68026.83	65819.98	53718.33	58327.39	61473.13477	1.0562	0.5035
O35381	Anp32a	Acidic leucine-rich nuclear phosphoprotein 32 family member A	54722.48828	55033.05	53852.61	67253.19	57715.33398	53621.91	51216.68	55596.25	58153.39	54647.05469	1.0561	0.4160
Q8BTS4	Nup54	Nuclear pore complex protein Nup54	8015.814941	6284.711	6781.574	6640.407	6930.626587	6861.575	6147.189	7429.038	5811.967	6562.442261	1.0561	0.5075
Q9D328	Tmem35a	Novel acetylcholine receptor chaperone	71323.34375	72572.89	67770.52	72163.55	70957.57422	61483.7	72655.48	70105.14	64513.53	67189.46094	1.0561	0.2234
Q9DB60	Prxl2b	Prostamide/prostaglandin F synthase	57010.17578	62165.3	56775.54	59559.11	58877.53125	66855.35	61003.12	42383.2	52805.94	55761.90039	1.0559	0.5888
Q6NV83	U2surp	U2 snRNP-associated SURP motif-containing protein	11455.69043	12025.73	6903.101	10065.11	10112.40881	9212.338	11780.97	9198.562	8118.444	9577.579468	1.0558	0.7128
O54931	Akap2	A-kinase anchor protein 2	5147.631348	7029.756	6155.214	6477.374	6202.493774	6195.603	5894.109	5428.732	5979.44	5874.471069	1.0558	0.4715
Q923D2	Blvrb	Flavin reductase (NADPH)	70699.3125	80972.73	95804.37	78387.65	81466.01563	75977.99	81728.29	58736.92	92189.8	77158.25	1.0558	0.6400
Q8BGD9	Eif4b	Eukaryotic translation initiation factor 4B	100939.9922	95627.7	117510.1	105867.3	104986.2891	97916.8	101283.6	96550.45	101999.5	99437.59766	1.0558	0.2961
P62874	Gnb1	Guanine nucleotide-binding protein G(I)/G(S)/G(T) subunit beta-1	716262.0625	769790.1	823605.8	903178.7	803209.1719	762843.8	750771.2	799173.9	730273.4	760765.5781	1.0558	0.3557
Q91YT8	Tmem63a	CSC1-like protein 1	10727.26758	10314.67	6404.489	10337.47	9445.974731	9960.474	12330.84	6701.544	6795.886	8947.186279	1.0557	0.7788
Q9WVJ3	Cpq	Carboxypeptidase Q	16523.29297	17857.5	19645.59	17227.07	17813.36572	13856.42	15798.92	21494.4	16341.32	16872.76587	1.0557	0.6127
O54984	Get3	ATPase GET3	85232.92188	91416.49	84403.25	88219.27	87317.98242	84636.78	83380.69	78628.64	84206.98	82713.27148	1.0557	0.0720
P41731	Cd63	CD63 antigen		6387.408	9554.939	4343.156	6761.834473	5911.279		6899.232		6405.255371	1.0557	0.8693
O08530	S1pr1	Sphingosine 1-phosphate receptor 1	96507.85938	80274.74	82022.91	95049.65	88463.79102	87519.36	87005.52	80667.21	80025.55	83804.41211	1.0556	0.3596
Q9DB27	Mcts1	Maligt T-cell-amplified sequence 1	65920.78906	63457.3	61417.94	56998.39	61948.60547	54487.45	57620.21	58296.45	64428.03	58708.03613	1.0552	0.2926
Q8BZN6	Dock10	Dedicator of cytokinesis protein 10	17037.56445	15016.77	14935.9	15642.11	15658.08447	16102.16	16130.32	12683.19	14444.74	14840.1001	1.0551	0.4237
Q9Z1X4	Ilf3	Interleukin enhancer-binding factor 3	50756.45703	45047.7	40273.32	42045.65	44530.78125	42372.75	44193.29	38878	43376.18	42205.05469	1.0551	0.4017
P70689	Gjb6	Gap junction beta-6 protein	55060.28125	43680.74	40942.96	42714.96	45599.73633	47012.49	44745.98	40580.58	40535.08	43218.5332	1.0551	0.5310
Q8K2H2	Otud6b	Deubiquitinase OTUD6B	17060.38672	16256.77	19231	17167.38	17428.88257	16412.84	18677.05	14355.84	16629.33	16518.76489	1.0551	0.4346
Q91W90	Txndc5	Thioredoxin domain-containing protein 5	57723.78125	55795.36	66055.83	58679.19	59563.54004	55329.61	55576.08	54330.32	60618.02	56463.50488	1.0549	0.2868
Q9CQB5	Cisd2	CDGSH iron-sulfur domain-containing protein 2	36902.09766	34376.81	45845.21	48112.65	41309.19141	37741.29	43939.15	37569.27	37400.51	39162.55273	1.0548	0.5835
P98191	Cds1	Phosphatidate cytidylyltransferase 1	16493.72461	15106.45	17283.52	12435.85	15329.88794	15460.06	12771.54	15419.47	14487.16	14534.55737	1.0547	0.5439
O88531	Ppt1	Palmitoyl-protein thioesterase 1	26545.71484	26888.13	38072.16	33471.55	31244.3877	27690.89	26798.11	32068.4	31949.53	29626.72949	1.0546	0.6211
Q8R4H9	Slc30a5	Proton-coupled zinc antiporter SLC30A5		16963.34			16963.33594		16085.69			16085.6875	1.0546	
Q9WVK4	Ehd1	EH domain-containing protein 1	48545.14844	48678.21	46532.39	42908.46	46666.05176	46379.97	45282.2	41550.51	43844.11	44264.19922	1.0543	0.2079
Q8VD65	Pik3r4	Phosphoinositide 3-kinase regulatory subunit 4	29241.45508	31259.84	29505.48	30548.48	30138.81445	28901.19	27550.18	29487.8	28413.52	28588.17334	1.0542	0.0470
Q80W21	Gstm7	Glutathione S-transferase Mu 7	75096.09375	74554.73	68447.16	72312.36	72602.58594	71264.57	71776.92	66369.82	66097.53	68877.21094	1.0541	0.1339
Q78JE5	Fbxo22	F-box only protein 22	19884.81055	22293.88	19449.55	23131.13	21189.84521	21183.37	22970.62	18220.35	18040.43	20103.69336	1.0540	0.4956
A2AQ25	Skt	Sickle tail protein	30796.27344	25354.33	29698.84	35495.88	30336.33252	26516.68	29176.01	29801.89	29633.51	28782.021	1.0540	0.5100
Q61165	Slc9a1	Sodium/hydrogen exchanger 1	40774.45703	36088.01	44397.83	39856.29	40279.14648	40697.45	39080.69	34683.79	38400.59	38215.62988	1.0540	0.3698
Q9JI90	Rnf14	E3 ubiquitin-protein ligase RNF14	65399.45313	60985.84	55513.64	63147.51	61261.60938	56459.3	58843.43	58381.22	58821.29	58126.30957	1.0539	0.2025

Q9R1C6	Dgke	Diacylglycerol kinase epsilon	58723.49609	60854.49	60073.79	72651.67	**63075.86133**	60494.05	62734.54	56780.75	59385.54	**59848.71973**	1.0539	0.3859
Q5U4D9	Thoc6	THO complex subunit 6 homolog	11325.01953	14266.77	9456.848	13657.67	**12176.57739**	11990.21	6294.752	15173.21	12756.95	**11553.78003**	1.0539	0.7848
Q68FF6	Git1	ARF GTPase-activating protein GIT1	45773.97656	48581.21	45307.14	42902.67	**45641.24805**	41004.98	43855.31	41945.58	46462.15	**43317.00586**	1.0537	0.2147
Q9Z1L5	Cacna2d3	Voltage-dependent calcium channel subunit alpha-2/delta-3	35520.0625	34426.32	30770.64	37486.84	**34550.96777**	32960.46	33216.19	34751.25	30239.48	**32791.84375**	1.0536	0.3390
Q9JHR7	Ide	Insulin-degrading enzyme	59887.68359	63536.73	55600.6	56424.49	**58862.37598**	46645.04	60913.76	57800.44	58113.41	**55868.15918**	1.0536	0.4419
Q9JLB0	Pals2	Protein PALS2	157258.4219	165961.6	186734.1	167525.9	**169370.0039**	161460.1	160606.6	166330.2	154626.9	**160755.9414**	1.0536	0.2435
O54824	Il16	Pro-interleukin-16	137049.4844	145784.3	141707.8		**141513.8542**	143245.7			125406.9	**134326.2891**	1.0535	0.4032
P41234	Abca2	ATP-binding cassette sub-family A member 2	15986.98047	15768.35	15033.93	18184.39	**16243.41357**	15578.39	14914.25	15372.23	15812.34	**15419.30225**	1.0534	0.2866
Q9R1P3	Psmb2	Proteasome subunit beta type-2	172489.9844	169069.3	175190.2	179087.4	**173959.2266**	165060.4	174340.7	162972	158170.3	**165135.8359**	1.0534	0.0694
P56389	Cda	Cytidine deaminase	48060.82031	47744.95	52115.18	52708.67	**50157.40332**	42025.52	43998.71	52582.89	51854.04	**47615.28906**	1.0534	0.4283
O08848	RO60	RNA-binding protein Ro60	25141.43359	24301.6	25271.97	24538.27	**24813.31982**	25038.01	22366.01	24263.09	22561.86	**23557.24121**	1.0533	0.1197
P61514	Rpl37a	Large ribosomal subunit protein eL43	14966.98438	21257.51	14099.17	19529.19	**17463.21387**	19515.69	21076.56	12698.11	13030.48	**16580.21167**	1.0533	0.7615
Q91W69	Epn3	Epsin-3	36777.66016	30723.4	31162.06	39694.7	**34589.45752**	30685.01	35939.35	30892.62	33851.3	**32842.06787**	1.0532	0.5151
Q6PDH0	Phldb1	Pleckstrin homology-like domain family B member 1			20594.6	12570.92	**16582.76172**	20029.59	8230.595	17722.49	17003.23	**15746.47388**	1.0531	0.8644
Q3UHD6	Snx27	Sorting nexin-27	41808.11328	38997.66	44648.26	44655.83	**42527.4668**	39794.86	45071.58	38962.09	37711.59	**40385.02832**	1.0531	0.3494
Q8CAE9	Podxl2	Podocalyxin-like protein 2	34558.47266	39203.2	39654.05	44747.52	**39540.80859**	51165.95	39989.92	28296.58	30761.57	**37553.50537**	1.0529	0.7344
Q149L6	Dnajb14	DnaJ homolog subfamily B member 14	9047.074219	6217.207	4615.083	5249.569	**6282.233154**	6877.284	7960.94	4743.528	4286.419	**5967.042847**	1.0528	0.8180
Q8CIN4	Pak2	Serine/threonine-protein kinase PAK 2	37920.51172	33569.96	36656.64	33730.8	**35469.47949**	37414.15	33241.3	31034.7	33070.04	**33690.04639**	1.0528	0.3411
O35855	Bcat2	Branched-chain-amino-acid aminotransferase, mitochondrial	21530.95703	18298.8	20970.07	18826.1	**19906.48486**	19911.71	17890.93	19586.81	18249.82	**18909.81885**	1.0527	0.3268
P35436	Grin2a	Glutamate receptor ionotropic, NMDA 2A	13288.05371	9977.164	9931.587	9997.181	**10798.49634**	10902.72	12171.58	9057.081	8900.616	**10258.00024**	1.0527	0.6525
Q9CR00	Psmd9	26S proteasome non-ATPase regulatory subunit 9	91110.1875	84593.73	89885.6	96115.96	**90426.36914**	90263.12	88435.3	84295.09	80612.49	**85901.50195**	1.0527	0.2075
Q6PEB6	Mob4	MOB-like protein phocein	22746.88086	24676.01	26400.57	24387.73	**24552.79639**	25127.94	20296.25	25752.01	22121.26	**23324.3667**	1.0527	0.4399
O55125	Nipsnap1	Protein NipSnap homolog 1	419133.5313	428042.5	428779	502474.8	**444607.4766**	419982.5	413656.8	394727.4	461109.4	**422369.0234**	1.0527	0.3885
P63001	Rac1	Ras-related C3 botulinum toxin substrate 1	766974.75	743097.4	775041.4	805884.5	**772749.5313**	781979.9	724450.8	748739.9	681236.9	**734101.875**	1.0526	0.1709
Q64735	Cr1l	Complement component receptor 1-like protein	13923.34375	13749.86	19232.8	12916.06	**14955.51587**	10530.92	12122.44	18728.09	15451.19	**14208.16016**	1.0526	0.7587
Q9JIX8	Acin1	Apoptotic chromatin condensation inducer in the nucleus	29003.71875	24080.56	28517.59	28546.98	**27537.21191**	25562.19	27205.47	27881.72	24005.54	**26163.72852**	1.0525	0.3793
P27671	Rasgrf1	Ras-specific guanine nucleotide-releasing factor 1	31942.05859	34850.34	30305.42	32688.35	**32446.54053**	31740.45	30582.41	29786.85	31209.72	**30829.85791**	1.0524	0.1685
Q61166	Mapre1	Microtubule-associated protein RP/EB family member 1	91831.0625	89572.13	77757.96	92758.31	**87979.86523**	82218.65	87005.48	79355.93	85811.52	**83597.89258**	1.0524	0.3024
Q8R3B1	Plcd1	1-phosphatidylinositol 4,5-bisphosphate phosphodiesterase delta-1	37112.28125	36328.91	36478.5	37550.9	**36867.65039**	34323.67	33704.54	36795.6	35308.73	**35033.13379**	1.0524	0.0460
O88447	Klc1	Kinesin light chain 1	64025.20703	60437.68	59893.76	66102.05	**62614.67578**	64140.09	66967.66	53367.46	53559.85	**59508.76465**	1.0522	0.4489
Q9CR67	Tmem33	Transmembrane protein 33	131875.6719	142768	119661	124416.6	**129680.3027**	126599.3	128012.8	119004.9	119412.4	**123257.3438**	1.0521	0.2919
Q91Y63	Slc13a3	Na(+)/dicarboxylate cotransporter 3	7445.70459	7089.927	6251.069	7295.676	**7020.594238**	7873.74	7751.714	6460.556	4605.917	**6672.981567**	1.0521	0.6810
O35972	Mrpl23	Large ribosomal subunit protein uL23m	16010.15039	16377.12	15682.36	17168.35	**16309.49512**	18238.47	16999.27	12839.62	13931.78	**15502.28491**	1.0521	0.5596
Q62186	Ssr4	Translocon-associated protein subunit delta	30849.0957	31335.55	29003.63	22969.19	**28539.36719**	31651.49	30300.77	19253.19	27307.06	**27128.12842**	1.0520	0.6907
Q91YP0	L2hgdh	L-2-hydroxyglutarate dehydrogenase, mitochondrial	20418.42578	13415.8	18267.14	19817.26	**17979.65747**	15697.48	18500.66	16646.88	17518.57	**17090.89819**	1.0520	0.6192
O08919	Numbl	Numb-like protein	37664.08203	39601.51	22385.02	42200.83	**35462.86035**	36464.95	36149.02	38080.55	24145.95	**33710.11914**	1.0520	0.7606
B1AWN6	Scn2a	Sodium channel protein type 2 subunit alpha	25712.41016	28803	25356.58	31019.05	**27722.75977**	30913.69	27384.89	22873.71	24240.89	**26353.29346**	1.0520	0.5630
Q9CQR4	Acot13	Acyl-coenzyme A thioesterase 13	192989.5313	174378	210046.2	208618.3	**196508.0273**	178589.2	179650.6	189838.4	199236.4	**186828.6563**	1.0518	0.3540
Q9CRA5	Golph3	Golgi phosphoprotein 3	11407.01172	12061.42	11579.29	12455.54	**11875.81616**	10388.88	10896.92	11760.66	12123.2	**11292.41528**	1.0517	0.2535
P97370	Atp1b3	Sodium/potassium-transporting ATPase subunit beta-3	399401.9688	408159.3	432543.4	379084.4	**404797.2734**	409689.5	383925.3	365092.8	381024	**384932.9063**	1.0516	0.2174
Q9DC07	Nebl	LIM zinc-binding domain-containing Nebulette	110281.8281	111987	95717.36	104833.9	**105705.0371**	106109.9	103153.8	98233.96	94573.77	**100517.8555**	1.0516	0.2899
Q6PGB6	Naa50	N-alpha-acetyltransferase 50	12981.31836	14461.38	11856.38	14516.23	**13453.82642**	10636.7	11179.95	14701.76	14658.11	**12794.12842**	1.0516	0.6215
Q91VR5	Ddx1	ATP-dependent RNA helicase DDX1	177894.8438	166076.3	165170.2	177852.6	**171748.4961**	166820.3	173591.9	149931.3	162967.8	**163327.7969**	1.0516	0.2172
Q3V0K9	Pls1	Plastin-1	38654.52734	35341.23	33290.93	31201.27	**34621.98828**	31894.65	33988.57	34004.14	31813.6	**32925.23926**	1.0515	0.3578
Q80UU9	Pgrmc2	Membrane-associated progesterone receptor component 2	48251.11719	43697.05	44280.74	38411.7	**43660.15234**	44161.73	45260.99	29531.69	47134.58	**41522.24902**	1.0515	0.6530
Q8VHH5	Agap3	Arf-GAP with GTPase, ANK repeat and PH domain-containing protein 3	49848.60547	49106.27	48443.3	46662.02	**48515.05078**	45736.94	44009.44	47962.5	46864.97	**46143.45996**	1.0514	0.0714
Q00623	Apoa1	Apolipoprotein A-I	168595.5625	121416.3	202260.9	192523.6	**171199.1016**	133366.8	120943.5	182860.1	214233.7	**162851.0156**	1.0513	0.7775
Q9D2G2	Dlst	Dihydrolipoyllysine-residue succinyltransferase component of 2-oxoglutara	945928.375	910434.9	1002756	975569.7	**958672.2656**	899938.9	859660.1	883120.8	1005139	**911964.7344**	1.0512	0.2623
Q99L20	Gstt3	Glutathione S-transferase theta-3	29858.65625	31233.32	32104.67	33347.66	**31636.07764**	28347.33	30116.53	30124.92	31792.25	**30095.2583**	1.0512	0.1805
P32020	Scp2	Sterol carrier protein 2	93560.01563	115191.9	128716.3	121197.5	**114666.4336**	120324.5	131014	101017.3	83993.15	**109087.252**	1.0511	0.6798
Q9JK23	Psmg1	Proteasome assembly chaperone 1	16324.26074	16082.57	15860.77		**16089.19987**	13285.36	16254.98	16019.33	15670.58	**15307.5603**	1.0511	0.3831
Q9QXS6	Dbn1	Drebrin	112197.0859	103858	119427.6	125822	**115326.1934**	106320.5	106738.2	112438.3	113406.3	**109725.8184**	1.0510	0.3125
P28661	Septin4	Septin-4	211223.2813	210519	245265.7	236204.9	**225803.1992**	214816.7	204673.5	223511.1	216386.9	**214847.0664**	1.0510	0.2989
Q8CBY8	Dctn4	Dynactin subunit 4	69698.05469	66086.27	65465.63	82012.01	**70815.49219**	74123.68	64925.12	67379.06	63147.06	**67393.72949**	1.0508	0.4793
Q62318	Trim28	Transcription intermediary factor 1-beta	56581.09375	51636.04	52432.39	53924.78	**53643.57617**	49593.17	53017.82	49262.99	52339.33	**51053.3291**	1.0507	0.1232
Q9D289	Trappc6b	Trafficking protein particle complex subunit 6B	16914.24023	20588.96	17640.96	21377.91	**19130.51758**	18012.16	18018.87	17158.74	19640.32	**18207.52148**	1.0507	0.4741
P68368	Tuba4a	Tubulin alpha-4A chain	3786203	3595100	3367641	3478106	**3556762.313**	3449877	3543755	2996157	3550868	**3385164.125**	1.0507	0.3225
Q9DBH5	Lman2	Vesicular integral-membrane protein VIP36	52573.3125	53092.57	50105.8	43604.23	**49843.97949**	47625.61	50767.12	47533.7	43831.98	**47439.60156**	1.0507	0.3908
E9Q3S4	Map3k19	Mitogen-activated protein kinase kinase kinase 19	235043.2344	242493.7	233668.6	239109.9	**237578.8359**	251132.9	226905.6	222112.9	204335.9	**226121.8281**	1.0507	0.2891
Q62432	Smad2	Mothers against decapentaplegic homolog 2	12375.05371	15157.59	18371.56	13996.3	**14975.12891**	15368.68	12421.1	15761.77	13460.43	**14252.99585**	1.0507	0.6460
Q8K009	Aldh1l2	Mitochondrial 10-formyltetrahydrofolate dehydrogenase	15598.71777	18581.31	13648.47	16652.99	**16120.37207**	18048.62	14421.83	15677.44	13227.45	**15343.83569**	1.0506	0.6132
Q9Z306	Slc22a4	Solute carrier family 22 member 4	31333.26758	31635.01	33841.85	37911.28	**33680.35156**	29019.77	35412.47	31599.71	32205.71	**32059.41602**	1.0506	0.4501
Q3UTH8	Arhgef9	Rho guanine nucleotide exchange factor 9	30974.33398	33219.68	18759.67	24552.61	**26876.57324**	26366.94	23631.62	27682.43	24662.09	**25585.77002**	1.0505	0.7166
Q9D8B7	Jam3	Junctional adhesion molecule C	79553.75	72013.48	86665.59	57273.01	**73876.45508**	66155.47	68188.77	72025.05	74952.69	**70330.49609**	1.0504	0.6099
Q8BXR1	Slc7a14	Probable cationic amino acid transporter	29665.1875	27578.45	25739.41	23068.33	**26512.84375**	26325.04	27064.76	21454.61	26129.95	**25243.59033**	1.0503	0.5282

Q05AA6	Drp2	Dystrophin-related protein 2	19242.12891	28446.09	27016.96	3025.149	**19432.58209**	23616.54	12852.52	11269.93	26272.56	**18502.88916**	1.0502	0.8979
P97952	Scn1b	Sodium channel subunit beta-1	38379.31641	32965.28	35700.97	31620.86	**34666.60742**	39776.41	33884.54	26960.32	31413.08	**33008.58643**	1.0502	0.6080
Q91WM2	Hdhd5	Haloacid dehalogenase-like hydrolase domain-containing 5	24823.63672	26748.1	25536.71	26776.05	**25971.12305**	25577.18	23250.9	25708.93	24389.84	**24731.71143**	1.0501	0.1492
P29758	Oat	Ornithine aminotransferase, mitochondrial	193266.4219	178481.2	191106.6	167481.3	**182583.8984**	182006.1	169620.7	168498.5	175359.8	**173871.2773**	1.0501	0.2444
Q9DCV4	Rmdn1	Regulator of microtubule dynamics protein 1	36284.26172	39133.2	32770.81	34343.08	**35632.83887**	34284.71	35194.74	32703.45	33559.58	**33935.61816**	1.0500	0.2919
Q8C419	Gpr158	Metabotropic glycine receptor	32272.66406	31087.72	29157.15	40861.39	**33344.73242**	31452.97	35897.84	29228	30457.07	**31758.97021**	1.0499	0.6122
O35682	Myadm	Myeloid-associated differentiation marker	50850.28906	50009.07	58730.49	48357.36	**51986.80469**	51851.46	47871.33	57606.55	40734.49	**49515.95801**	1.0499	0.5803
Q9JI19	Fibp	Acidic fibroblast growth factor intracellular-binding protein	16888.33984	13740.64		9108.652	**13245.87695**	12827.85	10812	12855.93	13969.98	**12616.44214**	1.0499	0.7706
Q9Z0V2	Kcnd2	Potassium voltage-gated channel subfamily D member 2	60738.27734	56120.67	62735.52	74877.31	**63617.94629**	66797.14	68824.55	54174.94	52583.49	**60595.0293**	1.0499	0.6209
P62242	Rps8	Small ribosomal subunit protein eS8	221225.375	233151	212302.2	206406.3	**218271.2344**	227356.5	228081.6	188369.4	187957.8	**207941.3047**	1.0497	0.4510
Q9Z1D1	Eif3g	Eukaryotic translation initiation factor 3 subunit G	34655.03906	32313.79	29758.67	33966.24	**32673.43652**	30243.45	30612.31	32992.43	30663.92	**31128.02588**	1.0496	0.2650
A2AJA9	Ajm1	Apical junction component 1 homolog	16251.60352	20042.78	18868.82	23066.51	**19557.4292**	16334.61	17150.41	22490.64	18559.22	**18633.72192**	1.0496	0.6548
Q8CBE3	Wdr37	WD repeat-containing protein 37	49060.80078	44359.63	51337.66	55720.5	**50119.64844**	48912.7	46002.91	45803.49	50319.34	**47759.60645**	1.0494	0.4013
Q9QYB5	Add3	Gamma-adducin	87069.13281	79281.82	88677.45	88050.33	**85769.68164**	83549.74	80827.15	82972.36	79575.38	**81731.1582**	1.0494	0.1401
Q63959	Kcnc3	Potassium voltage-gated channel subfamily C member 3	372062.5313	365575.7	396349.2	389519.7	**380876.7656**	378607.8	374432.4	357415.8	341349.5	**362951.3594**	1.0494	0.1600
Q99N96	Mrpl1	Large ribosomal subunit protein uL1m	8207.744141	7800.733	8791.593	7575.62	**8093.922607**	2718.808	8585.653	10958.74	8588.973	**7713.04425**	1.0494	0.8373
P21619	Lmnb2	Lamin-B2	68313.28906	67992.38	78631.24	78594.9	**73382.95117**	67993.34	67492.06	73079.27	71155.6	**69930.06641**	1.0494	0.3356
Q63829	Commd3	COMM domain-containing protein 3	11237.96973	13761.12	15286.53	15397.79	**13920.85034**	10998.67	14749.93	12582.73	14739.42	**13267.68555**	1.0492	0.6410
Q9Z0J4	Nos1	Nitric oxide synthase 1	22426.46484	18941.22	16382.16	18574.88	**19081.18213**	18210.8	17539.87	19496.84	17514.38	**18190.47363**	1.0490	0.5290
Q91VW3	Sh3bgrl3	SH3 domain-binding glutamic acid-rich-like protein 3	53296.05859	56712.39	68971.46	67894.58	**61718.62207**	57924.37	50466.3	63584.48	63379.81	**58838.7373**	1.0489	0.5861
P63024	Vamp3	Vesicle-associated membrane protein 3	193689.8594	187235.3	171018.8	208088.3	**190008.082**	187651.9	177165.7	174145.2	185731.1	**181173.457**	1.0488	0.3308
P28867	Prkcd	Protein kinase C delta type	15259.70313	15133.15	12391.19	9071.476	**12963.87891**	9780.986	15806.02	11571.26	12286.51	**12361.19507**	1.0488	0.7652
O54962	Banf1	Barrier-to-autointegration factor	32526.87695	36294.73	41600.7	36588.76	**36752.76514**	39413.24	35995.39	31377.02	33391.41	**35044.26416**	1.0488	0.5271
Q9DBR4	Apbb2	Amyloid beta precursor protein binding family B member 2	27903.40039	25148.23	19441.96	26278.45	**24693.0083**	28214.39	20548.83	19870.54	25548.43	**23545.54785**	1.0487	0.6880
Q8R127	Sccpdh	Saccharopine dehydrogenase-like oxidoreductase	346454.9063	372741.6	288071.5	301220.4	**327122.0859**	354455.8	350448.5	266233.2	276611.2	**311937.1641**	1.0487	0.6380
Q8BYL4	Yars2	Tyrosine--tRNA ligase, mitochondrial	17204.26758	16860.01	18368.21	10571.25	**15750.93579**	18015.53	14518.77	16820	10734.09	**15022.09741**	1.0485	0.7696
Q99JP7	Ggt7	Glutathione hydrolase 7	65236.88672	62044.64	58748.04	67261.34	**63322.72754**	61588.61	59901.5	58120.6	61963.66	**60393.59375**	1.0485	0.2053
P51150	Rab7a	Ras-related protein Rab-7a	484525.75	496561.5	477272	502212.3	**490142.8828**	504914.1	497148.6	430810.4	437176.9	**467512.5078**	1.0484	0.3068
P39447	Tjp1	Tight junction protein ZO-1	47535.57031	52423.68	59619.21	51667.16	**52811.4043**	53947.48	53545.41	49117.38	44882.56	**50373.20703**	1.0484	0.4871
Q8HW98	Iglon5	IgLON family member 5	52781.25	51445.3	49433.42	56640.08	**52575.0127**	47707.03	51499.26	49555.38	51845.89	**50151.8877**	1.0483	0.2261
Q8BVQ5	Ppme1	Protein phosphatase methylesterase 1	96667.82813	95614.97	85705.08	85773.77	**90940.41211**	83702.3	90905.57	75224.92	97203.36	**86759.03711**	1.0482	0.4841
P62281	Rps11	Small ribosomal subunit protein uS17	155758.5625	170909	143928.3	134057.1	**151163.2617**	146425.7	158559.1	131306.4	140560.9	**144213.0313**	1.0482	0.5038
Q8BZF8	Pgm5	Phosphoglucomutase-like protein 5	10847.07715	11098.28	6283.083	7798.947	**9006.846191**	9464.404	9977.441	6186.649	8745.969	**8593.615967**	1.0481	0.7848
A2A8U2	Tmem201	Transmembrane protein 201	11459.31738	12872.43	15691.36	11349.86	**12843.24243**	13552.54	14777.74	8385.649	12300.27	**12254.05005**	1.0481	0.7428
P63158	Hmgb1	High mobility group protein B1	132188.6719	139831.5	119909	121443.2	**128343.0977**	132041.2	123186.8	119745.8	114873.5	**122461.8223**	1.0480	0.3600
P80318	Cct3	T-complex protein 1 subunit gamma	310304.1563	309698.7	312001.7	313829.8	**311458.5703**	301523.7	302231.2	282308	302764	**297206.7109**	1.0480	0.0305
Q8BNU0	Armc6	Armadillo repeat-containing protein 6	29619.36523	28819.34	24724.84	30304.58	**28367.0332**	27854.94	29237.76	24644.58	26541.23	**27069.62646**	1.0479	0.4452
O35218	Cpsf2	Cleavage and polyadenylation specificity factor subunit 2	4446.643066	5273.304	3247.531	3940.212	**4226.922485**	5401.844	4003.131	2212.486	4517.498	**4033.73938**	1.0479	0.8164
Q6ZPJ3	Ube2o	(E3-independent) E2 ubiquitin-conjugating enzyme UBE2O	96597.10156	95784.26	95356.24	98283.33	**96505.23242**	94850.27	99506.31	84658.97	89374.2	**92097.43555**	1.0479	0.2294
Q99J77	s	Sialic acid synthase	73536.41406	71528.47	76061.13	71764.15	**73222.54102**	70907.09	71149.47	69948.43	67518.59	**69880.89648**	1.0478	0.0464
Q8BRF7	Scfd1	Sec1 family domain-containing protein 1	31534.66797	34679.16	33753.71	32433.74	**33100.32031**	34512.43	33554.9	31615.65	26676.09	**31589.76855**	1.0478	0.4521
Q99J39	Mlycd	Malonyl-CoA decarboxylase, mitochondrial	16122.19922	12116.99	13336.39	14897.85	**14118.35791**	14534.18	12570.8	15996.48	10800.17	**13475.40771**	1.0477	0.6697
Q80V26	Bpnt2	Golgi-resident adenosine 3',5'-bisphosphate 3'-phosphatase	24316.40039	19809.49	25703.66	26789.26	**24154.70264**	20171.17	24804.17	27557.13	19696.07	**23057.13623**	1.0476	0.6681
Q8K363	Ddx18	ATP-dependent RNA helicase DDX18	5076.765137		3873.746		**4475.255615**			4272.018		**4272.017578**	1.0476	
P61358	Rpl27	Large ribosomal subunit protein eL27	327834.0313	308228.3	308565	314209.4	**314709.1953**	316668.4	321033.4	309386.7	254659	**300436.8672**	1.0475	0.4098
P80313	Cct7	T-complex protein 1 subunit eta	176606.1719	187015.8	181508.5	191864.2	**184248.6719**	185349.3	177343.6	176219.9	164811.6	**175931.1055**	1.0473	0.1724
Q3UVL4	Vps51	Vacuolar protein sorting-associated protein 51 homolog	45289.17578	44455.37	44320.55	42984.44	**44262.38574**	43618.11	41627.58	42807.41	41009.49	**42265.64941**	1.0472	0.0383
P52623	Uck1	Uridine-cytidine kinase 1	21345.2832	22447.21	18404.33	25481.08	**21919.47656**	21275.13	22920.91	19915.29	19613.78	**20931.2749**	1.0472	0.5701
Q9DAM5	Slc25a19	Mitochondrial thiamine pyrophosphate carrier	20870.75781	18313.73	17232.62	20278.7	**19173.95166**	20290.9	17019.18	18822.54	17105.82	**18309.60938**	1.0472	0.4813
Q9ERD7	Tubb3	Tubulin beta-3 chain	861606.875	1048959	1036296	939132.4	**971498.5781**	912922.8	928346.3	975754.7	893969.8	**927748.3906**	1.0472	0.3918
O88736	Hsd17b7	3-keto-steroid reductase/17-beta-hydroxysteroid dehydrogenase 7	11217.23535	16020.38	10345.23	11410.72	**12248.39087**	14898.47	11029.09	11804.81	9067.478	**11699.96167**	1.0469	0.7661
Q8R086	Suox	Sulfite oxidase, mitochondrial	25390.85156	26423.5	30377.63	26096.74	**27072.17969**	29572.67	28369.77	23259.78	22240.65	**25860.71826**	1.0468	0.5922
A2APV2	Fmnl2	Formin-like protein 2	90350.21094	99223.94	87679.84	80560.43	**89453.60547**	96080.48	83850.08	78412.98	83470.16	**85453.42578**	1.0468	0.4854
Q921F4	Hnrnpll	Heterogeneous nuclear ribonucleoprotein L-like	18837.55859	19180.86	17002.12	18055.74	**18269.07031**	17909.63	17387.15	17470.78	17044.46	**17453.00537**	1.0468	0.1643
P31938	Map2k1	Dual specificity mitogen-activated protein kinase kinase 1	130062.1484	119551.3	123133.9	126458.4	**124801.4316**	113601.3	120070.3	119954.4	123311.4	**119234.3867**	1.0467	0.1160
Q69ZS7	Hbs1l	HBS1-like protein	11032.97656	9003.42	8566.47	9840.304	**9610.79248**	9125.73	8923.434	9435.681	9244.82	**9182.41626**	1.0467	0.4682
P14873	Map1b	Microtubule-associated protein 1B	671048.375	666275.2	777201.2	741879.4	**714101.0313**	695929.1	663834.2	689410.9	680401.8	**682393.9688**	1.0465	0.3022
Q64012	Raly	RNA-binding protein Raly	36695.52734	32560.03	37282.74	40064.54	**36650.70898**	31455.85	33506.27	41125.4	34013.14	**35025.16553**	1.0464	0.5571
Q921W4	Cryzl1	Quinone oxidoreductase-like protein 1	17573.4668	17364.52	17414.06	20993.17	**18336.30664**	17514.21	21154.11	17589.84	13839.06	**17524.30713**	1.0463	0.6566
P46412	Gpx3	Glutathione peroxidase 3	6195.699219	6215.601	4914.144	6479.642	**5951.271484**		5024.646	8262.86	3778.007	**5688.504639**	1.0462	0.8344
Q3TIX9	Usp39	Ubiquitin carboxyl-terminal hydrolase 39	12978.66406	12911.23	11928.73	12789.1	**12651.93188**	13958.3	12678.95	12868.86	8870.867	**12094.24585**	1.0461	0.6413
Q922Q1	Mtarc2	Mitochondrial amidoxime reducing component 2	85549.73438	103702.1	110887.4	102233.5	**100593.1875**	86078.62	96024.32	96960.21	105602.4	**96166.38477**	1.0460	0.5324
Q6ZQ29	Taok2	Serine/threonine-protein kinase TAO2	39446.96875	51840.57	44996.7	48973.93	**46314.54297**	43514.2	39200.73	49397.44	45018.49	**44282.71387**	1.0459	0.5732
Q61730	Il1rap	Interleukin-1 receptor accessory protein	34909.86328	28156.14	10425.94	23776.91	**24317.21655**	19073.65	28646.76	23435.19	21849.73	**23251.33203**	1.0458	0.8539
Q7TPS5	C2cd5	C2 domain-containing protein 5	8232.84082	12901.87	9993.076	11537.27	**10666.26392**	12785.75	10720.66	8974.089	8319.555	**10200.01367**	1.0457	0.7535

Q8C0E2	Vps26b	Vacuolar protein sorting-associated protein 26B	93967.71094	98839	81015.88	92101.84	91481.10547	91021.11	93796.38	79004.48	86113.93	87483.97461	1.0457	0.4519
Q8CFA2	Amt	Aminomethyltransferase, mitochondrial	29431.39648	30445.3	30366.98	34010.86	31063.63379	26788.18	32441.48	32833.16	26766.66	29707.36914	1.0457	0.5172
Q3TJZ6	Fam98a	Protein FAM98A	41259.44531	35874.77	31500.28	37306.11	36485.15234	38952.72	34878.16	32125.29	33621.47	34894.40967	1.0456	0.5466
Q6PGH2	Jpt2	Jupiter microtubule associated homolog 2	41972.50781	37581.95	25471.78	29966.8	33748.25781	35937.62	33473.11	30187.63	29512.37	32277.68262	1.0456	0.7257
Q64676	Ugt8	2-hydroxyacylsphingosine 1-beta-galactosyltransferase	69679.89063	62971.73	55621.29	45939.21	58553.03027	65419.79	62222.96	47451.6	48930.25	56006.15039	1.0455	0.7225
P98086	C1qa	Complement C1q subcomponent subunit A	38808.75781	28031.41	21173.31	37196.5	31302.49512	27261.7	31811.61	33037.9	27671.29	29945.62598	1.0453	0.7670
Q4KML4	Abracl	Costars family protein ABRACL	95552.53906	71602.77	79954.68	90044.4	84288.59766	77267.63	90682.91	77755.86	76834.99	80635.34766	1.0453	0.5825
Q9CQF4	Mtres1	Mitochondrial transcription rescue factor 1	14340.2207	15868.51	13013.13	13242.06	14115.97949	12537.79	14756.83	11592.26	15135.43	13505.57568	1.0452	0.5915
Q8K411	Pitrm1	Presequence protease, mitochondrial	42890.69141	32829.1	37152.52	35912.05	37196.09277	31180.3	37744.88	39521.98	33903.59	35587.68799	1.0452	0.5894
Q9WV55	Vapa	Vesicle-associated membrane protein-associated protein A	237527.6406	209306.8	208843.9	240438.8	224029.2734	222594.3	236951.3	192981.3	204848.7	214343.8711	1.0452	0.4840
O54734	Ddost	Dolichyl-diphosphooligosaccharide--protein glycosyltransferase 48 kDa sul	83738.05469	73003.78	85410.08	81241.73	80848.41016	75799.75	85771.23	71909.34	75934.79	77353.7793	1.0452	0.4202
Q9ESZ8	Gtf2i	General transcription factor II-I	19834.38086	19409.55	16002.99	13502	17187.23096	15586.78	16998.3	17411.58	15781.81	16444.61987	1.0452	0.6517
P61759	Vbp1	Prefoldin subunit 3	55605.95703	46206.06	43790.68	46155.35	47939.51172	53016.13	48208.09	37274.56	44980.58	45869.83691	1.0451	0.6410
Q9D1M0	Sec13	Protein SEC13 homolog	23470.41992	24280.7	25196.49	25513.07	24615.16846	26346.32	24099.31	22439.82	21327.26	23553.17969	1.0451	0.4048
Q3U7U3	Fbxo7	F-box only protein 7	25224.16406	28440.87	27802.76	29110.31	27644.5249	25323.12	24968.45	27738.93	27777.99	26452.12256	1.0451	0.3353
P97823	Lypla1	Acyl-protein thioesterase 1	52166.70313	49441.24	62046.46	63661.21	56828.90332	43197.23	46435.05	61836.39	66089.95	54389.65332	1.0448	0.7265
O70133	Dhx9	ATP-dependent RNA helicase A	84018.5	87269.25	83960.76	94455.83	87426.08398	88537.55	91654.65	80921.48	73612.13	83681.44922	1.0447	0.4593
O70443	Gnaz	Guanine nucleotide-binding protein G(z) subunit alpha	167142.5313	158295.2	161839.7	189159.6	169109.2422	160923.6	163112.9	161214.6	162222.5	161868.3945	1.0447	0.3373
Q9Z2G9	Htatip2	Oxidoreductase HTATIP2	11117.42578	11682.58	13104.24	14286.78	12547.75488	14775.84	9656.598	11446.08	12166.4	12011.23169	1.0447	0.6897
P57759	Erp29	Endoplasmic reticulum resident protein 29	157370.7969	143520.1	149139.2	140221.3	147562.8398	152849.1	139755	140116.5	132363.4	141271.0156	1.0445	0.3097
Q62384	Zpr1	Zinc finger protein ZPR1	6666.495605	4648.214	7053.581	7059.715	6357.001343	5332.625		6829.33	6096.592	6086.182129	1.0445	0.7402
Q8BWT5	Dip2a	Disco-interacting protein 2 homolog A	70621.27344	69752.32	64813.91	74542.59	69932.52344	66265.68	65048.24	67615.23	68927.26	66964.10156	1.0443	0.2200
Q9EQG9	Cert1	Ceramide transfer protein	9089.941406	7322.373	7885.583	11699.26	8999.289673	9242.816	9205.759	7549.501	8471.762	8617.459473	1.0443	0.7288
Q9CW03	Smc3	Structural maintece of chromosomes protein 3	16226.3584	17755.9	17770.18	15333.91	16771.58813	17376.26	15113.16	15026.49	16727.12	16060.75562	1.0443	0.4300
A2ARP1	Ppip5k1	Inositol hexakisphosphate and diphosphoinositol-pentakisphosphate kinas	9065.662109	7320.79	7309.298	5745.166	7360.229126	7749.412	3829.5	9729.878	6884.985	7048.443726	1.0442	0.8314
Q61831	Mapk10	Mitogen-activated protein kinase 10	60671.96484	77047.15	66488.96	65901.12	67527.29785	63121.17	65473.48	72830.35	57282.45	64676.86328	1.0441	0.5668
P08226	Apoe	Apolipoprotein E	134994.7188	135454.4	140549.3	151047.5	140511.4883	136610	135227.7	121664.8	144836.1	134584.6445	1.0440	0.3673
Q9QZB7	Actr10	Actin-related protein 10	49830.10547	50047.15	46196.8	53514.31	49897.09082	49804.22	50952.25	47694.71	42719.14	47792.58203	1.0440	0.4060
Q9Z0V8	Timm17a	Mitochondrial import inner membrane translocase subunit Tim17-A	13852.4209	11907.62	13433.1	14962.23	13538.84131	14450.32	13258.98	12173.64	11989.23	12968.04248	1.0440	0.5268
P14206	Rpsa	Small ribosomal subunit protein uS2	347433.6563	339097.3	351138	341103.6	344693.1563	345750.7	359962	283870.8	331342.4	330231.4453	1.0438	0.4212
Q9JKP5	Mbnl1	Muscleblind-like protein 1	40882.78516	47406.24	34472.39	44631.82	41848.31055	40803.59	42266.21	39794.7	37508.5	40093.24902	1.0438	0.5763
O35943	Fxn	Frataxin, mitochondrial	16389.35742	20084.83	22655.73	24674.78	20951.1748	21581.84	18991.4	22113.57	17607.13	20073.48584	1.0437	0.6880
Q60676	Ppp5c	Serine/threonine-protein phosphatase 5	87840.63281	79063.45	76229.06	88716.48	82962.40625	81430.58	82580.92	78423.79	75528.69	79490.99414	1.0437	0.3605
B7ZMP1	Xpnpep3	Xaa-Pro aminopeptidase 3	4390.486816	3195.921	1866.617	5412.635	3716.414886		3444.739	2864.869	4374.208	3561.271891	1.0436	0.8801
O35226	Psmd4	26S proteasome non-ATPase regulatory subunit 4	122520.6719	137482.6	132177.5	126337	129629.4648	130032.6	127435.4	114937.6	124478.1	124220.9355	1.0435	0.2893
Q9EQ80	Nif3l1	NIF3-like protein 1	88289.07031	99699.36	86385.73	91160.84	91383.75	94797.98	94495.61	68636.6	92363.84	87573.50586	1.0435	0.6051
Q3UH66	Wnk2	Serine/threonine-protein kinase WNK2	31563.36523	31511.59	23847.21	30488.65	29352.7041	34248.25	32626.55	25341.96	20299.46	28129.0542	1.0435	0.7547
Q91VT4	Cbr4	3-oxoacyl-[acyl-carrier-protein] reductase	41314.69531	48398.92	54832.38	45246.02	47448.00488	47503.61	43782.41	48072.54	42524.25	45470.70215	1.0435	0.5553
P97930	Dtymk	Thymidylate kinase	31075.80273	31661.3	28183.38	31282.1	30550.64551	32387.25	30068	23917.68	30737.4	29277.58203	1.0435	0.5511
Q3UIL6	Plekha7	Pleckstrin homology domain-containing family A member 7	10207.05957	5592.945	4317.737		6705.913737	11274.04		4362.629	3645.237	6427.303304	1.0433	0.9309
Q8VEK0	Tmem30a	Cell cycle control protein 50A	41640.78516	38442.02	36804.56	41614.41	39625.44238	37404.02	38597.61	36171.64	39754.54	37981.9502	1.0433	0.2939
Q8C181	Mbnl2	Muscleblind-like protein 2	49987.76172	52822.4	55691.66	44754.37	50814.04688	46937.78	48387.61	42674.39	56828.16	48706.9834	1.0433	0.5967
P17809	Slc2a1	Solute carrier family 2, facilitated glucose transporter member 1	426020.0625	411905.1	502388.6	465258.6	451393.0781	473044.9	462437.6	398115.2	397293.1	432722.7031	1.0431	0.5409
Q8VCH8	Ubxn4	UBX domain-containing protein 4	16117.71484	25481.07	17694.35	12902.38	18048.87939	18707.36	17341.13	15607.19	17557.4	17303.26978	1.0431	0.7951
Q9WTR5	Cdh13	Cadherin-13	42605.77734	32814.11	38228	34307.08	36988.74316	33784.09	30629.41	40680.24	36751.55	35461.32129	1.0431	0.6360
Q8C0N2	Gpat3	Glycerol-3-phosphate acyltransferase 3	12242.6377	8958.64	11408.11	8584.231	10298.4043	10387.27	10616.53	10522.24	7970.855	9874.223633	1.0430	0.7138
P12382	Pfkl	ATP-dependent 6-phosphofructokinase, liver type	173441.5469	178776.3	185623	183060.3	180225.2734	170036.3	178277.8	167472.6	175442.2	172807.2148	1.0429	0.0872
P33173	Kif1a	Kinesin-like protein KIF1A	81371.46094	82432.11	76160.21	85506.77	81367.63672	76373.56	77905.23	76591.17	81210.57	78020.13477	1.0429	0.1861
O09131	Gsto1	Glutathione S-transferase omega-1	54913.73047	57465.83	49340.78	57529.58	54812.48047	49787.76	53220.68	56686.96	50553.4	52562.20117	1.0428	0.3984
Q149F3	Gspt2	Eukaryotic peptide chain release factor GTP-binding subunit ERF3B	31453.70508	36156.54	33153.95	39128.55	34973.18311	35938.3	39771.12	25994.41	32458.45	33540.56982	1.0427	0.6864
P13439	Umps	Uridine 5'-monophosphate synthase	10974.35547	11441.4	9752.143	12733.86	11225.43823	10132.31	10858	12643.49	9437.605	10767.85254	1.0425	0.6382
P62482	Kcnab2	Voltage-gated potassium channel subunit beta-2	256297.2813	269310.7	281255.5	292707.5	274892.7422	249698	261537.6	270849.8	272743.1	263707.1328	1.0424	0.2807
Q8VDN2	Atp1a1	Sodium/potassium-transporting ATPase subunit alpha-1	1479105	1496187	1618610	1621729	1553907.719	1548945	1503541	1435576	1474928	1490747.406	1.0424	0.2121
Q9D1R1	Tmem126b	Complex I assembly factor TMEM126B, mitochondrial	12057.43262	11659.75			11858.5918	11376.74				11376.73926	1.0424	
P18052	Ptpra	Receptor-type tyrosine-protein phosphatase alpha	54366.51953	51061.85	49739.33	53539.41	52176.77832	50468.56	53198.67	48874.68	47691.86	50058.44141	1.0423	0.2347
Q61838	Pzp	Pregcy zone protein	43243.10156	33572.78	56213.37	52078.63	46276.9707	40106.09	41633.5	46231.14	49623.45	44398.5459	1.0423	0.7432
Q91WT9	Cbs	Cystathionine beta-synthase	26411.99414	23588.57	21662.77	23390.91	23763.56348	21949.98	20885.88	25413.5	22947.23	22799.14893	1.0423	0.5107
O88485	Dync1i1	Cytoplasmic dynein 1 intermediate chain 1	92076.03125	84373.39	80212.82	77149.8	83453.01172	79388.14	77841.36	73769.4	89298.05	80074.23633	1.0422	0.4918
Q5SSH8	Cyb5d2	Neuferricin	12279.0752	11346.51	13084.55	11611.18	12080.32959	11396.48	13354.64	12965.35	8649.326	11591.44995	1.0422	0.6821
P62880	Gnb2	Guanine nucleotide-binding protein G(I)/G(S)/G(T) subunit beta-2	1478664.125	1520439	1580407	1649509	1557254.688	1490282	1434563	1495122	1557066	1494258.281	1.0422	0.2094
Q9EQ06	Hsd17b11	Estradiol 17-beta-dehydrogenase 11	126679.6641	122468.1	138704	120910.9	127190.6777	129121.9	120530.1	124935.4	113636.1	122055.875	1.0421	0.3626
Q9DCN2	Cyb5r3	NADH-cytochrome b5 reductase 3	133571.2188	136060.8	185934.8	127716.1	145820.748	145363.2	136201.1	149929.7	128324.9	139954.7227	1.0419	0.6963
Q9D023	Mpc2	Mitochondrial pyruvate carrier 2	212387.7813	211062.2	224596.5	237360.3	221351.6836	220417.7	222372.8	196656.2	210420.3	212466.7617	1.0418	0.3366
Q9D1G5	Lrrc57	Leucine-rich repeat-containing protein 57	128604.6094	107739.4	117030.2	125874.6	119812.1934	127980.5	118386.2	106746.1	106900.7	115003.3691	1.0418	0.5154
O70311	Nmt2	Glycylpeptide N-tetradecanoyltransferase 2	8300.708008	7709.158	10325.73	6327.825	8165.855103	6392.838	4717.497	12284.38	7961.08	7838.949707	1.0417	0.8636

Q6P3A8	Bckdhb	2-oxoisovalerate dehydrogenase subunit beta, mitochondrial	27649.53125	26638.37	22097.15	21691.68	**24519.18164**	22960.28	22751.84	24513.18	23939.74	**23541.2583**	1.0415	0.5603
Q8C0P5	Coro2a	Coronin-2A	44873.27734	48716.64	48295.78	50151.26	**48009.23926**	44757.94	49706.91	45724.5	44215.05	**46101.10156**	1.0414	0.2970
P07934	Phkg1	Phosphorylase b kinase gamma catalytic chain, skeletal muscle/heart isofo	14060.10645	10539.06	13278.06	13410.63	**12821.96436**	7926.272	10337.38	14654.16	16343.77	**12315.39551**	1.0411	0.8161
Q9D906	Atg7	Ubiquitin-like modifier-activating enzyme ATG7	23151.08398	23688.66	23452.42	23975.17	**23566.8335**	22701.13	27562.14	22419.94	17863.2	**22636.60205**	1.0411	0.6565
Q9EST5	Anp32b	Acidic leucine-rich nuclear phosphoprotein 32 family member B	47324.66406	55347.91	46030.44	51393.95	**50024.23828**	48902.26	49247.63	49479.63	44573.99	**48050.87793**	1.0411	0.4443
O08749	Dld	Dihydrolipoyl dehydrogenase, mitochondrial	663038.625	665160.4	720278.1	726334.8	**693702.9844**	686274.8	681268	617759.1	680066.4	**666342.0781**	1.0411	0.2907
P08551	Nefl	Neurofilament light polypeptide	8005098	8259368	10172242	7828251	**8566239.625**	7989679	8361462	8051427	8512505	**8228768.125**	1.0410	0.5666
Q9DB50	Ap1s2	AP-1 complex subunit sigma-2	57538.79688	56297.48	49303.21	63705.74	**56711.30664**	53352.3	58835.11	49055.5	56667.61	**54477.63184**	1.0410	0.5621
Q99JB2	Stoml2	Stomatin-like protein 2, mitochondrial	59261.43359	57015.1	54938.23	74958.78	**61543.3877**	56396.27	60943.86	59275.96	59863.84	**59119.9834**	1.0410	0.6217
P53026	Rpl10a	Large ribosomal subunit protein uL1	167396.1719	171700.3	166923.4	167769.5	**168447.3398**	169668.5	166162.3	151629.1	159831.1	**161822.7461**	1.0409	0.1582
Q9DBL1	Acadsb	Short/branched chain specific acyl-CoA dehydrogenase, mitochondrial	71814.66406	70904.72	72135.83	73507.01	**72090.55469**	67643.91	68724.31	71255.34	69415.26	**69259.70703**	1.0409	0.0228
P99024	Tubb5	Tubulin beta-5 chain	2129221.5	2040065	2217361	2323384	**2177507.781**	2162449	2136570	2231626	1838812	**2092364.031**	1.0407	0.4522
Q91XD7	Creld1	Protein disulfide isomerase Creld1	30118.70703	31112.96	28316.24	23853.76	**28350.41602**	30235.13	27059.21	21376.34	30299.87	**27242.63818**	1.0407	0.6895
P51660	Hsd17b4	Peroxisomal multifunctional enzyme type 2	220568.8906	212640.4	195358.9	208209.2	**209194.3438**	212312.4	229004.1	178328.6	184452.5	**201024.3672**	1.0406	0.5534
P70168	Kpnb1	Importin subunit beta-1	185090.875	194564.8	183587.6	194719.6	**189490.7344**	185711.8	185680.1	171872.9	185121	**182096.4258**	1.0406	0.1542
Q9JHS3	Lamtor2	Ragulator complex protein LAMTOR2	17934.92578	24061.19	15190.75	16023.3	**18302.54248**	21212.82	21285.16	11660.2	16198.96	**17589.28516**	1.0406	0.8232
Q99KF1	Tmed9	Transmembrane emp24 domain-containing protein 9	101230.5781	90683.62	98547.98	76857.2	**91829.84375**	83164.09	86053.44	83165.31	100625.4	**88252.06055**	1.0405	0.6219
Q7TMQ7	Wdr91	WD repeat-containing protein 91	22638.78906	21408.39	17417.03	20106.99	**20392.79883**	17570.78	19508.22	18388.45	22934.22	**19600.41455**	1.0404	0.6433
Q8R527	Rhoq	Rho-related GTP-binding protein RhoQ	4917.442383	6168.604	6474.196	10604.58	**7041.205933**	5334.819	5464.668	7239.925	9032.645	**6768.014282**	1.0404	0.8625
Q9CQ54	Ndufc2	NADH dehydrogenase [ubiquinone] 1 subunit C2	311641.5	296149.1	300180.3	312523.6	**305123.625**	312825.7	310289.8	267565.5	282584.3	**293316.3047**	1.0403	0.3527
Q9QZB0	Rgs17	Regulator of G-protein signaling 17	28643.96875	26439.69		28122.01	**27735.22135**	21250.48	30224.76		28514.19	**26663.14258**	1.0402	0.7241
P70268	Pkn1	Serine/threonine-protein kinase N1	6937.70459	7822.032	11898.96	8844.089	**8875.695068**	9178.966	7467.675	7906.486	9581.098	**8533.556152**	1.0401	0.7837
Q99JR1	Sfxn1	Sideroflexin-1	64019.38281	56262.74	60972.94	67379.63	**62158.6709**	64796.95	59869.48	55194.32	59190.34	**59762.77344**	1.0401	0.4655
Q8BFR6	Zfand1	AN1-type zinc finger protein 1	9214.606445	13054.82	8252.722	7865.176	**9596.831299**		10625.29	7044.187	10013.81	**9227.760905**	1.0400	0.8350
O55135	Eif6	Eukaryotic translation initiation factor 6	30947.95898	29277.08	34741.9	21890.76	**29214.42334**	29499.42	31987.26	28270.8	22610.43	**28091.97754**	1.0400	0.7488
P97822	Anp32e	Acidic leucine-rich nuclear phosphoprotein 32 family member E	20209.8457	18256.96	17902.68	19371.63	**18935.27979**	16333.99	17274.98	19785.48	19446.7	**18210.28784**	1.0398	0.4913
O35409	Folh1	Glutamate carboxypeptidase 2	81395.39063	80847.35	93043.67	94522.38	**87452.19727**	77962.61	82395.58	82755.61	93307.79	**84105.39648**	1.0398	0.5205
Q922R8	Pdia6	Protein disulfide-isomerase A6	184486.0313	176243.5	196426.8	172109.8	**182316.5156**	193671.3	174584.4	163438.8	169671.1	**175341.3789**	1.0398	0.4403
Q80TT2	Baiap3	BAI1-associated protein 3	10308.76563	8286.323	6617.791	9242.584	**8613.865845**	8903.449	9411.541	8505.856	6319.951	**8285.199341**	1.0397	0.7622
Q99MU3	Adar	Double-stranded RNA-specific adenosine deaminase	9183.009766	9117.78	9378.966	10378.51	**9514.567627**	11725.38	9203.559	8868.396	6810.095	**9151.857056**	1.0396	0.7415
E9Q912	Rap1gds1	Rap1 GTPase-GDP dissociation stimulator 1	465814.75	493279	476590.4	408500.3	**461046.1016**	489310	447839.2	438721.5	398027.1	**443474.4531**	1.0396	0.5282
Q61941	Nnt	NAD(P) transhydrogenase, mitochondrial	62948.17578	61389.14	72884.74	64688.18	**65477.55859**	62404.82	60951.7	64921.57	63657.79	**62983.97168**	1.0396	0.3908
P01631		Ig kappa chain V-II region 26-10			4377.419		**4377.419434**			4211.112		**4211.111816**	1.0395	
P41216	Acsl1	Long-chain-fatty-acid--CoA ligase 1	47794.46875	47489.47	48699.73	44388.09	**47092.94043**	48586.52	47486.96	46018.2	39148.61	**45310.07324**	1.0393	0.4710
P46061	Rangap1	Ran GTPase-activating protein 1	18071.79102	19745.17	18720.56	21449.49	**19496.75244**	21561.07	17490.07	17816.2	18168.58	**18758.98242**	1.0393	0.5605
Q3UZP4	Svip	Small VCP/p97-interacting protein	53068.87109	60008.09	131868.4	62365.14	**76827.63281**	58058.39	57235.72	87328.55	93073.97	**73924.15527**	1.0393	0.8933
P42859	Htt	Huntingtin	29899.57422	29083.86	29498.29	32025.49	**30126.80078**	28620.19	28134.23	29489.49	29713.9	**28989.45117**	1.0392	0.1811
Q9CZT8	Rab3b	Ras-related protein Rab-3B	39127.77344	33552.53	37790.69	37120.33	**36897.83008**	36386	32064.98	38312.64	35259.71	**35505.83398**	1.0392	0.4614
Q99LI8	Hgs	Hepatocyte growth factor-regulated tyrosine kinase substrate	53174.46484	69067.41	61270.61	58232.56	**60436.26172**	61681.97	49941.65	58964.45	62043.99	**58158.0166**	1.0392	0.6203
Q80TM9	Nisch	Nischarin	41323.67969	38783.04	45952.76	40819.84	**41719.83008**	41278.25	35608.61	41116.83	42588.51	**40148.04883**	1.0391	0.4953
Q3ULF4	Spg7	Paraplegin	22352.41211	22220.52	20574.29	22473.99	**21905.30176**	21415.1	21971.87	20783.35	20155.72	**21081.5083**	1.0391	0.2153
Q9CPR4	Rpl17	Large ribosomal subunit protein uL22	129641.2266	145003.5	136066.4	130566.6	**135319.4492**	136571.3	132285.9	134776.5	117291.5	**130231.291**	1.0391	0.4017
Q6NS52	Dgkb	Diacylglycerol kinase beta	14861.28711	11528.03	14159.56	13087.45	**13409.07861**	15371.32	14667.12	10534.3	11047.58	**12905.07886**	1.0391	0.7367
Q8K021	Scamp1	Secretory carrier-associated membrane protein 1	123125.5781	121092.7	113045.4	126959.5	**121055.793**	123689.5	122660.1	110165.8	109560.3	**116518.9238**	1.0389	0.3848
Q9D6Z1	Nop56	Nucleolar protein 56	34169.02734	33274.68	30073.94	31565.78	**32270.85742**	34075.19	32723.08	30078.07	27375	**31062.83496**	1.0389	0.5135
O54901	Cd200	OX-2 membrane glycoprotein	77869.15625	63226.8	64069.2	66528.42	**67923.39551**	74005.41	63334.52	59590.01	64600.9	**65382.70996**	1.0389	0.5983
Q9CY66	Gar1	H/ACA ribonucleoprotein complex subunit 1	4866.393066	8995.465	7031.82	8836.173	**7432.462646**		4054.272	11218.27	6192.681	**7155.075928**	1.0388	0.9006
Q6NZR5	Skic2	Superkiller complex protein 2	23904.48633	18231.44	19553.09	20700.96	**20597.49365**	17848.79	19538.03	19208.83	22719.82	**19828.86963**	1.0388	0.6462
Q9WVE8	Pacsin2	Protein kinase C and casein kinase substrate in neurons protein 2	74701.98438	85032.55	88331.02	81272.91	**82334.61523**	73476.5	85658.6	81604.6	76332.13	**79267.95703**	1.0387	0.4713
Q99JH7	Clstn3	Calsyntenin-3	14023.38965	10911.46	12800.71	13012.38	**12686.98462**	13694.16	11608.02	12062.49	11494.51	**12214.79419**	1.0387	0.5876
Q3UUI3	Them4	Acyl-coenzyme A thioesterase THEM4	182682.0313	183471.9	173460.4	185417.3	**181257.8867**	172931.5	179140.7	180068.9	165985	**174531.5313**	1.0385	0.1611
P18760	Cfl1	Cofilin-1	2440497.25	2540208	2506758	2411219	**2474670.375**	2273178	2352179	2369572	2536544	**2382868.125**	1.0385	0.1939
P28741	Kif3a	Kinesin-like protein KIF3A	11769.50977	12976.25	11324.67	11458.81	**11882.30981**	10691.72	12916.19	11721.38	10441.83	**11442.78271**	1.0384	0.5407
Q9QYJ3	Dnajb1	DnaJ homolog subfamily B member 1	38800.43359	41217.57	34249.22	43433.23	**39425.1123**	39890	37501.2	33674.2	40814.86	**37970.06348**	1.0383	0.5864
Q9CYZ2	Tpd52l2	Tumor protein D54	102514.9531	91513.92	99131.21	94415.65	**96893.93359**	90405.53	94784.11	94831.02	93258.37	**93319.75586**	1.0383	0.2269
Q62446	Fkbp3	Peptidyl-prolyl cis-trans isomerase FKBP3	96646.30469	101319.2	105664.8	85876.91	**97376.78711**	100751.4	89662.76	86165.59	98568.6	**93787.07813**	1.0383	0.5384
O70172	Pip4k2a	Phosphatidylinositol 5-phosphate 4-kinase type-2 alpha	141583.8594	139192.8	132543.5	134494.5	**136953.6484**	132461.3	135169.8	129613.2	130412.7	**131914.2207**	1.0382	0.0827
Q5FWK3	Arhgap1	Rho GTPase-activating protein 1	62681.83984	57300.31	58703.7	66480.05	**61291.47656**	59006.5	58162.72	59415.87	59566.46	**59037.88672**	1.0382	0.3234
Q4VA53	Pds5b	Sister chromatid cohesion protein PDS5 homolog B	9822.557617	3295.951	13739.37	12500.13	**9839.501221**	8971.819	5430.997	9854.725	13656.61	**9478.538086**	1.0381	0.9043
Q8C033	Arhgef10	Rho guanine nucleotide exchange factor 10	19256.27539	17885.16	19781.17	16865.49	**18447.02393**	16876.27	19689.74	17839.65	16676.72	**17770.59766**	1.0381	0.5052
P19096	Fasn	Fatty acid synthase	281555.0625	298756.5	289135.8	292782.3	**290557.3984**	284328.6	291445.1	272230.3	271631.9	**279908.9688**	1.0380	0.1275
P83882	Rpl36a	Large ribosomal subunit protein eL42	31113.14648	24619.72	35265.02	40386.09	**32845.99414**	25104.77	49404.74	22169.07	29896	**31643.64209**	1.0380	0.8689
Q64737	Gart	Trifunctional purine biosynthetic protein adenosine-3	55658.66797	58152.4	60710.12	56932.17	**57863.33887**	56349.35	58240.4	53492.11	54911.26	**55748.28027**	1.0379	0.2029
Q60967	Papss1	Bifunctional 3'-phosphoadenosine 5'-phosphosulfate synthase 1	32412.75	34409.01	20033.37	20431.39	**26821.62939**	32945.18	30383.01	18889.09	21150.42	**25841.92529**	1.0379	0.8552
Q8VHX6	Flnc	Filamin-C	55369.46484	52633.58	42794.11	37802.84	**47149.99902**	52355.67	45540.05	39832.98	44006.42	**45433.78027**	1.0378	0.7369

Q9D5V5	Cul5	Cullin-5	32711.19531	33768.21	30928.35	31061.35	32117.27783	29849.2	30572.9	30912.73	32467.35	30950.54688	1.0377	0.2325
Q9JIF7	Copb1	Coatomer subunit beta	29910.26953	29998.1	28675.45	28970.95	29388.69189	30798.41	27450.26	27849.88	27189.63	28322.0459	1.0377	0.2809
O08583	Alyref	THO complex subunit 4	25968.04297	25155.79	23255.17	23657.57	24509.14111	21579.55	26278.46	23621.68	23015.55	23623.80908	1.0375	0.4781
Q8C8R3	Ank2	Ankyrin-2	210441.75	204168	206963.7	227656	212307.3789	214862.7	197031.3	199630.3	207034.3	204639.6367	1.0375	0.2913
Q9CX13	Cnih4	Protein cornichon homolog 4	32006.29102	25962.35	26212.02	20699.66	26220.07959	27174.54	29989.06	18158.44	25771.95	25273.49561	1.0375	0.7915
Q9D7A6	Srp19	Signal recognition particle 19 kDa protein	18335.16797	19739.22	17439.72	21656.79	19292.72754	20599.82	15109.26	19914.77	18761.34	18596.29932	1.0374	0.6649
Q80VP1	Epn1	Epsin-1	59676.17969	59943.05	57193.34	69252.98	61516.38477	65166.15	59888.38	56238.18	55891.76	59296.11426	1.0374	0.5400
P57780	Actn4	Alpha-actinin-4	37778.08984	37682.88	43793.03	34078.7	38333.17383	39516.8	36718.21	35640.59	35928.11	36950.92773	1.0374	0.5528
A2AJI0	Map7d1	MAP7 domain-containing protein 1	94887.57031	92887.23	95147.79	89392.96	93078.88867	92184.2	81500.65	93664.4	91560.45	89727.42188	1.0374	0.3181
Q7TNG5	Eml2	Echinoderm microtubule-associated protein-like 2	103020.0781	103340.1	112090.1	109510.9	106990.3086	102136.6	105369.2	103378.5	101701.2	103146.3379	1.0373	0.1615
Q8CIQ7	Dock3	Dedicator of cytokinesis protein 3	7911.477051	8816.322	8424.93	5167.742	7580.117676	7334.242	6901.988	8317.755	6680.781	7308.691528	1.0371	0.7735
Q6ZPJ0	Tex2	Testis-expressed protein 2	22328.26758	22579.29	33300.05	19174.99	24345.65088	25781.52	26436.51	24730.93	16949.02	23474.49365	1.0371	0.8258
Q9CQM5	Txndc17	Thioredoxin domain-containing protein 17	101054.125	112813.1	112501.6	108568.7	108734.3926	105710.9	99043.02	93956.71	120685.2	104848.9512	1.0371	0.5669
Q9D7V2	Lysmd2	LysM and putative peptidoglycan-binding domain-containing protein 2	12084.94043	14053.18	9493.951	7223.132	10713.80188	12669.27	13205.2	7618.107	7831.49	10331.01733	1.0371	0.8628
P62748	Hpcal1	Hippocalcin-like protein 1	153174.8438	163245	171688.5	160396.1	162126.1094	162221.5	154795.5	153952.8	154370.5	156335.0781	1.0370	0.2271
P80314	Cct2	T-complex protein 1 subunit beta	485310.9063	470857.7	499403.6	480775	484086.7969	467615.2	469056.3	471002	459579.7	466813.2813	1.0370	0.0364
P46935	Nedd4	E3 ubiquitin-protein ligase NEDD4	32883.91797	31904.28	34413.49	35040.06	33560.43701	34005.84	32838.46	29777.38	32834.64	32364.07861	1.0370	0.3394
Q9QXA5	Lsm4	U6 snRNA-associated Sm-like protein LSm4	23794.51563	28416.46	21370.55	30543.77	26031.32422	31626.34	25527.63	20577.69	22681.96	25103.40381	1.0370	0.7807
Q99KK7	Dpp3	Dipeptidyl peptidase 3	45797.07422	47611.29	45676.18	44839.5	45981.01172	44168.72	44859.04	40058.89	48283	44342.41113	1.0370	0.3942
Q9QZB9	Dctn5	Dynactin subunit 5	64090.49609	73772.63	73813	76509.51	72046.40723	76151.36	76542.38	57089.21	68134.99	69479.4834	1.0369	0.6463
P60202	Plp1	Myelin proteolipid protein	17391584	17266080	17199124	17270362	17281787.5	17317686	16645877	16761727	15947586	16668219	1.0368	0.0742
Q80VP0	Tecpr1	Tectonin beta-propeller repeat-containing protein 1	27785.3457	25092.14	24816.88	26215.91	25977.56689	26020.91	24678.12	24692.83	24847.43	25059.8208	1.0366	0.2655
Q8BU33	Ilvbl	2-hydroxyacyl-CoA lyase 2	38176.62891	44570.36	43491.69	46119.82	43089.62402	43698.3	42224.8	42899.61	37462.71	41571.35547	1.0365	0.5200
P47915	Rpl29	Large ribosomal subunit protein eL29	107636.1484	42037.31	86326.05	94926.24	82731.43945	95778.04	92828.88	53206.29	77477.96	79822.79395	1.0364	0.8717
P23506	Pcmt1	Protein-L-isoaspartate(D-aspartate) O-methyltransferase	175167.8438	165969.5	171788.1	177324.5	172562.4688	171001.7	164659.2	163669.5	166697.8	166507.0586	1.0364	0.0869
Q63932	Map2k2	Dual specificity mitogen-activated protein kinase kinase 2	46054.05469	43225.89	41971.71	45089.81	44085.36523	44550.88	42374.07	35864.2	47366.14	42538.8252	1.0364	0.5757
Q62448	Eif4g2	Eukaryotic translation initiation factor 4 gamma 2	42174.73047	39319.9	38311.07	43686.31	40873.00391	39114.34	42779.07	38394.41	37479.89	39441.92871	1.0363	0.4328
Q922V4	Plrg1	Pleiotropic regulator 1	16936.45703	14771.09	14941.1	19402.03	16512.66846	9219.907	22230.03	17343.14	14945.8	15934.71875	1.0363	0.8492
Q8VBW6	Nae1	NEDD8-activating enzyme E1 regulatory subunit	62484.69922	63185.73	57764.42	55982.96	59854.45313	59104.23	56234.3	62367.94	53352.32	57764.7002	1.0362	0.4550
Q61699	Hsph1	Heat shock protein 105 kDa	256795.9688	244096.4	213162.5	219550.7	233401.3867	240425.9	231647.6	206949	222019.8	225260.5703	1.0361	0.5395
P62823	Rab3c	Ras-related protein Rab-3C	48466.34766	46159.43	35637.27	46697.65	44240.17285	47478.78	44650.5	38239.46	40423.53	42698.06738	1.0361	0.6812
O88851	Rbbp9	Putative hydrolase RBBP9	27120.66016	31003.45	27921.04	33036.81	29770.48975	30588.22	27168.83	28687.78	28486.62	28732.86377	1.0361	0.5264
O54916	Reps1	RalBP1-associated Eps domain-containing protein 1	15019.44238	15456.44	13950.62	12697.97	14281.11743	15036.1	14458.75	13742.11	11897.39	13783.58765	1.0361	0.6076
E9QAT4	Sec16a	Protein transport protein Sec16A	8698.582031	6991.167	6827.102	7800.73	7579.395386	7301.741	6900.433	8257.041	6803.191	7315.601685	1.0361	0.6442
Q91Z61	Diras1	GTP-binding protein Di-Ras1	96352.57031	91980.56	91867.45	98348.42	94637.25195	85362.71	90679.83	95942.98	93389.57	91343.77344	1.0361	0.2815
Q9CX56	Psmd8	26S proteasome non-ATPase regulatory subunit 8	59034.72266	57919.57	60037.7	63079.62	60017.90332	56532.19	58717.89	54916.72	61558.08	57931.21875	1.0360	0.2942
Q9D0J4	Arl2	ADP-ribosylation factor-like protein 2	56132.16016	53860.13	57148.09	47115.89	53564.06738	56025.98	50434.69	55229.44	45122.64	51703.18652	1.0360	0.6019
Q9CZ42	Naxd	ATP-dependent (S)-NAD(P)H-hydrate dehydratase	53286.28516	51368.59	53897.54	49198.52	51937.73438	52619.22	52360.26	46031.71	49533.02	50136.05176	1.0359	0.3717
Q3UNZ8	Cryzl2	Quinone oxidoreductase-like protein 2	41069.53516	43061.48	52942.51	49691.58	46691.27637	44601.25	41820.34	49203.82	44670.36	45073.94238	1.0359	0.6285
O70591	Pfdn2	Prefoldin subunit 2	69001.47656	64964.34	73363.51	82616.86	72486.54688	67217.32	65085.4	67965.22	79645.52	69978.36328	1.0358	0.6345
Q61696	Hspa1a	Heat shock 70 kDa protein 1A	31457.5918	31230.93	39939.39	30203.85	33207.94141	30040.83	31083.29	32002.25	35114.91	32060.31738	1.0358	0.6638
Q9JIG7	Ccdc22	Coiled-coil domain-containing protein 22	44825.37109	40369.56	38913.08	40663.96	41192.99414	37265.64	40127.96	42571.67	39124.18	39772.36328	1.0357	0.4311
Q80U23	Snph	Syntaphilin	52508.28906	58790.63	51396.64	59400.69	55524.0625	55876.34	54627.3	50729.55	53242.92	53619.02734	1.0355	0.4490
Q9Z0E0	Ncdn	Neurochondrin	502193.875	551871.4	467008.2	535850.6	514231.0078	498552.5	502320.8	482985.2	502584.9	496610.8438	1.0355	0.3988
Q9JLC8	Sacs	Sacsin	30504.31641	30649.97	30532.07	29262.72	30237.26953	28514.64	28570.78	29125.08	30597.95	29202.1123	1.0354	0.1271
Q5RJI5	Brsk1	Serine/threonine-protein kinase BRSK1	29306.03516	26689.69	29111.91	30135.08	28810.67969	27943.15	28243.1	26507.01	28606.07	27824.83154	1.0354	0.3014
Q8VD66	Abhd4	(Lyso)-N-acylphosphatidylethanolamine lipase	37797.60938	35515.2	32675.28	31778.29	34441.5957	36849.05	35596.19	29410.13	31199.05	33263.60303	1.0354	0.6173
Q62348	Tsn	Translin	35215.76563	36857.96	37451.09	37123.52	36662.08496	33426.32	37059.21	35534.29	35615.23	35408.76074	1.0354	0.2123
P60766	Cdc42	Cell division control protein 42 homolog	360083.6875	365457.8	416916.3	423450.3	391477.0469	379154.7	375262.8	373611	384356.3	378096.2031	1.0354	0.4570
Q03157	Aplp1	Amyloid beta precursor like protein 1	57078.01953	47963.23	45900.27	32863.4	45951.23145	55639.52	44816.8	34855.97	42214.98	44381.81641	1.0354	0.8198
P62774	Mtpn	Myotrophin	56332.44141	56894.16	55886.15	60293.9	57351.66504	53899.81	51517.27	66647.8	49584.8	55412.41895	1.0350	0.6430
O88587	Comt	Catechol O-methyltransferase	40223.1875	37493.92	44130.14	34304.96	39038.05273	38038.77	39705.96	37850.08	35282.04	37719.20996	1.0350	0.5833
Q91XM9	Dlg2	Disks large homolog 2	33080.16797	35283.74	32590.09	35946.95	34225.23779	33623.19	32977.06	33052.73	32625.7	33069.66992	1.0349	0.2208
O88455	Dhcr7	7-dehydrocholesterol reductase	27249.22461	32282.86	27782.43	27191.07	28626.39648	29783.52	31142.02	25927.73	23787	27660.06641	1.0349	0.6609
Q8BLQ9	Cadm2	Cell adhesion molecule 2	36109.12109	31610.63	33230.7	38392.47	34835.72998	33615.27	38878.88	26195.26	35964.92	33663.58252	1.0348	0.7186
Q8BH93	Mapk1ip1l	MAPK-interacting and spindle-stabilizing protein-like	18346.78711	21749.27	16734.49	20959.53	19447.5166	19891.41	17771.43	18893.22	18625.46	18795.3833	1.0347	0.6177
P70336	Rock2	Rho-associated protein kinase 2	37695.19141	38293.29	36867.5	39615.07	38117.76367	34887.32	36471.99	38255.19	37749.14	36840.91016	1.0347	0.2268
Q9ESN6	Trim2	Tripartite motif-containing protein 2	114454.3594	118310.9	115516.9	125580.8	118465.7578	109579.6	113637.2	110945	123833.8	114498.9063	1.0346	0.3689
Q9Z2D6	Mecp2	Methyl-CpG-binding protein 2	57739.80469	58749.5	58260.08	62859.72	59402.27539	59529.3	59036.26	55387.02	55713.12	57416.42676	1.0346	0.2597
Q9Z2N8	Actl6a	Actin-like protein 6A	16565.65625	16474.18	11043.73	11018.21	13775.44336	12213.36	14110.41	12871.44	14065.72	13315.23218	1.0346	0.7899
Q9JIF0	Prmt1	Protein arginine N-methyltransferase 1	80274.69531	83192.88	77936.87	93213.64	83654.51953	80370.16	86826.61	84770.25	71483	80862.50391	1.0345	0.5808
P97441	Slc30a3	Probable proton-coupled zinc antiporter SLC30A3	32143.0957	36713.22	34001.95	38703.28	35390.38721	35975.18	36432.06	36219.02	28220.91	34211.79102	1.0344	0.6500
Q9CXW4	Rpl11	Large ribosomal subunit protein uL5	141251.2969	185740.7	200125	218205.2	186330.5508	179399	214616.8	177283.4	149215.9	180128.7852	1.0344	0.7797
Q61171	Prdx2	Peroxiredoxin-2	558841.375	608893.8	644720.1	684697.3	624288.1406	610898.8	641123.6	590463.6	571895.9	603595.4688	1.0343	0.5239
Q9Z0S1	Bpnt1	3'(2'),5'-bisphosphate nucleotidase 1	127713.4766	134719.7	129797.9	134320.7	131637.9355	133845.9	128352.8	120042.8	126857.5	127274.75	1.0343	0.2368

P52019	Sqle	Squalene monooxygenase	10346.56543	7807.027	10333.36	6153.325	**8660.068604**	7643.372	9007.065	8387.458	8455.296	**8373.297852**	1.0342	0.7966
P47911	Rpl6	Large ribosomal subunit protein eL6	364342.2813	362691.3	310860.7	358654.4	**349137.1875**	367694.3	363129.2	320632.8	298896.3	**337588.1563**	1.0342	0.6029
Q61543	Glg1	Golgi apparatus protein 1	14235.5127	10386.84	20267.42	21782.78	**16668.13794**	14749.43	14884	15383.38	19452.88	**16117.42383**	1.0342	0.8547
Q9WTQ5	Akap12	A-kinase anchor protein 12	82526.36719	79558.26	98136.37	86891.2	**86778.04883**	78752.7	87189.63	83678.59	86035.52	**83914.10938**	1.0341	0.5465
Q6A0D4	Rftn1	Raftlin	23632.10352	21395.84	19112.47	15849.32	**19997.43262**	19649.86	18374.85	18940.29	20387.33	**19338.08252**	1.0341	0.7145
Q9R0H0	Acox1	Peroxisomal acyl-coenzyme A oxidase 1	24703.31641	24034.84	20401.84	24682.56	**23455.63721**	24185.55	23243.94	21333.65	21978.21	**22685.33984**	1.0340	0.5484
Q9Z2W0	Dnpep	Aspartyl aminopeptidase	108721.9922	115991.8	113308.2	113077.6	**112774.875**	109282.5	108432.8	106474.8	112107.2	**109074.3066**	1.0339	0.1001
Q8R2Q4	Gfm2	Ribosome-releasing factor 2, mitochondrial	14487.52539	15926.94	13898.14	16978.74	**15322.83813**	16111.89	16641.78	13061.38	13465.48	**14820.13428**	1.0339	0.6762
Q9DBT5	Ampd2	AMP deaminase 2	341388.75	364894.1	357220.8	354525.7	**354507.3203**	338306.3	355163.7	336929.3	341117.3	**342879.1406**	1.0339	0.1210
B2RXS4	Plxnb2	Plexin-B2	18873.59961	16910.68	16106.1	15773.77	**16916.03857**	16598.46	17101.59	15926.25	15821.06	**16361.83984**	1.0339	0.4918
Q9R0N3	Syt11	Synaptotagmin-11	9202.731445	8846.38	8971.801	9145.796	**9041.677002**	9857.44	8886.271	9148.694	7091.892	**8746.074219**	1.0338	0.6364
P59279	Rab2b	Ras-related protein Rab-2B	17142.32422	15187.68	15432.85	14064.48	**15456.83301**	16937.72	13605.67	15289.91	13972.69	**14951.49878**	1.0338	0.6268
O88544	Cops4	COP9 signalosome complex subunit 4	108972.8359	111888.5	92415.42	101278.8	**103638.8906**	102986.1	101006.4	100638.9	96378.19	**100252.3965**	1.0338	0.4872
Q8BZX4	Srek1	Splicing regulatory glutamine/lysine-rich protein 1	5314.93457	5108.843	6254.499	5046.869	**5431.286255**	7068.854	5507.739	6253.842	2186.532	**5254.241821**	1.0337	0.8782
Q9QZE5	Copg1	Coatomer subunit gamma-1	16697.4707	14432.28	17724.15	14262.38	**15779.07153**	15062.77	14964.74	14809.63	16222.27	**15264.85327**	1.0337	0.5936
P52479	Usp10	Ubiquitin carboxyl-terminal hydrolase 10	31925.67969	30203.4	29168.69	30689.59	**30496.83789**	29824	28790.48	30333.19	29064.78	**29503.11279**	1.0337	0.1898
O35681	Syt3	Synaptotagmin-3	17396.29883	19099.54	19702.34	17752.4	**18487.64453**	19453.58	16626.28	17468.06	17994.18	**17885.52783**	1.0337	0.4837
P20029	Hspa5	Endoplasmic reticulum chaperone BiP	358200.8125	372678	353829.8	366138.3	**362711.7344**	368751.2	374302.4	329584.8	331075.6	**350928.5078**	1.0336	0.3880
Q8R1X6	Spart	Spartin	33835.41797	36923.79	33628.32	36454.73	**35210.56641**	32322.04	35312.54	32440.36	36193.56	**34067.12207**	1.0336	0.4168
P16460	Ass1	Argininosuccinate synthase	107090.2734	107379.5	122724.4	130576.5	**116942.6602**	110893.8	106512.6	121763.3	113421.6	**113147.8281**	1.0335	0.5891
Q9CQD1	Rab5a	Ras-related protein Rab-5A	147641.75	137551.9	138893.3	151451.8	**143884.6719**	144328.9	136161.7	141625.2	134747.5	**139215.8438**	1.0335	0.2937
Q9D6K8	Fundc2	FUN14 domain-containing protein 2	11863.73926	11718.72	10060.27	10030.32	**10918.26001**	10688.34	9784.434	11491.47	10292.62	**10564.21704**	1.0335	0.5888
O08547	Sec22b	Vesicle-trafficking protein SEC22b	87453.53906	85204.12	98598.64	92434.23	**90922.63281**	95195.43	89587.54	78390.52	88760.77	**87983.56445**	1.0334	0.5459
Q8BGC4	Ptgr3	Prostaglandin reductase-3	76145.75781	75438.91	73909.18	66442.9	**72984.18555**	70767.31	75232.01	68059.2	68441.86	**70625.0957**	1.0334	0.4275
Q9D920	Borcs5	BLOC-1-related complex subunit 5	23261.92773	22970.47	26873.88	19687.32	**23198.3999**	21131.85	25781.99	20338.17	22544.13	**22449.03662**	1.0334	0.7065
Q8K2Q0	Commd9	COMM domain-containing protein 9	12977.71289	14123.17	7606.433	11845.35	**11638.16577**	10976.89	14734.16	9355.005	9984.511	**11262.64136**	1.0333	0.8470
F6SEU4	Syngap1	Ras/Rap GTPase-activating protein SynGAP	21544.45703	21856.36	19109.46	25185.61	**21923.97217**	21499.86	19109.22	22344.49	21913.08	**21216.6626**	1.0333	0.6414
P01942	Hba	Hemoglobin subunit alpha	3304564.75	2937663	3100497	2372992	**2928929.25**	3085899	2833523	2556552	2862278	**2834562.813**	1.0333	0.6927
Q9CR57	Rpl14	Large ribosomal subunit protein eL14	316404.9688	285546.6	281918.4	290612.7	**293620.6641**	302680.5	298486.6	288298.3	247288.7	**284188.5313**	1.0332	0.5494
Q9JME7	Trappc2l	Trafficking protein particle complex subunit 2-like protein	32760.27344	26459.92	29924.02	34619	**30940.80225**	32253.29	33056.69	30506	23976.93	**29948.22803**	1.0331	0.7279
P50136	Bckdha	2-oxoisovalerate dehydrogenase subunit alpha, mitochondrial	22981.41211	23010.93	20604.3	21304.33	**21975.24512**	21464.61	22588.76	20723.11	20305.32	**21270.44971**	1.0331	0.4047
Q9DCL9	Paics	Bifunctional phosphoribosylaminoimidazole carboxylase/phosphoribosylar	157367.25	176674.4	157863.8	159644.1	**162887.3945**	163159.6	159877.7	143619.9	164001.8	**157664.7344**	1.0331	0.4614
Q03173	Enah	Protein enabled homolog	38783.19141	28875.09	32409.02	30743.2	**32702.625**	27509.03	31256.65	33278.46	34580.57	**31656.17822**	1.0331	0.7063
Q9WTX2	Prkra	Interferon-inducible double-stranded RNA-dependent protein kinase activa	28268.49609	25251.91	26693.54	31188.5	**27850.61328**	27440.5	24685.09	28072.57	27639.73	**26959.47119**	1.0331	0.5708
Q9QY42	Gpr37	Prosaposin receptor GPR37	42291.53125	41828.63	25415.4	31578.91	**35278.61621**	36843.71	35748.25	30513.12	33498.86	**34150.9834**	1.0330	0.8039
Q62426	Cstb	Cystatin-B	97815.80469	89822.36	93629.53	106928.9	**97049.1582**	91495.34	89433.78	91879.23	102985.5	**93948.45703**	1.0330	0.5408
P35802	Gpm6a	Neuronal membrane glycoprotein M6-a	167731.8906	189115.9	107079.7	228113.6	**173010.2598**	190418.3	190695.7	127516.7	161380.9	**167502.8828**	1.0329	0.8576
P55821	Stmn2	Stathmin-2	304113.5938	238981.6	226514.4	212547.1	**245539.168**	257489	243087.4	241749.4	208595.6	**237730.3555**	1.0328	0.7431
P62754	Rps6	Small ribosomal subunit protein eS6	285625.0938	247440.7	293500.4	278215.2	**276195.3477**	292143.9	279564.1	229369.7	268577	**267413.6758**	1.0328	0.6219
Q65CL1	Ctnna3	Catenin alpha-3	16515.375	13985.93	19218.15	13176.79	**15724.06323**	14866.35	14821.05	16634.25	14581.14	**15225.7002**	1.0327	0.7419
Q8CIE6	Copa	Coatomer subunit alpha	128105.7891	123414.4	126251.5	133069	**127710.1816**	126972.6	124754.6	127660.8	115367.8	**123688.9551**	1.0325	0.2934
Q9D5R2	Wdr20	WD repeat-containing protein 20	27763.55078	22048.34	32134.12	31009.12	**28238.78223**	24054.96	33987.97	32024.33	19333.92	**27350.29443**	1.0325	0.8359
P45377	Akr1b8	Aldose reductase-related protein 2	72327.23438	80411.03	77536.38	81897.47	**78043.0293**	71737.87	70738.43	80351.97	79547.13	**75593.84961**	1.0324	0.4851
Q9JJV2	Pfn2	Profilin-2	771137.375	821009.8	812144	797147.4	**800359.6563**	844680.3	753603.1	747531.1	755157.3	**775242.9375**	1.0324	0.3652
P60469	Ppfia3	Liprin-alpha-3	37560.57813	38296.44	42451.54	44297.83	**40651.59473**	37595.37	44345.52	36956.78	38624.52	**39380.54883**	1.0323	0.6071
Q80UG5	Septin9	Septin-9	134352.875	132045.7	126558	157340.9	**137574.3672**	117530.2	154453.6	136412.1	124708.1	**133275.9941**	1.0323	0.6975
P62843	Rps15	Small ribosomal subunit protein uS19	97171.26563	82528.73	80731.08	67644.59	**82018.91602**	99394.59	89994.07	65504.43	62932.99	**79456.52051**	1.0322	0.8213
Q62188	Dpysl3	Dihydropyrimidinase-related protein 3	947604.875	1015749	922213.5	935870	**955359.2344**	942036.3	970301.9	888136.6	901620.6	**925523.8438**	1.0322	0.3282
Q8BHN3	Ganab	Neutral alpha-glucosidase AB	99083	93660.73	91188.13	93125.19	**94264.25977**	94185.02	97407.46	87920.2	85804.77	**91329.36133**	1.0321	0.3921
P62270	Rps18	Small ribosomal subunit protein uS13	324608.3438	278697.1	327242	320986.7	**312883.5313**	324017	310711.6	287464.8	290432.2	**303156.3984**	1.0321	0.5237
Q64516	Gk	Glycerol kinase	125743.0703	129480.2	135970.8	132798.7	**130998.2109**	118743.1	116790.7	137772.7	134417.1	**126930.9258**	1.0320	0.5082
Q8BU31	Rap2c	Ras-related protein Rap-2c	23611.81836	28506.27	29597.71	28766.78	**27620.64502**	32004.91	27995.32	24776.02	22279.48	**26763.93213**	1.0320	0.7437
Q9ES00	Ube4b	Ubiquitin conjugation factor E4 B	15788.13867	15622.77	16524.85	18463.6	**16599.84058**	16159.03	17488.09	15763.68	14932.84	**16085.90967**	1.0319	0.5638
Q8K3J1	Ndufs8	NADH dehydrogenase [ubiquinone] iron-sulfur protein 8, mitochondrial	61793.92578	61045.42	55638.82	50607.5	**57271.41504**	52380.29	56628.84	48710.89	64289.61	**55502.40723**	1.0319	0.6913
Q5U5Q9	Uimc1	BRCA1-A complex subunit RAP80	80353.47656	71679.03	78557.31	86362.82	**79238.16016**	75171.42	68129.51	85132.53	78742.04	**76793.875**	1.0318	0.6188
P02088	Hbb-b1	Hemoglobin subunit beta-1	6242351.5	5582093	6017134	4903519	**5686274.125**	5862259	5378184	4997551	5807660	**5511413.125**	1.0317	0.6423
Q80U63	Mfn2	Mitofusin-2	44440.13672	42482.52	45300.94	41493.62	**43429.30469**	46276.11	44163.35	38890.71	39045.44	**42093.9043**	1.0317	0.5392
D3YZG8	Mthfd2l	Bifunctional methylenetetrahydrofolate dehydrogenase/cyclohydrolase 2, r	17723.73828	18671.85	17243.47	23163.51	**19200.6416**	20059.26	18803.75	18028.06	17553.51	**18611.14502**	1.0317	0.7004
Q8CGK3	Lonp1	Lon protease homolog, mitochondrial	193670.7188	193892.3	199480.6	207561.5	**198651.2773**	207397	188872.8	195899.9	178061.6	**192557.8398**	1.0316	0.4155
P28665	Mug1	Murinoglobulin-1	8551.135742	7209.54	16910.96	10447.08	**10779.67786**	9058.184	5405.17	10963.25	16370.02	**10449.15674**	1.0316	0.9195
Q9D924	Isca1	Iron-sulfur cluster assembly 1 homolog, mitochondrial	21604.94141	22329.87	28157.52	26857.65	**24737.49707**	21751.31	24139.74	23296.69	26731.24	**23979.74219**	1.0316	0.7086
Q9WTP6	Ak2	Adenylate kinase 2, mitochondrial	69905.44531	64934.28	68009.63	62899.59	**66437.23438**	66449.32	64116.73	62291.31	64760.07	**64404.35742**	1.0316	0.2976
Q9DBF1	Aldh7a1	Alpha-aminoadipic semialdehyde dehydrogenase	240022.5156	230911.2	234460.2	242937.4	**237082.8398**	239219.3	234225.5	214738.2	231162.1	**229836.2969**	1.0315	0.2690
P05213	Tuba1b	Tubulin alpha-1B chain	10416219	10781956	10229684	10728113	**10538993**	10623597	10307465	10053800	9882749	**10216902.75**	1.0315	0.1718
P14148	Rpl7	Large ribosomal subunit protein uL30	254730.2188	263244.4	236988.7	260443.8	**253851.7656**	263570.1	273741	212541.7	234567	**246104.9609**	1.0315	0.6268

Q9WV69	Dmtn	Dematin	106277.0313	89760.79	108107.7	100201.3	**101086.7227**	109954.2	103590	80618.55	97895.97	**98014.6875**	**1.0313**	**0.6977**
Q80XR2	Atp2c1	Calcium-transporting ATPase type 2C member 1	17031.16406	15712.7	14953.92	17555.04	**16313.20654**	16482.25	17568.1	12635.33	16584.69	**15817.59229**	**1.0313**	**0.7035**
Q6TEK5	Vkorc1l1	Vitamin K epoxide reductase complex subunit 1-like protein 1	37298.85547	27213.09	24230.01	34534	**30818.98828**	34190.04	33850.92	27090.7	24426.36	**29889.50488**	**1.0311**	**0.8203**
Q9JKY5	Hip1r	Huntingtin-interacting protein 1-related protein	21581.33789	23299.48	17815.31	23883.07	**21644.79834**	25356.58	20856.13	20242.89	17515.28	**20992.7207**	**1.0311**	**0.7692**
Q61035	Hars1	Histidine--tRNA ligase, cytoplasmic	177172.7969	173710.8	176286.8	168218.7	**173847.2813**	170229.6	170263	160654.8	173315.5	**168615.7383**	**1.0310**	**0.1758**
Q9JKX6	Nudt5	ADP-sugar pyrophosphatase	45404.28906	47855.47	48037.97	42757.06	**46013.69824**	45434.6	39598.08	42689.06	50795.44	**44629.29395**	**1.0310**	**0.6240**
Q8BJW6	Eif2a	Eukaryotic translation initiation factor 2A	21776.38086	23343.11	25555.13	21055.75	**22932.59131**	21573.98	22672.58	23047.19	21685.12	**22244.71777**	**1.0309**	**0.5406**
Q9CQZ6	Ndufb3	NADH dehydrogenase [ubiquinone] 1 beta subcomplex subunit 3	424802.6563	382883.3	442975.6	448199	**424715.1406**	399676.3	415488.3	394516.8	438231.1	**411978.125**	**1.0309**	**0.5006**
Q99104	Myo5a	Unconventional myosin-Va	160218.4063	149510.8	157632.3	161229	**157147.6094**	152065.1	153937	149523.7	154225	**152437.6992**	**1.0309**	**0.1517**
Q9WV27	Atp1a4	Sodium/potassium-transporting ATPase subunit alpha-4	328398.625	146094.8	321398.8	382850.3	**294685.6289**	286400.7	280324.6	304041	272701.4	**285866.9297**	**1.0308**	**0.8705**
P27659	Rpl3	Large ribosomal subunit protein uL3	172122.0156	167431.9	165378.5	171683.5	**169154.0078**	181706.7	176514.3	131284.8	166864.1	**164092.4727**	**1.0308**	**0.6747**
Q6P1I6	Psd2	PH and SEC7 domain-containing protein 2	26007.28125	30502.08	22254.45	27765.78	**26632.39746**	30124.63	29128.38	20190.99	23905.13	**25837.28467**	**1.0308**	**0.7929**
Q922H1	Prmt3	Protein arginine N-methyltransferase 3	9645.204102	10834.23	8156.03	10155.25	**9697.678101**	8672.414	9038.112	9164.922	10759.63	**9408.770752**	**1.0307**	**0.7070**
P61982	Ywhag	14-3-3 protein gamma	775164.8125	643112.6	685648.8	668432.6	**693089.7031**	460152.8	684189.9	768126.1	777486.5	**672488.8438**	**1.0306**	**0.8035**
P23116	Eif3a	Eukaryotic translation initiation factor 3 subunit A	40491.93359	43715.93	44534.66	39860.39	**42150.73047**	42785.93	41254.57	41836.63	37716.42	**40898.39063**	**1.0306**	**0.4643**
Q8BXQ2	Pigt	GPI transamidase component PIG-T	14202.65625	15365.82	16605.6	17606.65	**15945.18188**	14338.62	16711.26	13901.59	16935.3	**15471.69092**	**1.0306**	**0.6764**
P47740	Aldh3a2	Aldehyde dehydrogenase family 3 member A2	75013.33594	82206.7	73333.63	76385.13	**76734.70117**	77230.91	77632.15	76542.55	66422.95	**74457.14063**	**1.0306**	**0.5168**
Q8BL66	Eea1	Early endosome antigen 1	31013.14063	31328.79	33029.53	32717.64	**32022.2749**	31579.47	31600.81	32592.95	28528.2	**31075.35742**	**1.0305**	**0.3861**
Q6PHQ8	Naa35	N-alpha-acetyltransferase 35, NatC auxiliary subunit	8524.619141	6347.755	7268.027	6665.967	**7201.591919**	6943.961	6537.645	6409.071	8064.558	**6988.80896**	**1.0304**	**0.7393**
Q9QYI4	Dnajb12	DnaJ homolog subfamily B member 12	12123.58008	8161.641	7182.19	9818.071	**9321.370483**	8376.896	8606.541	9551.484	9652.479	**9046.850098**	**1.0303**	**0.8159**
Q9DBD5	Pelp1	Proline-, glutamic acid- and leucine-rich protein 1	6918.739746	6109.105	6480.888	13735.69	**8311.105347**	6458.971	7207.349	9767.487	8832.475	**8066.570435**	**1.0303**	**0.9051**
P60122	Ruvbl1	RuvB-like 1	55286.375	55463.11	62659.36	60331.49	**58435.08496**	60274.32	56321.3	54196.3	56090.94	**56720.71484**	**1.0302**	**0.4714**
Q9D154	Serpinb1a	Leukocyte elastase inhibitor A	259324.3281	248903.3	230258	263157.4	**250410.7539**	227159	232392.8	262262.3	250463.3	**243069.3398**	**1.0302**	**0.5278**
P59017	Bcl2l13	Bcl-2-like protein 13	17578.21289	17027.24	14141.42	15544.33	**16072.80029**	18564.91	17444.73	12188.88	14211.92	**15602.60962**	**1.0301**	**0.7861**
Q9Z1J3	Nfs1	Cysteine desulfurase	59255.36719	56732.72	61149.38	60926.81	**59516.06836**	53828.13	56956.96	62094.78	58257.14	**57784.25195**	**1.0300**	**0.4180**
Q8C1B7	Septin11	Septin-11	134784.0313	121838.4	133795.6	127357.6	**129443.8965**	134669.3	131028.5	111161.5	125864.8	**125681.0215**	**1.0299**	**0.5527**
Q8BP67	Rpl24	Large ribosomal subunit protein eL24	216166.9219	220542.8	229087.4	222278.7	**222018.957**	195439.7	225108.5	210570.2	231235	**215588.3398**	**1.0298**	**0.4745**
Q9D4J1	Efhd1	EF-hand domain-containing protein D1	128018.0078	68922.3	153110.5	159282.3	**127333.2656**	151459.3	131198.7	102348.2	109653.8	**123664.9727**	**1.0297**	**0.8806**
Q8R5H1	Usp15	Ubiquitin carboxyl-terminal hydrolase 15	11537.00293	10520.09	9937.556	10415.96	**10602.65259**	11859.97	11291.29	8757.98	9280.416	**10297.41528**	**1.0296**	**0.7245**
Q9Z204	Hnrnpc	Heterogeneous nuclear ribonucleoproteins C1/C2	268438.375	267285.3	262827.1	266475.4	**266256.5469**	281168.6	274349.9	250847.1	228002.9	**258592.1445**	**1.0296**	**0.5514**
Q9R1E6	Enpp2	Ectonucleotide pyrophosphatase/phosphodiesterase family member 2	32085.16797	19140.39	27107.01	20903.08	**24808.91016**	15265.76	28082.02	34517.31	18526.17	**24097.81494**	**1.0295**	**0.8980**
O08529	Capn2	Calpain-2 catalytic subunit	252286.9063	268449.6	299554.3	236508.2	**264199.7227**	266178.3	260684.6	243835.3	255925.9	**256656.0273**	**1.0294**	**0.6164**
Q8BM13	Olfm2	Noelin-2	33919.08594	30009.76	29456.95	38982.18	**33091.99219**	31142.62	31039.38	34035.69	32385.67	**32150.83887**	**1.0293**	**0.6977**
Q9QXJ4	Arl10	ADP-ribosylation factor-like protein 10	14712.16504	13380.39	13905.53	11708.49	**13426.64453**	13255.15	12403.21	13335.11	13186.31	**13044.94751**	**1.0293**	**0.5899**
P46638	Rab11b	Ras-related protein Rab-11B	509497.1563	473851.8	481591.5	498543.3	**490870.9453**	493935.5	503489.2	434077.4	476198.1	**476925.0469**	**1.0292**	**0.4522**
P55264	Adk	Adenosine kinase	130291.9609	138549.5	116266.9	127688.4	**128199.1641**	123302.2	127771.2	125414.1	121779.6	**124566.7871**	**1.0292**	**0.4763**
Q78ZA7	Nap1l4	Nucleosome assembly protein 1-like 4	59213.25781	59250.11	60565.68	66944.33	**61493.34375**	58501.85	57478.64	54673.18	68350.58	**59751.06348**	**1.0292**	**0.6366**
Q8CHT0	Aldh4a1	Delta-1-pyrroline-5-carboxylate dehydrogenase, mitochondrial	86139.9375	84472.59	78575.67	92632.63	**85455.20898**	93088	87980.99	75166.8	75938.63	**83043.60352**	**1.0290**	**0.6655**
Q921M7	Cyrib	CYFIP-related Rac1 interactor B	149667.2656	152078.3	147020	154998.6	**150941.0508**	150175.7	144626.1	135508.3	156435	**146686.2578**	**1.0290**	**0.4052**
Q91V12	Acot7	Cytosolic acyl coenzyme A thioester hydrolase	1651913.625	1797304	1604183	1696161	**1687390.313**	1762748	1644220	1544227	1608290	**1639871.156**	**1.0290**	**0.4701**
Q8K370	Acad10	Acyl-CoA dehydrogenase family member 10	18033.99805	15279.13	18782.43	18493.38	**17647.23315**	18882.17	16833.45	18068.04	14820.2	**17150.96533**	**1.0289**	**0.6924**
Q8BH59	Slc25a12	Electrogenic aspartate/glutamate antiporter SLC25A12, mitochondrial	816085.9375	792171.9	858285.4	891928.4	**839617.9063**	807836.5	825753.4	802231.1	828413.4	**816058.6094**	**1.0289**	**0.3468**
P22682	Cbl	E3 ubiquitin-protein ligase CBL	10936.93848	12584.76	8835.004	8442.313	**10199.75342**	9014.251	9696.806	11718.93	9227.37	**9914.338379**	**1.0288**	**0.8117**
Q80TR1	Adgrl1	Adhesion G protein-coupled receptor L1	28095.65625	30803.98	30118.48	22186.88	**27801.24756**	29010.84	24320.58	27772.83	26989.6	**27023.46338**	**1.0288**	**0.7352**
Q8BHS6	Armcx3	Armadillo repeat-containing X-linked protein 3	8539.363281	6955.386	5303.836	6546.455	**6836.260254**	5722.764	6844.768	6528.375	7485.128	**6645.258911**	**1.0287**	**0.8103**
Q9QXK3	Copg2	Coatomer subunit gamma-2	56205.64063	52462.09	55520.82	59387.34	**55893.9707**	57160.27	60170.06	50344.76	49667.77	**54335.71484**	**1.0287**	**0.6155**
Q61644	Pacsin1	Protein kinase C and casein kinase substrate in neurons protein 1	464245.5	414473.4	431601.4	486603.5	**449230.9531**	429173.4	457609	412916.3	447149.7	**436712.1016**	**1.0287**	**0.5334**
Q9CY27	Tecr	Very-long-chain enoyl-CoA reductase	145669.0781	153549.1	143926.4	154075.8	**149305.1016**	156904.6	149046.1	142564.7	132120.9	**145159.0781**	**1.0286**	**0.5061**
P62869	Elob	Elongin-B	99546.00781	94356.96	91556.21	82511.8	**91992.74414**	91772.5	94449.27	82907.35	88638.53	**89441.91211**	**1.0285**	**0.5786**
Q8K0T0	Rtn1	Reticulon-1	197771.8125	192324.8	179780.1	194318.1	**191048.6914**	195978	203506.2	170999.8	172529.5	**185753.3672**	**1.0285**	**0.5824**
Q60605	Myl6	Myosin light polypeptide 6	223557.1719	201038.5	239228.8	212729.4	**219138.4688**	205265.2	217107.7	220185.3	209740.5	**213074.6836**	**1.0285**	**0.5169**
Q91WQ3	Yars1	Tyrosine--tRNA ligase, cytoplasmic	101196.9141	107442.5	100442.8	102817.1	**102974.8145**	104455	102432.3	97514.64	96108.79	**100127.6758**	**1.0284**	**0.3027**
Q8VD04	Gripap1	GRIP1-associated protein 1	34797.58594	32055.11	34698.13	38179.07	**34932.47559**	35409.79	31004.31	35469.46	33986.17	**33967.43408**	**1.0284**	**0.5762**
Q922B2	Dars1	Aspartate--tRNA ligase, cytoplasmic	186380.0156	204099	185038	185776.5	**190323.375**	190191.3	195562	182630.7	171901.8	**185071.4688**	**1.0284**	**0.4748**
Q8R574	Prpsap2	Phosphoribosyl pyrophosphate synthase-associated protein 2	65643.80469	73040.49	58511.1	77324.55	**68629.9873**	65494.7	67430.52	69873.89	64146.2	**66736.32617**	**1.0284**	**0.6771**
Q8CIH9	Ppat	Amidophosphoribosyltransferase	18655.85742	23352.51	19917.08	19316.32	**20310.44238**	19497.07	14174.5	18865.22	26466.26	**19750.75977**	**1.0283**	**0.8450**
Q811P8	Arhgap32	Rho GTPase-activating protein 32	21816.61914	18428.7	13288.18	18023.75	**17889.31201**	20794.24	20440.54	12146.75	16205.35	**17396.71973**	**1.0283**	**0.8606**
Q9CQA9	Ntpcr	Cancer-related nucleoside-triphosphatase homolog	9268.322266	10774.1	14159.11	10427.01	**11157.1377**	9959.448	12560.74	8164.582	12715.49	**10850.06689**	**1.0283**	**0.8464**
Q9JII6	Akr1a1	Aldo-keto reductase family 1 member A1	381739.9375	378262.7	384288.7	375897.3	**380047.1484**	350207.5	364953	368295.7	394904.5	**369590.1719**	**1.0283**	**0.3128**
Q8K4K6	Pank1	Pantothenate kinase 1	10110.66602	9174.395	9414.434	7943.568	**9160.765503**	8150.587	8370.21	8964.946	10149.54	**8908.82019**	**1.0283**	**0.7058**
Q3TDQ1	Stt3b	Dolichyl-diphosphooligosaccharide--protein glycosyltransferase subunit ST	32096.89844	31938.04	28972.37	29382.57	**30597.46826**	31977.51	29930.45	28292.47	28840.71	**29760.2832**	**1.0281**	**0.4971**
Q9R1Q8	Tagln3	Transgelin-3	476319.625	438413.3	433899.8	452421.9	**450263.6563**	449018.6	405289.2	427436.4	470060.6	**437951.2188**	**1.0281**	**0.4934**
Q60805	Mertk	Tyrosine-protein kinase Mer	8618.894531	7084.003	6792.112	6287.955	**7195.741333**	7508.288	8845.972	6750.029	4892.344	**6999.157959**	**1.0281**	**0.8455**
Q3TZX3	Slc25a33	Solute carrier family 25 member 33	10093.28516	12085.97	12727.27		**11635.50618**	11692.89	12703.05	10597.64	10278.3	**11317.97314**	**1.0281**	**0.7470**
Q9JJZ2	Tuba8	Tubulin alpha-8 chain	46512.125	46425.01	52317.27	56726.12	**50495.13281**	50589.19	44985.73	51847.66	49047.58	**49117.53809**	**1.0280**	**0.6521**

Q9ES28	Arhgef7	Rho guanine nucleotide exchange factor 7	14955.73145	13665.5	13932.88	13798.95	**14088.2666**	16663.99	13704.03	12394.13	12054.9	**13704.26099**	1.0280	0.7365
O89112	Lancl1	Glutathione S-transferase LANCL1	152870.7031	152653.3	129059.4	126524.6	**140276.9961**	142733.5	132772.3	130206.2	140162.1	**136468.5176**	1.0279	0.6432
P98084	Apba2	Amyloid-beta A4 precursor protein-binding family A member 2	13427.21875	15878.42	17743.9	15856.27	**15726.4502**	14449.71	15252.9	17987.59	13510.54	**15300.18408**	1.0279	0.7557
Q9WVT6	Ca14	Carbonic anhydrase 14	140944.1719	136282.9	134579.6	120212.6	**133004.8047**	137430.5	117737.1	129765.2	132669.2	**129400.5137**	1.0279	0.5781
Q9WVL0	Gstz1	Maleylacetoacetate isomerase	99950.22656	102025.9	89414.18	106156.2	**99386.62305**	92179.05	96758.45	100974.3	96873.64	**96696.36914**	1.0278	0.5255
Q60770	Stxbp3	Syntaxin-binding protein 3	54880.70313	59161.32	56990.87	61819.41	**58213.07813**	59568.61	54504.55	55211.56	57264.81	**56637.38184**	1.0278	0.4323
Q64331	Myo6	Unconventional myosin-VI	64926.98047	65341.59	61152.16	58446.56	**62466.82227**	65400.96	59140.01	60151.79	58414.52	**60776.82031**	1.0278	0.4861
Q9JJI8	Rpl38	Large ribosomal subunit protein eL38	63478.57422	60455.59	53138.61	45264.49	**55584.31641**	57033.64	55013.21	51796.79	52488.66	**54083.0752**	1.0278	0.7355
P21460	Cst3	Cystatin-C	37913.58594	37059.16	40382.36	41069.21	**39106.07813**	39806.86	33221.95	38903.55	40273.89	**38051.56348**	1.0277	0.5983
P68404	Prkcb	Protein kinase C beta type	37768.24219	41238.49	38842.78	40445.21	**39573.68066**	39955.55	38459.91	37954.3	37658.57	**38507.08301**	1.0277	0.2966
Q78PY7	Snd1	Staphylococcal nuclease domain-containing protein 1	82413.57813	86369.92	84840.94	84757.84	**84595.57031**	83570.52	89056.75	78876.56	77775.18	**82319.75391**	1.0276	0.4315
O08989	Mras	Ras-related protein M-Ras	49428.35547	49591.06	46692.12	46336.05	**48011.89746**	44070.54	47038.48	46150.68	49635.37	**46723.76855**	1.0276	0.4065
Q9CPV4	Glod4	Glyoxalase domain-containing protein 4	159018.5156	162855.3	156354	154326.2	**158138.5234**	164287.3	159144.3	143615.7	148552.9	**153900.0547**	1.0275	0.4366
P54822	Adsl	Adenylosuccinate lyase	71655.1875	70979.2	65481.32	59137.38	**66813.26953**	66566.05	59976.61	63984.78	69570.57	**65024.50098**	1.0275	0.6322
P62311	Lsm3	U6 snRNA-associated Sm-like protein LSm3	123794.6328	116045.3	98397.82	111101.4	**112334.8027**	112245.1	113743.8	104308.3	107049.3	**109336.6289**	1.0274	0.6218
P62965	Crabp1	Cellular retinoic acid-binding protein 1	14781.43164	14014.66	12623.5	13752.73	**13793.08105**	12367.27	13184.05	13192.61	14957.28	**13425.30298**	1.0274	0.6209
O55022	Pgrmc1	Membrane-associated progesterone receptor component 1	131412.9219	135582.8	135557.1	150850.7	**138350.875**	144404.4	121190.5	134807.6	138294.5	**134674.2344**	1.0273	0.5930
Q9WUT3	Rps6ka2	Ribosomal protein S6 kinase alpha-2	42186.08984	36791.43	35977.89	39923.35	**38719.69238**	38965.24	35625.35	36938.61	39233.95	**37690.78613**	1.0273	0.5609
Q810A7	Ddx42	ATP-dependent RNA helicase DDX42	24479.17578	23724.15	26731.7	26985.64	**25480.16748**	20478.77	26919.1	24123.92	27703.97	**24806.4375**	1.0272	0.7247
C0HKE1;C0HKE2;C0HKI	H2ac4;H2ac6;H2ac7;H2ac8;	Histone H2A type 1-B;Histone H2A type 1-C;Histone H2A type 1-D;Histone	4659522	4484150	5475094	4624771	**4810884.125**	4264486	4313831	5049220	5108414	**4683987.5**	1.0271	0.7058
Q9DBG3	Ap2b1	AP-2 complex subunit beta	350797.4063	343921.9	330424.1	337860.6	**340750.9922**	366256	340827.2	307053.9	312928.8	**331766.4922**	1.0271	0.5537
P17427	Ap2a2	AP-2 complex subunit alpha-2	171715.7188	183918	172910.9	184661	**178301.3984**	179593.4	179703.8	168677.6	166467.8	**173610.6602**	1.0270	0.3789
P63087	Ppp1cc	Serine/threonine-protein phosphatase PP1-gamma catalytic subunit	167613.375	179979.8	154342	156162.1	**164524.3125**	163594.7	171206.5	150615	155420.4	**160209.1289**	1.0269	0.5844
Q61316	Hspa4	Heat shock 70 kDa protein 4	458297.3125	468352.3	426087.3	433728.7	**446616.3984**	456056	442208.7	410499.1	430853.8	**434904.4219**	1.0269	0.4309
P97351	Rps3a	Small ribosomal subunit protein eS1	135915.2813	142081.5	164687.2	152892.2	**148894.043**	150042.3	153757.8	131888.4	144284.4	**144993.2031**	1.0269	0.6403
Q02248	Ctnnb1	Catenin beta-1	97532.8125	94637.95	96149.82	91895.93	**95054.12891**	99177.19	100371.8	82826.21	87883.48	**92564.66406**	1.0269	0.5971
Q8BHW2	Oscp1	Protein OSCP1	7542.943848	10203.2	8834.323	10889.34	**9367.451782**	10300.89	7306.33	10717.05	8164.818	**9122.272827**	1.0269	0.8324
Q64511	Top2b	DNA topoisomerase 2-beta	16994.76367	13619.08	16997.42	14512.47	**15530.93384**	15586.42	15816.24	16072	13027.41	**15125.51758**	1.0268	0.7291
Q8CAY6	Acat2	Acetyl-CoA acetyltransferase, cytosolic	117682.0547	126848.5	112644.9	115570.8	**118186.5723**	114804.9	117899	101856	125861.2	**115105.2754**	1.0268	0.6178
Q80UK8	Ints2	Integrator complex subunit 2		18306.42			**18306.42383**	17830				**17829.99805**	1.0267	
P59999	Arpc4	Actin-related protein 2/3 complex subunit 4	451760.9063	507294.1	525335.6	505853.5	**497561.0313**	504295.1	480368.8	488488.5	465479.3	**484657.9219**	1.0266	0.4967
Q9Z1R2	Bag6	Large proline-rich protein BAG6	42543.10156	41251.48	39605.26	45674.83	**42268.66699**	42266.34	42510.24	39571.87	40342.01	**41172.61523**	1.0266	0.4850
Q9QXL2	Kif21a	Kinesin-like protein KIF21A	125346.1641	127413.6	122806.2	124165	**124932.7402**	122636.5	124037.7	117282.9	122819	**121694.0625**	1.0266	0.1208
O35316	Slc6a6	Sodium- and chloride-dependent taurine transporter	1332935.375	1429210	1240958	1363576	**1341669.969**	1375812	1356856	1272694	1222330	**1306922.938**	1.0266	0.5377
Q9D0K2	Oxct1	Succinyl-CoA:3-ketoacid coenzyme A transferase 1, mitochondrial	215488.9375	245582.9	219091.7	228684	**227211.8984**	242443.4	239996.3	207678.2	195306.8	**221356.1992**	1.0265	0.6806
Q9QXB9	Drg2	Developmentally-regulated GTP-binding protein 2	67650.02344	71892.2	66475.26	69180.01	**68799.37305**	65974.1	65414.46	68716.88	68024.69	**67032.53223**	1.0264	0.2580
Q9R1P1	Psmb3	Proteasome subunit beta type-3	98819.21094	98772.13	91650.84	102736.6	**97994.6875**	96654.17	96303.33	95743.45	93227.68	**95482.1582**	1.0263	0.3421
P50429	Arsb	Arylsulfatase B	24308.00977	22861.49	20028.39	21255.32	**22113.30127**	28379.65	23357.54	12763.7	21687.32	**21547.052**	1.0263	0.8727
Q9Z1G3	Atp6v1c1	V-type proton ATPase subunit C 1	236306.0469	227733.3	227891.6	238548.3	**232619.8008**	236121.4	231813.8	207962.4	230827.8	**226681.3516**	1.0262	0.4250
Q8BIF2	Rbfox3	RNA binding protein fox-1 homolog 3	9805.099609	9870.171	7846.922	8999.046	**9130.30957**	7431.422	9645.99	9943.666	8570.894	**8897.993042**	1.0261	0.7643
P56501	Ucp3	Putative mitochondrial transporter UCP3	16123.63281	15718.47	13335.34	14978.52	**15038.99072**	16639.41	23829.35	9962.885	8194.509	**14656.53735**	1.0261	0.9191
P97825	Jpt1	Jupiter microtubule associated homolog 1	33519.84766	29803.79	23106.98	26097.49	**28132.02881**	24371.88	23481.17	29235.49	32578.39	**27416.73291**	1.0261	0.8256
Q68FH0	Pkp4	Plakophilin-4	40776.47656	41674.73	37037.6	35387.71	**38719.13086**	42454.1	39975.66	32170.1	36350.52	**37737.59473**	1.0260	0.7281
P35700	Prdx1	Peroxiredoxin-1	1316669.375	1340796	1221741	1223279	**1275621.406**	1282369	1277891	1202163	1210771	**1243298.375**	1.0260	0.4241
Q80U49	Cep170b	Centrosomal protein of 170 kDa protein B	37198.61328	27168.05	32080.46	30951.39	**31849.62988**	29178.51	33148.4	30567.76	31282.12	**31044.19678**	1.0259	0.7301
Q9CZP5	Bcs1l	Mitochondrial chaperone BCS1	30332.86133	29360.35	33420.52	33920.31	**31758.51123**	31467.95	32434.78	29387.04	30532.81	**30955.64404**	1.0259	0.5598
Q8R0H9	Gga1	ADP-ribosylation factor-binding protein GGA1	15597.48145	15662.9	16245.84	19003.44	**16627.41479**	14645.83	14579.37	17678.4	17928.97	**16208.1438**	1.0259	0.7438
P52196	Tst	Thiosulfate sulfurtransferase	301995.5625	309474.9	318940.3	323659.8	**313517.6172**	302905.2	320943.6	309223.8	289382.6	**305613.8047**	1.0259	0.3705
Q99P72	Rtn4	Reticulon-4	654050.3125	615909.1	637579.7	588760.6	**624074.9219**	619244.4	577932.6	604329.1	632011.6	**608379.3906**	1.0258	0.4238
Q8BGB7	Enoph1	Enolase-phosphatase E1	61179.55469	60033.67	55553.41	49043.08	**56452.42773**	53905.67	58966.27	51491.19	55786.25	**55037.34473**	1.0257	0.6712
Q3U1J4	Ddb1	DNA damage-binding protein 1	81607.48438	81367.08	80397.78	87335.85	**82677.04883**	82416.41	82717.94	80225.42	77064.45	**80606.05469**	1.0257	0.3503
O55131	Septin7	Septin-7	220353.3125	219755.3	229350.4	224271.5	**223432.6172**	219536.5	226637.4	202946	222252.9	**217843.1875**	1.0257	0.3591
P63141	Kcna2	Potassium voltage-gated channel subfamily A member 2	137782.8594	140740.4	157062.8	159076.3	**148665.5938**	135446.1	136901.7	155793.3	151666.7	**144951.9453**	1.0256	0.6388
Q8VDU5	Snrk	SNF-related serine/threonine-protein kinase	13263.37207	15910.14	16984.29	12944.27	**14775.51855**	12040.59	15893.91		15285.9	**14406.80046**	1.0256	0.8205
Q99M08		Uncharacterized protein C4orf3 homolog	32420.9043	31769.66	28025.77	28987.85	**30301.04736**	30444.46	30619.77	28091.01	29031.49	**29546.68262**	1.0255	0.5594
Q14BB9	Map6d1	MAP6 domain-containing protein 1	92381.03906	89426.45	75131.8	87053.6	**85998.2207**	86975.86	83856.5	86927.91	77694.39	**83863.66602**	1.0255	0.6423
Q00PI9	Hnrnpul2	Heterogeneous nuclear ribonucleoprotein U-like protein 2	42747.88281	42504.4	34546.74	44566.46	**41091.37109**	40948.95	45789.85	37220.09	36349.16	**40077.01367**	1.0253	0.7544
Q8R151	Znfx1	NFX1-type zinc finger-containing protein 1	5586.949219	7746.229	21715.44	24726.66	**14943.81799**	3394.973	7357.274		32974.63	**14575.62427**	1.0253	0.9710
Q5H8C4	Vps13a	Intermembrane lipid transfer protein VPS13A	15911.24805	15069.07	15837.87	15240.33	**15514.62939**	15264.85	15939.36	14768.97	14563.45	**15134.15698**	1.0251	0.3458
E9PUL5	Prrt2	Proline-rich transmembrane protein 2	33223.28516	40014.36	51142.88	45113.93	**42373.61133**	52564.74	31861.92	31316.36	49605.23	**41337.06201**	1.0251	0.8842
Q9WTP7	Ak3	GTP:AMP phosphotransferase AK3, mitochondrial	194085.3125	191201.3	211113.5	175968.1	**193092.0508**	203086	194119.6	169451.6	186859.8	**188379.2383**	1.0250	0.6583
Q8BLE7	Slc17a6	Vesicular glutamate transporter 2	140727.7656	146487.7	122125.5	147454.8	**139198.9375**	149175.4	157704.8	113148.8	123236.2	**135816.3223**	1.0249	0.7885
P49769	Psen1	Presenilin-1	7456.483887	4816.346	4503.676		**5592.168457**		7355.671		3557.008	**5456.339355**	1.0249	0.9464
G5E8K5	Ank3	Ankyrin-3	189438.0938	179188.7	197939.4	198270.4	**191209.1289**	191080	190540.7	187236	177434.1	**186572.6836**	1.0249	0.4314
Q8BGY2	Eif5a2	Eukaryotic translation initiation factor 5A-2	11678.47363	19390.99	23826.85	13018.33	**16978.65845**	13701.64	15022.61	19780.66	17764.28	**16567.29639**	1.0248	0.9003

Q5SVR0	Tbc1d9b	TBC1 domain family member 9B	16665.69922	15948.9	20633.91	18025.2	**17818.42993**	19096.55	17181.51	15824.72	17446.17	**17387.23999**	**1.0248**	**0.7382**
P27612	Plaa	Phospholipase A-2-activating protein	61651.31641	59706.69	53331.59	59158.61	**58462.0498**	57728.07	57427.61	54586.64	58454.89	**57049.30371**	**1.0248**	**0.5029**
Q9CQ62	Decr1	2,4-dienoyl-CoA reductase [(3E)-enoyl-CoA-producing], mitochondrial	182185.1406	169954	202056.8	184202.2	**184599.5391**	178735.2	183968.5	179584.7	178299.7	**180147.0391**	**1.0247**	**0.5335**
P85094	Isoc2a	Isochorismatase domain-containing protein 2A	143212.6875	150131.5	166403.1	159857.2	**154901.1211**	145965.3	146977.5	145901.5	165834.8	**151169.7461**	**1.0247**	**0.6177**
O88342	Wdr1	WD repeat-containing protein 1	329217.875	347393.9	315300.2	318619.1	**327632.7734**	334535.8	325557.3	293015	326085.2	**319798.3359**	**1.0245**	**0.5269**
Q8BGS7	Cept1	Choline/ethanolaminephosphotransferase 1	74070.30469	80189.16	72072.34	62438.54	**72192.58301**	70622.59	73702.24	74044.5	63496.1	**70466.35742**	**1.0245**	**0.7097**
Q6A0A9	FAM120A	Constitutive coactivator of PPAR-gamma-like protein 1	18079.33203	17468.04	16261.97	15066.89	**16719.05859**	17133.79	17446.04	15228.14	15472.57	**16320.1355**	**1.0244**	**0.6645**
Q8BTY8	Scfd2	Sec1 family domain-containing protein 2	21789.52734	20808.17	21541.58	21019.14	**21289.60352**	20463.86	22064.01	20478.1	20120.95	**20781.72852**	**1.0244**	**0.3408**
P47738	Aldh2	Aldehyde dehydrogenase, mitochondrial	439025.2188	402346.6	498421.7	495808.4	**458900.4531**	431503.5	435501.6	475720.7	449297.1	**448005.7109**	**1.0243**	**0.6825**
P32037	Slc2a3	Solute carrier family 2, facilitated glucose transporter member 3	66936.91406	63687.14	66836.04	73157.34	**67654.35645**	61261.15	67861.27	66938.61	68150.05	**66052.76953**	**1.0242**	**0.5545**
Q8CCS6	Pabpn1	Polyadenylate-binding protein 2	39934.49609	36837.5	39319.92	22803.32	**34723.80859**	40184.77	34739.49	31180.5	29505.57	**33902.58252**	**1.0242**	**0.8662**
Q99KP6	Prpf19	Pre-mRNA-processing factor 19	68233.5	64966.46	70555.84	72581.05	**69084.21289**	69885.45	67506.83	66913.24	65500.82	**67451.58496**	**1.0242**	**0.4169**
O70194	Eif3d	Eukaryotic translation initiation factor 3 subunit D	46063.86719	45006.31	40375.27	45232.88	**44169.58301**	45181.66	47044.52	36070.39	44212.4	**43127.24219**	**1.0242**	**0.7171**
Q64191	Aga	N(4)-(beta-N-acetylglucosaminyl)-L-asparaginase	9047.162109	9150.273	9990.755	8640.31	**9207.125**	8402.379	7531.353	10723.34	9302.738	**8989.953003**	**1.0242**	**0.7785**
Q8BQU6	Gjc2	Gap junction gamma-2 protein	61887.03906	54104.88	49805.95	54333.57	**55032.8584**	65139.47	50444.82	50982.3	48372.75	**53734.83496**	**1.0242**	**0.7869**
Q9EQF6	Dpysl5	Dihydropyrimidinase-related protein 5	238732.5469	233035.1	217133.8	235443.8	**231086.3281**	242163.9	231834.4	204636.3	223933.5	**225642.0352**	**1.0241**	**0.5785**
Q8BZA9	Tigar	Fructose-2,6-bisphosphatase TIGAR	49838.70703	48351.44	47954.76	47280.27	**48356.29492**	43978.29	43942.64	45936.57	55018.4	**47218.97754**	**1.0241**	**0.6878**
Q9D1R9	Rpl34	Large ribosomal subunit protein eL34	181422.2813	169575.6	173929.1	198808.3	**180933.8125**	169353.4	201030.3	179345.3	157024.5	**176688.3594**	**1.0240**	**0.7205**
Q7TNE3	Spag7	Sperm-associated antigen 7	15129.17285	13532.06	17294.6	20289.2	**16561.25806**	16228.22	14775.09	16376.24	17318.45	**16174.5022**	**1.0239**	**0.8117**
P83741	Wnk1	Serine/threonine-protein kinase WNK1	40614.94531	41036.02	38445.74	38269.62	**39591.58008**	37374.97	39370	40871.93	37055.63	**38668.13379**	**1.0239**	**0.4519**
O35887	Calu	Calumenin	44812.47266	45310.73	53655.46	35262.5	**44760.29004**	36766.81	44006.21	43169.51	50933.17	**43718.92578**	**1.0238**	**0.8336**
O08997	Atox1	Copper transport protein ATOX1	95434.89063	93737.84	85810.17	95027.08	**92502.49609**	86768.3	95357.37	81878.52	97401.16	**90351.33984**	**1.0238**	**0.6338**
Q8K245	Uvrag	UV radiation resistance-associated protein	6845.24707	6350.964	8644.343	8315.313	**7538.966675**	6915.072	6188.516	8810.18	7545.328	**7364.774048**	**1.0237**	**0.8321**
Q9CZG9	Pdzd11	PDZ domain-containing protein 11	12798.76074	12705.58			**12752.17285**	10364.25	15368.24	10708.83	13394.09	**12458.84985**	**1.0235**	**0.8767**
Q7TQD2	Tppp	Tubulin polymerization-promoting protein	292578.8125	247534	251896	277412.7	**267355.3828**	253731.3	252894.9	270748.7	267460.5	**261208.8398**	**1.0235**	**0.6163**
Q922D8	Mthfd1	C-1-tetrahydrofolate synthase, cytoplasmic	142453.8438	138726	135274.1	130916.7	**136842.6367**	137924.6	136697.8	128608.8	131557.1	**133697.0625**	**1.0235**	**0.3761**
Q6PD26	Pigs	GPI transamidase component PIG-S	27200.63867	27645.08	21818.85	23850.32	**25128.72314**	25645.68	25391.45	25897.35	21269.91	**24551.09424**	**1.0235**	**0.7556**
Q8K2C9	Hacd3	Very-long-chain (3R)-3-hydroxyacyl-CoA dehydratase 3	164370.375	151170	155690.3	165771.5	**159250.5586**	169759.3	170673.7	136652.4	145324.4	**155602.4453**	**1.0234**	**0.7086**
Q8CHP8	Pgp	Glycerol-3-phosphate phosphatase	219565.9531	266476.6	245003	262512.9	**248389.6172**	249957.4	252582	214534.8	253738.3	**242703.1406**	**1.0234**	**0.7035**
Q9WV85	Nme3	Nucleoside diphosphate kinase 3	32709.94141	36462.84	32484.2	38422.09	**35019.76709**	36513.37	33677.84	31057.88	35633.29	**34220.59375**	**1.0234**	**0.6875**
Q03059	Chat	Choline O-acetyltransferase	11107.64063	11594.28	23493.22	7857.402	**13513.13464**	14486.52	13930.3	12121.12	12281.88	**13204.95288**	**1.0233**	**0.9323**
Q91W89	Man2c1	Alpha-mannosidase 2C1	43205.82422	46017.59	44226.51	42336.55	**43946.61914**	46459.37	41528.64	40515.43	43278.3	**42945.43652**	**1.0233**	**0.5356**
P97384	Anxa11	Annexin A11	18251.41992	18346.81	26135.24	22735.49	**21367.24121**	20259.66	20297.97	20075.13	22930.71	**20890.86719**	**1.0228**	**0.8215**
Q9Z0L0	Tpbg	Trophoblast glycoprotein	29229.85938	27167.58	23675.42	32579.48	**28163.08398**	30440.12	32476.09	24166.08	23059.88	**27535.54248**	**1.0228**	**0.8398**
Q9D8B3	Chmp4b	Charged multivesicular body protein 4b	59709.67969	58438.68	50220.13	47905.08	**54068.39258**	45915.79	47989.61	50562.49	66990.22	**52864.52539**	**1.0228**	**0.8378**
O88712	Ctbp1	C-terminal-binding protein 1	134447.875	133271.2	129362.7	134623.6	**132926.3535**	132170.4	136620.6	128210.7	122865.8	**129966.8613**	**1.0228**	**0.3867**
Q64669	Nqo1	NAD(P)H dehydrogenase [quinone] 1	29876.36719	33187.41	30419.95	31424.71	**31227.10645**	30243.49	27359.58	32368.54	32159.4	**30532.75293**	**1.0227**	**0.6304**
Q80W04	Tmcc2	Transmembrane and coiled-coil domains protein 2	12695.51758	11993.66	9163.139	10018.92	**10967.80884**	13206.8	10200.64	9465.146	10025.28	**10724.46802**	**1.0227**	**0.8434**
Q07417	Acads	Short-chain specific acyl-CoA dehydrogenase, mitochondrial	47836.87891	50936.96	54111.21	51551.91	**51109.24121**	53454.93	49829.39	48088.12	48532.71	**49976.28516**	**1.0227**	**0.5464**
Q99LG4	Ttc5	Tetratricopeptide repeat protein 5	7832.38623	7986.941	6900.818	6923.528	**7410.918579**	8836.247	8095.227	3739.597	8315.583	**7246.663513**	**1.0227**	**0.8968**
Q8BFZ3	Actbl2	Beta-actin-like protein 2	87511.96094	99305.55	71260.12	68548.49	**81656.5293**	97654.74	96633.8	63454.91	61652.88	**79849.08203**	**1.0226**	**0.8883**
Q9CR41	Hypk	Huntingtin-interacting protein K	5317.00293	6472.399	7966.051	4231.124	**5996.644165**	6237.778	3776.677	7420.123	6021.849	**5864.10675**	**1.0226**	**0.9084**
Q8R146	Apeh	Acylamino-acid-releasing enzyme	67069.14063	64169.45	62755.93	63292.3	**64321.70508**	63409.73	63686.23	56305.09	68202.27	**62900.82813**	**1.0226**	**0.6097**
P22892	Ap1g1	AP-1 complex subunit gamma-1	110349.5469	105459.1	99730.13	104659.6	**105049.6094**	107550.3	105140.3	97153.59	101072.5	**102729.1602**	**1.0226**	**0.4900**
Q9CZD3	Gars1	Glycine--tRNA ligase	255601.5469	245009.8	231504.6	233643.2	**241439.7734**	242151.6	244083.8	215989.2	242323.4	**236136.9883**	**1.0225**	**0.5662**
Q9WTN0	Ggps1	Geranylgeranyl pyrophosphate synthase	39070.07031	41753.49	45399.27	43488.49	**42427.83105**	44900	36038.38	41255.79	43797.36	**41497.88086**	**1.0224**	**0.7104**
Q3TY60	Fam131b	Protein FAM131B	20627.47656	17827.82	14381.39	16130.8	**17241.87231**	17096.72	20354.4	13769.22	16236.74	**16864.27002**	**1.0224**	**0.8492**
Q9Z0S6	Cldn10	Claudin-10	25685.61914	19372.3	21734.59	18649.17	**21360.42139**	21613.26	21516.34	20053.5	20396.91	**20895.00195**	**1.0223**	**0.7852**
P28740	Kif2a	Kinesin-like protein KIF2A	227963.9688	215671.9	224182.6	234506.5	**225581.2461**	228525	228037.6	211182.9	214930.4	**220668.9766**	**1.0223**	**0.4404**
Q9CYN2	Spcs2	Signal peptidase complex subunit 2	35507.49219	29349.16	33510.87	34153.96	**33130.37061**	36095.59	31713.14	28389.81	33440.34	**32409.72168**	**1.0222**	**0.7420**
Q6P1F6	Ppp2r2a	Serine/threonine-protein phosphatase 2A 55 kDa regulatory subunit B alpha	95078.04688	95681.94	87996.65	94587.78	**93336.10352**	98286.13	91914.45	89457.64	85570.51	**91307.17969**	**1.0222**	**0.5512**
Q8BJH1	Zc2hc1a	Zinc finger C2HC domain-containing protein 1A	32758.52734	35564.63	33861.16	37486.81	**34917.78223**	31039.52	34507.77	36737.74	34365.61	**34162.66064**	**1.0221**	**0.6463**
Q61598	Gdi2	Rab GDP dissociation inhibitor beta	693733.4375	706162.8	687206.8	650370.6	**684368.375**	692254.8	657877.5	656438	671742.6	**669578.2344**	**1.0221**	**0.3499**
P29533	Vcam1	Vascular cell adhesion protein 1	12149.01953	8840.485	11163.92	11172.78	**10831.552**	8980.733	10613.3	11504.94	11295.84	**10598.70166**	**1.0220**	**0.8058**
P56959	Fus	RNA-binding protein FUS	108739.9609	102574.1	103529.5	106356.9	**105300.0977**	114454.2	106526.3	100965	90237.23	**103045.6895**	**1.0219**	**0.6841**
O08788	Dctn1	Dynactin subunit 1	185942.6875	184158.6	186413.3	194116.3	**187657.707**	188478	182760.7	187299.9	176032.8	**183642.8672**	**1.0219**	**0.3051**
Q6ZQ58	Larp1	La-related protein 1	66060.8125	64288.8	63824.59	64902.72	**64769.23242**	62923.57	65594.59	66186.27	58838.25	**63385.66602**	**1.0218**	**0.4574**
P17156	Hspa2	Heat shock-related 70 kDa protein 2	2114081.25	2146295	2231329	2201621	**2173331.563**	2139121	2188645	2027343	2152670	**2126944.75**	**1.0218**	**0.3296**
B9EJ80	Pdzd8	PDZ domain-containing protein 8	25297.48438	24352.79	24371.59	22434.33	**24114.04688**	23446.95	25592.74	22977.64	22381.5	**23599.70703**	**1.0218**	**0.5973**
Q8BSZ2	Ap3s2	AP-3 complex subunit sigma-2	30856.9082	25463.56	25164.33	28986.25	**27617.7627**	28138.56	25130.5	27584.99	27271.58	**27031.40771**	**1.0217**	**0.7154**
Q3UU96	Cdc42bpa	Serine/threonine-protein kinase MRCK alpha	28405.61133	24094.89	26020.41	25280.97	**25950.47168**	25430.5	27799.51	23083.01	25285.88	**25399.7251**	**1.0217**	**0.6921**
Q9EQP6	Arr3	Arrestin-C	7581.017578	7803.046	11715.65	14913.5	**10503.30396**	7681.219	7319.712		15841.69	**10280.87354**	**1.0216**	**0.9459**
Q3UMF0	Cobll1	Cordon-bleu protein-like 1	7317.833008	7965.63	8982.162	3477.896	**6935.88031**	6893.247	6145.193	8664.789	5452.962	**6789.047852**	**1.0216**	**0.9191**
Q9D7X8	Ggct	Gamma-glutamylcyclotransferase	50059.15625	60247.46	51552.12	46675.51	**52133.55957**	54467.74	54726.98	45879.97	49064.99	**51034.9209**	**1.0215**	**0.7710**
P59481	Lman2l	VIP36-like protein	27617.97266	27144.28	20363.26	28605.93	**25932.85986**	9570.611	36366.54	32961.05	22650.46	**25387.16357**	**1.0215**	**0.9339**

P57776	Eef1d	Elongation factor 1-delta	300854.875	305575.2	284378.2	279834.6	292660.7266	286860.8	293257.7	291656.1	274322.2	286524.1797	1.0214	0.4485
Q8CHH9	Septin8	Septin-8	271654.0938	255067.3	233179.2	238569.2	249617.4375	259077.5	247122.8	225775.7	245578.8	244388.7031	1.0214	0.6543
P60670	Nploc4	Nuclear protein localization protein 4 homolog	18160.66602	19178.12	22805.46	16980.23	19281.11865	21560.16	21556.26	16648.96	15743.77	18877.28711	1.0214	0.8469
P51880	Fabp7	Fatty acid-binding protein, brain	212977.9375	228369.5	153436.7	224340.8	204781.2578	202787.8	209332.8	195507.5	194366.4	200498.5977	1.0214	0.8176
Q9CZM2	Rpl15	Large ribosomal subunit protein eL15	104984.0234	110417.6	94393.32	105356.9	103787.9629	111388.6	102015.1	94511.79	98575.84	101622.8262	1.0213	0.6758
P53798	Fdft1	Squalene synthase	18510.81836	14498.59	8516.888	15922.79	14362.26929	18284.52	18488.96	5613.784	13868.41	14063.91675	1.0212	0.9381
P32067	Ssb	Lupus La protein homolog	77369.08594	84629.65	74772.33	88067.91	81209.74219	79712.95	84670.54	71478.52	82246.57	79527.14453	1.0212	0.7038
P22723	Gabrg2	Gamma-aminobutyric acid receptor subunit gamma-2	16874.42188	16344.88	17656.78	22247.67	18280.93506	18124.84	17216.94	17298.97	18967.96	17902.18018	1.0212	0.7973
Q99L43	Cds2	Phosphatidate cytidylyltransferase 2	88971.53906	62855.64	80641.96	75798.89	77067.00879	85741.27	64535.65	69784.56	81827.95	75472.3584	1.0211	0.8364
Q7TMS5	Abcg2	Broad substrate specificity ATP-binding cassette transporter ABCG2	27145.9082	26699.85	40093.25	37386.57	32831.39697	35106.25	34179.36	30037.3	29286.06	32152.2417	1.0211	0.8623
Q9CZR3	Tomm40l	Mitochondrial import receptor subunit TOM40B	27161.13281	27650.72	22237.15	27600.67	26162.42041	26974.79	27642.94	23859.14	24011.61	25622.12109	1.0211	0.7531
A2A8L5	Ptprf	Receptor-type tyrosine-protein phosphatase F	26661.92383	26458.81	24985.74	21346.21	24863.17041	26493.16	23108.38	22587.56	25211.28	24350.09473	1.0211	0.7490
Q9D394	Rufy3	Protein RUFY3	455969.7813	476714.4	460544.8	437114.1	457585.7813	467060.6	459276.3	407461.9	458840.7	448159.8672	1.0210	0.5758
Q5SWU9	Acaca	Acetyl-CoA carboxylase 1	80054.78125	82955.09	74913.63	71304.63	77307.03125	79868.35	78700.57	71164.16	73138.45	75717.88281	1.0210	0.6521
Q9D8E6	Rpl4	Large ribosomal subunit protein uL4	264564.0938	258234.3	247180.6	261312	257822.7617	263187.5	267723.5	235343.8	243885.7	252535.1211	1.0209	0.5608
O55106	Strn	Striatin	51132.92578	46710.53	44555.54	44970.05	46842.26074	46679.33	47071.82	45349.23	44435.06	45883.85938	1.0209	0.5763
Q6GQT9	Nomo1	BOS complex subunit NOMO1	47961.39063	50107.31	42108.45	51383.43	47890.14355	48747.61	51885.61	45589.34	41419.8	46910.5918	1.0209	0.7579
Q6PF93	Pik3c3	Phosphatidylinositol 3-kinase catalytic subunit type 3	25089.74023	21753.95	21745.82	23543.27	23033.19629	21574.16	22464.01	24352.72	21866.89	22564.44482	1.0208	0.6617
Q8JZK9	Hmgcs1	Hydroxymethylglutaryl-CoA synthase, cytoplasmic	48818.17969	59613.77	44164.82	35241.37	46959.53418	50656.04	50559.49	41366.55	41433.54	46003.90625	1.0208	0.8729
Q9CXS4	Cenpv	Centromere protein V	43022.75781	48508.7	45160.45	53644.43	47584.08301	46825	45513.73	48516.31	45608.52	46615.8877	1.0208	0.7027
Q80TH2	Erbin	Erbin	15932.53027	17648.74	14667.04	15256.49	15876.19751	17916.11	14815.48	12914.32	16571.6	15554.37769	1.0207	0.8073
Q3USB7	Plcl1	Inactive phospholipase C-like protein 1	48122.80078	47084.95	42909.48	42701.17	45204.59961	46418.56	46839.95	39985.14	43910.39	44288.51074	1.0207	0.6790
P53612	Rabggtb	Geranylgeranyl transferase type-2 subunit beta	15893.71875	10944.37	15547.65	14953.54	14334.81836	14743.6	16822.98	11878.46	12734.2	14044.80835	1.0206	0.8614
Q9CPT3	p	N-acylneuraminate-9-phosphatase	11293.45508	12092.36	4470.283	6455.271	8577.841919	8047.497	8074.32	8903.724	8591.926	8404.366577	1.0206	0.9288
Q3UHX2	Pdap1	28 kDa heat- and acid-stable phosphoprotein	41408.46875	40395.96	42636.02	37567.91	40502.0918	37316.11	40339.14	37310.43	43767.55	39683.31055	1.0206	0.6782
Q9CQZ5	Ndufa6	NADH dehydrogenase [ubiquinone] 1 alpha subcomplex subunit 6	330473.4063	322448.3	344943.6	381770.1	344908.8594	347684.7	378250	280906.8	344983.1	337956.125	1.0206	0.7845
O88343	Slc4a4	Electrogenic sodium bicarbonate cotransporter 1	379819.0938	360949	339328	351531.9	357907.0078	354823.7	380586.4	326705.4	340702.5	350704.5	1.0205	0.6329
Q9CZN7	Shmt2	Serine hydroxymethyltransferase, mitochondrial	43698.76563	42533.8	48219.35	48401.29	45713.30176	43008.53	44419.16	51909.53	39842.96	44795.04492	1.0205	0.7680
P16388	Kcna1	Potassium voltage-gated channel subfamily A member 1	124770.6172	126600.1	155778.4	141048.3	137049.375	117394.9	126295.5	148213.7	145318.2	134305.582	1.0204	0.8003
Q6URW6	Myh14	Myosin-14	48948.07813	44420.2	57446.75	44251.49	48766.62891	51687.57	45996.54	46319.41	47157.76	47790.31934	1.0204	0.7813
Q6PDI5	Ecpas	Proteasome adapter and scaffold protein ECM29	35826.375	37552.13	34844.96	33054.64	35319.52734	34047.35	35131.45	35094.41	34178.13	34612.83398	1.0204	0.4994
Q9D8Z6	Atg101	Autophagy-related protein 101	17429.76953	13780.74	9728.521	8984.077	12480.77588	13034.96	12022.45	10624.19	13247.5	12232.27563	1.0203	0.9073
Q9D9V3	Echdc1	Ethylmalonyl-CoA decarboxylase	112871.6172	112673.4	100049.2	99585.21	106294.8672	107533.7	108405.2	100712.5	100064.4	104178.9512	1.0203	0.6432
Q5DQR4	Stxbp5l	Syntaxin-binding protein 5-like	32465.67188	31750.05	30835.85	30551.04	31400.65381	32896.11	33126.72	28823.61	28260.69	30776.78369	1.0203	0.6644
P03995	Gfap	Glial fibrillary acidic protein	5288227	5162707	3784556	3877611	4528274.938	4907640	4351652	4280745	4214840	4438718.875	1.0202	0.8433
P62071	Rras2	Ras-related protein R-Ras2	18624.62305	16423.3	12225.72	11404.12	14669.44287	19099.14	18872.4	8350.006	11195.81	14379.34033	1.0202	0.9311
O88545	Cops6	COP9 signalosome complex subunit 6	91192.40625	90063.38	83828.26	81578.48	86665.62891	81357.78	87444.63	85913.36	85095.47	84952.81055	1.0202	0.5460
Q8BMP6	Acbd3	Golgi resident protein GCP60	18441.61328	19066.67	16147.5	14630.17	17071.48828	17514.76	20184.47	15408.77	13829.06	16734.26831	1.0202	0.8508
P63321	Rala	Ras-related protein Ral-A	262968.4688	257628.2	276702.9	265264.8	265641.0781	261463.4	261969.7	250089.5	268056.8	260394.8594	1.0201	0.3766
Q6PHU5	Sort1	Sortilin	40516.83594	39867.6	33929.65	34488.82	37200.72656	35220.77	36362.71	39394.46	34887.52	36466.3623	1.0201	0.7282
Q7TQ95	Lnpk	Endoplasmic reticulum junction formation protein lunapark	63271.78125	53500.88	56397.8	61687.98	58714.61133	56044.84	56971.81	61494.75	55720.19	57557.89746	1.0201	0.6767
Q9DAR7	Dcps	m7GpppX diphosphatase	57407.02344	70155.52	63100.69	45204.83	58967.01758	65814.13	52295.3	59341.42	53803.25	57813.52637	1.0200	0.8563
Q9Z1Q9	Vars1	Valine--tRNA ligase	56651.15625	58818.07	58320.32	57648.15	57859.42578	58494.93	59195.73	52803.71	56426.61	56730.24805	1.0199	0.4827
P84096	Rhog	Rho-related GTP-binding protein RhoG	314276.0938	338598.4	331562.9	320022.2	326114.8906	322031.7	310449.1	315927.7	330683.8	319773.0781	1.0198	0.4001
P62908	Rps3	Small ribosomal subunit protein uS3	516664.3438	545297.9	515733.4	499235.7	519232.8203	531987.8	556273.1	452048.2	496257.5	509141.6484	1.0198	0.6960
Q9QY76	Vapb	Vesicle-associated membrane protein-associated protein B	287720.5	296357.8	289147.3	325924.9	299787.6094	304332.9	284803.1	278697.4	308118.3	293987.9453	1.0197	0.6311
Q11136	Pepd	Xaa-Pro dipeptidase	101084.6563	95667.77	91161.8	100828.9	97185.7793	95196.69	95981.29	94812.43	95232.96	95305.8418	1.0197	0.4591
Q60597	Ogdh	2-oxoglutarate dehydrogenase complex component E1	336897.25	330139.9	359826.7	370898.1	349440.4688	357988.8	342234.8	334980.1	335520.7	342681.1094	1.0197	0.5603
Q6GYP7	Ralgapa1	Ral GTPase-activating protein subunit alpha-1	16474.36133	13644.83	12949.71	14616.02	14421.22852	12153.04	16110.87	13542.04	14763.16	14142.28076	1.0197	0.8149
Q9D7A8	Armc1	Armadillo repeat-containing protein 1	51856.99219	49686.81	49009.21	48031.38	49646.09668	48546.31	45166.23	48580.84	52451.31	48686.17285	1.0197	0.5918
Q99LS3	Psph	Phosphoserine phosphatase	43163.08594	48973.48	47885.42	48214.04	47059.00684	43062.36	44089.18	46290.37	51154.54	46149.11133	1.0197	0.6975
P61079	Ube2d3	Ubiquitin-conjugating enzyme E2 D3	111057.6641	111936.1	103549.4	106366.5	108227.4258	107284.6	114056.4	100414.8	102793.6	106137.3477	1.0197	0.5821
Q8BZ98	Dnm3	Dynamin-3	794096.625	821811.8	784490.7	822651	805762.5156	785469	799954.5	761796.1	814028.2	790311.9531	1.0195	0.3363
O08600	Endog	Endonuclease G, mitochondrial	58197.88672	57870.55	56736.8	61713.06	58629.57422	57982.08	59357.95	57359.14	55325.9	57506.26758	1.0195	0.4413
Q8R191	Syngr3	Synaptogyrin-3	92900.84375	92682.37	112788.6	126942.6	106328.5957	103316.2	78722.77	117330.1	117839.7	104302.1973	1.0194	0.8754
P69566	Ranbp9	Ran-binding protein 9	6698.103516	8306.725		8254.356	7753.061523	2960.336	7365.674	12232.55	7864.847	7605.852234	1.0194	0.9511
Q8BWF0	Aldh5a1	Succinate-semialdehyde dehydrogenase, mitochondrial	310056.1563	289140.6	277993.8	309224.8	296603.8281	315415.6	300985.9	279440.2	268074.7	290979.1016	1.0193	0.6853
Q06185	Atp5me	ATP synthase subunit e, mitochondrial	1403110.125	1408751	1474667	1570005	1464133.031	1479047	1532094	1312863	1421533	1436384.344	1.0193	0.6649
Q07813	Bax	Apoptosis regulator BAX	10522.17969	9752.448	9031.277	9303.311	9652.303955	7586.429	8853.189	9043.862	12395.09	9469.641968	1.0193	0.8710
Q9CXT8	Pmpcb	Mitochondrial-processing peptidase subunit beta	52370.21875	48924.69	54425.93	58193.89	53478.68164	44663.79	50151.47	58718.7	56352.84	52471.70117	1.0192	0.7954
Q921M4	Golga2	Golgin subfamily A member 2	21126.50195	21319.05	14911.28	14467.89	17956.18091	9796.277	18740.67	18195.66	23741.77	17618.59521	1.0192	0.9253
Q9JJV5	Cacng3	Voltage-dependent calcium channel gamma-3 subunit	17104.8418	14768.08	11908.05	19193.16	15743.53223	15977.67	15185.74	14574.05	16053.95	15447.85522	1.0191	0.8599
Q62148	Aldh1a2	Retinal dehydrogenase 2	85695.66406	77962.43	72329.66	75045.68	77758.35742	79556.06	77664.55	78616.84	69370.84	76302.07422	1.0191	0.7087
Q8C522	Endod1	Endonuclease domain-containing 1 protein	381881.375	347192.6	367651.3	341597.2	359580.5938	344229.9	359521	340392	367324.8	352866.9297	1.0190	0.5730
Q9CPU2	Ndufb2	NADH dehydrogenase [ubiquinone] 1 beta subcomplex subunit 2, mitochor	260978.3125	246048.2	273578	258411.9	259754.1016	283811.7	220223.9	273291.3	242330.3	254914.2969	1.0190	0.7667

Q9DCT8	Crip2	Cysteine-rich protein 2	27893.44922	37186.77	29880.04	29490.09	31112.58838	34858.29	36443.38	22566.24	28268.54	30534.1123	1.0189	0.8841
Q9EP53	Tsc1	Hamartin	10744.37305	12683.1	12503.79	11877.02	11952.07202	13093.29	11678.16	11084.06	11064.9	11730.10376	1.0189	0.7433
O70293	Grk6	G protein-coupled receptor kinase 6	7980.390625	9947.741	12236.08	7074.694	9309.726807	11600.27	7085.355	9091.876	8770.604	9137.026611	1.0189	0.9107
Q5NCF2	Trappc1	Trafficking protein particle complex subunit 1	48541.42578	49051.5	51813.3	52873.64	50569.9668	48494.52	52607.13	50727.29	46699.2	49632.03809	1.0189	0.5934
Q9JM96	Cdc42ep4	Cdc42 effector protein 4	21241.2207	19894.02	25018.53	21628.87	21945.66016	21974.8	23759.03	19966.4	20455.97	21539.05029	1.0189	0.7789
P70441	Nherf1	Na(+)/H(+) exchange regulatory cofactor NHE-RF1	74623.35938	71365.93	71566.93	76108.06	73416.07031	66542.32	70659.95	75437.48	75591.52	72057.81836	1.0188	0.6008
P46664	Adss2	Adenylosuccinate synthetase isozyme 2	112485.1172	115333	123152.5	120180.2	117787.707	124487.8	114014.9	107369.1	116569.3	115610.2441	1.0188	0.6283
Q9QUR7	Pin1	Peptidyl-prolyl cis-trans isomerase NIMA-interacting 1	130643.375	128216.7	147689.8	136171.4	135680.3105	125950.6	124679.7	135026.4	147097.5	133188.541	1.0187	0.7248
P62320	Snrpd3	Small nuclear ribonucleoprotein Sm D3	176861.9531	170643.9	161316	181067.3	172472.3164	182287.2	190000.4	148171.2	156773.5	169308.0703	1.0187	0.7810
O08756	Hsd17b10	3-hydroxyacyl-CoA dehydrogenase type-2	196696.2188	197848.1	203615.8	202211	200092.7813	199843.5	205103	197515	183270.8	196433.0742	1.0186	0.4881
Q8BWL5	Rbms3	RNA-binding motif, single-stranded-interacting protein 3		15233.78			15233.77832			14335.99	15575.91	14955.94824	1.0186	
P62301	Rps13	Small ribosomal subunit protein uS15	411756.5	400471.1	420898.2	428472.6	415399.6172	406449	423981.5	406123.6	394741.5	407823.8906	1.0186	0.4089
Q9D358	Acp1	Low molecular weight phosphotyrosine protein phosphatase	260925.9531	305677.5	271881	270229	277178.3789	262856.4	265147.7	289181.1	271327.6	272128.1797	1.0186	0.6752
Q9WUB4	Dctn6	Dynactin subunit 6	32769.53125	35746.68	36351.85	33928.08	34699.03613	40846.43	33999.07	27710.97	33711.61	34067.021	1.0186	0.8294
Q9JLN9	Mtor	Serine/threonine-protein kinase mTOR	47315.38672	42511.07	41173.8	52978.25	45994.62598	48132.41	47248.77	42627.24	42620.03	45157.1123	1.0185	0.7931
Q9CWS0	Ddah1	N(G),N(G)-dimethylarginine dimethylaminohydrolase 1	293786.5313	286155	339321.2	305289.2	306137.9844	310763.4	298085	282295.7	311135.9	300570.0078	1.0185	0.6958
Q91Z31	Ptbp2	Polypyrimidine tract-binding protein 2	99499.53125	99549.27	89593.52	102325.6	97741.98242	105089.4	103618.4	87536.71	87616.88	95965.35352	1.0185	0.7618
Q9EQZ6	Rapgef4	Rap guanine nucleotide exchange factor 4	18020.65625	17364.09	15168.31	18495.58	17262.15796	16018.41	17708.69	18360.73	15707.2	16948.75684	1.0185	0.7594
Q9CQE1	Nipsnap3b	Protein NipSnap homolog 3B	40837.24219	34528.4	32435.11	28490.22	34072.74463	40144.27	36678.67	25647.48	31354.47	33456.2207	1.0184	0.8850
Q6PD28	Ppp2r5b	Serine/threonine-protein phosphatase 2A 56 kDa regulatory subunit beta is	46371.35547	51308.32	52810.01	41206.33	47924.00488	48570.28	51634.57	40372.11	47652.5	47057.36719	1.0184	0.8152
P19253	Rpl13a	Large ribosomal subunit protein uL13	189861.4063	183968.6	174977.7	184841.7	183412.3359	198026.6	199686.5	160013.5	162778.5	180126.2656	1.0182	0.7804
Q3U7R1	Esyt1	Extended synaptotagmin-1	212219.4844	212034.2	238456.2	219519.3	220557.3047	211158.6	216501.5	219383	219404.3	216611.8672	1.0182	0.5668
Q6DFV7	Ncoa7	Nuclear receptor coactivator 7	6433.924805	4439.427	4367.402	3554.219	4698.743286	5877.182	5019.521	4020.914	3541.225	4614.710571	1.0182	0.9202
O54988	Slk	STE20-like serine/threonine-protein kinase	19831.76172	26115.18	22683.33	22647.66	22819.48242	27789.27	17963.04	25785.01	18114.25	22412.89404	1.0181	0.8917
Q9D7G0	Prps1	Ribose-phosphate pyrophosphokinase 1	149600.5313	167006.1	163551.2	149002.8	157290.1289	156037.4	149688.7	151738.1	160516.8	154495.2461	1.0181	0.6136
P47809	Map2k4	Dual specificity mitogen-activated protein kinase kinase 4	77713.01563	82016.44	68523.53	79045.65	76824.6582	72454.46	70144.52	73277.96	85963.06	75460	1.0181	0.7767
Q91XL9	Osbpl1a	Oxysterol-binding protein-related protein 1	98239.625	106882.8	97220.73	100437.4	100695.1367	98547.87	95123.13	97317.29	104690.2	98919.62891	1.0179	0.5736
Q9JLR1	Sec61a2	Protein transport protein Sec61 subunit alpha isoform 2	26154.62109	10119.06	13701.27	21800.35	17943.8252	15277.31	19620.06	17739.42	17874.23	17627.75562	1.0179	0.9360
P26048	Gabra2	Gamma-aminobutyric acid receptor subunit alpha-2	8483.921875		8726.288	14313.22	10507.81022	9108.709	8415.932	15880.21	7886.392	10322.81116	1.0179	0.9485
Q62277	Syp	Synaptophysin	753595.25	732339.6	720916.4	863774.3	767656.375	832440.6	787442.4	750444.3	646372.1	754174.8594	1.0179	0.8020
P30416	Fkbp4	Peptidyl-prolyl cis-trans isomerase FKBP4	128338.6953	134223.8	126177.9	109951.7	124673.0195	136253.3	131788.6	103576.8	118346.5	122491.2852	1.0178	0.8168
Q8K2M0	Mrpl38	Large ribosomal subunit protein mL38	14937.98926	12163.01	11569.17	17541.52	14052.92212	11889.91	14219.44	16493.21	12627.33	13807.47461	1.0178	0.8907
P62069	Usp46	Ubiquitin carboxyl-terminal hydrolase 46	42048.82031	38924.86	40554.93	31015.64	38136.0625	31923.94	41178.46	34371.82	42408.16	37470.59473	1.0178	0.8574
Q9D1I6	Mrpl14	Large ribosomal subunit protein uL14m	11433.14941	11387.28	10175.57	11384.66	11095.16455	11824.58	11399.24	10618.33	9764.806	10901.74048	1.0177	0.7361
Q3UH60	Dip2b	Disco-interacting protein 2 homolog B	62892.75781	60459.68	60218.24	66753.61	62581.07031	60063.23	62893.27	59195.71	63811.25	61490.86426	1.0177	0.5824
Q8VDP4	Ccar2	Cell cycle and apoptosis regulator protein 2	42940.39844	46040.93	46074.13	50474.86	46382.58008	44755.58	48684.02	41339.61	47528.05	45576.81445	1.0177	0.7328
P61804	Dad1	Dolichyl-diphosphooligosaccharide--protein glycosyltransferase subunit D/	42459.57031	45702.8	52058.67	46541.04	46690.52051	49277.03	44748.28	42603.71	46904.52	45883.38477	1.0176	0.7535
Q9QUJ7	Acsl4	Long-chain-fatty-acid--CoA ligase 4	23885.70703	22424.74	30310.94	31817.89	27109.81689	29314.33	27685.73	24647.93	24917.67	26641.41602	1.0176	0.8620
P62631	Eef1a2	Elongation factor 1-alpha 2	1429419.875	1586066	1492072	1616483	1531010.125	1592422	1600666	1410280	1414973	1504585.031	1.0176	0.7123
Q8CCB4	Vps53	Vacuolar protein sorting-associated protein 53 homolog	32492.68945	33265.04	33295.05	34044.93	33274.43018	33107.3	32450.69	33017.6	32232.26	32701.96338	1.0175	0.1848
P40336	Vps26a	Vacuolar protein sorting-associated protein 26A	33559.08594	31451.13	31729.97	32430.93	32292.77734	31427.3	35991.55	31441.92	28091.79	31738.13916	1.0175	0.7537
P23591	Gfus	GDP-L-fucose synthase	22395.3457	19937.36	25003.43	20932.95	22067.27148	19059.44	23906.76	20491.14	23304.65	21690.49609	1.0174	0.8208
P58774	Tpm2	Tropomyosin beta chain	168455.7656	144686.1	191891.2	199359.8	176098.2266	161522.3	186122.3	167595.2	177127.9	173091.918	1.0174	0.8311
Q9WUL7	Arl3	ADP-ribosylation factor-like protein 3	187601.2031	187402.7	172117.3	175928	180762.2852	201515.1	173060.4	160467.5	175666.9	177677.4688	1.0174	0.7560
Q8BML9	Qars1	Glutamine--tRNA ligase	38847.92188	34858.35	34312.95	32042.86	35015.51953	35346.73	35186.39	33184.75	33955.59	34418.36523	1.0173	0.7055
Q9WUM3	Coro1b	Coronin-1B	62412.85547	57774.93	55794.56	65370.57	60338.22852	58820.01	62716.61	53391.62	62320.06	59312.07422	1.0173	0.7492
Q91VE0	Slc27a4	Long-chain fatty acid transport protein 4	110136.7422	106216	98876.83	92275.32	101876.2246	105575.7	106612.3	94317.25	94071.43	100144.1621	1.0173	0.7526
Q62422	Ostf1	Osteoclast-stimulating factor 1	77572.49219	88294.77	89216.84	86379.13	85365.80664	69249.2	87583.56	103229.8	75623.08	83921.39648	1.0172	0.8615
Q7TQH0	Atxn2l	Ataxin-2-like protein	27699.18164	27111.77	25859.02	26331.87	26750.45947	29784.66	27368.7	23902.15	24142.88	26299.59912	1.0171	0.7684
P12970	Rpl7a	Large ribosomal subunit protein eL8	150180.1094	145870.2	153184.8	149253	149622.0117	151506.9	158459.1	130003.5	148432	147100.3809	1.0171	0.7009
Q8CG72	Adprs	ADP-ribosylhydrolase ARH3	24019.75	24320.67	22122.66	24074.21	23634.32324	22041.41	23104.74	25186.6	22614	23236.69043	1.0171	0.6576
Q8CAQ8	Immt	MICOS complex subunit Mic60	320077	319379.3	337604.5	350517.5	331894.5625	331594.3	321741.9	328660.2	323285.4	326320.4531	1.0171	0.5042
Q9CY28	Gtpbp8	GTP-binding protein 8	10826.0957	11234.21	11161.86	12662.71	11471.21704	11028.09	8325.838	14527.51	11240.01	11280.36377	1.0169	0.8908
Q6R891	Ppp1r9b	Neurabin-2	32344.39063	30034.43	31686.99	32450.58	31629.09619	34469.57	30889.99	31076.95	27978.61	31103.78027	1.0169	0.7277
O70250	Pgam2	Phosphoglycerate mutase 2	656260.5	625374.8	487213.5	603150.8	592999.8984	629043.4	595173.2	463964.7	644425.4	583151.6875	1.0169	0.8643
O54786	Dffa	DNA fragmentation factor subunit alpha	27708.97852	28658.93	31124.92	22737.21	27557.51172	27346.32	24397.4	26863.17	29798.17	27101.26465	1.0168	0.8336
P62309	Snrpg	Small nuclear ribonucleoprotein G	56549.49219	54941.39	59316.28	66085.79	59223.23926	65757.58	65648.88	48148.82	53419.09	58243.59082	1.0168	0.8533
Q60634	Flot2	Flotillin-2	102636.5703	98952.49	104061.3	118668.6	106079.7363	107396.5	105976	104182.5	99748.15	104325.7871	1.0168	0.7184
Q9JIS5	Sv2a	Synaptic vesicle glycoprotein 2A	121123.5391	125138.7	113860.5	122876.2	120749.7363	115408.3	129668.1	112573.8	117364.8	118753.7422	1.0168	0.6722
Q9CR16	Ppid	Peptidyl-prolyl cis-trans isomerase D	93424.34375	94511.82	74590.55	77273.98	84950.17383	97122.34	90352.76	65609.13	81102.34	83546.64453	1.0168	0.8757
Q9JHW2	Nit2	Omega-amidase NIT2	33167.45313	32678.41	34328.32	32413.32	33146.87402	33573.18	32498.05	32599.26	31726.65	32599.28564	1.0168	0.3724
Q8R1Q8	Dync1li1	Cytoplasmic dynein 1 light intermediate chain 1	149813.0313	150528.1	158677.2	150799.4	152454.4258	151247	147937	151339.1	149248	149942.7773	1.0168	0.3054
Q99LC5	Etfa	Electron transfer flavoprotein subunit alpha, mitochondrial	455554.125	468049.8	488262.8	469952	470454.6719	448380.3	495689.4	449389.7	457459.3	462729.6641	1.0167	0.5755
Q8K354	Cbr3	Carbonyl reductase [NADPH] 3	78120.28906	97735.73	104673.5	109717.5	97561.75391	96257.2	86472.52	92046.24	109075.3	95962.82422	1.0167	0.8559
Q8K1Z0	Coq9	Ubiquinone biosynthesis protein COQ9, mitochondrial	43098.80859	37561.78	44141.38	43829.91	42157.96875	36176.13	39958.16	46757.46	42977.52	41467.31641	1.0167	0.8086

Q8BR90	Rimoc1	RAB7A-interacting MON1-CCZ1 complex subunit 1	19185.36328	18384.76	15659.24	17390.04	**17654.85107**	14531.91	17057.31	19358.94	18516.58	**17366.18457**	**1.0166**	**0.8319**
B1AY13	Usp24	Ubiquitin carboxyl-terminal hydrolase 24	10916.09766	11591.79	7191.921	13527.7	**10806.87769**	12867.93	8254.99	11837.86	9561.474	**10630.56201**	**1.0166**	**0.9204**
Q4VBD2	Tapt1	Transmembrane anterior posterior transformation protein 1	20830.93555	17166.12	14313.48	15007.01	**16829.38452**	14459.71	19742.15	16736.84	15281.53	**16555.05615**	**1.0166**	**0.8882**
P40142	Tkt	Transketolase	787200.3125	848740.3	765655.1	778994.6	**795147.5625**	796486.4	790924.5	743865.8	797746.2	**782255.7031**	**1.0165**	**0.5870**
P35288	Rab23	Ras-related protein Rab-23	39979.42578	38678.08	34445.04	34492.66	**36898.7998**	31762.59	36771.7	43064.91	33609.86	**36302.26465**	**1.0164**	**0.8418**
Q61263	Soat1	Sterol O-acyltransferase 1	3320.393066	2174.576	2255.191		**2583.386963**	1284.705		3798.795		**2541.750305**	**1.0164**	**0.9709**
Q64332	Syn2	Synapsin-2	826293.25	820490	780219.6	906259.2	**833315.5156**	850379.6	831728.9	822062.1	775467	**819909.375**	**1.0164**	**0.6788**
Q80XA6	Reps2	RalBP1-associated Eps domain-containing protein 2	12240.27734	10732.85	8587.465	9879.256	**10359.96094**	8620.918	9936.093	11272.59	10943.86	**10193.36523**	**1.0163**	**0.8694**
P70408	Cdh10	Cadherin-10	11983.1084	10074.56	8885.396	10216.57	**10289.90869**	12411.16	10121.49	8871.495	9094.561	**10124.67676**	**1.0163**	**0.8779**
Q9Z0E6	Gbp2	Guanylate-binding protein 2		2925.483	1.757554		**1463.620354**			1440.238		**1440.237793**	**1.0162**	
P52480	Pkm	Pyruvate kinase PKM	5541521	5793959	5844084	5693265	**5718207**	5503439	5770500	5447632	5786846	**5627104**	**1.0162**	**0.4419**
Q99LC3	Ndufa10	NADH dehydrogenase [ubiquinone] 1 alpha subcomplex subunit 10, mitoch	591000.5625	543039.9	551267.2	571112.8	**564105.1094**	574531.4	567054.4	534814	544084.4	**555121.0313**	**1.0162**	**0.5514**
Q99PV0	Prpf8	Pre-mRNA-processing-splicing factor 8	28696.3457	27820.36	24659.12	25389.32	**26641.28564**	27668.87	26921.74	26119.64	24162.37	**26218.15723**	**1.0161**	**0.7411**
P60879	Snap25	Synaptosomal-associated protein 25	651797.9375	633945.4	665337.8	757425.3	**677126.5938**	667739.7	637306.9	666556.2	694163.9	**666441.6875**	**1.0160**	**0.7329**
Q9QXV0	Pcsk1n	ProSAAS	752703.625	734742.9	774993.8	790771.3	**763302.9063**	741693.7	745376.3	806759.9	711399.5	**751307.3438**	**1.0160**	**0.6276**
Q8VEH5	Epm2aip1	EPM2A-interacting protein 1	64724.27344	65406.71	45636.42	59702.49	**58867.47461**	56868.48	59869.28	53478.22	61572.48	**57947.11523**	**1.0159**	**0.8579**
Q8BUV3	Gphn	Gephyrin	189206.75	189074.7	182279.3	177928.3	**184622.2461**	187418.6	188858	173287.7	177415.2	**181744.8633**	**1.0158**	**0.5622**
Q60780	Gas7	Growth arrest-specific protein 7	9682.342773	6777.164	10775.47		**9078.324219**	7377.826	7147.412	14639.41	6582.988	**8936.908569**	**1.0158**	**0.9565**
Q8K0L9	Zbtb20	Zinc finger and BTB domain-containing protein 20	937.0991211				**937.0991211**	634.3627			1210.722	**922.5422974**	**1.0158**	
P11983	Tcp1	T-complex protein 1 subunit alpha	337767.9375	340002.5	325726.9	339399.6	**335724.25**	353613.4	338592.8	324932	304919.6	**330514.4531**	**1.0158**	**0.6491**
Q8R0P4	Aamdc	Mth938 domain-containing protein	68094.70313	67540.53	63173.9	63165.29	**65493.60645**	66437.41	67060.88	53963.6	70453.84	**64478.93359**	**1.0157**	**0.8013**
O35474	Edil3	EGF-like repeat and discoidin I-like domain-containing protein 3	21571.75586	18600.5	24709.54	21815.05	**21674.20996**	20154.1	20477.74	24028.29	20694.28	**21338.60107**	**1.0157**	**0.8348**
Q9QZH6	Ecsit	Evolutionarily conserved signaling intermediate in Toll pathway, mitochondi	10206.91699	9730.842	11268.45	8629.466	**9958.91748**	9180.298	8236.46	12650.21	9153.27	**9805.058105**	**1.0157**	**0.8949**
P63328	Ppp3ca	Protein phosphatase 3 catalytic subunit alpha	446160.0625	426313.3	465246.8	443749.7	**445367.4297**	427035.5	420162.9	449727.8	457064.6	**438497.6797**	**1.0157**	**0.5848**
O55186	Cd59a	CD59A glycoprotein	2255.853516	3536.319	1806.836		**2533.003092**	3270.91	1004.951	1465.458	4235.42	**2494.18483**	**1.0156**	**0.9705**
Q8R2Y8	Ptrh2	Peptidyl-tRNA hydrolase 2, mitochondrial	53339.26172	57928.84	69487.86	69921.82	**62669.44629**	52494.97	61106.3	74315.38	58920.15	**61709.20313**	**1.0156**	**0.8819**
Q78IK2	Atp5mk	ATP synthase membrane subunit K, mitochondrial	529833.25	507477.8	503747.4	535711.6	**519192.5**	504735.3	505753	497971.3	536493.9	**511238.3516**	**1.0156**	**0.5226**
P51175	Ppox	Protoporphyrinogen oxidase	8431.591797	12443.13	14337.99	13042.76	**12063.86816**	9755.137	15123.17	11763.72	10874.16	**11879.04712**	**1.0156**	**0.9180**
Q8BTZ7	Gmppb	Mannose-1-phosphate guanyltransferase beta	55904.20703	52267.22	54702.38	49564.18	**53109.49609**	51305.8	54205.25	47439.77	56238.04	**52297.21777**	**1.0155**	**0.7434**
Q925N0	Sfxn5	Sideroflexin-5	239989.6719	271812.7	261448.5	285831.2	**264770.5**	264821.6	267446.3	273000.4	237646.3	**260728.6406**	**1.0155**	**0.7567**
P63276	Rps17	Small ribosomal subunit protein eS17	80503.03125	93099.59	91099.69	86281.32	**87745.9082**	89185.8	87371	91582.47	77497.72	**86409.24609**	**1.0155**	**0.7598**
Q9CR60	Golt1b	Vesicle transport protein GOT1B	22687.92773	23880.69	22751.59	21252.44	**22643.16113**	23065.33	21934.61	23204.47	20995.5	**22299.97803**	**1.0154**	**0.6626**
Q9WV60	Gsk3b	Glycogen synthase kinase-3 beta	88032.46875	93052.91	87656.33	91953.06	**90173.69336**	93150.7	92349.91	86080.23	83670.5	**88812.83594**	**1.0153**	**0.6325**
Q9R1X5	Abcc5	ATP-binding cassette sub-family C member 5	12367.66504	13864.77	10258.82	16145.91	**13159.29199**	13422.5	10952.34	12037.27	15433.25	**12961.33789**	**1.0153**	**0.9039**
Q8BXJ9	Tmem62	Transmembrane protein 62	9394.311523	8392.449	5619.314	5898.043	**7326.029541**	6715.684		7778.892	7153.145	**7215.906901**	**1.0153**	**0.9261**
O70228	Atp9a	Probable phospholipid-transporting ATPase IIA	31867.71094	30765.61	33532.29	36358.35	**33130.98926**	31960.24	34009.12	34089.84	30474.61	**32633.45215**	**1.0152**	**0.7509**
Q6PGN3	Dclk2	Serine/threonine-protein kinase DCLK2	62802.92188	63639.5	74291.86	82107.7	**70710.49512**	68820.69	69957.24	73082.7	66735.39	**69649.00391**	**1.0152**	**0.8323**
Q64520	Guk1	Guanylate kinase	62013.80859	59232.14	60042.79	55809.07	**59274.45117**	52272.05	49332.62	61330	70616.5	**58387.79102**	**1.0152**	**0.8646**
Q5XKN4	Jagn1	Protein jagunal homolog 1	12256.08301	24907.17	18928.69	20130.02	**19055.48901**	26240.28	10163.6	17027.63	21650.34	**18770.4624**	**1.0152**	**0.9494**
Q8BX57	Pxk	PX domain-containing protein kinase-like protein	12486.93164	10005.64	10093.71	8445.392	**10257.91724**	10164.56	12152.18	7687.329	10416.55	**10105.15308**	**1.0151**	**0.9060**
B2RXC1	Trappc11	Trafficking protein particle complex subunit 11	21333.61914	20082.61	19046.95	20306.6	**20192.4458**	20325.8	20710.57	19483.32	19049.49	**19892.29395**	**1.0151**	**0.6368**
P80315	Cct4	T-complex protein 1 subunit delta	214452.5781	213135.3	211536.1	225421.9	**216136.4648**	225279.6	215858.6	201857.3	208703.6	**212924.7773**	**1.0151**	**0.6071**
O70161	Pip5k1c	Phosphatidylinositol 4-phosphate 5-kinase type-1 gamma	59183.19922	61499.9	54337.1	58281.66	**58325.46484**	58399.62	54974.61	58959.41	57515.43	**57462.26855**	**1.0150**	**0.6361**
P26049	Gabra3	Gamma-aminobutyric acid receptor subunit alpha-3	32388.43945	34813.1	32220.2	43405.04	**35706.69385**	36702.66	38178.73	29062.22	36782.8	**35181.60498**	**1.0149**	**0.8805**
Q9D7S7	Rpl22l1	Ribosomal protein eL22-like 1	16330.32715	10302.8	9174.516	17843.01	**13412.66382**	13124.27	11194.62	14221.69	14324.9	**13216.36719**	**1.0149**	**0.9340**
P70452	Stx4	Syntaxin-4	25466.79883	26001.72	27071.53	29802.51	**27085.6416**	25657.74	27957.88	24589.7	28567.01	**26693.08154**	**1.0147**	**0.7806**
Q91XD6	Vps36	Vacuolar protein-sorting-associated protein 36	20086.98828	19704.51	18849.79	19320.66	**19490.4873**	18361.22	19647.9	20068.89	18755.61	**19208.40576**	**1.0147**	**0.5736**
Q8VE70	Pdcd10	Programmed cell death protein 10	39656.42969	43124.65	43281.68	42801.41	**42216.04297**	45280.7	41462.97	39785.57	39896.65	**41606.47559**	**1.0147**	**0.7067**
P50518	Atp6v1e1	V-type proton ATPase subunit E 1	558360	552163.8	557591	608247	**569090.4531**	589579.8	571381.4	516082.5	566897.5	**560985.3047**	**1.0144**	**0.7063**
Q8K0U4	Hspa12a	Heat shock 70 kDa protein 12A	1255889.5	1194285	1355520	1257733	**1265856.813**	1270400	1227717	1196043	1297252	**1247852.625**	**1.0144**	**0.6698**
Q64487	Ptprd	Receptor-type tyrosine-protein phosphatase delta	67596.60156	45487.91	62688.66	43478.38	**54812.88867**	61914.94	60527.33	43932.24	49777.75	**54038.06543**	**1.0143**	**0.9205**
A6X919	Dpy19l1	Probable C-mannosyltransferase DPY19L1	14630.17871	15813.49		9061.817	**13168.49674**	15059.87		10905.48		**12982.67139**	**1.0143**	**0.9560**
Q99JW2	Acy1	Aminoacylase-1	115670.8984	124031.1	112931.7	111324.2	**115989.4785**	115002	106510.2	114991.3	120935.8	**114359.8008**	**1.0143**	**0.7047**
Q8R050	Gspt1	Eukaryotic peptide chain release factor GTP-binding subunit ERF3A	28507.02344	30513.52	29074.84	24676.88	**28193.06885**	28308.46	25154.23	30251.25	27474.31	**27797.06201**	**1.0142**	**0.8165**
Q3UVK0	Ermp1	Endoplasmic reticulum metallopeptidase 1	51198.1875	51258.07	46228.13	46499.16	**48795.88477**	49304.63	52687.92	49893.4	40556.85	**48110.7002**	**1.0142**	**0.8256**
O08599	Stxbp1	Syntaxin-binding protein 1	1269608.75	1302265	1220900	1428734	**1305376.844**	1335076	1320155	1259016	1233985	**1287057.781**	**1.0142**	**0.7294**
Q9DAW9	Cnn3	Calponin-3	100507.875	105705.8	94637.84	101493.1	**100586.1641**	102594.3	100615.2	91843.47	101672.4	**99181.33789**	**1.0142**	**0.6912**
P61255	Rpl26	Large ribosomal subunit protein uL24	204832.6875	223168.3	167215.7	209087.7	**201076.0703**	222781.8	206490.6	193053.1	170782.4	**198276.957**	**1.0141**	**0.8688**
Q8BGD8	Coa6	Cytochrome c oxidase assembly factor 6 homolog	41447.45703	37207.97	34453.8	39200.89	**38077.53125**	37130.43	38368.79	37198.26	37492.57	**37547.51465**	**1.0141**	**0.7381**
P70236	Map2k6	Dual specificity mitogen-activated protein kinase kinase 6	29957.38086	31240.13	32981.58	27378.22	**30389.3291**	27855.19	26429.66	35400.1	30183.29	**29967.06006**	**1.0141**	**0.8601**
Q61879	Myh10	Myosin-10	181790.0156	184160.2	191349	180905.1	**184551.0938**	170570.9	190111.6	187830.6	179462.5	**181993.9102**	**1.0141**	**0.6296**
P80316	Cct5	T-complex protein 1 subunit epsilon	221657.4531	212529.3	203622.8	203935.1	**210436.1563**	210192.9	224814.6	191196.4	203949.2	**207538.2617**	**1.0140**	**0.7355**
Q8BIW1	Prune1	Exopolyphosphatase PRUNE1	27418.08203	26744.5	27794.85	29006.45	**27740.97314**	27916.49	26849.15	25227.66	29445.58	**27359.71924**	**1.0139**	**0.7180**
O35129	Phb2	Prohibitin-2	399678.5	406310.4	391522.9	412310.2	**402455.5**	418617	400568.3	400831.5	367773.5	**396947.5547**	**1.0139**	**0.6490**
Q3ULD5	Mccc2	Methylcrotonoyl-CoA carboxylase beta chain, mitochondrial	49591.00391	50771.12	49459.09	52908.52	**50682.43262**	53330.2	51258.37	49032.78	46338.11	**49989.86426**	**1.0139**	**0.6978**

Q8VDL4	Adpgk	ADP-dependent glucokinase	37462.26953	38046.49	44708.51	37937.23	**39538.62598**	43217.01	37161.35	34617.62	41000.62	**38999.14844**	**1.0138**	**0.8416**
P15508	Sptb	Spectrin beta chain, erythrocytic	176416.5625	178326.9	174820.6	175286.2	**176212.5508**	186040.6	177856.2	156848.1	174522.6	**173816.8789**	**1.0138**	**0.7126**
Q9EQH3	Vps35	Vacuolar protein sorting-associated protein 35	169699.2031	167448.4	162247.3	182869.8	**170566.1641**	168309.1	176119.7	162389.9	166191.9	**168252.6406**	**1.0138**	**0.6753**
P61458	Pcbd1	Pterin-4-alpha-carbinolamine dehydratase	41112.72656	45391.17	42381.59	42113.53	**42749.75293**	39268.61	39871.39	42223.89	47324.47	**42172.08691**	**1.0137**	**0.7877**
P14211	Calr	Calreticulin	501332.6875	490044.7	451904.1	491417.9	**483674.8516**	517027.5	540417.9	405428.5	445756.4	**477157.5703**	**1.0137**	**0.8504**
P97742	Cpt1a	Carnitine O-palmitoyltransferase 1, liver isoform	71662.91406	74459.68	68217.74	68116.95	**70614.32227**	69917.07	70363.63	71272.73	67098.92	**69663.08984**	**1.0137**	**0.6103**
P06801	Me1	NADP-dependent malic enzyme	187195.875	199642.8	173238.1	181037.9	**185278.6641**	192046.8	195636.6	175320.8	168180.9	**182796.2773**	**1.0136**	**0.7832**
Q501J6	Ddx17	Probable ATP-dependent RNA helicase DDX17	274311.3438	289048.8	265676.9	255268.3	**271076.332**	289491.8	278045.8	265899.6	236342.8	**267445.0156**	**1.0136**	**0.7967**
P10922	H1-0	Histone H1.0	290262.0313	263941.9	289243.7	260548.6	**275999.0547**	255110	315048.9	265240	253808.8	**272301.918**	**1.0136**	**0.8304**
Q6NXK7	Dpp10	Inactive dipeptidyl peptidase 10	30385.58789	24722.81	25188.02	27889.68	**27046.52344**	29019.84	28309.34	26028.3	23394.41	**26687.97266**	**1.0134**	**0.8509**
Q9QYF1	Rdh11	Retinol dehydrogenase 11	30676.01953	30858.89	28536.72	28706.73	**29694.59082**	33764.73	31317.99	27027.44	25093.71	**29300.96924**	**1.0134**	**0.8556**
Q01853	Vcp	Transitional endoplasmic reticulum ATPase	683409.625	682954.3	737332.6	731381.4	**708769.4688**	703960.6	707444.3	672281.4	713911.5	**699399.4688**	**1.0134**	**0.6113**
Q9D824	Fip1l1	Pre-mRNA 3'-end-processing factor FIP1	10501.55957	5867.582	8016.224	7130.72	**7879.021362**	7770.299	10341.18	5929.17	7060.313	**7775.241455**	**1.0133**	**0.9414**
Q9Z2Q6	Septin5	Septin-5	122841.3672	126534.1	113153.4	118562.1	**120272.7422**	105132.5	127112.3	116761.1	125764.7	**118692.6309**	**1.0133**	**0.7955**
Q8VHL1	Setd7	Histone-lysine N-methyltransferase SETD7	42769.11719	44544.9	40026.09	37214.79	**41138.72461**	39302.34	43171.16	40839.13	39095.46	**40602.02246**	**1.0132**	**0.7826**
Q9CQV8	Ywhab	14-3-3 protein beta/alpha	1537744.875	1584861	1395535	1321899	**1460009.969**	1427675	1623811	1231571	1480992	**1441012.281**	**1.0132**	**0.8579**
Q8BI08	Mal2	Protein MAL2	105621.0078	93720.62	90410.09	113995.1	**100936.6953**	108750.1	102741.3	93424.54	93645.45	**99640.3457**	**1.0130**	**0.8507**
A2A5R2	Arfgef2	Brefeldin A-inhibited guanine nucleotide-exchange protein 2	40338.77734	37988.31	37589.07	39328.31	**38811.11719**	38678.1	38539.04	38400.93	37634.31	**38313.09375**	**1.0130**	**0.4868**
Q8R4H2	Arhgef12	Rho guanine nucleotide exchange factor 12	25759.04492	18790.26	22821.85	24165.92	**22884.26563**	21327.56	25172.98	21555.16	22307.6	**22590.82422**	**1.0130**	**0.8712**
O55042	Snca	Alpha-synuclein	246179.7813	229099.7	207374.3	238179.9	**230208.4063**	238356.6	266553.9	199130	205007.5	**227261.9727**	**1.0130**	**0.8738**
Q7TSJ2	Map6	Microtubule-associated protein 6	667320.75	615736	654468.8	724740.6	**665566.5156**	656137.4	654154.3	639478.4	678458.9	**657057.25**	**1.0130**	**0.7346**
Q78PG9	Ccdc25	Coiled-coil domain-containing protein 25	18371.7793	11316.65	13056.7	15155.19	**14475.07861**	9822.578	20864.25	12821.67	13653.1	**14290.40186**	**1.0129**	**0.9494**
O08532	Cacna2d1	Voltage-dependent calcium channel subunit alpha-2/delta-1	75008.57031	75362.28	76182.05	86215.8	**78192.17578**	77180.73	77615.7	81451.66	72540.19	**77197.07031**	**1.0129**	**0.7696**
Q9WUR2	Eci2	Enoyl-CoA delta isomerase 2	63149.95703	70903.38	68425.13	71588.16	**68516.65723**	69464.8	72743.02	65209.38	63161.61	**67644.70215**	**1.0129**	**0.7720**
O35098	Dpysl4	Dihydropyrimidinase-related protein 4	67423.57813	67782.6	52176.63	59897.75	**61820.13867**	70703.03	70308.01	49839.69	53284.89	**61033.90332**	**1.0129**	**0.9096**
P52432	Polr1c	DNA-directed RNA polymerases I and III subunit RPAC1	71563.10938	71356.59		49776.1	**64231.9349**	79915.62	69817.95		40512.27	**63415.27995**	**1.0129**	**0.9558**
P17879	Hspa1b	Heat shock 70 kDa protein 1B	211854.9844	205579.5	238921.3	194493.9	**212712.4258**	232534	204535.2	184406.1	218634.8	**210027.5156**	**1.0128**	**0.8538**
P63085	Mapk1	Mitogen-activated protein kinase 1	269581.2813	267736.3	248744.8	239054.9	**256279.3086**	264015.7	264600.6	232146.2	251454.4	**253054.2344**	**1.0127**	**0.7717**
Q91V61	Sfxn3	Sideroflexin-3	463851.25	465510.6	521291.5	521833.9	**493121.8203**	482326.4	453105.1	512701	499789	**486980.375**	**1.0126**	**0.7786**
Q9CRD0	Ociad1	OCIA domain-containing protein 1	81977.00781	74341.62	72536	67136.26	**73997.7207**	79307.52	73303.37	71003.55	68706.96	**73080.34961**	**1.0126**	**0.8183**
Q7M753	Pank2	Pantothenate kinase 2, mitochondrial	10185.16309	6194.398	12991.05	11278.71	**10162.33044**	8707.208	12054.05	11276.13	8108.692	**10036.51978**	**1.0125**	**0.9445**
Q6VNB8	Wdfy3	WD repeat and FYVE domain-containing protein 3	19793.72266	15701.98	16000.09	18007.54	**17375.83447**	16674.35	17919.87	17490	16558.74	**17160.74072**	**1.0125**	**0.8383**
P16332	Mmut	Methylmalonyl-CoA mutase, mitochondrial	57475.81641	64624.16	69695.83	69150.2	**65236.50098**	65943.59	65449.82	65098.4	61228.13	**64429.98438**	**1.0125**	**0.7987**
P16283	Slc4a3	Anion exchange protein 3	9116.230469	9635.483	9741.558	9112.72	**9401.497803**	7363.865	9348.745	10834.41	9595.386	**9285.60083**	**1.0125**	**0.8803**
Q9D2R6	Coa3	Cytochrome c oxidase assembly factor 3 homolog, mitochondrial	35421.14453	29283.03	38294.59	30625.46	**33406.0542**	33173.93	28642.12	34544.77	35630.93	**32997.93701**	**1.0124**	**0.8803**
Q921T2	Tor1aip1	Torsin-1A-interacting protein 1	26490.82422	24674.85	28902.47	18490.62	**24639.68896**	24083.25	28792.4	26875.73	17609.35	**24340.18164**	**1.0123**	**0.9307**
Q9D710	Tmx2	Thioredoxin-related transmembrane protein 2	221778.4688	203422	221263.1	233979.2	**220110.6875**	207608.8	225117	234603	202423.7	**217438.1523**	**1.0123**	**0.7941**
Q3THK7	Gmps	GMP synthase [glutamine-hydrolyzing]	68582.05469	65842.1	63243.77	68642.22	**66577.53516**	66101.26	70826.15	58198.86	67955.86	**65770.53027**	**1.0123**	**0.7967**
Q80ZX8	Spag1	Sperm-associated antigen 1	26964.14258	22341.35	26516.01	27538.28	**25839.94482**	24917.79	25336.47	27207.28	24648.41	**25527.4873**	**1.0122**	**0.8205**
Q3V3R1	Mthfd1l	Monofunctional C1-tetrahydrofolate synthase, mitochondrial	95427.96875	96212.57	104928.1	103890.8	**100114.8438**	94989.27	101671	100381.3	98587.92	**98907.375**	**1.0122**	**0.6901**
P48036	Anxa5	Annexin A5	2059642.375	2130523	2012434	1850416	**2013253.813**	1961064	1968495	1894907	2131515	**1988995.219**	**1.0122**	**0.7660**
Q810J8	Zfyve1	Zinc finger FYVE domain-containing protein 1	13289.55664	14747.93	13879.91	17311.89	**14807.32007**	16462.97	14199.34	14365.16	13488.19	**14628.91602**	**1.0122**	**0.8758**
Q9CR86	Carhsp1	Calcium-regulated heat stable protein 1	110258.8359	107592.1	96828.94	94593.63	**102318.3652**	100412.4	97727.42	98175.27	108071.6	**101096.6719**	**1.0121**	**0.7978**
P14685	Psmd3	26S proteasome non-ATPase regulatory subunit 3	83068.875	83373.61	78221.7	83770.6	**82108.69531**	83123.6	81707.86	78229.65	81461.5	**81130.65234**	**1.0121**	**0.5781**
Q9Z0U1	Tjp2	Tight junction protein ZO-2	70575.04688	68457.61	87369.02	71909.14	**74577.70508**	75983.02	77967.08	74056.39	66762.3	**73692.19727**	**1.0120**	**0.8643**
Q921I1	Tf	Serotransferrin	263807.125	251115.2	288058.5	245206	**262046.7266**	260891.4	248154.6	252272.3	274503.4	**258955.4492**	**1.0119**	**0.7907**
P11352	Gpx1	Glutathione peroxidase 1	90930.96875	89983.22	90782.31	89861.49	**90389.49805**	95248.82	90395.26	81643.82	90022.64	**89327.63477**	**1.0119**	**0.7211**
Q6WVG3	Kctd12	BTB/POZ domain-containing protein KCTD12	40902.625	42527.57	39447.93	42018.15	**41224.07129**	40101.51	40888.38	41517.06	40460.41	**40741.84082**	**1.0118**	**0.5425**
P61264	Stx1b	Syntaxin-1B	1001626.875	949598.2	998977.9	1058450	**1002163.234**	1007008	1027467	962444.7	964934.2	**990463.3281**	**1.0118**	**0.6845**
Q8BVU5	Nudt9	ADP-ribose pyrophosphatase, mitochondrial	20773.15039	22160.42	23871.15	25973.73	**23194.61279**	19937.17	23597.18	24583.13	23578.23	**22923.92773**	**1.0118**	**0.8644**
Q5SYD0	Myo1d	Unconventional myosin-Id	254968.5	258556.8	257334.1	251830.4	**255672.457**	255511.4	247182.8	255872	252272.1	**252709.5703**	**1.0117**	**0.2805**
Q505F5	Lrrc47	Leucine-rich repeat-containing protein 47	46083.28516	43663.37	48804.57	47058.41	**46402.4082**	47091.36	45030.41	47478.85	43860.86	**45865.37109**	**1.0117**	**0.7092**
P42669	Pura	Transcriptional activator protein Pur-alpha	395222.9688	442264	392454.4	384926.2	**403716.8828**	445910.7	449981.5	350274.5	350013	**399044.9375**	**1.0117**	**0.8855**
Q9JHG2	Rcan2	Calcipressin-2	13552.35449	13038.37	13437.35	12361.06	**13097.2832**	14147.56	12163.08	12678.67	12793.81	**12945.77881**	**1.0117**	**0.7728**
A2AF47	Dock11	Dedicator of cytokinesis protein 11	19939.05078	21709.91	24804.04	25695.21	**23037.05176**	23307.03	23068.49	20192.53	24514.86	**22770.72803**	**1.0117**	**0.8751**
Q9QYI5	Dnajb2	DnaJ homolog subfamily B member 2	32055.40039	32347.28	26172.29	29305.18	**29970.03711**	30115.76	30728.9	28383.9	29265.98	**29623.63525**	**1.0117**	**0.8281**
Q8R4E6	Purg	Purine-rich element-binding protein gamma	36545.52344	37789.41	36496.82	30754.38	**35396.53271**	40026.27	35380.86	31864.25	32685.44	**34989.20313**	**1.0116**	**0.8720**
P50396	Gdi1	Rab GDP dissociation inhibitor alpha	578623	646412.1	633406.4	656234.6	**628669.0313**	608518.6	597559.3	633104.6	647055.1	**621559.3906**	**1.0114**	**0.7427**
Q80WG5	Lrrc8a	Volume-regulated anion channel subunit LRRC8A	41342.33203	42252.03	44781.88	41945.21	**42580.36328**	42301.64	43637.34	43580.37	38880.06	**42099.85156**	**1.0114**	**0.7340**
Q923T9	Camk2g	Calcium/calmodulin-dependent protein kinase type II subunit gamma	107993.2266	102837.3	97069.31	93190.3	**100272.5332**	106524.2	89414.85	94247.09	106381.7	**99141.96875**	**1.0114**	**0.8416**
Q8BVI4	Qdpr	Dihydropteridine reductase	255287.4375	256382.3	243502.3	245705.2	**250219.3086**	258000.5	249332.7	233782.2	248501.7	**247404.2734**	**1.0114**	**0.6555**
Q9WV92	Epb41l3	Band 4.1-like protein 3	448937.2813	442942.2	503205	448278.9	**460840.8516**	455732.6	448423.3	455486.5	463067.2	**455677.3984**	**1.0113**	**0.7339**
Q91YP2	Nln	Neurolysin, mitochondrial	22305.5293	19688.5	18946.14	21442.48	**20595.66357**	19984.16	20274.09	21209.29	19995.7	**20365.81104**	**1.0113**	**0.7901**
Q60864	Stip1	Stress-induced-phosphoprotein 1	625064.6875	603705.6	585256.3	609535.5	**605890.5156**	622618.2	632191.2	547829.3	593893	**599132.9063**	**1.0113**	**0.7546**
Q8C079	Strip1	Striatin-interacting protein 1	27113.47266	23306.33	21101.61	18131.6	**22413.25293**	22670.19	24180.37	20882.57	20921.19	**22163.5791**	**1.0113**	**0.9071**

Q8BPM0	Daam1	Disheveled-associated activator of morphogenesis 1	30207	31392.6	25498.85	29546.45	**29161.22412**	31274.95	24528.81	30800.4	28742.01	**28836.54248**	**1.0113**	**0.8764**
Q80UJ7	Rab3gap1	Rab3 GTPase-activating protein catalytic subunit	24691.49023	24636.03	25478.51	27611.6	**25604.40625**	25836.74	26982.62	23307.26	25150.74	**25319.34131**	**1.0113**	**0.7928**
Q9DBZ5	Eif3k	Eukaryotic translation initiation factor 3 subunit K	22828.48828	35186.98	22323.02	31979	**28079.37354**	31666.74	27186.31	29106.79	23113.9	**27768.43555**	**1.0112**	**0.9360**
Q52KR3	Prune2	Protein prune homolog 2	23349.07227	21559.7	25026.44	28424.35	**24589.88867**	25737.61	24788.35	24833.57	21919.59	**24319.78271**	**1.0111**	**0.8775**
O09061	Psmb1	Proteasome subunit beta type-1	112910.9922	118524.3	134812.8	136227.2	**125618.8105**	125310.6	129504.2	124966.9	117266.1	**124261.9531**	**1.0109**	**0.8384**
P29595	Nedd8	NEDD8	34514.94922	41369.36	48932.93	51877.28	**44173.62891**	29285.92	38068.09	45643.33	61790.01	**43696.83789**	**1.0109**	**0.9540**
P62751	Rpl23a	Large ribosomal subunit protein uL23	177750.6094	126275.1	140640.1	172172.8	**154209.6543**	163106.9	152318.2	136375.6	158409	**152552.4336**	**1.0109**	**0.9076**
P19536	Cox5b	Cytochrome c oxidase subunit 5B, mitochondrial	871193.75	907358.3	841089.9	909022.9	**882166.1875**	860604.3	920980.5	786537.9	922828.8	**872737.8594**	**1.0108**	**0.8023**
Q01768	Nme2	Nucleoside diphosphate kinase B	875073.625	863220.6	964232.4	909570.6	**903024.2969**	894539.3	851142.8	835315.3	992519	**893379.0938**	**1.0108**	**0.8259**
Q61768	Kif5b	Kinesin-1 heavy chain	78879.04688	74080.89	77189.8	66947.85	**74274.39648**	75964.14	75756.95	66980.43	75232.49	**73483.50195**	**1.0108**	**0.8246**
P23819	Gria2	Glutamate receptor 2	21192.1543	23659.34	20717.25	25111.03	**22669.94189**	22191.62	23159.87	23083.74	21280.57	**22428.94971**	**1.0107**	**0.8379**
P70335	Rock1	Rho-associated protein kinase 1	10253.9541	16292.71	12138.42	7685.161	**11592.56201**	10238.87	6629.993	17211.09	11799.55	**11469.87585**	**1.0107**	**0.9671**
P62484	Abi2	Abl interactor 2	49911.09766	56414.07	45895.03	48582.44	**50200.66016**	54130.62	49956.64	47725.34	46867.79	**49670.09863**	**1.0107**	**0.8539**
Q02013	Aqp1	Aquaporin-1		33546.36	34005.74	5110.266	**24220.78923**	23858.89	28568.5	17514.59	25917.78	**23964.94189**	**1.0107**	**0.9771**
Q6ZWZ2	Ube2r2	Ubiquitin-conjugating enzyme E2 R2	13602.90723	13197.38	12255	17393	**14112.07373**	16336.37	14745.28	12942.56	11827.85	**13963.01489**	**1.0107**	**0.9243**
Q9D428	GOLGA7B	Golgin subfamily A member 7B	28775.9707	31410.19	31945.36	28228.14	**30089.91553**	32609.12	28972.53	30377.66	27153.94	**29778.31348**	**1.0105**	**0.8402**
G3X8U3	QNG1	Queuosine 5'-phosphate N-glycosylase/hydrolase	39375.33203	37111.41	43405.59	43558.43	**40862.69238**	38609.22	40102.6	38863.25	44188.49	**40440.89063**	**1.0104**	**0.8432**
Q9EPW0	Inpp4a	Inositol polyphosphate-4-phosphatase type I A	18232.9043	17018.06	16947.47	18201.58	**17600.00586**	17534.59	16729.75	18019.16	17392.93	**17419.10596**	**1.0104**	**0.6985**
P84091	Ap2m1	AP-2 complex subunit mu	478422.7188	473672	481487.1	519639.9	**488305.4063**	493215.7	496521	458068	485546.9	**483337.9141**	**1.0103**	**0.7295**
Q9D1E6	Tbcb	Tubulin-folding cofactor B	98938.74219	107100.1	89099.71	93802.92	**97235.36523**	100297.1	104862.5	84565.8	95295.75	**96255.29688**	**1.0102**	**0.8718**
P31750	Akt1	RAC-alpha serine/threonine-protein kinase	19898.20508	17547.22	20785.93	17796.36	**19006.9292**	17666.87	18788.25	18438.54	20371.7	**18816.33838**	**1.0101**	**0.8517**
Q8BU30	Iars1	Isoleucine--tRNA ligase, cytoplasmic	67924.77344	68245.66	60760.23	65364.72	**65573.8457**	72132.26	67767.95	54370.45	65399.87	**64917.62891**	**1.0101**	**0.8798**
P63318	Prkcg	Protein kinase C gamma type	16626.95117	12643.79	14998.99	17932.54	**15550.5686**	17344.78	19532.23	11099.28	13607.47	**15395.94067**	**1.0100**	**0.9463**
Q812E0	Cpeb2	Cytoplasmic polyadenylation element-binding protein 2	15012.39355	12897.44	18207.07	14028.96	**15036.46729**	14701.85	13203.12	16176.42	15466.68	**14887.01807**	**1.0100**	**0.9127**
Q64310	Surf4	Surfeit locus protein 4	13020.81348	14403.56	16931.34	15540	**14973.927**	13789.64	17778.57	15214.37	12518.35	**14825.23169**	**1.0100**	**0.9189**
Q9WTU3	Scn8a	Sodium channel protein type 8 subunit alpha	52283.40234	49879.51	25174.44	22362.39	**37424.93408**	43093.76	42876.18	22772.54	39478.76	**37055.31055**	**1.0100**	**0.9695**
Q8VCT3	Rnpep	Aminopeptidase B	65483.58203	66301.3	59727.1	62582.94	**63523.73047**	67000.18	67729.34	55163.16	61699.21	**62897.97461**	**1.0099**	**0.8545**
Q9DCW4	Etfb	Electron transfer flavoprotein subunit beta	589245.625	578182.3	593638.7	618006	**594768.1406**	626933.8	604050.1	554303.4	570490.6	**588944.4688**	**1.0099**	**0.7622**
S4R2P9	Slc8a3	Sodium/calcium exchanger 3	15583.96191	15092.14	15501.88	18669.65	**16211.90869**	17364.29	15439.73	15602.11	15811.93	**16054.51514**	**1.0098**	**0.8722**
Q9D6F9	Tubb4a	Tubulin beta-4A chain	1028696.375	1063084	1040188	1078941	**1052727.297**	1095805	1058543	1078449	937371.7	**1042542.172**	**1.0098**	**0.7956**
Q9CXW2	Mrps22	Small ribosomal subunit protein mS22	20283.96484	17278.56	15347.3	18345.15	**17813.74512**	20262.89	16872.33	15856.85	17577.64	**17642.42725**	**1.0097**	**0.9064**
Q9WUA3	Pfkp	ATP-dependent 6-phosphofructokinase, platelet type	110329.125	110106.3	115618.7	117081.2	**113283.8359**	106453.8	102941	121994.4	117404.1	**112198.3301**	**1.0097**	**0.8298**
B9EKR1	Ptprz1	Receptor-type tyrosine-protein phosphatase zeta	125131.3203	120947.3	120862.1	115999.3	**120735.0137**	124725.5	123316.7	118072	112213.8	**119582.0117**	**1.0096**	**0.7461**
P98200	Atp8a2	Phospholipid-transporting ATPase IB	22194.04688	21826.99	18825	21702.4	**21137.1084**	23964.23	21693.82	19934.32	18149.97	**20935.58545**	**1.0096**	**0.8951**
Q9QZ08	Nagk	N-acetyl-D-glucosamine kinase	30355.06641	30739.83	36229.47	29886.06	**31802.60693**	30259.07	31543.54	33342.44	30855.62	**31500.16748**	**1.0096**	**0.8588**
Q62433	Ndrg1	Protein NDRG1	66681.52344	73264.3	86362.6	62346.97	**72163.84961**	71141.77	68102.08	71267.42	75413.05	**71481.07813**	**1.0096**	**0.9044**
Q9WUA2	Farsb	Phenylalanine--tRNA ligase beta subunit	96356.8125	96721.81	90342.11	89342.14	**93190.71875**	94933.92	95714.49	92217.41	86375.08	**92310.22461**	**1.0095**	**0.7697**
Q8BJZ4	Mrps35	Small ribosomal subunit protein mS35	12246.72363	12974.95	12218.27	10085.12	**11881.26709**	11964.74	9236.444	11248.86	14626.13	**11769.04443**	**1.0095**	**0.9328**
P21447	Abcb1a	ATP-dependent translocase ABCB1	21365.33984	22111.85	31121.06	25476.32	**25018.64209**	24687.29	26079.52	23235.16	25128.99	**24782.73975**	**1.0095**	**0.9216**
O09167	Rpl21	Large ribosomal subunit protein eL21	57322.54688	64030.11	54292.99	53171.79	**57204.36035**	64572.11	62805.1	45733.27	53551.03	**56665.37598**	**1.0095**	**0.9178**
Q9R0Q7	Ptges3	Prostaglandin E synthase 3	288279.75	288679.9	265991	235313.9	**269566.125**	266761.4	286754.3	222548.8	292098.3	**267040.6914**	**1.0095**	**0.9046**
Q64105	Spr	Sepiapterin reductase	208873.5313	212193.3	216722.8	214807.3	**213149.2227**	200361.9	207777.3	215867.1	220605.7	**211152.9727**	**1.0095**	**0.6907**
P35980	Rpl18	Large ribosomal subunit protein eL18	230740.6719	204503.9	203339	227071.7	**216413.8242**	222594	238535.7	179925	216526.9	**214395.3633**	**1.0094**	**0.8928**
Q9CR27	Washc3	WASH complex subunit 3	35986.60938	33599.94	33822.59	34890.28	**34574.85449**	33772.71	34092.88	38222.49	30926.29	**34253.5918**	**1.0094**	**0.8474**
O70480	Vamp4	Vesicle-associated membrane protein 4	4252.573242	5199.067	6356.049	9764.22	**6392.977295**	4810.321	6764.546	7904.872	5855.042	**6333.695313**	**1.0094**	**0.9669**
Q8C7B6	Fbxl22	F-box and leucine-rich protein 22	34322.41406	22197.17	23144.72	29286.52	**27237.70654**	24257.91	26061.78		30638.91	**26986.19727**	**1.0093**	**0.9486**
P09528	Fth1	Ferritin heavy chain	188773.375	204929.7	257191.3	236565.5	**221864.9688**	210562.1	207164.4	213351.5	248205	**219820.7188**	**1.0093**	**0.9138**
Q9D8S9	Bola1	BolA-like protein 1	26818.60547	24023.12	23885.8	17856.2	**23145.93164**	23286.57	24210.38	19857.62	24379.68	**22933.56104**	**1.0093**	**0.9250**
Q61102	Abcb7	Iron-sulfur clusters transporter ABCB7, mitochondrial	86975.86719	85536.82	87700.73	104557.4	**91192.70313**	88027.61	86045.17	99375.52	87977.09	**90356.34766**	**1.0093**	**0.8823**
P49443	Ppm1a	Protein phosphatase 1A	86911.07031	93147.72	96334.72	84209.42	**90150.73242**	92886.89	86676.6	91781.62	86005.3	**89337.60156**	**1.0091**	**0.8129**
Q3UE37	Ube2z	Ubiquitin-conjugating enzyme E2 Z	38428.23828	42000.82	34544.98	39137.95	**38527.99609**	35789.13	41942.07	39867.28	35126.31	**38181.19922**	**1.0091**	**0.8822**
Q9JIS8	Slc12a4	Solute carrier family 12 member 4	58420.20313	54687.73	28531.63	56507.35	**49536.72656**	22124.6	56067.15	66070.55	52104.66	**49091.74023**	**1.0091**	**0.9711**
P62835	Rap1a	Ras-related protein Rap-1A	560451.0625	580656.3	569985.4	532344.8	**560859.375**	553134.1	559740.3	544761.4	565690.9	**555831.6719**	**1.0090**	**0.6718**
Q8K4Z3	Naxe	NAD(P)H-hydrate epimerase	359873.625	382522.1	390216.5	382811.4	**378855.9141**	340371.5	371843.8	382818.5	406814.8	**375462.125**	**1.0090**	**0.8315**
Q9CWZ7	Napg	Gamma-soluble NSF attachment protein	358138.5625	356977.1	331947.8	356407.3	**350867.6875**	359664.8	364122.6	324512.3	342758.3	**347764.4922**	**1.0089**	**0.7875**
P17742	Ppia	Peptidyl-prolyl cis-trans isomerase A	2854205.5	3056985	3111745	3289197	**3078033.063**	2953600	2960307	3096754	3193024	**3050921**	**1.0089**	**0.8076**
Q8C163	Exog	Nuclease EXOG, mitochondrial	86167.14844	77915.5	75212.5	78170.24	**79366.34766**	85054.23	76500.07	72963.64	80174.06	**78673.00195**	**1.0088**	**0.8497**
Q99J45	Nrbp1	Nuclear receptor-binding protein	21593.12891	23557.58	24482.88	24885.47	**23629.76367**	20858.26	22614.4	25348.85	24899.93	**23430.36182**	**1.0085**	**0.8811**
Q6PH08	Erc2	ERC protein 2	31640.07617	24984.67	28726.74	25686.46	**27759.48535**	27642.95	34414.63	26877.47	21170.52	**27526.39111**	**1.0085**	**0.9427**
F8VPU2	Farp1	FERM, ARHGEF and pleckstrin domain-containing protein 1	42183.95703	44516.13	36386.82	37778.76	**40216.41797**	41263.66	40602.43	40629.23	37020.34	**39878.91113**	**1.0085**	**0.8790**
Q80WC7	Agfg2	Arf-GAP domain and FG repeat-containing protein 2	22002.16992	23682.16	19262.58	16604.96	**20387.96631**	22452.42	18059.49	20185.64	20170.97	**20217.13037**	**1.0085**	**0.9273**
Q93092	Taldo1	Transaldolase	226591.2031	228757.1	221480.3	213730.8	**222639.8398**	228221.5	225336.5	203831.4	225710.6	**220775**	**1.0084**	**0.7868**
Q91VI7	Rnh1	Ribonuclease inhibitor	55361.625	67884.88	57386.45	59968.19	**60150.28516**	61175.65	58891.47	60904.46	57628.07	**59649.91309**	**1.0084**	**0.8674**
Q922E4	Pcyt2	Ethanolamine-phosphate cytidylyltransferase	73347.29688	72370.29	65607.15	71771.67	**70774.10156**	74751.75	75707.95	66137.13	64150.52	**70186.83789**	**1.0084**	**0.8696**
O55143	Atp2a2	Sarcoplasmic/endoplasmic reticulum calcium ATPase 2	747983.375	721458.6	737620.4	778722.6	**746446.2656**	737631.1	712952.2	781906.6	728596.1	**740271.4844**	**1.0083**	**0.7572**

O88685	Psmc3	26S proteasome regulatory subunit 6A	116683.0313	119190.2	112966.1	119649.7	**117122.25**	122145.9	117635.3	115568.1	109296.9	**116161.5723**	**1.0083**	**0.7654**
P41105	Rpl28	Large ribosomal subunit protein eL28	91641.66406	100605.3	110446.2	85845.94	**97134.78125**	96582.22	119797.4	85448.82	83525.8	**96338.54883**	**1.0083**	**0.9386**
Q9WUM4	Coro1c	Coronin-1C	108897.9063	111080	114619.9	116898.6	**112874.0918**	119270.7	114133.5	103483	110914.7	**111950.4688**	**1.0083**	**0.8140**
Q8BXR9	Osbpl6	Oxysterol-binding protein-related protein 6	27626.96875	26692.35	28698.26	27331.26	**27587.20801**	27784.19	25654.64	28130.77	27881.22	**27362.70605**	**1.0082**	**0.7627**
Q9DBE8	Alg2	Alpha-1,3/1,6-mannosyltransferase ALG2	130511.0781	124751.8	113699.5	100937.4	**117474.9453**	135599.3	120731.1	103604.6	106146.4	**116520.3652**	**1.0082**	**0.9260**
P62137	Ppp1ca	Serine/threonine-protein phosphatase PP1-alpha catalytic subunit	76157.51563	80183.07	76745.06	67395.03	**75120.16992**	73561.32	82003.47	63481.8	78997.06	**74510.91309**	**1.0082**	**0.9051**
Q62443	Nptx1	Neuronal pentraxin-1	21944.35742	20898.87	18911.5	21046.66	**20700.34766**	21301.05	20807.69	21032.74	18988.99	**20532.61816**	**1.0082**	**0.8460**
Q8K212	Pacs1	Phosphofurin acidic cluster sorting protein 1	267880.0313	234525.7	255926.9	260951.1	**254820.9219**	251915.5	258646	263632.7	236832.1	**252756.5703**	**1.0082**	**0.8309**
P63028	Tpt1	Translationally-controlled tumor protein	51365.09766	48560.91	46106.41	44861.58	**47723.5**	49204.67	44944.06	42798.08	52414	**47340.20313**	**1.0081**	**0.8871**
Q0VBF8	Stum	Protein stum homolog	11060.69727	11078.35	11179.98	8999.918	**10579.73486**	11383.34	9402.577	8668.921	12526.91	**10495.43799**	**1.0080**	**0.9376**
Q8R2R9	Ap3m2	AP-3 complex subunit mu-2	64729.03125	64098.64	58205.08	69134.98	**64041.93262**	65722.01	67187.19	60980.66	60286.76	**63544.15332**	**1.0078**	**0.8659**
P09405	Ncl	Nucleolin	242051.75	227412.9	238430.3	225717.3	**233403.0859**	246837.6	240009.7	222405.2	217187.1	**231609.8789**	**1.0077**	**0.8324**
O08746	Matn2	Matrilin-2	8844.818359		8690.878	5656.499	**7730.731771**	4786.461			10556.43	**7671.446289**	**1.0077**	**0.9828**
Q9CZJ2	Hspa12b	Heat shock 70 kDa protein 12B	420233.2813	329982.3	489725.4	364966.4	**401226.8594**	412111.2	377888.1	387424.4	415223.5	**398161.7969**	**1.0077**	**0.9350**
Q3UYV9	Ncbp1	Nuclear cap-binding protein subunit 1	5924.75293		5853.995	3698.217	**5158.988444**	5316.317	3873.444	4922.437	6366.482	**5119.669922**	**1.0077**	**0.9654**
Q14B80	Kcnc2	Potassium voltage-gated channel subfamily C member 2	4858.391113	4328.38	5363.382	5343.112	**4973.316284**	4811.285	5304.747	4900.119	4725.701	**4935.462646**	**1.0077**	**0.8955**
Q8BH44	Coro2b	Coronin-2B	30779.30859	33424.76	33794.32	37329.49	**33831.96973**	34395.38	35073.69	31748.52	33089.17	**33576.6875**	**1.0076**	**0.8732**
Q99PJ0	Ntm	Neurotrimin	126276.3438	117047.4	109949.8	130817.3	**121022.707**	123267.8	125929.6	115983.2	115272	**120113.1738**	**1.0076**	**0.8711**
Q8BK08	Tmem11	Transmembrane protein 11, mitochondrial	86676.25781	81002.32	85855.83	72447.07	**81495.36914**	81974.25	87728.45	79816.02	74015.45	**80883.54297**	**1.0076**	**0.8921**
P62259	Ywhae	14-3-3 protein epsilon	807066.5	837579.2	863275.3	921430.2	**857337.7969**	846209.4	875870.6	807260.4	874378.6	**850929.75**	**1.0075**	**0.8330**
P29416	Hexa	Beta-hexosaminidase subunit alpha	27199.83984	27406.25	26842.08	19154.88	**25150.76172**	28142.21	20634.47	28089.27	22987.03	**24963.24805**	**1.0075**	**0.9478**
O35250	Exoc7	Exocyst complex component 7	31745.16992	28560.22	32114.64	29097.01	**30379.25781**	30249.92	31423.85	29756.63	29192.22	**30155.65674**	**1.0074**	**0.8341**
Q9QYK9	Pnck	Calcium/calmodulin-dependent protein kinase type 1B	11655.8877	9618.159	7632.087	10728.36	**9908.623535**	8637.961	10848.08	10488.08	9369.878	**9836.000488**	**1.0074**	**0.9447**
Q99LR1	Abhd12	Lysophosphatidylserine lipase ABHD12	110615.1641	108672.7	108983.6	113495.1	**110441.6211**	109996.3	107339.8	115728.2	105523.5	**109646.9512**	**1.0072**	**0.7599**
O55029	Copb2	Coatomer subunit beta'	86118.03125	80216.41	83605.26	76479.04	**81604.68359**	81300.57	85985	79922.12	76889.66	**81024.33789**	**1.0072**	**0.8439**
Q2TPA8	Hsdl2	Hydroxysteroid dehydrogenase-like protein 2	59157.14844	55243.25	57496.93	55633.23	**56882.64258**	59574.52	62500.3	53604.2	50239.65	**56479.66797**	**1.0071**	**0.8950**
Q9WU78	Pdcd6ip	Programmed cell death 6-interacting protein	118265.3516	133776.3	134700	133764.5	**130126.5293**	125851.1	129746.9	125441.8	135828.9	**129217.1719**	**1.0070**	**0.8509**
P62317	Snrpd2	Small nuclear ribonucleoprotein Sm D2	62969.95313	83512.23	66818.3	80362.37	**73415.71289**	74716.71	81733.48	71248.97	63931.95	**72907.7793**	**1.0070**	**0.9378**
Q8JZU2	Slc25a1	Tricarboxylate transport protein, mitochondrial	243501.2344	286719	259803.5	262414.1	**263109.4492**	282086.4	263748.3	251036.5	248338	**261302.2734**	**1.0069**	**0.8831**
O08576	Rundc3a	RUN domain-containing protein 3A	16781.69336	18884.44	19212.44	17378.38	**18064.2373**	15125.59	16526.61	21678.2	18430.77	**17940.29126**	**1.0069**	**0.9382**
Q9CZW5	Tomm70	Mitochondrial import receptor subunit TOM70	187743.4531	186869.6	171898.3	195162.1	**185418.375**	193475.3	195908.1	172834.7	174536.4	**184188.6133**	**1.0067**	**0.8800**
Q8K400	Stxbp5	Syntaxin-binding protein 5	77856.75	83141.15	77209.58	81525.7	**79933.29297**	81657.18	77583.73	78496.02	79876.17	**79403.27539**	**1.0067**	**0.7635**
Q9CQW1	Ykt6	Synaptobrevin homolog YKT6	84409.29688	89743.99	86399.82	85277.23	**86457.58398**	87389.29	84144.88	89553.66	82469.12	**85889.23633**	**1.0066**	**0.7832**
Q80YA3	Ddhd1	Phospholipase DDHD1	20107.46094	16439.18	11637.24	13740.92	**15481.19995**	17713.93	13976.01	15596.44	14236.81	**15380.79614**	**1.0065**	**0.9619**
Q9R1V6	Adam22	Disintegrin and metalloproteinase domain-containing protein 22	114012.6484	104868.2	110389.7	127531.6	**114200.5566**	115893.2	111255.2	111718.5	114989	**113463.9629**	**1.0065**	**0.8869**
Q9Z0X1	Aifm1	Apoptosis-inducing factor 1, mitochondrial	157223.0781	155022.5	166985.9	154317.7	**158387.2813**	156906.5	163279.7	154977	154305.1	**157367.0898**	**1.0065**	**0.7850**
Q80V42	Cpm	Carboxypeptidase M	103091.2344	95625.78	93982.95	96543.02	**97310.74609**	92304.95	102234.3	92646.91	99577.8	**96690.98828**	**1.0064**	**0.8526**
Q78XF5	Ostc	Oligosaccharyltransferase complex subunit OSTC	33917.64844	38292.68	38259.65	45856.27	**39081.56445**	38567.98	35709.74	40796.16	40272.73	**38836.65137**	**1.0063**	**0.9315**
O70310	Nmt1	Glycylpeptide N-tetradecanoyltransferase 1	71789.14844	75226.16	66123.74	83862.78	**74250.45703**	67161.17	85678.88	75837.68	66471.69	**73787.35547**	**1.0063**	**0.9393**
P97490	Adcy8	Adenylate cyclase type 8	32568.72852	27944.54	28166.37	26248.96	**28732.14795**	29520.65	32477.1	26886.92	25337.31	**28555.49414**	**1.0062**	**0.9347**
Q3U1V6	Uevld	Ubiquitin-conjugating enzyme E2 variant 3	5309.305664	6037.624	6056.096	5545.821	**5737.211426**	5365.079	5732.796	7063.368	4646.645	**5701.972046**	**1.0062**	**0.9500**
Q99KE1	Me2	NAD-dependent malic enzyme, mitochondrial	66009.9375	64725.45	63975.77	66731.15	**65360.5752**	66863.83	64474.23	67405.12	61105.03	**64962.05176**	**1.0061**	**0.8073**
P36916	Gnl1	Guanine nucleotide-binding protein-like 1	70823.28906	67227.99	67914.46	70926.17	**69222.97852**	64858.14	69116.06	72009.48	69225.97	**68802.41504**	**1.0061**	**0.8193**
Q99K85	Psat1	Phosphoserine aminotransferase	481067.25	489557.2	430061.6	438431	**459779.2578**	475079.7	466395.9	426133.1	460401.5	**457002.5391**	**1.0061**	**0.8849**
Q9DB73	Cyb5r1	NADH-cytochrome b5 reductase 1	68056.8125	68501.46	76893.38	71806.38	**71314.50977**	72729.77	74520.29	69613.47	66694.71	**70889.56055**	**1.0060**	**0.8789**
Q68FL6	Mars1	Methionine--tRNA ligase, cytoplasmic	45054	49896.62	45717.51	46656.11	**46831.05859**	47644.82	51220.13	43845.68	43505.31	**46553.98633**	**1.0060**	**0.8998**
Q9ESM3	Hapln2	Hyaluro and proteoglycan link protein 2	324217.375	326244.9	391360.9	411239.6	**363265.7031**	314360.3	325746.7	390544.5	413824.4	**361118.9766**	**1.0059**	**0.9502**
Q3TLI0	Trappc10	Trafficking protein particle complex subunit 10	13205.77539	11171.77	13764.92	16483.51	**13656.49512**	11234.71	13170.64	15249.17	14651.7	**13576.55591**	**1.0059**	**0.9567**
Q8K003	Tma7	Translation machinery-associated protein 7	1263.312256	3666.727	15957.56	9499.721	**7596.829773**		2247.573	5599.958	14810.14	**7552.555908**	**1.0059**	**0.9933**
P03930	Mtatp8	ATP synthase protein 8	103067.8438	101713.7	129070.2	98755.59	**108151.8438**	109572.7	101285	100033.2	119224.5	**107528.8535**	**1.0058**	**0.9427**
Q8R081	Hnrnpl	Heterogeneous nuclear ribonucleoprotein L	126818.3047	119594.4	115740.8	122277.1	**121107.6719**	127645.5	129774.3	114606.7	109668.7	**120423.7871**	**1.0057**	**0.9039**
Q9D8B4	Ndufa11	NADH dehydrogenase [ubiquinone] 1 alpha subcomplex subunit 11	139216.0781	120341.3	130174.7	147401.6	**134283.4258**	117920.4	131148.3	148304.4	136780.3	**133538.3359**	**1.0056**	**0.9337**
Q8VEK3	Hnrnpu	Heterogeneous nuclear ribonucleoprotein U	258084.1406	275423.8	250903	260202.2	**261153.293**	261896.3	298958.4	235636	242428.4	**259729.7578**	**1.0055**	**0.9280**
P21126	Ubl4a	Ubiquitin-like protein 4A	24372.57031	19747.96	21798.54	23874.81	**22448.46777**	22824.57	22112.61	19346.92	25020.57	**22326.16797**	**1.0055**	**0.9407**
Q61081	Cdc37	Hsp90 co-chaperone Cdc37	102993.9609	99558.92	98757.02	92318.24	**98407.03516**	103332.4	104079.8	90944.04	93147.43	**97875.91797**	**1.0054**	**0.9003**
Q9EQI8	Mrpl46	Large ribosomal subunit protein mL46	19407.60352	16280.84	17732.52	25636.71	**19764.42065**	17267.91	13923.88	28175.28	19264.69	**19657.94092**	**1.0054**	**0.9778**
Q9JLJ0	Litaf	Lipopolysaccharide-induced tumor necrosis factor-alpha factor homolog	18301.67773	46714.54	43583.64	51398.95	**39999.70264**	49112.98	52431.65	28415.88	29179.44	**39784.98926**	**1.0054**	**0.9832**
Q791V5	Mtch2	Mitochondrial carrier homolog 2	186158.1719	186373.3	184774.5	184808.4	**185528.5938**	190485.9	190844.5	174693.4	182133.9	**184539.4141**	**1.0054**	**0.8070**
B2RUR8	Otud7b	OTU domain-containing protein 7B	10930.77148	10892.27	11983.8	11603.63	**11352.61792**	11169.92	11936.21	10655.34	11411.58	**11293.2627**	**1.0053**	**0.8799**
Q8K2C6	Sirt5	NAD-dependent protein deacylase sirtuin-5, mitochondrial	22742.4707	22513.63	21353.76	21615.88	**22056.43555**	22310.86	22727.12	20810.08	21916.66	**21941.17969**	**1.0053**	**0.8358**
Q76MZ3	Ppp2r1a	Serine/threonine-protein phosphatase 2A 65 kDa regulatory subunit A alpha	644547.3125	610597.8	591201.1	614979.3	**615331.3594**	639015.2	611216.6	590923.5	607372.1	**612131.8281**	**1.0052**	**0.8368**
Q8R1G2	Cmbl	Carboxymethylenebutenolidase homolog	24694.4082	23666.78	29433.32	28868.93	**26665.8584**	28933.65	25300.66	26863.56	25011.99	**26527.46533**	**1.0052**	**0.9381**
Q3URE1	Acsf3	Malonate--CoA ligase ACSF3, mitochondrial	16895.5957	17726.32	20864.94	21308.88	**19198.93555**	19748.4	18817.63	19166.42	18666.99	**19099.86035**	**1.0052**	**0.9331**
P31230	Aimp1	Aminoacyl tRNA synthase complex-interacting multifunctional protein 1	35887.74219	40441.1	39642.69	34440.7	**37603.05664**	40066.02	39840.09	38293.39	31444	**37410.87451**	**1.0051**	**0.9410**
Q7TSE6	Stk38l	Serine/threonine-protein kinase 38-like	21231.4668	20640.42	21813.56	16338.8	**20006.06226**	16693.61	20351.96	21595.94	20983.13	**19906.15918**	**1.0050**	**0.9540**

Q8CDA1	Inpp5f	Phosphatidylinositide phosphatase SAC2	13104.19336	14343.09	13294.49	13183.63	**13481.35278**	11616.82	12287.48	15026.97	14725.1	**13414.09229**	1.0050	0.9432
P17751	Tpi1	Triosephosphate isomerase	941968.6875	962205.6	913983.4	1030530	**962171.9219**	869845.3	950946.2	983486.9	1025234	**957378.1094**	1.0050	0.9112
Q8K0S0	Phyhip	Phytanoyl-CoA hydroxylase-interacting protein	103839.2422	103419.7	93637.87	96812.12	**99427.22266**	101325.5	108621.6	91934.55	93862.16	**98935.94141**	1.0050	0.9178
Q8CGC7	Eprs1	Bifunctional glutamate/proline--tRNA ligase	55275.38281	53115.77	50676.11	49337.66	**52101.23242**	52302.63	55937.32	47121.78	52029.4	**51847.78223**	1.0049	0.9135
O35969	Gamt	Guanidinoacetate N-methyltransferase	14189.50586	12181.78	11173.5	12401.47	**12486.56396**	14073.73	12133.31	11907.86	11590.26	**12426.28979**	1.0049	0.9452
O70439	Stx7	Syntaxin-7	97839.85938	81844.43	96430.54	101360.4	**94368.79883**	94786.41	92153.27	93715.9	95038.82	**93923.60156**	1.0047	0.9218
Q8VDQ8	Sirt2	NAD-dependent protein deacetylase sirtuin-2	1982208.125	2275276	1947652	1972270	**2044351.656**	2111112	2087108	1948342	1992519	**2034770.375**	1.0047	0.9153
Q3TIR3	Ric8a	Synembryn-A	16367.10645	19318.35	19132.38	17853.74	**18167.89282**	16746.38	19396.35	16748.54	19442.22	**18083.37207**	1.0047	0.9373
P24288	Bcat1	Branched-chain-amino-acid aminotransferase, cytosolic	131661.9375	126510	130089	133288.7	**130387.4004**	127377.2	127204.2	123920.3	140635	**129784.1914**	1.0046	0.8844
Q9D7E4		UPF0449 protein C19orf25 homolog	10467.0332	13134.94	14270.21	12672.93	**12636.27734**	12758.7	11835.3	10962.68	14755.5	**12578.04492**	1.0046	0.9609
Q3UUG6	Tbc1d24	TBC1 domain family member 24	20277.81836	22645.21	22138.1	20660.75	**21430.46826**	20039.96	21698.65	23129.23	20463.85	**21332.92188**	1.0046	0.9171
Q80SW1	Ahcyl1	S-adenosylhomocysteine hydrolase-like protein 1	824979.5625	869356.2	777030.8	825933.9	**824325.1094**	859154.1	870229.8	761215.6	791862.3	**820615.4531**	1.0045	0.9125
Q8BGX2	Timm29	Mitochondrial import inner membrane translocase subunit Tim29	23939.80664	27017.18	15745.6	28095.07	**23699.41382**	29800.98	26362.45	21007.84	17200.8	**23593.01904**	1.0045	0.9794
Q91V41	Rab14	Ras-related protein Rab-14	197570.3594	197048.5	198041.9	189725.2	**195596.5156**	187464.8	195534.4	199463	196421.9	**194721.0117**	1.0045	0.7954
P04370	Mbp	Myelin basic protein	41710772	44921416	44936044	43229004	**43699309**	43609064	42017656	42098236	46293664	**43504655**	1.0045	0.8827
Q91WC3	Acsl6	Long-chain-fatty-acid--CoA ligase 6	287581.625	284354.1	274389.8	300577.7	**286725.7813**	293070.8	294332.6	283288.5	271126.4	**285454.5703**	1.0045	0.8730
Q9JHU4	Dync1h1	Cytoplasmic dynein 1 heavy chain 1	670222.75	660725.2	645777.9	629894.5	**651655.0938**	669718.5	655814.5	618850.6	650759.1	**648785.6719**	1.0044	0.8434
Q9Z0H8	Clip2	CAP-Gly domain-containing linker protein 2	72153.36719	69898.57	72234.68	62727.84	**69253.61426**	71314.05	69459.95	61395.41	73629.59	**68949.75**	1.0044	0.9332
Q99KQ4	Nampt	Nicotinamide phosphoribosyltransferase	101367.9922	105941.6	92389.88	91103.13	**97700.64844**	106360.4	98683.82	96225.54	87821.92	**97272.92969**	1.0044	0.9375
Q9EPL9	Acox3	Peroxisomal acyl-coenzyme A oxidase 3	14982.15723	16350.29	16714.88	14683.96	**15682.82349**	15140.31	15523.08	14680.5	17113.33	**15614.30444**	1.0044	0.9280
Q8VBT9	Aspscr1	Tether containing UBX domain for GLUT4	23406.08398	23481.33	20350.34	19502.82	**21685.14551**	21856.39	24529.27	17849.54	22126.63	**21590.45703**	1.0044	0.9580
A2RT62	Fbxl16	F-box/LRR-repeat protein 16	13463.45605	11536.02	10738.39	13992.55	**12432.60376**	13770.7	9577.494	12507.14	13659.83	**12378.79224**	1.0043	0.9669
P53668	Limk1	LIM domain kinase 1	31141.8457	32044.28	28216.53	27527.94	**29732.64746**	27908.92	28217.03	31255.61	31041.71	**29605.81787**	1.0043	0.9316
P29341	Pabpc1	Polyadenylate-binding protein 1	382243.75	368501.7	365717.6	360722.1	**369296.2813**	380735.1	391504.2	347471.2	351178.3	**367722.2031**	1.0043	0.8983
Q9CPW4	Arpc5	Actin-related protein 2/3 complex subunit 5	68461.71875	69245.5	61969.2	60297.21	**64993.40723**	66488.84	60230.93	55903.91	76267.63	**64722.82617**	1.0042	0.9583
Q9ERR1	Ndel1	Nuclear distribution protein nudE-like 1	24963.59961	19967.27	22213.89	20989.78	**22033.63379**	22223.81	23412.15	19036.33	23112.46	**21946.18994**	1.0040	0.9546
Q11011	Npepps	Puromycin-sensitive aminopeptidase	311807.9375	322399.6	292132.3	293821.5	**305040.3203**	303087.6	310464.4	287383.3	314385.2	**303830.1328**	1.0040	0.9020
Q91WL8	Wwox	WW domain-containing oxidoreductase	11305.74902	6925.942	6089.168	8830.007	**8287.716675**	8353.184	11686.67	8561.9	4418.265	**8255.004028**	1.0040	0.9867
Q6NSQ9	G6pc3	Glucose-6-phosphatase 3	19958.38086	18255.84	17720.45	22045.64	**19495.07617**	24429.89	19354.66	17341.24	16553.62	**19419.85449**	1.0039	0.9715
P68181	Prkacb	cAMP-dependent protein kinase catalytic subunit beta	51589.42578	46278.36	49233.74	29651.33	**44188.21289**	48276.25	53272.54	29818.12	44708.3	**44018.79932**	1.0038	0.9817
Q8BGH2	Samm50	Sorting and assembly machinery component 50 homolog	287123.7188	311992.6	286772.7	300845.5	**296683.625**	296081.1	296468.7	298745.5	290899	**295548.5703**	1.0038	0.8627
Q8BR63	Fam177a1	Protein FAM177A1	68654.8125	68376.43	62893.81	63964.23	**65972.32031**	69627.71	67570.15	62933.32	62749.68	**65720.2168**	1.0038	0.9151
P26883	Fkbp1a	Peptidyl-prolyl cis-trans isomerase FKBP1A	400970.3438	404255.7	319404.8	324244.2	**362218.7656**	391954.4	363088.4	358422	329963.9	**360857.1875**	1.0038	0.9608
Q3TLS3	Gdpgp1	GDP-D-glucose phosphorylase 1	16570.33008	16540.45	17786.98	16772.56	**16917.58105**	16225.07	17695.77	17909.52	15587.24	**16854.40161**	1.0037	0.9242
P00493	Hprt1	Hypoxanthine-guanine phosphoribosyltransferase	353476.2813	368976	390390.2	414789.8	**381908.0625**	374992.5	372678.9	385815	388491.3	**380494.4141**	1.0037	0.9222
Q8BW96	Camk1d	Calcium/calmodulin-dependent protein kinase type 1D	79702.83594	76319.39	80803.88	73787.31	**77653.35352**	77489.38	79390.11	75005.82	77579.3	**77366.15039**	1.0037	0.8810
P47964	Rpl36	Large ribosomal subunit protein eL36	155273.4844	154324	150228.7	146670.3	**151624.125**	153249.5	174298.7	137458	139263.9	**151067.5156**	1.0037	0.9513
Q5SSM3	Arhgap44	Rho GTPase-activating protein 44	51683.8125	47068.28	50925.5	48013.98	**49422.89063**	50108.27	51490.34	47683.06	47687.99	**49242.41504**	1.0037	0.9056
P20917	Mag	Myelin-associated glycoprotein	1435203.125	1342355	1333302	1173813	**1321168.188**	1274432	1321386	1305986	1363914	**1316429.406**	1.0036	0.9368
Q9WV80	Snx1	Sorting nexin-1	53524.59766	50409.43	44667.24	47851.98	**49113.3125**	45440.98	51198.36	51168.7	47941.57	**48937.4043**	1.0036	0.9426
Q3TXS7	Psmd1	26S proteasome non-ATPase regulatory subunit 1	96865.46094	94242.58	97923.16	106251.4	**98820.66016**	92489.73	104982.9	100753.7	95650.22	**98469.12695**	1.0036	0.9291
Q3TWN3	Cnnm2	Metal transporter CNNM2	5111.851563	9042.935	8072.999	9071.808	**7824.898193**	9424.346	7287.223	8187.719	6290.394	**7797.42041**	1.0035	0.9817
Q9JKF7	Mrpl39	Large ribosomal subunit protein mL39	16527.47266	16003.08	16307.69	16543.6	**16345.46143**	18642.55	19337.09	13462.66	13713.47	**16288.94116**	1.0035	0.9725
P68372	Tubb4b	Tubulin beta-4B chain	8780513	9830274	9372580	9744680	**9432011.75**	9050085	9266347	10077330	9204761	**9399630.75**	1.0034	0.9254
Q99LY9	Ndufs5	NADH dehydrogenase [ubiquinone] iron-sulfur protein 5	165150.3125	152777.4	171236.6	186826.9	**168997.7852**	158707.3	179283.1	150161.2	185554.7	**168426.543**	1.0034	0.9601
Q9CYW4	Hdhd3	Haloacid dehalogenase-like hydrolase domain-containing protein 3	33967.30078	30442.25	34334.1	33316.13	**33014.94629**	29805.44	27709.87	36426.27	37678.86	**32905.10889**	1.0033	0.9677
P47955	Rplp1	Large ribosomal subunit protein P1	254087.8906	237422	227543.1	227271.7	**236581.1836**	267365.1	262550.2	184916.1	228367	**235799.5898**	1.0033	0.9702
Q8VDQ1	Ptgr2	Prostaglandin reductase 2	43544.64063	48469.27	38259.23	37878.6	**42037.93555**	43544.72	44440.03	40133.53	39483.57	**41900.46387**	1.0033	0.9623
Q8R429	Atp2a1	Sarcoplasmic/endoplasmic reticulum calcium ATPase 1	293383.6875	285877.3	282841.3	301446.5	**290887.2188**	304581.5	289697.1	297115.6	268368.8	**289940.7266**	1.0033	0.9183
Q80W22	Thnsl2	Threonine synthase-like 2	5153.844727	7025.071	3577.561	6480.459	**5559.233826**	4782.946	4570.452	6011.76	6799.671	**5541.207031**	1.0033	0.9852
Q3UDE2	Ttll12	Tubulin--tyrosine ligase-like protein 12	44888.05859	44878.04	42292.63	38788.77	**42711.875**	42303.86	44722.9	40160.74	43109.41	**42574.22754**	1.0032	0.9391
Q920Q4	Vps16	Vacuolar protein sorting-associated protein 16 homolog	29602.32813	30672.43	28924.2	28631.86	**29457.70557**	24126.88	29778.41	31225.1	32321.82	**29363.0542**	1.0032	0.9614
Q8C7V8	Ccdc134	Coiled-coil domain-containing protein 134				3038.129	**3038.129395**	1907.628	2985.905	3340.278	3879.675	**3028.371368**	1.0032	
Q9R0N7	Syt7	Synaptotagmin-7	48122.60156	48864.81	41811.32	46493.43	**46323.04004**	51907.75	48725.96	39805.53	44261.93	**46175.29199**	1.0032	0.9633
Q8BFR5	Tufm	Elongation factor Tu, mitochondrial	877529.25	775799.5	875497.3	904829.9	**858413.9688**	846287.4	927156.1	804440.4	844967.4	**855712.8438**	1.0032	0.9460
P16858	Gapdh	Glyceraldehyde-3-phosphate dehydrogenase	2749802	3227649	3001682	3095641	**3018693.375**	2876244	2834057	3056899	3269864	**3009266.063**	1.0031	0.9491
Q9CYH5	Gfod2	Glucose-fructose oxidoreductase domain-containing protein 2		12658.79	21813.11		**17235.94971**			15055.19	19309.31	**17182.25**	1.0031	0.9925
Q9D051	Pdhb	Pyruvate dehydrogenase E1 component subunit beta, mitochondrial	911225.8125	948195.6	1003851	1036699	**974992.625**	937808.3	950955.7	1024246	975379.2	**972097.2969**	1.0030	0.9347
P63005	Pafah1b1	Platelet-activating factor acetylhydrolase IB subunit beta	71245.42188	74106.88	67888.85	68182.71	**70355.96484**	69096.38	75367.3	65216.55	70922.74	**70150.74609**	1.0029	0.9388
Q7TNC4	Luc7l2	Putative RNA-binding protein Luc7-like 2	13397.3623	12505.99	10702.12	10148.7	**11688.54224**	8995.066	10706.59	14674.63	12246.44	**11655.68042**	1.0028	0.9824
Q8K0G5	Eipr1	EARP and GARP complex-interacting protein 1	19472.92188	26952.45	26538.51	28440.35	**25351.05664**	25736.93	21728.66	28862.24	24795.25	**25280.76855**	1.0028	0.9783
Q6QD59	Bnip1	Vesicle transport protein SEC20	13541.38379	19441.67	11776.46	15390.18	**15037.42554**	17212.87	15072.47	14274.16	13423.89	**14995.84668**	1.0028	0.9826
P63242	Eif5a	Eukaryotic translation initiation factor 5A-1	192287.7344	205007.5	209534.6	202314.6	**202286.1094**	195514.7	202033.8	197935.8	211435.5	**201729.9336**	1.0028	0.9161
Q9D0M3	Cyc1	Cytochrome c1, heme protein, mitochondrial	490882.25	480561.9	504734.6	469731.3	**486477.5078**	464749.3	477730.4	474719.9	523379.4	**485144.75**	1.0027	0.9322
Q9WVA3	Bub3	Mitotic checkpoint protein BUB3	29859.48633	23788.8	23691.28	23261.18	**25150.18457**	22403.69	32919.75	23142.26	21862.23	**25081.98535**	1.0027	0.9829

P62852	Rps25	Small ribosomal subunit protein eS25	287521.4375	288952.3	296241.9	278788.8	**287876.1172**	301763.6	295237	267632.1	283763.1	**287098.9531**	**1.0027**	**0.9284**
P61961	Ufm1	Ubiquitin-fold modifier 1	90654.33594	90738.49	94050.64	95693.36	**92784.20703**	93225.01	97078.67	86447.41	93385.88	**92534.24219**	**1.0027**	**0.9249**
P61161	Actr2	Actin-related protein 2	121583.4922	133011	120436.5	122809.2	**124460.0527**	125821.1	124166.4	122976.9	123535.3	**124124.916**	**1.0027**	**0.9134**
P70372	Elavl1	ELAV-like protein 1	134923.5781	128867.5	127044.5	138604.7	**132360.0508**	138733.1	131113.7	130008	128177.7	**132008.1172**	**1.0027**	**0.9241**
Q9CWK8	Snx2	Sorting nexin-2	61455.85547	58692.18	57104.86	60845.28	**59524.54102**	61273.98	62802.88	56201.31	57189.73	**59366.97559**	**1.0027**	**0.9358**
Q8BMJ2	Lars1	Leucine--tRNA ligase, cytoplasmic	44353.08594	50871.13	52749.47	44329.23	**48075.72754**	46568.77	49175.55	46919.21	49134.58	**47949.52832**	**1.0026**	**0.9580**
Q60692	Psmb6	Proteasome subunit beta type-6	110398.1563	119450	116753.2	118161.8	**116190.7871**	110410.2	111721.4	122080.4	119354.5	**115891.6113**	**1.0026**	**0.9345**
P62830	Rpl23	Large ribosomal subunit protein uL14	129660.7344	139764.7	129041.9	145803.4	**136067.6953**	136302.2	124867.7	135874	145869.3	**135728.3223**	**1.0025**	**0.9561**
Q9CYG7	Tomm34	Mitochondrial import receptor subunit TOM34	34822.36719	31195.1	37395.34	31058.44	**33617.81006**	37396.85	37174.05	29226.14	30340.21	**33534.31543**	**1.0025**	**0.9760**
Q9DB20	Atp5po	ATP synthase subunit O, mitochondrial	2408412	2545451	2308586	2673810	**2484064.75**	2479935	2511462	2732129	2188740	**2478066.313**	**1.0024**	**0.9665**
Q91Z49	Fyttd1	UAP56-interacting factor	14807.94922	11618.92	14059.31	14460.55	**13736.68237**	14645.58	10215.1	14807.68	15147.53	**13703.97217**	**1.0024**	**0.9818**
Q08642	Padi2	Protein-arginine deiminase type-2	277479.3125	315097.1	236463.9	219531.1	**262142.8477**	275646	255668.2	250395.1	264374	**261520.8242**	**1.0024**	**0.9785**
Q9CY34	Ube2f	NEDD8-conjugating enzyme UBE2F	24428.25195	30791.19	17705.85	16127.35	**22263.15967**	22622.98	23271.83	21852.01	21100.02	**22211.70996**	**1.0023**	**0.9884**
P70699	Gaa	Lysosomal alpha-glucosidase	28352.14453	24022.87	24751.27	29911.12	**26759.35156**	29050.88	23971.78	27350.77	26422.58	**26699.00049**	**1.0023**	**0.9739**
Q3TWW8	Srsf6	Serine/arginine-rich splicing factor 6	35134.62891	35669.79	47826.96	51243.16	**42468.63379**	34954.55	41117.18	50201.13	43218.8	**42372.91797**	**1.0023**	**0.9859**
Q8VDJ3	Hdlbp	Vigilin	126200.0781	132128.9	132206.8	129960.9	**130124.1836**	132003.1	137483.7	124572.3	125271	**129832.5254**	**1.0022**	**0.9337**
Q3V1L4	Nt5c2	Cytosolic purine 5'-nucleotidase	32519.27539	33397.01	28188.09	32196.95	**31575.33154**	35061.28	35280.48	26787.42	28890.73	**31504.97705**	**1.0022**	**0.9780**
P50571	Gabrb1	Gamma-aminobutyric acid receptor subunit beta-1	26845.50781	25828.73	23184.25	28160.67	**26004.78857**	25955.83	26006.48	26013.35	25815.4	**25947.76563**	**1.0022**	**0.9587**
Q02357	Ank1	Ankyrin-1	99842.0625	97066.99	100563.2	108829.5	**101575.4414**	101996.1	100886.1	102063.9	100485.4	**101357.8652**	**1.0021**	**0.9351**
Q3THG9	Aarsd1	Alanyl-tRNA editing protein Aarsd1	32611.62695	36840.64	36195.71	38023.84	**35917.95361**	33799.06	37469.14	37441.2	34673.3	**35845.67383**	**1.0020**	**0.9632**
F6ZDS4	Tpr	Nucleoprotein TPR	9775.323242	8764.449	9518.291	9572.853	**9407.729004**	10181.38	9301.563	9044.512	9028.779	**9389.058838**	**1.0020**	**0.9592**
O55242	Sigmar1	Sigma non-opioid intracellular receptor 1	28341.06641	34132.43	32771.2	19019.34	**28566.00977**	22014.24	34143.49	27938.74	29944.27	**28510.18555**	**1.0020**	**0.9899**
Q04750	Top1	DNA topoisomerase 1	20285.65234	25859.76	18029.51	18082.1	**20564.25537**	20542.45	23883.23	18883.78	18793.58	**20525.75928**	**1.0019**	**0.9866**
Q64519	Sdc3	Syndecan-3	20646.47656	22068.84	16250.82	22522.25	**20372.09619**	22333.76	23550.15	18398.95	17053.91	**20334.19287**	**1.0019**	**0.9862**
Q99JX4	Eif3m	Eukaryotic translation initiation factor 3 subunit M	16747.83398	22102.38	22029.1	18610.07	**19872.34619**	22043.59	17494.6	19288.09	20516.33	**19835.65332**	**1.0018**	**0.9828**
Q9JHI5	Ivd	Isovaleryl-CoA dehydrogenase, mitochondrial	112434.8438	101040	111107.2	114538.9	**109780.25**	106781.2	114304.7	110659	106580.8	**109581.4336**	**1.0018**	**0.9567**
Q99LB6	Mat2b	Methionine adenosyltransferase 2 subunit beta	62022.4375	62340.76	56361.95	52556.82	**58320.49023**	59728.89	56960.88	56398.24	59771.68	**58214.92383**	**1.0018**	**0.9680**
Q8CGU1	Calcoco1	Calcium-binding and coiled-coil domain-containing protein 1	12596.62402	16226.76		15018.93	**14614.10514**	14453.33	14434.42	13338.68	16132.04	**14589.61938**	**1.0017**	**0.9835**
Q9CPP6	Ndufa5	NADH dehydrogenase [ubiquinone] 1 alpha subcomplex subunit 5	217597.5625	236497.8	234230.5	224278.4	**228151.0664**	216945.3	222042.3	234125.2	237967.1	**227769.9688**	**1.0017**	**0.9560**
Q9WUR9	Ak4	Adenylate kinase 4, mitochondrial	58278.00781	56227.5	48966.56	52680.96	**54038.25586**	56265.88	55893.03	51355.73	52285.94	**53950.14453**	**1.0016**	**0.9719**
Q5SRX1	Tom1l2	TOM1-like protein 2	159501.3281	172457.1	150655.6	170169.1	**163195.7969**	172259.6	163526.3	157182	158802.2	**162942.543**	**1.0016**	**0.9681**
Q8JZS0	Lin7a	Protein lin-7 homolog A	98894.66406	104757.9	105647.8	106210.4	**103877.6914**	107168.5	110556	89976.88	107170.8	**103718.0273**	**1.0015**	**0.9753**
Q9Z2D1	Mtmr2	Myotubularin-related protein 2	45949.48438	45310.77	43012.46	37788.43	**43015.28809**	44766.06	44092.85	40378.26	42563.62	**42950.19727**	**1.0015**	**0.9762**
P70265	Pfkfb2	6-phosphofructo-2-kinase/fructose-2,6-bisphosphatase 2	15574.22461	15053.16	15766.41	14689.84	**15270.90747**	15448.31	15777.13	14598.7	15169.35	**15248.37305**	**1.0015**	**0.9508**
Q8JZQ2	Afg3l2	AFG3-like protein 2	114196.4844	107268.3	110020.5	122166	**113412.8164**	117535.5	115933.6	114700.8	104824.5	**113248.6113**	**1.0014**	**0.9710**
Q8K010	Oplah	5-oxoprolinase	28491.97461	26460.03	29828.05	26547.48	**27831.88428**	26574.03	28286.17	29481.93	26828.71	**27792.70996**	**1.0014**	**0.9717**
Q9Z0N1	Eif2s3x	Eukaryotic translation initiation factor 2 subunit 3, X-linked	60961.66406	61906.07	64611.72	59566.25	**61761.42578**	64899.74	63028.53	58493.85	60276.79	**61674.72461**	**1.0014**	**0.9627**
O54784	Dapk3	Death-associated protein kinase 3	12940.92285	8484.716	11817.6	9210.246	**10613.37231**	9534.161	8243.925	12512.72	12107.07	**10599.47046**	**1.0013**	**0.9928**
Q3TWI9	Tmem63b	CSC1-like protein 2	36023.78906	31420.97	44887.62	36100.61	**37108.24707**	35725.82	36185	39413.61	36916.86	**37060.32617**	**1.0013**	**0.9875**
Q9CU62	Smc1a	Structural maintece of chromosomes protein 1A	18541.95898	15295.37	26858.49	20660.33	**20339.03809**	19926.28	19924.81	22612.57	18790.57	**20313.55762**	**1.0013**	**0.9924**
Q69ZW3	Ehbp1	EH domain-binding protein 1	9484.654297	12148.5	18958.68	15358.02	**13987.46191**	14473.99	10560.08	15819.56	15026.93	**13970.13892**	**1.0012**	**0.9944**
Q9R1R2	Trim3	Tripartite motif-containing protein 3	46095.65625	40867.13	42854.27	45693.31	**43877.5918**	41839.28	44748.06	46262.1	42447.59	**43824.25684**	**1.0012**	**0.9746**
P03911	Mtnd4	NADH-ubiquinone oxidoreductase chain 4	52952.50391	53435.97	49886.7	53215.48	**52372.66504**	52907.2	53798.3	57912.27	44628.3	**52311.51563**	**1.0012**	**0.9839**
P03899	mt-Nd3	NADH-ubiquinone oxidoreductase chain 3	301596.25	287363	307045.4	324537.3	**305135.4844**	294407.5	294859.3	335630.8	294238.3	**304783.9531**	**1.0012**	**0.9790**
Q99MR3	Slc12a9	Solute carrier family 12 member 9	11216.43457	10324	10322.28	11216.29	**10769.75195**	12000.07	11325.61	10473.69	9235.173	**10758.63599**	**1.0010**	**0.9869**
P60487	Pdxp	Chronophin	111103.7031	109276.2	105037.3	122103.1	**111880.1035**	106075.3	116394	109086.6	115506.2	**111765.5352**	**1.0010**	**0.9801**
P42125	Eci1	Enoyl-CoA delta isomerase 1, mitochondrial	237341.7031	233606.6	240058.8	244029.5	**238759.1406**	239516.9	229934.2	236937.3	247678.1	**238516.6094**	**1.0010**	**0.9566**
Q9CQ69	Uqcrq	Cytochrome b-c1 complex subunit 8	1144783.125	1085321	1051218	1158668	**1109997.438**	1152205	1171083	1012994	1099249	**1108882.672**	**1.0010**	**0.9804**
Q60803	Traf3	TNF receptor-associated factor 3	48060.21484	52374.51	49141.71	55051.39	**51156.95703**	49162.18	53947.97	51284.75	50030.14	**51106.26074**	**1.0010**	**0.9796**
Q60930	Vdac2	Voltage-dependent anion-selective channel protein 2	1215698.875	1347540	1249445	1260051	**1268183.531**	1277215	1330394	1248237	1211872	**1266929.313**	**1.0010**	**0.9745**
Q8BIJ6	Iars2	Isoleucine--tRNA ligase, mitochondrial	86274.02344	86457.4	84369.85	89032.8	**86533.51758**	87701.49	87080.2	85680.98	85342.11	**86451.19531**	**1.0010**	**0.9433**
Q9QUM9	Psma6	Proteasome subunit alpha type-6	157101.9063	170059.3	159374	161253.5	**161947.1758**	159095.5	160096.6	168127	159884.8	**161800.9609**	**1.0009**	**0.9684**
Q8CCX5	Krt222	Keratin-like protein KRT222	50678.90625	44263.63	59943.68	44307.53	**49798.43457**	51821.98	49301.27	47511.12	50385.46	**49754.95703**	**1.0009**	**0.9913**
Q60598	Cttn	Src substrate cortactin	124407.2891	119507.7	134135.3	132298.7	**127587.2285**	128403	134186.9	124018.4	123307.7	**127479.0078**	**1.0008**	**0.9805**
Q99KR3	Lactb2	Endoribonuclease LACTB2	22447.24023	12989.13	27247.97	23417.44	**21525.44434**	22126.15	23708.61	16833.06	23363.44	**21507.81396**	**1.0008**	**0.9961**
Q8BPN8	Dmxl2	DmX-like protein 2	116301.2031	116660.1	109071.5	122055	**116021.9629**	116028.9	115508.9	117020.1	115160	**115929.4648**	**1.0008**	**0.9737**
Q8VCW8	Acsf2	Medium-chain acyl-CoA ligase ACSF2, mitochondrial	204289.3125	214530.2	213614.5	207410.6	**209961.168**	215564.8	211996.9	198635.6	212979	**209794.082**	**1.0008**	**0.9717**
Q9DBR7	Ppp1r12a	Protein phosphatase 1 regulatory subunit 12A	27518.3125	29389.5	28168.08	25393.81	**27617.42529**	28873.69	29110.62	23119.82	29278.55	**27595.67041**	**1.0008**	**0.9903**
P13707	Gpd1	Glycerol-3-phosphate dehydrogenase [NAD(+)], cytoplasmic	399572.9063	423223.9	339272.2	375166.3	**384308.8438**	401173.6	421375.5	347379.7	366152.4	**384020.3047**	**1.0008**	**0.9910**
Q8VE37	Rcc1	Regulator of chromosome condensation	36313.16797	31168.46	30997.77	37150.52	**33907.47803**	32984.86	31114.82	35569.84	35860.18	**33882.42529**	**1.0007**	**0.9904**
O55126	Nipsnap2	Protein NipSnap homolog 2	241094.4375	233934.7	213178.4	285410.2	**243404.4258**	223868	273043.9	233503.7	242489.9	**243226.3789**	**1.0007**	**0.9927**
P50580	Pa2g4	Proliferation-associated protein 2G4	79228.5	80703.8	76520.27	80473.24	**79231.45313**	77288.98	89539.2	75003.61	74869.55	**79175.33594**	**1.0007**	**0.9882**
A3KFX0	Nt5c1a	Cytosolic 5'-nucleotidase 1A	6811.625	8581.456	8914.371	7625.845	**7983.324219**	6864.15	6725.161	9833.332	8488.405	**7977.761963**	**1.0007**	**0.9951**
P01831	Thy1	Thy-1 membrane glycoprotein	1888611.75	1709761	1604011	1797243	**1749906.625**	1773460	1717715	1778078	1725689	**1748735.219**	**1.0007**	**0.9857**
Q8R5J9	Arl6ip5	PRA1 family protein 3	101909.2656	98487.15	95265.3	81886.27	**94386.99805**	108940.4	103377.6	85912.09	79100.16	**94332.54297**	**1.0006**	**0.9950**

Q8BWT1	Acaa2	3-ketoacyl-CoA thiolase, mitochondrial	206114.0313	199495.8	213247	219749.4	**209651.5586**	213376	205632.3	200742.5	218399.1	**209537.4648**	1.0005	0.9852
Q8CA72	Gan	Gigaxonin		8837.157	8338.894	7249.518	**8141.85612**	7949.785	7553.56	9479.271	7567.398	**8137.503418**	1.0005	0.9950
Q8CBW3	Abi1	Abl interactor 1	58631.77734	55550.36	56901.53	62037.93	**58280.40039**	57793.16	62617.15	57505.95	55090.23	**58251.62305**	1.0005	0.9896
Q91W50	Csde1	Cold shock domain-containing protein E1	59324.95313	59773.73	54873.2	54328.16	**57075.0127**	59817.01	50444.57	55396.99	62556.96	**57053.88184**	1.0004	0.9946
Q9Z2Y8	Plpbp	Pyridoxal phosphate homeostasis protein	101376.4688	114743.2	108964	111829.2	**109228.2148**	113166	107540.8	107444.9	108622.9	**109193.6191**	1.0003	0.9917
P21836	Ache	Acetylcholinesterase	28028.30078	27074.84	27546.96	24683.51	**26833.40283**	23678.28	26898.44	29388.92	27337.51	**26825.78516**	1.0003	0.9958
P36536	Sar1a	GTP-binding protein SAR1a	56999.39063	67426.88	57177.97	67995.54	**62399.94336**	68462.57	60237.57	63466.25	57362.97	**62382.34082**	1.0003	0.9965
P58252	Eef2	Elongation factor 2	508844.3125	456366.2	448434.4	453966.1	**466902.75**	487888.8	489400.5	429219.2	460592.6	**466775.2656**	1.0003	0.9951
Q3TB82	Plekhf1	Pleckstrin homology domain-containing family F member 1	9391.633789	8662.643	5878.504	12064.36	**8999.286255**	8034.503	10108.78	8882.054	8962.767	**8997.026123**	1.0003	0.9987
Q8VE97	Srsf4	Serine/arginine-rich splicing factor 4	103129.9688	90466.08	75341.08	66567.09	**83876.05273**	74758.02	99208.91	75367.19	86087.49	**83855.40234**	1.0002	0.9984
Q811D0	Dlg1	Disks large homolog 1	107997.3594	104484	96041.49	100077.2	**102150.0137**	105405.5	105025.9	94647.46	103425	**102125.9629**	1.0002	0.9949
P97494	Gclc	Glutamate--cysteine ligase catalytic subunit	47232.01953	53994.77	52023.7	50051.11	**50825.39941**	47994.67	48729.42	51088.3	55447.39	**50814.94531**	1.0002	0.9964
Q9Z2U0	Psma7	Proteasome subunit alpha type-7	137112.4219	139745.7	144706.1	146380.3	**141986.1406**	139669.4	140137.5	142669.5	145357.6	**141958.5117**	1.0002	0.9916
Q78J03	Msrb2	Methionine-R-sulfoxide reductase B2, mitochondrial	40919.42578	43783.14	38758.45	39950.55	**40852.8916**	39457.11	38329.09	43158.59	42437.13	**40845.47949**	1.0002	0.9964
Q9CRC6	Borcs7	BLOC-1-related complex subunit 7	4148.1875	3475.933	3898.721	6309.079	**4457.980103**	4298.08	5832.103	2496.793	5202.414	**4457.347412**	1.0001	0.9995
P39688	Fyn	Tyrosine-protein kinase Fyn	22099.38867	23472.96	21157.58	18649.92	**21344.96191**	23111.92	20740.83	20376.19	21145.52	**21343.61426**	1.0001	0.9991
P14115	Rpl27a	Large ribosomal subunit protein uL15	414291.4063	470809.6	385035.3	408053.3	**419547.4063**	456777.6	431815.4	396435.2	393059.3	**419521.875**	1.0001	0.9992
Q03265	Atp5f1a	ATP synthase subunit alpha, mitochondrial	4457352	4758310	4762783	5106355	**4771199.75**	4771222	4605392	5139371	4568165	**4771037.375**	1.0000	0.9993
Q91V36	Nrbp2	Nuclear receptor-binding protein 2	14608.20605	15325.63	16952.54	15168.09	**15513.61792**	16015.09	15814.12	15595.96	14628.68	**15513.46313**	1.0000	0.9998
Q8BJ71	Nup93	Nuclear pore complex protein Nup93	16664.58203	17993.5	15970.1	14573.03	**16300.30225**	17868.76	16905.05	15339.87	15091.66	**16301.33545**	0.9999	0.9992
Q7TMY8	Huwe1	E3 ubiquitin-protein ligase HUWE1	65376.32813	58390.67	55432.47	58570.91	**59442.59668**	59203.04	60243.33	58732.29	59620.7	**59449.84082**	0.9999	0.9974
P13020	Gsn	Gelsolin	210702.25	205767.7	168041	146594.4	**182776.332**	190749.4	201321	151182.7	187950.7	**182800.9453**	0.9999	0.9990
Q8BHH2	Rab9b	Ras-related protein Rab-9B	40242.33594	42408.49	36450.24	34381.31	**38370.59375**	43923.54	40671.64	32399.08	36509.9	**38376.03906**	0.9999	0.9987
Q9JLJ2	Aldh9a1	4-trimethylaminobutyraldehyde dehydrogenase	143049.9063	145562.7	151112.5	153600.1	**148331.3008**	142537.4	149841.2	153304.2	147729.3	**148353.0156**	0.9999	0.9950
Q91ZZ3	Sncb	Beta-synuclein	1101619.25	1127686	991501.1	1152803	**1093402.328**	1163307	1137953	985188.6	1087808	**1093564.047**	0.9999	0.9977
Q9DB05	Napa	Alpha-soluble NSF attachment protein	167890.4219	191407.9	173292	185010.1	**179400.1016**	182173	180769.2	177731.8	177079.3	**179438.3242**	0.9998	0.9947
P35979	Rpl12	Large ribosomal subunit protein uL11	170208.4219	159290.4	170307.1	165452.3	**166314.5508**	160953.3	180866.8	173993.2	149627	**166360.0938**	0.9997	0.9953
Q64674	Srm	Spermidine synthase	150110.5781	142151.7	137112.6	147768.5	**144285.8398**	138539.9	142798.8	148805	147184.7	**144332.1172**	0.9997	0.9905
Q9WVA4	Tagln2	Transgelin-2	38389.97656	42146.73	39642.59	31232.75	**37853.0127**	35480.69	30114.27	39463.02	46408.34	**37866.58105**	0.9996	0.9975
Q921F2	Tardbp	TAR DNA-binding protein 43	121948.9922	140221.4	138161.2	146616	**136736.8926**	151812.7	146802.1	132226.8	116314.4	**136788.998**	0.9996	0.9958
P99029	Prdx5	Peroxiredoxin-5, mitochondrial	2163004.75	2140359	2253047	2339577	**2223997**	2149657	2230565	2255084	2264123	**2224857.25**	0.9996	0.9875
Q6RHR9	Magi1	Membrane-associated guanylate kinase, WW and PDZ domain-containing p	19120.125	19617.15	19345.9	19043.3	**19281.6167**	19998.27	20193.4	17048.48	19918.5	**19289.66064**	0.9996	0.9919
Q99M28	Rnps1	RNA-binding protein with serine-rich domain 1	32154.36523	32546.23	36778.69	30162.93	**32910.55225**	34653.09	37350.17	28031.83	31664.22	**32924.82666**	0.9996	0.9955
Q9JLT4	Txnrd2	Thioredoxin reductase 2, mitochondrial	26073.21484	23996.11	23812.52	24206.96	**24522.20215**	25202.37	25990.67	24067.34	22871.88	**24533.06152**	0.9996	0.9903
Q60932	Vdac1	Voltage-dependent anion-selective channel protein 1	2017995	1992456	1980386	2077176	**2017003.281**	2013207	2124893	2016871	1918043	**2018253.25**	0.9994	0.9798
Q9DCT2	Ndufs3	NADH dehydrogenase [ubiquinone] iron-sulfur protein 3, mitochondrial	798832.8125	772360.8	827166.5	828808	**806792.0156**	794126.2	804259.6	800612.8	830235.7	**807308.5625**	0.9994	0.9746
Q9D8X1	Cutc	Copper homeostasis protein cutC homolog	24885.60938	27191.31	32435.36	21441.85	**26488.53125**	30380.47	23164.7	25106.5	27371.89	**26505.89063**	0.9993	0.9952
Q91YQ5	Rpn1	Dolichyl-diphosphooligosaccharide--protein glycosyltransferase subunit 1	92114.11719	91329.64	92348.85	87892.9	**90921.37695**	95197.44	96129.66	89256.2	83352.09	**90983.84766**	0.9993	0.9848
Q9D8V7	Sec11c	Signal peptidase complex catalytic subunit SEC11C	25906.19727	26586.89	26204.02	21935.63	**25158.18555**	26692.92	26345.61	23019.1	24646.44	**25176.01709**	0.9993	0.9901
Q99LE6	Abcf2	ATP-binding cassette sub-family F member 2	37372.89844	34171.71	35849.02	37161.37	**36138.74805**	34266.63	37542.57	36316.82	36539.19	**36166.30176**	0.9992	0.9791
Q8CDG3	Vcpip1	Deubiquitinating protein VCPIP1	34863.62891	36962.35	38432.7	39905.07	**37540.93652**	39227.36	37679.78	37085.16	36288.36	**37570.16211**	0.9992	0.9820
Q91V92	Acly	ATP-citrate synthase	524494.625	503876.1	584288.3	497610.8	**527567.4531**	524897.4	519679.8	544825.2	522584.5	**527996.75**	0.9992	0.9840
Q8VBX6	Mpdz	Multiple PDZ domain protein	12846.50977	11504.5	11001.85	9508.964	**11215.45532**	10502.52	10633.39	11408.53	12354.15	**11224.64893**	0.9992	0.9913
G5E8V9	Arfip1	Arfaptin-1		4859.218	1302.219	5679.613	**3947.016927**	3792.813	2346.251	5368.805	4294.495	**3950.59082**	0.9991	0.9980
Q62393	Tpd52	Tumor protein D52	86762.89063	86867.25	65706.82	66316.23	**76413.29688**	87483.36	79937.09	68459.59	70052.7	**76483.18555**	0.9991	0.9928
Q8BIZ1	Anks1b	Ankyrin repeat and sterile alpha motif domain-containing protein 1B	24263.01758	24544.64	18914.94	21675.8	**22349.6001**	27990.3	22255.81	18034.23	21204.59	**22371.23291**	0.9990	0.9933
Q8VE95		UPF0598 protein C8orf82 homolog	42817.71875	38867.93	42587.07	45460.81	**42433.38184**	41586.34	44269.61	41952.49	42092.32	**42475.19043**	0.9990	0.9785
P50247	Ahcy	Adenosylhomocysteinase	288046.625	295696.1	341415.8	270619.8	**298944.5547**	309768.5	285850.2	295241.7	306134.3	**299248.6719**	0.9990	0.9855
Q5SSL4	Abr	Active breakpoint cluster region-related protein	41619.62109	40148.47	41582.67	40887.87	**41059.65723**	40906.5	43187.78	39870.02	40449.29	**41103.39551**	0.9989	0.9584
P70429	Evl	Ena/VASP-like protein	22437.59766	15945.2	17812.86	14202.03	**17599.42261**	16320.94	20079.34	15482.4	18590.39	**17618.26587**	0.9989	0.9930
Q8K2J7	Rell1	RELT-like protein 1	8329.412109	7662.15	10200.57		**8730.711263**		7981.432	6219.961	12019.32	**8740.238607**	0.9989	0.9962
P51174	Acadl	Long-chain specific acyl-CoA dehydrogenase, mitochondrial	208259.4375	204464.3	221857.6	247411	**220498.1016**	213384.5	210324	247491.4	211829.7	**220757.4102**	0.9988	0.9850
P59644	Inpp5j	Phosphatidylinositol 4,5-bisphosphate 5-phosphatase A	12077.0957	12140.38	14574.82	10933.42	**12431.42871**	12069.04	12619.84	12107.91	12989.71	**12446.625**	0.9988	0.9854
A2A935	Prdm16	Histone-lysine N-methyltransferase PRDM16	671170.25	615272.8	466126	662460.9	**603757.4844**	674204.4	674725.6	591177.8	477992.7	**604525.1172**	0.9987	0.9912
Q9JL62	Gltp	Glycolipid transfer protein	822578.8125	872497.9	782510.8	836596.7	**828546.0469**	844994.7	811707.7	877722.6	784121.5	**829636.625**	0.9987	0.9697
Q3UNH4	Gprin1	G protein-regulated inducer of neurite outgrowth 1	24757.57422	23283.63	21269.32	27462.74	**24193.31445**	24568.25	23050.36	25331.61	23950.54	**24225.18945**	0.9987	0.9824
O55234	Psmb5	Proteasome subunit beta type-5	128634	138230.1	123721	141367.4	**132988.1211**	126644.5	131180.3	140929.9	133909.7	**133166.1035**	0.9987	0.9732
P35585	Ap1m1	AP-1 complex subunit mu-1	97030.69531	91582.92	98253.06	103649.7	**97629.08789**	92669.22	100368.7	97226.61	100794.9	**97764.8418**	0.9986	0.9666
Q91WA3	Hdac11	Histone deacetylase 11	64134	60024.81	51934.89	62147.76	**59560.36523**	52125.15	58140.36	58718.39	69598.65	**59645.63574**	0.9986	0.9855
O35643	Ap1b1	AP-1 complex subunit beta-1	234615.3438	242736.6	235840.4	234842.4	**237008.7031**	246016.4	241936.9	234987.1	226494.7	**237358.793**	0.9985	0.9429
Q99K51	Pls3	Plastin-3	108670.7422	118740.4	120625.1	112156.5	**115048.1992**	105566	111265.5	121064.7	123014.4	**115227.6445**	0.9984	0.9724
Q8K199	Cmc2	COX assembly mitochondrial protein 2 homolog	4449.871094	3528.998	1675.426	2453.861	**3027.038879**		2928.105	3866.369	2300.991	**3031.821777**	0.9984	0.9955
P28063	Psmb8	Proteasome subunit beta type-8	45351.59375	45971.07	15208.87	48772.33	**38825.96509**	46863.72	47468.04	11592.77	49624.88	**38887.35352**	0.9984	0.9961
Q9Z127	Slc7a5	Large neutral amino acids transporter small subunit 1	38775.57031	63032.75	71410.26	60652.93	**58467.87695**	66548.74	38471.13	75243.35	53980.5	**58560.93066**	0.9984	0.9933
P35486	Pdha1	Pyruvate dehydrogenase E1 component subunit alpha, somatic form, mitoc	1010702.438	982867.3	1009044	1135613	**1034556.703**	1070305	1005953	1050102	1018688	**1036262.063**	0.9984	0.9650

Q9DC28	Csnk1d	Casein kinase I isoform delta	32172.49023	36419.09	37359.28	27109.04	**33264.97461**	34312.73	31814.33	35264.15	31888.03	**33319.80957**	0.9984	0.9832
P70195	Psmb7	Proteasome subunit beta type-7	66387.75781	56356.05	77309.67	71092.63	**67786.52539**	72355.44	59689.42	61950.78	77613.22	**67902.21289**	0.9983	0.9856
Q9D7N6	Mrpl30	Large ribosomal subunit protein uL30m	8638.081055	3820.478	4266.872	4366.978	**5273.102295**	7296.859	4919.995	5193.326	3719.272	**5282.363098**	0.9982	0.9948
Q9JMH9	Myo18a	Unconventional myosin-XVIIIa	54792.03516	54297.02	52700.36	49136.55	**52731.49316**	53235.94	56655	51174.28	50244.18	**52827.35059**	0.9982	0.9616
Q9JL26	Fmnl1	Formin-like protein 1	22636.94727	22332.05	14232.07	24631.72	**20958.19678**	20692.67	22912.46	22305.9	18078.18	**20997.30176**	0.9981	0.9882
Q8R361	Rab11fip5	Rab11 family-interacting protein 5	36588.46484	34895.34	38836.12	37037.19	**36839.28027**	33479.22	39378.52	36737.07	38038.71	**36908.37793**	0.9981	0.9648
Q6ZWY3	Rps27l	Small ribosomal subunit protein eS27-like	34385.09766	39855.04	34685.43	34653.72	**35894.82227**	38421.79	40193.66	34843.83	30392.66	**35962.9834**	0.9981	0.9794
Q8R0S2	Iqsec1	IQ motif and SEC7 domain-containing protein 1	87635.41406	84702.05	84337.26	89901.24	**86643.99023**	88661.63	81169.22	91074.33	86333.89	**86809.76563**	0.9981	0.9491
Q99JF5	Mvd	Diphosphomevalonate decarboxylase	20923.8457	26126.69	12175.3	15984.01	**18802.46118**	21802.39	22411.37	14049.04	17092.46	**18838.81543**	0.9981	0.9923
P36552	Cpox	Oxygen-dependent coproporphyrinogen-III oxidase, mitochondrial	11360.16211	14636.19	13225.68	13471.99	**13173.50391**	13256.87	13563.96	12119.24	13861.62	**13200.42065**	0.9980	0.9735
Q9CS84	Nrxn1	Neurexin-1	26103.62695	24896.33	30659.16	32593.7	**28563.20508**	30968.29	30296.56	26222.45	27006.6	**28623.47314**	0.9979	0.9788
Q8BHL5	Elmo2	Engulfment and cell motility protein 2	32159.63086	36072.45	28403.76	28399.53	**31258.8418**	31223.37	29654.74	29717.19	34705	**31325.0752**	0.9979	0.9768
Q6PB70	Ano8	Anoctamin-8	13668.40918	13332.67	13177.75	15615.33	**13948.54077**	14063.26	14720.24	13245.7	13883.29	**13978.12524**	0.9979	0.9647
Q920A5	Scpep1	Retinoid-inducible serine carboxypeptidase	8324.951172	7909.541	6526.636	8296.143	**7764.317505**	7188.72	12418.57	4431.829	7084.842	**7780.989136**	0.9979	0.9926
O88935	Syn1	Synapsin-1	1145102.75	1086188	1019015	1148986	**1099822.984**	1134452	1108162	1070754	1096019	**1102346.656**	0.9977	0.9420
P10126	Eef1a1	Elongation factor 1-alpha 1	653367.625	674569.5	624639.4	610185.2	**640690.4219**	650006.8	734998.5	599853	583792.7	**642162.7344**	0.9977	0.9695
Q925I1	Atad3	ATPase family AAA domain-containing protein 3	66654.25	59699.32	62166.78	65341.8	**63465.53906**	62429.43	66212.3	59903.09	65914.14	**63614.74121**	0.9977	0.9475
P68040	Rack1	Small ribosomal subunit protein RACK1	279815.875	303165.3	277045.3	276211.2	**284059.3984**	298641.5	299023.7	277084.3	264186.9	**284734.0859**	0.9976	0.9517
Q9D7X3	Dusp3	Dual specificity protein phosphatase 3	55817.37891	68666.84	63728.51	63840.05	**63013.19336**	53997.74	61601.9	69067.7	68014.66	**63170.49707**	0.9975	0.9725
P61965	Wdr5	WD repeat-containing protein 5	8583.000977	5936.885	16383.25	5688.151	**9147.821533**	6472.929	10291.49	9004.904	10914.79	**9171.026245**	0.9975	0.9934
P56395	Cyb5a	Cytochrome b5	37740.375	37300.23	37296.25	28282.35	**32654.80225**	34447.38	35193.16	28088.66	33222.77	**32737.99316**	0.9975	0.9804
Q8R123	Flad1	FAD synthase	21235.79297	20425.13	19977.05	18384.88	**20005.71338**	20370.9	20349.65	18628.61	20883.65	**20058.20313**	0.9974	0.9483
P08249	Mdh2	Malate dehydrogenase, mitochondrial	4415948.5	4398867	4282816	4845092	**4485680.75**	4438187	4510997	4562919	4478252	**4497588.25**	0.9974	0.9279
Q9QYB1	Clic4	Chloride intracellular channel protein 4	94838.82813	92204.03	83778.78	81293.38	**88028.75391**	87217.7	95693.73	87114.95	83037.48	**88265.96484**	0.9973	0.9568
O08586	Pten	Phosphatidylinositol 3,4,5-trisphosphate 3-phosphatase and dual-specifici	12170.36328	16683.21	14015.87	20042.04	**15727.87183**	23362.88	18040.02	10964.91	10714.32	**15770.53223**	0.9973	0.9907
P15116	Cdh2	Cadherin-2	115472.5781	111510.1	114220	130257.7	**117865.0898**	124386.7	124972.6	117946.2	105465.8	**118192.8281**	0.9972	0.9595
Q9QXJ1	Apbb1	Amyloid beta precursor protein binding family B member 1	15997.28516	12950.24	25304.05	27967.38	**20554.73926**	16142.82	25329.94	19255.37	21722.39	**20612.63037**	0.9972	0.9892
P07759	Serpina3k	Serine protease inhibitor A3K	28535.77148	22230.93	39904.5	33659.48	**31082.67139**	29003.52	24946.14	31657.26	39077.83	**31171.1875**	0.9972	0.9859
Q80Y17	Llgl1	Lethal(2) giant larvae protein homolog 1	82449.42969	86169.76	75056.22	78021.7	**80424.27539**	84343.09	81365.92	79927.44	76994.91	**80657.8418**	0.9971	0.9381
Q8CFE4	Scyl2	SCY1-like protein 2	14829.50977	15558.93	15042.77	15811.81	**15310.75562**	16286.92	15365.29	15843.24	13925.52	**15355.24097**	0.9971	0.9393
Q6PGB8	Smarca1	Probable global transcription activator SNF2L1	8929.512695	8742.165	12565.48	3061.782	**8324.735291**	6651.816	8356.067	9071.474	9316.971	**8349.081909**	0.9971	0.9909
Q80Z24	Negr1	Neuronal growth regulator 1	129512.3438	127647.6	113560.3	140842.1	**127890.6074**	147099.9	136223	111690.6	118053.3	**128266.7188**	0.9971	0.9709
Q9D1A2	Cndp2	Cytosolic non-specific dipeptidase	258466.1875	244922.7	251172.6	254744.1	**252326.4023**	245842.9	240457.2	255042.4	271163.9	**253126.6016**	0.9968	0.9165
Q03958	Pfdn6	Prefoldin subunit 6	61986.09375	56741.66	62174.56	56385.7	**59322.00195**	57041.76	55499.89	63188.68	62328.3	**59514.65527**	0.9968	0.9407
Q8R317	Ubqln1	Ubiquilin-1	79638.49219	78174.71	73951.62	66845.27	**74652.52148**	83264.27	70999.3	75844.82	69476.78	**74896.29492**	0.9967	0.9559
P80317	Cct6a	T-complex protein 1 subunit zeta	292882.0938	305339.8	295429.93	296510.7	**297681.1953**	305719	304215.9	280382.6	304403.2	**298680.1875**	0.9967	0.8858
Q9CR61	Ndufb7	NADH dehydrogenase [ubiquinone] 1 beta subcomplex subunit 7	86917.89844	82482.56	80405.2	82350.48	**83039.03516**	89992.91	82567.47	89086.47	71663.05	**83327.47266**	0.9965	0.9504
G3X9K3	Arfgef1	Brefeldin A-inhibited guanine nucleotide-exchange protein 1	17369.46094	16249.89	16950.79	15354.14	**16481.07275**	17148.46	18186.83	16045.76	14773.07	**16538.53003**	0.9965	0.9486
Q9WV54	Asah1	Acid ceramidase	60179.89063	54915.52	59648.01	65856.36	**60149.94629**	56645.79	63367.04	58321.89	63103.98	**60359.67383**	0.9965	0.9429
Q6GV12	Kdsr	3-ketodihydrosphingosine reductase	21345.54297	16827.58	17003.79	17107.45	**18071.09229**	18774.54	20003.25	20046.27	13716.96	**18135.25562**	0.9965	0.9736
Q9D404	Oxsm	3-oxoacyl-[acyl-carrier-protein] synthase, mitochondrial	26748.07227	25566.04	30737.8	28420.88	**27868.19873**	25578.71	26272.35	28726.41	31291.66	**27967.28125**	0.9965	0.9558
Q91WC0	Setd3	Actin-histidine N-methyltransferase	19011.26367	19238.04	16686.63	9811.697	**16186.90771**	16822.52	19191.62	15548.22	13417.35	**16244.92651**	0.9964	0.9823
P63143	Kcnab1	Voltage-gated potassium channel subunit beta-1	19794.58984	20806.76	17622.94	20458.56	**19670.71338**	22254.37	18696.12	18659.33	19359.43	**19742.31152**	0.9964	0.9508
P23242	Gja1	Gap junction alpha-1 protein	241646.7031	260891.3	233005.7	249979.9	**246380.9063**	241228.9	262990.6	230918	253989.8	**247281.793**	0.9964	0.9254
Q9WUM5	Suclg1	Succinate--CoA ligase [ADP/GDP-forming] subunit alpha, mitochondrial	350125.75	344922.7	346677.2	366068.6	**351948.5313**	386823.1	340295.8	321186.7	364715.1	**353255.1719**	0.9963	0.9338
Q99KK2	Cmas	N-acylneuraminate cytidylyltransferase	61264.3125	66454.82	52396.35	62287.26	**60600.68555**	60632.3	62059.83	63799.09	56821.8	**60828.25586**	0.9963	0.9474
Q9WU79	Prodh	Proline dehydrogenase 1, mitochondrial	23865.77734	27133.56	25758.07	26285.65	**25760.76416**	26586.94	23395.73	28315.45	25133.89	**25858.00146**	0.9962	0.9408
Q922Q4	Pycr2	Pyrroline-5-carboxylate reductase 2	67852.125	72700.48	75429.93	77875.43	**73464.49023**	73137.87	75095.6	73275.3	73489.66	**73749.60547**	0.9961	0.9010
Q3UHB1	Nt5dc3	5'-nucleotidase domain-containing protein 3	71769.72656	69126.09	61317.14	65870.41	**67020.84277**	58810.43	67890.6	70774.73	71651.3	**67281.76758**	0.9961	0.9461
O55023	Impa1	Inositol monophosphatase 1	304443.6563	300740.2	264067.9	295803.3	**291263.7656**	293780	302524.2	279991.5	293303.6	**292399.8203**	0.9961	0.9161
Q9ES56	Trappc4	Trafficking protein particle complex subunit 4	32540.01172	33497.3	34148.16	39558	**34935.86914**	38547.12	33481.85	33675.33	34584.61	**35072.22656**	0.9961	0.9471
P63094;Q6R0H7	Gnas	Guanine nucleotide-binding protein G(s) subunit alpha isoforms short;Guai	195817.2188	192801.8	191005.1	191597.3	**192805.3398**	195324.4	188076.2	190811.4	200031	**193560.7578**	0.9961	0.7987
Q9D1Q6	Erp44	Endoplasmic reticulum resident protein 44	38012.28516	37795.84	35151.98	37085.82	**37011.48242**	41054.97	35377.76	35637.3	36565.02	**37158.7627**	0.9960	0.9237
Q9JHK4	Rabggta	Geranylgeranyl transferase type-2 subunit alpha	70700.01563	76644.36	71846.34	70246.27	**72359.24414**	70775.16	72757.34	71559.33	75522.15	**72653.49609**	0.9959	0.8754
Q9D4C9	Clvs1	Clavesin-1	72112.80469	69463.91	71252.2	79072.88	**72975.44531**	68907.79	72552.26	74758.02	76872.15	**73272.55273**	0.9959	0.9162
Q8C7D2	Crbn	Protein cereblon	23327.59766	20063.89	19750.84	23453.84	**21649.04346**	21488.66	20438.02	21472.28	23553.49	**21738.11133**	0.9959	0.9433
Q9D8U8	Snx5	Sorting nexin-5	42547.49219	39648.97	40311.64	45524.24	**42008.08594**	39311.63	43518.6	41759.98	44133.9	**42181.02539**	0.9959	0.9228
P62858	Rps28	Small ribosomal subunit protein eS28	276094.2813	234860.9	231223.5	243991.2	**246542.4688**	258743.1	256340	257512.5	217641.7	**247559.3242**	0.9959	0.9455
Q9WU40	Lemd3	Inner nuclear membrane protein Man1	9454.548828	10109.08	4744.628		**8102.751465**	10364.92	8736.704	5927.69	7516.106	**8136.355713**	0.9959	0.9858
Q6NVF9	Cpsf6	Cleavage and polyadenylation specificity factor subunit 6	12334.22656	12283.53	12890.11	11630.64	**12284.62476**	8954.015	16604.23	12696.41	11091.57	**12336.55542**	0.9958	0.9757
P60710	Actb	Actin, cytoplasmic 1	6458772.5	6522803	6587191	6375287	**6486013.25**	6389825	6285876	6505506	6872644	**6513462.625**	0.9958	0.8463
Q9ESN9	Mapk8ip3	C-Jun-amino-terminal kinase-interacting protein 3	51810.58594	49331.75	45373.31	47084.3	**48399.98633**	50281.93	50783.12	47553.47	45803	**48605.37988**	0.9958	0.9140
Q9Z0H3	Smarcb1	SWI/SNF-related matrix-associated actin-dependent regulator of chromatir	15380.49023	13212.1	22860.05	19172.29	**17656.23145**	18169.2	16664.84	17438.39	18654.44	**17731.7168**	0.9957	0.9734
Q99LF4	Rtcb	RNA-splicing ligase RtcB homolog	92215.96094	86334.84	79061.51	81682.38	**84823.66992**	86057.81	84211.89	85981.09	84505.41	**85189.04883**	0.9957	0.9047
Q8BYK6	Ythdf3	YTH domain-containing family protein 3	21364.03125	17943.22	15388.71	7535.486	**15557.86414**	14086.47	13835.17	19061.16	15521.08	**15625.96802**	0.9956	0.9836

Q9R1C7	Prpf40a	Pre-mRNA-processing factor 40 homolog A	9763.022461	14153.27	7011.636	3678.59	**8651.629578**	6890.117	12967.98	10771.04	4129.118	**8689.562988**	**0.9956**	**0.9902**
Q91XY4	Pcdhga4	Protocadherin gamma-A4	31861.50781	29396.33	25524.21	27471.82	**28563.46777**	26118.75	32789.3	27999.42	27860.42	**28691.97217**	**0.9955**	**0.9501**
Q8C878	Uba3	NEDD8-activating enzyme E1 catalytic subunit	37522.76953	42919.6	40764.94	46348.91	**41889.05469**	47274.35	47803.5	35393.63	37840.02	**42077.875**	**0.9955**	**0.9609**
Q9D5V6	Syap1	Synapse-associated protein 1	9707.05957	7186.906	8456.419	6287.589	**7909.493408**	9097.395	6180.902	8520.444	7982.991	**7945.432983**	**0.9955**	**0.9719**
Q8BK30	Ndufv3	NADH dehydrogenase [ubiquinone] flavoprotein 3, mitochondrial	31559.96094	28564.97	37057.6	40403.03	**34396.38916**	32887.91	34678.24	34779.88	35877.6	**34555.90918**	**0.9954**	**0.9554**
Q05909	Ptprg	Receptor-type tyrosine-protein phosphatase gamma	9843.05957	10478.11	9734.818	12757.02	**10703.25171**	13841.68	11635.41	12591.69	4947.246	**10754.00415**	**0.9953**	**0.9816**
Q3TCJ1	Abraxas2	BRISC complex subunit Abraxas 2	32727.69727	29970.44	30758.01	28719.11	**30543.81592**	30151.51	35159	29935.79	27508.98	**30688.82129**	**0.9953**	**0.9388**
Q8VDT9	Mrpl50	Large ribosomal subunit protein mL50	12718.5918	13831.41	12817.92	8746.352	**12028.56909**	15096.1	14678.49	9079.031	9492.253	**12086.46777**	**0.9952**	**0.9775**
Q80UP8	Slc20a2	Sodium-dependent phosphate transporter 2	23104.38867	24077.04	22795.67	17284.81	**21815.479**	23650.34	17341.91	22342.68	24351.85	**21921.69385**	**0.9952**	**0.9631**
P05064	Aldoa	Fructose-bisphosphate aldolase A	1647922.125	1732618	1674735	1731286	**1696640.281**	1752630	1562667	1856040	1648637	**1704993.656**	**0.9951**	**0.9048**
P53811	Pitpnb	Phosphatidylinositol transfer protein beta isoform	19319.06445	16271.8	17263.52	13754.53	**16652.22974**	16546.29	17540.36	18315.7	14536.9	**16734.81055**	**0.9951**	**0.9554**
P48774	Gstm5	Glutathione S-transferase Mu 5	75551.44531	82994.64	78914.6	83283.68	**80186.0918**	74012.94	81403.61	83073.3	83882.66	**80593.125**	**0.9949**	**0.8933**
Q8K097	Faim2	Protein lifeguard 2	23108.29102	22511.19	14220	4020.965	**15965.11005**	17913.77	17238.06	14645.57	14395.92	**16048.33081**	**0.9948**	**0.9860**
P54728	Rad23b	UV excision repair protein RAD23 homolog B	84174.57031	77983.72	85066.05	80704.68	**81982.25586**	89566.8	83792.23	73534.23	82791.2	**82421.11523**	**0.9947**	**0.9094**
P62821	Rab1A	Ras-related protein Rab-1A	194134.4219	174865.1	189784	187335.5	**186529.7695**	157086	176866.8	215023.3	201141.4	**187529.375**	**0.9947**	**0.9434**
Q7TMF3	Ndufa12	NADH dehydrogenase [ubiquinone] 1 alpha subcomplex subunit 12	474297.0938	420894.4	494777.9	489268.8	**469809.5547**	452179	456571.9	469591.9	511008.6	**472337.8516**	**0.9946**	**0.9104**
Q8VDD8	Washc1	WASH complex subunit 1	12028.39648	11713.49	14364.66	11624.11	**12432.66406**	12605.76	12019.2	12727.82	12647.14	**12499.97876**	**0.9946**	**0.9232**
P14824	Anxa6	Annexin A6	762996.9375	796528.4	902758.1	762627.5	**806227.75**	760600.3	810657.5	830523.6	840752.2	**810633.375**	**0.9946**	**0.9106**
Q9DCS3	Mecr	Enoyl-[acyl-carrier-protein] reductase, mitochondrial	63506.68359	61973.07	60700.02	62302.56	**62120.58398**	67181.88	66498.72	56436.78	59754.84	**62468.05566**	**0.9944**	**0.9011**
P17665	Cox7c	Cytochrome c oxidase subunit 7C, mitochondrial	122620.75	129341.7	87697.38	123710.2	**115842.4941**	117126.9	117511.2	121541.2	109797.2	**116494.123**	**0.9944**	**0.9492**
Q9CQN7	Mrpl41	Large ribosomal subunit protein mL41	16784.66211	13076.89	16057.61	17101.47	**15755.15845**	18260.73	14953.35	15442.71	14723.51	**15845.07471**	**0.9943**	**0.9442**
Q9D3A9	Ttyh1	Protein tweety homolog 1	151116.0938	175450	159837	160832.8	**161808.9922**	165279	162192.7	165815.8	157718.8	**162751.5586**	**0.9942**	**0.8666**
Q91XF0	Pnpo	Pyridoxine-5'-phosphate oxidase	38027.25	34669.42	41922.57	42762.02	**39345.3125**	31951.87	32770.62	39189.67	54391.33	**39575.87354**	**0.9942**	**0.9681**
P50171	Hsd17b8	(3R)-3-hydroxyacyl-CoA dehydrogenase	75365.57813	76831.66	70601.41	87017.27	**77453.97852**	81256.57	81696.13	74309.39	74378.43	**77910.13086**	**0.9941**	**0.9134**
Q61701	Elavl4	ELAV-like protein 4	90246.85156	99845.46	93099.34	99990.9	**95795.63867**	95789.38	106579.6	94364.88	88721.04	**96363.7168**	**0.9941**	**0.9029**
Q8JZP2	Syn3	Synapsin-3	52101.58984	51262.64	38243.3	54939.93	**49136.86328**	55679.07	53418.98	43908.36	44713.43	**49429.95801**	**0.9941**	**0.9530**
Q6P8X1	Snx6	Sorting nexin-6	35800.43359	38358.78	34993.7	35661.83	**36203.68652**	37844.31	39946.52	35690.31	32203.88	**36421.25586**	**0.9940**	**0.9083**
Q8R1G6	Pdlim2	PDZ and LIM domain protein 2	14087.33789	13257.06	12952.69	10655.21	**12738.07202**	14255.62	12432.61	12690.68	11882.47	**12815.34399**	**0.9940**	**0.9339**
Q8QZT1	Acat1	Acetyl-CoA acetyltransferase, mitochondrial	803182.75	734801.3	730100.4	775026.9	**760777.8125**	762443	772956.7	766035.1	760243	**765419.4531**	**0.9939**	**0.8006**
Q8CHC4	Synj1	Synaptojanin-1	271395.2813	268364.5	253830.3	278303.6	**267973.3906**	261009.1	270167.6	275983.4	271319	**269619.7813**	**0.9939**	**0.7940**
Q9ERK4	Cse1l	Exportin-2	12716252	13078460	11617946	12444913	**12464392.75**	13844194	13466257	10852962	12000799	**12541053**	**0.9939**	**0.9225**
Q921S7	Mrpl37	Large ribosomal subunit protein mL37	14565.83301	14085.81	16782.44	16227.66	**15415.43359**	13959.1	15658.41	18017.13	14408.38	**15510.75659**	**0.9939**	**0.9347**
Q99L47	St13	Hsc70-interacting protein	408646.9375	405869.1	439117.8	407264.3	**415224.5156**	425983.5	417346.2	409338.4	418591.1	**417814.7891**	**0.9938**	**0.7755**
B1AZI6	Thoc2	THO complex subunit 2				3944.227	**3944.227295**			3969.193		**3969.192871**	**0.9937**	
A2ASS6	Ttn	Titin	165832.625	98787.76	122194.1	120960.9	**126943.8633**	187932.8	84968.8	100228.7	137873.2	**127750.8965**	**0.9937**	**0.9770**
P05201	Got1	Aspartate aminotransferase, cytoplasmic	2311594.75	2345474	2231804	2369291	**2314540.625**	2231426	2367561	2288367	2429998	**2329337.75**	**0.9936**	**0.7893**
Q6PE13	Prrt3	Proline-rich transmembrane protein 3	29742.24023	26798.29	31750.32	34723.21	**30753.5166**	29541.04	32902.56	32280.57	29077.37	**30950.38574**	**0.9936**	**0.9219**
Q99P58	Rab27b	Ras-related protein Rab-27B	45470.48438	48267.35	42342.69	47134.7	**45803.80762**	38542.47	47499.75	51520.61	46828.03	**46097.71387**	**0.9936**	**0.9255**
P24472	Gsta4	Glutathione S-transferase A4	234410.3125	224082.1	232893.7	228341.8	**229931.9844**	220157	238588.4	218220.3	248687.3	**231413.2656**	**0.9936**	**0.8543**
P53702	Hccs	Holocytochrome c-type synthase	34948.19922	23026.2	31397.07	29411.45	**29695.72949**	27925.85	21643.87	34961.31	35019.19	**29887.5542**	**0.9936**	**0.9640**
Q9CR39	Wdr45b	WD repeat domain phosphoinositide-interacting protein 3	7307.762695	7780.672	7946.057	9061.155	**8023.911743**	4951.896	5979.529	13129.92	8241.718	**8075.766602**	**0.9936**	**0.9786**
Q921L3	Tmco1	Calcium load-activated calcium channel	22140.04688	19743.88	26058.96	25179.68	**23280.64404**	22316.1	22447	28095.48	20870.67	**23432.31006**	**0.9935**	**0.9461**
Q8K0C9	Gmds	GDP-mannose 4,6 dehydratase	26603.5918	20390.92	21565.96	21472.65	**22508.2793**	18902.14	23893.18	23380.5	24446.33	**22655.53857**	**0.9935**	**0.9402**
Q8R164	Bphl	Valacyclovir hydrolase	157486.1719	151360.2	156173.4	164917.3	**157484.2773**	158948.4	163369.4	154097.3	157651.8	**158516.7266**	**0.9935**	**0.7715**
P09813	Apoa2	Apolipoprotein A-II	33962.28516	24253.19	34373.7	32350.51	**31234.92334**	26624.14	23540.78	36643.07	38956.69	**31441.16846**	**0.9934**	**0.9644**
Q8K353	Cystm1	Cysteine-rich and transmembrane domain-containing protein 1	36898.16797	39277.66	50303.03	47466.37	**43486.30566**		30370.95	53565.33	47387.94	**43774.74023**	**0.9934**	**0.9684**
P35564	Canx	Calnexin	429159.625	393713.8	383701.7	418858.1	**406358.3125**	414029.5	431957.3	379488.4	410916.6	**409097.9375**	**0.9933**	**0.8630**
P08113	Hsp90b1	Endoplasmin	558139.0625	554883.2	593750.1	493638.9	**550102.8281**	576391.3	565464	502079.2	571620.3	**553888.7109**	**0.9932**	**0.8935**
Q9R0D8	Wdr54	WD repeat-containing protein 54	38758.09375	41728.84	37720.77	45172.7	**40845.10059**	39051	38564.69	43438.04	43453.46	**41126.7959**	**0.9932**	**0.8998**
Q9R0E1	Plod3	Multifunctional procollagen lysine hydroxylase and glycosyltransferase LH3		14296.44		22903.38	**18599.91162**	21044.1	16413.76			**18728.93164**	**0.9931**	**0.9813**
Q6ZPE2	Sbf1	Myotubularin-related protein 5	64619.0625	55733.21	58735.93	60733.32	**59955.38184**	59012.93	61186.73	59569.52	61716.63	**60371.45215**	**0.9931**	**0.8398**
Q9JME5	Ap3b2	AP-3 complex subunit beta-2	122406.3359	113735.6	115451	125598.1	**119297.748**	123743	125502	117360.6	113905.6	**120127.7734**	**0.9931**	**0.8389**
P10852	Slc3a2	Amino acid transporter heavy chain SLC3A2	458722.9375	464031.9	400028.1	471770.9	**448638.4609**	434343.8	461971.5	438437.4	472312.9	**451766.4141**	**0.9931**	**0.8734**
P97352	S100a13	Protein S100-A13	167546.9531	143354.1	147082.4	144398	**150595.3516**	166468.1	148575.2	147680	143864.6	**151647.0039**	**0.9931**	**0.8947**
Q9JKF6	Nectin1	Nectin-1	15625.59277	14923.54	15938.57	21912.1	**17099.95142**	22993.04	20813.82	14138.91	10932.68	**17219.6145**	**0.9931**	**0.9718**
O55137	Acot1	Acyl-coenzyme A thioesterase 1	274667.9063	282172.6	269479.6	284296.4	**277654.1406**	278767.2	282874.6	267224.3	289638.8	**279626.2031**	**0.9929**	**0.7460**
Q99JA0	Calca	Calcitonin gene-related peptide 1	19101.44141	19251.55	14433.76	15802.53	**17147.32056**	18984.46	17897.21	17658.6	14538.01	**17269.56885**	**0.9929**	**0.9392**
Q8BP48	Metap1	Methionine aminopeptidase 1	14546.53613	14305.72	18151.77	14265.71	**15317.43335**	13184.39	15348.91	19620.46	13552.78	**15426.63599**	**0.9929**	**0.9524**
Q63918	Cavin2	Caveolae-associated protein 2	9093.148438	8968.26	12807.07	5131.869	**9000.085938**	7906.785	8935.435	11030.55	8385.306	**9064.519165**	**0.9929**	**0.9712**
Q8R0N6	Adhfe1	Hydroxyacid-oxoacid transhydrogenase, mitochondrial	8909.057617	9137.981	6922.726	7932.417	**8225.545532**		10119.57	5771.361	8963.37	**8284.765788**	**0.9929**	**0.9638**
Q9DB72	Btbd17	BTB/POZ domain-containing protein 17	60019.83594	63731.8	70008.68	80581	**68585.3291**	63783.94	65186.62	78576.03	68778.71	**69081.3252**	**0.9928**	**0.9323**
Q9JLZ3	Auh	Methylglutaconyl-CoA hydratase, mitochondrial	781374.9375	701076.3	796681.6	796868.7	**769000.3594**	724147.6	769725.2	790364.1	814016.6	**774563.375**	**0.9928**	**0.8582**
B2RY04	Dock5	Dedicator of cytokinesis protein 5	22810.72266	26910.71	23044.73	24417.59	**24295.94043**	26771.67	22817.28	25318.88	22981.88	**24472.42529**	**0.9928**	**0.8996**
P21107	Tpm3	Tropomyosin alpha-3 chain	159502.8906	149231.2	167563.8	163395.1	**159923.2461**	160033.2	169723.1	140770	173823.4	**161087.418**	**0.9928**	**0.8936**
Q71LX4	Tln2	Talin-2	85576.125	85071.16	81686.03	89546.16	**85469.86914**	83646.8	87596.07	91037.53	82089.75	**86092.53711**	**0.9928**	**0.8173**

Q8BPU7	Elmo1	Engulfment and cell motility protein 1	28706.48633	26622.79	22185.05	25706.72	25805.2627	28393.04	26511.93	22770.08	26298.14	25993.29883	0.9928	0.9200
Q9QZQ1	Afdn	Afadin	14157.8877	12665.54	14538.02	14316.76	13919.55103	13969.29	14467.96	14310.35	13337.41	14021.25439	0.9927	0.8436
P62996	Tra2b	Transformer-2 protein homolog beta	35518.13672	34327.76	32227.92	34613.05	34171.71484	36598.36	34708.48	30843.35	35543.61	34423.45166	0.9927	0.8665
P05202	Got2	Aspartate aminotransferase, mitochondrial	1938542.125	1945111	1923126	1987393	1948542.844	1982564	1955609	1973283	1940532	1962996.969	0.9926	0.4179
Q9CWD3	Nudt17	Nucleoside diphosphate-linked moiety X motif 17	13714.83984	13808.36	17366.75	14082.39	14743.08569	15192.96	15390.97		13977.64	14853.85677	0.9925	0.9239
Q6NZJ6	Eif4g1	Eukaryotic translation initiation factor 4 gamma 1	55350.71094	59343.65	53683.55	48987.13	54341.25879	52548.01	62682.36	51645.78	52125.46	54750.40137	0.9925	0.9084
Q8BUB4	Wdfy2	WD repeat and FYVE domain-containing protein 2				1936.746	1936.745972	1477.333	1329.133	1171.32	3827.78	1951.391571	0.9925	
Q91ZR1	Rab4b	Ras-related protein Rab-4B	79566.35156	81073.89	68256.56	73996.52	75723.33203	83267.78	78979.52	71788.73	71158.54	76298.64453	0.9925	0.8938
P97819	Pla2g6	85/88 kDa calcium-independent phospholipase A2	12789.9082	15826.89	10848.04	11359.13	12705.99268	11483.66	13694.07	12707.69	13325.59	12802.75098	0.9924	0.9393
Q8C4X2	Csnk1g3	Casein kinase I isoform gamma-3	13666.02441	13940.92	11535.94	13846.43	13247.3291	12713.14	13013.29	13115.59	14551.96	13348.49536	0.9924	0.8906
Q8BTY1	Kyat1	Kynurenine--oxoglutarate transaminase 1	29225.48047	17731.11	29967.39	32159.59	27270.89258	27512.35	25773.44	29369.7	27269.3	27481.19873	0.9923	0.9516
Q9QUP5	Hapln1	Hyaluro and proteoglycan link protein 1	139017.3438	142513.2	137770.8	135412.5	138678.4805	123024.5	141306	147890.1	146776.9	139749.3711	0.9923	0.8630
Q61249	Igbp1	Immunoglobulin-binding protein 1	15290.81055	17174.36	15228.52	17013.6	16176.82422	15141.31	15951.17	16764.21	17354.67	16302.83789	0.9923	0.8663
Q6P5E8	Dgkq	Diacylglycerol kinase theta	13806.52539	13263.52	15801.32	15175.84	14511.80249	13691.52	14946.08	16652.44	13211.1	14625.28711	0.9922	0.9105
Q8CHU3	Epn2	Epsin-2	39949.125	35575.38	37274.26	38069.07	37716.95703	37780.29	34739.31	39378.24	40152.03	38012.46777	0.9922	0.8506
Q9JKC8	Ap3m1	AP-3 complex subunit mu-1	60900.82031	58293.73	64432.62	66740.67	62591.96094	67149.81	62174.53	61275.68	61730.49	63082.62988	0.9922	0.8393
Q9D6G9	Cmtm5	CKLF-like MARVEL transmembrane domain-containing protein 5	83824.82031	81505.75	79988.64	69757.75	78769.24023	82198.13	87281.92	74460.3	73681.4	79405.4375	0.9920	0.8922
P61953	Gng11	Guanine nucleotide-binding protein G(I)/G(S)/G(O) subunit gamma-11	28754.51367	26730.23	24473.29	18545.87	24625.97656	23221.49	21516.88	27100.22	27465.23	24825.95801	0.9919	0.9422
P31324	Prkar2b	cAMP-dependent protein kinase type II-beta regulatory subunit	141833.1094	145729.5	123745.8	140654.9	137990.8086	139979.7	151288.8	127737.7	137456.7	139115.7363	0.9919	0.8752
P62806	H4c1	Histone H4	2819505.25	2927468	3140373	2793754	2920274.688	2987987	2955077	3225234	2608169	2944116.563	0.9919	0.8786
O35345	Kpna6	Importin subunit alpha-7	44290.75781	49883.42	44399.45	41229.48	44950.77539	43779.16	45192.25	45690.3	46612.18	45318.47363	0.9919	0.8526
Q91X96	Rabif	Guanine nucleotide exchange factor MSS4	45994.95313	39964.77	33389.57	44733.25	41020.63574	42960.73	41296.59	38981.45	42192.65	41357.85352	0.9918	0.9137
P28652	Camk2b	Calcium/calmodulin-dependent protein kinase type II subunit beta	722054	701846.8	723125.6	828932.6	743989.75	702029.7	679069.3	810461.2	808914.6	750118.6719	0.9918	0.8963
Q80TV8	Clasp1	CLIP-associating protein 1	103329.8906	103260.3	108219.2	97197.2	103001.6406	102302.8	103845.1	99968.88	109310.3	103856.7949	0.9918	0.7855
Q9D1D4	Tmed10	Transmembrane emp24 domain-containing protein 10	59987.69531	54469.2	54034.23	52644.64	55283.94336	61268.61	58158	51434.96	52111.19	55743.19043	0.9918	0.8785
Q9R1P0	Psma4	Proteasome subunit alpha type-4	221127.4844	241334.2	236847.5	239875.3	234796.1445	241053.6	235011.8	232039.3	238907.3	236752.9883	0.9917	0.7126
P47963	Rpl13	Large ribosomal subunit protein eL13	237025	224757.2	219617.7	243325.7	231181.3906	276438.1	234738.4	201756	219505.8	233109.5586	0.9917	0.9126
P07309	Ttr	Transthyretin	20628.12695	28381.64	40164.83	28552.09	29431.67236	27036.77	22951.52	33768.39	34955.39	29678.01807	0.9917	0.9618
Q8C1A5	Thop1	Thimet oligopeptidase	54241.73047	57728.98	51045.01	54357.04	54343.19043	50932.73	54853.51	59552.47	53858.67	54799.34668	0.9917	0.8461
P56528	Cd38	ADP-ribosyl cyclase/cyclic ADP-ribose hydrolase 1	17285.79883	17632.59	17810.34	13052.39	16445.2793	21259.05	15173.53	15306.69	14595.42	16583.67212	0.9917	0.9453
Q920I9	Wdr7	WD repeat-containing protein 7	95805.15625	97799.92	96251.44	101806.5	97915.74414	98613.34	97704.59	99756.81	98886.16	98740.22461	0.9917	0.5851
Q9D172	Gatd3	Glutamine amidotransferase-like class 1 domain-containing protein 3, mito	460791.9375	429929	441521.9	449651.6	445473.6094	452555.1	428770.9	439143.2	476471.9	449235.2969	0.9916	0.7680
P11499	Hsp90ab1	Heat shock protein HSP 90-beta	3509518	3366140	2988581	3039662	3225975.313	3435550	3426777	2952895	3198035	3253313.938	0.9916	0.8777
P58389	Ptpa	Serine/threonine-protein phosphatase 2A activator	156837.3594	169492.1	166571	167845.4	165186.457	163812.8	161495.6	161696.8	179346.9	166588	0.9916	0.7944
Q99KI0	Aco2	Aconitate hydratase, mitochondrial	2736927.75	2753301	2776862	2837340	2776107.5	2823256	2784479	2806665	2784327	2799681.625	0.9916	0.3628
Q920E5	Fdps	Farnesyl pyrophosphate synthase	99757.61719	118889.4	108504.5	94470.28	105405.4453	122145.7	121576.6	89702.05	91832.4	106314.1875	0.9915	0.9336
Q61584	Fxr1	RNA-binding protein FXR1	13347.33105	14523.6	17144.11	15772.7	15196.93506	16901.6	16674.18	15447.65	12289.57	15328.24756	0.9914	0.9251
P46096	Syt1	Synaptotagmin-1	167423.8594	159306.8	175657.3	198571	175239.75	175089.5	176032.1	173971.2	181954.8	176761.875	0.9914	0.8661
Q61335	Bcap31	B-cell receptor-associated protein 31	12871.67188	13338.55	14285.73	12074.05	13142.50244	12610.68	14066.63	14452.67	11898.57	13257.13745	0.9914	0.8849
Q9Z1T1	Ap3b1	AP-3 complex subunit beta-1	13128.31396	12889.45	18900.62	11823.4	14185.448	14400.42	20273.23	13296.85	9268.161	14309.66479	0.9913	0.9658
P54731	Faf1	FAS-associated factor 1	27501.9668	32747.99	27797.55	33647.81	30423.8291	31116.27	27056.3	34255.58	30333.61	30690.44287	0.9913	0.9070
Q3UX10	Tubal3	Tubulin alpha chain-like 3	22716.77344	26122.49	20931.8	12556.97	20582.00806	24105.7	23979.89	16017.21	18949.51	20763.07617	0.9913	0.9604
Q9Z1Q2	Abhd16a	Phosphatidylserine lipase ABHD16A	68257.14063	64367.26	58669.07	58933.19	62556.66406	69540.45	69000.66	57400.61	56487.33	63107.2627	0.9913	0.9011
Q8BMS1	Hadha	Trifunctional enzyme subunit alpha, mitochondrial	359810.0938	356521.8	376949.8	354772.8	362013.6484	372131.8	365988.4	371863.9	350988.7	365243.2188	0.9912	0.6654
Q8BLJ3	Plcxd3	PI-PLC X domain-containing protein 3	65882.64844	61061.25	67599.48	75012.65	67389.00879	74147.52	64197.77	66345.66	67272.34	67990.82031	0.9911	0.8729
Q99LB2	Dhrs4	Dehydrogenase/reductase SDR family member 4	55480.64453	59301.95	62683.63	59554.81	59255.25586	62046.77	56034.64	60432.55	60668.64	59795.64941	0.9910	0.7928
O08553	Dpysl2	Dihydropyrimidinase-related protein 2	3748669.75	3714233	3767977	3820991	3762967.438	3731203	3671261	3919656	3867206	3797331.563	0.9910	0.5993
Q9DC69	Ndufa9	NADH dehydrogenase [ubiquinone] 1 alpha subcomplex subunit 9, mitocho	474825.5938	452166.5	449960.8	503527.8	470120.1719	490037	498629.2	462929.3	446070.3	474416.4531	0.9909	0.8132
Q64518	Atp2a3	Sarcoplasmic/endoplasmic reticulum calcium ATPase 3	218697.8438	230437.3	260416.1	261814.2	242841.3711	229240.4	239397.3	245108.9	266509.1	245063.9297	0.9909	0.8735
Q3UGY8	Arfgef3	Brefeldin A-inhibited guanine nucleotide-exchange protein 3	18830.54102	17331.96	16870.09	18731.87	17941.11523	19409.16	18588.41	15899.35	18524.56	18105.37012	0.9909	0.8625
Q811U4	Mfn1	Mitofusin-1	19330.98242	17462.14	20730.52	20964.84	19622.12012	18733.51	20864.24	21551.07	18060.27	19802.27344	0.9909	0.8817
Q6ZPQ6	Pitpnm2	Membrane-associated phosphatidylinositol transfer protein 2	11746.8623	11262.38	12304.68	11989.52	11825.86157	11809.48	10918.17	12622.93	12389.39	11934.99072	0.9909	0.8118
Q99J08	Sec14l2	SEC14-like protein 2	58414.38281	56722.89	42239.98	55782.15	53289.85156	55155.01	55490.54	45850.16	58636.74	53783.11133	0.9908	0.9187
Q9WTT4	Atp6v1g2	V-type proton ATPase subunit G 2	120487.9688	112771.4	120657.5	128042.2	120489.7598	103255.6	113393.5	122601.6	147170	121605.1797	0.9908	0.9139
Q9D0B6	Pbdc1	Protein PBDC1	23968.80664	25725.13	21753.7	16013.55	21865.29688	27330.37	23122.18	19426.11	18394.07	22068.18164	0.9908	0.9470
Q9D7N9	Apmap	Adipocyte plasma membrane-associated protein	67297.4375	64860.22	69759.39	65246.16	66790.80273	68700.55	71488.73	59317.71	70136.27	67410.81738	0.9908	0.8419
P17183	Eno2	Gamma-enolase	667583.4375	838203.6	722504.6	781706.1	752499.4375	745888.1	688908.1	821825.8	781366	759496.9844	0.9908	0.8851
Q6P6J9	Txndc15	Thioredoxin domain-containing protein 15	9062.015625	8121.951	4483.518	12192.64	8465.030029	13880.11	11329.39	4154.647	4811.934	8544.019897	0.9908	0.9790
Q9WVM1	Racgap1	Rac GTPase-activating protein 1	56640.75781	57641.28	59885.71	63578.8	59436.6377	64940.97	57298.77	58506.86	59218.48	59991.26953	0.9908	0.8167
P60603	Romo1	Reactive oxygen species modulator 1	50866.79297	46236.15	43899.81	33218.39	43555.28613	44280.85	55904.94	29265.25	46397.08	43962.03027	0.9907	0.9533
Q8VEM8	Slc25a3	Solute carrier family 25 member 3	854204	890517.6	927312.1	983830.9	913966.1406	943318.5	953098.9	886459.3	907509.7	922596.5781	0.9906	0.7947
Q64433	Hspe1	10 kDa heat shock protein, mitochondrial	2137304	1908630	2030300	2123415	2049912.406	2080103	2050546	1999712	2147658	2069504.719	0.9905	0.7594
Q640R3	Hepacam	Hepatic and glial cell adhesion molecule	301211.875	263902	300281.8	295518.8	290228.6094	272750.7	297208.7	309234.6	292894.7	293022.1641	0.9905	0.8188
Q6PHZ2	Camk2d	Calcium/calmodulin-dependent protein kinase type II subunit delta	107224.0703	102936.3	103486.5	98715.45	103090.5762	106364	111527	101178.7	97280.17	104087.459	0.9904	0.7886
Q6P5E4	Uggt1	UDP-glucose:glycoprotein glucosyltransferase 1	26317.31641	24199.4	26394.96	22531.79	24860.86768	25556.65	27277.48	24700.44	22871.5	25101.51758	0.9904	0.8597

Q9D0F9	Pgm1	Phosphoglucomutase-1	439675.5	478418.5	452817.7	471569.4	**460620.2891**	448357	460694.2	480141.6	471147.9	**465085.1875**	0.9904	0.7033
Q62167	Ddx3x	ATP-dependent RNA helicase DDX3X	106114.7031	108489.7	97861.59	97643.43	**102527.3652**	111069.9	112840.6	91417.2	98780.52	**103527.0645**	0.9903	0.8693
Q05816	Fabp5	Fatty acid-binding protein 5	757946.75	860374.1	619975.7	660906.9	**724800.9844**	729754.6	811158.3	718124.1	668791.2	**731957.0469**	0.9902	0.9108
P11438	Lamp1	Lysosome-associated membrane glycoprotein 1	23155.09766	34801.3	21584.32	18440.87	**24495.396**	22796.25	23630.63	33926.34	18600.86	**24738.51953**	0.9902	0.9615
Q9CZ44	Nsfl1c	NSFL1 cofactor p47	104006.0234	100368.9	95477.38	99808.89	**99915.29883**	99723.09	96605.68	111850.8	95457.5	**100909.2676**	0.9901	0.8184
P59016	Vps33b	Vacuolar protein sorting-associated protein 33B	29989.07422	29366.79	31052.09	33290.93	**30924.72168**	29838.39	30680.94	30412.36	33998.29	**31232.49805**	0.9901	0.8172
Q8BP47	NARS1	Asparagine--tRNA ligase, cytoplasmic	152174.375	159021.7	159286.2	146847.4	**154332.4219**	149647.2	155860.4	166707.2	151306.3	**155880.2773**	0.9901	0.7612
Q9ER00	Stx12	Syntaxin-12	65653.64844	64066.6	69130.42	67402.2	**66563.21582**	66876.37	71073.87	68533.02	62443.01	**67231.56543**	0.9901	0.7631
Q8BW75	Maob	Amine oxidase [flavin-containing] B	181294.5313	185460.6	162951.5	213491.3	**185799.457**	205211	189142	178868.1	177447.8	**187667.2227**	0.9900	0.8838
Q60899	Elavl2	ELAV-like protein 2	29697.20898	32275.85	24544.22	27683.5	**28550.19531**	29955.88	37793.45	36877.87	10723.93	**28837.78052**	0.9900	0.9661
Q80TZ3	Dnajc6	Putative tyrosine-protein phosphatase auxilin	115967.6797	108906	94158.75	108705.2	**106934.418**	115916.8	116689.2	99682.72	99764.06	**108013.2051**	0.9900	0.8760
Q8VBV7	Cops8	COP9 signalosome complex subunit 8	109220.3516	107575.2	102943.3	97921.13	**104414.9941**	103590.1	106963.5	94159.85	117165.4	**105469.7188**	0.9900	0.8512
P16330	Cnp	2',3'-cyclic-nucleotide 3'-phosphodiesterase	7119345.5	6842337	6635381	6447490	**6761138.125**	6899037	6870661	6821257	6728383	**6829834.375**	0.9899	0.6607
Q9CPX6	Atg3	Ubiquitin-like-conjugating enzyme ATG3	40257.09766	39768.11	32476.71	38059.15	**37640.26855**	38612.21	41393.11	33541.8	38547.45	**38023.64551**	0.9899	0.8793
Q8R010	Aimp2	Aminoacyl tRNA synthase complex-interacting multifunctional protein 2	43657.55469	44463.24	40022.24	38689.11	**41708.03613**	43184.68	40516.43	43173.03	41673.08	**42136.80469**	0.9898	0.7897
P56376	Acyp1	Acylphosphatase-1	201317.8594	197667.1	179772.1	199267.8	**194506.1914**	190284.9	197927.8	195479.5	202390.1	**196520.6133**	0.9897	0.7301
Q80ZK0	Mrps10	Small ribosomal subunit protein uS10m	18700.6582	14136.63	16055.41	15830.64	**16180.83423**	17372.9	16638.73	17359.69	14028.95	**16350.06885**	0.9896	0.8952
Q9JHQ5	Lztfl1	Leucine zipper transcription factor-like protein 1	30540.33008	30066.74	29834.9	26256.42	**29174.59863**	28565.94	29115.47	27940.88	32310.79	**29483.26904**	0.9895	0.8308
Q8K274	Fn3krp	Ketosamine-3-kinase	33860.67969	27505.67	32378.31	33927.62	**31918.0708**	30324.4	33051.75	32577.05	33071.04	**32256.05908**	0.9895	0.8444
E9Q6P5	Ttc7b	Tetratricopeptide repeat protein 7B	29835.25195	30467.68	28977.73	27648.18	**29232.21094**	28169.52	29729.34	32195.35	28073.57	**29541.9458**	0.9895	0.7949
Q9D8V0	Hm13	Minor histocompatibility antigen H13	21482.52539	25753.98		22478.01	**23238.17057**		30515.87	16452.94		**23484.40332**	0.9895	0.9671
Q99KH8	Stk24	Serine/threonine-protein kinase 24	44505.14453	46375.05	43637.21	43917.86	**44608.81641**	43910.16	44732.09	46737.98	44957.98	**45084.55469**	0.9894	0.5987
Q8VD37	Sgip1	SH3-containing GRB2-like protein 3-interacting protein 1	34412.85156	33190.72	33433.14	35510.92	**34136.90723**	34101.76	36011.58	33803.49	34087.83	**34501.16602**	0.9894	0.6371
P54797	Tango2	Transport and Golgi organization 2 homolog	27173.32227	26554.47	28876.57	25844.42	**27112.1958**	28173.57	25832.61	26875.59	28728.33	**27402.52588**	0.9894	0.7627
O08810	Eftud2	116 kDa U5 small nuclear ribonucleoprotein component	192008.0469	190178.9	183232.9	186588.5	**188002.0742**	208065.3	194482.5	167735.9	189810.7	**190023.6211**	0.9894	0.8220
Q61627	Grid1	Glutamate receptor ionotropic, delta-1	2240.66748	3104.421	5138.123	4456.311	**3734.880432**		3497.479	5380.777	2447.307	**3775.187744**	0.9893	0.9710
P07901	Hsp90aa1	Heat shock protein HSP 90-alpha	1613588.5	1525761	1496806	1465736	**1525472.781**	1623350	1547237	1429218	1567954	**1541939.625**	0.9893	0.7613
O35379	Abcc1	Multidrug resistance-associated protein 1	11245.37207	9967.188	11934.62	11027.26	**11043.60962**	11757.78	11957.34	10012.44	10925.77	**11163.33179**	0.9893	0.8491
Q6ZQ18	Efr3b	Protein EFR3 homolog B	54054.53906	53588.4	55435.73	54147.06	**54306.43262**	55936.41	53517.17	55841.83	54292.69	**54897.02734**	0.9892	0.4399
Q8BXK8	Agap1	Arf-GAP with GTPase, ANK repeat and PH domain-containing protein 1	16741.69922	11934.53	13435.39	17116.04	**14806.9126**	15658.29	14027.17	13671.5	16518.18	**14968.7854**	0.9892	0.9138
Q91WS0	Cisd1	CDGSH iron-sulfur domain-containing protein 1	516346.6563	497773.7	459757.4	464032.9	**484477.6641**	489224.7	520826.5	477824.9	471244.7	**489780.1953**	0.9892	0.7720
Q00612	G6pdx	Glucose-6-phosphate 1-dehydrogenase X	82882.74219	86921.3	81147.74	79395.82	**82586.90039**	87057.9	86869.69	80123.59	79950.14	**83500.32813**	0.9891	0.7343
P15105	Glul	Glutamine synthetase	1918287	2171414	1965871	2137043	**2048153.625**	2118760	2017183	2039303	2108162	**2070851.688**	0.9890	0.7473
Q8R0Y6	Aldh1l1	Cytosolic 10-formyltetrahydrofolate dehydrogenase	244632.5781	243270.1	212766.8	232521.3	**233297.6797**	245323.7	248275.3	229224.5	220712.4	**235884**	0.9890	0.8019
Q91X56	S1pr5	Sphingosine 1-phosphate receptor 5	72039.07031	65566.35	58890.01	70300.33	**66698.93945**	61887.91	62127.25	73293.69	72464.91	**67443.4375**	0.9890	0.8684
Q62376	Snrnp70	U1 small nuclear ribonucleoprotein 70 kDa	61430.40625	56787.56	61257.39	69508.02	**62245.84375**	60843.09	63952.87	61395.64	65585.2	**62944.20117**	0.9889	0.8160
Q5IRJ6	Slc30a9	Proton-coupled zinc antiporter SLC30A9, mitochondrial	53620.50781	53235.29	55227.32	59126.04	**55302.29004**	56326.3	51601.28	57937.91	57834.76	**55925.06348**	0.9889	0.7667
Q78RX3	Smim12	Small integral membrane protein 12	16546.41406	9931.394	6155.21	4483.615	**9279.158081**	9664.873	10651.41	6597.698	10621.72	**9383.925171**	0.9888	0.9718
P05132	Prkaca	cAMP-dependent protein kinase catalytic subunit alpha	166098.7031	148989.2	143443	195143.1	**163418.4961**	173245.5	173957.4	162394.6	151457.2	**165263.6914**	0.9888	0.8899
Q6P9J5	Kank4	KN motif and ankyrin repeat domain-containing protein 4	20322.92383	17910.08	19418.94	21685.87	**19834.45215**	23099.86	18387.45	19552.67	19195.33	**20058.82764**	0.9888	0.8696
Q5DTY9	Kctd16	BTB/POZ domain-containing protein KCTD16	16070.56543	12249.36	10984.34	13832.43	**13284.17358**	14010.72	15265.6	11660.09	12802.63	**13434.76074**	0.9888	0.9144
O55091	Impact	Protein IMPACT	40197.04688	36271.63	34472.39	32641.54	**35895.65234**	33969.84	36506.96	37294.31	37445.12	**36304.05566**	0.9888	0.8284
Q3URD3	Slmap	Sarcolemmal membrane-associated protein	19607.05469	20021.61	20111.73	18289.8	**19507.54883**	21955.04	20112.52	17735.15	19115.82	**19729.63428**	0.9887	0.8286
Q61234	Snta1	Alpha-1-syntrophin	102061.8359	108583.3	119260.2	101916.6	**107955.4863**	110303.3	112553.4	104613.9	109300.6	**109192.7969**	0.9887	0.7883
P97445	Cacna1a	Voltage-dependent P/Q-type calcium channel subunit alpha-1A	34666.52344	31716.31	26183.11	29623.09	**30547.25635**	34024.39	31100.94	27337.09	31127.97	**30897.59863**	0.9887	0.8814
P51859	Hdgf	Hepatoma-derived growth factor	58665.88672	58347.2	59132.18	61828.7	**59493.49023**	54255.79	57868.62	66226.66	62353.71	**60176.19629**	0.9887	0.8107
Q9QYS9	Qki	KH domain-containing RNA-binding protein QKI	50580.1875	53050.3	46116.13	42877.54	**48156.03809**	45026.88	53939.05	50318.7	45556.82	**48710.3623**	0.9886	0.8640
Q80U19	Daam2	Disheveled-associated activator of morphogenesis 2	26567.78125	20834.28	19299.26	23530.79	**22558.02686**	24411.49	22393.84	22483.61	21983.25	**22818.04736**	0.9886	0.8825
Q9CWU6	Uqcc1	Ubiquinol-cytochrome-c reductase complex assembly factor 1	41470.98828	38438.7	42031.37	38592.08	**40133.28516**	42613.28	42895.87	38439.21	38463.04	**40602.84961**	0.9884	0.7736
Q8R1F6	Hid1	Protein HID1	5075.111816	5953.269		5466.412	**5498.26416**	6739.092	4556.521	5395.027	5560.694	**5562.833374**	0.9884	0.9146
Q3TYP4	Tmem88b	Transmembrane protein 88B	17075.77148	15999.06	13764.09	2881.165	**12430.0213**	15431.56	15445.64	11145.73	8281.666	**12576.15186**	0.9884	0.9698
Q8BGN8	Synpr	Synaptoporin	40120.65625	43202.72	32471.88	47607.52	**40850.69434**	47049.16	43617.51	46570.51	28088.28	**41331.36523**	0.9884	0.9332
Q8BKC5	Ipo5	Importin-5	70271.50781	70512.08	71262.76	61063.04	**68277.34473**	72057.51	71694.64	58952.12	73637.26	**69085.38086**	0.9883	0.8529
P28271	Aco1	Cytoplasmic aconitate hydratase	181493.1406	182443.2	168011.5	169192.3	**175285.0352**	171463.2	183416.9	171268.1	183310	**177364.5625**	0.9883	0.7028
P42208	Septin2	Septin-2	265847.0938	266469.1	255164.7	225491.7	**253243.1289**	269414.6	262532.3	226472.3	266609.2	**256257.082**	0.9882	0.8354
Q9D0M1	Prpsap1	Phosphoribosyl pyrophosphate synthase-associated protein 1	45055.36719	46577.49	53538.74	52197.75	**49342.33691**	50735.63	48793.14	48281.41	51917.33	**49931.87793**	0.9882	0.8015
Q9CR95	Necap1	Adaptin ear-binding coat-associated protein 1	146699.7813	146631.6	147798.8	165752	**151720.5352**	160163.1	146259.9	156679.5	151046	**153537.1133**	0.9882	0.7567
P32921	Wars1	Tryptophan--tRNA ligase, cytoplasmic	187906.625	182322	174103.3	177654.7	**180496.6367**	184090	179048.2	169316.7	198176.8	**182657.9258**	0.9882	0.7585
Q8K310	Matr3	Matrin-3	122257.2891	125428.1	112128	142012.7	**125456.5156**	132329.5	133532.2	122758.5	119215.1	**126958.8223**	0.9882	0.8403
Q8BGN2	D3Ertd751e	UPF0462 protein C4orf33 homolog	8002.41748	11079.23	15247.98	23325.31	**14413.73596**	5157.537	21324.97	9231.915	22636.15	**14587.64331**	0.9881	0.9757
Q9QZ88	Vps29	Vacuolar protein sorting-associated protein 29	104805.9844	92275.9	107195.3	109403.4	**103420.125**	113552.8	97957.34	95895.86	111267.4	**104668.3262**	0.9881	0.8400
Q64521	Gpd2	Glycerol-3-phosphate dehydrogenase, mitochondrial	428963.5	419952	408122.2	400800.1	**414459.4453**	441840.3	440412.7	397241.6	398469.9	**419491.1484**	0.9880	0.7310
Q9QZS3	Numb	Protein numb homolog	12332.64453	12203.71	15897.45	12462.56	**13224.0896**	15341.95	11337.29	11150.07	15710.45	**13384.93994**	0.9880	0.9196
Q8CGY8	Ogt	UDP-N-acetylglucosamine--peptide N-acetylglucosaminyltransferase 110 [illegible]	54329.22656	56090.43	52553.58	55796.83	**54692.51563**	60555.45	57286.96	49421.93	54174.9	**55359.81152**	0.9879	0.7988
Q6P5F9	Xpo1	Exportin-1	40508.14844	42886.12	38873.73	37136.85	**39851.21387**	40917.84	42863.88	39420.48	38155.48	**40339.41895**	0.9879	0.7690

Q61330	Cntn2	Contactin-2	149517.3594	138967	153952.2	160439.5	150719.0117	143275.5	144065	156666	166260.6	152566.7734	0.9879	0.8038
Q8CI51	Pdlim5	PDZ and LIM domain protein 5	32955.56641	33712.4	34344.76	36459.17	34367.97363	35409.43	38109.39	32681.52	32988.96	34797.32764	0.9877	0.7799
A2AN08	Ubr4	E3 ubiquitin-protein ligase UBR4	51330.35547	53342.38	48658.11	47908.04	50309.71973	49927.16	51546.85	49922.17	52362.45	50939.65723	0.9876	0.6664
Q80UP5	Ankrd13a	Ankyrin repeat domain-containing protein 13A	19544.79492	17094.04	12351.71	14485.39	15868.98389	15662.18	14980.66	17729.89	15902.02	16068.68799	0.9876	0.9087
Q80WJ7	Mtdh	Protein LYRIC	37078.08594	30529.44	34961.6	36820.46	34847.39795	37797.63	34436.92	33426.19	35485.33	35286.51855	0.9876	0.8134
Q8R3V5	Sh3glb2	Endophilin-B2	188693.3438	192268	184712.6	209423.1	193774.2617	198328.5	198850.9	192934.1	194753.9	196216.8633	0.9876	0.6792
Q9CXR1	Dhrs7	Dehydrogenase/reductase SDR family member 7	25067.5293	25444	29529.54	26737.27	26694.58398	26615.06	26496.22	28038.13	26975.16	27031.146	0.9875	0.7637
Q8VHC3	Selenom	Selenoprotein M	17149.24805	22354.25	17708.23	19913.22	19281.2373	16595.1	17673.65	23894.1	19939	19525.46387	0.9875	0.9069
Q9Z1Z2	Strap	Serine-threonine kinase receptor-associated protein	53813.80859	53517.18	46423.16	48606.24	50590.09473	52092.92	50447.52	47995.5	54393.41	51232.33691	0.9875	0.7872
P17563	Selenbp1	Methanethiol oxidase	87988.75781	86631.74	94601.84	92499.79	90430.5332	85497.27	87728.28	99570.88	93523.33	91579.9375	0.9874	0.7647
P27773	Pdia3	Protein disulfide-isomerase A3	402196.1563	390008.2	370495	361615.9	381078.8281	395294.3	396965.3	392659.3	358773.1	385922.9922	0.9874	0.7210
Q9Z0V7	Timm17b	Mitochondrial import inner membrane translocase subunit Tim17-B	19492.62109	18546.07	22317.85	21141.48	20374.50635	21791.71	16835.4	22707.68	21199.88	20633.66602	0.9874	0.8728
Q5U458	Dnajc11	DnaJ homolog subfamily C member 11	50787.53516	50884.02	52643.09	56366.29	52670.2334	55562.77	48734.03	60264.38	48804.43	53341.39941	0.9874	0.8356
Q7TMK9	Syncrip	Heterogeneous nuclear ribonucleoprotein Q	134021.9063	155335.1	146804.6	140794.2	144238.9492	150497.5	152434.6	143608.7	137779.1	146079.9961	0.9874	0.7549
O70492	Snx3	Sorting nexin-3	159772.1875	145927.4	142479.2	179070.2	156812.2578	169924.6	153177.3	165507.8	146660.2	158817.4648	0.9874	0.8462
Q8BGQ7	Aars1	Alanine--tRNA ligase, cytoplasmic	73515.64844	70731.17	75635.14	66232.23	71528.54883	70113.27	72923.96	70155.52	76589.55	72445.57422	0.9873	0.7307
P63054	Pcp4	Calmodulin regulator protein PCP4	550457.5	1032528	724355.6	684448.9	747947.5469	568527.5	882554.9	854476.8	724662.3	757555.3438	0.9873	0.9411
Q02053	Uba1	Ubiquitin-like modifier-activating enzyme 1	526208.5625	526588	521406.7	529424.6	525906.9609	526425.2	532063.5	533485.7	538729.1	532675.875	0.9873	0.0667
O35387	Hax1	HCLS1-associated protein X-1	41314.62891	40551.75	48631.5	47368.41	44466.57324	44056.17	46019.83	48223.58	41870.45	45042.50684	0.9872	0.8233
Q923Z0	Gprc5b	G-protein coupled receptor family C group 5 member B	42143.02344	49395.71	51617.46	57626.91	50195.77637	49541.87	50933.25	49919.75	52993.91	50847.19434	0.9872	0.8496
Q9Z307	Kcnj16	Inward rectifier potassium channel 16	11093.0332	8409.42	6445.749	3993.404	7485.401489	8041.254	8778.983	7375.768	6135.42	7582.856445	0.9871	0.9536
Q8C1B1	Camsap2	Calmodulin-regulated spectrin-associated protein 2	8149.124512	7362.204	7819.742	8344.578	7918.912231	8470.985	7351.275	7419.911	8846.2	8022.092896	0.9871	0.8195
Q8R326	Pspc1	Paraspeckle component 1	49306.63281	50411.91	48327.32	54638.21	50671.0166	53261.59	51294.36	49657.64	51114.69	51332.07031	0.9871	0.6892
Q6PIE5	Atp1a2	Sodium/potassium-transporting ATPase subunit alpha-2	13501137	13651948	13777533	14301703	13808080.25	14624319	14510682	13528854	13296449	13990076	0.9870	0.6487
Q9CQ79	Txndc9	Thioredoxin domain-containing protein 9	39661.48438	41219.9	33270.31	49301.47	40863.29102	43701.29	44116.1	40954.14	36838.63	41402.53809	0.9870	0.8888
Q9JKB1	Uchl3	Ubiquitin carboxyl-terminal hydrolase isozyme L3	63474.25391	60843.24	55132.87	56191.71	58910.51953	60145.52	58629.28	57940.41	62036.91	59688.03223	0.9870	0.7315
O88844	Idh1	Isocitrate dehydrogenase [NADP] cytoplasmic	340279.5625	351289.9	349409.7	336259.5	344309.6641	380950.1	366783.3	343532	304195.8	348865.2969	0.9869	0.7994
Q99LX0	Park7	Parkinson disease protein 7 homolog	430844.875	491626.9	463082.6	469369.5	463730.9531	438984.3	446646	488986.9	504876.3	469873.3594	0.9869	0.7731
Q3TES0	Iqsec3	IQ motif and SEC7 domain-containing protein 3	19160.64063	21845.02	20654.11	20665.38	20581.28857	22194.48	19997.33	20370.51	20853.46	20853.94531	0.9869	0.7216
P28660	Nckap1	Nck-associated protein 1	55375.45703	53571.59	51936.14	56689.39	54393.14355	59931.61	58929.83	49949.5	51652.48	55115.85254	0.9869	0.8000
P10637	Mapt	Microtubule-associated protein tau	249028.5625	225329.4	251380.1	251407.9	244286.4961	241609	240287.2	230458.3	277792.3	247536.7031	0.9869	0.7984
Q9JLQ0	Cd2ap	CD2-associated protein	19794.77344	18811.19	23238.88	22540.71	21096.38818	22576.59	22707.65	21091.07	19135.59	21377.72314	0.9868	0.8419
Q9D753	Exosc8	Exosome complex component RRP43	10480.02441	7401.847	9445.985	9031.693	9089.887451	10380.79	11089.69	8611.545	6765.704	9211.933105	0.9868	0.9196
Q9Z224	Mocs2	Molybdopterin synthase sulfur carrier subunit	10658.59668	10693.84	9999.944	10725.58	10519.49194	11874.15	12278.53	7911.886	10578.85	10660.85242	0.9867	0.8923
P48771	Cox7a2	Cytochrome c oxidase subunit 7A2, mitochondrial	71083.85156	63423.43	57795.66	71071.87	65843.70117	66332.77	64213.04	68991.32	67377.74	66728.71582	0.9867	0.8025
Q91YR1	Twf1	Twinfilin-1	79467.92188	54555.5	73346.96	72659.16	70007.38379	76354.83	53015.46	78186.83	76259.25	70954.09277	0.9867	0.9102
P48453	Ppp3cb	Serine/threonine-protein phosphatase 2B catalytic subunit beta isoform	409226.9375	451933.3	446494.1	428094.6	433937.2266	453684.4	434433.8	423578	447534.2	439807.6016	0.9867	0.6365
Q64133	Maoa	Amine oxidase [flavin-containing] A	364053.875	354143.6	347215.9	353030.4	354610.9297	372685.4	354374.1	363093.6	347744.7	359474.4375	0.9865	0.4788
Q3ULJ0	Gpd1l	Glycerol-3-phosphate dehydrogenase 1-like protein	93296.11719	114638.5	97850.9	109790.6	103894.0371	111221	104432.1	104560.1	101083.1	105324.0742	0.9864	0.8009
P84228	H3c2	Histone H3.2	2710816.75	2849099	2663875	2196663	2605113.5	3286104	2922758	1869188	2487444	2641373.469	0.9863	0.9176
Q64096	Mcf2l	Guanine nucleotide exchange factor DBS	13429.14355	12386.26	7004.314	13804.58	11656.07483	13786.39	12036.39		9632.589	11818.4541	0.9863	0.9420
Q99JI4	Psmd6	26S proteasome non-ATPase regulatory subunit 6	47560.30078	47305.91	38408.62	43008.29	44070.7793	45116.07	46027.85	46784	40823.95	44687.96875	0.9862	0.8158
Q7TPM6	Fsd1	Fibronectin type III and SPRY domain-containing protein 1	65966.98438	71222.58	56536.22	58040.34	62941.53125	67276.82	70530.66	54189.63	63307.71	63826.20703	0.9861	0.8637
Q9CPY7	Lap3	Cytosol aminopeptidase	371070.375	361056.8	346006.1	386895.7	366257.2344	346548.6	379363.1	372530.1	387326.8	371442.125	0.9860	0.6886
P70302	Stim1	Stromal interaction molecule 1	16943.37305	16873.93	17314.58	18701.42	17458.32422	19214.94	20222.93	15170.81	16217.84	17706.62817	0.9860	0.8517
Q8BHN0	Ppm1l	Protein phosphatase 1L	15723.7334	22767.39	19435.56	15022.09	18237.19287	18341.69	15497.48	19802.07	20347.65	18497.22144	0.9859	0.9054
Q8CHG7	Rapgef2	Rap guanine nucleotide exchange factor 2	32817.57813	33050.53	34134.58	31025.28	32756.99072	34888.01	37931.02	29707.48	30373.08	33224.89551	0.9859	0.8270
Q8BGW1	Fto	Alpha-ketoglutarate-dependent dioxygenase FTO	14952.20117	14174.56	11542.13	14525.21	13798.52612	15012.1	13575.25	12448.25	14947.12	13995.68359	0.9859	0.8477
Q9EQU5	Set	Protein SET	30578.47656	31876.58	33452.21	33222.28	32282.3877	36884.04	32230.36	35161.84	26704.82	32745.26514	0.9859	0.8490
Q8BUK6	Hook3	Protein Hook homolog 3	13401.8877	17125.04	14724.85	14714.28	14991.51343	15276.11	11724.67	18491.29	15338.18	15207.56396	0.9858	0.8961
Q8R2H9	Phospho1	Phosphoethanolamine/phosphocholine phosphatase	28080.60352	27659.6	28341.33	23813.13	26973.66406	30025.98	25346.46	28082.4	25996.6	27362.85889	0.9858	0.8043
Q9WTM5	Ruvbl2	RuvB-like 2	48389.84766	41120.5	45693.58	41792.75	44249.17188	44357.58	44452.29	47025.82	43718.09	44888.44629	0.9858	0.7426
P17710	Hk1	Hexokinase-1	1511956.875	1532188	1440014	1609520	1523419.75	1544009	1580920	1524372	1532417	1545429.25	0.9858	0.5740
Q9CPW0	Cntnap2	Contactin-associated protein-like 2	109570.6172	108591.5	112614	122163.5	113234.9004	110783.8	105010	119081.8	124612.8	114872.1113	0.9857	0.7693
Q80X50	Ubap2l	Ubiquitin-associated protein 2-like	38514.63281	33889.09	30459.39	33069.43	33983.1377	35278.4	35023.71	32747.02	34852.02	34475.28906	0.9857	0.7910
Q6PAR5	Gapvd1	GTPase-activating protein and VPS9 domain-containing protein 1	34989.64453	38455.59	31884.6	38726.32	36014.03955	38146.95	36868.73	34767.59	36365.55	36537.20605	0.9857	0.7766
P97807	Fh	Fumarate hydratase, mitochondrial	659089.8125	627637	661491.8	675397.7	655904.0625	664430.1	686193.6	649945.4	661172.1	665435.3125	0.9857	0.4786
Q8BWW4	Larp4	La-related protein 4		3523.716			3523.71582				3575.057	3575.057129	0.9856	
Q3UHJ0	Aak1	AP2-associated protein kinase 1	137057.5156	132563.4	134216	140601.7	136109.6289	136187.1	135176.3	141515.7	139513.4	138098.1211	0.9856	0.4192
Q9EPU0	Upf1	Regulator of nonsense transcripts 1	40237.33203	36251.78	34807.85	36173.88	36867.70996	36961.07	39504.2	36228.7	36953.71	37411.9209	0.9855	0.7057
Q91VR2	Atp5f1c	ATP synthase subunit gamma, mitochondrial	1160267	1111521	1213680	1232095	1179390.5	1208493	1210737	1102154	1266290	1196918.281	0.9854	0.7030
Q920M5	Coro6	Coronin-6	48950.75781	51044.43	50546.74	38134.96	47169.22168	45810.48	46847.02	47865.53	50971.37	47873.59961	0.9853	0.8352
Q62261	Sptbn1	Spectrin beta chain, non-erythrocytic 1	963319.5625	953707.1	1015068	943567.9	968915.5469	997010.3	968787.1	992510.4	975382.1	983422.4688	0.9852	0.4332
P49442	Inpp1	Inositol polyphosphate 1-phosphatase	82577.36719	81393.56	76790.57	80815.45	80394.23633	82234.55	81793.31	82024.02	80368.4	81605.06836	0.9852	0.3960
Q9DCS9	Ndufb10	NADH dehydrogenase [ubiquinone] 1 beta subcomplex subunit 10	532307.125	540447.8	550170.6	569552.9	548119.6094	567570.3	569358.4	506376.6	582282.4	556396.9141	0.9851	0.6750

Q9D1X0	Nol3	Nucleolar protein 3	40321.14453	42658.18	46936.96	36388.25	41576.13281	45404.35	39124.8	39016.33	45275.95	42205.35742	0.9851	0.8328
P62245	Rps15a	Small ribosomal subunit protein uS8	192422.5	195015	175110	175252.5	184449.9922	215878.7	187845.3	176863.4	168380.9	187242.082	0.9851	0.8187
Q61655	Ddx19a	ATP-dependent RNA helicase DDX19A	43743.625	44316.09	45767.88	39136.7	43241.07227	45051.93	43534.23	41553.06	45443.78	43895.75195	0.9851	0.7107
P00920	Ca2	Carbonic anhydrase 2	660158.75	626471.3	620135.7	605709.9	628118.8906	596634.9	582140.5	695849.7	676007.6	637658.1719	0.9850	0.7656
Q4KMM3	Oxr1	Oxidation resistance protein 1	93991.17969	88941.51	90256.15	99044.45	93058.32031	95304.13	97632.59	91520.55	93433.92	94472.79688	0.9850	0.6079
P35235	Ptpn11	Tyrosine-protein phosphatase non-receptor type 11	72868.44531	68056.5	59326.88	59156.77	64852.14844	65433.06	69195.27	63550.29	65182.41	65840.25879	0.9850	0.7923
D3YZP9	Ccdc6	Coiled-coil domain-containing protein 6	39981.71094	45257.32	38097.15	36435.41	39942.89844	40268.12	42743.37	38576.04	40619.06	40551.64844	0.9850	0.7813
P23953	Ces1c	Carboxylesterase 1C	16041.83203	8171.755	12939.46	11621.7	12193.68713	15222.33	11664.04	10414.33	12219.97	12380.17065	0.9849	0.9259
P21278	Gna11	Guanine nucleotide-binding protein subunit alpha-11	75755.64844	73482.43	71937.27	59672.87	70212.05371	70881.45	73015.87	72431.64	68826.19	71288.78711	0.9849	0.7819
Q3UMC0	Afg2a	ATPase family gene 2 protein homolog A	934338.375	899668.6	870135.8	955343.4	914871.5313	916437.6	918949.3	967170.8	913052.6	928902.5469	0.9849	0.5603
Q99JT9	Adi1	Acireductone dioxygenase	23643.02539	22245.44	19491.39	21542.25	21730.52637	22565.25	21059.62	20938.94	23694.08	22064.47461	0.9849	0.7689
P57080	Usp25	Ubiquitin carboxyl-terminal hydrolase 25		25549.18		26452.88	26001.03223	23542.36	29259.95			26401.1543	0.9848	0.9027
Q69ZF3	Gba2	Non-lysosomal glucosylceramidase	9297.911133	9764.663	9718.981	11462.2	10060.93921	8608.382	10249.16	10799.32	11212.36	10217.30518	0.9847	0.8408
Q6PFX8	Rimkla	N-acetylaspartylglutamate synthase A	48543.59766	53293.1	54909.68	40994.58	49435.23926	51174.74	50335.63	54549.19	44756.19	50203.93945	0.9847	0.8433
Q9CRY7	Gdpd1	Lysophospholipase D GDPD1	85393.64844	87192.2	78552.91	83342.91	83620.41992	91357.84	86589.8	81141.27	80612.26	84925.29297	0.9846	0.6927
O08539	Bin1	Myc box-dependent-interacting protein 1	273930.375	269108.3	264230.1	294488.6	275439.3438	299364	276936.3	277529.7	265156.2	279746.5391	0.9846	0.6742
Q91VD9	Ndufs1	NADH-ubiquinone oxidoreductase 75 kDa subunit, mitochondrial	758035.875	745590.6	768977.8	777544.8	762537.2656	756588.8	784461.1	792192.9	764625.5	774467.0625	0.9846	0.3124
Q9Z2U1	Psma5	Proteasome subunit alpha type-5	165932.9531	156666	164636.8	169676.7	164228.1211	148990.2	167777.7	177920.6	172566.1	166813.6328	0.9845	0.7193
Q9QXT0	Cnpy2	Protein canopy homolog 2	20529.09961	19967.93	23198.46	21226.33	21230.45605	19047.07	23709.26	20539.62	22969.68	21566.40479	0.9844	0.8030
Q05512	Mark2	Serine/threonine-protein kinase MARK2	7734.550293	6005.522	9114.781	7352.173	7551.756592	8913.183	3921.421	8796.31	9059.331	7672.561096	0.9843	0.9343
P39054	Dnm2	Dynamin-2	131707.5469	125455.6	151583.9	136699	136361.5195	155200.3	139001.1	134510.6	125462.4	138543.5859	0.9842	0.8027
Q9CQU3	Rer1	Protein RER1	30985.67188	26518.71	25139.66	28669.5	27828.38623	30194.4	25411.69	30561.63	26928.24	28273.99268	0.9842	0.8119
Q9Z1G4	Atp6v0a1	V-type proton ATPase 116 kDa subunit a 1	644240.6875	662566.6	629423.1	673696.9	652481.8281	697144.8	667692.3	642632.3	644257.7	662931.75	0.9842	0.5400
Q9QYA2	Tomm40	Mitochondrial import receptor subunit TOM40 homolog	42916.00391	64841.12	56058.25	68710.59	58131.49023	67783.41	58622.65	49647	60198.18	59062.80957	0.9842	0.8959
P16014	Chgb	Secretogranin-1	12631.25879	9623.827	12957.47	11793.31	11751.46729	12541.12	11339.39			11940.25146	0.9842	0.8811
P52825	Cpt2	Carnitine O-palmitoyltransferase 2, mitochondrial	61978.31641	62845.32	71760.44	65932.07	65629.03613	66102.13	60471.53	71061.82	69105.62	66685.27344	0.9842	0.7524
Q5SS80	Dhrs13	Dehydrogenase/reductase SDR family member 13	13010.64453	14665.55	10199.39	11018.45	12223.50684	12476.17	12448.63	12002.31	12760.16	12421.81787	0.9840	0.8519
Q3KNM2	Marchf5	E3 ubiquitin-protein ligase MARCHF5	25099.4082	23952.11	27029.81	33910.78	27498.02734	30500.44	31254.43	26105.89	23916.05	27944.20313	0.9840	0.8803
P16546	Sptan1	Spectrin alpha chain, non-erythrocytic 1	804145.9375	773468.4	827851.7	773795.6	794815.3906	832915.3	797113.8	794747.6	806089.7	807716.5781	0.9840	0.4453
Q8BNW9	Kbtbd11	Kelch repeat and BTB domain-containing protein 11	55178.58203	54218.46	56069.12	57783.86	55812.50293	56307.85	57157.18	54133.33	59283.98	56720.58594	0.9840	0.5134
Q8BKE6	Cyp20a1	Cytochrome P450 20A1	3888.851074		5267.535		4578.192871		3372.962	6374.338	4212.052	4653.117432	0.9839	0.9564
Q9D1L9	Lamtor5	Ragulator complex protein LAMTOR5	9669.683594	7963.74	8317.37	9718.11	8917.226074	7993.553	9842.067	9375.688	9042.215	9063.380981	0.9839	0.8158
Q8BH04	Pck2	Phosphoenolpyruvate carboxykinase [GTP], mitochondrial	40667.75781	38470.72	41155.48	43139.49	40858.36328	37588.57	40438.84	41715.68	46370.25	41528.33691	0.9839	0.7567
Q99N28	Cadm3	Cell adhesion molecule 3	244212.7969	215694	226891.9	211827.1	224656.4492	211868.5	217657.4	231309.6	252739.8	228393.8008	0.9836	0.7588
D3YXK2	Safb	Scaffold attachment factor B1	46313.16016	47067.06	43416.07	42886.03	44920.58105	45066.82	49315.94	44674.67	43615.64	45668.26855	0.9836	0.6623
Q91YT7	Ythdf2	YTH domain-containing family protein 2		1613.348		4280.446	2946.896973	1295.945	4329.138	3588.174	2771.28	2996.133911	0.9836	0.9710
P97427	Crmp1	Dihydropyrimidinase-related protein 1	1558654.25	1455157	1371112	1452608	1459382.813	1582510	1643472	1369976	1340061	1484004.531	0.9834	0.7817
P24527	Lta4h	Leukotriene A-4 hydrolase	110240.75	122477.2	111981.5	111517.1	114054.1426	118144.2	119570.3	108152	118074.9	115985.3555	0.9833	0.6353
Q9DCU6	Mrpl4	Large ribosomal subunit protein uL4m	15505.9834	13993.83	16904.41	12772.16	14794.09546	14563.53	14493.3	15402	15725.9	15046.18335	0.9832	0.7995
Q9CVB6	Arpc2	Actin-related protein 2/3 complex subunit 2	104273.4609	110979	94706.98	99347.07	102326.623	110087.4	106146.2	101005	99056.41	104073.7656	0.9832	0.6978
Q59J78	Ndufaf2	NADH dehydrogenase [ubiquinone] 1 alpha subcomplex assembly factor 2	110637.5547	103466.1	102143.7	102384.6	104657.9824	105603.6	106059.6	103116.9	111041.8	106455.4941	0.9831	0.5167
P60843	Eif4a1	Eukaryotic initiation factor 4A-I	551546.8125	558227.7	540414	504955.7	538786.0391	567899.4	586725.9	510921.8	526647.9	548048.75	0.9831	0.6780
P57746	Atp6v1d	V-type proton ATPase subunit D	283902.0313	277793	283182.5	298058.8	285734.0703	300944.8	305492.4	276038.5	280149.2	290656.2031	0.9831	0.5853
Q62351	Tfrc	Transferrin receptor protein 1	55807.33984	54525.16	51556.03	57367.79	54814.08008	62741.91	58848.02	54488.65	46979.94	55764.62793	0.9830	0.8004
Q3UMB9	Washc4	WASH complex subunit 4	12060.08691	11517.91	12033.34	14033.76	12411.27417	14833.65	13657.03	12312.35	9703.867	12626.72339	0.9829	0.8671
Q3U5Q7	Cmpk2	UMP-CMP kinase 2, mitochondrial	63736.87109	69469.18	64894.19	65458.66	65889.72559	65679.39	66103.85	67677.37	68673.63	67033.56055	0.9829	0.4533
P55066	Ncan	Neurocan core protein	144822.4219	191201.5	143053.8	152535.7	157903.3516	148054.2	185753	156850.9	151952.8	160652.7031	0.9829	0.8525
Q9Z1S5	Septin3	Neuronal-specific septin-3	131455.7031	158936	120268.7	110077.5	130184.4648	162369.1	163730.7	74938.2	128791.9	132457.4922	0.9828	0.9255
Q8C7X2	Emc1	ER membrane protein complex subunit 1	37847.60156	35922.5	36605.98	33159.85	35883.98145	39477.65	37532.43	30809.98	38229.86	36512.47852	0.9828	0.7829
O88703	Hcn2	Potassium/sodium hyperpolarization-activated cyclic nucleotide-gated cha	117695.9375	113569.6	110075.2	102705.1	111011.4668	121094.1	116050.9	106394.4	108299.6	112959.7813	0.9828	0.6910
P47754	Capza2	F-actin-capping protein subunit alpha-2	383747.5625	329261.3	363715.3	374794.8	362879.7344	347028.5	358458.9	399970.2	371539.5	369249.2578	0.9828	0.7128
Q9EP69	Sacm1l	Phosphatidylinositol-3-phosphatase SAC1	203575.4531	213764.2	202688.3	208500.1	207132.0039	219955	209231	198236.3	215671.1	210773.3477	0.9827	0.5230
P0DP60	Lynx1	Ly-6/neurotoxin-like protein 1	49988.16016	52908.43	44253.93	49167.42	49079.48535	47438.96	48624.58	51933.37	51796.64	49948.38867	0.9826	0.6968
Q5F2E7	Nufip2	FMR1-interacting protein NUFIP2	13035.06738	14545.87	18597.9	2686.484	12216.32971	11173.47	17674.38	9803.019	11080.26	12432.78345	0.9826	0.9567
Q9D4H8	Cul2	Cullin-2	44454.74219	47006.32	38157.95	36741.26	41590.06641	44378.93	44249.45	40750.98	39933.74	42328.27637	0.9826	0.7954
Q5M8N4	Sdr39u1	Epimerase family protein SDR39U1	110553.7344	109596.3	131468.1	114042.3	116415.1191	115785.7	121469.7	116573.1	120159	118496.8711	0.9824	0.7075
Q3UHE1	Pitpnm3	Membrane-associated phosphatidylinositol transfer protein 3	12105.37793	9628.263	10843.8	11992.58	11142.50708	12590.55	12536.03	11581.35	8662.146	11342.52075	0.9824	0.8604
Q61425	Hadh	Hydroxyacyl-coenzyme A dehydrogenase, mitochondrial	102660.9688	105586.7	97563.3	100961.9	101693.2207	109552.4	101878.1	98306.26	104359.4	103524.0547	0.9823	0.5506
P61222	Abce1	ATP-binding cassette sub-family E member 1	51513.91406	49696.2	53469.95	48159.86	50709.97949	55831.41	55957.35	43897.35	50824.92	51627.75684	0.9822	0.7746
Q8BJY1	Psmd5	26S proteasome non-ATPase regulatory subunit 5	103100.0625	102454.1	105205.7	98520.59	102320.1211	106528.4	104286	100149.3	105748.2	104177.9551	0.9822	0.3870
Q9CPX8	Uqcr11	Cytochrome b-c1 complex subunit 10	75436.41406	84350.16	76575.14	82098.87	79615.14453	85982.48	80087.95	72826.9	85374.4	81067.92969	0.9821	0.7103
Q9R0Q6	Arpc1a	Actin-related protein 2/3 complex subunit 1A	195082.75	198427.9	170384.1	176139	185008.4297	189051.1	202394.5	178037	184065.9	188387.1289	0.9821	0.7094
P70333	Hnrnph2	Heterogeneous nuclear ribonucleoprotein H2	72512.3125	80988.22	73109.48	75988.7	75649.67578	73034.97	79501.59	81832.16	73775.21	77035.98047	0.9820	0.6491
Q60631	Grb2	Growth factor receptor-bound protein 2	81823.9375	87013.47	81162.12	81012.11	82752.9082	88462.05	85534.28	81984.91	81107.55	84272.19531	0.9820	0.5187
Q9CWL8	Ctnnbl1	Beta-catenin-like protein 1	21384.75391	19631.25	15649.66	20966.65	19408.07886	20719.8	21535.4	16758.05	20045.99	19764.81104	0.9820	0.8384

Q9CS42	Prps2	Ribose-phosphate pyrophosphokinase 2	22184.95508	23122.63	24016.02	22565.32	22972.23193	19217.37	23521.7	23224.52	27618.4	23395.49561	0.9819	0.8181
Q99N84	Mrps18b	Small ribosomal subunit protein mS40	7918.771973	12142.84	9402.03	11834.08	10324.42957	9623.025	10748.18	12517.54	9171.148	10514.97534	0.9819	0.8843
Q9QUR6	Prep	Prolyl endopeptidase	57827.60156	55602.88	50707.89	57203.84	55335.55078	55727.95	54482.1	54776.69	60458.19	56361.23047	0.9818	0.6471
Q8CI32	Bag5	BAG family molecular chaperone regulator 5	9534.955078	9882.103	12541.11	11892.73	10962.72412	11487.5	10646.42	12383.5	10150.9	11167.08179	0.9817	0.8255
Q8CHT1	Ngef	Ephexin-1	20537.92578	21942.28	9606.127	18064.81	17537.78711	17166.74	18811.26	18483.82	16997.62	17864.86133	0.9817	0.9108
Q8C3W1		Uncharacterized protein C1orf198 homolog	51678.88281	48122.23	49170.2	46419.93	48847.80957	48767.94	53017.2	50407.84	46847.5	49760.12109	0.9817	0.6126
E9PVA8	Gcn1	Stalled ribosome sensor GCN1	24818.6875	23864.47	24162.34	21165.51	23502.75098	25623.02	24086.45	23987.56	22071.83	23942.21387	0.9816	0.6993
P55012	Slc12a2	Solute carrier family 12 member 2	465418.5	454791.9	437781.2	421632.2	444905.9453	457787.9	472405.9	432743.9	450000.8	453234.6406	0.9816	0.5357
Q62108	Dlg4	Disks large homolog 4	46837.52344	44763.68	47041.87	52719.16	47840.55957	46809.68	49203.54	51879.54	47057.7	48737.6123	0.9816	0.6803
Q921G7	Etfdh	Electron transfer flavoprotein-ubiquinone oxidoreductase, mitochondrial	82559.46875	79425.83	81161.92	78235.63	80345.71289	84004.36	86311.77	82704.53	74425.36	81861.50391	0.9815	0.6024
Q7TN29	Smap2	Stromal membrane-associated protein 2	107871.1328	105276.8	96287.2	110955.2	105097.584	109743	110103	102251	106259.4	107089.0898	0.9814	0.6050
Q9CQ22	Lamtor1	Ragulator complex protein LAMTOR1	38682.63281	37664	40224.41	43866.87	40109.48047	45647.32	41996.12	39378.07	36472.83	40873.58398	0.9813	0.7588
A2ASZ8	Slc25a25	Mitochondrial adenyl nucleotide antiporter SLC25A25	111321.0547	95157.95	100652.1	101036.4	102041.8691	112788.4	111733.4	94815.55	96632.09	103992.3516	0.9812	0.7506
Q71RI9	Kyat3	Kynurenine--oxoglutarate transaminase 3	67047.46094	67942.55	62985.7	76183.61	68539.83203	73499.65	67350.89	67908.09	70650.23	69852.21484	0.9812	0.6874
Q9ER73	Elp4	Elongator complex protein 4	8272.987305	8406.784	5767.246	5032.438	6869.864014		9965.3	5805.752	5233.453	7001.501465	0.9812	0.9381
O35343	Kpna4	Importin subunit alpha-3	15085.0625	14323.84	11461.85	13003.27	13468.50732	12634.83	13009.89	15216.83	14044.93	13726.62183	0.9812	0.8018
Q9JHZ2	Ankh	Progressive ankylosis protein	7731.055176	7122.466	5546.431	7153.028	6888.244873	7830.381	8095.605	6095.993	6061.823	7020.950439	0.9811	0.8598
Q8BPQ7	Sgsm1	Small G protein signaling modulator 1	31242.41016	23718.68	21122.23	23379.56	24865.72217	27503.49	23604.91	24072.57	26200.62	25345.39502	0.9811	0.8472
P01899	H2-D1	H-2 class I histocompatibility antigen, D-B alpha chain	8215.697266	6688.151	8295.661	7546.447	7686.489136	6272.256	7266.957	10650.79	7149.542	7834.886475	0.9811	0.8906
Q3UJB9	Edc4	Enhancer of mRNA-decapping protein 4	12566.38281	11958.84	9895.617	11001.45	11355.57104	11915.21	12313.79	10163.8	11906.74	11574.88525	0.9811	0.7814
Q9CQC7	Ndufb4	NADH dehydrogenase [ubiquinone] 1 beta subcomplex subunit 4	407903.875	389216.2	398896.3	446206.1	410555.6172	431498.8	419541.7	424894.4	398067.4	418500.5781	0.9810	0.6017
P62715	Ppp2cb	Serine/threonine-protein phosphatase 2A catalytic subunit beta isoform	361840.4688	356016.3	347554.1	375431.8	360210.6641	387724	353805.5	354859	372345.4	367183.4766	0.9810	0.5103
Q9CQC9	Sar1b	GTP-binding protein SAR1b	172999.4375	196519.1	164174.2	169126.9	175704.9063	196825.1	186836.1	162186.1	170642	179122.3242	0.9809	0.7581
Q8K298	Anln	Anillin	32523.44336	30047.57	29763.05	34215.33	31637.34912	32139.45	29829.63	34445.36	32614.31	32257.18799	0.9808	0.6783
P00405	Mtco2	Cytochrome c oxidase subunit 2	2006526.75	2149077	2318960	2422151	2224178.625	2350719	2369909	2084138	2266367	2267783.531	0.9808	0.7120
Q80TN5	Zdhhc17	Palmitoyltransferase ZDHHC17	27316.59766	18077.45	6973.45	13023.42	16347.7301	19088.52	20596.3	4046.137	22944.15	16668.7749	0.9807	0.9595
Q8BTG7	Ndrg4	Protein NDRG4	144684.4063	159834.8	150852	137679.8	148262.7305	161630.5	150831.5	141198.7	151063.3	151181	0.9807	0.6589
P63038	Hspd1	60 kDa heat shock protein, mitochondrial	637202.6875	641028.8	647199.9	655543.1	645243.6094	675082.4	674683.2	645633.8	636778.2	658044.4063	0.9805	0.2754
Q91YM2	Arhgap35	Rho GTPase-activating protein 35	29224.73828	30234.44	24282.06	23557.81	26824.76123	26583.41	27211.66	27602.93	28031.71	27357.42871	0.9805	0.7678
Q62093	Srsf2	Serine/arginine-rich splicing factor 2	81898.22656	68513.79	82514.1	83213.51	79034.90625	78411.05	81816.13	84452.09	77740.04	80604.82422	0.9805	0.6975
Q920B9	Supt16h	FACT complex subunit SPT16	8149.154297	7538.559	8323.222	8747.563	8189.624268	8904.01	7937.335	7179.412	9389.316	8352.518311	0.9805	0.7786
Q9D1H8	Mrpl53	Large ribosomal subunit protein mL53	9190.043945	12787.42	12326.29	8461.019	10691.19287	9501.413	8476.053	15326.5	10311.55	10903.87939	0.9805	0.9133
Q9QWY8	Asap1	Arf-GAP with SH3 domain, ANK repeat and PH domain-containing protein 1	12276.51367	11914.73	11099.49	14035.73	12331.61475	12412.01	11696.76	14152.15	12049.15	12577.51807	0.9804	0.7756
P09411	Pgk1	Phosphoglycerate kinase 1	1472531	1507769	1435762	1482203	1474566.25	1503129	1480295	1415999	1616869	1504073.094	0.9804	0.5315
Q9CZ13	Uqcrc1	Cytochrome b-c1 complex subunit 1, mitochondrial	893759.625	933609.1	932256.2	1003229	940713.5469	976179.8	967646.6	978798.5	915674.6	959574.875	0.9803	0.5138
Q99J99	Mpst	3-mercaptopyruvate sulfurtransferase	33463.30859	35656.04	39927.59	39514.06	37140.25	37422.69	35002.09	41649.98	37467.65	37885.60547	0.9803	0.7326
Q8BHL8	Psmf1	Proteasome inhibitor PI31 subunit	34670.53906	39011.71	40142.38	41888.44	38928.26758	31360.77	38750.17	41739.35	46989.52	39709.9541	0.9803	0.8356
A2AGT5	Ckap5	Cytoskeleton-associated protein 5	111339.2891	112209.5	107701	111536.5	110696.5938	112588.6	112573.3	111521	114996	112919.7441	0.9803	0.1267
Q810U3	Nfasc	Neurofascin	392077.6563	362083.3	385361.5	403998.6	385880.2656	382760.9	384761.2	398560	408473.2	393638.8438	0.9803	0.4958
P52503	Ndufs6	NADH dehydrogenase [ubiquinone] iron-sulfur protein 6, mitochondrial	148557.4375	136901	142134.8	147210.5	143700.9219	147607.5	145901	141297	151586.4	146597.9883	0.9802	0.4274
Q9DCZ4	Apoo	MICOS complex subunit Mic26	299262.3125	283460.4	293414.6	291282.8	291855.0234	298734.6	288089.1	288138.3	316036.9	297749.7266	0.9802	0.4534
Q8BVE3	Atp6v1h	V-type proton ATPase subunit H	403309.0938	378811.5	382686.8	405913.2	392680.1484	407210.8	393699.9	388650.4	412918.5	400619.8828	0.9802	0.4103
P70188	Kifap3	Kinesin-associated protein 3	18539.25781	17165.02	17998.45	23116.2	19204.72949	20189.03	22226.37	20805.11	15153.52	19593.50732	0.9802	0.8550
P09103	P4hb	Protein disulfide-isomerase	173131.75	174937.7	171283.9	172927.5	173070.1992	192489.3	186525.9	159588.2	167705.1	176577.1094	0.9801	0.6680
P52760	Rida	2-iminobutanoate/2-iminopropanoate deaminase	717699.875	770895.3	748397.8	740748.8	744435.4531	711563.3	692905.2	858097.3	775566.9	759533.1563	0.9801	0.7113
Q91VK4	Itm2c	Integral membrane protein 2C	20128.23633	13165.1	17381.38	17264.63	16984.83667	16210.46	17858.13	17442.19	17807.5	17329.56787	0.9801	0.8242
Q5SUR0	Pfas	Phosphoribosylformylglycinamidine synthase	22824.25977	26582.01	28940.51	27907.44	26563.55566	22707.61	27684.52	30935.27	27093.87	27105.31787	0.9800	0.8100
O35874	Slc1a4	Neutral amino acid transporter A	62791.95703	59771.43	64395.63	58803.86	61440.71875	64705.93	64571.06	61999.75	59501.46	62694.54785	0.9800	0.5104
O35382	Exoc4	Exocyst complex component 4	57451.05859	54468.99	49773.34	50490.72	53046.02832	54378.78	57846.78	51707.66	52592.3	54131.38086	0.9799	0.6467
Q9CR68	Uqcrfs1	Cytochrome b-c1 complex subunit Rieske, mitochondrial	370726.5313	349089.8	381146.7	426457.8	381855.2109	394523.2	374218.1	397791.9	392224.1	389689.3281	0.9799	0.6636
P62077	Timm8b	Mitochondrial import inner membrane translocase subunit Tim8 B	20669.29688	16109.75	18415.05	16936.24	18032.58374	19962.86	21561.96	14019.73	18067.73	18403.06812	0.9799	0.8525
Q9DCB8	Isca2	Iron-sulfur cluster assembly 2 homolog, mitochondrial	41301.91797	38816.52	40957.26	51948.13	43255.95508	44295.88	40858.08	47831.98	43603.09	44147.25488	0.9798	0.7949
O55229	Chkb	Choline/ethanolamine kinase	29159.4668	30993.01	32489.48	32175.99	31204.48633	36079.69	33554.09	24990.55	32770.12	31848.61328	0.9798	0.8059
O08663	Metap2	Methionine aminopeptidase 2	37064.53906	39470.11	34019.56	30671.73	35306.48242	38231.14	35334.68	33290.85	37290.59	36036.8125	0.9797	0.7510
P62192	Psmc1	26S proteasome regulatory subunit 4	96521.38281	94470.31	92992.56	87398.09	92845.58789	92303.83	97508.58	94589.24	94680.9	94770.63672	0.9797	0.4204
Q91VN4	Chchd6	MICOS complex subunit Mic25	20015.60938	20677.78	18475.49	22008.39	20294.31836	19825.33	20728.81	21700.04	20614.3	20717.12109	0.9796	0.6282
P51863	Atp6v0d1	V-type proton ATPase subunit d 1	677629.8125	637118.6	635007.4	675077.6	656208.3438	660480.8	663015.6	672794.8	683498.5	669947.4063	0.9795	0.3235
Q9QYG0	Ndrg2	Protein NDRG2	1145936.125	1155196	961659	964308.7	1056774.828	1106469	1112411	1028918	1067865	1078915.609	0.9795	0.7137
Q5DU25	Iqsec2	IQ motif and SEC7 domain-containing protein 2	11185.9834	12048.14	12095.97	9924.616	11313.67603	10752.29	10971.94	12734.73	11746.78	11551.43335	0.9794	0.7377
O35604	Npc1	NPC intracellular cholesterol transporter 1	15480.47461	16050.35	18513.37	17245.75	16822.48657	17083.97	16952.11	16315.56	18354.59	17176.55762	0.9794	0.6725
Q9JIA1	Lgi1	Leucine-rich glioma-inactivated protein 1	321354.9375	307062.9	301551.1	345419.5	318847.0938	331740.1	326836.5	323714.3	320088	325594.7266	0.9793	0.5288
Q9CQI6	Cotl1	Coactosin-like protein	151775.2188	182769.4	194077.7	157398.7	171505.2539	175202.7	177866.9	172007.7	175485.7	175140.7578	0.9792	0.7331
Q8C0C7	Farsa	Phenylalanine--tRNA ligase alpha subunit	85176.14063	88042.73	79015.78	82495.84	83682.62305	93691.82	82949.76	71944.76	93241.93	85457.06641	0.9792	0.7575
Q8BG73	Sh3bgrl2	SH3 domain-binding glutamic acid-rich-like protein 2	97283.48438	110705.5	104149.3	100720.6	103214.7383	102903.5	104569.5	115196.6	98952.46	105405.5059	0.9792	0.6435
Q8C167	Prepl	Prolyl endopeptidase-like	33204.21484	35582.16	35499.79	36970.98	35314.28516	35679.8	34423.89	35725.36	38436.29	36066.33594	0.9791	0.5375

Q8BGU5	Ccny	Cyclin-Y	23784.43359	24572.3	27445.16	22607.71	**24602.40088**	26866.28	27173.6	24921.71	21547.9	**25127.37012**	0.9791	0.7616
Q99020	Hnrnpab	Heterogeneous nuclear ribonucleoprotein A/B	47783.98438	40358.01	45286.18	39541.3	**43242.36816**	45351.51	48172.48	44224.31	38922.14	**44167.6084**	0.9791	0.7493
Q9WV95	Phlda3	Pleckstrin homology-like domain family A member 3	11033.77441	10223.97	7297.384	6461.461	**8754.147705**	9804.943	11064.7	6787.233	8109.225	**8941.524658**	0.9790	0.9016
O35678	Mgll	Monoglyceride lipase	66045.73438	62089.4	45616.99	54214.59	**56991.68066**	66017.74	63885.75	50883.68	52069.19	**58214.08984**	0.9790	0.8449
Q9CRC8	Lrrc40	Leucine-rich repeat-containing protein 40	21415.46875	20314.37	17898.78	19511.82	**19785.11035**	21342.13	19604.31	21355.49	18537.55	**20209.87207**	0.9790	0.6898
P61924	Copz1	Coatomer subunit zeta-1	94810.6875	85958.63	85116.47	91483.57	**89342.33984**	97225.2	101362.3	87087.58	79370.44	**91261.36719**	0.9790	0.7381
P63248	Pkia	cAMP-dependent protein kinase inhibitor alpha	16522.02539	27238.79	20572.26	24966.45	**22324.88379**	33806.83	17268.96	23378.7	16765	**22804.87158**	0.9790	0.9207
Q99KB8	Hagh	Hydroxyacylglutathione hydrolase, mitochondrial	34965.31641	41905.23	37943.43	36504.34	**37829.57813**	34142.28	38356.39	40701.48	41377.13	**38644.32324**	0.9789	0.7251
Q9CPQ8	Atp5mg	ATP synthase subunit g, mitochondrial	1215041.5	1207028	1288886	1380215	**1272792.656**	1330560	1265503	1320271	1285022	**1300338.938**	0.9788	0.5457
Q8R0S4	Cacnb4	Voltage-dependent L-type calcium channel subunit beta-4	25698.78516	27074.19	24742.84	26082.4	**25899.55469**	26186.9	24973.08	27653.03	27027.37	**26460.09424**	0.9788	0.4853
Q6PIC6	Atp1a3	Sodium/potassium-transporting ATPase subunit alpha-3	4746635.5	5030921	5057704	5008576	**4960959.125**	5141578	5115103	4881835	5135458	**5068493.25**	0.9788	0.3029
Q9Z2E9	Bscl2	Seipin		10847.58	9593.214		**10220.39453**	10311.33	10026.39	12144.39	9286.018	**10442.03076**	0.9788	0.8336
P55096	Abcd3	ATP-binding cassette sub-family D member 3	34141.97656	40330.05	37742.3	40323.95	**38134.57227**	39720.69	38432.83	38731.87	38961.65	**38961.76074**	0.9788	0.5987
P67871	Csnk2b	Casein kinase II subunit beta	37993.38672	28002.81	37106.15	34685.97	**34447.07861**	29283.71	38756.68	34388.27	38352.38	**35195.2583**	0.9787	0.8205
Q9CZX0	Elp3	Elongator complex protein 3	8620.03418	11025.24	10975.83	9388.046	**10002.28662**	9404.365	10883.34	11044.21	9547.075	**10219.74927**	0.9787	0.7779
Q8BXC6	Commd2	COMM domain-containing protein 2	22211.33789	29385.04	27538	25140.06	**26068.60742**	24959.74	26237.76	26864.58	28483.12	**26636.2998**	0.9787	0.7520
P34914	Ephx2	Bifunctional epoxide hydrolase 2	26898.72656	25560.25	22887.24	21686.96	**24258.29248**	24006.3	23426.76	24459.57	27258.9	**24787.88379**	0.9786	0.7305
Q6Q477	Atp2b4	Plasma membrane calcium-transporting ATPase 4	114734.2578	116690.5	111099.8	136829.4	**119838.4941**	127790.6	122700.8	122266.3	117090.2	**122461.9785**	0.9786	0.6860
Q80U56	Avl9	Late secretory pathway protein AVL9 homolog	24905.53711	25588.98	26778.39	29488.16	**26690.26709**	27727.32	26252.11	28108.19	27011.31	**27274.7334**	0.9786	0.6110
Q9DCJ5	Ndufa8	NADH dehydrogenase [ubiquinone] 1 alpha subcomplex subunit 8	427833.5938	400302.3	412708.1	466920.1	**426941.0234**	448411.3	429271.9	420991.6	446609.4	**436321.0469**	0.9785	0.5776
Q80XN0	Bdh1	D-beta-hydroxybutyrate dehydrogenase, mitochondrial	391254.9375	420585.8	331365.2	335801.8	**369751.9375**	419577.8	430805.5	325723.6	335614.4	**377930.3047**	0.9784	0.8231
Q922H2	Pdk3	[Pyruvate dehydrogenase (acetyl-transferring)] kinase isozyme 3, mitochon	69878.07813	73350.84	88288.52	72033.56	**75887.75**	68097.66	75206.92	92596.26	74366.6	**77566.86133**	0.9784	0.8111
Q6PHN9	Rab35	Ras-related protein Rab-35	54668.97656	45074.66	61866.44	62497	**56026.76855**	64034.54	59281.26	58796.68	46969.95	**57270.60547**	0.9783	0.8269
P56135	Atp5mf	ATP synthase subunit f, mitochondrial	2246009	2258071	2431837	2556043	**2372989.938**	2493709	2307716	2598392	2302982	**2425699.438**	0.9783	0.6303
Q8VD62	Bles03	UPF0696 protein C11orf68 homolog	45395.03516	51239.05	41033.59	44428.29	**45523.99023**	45540.85	47459.91	44303.16	48837.19	**46535.27441**	0.9783	0.6818
Q91XV3	Basp1	Brain acid soluble protein 1	195254.8438	161317.6	184256.5	206494.4	**186830.832**	193406.9	180756.3	190719.1	199055.6	**190984.4609**	0.9783	0.7027
P28663	Napb	Beta-soluble NSF attachment protein	328640.3438	299086.2	330256.1	292329.6	**312578.0703**	315925.2	320929	308872.6	332388.4	**319528.7891**	0.9782	0.5513
Q922F4	Tubb6	Tubulin beta-6 chain	3109120	3483930	3360791	3457241	**3352770.688**	3240934	3359566	3727033	3382007	**3427384.875**	0.9782	0.6005
Q6A058	Armcx2	Armadillo repeat-containing X-linked protein 2	4703.681152	2946.121		5265.306	**4305.036051**	3873.644	4440.291	6454.449	2835.362	**4400.936279**	0.9782	0.9323
Q99JY0	Hadhb	Trifunctional enzyme subunit beta, mitochondrial	793889.375	757696.2	875618.6	827741.8	**813736.5**	860087.3	871216.8	785905.5	810285.1	**831873.6406**	0.9782	0.5943
Q9Z0R6	Itsn2	Intersectin-2	19339.66602	16273.32	19463.32	18450.87	**18381.79443**	18578.92	21109	16852.73	18627.15	**18791.95068**	0.9782	0.7325
Q8CI71	Vps50	Syndetin	16174.30371	14357.04	15228.86	12811.98	**14643.04736**	17628.81	14940	13209.05	14101.69	**14969.88965**	0.9782	0.7931
O35737	Hnrnph1	Heterogeneous nuclear ribonucleoprotein H	186488.6094	170536.1	177238.3	188903.9	**180791.7344**	187360.1	203307.6	177535.1	171141.4	**184836.0547**	0.9781	0.6389
Q8K1A6	Cc2d1a	Coiled-coil and C2 domain-containing protein 1A	18894.22461	17130.48	18355.9	16739.16	**17779.94141**	18702.93	18117.24	18269.68	17624.72	**18178.64111**	0.9781	0.4982
Q8K0V4	Cnot3	CCR4-NOT transcription complex subunit 3	4431.317383	3289.745		5782.598	**4501.220215**	3863.367	3700.56	4467.246	6378.911	**4602.520752**	0.9780	0.9188
B0V2N1	Ptprs	Receptor-type tyrosine-protein phosphatase S	11710.95117	8587.872	8909.639	10884.3	**10023.19067**	10685.69	10253.18	8956.247	11100.98	**10249.02271**	0.9780	0.8079
Q9ERE7	Mesd	LRP chaperone MESD	16054.05078	15288.83	22357.2	17011.79	**17677.96899**	18689.17	18649.15	13748.72	21219.37	**18076.60229**	0.9779	0.8644
Q99KJ8	Dctn2	Dynactin subunit 2	277769.375	298934.7	279327.4	277150.2	**283295.3984**	296003.8	303730.2	283908.6	275103	**289686.3984**	0.9779	0.4667
P46460	Nsf	Vesicle-fusing ATPase	1139914.375	1138478	1070214	1224483	**1143272.375**	1176013	1177307	1147607	1175528	**1169113.5**	0.9779	0.4553
P63046	Sult4a1	Sulfotransferase 4A1	85608.53906	77467.08	78485.13	79721.44	**80320.54688**	79888.22	83969.04	81894.85	82805.43	**82139.38477**	0.9779	0.4016
P70362	Ufd1	Ubiquitin recognition factor in ER-associated degradation protein 1	55596.04688	54492.34	61865.44	61063.96	**58254.44824**	56206.36	61442.4	61288.07	59358.16	**59573.74707**	0.9779	0.5766
Q01065	Pde1b	Dual specificity calcium/calmodulin-dependent 3',5'-cyclic nucleotide pho	52639.79297	53584.63	51995.78	51982.8	**52550.75**	49403.89	54622.36	55513.79	55435.21	**53743.81348**	0.9778	0.4591
P70202	Lxn	Latexin	340164.25	369304.9	444080.1	364387.4	**379484.1563**	324140.7	370949.4	445033	412332.6	**388113.9375**	0.9778	0.8107
P70295	Aup1	Lipid droplet-regulating VLDL assembly factor AUP1	7768.980469	7248.276	10366.14	5681.386	**7766.196411**	7472.689	7500.521	9761.63	7036.63	**7942.867676**	0.9778	0.8831
Q9Z2I8	Suclg2	Succinate--CoA ligase [GDP-forming] subunit beta, mitochondrial	47520.11328	47086.72	46593.64	48737.61	**47484.52051**	46661.99	49717.67	48032.35	49852.84	**48566.21191**	0.9777	0.2678
Q8C1Y8	Ccz1	Vacuolar fusion protein CCZ1 homolog	40356.94531	35738.77	46826.94	26375.13	**37324.44531**	38530.96	41090.62	21483.92	51602.97	**38177.11621**	0.9777	0.9141
P57784	Snrpa1	U2 small nuclear ribonucleoprotein A'	28635.3457	28186.63	26141.66	30730.1	**28423.43164**	28839.71	29857.13	33070.82	24524.94	**29073.14648**	0.9777	0.7562
Q8K448	Abca5	Cholesterol transporter ABCA5	27935.39453	28694.83	17790.82	18380.14	**23200.26953**	28493.2	23503.6	20857.56	22068.29	**23730.66357**	0.9776	0.8812
Q9Z0R4	Itsn1	Intersectin-1	24260.78711	21105.43	21947.2	23280.45	**22648.4668**	22862.18	22392.85	25041.58	22371.8	**23167.1001**	0.9776	0.6028
P20934	Evi2a	Protein EVI2A	21124.55469	15277.71	19187.67	16163.14	**17938.26685**	18149.23	15821.61	21211.79	18214.29	**18349.2312**	0.9776	0.8218
Q3UGX3	Nat8l	N-acetylaspartate synthetase	45854.61719	44875.85	45534.97	45529.22	**45448.66406**	47689.66	44913.64	48176.66	45181.91	**46490.46777**	0.9776	0.2740
Q8BMB3	Eif4e2	Eukaryotic translation initiation factor 4E type 2	13378.89063	14047.87	15699.04	13102.13	**14056.98242**	14552.89	15585.51	13024.07	14359.22	**14380.42432**	0.9775	0.6945
P53810	Pitpna	Phosphatidylinositol transfer protein alpha isoform	134851.1875	132959.2	139798.6	132028	**134909.2461**	137212.6	130306.3	141381.3	143178.8	**138019.7539**	0.9775	0.3880
Q8C437	Pex5l	PEX5-related protein	118698.4609	130523.5	111481.3	111751.7	**118113.75**	129176.5	128565	113767.9	111839.4	**120837.1953**	0.9775	0.6875
E9Q8I9	Fry	Protein furry homolog	10801.39648	11941.24	9400.953	10091.4	**10558.74707**	10124.74	11074.94	11620.63	10391.58	**10802.97485**	0.9774	0.7155
Q08890	Ids	Iduronate 2-sulfatase				5552.357	**5552.357422**		3872.613		7489.232	**5680.922607**	0.9774	
Q6A065	Cep170	Centrosomal protein of 170 kDa	34901.28125	35864.37	31357.78	34572.33	**34173.94043**	36320.89	38149.16	30789.11	34603.95	**34965.77783**	0.9774	0.6834
Q6PDM2	Srsf1	Serine/arginine-rich splicing factor 1	117821.0156	107733.5	117908.9	111191.1	**113663.6289**	113870.5	121338.7	121321.6	108671.6	**116300.6035**	0.9773	0.5335
P48318	Gad1	Glutamate decarboxylase 1	47750.13281	69399.8	64353.66	66813.73	**62079.3291**	70644.12	64546.66	61884.78	57010.16	**63521.42969**	0.9773	0.8071
Q8BTI8	Srrm2	Serine/arginine repetitive matrix protein 2	21149.29883	15678.51	18386.74	20383.84	**18899.59741**	21165.03	20203.5	19873.52	16112.7	**19338.69019**	0.9773	0.7991
Q1RLL3	Cpne9	Copine-9	38128.52344	45400.71	36909.66	30253.87	**37673.19141**	44499.7	60379.47	33362.64	15957.66	**38549.8667**	0.9773	0.9320
O88809	Dcx	Neuronal migration protein doublecortin	42376.91016	39058.39	37239.13	38005.14	**39169.89258**	41717.58	37908.52	38949.35	41755.75	**40082.79883**	0.9772	0.5642
P32883	Kras	GTPase KRas	41710.43359	40216.07	41639.81	40029.53	**40898.96094**	46560.91	47487.89	36174.82	37187.33	**41852.73633**	0.9772	0.7638
Q9CZC8	Scrn1	Secernin-1	112411.3203	126742.6	132567.9	116464.7	**122046.625**	117863.2	116385.5	126207.3	139139.8	**124898.9648**	0.9772	0.6966
P53994	Rab2a	Ras-related protein Rab-2A	314363.5938	342432.1	313459.7	303478.5	**318433.4766**	342366.8	320917.3	318112	322180.4	**325894.1406**	0.9771	0.4858

Q9CQ60	Pgls	6-phosphogluconolactonase	88403.00781	87667.76	91321.83	80496.8	**86972.34961**	87598.5	83708.2	92491.57	92248.1	**89011.5918**	**0.9771**	**0.5364**
O35593	Psmd14	26S proteasome non-ATPase regulatory subunit 14	116645.6172	111665.2	97622.74	110475.7	**109102.3047**	113209.6	109284.5	111922.1	112232.6	**111662.2207**	**0.9771**	**0.5590**
Q80UG2	Plxna4	Plexin-A4	38966.35938	40950.07	41605.76	46237.32	**41939.87988**	41275.54	42453.8	46248.04	41723.42	**42925.19922**	**0.9770**	**0.6246**
Q9QYC0	Add1	Alpha-adducin	261263.4063	238423.7	264784.7	233567.7	**249509.8672**	247610.8	235053.6	268260.9	270565.5	**255372.6797**	**0.9770**	**0.6317**
Q91VR7	Map1lc3a	Microtubule-associated proteins 1A/1B light chain 3A	624103.1875	620234.4	640143.6	618634.8	**625779**	690456.1	620677.7	619429.3	631387.3	**640487.5938**	**0.9770**	**0.4347**
P35293	Rab18	Ras-related protein Rab-18	263703.1563	279148.8	245578.8	237608.7	**256509.8555**	285616.6	263764.3	256257.2	244562.9	**262550.2773**	**0.9770**	**0.6513**
Q9Z2H5	Epb41l1	Band 4.1-like protein 1	174439.9844	176296.8	161016.2	190778.2	**175632.8008**	187556.2	188358.9	163235.4	179936.3	**179771.6992**	**0.9770**	**0.6409**
Q8QZS1	Hibch	3-hydroxyisobutyryl-CoA hydrolase, mitochondrial	89235.59375	94158.3	83419.49	92858.77	**89918.04102**	91908.63	88040.35	94573.86	93628.19	**92037.75586**	**0.9770**	**0.4781**
Q6ZWN5	Rps9	Small ribosomal subunit protein uS4	307301.7188	308745.6	288603.3	277084.8	**295433.875**	326870.7	318508	278569.8	285673.1	**302405.3828**	**0.9769**	**0.6401**
P70296	Pebp1	Phosphatidylethanolamine-binding protein 1	890696.125	910540.7	907544.4	875816.2	**896149.3438**	852155.6	868400.7	965053.4	983619	**917307.1719**	**0.9769**	**0.5597**
P63073	Eif4e	Eukaryotic translation initiation factor 4E	36799.32031	32307.79	36209.88	34122.36	**34859.83887**	31541.32	32586.61	38743.96	39861.3	**35683.30029**	**0.9769**	**0.7379**
Q03517	Scg2	Secretogranin-2	146886.6094	149244.9	149513	169424.4	**153767.2422**	157911.7	159219	160109.3	152412.2	**157413.0586**	**0.9768**	**0.5341**
P06745	Gpi	Glucose-6-phosphate isomerase	576195.875	646362.3	634877.3	645042.1	**625619.3594**	636295.1	602392	647429.1	675824.3	**640485.1094**	**0.9768**	**0.5342**
Q810B6	Ankfy1	Rabankyrin-5	30539.95117	30916.73	30916.21	26901.5	**29818.59766**	33137.87	34055.57	27414.78	27506.2	**30528.60449**	**0.9767**	**0.7386**
P62812	Gabra1	Gamma-aminobutyric acid receptor subunit alpha-1	10026.85547	10748.97	6681.959	11455.44	**9728.307495**	8369.267	12297.8	9073.357	10099.51	**9959.981934**	**0.9767**	**0.8703**
Q80ZJ1	Rap2a	Ras-related protein Rap-2a	65091.87109	58766.61	68721.64	58358.59	**62734.67773**	68163.99	60335.98	65511.89	62926.8	**64234.66504**	**0.9766**	**0.6383**
Q8C2Q3	Rbm14	RNA-binding protein 14	21433.62891	23057.72	18732.04	23033.23	**21564.1543**	23685.57	24232.12	20808.8	19597.08	**22080.89209**	**0.9766**	**0.7441**
Q8R307	Vps18	Vacuolar protein sorting-associated protein 18 homolog	22838.29688	24952.84	24670.34	23921.7	**24095.79248**	27180.57	19735.31	22864.45	28915.1	**24673.85693**	**0.9766**	**0.7955**
Q91Z53	Grhpr	Glyoxylate reductase/hydroxypyruvate reductase	220159.2656	250798.2	234397	241371	**236681.375**	250807.7	238218	247622	232912.2	**242389.9531**	**0.9764**	**0.4845**
O70435	Psma3	Proteasome subunit alpha type-3	86853.21875	89899.17	86107.52	94905.1	**89441.25195**	93213.94	96994.06	83959.97	92230.37	**91599.58398**	**0.9764**	**0.5485**
Q9ERS2	Ndufa13	NADH dehydrogenase [ubiquinone] 1 alpha subcomplex subunit 13	861451.1875	823328.8	823250.1	830954.2	**834746.0781**	894629.6	884456.9	813322.3	827387.6	**854949.0781**	**0.9764**	**0.3984**
Q3U487	Hectd3	E3 ubiquitin-protein ligase HECTD3	21734.21094	20028.99	20154.81	20322.19	**20560.04932**	20320.75	21775.97	19933.41	22202.3	**21058.11035**	**0.9763**	**0.4902**
Q80YA7	Dpp8	Dipeptidyl peptidase 8	14581.88672	11902.98	14063.3	16535.29	**14270.86328**	14168	12542.47	17705.81	14056.21	**14618.11987**	**0.9762**	**0.8187**
Q8VIM9	Irgq	Immunity-related GTPase family Q protein	60547.41797	52021.75	60299.16	61420.93	**58572.31348**	58282.63	57623.67	60078.8	64010.81	**59998.97656**	**0.9762**	**0.6062**
Q8BFS6	Cpped1	Serine/threonine-protein phosphatase CPPED1	29838.80859	23733.2	27553.13	30167.51	**27823.16162**	26239.13	26008.13	31286.46	30470.82	**28501.13281**	**0.9762**	**0.7495**
Q9D0R2	Tars1	Threonine--tRNA ligase 1, cytoplasmic	64615.75	68933.34	60693.21	62007.07	**64062.34277**	63220.36	67242.55	68179.9	63878.28	**65630.27246**	**0.9761**	**0.5010**
P61164	Actr1a	Alpha-centractin	276449.9375	272301.3	246349	287775.2	**270718.8555**	283747.9	276545.2	283446.8	265649.5	**277347.3438**	**0.9761**	**0.5211**
Q9D6D0	Slc25a27	Mitochondrial uncoupling protein 4	17116.01367	19350.63	15763.5	19526.01	**17939.03662**	15854.4	20776.38	18296.75	18587.85	**18378.84521**	**0.9761**	**0.7568**
P20352	F3	Tissue factor	29055.70898	23323.98	24815.84	25700.48	**25724.00049**	29701.62	27081.17	27372.85	21269.1	**26356.18604**	**0.9760**	**0.7802**
P62315	Snrpd1	Small nuclear ribonucleoprotein Sm D1	77554.76563	86415.98	71919.3	67180.48	**75767.63281**	82003.19	79536.42	63984.19	85007.31	**77632.77832**	**0.9760**	**0.7754**
P23492	Pnp	Purine nucleoside phosphorylase	82224.17969	89479.57	79572.98	78355.16	**82407.97266**	87139.58	85316.59	80152.5	85160	**84442.16602**	**0.9759**	**0.5104**
P09671	Sod2	Superoxide dismutase [Mn], mitochondrial	811912.625	829495.9	767710.8	812151.9	**805317.7969**	884297.1	865212.8	750214.6	801727.3	**825362.9219**	**0.9757**	**0.5699**
Q8R5M8	Cadm1	Cell adhesion molecule 1	47377.70703	43180.69	45597.98	40485.14	**44160.37988**	44464.41	46237.92	38295.42	52044.46	**45260.55371**	**0.9757**	**0.7428**
O88845	Akap10	A-kinase anchor protein 10, mitochondrial	14851.25977	12271.36	13533.66	12183.14	**13209.85449**	9395.579	13464.47	16783.81	14512.33	**13539.04736**	**0.9757**	**0.8501**
P17182	Eno1	Alpha-enolase	1771590.125	1787536	1793929	1925096	**1819537.656**	1765955	1813649	1923655	1956389	**1864911.844**	**0.9757**	**0.4584**
Q05920	Pc	Pyruvate carboxylase, mitochondrial	285310	278616.8	284991.9	299120	**287009.6875**	307127.5	286241.6	294063.5	289284	**294179.1328**	**0.9756**	**0.2996**
Q80VC9	Camsap3	Calmodulin-regulated spectrin-associated protein 3	17415.33398	16240.69	17928.45	18441.07	**17506.38672**	19307.38	18371.16	17465.91	16632.66	**17944.27881**	**0.9756**	**0.5779**
Q9CQN1	Trap1	Heat shock protein 75 kDa, mitochondrial	74966.28906	70548.39	63211.44	70436.34	**69790.61621**	77022.92	69817.14	69757.16	69551.41	**71537.1582**	**0.9756**	**0.5870**
Q9D819	Ppa1	Inorganic pyrophosphatase	162694.4219	168097.6	145627.5	155168.3	**157896.9414**	162896.1	157887.3	158993.1	167669.7	**161861.5352**	**0.9755**	**0.4869**
Q8BMG7	Rab3gap2	Rab3 GTPase-activating protein non-catalytic subunit	54834.10156	50987.14	54794.22	57352.38	**54491.96094**	49914.52	58943.62	58950.3	55634.14	**55860.64551**	**0.9755**	**0.6041**
P62334	Psmc6	26S proteasome regulatory subunit 10B	53536.69141	53429.9	48740.91	56912.23	**53154.93359**	54256.81	57916.69	52678.85	53115.21	**54491.8916**	**0.9755**	**0.5398**
Q07235	Serpine2	Glia-derived nexin	42915.90625	40335.73	45416.12	40606.46	**42318.55469**	39319.13	43092.7	43845.34	47275.16	**43383.08203**	**0.9755**	**0.6164**
Q8K215	Lyrm4	LYR motif-containing protein 4	51903.27344	46089	48558.28	55210.95	**50440.375**	55588.53	49540.26	50034.1	51678.71	**51710.40137**	**0.9754**	**0.6177**
Q9WVQ1	Magi2	Membrane-associated guanylate kinase, WW and PDZ domain-containing p	19448.98242	20408.74	17145.45	19970.75	**19243.47949**	22534.09	18186.3	17396.15	20798.54	**19728.76904**	**0.9754**	**0.7388**
Q9Z2H2	Rgs6	Regulator of G-protein signaling 6	20664.07813	24706.67	31984.21	21589.15	**24736.02588**	26898.67	29945.37	22010.64	22596.48	**25362.78955**	**0.9753**	**0.8502**
P08003	Pdia4	Protein disulfide-isomerase A4	52176.29297	43491.48	48695.14	42926.27	**46822.29688**	46664.38	53183.48	43929.84	48258.87	**48009.13965**	**0.9753**	**0.7004**
Q3TY86	Aifm3	Apoptosis-inducing factor 3	20778.72461	21809.07	25520.1	24513.36	**23155.31445**	20812.51	31791.39	23686.65	18682.5	**23743.26514**	**0.9752**	**0.8549**
P42337	Pik3ca	Phosphatidylinositol 4,5-bisphosphate 3-kinase catalytic subunit alpha isof	10432.46484	10917		10270.02	**10539.8252**	11565.7	10066.65	11401.48	10197.29	**10807.77808**	**0.9752**	**0.6095**
Q924S8	Spred1	Sprouty-related, EVH1 domain-containing protein 1	18227.17773	13711.82	19726.74	15502.74	**16792.12036**	19169.69	21305.89	14043.63	14360.13	**17219.83496**	**0.9752**	**0.8553**
P19783	Cox4i1	Cytochrome c oxidase subunit 4 isoform 1, mitochondrial	1362520.625	1318523	1372975	1373116	**1356783.781**	1340862	1368623	1418813	1437154	**1391363.031**	**0.9751**	**0.2275**
Q64514	Tpp2	Tripeptidyl-peptidase 2	147916.9688	144993.3	138172.5	147996.4	**144769.7773**	150524.5	143004.6	147127.4	153201.6	**148464.5273**	**0.9751**	**0.2909**
P58059	Mrps21	Small ribosomal subunit protein bS21m	13035.59961	12897.6	14140.67	12341.68	**13103.88818**	11955.53	12910.13	14757.1	14139.3	**13440.5144**	**0.9750**	**0.6613**
P99027	Rplp2	Large ribosomal subunit protein P2	108236.6641	109864.3	109212.7	97839.16	**106288.2109**	118775.3	109137.4	107957.9	100280.1	**109037.6973**	**0.9748**	**0.5827**
P34022	Ranbp1	Ran-specific GTPase-activating protein	98050.58594	100688.3	83906.63	102889	**96383.62891**	91153.64	106409.6	98748.95	99209.44	**98880.41016**	**0.9747**	**0.6536**
Q61656	Ddx5	Probable ATP-dependent RNA helicase DDX5	96826.48438	98122.66	100971.4	102484.6	**99601.2832**	102696.9	101906.1	105287.3	98838	**102182.0527**	**0.9747**	**0.2133**
Q9D0I9	Rars1	Arginine--tRNA ligase, cytoplasmic	114544.3281	124927	108982.7	111317.6	**114942.8809**	125081.6	127363.1	105812.7	113428.8	**117921.5898**	**0.9747**	**0.6460**
P51791	Clcn3	H(+)/Cl(-) exchange transporter 3	6698.100098	7082.701	6846.53	6556.457	**6795.947021**	7429.771	3482.275	10286.79	6690.096	**6972.234253**	**0.9747**	**0.9041**
O88848	Arl6	ADP-ribosylation factor-like protein 6	17349.73828	17883.97	15925.5	16928.7	**17021.97925**	17652.77	15732.67	17437.73	19032.01	**17463.79492**	**0.9747**	**0.5978**
Q9CPU0	Glo1	Lactoylglutathione lyase	375878.5	390639.6	333920.5	350598	**362759.1328**	365796	376665.4	350175.3	396386.3	**372255.75**	**0.9745**	**0.5738**
Q9D032	Ssbp3	Single-stranded DNA-binding protein 3	4343.850098				**4343.850098**		4457.609			**4457.608887**	**0.9745**	
Q6GQT5	Tmem151a	Transmembrane protein 151A	13388.20996	11415.32	11535.07	8694.818	**11258.35474**	13997.77	10100.85	11785.59	10330.61	**11553.70532**	**0.9744**	**0.8301**
P46097	Syt2	Synaptotagmin-2	773882.375	784648.1	730209.2	757477.8	**761554.3594**	850631.9	769588.8	745756.4	760181.8	**781539.7031**	**0.9744**	**0.4770**
Q8BJI1	Slc6a17	Sodium-dependent neutral amino acid transporter SLC6A17	30640.12695	30275.32	30524.39	34296.74	**31434.14453**	30402.28	33417.2	34277.05	30946.44	**32260.74121**	**0.9744**	**0.5603**
P51855	Gss	Glutathione synthetase	59450.49219	68760.17	58752.81	64591.05	**62888.63281**	65887.49	63586.73	65653.31	63042.11	**64542.41113**	**0.9744**	**0.5261**
Q9ESW4	Agk	Acylglycerol kinase, mitochondrial	179902.6719	178452.1	158575.1	160148.7	**169269.6523**	176208.3	186634.6	166837.8	165209.2	**173722.4844**	**0.9744**	**0.5779**

Q9Z1M8	Ik	Protein Red	8676.036133	10593.31	9843.313	8340.087	**9363.185547**	11010.39	9763.339	9065.66	8606.756	**9611.536377**	**0.9742**	**0.7482**
Q921E2	Rab31	Ras-related protein Rab-31	33266.54688	29173.96	30593.04	33523.76	**31639.32617**	35200.63	33654.77	29307.48	31751.28	**32478.53955**	**0.9742**	**0.6296**
Q0KL02	Trio	Triple functional domain protein	15085.01855	14190.94	15024.22	12973.71	**14318.47266**	14249.62	14267.92	14343.06	15944.35	**14701.23755**	**0.9740**	**0.5739**
Q9Z0G0	Gipc1	PDZ domain-containing protein GIPC1	7745.293457	5728.18	8188.509	6171.561	**6958.385986**	5662.292	6140.845	8878.592	7896.208	**7144.484131**	**0.9740**	**0.8526**
Q9CY64	Blvra	Biliverdin reductase A	171672.6719	186230.6	159794.1	166842	**171134.8555**	169914.5	172865.9	176556	183511.2	**175711.8828**	**0.9740**	**0.4958**
P63080	Gabrb3	Gamma-aminobutyric acid receptor subunit beta-3	30597.08398	28315.12	26702.88	32375.54	**29497.65479**	30879.9	29873.55	31325.2	29069.5	**30287.03857**	**0.9739**	**0.5793**
Q8BFP9	Pdk1	[Pyruvate dehydrogenase (acetyl-transferring)] kinase isozyme 1, mitochon	19059.1543	20443.13	20128.76	18690.88	**19580.48096**	21456.78	22611.3	17247.06	19104.65	**20104.94727**	**0.9739**	**0.6942**
O35927	Ctnnd2	Catenin delta-2	31289.98633	33190.95	29051.38	33057.05	**31647.34082**	29899.1	31608.44	35034.03	33439.26	**32495.20654**	**0.9739**	**0.5862**
Q91ZU6	Dst	Dystonin	45433.87891	41790.17	42630.47	43546.06	**43350.14453**	46870.59	44899.1	43642.82	42654.94	**44516.86133**	**0.9738**	**0.3681**
P31786	Dbi	Acyl-CoA-binding protein	573542.375	549170.8	559400.6	580164.7	**565569.625**	516910.1	527540.6	606520.4	672417.6	**580847.1484**	**0.9737**	**0.6951**
Q9WVJ2	Psmd13	26S proteasome non-ATPase regulatory subunit 13	75566.97656	83703.04	75421.6	78102.2	**78198.45508**	82247.91	85274.93	79053.98	74669.85	**80311.66992**	**0.9737**	**0.5052**
P35282	Rab21	Ras-related protein Rab-21	110595.9375	110253.2	104168.6	101829.7	**106711.8574**	115130.1	106174	106295.9	110807.9	**109601.9492**	**0.9736**	**0.3819**
Q5SSH7	Zzef1	Zinc finger ZZ-type and EF-hand domain-containing protein 1	14252.41699	12727.72	9244.74	11678.94	**11975.9541**	12286.52	10920.7	10241.93	15753.18	**12300.58179**	**0.9736**	**0.8475**
Q80X80	C2cd2l	Phospholipid transfer protein C2CD2L	24999.07227	23339.44	21821.97	16574.96	**21683.85986**	20963.3	23669.48	24279.79	20174.41	**22271.74365**	**0.9736**	**0.7870**
Q03141	Mark3	MAP/microtubule affinity-regulating kinase 3	27575.60352	26819.16	26407.58	28515.42	**27329.44141**	28789.44	29822.44	26922.95	26747.89	**28070.67969**	**0.9736**	**0.4304**
Q8K3G9	Appl2	DCC-interacting protein 13-beta	10496.52148	11308.27	11680.01	11375.37	**11215.04248**	11343.19	11662.36	11191.51	11883.1	**11520.03931**	**0.9735**	**0.3440**
Q8CCM6	Timm21	Mitochondrial import inner membrane translocase subunit Tim21	14348.50391	15468.69	15412.16	12705.56	**14483.72754**	16887.92	16596.72	14091.23	11937.44	**14878.32837**	**0.9735**	**0.7769**
Q9ERF3	Skic8	Superkiller complex protein 8	3209.520264	6489.322	7559.428	3303.239	**5140.377441**	6770.629	6547.359	3492.107	4312.751	**5280.711609**	**0.9734**	**0.9221**
Q9Z2W8	Gria4	Glutamate receptor 4	18640.19336	19229.42	25942.31	24601.9	**22103.45752**	20289.73	24993.32	24707.83	20839.74	**22707.65625**	**0.9734**	**0.7957**
Q8BSS9	Ppfia2	Liprin-alpha-2	17383.89648	14844.27	14032.86	17570.13	**15957.78833**	16616.48	17411.2	14928.07	16624.17	**16394.98242**	**0.9733**	**0.6876**
Q60766	Irgm1	Immunity-related GTPase family M protein 1	13105.03906	9058.455	14742.46	12687.25	**12398.30322**	12546.17	13394.65	13038.07	11978.8	**12739.4231**	**0.9732**	**0.7920**
Q8BRT1	Clasp2	CLIP-associating protein 2	46490.8125	44752.15	41188.14	41014.62	**43361.43066**	44394.25	46941.64	41839.07	45051.45	**44556.60352**	**0.9732**	**0.5120**
Q64524	H2bc21	Histone H2B type 2-E	124929.5938	194002.8	211215.5	235805.6	**191488.3789**	196442.7	193917.3	202841	193921.3	**196780.5625**	**0.9731**	**0.8320**
P11404	Fabp3	Fatty acid-binding protein, heart	415417.5938	422152.5	371109.1	433471.9	**410537.7734**	439612.2	412152.4	402086.8	433772.4	**421905.9453**	**0.9731**	**0.5112**
Q9QZ23	Nfu1	NFU1 iron-sulfur cluster scaffold homolog, mitochondrial	55308.66016	56333.91	55561.77	61878	**57270.58496**	57220.89	59168.64	64846.34	54195.58	**58857.8623**	**0.9730**	**0.5817**
P46737	Brcc3	Lys-63-specific deubiquitinase BRCC36	10613.10156	7964.419	10559.48	6673.477	**8952.618286**	10839.04	7977.514	11687.13	6300.441	**9201.030762**	**0.9730**	**0.8809**
O70400	Pdlim1	PDZ and LIM domain protein 1	23401.45313	19063.75	21316.73	21293.65	**21268.89453**	22891.41	21350.94	22555.97	20642.41	**21860.18066**	**0.9730**	**0.5864**
P63213	Gng2	Guanine nucleotide-binding protein G(I)/G(S)/G(O) subunit gamma-2	60376.69922	56486.25	52798.45	61753.57	**57853.74023**	53670.83	56073.48	55890.96	72233.94	**59467.30176**	**0.9729**	**0.7453**
Q68FH4	Galk2	N-acetylgalactosamine kinase		11097.28	17237.52		**14167.40381**	15064.03	10213.86	17925.52	15047.04	**14562.6123**	**0.9729**	**0.9031**
Q9D0E1	Hnrnpm	Heterogeneous nuclear ribonucleoprotein M	98019.26563	99265.58	90692.66	95791.97	**95942.36719**	108328.8	106758.3	88320.53	91091.85	**98624.86133**	**0.9728**	**0.6445**
O54833	Csnk2a2	Casein kinase II subunit alpha'	12353.57715	13252.9	13343.56	13639.71	**13147.43579**	14521.23	13985.37	14092.33	11461.26	**13515.0498**	**0.9728**	**0.6404**
Q7TT37	Elp1	Elongator complex protein 1	30261.03906	28609.8	29256.2	32382.26	**30127.3252**	30958.65	32351.11	30178.44	30394.91	**30970.77832**	**0.9728**	**0.4128**
O54890	Itgb3	Integrin beta-3			5138.422		**5138.421875**			6318.862	4246.651	**5282.756836**	**0.9727**	
Q8C460	Eri3	ERI1 exoribonuclease 3	10960.41406	15246.04	12670.6	14476.16	**13338.30273**	13733.2	14611.64	10870.15	15642.34	**13714.33228**	**0.9726**	**0.7978**
Q9QWR8	Naga	Alpha-N-acetylgalactosaminidase	9480.036133	10607.21	12370.09	8938.536	**10348.96875**	12780.15	8894.087	9474.083	11416.24	**10641.13989**	**0.9725**	**0.8115**
Q9D6W8	Borcs6	BLOC-1-related complex subunit 6	13483.87012	12505.44	14453.67		**13480.99642**	13155.28	12395.49	16335.88	13560.67	**13861.82959**	**0.9725**	**0.7477**
Q9R1V7	Adam23	Disintegrin and metalloproteinase domain-containing protein 23	158488.8281	148409.2	162335.2	145556.5	**153697.4375**	150254.5	157352.1	153461.4	171099	**158041.7656**	**0.9725**	**0.5021**
Q761V0	Slc6a5	Sodium- and chloride-dependent glycine transporter 2	62623.96875	61633.18	61045.29	64614.35	**62479.19727**	83730.73	70170.03	44283.73	58822.3	**64251.69824**	**0.9724**	**0.8402**
Q61578	Fdxr	NADPH:adrenodoxin oxidoreductase, mitochondrial	22187.80273	20735.32	20813.49	20485.93	**21055.63525**	23582.98	21409.65	21025.51	20598.77	**21654.22754**	**0.9724**	**0.4647**
O35215	Ddt	D-dopachrome decarboxylase	352310.125	348960.3	320734.1	355319.4	**344330.9688**	339828.9	334489.4	359442.5	382729.3	**354122.5313**	**0.9723**	**0.4967**
Q64018	Glra1	Glycine receptor subunit alpha-1	15760.84375	14249.12	13637.02	15131.36	**14694.58472**	14917.47	15184.53	14850.38	15497.47	**15112.46313**	**0.9723**	**0.4283**
O88602	Cacng2	Voltage-dependent calcium channel gamma-2 subunit	42370.09375	44226.7	41740.31	46226.89	**43641**	45864.23	41052.88	49260.76	43363.32	**44885.29688**	**0.9723**	**0.5621**
Q61390	Cct6b	T-complex protein 1 subunit zeta-2	7002.326172	16051.41	12820.86	13994.43	**12467.25586**	10671.92	9657.979	16976.29	13985.26	**12822.86035**	**0.9723**	**0.8939**
O55057	Pde6d	Retinal rod rhodopsin-sensitive cGMP 3',5'-cyclic phosphodiesterase subur	42652.39063	41354.61	32621.79	39518.57	**39036.84033**	42483.97	43982.58	34786.1	39369.37	**40155.50488**	**0.9721**	**0.7237**
Q9JLJ5	Elovl1	Elongation of very long chain fatty acids protein 1	6720.659668	10500.07	7271.644		**8164.123372**	9467.716	7036.518	8690.225		**8398.152832**	**0.9721**	**0.8735**
Q9WV96	Timm10b	Mitochondrial import inner membrane translocase subunit Tim10 B	23898.82813	21441.87	18825.63	18940.07	**20776.59863**	20873.13	22842.99	23336.41	18436.56	**21372.27051**	**0.9721**	**0.7288**
Q9CY50	Ssr1	Translocon-associated protein subunit alpha	44591.04688	59338.45	44074.51	48919.13	**49230.78418**	56353.89	56270.24	38488.71	51473.97	**50646.7002**	**0.9720**	**0.8055**
Q8BFY9	Tnpo1	Transportin-1	46584.03125	46069.04	43615.91	41713.44	**44495.60254**	43903.06	47669.27	47100.64	44428.73	**45775.4248**	**0.9720**	**0.4181**
Q7TNC9	Inpp5a	Inositol polyphosphate-5-phosphatase A	15101.9707	13375.11	13794.86	9139.693	**12852.90869**	15688.07	11270.22	11534.87	14398.46	**13222.90186**	**0.9720**	**0.8336**
Q8BYN3	Itpk1	Inositol-tetrakisphosphate 1-kinase	12290.74414	17869.5	8375.435	11162.24	**12424.4812**	9606.931	13757.08	17114.62	10650.36	**12782.24683**	**0.9720**	**0.8956**
Q9Z2I0	Letm1	Mitochondrial proton/calcium exchanger protein	132332.7969	119165.4	108653.9	119798.5	**119987.6602**	118977.2	127953.8	119775.6	127065.5	**123443.0137**	**0.9720**	**0.5451**
Q8K1E0	Stx5	Syntaxin-5	15803.90332	16254.72	13703.35	11169.84	**14232.95532**	16224.54	12820.38	14651.28	14881.25	**14644.3623**	**0.9719**	**0.7720**
Q80W37	Snupn	Snurportin-1	7292.360352	5506.577	6057.887	7594.589	**6612.853394**	6650.552	7559.731	4990.138	8017.47	**6804.472778**	**0.9718**	**0.8256**
Q8BGD5	Cpt1c	Carnitine O-palmitoyltransferase 1, brain isoform	8217.816406	8467.574	9947.083	11081.71	**9428.546875**	7668.031	10368.97	7025.255	13745.75	**9702.000854**	**0.9718**	**0.8754**
Q8C863	Itch	E3 ubiquitin-protein ligase Itchy	7418.690918	7621.626	5501.701	8880.616	**7355.658569**	10746.64	5719.435	7298.576	6512.137	**7569.196289**	**0.9718**	**0.8757**
Q99PT1	Arhgdia	Rho GDP-dissociation inhibitor 1	108641.1172	95502	91448.98	93160.36	**97188.11523**	91378.66	100001.3	110374.3	98343.19	**100024.375**	**0.9716**	**0.6268**
Q8BN59	Larp6	La-related protein 6	11989.71289	11807.99	8537.041	13184.81	**11379.8894**	7620.413	10977.49	15074.27	13183.3	**11713.86841**	**0.9715**	**0.8652**
O35344	Kpna3	Importin subunit alpha-4	42692.97266	44408.98	40269.69	29944.33	**39328.99463**	45594.41	42718.71	32648.58	40971.66	**40483.33838**	**0.9715**	**0.7960**
Q91ZX7	Lrp1	Prolow-density lipoprotein receptor-related protein 1	67663.09375	68293.86	66556.89	70257.04	**68192.7207**	68866.6	71499.6	71314.81	69098.32	**70194.83398**	**0.9715**	**0.1044**
Q68ED2	Grm7	Metabotropic glutamate receptor 7	13483.15527	13542.89	18043.8	13656.62	**14681.61719**	15275.82	14347.93	15955.51	14871.94	**15112.79956**	**0.9715**	**0.7255**
Q8CCN5	Bcas3	BCAS3 microtubule associated cell migration factor	16279.65332	15866.34	14814.14	14382.37	**15335.62378**	16660.43	15079.27	15526.98	15879.32	**15786.49927**	**0.9714**	**0.4474**
Q9Z1P6	Ndufa7	NADH dehydrogenase [ubiquinone] 1 alpha subcomplex subunit 7	65623.54688	61081.04	70124.75	67266.12	**66023.8623**	48317.38	65772.23	86043.98	71745.98	**67969.89453**	**0.9714**	**0.8167**
Q6PCP5	Mff	Mitochondrial fission factor	46889.04297	43872.05	38651.81	57431.97	**46711.21777**	44758.1	47998.17	46034.13	53566.07	**48089.11816**	**0.9713**	**0.7652**
Q91V14	Slc12a5	Solute carrier family 12 member 5	207432.4531	242259.9	225442.9	231578.7	**226678.4922**	262017.3	247841.4	207195.6	216487.8	**233385.5117**	**0.9713**	**0.6670**
P70245	Ebp	3-beta-hydroxysteroid-Delta(8),Delta(7)-isomerase	45603.94531	42092.64	54152.17	47973.81	**47455.6416**	44779.94	43120.27	50591.7	56967.34	**48864.81055**	**0.9712**	**0.7390**

P03893	mt-Nd2	NADH-ubiquinone oxidoreductase chain 2	20451.53516	24608.8	17361.82	15871.97	**19573.53101**	20482.91	28167.75	16807.61	15167.25	**20156.38086**	**0.9711**	**0.8724**
P60335	Pcbp1	Poly(rC)-binding protein 1	251744.5469	277083.8	240000	223401.4	**248057.4492**	255112.5	247881.4	269023.7	249783.2	**255450.1914**	**0.9711**	**0.5685**
Q9Z2I9	Sucla2	Succinate--CoA ligase [ADP-forming] subunit beta, mitochondrial	555842.875	566435.9	554967.1	561499.9	**559686.4688**	603685.3	585001	561374.6	555416.6	**576369.375**	**0.9711**	**0.1950**
P49025	Cit	Citron Rho-interacting kinase	25695.29102	25578.37	26115.57	27963.89	**26338.28223**	26560.15	25446.55	27386.62	29106.02	**27124.83447**	**0.9710**	**0.4390**
Q5M8N0	Cnrip1	**CB1 cannabinoid receptor-interacting protein 1**	185219.3594	191727.8	170268	159351.5	**176641.668**	187053.9	182456.2	173929.5	184240.2	**181919.9297**	**0.9710**	**0.5256**
O70493	Snx12	Sorting nexin-12	92586.88281	99841.22	106075	101834.2	**100084.3242**	93124.62	114252.4	107067	97874.79	**103079.709**	**0.9709**	**0.6052**
Q9CQM9	Glrx3	Glutaredoxin-3	281621.75	257593.2	235133.5	259475.8	**258456.0508**	279285.3	256387.7	250057.2	279038.1	**266192.0625**	**0.9709**	**0.5482**
Q9Z1Z0	Uso1	General vesicular transport factor p115	75214.35938	73529.34	67991.02	67196.45	**70982.79102**	72222.63	76473.61	69552.05	74193.88	**73110.54297**	**0.9709**	**0.4233**
P97765	Wbp2	WW domain-binding protein 2	61825.93359	57764.82	71634.93	57384.18	**62152.4668**	56284.31	55505.7	73579.41	70694.57	**64015.99512**	**0.9709**	**0.7579**
Q6ZQ08	Cnot1	CCR4-NOT transcription complex subunit 1	17541.99609	13623.21	15887.75	16859.97	**15978.2312**	17437.76	17298.78	15950.36	15145.05	**16457.98755**	**0.9708**	**0.6540**
Q9ES97	Rtn3	Reticulon-3	419784.5625	406554.5	410763.4	401091.6	**409548.5156**	423752.9	408449.8	410051.4	445216.4	**421867.625**	**0.9708**	**0.2369**
Q99L13	Hibadh	3-hydroxyisobutyrate dehydrogenase, mitochondrial	131903.2656	138059.4	153285	149256.6	**143126.0664**	130545.1	136360.4	167712.7	155180.9	**147449.7539**	**0.9707**	**0.6770**
P63017	Hspa8	Heat shock cognate 71 kDa protein	1733393.625	1838664	1643372	1692339	**1726941.969**	1832455	1746903	1798736	1738469	**1779140.688**	**0.9707**	**0.3102**
Q8BH66	Atl1	Atlastin-1	149243.25	159028.3	158383.9	148242.8	**153724.5625**	150094.9	167803.6	154624.7	160992.4	**158378.8867**	**0.9706**	**0.3712**
Q9DBC7	Prkar1a	cAMP-dependent protein kinase type I-alpha regulatory subunit	100993.6484	99919.05	107537.9	112993.3	**105360.9785**	106856.6	113809.5	112494.3	101070.1	**108557.627**	**0.9706**	**0.4774**
P05063	Aldoc	Fructose-bisphosphate aldolase C	2837647.75	2947798	2641792	3069936	**2874293.5**	2977171	2801297	3100937	2967767	**2961792.938**	**0.9705**	**0.4556**
Q9CQE8	RTRAF	RNA transcription, translation and transport factor protein	69593.92188	73227.64	50986.03	58276.85	**63021.11035**	69981.95	62691.09	65622.06	61478.08	**64943.29688**	**0.9704**	**0.7368**
Q9DCH4	Eif3f	Eukaryotic translation initiation factor 3 subunit F	40282.38672	36144.95	40429.52	43349.98	**40051.70996**	43234.11	44115.61	39016.66	38735.7	**41275.51855**	**0.9704**	**0.5700**
Q6A009	Ltn1	E3 ubiquitin-protein ligase listerin		5645.085			**5645.085449**	10182.68		2439.666	4832.06	**5818.135417**	**0.9703**	
P35276	Rab3d	Ras-related protein Rab-3D	268605.2188	302507.9	274570.8	322534.6	**292054.6016**	345773.2	329882.7	244223	284231.8	**301027.6797**	**0.9702**	**0.7437**
Q99PU5	Acsbg1	Long-chain-fatty-acid--CoA ligase ACSBG1	243325.8438	233490.1	212074.3	235543.4	**231108.3984**	241894.8	247084.7	236798.2	227080.5	**238214.5625**	**0.9702**	**0.4049**
Q8BKZ9	Pdhx	Pyruvate dehydrogenase protein X component, mitochondrial	165340.6406	170543.3	155427.1	169978.2	**165322.3086**	156478.8	193543.6	168379.2	163262.6	**170416.043**	**0.9701**	**0.5842**
P97467	Pam	Peptidyl-glycine alpha-amidating monooxygenase	11720.3252	15267.34	3990.248	12034.35	**10753.06702**	11152.29	14867.1	9010.461	9308.204	**11084.5127**	**0.9701**	**0.9079**
Q8VD63	Tspyl4	Testis-specific Y-encoded-like protein 4	15356.8418	14529.24	11674.33	13686.38	**13811.69678**	16102.18	16444.46	12930.75	11474.96	**14238.08838**	**0.9701**	**0.7784**
Q9CZU3	Mtrex	Exosome RNA helicase MTR4	33981.13672	40421.45	28974.83	35184.64	**34640.51318**	42810.36	35727.18	29123.88	35180.3	**35710.43408**	**0.9700**	**0.7796**
Q8R2U0	Seh1l	Nucleoporin SEH1	10511.17773	12501.33	9484.056	12196.14	**11173.17725**	11747.32	12737.57	12424.04	9169.143	**11519.51782**	**0.9699**	**0.7592**
Q60737	Csnk2a1	Casein kinase II subunit alpha	53683.24219	55042.81	61620.11	61511.6	**57964.44141**	65694.85	60041.57	56218.43	57091.6	**59761.6123**	**0.9699**	**0.5707**
P32233	Drg1	Developmentally-regulated GTP-binding protein 1	22510.59961	21195.91	20806.22	16187.28	**20175.00439**	22810.08	23632.81	17814.84	18945.32	**20800.76025**	**0.9699**	**0.7631**
Q91VC3	Eif4a3	Eukaryotic initiation factor 4A-III	52209.0625	53670.45	52347.23	50873.48	**52275.05566**	54797.19	55583.54	52577.81	52633.06	**53897.89941**	**0.9699**	**0.1397**
Q8BGG7	Ubash3b	Ubiquitin-associated and SH3 domain-containing protein B	11607.22852	15368.39	14169.47	13212.43	**13589.37817**	10514.05	16120.56	16084.06	13328.76	**14011.85596**	**0.9698**	**0.7950**
Q60902	Eps15l1	Epidermal growth factor receptor substrate 15-like 1	52303.94141	51148.76	54098.39	55603.2	**53288.57422**	53197.41	53804.16	57565.34	55216.45	**54945.83887**	**0.9698**	**0.2751**
Q9QZE7	Tsnax	Translin-associated protein X	33472.33984	33894.04	32830.05	32723.12	**33229.88525**	36833.46	32631.79	35439.51	32153.98	**34264.68506**	**0.9698**	**0.4049**
Q61235	Sntb2	Beta-2-syntrophin	38129.56641	39685.23	55210.04	32528.67	**41388.37744**	40657.44	39866.79	42281.03	47917.39	**42680.66504**	**0.9697**	**0.8115**
P07724	Alb	Albumin	1089165.375	1078670	1060556	911660	**1035012.844**	1211473	1156625	964274.9	937004.3	**1067344.391**	**0.9697**	**0.7006**
P47857	Pfkm	ATP-dependent 6-phosphofructokinase, muscle type	374016.875	366890.7	357642.1	366014.6	**366141.0703**	380119.9	380450.8	366528.9	383260.3	**377589.9688**	**0.9697**	**0.0632**
Q6IRU2	Tpm4	Tropomyosin alpha-4 chain	22628.22461	28821.96	39623.33	36470.7	**31886.05273**	26824.87	35526.04	31147.75	38034.18	**32883.21094**	**0.9697**	**0.8341**
Q920L1	Fads1	Acyl-CoA (8-3)-desaturase	14554.36621	15365.92	13413.16	13304.72	**14159.54102**	13609.36	16094.03	14430.87	14278.8	**14603.26587**	**0.9696**	**0.5611**
O88569	Hnrnpa2b1	Heterogeneous nuclear ribonucleoproteins A2/B1	353794.5625	388439.1	348668.7	359375.3	**362569.4297**	385589	382692.4	364522.6	362969.9	**373943.4609**	**0.9696**	**0.3282**
P27601	Gna13	Guanine nucleotide-binding protein subunit alpha-13	134860.7656	141021	143181	140797.6	**139965.0781**	145211.4	137112.2	149843.4	145260.2	**144356.7773**	**0.9696**	**0.2181**
Q99NB9	Sf3b1	Splicing factor 3B subunit 1	34965.62891	40348.39	36270.17	38788.18	**37593.08984**	39063.81	32979.38	42130.22	40928.16	**38775.39258**	**0.9695**	**0.6352**
Q8BMF4	Dlat	Dihydrolipoyllysine-residue acetyltransferase component of pyruvate dehyd	818511.1875	889804.4	816859.8	916165.1	**860335.1094**	896898.3	869198.4	924748.7	858737.9	**887395.8281**	**0.9695**	**0.3905**
O35685	Nudc	Nuclear migration protein nudC	48001.96484	52151.36	58704.93	48850.03	**51927.07227**	50771.7	50586.2	56272.98	56638.5	**53567.34375**	**0.9694**	**0.5981**
Q9JKR6	Hyou1	Hypoxia up-regulated protein 1	82057.72656	75250.34	78363.51	72894.23	**77141.45313**	81443.21	80370.19	77514.9	78992.85	**79580.28711**	**0.9694**	**0.3020**
Q8BHF7	Pgs1	CDP-diacylglycerol--glycerol-3-phosphate 3-phosphatidyltransferase, mito	36873.59375	34486.84	37674.92	36124.23	**36289.89551**	35695.24	35203.14	42170.07	36695.45	**37440.97266**	**0.9693**	**0.5338**
P56399	Usp5	Ubiquitin carboxyl-terminal hydrolase 5	215374.9063	219746.4	214279.8	213986.1	**215846.8281**	213524.9	230280.7	224355.7	222620.8	**222695.5352**	**0.9692**	**0.1149**
Q99MR8	Mccc1	Methylcrotonoyl-CoA carboxylase subunit alpha, mitochondrial	87610.35156	82716.2	82259.86	87226.92	**84953.33203**	85991.63	91458.7	84500.91	88651.78	**87650.75586**	**0.9692**	**0.2453**
Q9R0P9	Uchl1	Ubiquitin carboxyl-terminal hydrolase isozyme L1	1775961.125	1881021	1818832	1902721	**1844633.875**	1696295	1608058	2059027	2249622	**1903250.594**	**0.9692**	**0.7164**
P17426	Ap2a1	AP-2 complex subunit alpha-1	776306.75	806498.1	753319.4	819796.5	**788980.1719**	848431.7	816805.5	791680.3	799419.1	**814084.1563**	**0.9692**	**0.2468**
Q9CYT6	Cap2	Adenylyl cyclase-associated protein 2	49153.52734	41108.65	46138.38	50384.9	**46696.36426**	45389.43	52243.56	47391.67	47718.69	**48185.83887**	**0.9691**	**0.5763**
Q9CQJ8	Ndufb9	NADH dehydrogenase [ubiquinone] 1 beta subcomplex subunit 9	175747.2031	192008.2	181986.4	189381.5	**184780.8203**	193875.5	185494	189154.8	194174.9	**190674.7891**	**0.9691**	**0.2127**
P27048;P63163	Snrpb;Snrpn	Small nuclear ribonucleoprotein-associated protein B;Small nuclear ribonu	135838.125	155978.1	95042.96	133614.4	**130118.3965**	150479.3	139268.6	120495	126893.5	**134284.0859**	**0.9690**	**0.7816**
Q7TPM9	Mtmr10	Myotubularin-related protein 10	40277.59766	40234.27	35833.65	35169.66	**37878.79492**	42411.21	37273.2	35862.68	40832.51	**39094.89746**	**0.9689**	**0.5754**
Q921I0;Q9CQZ0	Ormdl1;Ormdl2	ORM1-like protein 1;ORM1-like protein 2	2776.164795	2980.754	1718.406		**2491.77478**			2089.051	3055.02	**2572.035522**	**0.9688**	**0.9052**
Q91W61	Fbxl15	F-box/LRR-repeat protein 15	16403.89844	18629.62	20580.41	12718.5	**17083.10742**	15962.17	16705.23	14517.74	23351.8	**17634.23413**	**0.9687**	**0.8382**
Q9D4H1	Exoc2	Exocyst complex component 2	24664.58008	26712.98	24896.96	23384.89	**24914.85205**	27007.62	27306.82	23689.62	24879.63	**25720.91846**	**0.9687**	**0.4930**
Q8BX70	Vps13c	Intermembrane lipid transfer protein VPS13C	26015.3125	25101.02	24386.43	28849.77	**26088.13232**	25300.34	25264.67	28743.82	28433.71	**26935.63672**	**0.9685**	**0.5586**
O70252	Hmox2	Heme oxygenase 2	60869.90234	63847.84	58772.51	58957.27	**60611.88281**	65735.82	63403.78	63056.84	58129.08	**62581.37988**	**0.9685**	**0.3597**
Q9D3E6	Stag1	Cohesin subunit SA-1	1840.701782				**1840.701782**				1900.557	**1900.557007**	**0.9685**	
Q8K406	Lgi3	Leucine-rich repeat LGI family member 3	208927.3906	203757.8	254826.5	246601.6	**228528.3203**	218634.2	234540	238552.3	252122.2	**235962.168**	**0.9685**	**0.6307**
Q8BGH7	Cdc42se2	CDC42 small effector protein 2	23122.95117	21373.56	21526.9	15609.26	**20408.16797**	21957.78	24821.38	18223.16	19297.52	**21074.96191**	**0.9684**	**0.7732**
Q9WVK8	Cyp46a1	Cholesterol 24-hydroxylase	5944.57959	7075.688	5008.864	5401.44	**5857.643066**	6993.568	6578.5	5948.314	4675.755	**6049.034058**	**0.9684**	**0.7867**
Q9D3P8	Plgrkt	Plasminogen receptor (KT)	18431.9707	11912.06	17278.72	17263.81	**16221.63916**	17204.54	18299.47	17618.07	13885.17	**16751.81201**	**0.9684**	**0.7736**
Q8CIG8	Prmt5	Protein arginine N-methyltransferase 5	29888.85352	36675.77	32012.49	33854.91	**33108.00488**	34686.46	34488.32	30061.33	37530.88	**34191.74854**	**0.9683**	**0.6258**
Q6NXK8	Asic1	Acid-sensing ion channel 1	5650.350586	7505.711	6587.408	11330.2	**7768.416626**	7501.609	8593.907	8452.101	7545.135	**8023.187988**	**0.9682**	**0.8488**
O54774	Ap3d1	AP-3 complex subunit delta-1	88406.875	92562.82	74816.27	83466.48	**84813.11328**	95193.97	88528.88	82041.65	84619.76	**87596.06445**	**0.9682**	**0.5809**

Q9CPQ1	Cox6c	Cytochrome c oxidase subunit 6C	602195	566634.6	607671.4	606921.6	**595855.625**	622575.4	612014.8	571462.1	655592.1	**615411.1094**	0.9682	0.3643
Q80TA1	Selenoi	Ethanolaminephosphotransferase 1	40789.69531	38997.14	44899.66	38649.88	**40834.09473**	44125.47	45811.93	41207.29	37554.55	**42174.8125**	0.9682	0.5827
Q6ZQ38	Cand1	Cullin-associated NEDD8-dissociated protein 1	443459.6563	463861.6	441176	446266.5	**448690.9531**	453347	455737.9	472751.7	471869	**463426.375**	0.9682	0.0899
Q924Y0	Bbox1	Gamma-butyrobetaine dioxygenase	11158.82129	15063.5	14011.23	12005.21	**13059.69092**	12121.53	14645.08	14930.49	12257.69	**13488.69946**	0.9682	0.7266
Q8BMS4	Coq3	Ubiquinone biosynthesis O-methyltransferase, mitochondrial	23141.31836	30883.99	25771.29	35558.56	**28838.79102**	30386.99	25271.88	36463.17	27032.4	**29788.6084**	0.9681	0.8059
Q99LB7	Sardh	Sarcosine dehydrogenase, mitochondrial	22299.60352	22073.24	21912.57	23621.04	**22476.61426**	22327.16	24993.47	20873.56	24676.11	**23217.57422**	0.9681	0.5093
Q62241	Snrpc	U1 small nuclear ribonucleoprotein C	11067.82617	11130.88	8724.672		**10307.79102**	13264.44	11621.57	6770.156	10937.58	**10648.43762**	0.9680	0.8545
O70251	Eef1b	Elongation factor 1-beta	102494.3281	88482.8	80209.41	82210.62	**88349.28711**	98495.51	91158.88	85750.9	89707.56	**91278.21094**	0.9679	0.6254
P48962	Slc25a4	ADP/ATP translocase 1	5139370	5351354	4696082	5116384	**5075797.25**	5306920	5458809	5156470	5054774	**5244243**	0.9679	0.3416
Q91ZJ5	Ugp2	UTP--glucose-1-phosphate uridylyltransferase	194134.2813	186564	177995.6	168475.3	**181792.2891**	183103.3	184789.1	204248.5	179239	**187845.0117**	0.9678	0.4706
O88746	Tom1	Target of Myb1 membrane trafficking protein	55746.85547	63760.05	54404.21	44006.33	**54479.36426**	58864.53	52240.55	57242.73	56829.43	**56294.3125**	0.9678	0.6875
Q8K0D5	Gfm1	Elongation factor G, mitochondrial	47572.07031	49914.48	43409.37	51755.08	**48162.75098**	50339.5	50431.46	51219.79	47083.4	**49768.53906**	0.9677	0.4571
Q8JZN5	Acad9	Complex I assembly factor ACAD9, mitochondrial	102044.8594	110115.9	104865.4	104755.8	**105445.5059**	107544	109177.2	111437.8	107692.7	**108962.9336**	0.9677	0.1158
P84075	Hpca	Neuron-specific calcium-binding protein hippocalcin	119640.8828	128169.4	120122.5	110655.5	**119647.0605**	124492	122533.9	135405.5	112153.2	**123646.1328**	0.9677	0.5271
P61082	Ube2m	NEDD8-conjugating enzyme Ubc12	133137.5781	151411.3	144515.5	136789.3	**141463.4258**	134422.5	145261.8	137111.8	167988.4	**146196.1211**	0.9676	0.6037
Q3UV70	Pdp1	[Pyruvate dehydrogenase [acetyl-transferring]]-phosphatase 1, mitochondri	33783.83203	33341.55	27786.9	32622.6	**31883.72021**	32964.56	37419.43	29377.7	32045.12	**32951.70264**	0.9676	0.6404
P27545	Cers1	Ceramide synthase 1	23248.6582	21134.49	19836.16	20214.68	**21108.49609**	21658.67	26775.84	15408.86	23421.15	**21816.12988**	0.9676	0.7870
Q9CZ30	Ola1	Obg-like ATPase 1	240436.0469	228353.3	196920.2	230411.3	**224030.1992**	234579.6	253070.3	205071.6	233456.9	**231544.5977**	0.9675	0.6022
Q62048	Pea15	Astrocytic phosphoprotein PEA-15	77118.29688	94694.37	80371.14	62735.55	**78729.83887**	78128.36	79419.12	80533.22	87417.98	**81374.66797**	0.9675	0.7138
Q9JLV5	Cul3	Cullin-3	78721.23438	62792.7	70317.41	75776.93	**71902.06934**	77837.29	74540.5	68286.27	76640.08	**74326.0332**	0.9674	0.5755
Q9ERU9	Ranbp2	E3 SUMO-protein ligase RanBP2	26109.26953	23128.43	26314.33	25767.69	**25329.92969**	22815.72	23841.98	28320.36	29773.9	**26187.9917**	0.9672	0.6585
Q8BGY7	Fam210a	Protein FAM210A	42747.87109	44562.96	41005.32	43144.39	**42865.13477**	50078.93	47456.68	37376.8	42374.75	**44321.79199**	0.9671	0.6342
Q8CGV2	Tph2	Tryptophan 5-hydroxylase 2	11798.32227	14170.38	14694.31	15907.9	**14142.72754**	19023.91	9962.562	12988.5	16522.81	**14624.44629**	0.9671	0.8314
Q9DBS1	Tmem43	Transmembrane protein 43	24092.76367	19257.41	24237.57	19819.43	**21851.79541**	20869.93	17866.89	28046.13	23613.28	**22599.05664**	0.9669	0.7788
Q99JY8	Plpp3	Phospholipid phosphatase 3	74491.46094	70410.23	51224.48	76750.53	**68219.17578**	72003.2	76467.95	72638.68	61112.44	**70555.56836**	0.9669	0.7387
P70290	Mpp1	55 kDa erythrocyte membrane protein	93229.17969	86693.52	96225.8	95233.37	**92845.46875**	92873.88	95686.77	94795.79	100755.8	**96028.05859**	0.9669	0.2870
Q3TZM9	Alg11	GDP-Man:Man(3)GlcNAc(2)-PP-Dol alpha-1,2-mannosyltransferase	9165.183594	15847.91	13346.86	8873.705	**11808.41455**	14188.31	11696.43	11397.56	11572.22	**12213.63086**	0.9668	0.8307
Q3V038	Ttc9	Tetratricopeptide repeat protein 9A	13494.09668	21722.78	22518.67	15581.44	**18329.24731**	13435.03	14263.72	21574.51	26567.67	**18960.23364**	0.9667	0.8750
O35136	Ncam2	Neural cell adhesion molecule 2	85116.625	72040.12	65937.98	77396.5	**75122.80469**	83668.89	76114.38	78651.29	72408.64	**77710.79883**	0.9667	0.6024
Q9D7S9	Chmp5	Charged multivesicular body protein 5	50359.37891	55622.23	54612.52	38709.23	**49825.83789**	54126.05	58883.59	48071.44	45089.51	**51542.64746**	0.9667	0.7408
Q80UW2	Fbxo2	F-box only protein 2	242210.2969	260758.2	270707.6	223867.8	**249385.9805**	244531.8	238597	260202	288634	**257991.1914**	0.9666	0.5928
Q9R013	Ctsf	Cathepsin F	5884.273438	5978.331		7768.34	**6543.648112**		6436.291	7107.363		**6771.827148**	0.9663	0.8012
P11859	Agt	Angiotensinogen	6250.890625	9661.281	6644.933	6667.723	**7306.206909**		9766.604	6051.954	6864.733	**7561.096842**	0.9663	0.8554
Q8BMG8	Slc25a32	Mitochondrial folate transporter/carrier	6243.623535	7648.628	7242.574	8929.45	**7516.06897**	7333.988	7499.494	9593.362	6689.335	**7779.044922**	0.9662	0.7648
Q5HZI9	Slc25a51	Mitochondrial nicotinamide adenine dinucleotide transporter SLC25A51	57427.12109	52070.75	51183.87	53571.24	**53563.24414**	59201.8	57731.63	52307.55	52522.05	**55440.75684**	0.9661	0.4353
Q9JI78	Ngly1	Peptide-N(4)-(N-acetyl-beta-glucosaminyl)asparagine amidase	14936.87891	16145.82	16653.78	16416.5	**16038.24268**	17651.58	16606.31	14360.54	17791.83	**16602.56494**	0.9660	0.5449
Q8BK03	Miga2	Mitoguardin 2	34268.47266	38914.63	36591.8	41334.62	**37777.38086**	38770.28	39072.68	38243.8	40349.3	**39109.01563**	0.9660	0.4325
O35083	Agpat1	1-acyl-sn-glycerol-3-phosphate acyltransferase alpha	59433.67969	52349.55	60843.31	59569.03	**58048.89355**	58121.71	60038.86	60402.71	61833.48	**60099.19043**	0.9659	0.3607
Q8CI94	Pygb	Glycogen phosphorylase, brain form	453195	498602.8	445616	438145.7	**458889.8906**	471866.6	479561.8	484176.4	464793.6	**475099.6016**	0.9659	0.2985
Q9ER72	Cars1	Cysteine--tRNA ligase, cytoplasmic	57282.125	58847.44	54537.19	58961.29	**57407.00977**	61270.38	57771.33	59922.89	58801.1	**59441.42578**	0.9658	0.1618
Q922G0	Slc25a36	Solute carrier family 25 member 36	48719.46875	52196.93	47525.3	57828.32	**51567.50684**	51378.8	55988.91	55386.83	50834.6	**53397.28711**	0.9657	0.5183
Q8BHC1	Rab39b	Ras-related protein Rab-39B	61342.16016	64634.33	74205.2	74493.28	**68668.74414**	73304.73	69391.66	70414.16	71311.69	**71105.56055**	0.9657	0.5065
P60764	Rac3	Ras-related C3 botulinum toxin substrate 3	53173.85547	45816.49	43079.2	55025.78	**49273.83008**	48209.48	48932.97	52890.71	54056.6	**51022.44043**	0.9657	0.6055
Q9DB41	Slc25a18	Mitochondrial glutamate carrier 2	357068.5313	401243.9	428438.6	449392.2	**409035.7969**	405900.2	402375.3	471500	414500.4	**423568.9609**	0.9657	0.5918
P48428	Tbca	Tubulin-specific chaperone A	49900.375	44139.16	63682.61	35854.64	**48394.19824**	37389.18	43123.07	56154.51	63798.94	**50116.42188**	0.9656	0.8443
P08752	Gnai2	Guanine nucleotide-binding protein G(i) subunit alpha-2	461586.875	479918.4	503553.4	457948.8	**475751.875**	491521.3	447017.7	556632.4	475574.2	**492686.375**	0.9656	0.5306
P47757	Capzb	F-actin-capping protein subunit beta	252880.5781	257663.1	223139	227323.3	**240251.4766**	264315.4	270217.8	235174.1	225507.1	**248803.582**	0.9656	0.5636
Q9QYY0	Gab1	GRB2-associated-binding protein 1	21232.22266	21982.06	21173.79	13070.17	**19364.56079**	16978.73	18510.49	22556.6	22171.7	**20054.38037**	0.9656	0.7930
P68254	Ywhaq	14-3-3 protein theta	112765.0625	112953.4	111846.6	115252.4	**113204.3555**	102831.5	113581.2	130929.9	121677.2	**117254.9727**	0.9655	0.5259
Q3UHQ6	Dop1b	Protein dopey-2		5439.122	4139.553		**4789.337646**			6903.616	3017.827	**4960.721802**	0.9655	0.9409
Q61474	Msi1	RNA-binding protein Musashi homolog 1	13090.18262	13141.19	8791.863	6586.514	**10402.43689**	11533.21	11644.48	9027.105	10895.02	**10774.9519**	0.9654	0.8375
Q58A65	Spag9	C-Jun-amino-terminal kinase-interacting protein 4	44702.92188	38888.95	35966.21	40361.72	**39979.95215**	41153.23	48233	36221.48	40053.55	**41415.31543**	0.9653	0.6594
P08775	Polr2a	DNA-directed RNA polymerase II subunit RPB1	969.1408691	383.8234	80.36805	613.5278	**511.7150364**	283.8623		897.879	408.5373	**530.0928548**	0.9653	0.9487
P58044	Idi1	Isopentenyl-diphosphate Delta-isomerase 1	63975.32031	59773.28	56348.79	54835.49	**58733.21973**	57648.41	58651.43	59281.48	67799.11	**60845.10547**	0.9653	0.5210
Q3U0V1	Khsrp	Far upstream element-binding protein 2	85084.47656	85622.64	75286.73	84400.73	**82598.64648**	92182.38	92994.23	77106.92	80026.89	**85577.60547**	0.9652	0.5554
Q6NVF0	Ocrl	Inositol polyphosphate 5-phosphatase OCRL	12328.0791	13461.99	11387.12	13088.11	**12566.32568**	12881.16	13429.52	12583.21	13190.86	**13021.18579**	0.9651	0.3927
Q9Z2S7	Tsc22d3	TSC22 domain family protein 3	86600.05469	88435.28	92904.27	87519.55	**88864.78711**	85353.72	80627.68	93768.32	108623.4	**92093.27148**	0.9649	0.6267
Q8BTV2	Cpsf7	Cleavage and polyadenylation specificity factor subunit 7	22412.1543	20320.73	21143.41	18770.95	**20661.8125**	23455.01	19038.52	21932.17	21227.86	**21413.39063**	0.9649	0.5521
Q3UFK8	Frmd8	FERM domain-containing protein 8	18527.52734	18960.34	19132.32	16729.48	**18337.41699**	18070.72	16556.79	20414.9	20980.76	**19005.79443**	0.9648	0.5883
A2TJV2	Palm3	Paralemmin-3	9474.49707	6424.799			**7949.647949**	8499.602	9594.29	6215.13	8648.915	**8239.484253**	0.9648	0.8487
Q61151	Ppp2r5e	Serine/threonine-protein phosphatase 2A 56 kDa regulatory subunit epsilor	71014.03125	69225.59	71856.11	72324.5	**71105.05664**	75261.62	74647.31	72626.31	72275.7	**73702.73438**	0.9648	0.0414
Q9CRB9	Chchd3	MICOS complex subunit Mic19	120351.8203	101540.9	105908	111867.9	**109917.1602**	113598.5	101645.8	113899.3	126660.2	**113950.9355**	0.9646	0.5596
Q8CFV9	Rfk	Riboflavin kinase	27046.17188	31532.46	28121.03	23406.19	**27526.4624**	27979.11	29454.27	26253.06	30461.88	**28537.08154**	0.9646	0.6153
Q61704	Itih3	Inter-alpha-trypsin inhibitor heavy chain H3	38459.97656	37339.54	45811.52	38741.89	**40088.22852**	44804.26	38817.96	42056.87	40564.66	**41560.9375**	0.9646	0.5474
Q80WM4	Hapln4	Hyaluro and proteoglycan link protein 4	81408.16406	89898.36	87893.29	85582.11	**86195.48047**	87543.76	89628.63	95180.86	85135.63	**89372.2168**	0.9645	0.3020

Q9D880	Timm50	Mitochondrial import inner membrane translocase subunit TIM50	48926.09766	48499.57	53329.63	47087.66	**49460.74023**	50341.51	52765.95	47744.15	54285.32	**51284.2334**	0.9644	0.3897
P07214	Sparc	SPARC	16194.51465	14418.58	16497.28	12584.11	**14923.62061**	15306.17	19344.61	13272.45	13973.33	**15474.14038**	0.9644	0.7472
P62313	Lsm6	U6 snRNA-associated Sm-like protein LSm6	20093.90234	22167.67	20432.31	19665.38	**20589.81592**	20979.91	20854.89	22810.56	20752.93	**21349.57324**	0.9644	0.3413
Q8VHN8	Nudt16l1	Tudor-interacting repair regulator protein	14343.5127	19844.7	17498.17	17657.74	**17336.03247**	17637.18	17884.06	20285.84	16096.57	**17975.91064**	0.9644	0.6692
Q9DC71	Mrps15	Small ribosomal subunit protein uS15m	14670.6416	15808.07	15114.31	17476.69	**15767.42993**	17585.57	14221.05	18455.98	15136.33	**16349.73267**	0.9644	0.6373
D3YZU1	Shank1	SH3 and multiple ankyrin repeat domains protein 1	23193.31055	25002.57	20844.27	27660.46	**24175.15332**	25269.74	24589.84	25635.51	24778.6	**25068.42188**	0.9644	0.5630
Q3TFQ1	Spryd7	SPRY domain-containing protein 7	7821.856445	13924.09	13018.8	17712.92	**13119.41602**	13792.41	14571.04	15617.86	10439.13	**13605.10693**	0.9643	0.8414
Q80TJ1	Cadps	Calcium-dependent secretion activator 1	290149.3125	296551	270568.5	288873.6	**286535.5938**	300837.5	311726.6	279937.1	296093.2	**297148.5938**	0.9643	0.2657
Q9JJ59	Abcb9	ABC-type oligopeptide transporter ABCB9	38102.9375	34133.1	38280.55	36108.41	**36656.25098**	35783.69	37626.63	37415.54	41240.4	**38016.56641**	0.9642	0.4018
P62743	Ap2s1	AP-2 complex subunit sigma	151120.1094	113665.1	130453	133611.8	**132212.5**	132214.9	136272.3	145893.5	134098.2	**137119.7188**	0.9642	0.5739
Q6PE15	Abhd10	Palmitoyl-protein thioesterase ABHD10, mitochondrial	108912.4141	95618.46	101719.1	112105.9	**104588.9648**	109558	107471.1	113156.3	103708.8	**108473.5371**	0.9642	0.3897
Q91VR8	Brk1	Protein BRICK1	20918.47852	14191.29	14177.22	12326.27	**15403.31567**	11946.33	19441.19		16542.83	**15976.78516**	0.9641	0.8504
P35505	Fah	Fumarylacetoacetase	217967.0313	197870.8	216545.1	234675.9	**216764.6992**	222643.6	225784.4	207681	243350.4	**224864.8477**	0.9640	0.4696
Q9CQV1	Pam16	Mitochondrial import inner membrane translocase subunit TIM16	16432.49609	10592.92	15293.9	16191.41	**14627.67896**	12410.99	19488.95	13764.7	15032.59	**15174.30908**	0.9640	0.7992
B2RUJ5	Apba1	Amyloid-beta A4 precursor protein-binding family A member 1	10253.08594	11689.7	12670.9	12551.36	**11791.26099**	10574.87	12509.66	13804.44	12038.9	**12231.96606**	0.9640	0.6301
Q9QYF9	Ndrg3	Protein NDRG3	132858.0781	133715	114364.2	111220.8	**123039.5078**	130810	137569.6	128149.7	114023.8	**127638.2891**	0.9640	0.5743
Q9D0D3	Mtpap	Poly(A) RNA polymerase, mitochondrial	6688.790039	6691.885	5848.817	4703.67	**5983.290649**	5933.381	4481.787	7149.587	7264.319	**6207.268799**	0.9639	0.7893
Q9CRB8	Mtfp1	Mitochondrial fission process protein 1	21938.98633	19133.6	20779.55	25415.45	**21816.89746**	26180.12	23917.18	18298.92	22138.4	**22633.65527**	0.9639	0.7147
Q62288	Spock1	Testican-1	39890.82422	33769.93	32591.52	33436.27	**34922.13428**	38143.04	36359.04	38736.64	31681.06	**36229.94531**	0.9639	0.5926
P70663	Sparcl1	SPARC-like protein 1	28069.85742	28753.77	25200.23	25716.21	**26935.01611**	27924.29	31529.39	27199.91	25133.93	**27946.88184**	0.9638	0.5484
Q3UM45	Ppp1r7	Protein phosphatase 1 regulatory subunit 7	160620.7969	147572.5	155317.2	153481.5	**154247.9922**	154097.8	154791	170863.4	160421.2	**160043.3359**	0.9638	0.2653
Q91YK0	Lrrc49	Leucine-rich repeat-containing protein 49	3980.667236				**3980.667236**	3893.277	4367.971			**4130.624023**	0.9637	
Q80YD1	Supv3l1	ATP-dependent RNA helicase SUPV3L1, mitochondrial	7854.17627	10184.35	6132.358	10705.5	**8719.096191**	8283.948	8739.607	10118.58	9048.442	**9047.643799**	0.9637	0.7813
Q9Z210	Pex11b	Peroxisomal membrane protein 11B	8909.492188	6603.585	6517.31	5938.736	**6992.280884**	7542.263	6265.378	6801.632	8414.264	**7255.884155**	0.9637	0.7544
Q62418	Dbnl	Drebrin-like protein	22310.22461	20048.38	24398.88	24271.64	**22757.28174**	21229.11	24441.72	24672.26	24118.87	**23615.48828**	0.9637	0.5336
Q9D6J6	Ndufv2	NADH dehydrogenase [ubiquinone] flavoprotein 2, mitochondrial	561315.8125	550769.1	564773.9	575274.1	**563033.2031**	585737.6	589218.1	563379.8	598812.6	**584287**	0.9636	0.0570
P70297	Stam	Signal transducing adapter molecule 1	46993.46484	50249.91	44765.47	41621.74	**45907.64746**	51424.87	45276.81	44643.79	49234.62	**47645.02246**	0.9635	0.5023
B9EJI9	Tmem229a	Transmembrane protein 229A	9635.796875	7547.914	12121.44	6486.461	**8947.9021**	6605.965	7297.348	13270.22	9974.251	**9286.946655**	0.9635	0.8683
Q9JMA1	Usp14	Ubiquitin carboxyl-terminal hydrolase 14	130578.5313	141109.6	142382.3	135061.9	**137283.0859**	128720.9	145399.7	150109.6	145715.4	**142486.3906**	0.9635	0.3770
Q8K4Q0	Rptor	Regulatory-associated protein of mTOR	17750.59766	16721.99	20045.71	20684.88	**18800.79248**	21262.73	21837.42	17412.51	17549.21	**19515.46631**	0.9634	0.6521
Q8VDM4	Psmd2	26S proteasome non-ATPase regulatory subunit 2	89377.46875	85088.6	71521.56	75484.76	**80368.09766**	86411.91	86809.05	81505.91	78968.11	**83423.74609**	0.9634	0.5277
P97315	Csrp1	Cysteine and glycine-rich protein 1	285887.4688	308634.4	325536.3	305256.8	**306328.7344**	317043.5	296165.7	334993.4	323709.8	**317978.0938**	0.9634	0.3509
Q922P9	Glyr1	Cytokine-like nuclear factor N-PAC	14036.34473	14463.5	11315.33	12469.97	**13071.28613**	11946.39	15679.13	12665.83	13985.29	**13569.15942**	0.9633	0.6653
Q9DBL9	Abhd5	1-acylglycerol-3-phosphate O-acyltransferase ABHD5	24303.21289	24272.09	19295.37	17901.53	**21443.04932**	26168.09	22743.88	17042.1	23090.49	**22261.13867**	0.9633	0.7573
Q7TQA1	Igsf1	Immunoglobulin superfamily member 1	8913.262695	10672.51	7782.651	7763.168	**8782.897827**	7356.85	10018.86	10756.59	8342.504	**9118.702515**	0.9632	0.7564
P84089	Erh	Enhancer of rudimentary homolog	124921.9766	126014.2	110760.8	112196.9	**118473.4727**	140425.7	124548.6	118664.4	108388.7	**123006.8418**	0.9631	0.5837
Q8K4Z0	Lgi2	Leucine-rich repeat LGI family member 2	30431.55078	29493.87	29771.23	31089.49	**30196.53418**	27188.29	29070.93	34040.69	35112.81	**31353.17969**	0.9631	0.5740
P10605	Ctsb	Cathepsin B	58012.05078	52404.83	56658.03	52942.42	**55004.33398**	53970.75	55680.05	58433.02	60369.07	**57113.22168**	0.9631	0.3279
Q9CZB0	Sdhc	Succinate dehydrogenase cytochrome b560 subunit, mitochondrial	87713.80469	93080.81	93866.86	106238.1	**95224.89648**	100680.9	93049.45	104463.3	97324.98	**98879.63867**	0.9630	0.4581
P42932	Cct8	T-complex protein 1 subunit theta	430879.375	434942.8	389928.4	411855.2	**416901.4219**	443933.7	460018.1	419193.1	408475.7	**432905.1094**	0.9630	0.3441
O88952	Lin7c	Protein lin-7 homolog C	58820.01563	43221.7	66138.9	74453.23	**60658.46094**	68416	61173.84	67024.7	55333.52	**62987.01367**	0.9630	0.7598
Q9D2N4	Dtna	Dystrobrevin alpha	28551.86914	26977.79	36538.63	31729.45	**30949.43262**	32380.79	33880.81	34052.85	28243.39	**32139.46094**	0.9630	0.6515
Q6ZPZ3	Zc3h4	Zinc finger CCCH domain-containing protein 4	12529.29199	10896.35	3288.111	9157.647	**8967.849915**	14142.15	11036.29	6738.579	5335.013	**9313.008545**	0.9629	0.9075
Q2PFD7	Psd3	PH and SEC7 domain-containing protein 3	16679.65039	14807.9	15239.71	13536.8	**15066.01489**	14189.44	13892.83	18287.27	16218.61	**15647.03662**	0.9629	0.6478
Q9JIK9	Mrps34	Small ribosomal subunit protein mS34	36810.45703	32318.12	35313.89	32337.43	**34194.97314**	33921.49	38661.21	39790.13	29684.69	**35514.37891**	0.9628	0.6271
Q3UFS0	Zyg11b	Protein zyg-11 homolog B	10372.51855	13556.52	14866.1	13466.74	**13065.46948**	12298.21	13334.67	16028.63	12623.21	**13571.1814**	0.9627	0.7054
Q9D7P6	Iscu	Iron-sulfur cluster assembly enzyme ISCU	58641.82422	59160.97	59168.64	59019.91	**58997.83594**	60393.65	61423.06	62930.45	60385.14	**61283.07324**	0.9627	0.0098
Q8K268	Abcf3	ATP-binding cassette sub-family F member 3	10359.87305	13038.7	15940.8	13373.43	**13178.20264**	10490.32	11605.19	16614.31	16050.33	**13690.03735**	0.9626	0.7989
Q505D7	Opa3	Optic atrophy 3 protein homolog	67701.61719	76687.56	60120.7	74506.43	**69754.07617**	77914.1	70895.63	73686.31	67363.49	**72464.88281**	0.9626	0.5563
Q8BFR4	Gns	N-acetylglucosamine-6-sulfatase	10585.32227	12244.5	12605.96	11018.9	**11613.66968**	11503.13	11498.04	13386.06	11874.17	**12065.3501**	0.9626	0.5187
Q8VE22	Mrps23	Small ribosomal subunit protein mS23	33928.36719	33958.63	35700.62	37139.29	**35181.72656**	36886.71	34623.05	37458.48	37237.39	**36551.4082**	0.9625	0.2247
Q8JZR6	Slc4a8	Electroneutral sodium bicarbonate exchanger 1	21359.09375	20284.06	21858.22	23603.36	**21776.18213**	24848.19	23490.98	23366.85	18795.17	**22625.29736**	0.9625	0.5895
Q8R310	Tmcc3	Transmembrane and coiled-coil domain protein 3	22270.39453	28826.67	30320.66	22813.25	**26057.74316**	32872.82	31159.04	23527.92	20744.08	**27075.96289**	0.9624	0.7856
Q60771	Cldn11	Claudin-11	136149.9531	102546.3	174761.2	136015.2	**137368.1484**	107771.3	109690.1	180640.2	172844.1	**142736.4434**	0.9624	0.8346
Q80XI3	Eif4g3	Eukaryotic translation initiation factor 4 gamma 3	34436.10547	33181.87	34512.03	30413.48	**33135.87207**	31701.62	36801.48	35617.19	33634.56	**34438.71533**	0.9622	0.4111
O08759	Ube3a	Ubiquitin-protein ligase E3A	18425.51758	20684.56	19036.49	16315.15	**18615.43091**	18064.59	19445.04	20842.99	19039.31	**19347.98096**	0.9621	0.5196
Q9D8W5	Psmd12	26S proteasome non-ATPase regulatory subunit 12	70777.58594	74758.9	62564.41	68658.09	**69189.74805**	77642.45	71612.86	67503.42	70899.23	**71914.49023**	0.9621	0.4413
Q811I0	Atpaf1	ATP synthase mitochondrial F1 complex assembly factor 1	32619.86523	28873.48	28488.28	29731.6	**29928.30811**	30610.07	36194.01	31141.38	26494.83	**31110.07031**	0.9620	0.6098
Q6PDL0	Dync1li2	Cytoplasmic dynein 1 light intermediate chain 2	123329.5156	119638.2	120584.6	110985.9	**118634.5547**	122534.9	122106.5	121969.3	126680	**123322.6836**	0.9620	0.1565
O54941	Smarce1	SWI/SNF-related matrix-associated actin-dependent regulator of chromatir	13389.79004	11626.87	10087.69	12216.64	**11830.24707**	12944.61	12666.11	12507.5	11076.31	**12298.63403**	0.9619	0.5812
P28028	Braf	Serine/threonine-protein kinase B-raf	43869.39453	41433.69	40702.96	44451.93	**42614.49512**	43723	39814.72	48423.81	45246.63	**44302.04297**	0.9619	0.4327
Q9D387	Lamp5	Lysosome-associated membrane glycoprotein 5	106002.7656	108768.1	69520.5	89992.08	**93570.85547**	110776.4	112473.6	88694.69	77177.06	**97280.43164**	0.9619	0.7762
Q8VDZ4	Zdhhc5	Palmitoyltransferase ZDHHC5	10093.35059	6437.534	6034.85	8684.697	**7812.608032**	8035.405	7941.802	9429.649	7083.144	**8122.499878**	0.9618	0.7827
P18242	Ctsd	Cathepsin D	296299.3438	302252.7	327818.8	327284.7	**313413.875**	294465.3	322778.1	328256.3	357934.9	**325858.6328**	0.9618	0.4500
Q9QYE6	Golga5	Golgin subfamily A member 5	7993.983887	673.3331	5638.34		**4768.552266**	1789.107	9520.507	3940.819	4581.245	**4957.919403**	0.9618	0.9457

Q08331	Calb2	Calretinin	527710.5	591985.9	582145.2	512118.4	553490	508449.7	573215.3	567284.6	653114.4	575516.0078	0.9617	0.5596
P46471	Psmc2	26S proteasome regulatory subunit 7	72201.34375	79197.69	77241.14	72553.52	75298.42383	76556.47	79834.69	79975.34	76820.75	78296.8125	0.9617	0.1785
Q9ESJ4	Nckipsd	NCK-interacting protein with SH3 domain	100785.0625	99622.66	110570.4	103676.9	103663.7676	102375.7	104442.9	108344.6	116089	107813.0293	0.9615	0.3277
Q9CQQ7	Atp5pb	ATP synthase F(0) complex subunit B1, mitochondrial	784336.5625	749224.3	698219.3	738364.8	742536.2188	769113.9	789892.2	753612	776533.2	772287.8125	0.9615	0.1737
Q9JLM8	Dclk1	Serine/threonine-protein kinase DCLK1	125446.6563	108884.1	106576.7	127416.2	117080.9355	129009	121527.2	120064.7	116531.5	121783.123	0.9614	0.4655
P70271	Pdlim4	PDZ and LIM domain protein 4	10770.72949	3453.792	4286.216	11580.24	7522.744507		7799.468	6547.623	9129.386	7825.492025	0.9613	0.9114
P62761	Vsnl1	Visinin-like protein 1	1167772.625	1273813	1058054	1100507	1150036.469	1196027	1229602	1213198	1146522	1196337.219	0.9613	0.3933
Q9CX60	Lbh	Protein LBH	32059.83203	30040.05	26878.97	29583.17	29640.50488	26941.38	30096.06	31881.88	34424.26	30835.89453	0.9612	0.5525
Q3UHH2	Slc22a23	Solute carrier family 22 member 23	16108.73828	16413.58	11709.95	13505.43	14434.42554	15796.21	11879.5	15067.62	17328.41	15017.93311	0.9611	0.7282
Q8R105	Vps37c	Vacuolar protein sorting-associated protein 37C	21950.72461	23030.25	19887.33	20507.55	21343.96387	24086.29	20247.69	23529.18	20965.25	22207.10205	0.9611	0.4919
Q8VEJ9	Vps4a	Vacuolar protein sorting-associated protein 4A	85514.42969	89495.75	80541.68	84706.66	85064.63086	88318.73	89131.69	93951.2	82657.71	88514.83008	0.9610	0.2871
Q99K70	Rragc	Ras-related GTP-binding protein C	33236.45703	32939.35	33156.31	25784.25	31279.09277	33637.2	36789.43	29388.78	30380.38	32548.94775	0.9610	0.6277
P62918	Rpl8	Large ribosomal subunit protein uL2	160742.8125	169539.5	156902.8	162735.3	162480.1172	160269.1	184811.8	168550.1	162752.3	169095.8438	0.9609	0.3212
Q9R1T4	Septin6	Septin-6	123149.2578	140316.4	159957.2	200518.6	155985.377	172504.9	162950.8	170477	143449.5	162345.5508	0.9608	0.7347
Q9D1G1	Rab1b	Ras-related protein Rab-1B	46932.92969	43840.04	51069.48	47743.93	47396.59375	50517.38	46412.11	51147.17	49245.13	49330.44531	0.9608	0.3286
Q3TPX4	Exoc5	Exocyst complex component 5	15198.50293	17080.46	16498.92	17836.11	16653.49536	20817.61	17926.94	14641.67	15947.95	17333.5415	0.9608	0.6566
P43406	Itgav	Integrin alpha-V	48614.07031	52384.52	44316.92	43623.8	47234.82715	50381.14	49410.74	45517.81	51377.71	49171.84766	0.9606	0.4523
Q9D009	Lipt2	Putative lipoyltransferase 2, mitochondrial	6650.754395	7308.552	6076.465	5497.217	6383.247192	7107.563	5603.631	5702.682	8166.747	6645.15564	0.9606	0.7303
Q99KW9	Itfg1	T-cell immunomodulatory protein	18596.28711	17992.8	16342.36	14175.01	16776.61548	17124.33	18596.47	16891.84	17253.45	17466.52246	0.9605	0.5397
P46660	Ina	Alpha-internexin	2726756.25	2751830	2995444	2804431	2819615.313	2861251	2813587	2993457	3074491	2935696.563	0.9605	0.2227
Q922S4	Pde2a	cGMP-dependent 3',5'-cyclic phosphodiesterase	6729.538574	6022.35	5879.056	8080.743	6677.921753	6590.466	7611.858	6180.871	7428.666	6952.965088	0.9604	0.6666
Q9R0K7	Atp2b2	Plasma membrane calcium-transporting ATPase 2	589505.3125	572082.6	569927.4	583087.8	578650.7656	602166.9	607205.9	610446.4	590211.1	602507.5781	0.9604	0.0098
Q8R570	Snap47	Synaptosomal-associated protein 47	144579.6563	139671.3	127215.7	147379.9	139711.6406	148090.1	150715.8	143527.9	139562.5	145474.0586	0.9604	0.3014
P67778	Phb1	Prohibitin 1	424551.3125	450722.7	436617.4	412409.6	431075.2422	432658	449362.1	459738.1	453732.7	448872.7031	0.9604	0.1270
P12658	Calb1	Calbindin	252322.7031	259061.6	222129	276753.8	252566.7617	333634	232477.9	239650.7	246219.3	262995.4648	0.9603	0.7054
P70670;Q60817	Naca	Nascent polypeptide-associated complex subunit alpha, muscle-specific f	51762.16797	49033.42	48629.52	43950	48343.77734	52567.57	50453.96	51446.46	46908.96	50344.2373	0.9603	0.3628
P48168	Glrb	Glycine receptor subunit beta	25805.62109	23521.29	24184.05	23349.96	24215.229	23339.84	22794.29	27747.2	26988.25	25217.39355	0.9603	0.4936
Q8K2T1	Nmral1	NmrA-like family domain-containing protein 1	84169.57031	97852.33	83001.52	82563.62	86896.75781	81762.95	95306.03	97628.97	87285.41	90495.8418	0.9602	0.5132
Q8BIQ5	Cstf2	Cleavage stimulation factor subunit 2	24898.42969	20761.76	16022.61	21995.35	20919.53687	23373.39	22552.67	18847.1	22371.66	21786.20313	0.9602	0.6946
Q9D338	Mrpl19	Large ribosomal subunit protein bL19m	25662	30322.04	30009.72	33841.05	29958.70557	37059.42	30035.42	30633.17	27079.17	31201.79492	0.9602	0.6600
P12960	Cntn1	Contactin-1	713330	722761.9	703169.8	731221.3	717620.7188	740369	712940.1	790987.3	745353.6	747412.4844	0.9601	0.1353
Q8BLV3	Slc9a7	Sodium/hydrogen exchanger 7	19240.41016	17646.69	13631.58	19561.09	17519.94141	20252.96	19780.99	15237.52	17720.73	18248.05078	0.9601	0.6965
P00375	Dhfr	Dihydrofolate reductase	6590.739746	6710.586	7971.195	5969.495	6810.503906	5845.114	5740.383	8524.024	8266.76	7094.070313	0.9600	0.7535
Q04690	Nf1	Neurofibromin	45852.39453	49027.64	46586.28	45583.93	46762.55957	49049.82	50638.97	48597.12	46557.71	48710.9043	0.9600	0.1411
P20357	Map2	Microtubule-associated protein 2	905961.9375	846319.3	815657.8	875015.4	860738.6094	906744.8	936317.3	848940.4	894945.2	896736.8906	0.9599	0.2236
Q8K2B3	Sdha	Succinate dehydrogenase [ubiquinone] flavoprotein subunit, mitochondrial	463904.3125	450071.7	441527.5	449506.2	451252.4297	472317.8	482689.2	466626.4	459021.8	470163.7813	0.9598	0.0322
Q91ZM2	Sh2b1	SH2B adapter protein 1	13327.42578	17357.87	14744.45	12514.82	14486.14185	19522.11	11004.08	17092.1	12756.37	15093.66504	0.9597	0.7939
Q9DB77	Uqcrc2	Cytochrome b-c1 complex subunit 2, mitochondrial	972211.9375	969303.6	964057	1036538	985527.5781	1030133	1030994	1013468	1033026	1026905.234	0.9597	0.0578
Q8K3H0	Appl1	DCC-interacting protein 13-alpha	54339.14063	54926.11	47503.84	56351.21	53280.0752	54458.71	54391.76	53574	59645.02	55517.37402	0.9597	0.3895
P61979	Hnrnpk	Heterogeneous nuclear ribonucleoprotein K	243874.125	231272.3	232335.9	237389.8	236218.0469	252729.7	251218.9	243442.8	237250.7	246160.5469	0.9596	0.0744
Q99JY9	Actr3	Actin-related protein 3	139637.125	151262.7	140050.5	134315.4	141316.4219	145508.5	144555.2	150787.1	148320.7	147292.8672	0.9594	0.1700
Q9QYB8	Add2	Beta-adducin	200932.5625	200899.4	210656.3	215725.8	207053.5	217427.4	217041.5	218970.1	209839.2	215819.5469	0.9594	0.0828
Q68FD5	Cltc	Clathrin heavy chain 1	1862257	1916755	1906585	1925343	1902734.781	1932274	1941639	2049984	2009381	1983319.469	0.9594	0.0425
P70288	Hdac2	Histone deacetylase 2	28151.90039	29869.21	21184.49	21872.1	25269.42432	29801.17	29033.13	23463.29	23062.14	26339.92969	0.9594	0.7180
Q6ZWX6	Eif2s1	Eukaryotic translation initiation factor 2 subunit 1	115235.8984	119510.5	100116.8	103095.4	109489.6758	110623.4	113258.5	113277.5	119424.4	114145.9434	0.9592	0.3906
Q60649	Clpb	Mitochondrial disaggregase	12234.61426	10241.64	9982.571	6851.723	9827.637817	10981.13	11174.39	9796.59	9030.768	10245.71948	0.9592	0.7440
Q9CZ04	Cops7a	COP9 signalosome complex subunit 7a	118240.4922	132002.9	124889	112155.6	121822.0098	126037.4	119982	133615.7	128482.8	127029.4941	0.9590	0.3490
Q9DB16	Cab39l	Calcium-binding protein 39-like	60716.71875	46497.76	74288.05	59717.49	60305.00488	69393.83	58314.62	56542.26	67288.61	62884.8291	0.9590	0.7059
Q9D0A3	Arpin	Arpin	39658.33203	23285.99	27323.75	34359.29	31156.8418	24453.7	29165.09	37346.15	39002.24	32491.7959	0.9589	0.7987
Q9Z2E1	Mbd2	Methyl-CpG-binding domain protein 2	14295.7793	20380.66	21806.32	24870.96	20338.42871	21078.53	17878.79	21887.29	24000.53	21211.28516	0.9588	0.7446
Q9CZ62	Cep97	Centrosomal protein of 97 kDa	13190.30859	11094.85			12142.58008	11895.89	11542.43	14553.4		12663.90885	0.9588	0.7437
Q7M764	Usp17le	Ubiquitin carboxyl-terminal hydrolase 17-like protein E	20529.72266	21248.23	22104.91	27840.58	22930.85938	22488.02	22481.82	27994.85	22714.9	23919.89941	0.9587	0.6620
Q9WTX8	Mad1l1	Mitotic spindle assembly checkpoint protein MAD1		11462.72	7942.549	13292.85	10899.3724	8841.899		12643.33	12627.62	11370.94922	0.9585	0.8265
Q9CQH3	Ndufb5	NADH dehydrogenase [ubiquinone] 1 beta subcomplex subunit 5, mitochon	244475.9844	270487.1	253856.4	275765.6	261146.2617	262601.3	275744.8	287084.7	264351.8	272445.6406	0.9585	0.2662
Q8R0F8	Fahd1	Acylpyruvase FAHD1, mitochondrial	64076.27734	61115.18	59125.63	69130.48	63361.8916	52851.49	68369.02	68829.01	74365.85	66103.84375	0.9585	0.6108
Q63912	Omg	Oligodendrocyte-myelin glycoprotein	805893.5625	828780.7	773192.1	817789	806413.8281	795219.4	834147.8	881662.8	854284.6	841328.625	0.9585	0.1605
Q9JJ28	Flii	Protein flightless-1 homolog	20389.63867	21354.14	22528.24	20905.84	21294.46582	22353.69	21065.66	23786.8	21660.94	22216.77393	0.9585	0.2604
P28658	Atxn10	Ataxin-10	35012.76172	36657.15	34835.36	37685.23	36047.625	37658.59	34921.08	42513.07	35345.4	37609.53516	0.9585	0.4357
P62827	Ran	GTP-binding nuclear protein Ran	391103.8438	411340.9	372799.8	373124.7	387092.3203	397822.7	388969.9	397510.4	431210.7	403878.4219	0.9584	0.2464
Q9CR62	Slc25a11	Mitochondrial 2-oxoglutarate/malate carrier protein	636423.4375	648198	640817.9	685456.8	652724.0156	663771.4	677439.6	728758.9	654367.8	681084.3906	0.9584	0.2059
Q8BH60	Gopc	Golgi-associated PDZ and coiled-coil motif-containing protein	4455.046387	11144.58	10063.42	3467.44	7282.621216		7599.732			7599.731934	0.9583	
Q920N2	Hlcs	Biotin--protein ligase	20398.66406	21454.05	24553.44	20650.69	21764.21143	19536	19390.48	27443.75	24481.45	22712.91992	0.9582	0.6802
Q6PER3	Mapre3	Microtubule-associated protein RP/EB family member 3	423943.7188	446960.3	417436.5	433723.4	430515.9688	434930.5	451497.2	454844.7	456053.1	449331.3594	0.9581	0.0587
D3YWQ0	Dgki	Diacylglycerol kinase iota	21047.85742	18630.14	24465.46	22129.26	21568.1792	24223.09	18458.8	22831.99	24531.49	22511.34326	0.9581	0.6287
Q8BGP6	Slc25a40	Probable mitochondrial glutathione transporter SLC25A40	41141.83594	41686.2	31040.02	41336.33	38801.09473	47148.42	40863.26	39124.07	34857.41	40498.28809	0.9581	0.6570

Q3U186	Rars2	Probable arginine--tRNA ligase, mitochondrial	9473.619141	8574.68	5765.993	9803.518	8404.452271	7883.715	7565.498	10157.65	9483.679	8772.635498	0.9580	0.7512
Q8CHY3	Dym	Dymeclin	4312.074707	5740.335	7507.351	3276.922	5209.170776	5898.186		4270.563	6144.648	5437.798665	0.9580	0.8554
Q8C0J2	Atg16l1	Autophagy-related protein 16-1	17282.04688	20046.82	11116.14	13570.41	15503.85376	17676.76	17405.8	14310.48	15352	16186.25952	0.9578	0.7601
Q8R1V4	Tmed4	Transmembrane emp24 domain-containing protein 4	17938.6875	14724.51	17302.45	16789.36	16688.75122	18231.17	18040.98	18171.39	15251.4	17423.7356	0.9578	0.4921
Q60668	Hnrnpd	Heterogeneous nuclear ribonucleoprotein D0	199908.6563	203623.9	184219.1	196020.3	195942.9844	212968.4	212065	211082.2	182220.9	204584.1133	0.9578	0.3521
Q6PFR5	Tra2a	Transformer-2 protein homolog alpha	23176.94727	20609.88	22800.8	12061.87	19662.37207	17827.63	17304.09	23860.21	23135.87	20531.94922	0.9576	0.7895
P62196	Psmc5	26S proteasome regulatory subunit 8	87764.77344	88482.2	74822.41	78398.72	82367.02344	86731.33	90250.13	78244.95	88827.09	86013.37109	0.9576	0.4329
Q8JZL3	Thtpa	Thiamine-triphosphatase	32873.57031	38749.65	39659.2	29374.8	35164.30469	38973.88	36075.47	34092.24	37746.48	36722.0166	0.9576	0.5803
Q9D5T0	Atad1	Outer mitochondrial transmembrane helix translocase	49486.07813	49585.5	42007.09	38887.17	44991.45898	50194.79	45925.18	46902.68	44918.95	46985.39941	0.9576	0.5219
Q9Z110	Aldh18a1	Delta-1-pyrroline-5-carboxylate synthase	44773.30078	50559.19	48378.56	49847.85	48389.72559	53918.63	50824.65	49551.94	47868.05	50540.81836	0.9574	0.2807
Q9QYR6	Map1a	Microtubule-associated protein 1A	747285.6875	754350.3	735194.9	752788.6	747404.875	778615.3	780295.4	773476.1	790184.3	780642.7656	0.9574	0.0010
Q9CQV4	Retreg3	Reticulophagy regulator 3	19761.37305	20076.82	20367.45	20816.26	20255.47656	21786.47	20080.57	23431.84	19327.4	21156.56934	0.9574	0.3764
P70398	Usp9x	Probable ubiquitin carboxyl-terminal hydrolase FAF-X	101239.7344	100256.9	98367.11	100692.1	100138.959	106622.4	105217.4	100658.3	105880	104594.5195	0.9574	0.0237
P28650	Adss1	Adenylosuccinate synthetase isozyme 1	215386.7344	220658.3	169114	181808.7	196741.9063	214023.2	215677.9	185221.4	207099.4	205505.457	0.9574	0.5657
Q8CIM7	Cyp2d26	Cytochrome P450 2D26	11769.92188	12533.33	14493.95	13272.53	13017.43213	12185.25	13421	14300.82	14485.76	13598.20679	0.9573	0.4859
Q8VD75	Hip1	Huntingtin-interacting protein 1	16897.75	16061.76	15296.71	19394.73	16912.73779	17495.9	18355.83	19937.64	14883.35	17668.17969	0.9572	0.6042
P62892	Rpl39	Large ribosomal subunit protein eL39	45796.53516	46261.23	15299.69	36045.14	35850.64917	37788.33	50184.41	30440.51	31395.32	37452.1416	0.9572	0.8576
Q3TW96	Uap1l1	UDP-N-acetylhexosamine pyrophosphorylase-like protein 1	29669.08789	32376.15	34925.76	32774.37	32436.34277	29449.83	33684.46	35065.21	37341.76	33885.31543	0.9572	0.4918
Q9Z1W9	Stk39	STE20/SPS1-related proline-alanine-rich protein kinase	76108.02344	92011.07	77623.83	82973.41	82179.08398	89287.02	84755.47	85154.84	84225.52	85855.70898	0.9572	0.3677
Q9WU63	Hebp2	Heme-binding protein 2	24778.14453	25653.81	23955.58	24812.33	24799.96631	23443.24	24055.27	27164.35	28981.88	25911.18506	0.9571	0.4430
Q3UGC7	Eif3j1	Eukaryotic translation initiation factor 3 subunit J-A	22082.25586	21313.36	23864.95	22942.91	22550.86719	22685.46	22707.78	22559.68	26306.1	23564.75439	0.9570	0.3787
P43024	Cox6a1	Cytochrome c oxidase subunit 6A1, mitochondrial	147533.6563	119831.3	189827.1	131952.8	147286.2012	142941.2	136821.2	183533.1	152391.8	153921.7969	0.9569	0.7316
Q1HFZ0	Nsun2	RNA cytosine C(5)-methyltransferase NSUN2	2486.455566	5340.255	7022.561	9746.572	6148.960938	8557.155	4304.448	6429.166	6416.583	6426.838135	0.9568	0.8792
Q9CXJ4	Abcb8	Mitochondrial potassium channel ATP-binding subunit	32814.99609	29071.25	24783.69	26430.72	28275.16309	29434.99	32998.09	26743.25	29037.02	29553.3374	0.9568	0.5786
Q69ZR2	Hectd1	E3 ubiquitin-protein ligase HECTD1	9802.911133	9085.362	11102.94	10868.78	10214.99731	10519.16	10533.68	10656.39	11001.11	10677.58252	0.9567	0.3762
Q9EPK2	Rp2	Protein XRP2	12238.6416	10687.39	16012.44	17081.37	14004.95947	12474.77	22024.82	11856.04	12204.43	14640.01538	0.9566	0.8336
P16054	Prkce	Protein kinase C epsilon type	28148.54883	32245.69	24524.35	27447.64	28091.55713	30086.21	28283.49	30992.49	28123.81	29371.5	0.9564	0.4894
P58281	Opa1	Dynamin-like 120 kDa protein, mitochondrial	151371.4219	146538	133260.9	138695.3	142466.3828	151273.5	153371.1	147290.1	143927	148965.418	0.9564	0.2026
Q5BKP2	Usp13	Ubiquitin carboxyl-terminal hydrolase 13	57258.23828	56235.21	75286.66	61607.64	62596.9375	79638.49	47472.61	65277.64	69429.59	65454.58105	0.9563	0.7337
Q8BLY2	Tars3	Threonine--tRNA ligase 2, cytoplasmic	82113.77344	84866.95	91686.48	84409.93	85769.2832	90672.02	87932.57	89670.67	90469.1	89686.08984	0.9563	0.1189
Q9JJL8	Sars2	Serine--tRNA ligase, mitochondrial	12288.15625	13789.33	11653.19	13864.03	12898.677	15126.05	13492.34	14612.25	10720.81	13487.86328	0.9563	0.6200
Q9Z0P5	Twf2	Twinfilin-2	35215.41016	42170.31	38196.87	40887.02	39117.40234	44224.11	43336.66	36962.34	39100.61	40905.93066	0.9563	0.4689
P56375	Acyp2	Acylphosphatase-2	139188.3125	157185.7	126612.7	139236.8	140555.8828	137132.4	143214.6	149055.1	158538.2	146985.1094	0.9563	0.4394
Q9WUK2	Eif4h	Eukaryotic translation initiation factor 4H	56393.60547	59968.32	61145.94	56307.23	58453.77344	63695.55	57278.14	57235.85	66303.96	61128.37598	0.9562	0.3450
Q8CGF6	Wdr47	WD repeat-containing protein 47	47802.51563	42261.92	44801.84	46582.72	45362.24902	54442.93	46964.8	46135.99	42208.1	47437.95703	0.9562	0.4901
Q9Z160	Cog1	Conserved oligomeric Golgi complex subunit 1	6083.438477	8647.468		8947.248	7892.718099	5062.094	10741.83	5660.966	11552.29	8254.295288	0.9562	0.8719
Q8BGZ4	Cdc23	Cell division cycle protein 23 homolog	7757.629883	13261.39	9078.414	10305.71	10100.78564	8808.454	11819.43	8328.187	13307.14	10565.80273	0.9560	0.7909
P54775	Psmc4	26S proteasome regulatory subunit 6B	105083.6641	97214.03	97625.3	96378.14	99075.28516	99502.8	104296.5	109071.5	101753.6	103656.0918	0.9558	0.1628
Q8VED9	Lgalsl	Galectin-related protein	37953.35156	47660.95	34467.5	51303.69	42846.37402	37398.17	44787.11	53833.58	43303.9	44830.68945	0.9557	0.7171
O70589	Cask	Peripheral plasma membrane protein CASK	49707.85938	46224.78	45700.38	52419.07	48513.02344	51641.89	54800.96	47680.17	48958.71	50770.43652	0.9555	0.3504
Q2TBE6	Pi4k2a	Phosphatidylinositol 4-kinase type 2-alpha	24703.1582	24760	29275.85	26237.43	26244.1084	30334.36	26195.43	27081.55	26250.23	27465.39307	0.9555	0.4320
P58021	Tm9sf2	Transmembrane 9 superfamily member 2	20587.47852	22640.27	18741.57	23702.97	21418.07031	19259.88	21266.59	25539.67	23596.82	22415.74268	0.9555	0.5905
Q8K2Q5	Chchd7	Coiled-coil-helix-coiled-coil-helix domain-containing protein 7	8112.250488	7437.909		12499.53	9349.895508	8796.11	12298.01	8434.834	9616.942	9786.473145	0.9554	0.8056
Q9Z1K5	Arih1	E3 ubiquitin-protein ligase ARIH1	8803.28125	13302.54	13520.53	17921.78	13387.03076	14761.96	15151.78		12123.67	14012.46842	0.9554	0.8001
Q8C050	Rps6ka5	Ribosomal protein S6 kinase alpha-5	19875.15625	21759.82	16573.66	19777.04	19496.41748	20578.19	19541.58	23101.02	18416.81	20409.39795	0.9553	0.5571
Q91VM5	Rbmxl1	RNA binding motif protein, X-linked-like-1	144984.125	155841.5	135385.1	128568.4	141194.7676	143261.3	151665	158196.9	138110.3	147808.3555	0.9553	0.4067
Q9CPU4	Mgst3	Glutathione S-transferase 3, mitochondrial	83242.70313	94430.3	69892.01	70625.4	79547.60156	83139.07	79976.09	86867.6	83116.69	83274.86328	0.9552	0.5572
Q9R087	Gpc6	Glypican-6			4823.387	6739.354	5781.370605	4199.567		7905.02		6052.293457	0.9552	0.9085
Q8BFZ2	Plppr1	Phospholipid phosphatase-related protein type 1	34886.85547	41664.14	25471.94	39778.03	35450.24365	39533.5	40697.06	37465.9	30750.58	37111.76123	0.9552	0.7092
Q9CWE0	Mtfr1l	Mitochondrial fission regulator 1-like	27978.96484	27380.41	29364.36	27860.28	28146.00146	27530.99	33801.95	29766.31	26763.44	29465.67383	0.9552	0.4507
A2ALU4	Shroom2	Protein Shroom2	20351.71094	21305.08	19566.89	18102.04	19831.4292	20013.68	18550.04	23707.78	20774.38	20761.46729	0.9552	0.4945
Q91YE6	Ipo9	Importin-9	23380.98242	31804.3	26827.25	25539.61	26888.03516	26559.79	32330.09	25663.84	28043.04	28149.18896	0.9552	0.6060
Q8K3C3	Lzic	Protein LZIC	70744.42969	79115.25	50595.71	41243.96	60424.83887	73974.88	68663.13	49687.06	60713.35	63259.60547	0.9552	0.7909
P63042	Stmn4	Stathmin-4			8852.322		8852.322266	9146.93	10434.83	8225.338		9269.030924	0.9550	
Q9WTR1	Trpv2	Transient receptor potential cation channel subfamily V member 2	6729.42041	7206.404	10137.38	8353.498	8106.674683	8052.585	8798.505	7940.81	9166.362	8489.565552	0.9549	0.6544
Q9CR20	Ier3ip1	Immediate early response 3-interacting protein 1	35888.64844	38727.05	34129.31	35523.28	36067.07227	35910.89	43376.71	35654.05	36141.62	37770.81738	0.9549	0.4492
Q9Z1N5	Ddx39b	Spliceosome RNA helicase Ddx39b	189966	200664.1	181684.9	183731.5	189011.6172	194860.7	205358.9	183350.6	208192.9	197940.7656	0.9549	0.2538
P61922	Abat	4-aminobutyrate aminotransferase, mitochondrial	1255627.5	1232875	1174171	1258820	1230373.406	1304813	1299560	1310409	1239857	1288659.844	0.9548	0.0629
Q99MN1	Kars1	Lysine--tRNA ligase	60164.86719	63786.96	54962.49	58631.98	59386.57617	62902.92	63363.81	60073.52	62478.55	62204.70215	0.9547	0.2025
Q9Z130	Hnrnpdl	Heterogeneous nuclear ribonucleoprotein D-like	47775.91016	49532.93	50450.61	48519.1	49069.63672	54341.06	47491.92	55907.44	47858.48	51399.72559	0.9547	0.3408
Q99N93	Mrpl16	Large ribosomal subunit protein uL16m	11175.81152	8898.38	14003.93	15715.12	12448.30908	10646.06	12478.49	12757.58	16275.7	13039.45776	0.9547	0.7677
P48722	Hspa4l	Heat shock 70 kDa protein 4L	439605.375	423955	400151	405977	417422.0859	429656.7	444559.4	440131.2	434632.7	437244.9844	0.9547	0.0828
P56480	Atp5f1b	ATP synthase subunit beta, mitochondrial	4420398	4588603	4607612	4822540	4609788.25	4675306	4632513	4990825	5016243	4828721.375	0.9547	0.1450
Q8VDM6	Hnrnpul1	Heterogeneous nuclear ribonucleoprotein U-like protein 1	14174.11328	16708.64	12008.91	12425.68	13829.33521	13216.45	14376.12	14619.35	15732.79	14486.17944	0.9547	0.5998
Q810U4	Nrcam	Neuronal cell adhesion molecule	27281.64453	27480.47	26303.27	30167.95	27808.33545	28366.93	29833.9	28925.09	29390.8	29129.17822	0.9547	0.1863

Q923Z3	Mto1	Protein MTO1 homolog, mitochondrial	9128.196289	6295.346	7387.965	11199.06	**8502.64209**	10196.76	9976.446	6397.051	9056.709	**8906.741455**	**0.9546**	**0.7798**
P68510	Ywhah	14-3-3 protein eta	105146.125	110139.6	141140.5	130857.1	**121820.834**	112393.9	82285.61	163276.3	152491.2	**127611.7402**	**0.9546**	**0.7871**
Q8BGN3	Enpp6	Glycerophosphocholine cholinephosphodiesterase ENPP6	206730.6406	206257.1	175468.4	178698.1	**191788.5664**	205434.8	210897	196333.8	190978.1	**200910.9492**	**0.9546**	**0.3795**
O70325	Gpx4	Phospholipid hydroperoxide glutathione peroxidase	288310.1875	295530.3	265341.8	283661.4	**283210.9141**	295812.7	287769.8	296999.7	306171.7	**296688.4609**	**0.9546**	**0.1208**
Q9CWX2	Ndufaf1	Complex I intermediate-associated protein 30, mitochondrial	18932.9375	17752.43	18688.53	17523.26	**18224.28809**	19928.88	16691.67	22757.54	16988.19	**19091.56885**	**0.9546**	**0.5755**
Q8C0L0	Tmx4	Thioredoxin-related transmembrane protein 4	54272.51172	55006.44	53684.81	55435.25	**54599.75488**	60074.19	60015.29	54174.81	54543.53	**57201.95508**	**0.9545**	**0.1742**
Q9D2R0	Aacs	Acetoacetyl-CoA synthetase	29066.11328	33753.14	27791.97	30899.68	**30377.72559**	33735.86	35850.44	26199.53	31523.17	**31827.25049**	**0.9545**	**0.5747**
Q8BW41	Pomgnt2	Protein O-linked-mannose beta-1,4-N-acetylglucosaminyltransferase 2	47908.67578	44329.4	43326.61	42622.11	**44546.7002**	52433.73	45823.27	43031.86	45421.37	**46677.55664**	**0.9543**	**0.3961**
Q5FWH7	Slc39a12	Zinc transporter ZIP12	58222.23047	46130.31	34532.17	43025.04	**45477.4375**	49588.91	46955.59	46697.75	47377.63	**47654.9707**	**0.9543**	**0.6753**
P13595	Ncam1	Neural cell adhesion molecule 1	988549.5625	966227.1	971619.1	1011057	**984363.2969**	1067299	1044934	981199.6	1032620	**1031513.063**	**0.9543**	**0.0644**
O89023	Tpp1	Tripeptidyl-peptidase 1	36772.09766	39158.48	34139.42	37075.86	**36786.46191**	37167.47	34993.07	45277.17	36804.24	**38560.4873**	**0.9540**	**0.5062**
P56371	Rab4a	Ras-related protein Rab-4A	44322.96094	38106.48	36902.86	42666.52	**40499.7041**	46738.16	40850.35	44109.17	38128.39	**42456.51953**	**0.9539**	**0.4782**
P10630	Eif4a2	Eukaryotic initiation factor 4A-II	89783.77344	86792.17	81842.47	77199.13	**83904.38477**	94586.21	90243.7	75575.37	91477.01	**87970.57227**	**0.9538**	**0.4521**
Q99K23	Ufsp2	Ufm1-specific protease 2	23292.91797	24586.73	20604.29	17518.57	**21500.62598**	24554.74	22808.58	22139.99	20676.63	**22544.9873**	**0.9537**	**0.5745**
P57774	Npy	Pro-neuropeptide Y	19618.62109	24876.46	23724.88	35443.2	**25915.79053**	26814.82	25603.69	30415.91	25864.42	**27174.71094**	**0.9537**	**0.7349**
P12815	Pdcd6	Programmed cell death protein 6	60117.33203	74387.26	69147.75	61652.23	**66326.14258**	68789.82	58379.88	77346.24	73679.86	**69548.95117**	**0.9537**	**0.5651**
P60229	Eif3e	Eukaryotic translation initiation factor 3 subunit E	29216.16016	28247.68	29536.92	23816.38	**27704.28516**	30357.75	31627.72	27700.91	26527.3	**29053.41895**	**0.9536**	**0.4748**
Q6P4T2	Snrnp200	U5 small nuclear ribonucleoprotein 200 kDa helicase	21928.02539	21844.31	23298.6	23264.1	**22583.75732**	24463.89	25455.47	24071.46	20748.55	**23684.84326**	**0.9535**	**0.3546**
P18894	Dao	D-amino-acid oxidase	39354.93359	41774.83	33109.82	29127.1	**35841.6709**	41534.52	40557.91	35177.23	33092.56	**37590.55371**	**0.9535**	**0.6390**
Q8C2E7	Washc5	WASH complex subunit 5	21702.21875	19162.88	18990	15778.4	**18908.37402**	17521.36	22036.51	19785.23	19982.82	**19831.479**	**0.9535**	**0.5671**
Q9ET01	Pygl	Glycogen phosphorylase, liver form	1546292	1668507	1003938	1084472	**1325802.109**	1507335	1482556	1158353	1414206	**1390612.625**	**0.9534**	**0.7362**
Q62523	Zyx	Zyxin	21747.30273	19266.91	25085.34	12483.49	**19645.7627**	17041.77	19428.1	22652.75	23302.6	**20606.30371**	**0.9534**	**0.7628**
Q62425	Ndufa4	Cytochrome c oxidase subunit NDUFA4	1759284	1593329	1453175	1552255	**1589510.688**	1711621	1634871	1751478	1571186	**1667288.875**	**0.9534**	**0.3418**
P12787	Cox5a	Cytochrome c oxidase subunit 5A, mitochondrial	1228686.25	1242903	1141142	1148585	**1190328.875**	1289019	1274415	1180322	1250587	**1248585.563**	**0.9533**	**0.1546**
P51881	Slc25a5	ADP/ATP translocase 2	5148705.5	5303694	4533757	4909486	**4973910.375**	5396160	5587505	4886231	5000078	**5217493.125**	**0.9533**	**0.3400**
Q8WUR0		Protein C19orf12 homolog	13324.44531	14928.37	12660.19	10360.51	**12818.37793**	16196.08		12171.63	11974.18	**13447.29492**	**0.9532**	**0.7116**
P15626	Gstm2	Glutathione S-transferase Mu 2	692879.75	705796	566540.5	592551.9	**639442.0469**	732025.3	709023.3	498830.3	743463.3	**670835.5156**	**0.9532**	**0.6587**
Q9ERA0	Tfcp2	Alpha-globin transcription factor CP2	13420.46484	17155.96	15484.48	10307.65	**14092.13965**	11633.97	19947.74	13999.25	13559.52	**14785.11792**	**0.9531**	**0.7756**
Q8C7R4	Uba6	Ubiquitin-like modifier-activating enzyme 6	24556.50391	20288.31	23136.76	22535.36	**22629.23535**	23781.84	24550.68	24154.68	22513.08	**23750.06982**	**0.9528**	**0.3014**
Q8BGT8	Phyhipl	Phytanoyl-CoA hydroxylase-interacting protein-like	189476.375	213668.1	172066.2	179248.8	**188614.8672**	212288.4	206039.1	197668.6	175846.9	**197960.7734**	**0.9528**	**0.4683**
Q9D024	Ccdc47	PAT complex subunit CCDC47	17818.54883	17847.09	14309.9	14861.78	**16209.33228**	18029.51	17741.88	17134.52	15146.36	**17013.06641**	**0.9528**	**0.5094**
Q99NB1	Acss1	Acetyl-coenzyme A synthetase 2-like, mitochondrial	36983.57813	31997.54	31563.24	31037.37	**32895.4292**	34200.68	35815.63	36109.56	31987.94	**34528.44971**	**0.9527**	**0.3659**
Q9DC70	Ndufs7	NADH dehydrogenase [ubiquinone] iron-sulfur protein 7, mitochondrial	85670.91406	90122.67	83434.2	79592.95	**84705.18359**	72764.87	104894.9	86792.96	91197.7	**88912.5957**	**0.9527**	**0.5685**
Q9CRA7	Dmac2l	ATP synthase subunit s, mitochondrial	52182.50781	54368.3	46890.48	50567.05	**51002.08594**	54503.4	52913.69	56044.68	50681.75	**53535.87988**	**0.9527**	**0.2413**
P80560	Ptprn2	Receptor-type tyrosine-protein phosphatase N2	31058.74609	30396.18	28527.61	30347.59	**30082.53271**	31503.23	31704.92	31297.83	31804.2	**31577.54541**	**0.9527**	**0.0358**
O88448	Klc2	Kinesin light chain 2	63590.23828	62839.33	62565.09	59538.89	**62133.3877**	63123.14	59739.04	68422.79	69604.97	**65222.48438**	**0.9526**	**0.2584**
Q99NH2	Pard3	Partitioning defective 3 homolog	10313.84082	11580.92	9059.617	9405.326	**10089.92725**	11840.45	11277.7	9619.771	9632.957	**10592.71948**	**0.9525**	**0.5533**
Q91V24	Abca7	ATP-binding cassette sub-family A member 7	5503.814941	6980.457	4605.268	7690.538	**6195.019409**	7491.563	6694.757	5076.996	6755.36	**6504.669189**	**0.9524**	**0.7325**
Q3UGS4	Mcrip1	Mapk-regulated corepressor-interacting protein 1	20057.74805	8024.952	19065.09	15977.33	**15781.27942**	16623.07	13311.25	16921.13	19431.47	**16571.73389**	**0.9523**	**0.8012**
Q9CZW4	Acsl3	Fatty acid CoA ligase Acsl3	54317.63281	59622.16	55140.91	58263.93	**56836.15723**	55245.7	54633.54	65842.62	63077.73	**59699.89746**	**0.9520**	**0.3880**
Q9CQS8	Sec61b	Protein transport protein Sec61 subunit beta	18116.01367	15629.22	12689.55	13214.78	**14912.39111**	25989.81	9893.311	17176.33	9596.335	**15663.94849**	**0.9520**	**0.8592**
Q8K3K8	Optn	Optineurin	11109.60742	12550.26	14578.23	9664.931	**11975.75659**	13725.94	11475.25	13746.83	11371.1	**12579.78052**	**0.9520**	**0.6443**
Q8VDN4	Ccdc92	Coiled-coil domain-containing protein 92	77406.96875	72104.72	78178.18	84278.71	**77992.14453**	84175.75	84075.88	83501.7	75980.38	**81933.42578**	**0.9519**	**0.2628**
Q3UHL1	Camkv	CaM kinase-like vesicle-associated protein	16763.03516	15751.23	13300.1	17734.48	**15887.21143**	18766.53	17344.92	15358.19	15290.99	**16690.15674**	**0.9519**	**0.5506**
Q8CGF7	Tcerg1	Transcription elongation regulator 1	249877.9531	311187	227707	223831.5	**253150.8633**	308189.9	298660.2	214657.1	242368.4	**265968.8906**	**0.9518**	**0.6858**
Q8BL65	Ablim2	Actin-binding LIM protein 2	47782.05859	49150.17	52172.89	45191.57	**48574.17285**	49868.88	47593.4	57800.27	48875.73	**51034.57031**	**0.9518**	**0.4011**
Q9CQ88	Tspan31	Tetraspanin-31	14316.02441		17129.99		**15723.00928**	14857.08		20399.1	14303.47	**16519.88151**	**0.9518**	**0.7888**
Q9Z0Y1	Dctn3	Dynactin subunit 3	129321.0859	136442.8	109991	117537.2	**123323.0273**	139889.9	125366.2	125918.8	127183.1	**129589.4961**	**0.9516**	**0.3954**
Q8VE33	Gdap1l1	Ganglioside-induced differentiation-associated protein 1-like 1	82041.99219	81681.73	74504.34	82217.58	**80111.41211**	81709.27	81053.68	84790.41	89218.35	**84192.92773**	**0.9515**	**0.1732**
Q9D2M8	Ube2v2	Ubiquitin-conjugating enzyme E2 variant 2	423743	439706.9	420003.9	456669.5	**435030.8203**	434142.1	436490.8	502128.9	456104.7	**457216.6172**	**0.9515**	**0.2603**
Q8BYM5	Nlgn3	Neuroligin-3	41959.17578	39402.74	33837.9	39993.56	**38798.34473**	41482.56	40960.02	38138.53	42534.02	**40778.78516**	**0.9514**	**0.3555**
Q62420	Sh3gl2	Endophilin-A1	478266.0938	444900	459187.7	453338.1	**458922.9844**	491234.8	479066.1	479847.5	479308	**482364.1094**	**0.9514**	**0.0224**
Q9D554	Sf3a3	Splicing factor 3A subunit 3	12054.73926	17580.48	24786.68	17329.84	**17937.93335**	23871.47	13213.55	22207.44	16127.34	**18854.9502**	**0.9514**	**0.8087**
B2RQC6	Cad	CAD protein	31321.28906	28948.62	29401.36	29573.67	**29811.23291**	30147.32	34633.88	29937.03	30637.26	**31338.87402**	**0.9513**	**0.2586**
Q2KN98	Specc1l	Cytospin-A	7453.042969	7415.584		4191.365	**6353.330729**	4102.533	11417.74	4285.792	6914.69	**6680.188965**	**0.9511**	**0.8882**
Q60900	Elavl3	ELAV-like protein 3	43931.60156	43321.08	39811.71	40663.39	**41931.94629**	42568.85	42547.56	46985.39	44256.98	**44089.69824**	**0.9511**	**0.1866**
O88507	Cntfr	Ciliary neurotrophic factor receptor subunit alpha	35287.65234	34877.62	23980.37	27495.27	**30410.229**	36479.07	33382.29	27962.23	30089.67	**31978.31201**	**0.9510**	**0.6572**
O88696	Clpp	ATP-dependent Clp protease proteolytic subunit, mitochondrial	54387.20313	46995.25	49664.09	50372.8	**50354.83496**	61972.7	51150.42	47957.84	50728.52	**52952.36914**	**0.9509**	**0.4795**
P62264	Rps14	Small ribosomal subunit protein uS11	136355.4375	134267.6	130809.2	114205.5	**128909.4434**	144625.2	140411.8	131978	125221.8	**135559.2051**	**0.9509**	**0.3554**
Q922J3	Clip1	CAP-Gly domain-containing linker protein 1	13094.18555	10900.54	13477.9	11282.85	**12188.86743**	11025.98	13236.84	14407.42	12601.74	**12817.99707**	**0.9509**	**0.5341**
Q8BX94	Osbpl2	Oxysterol-binding protein-related protein 2	34363.64453	34004.88	37014.68	34054.72	**34859.48145**	31028.8	33488.39	49934.48	32205.78	**36664.36182**	**0.9508**	**0.7029**
Q3TRM4	Pnpla6	Patatin-like phospholipase domain-containing protein 6	18871.84766	17996.18	19007.84	16804.03	**18169.97412**	18531.99	19875.58	20108.4	17927.21	**19110.79443**	**0.9508**	**0.2453**
Q99MP8	Brap	BRCA1-associated protein	3486.39917	5133.895	4386.616	4347.444	**4338.588318**			5719.298	3408.648	**4563.972778**	**0.9506**	**0.8082**
Q8QZY6	Tspan14	Tetraspanin-14	7997.41748	7112.365	6136.591	7194.469	**7110.210449**		6633.178	8390.296	7415.943	**7479.805664**	**0.9506**	**0.5769**
Q9EPJ9	Arfgap1	ADP-ribosylation factor GTPase-activating protein 1	44527.94141	48989.86	22841.77	40841.48	**39300.26172**	57145.38	41475.68	36259.3	30497.54	**41344.47705**	**0.9506**	**0.8092**

Q61206	Pafah1b2	Platelet-activating factor acetylhydrolase IB subunit alpha2	647306.3125	648507.3	589263.9	669966.8	**638761.0625**	645694.6	616372.7	666450.4	760136.5	**672163.5469**	**0.9503**	**0.3839**
Q3UMR5	Mcu	Calcium uniporter protein, mitochondrial	103736.9063	93938.42	91772.71	99459.82	**97226.96484**	100725	100067.1	111666.8	96808.7	**102316.8711**	**0.9503**	**0.2727**
Q9JM14	Nt5c	5'(3')-deoxyribonucleotidase, cytosolic type	56181.83203	54239.22	52788.62	54169.53	**54344.79883**	53762.91	52563.41	61387.48	61052.43	**57191.55957**	**0.9502**	**0.2879**
Q8VC85	Lsm1	U6 snRNA-associated Sm-like protein LSm1	15896.56152	14159.94	11297.4	6986.51	**12085.10168**	14515.92	11986.38	10603.92	13776.29	**12720.62915**	**0.9500**	**0.7762**
Q9CZX8	Rps19	Small ribosomal subunit protein eS19	94838.39063	91989.34	90485.52	72880.2	**87548.35938**	96402.78	90166.08	89456.81	92602.83	**92157.125**	**0.9500**	**0.4107**
Q3THE2	Myl12b	Myosin regulatory light chain 12B	151009.5313	162302.1	157065.8	146272.8	**154162.5625**	178129	173323.9	141580.4	156098.3	**162282.8984**	**0.9500**	**0.4050**
Q8CI78	Rmnd1	Required for meiotic nuclear division protein 1 homolog	15117.63574	17819.33	18287.02	16199.64	**16855.90845**	20325.27	16734.74	17072.03	16843.35	**17743.84766**	**0.9500**	**0.4626**
A2ALS5	Rap1gap	Rap1 GTPase-activating protein 1	43286.86719	43401.05	39754.64	43428.71	**42467.81934**	43585.08	45831.29	44466.98	44948.47	**44707.9541**	**0.9499**	**0.0703**
Q61205	Pafah1b3	Platelet-activating factor acetylhydrolase IB subunit alpha1	118458.6797	132973.7	136137.5	118539.9	**126527.4434**	109545.4	120544.8	165835.1	136916.4	**133210.4141**	**0.9498**	**0.6283**
P22315	Fech	Ferrochelatase, mitochondrial	68173.38281	72444.56	75377.7	71871.1	**71966.6875**	72971.84	71244.75	83059.53	75811.35	**75771.86719**	**0.9498**	**0.2511**
Q8BHC4	Dcakd	Dephospho-CoA kinase domain-containing protein	31608.66992	27629.35	28040.15	25488.1	**28191.56592**	31271.21	30734.79	29137.75	27592.19	**29683.98242**	**0.9497**	**0.3633**
Q6PB44	Ptpn23	Tyrosine-protein phosphatase non-receptor type 23	25764.47266	23180.47	20924.97	19150.45	**22255.09082**	25467.11	25547.97	21296.8	21427.74	**23434.90527**	**0.9497**	**0.5505**
P56873	Znrd2	Protein ZNRD2	32233.10547	30462.81	32022.59	31251.07	**31492.39307**	35242.73	37158.99	31721.26	28528.3	**33162.82178**	**0.9496**	**0.4254**
Q9DCD0	Pgd	6-phosphogluconate dehydrogenase, decarboxylating	105336.3203	110659.8	108274.7	95172.19	**104860.752**	107486.8	110556.4	110434.5	113228.5	**110426.5723**	**0.9496**	**0.1735**
P39087	Grik2	Glutamate receptor ionotropic, kainate 2	9227.100586	12613.48	14111.47	11787.98	**11935.00952**	14571.32	12553.21	11999.49	11150.58	**12568.65186**	**0.9496**	**0.6316**
Q8K0T4	Katnal1	Katanin p60 ATPase-containing subunit A-like 1	34340.26563	37007.18	42292.16	38951.35	**38147.73633**	39552.37	37254.27	43146.56	40759.57	**40178.19141**	**0.9495**	**0.3658**
Q9CQF0	Mrpl11	Large ribosomal subunit protein uL11m	28913.29102	29374.5	30381.5	34462.46	**30782.93799**	29808.25	32334.05	34628.51	32915.44	**32421.56299**	**0.9495**	**0.3482**
Q8CFV4	Nrn1	Neuritin	17789.98633	14833.63	10915.18	12631.88	**14042.66992**	15381.95	13984.69	15026.43	14775.14	**14792.052**	**0.9493**	**0.6382**
Q9DBJ1	Pgam1	Phosphoglycerate mutase 1	1512825	1554014	1494767	1550544	**1528037.438**	1519270	1482103	1630966	1806483	**1609705.531**	**0.9493**	**0.3135**
Q8C0M9	Asrgl1	Isoaspartyl peptidase/L-asparaginase	256286.2813	265856.8	229029.5	252103.6	**250819.0586**	282628.4	272350.1	247477.4	254476.8	**264233.1797**	**0.9492**	**0.2773**
Q91YJ2	Snx4	Sorting nexin-4	30778.05859	27491.96	29607.88	29405.27	**29320.7915**	31253.06	31493.74	31369.64	29444.75	**30890.29785**	**0.9492**	**0.1093**
Q8BK63	Csnk1a1	Casein kinase I isoform alpha	27415.41797	35741.35	30121.15	28535.32	**30453.31201**	32296.82	36455.41	31668.25	27920.32	**32085.20068**	**0.9491**	**0.5449**
P39053	Dnm1	Dynamin-1	1403951.75	1417462	1344926	1390905	**1389311.344**	1503111	1485120	1463531	1403399	**1463790.094**	**0.9491**	**0.0321**
O35954	Pitpnm1	Membrane-associated phosphatidylinositol transfer protein 1	90289.39063	89217.75	87700.17	88373.88	**88895.29688**	89231.21	99193.77	92986.66	93267.88	**93669.87891**	**0.9490**	**0.0665**
Q80U28	Madd	MAP kinase-activating death domain protein	21649.97852	19333.63	19341.41	20254.35	**20144.84229**	20695.21	20339.1	20735.86	23138.81	**21227.24414**	**0.9490**	**0.2470**
G5E829	Atp2b1	Plasma membrane calcium-transporting ATPase 1	181652.4219	185451.5	168196.7	198183	**183370.9102**	200326.1	198945.2	194013.5	179658.2	**193235.75**	**0.9489**	**0.2513**
Q8BR92	Palm2	Paralemmin-2	49108.8125	48421.75	34804.15	48623.76	**45239.61914**	53824.95	55035.86	39855.55	41994.61	**47677.74121**	**0.9489**	**0.6588**
Q8CG76	Akr7a2	Aflatoxin B1 aldehyde reductase member 2	76255.28906	62636.54	71249.29	54279.95	**66105.2666**	65670.7	73152.05	66496.09	73353.31	**69668.03906**	**0.9489**	**0.5241**
Q8VIJ6	Sfpq	Splicing factor, proline- and glutamine-rich	138250.0938	121846.1	114216.3	126379.7	**125173.0527**	130729.9	143691.9	126074.7	127280.7	**131944.3008**	**0.9487**	**0.3343**
Q0KK55	Kndc1	Kinase non-catalytic C-lobe domain-containing protein 1	5570.448242	5104.877	3793.277	2004.697	**4118.324768**	4060.904	4597.132	4288.562	4421.623	**4342.055237**	**0.9485**	**0.7908**
Q9DBS5	Klc4	Kinesin light chain 4	109362.5469	120214.9	106795	109206.3	**111394.6777**	114738.5	123266.8	112055.4	119726.7	**117446.8477**	**0.9485**	**0.1725**
Q3UYC0	Ppm1h	Protein phosphatase 1H	23520.40625	26175.63	21977.79	24140.49	**23953.57959**	24633.74	27183.67	24550.85	24716.71	**25271.24023**	**0.9479**	**0.2676**
O35621	Pmm1	Phosphomannomutase 1	12799.61133	17169.83	18551.82	19363.57	**16971.20654**	17574.78	21070.42	17801.11	15176.18	**17905.62085**	**0.9478**	**0.6401**
Q6NVG1	Lpcat4	Lysophospholipid acyltransferase LPCAT4	41919.03906	40280.54	35773.07	38624.54	**39149.29785**	46055.36	41232.02	41874.88	36069.02	**41307.81836**	**0.9477**	**0.4088**
Q8CDN6	Txnl1	Thioredoxin-like protein 1	231305.6406	241842.7	259420.5	238932.6	**242875.375**	232354.6	244867	277264.2	270677.5	**256290.8164**	**0.9477**	**0.3122**
Q8C729	Hycc2	Hyccin 2	28050.54297	34236.36	27334.35	25967.7	**28897.23779**	25426.91	35362.79	32072.79	29111.46	**30493.48779**	**0.9477**	**0.5892**
P42225	Stat1	Signal transducer and activator of transcription 1	25693.05859	20701.36	30887.2	20600.2	**24470.45508**	24816.75	24784.21	23965.97	29723	**25822.48242**	**0.9476**	**0.6437**
Q80UM7	Mogs	Mannosyl-oligosaccharide glucosidase	30736.45117	22976.97	32917.73	33366.94	**29999.52148**	32922.55	29737.82	33991.61	29980.26	**31658.06104**	**0.9476**	**0.5521**
Q922B1	Macrod1	ADP-ribose glycohydrolase MACROD1	39866.59375	43130.64	38525.11	48793.76	**42579.02441**	42511.03	42565.38	48733.61	45934.85	**44936.21777**	**0.9475**	**0.4216**
Q9D7I5	Lhpp	Phospholysine phosphohistidine inorganic pyrophosphate phosphatase	11274.72754	11416.79	5628.513	4489.471	**8202.376221**	7736.299	8937.605	8262.642	9690.613	**8656.789795**	**0.9475**	**0.8169**
Q99NH0	Ankrd17	Ankyrin repeat domain-containing protein 17	8654.861328	4083.781	3590.642	5687.653	**5504.234375**	8405.646	6906.19	2627.535	5298.947	**5809.579651**	**0.9474**	**0.8620**
Q9WV02	Rbmx	RNA-binding motif protein, X chromosome	13671.29199	12179.37	15385.18	12183.54	**13354.8457**	18763.45	18384.18	5764.585	13473.3	**14096.37915**	**0.9474**	**0.8201**
P59764	Dock4	Dedicator of cytokinesis protein 4	31064.74219	22798.13	15383.03	21815.27	**22765.29492**	19577.21	27264.75	23896.41	25385.46	**24030.9585**	**0.9473**	**0.7379**
Q9CXZ1	Ndufs4	NADH dehydrogenase [ubiquinone] iron-sulfur protein 4, mitochondrial	214936.9844	201101.8	195023.9	203011.6	**203518.5781**	222106.3	208045.8	225769.9	203504.9	**214856.7188**	**0.9472**	**0.1467**
O08709	Prdx6	Peroxiredoxin-6	1196272.5	1400402	1155382	1160751	**1228201.813**	1292288	1324726	1227437	1342187	**1296659.438**	**0.9472**	**0.3215**
Q99KY4	Gak	Cyclin-G-associated kinase	29670.75781	31090.07	34194.04	33465.05	**32104.97949**	33340.43	32604.13	39138.06	30513.42	**33899.0083**	**0.9471**	**0.4304**
P12023	App	Amyloid-beta precursor protein	45800.75391	31818.25	44403.57	43126.95	**41287.38135**	38593.43	35571.7	49952.14	50270.57	**43596.95898**	**0.9470**	**0.6591**
Q9CQK7	Rwdd1	RWD domain-containing protein 1	10998.7041	15862.41	9073.011	12781.29	**12178.85327**	12541.09	12957.3	12231.31	13711.23	**12860.23218**	**0.9470**	**0.6610**
Q9JKV1	Adrm1	Proteasomal ubiquitin receptor ADRM1	48999.45313	48990.05	38916.33	45417.16	**45580.74902**	55397.96	41978.25	42075.53	53073.66	**48131.35156**	**0.9470**	**0.5727**
Q80TL0	Ppm1e	Protein phosphatase 1E	37392.23438	43918.63	36116.97	35553.82	**38245.41211**	41715.67	40122.67	37136.79	42576.47	**40387.90137**	**0.9470**	**0.3819**
O89106	Fhit	Bis(5'-adenosyl)-triphosphatase	18657.24609	19595.31	22674.24	14110.4	**18759.30005**	20959.53	22374.89	18244.15	17666.24	**19811.2041**	**0.9469**	**0.6333**
Q8VCF0	Mavs	Mitochondrial antiviral-signaling protein	15868.95117	16262.1	14168.31	16992.65	**15823.00269**	14734.79	16210.27	19842.08	16054.44	**16710.39526**	**0.9469**	**0.5038**
Q9ESX5	Dkc1	H/ACA ribonucleoprotein complex subunit DKC1	18918.0957	11289.04	18070.34	16581.64	**16214.7793**	18857.57	16159.89	16465.51	17018.52	**17125.37354**	**0.9468**	**0.6337**
Q9D967	Mdp1	Magnesium-dependent phosphatase 1	37327.17578	40252.95	44288.76	39026.03	**40223.72852**	42390.36	43200.28	46253.46	38090.06	**42483.54004**	**0.9468**	**0.3526**
Q9D1P0	Mrpl13	Large ribosomal subunit protein uL13m	9424.722656	8129.677	5234.158	9005.876	**7948.608521**		8389.307	8985.772	7812.938	**8396.005859**	**0.9467**	**0.7139**
Q8CC35	Synpo	Synaptopodin	9409.663086	7156.637	8049.242	9516.834	**8533.094116**	5930.784	12926.09	6451.143	10746.49	**9013.626831**	**0.9467**	**0.7968**
Q8CC88	Vwa8	von Willebrand factor A domain-containing protein 8	68597.9375	74702.98	71965.84	79639.73	**73726.61914**	76456.64	76434.06	80457.41	78181.88	**77882.5**	**0.9466**	**0.1501**
Q8BTG3	Tcp11l1	T-complex protein 11-like protein 1	43761.48828	44382.72	38726.82	38886.93	**41439.49023**	44057.32	45112.39	42495.39	43441.31	**43776.60352**	**0.9466**	**0.1995**
Q91YT0	Ndufv1	NADH dehydrogenase [ubiquinone] flavoprotein 1, mitochondrial	743324.4375	710119.1	670521.6	705060.9	**707256.5**	768823.7	782865.5	708919.9	728204	**747203.2813**	**0.9465**	**0.1301**
O54950	Prkag1	5'-AMP-activated protein kinase subunit gamma-1	24455.68945	20430.16	23363.48	14980.27	**20807.40063**	23578.37	24730.98	21632.06	17998.56	**21984.9917**	**0.9464**	**0.6644**
Q8R0F6	Ilkap	Integrin-linked kinase-associated serine/threonine phosphatase 2C	12152.4541	13925.76	10893.37	7013.842	**10996.35608**	12544.01	12191.6	11637.29	10102.86	**11618.93628**	**0.9464**	**0.7040**
P47941	Crkl	Crk-like protein	161315.9688	153567.3	160397.4	147774.7	**155763.8281**	166321.1	159135.3	159375	173543.7	**164593.7813**	**0.9464**	**0.1072**
P06837	Gap43	Neuromodulin	57869.41797	46548.87	41766.75	45267.5	**47863.13281**	48251.68	48242.07	74774.61	31057.21	**50581.39355**	**0.9463**	**0.7882**
P35285	Rab22a	Ras-related protein Rab-22A	29985.5	27561.25	36114.77	27754.91	**30354.10791**	34812.58	25094.6	36411.95	32000.75	**32079.97168**	**0.9462**	**0.6091**
Q3UHC7	Dab2ip	Disabled homolog 2-interacting protein	9645.49707	9726.309	5129.922	9006.711	**8377.109741**	8844.911	12422.13	6252.019	7895.76	**8853.704712**	**0.9462**	**0.7889**

Q8K4R4	Pitpnc1	Cytoplasmic phosphatidylinositol transfer protein 1	29233.11914	30789.86	25142.63	25420.66	**27646.56738**	31565.61	28285.73	27690.22	29348.96	**29222.63086**	**0.9461**	**0.3742**
P47791	Gsr	Glutathione reductase, mitochondrial	35475.55859	39397.49	41320.03	39696.13	**38972.30176**	40376.49	39103.54	42187.03	43111.01	**41194.5166**	**0.9461**	**0.1970**
Q9D1H6	Ndufaf4	NADH dehydrogenase [ubiquinone] 1 alpha subcomplex assembly factor 4	15087.99316	16801.3	17517.46	16081.09	**16371.96387**	18073.4	16905.51	16870.72	17383.22	**17308.2124**	**0.9459**	**0.1635**
Q9CZU6	Cs	Citrate synthase, mitochondrial	2892272	2965737	2947642	2904800	**2927612.625**	3221955	3269752	2877188	3011627	**3095130.375**	**0.9459**	**0.1230**
P14152	Mdh1	Malate dehydrogenase, cytoplasmic	4757483.5	5162594	4441184	4836239	**4799374.875**	4923532	4986056	5069524	5320387	**5074874.5**	**0.9457**	**0.1600**
Q8K4Z5	Sf3a1	Splicing factor 3A subunit 1	16946.28906	17145.37	16578.67	17239.86	**16977.54736**	17705.21	18243.22	19763.71	16098.83	**17952.74658**	**0.9457**	**0.2524**
P50544	Acadvl	Very long-chain specific acyl-CoA dehydrogenase, mitochondrial	55039.23828	58757.22	63985.23	54842.93	**58156.1543**	59602.17	62112.4	66073.03	58212.13	**61499.93262**	**0.9456**	**0.2696**
Q8BHG2	Czib	CXXC motif containing zinc binding protein	19361.35352	30060.66	28894.13	23043.51	**25339.91113**	22721.96	26716.01	24627.3	33135.7	**26800.24463**	**0.9455**	**0.6812**
Q62421	Sh3gl3	Endophilin-A3	29057.0918	23994.59	22382.79	26217.98	**25413.11035**	23703.03	25564.59	30837.44	27416.64	**26880.4248**	**0.9454**	**0.5108**
Q8CCJ4	Amer2	APC membrane recruitment protein 2	24338.16992	22953.79	18898.86	24016.54	**22551.84033**	26059.06	26804.2	20428.27	22127.4	**23854.73047**	**0.9454**	**0.5353**
Q8QZY1	Eif3l	Eukaryotic translation initiation factor 3 subunit L	32824.45313	27702.17	29468.41	27710.34	**29426.34326**	30441.76	31202.96	33606.93	29278.72	**31132.59082**	**0.9452**	**0.3028**
O35286	Dhx15	ATP-dependent RNA helicase DHX15	44439.13281	42742.08	44690.98	42527.93	**43600.0332**	45507.82	48693.15	45062.17	45251.45	**46128.64648**	**0.9452**	**0.0489**
Q61191	Hcfc1	Host cell factor 1	20507.12109	23047.26	16332.75	19900.19	**19946.83081**	21518.35	21764.55	20338.38	20793.72	**21103.75049**	**0.9452**	**0.4471**
Q8VCM8	Ncln	BOS complex subunit NCLN	21203.13672	18044.12	17161.87	18141.8	**18637.7334**	18663.93	21108.97	19565.06	19546.3	**19721.06738**	**0.9451**	**0.3286**
Q8CJG0	Ago2	Protein argonaute-2	4177.722168	3917.813	4131.727	4755.08	**4245.585449**	3917.678	5937.63	4871.535	3243.056	**4492.475037**	**0.9450**	**0.7011**
Q9DCP2	Slc38a3	Sodium-coupled neutral amino acid transporter 3	57179.22266	61630.74	53815.43	74243.45	**61717.21094**	72306.62	68645.73	64279.59	55996.24	**65307.04395**	**0.9450**	**0.5510**
Q9Z315	Sart1	U4/U6.U5 tri-snRNP-associated protein 1		5458.677	7059.781		**6259.229004**				6623.5	**6623.500488**	**0.9450**	
O35658	C1qbp	Complement component 1 Q subcomponent-binding protein, mitochondria	53126.38281	46502.47	48552.44	46282.78	**48616.0166**	52476.69	54918.83	46429.54	51960.39	**51446.36328**	**0.9450**	**0.2819**
Q8R1S0	Coq6	Ubiquinone biosynthesis monooxygenase COQ6, mitochondrial	10405.96973	6693.231	10352.65	12087.14	**9884.746216**	9655.215	9286.492	11623.04	11281.78	**10461.63062**	**0.9449**	**0.6674**
Q9ERN0	Scamp2	Secretory carrier-associated membrane protein 2	2510.584229	4448.174	1326.378	2682.654	**2741.947571**	3250.635	4937.761	2265.263	1154.9	**2902.139862**	**0.9448**	**0.8813**
Q9DCX2	Atp5pd	ATP synthase subunit d, mitochondrial	515637.3438	483717.2	535710.3	509964.1	**511257.2266**	534558.9	528652.8	565509.3	535784.8	**541126.4531**	**0.9448**	**0.0695**
Q80TL4	Phf24	PHD finger protein 24	85516.00781	91363.99	82974.52	87628.59	**86870.7793**	94157.34	88264.15	92864.94	92506.27	**91948.17383**	**0.9448**	**0.0593**
Q9Z2W9	Gria3	Glutamate receptor 3	36672.58203	33486.17	36245.62	35059.84	**35366.05273**	38939.63	37952.88	37797.94	35049.11	**37434.88965**	**0.9447**	**0.1085**
Q8VBY2	Camkk1	Calcium/calmodulin-dependent protein kinase kinase 1	34565.59375	33258.37	31871.44	31623.61	**32829.75244**	32971.79	35544.69	34635.76	35855.48	**34751.92969**	**0.9447**	**0.0868**
Q9D020	Nt5c3a	Cytosolic 5'-nucleotidase 3A	15881.15918	17123.22	12155.13	16653.02	**15453.13208**	15794.17	16685.5	16118.86	16846.39	**16361.23071**	**0.9445**	**0.4617**
Q8BKX1	Baiap2	Brain-specific angiogenesis inhibitor 1-associated protein 2	17290.67383	15946.91	16396.98	15429.55	**16266.02661**	19008.08	16849.26	17401.12	15630.94	**17222.34863**	**0.9445**	**0.2793**
Q8VCN9	Tbcc	Tubulin-specific chaperone C	28768.57227	32322.32	23747.71	26917.25	**27938.96143**	30713.97	28852.57	30317.55	28445.93	**29582.50537**	**0.9444**	**0.4143**
Q9DC61	Pmpca	Mitochondrial-processing peptidase subunit alpha	27615.71484	30203.63	24803.08	27569.87	**27548.07422**	29462.93	28709.79	29759.84	28742.17	**29168.68359**	**0.9444**	**0.2028**
O54991	Cntnap1	Contactin-associated protein 1	313377.625	310433.8	306933.3	323112.4	**313464.2969**	314919.6	325138.7	351230.8	336354.6	**331910.9219**	**0.9444**	**0.0737**
Q8K1M6	Dnm1l	Dynamin-1-like protein	306851	323979.8	276421.8	295388.5	**300660.2969**	327275.2	340789.2	303111.8	302252.3	**318357.1016**	**0.9444**	**0.2458**
P60867	Rps20	Small ribosomal subunit protein uS10	102045.9453	108978.4	102677.2	109721.5	**105855.7773**	112763.8	125452.2	107440.1	102696	**112088.0215**	**0.9444**	**0.2849**
Q9Z2X2	Psmd10	26S proteasome non-ATPase regulatory subunit 10	6715.376953		5459.427		**6087.4021**	6750.08	7041.376	6768.129	5225.294	**6446.220093**	**0.9443**	**0.6481**
Q00560	Il6st	Interleukin-6 receptor subunit beta	10906.20801	9711.899	11579.01	11199.15	**10849.06812**	12047.58	12757.17	11467.3	9686.183	**11489.55835**	**0.9443**	**0.4376**
Q9CZ28	Snf8	Vacuolar-sorting protein SNF8	15275.85547	15466.98	14753.22	13788.09	**14821.03442**	15965.04	14966.05	17675.08	14184.03	**15697.54956**	**0.9442**	**0.3379**
Q9QZ06	Tollip	Toll-interacting protein	70683.78125	62087.41	62600.54	67737.23	**65777.24121**	68010.7	68840.77	73400.48	68437.37	**69672.33008**	**0.9441**	**0.1592**
Q8R3Q2	Ppp6r2	Serine/threonine-protein phosphatase 6 regulatory subunit 2	25935.72852	26764.36	18294.38	26092.36	**24271.70801**	25868.09	25548.69	24765.12	26669.85	**25712.93604**	**0.9439**	**0.5062**
Q9CR09	Ufc1	Ubiquitin-fold modifier-conjugating enzyme 1	53994.30469	57802.29	56989.25	45822.39	**53652.05859**	54013.03	61343.97	55572.27	56428.11	**56839.3457**	**0.9439**	**0.3521**
Q8VCD6	Reep2	Receptor expression-enhancing protein 2	34497.77344	34245.39	33122.71	34451.71	**34079.39648**	36944.01	39538.55	35187.95	32759.47	**36107.49414**	**0.9438**	**0.2158**
Q6PB66	Lrpprc	Leucine-rich PPR motif-containing protein, mitochondrial	70567.65625	69484.32	67663.73	66994.13	**68677.45898**	72990.78	69612.57	75829.4	72626.83	**72764.89453**	**0.9438**	**0.0355**
Q8R3I2	Mboat2	Lysophospholipid acyltransferase 2	11158.10742	12160.28	9776.562	11111.25	**11051.54932**	14196.68	11499.14	10943.69	10200.73	**11710.0603**	**0.9438**	**0.5340**
Q8BP40	Acp6	Lysophosphatidic acid phosphatase type 6	19820.25586	17377.19	19054.17	17901.34	**18538.23926**	17881.94	22431.74	19416.23	18843.44	**19643.33887**	**0.9437**	**0.3645**
P24549	Aldh1a1	Aldehyde dehydrogenase 1A1	158160.375	150789.1	152719.8	128740.9	**147602.5371**	158156.7	159276.9	153101.3	155153.2	**156422.0234**	**0.9436**	**0.2317**
P38647	Hspa9	Stress-70 protein, mitochondrial	556193	549554.1	529476.3	584602.6	**554956.4844**	558419.1	548960.3	615535.8	629843.3	**588189.625**	**0.9435**	**0.2022**
Q6PA06	Atl2	Atlastin-2	22703.69531	21484.71	23717.97	24004.36	**22977.68262**	24561.46	25808.58	23908.93	23161.72	**24360.17188**	**0.9432**	**0.1348**
Q9R1P4	Psma1	Proteasome subunit alpha type-1	91608.625	100554.6	97475.26	96435.63	**96518.5293**	98699.95	94595.97	114090.1	101932.4	**102329.6152**	**0.9432**	**0.2524**
P19157	Gstp1	Glutathione S-transferase P 1	365151.0625	373136.2	345515	336153.2	**354988.8438**	367129.4	349398.9	390234.6	398803.4	**376391.5781**	**0.9431**	**0.1798**
Q5SQX6	Cyfip2	Cytoplasmic FMR1-interacting protein 2	61847.56641	61096.65	55564.09	54552.33	**58265.16016**	67116.73	66675.48	55998.46	57341.65	**61783.08008**	**0.9431**	**0.3544**
Q8VE38	Oxnad1	Oxidoreductase NAD-binding domain-containing protein 1	25732.26758	27619.89	28453.68	30767.31	**28143.28857**	28258.72	26326.58	28817.74	35973.13	**29844.04102**	**0.9430**	**0.4974**
Q9CZR8	Tsfm	Elongation factor Ts, mitochondrial	25601.14258	27835.93	24274.22	22684.17	**25098.86572**	27757.57	27832.79	25749.36	25132.74	**26618.1123**	**0.9429**	**0.2837**
P59672	Anks1a	Ankyrin repeat and SAM domain-containing protein 1A	9575.866211	9748.991	11830.2	7585.701	**9685.190186**	10786.82	12266.7	10669.05	7371.89	**10273.61511**	**0.9427**	**0.6780**
Q3U2V3	Nudt18	8-oxo-dGDP phosphatase NUDT18	84109.46094	72832.2	75630.96	90750.91	**80830.88281**	96013.05	88510.97	76114.66	82349.2	**85746.9707**	**0.9427**	**0.4365**
Q9WUZ9	Entpd5	Ectonucleoside triphosphate diphosphohydrolase 5	8552.894531	6094.029	7077.814	6961.34	**7171.519409**	9681.465	7342.64	6016.886	7393.069	**7608.515015**	**0.9426**	**0.6502**
Q3SXD3	Hddc2	5'-deoxynucleotidase HDDC2	25759.40625	20294.1	20329.79	8456.424	**18709.93018**	20591.33	20070.42	20663.74	18075.46	**19850.2373**	**0.9426**	**0.7684**
Q6NTA4	Rragb	Ras-related GTP-binding protein B	47103.0625	51849.17	48441.39	48941.79	**49083.85449**	49530.92	51068.38	52480.9	55227.19	**52076.84668**	**0.9425**	**0.1053**
Q9DBR3	Armc8	Armadillo repeat-containing protein 8	19427.00391	19542.21	18926.3	22278.08	**20043.39697**	24080.68	20676.52	20811.66	19494.77	**21265.90918**	**0.9425**	**0.3627**
Q9CQF9	Pcyox1	Prenylcysteine oxidase 1	59347.99609	61988.09	52058.68	53758.79	**56788.38672**	59738.73	64701.42	61928.66	54643.14	**60252.9873**	**0.9425**	**0.3143**
Q8CEE7	Rdh13	Retinol dehydrogenase 13	6705.650879	7763.387	186.297	6636.736	**5323.017849**	8239.455	5469.98	2992.132	5892.353	**5648.480103**	**0.9424**	**0.8784**
Q9ET30	Tm9sf3	Transmembrane 9 superfamily member 3	21865.06055	22217.67	22139.36	19506.73	**21432.20508**	21916.21	20700.34	25004.49	23358.02	**22744.76514**	**0.9423**	**0.2901**
Q3UPL0	Sec31a	Protein transport protein Sec31A	64804.19531	62547	58022.82	67032.54	**63101.6377**	68541.45	70248.03	63353.7	65731.05	**66968.55664**	**0.9423**	**0.1662**
P12367	Prkar2a	cAMP-dependent protein kinase type II-alpha regulatory subunit	61471.62891	68731.59	59751.62	62864.22	**63204.76465**	67228.3	69697.13	63413.29	68000.42	**67084.78223**	**0.9422**	**0.1511**
P70404	Idh3g	Isocitrate dehydrogenase [NAD] subunit gamma 1, mitochondrial	452556.25	480687.2	383488.5	450446.8	**441794.6797**	503310.1	494349.8	445303.2	433121.5	**469021.1563**	**0.9420**	**0.3528**
P84086	Cplx2	Complexin-2	80976.28906	86011.98	71882.39	85652.54	**81130.80078**	87400.6	69473.28	95228.14	92425.47	**86131.87305**	**0.9419**	**0.4807**
Q99MN9	Pccb	Propionyl-CoA carboxylase beta chain, mitochondrial	117883.0156	119363.6	123067.9	103220.2	**115883.6836**	127281.1	115976.6	125144.3	123721.7	**123030.9238**	**0.9419**	**0.2034**
P62881	Gnb5	Guanine nucleotide-binding protein subunit beta-5	67399.99219	63767.43	61968.68	71469.63	**66151.43066**	66628.64	75495.98	70516.71	68289.91	**70232.8125**	**0.9419**	**0.2023**
Q9D0M5	Dynll2	Dynein light chain 2, cytoplasmic	153737	151074	132593.9	134612.7	**143004.4063**	160301.8	143145.8	149296.5	154621.1	**151841.3008**	**0.9418**	**0.2282**

Q8BHZ0	Cyria	CYFIP-related Rac1 interactor A	32872.84375	21339.5	29742.82	34656.45	**29652.90381**	32353.25	30566.57	33616.77	29412.94	**31487.38281**	**0.9417**	**0.5750**
Q61823	Pdcd4	Programmed cell death protein 4	6852.73291		3762.513		**5307.623169**				5636.473	**5636.473145**	**0.9417**	
P48758	Cbr1	Carbonyl reductase [NADPH] 1	234280.125	248915.2	224887.5	239757.8	**236960.168**	247207.7	252816.6	254449.1	252103	**251644.1133**	**0.9416**	**0.0317**
Q8BYY4	Ttc39b	Tetratricopeptide repeat protein 39B	15815.11328	12362.86	17034.95	15054.57	**15066.87183**	13660.78	18103.17	16546.94	15696.1	**16001.74902**	**0.9416**	**0.5160**
Q9R0X4	Acot9	Acyl-coenzyme A thioesterase 9, mitochondrial	96828.07031	96088.94	100482.9	102426.9	**98956.70703**	102771.6	105539.8	106988.7	105095.9	**105099.0215**	**0.9416**	**0.0124**
O54983	Crym	Ketimine reductase mu-crystallin	15378.32813	10899.77	6462.386	12559.6	**11325.01978**	18048.33	13320.58	7272.224	9477.628	**12029.69177**	**0.9414**	**0.8228**
Q9R008	Mvk	Mevalonate kinase	30360.00586	37462.11	30900.96	28027.9	**31687.74365**	35126.91	38027.58	31046.32	30439.77	**33660.14404**	**0.9414**	**0.4928**
Q8BGR9	Ublcp1	Ubiquitin-like domain-containing CTD phosphatase 1	27332.67969	34035.16	30710.09	35660.56	**31934.625**	32779.88	33310.77	32609.88	36989.08	**33922.40283**	**0.9414**	**0.3840**
Q80Y98	Ddhd2	Phospholipase DDHD2	9476.205078	8347.433	7296.944	7339.225	**8114.951782**	8794.421	9180.126	8491.909	8016.511	**8620.741821**	**0.9413**	**0.4093**
Q810C0	Slitrk2	SLIT and NTRK-like protein 2	2130.504883	4183.553	3737.113	5394.272	**3861.360718**	3783.502			4421.272	**4102.386963**	**0.9412**	**0.8266**
Q8VHI6	Wasf3	Actin-binding protein WASF3	47420.78906	42865.78	34477.81	33723.39	**39621.94141**	46450.83	42471.91	37807.93	41655.9	**42096.64258**	**0.9412**	**0.5356**
P48320	Gad2	Glutamate decarboxylase 2	158469.7031	162721.6	132681	173288	**156790.082**	158138.5	159657.1	169139.2	179410.2	**166586.2383**	**0.9412**	**0.3617**
P45591	Cfl2	Cofilin-2	226849.5313	235089.8	284501.8	259336.4	**251444.4102**	226515.8	226378.6	290293.3	325597.7	**267196.3516**	**0.9410**	**0.5919**
Q9JI10	Stk3	Serine/threonine-protein kinase 3	19835.05664	21913.04	16974.59	16629.38	**18838.01709**	22590.7	25218.53	17577.98	14685.37	**20018.14551**	**0.9410**	**0.6763**
Q922J6	Tspan2	Tetraspanin-2	31652.54102	30892.77	24811.61	24129	**27871.47949**	29703.69	28785.12	31498.61	28488.72	**29619.03125**	**0.9410**	**0.4346**
Q3TLP5	Echdc2	Enoyl-CoA hydratase domain-containing protein 2, mitochondrial	8546.260742	9949.65	10039.09	7860.985	**9098.996094**	8855.357	8140.126	10492.96	11190.18	**9669.656372**	**0.9410**	**0.5437**
Q9JJK2	Lancl2	LanC-like protein 2	50781.57422	52338.59	52046.14	48319.77	**50871.52051**	52639.32	55364.23	53381.51	54870.86	**54063.98047**	**0.9410**	**0.0286**
P35283	Rab12	Ras-related protein Rab-12	23378.48633	23284.33	23403.34	25073.88	**23785.00879**	24640.54	26264.4	28407.04	21807.08	**25279.76709**	**0.9409**	**0.3442**
Q80XI4	Pip4k2b	Phosphatidylinositol 5-phosphate 4-kinase type-2 beta	27700.72266	27319.69	26062.97	29303.2	**27596.64453**	31919.11	29747.24	27193.49	28479.16	**29334.75**	**0.9407**	**0.2003**
Q9D6U8	Fam162a	Protein FAM162A	88076.76563	80682.36	82581.59	83339.03	**83669.9375**	87502.15	106547.5	81254.59	80466.74	**88942.73438**	**0.9407**	**0.4330**
Q62059	Vcan	Versican core protein	170910.2188	163694.1	170202.6	173097.8	**169476.1563**	160640.1	156976.9	199881	203178.8	**180169.1836**	**0.9407**	**0.4265**
P62814	Atp6v1b2	V-type proton ATPase subunit B, brain isoform	803989.3125	863734.5	837765.7	848573.4	**838515.7344**	845238.8	868297.7	948133.1	904045.6	**891428.7969**	**0.9406**	**0.0859**
O88532	Zfr	Zinc finger RNA-binding protein	13040.41504	15447.99	11091.54	11910.37	**12872.57666**	14242.24	14454.35	14358.17	11688.04	**13685.698**	**0.9406**	**0.5090**
Q3V1U8	Elmod1	ELMO domain-containing protein 1	12237.08789	11350.58	12539.82	12508.27	**12158.94067**	15818.08	15319.21	10024.89	10546.93	**12927.27808**	**0.9406**	**0.6392**
Q9JJC6	Rilpl1	RILP-like protein 1	15820.7168	15923.84	12685.32	11458.61	**13972.12109**	15156.89	16033.82	12537.25	15697.44	**14856.35229**	**0.9405**	**0.5446**
Q5PR69	Cracd	Capping protein-inhibiting regulator of actin dynamics	7706.375	9089.701	6254.067	7143.864	**7548.501709**	6176.698	8825.091	7094.29	10010.7	**8026.69397**	**0.9404**	**0.6634**
Q8BKY8	Mterf2	Transcription termination factor 2, mitochondrial	8971.978516	11538.39	9688.717	7288.643	**9371.931885**	9128.208	11180.58	10675.93	8878.008	**9965.682617**	**0.9404**	**0.5913**
Q9CQE5	Rgs10	Regulator of G-protein signaling 10	13720.18457	10995.84	9387.602	14012.44	**12029.01685**	16225.41	11060.65	11801.34	12077.11	**12791.12866**	**0.9404**	**0.6527**
Q8BK75	Elp6	Elongator complex protein 6	7092.323242	16785.25	9041.411		**10972.99414**	12070.47	12293.75	10640.4		**11668.20671**	**0.9404**	**0.8284**
Q6NVE8	Wdr44	WD repeat-containing protein 44	13101.53711	12424.38	8832.613	11375.54	**11433.5166**	12979.69	11492.66	12318.15	11850.77	**12160.31665**	**0.9402**	**0.4907**
Q9CPQ3	Tomm22	Mitochondrial import receptor subunit TOM22 homolog	103586.9844	101636.4	92649.71	104284.7	**100539.459**	103067.1	102043.7	117639.2	104989.8	**106934.9395**	**0.9402**	**0.2059**
Q9D1K2	Atp6v1f	V-type proton ATPase subunit F	100837.6875	106479.1	92884.07	96756.36	**99239.31445**	105348.9	89716.72	109985.3	117159.6	**105552.6113**	**0.9402**	**0.3688**
Q922Y1	Ubxn1	UBX domain-containing protein 1	21372.13477	20894.26	23019.66	18383.11	**20917.28955**	19087.52	26098.64	21702.38	22108.27	**22249.20361**	**0.9401**	**0.4722**
Q9WV34	Mpp2	MAGUK p55 subfamily member 2	30982.29688	31230.46	26469.31	28674.49	**29339.13965**	30388.12	30641.58	31618.41	32190.27	**31209.59424**	**0.9401**	**0.1680**
Q924L1	Letmd1	LETM1 domain-containing protein 1	14000.74707	13718.75	16381.08	11600.94	**13925.3772**	15650.13	14496.91	14111.28	15004.21	**14815.62988**	**0.9399**	**0.4220**
Q8R366	Igsf8	Immunoglobulin superfamily member 8	195411.1875	207910.2	177686.4	184636.6	**191411.0898**	188622.4	190968.6	219022.4	216027.6	**203660.2578**	**0.9399**	**0.2836**
Q9JLR9	Higd1a	HIG1 domain family member 1A, mitochondrial	23962.20703	32320.02	64369.71	27827.66	**37119.89941**	27938.72	37523.63	55453.64	37080.8	**39499.19727**	**0.9398**	**0.8343**
P26040	Ezr	Ezrin	15924.04492	16967.46	16903.69	16071.27	**16466.61841**	18771.91	19790.34	15853.74	15691.75	**17526.93506**	**0.9395**	**0.3599**
Q91X21	Kiaa2013	Uncharacterized protein KIAA2013	23701.93164	48665.39			**36183.65918**	44232.62	62822.03	8506.713		**38520.45508**	**0.9393**	**0.9241**
P46467	Vps4b	Vacuolar protein sorting-associated protein 4B	13883.96582	13760.8	18003.26	12877.2	**14631.30493**	16644.91	14250.79	16019.27	15389.93	**15576.22363**	**0.9393**	**0.4799**
P0DP26;P0DP27;P0DP2	Calm1;Calm2;Calm3	Calmodulin-1;Calmodulin-2;Calmodulin-3	732850.3125	966170	837532.3	748024.5	**821144.2813**	857435.9	766426.8	1071252	801705.1	**874204.9531**	**0.9393**	**0.5635**
Q91YQ3	Csdc2	Cold shock domain-containing protein C2	15089.69629	14943.29	14145.48	12688.14	**14216.65283**	14688.93	14295.44	14777.05	16782.44	**15135.96436**	**0.9393**	**0.2855**
Q61937	Npm1	Nucleophosmin	98740.33594	87046.91	111534.7	91064.43	**97096.5918**	116108.5	94380.13	95294.7	107753.8	**103384.2754**	**0.9392**	**0.4343**
Q9D8U2	Tmem41a	Transmembrane protein 41A	9280.182617	7405.012	7796.743	5666.654	**7537.147949**	9382.424	6562.988	8130.606		**8025.339355**	**0.9392**	**0.6792**
Q6A4J8	Usp7	Ubiquitin carboxyl-terminal hydrolase 7	31873.98047	33646.67	33190.65	30800.16	**32377.86523**	32499.87	36256.15	34511.6	34640.4	**34477.00342**	**0.9391**	**0.0817**
Q8BWP8	B4gat1	Beta-1,4-glucuronyltransferase 1	5248.22998	4868.693	2611.19	4722.314	**4362.606934**	3450.324	4569.073		5916.947	**4645.447998**	**0.9391**	**0.7714**
Q9Z2A0	Pdpk1	3-phosphoinositide-dependent protein kinase 1	22381.16992	18778.15	28932.73	21096.63	**22797.16943**	23379.97	24294.16	23853.09	25587.58	**24278.70068**	**0.9390**	**0.5308**
Q6NXM2	Rcbtb1	RCC1 and BTB domain-containing protein 1	9079.381836	8205.023	5918.681	6132.553	**7333.90979**	8225.593	7467.134	7897.454	7660.966	**7812.786743**	**0.9387**	**0.5687**
Q8BHG1	Nrdc	Nardilysin	28990.97461	27795.53	35605.14	28949.35	**30335.24805**	29308.76	31636.92	30006.98	38327.45	**32320.02734**	**0.9386**	**0.4934**
P61967	Ap1s1	AP-1 complex subunit sigma-1A	25126.43945	22663.85	21194.03	29387.17	**24592.87109**	21627.18	25563.35	36272.42	21349.42	**26203.09131**	**0.9385**	**0.6959**
Q8CGC4	Lsm14b	Protein LSM14 homolog B		5937.176			**5937.17627**	7334.147	6699.134	4160.708	7110.483	**6326.118164**	**0.9385**	
P26443	Glud1	Glutamate dehydrogenase 1, mitochondrial	1666277.125	1759146	1683729	1722505	**1707914.344**	1829974	1813613	1906969	1729690	**1820061.625**	**0.9384**	**0.0365**
Q7TSQ8	Pdpr	Pyruvate dehydrogenase phosphatase regulatory subunit, mitochondrial	63361.4375	56750.43	62244.97	62411.59	**61192.10645**	66596.52	65393.9	62180.73	66728.37	**65224.87891**	**0.9382**	**0.0705**
A2A5Y0;O76009;Q1452	Krt31;Krt33b;;Krt33a;;;	Keratin, type I cuticular Ha1;Keratin, type I cuticular Ha3-II;;Keratin, type I c	55828.39453	55794.19	53051.48	62215.62	**56722.42285**	66227.77	62398.55	57930.79	55293.24	**60462.58496**	**0.9381**	**0.2732**
Q8VEB6	Elac1	Zinc phosphodiesterase ELAC protein 1	7408.713867	6735.341	9959.38	6026.411	**7532.461304**	6625.542	8752.363	7954.51	8788.728	**8030.285767**	**0.9380**	**0.6347**
Q91WJ8	Fubp1	Far upstream element-binding protein 1	31309.89453	37872.1	30673.58	32876.09	**33182.91797**	31957.39	38643.5	36745.4	34159.06	**35376.33838**	**0.9380**	**0.3554**
Q91VK1	Bzw2	eIF5-mimic protein 1	3497.49707	1632.634	3125.971		**2752.033895**		2934.037			**2934.036865**	**0.9380**	
Q6P1B1	Xpnpep1	Xaa-Pro aminopeptidase 1	49556.72656	57557.82	45333.86	56887.74	**52334.03516**	56166.11	56912.01	57894.42	52250.74	**55805.82031**	**0.9378**	**0.3200**
P47934	Crat	Carnitine O-acetyltransferase	28266.8418	29490.26	32277.45	25717.33	**28937.96924**	30132.9	31709.99	33069.73	28530.59	**30860.80273**	**0.9377**	**0.2958**
Q9D0G0	Mrps30	Large ribosomal subunit protein mL65	8381.585938	12948.83	14767.4	12836.58	**12233.60132**	14264.57	8933.363	14200.79	14794.51	**13048.30664**	**0.9376**	**0.6884**
Q99MK8	Grk2	Beta-adrenergic receptor kinase 1	48447.41016	48309.61	38018.64	44483.63	**44814.82324**	50194.25	50744.18	45784.82	44503.79	**47806.76172**	**0.9374**	**0.3422**
Q8CFI0	Nedd4l	E3 ubiquitin-protein ligase NEDD4-like	10952.88477	12093.26	13112.38	12101.55	**12065.01929**	13725.12	10759.38	14705.52	12292.25	**12870.56763**	**0.9374**	**0.4368**
O35114	Scarb2	Lysosome membrane protein 2	34181.4375	38337.2	23436.97	16459.92	**28103.88232**	28733.43	30821.7	28733.92	31637.12	**29981.54004**	**0.9374**	**0.7226**
Q9QZM0	Ubqln2	Ubiquilin-2	54970.25391	57490.66	62203.4	65838.1	**60125.60254**	60931.21	58250.2	72127.32	65292.43	**64150.29102**	**0.9373**	**0.3395**
Q80V91	Dtx3	Probable E3 ubiquitin-protein ligase DTX3	11075.55273	10922.51	10647.15	7153.557	**9949.691895**	9452.905	10377.62	13072.85	9564.471	**10616.96118**	**0.9372**	**0.6156**

P16406	Enpep	Glutamyl aminopeptidase	10130.70898	6086.058	7031.04	7814.42	**7765.556641**	7903.973	8393.434	9405.882	7444.456	**8286.936035**	**0.9371**	**0.6069**
Q9WTX6	Cul1	Cullin-1	31464.89453	32344.93	28149.63	30902.43	**30715.4707**	31602.22	29734.82	35390.07	34389.05	**32779.03906**	**0.9370**	**0.2390**
Q3TDN2	Faf2	FAS-associated factor 2	47433.39844	42708.85	48638.05	37257.16	**44009.36523**	50443.92	50152.62	43475.23	43795.73	**46966.875**	**0.9370**	**0.3947**
Q9D287	Bcas2	Pre-mRNA-splicing factor SPF27	10444.84961	6950.628	5945.199	8166.216	**7876.723267**	10442.47	10145.43	5580.522	7456.224	**8406.161255**	**0.9370**	**0.7377**
P63030	Mpc1	Mitochondrial pyruvate carrier 1	52495.01563	44588.65	51705.43	32300.69	**45272.44775**	39094.41	59345.06	58322.39	36530.7	**48323.13867**	**0.9369**	**0.7050**
O88983	Stx8	Syntaxin-8	13573.99512	13537.12	12958.46	10250.7	**12580.06836**	14449.72	12082.74	13670.15	13508.8	**13427.85205**	**0.9369**	**0.3974**
Q04447	Ckb	Creatine kinase B-type	4817970.5	5007590	4713946	4600627	**4785033.375**	4604035	4872513	5215068	5738514	**5107532.125**	**0.9369**	**0.2603**
Q9JMG3	Tmub1	Transmembrane and ubiquitin-like domain-containing protein 1			1768.652	5105.422	**3437.03717**			3241.938	4096.927	**3669.432495**	**0.9367**	**0.9050**
P30275	Ckmt1	Creatine kinase U-type, mitochondrial	1332950.75	1359187	1380182	1483061	**1388845**	1487890	1358418	1568283	1517014	**1482901.063**	**0.9366**	**0.1409**
Q9D8N0	Eef1g	Elongation factor 1-gamma	627291.4375	605188.6	521902.1	574378.1	**582190.0703**	637948.4	630010.8	583763.3	635107.6	**621707.5**	**0.9364**	**0.1816**
O08992	Sdcbp	Syntenin-1	36301.91016	36804.2	41040.1	33152.38	**36824.64746**	38455.07	38485.82	38790.72	41569.72	**39325.33301**	**0.9364**	**0.2112**
Q3UJU9	Rmdn3	Regulator of microtubule dynamics protein 3	13069.49023	16007.55	10875.93	13727.17	**13420.03345**	14298.83	15972.46	14427.44	12645.76	**14336.12378**	**0.9361**	**0.4932**
O35551	Rabep1	Rab GTPase-binding effector protein 1	19677.25977	16667.83	19978.59	20053.19	**19094.21973**	19646.67	20937.23	19702.56	21304.86	**20397.82861**	**0.9361**	**0.2050**
Q91WG4	Elp2	Elongator complex protein 2	10116.44629	14545.72	11255.55	12423.61	**12085.33203**	13590.12	12253.39	11756.08	14048.81	**12912.09888**	**0.9360**	**0.4768**
Q71KT5	Tm7sf2	Delta(14)-sterol reductase TM7SF2		8033.871			**8033.870605**	11269.05	7473.713	9355.226	6236.651	**8583.659058**	**0.9359**	
O35988	Sdc4	Syndecan-4	9762.785156	14139.46	13601.23	11863.3	**12341.69409**	12831.72	14798.93	11278.37	13848.5	**13189.38208**	**0.9357**	**0.5203**
Q99LH2	Ptdss1	Phosphatidylserine synthase 1	32005.27148	38128.69	36567.79	27358.35	**33515.02441**	36584.16	35865.52	33292.54	37572.26	**35828.61719**	**0.9354**	**0.4070**
Q924C1	Xpo5	Exportin-5	11462.17969	11412.75	11507.28	10470.35	**11213.14136**	11609.55	14451.19	10353.03	11535.55	**11987.33179**	**0.9354**	**0.4251**
P28738	Kif5c	Kinesin heavy chain isoform 5C	75856.03125	81158.37	84369.87	69208.88	**77648.28711**	83116.03	85502.77	78720.79	84858.88	**83049.61914**	**0.9350**	**0.1895**
P62862	Fau	Ubiquitin-like FUBI-ribosomal protein eS30 fusion protein	168830.9219	163410.8	154939.8	185797.7	**168244.7969**	181526.6	162002.7	186578	189711.8	**179954.7539**	**0.9349**	**0.2411**
Q8BKH7	Mapkap1	Target of rapamycin complex 2 subunit MAPKAP1	7428.348145	6929.204	7215.969	7543.942	**7279.365845**	7031.326	5802.948	8473.742	9837.365	**7786.345337**	**0.9349**	**0.5876**
O35393	Efnb3	Ephrin-B3	37466.5	32117.37	27695.79	24881.54	**30540.29932**	32140.28	31299.2	32876.83	34356.04	**32668.0874**	**0.9349**	**0.4795**
Q9JI59	Jam2	Junctional adhesion molecule B	29513.64453	26181.38	28031.81	24791.54	**27129.59131**	32028.19	31570.32	29273.79	23220.49	**29023.2002**	**0.9348**	**0.4371**
Q91ZW2	Pofut1	GDP-fucose protein O-fucosyltransferase 1	6646.318848				**6646.318848**		7674.699		6545.777	**7110.238037**	**0.9348**	
Q8BNY6	Ncs1	Neuronal calcium sensor 1	60203.63281	60260.13	60062.75	51376.54	**57975.76172**	65917.99	69363.14	61066.95	51749.65	**62024.43457**	**0.9347**	**0.3942**
Q8BG51	Rhot1	Mitochondrial Rho GTPase 1	83144.98438	81929.06	77316.56	80064.67	**80613.82031**	89841.45	88747.24	85195.73	81201.12	**86246.38672**	**0.9347**	**0.0519**
Q8R2Y0	Abhd6	Monoacylglycerol lipase ABHD6	26505.36914	32132.95	27546.64	27194.02	**28344.74609**	33058.86	32704.74	26933.86	28605.13	**30325.64502**	**0.9347**	**0.3569**
Q9D883	U2af1	Splicing factor U2AF 35 kDa subunit	29263.70313	29635.84	29437.59	31133.56	**29867.67236**	26269.04	42561.54	27026.8	31991.19	**31962.13916**	**0.9345**	**0.5994**
Q5SUC9	Sco1	Protein SCO1 homolog, mitochondrial	19152.4668	14623.91	19458.11	20817.4	**18512.97168**	10628.93	16427.2	31115.55	21078.11	**19812.4458**	**0.9344**	**0.7841**
Q91YD9	Wasl	Actin nucleation-promoting factor WASL	22646.7168	23896.37	26980.4	26318.89	**24960.59277**	30080.68	20396.12	31025.61	25363.74	**26716.53564**	**0.9343**	**0.5317**
Q8VI75	Ipo4	Importin-4	19576.21094	21371.86	17812.89	20462.67	**19805.90723**	23774.28	19369.64	23016.45	18637.33	**21199.42188**	**0.9343**	**0.3867**
Q9QYS2	Grm3	Metabotropic glutamate receptor 3	23528.23438	23273.06	25662.73	25165.9	**24407.48047**	29617.94	29207.17	22134.75	23562.68	**26130.6333**	**0.9341**	**0.4238**
Q7TPM1	Prrc2b	Protein PRRC2B	3167.199219	5428.059	4138.476	5601.403	**4583.784302**		4096.605	6392.662	4236.66	**4908.642253**	**0.9338**	**0.7386**
Q8BWY3	Etf1	Eukaryotic peptide chain release factor subunit 1	19063.91602	18648.88	19431.03	19264.19	**19102.00439**	20782.69	22462.14	20696.92	17884.78	**20456.63135**	**0.9338**	**0.2094**
Q91ZA3	Pcca	Propionyl-CoA carboxylase alpha chain, mitochondrial	82843.86719	82771.7	80147.26	80095.6	**81464.60742**	90022.68	88200.27	91681.64	79132.33	**87259.23047**	**0.9336**	**0.0932**
P59648	Fxyd7	FXYD domain-containing ion transport regulator 7			299873.8	330113.4	**314993.5781**			381201.2	293650.3	**337425.7656**	**0.9335**	**0.6760**
P23198	Cbx3	Chromobox protein homolog 3	27333.66602	26586.7	26486.33	27200.09	**26901.69629**	29663.4	29610.23	30132.26	25865.15	**28817.76025**	**0.9335**	**0.1077**
P20108	Prdx3	Thioredoxin-dependent peroxide reductase, mitochondrial	789391.8125	719667.2	756121.5	790905.9	**764021.6094**	827456.6	780205.9	829869.4	836377.1	**818477.2813**	**0.9335**	**0.0424**
P50516	Atp6v1a	V-type proton ATPase catalytic subunit A	726327	742105.7	712598.1	713523.3	**723638.5**	785958.7	772319.5	746664.4	795936.1	**775219.6875**	**0.9335**	**0.0067**
P99026	Psmb4	Proteasome subunit beta type-4	120377.1094	112935.2	109037.7	107174.8	**112381.209**	120651.4	125441.8	112700.3	122821.9	**120403.8457**	**0.9334**	**0.0925**
Q8VCE6	Nt5m	5'(3')-deoxyribonucleotidase, mitochondrial	14537.70313	12682.27	15434.16	14525.8	**14294.98364**	16054.17	13148.89	16739.52	15320.41	**15315.74658**	**0.9334**	**0.3329**
Q8K0C4	Cyp51a1	Lanosterol 14-alpha demethylase	105683.3125	98101.1	100631.8	110537	**103738.3027**	122766.6	119733.2	116799.7	85477.14	**111194.1523**	**0.9329**	**0.4433**
Q8K3W0	Babam2	BRISC and BRCA1-A complex member 2	14100.06055	12738.9	12888.17	12340.77	**13016.9751**	14393.73	14256.27	13578.39	13589.13	**13954.38013**	**0.9328**	**0.0752**
O08908	Pik3r2	Phosphatidylinositol 3-kinase regulatory subunit beta	8115.580566	5615.125	7571.102	4331.53	**6408.334473**	7446.638	6585.505	7905.678	5541.637	**6869.864624**	**0.9328**	**0.6665**
P97450	Atp5pf	ATP synthase-coupling factor 6, mitochondrial	443684.6563	444216.9	442084	456193.5	**446544.7656**	460572.6	451287.3	485597.5	517408.4	**478716.4297**	**0.9328**	**0.0778**
O09111	Ndufb11	NADH dehydrogenase [ubiquinone] 1 beta subcomplex subunit 11, mitoch	182489.9219	181787.7	210359.7	182669.8	**189326.7734**	202638.2	203904	193510.6	211839.5	**202973.0938**	**0.9328**	**0.1371**
Q3THS6	Mat2a	S-adenosylmethionine synthase isoform type-2	57677.24219	59441.9	57957.07	58200.78	**58319.24902**	60758.54	61117.41	62314.2	65917.88	**62527.00488**	**0.9327**	**0.0147**
Q8R4C2	Rufy2	RUN and FYVE domain-containing protein 2	48646.875	45278.42	43125.62	31948.46	**42249.84277**	43855.77	49635.98	34976.38	52729.18	**45299.32813**	**0.9327**	**0.5873**
P0C7M9	Clec2l	C-type lectin domain family 2 member L	49725.60547	48367.41	43134.85	58857.2	**50021.2666**	53713.63	46995.58	54668.14	59158.48	**53633.95703**	**0.9326**	**0.4146**
Q3TDK6	Rogdi	Protein rogdi homolog	48862.74219	48092.47	43344.36	43830.95	**46032.63086**	49720.44	51374.73	46066.86	50312.1	**49368.53418**	**0.9324**	**0.1184**
Q9QZJ6	Mfap5	Microfibrillar-associated protein 5	6043.789551		20349.02	35699.07	**20697.29248**				22198.17	**22198.16602**	**0.9324**	
Q9CXF4	Tbc1d15	TBC1 domain family member 15	12582.46094	13984.31	11231.73	12661.31	**12614.95068**	13047.55	12418.88	14898.2	13760.54	**13531.29224**	**0.9323**	**0.2811**
P11103	Parp1	Poly [ADP-ribose] polymerase 1	8006.467285	8107.833	9257.558	10940.44	**9078.074219**	10622.27	7829.607	10791.76	9719.944	**9740.895752**	**0.9320**	**0.5169**
Q6IEE6	Tmem132e	Transmembrane protein 132E	11671.41602	13342.06	14764.73	8239.722	**12004.48291**	15584.15	12546.36	13323.07	10071.04	**12881.15601**	**0.9319**	**0.6449**
Q80UM3	Naa15	N-alpha-acetyltransferase 15, NatA auxiliary subunit	24672.41211	25273.67	19256.4	19905.41	**22276.97217**	26643.53	26716.37	19617.82	22641.52	**23904.81006**	**0.9319**	**0.5100**
Q3B7Z2	Osbp	Oxysterol-binding protein 1	38521.07031	40636.27	43863.76	45987.31	**42252.10156**	47639.86	45878.21	41578.77	46285.14	**45345.49414**	**0.9318**	**0.1940**
Q8BRJ3	Rell2	RELT-like protein 2	27763.49414	28268.8	20600.03	24205.71	**25209.50586**	23345.21	29477.07	25557.67	29841.3	**27055.31396**	**0.9318**	**0.4668**
Q9DCC4	Pycr3	Pyrroline-5-carboxylate reductase 3	53411.0625	52463.18	48865.78	42760.67	**49375.17383**	49210.43	53176.03	55918.88	53658.59	**52990.97852**	**0.9318**	**0.2421**
Q923M0	Ppp1r16a	Protein phosphatase 1 regulatory subunit 16A	8331.820313	8396.521	5748.328	8037.062	**7628.432739**	8015.36	10781.93	7518.706	6441.641	**8189.410034**	**0.9315**	**0.6342**
Q9CX86	Hnrnpa0	Heterogeneous nuclear ribonucleoprotein A0	18944.46289	21598.09	18811.27	18283.54	**19409.3418**	21338.68	21997.3	20484.75	19528.9	**20837.40869**	**0.9315**	**0.1699**
Q9Z266	Snapin	SNARE-associated protein Snapin	29513.29492	27317.5	23575.91	28057.44	**27116.03516**	29870.46	28270.1	30077.27	28239.77	**29114.39844**	**0.9314**	**0.1920**
Q6P4S6	Sik3	Serine/threonine-protein kinase SIK3	23307.51172	25718.55	21570.59	19027.18	**22405.95898**	23669.56	24482.2	22626.4	25465.57	**24060.93213**	**0.9312**	**0.3223**
Q91W86	Vps11	Vacuolar protein sorting-associated protein 11 homolog	29324.06445	30240.83	24881.83	23533.8	**26995.13232**	29166.52	28911.27	29065.13	28820.3	**28990.80176**	**0.9312**	**0.2707**
Q3TYX3	Smyd5	Histone-lysine N-trimethyltransferase SMYD5	21780.31641	30642.63	33115.72	22079.29	**26904.48975**	18262.11	22587.51	31632.97	43093.62	**28894.05127**	**0.9311**	**0.7599**
Q9Z2M7	Pmm2	Phosphomannomutase 2	33568.22266	39688.08	46708.22	46248.67	**41553.29785**	40940.23	46113.25	47496.59	43960.17	**44627.55859**	**0.9311**	**0.4033**

E9Q3L2	Pi4ka	Phosphatidylinositol 4-kinase alpha	46862.5	41143.13	43008.2	45908.1	**44230.48242**	45312	46380.81	54229.54	44103.05	**47506.34766**	0.9310	0.2610
Q8C129	Lnpep	Leucyl-cystinyl aminopeptidase	40042.89453	39737.36	44123.46	39585.66	**40872.3418**	49063.11	44642.57	38917.05	43003.54	**43906.56641**	0.9309	0.2466
Q9JMH6	Txnrd1	Thioredoxin reductase 1, cytoplasmic	143622.5781	136367.8	144773.8	132996.4	**139440.1523**	142530.2	166997.2	141026.6	148635.6	**149797.4102**	0.9309	0.1680
Q8VCB3	Gys2	Glycogen [starch] synthase, liver	25927.58008	12654.73	22868.57		**20483.62598**	17115.83	26896.78			**22006.30566**	0.9308	0.8255
O08914	Faah	Fatty-acid amide hydrolase 1	13084.71387	9781.232	12510.98	12759.83	**12034.19043**	13459.16	12715.53	12562.24	12983.1	**12930.00537**	0.9307	0.2974
Q9D7B6	Acad8	Isobutyryl-CoA dehydrogenase, mitochondrial	57117.95703	47721.15	53881.26	48361.04	**51770.35059**	62523.2	50796.82	61739.52	47471.86	**55632.85156**	0.9306	0.4171
O88958	Gnpda1	Glucosamine-6-phosphate isomerase 1	58767.82813	65738.97	53319.25	56175.86	**58500.47852**	71255.48	61636.02	57131.48	61443.23	**62866.55273**	0.9306	0.3164
Q8VHQ9	Acot11	Acyl-coenzyme A thioesterase 11	107070.9766	123974.6	77404.2	93695.48	**100536.3184**	122604.1	123903.9	95787.52	89883.72	**108044.8105**	0.9305	0.5924
P08414	Camk4	Calcium/calmodulin-dependent protein kinase type IV	21146.86914	25139.94	24750.31	24686.1	**23930.8042**	28106.94	24483.99	27585.65	22708.52	**25721.2749**	0.9304	0.3024
P62073	Timm10	Mitochondrial import inner membrane translocase subunit Tim10	32206.54297	36312.17	31736.75	36947.17	**34300.6582**	45397.79	37104.88	32460.3	32509.3	**36868.06787**	0.9304	0.4703
O35435	Dhodh	Dihydroorotate dehydrogenase (quinone), mitochondrial	19886.76172	20424.8	18771.76	22173.84	**20314.29053**	20018.36	19337.4	26570.7	21419.15	**21836.40332**	0.9303	0.4262
P35922	Fmr1	Fragile X messenger ribonucleoprotein 1	7638.26416	7872.857	8133.78	3944.632	**6897.383179**	6197.782	8981.244	7490.52	6987.981	**7414.381836**	0.9303	0.6688
Q4QQM4	Trp53i11	Tumor protein p53-inducible protein 11	13431.24414	21105.8	15422.85	18111.85	**17017.93652**	17382.95	12644.95	23588.84	19557.92	**18293.66626**	0.9303	0.6673
Q61885	Mog	Myelin-oligodendrocyte glycoprotein	2702911.25	2811631	2762964	2819481	**2774246.625**	2942740	2894651	3100009	2991731	**2982282.5**	0.9302	0.0068
P0DN34	Ndufb1	NADH dehydrogenase [ubiquinone] 1 beta subcomplex subunit 1	65094.66016	57180.96	57757.22	54891.93	**58731.19238**	62780.81	62473.21	61178.2	66169.53	**63150.44043**	0.9300	0.1216
Q9WUB3	Pygm	Glycogen phosphorylase, muscle form	702548.0625	773377.1	631185.6	665359.9	**693117.6563**	744856.5	736219.4	732886.5	767249.4	**745302.9531**	0.9300	0.1479
Q69ZS6	Sv2c	Synaptic vesicle glycoprotein 2C	10981.96777	19780.99	18818	15934.74	**16378.92456**	21507.83	19009.46	16682.6	13259.62	**17614.8772**	0.9298	0.6565
Q9JM52	Mink1	Misshapen-like kinase 1	28464.71094	28299.77	25806.67	26188.35	**27189.875**	28960.88	33908.79	27245.09	26861.25	**29244.00293**	0.9298	0.2881
Q68ED7	Crtc1	CREB-regulated transcription coactivator 1	8815.253906	9249.598	8438.388	8880.845	**8846.020996**	8308.1	9160.069	7418.072	13172.25	**9514.621826**	0.9297	0.6204
Q8QZR5	Gpt	Alanine aminotransferase 1	19637.95898	16590.68	24733.62	24620.45	**21395.67822**	21319.63	20518.4	22228.88	27984.91	**23012.95361**	0.9297	0.5592
Q64455	Ptprj	Receptor-type tyrosine-protein phosphatase eta	4866.723633	4375.383	4475.513	4847.638	**4641.314453**	3030.026	6610.385	5414.687	4919.66	**4993.689636**	0.9294	0.6572
Q8VCX5	Micu1	Calcium uptake protein 1, mitochondrial	20765.59961	21931.63	20417.17	17598.52	**20178.22949**	23089.76	24289.44	19718.21	19746.51	**21710.97803**	0.9294	0.3422
Q8C996	Tmem163	Transmembrane protein 163	29999.54492	28563.29	28583.7	31184.45	**29582.74365**	31445.46	31853.94	33275.17	30753.08	**31831.90967**	0.9293	0.0344
Q9D892	Itpa	Inosine triphosphate pyrophosphatase	35605.23828	43538.52	30936.88	38420.23	**37125.21777**	38614.8	36770.75	43883.04	40531.34	**39949.98145**	0.9293	0.3891
Q8BGT5	Gpt2	Alanine aminotransferase 2	21146.55469	16401.8	19941.61	19420.35	**19227.57813**	22437.02	21646.18	19913.06	18778.85	**20693.77783**	0.9291	0.3042
Q9ET54	Palld	Palladin		3107.925			**3107.924805**			3344.986		**3344.985596**	0.9291	
Q3U4G3	Xxylt1	Xyloside xylosyltransferase 1	9305.493164	14915.49	19220.61	12420.9	**13965.62354**	13672.85	19351.09	15573.05	11536.16	**15033.28735**	0.9290	0.7032
Q9JKD3	Scamp5	Secretory carrier-associated membrane protein 5	230067.7188	216575.1	174692.8	185520.9	**201714.1367**	234881.2	231072.5	206024.3	196643.6	**217155.4102**	0.9289	0.3716
Q8C6I2	Sdhaf2	Succinate dehydrogenase assembly factor 2, mitochondrial	7373.375488	5414.27	6850.99	6773.383	**6603.004395**	7530.428	6854.447	6145.393	7904.234	**7108.625488**	0.9289	0.4093
Q6PD10	Ip6k1	Inositol hexakisphosphate kinase 1	10950.83105	9818.084	12962.62	6361.813	**10023.33704**	9142.932	10148.4	10647.76	13229.57	**10792.16675**	0.9288	0.6546
Q6Y685	Tacc1	Transforming acidic coiled-coil-containing protein 1	30842.65625	23797.79	26248.87	23714.21	**26150.88135**	27102.03	28982.16	28503.07	28040.14	**28156.84814**	0.9288	0.2873
P14231	Atp1b2	Sodium/potassium-transporting ATPase subunit beta-2	657060.4375	633922.3	658943.2	620433.4	**642589.8281**	629811.3	656249.6	744331.4	737623.8	**692004.0313**	0.9286	0.1539
Q9DC50	Crot	Peroxisomal carnitine O-octanoyltransferase	17840.08398	17701.34	14973.69	14614.45	**16282.39063**	15778.47	15241.3	20110.95	19020.61	**17537.83105**	0.9284	0.4275
Q3TH73	Ttyh2	Protein tweety homolog 2	23109.41016	22737.84	17372.28	24717.93	**21984.36426**	23495.24	24389.54	28679.55	18169.59	**23683.47998**	0.9283	0.5501
Q4VAE3	Tmem65	Transmembrane protein 65	140122.2031	134946.5	118187.6	158803.1	**138014.8379**	149885.4	138092.7	159776	147077.8	**148707.9531**	0.9281	0.3024
P59108	Cpne2	Copine-2	27323.36523	26083.06	31604.65	25081.99	**27523.2666**	30005.7	27474.49	31548.5	29604.96	**29658.41064**	0.9280	0.2466
P10639	Txn	Thioredoxin	140269.9219	151274.5	137708.1	117269.7	**136630.5625**	136687	118906.9	166504.8	166841.8	**147235.1152**	0.9280	0.4703
P48024	Eif1	Eukaryotic translation initiation factor 1	5855.621094	5252.38	3657.594	6144.193	**5227.447266**	6508.003	5170.096	5676.528	5179.624	**5633.562866**	0.9279	0.5480
Q8BH86	Dglucy	D-glutamate cyclase, mitochondrial	26833.60156	26927.43	24966.75	29233.46	**26990.31201**	28509.26	27690.88	30191.07	29963.29	**29088.62695**	0.9279	0.0945
Q9Z2I2	Fkbp1b	Peptidyl-prolyl cis-trans isomerase FKBP1B	33487.63281	28649.18	44194.75	31744.88	**34519.10889**	30779.85	31819.76	44136.17	42079.81	**37203.89746**	0.9278	0.5978
O88291	Znf326	DBIRD complex subunit ZNF326	9658.921875	10010.99	5658.476	5449.68	**7694.517334**	10706.6	10323.83	5865.854	6283.643	**8294.98291**	0.9276	0.7482
Q8BIG7	Comtd1	Catechol O-methyltransferase domain-containing protein 1	31695.73047	35348.26	28999.95	31618.06	**31915.49951**	41028.64	39676.25	32155.52	24764.36	**34406.19287**	0.9276	0.5545
Q9Z1B3	Plcb1	1-phosphatidylinositol 4,5-bisphosphate phosphodiesterase beta-1	49248.58203	45645.05	50144.99	49093.53	**48533.03906**	52515.25	56176.29	51792.3	48800.76	**52321.14941**	0.9276	0.0813
P56564	Slc1a3	Excitatory amino acid transporter 1	321923.6563	345122.4	311773.9	309864.4	**322171.1016**	389868.8	352016.4	344587.3	302845.9	**347329.6016**	0.9276	0.2464
Q8R0A7	Kiaa0513	Uncharacterized protein KIAA0513	46882.28125	46096.59	39309.97	40701.12	**43247.48926**	47299.27	45766.36	44539.59	48917.17	**46630.59668**	0.9274	0.1622
Q8C6E0	Cfap36	Cilia- and flagella-associated protein 36	28556.90234	24964.1	24366.37	23110.72	**25249.52295**	27561.62	29506.45	23745.25	28088.6	**27225.48145**	0.9274	0.2884
Q9CXI0	Coq5	2-methoxy-6-polyprenyl-1,4-benzoquinol methylase, mitochondrial	11135.24121	8509.925	11034.94	10348.5	**10257.15137**	10051.73	9877.171	13400.31	10910.55	**11059.94067**	0.9274	0.4589
Q9DA03	Lyrm7	Complex III assembly factor LYRM7	11759.83301	13423.06	10225	16937.56	**13086.36426**	14513.79	14197.74	18667.43	9066.379	**14111.33643**	0.9274	0.6887
P49446	Ptpre	Receptor-type tyrosine-protein phosphatase epsilon	18639.39648	15207.14	18428.21	16663.59	**17234.58276**	19650.49	19262.82	16605.5	18819.47	**18584.5708**	0.9274	0.2487
Q9DBG5	Plin3	Perilipin-3	108602.7422	112814.3	102616	104251.8	**107071.2246**	110868.5	113030.7	119656.8	118402.7	**115489.6895**	0.9271	0.0354
Q07797	Lgals3bp	Galectin-3-binding protein				2234.086	**2234.085938**			2409.888		**2409.887939**	0.9270	
Q9JJU8	Sh3bgrl	Adapter Sh3bgrl	21128.28906	31008.09	21506.19	19697.21	**23334.94482**	26962.83	23424.23	26714.96	23592.84	**25173.71338**	0.9270	0.5301
Q9D0K0	Tbc1d7	TBC1 domain family member 7	9754.586914	7383.896	7955.881	6332.983	**7856.836792**	8907.231	8587.671	7832.245	8577.363	**8476.127686**	0.9269	0.4415
Q80UW8	Polr2e	DNA-directed RNA polymerases I, II, and III subunit RPABC1	11396.98438		12767.87	12449.33	**12204.72754**	12917.66	13839.59	12744.34		**13167.19727**	0.9269	0.1469
P42232	Stat5b	Signal transducer and activator of transcription 5B	8817.726563	10105.33	6754.37	9301.388	**8744.703613**	10353.85	9991.083	8967.685	8430.759	**9435.844482**	0.9268	0.4431
Q3TAS6	Emc10	ER membrane protein complex subunit 10	26047.3418	25612.18	24693.66	16042.02	**23098.79907**	24714.5	28034.96	19850.07	27100.25	**24924.94531**	0.9267	0.5643
Q9JLB2	Pals1	Protein PALS1	16119.95703	12608.09	13347.53	13859.36	**13983.73242**	13102.94	15907.81	16943.21	14403.25	**15089.30176**	0.9267	0.3668
Q9R0A0	Pex14	Peroxisomal membrane protein PEX14	33295.47656	29569.93	29320.62	34339.77	**31631.44922**	34989.72	31249.19	34201.5	36096.13	**34134.13623**	0.9267	0.1797
P97797	Sirpa	Tyrosine-protein phosphatase non-receptor type substrate 1	107634.4141	100285.9	88360.46	95942.38	**98055.77539**	97075.65	102333.5	114921.5	108925.9	**105814.1348**	0.9267	0.2152
Q9R1T2	Sae1	SUMO-activating enzyme subunit 1	31230.37695	34711.98	31161.27	28257.23	**31340.21484**	36291.19	36411.17	31221.67	31365.59	**33822.4043**	0.9266	0.2542
Q80SY6	Adal	Adenosine deaminase-like protein	6847.710938	6737.24	5154.198	4483.113	**5805.56543**	6515.711	7166.505	4596.95	6785.508	**6266.168701**	0.9265	0.5944
Q8CJF9	Ago3	Protein argonaute-3	17976.20703	18684.23	17623.99	17900.1	**18046.13184**	19189.57	21061.43	19893.27	17777.21	**19480.36963**	0.9264	0.0944
Q78IK4	Apool	MICOS complex subunit Mic27	16248.45605	17871.01	20028.21	17174.83	**17830.62573**	19916.27	20954.21	18897.7	17231.23	**19249.85352**	0.9263	0.2557
P31001	Des	Desmin			11602.48	17004.86	**14303.66748**	8611.507		1468.297	36260.19	**15446.66361**	0.9260	0.9394
Q80U95	Ube3c	Ubiquitin-protein ligase E3C	25215.58203	22373.65	19467.54	24747.67	**22951.10986**	23091.94	25136.64	25504.44	25419.54	**24788.14063**	0.9259	0.2480

Q9JHW4	Eefsec	Selenocysteine-specific elongation factor			7392.511	2855.914	**5124.212769**		7799.829	3269.098		**5534.463257**	**0.9259**	**0.9099**
Q9DBP5	Cmpk1	UMP-CMP kinase	230713.1563	216536.8	196376.1	203072.7	**211674.7031**	233980	225785.5	219575.9	235157.3	**228624.668**	**0.9259**	**0.0915**
D3Z4S3	Ptrhd1	Putative peptidyl-tRNA hydrolase PTRHD1	8044.0625	10978.12	5113.881	6819.669	**7738.933228**	7725.139	8264.526	5205.979	12241.58	**8359.305542**	**0.9258**	**0.7563**
Q9Z1W8	Atp12a	Potassium-transporting ATPase alpha chain 2	582786.9375	449865	526125.1	579109.7	**534471.6953**	595872.9	605886.6	553751.8	553826.5	**577334.4375**	**0.9258**	**0.2535**
Q7TQK5	Ccdc93	Coiled-coil domain-containing protein 93	13998.17578	13668.68	12146.89	12367.56	**13045.32495**	14325	14117.92	14052.87	13871.57	**14091.84082**	**0.9257**	**0.0683**
Q9Z218	Dpp6	Dipeptidyl aminopeptidase-like protein 6	113952.3203	108723.6	100703.7	118023.5	**110350.7949**	124946.2	114968	115985.1	120923.1	**119205.582**	**0.9257**	**0.0906**
A2AHC3	Camsap1	Calmodulin-regulated spectrin-associated protein 1	6977.597656	8372	6453.921	10706.36	**8127.470459**	9618.128	9504.105	6951.904	9048.585	**8780.680664**	**0.9256**	**0.5860**
Q9R0Y5	Ak1	Adenylate kinase isoenzyme 1	577530	666812.7	599874.3	547670.9	**597971.9688**	660274.3	630902	638038.4	655056.8	**646067.8594**	**0.9256**	**0.1166**
Q8BUR4	Dock1	Dedicator of cytokinesis protein 1	8972.825195	6963.191	7531.016	8362.282	**7957.328735**	9422.133	7667.536	9580.565	7727.932	**8599.541504**	**0.9253**	**0.3847**
Q80U87	Usp8	Ubiquitin carboxyl-terminal hydrolase 8	30816.72656	29483.92	27740.97	32071.5	**30028.2793**	32334.35	31775.41	30831.98	34873.39	**32453.78467**	**0.9253**	**0.1042**
Q9QXX4	Slc25a13	Electrogenic aspartate/glutamate antiporter SLC25A13, mitochondrial	22429.33984	23406.04	20660.77	21052.47	**21887.15576**	22640.69	22354.48	25955.35	23672.81	**23655.83252**	**0.9252**	**0.1378**
Q3TYD6	Lmtk2	Serine/threonine-protein kinase LMTK2	5745.208984	2950.98	3042.585	3008.666	**3686.859924**	3971.842		4215.408	3768.801	**3985.350016**	**0.9251**	**0.7305**
O54781	Srpk2	SRSF protein kinase 2	5713.818848	17137.92	16263.62	10140.51	**12313.96814**	14809.34	15938.85	9407.936	13100.52	**13314.16064**	**0.9249**	**0.7541**
Q3UMB5	Smcr8	Guanine nucleotide exchange protein SMCR8	10033.01172	8178.128	8253.25	8496.58	**8740.242432**	8820.106	9676.008	10417.17	8889.801	**9450.771484**	**0.9248**	**0.2635**
Q8BGE6	Atg4b	Cysteine protease ATG4B	24679.07031	20446.94	14936.08	13690.02	**18438.02856**	19280.61	21969.09	20162.82	18336.54	**19937.26758**	**0.9248**	**0.5936**
Q8CC86	Naprt	Nicotinate phosphoribosyltransferase	8969.673828	10352.74	10394	9911.022	**9906.859375**	9546.135	10656.64	11293.6	11354.75	**10712.78296**	**0.9248**	**0.1823**
Q9CXY9	Pigk	GPI-anchor transamidase	30893.57227	24615.19	24435.39	16504.78	**24112.23193**	28592.92	24972.76	23429.76	27318.12	**26078.38867**	**0.9246**	**0.5575**
Q9JM63	Kcnj10	ATP-sensitive inward rectifier potassium channel 10	277817.4375	282613.3	240574.3	246412	**261854.2656**	290415.3	300677.1	257644.8	284327.3	**283266.1328**	**0.9244**	**0.1799**
Q5XG69	Fam169a	Soluble lamin-associated protein of 75 kDa	16493.01563	19695.02	13340.51	20548.74	**17519.32227**	14746.74	21808.78	19728.36	19526.58	**18952.6145**	**0.9244**	**0.5426**
Q6PEV3	Wipf2	WAS/WASL-interacting protein family member 2	30834.02344	27646.87	31950.7	31440.41	**30468**	31233.76	33227.59	36675.27	30709.1	**32961.42871**	**0.9244**	**0.1843**
Q924D0	Rtn4ip1	Reticulon-4-interacting protein 1, mitochondrial	32139.64063	34230.81	33578.51	27782.69	**31932.9126**	32779.31	33598.52	34913	36896.09	**34546.73145**	**0.9243**	**0.1764**
Q9D6K5	Synj2bp	Synaptojanin-2-binding protein	28890.01953	30609.47	26934	30022.34	**29113.95654**	32559.96	33870.29	28299.88	31273.03	**31500.78857**	**0.9242**	**0.1486**
Q9DCA2	Mrps11	Small ribosomal subunit protein uS11m	8624.513672	10159.84	9335.097	9875.93	**9498.845459**	11572.93	9978.005	7688.143	11871.96	**10277.75818**	**0.9242**	**0.4723**
P42567	Eps15	Epidermal growth factor receptor substrate 15	47373.00781	46775.63	39453.41	36372.09	**42493.5332**	46809.06	48301.35	45974.96	42853.17	**45984.63281**	**0.9241**	**0.2820**
Q8BKC8	Pi4kb	Phosphatidylinositol 4-kinase beta	22127.91602	43903.04	27132.04		**31054.32878**	42662.92	18299.84	48932.8	24528.17	**33605.93311**	**0.9241**	**0.8126**
P68037	Ube2l3	Ubiquitin-conjugating enzyme E2 L3	62253.52344	68232.85	52462.06	49630.44	**58144.71777**	73601.71	67671.85	52597.77	57821.44	**62923.19434**	**0.9241**	**0.4840**
Q7TQF7	Amph	Amphiphysin	429873.3438	416814.3	418500.2	406274.1	**417865.4688**	491626.3	452746.5	424737.4	439784.3	**452223.625**	**0.9240**	**0.0635**
Q61990	Pcbp2	Poly(rC)-binding protein 2	92526.82813	98499.73	92974.38	100291.7	**96073.15234**	108866.8	112601.3	102079.9	92384.91	**103983.2383**	**0.9239**	**0.1539**
Q9CRD2	Emc2	ER membrane protein complex subunit 2	17028.01953	14933.77	16576.84	13184.68	**15430.82837**	17365.37	17467.76	16706.66	15269.88	**16702.41626**	**0.9239**	**0.2547**
P52194	Clgn	Calmegin	12362.87598	10826	8570.655	6916.679	**9669.052612**	14086.86	12375.38	9155.449	6247.469	**10466.29114**	**0.9238**	**0.7191**
Q9D6R2	Idh3a	Isocitrate dehydrogenase [NAD] subunit alpha, mitochondrial	936332	979849.8	953479.8	981475.4	**962784.25**	1036194	1011642	1085398	1036052	**1042321.328**	**0.9237**	**0.0057**
Q8R1I1	Uqcr10	Cytochrome b-c1 complex subunit 9	941901.3125	902739.5	858848.5	848608.1	**888024.3594**	945551.5	904207.8	1051922	943951.3	**961408.2031**	**0.9237**	**0.1034**
Q9CXK8	Nip7	60S ribosome subunit biogenesis protein NIP7 homolog	6496.137695	7557.193		3895.29	**5982.873779**		5413.963	6469.658	7548.389	**6477.336426**	**0.9237**	**0.7127**
Q3UHK1	Slc2a13	Proton myo-inositol cotransporter	10899.18066	10318.08	9545.11	10978.33	**10435.17505**	9860.981	11831.28	11812.71	11687.66	**11298.15771**	**0.9236**	**0.1895**
P56546	Ctbp2	C-terminal-binding protein 2	64113.79297	71993.05	39853.35	75342.36	**62825.63867**	62150.34	76130.76	65850.28	68031.95	**68040.83398**	**0.9234**	**0.5638**
Q9DCM2	Gstk1	Glutathione S-transferase kappa 1	33424.98828	34465.86	29685.11	29827.84	**31850.94971**	38283.84	35076.15	33266.32	31355.78	**34495.51953**	**0.9233**	**0.2172**
Q9QXZ0	Macf1	Microtubule-actin cross-linking factor 1, isoforms 1/2/3/4	137424.6563	137861	143514	139304.6	**139526.0508**	154365.5	155229.8	153256.7	141629	**151120.2695**	**0.9233**	**0.0157**
Q9D3D9	Atp5f1d	ATP synthase subunit delta, mitochondrial	455680.0938	618827.7	289569.7	107402.7	**367870.0488**	411518.9	516419.2	327150.3	338919	**398501.875**	**0.9231**	**0.8040**
O54754	Aox1	Aldehyde oxidase 1		8307.021		3131.803	**5719.412354**	8091.734			4301.368	**6196.550781**	**0.9230**	**0.8954**
Q9Z2C5	Mtm1	Myotubularin	18166.1582	15634.97	14000.49	16297.74	**16024.84155**	16608.32	16159.92	18680.99	18001.35	**17362.64624**	**0.9229**	**0.2472**
O35668	Hap1	Huntingtin-associated protein 1	26470.20313	30278.99	18663.93	24155.16	**24892.07227**	29035.74	30385.11	24994.34	23494.46	**26977.4126**	**0.9227**	**0.5028**
Q8BK64	Ahsa1	Activator of 90 kDa heat shock protein ATPase homolog 1	253170.4844	232854.6	243600.1	247902.9	**244382.0234**	273792.8	260875	247129.1	277736.3	**264883.293**	**0.9226**	**0.0457**
O88533	Ddc	Aromatic-L-amino-acid decarboxylase	20212.53125	16343.52	16883.28	23120.95	**19140.07129**	16431.2	22256.99	21807.35	22495.24	**20747.69385**	**0.9225**	**0.4811**
P60840	Ensa	Alpha-endosulfine	24743.78711	28682.93	20382.62	22638.87	**24112.05029**	29924.63	26164.55	26440.04	22022.92	**26138.03467**	**0.9225**	**0.4297**
D3Z7P3	Gls	Glutaminase kidney isoform, mitochondrial	166285.2969	160112.8	158616.8	173566.3	**164645.3008**	163840.3	170204.5	193921.9	185982.6	**178487.3125**	**0.9224**	**0.1235**
Q4KUS2	Unc13a	Protein unc-13 homolog A	21879.76758	18082.34	19936.77	16487.87	**19096.68701**	20625	18478.79	22569.37	21156.72	**20707.46826**	**0.9222**	**0.3065**
Q9CWM4	Pfdn1	Prefoldin subunit 1	55415.55859	42645.62	55623.71	55692.83	**52344.42969**	57577.92	57823.79	53411.18	58232.73	**56761.4043**	**0.9222**	**0.2445**
P10649	Gstm1	Glutathione S-transferase Mu 1	794421.6875	816852	803774.3	854708.2	**817439.0313**	850407.9	834937.6	897653.4	963432.3	**886607.8281**	**0.9220**	**0.0723**
Q8R4N0	Clybl	Citramalyl-CoA lyase, mitochondrial	67828.34375	62258.93	68070.73	71710.74	**67467.18652**	74842.66	69007.41	78367.16	70511.13	**73182.0918**	**0.9219**	**0.0948**
Q61033	Tmpo	Lamina-associated polypeptide 2, isoforms alpha/zeta				15999.37	**15999.37109**	17355.43				**17355.43164**	**0.9219**	
Q8BK72	Mrps27	Small ribosomal subunit protein mS27	18545.92773	18588.07	17606.82	15915.72	**17664.13354**	19179.09	19007.07	17447.96	21013.98	**19162.02637**	**0.9218**	**0.1701**
Q8C5H8	Nadk2	NAD kinase 2, mitochondrial	43892.61328	38716	40284.9	38373.76	**40316.81934**	43081.89	42793.55	43254.12	45904.15	**43758.42871**	**0.9213**	**0.0558**
P54818	Galc	Galactocerebrosidase	17648.47656	19102.52	11672.24	15055.56	**15869.69922**	17989.78	16463.61	16517.34	17927.9	**17224.65918**	**0.9213**	**0.4519**
O88704	Hcn1	Potassium/sodium hyperpolarization-activated cyclic nucleotide-gated cha	15474.99316	14694.68	8145.427	7154.853	**11367.48926**	13922.17	13257.63	9620.872	12559.42	**12340.02051**	**0.9212**	**0.6946**
P63166	Sumo1	Small ubiquitin-related modifier 1	19854.92969	28482.61		21174.38	**23170.63802**	20593.07	24545.91	34569.87	20911.35	**25155.0498**	**0.9211**	**0.6754**
P70460	Vasp	Vasodilator-stimulated phosphoprotein	8009.85498	13345.7	11469.09	13045.12	**11467.43835**	13789.31	12743.48	10275.47	12990.22	**12449.62109**	**0.9211**	**0.5206**
B1AR13	Cisd3	CDGSH iron-sulfur domain-containing protein 3, mitochondrial	10854.6875	10991.64	6389.416	13456.45	**10423.0498**	10659.85	8807.25	12168.31	13632.66	**11317.01831**	**0.9210**	**0.6368**
P26638	Sars1	Serine--tRNA ligase, cytoplasmic	237749.2813	237341.5	232952.2	239364.5	**236851.8789**	260817.8	269451.9	248233.4	250220	**257180.8047**	**0.9210**	**0.0074**
Q8CJH3	Plxnb1	Plexin-B1	32410.3125	30532.58	28021.13	28987.29	**29987.82764**	35463.45	32101.27	31609.37	31079.77	**32563.46484**	**0.9209**	**0.1107**
Q8R138	Tmem119	Transmembrane protein 119	29527.21094	30743.47			**30135.33887**		30380.38		35076.12	**32728.24805**	**0.9208**	**0.3970**
P31650	Slc6a11	Sodium- and chloride-dependent GABA transporter 3	409924.9688	397217.6	327385	384041.8	**379642.3359**	447138.8	471183.4	361001	370053	**412344.0625**	**0.9207**	**0.3601**
P62075	Timm13	Mitochondrial import inner membrane translocase subunit Tim13	231876.2188	239521	205526.7	236497.6	**228355.3594**	252811.9	233947.4	258448.2	246988.9	**248049.1016**	**0.9206**	**0.0805**
O88653	Lamtor3	Ragulator complex protein LAMTOR3	13285.73047	16234.18	17166.44	14831.56	**15379.47778**	16243.85	16405.82	17870.93	16307.09	**16706.92041**	**0.9205**	**0.2043**
P61750	Arf4	ADP-ribosylation factor 4	45139.96484	61122.54	52588.29	50253.13	**52275.97949**	58808.72	58793.34	54283.25	55273.08	**56789.59766**	**0.9205**	**0.2490**
Q91ZR2	Snx18	Sorting nexin-18	7296.956055	5398.803	9361.135	5810.367	**6966.815186**	9625.775	7068.819	6827.199	6764.132	**7571.481323**	**0.9201**	**0.6117**

O70481	Ubr1	E3 ubiquitin-protein ligase UBR1	13500.34277	12750.61	13009.64	13942.36	**13300.73633**	14438.58	16851.9	13495.22	13036.68	**14455.59473**	0.9201	0.2423
Q8BH55	Thnsl1	Threonine synthase-like 1	21511.75195	24420.18	18544.75	21111.81	**21397.12354**	24514.32	23494.51	22766.04	22248.87	**23255.93506**	0.9201	0.2025
Q7TMC8	Fcsk	L-fucose kinase	6470.083496	6326.059	9884.575	6393.328	**7268.511353**	7425.999	7513.863	6464.042	10196.46	**7900.089844**	0.9201	0.6131
O70305	Atxn2	Ataxin-2	14424.13574	15791.87	11379.54	14673.13	**14067.16943**	15051.68	15211.36	14118.17	16780.61	**15290.45313**	0.9200	0.3061
A6H630	Armt1	Damage-control phosphatase ARMT1	16529.11133	19224.76	15698.45	14346.74	**16449.76489**	21448.87	19363.67	18768.13	11958.57	**17884.80884**	0.9198	0.5556
Q91Z67	Srgap2	SLIT-ROBO Rho GTPase-activating protein 2	9834.701172	7042.348	8768.146	10458.89	**9026.02124**	9506.578	9009.479	9963.522	10774.46	**9813.51001**	0.9198	0.3828
Q91WD5	Ndufs2	NADH dehydrogenase [ubiquinone] iron-sulfur protein 2, mitochondrial	293463.5625	297854.8	286998.8	277446.8	**288941.0234**	317194.7	316866.8	329701.4	293182	**314236.2422**	0.9195	0.0285
P35438	Grin1	Glutamate receptor ionotropic, NMDA 1	30019.73047	26966.62	28687.98	36308.14	**30495.61719**	40953.83	33718.43	29246.31	28746.63	**33166.30029**	0.9195	0.4723
P61205;P84078	Arf3;Arf1	ADP-ribosylation factor 3;ADP-ribosylation factor 1	640568.4375	704124.3	597298.1	636774.9	**644691.4219**	680285.6	691615.2	761216.6	672088.5	**701301.4844**	0.9193	0.1086
E9Q735	Ube4a	Ubiquitin conjugation factor E4 A	31169.00586	32701.75	35103.97	26874.41	**31462.2832**	31876.3	32993.18	37762.06	34270.37	**34225.47705**	0.9193	0.2461
P70303	Ctps2	CTP synthase 2	16300.57324	15117.35	16268.15	16097.04	**15945.77832**	16382.16	15551.94	19627.86	17842.37	**17351.08032**	0.9190	0.1844
Q91YN0	D6Wsu163e	Protein C12orf4 homolog	11946.39941	11622.32	10700.74	11636.57	**11476.50732**	11608.2	11913.55	13200.57	13230.31	**12488.15698**	0.9190	0.0908
Q91X72	Hpx	Hemopexin	13190.60742	10437.4	13626.5	14568.7	**12955.80151**	10926.35	12183.08	18276.05	15018.77	**14101.0647**	0.9188	0.5605
Q5U4C1	Gprasp1	G-protein coupled receptor-associated sorting protein 1		9838.353			**9838.352539**	10708.6				**10708.59863**	0.9187	
Q8VEH3	Arl8a	ADP-ribosylation factor-like protein 8A	51161.80859	52588.97	47719.37	33807.8	**46319.4873**	46597.22	45300.41	57978.52	51863.29	**50434.86035**	0.9184	0.4567
P09925	Surf1	Surfeit locus protein 1	35331.5	35513.63	35073.14	37136.49	**35763.68848**	35899.16	35075.65	45517.25	39297.18	**38947.30859**	0.9183	0.2361
Q9Z280	Pld1	Phospholipase D1	10825.79688	8385.008	8532.685	5212.914	**8239.10083**	9597.304	9118.14	7435.902	9739.667	**8972.753174**	0.9182	0.5841
Q6PE01	Snrnp40	U5 small nuclear ribonucleoprotein 40 kDa protein	10310.70801	11470.02	5175.983	4468.888	**7856.39856**	7659.339	9703.796	8699.566	8162.465	**8556.291748**	0.9182	0.7148
Q5F285	Tmem256	Transmembrane protein 256	49516.54688	46568.92	33507.74	47863.19	**44364.09863**	52511.07	51825.78	45309.36	43629.72	**48318.98242**	0.9182	0.3937
O88456	Capns1	Calpain small subunit 1	178497.4844	211280.5	179156.3	166292.9	**183806.7813**	213824.3	197454.2	199308.3	190209	**200198.9414**	0.9181	0.1806
O89086	Rbm3	RNA-binding protein 3	31966.38477	27702.49	60316.25	38955.6	**39735.18164**	12669.62	51744.98	56268.43	52466.55	**43287.39307**	0.9179	0.7867
Q99MS8	Tpgs1	Tubulin polyglutamylase complex subunit 1	9837.81543	10536.6	9567.523	11266.72	**10302.16602**	10318.7	11008.69	12644.55	10924.07	**11224.00195**	0.9179	0.1918
Q6I6G8	Hecw2	E3 ubiquitin-protein ligase HECW2	16048.89453	15709.28	17110.77	32335.02	**20300.99194**	29763.59	24719.58	16481.52	17509.05	**22118.43652**	0.9178	0.7339
Q8C854	Myef2	Myelin expression factor 2	105177.2969	130762.5	134850.7	133049.3	**125959.9375**	147349	141972.3	135309.4	124375	**137251.4199**	0.9177	0.2350
P36993	Ppm1b	Protein phosphatase 1B	11904.41113	13460.36	13827.37	9066.548	**12064.67139**	13528.12	13856.37	11270.01	13931.49	**13146.49951**	0.9177	0.4213
Q80XE1	Ric8b	Synembryn-B	14039.16699	15843.23	16794.97	14991.82	**15417.29663**	17291.01	17431.94	15902.12	16581.97	**16801.76099**	0.9176	0.0903
Q99L04	Dhrs1	Dehydrogenase/reductase SDR family member 1	170796.7969	182429	141147.8	140666.7	**158760.0781**	183366.5	189700.3	165885.5	153263.6	**173053.9805**	0.9174	0.3286
O35864	Cops5	COP9 signalosome complex subunit 5	106454.7188	105377.4	102919.7	94997.16	**102437.2441**	112270	107803.3	102773.4	123798.6	**111661.3281**	0.9174	0.1252
Q9CXY6	Ilf2	Interleukin enhancer-binding factor 2	70422.44531	54122.17	50555.2	56038.49	**57784.57715**	60747.61	72096.26	56340.07	62780.41	**62991.08789**	0.9173	0.3789
P54071	Idh2	Isocitrate dehydrogenase [NADP], mitochondrial	187505.5	196413.4	197990.6	189518.9	**192857.0938**	212781.5	210926	207663.8	209710.3	**210270.3867**	0.9172	0.0008
Q9EPL8	Ipo7	Importin-7	25935.98633	28714.99	22289.93	23160.13	**25025.25928**	28479.7	29371.96	26417.28	24888.78	**27289.4292**	0.9170	0.2486
Q80VD1	Fam98b	Protein FAM98B	17913.52539	18525.53	18127.25	19511.43	**18519.43262**	19471.32	19897.44	17948.96	23481.67	**20199.84912**	0.9168	0.2187
P32848	Pvalb	Parvalbumin alpha	123264.7422	136594.3	145980.4	144257.9	**137524.3184**	114966.6	129235.6	194333.3	161519.7	**150013.7988**	0.9167	0.5233
Q3UQ84	Tars2	Threonine--tRNA ligase, mitochondrial	6605.862305	13908.41	5588.335		**8700.869792**	12176.22	9167.117		7136.548	**9493.295736**	0.9165	0.8048
Q8R3F5	Mcat	Malonyl-CoA-acyl carrier protein transacylase, mitochondrial	8913.068359	8816.94	9545.886	9274.272	**9137.541748**	10328.79	8706.002	9768.778	11075.75	**9969.830078**	0.9165	0.1651
P15532	Nme1	Nucleoside diphosphate kinase A	188113.4375	177661.9	169694.8	158564.3	**173508.5781**	202726.6	178354.5	180550.7	195880.5	**189378.0547**	0.9162	0.1147
Q9CQ80	Vps25	Vacuolar protein-sorting-associated protein 25	27200.80859	30555.38	30340.6	27695.98	**28948.19287**	30830.28	32384.49	38066.51	25105.38	**31596.66504**	0.9162	0.3814
Q924K8	Mta3	Metastasis-associated protein MTA3	9650.71582	11320.8	12273.99	13102.49	**11586.99927**	14168.54	14244.87	9454.839	12720.57	**12647.20581**	0.9162	0.4600
P26450	Pik3r1	Phosphatidylinositol 3-kinase regulatory subunit alpha	12704.80957	13642.68	13778.05	7330.105	**11863.91089**	9547.039	18615.47	11257.92	12377.48	**12949.47754**	0.9162	0.6792
Q9CQY6	Uqcc2	Ubiquinol-cytochrome-c reductase complex assembly factor 2	34239.75	30836.57	34208.71	33792.02	**33269.26221**	34126.9	32337.81	37453.13	41340.1	**36314.48486**	0.9161	0.2053
A2APY7	Ndufaf5	Arginine-hydroxylase NDUFAF5, mitochondrial	8190.638184	9538.17	8317.305	9420.778	**8866.722778**	7635.025	8406.603	15117.82	7560.51	**9679.990234**	0.9160	0.6768
Q9CQA1	Trappc5	Trafficking protein particle complex subunit 5	22743.5	27067.95	22146.26	22795.03	**23688.18359**	26832.53	27829.88	26730.78	22078.91	**25868.02393**	0.9157	0.2512
P97855	G3bp1	Ras GTPase-activating protein-binding protein 1	14161.59961	13481.17	12445.99	12631.56	**13180.07764**	14855.89	16731.79	11668.63	14335.76	**14398.01538**	0.9154	0.3179
E9Q401	Ryr2	Ryanodine receptor 2	9138.845703	9730.006	8934.159	10354.23	**9539.309326**	9646.016	8920.89	11514.6	11604.08	**10421.39746**	0.9154	0.2816
Q9R060	Nubp1	Cytosolic Fe-S cluster assembly factor NUBP1	4409.526367	5631.668	3547.39	5255.533	**4711.029541**	10164.89	3947.716	3624.076	2850.071	**5146.688538**	0.9154	0.8118
Q3UGR5	Hdhd2	Haloacid dehalogenase-like hydrolase domain-containing protein 2	94863.08594	111906.9	87984.32	82668.95	**94355.80469**	98090.3	103809.2	104943.2	105491	**103083.418**	0.9153	0.2332
P70392	Rasgrf2	Ras-specific guanine nucleotide-releasing factor 2	12919.98242	10767.22	10817.77	11498.94	**11500.97827**	13934.14	12028.13	12206.06	12091.59	**12564.97827**	0.9153	0.1682
P26369	U2af2	Splicing factor U2AF 65 kDa subunit	40479.70703	38565.18	31478.13	30122.19	**35161.30322**	43735.13	43610.15	31433.54	34895.19	**38418.50439**	0.9152	0.4502
Q6DFW0	C9orf72	Guanine nucleotide exchange factor C9orf72 homolog	17418.26172	14989.96	12388.83	13181.91	**14494.73901**	14293.59	17755.56	15099.88	16206.53	**15838.89111**	0.9151	0.3560
Q9CWD8	Nubpl	Iron-sulfur protein NUBPL	10954.74609	12947.34	10413.75	12079.83	**11598.91699**	14909.7	12124.4	13258.11	10415.21	**12676.85327**	0.9150	0.3664
Q8QZV4	Stk32c	Serine/threonine-protein kinase 32C	8179.567383	6900.083	6383.765	5790.741	**6813.539063**	6444.915	6208.121	9762.842	7371.637	**7446.878662**	0.9150	0.5331
Q3UUF8	Ankrd34b	Ankyrin repeat domain-containing protein 34B	13605.77148	11454.56	12820.52	9536.777	**11854.40576**	10857.57	12995.38	13423.44	14549.89	**12956.56934**	0.9149	0.3862
O55201	Supt5h	Transcription elongation factor SPT5	8106.192871	9625.896	9842.222	7096.349	**8667.665039**	10072.99	10408.59	8159.648	9257.287	**9474.629272**	0.9148	0.3636
Q8R5H6	Wasf1	Actin-binding protein WASF1	33337.875	32518.08	31211.14	25965.43	**30758.13281**	33845.38	35566.82	30517.08	34561.17	**33622.61328**	0.9148	0.1991
Q9D8W7	Ociad2	OCIA domain-containing protein 2	28577.63672	26010.42	37212	35696.1	**31874.0376**	39883.81	26981.38	38765.64	33756.79	**34846.90576**	0.9147	0.4856
Q9DCM0	Ethe1	Persulfide dioxygenase ETHE1, mitochondrial	64091.24609	68351.16	68273.16	68344.34	**67264.97754**	71613.96	64135.78	87481.38	70945.18	**73544.07617**	0.9146	0.2606
Q9D9H8		Mitochondrial protein C2orf69 homolog		2249.279	3717.737	10012.14	**5326.385986**	2357.767	8468.36	6646.566		**5824.23112**	0.9145	0.8759
Q91W97	Hkdc1	Hexokinase HKDC1	114302.8828	124880.4	93741.28	105375.1	**109574.918**	129233.2	125396.7	119900.8	104752.7	**119820.8398**	0.9145	0.2746
P62960	Ybx1	Y-box-binding protein 1	14895.18164	13450.11	18229.54	12553.62	**14782.1123**	15159.4	17151.17	16303.89	16066.98	**16170.35815**	0.9141	0.3307
Q9Z2A9	Ggt5	Glutathione hydrolase 5 proenzyme	4741.019531	4017.898	2291.794		**3683.570557**	4294.075		5005.678	2789.579	**4029.777507**	0.9141	0.7410
Q9DAK9	Phpt1	14 kDa phosphohistidine phosphatase	26980.67578	30797.74	32607	35119.13	**31376.13525**	29349.66	30833.4	35687.39	41440.01	**34327.61475**	0.9140	0.3950
O88307	Sorl1	Sortilin-related receptor	12705.66699	12339.37	11755.53	14371.73	**12793.0752**	15831.43	13051.14	15525.68	11579.8	**13997.01367**	0.9140	0.3404
P70175	Dlg3	Disks large homolog 3	10133.05176	6282.741	9607.483	10191.79	**9053.767334**	9969.731	8190.179	11071.41	10394.26	**9906.396851**	0.9139	0.4745
Q8C4Y3	Nelfb	Negative elongation factor B	10223.67773	10066.74		8420.504	**9570.30599**	10378.66			10566.18	**10472.41846**	0.9139	0.3141
P61166	Tmem258	Transmembrane protein 258	22850.47461	35336.54	32552.46	29209.13	**29987.15039**	44511.5	33312.48	23638.28	29833.2	**32823.86426**	0.9136	0.6009

A2ADY9	Ddi2	Protein DDI1 homolog 2	20564.44336	25674.59	24962.86	29513.34	**25178.80859**	26754.73	24862.04	27845.62	30783.94	**27561.58203**		0.9135	0.3230
Q8VCL2	Sco2	Protein SCO2 homolog, mitochondrial	21791.04297	28579.39	13354.87	15610.24	**19833.88574**	22101.84	16332.14	22833.91	25578.52	**21711.60596**		0.9135	0.6498
Q8R4U7	Luzp1	Leucine zipper protein 1	14031.90137	14103.3	14327.67	12625.06	**13771.9834**	15020.37	17127.09	13211.63	14945.53	**15076.15625**		0.9135	0.1932
Q61923	Kcna6	Potassium voltage-gated channel subfamily A member 6	5551.392578	2352.305	2772.427	1847.858	**3130.995514**	7242.589	1653.547	2146.699	2667.512	**3427.587036**		0.9135	0.8529
P63101	Ywhaz	14-3-3 protein zeta/delta	377929.7813	372811.8	401701.9	431241.3	**395921.2188**	423436.9	330905.3	533064.3	446314.2	**433430.1719**		0.9135	0.4230
Q9WV98	Timm9	Mitochondrial import inner membrane translocase subunit Tim9	76776.0625	80262.15	61777.01	66425.2	**71310.10352**	81358.38	84429.48	73551.43	72926.75	**78066.50781**		0.9135	0.2405
P47199	Cryz	Quinone oxidoreductase	55535.60547	62530.2	60018.63	54217.75	**58075.5459**	64235.04	63042.45	62071.22	65009.65	**63589.58789**		0.9133	0.0355
Q91W39	Ncoa5	Nuclear receptor coactivator 5	11167.21484	5287.961	10014.09	6093.879	**8140.785278**	10614.2	8493.304	7740.211	8807.617	**8913.83374**		0.9133	0.6392
E9Q6B2	Ccdc85c	Coiled-coil domain-containing protein 85C	19047.46289			11789.49	**15418.4751**	16672.85	19113.08	12575.55	19171.04	**16883.13159**		0.9132	0.6724
Q8R404	Micos13	MICOS complex subunit MIC13	128262.1953	117974.9	105106.9	85481.65	**109206.4043**	119247.3	121729.3	110696.6	126675.2	**119587.0957**		0.9132	0.3305
Q9WTU6	Mapk9	Mitogen-activated protein kinase 9	12714.9834	14392.53	12212.74	10743.13	**12515.84546**	14250.08	13953.88	10190	16429.43	**13705.84888**		0.9132	0.4573
Q3TDD9	Ppp1r21	Protein phosphatase 1 regulatory subunit 21	15396.33887	14633.63	12845.76	13237.8	**14028.3833**	16203.31	15938.48	15446.29	13865.9	**15363.49561**		0.9131	0.1433
Q62179	Sema4b	Semaphorin-4B	17032.93945	11783.77		8905.624	**12574.1123**	17720.32	10632.58	15326.42	11406.11	**13771.35742**		0.9131	0.6872
O08664;Q921K9;Q9CXE	Bcl7c;Bcl7b;Bcl7a	B-cell CLL/lymphoma 7 protein family member C;B-cell CLL/lymphoma 7 p	15248.78809	15272.91	16704.19	17408.9	**16158.69775**	14760	19159.01		19192.55	**17703.85352**		0.9127	0.3157
Q91WV0	Dr1	Protein Dr1	27667.23047	26395.39	20284.26	23561.91	**24477.1958**	30606.46	30148.59	22093.72	24437.26	**26821.50488**		0.9126	0.4141
Q9D0B5	Tstd3	Thiosulfate sulfurtransferase/rhodanese-like domain-containing protein 3	17134.02734	18303.13	17824.53	20614.72	**18469.10205**	21789.06	17703.89	21183.56	20308.33	**20246.21094**		0.9122	0.1810
Q61361	Bcan	Brevican core protein	142301.75	144092.8	130434.9	119288.4	**134029.4609**	125267.2	154275.7	151968.2	156201.8	**146928.2246**		0.9122	0.2141
Q8CBC4	Cnst	Consortin	7553.438477	6549.37	8101.925	6764.79	**7242.380737**	9177.555	8448.112	7276.781	6858.769	**7940.304077**		0.9121	0.3186
Q8R3P0	Aspa	Aspartoacylase	144310.5469	158691.3	142530.1	124585.4	**142529.3145**	154901	146713.8	164966.6	158479.9	**156265.3398**		0.9121	0.1351
Q5SVL6	Rap1gap2	Rap1 GTPase-activating protein 2	24871.53711	27292.58	22470.65	23612.32	**24561.77295**	27742.39	25386.33	29418.01	25172.04	**26929.69336**		0.9121	0.1530
Q6P9K8	Caskin1	Caskin-1	101947.7734	86901.38	94413.64	110052.5	**98328.83203**	113355.2	125336.9	94929.27	97622.59	**107810.9883**		0.9120	0.3166
P45878	Fkbp2	Peptidyl-prolyl cis-trans isomerase FKBP2	167214.4375	166004.4	142149.9	156947.3	**158079.0195**	191523.2	189313.9	155627.5	157054.1	**173379.6875**		0.9118	0.2290
Q8C0I1	Agps	Alkyldihydroxyacetonephosphate synthase, peroxisomal	16549.73242	15725.45	19786	9345.759	**15351.73438**	15673.8	17677.81	19747.23	14259.31	**16839.53784**		0.9116	0.5722
Q6NVE9	Pptc7	Protein phosphatase PTC7 homolog	37103.40234	40263.22	37285.29	37108.28	**37940.04883**	41592.01	40286.72	43048.34	41553.07	**41620.03613**		0.9116	0.0086
O70378	Emc8	ER membrane protein complex subunit 8	23747.55273	19211.68	23104.77	24773.53	**22709.3833**	24356.99	27987.64	25179.48	22124.94	**24912.26416**		0.9116	0.2466
P15209	Ntrk2	BDNF/NT-3 growth factors receptor	18961.55078	14793.63	13083.6	15196.23	**15508.75171**	15498.16	16746.13	18169.1	17640.52	**17013.47632**		0.9116	0.3140
P97300	Nptn	Neuroplastin	443976.6563	450267.8	414181.1	409860.3	**429571.4688**	449506.2	461644.5	451104.7	522828.5	**471270.9688**		0.9115	0.0844
Q61301	Ctnna2	Catenin alpha-2	67097.78125	65607.49	49977.82	56838.93	**59880.50781**	63805.07	71307.73	66029.95	61650.68	**65698.35547**		0.9114	0.2443
P83510	Tnik	Traf2 and NCK-interacting protein kinase	8158.97168	11775.02	9505.69	13073.51	**10628.29907**	13686.28	8263.768	14055.28	10639.18	**11661.12549**		0.9114	0.5782
O88951	Lin7b	Protein lin-7 homolog B	44163.10938	41203.17	37462.58	37698.61	**40131.86719**	40978.46	52628.66	42697.32	39835.06	**44034.87402**		0.9114	0.2856
P0C0A3	Chmp6	Charged multivesicular body protein 6	15424.4707	15113.34	15971.93	17881.97	**16097.92896**	17053.54	14870.11	19889.68	18842.9	**17664.05811**		0.9113	0.2613
Q4VAA7	Snx33	Sorting nexin-33	21056.01367	16083.27	21347.02	13251.48	**17934.44629**	21150.27	24214.01	15108.76	18245.48	**19679.6333**		0.9113	0.5526
Q80TY0	Fnbp1	Formin-binding protein 1	53336	47375.57	40526.45	34940.89	**44044.72852**	46322.73	49409.27	51382.5	46215.16	**48332.41602**		0.9113	0.3467
A2BDX3	Mocs3	Adenylyltransferase and sulfurtransferase MOCS3	62168.49609	57067.69	74234.24	65395.8	**64716.55762**	104656.8	61314.89	61421.41	56802.44	**71048.88672**		0.9109	0.6113
Q8R1T1	Chmp7	Charged multivesicular body protein 7	13526.58105	12662.44	5006.184	10024.62	**10304.95569**	5728.228	13530.18	12702.6	13296.34	**11314.33667**		0.9108	0.7192
Q8JZN7	Rhot2	Mitochondrial Rho GTPase 2	25397.35352	24524.41	22601.16	20889.32	**23353.06055**	25458.33	26939.67	24339.95	25825.74	**25640.91992**		0.9108	0.0919
Q8BHE8	Maip1	m-AAA protease-interacting protein 1, mitochondrial	9356.117188	10689.63	6386.293	10511.25	**9235.822388**	9296.677	9984.813	11747.12	9534.765	**10140.84399**		0.9108	0.4570
P47708	Rph3a	Rabphilin-3A	67110.82031	66970.84	65940.44	65936.91	**66489.75**	75812.49	72643.91	74580.4	69015.41	**73013.05078**		0.9107	0.0051
P28352	Apex1	DNA-(apurinic or apyrimidinic site) endonuclease	17137.63867	18845.25	16437.81	18864.17	**17821.21924**	22512.99	20126.21	16766.25	18874.52	**19569.9917**		0.9106	0.2424
Q3V4B5	Commd6	COMM domain-containing protein 6	13377.1875	13101.87	9220.579	6693.814	**10598.36145**	11371.96	10478.31	13462.18	11242.33	**11638.698**		0.9106	0.5702
Q3UHD2	Gfod1	Glucose-fructose oxidoreductase domain-containing protein 1	16431.29688	17954.86	15530.64	19755.15	**17417.98779**	18824.12	18865.43	19201	19622.73	**19128.31885**		0.9106	0.1200
Q8VE47	Uba5	Ubiquitin-like modifier-activating enzyme 5	20718.39258	20103.33	17899.91	19182.96	**19476.14893**	18976.36	20875.56	24209.06	21496.38	**21389.33936**		0.9106	0.1748
Q9CRT8	Xpot	Exportin-T	9613.724609	10196.92	11331.72	11239.48	**10595.45996**	11708.25	12847.51	9678.086	12311.75	**11636.39795**		0.9105	0.2453
Q99LJ1	Fuca1	Tissue alpha-L-fucosidase	16490.56836	18378.75	19303.38	15010.51	**17295.80298**	18805.39	20223.64	20705.63	16259.06	**18998.4314**		0.9104	0.2651
Q8K211	Slc31a1	High affinity copper uptake protein 1	6568.35498	7130.614	3571.338	7428.124	**6174.60791**	8101.656			5464.678	**6783.166748**		0.9103	0.7155
Q8BWM0	Ptges2	Prostaglandin E synthase 2	34580.92188	38083.7	36267.15	41204.32	**37534.02344**	43176.5	43042.55	39782.97	38934.42	**41234.1123**		0.9103	0.0845
Q8BMI3	Gga3	ADP-ribosylation factor-binding protein GGA3	61475.76563	55791.08	48063.57	49517.13	**53711.8877**	55000.15	61853.62	55243.79	63944.2	**59010.44043**		0.9102	0.2167
Q5SX40	Myh1	Myosin-1		5472.725		8051.032	**6761.878174**				7428.938	**7428.9375**		0.9102	
P16125	Ldhb	L-lactate dehydrogenase B chain	2272146.25	2536749	2335897	2388085	**2383219.25**	2530247	2390391	2703391	2849923	**2618487.938**		0.9102	0.0868
Q99LD4	Gps1	COP9 signalosome complex subunit 1	84950.8125	81924.29	78136.27	73815.3	**79706.66602**	90432.5	91248.72	84366.55	84287.93	**87583.92578**		0.9101	0.0421
Q6PHS9	Cacna2d2	Voltage-dependent calcium channel subunit alpha-2/delta-2	20091.32813	18474.65	18532.46	16637.4	**18433.95947**	19980.69	21577.21	20137.47	19350.41	**20261.44482**		0.9098	0.0747
Q8C650	Septin10	Septin-10	16503.01953	18239.91	17084.13	9705.571	**15383.15796**	21246.47	15010.03	14861.24	16523.42	**16910.29175**		0.9097	0.5541
Q9CX34	Sugt1	Protein SGT1 homolog	89152.03906	67517.61	93002.85	87187.82	**84215.08008**	87906.53	84345.66	98034.09	100030.9	**92579.29688**		0.9097	0.2683
Q64444	Ca4	Carbonic anhydrase 4	15860.82617	13415.4	12540.49	16141.71	**14489.60718**	15289.53	15627.93	18000.76	14803.94	**15930.53979**		0.9095	0.2535
E9Q7G0	Numa1	Nuclear mitotic apparatus protein 1	11501.91016	11169.23	7119.911	8821.31	**9653.089478**	12736.47	9640.907	10808.06	9267.719	**10613.28931**		0.9095	0.4864
P97382	Kcnab3	Voltage-gated potassium channel subunit beta-3	13524.83008	14634.51	15707.11	15305.86	**14793.07739**	13860.37	14630.35	18706.2	17866.57	**16265.86963**		0.9095	0.2942
Q4VAA2	Cdv3	Protein CDV3	41842.41016	40662.62	34659.33	43391.29	**40138.91113**	36822.26	35396.54	51482.43	52841.32	**44135.64063**		0.9094	0.4570
Q8CHG3	Gcc2	GRIP and coiled-coil domain-containing protein 2	5174.09668	5238.721	5483.396	2179.676	**4518.972107**	1892.096	5971.167	4252.141	7762.929	**4969.582916**		0.9093	0.7705
Q8VHK9	Dhx36	ATP-dependent DNA/RNA helicase DHX36	8788.904297	9553.979	7465.055	6715.707	**8130.911377**	12038.51	11726.09	7648.687	4368.25	**8945.383179**		0.9090	0.6882
Q9D1L0	Chchd2	Coiled-coil-helix-coiled-coil-helix domain-containing protein 2	50702.54688	52293.13	21170.15	39479.3	**40911.28271**	45070.41	46982.57	50591.25	37465.28	**45027.375**		0.9086	0.6116
P63011	Rab3a	Ras-related protein Rab-3A	1200967.625	1308035	1010998	1109542	**1157385.719**	1313339	1301302	1268695	1213190	**1274131.344**		0.9084	0.1334
Q8BQP9	Rgs7bp	Regulator of G-protein signaling 7-binding protein	15010.01953	19347.8	6927.527	8998.939	**12571.07166**	8766.115	18085.84	12263.4	16246.12	**13840.36914**		0.9083	0.7306
Q8K0X8	Fez1	Fasciculation and elongation protein zeta-1	11991.36816	13056.58	16720.29	9340.293	**12777.13281**	17024.28	11532.99		13648.5	**14068.58529**		0.9082	0.5909
Q91XI1	Dus3l	tRNA-dihydrouridine(47) synthase [NAD(P)(+)]-like	6858.540527	10494.12	10096.3	5933.838	**8345.699341**	10166.87	6789.774	10325.43	9487.667	**9192.435181**		0.9079	0.5697
P21550	Eno3	Beta-enolase	50033.59375	194555.3	32751.17	37735.16	**78768.81348**	57401.96	149238.1	55582.5	84880.01	**86775.64258**		0.9077	0.8632

Q61545	Ewsr1	RNA-binding protein EWS	25370.06836	23127.93	25337.79	19718.87	**23388.66455**	21801.57	21108.8	30699.45	29491.1	**25775.22998**	**0.9074**	**0.4331**
P51163	Uros	Uroporphyrinogen-III synthase	21582.69922	25213.83	22279.22	24668.3	**23436.01172**	28139.78	21193.55	28182.97	25795.05	**25827.83789**	**0.9074**	**0.2474**
Q7TQI3	Otub1	Ubiquitin thioesterase OTUB1	140873.4844	142484.5	138711.5	124872.6	**136735.5293**	135856.4	153151.9	157048.8	156717.4	**150693.6289**	**0.9074**	**0.0733**
Q3TCN2	Plbd2	Putative phospholipase B-like 2	30455.92383	31483.49	20293.24	28636.58	**27717.30811**	32130.69	29039.85	31135.78	29899.82	**30551.53271**	**0.9072**	**0.3231**
Q9WV18	Gabbr1	Gamma-aminobutyric acid type B receptor subunit 1	19206.73828	22747.67	15277.59	24936.56	**20542.13916**	25205.2	21279.18	22297.06	21790.3	**22642.93213**	**0.9072**	**0.3944**
Q9DB26	Phyhd1	Phytanoyl-CoA dioxygenase domain-containing protein 1	5818.752441	8439.854	7479.021	5320.378	**6764.501343**	7575.893	5534.47	6311.727	10403.39	**7456.369141**	**0.9072**	**0.6113**
Q9Z2Y3	Homer1	Homer protein homolog 1	16352.55859	13646.36	12631.63	18479.62	**15277.54126**	16255.15	16290.89	17287.15	17531.35	**16841.13599**	**0.9072**	**0.2960**
P63040	Cplx1	Complexin-1	210208.5938	309599	236886	200308.7	**239250.582**	380457.5	273114.2	207764.6	193801.6	**263784.4648**	**0.9070**	**0.6358**
Q80U78	Pum1	Pumilio homolog 1	9134.849609	8726.074	11549.82	11862.52	**10318.31494**	10708.24	10476.69	11256.96	13070.48	**11378.09497**	**0.9069**	**0.3296**
P54923	Adprh	ADP-ribosylhydrolase ARH1	41765.04297	38414.15	34261.98	34523.45	**37241.15625**	44129.49	43168.56	39235.25	37735.86	**41067.29297**	**0.9068**	**0.1548**
Q6P9R4	Arhgef18	Rho guanine nucleotide exchange factor 18	4991.789063	4555.187	8663.949	5205.807	**5854.182983**	6247.425	6554.527	7030.901	5997.575	**6457.607178**	**0.9066**	**0.5576**
Q99LU0	Chmp1b1	Charged multivesicular body protein 1B1	9662.609375	14016.25		18362.03	**14013.62923**		15335.01	13157.15	17883.65	**15458.60579**	**0.9065**	**0.6398**
Q9D0I6	Wdsub1	WD repeat, SAM and U-box domain-containing protein 1		3983.595			**3983.595459**		4615.195	3627.813	4941.44	**4394.816243**	**0.9064**	
Q9JLI8	Sart3	Squamous cell carcinoma antigen recognized by T-cells 3	19450.89063	19544.39	18984.6	20365.34	**19586.30615**	20005.08	21209.71	22384.12	22841.58	**21610.12207**	**0.9063**	**0.0273**
Q4LDD4	Arap1	Arf-GAP with Rho-GAP domain, ANK repeat and PH domain-containing prot	4525.537598	4647.399		1843.985	**3672.30721**	4293.503		5967.545	1895.015	**4052.020996**	**0.9063**	**0.8120**
Q9CR26	Vta1	Vacuolar protein sorting-associated protein VTA1 homolog	128483.8516	161874.4	151322.5	138476.1	**145039.2012**	167281.6	159083.9	160562.7	153402.9	**160082.7617**	**0.9060**	**0.1035**
Q9CTY5	Micu3	Calcium uptake protein 3, mitochondrial	18802.12109	15335.24	11709.45	14906.13	**15188.23438**	16718.03	15949.5	17561.46	16834.95	**16765.98584**	**0.9059**	**0.3298**
Q60759	Gcdh	Glutaryl-CoA dehydrogenase, mitochondrial	42003.25	42911.34	42495.89	38385.94	**41449.10645**	45182.37	47966.49	44039.5	45839.67	**45757.00879**	**0.9059**	**0.0175**
Q9D855	Uqcrb	Cytochrome b-c1 complex subunit 7	231342.4375	229335.2	221895.3	231707.1	**228570.0039**	249154	244747.2	276605.7	238863.1	**252342.5039**	**0.9058**	**0.0336**
P14094	Atp1b1	Sodium/potassium-transporting ATPase subunit beta-1	6995462.5	6572822	5840763	6383112	**6448039.75**	7029429	7135401	6865319	7445274	**7118855.625**	**0.9058**	**0.0468**
Q5EBJ4	Ermn	Ermin	26708.06641	22718.18	23461.29	25688.18	**24643.9292**	28680.14	25196.25	26767.08	28201.49	**27211.24023**	**0.9057**	**0.0800**
Q8JZX4	Rbm17	Splicing factor 45		3535.689	3675.153	4337.15	**3849.330566**	4808.667	4568.254	1990.574	5634.192	**4250.421783**	**0.9056**	**0.6916**
Q924M7	Mpi	Mannose-6-phosphate isomerase	56242.96875	53814.93	55468.48	43641.74	**52292.02734**	59949.7	59748.98	52512.53	58784.3	**57748.87988**	**0.9055**	**0.1615**
Q60996	Ppp2r5c	Serine/threonine-protein phosphatase 2A 56 kDa regulatory subunit gamma	38688.81641	55381.23	34383.71	40595.25	**42262.25293**	38437.56	51974.81	50689.13	45624.42	**46681.47754**	**0.9053**	**0.4523**
Q9D061	Acbd6	Acyl-CoA-binding domain-containing protein 6	11376.19922	12727.55	8614.426	12000.44	**11179.65552**	12644.06	10633.16	13291.93	12827.85	**12349.2522**	**0.9053**	**0.3179**
Q8CHX7	Rftn2	Raftlin-2	13565.9834	22521.93	20625.36	13885.83	**17649.77563**	20248.79	26635.19	17285.73	13819.51	**19497.30688**	**0.9052**	**0.6224**
Q6ZWR6	Syne1	Nesprin-1	11466.39355	11888.17	12570.93	12128.21	**12013.42554**	13587.3	13165.83	13456.32	12881.24	**13272.67188**	**0.9051**	**0.0041**
Q9DB15	Mrpl12	Large ribosomal subunit protein bL12m	75289.77344	74476.09	58051.59	57159.82	**66244.31836**	80856.9	78640.14	70727.65	62787.54	**73253.05762**	**0.9043**	**0.3201**
B1AVY7	Kif16b	Kinesin-like protein KIF16B	19818.17188	21682.35	15804.76	19514.73	**19205.00269**	23941.98	21841.59	17952.59	21221.67	**21239.45654**	**0.9042**	**0.2885**
P70671	Irf3	Interferon regulatory factor 3		3998.024	4935.254	4835.968	**4589.749023**	4060.615		5729.826	5438.33	**5076.256836**	**0.9042**	**0.4590**
Q5SW19	Cluh	Clustered mitochondria protein homolog	18461.01953	16587.2	16391.07	18431.44	**17467.68213**	19094.39	20212.28	21240.85	16733.16	**19320.17041**	**0.9041**	**0.1495**
Q91VZ6	Smap1	Stromal membrane-associated protein 1	80340.46094	81754.98	75813.35	70507.27	**77104.01563**	81318.89	79674.15	90293.41	89855.45	**85285.47461**	**0.9041**	**0.0730**
Q91WK1	Spryd4	SPRY domain-containing protein 4	24713.41992	21261.56	24201.08	20643.62	**22704.9209**	23902.36	27622.65	27232.9	21702.15	**25115.01367**	**0.9040**	**0.2162**
Q922D4	Ppp6r3	Serine/threonine-protein phosphatase 6 regulatory subunit 3	7064.253418		8445.543	10541.75	**8683.849772**	8921.332	15734.4	4323.596	9451.26	**9607.646118**	**0.9038**	**0.7629**
Q9CXW3	Cacybp	Calcyclin-binding protein	101903.1484	94058.38	82762.82	76737.7	**88865.51172**	94634.76	99898.63	100198	98608.34	**98334.94141**	**0.9037**	**0.1525**
Q8BH95	Echs1	Enoyl-CoA hydratase, mitochondrial	229989.2813	239214.8	238033.7	257620	**241214.4336**	268287.1	274749.1	262490.8	262283.3	**266952.5781**	**0.9036**	**0.0077**
P61087	Ube2k	Ubiquitin-conjugating enzyme E2 K	86227.94531	82136.93	67168.98	77038.79	**78143.16211**	85815.02	84795.87	84084.34	91289.37	**86496.14844**	**0.9034**	**0.1081**
Q9JKW0	Arl6ip1	ADP-ribosylation factor-like protein 6-interacting protein 1	13923.56738	15216.19	9212.739	13821.91	**13043.60278**	15374.72	11255.11	13485.45	17641.68	**14439.23901**	**0.9033**	**0.4886**
Q80UC6	Gpr62	G-protein coupled receptor 62	7933.361328	8920.372	10740.42	7605.583	**8799.934937**	7596.194	9381.877	11894.24	10094.37	**9741.6698**	**0.9033**	**0.4383**
Q6NS82	Retreg2	Reticulophagy regulator 2	35834.74219	39164.62	25036.63	20973.9	**30252.47363**	36072.06	30088.95	30633.81	37208.61	**33500.85596**	**0.9030**	**0.5146**
Q80VL1	Tdrkh	Tudor and KH domain-containing protein	31373.66992	34541.1	31124.72	32374.17	**32353.41602**	38954.52	31906.34	39789.71	32671.53	**35830.52393**	**0.9030**	**0.1650**
P97411	Ica1	Islet cell autoantigen 1	9387.881836	27601.51	27037.42	25375.4	**22350.55151**	12534.58	26454.25	28327.71	31698.86	**24753.84937**	**0.9029**	**0.7052**
F8VPK0	Skic3	Superkiller complex protein 3	9282.317383	9607.973	10188.38	9253.871	**9583.135986**	9765.71	15493.48	8003.23	9200.436	**10615.71472**	**0.9027**	**0.5616**
P18872	Gnao1	Guanine nucleotide-binding protein G(o) subunit alpha	1237886.5	1235897	1121719	1223025	**1204631.594**	1356469	1315436	1440452	1226768	**1334781.219**	**0.9025**	**0.0476**
Q99J36	Thumpd1	THUMP domain-containing protein 1	5078.422852	5812.275	6240.299	4019.782	**5287.694885**	5605.814	6172.187	4457.129	7202.121	**5859.312744**	**0.9024**	**0.4753**
O54865	Gucy1b1	Guanylate cyclase soluble subunit beta-1	31916.59766	35843.76	27745.68	33604.35	**32277.5957**	37920.32	34125.75	38134.11	32893.33	**35768.37891**	**0.9024**	**0.1583**
Q8BRK8	Prkaa2	5'-AMP-activated protein kinase catalytic subunit alpha-2			2994.379		**2994.378906**			3318.392		**3318.392334**	**0.9024**	
P24457	Cyp2d11	Cytochrome P450 2D11	12998.3877	12655.4	15809.31	13577.93	**13760.25562**	12804.39	12274.37	19727.78	16224.24	**15257.69238**	**0.9019**	**0.4533**
O35295	Purb	Transcriptional activator protein Pur-beta	69274.21875	75215.79	63103.19	61080.79	**67168.49609**	77898.8	75500.61	76848.41	67672.46	**74480.06836**	**0.9018**	**0.1138**
Q9CR98	Fam136a	Protein FAM136A	36307.00781	32175.88	22646.3	30456.3	**30396.37354**	36351.39	35201.68	37985.98	25283.62	**33705.6709**	**0.9018**	**0.4449**
P35546	Ret	Proto-oncogene tyrosine-protein kinase receptor Ret				4298.226	**4298.226074**	5699.788		4127.577	4473.305	**4766.889974**	**0.9017**	
Q6PDS3	Sarm1	NAD(+) hydrolase SARM1	19306.88477	17096.31	18337.23	16184.82	**17731.31104**	19327.13	18701.18	19038.52	21592.83	**19664.91553**	**0.9017**	**0.0876**
Q921H9	Coa7	Cytochrome c oxidase assembly factor 7		9609.001	9901.86	11974.9	**10495.25326**	12824.74	13402.52		8698.194	**11641.81673**	**0.9015**	**0.5272**
Q01147	Creb1	Cyclic AMP-responsive element-binding protein 1	9797.503906	8739.755	7722.752	5191.022	**7862.758301**	10411	7292.931	8461.705		**8721.877279**	**0.9015**	**0.5644**
Q62087	Pon3	Serum paraoxonase/lactonase 3	10756.40918	13487.17	19685.09	7463.559	**12848.05823**	15228.81	12041.56	15732.84	14006.33	**14252.3855**	**0.9015**	**0.6238**
Q61285	Abcd2	ATP-binding cassette sub-family D member 2	15195.67969	16974.96	14655.99	11464.6	**14572.80859**	17517.47	15630.61	15595.04	15929.62	**16168.18604**	**0.9013**	**0.2442**
Q9Z108	Stau1	Double-stranded RNA-binding protein Staufen homolog 1	3880.577393	7503.557	819.5087		**4067.8809**	4650.466	6961.97	3294.674	3146.115	**4513.306152**	**0.9013**	**0.8262**
C0HK79;C0HK80	Arxes1;Arxes2	Adipocyte-related X-chromosome expressed sequence 1;Adipocyte-related	18787.21094	15299.96	8237.146	11989.48	**13578.44946**	15861.57	17084	11612.36	15703.56	**15065.37085**	**0.9013**	**0.5814**
P61963	Dcaf7	DDB1- and CUL4-associated factor 7	17822.875	18497.49	13204.61	17572.34	**16774.32837**	18283.9	17354.37	19680.09	19144.96	**18615.82813**	**0.9011**	**0.2091**
Q8BH58	Tiprl	TIP41-like protein	31826.66992	22450.46	21799.74	24096.75	**25043.40723**	25209.34	31231.5	24584.95	30168.98	**27798.69336**	**0.9009**	**0.3735**
Q3UMT1	Ppp1r12c	Protein phosphatase 1 regulatory subunit 12C	31085.28711	15700.98	10338.01	18229.48	**18838.43823**	21750.85	22232.51	20554.44	19108.16	**20911.49023**	**0.9009**	**0.6582**
Q812A2	Srgap3	SLIT-ROBO Rho GTPase-activating protein 3	20765.22266	20856.98	22114.35	19828.67	**20891.30518**	22948.07	21314.96	24300.99	24199.07	**23190.77197**	**0.9008**	**0.0339**
Q7TN99	Cpeb3	Cytoplasmic polyadenylation element-binding protein 3	15608.20117	17505.57	13681.55	12569.83	**14841.28857**	15934	18018.16	15935.81	16013.44	**16475.35352**	**0.9008**	**0.2232**
Q3TFD2	Lpcat1	Lysophosphatidylcholine acyltransferase 1	11415.67871	11089.31	8734.293	9769.333	**10252.1543**	10463.94	17082.87	9738.778	8252.275	**11384.46753**	**0.9005**	**0.6007**
Q9QUH0	Glrx	Glutaredoxin-1	151816.5469	202073.9	125096.9	173398.3	**163096.4258**	170841.3	157183.8	207463.9	189140.7	**181157.4219**	**0.9003**	**0.3935**

B1AVZ0	Uprt	Uracil phosphoribosyltransferase homolog	18468.16406	13163.85	14240.67	12006.82	**14469.87378**	19047.1	15492.7	16120.55	13642.62	**16075.74365**	**0.9001**	**0.4068**
Q3TC93	Hs1bp3	HCLS1-binding protein 3	19937.7793	18326.88	17996.76	11257.74	**16879.79004**	16708.92	18921.7	18499.38	20903.53	**18758.38281**	**0.8999**	**0.4066**
Q8C398	Pigw	Phosphatidylinositol-glycan biosynthesis class W protein	114940.2578	113652.5	105786.4	96374.16	**107688.3496**	134106.6	131088.5	90291.69	123484.9	**119742.9121**	**0.8993**	**0.3127**
Q99K01	Pdxdc1	Pyridoxal-dependent decarboxylase domain-containing protein 1	43718.16016	43483.85	42716.99	36804.7	**41680.92383**	46388.64	40943.01	51650.11	46420.04	**46350.45117**	**0.8993**	**0.1383**
P49312	Hnrnpa1	Heterogeneous nuclear ribonucleoprotein A1	101675.7188	106066.1	105798.7	95906.71	**102361.8262**	113889.5	113665.4	118068	109723	**113836.4395**	**0.8992**	**0.0078**
P84104	Srsf3	Serine/arginine-rich splicing factor 3	182426.5156	160402.4	183491.5	190050.5	**179092.7305**	221206.8	201505.8	179214.7	194939.4	**199216.6797**	**0.8990**	**0.1125**
P04925	Prnp	Major prion protein	51057.19531	40098.52	32276.41	25265.4	**37174.38135**	45844.1	44988.86	37248.34	37327.77	**41352.2666**	**0.8990**	**0.5130**
Q8BQZ4	Ralgapb	Ral GTPase-activating protein subunit beta	14741.64551	14627.21	12746.81	12188.61	**13576.06885**	14722.68	15299.76	13219.37	17180.52	**15105.58057**	**0.8987**	**0.1939**
Q9JKV5	Scamp4	Secretory carrier-associated membrane protein 4	51248.22266	24078.14	41719.63	41713.56	**39689.88867**	48242.68	47750.64	43199.31	37477.19	**44167.4541**	**0.8986**	**0.4971**
Q9R226	Khdrbs3	KH domain-containing, RNA-binding, signal transduction-associated protei	11447.09277	16052.14	8537.765	10739.54	**11694.13354**	17294.71	16021.99	13669.45	5104.048	**13022.54993**	**0.8980**	**0.6894**
Q9JKK7	Tmod2	Tropomodulin-2	196474.375	179711.1	186533.6	163488	**181551.7539**	217038	198467.5	193150.3	200067.7	**202180.8633**	**0.8980**	**0.0544**
O55102	Bloc1s1	Biogenesis of lysosome-related organelles complex 1 subunit 1		6750.581		4085.364	**5417.972656**	4564.43	7504.334			**6034.381836**	**0.8979**	**0.7854**
Q8C5L3	Cnot2	CCR4-NOT transcription complex subunit 2	8510.686523	8697.448	8703.505	9230.488	**8785.531982**	9105.857	9408.861	10556.35	10076.24	**9786.826172**	**0.8977**	**0.0325**
Q9Z2B2	Slc25a14	Brain mitochondrial carrier protein 1		2805.109		1875.346	**2340.227722**			2904.082	2311.137	**2607.609497**	**0.8975**	**0.6756**
Q8R3Z5	Cacnb1	Voltage-dependent L-type calcium channel subunit beta-1		23529.87	14120	13160.38	**16936.75065**	16896.91	20860.44	14516.8	23217.57	**18872.93042**	**0.8974**	**0.6142**
P21300	Akr1b7	Aldo-keto reductase family 1 member B7	100425.3125	107952.3	85769.05	89263.73	**95852.59766**	103921.1	96832.26	118771	107747.9	**106818.0742**	**0.8973**	**0.1609**
Q7TSI3	Ppp6r1	Serine/threonine-protein phosphatase 6 regulatory subunit 1	7327.757813	5992.499	2683.783	2876.763	**4720.200439**	4989.073	2125.554	6501.105	7431.45	**5261.795471**	**0.8971**	**0.7519**
Q924T2	Mrps2	Small ribosomal subunit protein uS2m	10561.08203	10599.7	12698.62	12658.3	**11629.42529**	15662.58	12452.99	14705.66	9042.051	**12965.82031**	**0.8969**	**0.4330**
Q8CCP0	Nemf	Ribosome quality control complex subunit NEMF	5562.064453	6866.127	4442.577	3917.419	**5197.046814**	6769.665	3525.789	8695.84	4188.538	**5794.957642**	**0.8968**	**0.6758**
Q8BL80	Arhgap22	Rho GTPase-activating protein 22	135034.9531	120168.2	110503.6	102992.7	**117174.8691**	125518.3	128698.9	127137.4	141333	**130671.8867**	**0.8967**	**0.1343**
Q8K183	Pdxk	Pyridoxal kinase	104309.0547	99565.96	95741.23	88228.51	**96961.1875**	108612.8	105983.3	106935	111153.7	**108171.1973**	**0.8964**	**0.0203**
P56391	Cox6b1	Cytochrome c oxidase subunit 6B1	866504.625	847679.2	836087.9	697867.6	**812034.8281**	812695.9	845767.3	1019464	946398.3	**906081.2344**	**0.8962**	**0.1742**
Q5SWP3	Nacad	NAC-alpha domain-containing protein 1	18143.91797	18804.28	15832.66	13623.5	**16601.09058**	17188.92	19582.04	18209.96	19119.38	**18525.07373**	**0.8961**	**0.1872**
Q00262	Stx2	Syntaxin-2			2363.295		**2363.295166**	2450.067	2753.215		2708.457	**2637.246501**	**0.8961**	
Q3UPH7	Arhgef40	Rho guanine nucleotide exchange factor 40	2186.979492		4035.445		**3111.21228**	3031.792	2099.687	4845.895	3911.052	**3472.106384**	**0.8961**	**0.7481**
Q9CQ20	Mid1ip1	Mid1-interacting protein 1	7838.121094	6590.806	8556.998	5382.27	**7092.048584**	7458.647	5556.03	10010.73	8633.514	**7914.730103**	**0.8961**	**0.5099**
O09126	Sema4d	Semaphorin-4D	12369.49707	11519.09	12370.71	9775.133	**11508.6084**	14475.93	11633.06	10263.9	15005.93	**12844.7063**	**0.8960**	**0.3400**
Q8R1I2	Grp	Gastrin-releasing peptide	4356.396484	7862.87	14494.5	16175.32	**10722.26965**	6031.03	7355.167	12181.67	22317.85	**11971.43091**	**0.8957**	**0.7960**
Q91ZP9	Necab2	N-terminal EF-hand calcium-binding protein 2	42701.36719	37247.12	30236.16	39270.03	**37363.66846**	43552.61	42718.85	40344.82	40338.57	**41738.71387**	**0.8952**	**0.1634**
O55013	Trappc3	Trafficking protein particle complex subunit 3	19098.35742	25820.97	22385.04	22383.45	**22421.95557**	17535.92	23428.36	36034.01	23196.76	**25048.76123**	**0.8951**	**0.5493**
Q8VEK2	Rhbdd2	Rhomboid domain-containing protein 2	12997.09766	13588.37	13676.02	15311.6	**13893.27124**	17172.1	16490.47	14796.09	13637.97	**15524.15601**	**0.8949**	**0.1348**
Q9CSU0	Rprd1b	Regulation of nuclear pre-mRNA domain-containing protein 1B	7701.972168	7958.542	5452.482	5951.257	**6766.063232**	10836.84	8506.754	3455.682	7446.165	**7561.361389**	**0.8948**	**0.6494**
Q8CDM8	Fhip2a	FHF complex subunit HOOK interacting protein 2A	14022.43164	14202.66	6611.45	2919.935	**9439.118958**	9967.328	11411.46	8622.795	12195.2	**10549.19458**	**0.8948**	**0.7161**
P97393	Arhgap5	Rho GTPase-activating protein 5	19109.59961	19023.44	18235.21	14231.81	**17650.0166**	21614.7	19598.32	18769.52	18933.55	**19729.02295**	**0.8946**	**0.1686**
P30677	Gna14	Guanine nucleotide-binding protein subunit alpha-14	8437.808594	10441.46	3209.764	2.44334	**5522.868464**	2.418788	16583.04	3780.358	4329.31	**6173.78237**	**0.8946**	**0.8852**
Q8K394	Plcl2	Inactive phospholipase C-like protein 2	11763.2334	11623.22	10264.05	13877.94	**11882.11304**	12309.34	12840.88	15421.1	12560.77	**13283.02124**	**0.8945**	**0.2257**
Q8BVU0	Lrch3	DISP complex protein LRCH3			1475.077	8481.404	**4978.240662**			5565.832		**5565.832031**	**0.8944**	
P50153	Gng4	Guanine nucleotide-binding protein G(I)/G(S)/G(O) subunit gamma-4	131878.5938	132707.3	133255.4	132943.5	**132696.1992**	155678.2	151598.6	151245	134918.8	**148360.1484**	**0.8944**	**0.0144**
A3KMP2	Ttc38	Tetratricopeptide repeat protein 38	9379.760742	8169.441	6183.977	5401.771	**7283.737427**	8363.654	8007.459	8335.112	7870.791	**8144.25415**	**0.8943**	**0.3846**
P61089	Ube2n	Ubiquitin-conjugating enzyme E2 N	598789.375	711532.9	540817.5	532248.4	**595847.0625**	670892.7	643796.3	661971	688763.4	**666355.8281**	**0.8942**	**0.1469**
Q921G8	Tubgcp2	Gamma-tubulin complex component 2	9820.353516	8299.036	8345.988	7896.375	**8590.43811**	9980.27	9404.658	9365.415	9679.021	**9607.341064**	**0.8942**	**0.0626**
Q8BG05	Hnrnpa3	Heterogeneous nuclear ribonucleoprotein A3	190041.8281	187035.7	188343.1	165393.8	**182703.6133**	197248.3	200255.2	211070.4	208870.8	**204361.1953**	**0.8940**	**0.0177**
Q8BLK3	Lsamp	Limbic system-associated membrane protein	64184.84375	55013.43	45535.09	52779.42	**54378.19824**	59073.7	63962.5	59024.38	61266.59	**60831.79199**	**0.8939**	**0.1593**
P28656	Nap1l1	Nucleosome assembly protein 1-like 1	63555.94141	74600.95	56264.41	48991.62	**60853.23047**	79363.66	67838.2	56925.04	68335.8	**68115.67383**	**0.8934**	**0.3477**
P40630	Tfam	Transcription factor A, mitochondrial	38281.82813	40246.7	45241.12	45326.46	**42274.02441**	44329.68	47317.7	44414.07	53228.15	**47322.40039**	**0.8933**	**0.1156**
Q3TKY6	Cwc27	Spliceosome-associated protein CWC27 homolog	2376.424316	4249.835			**3313.129639**	3147.732	3454.778	5507.319	2727.012	**3709.210327**	**0.8932**	**0.7346**
Q3TCH7	Cul4a	Cullin-4A	2011.47583	3110.477	6863.667	7598.15	**4895.942383**	8902.248	2779.949	5280.863	4963.301	**5481.590332**	**0.8932**	**0.7648**
Q02819	Nucb1	Nucleobindin-1	23741.17383	21455.08	21081.85	24337.46	**22653.89307**	22922.39	27871.37	24950.29	25714.59	**25364.65918**	**0.8931**	**0.0832**
Q9JKN6	Nova1	RNA-binding protein Nova-1	77211.82813	81336.48	42672.02	95063.45	**74070.94531**	82217.12	82801.48	88685.58	78110.13	**82953.57422**	**0.8929**	**0.4636**
P01863;P01865	Ighg;Igh-1a	Ig gamma-2A chain C region, A allele;Ig gamma-2A chain C region, membrane-bound form		1770.322		1352.524	**1561.42334**	1894.601	1706.386	1855.315	1538.91	**1748.802887**	**0.8929**	**0.3477**
Q9Z2D8	Mbd3	Methyl-CpG-binding domain protein 3	9384.044922	5453.401	7674.876	6585.305	**7274.406738**	10858.39	5075.852	7869.996	8787.474	**8147.928711**	**0.8928**	**0.5722**
B2RY56	Rbm25	RNA-binding protein 25	17008.24805	14962.95	17224.04	14160.68	**15838.97803**	18484.12	20905.19	17071.26	14511.36	**17742.97974**	**0.8927**	**0.2614**
Q99ME2	Wdr6	tRNA (34-2'-O)-methyltransferase regulator WDR6	9445.881836	9285.969	5120.142	4686.675	**7134.666748**	8887.284	8521.811	8500.258	6066.052	**7993.851196**	**0.8925**	**0.5739**
Q8BH69	Sephs1	Selenide, water dikinase 1	19005.10352	16209.98	17022.28	19064.09	**17825.36255**	32638.05	14472.58	18675.82	14117.97	**19976.10303**	**0.8923**	**0.6427**
Q8BTJ4	Enpp4	Bis(5'-adenosyl)-triphosphatase enpp4	15762.8291	8948.359	16252.88	7847.633	**12202.92627**	7964.729	12418.12	19461.34	14870.73	**13678.7323**	**0.8921**	**0.6669**
Q8CFE6	Slc38a2	Sodium-coupled neutral amino acid symporter 2	11606.69629	9794.027	11244.19	10709.46	**10838.59302**	12256.88	14188.6	10311.61	11866.32	**12155.85205**	**0.8916**	**0.1890**
Q3UIU2	Ndufb6	NADH dehydrogenase [ubiquinone] 1 beta subcomplex subunit 6	269634.2813	233457.6	264487.7	244952.6	**253133.0508**	274969.7	291870.8	306628.8	262792.9	**284065.5469**	**0.8911**	**0.0519**
Q8BY87	Usp47	Ubiquitin carboxyl-terminal hydrolase 47	8983.666016	12865.16	9821.302	11866.68	**10884.20361**	13268.33	12828.41	10972.55	11789.09	**12214.59619**	**0.8911**	**0.2458**
O55100	Syngr1	Synaptogyrin-1	57349.15625	113943.7	106028.6	102004.3	**94831.45313**	81987.85	97572.66	172582.7	73627.34	**106442.6426**	**0.8909**	**0.6701**
P17918	Pcna	Proliferating cell nuclear antigen		5212.616	2261.185	4112.587	**3862.129395**	3771.178	5784.748		3450.61	**4335.511882**	**0.8908**	**0.6966**
Q6PEE3	Rrm2b	Ribonucleoside-diphosphate reductase subunit M2 B	4281.495605	2458.234	2923.762		**3221.164063**	1762.031	6126.182	2929.737	3649.565	**3616.878723**	**0.8906**	**0.7517**
Q6PNC0	Dmxl1	DmX-like protein 1	11928.69824	9456.007	11238.21	9403.469	**10506.59668**	12271.39	10563.29	13420.15	10935.19	**11797.50659**	**0.8906**	**0.2071**
Q9D882	Fam241b	Protein FAM241B	13754.14844	12238.53	7674.359	2960.387	**9156.856995**	12082.18	12780.04		6000.858	**10287.69336**	**0.8901**	**0.7529**
Q9JL35	Hmgn5	High mobility group nucleosome-binding domain-containing protein 5	16208.38965	19625.05	12948.37	11945.57	**15181.84546**	16441.84	20292.09	14191.99	17321.85	**17061.94189**	**0.8898**	**0.4151**
Q61503	Nt5e	5'-nucleotidase	20112.26758	14611.78	15573.54	14039.16	**16084.18457**	16610.03	20278.39	18174.25	17250.33	**18078.25049**	**0.8897**	**0.2578**

Q8BZH4	Pogz	Pogo transposable element with ZNF domain	2910.930176	1577.177	1832.554		**2106.887044**		1422.715	3507.149	2175.873	**2368.578857**	0.8895	0.7394
Q9CPT4	Mydgf	Myeloid-derived growth factor	28134.97266	23363.72	25138.87	29011.13	**26412.17139**	31392.3	27767.62	34688.14	24928.86	**29694.22949**	0.8895	0.2369
O88741	Gdap1	Ganglioside-induced differentiation-associated protein 1	51574.35938	48274.48	48875.21	51339.68	**50015.93457**	53632.38	52064.82	64585.78	54791.94	**56268.72949**	0.8889	0.0784
Q05186	Rcn1	Reticulocalbin-1	3215.109375		4416.745	4893.632	**4175.161947**		3753.201	5641.312		**4697.25647**	0.8889	0.6224
Q99N85	Mrps18a	Large ribosomal subunit protein mL66		11525.13	10167.82	7213.283	**9635.412598**	13558.08	11431	12123.07	6260.242	**10843.09924**	0.8886	0.6006
Q8C0L9	Gpcpd1	Glycerophosphocholine phosphodiesterase GPCPD1	25402.96289	26871.76	21484.13	21994.82	**23938.41992**	25265.33	25979.9	26721.8	29791.82	**26939.71289**	0.8886	0.1178
Q91VM9	Ppa2	Inorganic pyrophosphatase 2, mitochondrial	88514.5	93495.34	79606.18	75198.45	**84203.61719**	98842.88	86373.12	92083.62	101789.6	**94772.29102**	0.8885	0.0984
Q920N7	Syt12	Synaptotagmin-12	55950.07422	59414.28	62751.48	58939.87	**59263.92676**	69204.89	72358.75	60748.64	64507.19	**66704.86621**	0.8884	0.0432
Q8BMF3	Me3	NADP-dependent malic enzyme, mitochondrial	42538.92188	42707.14	40638.14	42659.46	**42135.91504**	43450.89	47508.21	48795.02	49990.91	**47436.25879**	0.8883	0.0126
Q61771	Kif3b	Kinesin-like protein KIF3B	6162.384277	6542.927	6077.851	5114.758	**5974.480103**	9487.521	6607.673	5220.64	5589.416	**6726.312622**	0.8882	0.4859
Q3UUQ7	Pgap1	GPI inositol-deacylase	12363.20215	10853.78	10600.49	8817.553	**10658.75562**	12618.33	10237.51	13728.56	11420.68	**12001.27026**	0.8881	0.2470
Q9DB32	Haghl	Hydroxyacylglutathione hydrolase-like protein	28914.60352	29945.85	27016.34	29084.06	**28740.21338**	31093.26	28466.85	34144.05	35737.84	**32360.50098**	0.8881	0.0813
P97355	Sms	Spermine synthase	23138.47266	28324.3	23555.31	24678.69	**24924.19287**	24153.76	27448.08	32102.47	28558.44	**28065.68848**	0.8881	0.1707
Q9D125	Mrps25	Small ribosomal subunit protein mS25	12090.50195	12597.22	9566.192	9368.835	**10905.68848**	11704.81	11983.82	13203.14	12248.6	**12285.08984**	0.8877	0.1757
P05532	Kit	Mast/stem cell growth factor receptor Kit	5238.854004	1025.83		6466.747	**4243.810059**	6845.327	4366.817	4234.944	3675.424	**4780.627991**	0.8877	0.7525
Q9R0P3	Esd	S-formylglutathione hydrolase	183182.9844	285430.1	192203.3	140789.1	**200401.3711**	264999.7	217703.5	212981.6	207458.3	**225785.7695**	0.8876	0.4739
Q8VC88	Gca	Grancalcin	20998.50781	23251.96	21026.86	13654.55	**19732.96899**	24190.11	16123.58		26401.66	**22238.45215**	0.8873	0.5176
P55288	Cdh11	Cadherin-11	13425.19336	14191.65	12714	10772.35	**12775.79663**	16324.09	12176.14	15747.02	13351.92	**14399.79126**	0.8872	0.2331
Q61418	Clcn4	H(+)/Cl(-) exchange transporter 4	41034.48438	38636.84	35289.99	37124.38	**38021.4248**	44083.51	53081.32	34823.35	39432.22	**42855.09863**	0.8872	0.2812
Q61550	Rad21	Double-strand-break repair protein rad21 homolog	15717.08008	17440.2	13720.1	8625.83	**13875.80151**	15731.62	18349.64	12280.51	16212.3	**15643.51904**	0.8870	0.4685
Q9Z0M5	Lipa	Lysosomal acid lipase/cholesteryl ester hydrolase	13840.79785	7499.705	14474.55	13261.7	**12269.18738**	18121.37	16883.1	7933.288	12407.9	**13836.41321**	0.8867	0.5988
Q6ZWU9	Rps27	Small ribosomal subunit protein eS27	20211.1582	19277.3	10718.33	17337.75	**16886.13599**	25404.05	20617.66	15959.24	14192.41	**19043.34253**	0.8867	0.5380
Q9QYJ0	Dnaja2	DnaJ homolog subfamily A member 2	78358.85156	71346.74	72456.53	59534.65	**70424.19434**	83478.76	81790.91	73523.66	78933.11	**79431.60742**	0.8866	0.0925
P05977	Myl1	Myosin light chain 1/3, skeletal muscle isoform		47588.96		19076.55	**33332.75195**	7822.111	43013.38		61958.2	**37597.89632**	0.8866	0.8651
Q8BI72	Cdkn2aip	CDKN2A-interacting protein	5682.510742		7652.782	6413.914	**6583.068685**	6241.318		9378.237	6659.264	**7426.273112**	0.8865	0.5003
Q9D7M1	Gid8	Glucose-induced degradation protein 8 homolog	10733.10449	16258.55	16062.73	12565.08	**13904.86646**	18601.23	17219.77	10958.4	15967.23	**15686.65845**	0.8864	0.4384
Q6ZWM4	Lsm8	U6 snRNA-associated Sm-like protein LSm8	17393.98633	11974.78	13108.18	14448.27	**14231.30347**	15247.18	14952.36	18787.74	15256.25	**16060.88306**	0.8861	0.2633
Q9CRA9	Fgfr1op2	FGFR1 oncogene partner 2 homolog	13334.28223	8565.422	6953.261	11544.08	**10099.26233**	9326.065	12253.6	12971.67	11052.45	**11400.9458**	0.8858	0.4586
P62897	Cycs	Cytochrome c, somatic	1235032.5	1193665	1040106	1159842	**1157161.172**	1258301	1270923	1296544	1401669	**1306859.156**	0.8855	0.0304
O54829	Rgs7	Regulator of G-protein signaling 7	30022.38867	30186.83	27551.69	26664.97	**28606.46924**	30446.39	33079.7	32987.73	32722.17	**32309**	0.8854	0.0142
P43006	Slc1a2	Excitatory amino acid transporter 2	721184.5	730915.1	758778.4	757803.2	**742170.2969**	685022.6	730758.2	1148912	789487.2	**838544.9063**	0.8851	0.3986
Q7TNM2	Trim46	Tripartite motif-containing protein 46	9920.22168	7326.433	7452.115	7363.272	**8015.510376**	10154.41	8025.732	8468.434	9589.854	**9059.607422**	0.8848	0.2414
Q8BGC0	Htatsf1	HIV Tat-specific factor 1 homolog	11850.45117	10799.07	8042.087	13749.51	**11110.28113**	11070.76	12384.33	12048.72	14728.1	**12557.97607**	0.8847	0.3476
P47856	Gfpt1	Glutamine--fructose-6-phosphate aminotransferase [isomerizing] 1	16848.48242	15815.46	16354.98	15242.55	**16065.36597**	16477.69	18730.04	19736.16	17694.91	**18159.70166**	0.8847	0.0362
Q9R0P6	Sec11a	Signal peptidase complex catalytic subunit SEC11A	14312.73438	11841.52	11702.19	12674.02	**12632.61548**	9037.455	15764.95	7032.085	25289.22	**14280.92822**	0.8846	0.7057
Q8K209	Adgrg1	Adhesion G-protein coupled receptor G1	28436.65234	26270.23	30784.79	33096.42	**29647.02148**	33340.16	32834.87	35714.23	32182.17	**33517.85693**	0.8845	0.0588
Q6PD19	Armh3	Armadillo-like helical domain-containing protein 3	11778.08789	7635.767	8311.354	4272.44	**7999.411987**	13078.96	9566.124		4487.276	**9044.120605**	0.8845	0.7214
Q91YJ5	Mtif2	Translation initiation factor IF-2, mitochondrial	8172.22168	8308.873	14227.43	17726.63	**12108.78833**	17421.9	14917.36	16935.7	5498.718	**13693.42041**	0.8843	0.6786
P63239	Pcsk1	Neuroendocrine convertase 1	15066.75293	11405.58	11421.11	14978.17	**13217.90234**	15464.62	15269.17	15246.56	13812.03	**14948.09326**	0.8843	0.1700
O88520	Shoc2	Leucine-rich repeat protein SHOC-2	149592.75	148188.3	113365.7	138435	**137395.4297**	152165.9	153013.3	153208	163175.3	**155390.6484**	0.8842	0.0863
Q80YV4	Pank4	4'-phosphopantetheine phosphatase	25312.92188	23991.05	21098.19	26005.7	**24101.96533**	27625.45	30266.37	26581.82	24567.57	**27260.30176**	0.8841	0.0970
Q9WU28	Pfdn5	Prefoldin subunit 5	15259.82813	14930.49	14540.75	12857.56	**14397.15625**	15502.06	14276.86	17524.36	17853.26	**16289.1377**	0.8839	0.1081
Q78T54	Vma21	Vacuolar ATPase assembly integral membrane protein Vma21	79536.57813	88912.73	73036.04	83210.66	**81174.00195**	103601.1	82725.88	112426.6	68697.18	**91862.68359**	0.8836	0.3464
Q9ES89	Extl2	Exostosin-like 2	22405.37891	20232.15	15271.88	14128.17	**18009.39502**	23876.48	20921.87	19685	17047.94	**20382.81982**	0.8836	0.3666
Q99K28	Arfgap2	ADP-ribosylation factor GTPase-activating protein 2	10664.1416	8627.757	10701.46	8494.717	**9622.017822**	11448.02	8604.305	12180.91	11337.01	**10892.56152**	0.8834	0.2494
E9Q4P1	Wdfy1	WD repeat and FYVE domain-containing protein 1	23696.07813	27474.53	8668.488		**19946.36589**	25405.82	25303.69	17514.18	22113.21	**22584.22705**	0.8832	0.6387
Q8CH72	Trim32	E3 ubiquitin-protein ligase TRIM32	16201.55566	17872.49	15027.76	13233.31	**15583.77881**	24241.04	16704.71	20973.94	8663.301	**17645.74512**	0.8831	0.5780
Q8K3E5	Ahi1	Jouberin	18786.59766	19377.54	17492.77	20236.73	**18973.40967**	20609.51	18880.91	17811.29	28645.87	**21486.89404**	0.8830	0.3574
Q8VCA8	Scrn2	Secernin-2	14601.41211	14811.81	12680.08	10579.88	**13168.29736**	14654.92	16836.38	12733.77	15452.53	**14919.40088**	0.8826	0.2288
Q99M87	Dnaja3	DnaJ homolog subfamily A member 3, mitochondrial	53494.05078	49842.63	52687.24	44409.27	**50108.29492**	59068.16	65393.64	58867.3	43800.05	**56782.29004**	0.8825	0.2324
P0DN89	Tmem254	Transmembrane protein 254	16676.91602	18457.66			**17567.28711**	18227.08		19805.86	21688.65	**19907.19596**	0.8825	0.2055
Q8VHP7	Serpinb1b	Leukocyte elastase inhibitor B	196236.75	203161.3	168898.7	234012	**200577.1797**	213205.9	207273.7	232656.8	256103	**227309.8438**	0.8824	0.1739
P56212	Arpp19	cAMP-regulated phosphoprotein 19	29505.05664	30030.73	26988.38	23842.07	**27591.55811**	30491.37	33456.53	30418	30764.17	**31282.51758**	0.8820	0.0595
P62331	Arf6	ADP-ribosylation factor 6	66786.66406	74581.16	70230.69	71604.34	**70800.71289**	89421.28	71082.28	86293.32	74379.58	**80294.11523**	0.8818	0.0925
Q8VHJ5	Mark1	Serine/threonine-protein kinase MARK1	21823.41797	20019.1	10382.7	17039.11	**17316.08081**	20368.33	20673.9	21047.38	16475.88	**19641.37402**	0.8816	0.4269
P16254	Srp14	Signal recognition particle 14 kDa protein	19926.89453	25253.15	16722.76	15469.36	**19343.04126**	22870.61	23050.04	20461.08	21385.49	**21941.8042**	0.8816	0.2955
Q4PJX1	Odr4	Protein odr-4 homolog	14127.25879	12334.71	11518.45	12956.09	**12734.12573**	16743.07	12210.29	13423.95	15413.56	**14447.71606**	0.8814	0.1869
Q9DB34	Chmp2a	Charged multivesicular body protein 2a	38401.82813	29844.68	23494.83	22443.77	**28546.2749**	31754.83	33643.83	31441.93	32714.16	**32388.68848**	0.8814	0.3394
Q8BU88	Mrpl22	Large ribosomal subunit protein uL22m	10615.99512	9798.588	8034.647	8569.699	**9254.7323**	9882.161	11039.23	11706.35	9379.671	**10501.85132**	0.8812	0.1655
O35526	Stx1a	Syntaxin-1A	18194.75586	16482.07	12978.99	14180.12	**15458.98389**	20547.51	19237.65	15804.4	14579.64	**17542.30103**	0.8812	0.2974
Q9D6J5	Ndufb8	NADH dehydrogenase [ubiquinone] 1 beta subcomplex subunit 8, mitochor	183435.9844	181820.9	143554.9	172846.2	**170414.5234**	186969.5	189787.5	208154.5	188663.1	**193393.6484**	0.8812	0.0711
Q80U40	Rimbp2	RIMS-binding protein 2	9762.584961	11477.93	10687.56	9242.717	**10292.69751**	13195.64	9357.19	13096.54	11092.48	**11685.46167**	0.8808	0.2292
Q3V3V9	Carmil2	Capping protein, Arp2/3 and myosin-I linker protein 2	16766.99609	11964.1	16987.59	15858.58	**15394.31714**	18578.02	15884.7	17533.53	17955.55	**17487.94824**	0.8803	0.1593
Q91YX5	Lpgat1	Acyl-CoA:lysophosphatidylglycerol acyltransferase 1	19103.56445	21652.79	19253.37	13750.24	**18439.99219**	24030.94	18614.12	24901.36	16247.91	**20948.58105**	0.8803	0.3851
Q3V384	Afg1l	AFG1-like ATPase	21948.30273	22541.96	26848.31	21239.16	**23144.43359**	24760.24	23529.79	30542.46	26339.94	**26293.10889**	0.8802	0.1634

Q9CQ40	Mrpl49	Large ribosomal subunit protein mL49	14344.05566	13100.27	12058.18	9199.505	**12175.50269**	16582.16	13187.31	14181.61	11381.91	**13833.24854**	**0.8802**	**0.3237**
Q8K4F5	Abhd11	Protein ABHD11	23494.68555	24708.71	21155.07	21023.9	**22595.5918**	24140	23580.38	27531.92	27450.91	**25675.80322**	**0.8800**	**0.0685**
Q925E7	Ppp2r2d	Serine/threonine-protein phosphatase 2A 55 kDa regulatory subunit B delta	13593.89941	16579.81	17510.74	16751.05	**16108.87378**	29748.88	13381.85	18084.86	12015.85	**18307.85938**	**0.8799**	**0.6127**
P60521	Gabarapl2	Gamma-aminobutyric acid receptor-associated protein-like 2	39296.375	40930.55	34597.14	39483.91	**38576.99316**	39019.12	44283.87	49344.07	42742.48	**43847.38379**	**0.8798**	**0.0837**
Q8C008	Dzank1	Double zinc ribbon and ankyrin repeat-containing protein 1	21747.12109	18144.79	19859.5	10631.67	**17595.77124**	21669.71	22432.12	18826.53	17085.52	**20003.46924**	**0.8796**	**0.4125**
Q60931	Vdac3	Voltage-dependent anion-selective channel protein 3	761115.1875	800620.6	717781.1	721758.9	**750318.9531**	826909.5	847670.5	915624.3	821970	**853043.5625**	**0.8796**	**0.0122**
Q9Z255	Ube2a	Ubiquitin-conjugating enzyme E2 A		6992.928	6387.583		**6690.255127**	7608.395				**7608.39502**	**0.8793**	
Q3TIV5	Zc3h15	Zinc finger CCCH domain-containing protein 15	5354.615234	7478.95	6939.574	7048.303	**6705.360474**	6896.977	7979.501	8000.9	7629.686	**7626.765747**	**0.8792**	**0.1338**
P99028	Uqcrh	Cytochrome b-c1 complex subunit 6, mitochondrial	31712.49219	33492.83	23224.3	38547.21	**31744.20947**	51011.25	40413.85	21864.88	31179.48	**36117.36816**	**0.8789**	**0.5557**
P18826	Phka1	Phosphorylase b kinase regulatory subunit alpha, skeletal muscle isoform	13899.55859	11097.36	11868.85	6460.197	**10831.4917**	10741.63	10786.05	13005.97	14796.25	**12332.47632**	**0.8783**	**0.4484**
Q8BM55	Tmem214	Transmembrane protein 214	1362.790894	2175.233	2296.427		**1944.816854**	3065.071	1957.808	2386.422	1452.242	**2215.385651**	**0.8779**	**0.5918**
Q8C8T7	Elfn1	Protein ELFN1	7300.729004	7996.13	6082.235	7087.187	**7116.57019**	9171.453	8197.82	6713.196	8345.72	**8107.047241**	**0.8778**	**0.1765**
Q8BSF4	Pisd	Phosphatidylserine decarboxylase proenzyme, mitochondrial	12984.9375	14561.22	16142.5	12011.68	**13925.08472**	18491.91	14699.68	15756.08	14507.93	**15863.90088**	**0.8778**	**0.1836**
Q6PAK3	Prmt8	Protein arginine N-methyltransferase 8	24652.81836	27143.26	21649.21	22220.97	**23916.56543**	27912.26	28912.65	28821.23	23369.04	**27253.7959**	**0.8775**	**0.1162**
Q80U35	Arhgef17	Rho guanine nucleotide exchange factor 17	3895.059814	8028.367	127.3165	3345.549	**3849.073076**				4386.92	**4386.919922**	**0.8774**	
Q5SV85	Synrg	Synergin gamma	14257.38477	13759.4	14915.14	13825.19	**14189.27979**	15872.08	17825.8	16275.39	14718.7	**16172.99341**	**0.8773**	**0.0290**
Q63810	Ppp3r1	Calcineurin subunit B type 1	21686.42188	41877.79	16686.74	19881.97	**25033.22852**	30662.78	26856.82	32350.95	24282.17	**28538.18018**	**0.8772**	**0.5800**
Q80X71	Tmem106b	Transmembrane protein 106B	9093.335938	9640.872	11529.75	11030.79	**10323.68677**	11046.79	18205.31	10909.38	6924.399	**11771.47131**	**0.8770**	**0.5711**
Q6VNS1	Ntrk3	NT-3 growth factor receptor	10068.83301		14071.1	7862.903	**10667.61133**		7857.119	12584.17	16052.75	**12164.68001**	**0.8769**	**0.6429**
Q64314	Cd34	Hematopoietic progenitor cell antigen CD34	5135.434082			9840.268	**7487.85083**	8541.918				**8541.917969**	**0.8766**	
Q9WUP7	Uchl5	Ubiquitin carboxyl-terminal hydrolase isozyme L5	19227.29102	20584.83	19545.51	18214.81	**19393.10938**	17788.58	20684.77	24982.81	25045.21	**22125.33838**	**0.8765**	**0.1872**
Q9DBC0	Selenoo	Protein adenylyltransferase SelO, mitochondrial	16066.3584	17544.06	14554.56	18878.78	**16760.93945**	16364.43	17664.63	24881.15	17629.24	**19134.86084**	**0.8759**	**0.3122**
Q9CR30	Josd2	Josephin-2	24154.48438	24202.8	20645.44	16053.91	**21264.15649**	24160.76	22961.68	24229.22	25791.36	**24285.75488**	**0.8756**	**0.1838**
Q08943	Ssrp1	FACT complex subunit SSRP1	13696.52734	10845.09	6750.909	12543.7	**10959.05847**	10808.31	14785.1	11999.33	12474.6	**12516.83374**	**0.8755**	**0.4035**
Q8K2F8	Lsm14a	Protein LSM14 homolog A			2566.996	1535.807	**2051.40155**		1974.979	3358.757	1696.186	**2343.307251**	**0.8754**	**0.7288**
Q7TSS2	Ube2q1	Ubiquitin-conjugating enzyme E2 Q1	38708.98438	40578.98	35916.79	30309.68	**36378.60986**	42115.71	39030.82	46522.43	38581.23	**41562.54688**	**0.8753**	**0.1232**
Q9JIX0	Eny2	Transcription and mRNA export factor ENY2	5115.958496	7642.757	11840.25	8643.524	**8310.621338**	10547.21	10722.8	7034.645	9678.415	**9495.767456**	**0.8752**	**0.4948**
O88737	Bsn	Protein bassoon	35696.83203	29452.92	35416.63	40422.07	**35247.11133**	31883.9	37660.08	43297.28	48289.43	**40282.6748**	**0.8750**	**0.2753**
Q14C51	Ptcd3	Small ribosomal subunit protein mS39	26070.04688	25977.71	24784.23	25439.11	**25567.77295**	30378.38	32427.73	27474.62	26608.94	**29222.4165**	**0.8749**	**0.0372**
Q6NZC7	Sec23ip	SEC23-interacting protein	23466.16211	29093.73	23849.03	19817.46	**24056.59375**	27557.89	27333.46	26676.96	28414.75	**27495.76367**	**0.8749**	**0.1270**
Q3URJ8	Nkain3	Sodium/potassium-transporting ATPase subunit beta-1-interacting protein	34920.45703		52879.39		**43899.92383**	43753.26	51775	62651.21	42525.41	**50176.2207**	**0.8749**	**0.5181**
Q80XK6	Atg2b	Autophagy-related protein 2 homolog B	9202.714844	8000.336	5957.901	6134.788	**7323.934937**	7557.986	12713.69	6722.955	6502.867	**8374.373901**	**0.8746**	**0.5498**
P62254	Ube2g1	Ubiquitin-conjugating enzyme E2 G1	20730.87891	21290.78	17928.9	20664.89	**20153.86182**	22459.76	22404.56	24836.16	22487.35	**23046.95801**	**0.8745**	**0.0238**
Q8R2U4	Ntmt1	N-terminal Xaa-Pro-Lys N-methyltransferase 1	17659.27344	7798.843	20977.52	19043.15	**16369.69666**		18927.57		18516.69	**18722.13281**	**0.8743**	**0.6218**
Q9ERB0	Snap29	Synaptosomal-associated protein 29	17950.03906	12662.23	13575.15	10905.21	**13773.15479**	18885.28	10253.79	14316.46	19571.24	**15756.69165**	**0.8741**	**0.4809**
Q0GNC1	Inf2	Inverted formin-2	26370.5	31780.46	32386.95	20434.88	**27743.19629**	28915.49	32668.51	34508.66	30867.5	**31740.04004**	**0.8741**	**0.2357**
Q3UW53	Niban1	Protein Niban 1	10345.75391	8584.893	6954.557	5752.903	**7909.526489**	3814.381	7660.112	11857.87	12865.71	**9049.518372**	**0.8740**	**0.6384**
Q6P9Q6	Fkbp15	FK506-binding protein 15	13076.57422	9903.729	10792.03	13318.51	**11772.71167**	11924.76	15674.64	12801.12	13485.89	**13471.60181**	**0.8739**	**0.1945**
P47753	Capza1	F-actin-capping protein subunit alpha-1	40901.94531	70282.9	43717.09	46481.77	**50345.92578**	54940.14	72157.91	50069.04	53301.22	**57617.07715**	**0.8738**	**0.4181**
Q9CZS1	Aldh1b1	Aldehyde dehydrogenase X, mitochondrial	198552	212185.2	242721.1	289189.1	**235661.8555**	286061.1	304540.6	288420.2	199774.8	**269699.1797**	**0.8738**	**0.3149**
Q9CQV7	Dnajc19	Mitochondrial import inner membrane translocase subunit TIM14	22926.94141	25642.59	18615.98	26568.97	**23438.62256**	27512.97	27993.95	28299.12	23594.75	**26850.19727**	**0.8729**	**0.1544**
Q9EQJ9	Magi3	Membrane-associated guanylate kinase, WW and PDZ domain-containing p	9285.860352	8784.538	9802.47	7829.103	**8925.492676**	11259.74	10903.77	9063.445	9685.087	**10228.01172**	**0.8727**	**0.0975**
O35623	Bet1	BET1 homolog	5811.338867	3311.18	4005.187		**4375.901937**	6651.17	5851.42	4387.728	3169.825	**5015.035828**	**0.8726**	**0.5888**
Q9WVL3	Slc12a7	Solute carrier family 12 member 7	13592.43848	14099.45	11547.96	13679.81	**13229.91479**	16539.26	14522.22	14592.97	15001.96	**15164.10181**	**0.8724**	**0.0400**
Q14CH0	Fam171b	Protein FAM171B	7615.976074	7937.244		5663.857	**7072.358887**	8428.278	9945.66	6376.464	7682.178	**8108.14502**	**0.8723**	**0.3746**
Q9CR89	Ergic2	Endoplasmic reticulum-Golgi intermediate compartment protein 2	2813.076416	1531.551			**2172.313782**	833.0978		3581.225	3058.215	**2490.845907**	**0.8721**	**0.8060**
Q8CCA0	Dcun1d4	DCN1-like protein 4	10302.73242	7175.392	9185.719	8259.838	**8730.920288**	8540.779	9273.658	12322.53	9913.591	**10012.64038**	**0.8720**	**0.2704**
Q91ZH7	Abhd3	Phospholipase ABHD3	13950.37891	15339.26	17003.61	18621.03	**16228.5686**	12965.43	16474.42	23301.84	21724.25	**18616.48462**	**0.8717**	**0.3919**
O35857	Timm44	Mitochondrial import inner membrane translocase subunit TIM44	45972.33594	38708.56	46136.49	40904.79	**42930.54395**	52914.09	57671.45	42352.49	44105.92	**49260.98633**	**0.8715**	**0.1718**
P28653	Bgn	Biglycan			50633.13		**50633.12891**			58105.23		**58105.23047**	**0.8714**	
P59235	Nup43	Nucleoporin Nup43	7762.375488	6748.92	5988.368	6937.225	**6859.222168**	8972.104	7910.26	8085.38	6529.018	**7874.190796**	**0.8711**	**0.1542**
Q9EP89	Lactb	Serine beta-lactamase-like protein LACTB, mitochondrial	27042.71094	23476.09	19404.85	20165.71	**22522.33838**	24936.34	25568.22	28705.18	24211.71	**25855.36182**	**0.8711**	**0.1479**
P53986	Slc16a1	Monocarboxylate transporter 1	35402.62109	55686.39	51468.82	37899.21	**45114.26172**	48085.39	50095.45	54997.59	54024.77	**51800.7998**	**0.8709**	**0.2497**
Q9D8Y0	Efhd2	EF-hand domain-containing protein D2	133056.8906	166827.3	131548.8	119962.2	**137848.7852**	143681.9	172658.5	160152.1	157051.5	**158385.9961**	**0.8703**	**0.1302**
P56394	Cox17	Cytochrome c oxidase copper chaperone	15868.76465	14666.48	12100.87	15232.68	**14467.19775**	17182.24	13863.63	21807.19	13646.15	**16624.80371**	**0.8702**	**0.3393**
Q9EST4	Psmg2	Proteasome assembly chaperone 2	50972.85156	15721.98	10023.8	18773.33	**23872.99072**	52397.85	33706.46	11565.96	12074.94	**27436.30176**	**0.8701**	**0.7998**
Q8BTV1	Tusc3	Tumor suppressor candidate 3	21474.61914	22165.79	20627.01	24168.67	**22109.02344**	24080.51	26584.21	26096.86	24922.46	**25421.00977**	**0.8697**	**0.0127**
Q60749	Khdrbs1	KH domain-containing, RNA-binding, signal transduction-associated protei	30794.82617	26919.7	32772.6	32781.99	**30817.27881**	29317.46	37082.23	37281.43	38054.66	**35433.94385**	**0.8697**	**0.1110**
Q8CH09	Sugp2	SURP and G-patch domain-containing protein 2	9177.443359	8117.277	8011.166	7041.462	**8086.837036**	9070.875	12002.78	10484.76	5648.569	**9301.746704**	**0.8694**	**0.4268**
Q8C8T8	Tsr2	Pre-rRNA-processing protein TSR2 homolog	24557.52734	22025.24	25410.94	25475.88	**24367.39893**	25935.62	24228.11	31628.77	30334.59	**28031.77197**	**0.8693**	**0.1071**
Q3UZV7	Elapor2	Endosome/lysosome-associated apoptosis and autophagy regulator family	10188.79395	9527.484	5822.3	10140.93	**8919.875977**	14919.28	7973.507	7140.999	11042.29	**10269.01917**	**0.8686**	**0.5345**
Q62193	Rpa2	Replication protein A 32 kDa subunit	5573.012695	6741.94	7633.738		**6649.563477**	5393.296	7109.349	9728.164	8394.915	**7656.431152**	**0.8685**	**0.4406**
Q9WVR4	Fxr2	RNA-binding protein FXR2	27161.43555	24771.35	25743.85	21480.46	**24789.27246**	29610.59	29449.58	28128.61	26994.08	**28545.71436**	**0.8684**	**0.0323**
Q9D8Z2	Triap1	TP53-regulated inhibitor of apoptosis 1		4261.638			**4261.638184**	4341.59		5475.191		**4908.390625**	**0.8682**	
Q9CQA3	Sdhb	Succinate dehydrogenase [ubiquinone] iron-sulfur subunit, mitochondrial	336420.6875	364773.1	311688	326697.7	**334894.8906**	376064.7	383250	413202.8	370715.9	**385808.3438**	**0.8680**	**0.0133**

Q99LT0	Dpy30	Protein dpy-30 homolog	11643.52734	13208.68	2029.402	1346.027	**7056.909821**	6268.109	9430.902	9180.465	7640.339	**8129.953735**	**0.8680**	**0.7492**
Q3TMH2	Scrn3	Secernin-3	37681.90625	32905.43	33871.58	24589.23	**32262.03809**	38458.73	38922.31	34535.27	36814.76	**37182.7666**	**0.8677**	**0.1441**
P70340	Smad1	Mothers against decapentaplegic homolog 1	9204.130859	9113.339	11759.48	9202.133	**9819.771484**	10812.34	11298.56	12543.64	10620.3	**11318.71143**	**0.8676**	**0.1024**
Q99KN9	Clint1	Clathrin interactor 1	21124.61914	23558.68	17077.73	15489.17	**19312.55298**	22436.92	25907.58	19804.63	20902.62	**22262.93652**	**0.8675**	**0.2424**
Q9DC16	Ergic1	Endoplasmic reticulum-Golgi intermediate compartment protein 1	35114.12891	42885.05	34294.95	30614.29	**35727.10596**	40224.51	42692.12	41781.32	40095.8	**41198.43652**	**0.8672**	**0.0849**
P60824	Cirbp	Cold-inducible RNA-binding protein	15112.74316	25513.19	34678.14	29338.22	**26160.57446**	26469.58	27711.54	34262.98	32278.96	**30180.76367**	**0.8668**	**0.4088**
P63330	Ppp2ca	Serine/threonine-protein phosphatase 2A catalytic subunit alpha isoform	50246.875	43080.24	37824.38	33672.11	**41205.90234**	44314.65	43752.5	50957.57	51188.32	**47553.26367**	**0.8665**	**0.1739**
Q9WTX5	Skp1	S-phase kinase-associated protein 1	90175.79688	70647.16	84787.46	94006.53	**84904.23828**	120593.5	82584.02	95006.27	94066.51	**98062.57422**	**0.8658**	**0.2160**
P62257	Ube2h	Ubiquitin-conjugating enzyme E2 H	12705.12891	12869.27	9837.856	9591.436	**11250.92261**	13214.06	14419.56	11459.8	12913.52	**13001.7334**	**0.8653**	**0.1552**
Q9D8P4	Mrpl17	Large ribosomal subunit protein bL17m	31726.00977	27775.76	21629.89	24449.51	**26395.29199**	30086.4	31919.78	30583.64	29457.72	**30511.88428**	**0.8651**	**0.1155**
Q9DB10	Smdt1	Essential MCU regulator, mitochondrial	25142.71289	30119.1	15709.39	28512.22	**24870.85767**	36058.95	36360.84		13860.22	**28760.00065**	**0.8648**	**0.6174**
Q8BX17	Gemin5	Gem-associated protein 5	3150.942383	3380.097		2903.845	**3144.961507**	4255.121	3496.833		3159.192	**3637.048584**	**0.8647**	**0.2347**
Q9DCC8	Tomm20	Mitochondrial import receptor subunit TOM20 homolog	4711.899414	4860.502	6154.337	3890.981	**4904.429993**	7255.326	5139.088	5290.465	5008.173	**5673.262939**	**0.8645**	**0.3188**
Q60963	Pla2g7	Platelet-activating factor acetylhydrolase	15846.04785	14281.22	15883.53	13315.78	**14831.64453**	18751.1	16043.31	17698.37	16137.12	**17157.4729**	**0.8644**	**0.0425**
Q8BFT9	Svop	Synaptic vesicle 2-related protein	16057.62109	9309.278	13281.94	7260.733	**11477.39355**	15249.55	14049.76	12851.69	10966.65	**13279.40918**	**0.8643**	**0.4389**
Q8BTU1	Cfap20	Cilia- and flagella-associated protein 20	8283.567383	4847.643	3875.079	6268.702	**5818.747803**	6248.606		7894.505	6055.909	**6733.006673**	**0.8642**	**0.4919**
Q6IRU5	Cltb	Clathrin light chain B	222578.6719	199397.9	190800.3	202715.4	**203873.0664**	235143.4	242704	265343	200486	**235919.1289**	**0.8642**	**0.0770**
Q91WK5	Gcsh	Glycine cleavage system H protein, mitochondrial	69239.41406	56892.16	62617.48	72182.72	**65232.94238**	66554.05	69230.9	85674.44	80498.28	**75489.41602**	**0.8641**	**0.1216**
Q8CH18	Ccar1	Cell division cycle and apoptosis regulator protein 1	10572.69629	10546.72	9194.081	11133.77	**10361.81714**	13119.33	11908.23	13524.3	9435.916	**11996.94678**	**0.8637**	**0.1560**
A2RSY6	Trmt1l	TRMT1-like protein	35368.04688				**35368.04688**	37875.96	39833.77		45193.36	**40967.69922**	**0.8633**	
Q9EPU5	Tnfrsf21	Tumor necrosis factor receptor superfamily member 21	2069.050049		3861.167	3009.071	**2979.762777**	2590.679	2885.718	5300.417	3035.719	**3453.133362**	**0.8629**	**0.6035**
P50114	S100b	Protein S100-B	508320.4375	625700.7	394874.8	341824.7	**467680.1484**	492359.6	461857	581159.3	632852.3	**542057.0313**	**0.8628**	**0.3561**
O70362	Gpld1	Phosphatidylinositol-glycan-specific phospholipase D	17415.32031	25872.41	20354.53	18658.58	**20575.21045**	26697.75	26998.73	19726.57	22003.23	**23856.57227**	**0.8625**	**0.2514**
P56380	Nudt2	Bis(5'-nucleosyl)-tetraphosphatase [asymmetrical]	59436.15234	62865.46	62411.7	54520.93	**59808.56055**	69112.02	66012.05	64075.12	78196.1	**69348.82129**	**0.8624**	**0.0406**
Q6P5G6	Ubxn7	UBX domain-containing protein 7			6293.96	10557.85	**8425.90625**	11067.25	10228.83	8019.006		**9771.694987**	**0.8623**	**0.5446**
O08579	Emd	Emerin	12484.0752	7786.738	12042	11043.94	**10839.1875**	12606.72	13064	11379.44	13238.07	**12572.05957**	**0.8622**	**0.1796**
Q3UX61	Naa11	N-alpha-acetyltransferase 11		4767.905			**4767.904785**		4247.791		6814.487	**5531.13916**	**0.8620**	
P23780	Glb1	Beta-galactosidase	15202.08105	16706.37	13876.27	17330.27	**15778.74756**	19978.18	18191.82	17996.47	17055.07	**18305.38525**	**0.8620**	**0.0429**
Q9JHR9	Nrip2	Nuclear receptor-interacting protein 2	31466.56445	27917.77	22773.88	19572.85	**25432.76514**	37825.5	28016.57	22515.64	29688.06	**29511.44141**	**0.8618**	**0.3611**
Q8BS40	Cptp	Ceramide-1-phosphate transfer protein	8030.188477	16759.36	9510.286	10897.93	**11299.44116**	10949.23	8898.899	18470.72	14143.91	**13115.68994**	**0.8615**	**0.5447**
Q5EBG8		Uncharacterized protein C1orf50 homolog				3357.846	**3357.846436**				3899.041	**3899.04126**	**0.8612**	
Q9D4V0	Etnk1	Ethanolamine kinase 1	3663.281006	6092.499			**4877.890015**	7726.917	5561.19	3804.426	5566.857	**5664.847717**	**0.8611**	**0.6078**
Q7TQG1	Plekha6	Pleckstrin homology domain-containing family A member 6	18131.17578	18259.67	21162.29	17373.68	**18731.70264**	25838.54	16028.2	24296.29	20907.45	**21767.62036**	**0.8605**	**0.2398**
Q9EQC5	Scyl1	N-terminal kinase-like protein	10514.41895	9807.945	7726.83	8826.288	**9218.870483**	11066.78	9389.444	12090.15	10311.28	**10714.41382**	**0.8604**	**0.1230**
Q99PW4	Tp53rkb	EKC/KEOPS complex subunit Tp53rkb	8639.574219	9214.891	8553.611	3464.209	**7468.07135**	10332.06	8706.901	5975.398	9705.604	**8679.991211**	**0.8604**	**0.4907**
Q9D6Y9	Gbe1	1,4-alpha-glucan-branching enzyme	18099.26953	18711.76	20811.57	20847.96	**19617.64063**	19413.11	22036.48	22230.74	27549.07	**22807.35059**	**0.8601**	**0.1352**
Q9CX30	Yif1b	Protein YIF1B	8233.892578	4936.639	1307.343	4088.368	**4641.560455**	4326.236	7380.472	7130.798	2750.414	**5396.980225**	**0.8600**	**0.6916**
P97772	Grm1	Metabotropic glutamate receptor 1	9703.051758	6112.363	6172.348	7064.627	**7263.097412**	10288.38	7866.108	7012.685	8615.649	**8445.706055**	**0.8600**	**0.3206**
Q9CQ86	Mien1	Migration and invasion enhancer 1	26101.32422	33980.23	23401.5	25209.89	**27173.23389**	35968.17	34948.76	29573.58	25954.56	**31611.26611**	**0.8596**	**0.2291**
Q3UFF7	Lyplal1	Lysophospholipase-like protein 1	13171.2168	13237.88	9483.707	11657.32	**11887.53027**	19157.49	11643.37	8808.356	15725.94	**13833.78857**	**0.8593**	**0.4550**
Q9D0W5	Ppil1	Peptidyl-prolyl cis-trans isomerase-like 1	11818.82031	9227.666	10587.08	13040.62	**11168.54761**	15764.97	10186.78	13873.76	12166.94	**12998.11279**	**0.8592**	**0.2523**
Q62415	Ppp1r13b	Apoptosis-stimulating of p53 protein 1	23882.42188	21119.64	19052.11	23025.13	**21769.82666**	23759.38	23839.72	24760.23	28996.97	**25339.07422**	**0.8591**	**0.0725**
Q99M71	Epdr1	Mammalian ependymin-related protein 1	107078.5156	103050.9	78463.9	89615.44	**94552.1875**	104560	100539.9	126976.6	108215.8	**110073.0898**	**0.8590**	**0.1271**
Q9Z0Z4	Heph	Hephaestin	10233.33398	12042.88	8277.342	9427.865	**9995.355957**	13411.21	13057.23	10390.54	9691.71	**11637.67139**	**0.8589**	**0.2288**
Q9D1B9	Mrpl28	Large ribosomal subunit protein bL28m	11552.15332	9595.013	15327.3	9325.693	**11450.04053**	10360.31	10802.99	18380.35	13783.79	**13331.85791**	**0.8588**	**0.4460**
Q9DAW6	Prpf4	U4/U6 small nuclear ribonucleoprotein Prp4	6211.483398		6142.327	5636.12	**5996.643392**	3715.156	10196.82	7105.12	6932.83	**6987.481201**	**0.8582**	**0.5565**
Q8C0D5	Efl1	Elongation factor-like GTPase 1	16913.07617	18593.61	16828.04	15264.49	**16899.8042**	17514.77	23274.31	18467.98	19512.98	**19692.51123**	**0.8582**	**0.0993**
Q9CT10	Ranbp3	Ran-binding protein 3	17194.04883	16626.93	13329.92	15403.24	**15638.53418**	19478.61	19917.34	17376.31	16136.06	**18227.07959**	**0.8580**	**0.0809**
Q8K0P3	Meak7	MTOR-associated protein MEAK7	7392.800781	8726.382		2876.33	**6331.837402**	7782.955	7836.596	6498.611	7431.262	**7387.355835**	**0.8571**	**0.5198**
Q8WTY4	Ciapin1	Anamorsin	11985.46191	15373.92	9677.975	11899.54	**12234.22534**	15234.22	14178.09	13171.28	14599.34	**14295.73145**	**0.8558**	**0.1508**
Q7TSH2	Phkb	Phosphorylase b kinase regulatory subunit beta	13826.27832	15500.08	12100.46	12109.87	**13384.17139**	19148.23	15251.39	13878.21	14280.64	**15639.61597**	**0.8558**	**0.1717**
Q8BWU8	Etnppl	Ethanolamine-phosphate phospho-lyase	10877.66992	12823.55	12805.49	12417.31	**12231.00415**	14118.45	11528.16	15164.39	16378.54	**14297.38623**	**0.8555**	**0.1173**
Q3UMU9	Hdgfl2	Hepatoma-derived growth factor-related protein 2	66007.72656	19754.62	54671.5	42181.59	**45653.85693**	71848.54	58465.91	18668.25	64493.85	**53369.1377**	**0.8554**	**0.6358**
Q6DVA0	Lemd2	LEM domain-containing protein 2	27130.66992	8887.18	34031.27	28959.66	**24752.19678**	25094.94	24641.39	31795.23	34210.59	**28935.53906**	**0.8554**	**0.5110**
Q8BUN9	Slc24a2	Sodium/potassium/calcium exchanger 2	38919.91406	39390.37	32706.1	55445.58	**41615.48975**	57087.7	37016.94	50373.81	50118.52	**48649.24121**	**0.8554**	**0.3152**
Q60960	Kpna1	Importin subunit alpha-5	16313.77246	15818.43	9566.945	14626.69	**14081.45776**	15431.16	15742.56	16782.72	17889.71	**16461.53906**	**0.8554**	**0.1977**
A2AAY5	Sh3pxd2b	SH3 and PX domain-containing protein 2B	18412.55859	13599.63	16863.83	16934.5	**16452.62964**	20058.13	21947.33	14734.32	20194.86	**19233.65918**	**0.8554**	**0.1859**
Q9JL15	Lgals8	Galectin-8	5397.507813	5294.179	8518.821	6388.55	**6399.764648**	7659.732	8660.014		6128.758	**7482.834635**	**0.8553**	**0.3613**
Q6P9S0	Mtss2	Protein MTSS 2	21659.06836	21643.51	16842.64	23606.72	**20937.98291**	22415.93	23318.67	28587.13	23670.26	**24497.99805**	**0.8547**	**0.1255**
Q8VDG5	Ppcs	Phosphopantothenate--cysteine ligase	4629.549805	13909.92	9206.891	13351.74	**10274.52417**	11253.67	17015.36	8418.861	11433.62	**12030.37573**	**0.8540**	**0.5546**
P12849	Prkar1b	cAMP-dependent protein kinase type I-beta regulatory subunit	30682.82422	35172.86	35671.69	29684.44	**32802.95459**	34771.5	33802.34	40848.16	44213.36	**38408.83984**	**0.8540**	**0.1031**
Q9QUR8	Sema7a	Semaphorin-7A	19020.50195	20863.51	20862.23	21204.01	**20487.5625**	23807.6	28071.04	24699.54	19377.51	**23988.92041**	**0.8540**	**0.1084**
Q99N94	Mrpl9	Large ribosomal subunit protein bL9m	13343.35645	12395.78	10772.49	17685.47	**13549.2749**	17441.43	13652.39	17716.31	14659.05	**15867.29639**	**0.8539**	**0.2429**
Q9Z0W1	Ngfr	Tumor necrosis factor receptor superfamily member 16		3900.396			**3900.396484**	5042.599	1422.108	6119.03	5690.13	**4568.466888**	**0.8538**	
O55074;Q7TN79	Akap7	A-kinase anchor protein 7 isoform alpha;A-kinase anchor protein 7 isoform	2707.442139		4111.701	5984.555	**4267.899333**	4009.861		6293.801	4694.014	**4999.225098**	**0.8537**	**0.5645**

A6H6A9	Rabgap1l	Rab GTPase-activating protein 1-like	17245.32227	9171.533	10848.78	10644.16	**11977.44824**	8736.237	31130.99	7521.028	8753.067	**14035.3302**	0.8534	0.7426
P18653	Rps6ka1	Ribosomal protein S6 kinase alpha-1	16085.06152	17495.44	22101.87	22644	**19581.59448**	18963.11	21378.42	29912.1	21532.39	**22946.50342**	0.8534	0.2905
Q99LG2	Tnpo2	Transportin-2	19081.33984	23935.65	17321.56	13882.88	**18555.35913**	18391.49	24059.83	22174.4	22360.78	**21746.62402**	0.8533	0.2338
Q9CQ71	Rpa3	Replication protein A 14 kDa subunit		19425.36			**19425.36328**	33474.81		17521.88	17343.44	**22780.04232**	0.8527	
P57722	Pcbp3	Poly(rC)-binding protein 3	197991.3438	239908.7	173814.8	202995.4	**203677.5742**	239489.8	223214.7	289212.6	203744.9	**238915.4844**	0.8525	0.1736
Q2MKA5	Chrna5	Neuronal acetylcholine receptor subunit alpha-5		38242.03	44094.23	35129.72	**39155.32422**	51301.41			40591.37	**45946.38867**	0.8522	0.2855
Q61176	Arg1	Arginase-1	6944.96582	8551.144	16857.87	5274.543	**9407.13147**	10763.22	11986.16	11947.13	9469.081	**11041.39575**	0.8520	0.5587
Q8BGR6	Arl15	ADP-ribosylation factor-like protein 15			2612.35	2751.102	**2681.725708**				3149.637	**3149.636719**	0.8514	
Q80XQ2	Tbc1d5	TBC1 domain family member 5	12245.42773	10898.28	9263.794	8720.003	**10281.87622**	8353.276	17163.91	10893.75	11901.35	**12078.0708**	0.8513	0.4078
Q9CWP6	Mospd2	Motile sperm domain-containing protein 2	9342.71875	7771.808	3830.754	5344.888	**6572.542297**	7194.805	6210.726	10744.11	6737.807	**7721.862793**	0.8512	0.5001
P52483;Q91W82	Ube2e3;Ube2e2	Ubiquitin-conjugating enzyme E2 E3;Ubiquitin-conjugating enzyme E2 E2	24408.58008	23522.57	15841.3	17619.22	**20347.91724**	25501.09	25280.75	24841.79	20006.46	**23907.521**	0.8511	0.2039
Q6PAL8	Dennd5a	DENN domain-containing protein 5A	14112.32227	2309.267	1.135079	15218.76	**7910.372332**	17737.59			851.2203	**9294.404083**	0.8511	0.8686
Q3TWL2	Pip4p1	Type 1 phosphatidylinositol 4,5-bisphosphate 4-phosphatase	6749.273926	6862.206	11038.77	6083.663	**7683.478271**	5197.553	7907.566	15387.83	7619.219	**9028.042603**	0.8511	0.6070
P98192	Gnpat	Dihydroxyacetone phosphate acyltransferase	7613.358398	5631.058	7797.22		**7013.878906**		11455.28	5028.044		**8241.662598**	0.8510	0.6640
Q78WH7	Camk2n2	Calcium/calmodulin-dependent protein kinase II inhibitor 2	9940.498047	9647.845	8932.025	8152.963	**9168.332886**	11318.06	11162.6	10823.81	9794.386	**10774.71533**	0.8509	0.0224
P61971	Nutf2	Nuclear transport factor 2	95697.29688	125420.2	89135.2	87092.02	**99336.17188**	124624.1	126271.8	92162.46	123977	**116758.832**	0.8508	0.2000
P61022	Chp1	Calcineurin B homologous protein 1	76658.96094	80026.47	74146.21	66024.55	**74214.04688**	93536.66	78512.91	95570.8	81576.63	**87299.25391**	0.8501	0.0454
Q64523;Q6GSS7	H2ac20;Hist2h2aa1	Histone H2A type 2-C;Histone H2A type 2-A	25077.03125	21713.3	16361.62	8795.212	**17986.78906**	20079.41	21939.41	20497.12	22142.14	**21164.51953**	0.8499	0.4099
P49070	Camlg	Guided entry of tail-anchored proteins factor CAMLG	10611.5918	14000.99	11581.2	8234.449	**11107.05713**	14399.02	16323.55	14564.36	7030.709	**13079.41199**	0.8492	0.4396
Q62226	Shh	Sonic hedgehog protein	9132.814453	15151.22	17418.15	13029.62	**13682.95288**	15696.82		15680.29	16969.21	**16115.4375**	0.8491	0.3021
Q7TNF0	Doc2a	Double C2-like domain-containing protein alpha	14461.38672	27796.61	22293.76	13568.73	**19530.12231**		22073.65	17255.3	29732.51	**23020.48893**	0.8484	0.5182
Q8BGS2	Bola2	BolA-like protein 2	51095.80078	41206.45	57609.46	39982.61	**47473.5791**	50097.27	52738.81	59476.9	61629.91	**55985.71973**	0.8480	0.1398
Q8BP92	Rcn2	Reticulocalbin-2	23960.51367	22321.2	25485.29	22014.35	**23445.3374**	26828.81	27215.62	27888.05	28693.74	**27656.5542**	0.8477	0.0034
Q8CI43	Myl6b	Myosin light chain 6B	33019.09375	25539.55	35033.29	16644.83	**27559.18994**	30726.64	34191.45	29805.11	35331.2	**32513.59668**	0.8476	0.3011
Q8R5C5	Actr1b	Beta-centractin	79133.67969	81765.55	75198.18	70711.43	**76702.21094**	106457.7	81263.9	102129.5	72286.48	**90534.38867**	0.8472	0.1567
Q9DBE0	Csad	Cysteine sulfinic acid decarboxylase	12237.79395	11962.03	9555.44	8903.848	**10664.77808**	12409.75	13022.97	11344	13581.45	**12589.54321**	0.8471	0.0939
Q9CYI4	Luc7l	Putative RNA-binding protein Luc7-like 1	13982.10156	11770.27	8434.439	7103.252	**10322.51526**	15215.58	12091.83	10354.15	11081.79	**12185.83838**	0.8471	0.3639
Q8K4P8	Hecw1	E3 ubiquitin-protein ligase HECW1	4746.760742	2128.865		4767.051	**3880.892171**	2949.667	4395.566	4426.124	6569.122	**4585.119751**	0.8464	0.5661
Q69ZZ6	Tmcc1	Transmembrane and coiled-coil domains protein 1	6552.996582	5488.362	5010.188	4312.424	**5340.992798**	8002.418	6806.858	5245.882	5191.448	**6311.651489**	0.8462	0.2835
Q8BGQ1	Vipas39	Spermatogenesis-defective protein 39 homolog	9088.93457	7884.966	10255.21	921.9419	**7037.764282**	8773.676	7797.225	8519.557	8199.437	**8322.473511**	0.8456	0.5642
Q9QZF2	Gpc1	Glypican-1	15743.89355	12581.6	6109.632	12060.6	**11623.93066**	17508.91	13395.72	13002.78	11088.09	**13748.87769**	0.8454	0.4140
Q91WG8	Gne	Bifunctional UDP-N-acetylglucosamine 2-epimerase/N-acetylmannosamin	7088.99707	5062.002	8936.965	5644.301	**6683.066284**	6268.823	7706.985	7778.779	9864.816	**7904.850952**	0.8454	0.3240
Q8BVF2	Pdcl3	Phosducin-like protein 3		11690.63		8944.262	**10317.44531**		12423.89		11998.57	**12211.22998**	0.8449	0.3061
Q19LI2	A1bg	Alpha-1B-glycoprotein	7721.566895	10842.63			**9282.096436**	11013.84	10968.94			**10991.38672**	0.8445	0.3877
Q05CL8	Larp7	La-related protein 7	5434.529297	5756.18		2978.15	**4722.953125**			4912.178	6283.922	**5598.050293**	0.8437	0.5320
Q5XG73	Acbd5	Acyl-CoA-binding domain-containing protein 5	11449.6748	11622.46	8724.09	7761.898	**9889.530273**	12714.84	11847.33	11526.69	10800.95	**11722.45264**	0.8436	0.1312
P16110	Lgals3	Galectin-3	8314.728516	3007.319	9147.337	4463.142	**6233.131714**	7390.602				**7390.601563**	0.8434	
Q64152	Btf3	Transcription factor BTF3	15720.91211	14590.33	11410.37	14270.21	**13997.95483**	13932.37	17790.37	16761.66	17905.95	**16597.58618**	0.8434	0.0929
Q62419	Sh3gl1	Endophilin-A2	9251.867188	11570.63	7744.6	16024.6	**11147.92419**	12114	10339.19	13772.47	16658.98	**13221.15991**	0.8432	0.3925
P06802	Enpp1	Ectonucleotide pyrophosphatase/phosphodiesterase family member 1	15719.32031	13776.67	13367.83	7770.025	**12658.46191**	14068.84	15658.57	16030.2	14338.84	**15024.11328**	0.8425	0.2310
Q9CQ06	Mrpl24	Large ribosomal subunit protein uL24m	15306.18262	14215.26	13637.54	10162.98	**13330.49268**	13175.19	16125.51	16415.27	17631.58	**15836.88843**	0.8417	0.1366
P63002	Tle5	TLE family member 5	12021.87207	10325.46	10894.96	8957.747	**10550.0083**	11973.31	11263.6		14398.48	**12545.13216**	0.8410	0.1280
P61211	Arl1	ADP-ribosylation factor-like protein 1	21607.41406	26047.97	29288	16081.67	**23256.26367**	41477.74	22959.78	24701.41	21482.17	**27655.2749**	0.8409	0.4515
Q6PGE7	Slc6a7	Sodium-dependent proline transporter	20532.01172	18045.52	12381.49	17116.56	**17018.89722**	18364.31	21068	21702.91	19827.92	**20240.78369**	0.8408	0.1336
Q7TNS2	Micos10	MICOS complex subunit Mic10	15389.59668	20998.23		9490.461	**15292.76335**	12740.64	26669.61		15174.12	**18194.78906**	0.8405	0.6214
Q5XPI3	Rnf123	E3 ubiquitin-protein ligase RNF123	8828.614258	8616.615	6167.186	6993.312	**7651.431763**	6755.734	15549.86	6154.286	7963.999	**9105.969238**	0.8403	0.5459
Q8R349	Cdc16	Cell division cycle protein 16 homolog			19750.3	12183.04	**15966.66699**	15569.92		22556.66	18917.56	**19014.71289**	0.8397	0.4852
P55937	Golga3	Golgin subfamily A member 3	3607.020752	4297.089	4388.501	4456.319	**4187.232361**	6588.409	5099.41	3756.335	4504.125	**4987.069702**	0.8396	0.2523
Q8BMS9;Q8CB96	Rassf2;Rassf4	Ras association domain-containing protein 2;Ras association domain-conta	9012.353516		6692.829	4201.906	**6635.695964**		7903.321			**7903.321289**	0.8396	
P11862	Gas2	Growth arrest-specific protein 2	11671.06836	8572.563		10135.13	**10126.25326**				12063.44	**12063.44043**	0.8394	
Q923S9	Rab30	Ras-related protein Rab-30	10415.62305	9978.16	34114.65	33911.8	**22105.05811**	7895.087	34232.95	27560.51	35686.01	**26343.6405**	0.8391	0.6677
Q8CGZ0	Cherp	Calcium homeostasis endoplasmic reticulum protein	3104.947998	4024.796	3301.734		**3477.159261**	6972.135	5692.414	1336.015	2578.073	**4144.65918**	0.8389	0.6889
Q8K2Y7	Mrpl47	Large ribosomal subunit protein uL29m	16635.1582	18433.7	15734.48	19571.41	**17593.68677**	21162.96	23994.74	18998.84	19776.74	**20983.31934**	0.8385	0.0517
Q8C080	Snx16	Sorting nexin-16	9478.255859	15324.74	13305.42	13984.77	**13023.29517**	15459.25	15230.05	18691.58	12758.47	**15534.84009**	0.8383	0.2007
Q03137	Epha4	Ephrin type-A receptor 4	2709.39502	2183.957	1990.425		**2294.592244**	2918.355	2695.316	3258.942	2077.793	**2737.601685**	0.8382	0.2550
Q8CF89	Tab1	TGF-beta-activated kinase 1 and MAP3K7-binding protein 1	5149.873535	6986.375	2586.174	5918.716	**5160.284729**	6174.199		6147.691		**6160.944824**	0.8376	0.5158
Q14CH7	Aars2	Alanine--tRNA ligase, mitochondrial	7741.688477	8602.256	6781.966		**7708.636882**	9923.883	9766.613	8769.126	8367.717	**9206.834717**	0.8373	0.0628
Q8K2I1	Fntb	Protein farnesyltransferase subunit beta	33024.67188	35166.67	26120.5	19432.08	**28435.98096**	36881.1	34309.66	33953.66	30732.03	**33969.11377**	0.8371	0.1940
Q60865	Caprin1	Caprin-1	49045.75391	56348.07	56980.52	44895.38	**51817.42969**	67880.39	71121.09	51262.51	57463.12	**61931.7793**	0.8367	0.1129
Q99PU8	Dhx30	ATP-dependent RNA helicase DHX30	14483.37207	16318.28	15335.31	12435.03	**14642.99805**	17256.56	17434.19	18051.51	17265.75	**17502.00098**	0.8366	0.0149
Q923B0	Ggact	Gamma-glutamylaminecyclotransferase	20327.33398	27188.95	19218.75	19374.35	**21527.3457**	24474.69	28862.67	25658.42	23947.18	**25735.73975**	0.8365	0.1042
P70388	Rad50	DNA repair protein RAD50			65637.75	69493.13	**67565.4375**	78715.79			82891.05	**80803.42188**	0.8362	0.0431
Q8K358	Pigu	Phosphatidylinositol glycan anchor biosynthesis class U protein		14844.54	11477.55	7609.692	**11310.59229**	15064.17		14059.27	11457.87	**13527.10254**	0.8361	0.3990
Q8BFQ4	Wdr82	WD repeat-containing protein 82	7144.104004	6475.912	3691.933	7959.991	**6317.984985**	9213.412	6786.601	8215.443	6014.094	**7557.387573**	0.8360	0.3306
O35239	Ptpn9	Tyrosine-protein phosphatase non-receptor type 9	17455.69922	23254.17	16504.99	17261.69	**18619.13623**	17151.65	25634.34	24436.27	21891.05	**22278.32813**	0.8358	0.1845

Q9CQ89	Cuta	Protein CutA	131334.2969	145027.7	79074.92	51537.72	101743.665	105531.7	121343.6	125583.8	134532.1	121747.8125	0.8357	0.4136
A2AR02	Ppig	Peptidyl-prolyl cis-trans isomerase G	9914.054688	12478.87	6574.281	6438.101	8851.327026	9176.756	12930.99	10161.82	10112.35	10595.47827	0.8354	0.3347
Q9CX00	Ist1	IST1 homolog	26942.16992	30826.76	21889.35	23080.31	25684.64746	25218.59	33348.82	32946.81	31485.92	30750.03369	0.8353	0.1170
Q0VGU4	Vgf	Neurosecretory protein VGF	16087.99707	13148.86	10455.39	11922.6	12903.71216	17694.85	19226.61	11523.03	13356.82	15450.32544	0.8352	0.2841
Q61214	Dyrk1a	Dual specificity tyrosine-phosphorylation-regulated kinase 1A	6183.605957	5009.646	4595.555	3976.332	4941.284424	4431.083		5592.342	7729.828	5917.751139	0.8350	0.3639
Q8K221	Arfip2	Arfaptin-2	9824.413086	9801.66	6506.146	8025.444	8539.415894	10389.03	10467.7	10084.78	9967.274	10227.19482	0.8350	0.0814
P34884	Mif	Macrophage migration inhibitory factor	778536.25	813315.3	650442.4	735672.1	744491.4844	861526.3	815463.3	889168.5	1000558	891678.9063	0.8349	0.0316
Q99J83	Atg5	Autophagy protein 5	8325.799805	8623.307	7526.97	9971.694	8611.942627	10653.56	10717.48	9034.501	10871.95	10319.37012	0.8345	0.0429
Q641K1	Agtpbp1	Cytosolic carboxypeptidase 1	4415.741211	2794.573	5831.418	5573.404	4653.784119	4948.334	5890.953	4986.017	6482.833	5577.03418	0.8345	0.2845
P21812	Mcpt4	Mast cell protease 4		200.5401			200.5401306	265.8575	214.9541			240.405838	0.8342	
Q9CSN1	Snw1	SNW domain-containing protein 1	6872.020996	10117.62	3674.487	11997.42	8165.385254	11635.99	9724.718	11398.63	6399.695	9789.75769	0.8341	0.4873
P01868;P01869	Ighg1	Ig gamma-1 chain C region secreted form;Ig gamma-1 chain C region, membrane-bound form	4311.554	4014.385	4777.478		4367.80542	4588.001	10080.38	3254.196	3029.773	5238.088623	0.8339	0.6758
Q99MR6	Srrt	Serrate RNA effector molecule homolog	23228.29492	21907.77	15030.45	19739.92	19976.60742	22605.04	36680.48	16659.57	19887.39	23958.12061	0.8338	0.4353
Q8BVL3	Snx17	Sorting nexin-17	7346.271973	9239.228	10397.59	8020.263	8750.838501	8963.004	12227.93	10081.05	10714.77	10496.69043	0.8337	0.1184
Q91YS8	Camk1	Calcium/calmodulin-dependent protein kinase type 1	7225.337891	7913.453	9120.358	6488.354	7686.875854	7763.315	11534.57	10325.67	7304.657	9232.053101	0.8326	0.2309
Q9Z1F9	Uba2	SUMO-activating enzyme subunit 2	8006.563965	12654.6	9657.463	12205.11	10630.93298	13003.31	13758.55	12446.84	11866.63	12768.83203	0.8326	0.1168
Q9JMA2	Qtrt1	Queuine tRNA-ribosyltransferase catalytic subunit 1	15308.0459	11200.97	9747.197		12085.40495		14517.09			14517.08984	0.8325	
Q3UHH1	Zswim8	Zinc finger SWIM domain-containing protein 8	2793.842041		1959.769	1989.901	2247.837565		2373.929	2724.748	3004.069	2700.915365	0.8323	0.2398
P55088	Aqp4	Aquaporin-4	113695.3125	144707.7	67941.94	109575	108979.9902	147542.1	164449.5	110375.7	101591.3	130989.6387	0.8320	0.3502
Q9CQP2	Trappc2	Trafficking protein particle complex subunit 2	7719.730469	9494.916	6631.696	4367.159	7053.375244	9795.061	9162.051	6050.134	8910.359	8479.401245	0.8318	0.3336
F8VPZ3	Usp32	Ubiquitin carboxyl-terminal hydrolase 32	12586.02539	10638.67	5764.344	4931.013	8480.013428	10890.11	11680.05	6973.154	11254.12	10199.35828	0.8314	0.4552
Q80YE4	Aatk	Serine/threonine-protein kinase LMTK1	4420.673828	3808.965	3387.688	4082.08	3924.851624	4845.186	4210.695	5230.42	4598.75	4721.262573	0.8313	0.0405
Q8VDR9	Dock6	Dedicator of cytokinesis protein 6	1288101.625	816835.3	840088.3	572341.6	879341.7031	1585441	961671.1		629644.8	1058918.896	0.8304	0.5672
E9Q137	Tex264	Testis-expressed protein 264 homolog	5620.991699	4221.058	6524.123	10125.15	6622.829468	4466.41	13894.43	5400.573	8161.47	7980.719727	0.8299	0.6020
A1L3P4	Slc9a6	Sodium/hydrogen exchanger 6	15210.70313	15931.54	13001.71	12978.91	14280.71289	16372.39	16468.51	17697.78	18379.46	17229.5332	0.8289	0.0171
P11031	Sub1	Activated RNA polymerase II transcriptional coactivator p15	18804.82617	21223.61	19688.73	12131.83	17962.24731	24303.64	22630.05	19815.12	19961.11	21677.48047	0.8286	0.1548
Q569Z6	Thrap3	Thyroid hormone receptor-associated protein 3	19153.45117	18543.87	11780.31	19559.6	17259.30664	24201.75	24277.97	14508.55	20359.39	20836.91528	0.8283	0.2699
Q8VC42	Rmc1	Regulator of MON1-CCZ1 complex	12689.0332	11971.02	10135.67	6930.801	10431.63	15242.75	11562.54	10900.61	12673.87	12594.94336	0.8282	0.2254
P97434	Mprip	Myosin phosphatase Rho-interacting protein	6735.117676	7991.778	5946.027	5447.34	6530.065918	6153.882	7107.723	8617.137	9659.637	7884.594727	0.8282	0.2065
P46414	Cdkn1b	Cyclin-dependent kinase inhibitor 1B	15366.08301	10673.57	13221.41	10757.26	12504.57788	13055.15	15941.99	8779.962	22653.39	15107.62329	0.8277	0.4364
Q9WVQ5	Apip	Methylthioribulose-1-phosphate dehydratase	22308.29883	22875.48	16461.46	18261.43	19976.66895	33320.98	20917.4	17285.76	25061.11	24146.31104	0.8273	0.3125
P84084	Arf5	ADP-ribosylation factor 5	24624.61328	28518.33	21819.75	15364.52	22581.80347	22989.5	31308.85	30681.88	24249.68	27307.47656	0.8269	0.2263
Q80VJ2	Sra1	Steroid receptor RNA activator 1	6026.434082	6251.79	6438.568	3041.192	5439.496033	5928.407	7096.933	6101.912	7186.979	6578.557495	0.8269	0.2374
Q6P1D5	Sez6l	Seizure 6-like protein	18054.10156	11080.75	10936.59	12759.19	13207.65942	17133.43	13987.61	17161.56	15611.98	15973.64502	0.8268	0.1815
Q8C6G8	Wdr26	WD repeat-containing protein 26	3687.235107	3837.177	1070.032	2607.023	2800.366821	2494.444	2210.287	3881.72	4967.626	3388.519226	0.8264	0.5396
Q9Z2D3	Gsdme	Gasdermin-E	7752.00293	12051.22	13712.13	12232.26	11436.90552	11869.81	13176.61	19299.86	11061.32	13851.90137	0.8257	0.3275
O88597	Becn1	Beclin-1	3442.823975			3334.226	3388.525146		5989.802	3384.184	2939.286	4104.424154	0.8256	0.6011
Q9DCT1	Akr1e2	1,5-anhydro-D-fructose reductase	21897.0332	18267.55	15932.3	9317.581	16353.61523	15924.57	20963.41	25278.92	17073.98	19810.22095	0.8255	0.3472
Q9DCF9	Ssr3	Translocon-associated protein subunit gamma	9425.743164	6274.351	6383.17		7361.088053	9530.17	8304.142			8917.155762	0.8255	0.3502
Q61586	Gpam	Glycerol-3-phosphate acyltransferase 1, mitochondrial	9210.039063	10713.12	9628.268	6060.021	8902.861084	11522.21	13396.96	9192.066	9029.05	10785.07324	0.8255	0.2397
Q91W18	Tdrd3	Tudor domain-containing protein 3		5766.504	3733.288	5355.519	4951.770182	6292.918	4272.989		7431.741	5999.215983	0.8254	0.3999
Q8BZF2	Nipal4	Magnesium transporter NIPA4	9320.391602	11334.54	8660.537	7677.005	9248.119385	9058.495	11994.68	12282.08	11492.75	11206.99976	0.8252	0.1159
Q8BFV2	Pcid2	PCI domain-containing protein 2	5349.530273	2533.369	3850.396	2515.774	3562.267456	6188.939	5459.124	3328.101	2294.58	4317.686096	0.8250	0.5285
Q9D1M4	Eef1e1	Eukaryotic translation elongation factor 1 epsilon-1	12373.46777	10898.23	9889.375	10543.25	10926.07935	12624.83	14390.05	10309.25	15650.65	13243.69214	0.8250	0.1183
Q9D273	Mmab	Corrinoid adenosyltransferase MMAB	16000.43652	15140.52	10376.11	16656.53	14543.39966	15480.48	16258.01	21695.24	17113.09	17636.70361	0.8246	0.1714
Q91VX2	Ubap2	Ubiquitin-associated protein 2	11761.55176	6668.108	6107.604	3949.856	7121.780151	9900.839	9790.622	8049.201	6857.966	8649.65686	0.8234	0.4307
Q9CR21	Ndufab1	Acyl carrier protein, mitochondrial	38221.35938	40923.13	40039.39	35540.18	38681.01563	49115.57	48307.56	44358.7	46146.54	46982.08887	0.8233	0.0021
Q3TC72	Fahd2	Fumarylacetoacetate hydrolase domain-containing protein 2A	31712.36914	30740.3	40131.36	33608.16	34048.04639	39564.46	39283.91	43335.89	43269.95	41363.55371	0.8231	0.0223
O35633	Slc32a1	Vesicular inhibitory amino acid transporter	46863.38281	62791.54	45444.59	54810.41	52477.48145	56235.33	73787.78	73277.28	51718.83	63754.80566	0.8231	0.1576
Q922R1	Phaf1	Phagosome assembly factor 1	7773.508301	7953.56	5291.473	6997.951	7004.122925	8193.741	8135.865	8643.443	9080.159	8513.302246	0.8227	0.0582
B1AZP2	Dlgap4	Disks large-associated protein 4	4474.166016	5235.912	3189.921	5339.105	4559.775818	4773.099	2465.839	10946.55	3984.236	5542.432007	0.8227	0.6286
Q8BMJ3	Eif1ax	Eukaryotic translation initiation factor 1A, X-chromosomal	11556.94824	13794.16	14491.64	9325.763	12292.12842	13558.11	11741.82	18386.5	16136.59	14955.75708	0.8219	0.2038
Q9DBR1	Xrn2	5'-3' exoribonuclease 2	2462.211182	1813.668	992.4687		1756.115946	2279.748	1556.954	2146.276	2566.006	2137.246063	0.8217	0.4216
P55014	Slc12a1	Solute carrier family 12 member 1	88608.76563	79681.2	98772.33	19538.1	71650.09961	95298.05	99318.12	74909.18	79349.34	87218.67383	0.8215	0.4386
Q61387	Cox7a2l	Cytochrome c oxidase subunit 7A-related protein, mitochondrial	18394.71875	19516.99	18889.43	10563.59	16841.18359	20488.08	19934.9	19252.6	22364.5	20510.02197	0.8211	0.1477
Q8R3G1	Ppp1r8	Nuclear inhibitor of protein phosphatase 1	9240.848633	8516.531	5328.93	9619.322	8176.407959	10149.59	10359.42	11484.72	7846.61	9960.085815	0.8209	0.2001
Q9R1K9	Cetn2	Centrin-2	5908.845703	6536.042	6182.874	2999.592	5406.83844	6524.525	8373.646	5195.428	6262.777	6589.093994	0.8206	0.3021
Q60604	Scin	Scinderin	15484.75195	17368.16		15342.59	16065.16797	21354.63			17810.23	19582.42773	0.8204	0.1105
Q80ZJ7	Snx32	Sorting nexin-32	6600.370605	6329.793	4971.278	3852.769	5438.552795	7394.638	6889.7	5892.599	6341.861	6629.699585	0.8203	0.1474
Q6SKR2	N6amt1	Methyltransferase N6AMT1	4617.130859	7034.502	7561.767	4809.86	6005.815186	5587.544	5695.131	11358.56	6659.428	7325.166748	0.8199	0.4303
Q6A0A2	Larp4b	La-related protein 4B	10835.34375	5625.589	4789.936	8640.552	7472.855225	8162.69	8396.467	10491.86	9408.887	9114.975098	0.8198	0.3130
Q91YN5	Uap1	UDP-N-acetylhexosamine pyrophosphorylase			9447.604	5035.011	7241.307373			10167.09	7520.365	8843.72998	0.8188	0.5969
Q9D1F4	Akt1s1	Proline-rich AKT1 substrate 1	25537.18555	24555.78	18017.63	14618.9	20682.3728	28104.77	25357.57	23235.03	24417.8	25278.79346	0.8182	0.1541
Q9EPU4	Cpsf1	Cleavage and polyadenylation specificity factor subunit 1	6802.023438	8345.173	10838.65	6009.194	7998.759399	9684.73	7202.13		12474.34	9787.068034	0.8173	0.3638
Q8VBT0	Tmx1	Thioredoxin-related transmembrane protein 1	13455.97363	15961.23	12197.89	11852.66	13366.93628	19518.47	15412.15	15793.15	14714.49	16359.56665	0.8171	0.0802

Q8BH64	Ehd2	EH domain-containing protein 2	22085.56055	13558.91	26882.03	12602.85	18782.33716	25260.68	31314.59	15344.63	20061.08	22995.24585	0.8168	0.4194
Q03963	Eif2ak2	Interferon-induced, double-stranded RNA-activated protein kinase		12772.62	2958.094	8040.007	7923.574626	14234.66	11463.92	6035.578	7098.458	9708.155884	0.8162	0.6095
Q80UK0	Sestd1	SEC14 domain and spectrin repeat-containing protein 1	6487.48291	5989.542	5364.44	7246.332	6271.949219	8087.187	10172.05	7853.585	4638.578	7687.850952	0.8158	0.2861
O35066	Kif3c	Kinesin-like protein KIF3C	12582.70996	12184.37	17449.13		14072.06803	26134.35	3955.777	2294.686	36643.43	17257.06049	0.8154	0.7650
P56565	S100a1	Protein S100-A1	35426.85547	35054.86	28108.81	28348.6	31734.78076	42513.85	38625.38	38636.02	35938.24	38928.37305	0.8152	0.0255
Q5RJG7	Crppa	D-ribitol-5-phosphate cytidylyltransferase	9141.00293	12369.58	11431.06		10980.54753	12272.37	15191.77	14865.4	11563.24	13473.19385	0.8150	0.1230
O70176	Adcyap1	Pituitary adenylate cyclase-activating polypeptide	3137.026123	4551.777	5181.533		4290.112061	3477.485	4027.464	6902.908	6657.802	5266.414612	0.8146	0.4385
Q8BIV3	Ranbp6	Ran-binding protein 6	6732.287598	14022.81	15748.77	9634.657	11534.63074	12153.88	15049.59	15764.41	13737.45	14176.33252	0.8137	0.2755
O35127	Grcc10	Protein C10	26658.88477	26959.21	20775.58	28679.94	25768.40381	30269.69	28467.52	36108.14	31949.06	31698.60303	0.8129	0.0466
Q8BM72	Hspa13	Heat shock 70 kDa protein 13	8763.567383	5319.683	7313.226	5210.404	6651.720093	3343		11408.13	9796.829	8182.653809	0.8129	0.5344
Q99MI1	Erc1	ELKS/Rab6-interacting/CAST family member 1	1962.890503	5967.789	7423.1	2319.224	4418.250824	6010.19		4077.803	6220.673	5436.222168	0.8127	0.5745
Q9D0L8	Rnmt	mRNA cap guanine-N7 methyltransferase	17660.15039	18572.01	17879.33	29365.01	20869.125	23561.2	31435.98	25273.43	22541.24	25702.96191	0.8119	0.2128
Q80UY2	Kcmf1	E3 ubiquitin-protein ligase KCMF1	5560.990234	5183.666	3706.648	5939.09	5097.598572	7819.667	6097.344	5340.503	5867.121	6281.158569	0.8116	0.1541
Q9WVD5	Slc25a15	Mitochondrial ornithine transporter 1	6147.469727	4659.813	8501.979	5665.548	6243.702271	4449.034	6069.036	11157.64	9162.989	7709.674805	0.8099	0.4254
Q04519	Smpd1	Sphingomyelin phosphodiesterase	12326.5293	13641.29	12440.38	9162.587	11892.69604	15699.18	11162.84	15709.85	16206.32	14694.5481	0.8093	0.1152
Q91WF7	Fig4	Polyphosphoinositide phosphatase		2845.65	4015.262	1007.82	2622.910909	2175.446	4314.348	3612.25	2874.756	3244.200134	0.8085	0.5267
Q9WTK3	Gpaa1	Glycosylphosphatidylinositol anchor attachment 1 protein	14355.91992	18921.24	15631.39	16464.65	16343.29907	23030.76	20257.03	18813.08	18771.58	20218.11084	0.8083	0.0315
Q9CQ91	Ndufa3	NADH dehydrogenase [ubiquinone] 1 alpha subcomplex subunit 3	33617.78516	23892.14	26506.29	16050.2	25016.60522	28801.11	31271.23	34595.26	29146.6	30953.55029	0.8082	0.1753
Q61036	Pak3	Serine/threonine-protein kinase PAK 3	17830.4707	18079.59	25825.18	10473.84	18052.26807	26805.05	25642.57	20787.62	16119.04	22338.56934	0.8081	0.3226
P61290	Psme3	Proteasome activator complex subunit 3	7362.704102	6211.693	7552.649	5845.442	6743.12207	8045.778	8908.875	8041.862	8381.293	8344.452026	0.8081	0.0141
Q62203	Sf3a2	Splicing factor 3A subunit 2	5397.038574	8593.114	3335.011	4942.381	5566.88623	10658.89	4615.124	5683.382	6609.704	6891.775513	0.8078	0.4702
Q9D1I5	Mcee	Methylmalonyl-CoA epimerase, mitochondrial	15690.14941	17790.89	7416.007	9511.756	12602.20178	18944.26	11212.56	17454.56	14903.87	15628.81104	0.8063	0.3504
Q9D4F2	Plpp6	Polyisoprenoid diphosphate/phosphate phosphohydrolase PLPP6	4803.246094	6182.694	3434.453	4936.054	4839.111877	6932.552	5763.186	5581.019	5741.148	6004.476196	0.8059	0.1198
Q9D898	Arpc5l	Actin-related protein 2/3 complex subunit 5-like protein	83245.40625	99606.92	74844.05	71155.22	82212.89844	118288.4	99115.63	99249.94	91789.15	102110.7695	0.8051	0.0576
Q9R1Z7	Pts	6-pyruvoyl tetrahydrobiopterin synthase	15430.94727	11897.39		15030.67	14119.6696	12397.4	13558.81	19821.91	24425.42	17550.88599	0.8045	0.3664
Q8CJ19	Mical3	[F-actin]-monooxygenase MICAL3	5297.935059	7322.915	8261.776	5426.94	6577.391724	11191.26	8229.979	8178.501	5111.178	8177.729614	0.8043	0.3086
Q9D938	Tmem160	Transmembrane protein 160	38374.17969	36953.7	19813.96	28386.12	30881.98828	31205.33	35856.49	46408.47	40168.49	38409.69482	0.8040	0.2112
Q6ZWQ7	Spcs3	Signal peptidase complex subunit 3	6282.59082	3457.65	6688.064	2112.863	4635.291809	6645.456	5759.098	5913.472	4750.211	5767.059326	0.8038	0.3718
P70261	Pald1	Paladin	6977.106445	7199.846	9629.003	5767.607	7393.390625	7593.236	10578.08	9732.489	8894.095	9199.475586	0.8037	0.1298
Q8BGF9	Slc25a44	Solute carrier family 25 member 44	11146.77734	21384.47		17118.77	16550.00651	21228.29	25313.56	13822.93	22054.83	20604.90186	0.8032	0.3343
Q80W00	Ppp1r10	Serine/threonine-protein phosphatase 1 regulatory subunit 10	18329.90625				18329.90625	22825.75				22825.74609	0.8030	
P56213	Gfer	FAD-linked sulfhydryl oxidase ALR	13468.58984	20316.59	24588.74	29002.5	21844.104	33400.75	12880.33	34840.87	27690.5	27203.11304	0.8030	0.4069
P08103	Hck	Tyrosine-protein kinase HCK	5064.633301	4332.951	14476.58		7958.056478	6456.369			13375.97	9916.168457	0.8025	0.7185
P09242	Alpl	Alkaline phosphatase, tissue-nonspecific isozyme	2688.818604	3225.3	13617.18	153.6923	4921.248272	10245.04	4018.231	5031.306	5236.889	6132.865662	0.8024	0.7250
Q3TJD7	Pdlim7	PDZ and LIM domain protein 7		4866.353			4866.352539		6066.18			6066.179688	0.8022	
Q99JX3	Gorasp2	Golgi reassembly-stacking protein 2	17313.91797	19257.47	17116.35	17763.92	17862.91406	22274.34	21033.09	23162.74	22609.39	22269.8916	0.8021	0.0006
P42703	Lifr	Leukemia inhibitory factor receptor			1183.256	2177.654	1680.454651		1629.217	3628.375	1029.119	2095.570313	0.8019	0.7263
Q8CGQ8	Slc24a4	Sodium/potassium/calcium exchanger 4	13887.12891	13260.44	9464.605	10886.94	11874.7771	17909.45	16993.19	10567.79	13793.57	14815.99585	0.8015	0.1844
Q8R3U1	Plaat3	Phospholipase A and acyltransferase 3	4977.785156	9529.946	7028.739	4044.801	6395.317993	7987.579	5386.453	13046.32	5509.429	7982.444824	0.8012	0.4912
Q9D1C3	Pyurf	Protein preY, mitochondrial	4354.000488	6577.795	2767.809	9357.525	5764.282471	4193.002	3757.2	15041.71	5834.829	7206.685512	0.7999	0.6489
Q6Y7W8	Gigyf2	GRB10-interacting GYF protein 2	3672.434326	4051.782	2088.823		3271.013265		3648.472	2487.6	6135.166	4090.412354	0.7997	0.5425
P54869	Hmgcs2	Hydroxymethylglutaryl-CoA synthase, mitochondrial	79264.04688	73547.29	36867.52	8651.534	49582.59741	76863.86	56911.73	57164.3	57459.8	62099.91992	0.7984	0.4961
Q50H33	Kctd8	BTB/POZ domain-containing protein KCTD8	3122.372559	3701.354	3232.034	3015.639	3267.849731	3614.406	3808.887	5049.718	3899.925	4093.233887	0.7984	0.0605
O08648	Map3k4	Mitogen-activated protein kinase kinase kinase 4	2828.529541	13079.49	11168.67	6386.735	8365.856018	10602.88	10439.04	14905.51	6000.454	10486.96851	0.7977	0.4989
Q6ZQ88	Kdm1a	Lysine-specific histone demethylase 1A	3443.107178	3001.314		3753.677	3399.36613	4358.739	6819.006	3610.253	2274.125	4265.530762	0.7969	0.4830
P22005	Penk	Proenkephalin-A	38232.44922	34504.84	36681.29	41990.98	37852.3877	39894.41	34593.39	62398	53131.98	47504.44727	0.7968	0.1885
Q8R1H0	Hopx	Homeodomain-only protein	12982.74219	13325.86	7554.263	9248.679	10777.88623	14306.95	13239.21	13027.87	13554.11	13532.03613	0.7965	0.1051
Q3TC33	Ccdc127	Coiled-coil domain-containing protein 127	12699.0791	13457.08	10720.13	7022.917	10974.80359	13771.07	12971.06	15457.41	12974.59	13793.5332	0.7956	0.1194
Q641K5	Nuak1	NUAK family SNF1-like kinase 1	37163.16016	33291.64	25058.47	19829.43	28835.67334	30057.42	44370.26		34398.73	36275.4694	0.7949	0.2587
Q9CYL5	Glipr2	Golgi-associated plant pathogenesis-related protein 1	10930.33789	13050.25	18192.86	9834.908	13002.08813	11624.62	14282.95	19498.79	20038.46	16361.20483	0.7947	0.2692
Q9EPC1	Parva	Alpha-parvin	6730.034668	9640.383	7069.633	62.76063	5875.702853	6888.193	8391.801	6573.07	7761.398	7403.615723	0.7936	0.4913
Q8BHS3	Rbm22	Pre-mRNA-splicing factor RBM22	4009.448975	3141.071		3251.779	3467.433024	4589.968	4030.988	5003.866	3854.187	4369.752197	0.7935	0.0666
Q8R238	Sdsl	Serine dehydratase-like	10950.68066	7926.435	8266.351	10171.6	9328.767212	9600.201	14624.29	10703.91	12106.75	11758.78833	0.7933	0.1126
Q8C6G1	Cfap410	Cilia- and flagella-associated protein 410	4238.537598			2757.61	3498.07373		4893.374	5056.225	3285.572	4411.723796	0.7929	0.3915
P24788	Cdk11b	Cyclin-dependent kinase 11B			5269.297	2064.076	3666.686768	2433.103		6841.299	4604.429	4626.277262	0.7926	0.6689
Q9WTK7	Stk11	Serine/threonine-protein kinase STK11	3977.023438	10214.89	3442.107	3392.14	5256.539673	8172.942	7895.06	5305.228	5165.662	6634.7229	0.7923	0.4834
Q9CQJ6	Denr	Density-regulated protein	16935.88281	32629.33	27740.68	23525.79	25207.91943	34732.46	36543.81	33584.51	22512.61	31843.34863	0.7916	0.1988
Q69ZS8	Kazn	Kazrin	19228.57617	18504.04	13794.09	14674.48	16550.29736	23504.38	20123.97	20565.97	19439.21	20908.38477	0.7916	0.0365
P58158	B3gat3	Galactosylgalactosylxylosylprotein 3-beta-glucuronosyltransferase 3	21080.74805	25051.59	19738.13	29194.09	23766.13867	33466.7	24964.1	34888.15	26814.09	30033.25977	0.7913	0.1012
Q91ZV0	Mia2	Melanoma inhibitory activity protein 2	4559.128906	9649.756	8523.307	3887.789	6654.995117	10009.62	4545.46	7646.342	11443.69	8411.279053	0.7912	0.4303
Q8BJL0	Smarcal1	SWI/SNF-related matrix-associated actin-dependent regulator of chromatin	7135.314453	7630.583			7382.948975			9339.634		9339.633789	0.7905	
Q8BHA3	Dtd2	D-aminoacyl-tRNA deacylase 2	19669.98438	24419.7	18566.95	894.4086	15887.76181	16636.47	23288.36	15269.9	25301.89	20124.15356	0.7895	0.4864
Q9JII5	Dazap1	DAZ-associated protein 1	20775.92578	16456.79	26493.97	18674.33	20600.25342	21158.91	25379.67	30838.25	27075.69	26113.12988	0.7889	0.1102
Q8CAB8	Castor2	Cytosolic arginine sensor for mTORC1 subunit 2	16463.62695	17896.29	7041.346	6099.766	11875.25647	16304.64	18044.41	11590.58	14284.26	15055.97095	0.7887	0.3831
Q2YDW2	Msto1	Protein misato homolog 1	14444.9082	10909.32	14739.19	14087.04	13545.11523	19852.35	18520.32	13141.9	17191.26	17176.4563	0.7886	0.0767

Q8CEC5	Nkiras1	NF-kappa-B inhibitor-interacting Ras-like protein 1	16490.35742	13585.68	8315.231	21823.62	15053.72266	15660.16	15077.5	26003.83	19638.03	19094.87866	0.7884	0.3261
Q91ZW3	Smarca5	SWI/SNF-related matrix-associated actin-dependent regulator of chromatir	1693.904175	1301.474		10837.88	4611.084961	3642.485	5861.54	8073.819		5859.281494	0.7870	0.7297
Q6GQT6	Scap	Sterol regulatory element-binding protein cleavage-activating protein		1338.275			1338.275024	1322.288	2079.247			1700.767456	0.7869	
Q8CHP5	Pym1	Partner of Y14 and mago	10497.89258	9095.578	10848.05	11019.8	10365.3291	12648.56	16916.28	10675.93	12491.92	13183.16919	0.7863	0.0895
Q9WUA6	Akt3	RAC-gamma serine/threonine-protein kinase	9084.754883	9737.8	8150.596	6847.933	8455.270752	13565.35	9085.472	10361.61	10012.36	10756.19702	0.7861	0.0943
Q91VC9	Ghitm	Growth hormone-inducible transmembrane protein		25011.68	37196.95	18896.22	27034.94987	51255.31		20291.11	31636.8	34394.40625	0.7860	0.5229
D3YXJ0	Dgkh	Diacylglycerol kinase eta	4056.890869	7953.354	9203.808	11068.04	8070.522766	11036.5	11762.87	11318.98	7035.523	10288.46912	0.7844	0.2742
Q9D1G3	Hhatl	Protein-cysteine N-palmitoyltransferase HHAT-like protein	8491.052734	16588.74	8627.593	11147.5	11213.72241	13188.42	18863.35	13424.57	11711.11	14296.86353	0.7843	0.2564
P97493	Txn2	Thioredoxin, mitochondrial	11208.80469	10765.43	10966.58	11516.1	11114.22925	15397.58	13625.45	15601.31	12088.65	14178.24634	0.7839	0.0108
O88895	Hdac3	Histone deacetylase 3	47569.53125	43035.66	44252.79	32793.67	41912.91406	60498.98	51938.93	55546.6	45892.83	53469.33594	0.7839	0.0402
Q8VC30	Tkfc	Triokinase/FMN cyclase	9242.591797	9279.238	9532.457	8789.447	9210.933594	12603.25	11633.48	11602.78	11179.42	11754.73193	0.7836	0.0003
Q68FE2	Atg9a	Autophagy-related protein 9A	6604.213379	8174.35	6735.002	9678.065	7797.907715	12553.77	10136.83	10025.04	7107.242	9955.719482	0.7833	0.1551
Q3TKT4	Smarca4	Transcription activator BRG1	4390.591797	5824.962	3237.521		4484.358561	5629.087	9178.515	3460.816	4672.892	5735.327332	0.7819	0.4657
Q8K0D0	Cdk17	Cyclin-dependent kinase 17	10464.03809	27147.96	26128.28	13798.33	19384.6521	29953.45	28391.03	21903.35	18987.52	24808.83887	0.7814	0.3183
O88998	Olfm1	Noelin	18447.9375	19235.65	16070.36	17404.67	17789.65332	22483.11	22963.43	23805.86	21937.93	22797.58252	0.7803	0.0007
Q920Q6	Msi2	RNA-binding protein Musashi homolog 2	13266.89746	14151.89	11396.75	9515.842	12082.84448	17524.28	15285.85	17634.14	11499.4	15485.9209	0.7802	0.1023
Q9R020	Zranb2	Zinc finger Ran-binding domain-containing protein 2	1255.226807	2509.992		2788.455	2184.557943	3333.545	1176.097		3893.042	2800.894613	0.7800	0.5531
Q61070	Ei24	Etoposide-induced protein 2.4	39258.87109	40759.59	30192.38	30744.48	35238.83057		45195.58			45195.58203	0.7797	
Q9CZP7	Cdc37l1	Hsp90 co-chaperone Cdc37-like 1	6614.507813	8450.168	8795.791	6049.329	7477.448853	9733.355	7847.668	8294.609	12487.42	9590.762695	0.7797	0.1405
Q8R001	Mapre2	Microtubule-associated protein RP/EB family member 2	83136.40625	91956.4	59591.7	47931.29	70653.94922	98731.27	82737.41	95149.74	86040.7	90664.78125	0.7793	0.1152
Q9CY16	Mrps28	Small ribosomal subunit protein bS1m	12367.75586	10184.08	7505.008	10794.7	10212.88599	14644.93	13523.96	14323.41	9945.866	13109.54175	0.7790	0.0983
Q571J5	Znf354c	Zinc finger protein 354C	28619.58789	9077.344	31936.68	30758.43	25098.00928	31199.14	28903.41	38870.84	29980.54	32238.4834	0.7785	0.2672
Q3TXX4	Slc17a7	Vesicular glutamate transporter 1				12705.33	12705.32617			16331.21		16331.21191	0.7780	
Q05D44	Eif5b	Eukaryotic translation initiation factor 5B	14668.28516	9587.972	9147.099	7915.941	10329.82422	14125.95	13512.94	12290.27	13218.92	13287.02026	0.7774	0.1027
Q9CYR0	Ssbp1	Single-stranded DNA-binding protein, mitochondrial	28985.63086	23405.29	23857.37	17015.52	23315.95215	28745.49	30370.46	30208.39	30732.36	30014.1748	0.7768	0.0361
B0F2B4	Nlgn4l	Neuroligin 4-like	16776.66016	14300.36	11094.5	8257.097	12607.15259	18422.48	16851.59	15060.2	14684.94	16254.80347	0.7756	0.1254
Q3U2P1	Sec24a	Protein transport protein Sec24A	12298.98242	11391.53	6912.102	9916.875	10129.87134	13142.46	19695.84	10239.77	9168.225	13061.57568	0.7755	0.3098
Q66X03	Nlrp9a	NACHT, LRR and PYD domains-containing protein 9A	16025.24902	11885.57	13343.14		13751.32161	17531.15		17851.69	17821.71	17734.85091	0.7754	0.0307
Q6NZB0	Dnajc8	DnaJ homolog subfamily C member 8	13603.73535	15287.32	27101.03	16584.55	18144.1604	15883.25	29645.38	22304.23	25791.95	23406.20166	0.7752	0.2591
Q9EPB5	Serhl	Serine hydrolase-like protein	2980.925293	2874.065	3023.167	1794.895	2668.263092				3442.205	3442.205078	0.7752	
Q9CY73	Mrpl44	Large ribosomal subunit protein mL44	6056.902832	4748.984	3954.626	4904.634	4916.286682	6814.622	5828.324	7462.704	5265.278	6342.732056	0.7751	0.0725
P54754	Ephb3	Ephrin type-B receptor 3	16749.98828	16666.26	12390.98	17756.23	15890.86523	29564.14	5823.341	27698.92	18940.77	20506.79419	0.7749	0.4370
Q9JMG1	Edf1	Endothelial differentiation-related factor 1	10428.14941	5329.089	6867.662	4657.603	6820.625732	10014.29	10813.45	10284.12	4108.092	8804.989746	0.7746	0.3670
Q8BLK9	Rps6kc1	Ribosomal protein S6 kinase delta-1	6963.435059	4984.712	1826.139		4591.428589	6148.585	7045.417	4815.643	5699.544	5927.297363	0.7746	0.3733
Q8R035	Mrpl58	Large ribosomal subunit protein mL62		3662.719	4101.807		3882.262939		4752.428	5274.07		5013.249268	0.7744	0.0801
Q8BP00	Iqcb1	IQ calmodulin-binding motif-containing protein 1	9439.825195		6457.068		7948.446533	10459.01	11985.67		8457.854	10300.84635	0.7716	0.2659
Q569Z5	Ddx46	Probable ATP-dependent RNA helicase DDX46	8730.417969	10981.43	8357.647	7600.973	8917.618286	10596.4	12013.54	10226.14	13430.71	11566.69604	0.7710	0.0424
P46938	Yap1	Transcriptional coactivator YAP1	5410.047852			3456.178	4433.112915			5756.105		5756.10498	0.7702	
P56379	Atp5mpl	ATP synthase subunit ATP5MPL, mitochondrial	220401.6875	209560.4	202530.8	169237.7	200432.668	265832.3	248587.7	249908.2	277233.9	260390.5156	0.7697	0.0036
Q8CGB6	Tns2	Tensin-2	1663.924194				1663.924194	1839.548	1731.803	2855.029	2238.885	2166.316254	0.7681	
Q99LP6	Grpel1	GrpE protein homolog 1, mitochondrial	27554.92578	21491.69	25887.63	24674.92	24902.28955	32019.04	30792.96	28141.07	38833.73	32446.70117	0.7675	0.0278
Q9CYK1	Wars2	Tryptophan--tRNA ligase, mitochondrial	5900.840332	6835.839	8226.903		6987.86084	8684.896	9390.118	9377.87	9018.838	9117.930664	0.7664	0.0163
Q99J27	Slc33a1	Acetyl-coenzyme A transporter 1			1942.777	1537.757	1740.267273			2272.949		2272.948975	0.7656	
Q80XU3	Nucks1	Nuclear ubiquitous casein and cyclin-dependent kinase substrate 1		2651.623		6839.524	4745.573364	5274.57		7178.271	6184.021	6212.287598	0.7639	0.4552
Q9DBG7	Srpra	Signal recognition particle receptor subunit alpha	6046.744629	9678.316	9977.465	7210.41	8228.233887	9792.97	16899.74	10543.39	5871.07	10776.79395	0.7635	0.3429
E9Q555	Rnf213	E3 ubiquitin-protein ligase RNF213		2730.284			2730.283691	3067.297	4088.471			3577.883911	0.7631	
Q3UV17	Krt76	Keratin, type II cytoskeletal 2 oral	71549.40625	156671.3	278698.4	122487.1	157351.5625	165362.7	177154.5	347720.5	135043.3	206320.2266	0.7627	0.4806
P82198	Tgfbi	Transforming growth factor-beta-induced protein ig-h3	8294.365234	140208.5	83574.51	2940.048	58754.35138	11239.98	197062.3	44038.16	56097.81	77109.5625	0.7620	0.7389
Q6PFD9	Nup98	Nuclear pore complex protein Nup98-Nup96	4616.028809	6222.326	6851.885	6427.93	6029.542358	9437.195	5766.82	7591.996	8871.719	7916.932495	0.7616	0.0940
Q8C407	Yipf4	Protein YIPF4	11788.95508	9707.634	10789.28	6278.94	9641.201904	15776.42	10980.72	9975.199	13963.71	12674.01343	0.7607	0.1421
Q8VHR5	Gatad2b	Transcriptional repressor p66-beta	8524.951172	7410.361	4873.407	5667.372	6619.022705	9477.065	6533.445	9539.664	9289.286	8709.865234	0.7599	0.1065
Q3TYS2	Cybc1	Cytochrome b-245 chaperone 1	13517.78125	11282.03	12512.52	17950.01	13815.58374	17666.31	24459.94	17258.54	13341.04	18181.45825	0.7599	0.1606
Q920Q8	Ivns1abp	Influenza virus NS1A-binding protein homolog	2559.194092		3446.547		3002.870605			4045.083	3864.246	3954.664795	0.7593	0.1703
Q8BMD6	Tmem260	Protein O-mannosyl-transferase TMEM260			1227.76		1227.759766	1479.245	2176.041		1200.134	1618.473226	0.7586	
P24529	Th	Tyrosine 3-monooxygenase	3302.351074	6536.149	4377.865	2213.453	4107.454773	9159.426	5305.184	3826.304	3382.539	5418.362915	0.7581	0.4452
Q91WK2	Eif3h	Eukaryotic translation initiation factor 3 subunit H	21707.60352	15451.03	19537.02	17552.43	18562.02148	21076.75	17815.25	30232.62	28858.11	24495.68213	0.7578	0.1213
Q9JHU2	Palmd	Palmdelphin	9751.573242	9018.965	14111.6	8933.609	10453.93604	18411.14	11637.76	13362.54	11774.83	13796.56763	0.7577	0.1473
O55003	Bnip3	BCL2/adenovirus E1B 19 kDa protein-interacting protein 3	4843.030762	3050.385		4306.251	4066.55542	3591.941		4494.291	8021.568	5369.266683	0.7574	0.4204
Q8VDC0	Lars2	Probable leucine--tRNA ligase, mitochondrial	13204.05664	17881.07	10907.89	7173.82	12291.70972	19100.24	19035.89	13994.94	12842.24	16243.32861	0.7567	0.2051
Q9DCI3	Stard3nl	STARD3 N-terminal-like protein	8610.744141	12278.49	19249.96	5973.74	11528.23328	15441.15	9077.869	18823.24	17596.98	15234.80908	0.7567	0.3435
P60755	Mdga2	MAM domain-containing glycosylphosphatidylinositol anchor protein 2	5698.880859	4769.443	5153.388	7039.443	5665.288574	7674.297	6105.428	7955.688	8260.772	7499.046265	0.7555	0.0377
Q32NY4	Cnnm3	Metal transporter CNNM3	5869.472168	4894.169	6340.206		5701.282227	4433.965		10693.26		7563.611084	0.7538	0.4938
Q8R5L3	Vps39	Vam6/Vps39-like protein	10491.58496	12099.66	14072.01	9442.009	11526.31641	14132.94	17842.68	14091.29	15164.73	15307.90967	0.7530	0.0302
Q8BG95	Ppp1r12b	Protein phosphatase 1 regulatory subunit 12B	100518.7266	41622.94	98235.67	80719.98	80274.33008	90435.95	97954.39	56676.63	182372.6	106859.8857	0.7512	0.4095
P84309	Adcy5	Adenylate cyclase type 5	10084.91211	15584.92	8528.465	7706.015	10476.07715	17266.65	16758.3	11815.94	9973.309	13953.54858	0.7508	0.2188

Q5DU57	Spata13	Spermatogenesis-associated protein 13	3855.502441	3116.652			3486.077393			4645.559		4645.559082	0.7504	
Q9D9E0	Slc22a17	Solute carrier family 22 member 17	16746.67773	26999.41	22047.93	18423.49	21054.37744	40744.19	26287.27	22557.34	22647.82	28059.15723	0.7504	0.2009
Q641P0	Actr3b	Actin-related protein 3B	26820.44336	22704.41	16171.95	10979.61	19169.10303	26883.43	26378.13	22468.44	26465.51	25548.87744	0.7503	0.1311
Q8K1A5	Tmem41b	Transmembrane protein 41B	6662.934082	7957.344			7310.138916	9769.629	9717.685			9743.656738	0.7502	0.0641
Q91ZS8	Adarb1	Double-stranded RNA-specific editase 1	7282.623047	4298.829	3368.987	4872.095	4955.633667	7906.14	5840.979	10005.69	2680.253	6608.266174	0.7499	0.3866
Q8VE19	Mios	GATOR complex protein MIOS	4539.811035	6447.106	5008.498		5331.805176	6194.886			8030.271	7112.578369	0.7496	0.1756
Q6DIC0	Smarca2	Probable global transcription activator SNF2L2	6012.310547	7714.139		2191.763	5306.070801	6323.687	10178.63	7153.749	4668.199	7081.065918	0.7493	0.4004
Q5SQY2	Bod1	Biorientation of chromosomes in cell division protein 1	6138.303711	3242.413	3156.799		4179.171956	6112.497	5429.851	5205.584	5591.952	5584.970947	0.7483	0.1595
P70279	Surf6	Surfeit locus protein 6		1739.974			1739.973755			1892.131	2761.523	2326.827026	0.7478	
Q9JIM1	Slc29a1	Equilibrative nucleoside transporter 1			12723.65		12723.6543	14832.22			19296.66	17064.43945	0.7456	
Q8BTR5	Dusp28	Dual specificity phosphatase 28			8857.994		8857.994141			11942.05	11828.98	11885.51807	0.7453	
Q8VHI3	Pofut2	GDP-fucose protein O-fucosyltransferase 2		4180.392	3167.557	2875.193	3407.713867	4145.48	5000.144			4572.812012	0.7452	0.1483
Q6W8Q3	Pcp4l1	Purkinje cell protein 4-like protein 1	19459.81445	23370.65	20668.17	24718.08	22054.17822	33144.85	27111.52	30662.52	27479.74	29599.65674	0.7451	0.0068
Q64362	Aktip	AKT-interacting protein	3331.909668	7780.466	8202.705		6438.360189	10922.8	9429.869	8522.735	5790.642	8666.512817	0.7429	0.2764
Q99LC8	Eif2b1	Translation initiation factor eIF-2B subunit alpha	6411.369629	3650.009	6591.245	4627.876	5320.124756	6628.371	8307.532	8954.801	4835.281	7181.496216	0.7408	0.1613
Q8BV13	Cops7b	COP9 signalosome complex subunit 7b	3532.967773	3503.704	2616.207	1779.19	2858.017212	547.0002	4997.862	5648.524	4270.891	3866.069382	0.7393	0.4387
Q9JKB3	Ybx3	Y-box-binding protein 3	3951.850342	5613.787	4186.504	4296.932	4512.268127			8208.665	4002.141	6105.403076	0.7391	0.3202
P57724	Pcbp4	Poly(rC)-binding protein 4	32180.07813	17709.79	14700.26	24872.06	22365.54932	15382.55	45171.62	40272.08	20273.42	30274.92041	0.7387	0.3774
Q9ER99	Rsc1a1	Regulatory solute carrier protein family 1 member 1	10091.12109	9009.362	11559.38	7688.155	9587.003418	13145.49	12987.58	11893.84	13917.41	12986.08032	0.7383	0.0102
Q8CFI2	Cdc34	Ubiquitin-conjugating enzyme E2 R1	10149.85449	7313.674	5100.641	5502.039	7016.552124	9908.867	7869.618	11175.62	9183.164	9534.31604	0.7359	0.1097
Q6A026	Pds5a	Sister chromatid cohesion protein PDS5 homolog A		3648.324			3648.32373			4962.136		4962.13623	0.7352	
Q924N4	Slc12a6	Solute carrier family 12 member 6	11403.83301	4490.735	9824.229	11581.11	9324.977783	15774.43	8275.587	14099.39	12637.87	12696.81958	0.7344	0.1946
P62500	Tsc22d1	TSC22 domain family protein 1	5339.913574	6555.749	6618.363	5747.665	6065.422607	7680.555	10772.65	7641.914	6941.399	8259.129639	0.7344	0.0526
Q3UHU5	Mtcl1	Microtubule cross-linking factor 1	2203.258545				2203.258545				3002.576	3002.575928	0.7338	
Q8K2X3	Stn1	CST complex subunit STN1		6229.761	8658.603		7444.181641	10244.91		10063.03		10153.97314	0.7331	0.1560
Q8JZW4	Cpne5	Copine-5	15806.29004	14271.08		10753.02	13610.13118	19906.1	16481.02	17147.9	20779.45	18578.61523	0.7326	0.0368
P70206	Plxna1	Plexin-A1	17839.74023	13784.74	10590.18	13357.23	13892.97437	18889.82	21364.65	16213.4	19437.17	18976.26001	0.7321	0.0323
Q99LD9	Eif2b2	Translation initiation factor eIF-2B subunit beta	6600.340332	12620.08	4586.017	9241.067	8261.875977	14100.12	13165.04	9151.217	8724.57	11285.23633	0.7321	0.2210
Q9DCE9	Igtp	Immunity-related GTPase family M protein 3	2342.38623	1203.285	2319.502	9300.855	3791.50705	6052.338		3428.664	6058.465	5179.822591	0.7320	0.5748
Q8BU03	Pwp2	Periodic tryptophan protein 2 homolog		46897.45	1640433	866807	851379.2344	956596.6	1126417		1410483	1164498.604	0.7311	0.5488
Q570Y9	Deptor	DEP domain-containing mTOR-interacting protein	6180.342285	6154.242	6863.457		6399.347005	3591.155	5761.293	13123.19	12557.62	8758.313721	0.7307	0.4451
Q8R3I3	Cog6	Conserved oligomeric Golgi complex subunit 6	3234.722168	4468.125	7220.807		4974.551432	7206.832	5889.885	3301.172	10858.92	6814.202637	0.7300	0.4225
Q9WTS2	Fut8	Alpha-(1,6)-fucosyltransferase			1323.87		1323.869751			1813.501		1813.501465	0.7300	
Q6ZPS2	Carns1	Carnosine synthase 1	14750.83008	16058.43	12556.71	23568.77	16733.6853	26759.41	24652.95	23053.72	17226.18	22923.06445	0.7300	0.0967
Q9DBN4	P33monox	Putative monooxygenase p33MONOX	7059.071289	7338.999	2623.092	4678.868	5425.007324	5634.317	7797.472	8926.664		7452.817871	0.7279	0.2452
O08677	Kng1	Kininogen-1	18960.28125	14147.35	16361.09	18074.83	16885.88892	24330.86	29041.38		16283.57	23218.60254	0.7273	0.1181
Q9Z321	Top3b	DNA topoisomerase 3-beta-1	5436.687012	5655.568	3011.909	4392.25	4624.103516	7480.481			5246.932	6363.706299	0.7266	0.2001
Q9EPC2	Fgf23	Fibroblast growth factor 23			2584.889	1911.974	2248.431519	3659.406	2673.367	2686.626	3364.553	3095.988037	0.7262	0.1167
Q9EQG7	Enpp5	Ectonucleotide pyrophosphatase/phosphodiesterase family member 5	10978.57422	10146.48	6761.86	4158.995	8011.477295	12653.65	12241.23	12812.25	6442.157	11037.31946	0.7259	0.2182
Q7TS72	Itpkc	Inositol-trisphosphate 3-kinase C				12642.09	12642.08691			17538.41	17384.05	17461.23438	0.7240	
Q91VM3	Wdr45	WD repeat domain phosphoinositide-interacting protein 4	12203.08203	16547.76	8595.713	11344.81	12172.84155		20038.16	15618.17	14787.13	16814.48568	0.7239	0.1086
A2A863	Itgb4	Integrin beta-4	317.1443176		5497.489		2907.316788	2145.028	7268.023	4443.552	2224.09	4020.173218	0.7232	0.6678
Q8CD19	Lancl3	LanC-like protein 3		9732.239	8500.524	4769.842	7667.535319		10686.81	7260.829	13879.01	10608.88477	0.7227	0.2918
Q6P8J7	Ckmt2	Creatine kinase S-type, mitochondrial	765941.875	777805	37918.41	697241.2	569726.6172	809028.5	790236.6	825957.7	738565.9	790947.1719	0.7203	0.2631
E9Q5F9	Setd2	Histone-lysine N-methyltransferase SETD2	6756.005859	9256.084	1801.576	7544.954	6339.654816	6947.238	7648.594	11810.15		8801.993652	0.7203	0.3294
Q9D711	Pir	Pirin	3809.481201	2883.523	7140.497	6406.29	5059.947754	6310.712	3774.808	10115.32	7994.648	7048.87207	0.7178	0.2821
Q9CZY3	Ube2v1	Ubiquitin-conjugating enzyme E2 variant 1	15607.15625	31169.04	26650.65	20631.91	23514.68848	39497.9	40900.87	44728.82	6059.308	32796.72546	0.7170	0.3712
Q9ER41	Tor1b	Torsin-1B	18830.09961			26558.91	22694.50586	33783.11	26836.28	39069.42	27183.54	31718.08691	0.7155	0.1446
Q8VCM5	Mul1	Mitochondrial ubiquitin ligase activator of NFKB 1	9578.09668	13387.56	10109.43		11025.02897	17163.39		14308.49	14871.87	15447.91309	0.7137	0.0401
Q9CWQ0	Dph5	Diphthine methyl ester synthase	1298.916504	2250.071	1600.498		1716.495158	1830.743	2979.561			2405.152222	0.7137	0.3071
Q69ZK0	Prex1	Phosphatidylinositol 3,4,5-trisphosphate-dependent Rac exchanger 1 prote	10444.59668	6642.998	7269.518	11407.11	8941.05603	10457.63	16150.07	11678.88	11940.06	12556.65967	0.7121	0.0782
Q9DAU1	Cnpy3	Protein canopy homolog 3	9082.007813	4328.452			6705.22998		7272.554	13289.43	7747.942	9436.641113	0.7106	0.4376
Q9Z1K6	Arih2	E3 ubiquitin-protein ligase ARIH2	7011.482422	8433.012	7373.862	9097.81	7979.041504	7495.142	6968.769	17817.14	12649.26	11232.57751	0.7103	0.2551
Q3TIR1	Trappc13	Trafficking protein particle complex subunit 13	2327.362305	2244.401	4205.394	2643.421	2855.144592	5600.416	3753.094	3328.827	3429.589	4027.981323	0.7088	0.1458
P11930	Nudt19	Acyl-coenzyme A diphosphatase NUDT19	6063.960449	7621.295	6906.195	6890.485	6870.483887	10803.18	5759.661	12518.56	9809.328	9722.68103	0.7066	0.1003
Q80Y14	Glrx5	Glutaredoxin-related protein 5, mitochondrial	39649.33984	38124.17	53648.81	44535.04	43989.3418	68993.12	73432.06	44434.95	62414.54	62318.66895	0.7059	0.0453
Q8CCT4	Tceal5	Transcription elongation factor A protein-like 5	30954.7832	10916.38	36534.11	22814.08	25304.83643	43683.87	18277.01	39490.36	42305.22	35939.11279	0.7041	0.2396
Q9JM13	Rabgef1	Rab5 GDP/GTP exchange factor	3249.523682	8468.268	4508.28	5756.476	5495.636902	8203.216	12624.62	5746.271	4717.226	7822.833618	0.7025	0.3067
Q80WT5	Aftph	Aftiphilin		2229.669			2229.669189	3182.998				3182.997559	0.7005	
Q9WUU7	Ctsz	Cathepsin Z	3683.303223		6264.162		4973.732666	8513.691		2514.621	10302.74	7110.351237	0.6995	0.5508
P0C192	Lrrc4b	Leucine-rich repeat-containing protein 4B	8347.393555	12945.73	5427.908	5761.648	8120.669678	13342.58	12075.67	10744.86	10441.28	11651.09668	0.6970	0.1063
Q9JKZ2	Slc5a3	Sodium/myo-inositol cotransporter	2514.131592	3959.839	1839.705		2771.22526		3813.231		4160.573	3986.9021	0.6951	0.2338
A2A6T1	Cdr2l	Cerebellar degeneration-related protein 2-like	7627.084961	18020.52	15570.51	7894.109	12278.05737	17312.06	7558.542	28379.32		17749.97298	0.6917	0.3987
O70370	Ctss	Cathepsin S	6598.984375	5908.35	7202.511	4179.682	5972.381836	15794.8	8030.886	4270.761	6545.375	8660.4552	0.6896	0.3384
P01837	Igkc	Immunoglobulin kappa constant	8792.960938	10313.47	699.2991	4074.458	5970.047928	5594.027	8018.314	9948.962	11092.73	8663.507324	0.6891	0.3245

P61327	Magoh	Protein mago nashi homolog	11454.08398	10998.12	8349.197	4726.784	**8882.047363**	14065.06	13209.52	11503.47	12834.69	**12903.18433**	**0.6884**	**0.0491**
Q9JIH2	Nup50	Nuclear pore complex protein Nup50	3496.708008	2185.535		3834.107	**3172.116618**	3468.596	4971.548	3826.458	6215.278	**4620.470215**	**0.6865**	**0.1477**
Q63ZW7	Patj	InaD-like protein	13936.6582	23935.68	8642.04	9846.824	**14090.30151**	12522.81	25415.92	17683.13	26558.71	**20545.14307**	**0.6858**	**0.2278**
Q9DBY1	Syvn1	E3 ubiquitin-protein ligase synoviolin				3258.33	**3258.32959**			6873.203	2654.367	**4763.785278**	**0.6840**	
Q80Y56	Rbsn	Rabenosyn-5	7172.797363	7591.167	10803.25	10468.76	**9008.99353**	10877.44	18180.48	10365.39	13263.75	**13171.76343**	**0.6840**	**0.0850**
Q9CQ49	Ncbp2	Nuclear cap-binding protein subunit 2	5222.506348	4981.421		5746.553	**5316.826986**	6582.371	6728.488	10068.18		**7793.012207**	**0.6823**	**0.0998**
Q91WE1	Snx15	Sorting nexin-15	7702.212402	8003.887	3576.235	3127.602	**5602.484009**	5411.94	6351.285	12495.33	8596.778	**8213.833618**	**0.6821**	**0.2489**
Q921L5	Cog2	Conserved oligomeric Golgi complex subunit 2	2877.77417	3185.764	4074.861	3007.185	**3286.396118**	4052.776	3801.153	7210.838	4247.008	**4827.943909**	**0.6807**	**0.1176**
Q91VJ5	Pqbp1	Polyglutamine-binding protein 1	1019.230225	1994.051		3272.787	**2095.356038**	2455.681	3192.619	4394.966	2295.614	**3084.719666**	**0.6793**	**0.2639**
Q8BHD7	Ptbp3	Polypyrimidine tract-binding protein 3	5733.731445	1256.38	2027.292	2727.026	**2936.107208**	1040.125	1855.815	11583.02	2863.87	**4335.70755**	**0.6772**	**0.6142**
Q9Z103	Adnp	Activity-dependent neuroprotector homeobox protein				1913.135	**1913.13501**			2575.255	3083.048	**2829.151733**	**0.6762**	
Q8BG26	Rusc1	AP-4 complex accessory subunit RUSC1			4310.764	3019.344	**3665.054077**	4559.772		5564.97	6144.106	**5422.949544**	**0.6758**	**0.1058**
Q8VE99	Ccdc115	Coiled-coil domain-containing protein 115	1370.89563	2037.934	2611.579		**2006.802612**		2450.617	3097.443	3364.525	**2970.861816**	**0.6755**	**0.0986**
Q91VH6	Memo1	Protein MEMO1	6806.891602	10647.82	7875.117	8905.436	**8558.815186**	7681.346	10368.76	19785.33	13090.69	**12731.53137**	**0.6723**	**0.1763**
Q80WR1	Tspan18	Tetraspanin-18			7065.785	3382.718	**5224.251465**			7789.607		**7789.606934**	**0.6707**	
Q8K1N1	Pnpla8	Calcium-independent phospholipase A2-gamma	9721.161133	11111.99	3882.339	14933.54	**9912.25708**	16339.49	14084.74	9968.138	18747.38	**14784.9353**	**0.6704**	**0.1504**
Q6P5D8	Smchd1	Structural maintece of chromosomes flexible hinge domain-containing protein 1		44292.25	13494.38	16191.83	**24659.48633**	16776.44	72782.77	37411.25	20424.3	**36848.69189**	**0.6692**	**0.5110**
Q8VDP2	Steep1	STING ER exit protein	2365.269531	1700.104			**2032.686768**			3190.933	2892.277	**3041.605103**	**0.6683**	**0.1095**
O55203	Ldb2	LIM domain-binding protein 2	8062.945801	9955.635		4195.157	**7404.579264**		9168.918	12999.38		**11084.14746**	**0.6680**	**0.2538**
Q99N16;Q9EP75	Cyp4f3;Cyp4f14	Cytochrome P450 4F3;Leukotriene-B4 omega-hydroxylase 3	1510.664673				**1510.664673**		1772.682		2763.383	**2268.032715**	**0.6661**	
Q9JHG6	Rcan1	Calcipressin-1	7694.477051	10541.29	9290.272	6974.174	**8625.054565**	15614.06	10679.08	16336.9	9342.739	**12993.1958**	**0.6638**	**0.0637**
Q8CGF5	Tlcd4	TLC domain-containing protein 4	307.7319336	5312.011	5914.641	6515.257	**4512.410156**	4958.47	6744.152	11349.84	4380.516	**6858.245728**	**0.6580**	**0.3121**
Q9JLH8	Tmod4	Tropomodulin-4			4902.542		**4902.541992**	7452.376				**7452.376465**	**0.6578**	
Q9JHW9	Aldh1a3	Retinaldehyde dehydrogenase 3	12848.77539				**12848.77539**		12973.31		26186.7	**19580.00342**	**0.6562**	
Q923X4	Glrx2	Glutaredoxin-2, mitochondrial		5623.454	8787.456	7806.457	**7405.788737**	9278.307	9591.93		15090.37	**11320.20215**	**0.6542**	**0.1366**
O08750	Nfil3	Nuclear factor interleukin-3-regulated protein	2040.155029	3740.313			**2890.234009**	4878.732		3643.989	4777.293	**4433.337891**	**0.6519**	**0.1543**
Q9D281	Fam114a1	Protein Noxp20	3892.283203				**3892.283203**	6053.068		4189.231	7712.426	**5984.908529**	**0.6503**	
P25785	Timp2	Metalloproteinase inhibitor 2		1952.884	27.54158	2376.723	**1452.383**	2346.281	1706.341	3034.707	1846.522	**2233.462585**	**0.6503**	**0.3161**
Q8R1U1	Cog4	Conserved oligomeric Golgi complex subunit 4		1800.318			**1800.317993**		1671.739	2747.129	3927.201	**2782.023193**	**0.6471**	
Q67BT3	Slc13a5	Na(+)/citrate cotransporter	7343.482422	3332.972	2458.483	3805.772	**4235.177124**	6686.646	5555.267	6459.035	7518.405	**6554.838379**	**0.6461**	**0.0895**
Q80XH1	Kxd1	KxDL motif-containing protein 1	5095.930664	5272.283	2982.471	2847.61	**4049.57373**	5846.726	8422.289	5068.915	5863.402	**6300.332886**	**0.6428**	**0.0619**
Q3V009	Tmed1	Transmembrane emp24 domain-containing protein 1	994.6583252	1477.512			**1236.084961**		1925			**1924.999634**	**0.6421**	
Q80UP3	Dgkz	Diacylglycerol kinase zeta	9509.65625	10065.69	10990.11	11043.13	**10402.14502**	16786.83	13729.39	27167.21	7373.375	**16264.20142**	**0.6396**	**0.2071**
Q8BZ60	Ston2	Stonin-2	3824.251709		4081.172	3397.224	**3767.549316**	6649.204	8850.07	4070.38	4011.264	**5895.229553**	**0.6391**	**0.1848**
Q9JLF6	Tgm1	Protein-glutamine gamma-glutamyltransferase K			5204.702		**5204.702148**			9285.633	7036.124	**8160.878418**	**0.6378**	
P97346	Nxn	Nucleoredoxin	81600.9375	76949.73	50160.89	7486.616	**54049.54358**	93741.72	94824.84	85577.35	65408.37	**84888.07031**	**0.6367**	**0.1431**
P50096	Impdh1	Inosine-5'-monophosphate dehydrogenase 1	5360.736328				**5360.736328**			8431.46		**8431.459961**	**0.6358**	
O09117	Sypl1	Synaptophysin-like protein 1	10196.08203	9257.282	19457.9	116.4351	**9756.924938**	10799.4	128.9114	25855.67	24686.29	**15367.56866**	**0.6349**	**0.4706**
P13541	Myh3	Myosin-3				9107.648	**9107.648438**	9256.357	24681.88		9125.876	**14354.70475**	**0.6345**	
Q8VDB2	Alg12	Dol-P-Man:Man(7)GlcNAc(2)-PP-Dol alpha-1,6-mannosyltransferase			2591.218		**2591.217529**				4088.492	**4088.492188**	**0.6338**	
Q9WUC3	Ly6h	Lymphocyte antigen 6H	15243.11426		10784.11	14200.49	**13409.23893**			22985.61	19344.71	**21165.16309**	**0.6336**	**0.0390**
Q8C0T5	Sipa1l1	Signal-induced proliferation-associated 1-like protein 1	6565.145996	7520.389	4049.936	3754.366	**5472.459106**	7653.871	11503.07		6877.728	**8678.223307**	**0.6306**	**0.1056**
P50285	Fmo1	Flavin-containing monooxygenase 1			12784.33		**12784.32715**			20333.11		**20333.10742**	**0.6287**	
O55098	Stk10	Serine/threonine-protein kinase 10	8848.491211	6958.478	3335.994	5089.26	**6058.055786**	15455.51	7244.603	10115.95	5725.931	**9635.498413**	**0.6287**	**0.1946**
P62322	Lsm5	U6 snRNA-associated Sm-like protein LSm5	10183.90918	20044.24	42308	13744.48	**21570.15698**	44003.31	15920.33	42573.53	34762.82	**34314.99829**	**0.6286**	**0.2359**
Q8K2Y9	Ccm2	Cerebral cavernous malformations protein 2 homolog	5010.365723	4530.074		4788.058	**4776.165853**		4551.608	13931.46	4350.232	**7611.099609**	**0.6275**	**0.4209**
P09803	Cdh1	Cadherin-1	94507.61719		9088.503	144801	**82799.05046**		146837.8	117736.8		**132287.2891**	**0.6259**	**0.4136**
Q925F2	Esam	Endothelial cell-selective adhesion molecule		3886.729	2511.991	2950.599	**3116.439779**			6161.526	3806.429	**4983.977295**	**0.6253**	**0.1651**
P51807	Dynlt1	Dynein light chain Tctex-type 1	4585.67627	13977.98	11565.33	4980.675	**8777.415649**	17396.47	15042.83	5479.992	18313.11	**14058.09888**	**0.6244**	**0.2109**
P07310	Ckm	Creatine kinase M-type	227169.7344	146615.3	64694.41	57040.3	**123879.9229**	91672.08	282726.8	213682.8	207323.8	**198851.3438**	**0.6230**	**0.2310**
Q8C561	Lmbrd2	G-protein coupled receptor-associated protein LMBRD2	967.480957			1015.085	**991.2831726**			2765.755	419.27	**1592.512299**	**0.6225**	**0.6594**
Q9CY18	Snx7	Sorting nexin-7	1777.734985			5.386849	**891.5609171**	1967.604	1708.361	1107.269	947.9933	**1432.806595**	**0.6222**	**0.4537**
Q8VD33	Sgtb	Small glutamine-rich tetratricopeptide repeat-containing protein beta	5598.046387	17940.19	5808.575	8042.351	**9347.290527**	8605.715	16662.36	18243.04	16877.62	**15097.18359**	**0.6191**	**0.1661**
Q80U93	Nup214	Nuclear pore complex protein Nup214		8024.014	6296.669	1371.801	**5230.828125**	8139.898	7215.047	10055.76	8392.424	**8450.78186**	**0.6190**	**0.1351**
P0C673	Igsf11	Immunoglobulin superfamily member 11	12222.93262	13575.37	11600.35	10627.59	**12006.55981**	23195.67	18225.69	14808.3	21383.63	**19403.31982**	**0.6188**	**0.0089**
A2A8Z1	Osbpl9	Oxysterol-binding protein-related protein 9	27657.91797	182211.5	20140.77	195319.9	**106332.5356**	186793.1	177131.2	147451.1	177935.4	**172327.7109**	**0.6170**	**0.2221**
Q8CBY0	Gatc	Glutamyl-tRNA(Gln) amidotransferase subunit C, mitochondrial		3498.906		2050.303	**2774.60437**			4497.11		**4497.110352**	**0.6170**	
Q8VEG4	Exd2	Exonuclease 3'-5' domain-containing protein 2	2906.522949	3790.393			**3348.457764**	10280.88	3023.287	4147.107	4370.248	**5455.381165**	**0.6138**	**0.4413**
Q9QXY9	Pex3	Peroxisomal biogenesis factor 3	1248.092407	667.1666			**957.6295166**			2589.681	538.1111	**1563.895996**	**0.6123**	**0.6269**
Q9D826	Pipox	Peroxisomal sarcosine oxidase	5524.07373	5600.479	2927.042		**4683.864909**	7046.848	9502.512	6276.879	7784.242	**7652.620117**	**0.6121**	**0.0426**
Q8BZB2	Ppcdc	Phosphopantothenoylcysteine decarboxylase		5537.42	5069.643		**5303.531738**	8052.347		8299.273	9728.238	**8693.286133**	**0.6101**	**0.0166**
Q9CZE3	Rab32	Ras-related protein Rab-32			6593.766		**6593.765625**		10860.79			**10860.78613**	**0.6071**	
Q02788	Col6a2	Collagen alpha-2(VI) chain	2307.432861		114754.9	10128.94	**42397.09709**	132432.7	122179.6	6906.325	18095.47	**69903.52173**	**0.6065**	**0.6039**
Q8CGK7	Gnal	Guanine nucleotide-binding protein G(olf) subunit alpha	8215.520508	7004.552	39.54123	2383.651	**4410.816155**	10842.1	6452.513	7852.968	4404.673	**7388.062622**	**0.5970**	**0.2524**
Q9Z0W3	Nup160	Nuclear pore complex protein Nup160	4065.487793	2053.399		5301.67	**3806.852295**	9348.696	3445.945			**6397.320557**	**0.5951**	**0.3790**

Q61554	Fbn1	Fibrillin-1		17516.6	18593.65	35950.4	**24020.21615**	105442.7	39735.07	12974.48	3712.639	**40466.22424**	0.5936	0.5776
Q8BGK9		Uncharacterized protein C3orf18 homolog	9041.705078			3865.133	**6453.419189**	8712.46	14110.7	9886.705		**10903.28939**	0.5919	0.2180
Q9DC23	Dnajc10	DnaJ homolog subfamily C member 10	17048.64648	16029.64	11272.53	10169.86	**13630.17065**	51077.31	8792.256	10307.29		**23392.28516**	0.5827	0.4444
Q69ZH9	Arhgap23	Rho GTPase-activating protein 23	15747.64063	11209.94	9649.893	1414.188	**9505.416138**	13786.13	11490.7	23428.74	16549.86	**16313.85742**	0.5827	0.1359
Q6WQJ1	Dagla	Diacylglycerol lipase-alpha		2121.5			**2121.499512**	3233.99	4036.576	5736.799	1587.794	**3648.789673**	0.5814	
P55099	Tac3	Tachykinin-3	13162.67969	12027.18	5567.383	14727.95	**11371.29675**	20985.14	18487.38	20575.45	18282.51	**19582.61914**	0.5807	0.0084
Q811S7	Ubp1	Upstream-binding protein 1	3821.661377	12642.23	14932.6	20982.23	**13094.67999**	25656.82	23338.32	22004.99	19311.39	**22577.87939**	0.5800	0.0466
Q9DBC3	Cmtr1	Cap-specific mRNA (nucleoside-2'-O-)-methyltransferase 1	6658.806152	4519.437	1498.85	4039.678	**4179.192657**	10320.66	6067.082	5786.388	6686.143	**7215.067383**	0.5792	0.0883
Q99N50	Sytl2	Synaptotagmin-like protein 2		4437.385	3191.565	2317.677	**3315.542155**			5736.529		**5736.529297**	0.5780	
Q91W34	Rusf1	RUS family member 1	4866.594727	2637.948	3750.076	1441.215	**3173.958405**		3882.043	7131.288	5623.379	**5545.570231**	0.5723	0.0989
Q3UBX0	Tmem109	Transmembrane protein 109	6940.543945	640.1245	17071.63		**8217.433757**	16966.11		11762.21		**14364.1582**	0.5721	0.4126
O88398	Avil	Advillin	21926.57617	21952.67	32179.13	29391.59	**26362.48975**	42006.95	46372.79	50653.68	45621.21	**46163.65625**	0.5711	0.0008
Q8VDS4	Rprd1a	Regulation of nuclear pre-mRNA domain-containing protein 1A	5881.43457	2048.805	2200.65		**3376.963216**	2915.025	7262.653	7879.635	5603.305	**5915.154419**	0.5709	0.1912
Q80ZE5	Paqr8	Membrane progestin receptor beta	294.0411072	1359.326	916.9907	903.2573	**868.4038773**	1475.48	2290.131		834.7937	**1533.468302**	0.5663	0.1887
Q64378	Fkbp5	Peptidyl-prolyl cis-trans isomerase FKBP5	77.14763641	5103.253	4579.551	6106.374	**3966.581465**	4691.258	6035.77	6870.005	10664.51	**7065.38562**	0.5614	0.1449
P00416	mt-Co3	Cytochrome c oxidase subunit 3	5884.371582	5574.831	12174.21	2752.81	**6596.554749**	22894.92	2395.835	8222.625	13853.16	**11841.63428**	0.5571	0.3160
Q9JK53	Prelp	Prolargin	45786.89063	22361.91	45707.63	46737.08	**40148.37598**	41536.41	59475.52	92642.66	96325.39	**72494.99414**	0.5538	0.0672
P70158	Smpdl3a	Acid sphingomyelinase-like phosphodiesterase 3a		414.8404	501.5415		**458.190918**		628.551	1032.849		**830.6997986**	0.5516	0.2134
Q08288	Lyar	Cell growth-regulating nucleolar protein			1815.682	2322.344	**2069.012878**			4332.511	3175.628	**3754.06958**	0.5511	0.1164
Q8BQM8	Eml5	Echinoderm microtubule-associated protein-like 5	1915.66394			6772.013	**4343.838318**	1792.081	10432.24	15802.68	3569.245	**7899.061768**	0.5499	0.5213
P62878	Rbx1	E3 ubiquitin-protein ligase RBX1		370.0276	85565.15	70816.22	**52250.46494**			100226.9	90249.57	**95238.25**	0.5486	0.2970
Q99M04	Lias	Lipoyl synthase, mitochondrial	3130.0354	2696.745	2997.809	1374.454	**2549.760651**	5515.183	2542.507	5934.467	4643.773	**4658.982727**	0.5473	0.0487
Q9D6L8	Ppil3	Peptidyl-prolyl cis-trans isomerase-like 3				2962.818	**2962.817871**			5426.571		**5426.571289**	0.5460	
Q9D708	S100a16	Protein S100-A16	9788.794922	15381.7	4373.259	4028.485	**8393.059753**	16875.11	16306.51	16396.88	12059.64	**15409.53638**	0.5447	0.0521
Q7TPH6	Mycbp2	E3 ubiquitin-protein ligase MYCBP2		3406.559	2006.302	4895.35	**3436.070272**	6801.367			5930.339	**6365.852783**	0.5398	0.0800
Q80TL1	Adcy2	Adenylate cyclase type 2				1448.69	**1448.69043**			2691.78		**2691.779541**	0.5382	
Q8BGR2	Lrrc8d	Volume-regulated anion channel subunit LRRC8D	4795.965332			16274.13	**10535.04517**		23249.35		16603.46	**19926.4082**	0.5287	0.2924
Q8QZY9	Sf3b4	Splicing factor 3B subunit 4	2765.969971	1910.987	2575.463	2146.88	**2349.825104**	4070.397	5106.3	4713.776	4173.295	**4515.941956**	0.5203	0.0004
Q922K7	Nop2	Probable 28S rRNA (cytosine-C(5))-methyltransferase	861.2623901	780.8189	1123.773	795.1391	**890.2484283**		1162.958	2024.826	1976.051	**1721.27828**	0.5172	0.0216
Q9D3B1	Hacd2	Very-long-chain (3R)-3-hydroxyacyl-CoA dehydratase 2	13683.5957	31174.34	14344.28	10783.28	**17496.37524**	35756.25	28974.13		36906.79	**33879.05534**	0.5164	0.0380
P20918	Plg	Plasminogen		4132.293		5127.344	**4629.818604**	12540.48			5453.905	**8997.192627**	0.5146	0.3466
Q5SUF2	Luc7l3	Luc7-like protein 3	3393.307861	2747.268	18.00061	2578.487	**2184.265717**		3633.434	7922.525	1313.022	**4289.6604**	0.5092	0.3050
Q3TVI8	Pbxip1	Pre-B-cell leukemia transcription factor-interacting protein 1		3140.259	2641.124	1133.775	**2305.05245**	3425.267	5036.656	5307.168		**4589.696777**	0.5022	0.0533
Q9EQX4	Aif1l	Allograft inflammatory factor 1-like	5549.35791		8552.461		**7050.909424**	1868.492		26341.93		**14105.21198**	0.4999	0.6249
Q99PG2	Ogfr	Opioid growth factor receptor	106922.9844	104729.8	95855.97		**102502.9297**	313441.1	97306.91			**205373.9883**	0.4991	0.2920
Q04207	Rela	Transcription factor p65	6315.589844	4128.93	308.2913	294.4803	**2761.822899**	5803.35	4319.8	6026.083	6348.689	**5624.480469**	0.4910	0.1153
O88630	Gosr1	Golgi SNAP receptor complex member 1	14267.5332	5623.861		3895.658	**7929.017415**	14850.88	16666.61	17866.68	15208.63	**16148.19824**	0.4910	0.0327
B1AVD1	Xpnpep2	Xaa-Pro aminopeptidase 2		21104.92			**21104.91992**	43162.41				**43162.41016**	0.4890	
Q9Z2W1	Stk25	Serine/threonine-protein kinase 25		782.4346			**782.4346313**				1609.803	**1609.802612**	0.4860	
Q9QYH6	Maged1	Melanoma-associated antigen D1			1110.318		**1110.318359**	2812.804		854.753	3242.439	**2303.332052**	0.4820	
Q62095	Ddx3y	ATP-dependent RNA helicase DDX3Y	20583.11328	21298.81	12253.58	12292.69	**16607.04932**	80716.91	24111.53	13368.39	21149.45	**34836.57275**	0.4767	0.2886
Q02614	Sap30bp	SAP30-binding protein	3496.784424	962.0231		1817.34	**2092.049093**	7184.976			1639.959	**4412.467346**	0.4741	0.3836
P47811	Mapk14	Mitogen-activated protein kinase 14	5362.588379		3646.556	2210.356	**3739.833333**	6411.188	14260.01		3559.246	**8076.815104**	0.4630	0.2623
Q8BX10	Pgam5	Serine/threonine-protein phosphatase PGAM5, mitochondrial	18815.12891	16781.75		11513.5	**15703.45801**	44881.46	37997.87	29269.46	23789.14	**33984.48389**	0.4621	0.0255
Q9JHS4	Clpx	ATP-dependent Clp protease ATP-binding subunit clpX-like, mitochondrial	1995.497559	3106.636	383.9601	278.2468	**1441.08503**	3267.436	2852.221	2868.439	3561.805	**3137.475403**	0.4593	0.0519
P59708	Sf3b6	Splicing factor 3B subunit 6		4053.828	4334.276		**4194.052002**	17749.31	5811.848	9380	3896.971	**9209.532532**	0.4554	0.3367
P97313	Prkdc	DNA-dependent protein kinase catalytic subunit		1634.759			**1634.758911**			4429.342	2756.704	**3593.022949**	0.4550	
Q91W52	Tmem19	Transmembrane protein 19	5455.816406		6326.045		**5890.930664**			22530.38	3698.374	**13114.37756**	0.4492	0.5236
P07091	S100a4	Protein S100-A4	414.291626	1012.01	5171.866		**2199.38914**	17700.12	555.7188	604.9753	786.3721	**4911.795853**	0.4478	0.6240
P08032	Spta1	Spectrin alpha chain, erythrocytic 1	45331.46484	104218	39307.03	5579.99	**48609.11658**	117787.8	112724.2	107436.6	100560.2	**109627.2051**	0.4434	0.0263
Q3U1F9	Pag1	Phosphoprotein associated with glycosphingolipid-enriched microdomains 1		1024.605	2051.314	772.2032	**1282.707479**	2426.667	2080.713		4446.034	**2984.471029**	0.4298	0.1112
Q8BS95	Gpr89	Golgi pH regulator	5335.254395			5846.069	**5590.661621**				13122.29	**13122.28809**	0.4260	
Q9Z277	Baz1b	Tyrosine-protein kinase BAZ1B	6193.649902		5989.243	4800.295	**5661.062663**	4420.758		28958.16	6528.472	**13302.46468**	0.4256	0.3862
Q9Z2L7	Crlf3	Cytokine receptor-like factor 3			7127.028	3908.5	**5517.764404**	9419.59	17781.15	9377.637	16631.92	**13302.57324**	0.4148	0.0927
P97457	Myl11	Myosin regulatory light chain 11	830.6207886			3428.28	**2129.450531**		6355.812		3945.803	**5150.807739**	0.4134	0.2303
Q8K3X4	Irf2bpl	Probable E3 ubiquitin-protein ligase IRF2BPL				6515.218	**6515.217773**				15760.54	**15760.53809**	0.4134	
Q3U9G9	Lbr	Delta(14)-sterol reductase LBR	4879.6875	18999.12		4400.799	**9426.536621**	10850.56	13559.99	61524.91	5385.049	**22830.12683**	0.4129	0.4379
A6H584	Col6a5	Collagen alpha-5(VI) chain	9207.62793		1625.274		**5416.451172**	12151.35		22435.75	4792.421	**13126.50716**	0.4126	0.3618
Q9ERA6	Tfip11	Tuftelin-interacting protein 11	3472.713379				**3472.713379**		8597.987	8456.088		**8527.037598**	0.4073	
Q6PAM1	Txlna	Alpha-taxilin	1582.509521	295.301			**938.9052582**	1665.232	2335.29	3380.796	1858.524	**2309.960297**	0.4065	0.1208
Q9D7J4	Cox20	Cytochrome c oxidase assembly protein COX20, mitochondrial	7805.810059			9548.495	**8677.152588**	30662.91			12446.2	**21554.55566**	0.4026	0.2946
Q8R173	Zdhhc3	Palmitoyltransferase ZDHHC3	1991.654541		880.0788		**1435.866669**				3724.8	**3724.800049**	0.3855	
O70622	Rtn2	Reticulon-2				4658.929	**4658.929199**			12102.2		**12102.20313**	0.3850	
Q2UY11	Col28a1	Collagen alpha-1(XXVIII) chain		6180.285	1714.317		**3947.301025**	16405.73	5568.245	3550.377	15917.67	**10360.50739**	0.3810	0.2886
Q01279	Egfr	Epidermal growth factor receptor			1999.955		**1999.954956**		4369.564	5952.365	5484.331	**5268.753255**	0.3796	

Q9ERG0	Lima1	LIM domain and actin-binding protein 1		8789.508	5782.968		**7286.238037**	19413.08				**19413.08203**	0.3753	
Q61646	Hp	Haptoglobin			6015.121		**6015.121094**			16272.4		**16272.39941**	0.3697	
Q8CH40	Nudt6	Nucleoside diphosphate-linked moiety X motif 6			6494.241		**6494.241211**			17735.87		**17735.87109**	0.3662	
Q80YT7	Pde4dip	Myomegalin	10031.06152			3201.705	**6616.383301**	17729.25		18635.96		**18182.60352**	0.3639	0.0784
O43790;P97861;Q1453	Krt86;;Krt81	Keratin, type II cuticular Hb6;;Keratin, type II cuticular Hb1	453004.8125	538976.9	19613.32	513295	**381222.5107**	524950.8	607595.9	1719886	1493292	**1086431**	0.3509	0.0750
Q8R015	Bloc1s5	Biogenesis of lysosome-related organelles complex 1 subunit 5				959.2187	**959.218689**			2590.785	2980.414	**2785.599854**	0.3443	
Q6QI06	Rictor	Rapamycin-insensitive companion of mTOR			3053.402		**3053.402344**				9141.832	**9141.832031**	0.3340	
P01878		Ig alpha chain C region	2517.446045	2086.777			**2302.111328**			6905.931		**6905.931152**	0.3334	
A6X935	Itih4	Inter alpha-trypsin inhibitor, heavy chain 4	4809.050293	3339.114	1980.762		**3376.308797**	9228.299			11541.89	**10385.09521**	0.3251	0.0142
Q5DTU0	Afap1l2	Actin filament-associated protein 1-like 2			9628.776		**9628.776367**	17240.31		42520.76		**29880.53516**	0.3222	
Q91VW5	Golga4	Golgin subfamily A member 4		1544.638	2085.188		**1814.913025**		5861.436			**5861.436035**	0.3096	
Q91YL2	Rnf126	E3 ubiquitin-protein ligase RNF126			6995.478		**6995.477539**	2248.226		68511.3	3467.542	**24742.35767**	0.2827	
P50427	Sts	Steryl-sulfatase			14715.24	5794.586	**10254.91113**	65194.42		7528.94		**36361.6792**	0.2820	0.4653
Q8BUJ9	Lrp12	Low-density lipoprotein receptor-related protein 12			20394.34	780.8802	**10587.60907**	30664.3		46664.89		**38664.59668**	0.2738	0.1568
P54729	Nub1	NEDD8 ultimate buster 1		4417.804			**4417.803711**				16359.84	**16359.84277**	0.2700	
Q91XU0	Wrnip1	ATPase WRNIP1		2243.944		3389.083	**2816.513794**	13300.88	7711.125			**10506.00024**	0.2681	0.1145
P54763	Ephb2	Ephrin type-B receptor 2				937.3344	**937.3343506**	3680.334		6270.465	740.5439	**3563.781006**	0.2630	
Q6A098	Secisbp2l	Selenocysteine insertion sequence-binding protein 2-like	13840.33887	7707.644			**10773.99121**	52596.36			32885.43	**42740.89453**	0.2521	0.0903
Q99LL5	Pwp1	Periodic tryptophan protein 1 homolog		966.7356			**966.7355957**	6376.075	1313.276			**3844.67511**	0.2514	
Q9D0Q7	Mrpl45	Large ribosomal subunit protein mL45	1074.330688			1.015087	**537.6728879**		2158.198			**2158.197998**	0.2491	
P52332	Jak1	Tyrosine-protein kinase JAK1	6365.654297	9143.911	8392.435	4158.32	**7015.080078**	12811.72		7625.743	64116.41	**28184.62467**	0.2489	0.2209
Q8K2A1	Gulp1	PTB domain-containing engulfment adapter protein 1			4307.493		**4307.493164**		17554.49			**17554.48828**	0.2454	
Q61510	Trim25	E3 ubiquitin/ISG15 ligase TRIM25				463.9622	**463.9621887**		1832.562		2165.142	**1998.851685**	0.2321	
G5E897	Poglut3	Protein O-glucosyltransferase 3	4419.361328		6009.056	22411.46	**10946.62614**	134988.7	4081.636		6500.12	**48523.48641**	0.2256	0.4376
Q8BG58	P4htm	Transmembrane prolyl 4-hydroxylase	16019.15527	7464.66			**11741.90747**	12627.6	183242.7	13220.33	3839.399	**53232.50623**	0.2206	0.5588
O09005	Degs1	Sphingolipid delta(4)-desaturase DES1				801.6794	**801.6793823**	1416.746	4679.216	8003.779	783.2688	**3720.752502**	0.2155	
Q9ERR7	Selenof	Selenoprotein F	5233.768555		1245.874		**3239.82135**			27449.1	5568.172	**16508.63403**	0.1963	0.3552
Q8C3X8	Lmf2	Lipase maturation factor 2	2348.021973	859.1783			**1603.600159**		15724.4		1593.578	**8658.988586**	0.1852	0.4253
P10107	Anxa1	Annexin A1			11657.37	11.33048	**5834.348833**	16704.16		47675.41		**32189.7832**	0.1812	0.2522
Q8K440	Abca8b	ABC-type organic anion transporter ABCA8B			1015.254	464.7595	**740.0066528**	1253.149	2197.32	2067.781	11019.61	**4134.464325**	0.1790	0.3822
Q8VI36	Pxn	Paxillin			945.7133		**945.7132568**		5349.913			**5349.913086**	0.1768	
Q61334	Bcap29	B-cell receptor-associated protein 29	1179.567261	2290.32		839.3743	**1436.420532**	4183.717	2701.277	1635.553	27200.31	**8930.214142**	0.1608	0.3482
Q6P5H2	Nes	Nestin		20.10015	2140.958	22.43728	**727.831974**			9418.902	30.5662	**4724.73427**	0.1540	0.3497
Q3UQ44	Iqgap2	Ras GTPase-activating-like protein IQGAP2		7728.459	21065.39	11268.64	**13354.16569**	169404.4			13048.82	**91226.62891**	0.1464	0.2752
P62700	Ypel5	Protein yippee-like 5			5751.807		**5751.806641**			40161.62	42585.16	**41373.39063**	0.1390	
Q61702	Itih1	Inter-alpha-trypsin inhibitor heavy chain H1	6613.233887	14395.34	3045.556	15647.14	**9925.317322**	6727.287	13203.18	277641.9	9978.553	**76887.72351**	0.1291	0.3562
Q9QZ47	Tnnt3	Troponin T, fast skeletal muscle				3684.425	**3684.425293**	3179.645	8187.736		90301.72	**33889.69995**	0.1087	
P02301;P84244	H3-5;H3-3a	Histone H3.3C;Histone H3.3		358.7373			**358.7373352**		3427.692			**3427.692383**	0.1047	
Q8K297	Colgalt1	Procollagen galactosyltransferase 1		1723.777	3075.837		**2399.80719**			7408.119	42313.95	**24861.03589**	0.0965	0.3272
Q8R1S4	Mtss1	Protein MTSS 1	193.7007599				**193.7007599**	870.9463		3320.422		**2095.684082**	0.0924	
O89114	Dnajb5	DnaJ homolog subfamily B member 5	2192.104248				**2192.104248**		26666.64			**26666.63672**	0.0822	
P01029	C4b	Complement C4-B			3134.057	1585.206	**2359.631775**	109950.8	3340.123	5211.709	2099.006	**30150.39905**	0.0783	0.5246
P18608	Hmgn1	Non-histone chromosomal protein HMG-14		14.92676			**14.92676163**	14.39357		1127.516		**570.954721**	0.0261	
Q8K0G8	Esrp2	Epithelial splicing regulatory protein 2		5.652346	2.984259		**4.318302155**			1906.732		**1906.732178**	0.0023	
P28184	Mt3	Metallothionein-3			2.491624		**2.491624355**			1183.788		**1183.787842**	0.0021	
Q91VU6	Dcaf11	DDB1- and CUL4-associated factor 11								612.8173		**612.8172607**	0.0000	
Q8C838	Trarg1	Trafficking regulator of GLUT4 1									126.3885	**126.3884888**	0.0000	
Q8BXN9	Tmem87a	Transmembrane protein 87A								1662.611		**1662.610962**	0.0000	
P35441	Thbs1	**Thrombospondin-1**						9175.637				**9175.636719**	0.0000	
Q8K2Y0	Obi1	ORC ubiquitin ligase 1								627.8229		**627.822876**	0.0000	
Q8K013	Gtpbp10	GTP-binding protein 10						835.2827				**835.2827148**	0.0000	
Q8K1L5	Ppp1r11	E3 ubiquitin-protein ligase PPP1R11							18974.63	2943.427	6991.383	**9636.480876**	0.0000	
Q52KI8	Srrm1	Serine/arginine repetitive matrix protein 1						29346.59				**29346.58594**	0.0000	
Q6P9P0	Slf2	SMC5-SMC6 complex localization factor protein 2								831.8702		**831.8702393**	0.0000	
Q6NWW9	Fndc3b	Fibronectin type III domain-containing protein 3B								24002.39		**24002.39453**	0.0000	
P49586	Pcyt1a	Choline-phosphate cytidylyltransferase A						1245.471		1495.345		**1370.407776**	0.0000	
Q9CQU5	Zwint	ZW10 interactor						3842.06				**3842.05957**	0.0000	
O55033	Nck2	Cytoplasmic protein NCK2								2375.434		**2375.433838**	0.0000	
P13864	Dnmt1	DNA (cytosine-5)-methyltransferase 1							14704.54	12271.28	11673.12	**12882.97949**	0.0000	
P19221	F2	Prothrombin								7126.812		**7126.811523**	0.0000	
O88796	Rpp30	Ribonuclease P protein subunit p30						867.5433				**867.543335**	0.0000	
Q99LC2	Cstf1	Cleavage stimulation factor subunit 1						2623.125				**2623.124512**	0.0000	
Q60973	Rbbp7	Histone-binding protein RBBP7						3737.35	10158.07			**6947.710693**	0.0000	
Q61112	Sdf4	45 kDa calcium-binding protein								3255.737		**3255.736816**	0.0000	
Q3ULW8	Parp3	Protein mono-ADP-ribosyltransferase PARP3								3542.368		**3542.368408**	0.0000	

Q6J756	Capn11	Calpain-11				20242.46		20242.45508	0.0000	
Q9EPX2	Papln	Papilin					3341.207	3341.206543	0.0000	
Q3UKC1	Tax1bp1	Tax1-binding protein 1 homolog		16250.87	15340.66	1243.11	13225.63	11515.06958	0.0000	
O09164	Sod3	Extracellular superoxide dismutase [Cu-Zn]		97875.06				97875.0625	0.0000	
Q8BR07	Bicd1	Protein bicaudal D homolog 1					1708.028	1708.028198	0.0000	
Q8BHB4	Wdr3	WD repeat-containing protein 3		1388.375				1388.374878	0.0000	
Q3UPF5	Zc3hav1	Zinc finger CCCH-type antiviral protein 1			1757.417			1757.416626	0.0000	
Q4PZA2	Ece1	Endothelin-converting enzyme 1			12529.93	12051.33	8174.851	10918.70475	0.0000	
P13516	Scd1	Acyl-CoA desaturase 1				3164.082		3164.081787	0.0000	
P58466	Ctdsp1	Carboxy-terminal domain RNA polymerase II polypeptide A small phosphatase 1				3494.42		3494.419922	0.0000	
Q9DC53	Cpne8	Copine-8		2359.655			22048.01	12203.8324	0.0000	
Q9JHJ3	Glmp	Glycosylated lysosomal membrane protein		4171.916	4822.93			4497.422852	0.0000	
Q8BZQ7	Anapc2	Anaphase-promoting complex subunit 2		10963.48		2849.055		6906.269775	0.0000	
Q08274	Dmwd	Dystrophia myotonica WD repeat-containing protein				3977.845		3977.844727	0.0000	
Q8CI70	Lrrc20	Leucine-rich repeat-containing protein 20			7669.988			7669.988281	0.0000	
P61957	Sumo2	Small ubiquitin-related modifier 2				1334.921		1334.920532	0.0000	
Q6GUQ1	Egfl8	Epidermal growth factor-like protein 8			551.0964			551.0963745	0.0000	
Q3UH53	Sdk1	Protein sidekick-1		5388.2				5388.199707	0.0000	
Q5SVQ0	Kat7	Histone acetyltransferase KAT7				1272.147		1272.147217	0.0000	
Q8C7E7	Stbd1	Starch-binding domain-containing protein 1					6821.902	6821.901855	0.0000	
Q62084	Ppp1r14b	Protein phosphatase 1 regulatory subunit 14B				1881.306		1881.30603	0.0000	
Q3U319	Rnf40	E3 ubiquitin-protein ligase BRE1B				8589.389		8589.388672	0.0000	
O70624	Myoc	Myocilin			3311.135	4244.999		3778.067139	0.0000	
P43135	Nr2f2	COUP transcription factor 2		9923.666				9923.666016	0.0000	
Q8BH97	Rcn3	Reticulocalbin-3		122739.6				122739.625	0.0000	
Q99KK1	Reep3	Receptor expression-enhancing protein 3					10248.38	10248.38477	0.0000	
Q9JHI7	Exosc9	Exosome complex component RRP45			1116.695	1367.461		1242.077881	0.0000	
Q61555	Fbn2	Fibrillin-2					2014.113	2014.112671	0.0000	
Q8CFQ3	Aqr	RNA helicase aquarius		151.6727				151.6727448	0.0000	
Q61830	Mrc1	Macrophage mannose receptor 1		6855.71			12270.79	9563.250244	0.0000	
Q80VI1	Trim56	E3 ubiquitin-protein ligase TRIM56			941.6949			941.6949463	0.0000	
Q62440;Q62441	Tle1;Tle4	Transducin-like enhancer protein 1;Transducin-like enhancer protein 4					11112.76	11112.76074	0.0000	
Q9Z2R6	Unc119	Protein unc-119 homolog A		3777.32				3777.320313	0.0000	
Q9ER60	Scn4a	Sodium channel protein type 4 subunit alpha		15251.45				15251.44824	0.0000	
P40936	Inmt	Indolethylamine N-methyltransferase				2684.083		2684.083252	0.0000	
Q91ZP6	Ndfip2	NEDD4 family-interacting protein 2 (Fragment)				4318.861		4318.861328	0.0000	
P62984	Uba52	Ubiquitin-ribosomal protein eL40 fusion protein				351.2314		351.2314148	0.0000	
Q9D975	Srxn1	Sulfiredoxin-1				8450.118		8450.118164	0.0000	
Q9DCB1	Hmgn3	High mobility group nucleosome-binding domain-containing protein 3				3564.458		3564.458496	0.0000	
P16092	Fgfr1	Fibroblast growth factor receptor 1				7372.652	3971.957	5672.304565	0.0000	
Q00422	Gabpa	GA-binding protein alpha chain			1898.745			1898.744995	0.0000	
P35330	Icam2	Intercellular adhesion molecule 2			2369.134			2369.134033	0.0000	
Q9EQS3	Mycbp	c-Myc-binding protein			9892.947	18562.34	11019.52	13158.26986	0.0000	
Q91WG7	Dgkg	Diacylglycerol kinase gamma				3666.698	8095.717	5881.207764	0.0000	
P51655	Gpc4	Glypican-4				36817.83		36817.82813	0.0000	
Q62313	Tgoln1	Trans-Golgi network integral membrane protein 1				5803.428		5803.428223	0.0000	
Q60575	Kif1b	Kinesin-like protein KIF1B		3614.491				3614.491211	0.0000	
Q9D2P4	Urm1	Ubiquitin-related modifier 1				4534.743		4534.742676	0.0000	
P98078	Dab2	Disabled homolog 2		1352.124			1176.325	1264.22467	0.0000	
Q9JJE7	Fads3	Fatty acid desaturase 3				3159.394		3159.394287	0.0000	
Q8CG19	Ltbp1	Latent-transforming growth factor beta-binding protein 1		10046.31	15010.67			12528.48975	0.0000	
A2AJ15	Man1b1	Endoplasmic reticulum mannosyl-oligosaccharide 1,2-alpha-mannosidase					15570.78	15570.7793	0.0000	
E9Q7T7	Chadl	Chondroadherin-like protein					5725.458	5725.458496	0.0000	
Q7TQI7	Abtb2	Ankyrin repeat and BTB/POZ domain-containing protein 2				7242.654	8391.587	7817.120605	0.0000	
Q9WUI1	Mapk11	Mitogen-activated protein kinase 11		4430.233	6611.407	3106.302		4715.980469	0.0000	
Q6WKZ8	Ubr2	E3 ubiquitin-protein ligase UBR2				6668.413		6668.412598	0.0000	
Q923D4	Sf3b5	Splicing factor 3B subunit 5				12547.38	6598.622	9573.003418	0.0000	
Q9R0G7	Zeb2	Zinc finger E-box-binding homeobox 2			5447.163			5447.162598	0.0000	
Q5ND34	Wdr81	WD repeat-containing protein 81					870.5014	870.5014038	0.0000	
P0CW02	Ly6c1	Lymphocyte antigen 6C1		3224.643				3224.643311	0.0000	
Q08481	Pecam1	Platelet endothelial cell adhesion molecule				2509.423	4034.422	3271.922119	0.0000	
Q9Z239	Fxyd1	Phospholemman				40702.98	41838.03	41270.50781	0.0000	
Q923Q2	Stard13	StAR-related lipid transfer protein 13		4831.324				4831.32373	0.0000	
Q9CY52	Thg1l	Probable tRNA(His) guanylyltransferase					7351.312	7351.311523	0.0000	
Q8BHL7	Cdc42se1	CDC42 small effector protein 1					6451.678	6451.678223	0.0000	

Q8BIE6	Frmd4a	FERM domain-containing protein 4A							5266.613		**5266.612793**	**0.0000**	
E9PY46	Ift140	Intraflagellar transport protein 140 homolog						4228.952	4778.222		**4503.58667**	**0.0000**	
Q6DID3	Scaf8	SR-related and CTD-associated factor 8						1886.958		2613.123	**2250.040222**	**0.0000**	
Q8BX90	Fndc3a	Fibronectin type-III domain-containing protein 3A							1176.476		**1176.475708**	**0.0000**	
Q6ZQH8	Nup188	Nucleoporin NUP188					2127.424				**2127.423828**	**0.0000**	
Q8BH15	Cnot10	CCR4-NOT transcription complex subunit 10					49575.96		15292.53		**32434.24658**	**0.0000**	
Q921V5	Mgat2	Alpha-1,6-mannosyl-glycoprotein 2-beta-N-acetylglucosaminyltransferase					3751.292				**3751.292236**	**0.0000**	
P97760	Polr2c	DNA-directed RNA polymerase II subunit RPB3								27799.79	**27799.79297**	**0.0000**	
Q62469	Itga2	Integrin alpha-2					5417.947				**5417.947266**	**0.0000**	
Q8C6M1	Usp20	Ubiquitin carboxyl-terminal hydrolase 20						3301.015			**3301.014893**	**0.0000**	
P70182	Pip5k1a	Phosphatidylinositol 4-phosphate 5-kinase type-1 alpha								2090.661	**2090.661377**	**0.0000**	
Q8BGD6	Slc38a9	Neutral amino acid transporter 9						3295.18			**3295.18042**	**0.0000**	
P97363	Sptlc2	Serine palmitoyltransferase 2					6133.838		5634.422		**5884.130127**	**0.0000**	
Q04888	Sox10	Transcription factor SOX-10						13606.44	388.5879		**6997.515121**	**0.0000**	
Q9JJR8	Tmem9b	Transmembrane protein 9B								1833.011	**1833.01123**	**0.0000**	
Q8BZ94	Zmat4	Zinc finger matrin-type protein 4							1769.905	1148.158	**1459.03186**	**0.0000**	
Q8BL03	Slc25a29	Mitochondrial basic amino acids transporter							4710.599		**4710.598633**	**0.0000**	
Q8R3Q0	Saraf	Store-operated calcium entry-associated regulatory factor							5974.1	2661.862	**4317.980957**	**0.0000**	
Q91XB0	Trex1	Three-prime repair exonuclease 1						2626.52			**2626.519775**	**0.0000**	
O89116	Vti1a	Vesicle transport through interaction with t-SNAREs homolog 1A							16452.74		**16452.74023**	**0.0000**	
Q8CHT3	Ints5	Integrator complex subunit 5						1334.606			**1334.606201**	**0.0000**	
Q9JMC8	Epb41l4b	Band 4.1-like protein 4B						2801.525			**2801.524658**	**0.0000**	
Q6P5C5	Smug1	Single-strand selective monofunctional uracil DNA glycosylase							10675.01		**10675.00879**	**0.0000**	
Q6A051	Atrnl1	Attractin-like protein 1							25100.88	3781.011	**14440.94775**	**0.0000**	
Q61469	Plpp1	Phospholipid phosphatase 1							1206.151		**1206.150757**	**0.0000**	
Q8R3Y8	Irf2bp1	Interferon regulatory factor 2-binding protein 1					4831.485				**4831.484863**	**0.0000**	
P26350	Ptma	Prothymosin alpha							18071.32		**18071.32227**	**0.0000**	
Q9WV70	Noc2l	Nucleolar complex protein 2 homolog						4349.188			**4349.188477**	**0.0000**	
Q04646	Fxyd2	Sodium/potassium-transporting ATPase subunit gamma							5181.938	1416.397	**3299.167664**	**0.0000**	
Q9CQE7	Ergic3	Endoplasmic reticulum-Golgi intermediate compartment protein 3					1690.626				**1690.62561**	**0.0000**	
Q61391	Mme	Neprilysin						1511.731			**1511.730591**	**0.0000**	
Q9D7V9	Naaa	N-acylethanolamine-hydrolyzing acid amidase							2149.97	887.0781	**1518.523926**	**0.0000**	
Q9CWG9	Bloc1s2	Biogenesis of lysosome-related organelles complex 1 subunit 2							25246.84		**25246.83984**	**0.0000**	
Q8BVL9	Jakmip1	Janus kinase and microtubule-interacting protein 1							5512.646		**5512.645508**	**0.0000**	
P28828	Ptprm	Receptor-type tyrosine-protein phosphatase mu					8651.726	11634.24			**10142.98438**	**0.0000**	
Q921Q3	Alg1	Chitobiosyldiphosphodolichol beta-mannosyltransferase					886.1377		1914.364	29137.04	**10645.84871**	**0.0000**	
Q61245	Col11a1	Collagen alpha-1(XI) chain							1789.39		**1789.390137**	**0.0000**	
Q921U8	Smtn	Smoothelin								166860.7	**166860.7188**	**0.0000**	
Q9D4H2	Gcc1	GRIP and coiled-coil domain-containing protein 1					5996.251				**5996.250977**	**0.0000**	
Q9DCT6	Bap18	Chromatin complexes subunit BAP18					5339.617		6064.651		**5702.134033**	**0.0000**	
Q3T9E4;Q62293	Tgtp2;Tgtp1	T-cell-specific guanine nucleotide triphosphate-binding protein 2;T-cell-specific guanine nucleotide triphosphate-binding protein 1							472879.6		**472879.625**	**0.0000**	
Q8BYM8	Cars2	Probable cysteine--tRNA ligase, mitochondrial							4794.709	3366.81	**4080.759521**	**0.0000**	
Q9CVD2	Atxn3	Ataxin-3							3253.197	1658.5	**2455.848633**	**0.0000**	
Q9Z1S3	Rasgrp1	RAS guanyl-releasing protein 1							59997.54		**59997.54297**	**0.0000**	
Q9DAT5	Trmu	Mitochondrial tRNA-specific 2-thiouridylase 1							4166.568		**4166.567871**	**0.0000**	
Q8R4K2	Irak4	Interleukin-1 receptor-associated kinase 4							1667.08		**1667.079834**	**0.0000**	
Q3U0D9	Hace1	E3 ubiquitin-protein ligase HACE1							1		**1**	**0.0000**	
Q6PAN7	Prr18	Proline-rich protein 18							1427.132		**1427.132202**	**0.0000**	
Q9R1W5	Calcrl	Calcitonin gene-related peptide type 1 receptor							5375.763	2855.022	**4115.392456**	**0.0000**	
D3YVF0	Akap5	A-kinase anchor protein 5							8129.744	1976.867	**5053.305664**	**0.0000**	
Q9QXW9	Slc7a8	Large neutral amino acids transporter small subunit 2						4779.385		6354.319	**5566.852295**	**0.0000**	
Q8BL06	Usp54	Inactive ubiquitin carboxyl-terminal hydrolase 54							4567.943	10703.01	**7635.475098**	**0.0000**	
Q9ES07	Slc15a2	Solute carrier family 15 member 2							10095.08		**10095.07715**	**0.0000**	
P28798	Grn	Progranulin											
Q8VEB4	Pla2g15	Phospholipase A2 group XV			3478.869	**3478.868896**							
Q9D8C4	Ifi35	Interferon-induced 35 kDa protein homolog											
Q3URQ7	Mthfsd	Methenyltetrahydrofolate synthase domain-containing protein	5112.920898			**5112.920898**							
Q80WV3	Chst2	Carbohydrate sulfotransferase 2											
Q9JKS4	Ldb3	LIM domain-binding protein 3		6074.258		**6074.257813**							
Q91WJ7	Spats2l	SPATS2-like protein		17111.45		**17111.44727**							
P29788	Vtn	Vitronectin			8239.105	**8239.105469**							
Q05117	Acp5	Tartrate-resistant acid phosphatase type 5											
P20801	Tnnc2	Troponin C, skeletal muscle											
P31266	Rbpj	Recombining binding protein suppressor of hairless											
E9Q1P8	Irf2bp2	Interferon regulatory factor 2-binding protein 2											

Q8VC65	Nrm	Nurim					
P59900	Emilin3	EMILIN-3					
Q62147	Sspn	Sarcospan					
Q9Z1T2	Thbs4	**Thrombospondin-4**					
Q99JP6	Homer3	Homer protein homolog 3					
Q3TBT3	Sting1	Stimulator of interferon genes protein					
A2BH40	Arid1a	AT-rich interactive domain-containing protein 1A					
P27005	S100a8	Protein S100-A8			23710.66		**23710.66016**
Q9JK37	Myoz1	Myozenin-1					
Q8K3H5	Myo3a	Myosin-IIIa					
O88693	Ugcg	Ceramide glucosyltransferase					
Q64261	Cdk6	Cyclin-dependent kinase 6					
P13412	Tnni2	Troponin I, fast skeletal muscle					
Q9JKN1	Slc30a7	Zinc transporter 7					
Q9JMF7	Dolpp1	Dolichyldiphosphatase 1					
P97314	Csrp2	Cysteine and glycine-rich protein 2					
P42925	Pxmp2	Peroxisomal membrane protein 2				844.3657	**844.3656616**
Q8C0X8		Sperm motility kinase X					
Q8BNE1	Tcaf1	TRPM8 channel-associated factor 1					
Q8BUH8	Senp7	Sentrin-specific protease 7					
P31725	S100a9	Protein S100-A9			63063.36	4965.355	**34014.35913**
Q64512	Ptpn13	Tyrosine-protein phosphatase non-receptor type 13					
Q62234	Myom1	Myomesin-1					
Q60590	Orm1	Alpha-1-acid glycoprotein 1				3492.354	**3492.35376**
Q8BFY0	Pirt	Phosphoinositide-interacting protein					
Q9CXG3	Ppil4	Peptidyl-prolyl cis-trans isomerase-like 4					
Q61247	Serpinf2	Alpha-2-antiplasmin					
Q3UA37	Qrich1	Transcriptional regulator QRICH1					
O88878	Zfand5	AN1-type zinc finger protein 5					
Q80TE4	Sipa1l2	Signal-induced proliferation-associated 1-like protein 2					
Q9WUH1	Tmem115	Transmembrane protein 115					
Q07113	Igf2r	Cation-independent mannose-6-phosphate receptor					
Q8C3P7	Mettl3	N6-adenosine-methyltransferase subunit METTL3					
Q80TY4	St18	Suppression of tumorigenicity 18 protein					
Q9D0B0	Srsf9	Serine/arginine-rich splicing factor 9					
Q80XM9	Slc66a2	Solute carrier family 66 member 2					
Q3UP87	Elane	Neutrophil elastase	16016.42285		9288.446		**12652.43457**
P41245	Mmp9	Matrix metalloproteinase-9			5249.767		**5249.766602**
P11672	Lcn2	Neutrophil gelatinase-associated lipocalin			25123.07		**25123.07031**
Q8BHD4	Frmd3	FERM domain-containing protein 3					
O70255	Mpzl2	Myelin protein zero-like protein 2					
Q9D1H9	Mfap4	Microfibril-associated glycoprotein 4					
Q7TT15	Galnt17	Polypeptide N-acetylgalactosaminyltransferase 17	2179.82666				**2179.82666**
Q91XL3	Uxs1	UDP-glucuronic acid decarboxylase 1					
Q08093	Cnn2	Calponin-2				3316.022	**3316.021973**
Q9QXD8	Limd1	LIM domain-containing protein 1			2139.014		**2139.014404**
Q99LC9	Pex6	Peroxisomal ATPase PEX6					
Q6P1H6	Ankle2	Ankyrin repeat and LEM domain-containing protein 2					
O08692	Ngp	Neutrophilic granule protein	48862.26563		47192.56	5947.464	**34000.76286**
Q921Y4	Mfsd5	Molybdate-anion transporter					
P04117	Fabp4	Fatty acid-binding protein, adipocyte					
P59326	Ythdf1	YTH domain-containing family protein 1					
Q9ERY9	Erg28	Ergosterol biosynthetic protein 28 homolog		10704.04		10461.2	**10582.61963**
Q80Z19	Muc2	Mucin-2					
Q8K2V1	Ppp4r1	Serine/threonine-protein phosphatase 4 regulatory subunit 1					
Q8K1B8	Fermt3	Fermitin family homolog 3		9653.771	8089.861		**8871.815674**
P11247	Mpo	Myeloperoxidase			24927.93		**24927.92773**
P51912	Slc1a5	Neutral amino acid transporter B(0)					
Q80XL6	Acad11	Acyl-CoA dehydrogenase family member 11			43341.97		**43341.96875**
Q925J9	Med1	Mediator of RNA polymerase II transcription subunit 1					
Q8R242	Ctbs	Di-N-acetylchitobiase					
Q6NSU3	Glt8d1	Glycosyltransferase 8 domain-containing protein 1					
P61372	Isl1	Insulin gene enhancer protein ISL-1					
Q8BQS4	Eeig2	EEIG family member 2					
P04247	Mb	Myoglobin			5842.556	1341.626	**3592.091064**

Q6QIY3	Scn10a	Sodium channel protein type 10 subunit alpha					
Q7TSG2	Ctdp1	RNA polymerase II subunit A C-terminal domain phosphatase					
O35309	Nmi	N-myc-interactor					
Q6A028	Swap70	Switch-associated protein 70					
Q8K1C9	Lrrc41	Leucine-rich repeat-containing protein 41					
P54320	Eln	Elastin					
O54965	Rnf13	E3 ubiquitin-protein ligase RNF13					
Q6DTY7	Pfkfb4	6-phosphofructo-2-kinase/fructose-2,6-bisphosphatase 4					
Q8CIZ8	Vwf	von Willebrand factor					
O89020	Afm	Afamin					
Q5S006	Lrrk2	Leucine-rich repeat serine/threonine-protein kinase 2	2345.784424				**2345.784424**
Q61508	Ecm1	Extracellular matrix protein 1					
Q9ESB3	Hrg	Histidine-rich glycoprotein					
A6H6A4	Lrriq4	Leucine-rich repeat and IQ domain-containing protein 4			3992.511		**3992.511475**
Q99JP0	Map4k3	Mitogen-activated protein kinase kinase kinase kinase 3					
Q61129	Cfi	Complement factor I					
O09012	Pex5	Peroxisomal targeting signal 1 receptor					
Q9R1Q7	Plp2	Proteolipid protein 2					
Q99JR8	Smarcd2	SWI/SNF-related matrix-associated actin-dependent regulator of chromatin subfamily D member 2					
O35744	Chil3	Chitinase-like protein 3			92795.85	2259.466	**47527.65869**
Q99LQ7	PEDS1	Plasmanylethanolamine desaturase 1					
Q9CZU4	Eral1	GTPase Era, mitochondrial					
P28481	Col2a1	Collagen alpha-1(II) chain					
Q99J95	Cdk9	Cyclin-dependent kinase 9					
Q60994	Adipoq	Adiponectin					
P39876	Timp3	Metalloproteinase inhibitor 3				2781.371	**2781.37085**
Q05144	Rac2	Ras-related C3 botulinum toxin substrate 2			12434.79	2136.859	**7285.823608**
Q61878	Prg2	Bone marrow proteoglycan			38309.31	2964.604	**20636.9563**
Q8CEC0	Nup88	Nuclear pore complex protein Nup88		6988.935			**6988.935059**
Q8VC03	Eml3	Echinoderm microtubule-associated protein-like 3		2309.309	3090.194		**2699.751587**
P49290	Epx	Eosinophil peroxidase			16725.72		**16725.72266**
Q78KK3	Slc22a18	Solute carrier family 22 member 18					
P51437	Camp	Cathelicidin antimicrobial peptide			14953.52		**14953.51563**
Q91YV9	Pgap4	Post-GPI attachment to proteins factor 4					
Q8C1D8	Iws1	Protein IWS1 homolog					
Q9DBX2	Pdcl	Phosducin-like protein		6389.107	7643.021	7157.435	**7063.187826**
Q9Z2F7	Bnip3l	BCL2/adenovirus E1B 19 kDa protein-interacting protein 3-like		4618.682			**4618.681641**
Q80U38	Khnyn	Protein KHNYN			32344.64		**32344.64063**
Q8CFX1	H6pd	GDH/6PGL endoplasmic bifunctional protein					
Q8CD91	Smoc2	SPARC-related modular calcium-binding protein 2					
Q8R0A0	Gtf2f2	General transcription factor IIF subunit 2					
Q9QZD4	Ercc4	DNA repair endonuclease XPF					
Q6Q899	Rigi	Antiviral innate immune response receptor RIG-I	4625.486816				**4625.486816**
Q6VVW5	Npr2	Atrial natriuretic peptide receptor 2		4072.925			**4072.924561**
P15306	Thbd	Thrombomodulin					
Q6P2K6	Ppp4r3a	Serine/threonine-protein phosphatase 4 regulatory subunit 3A					
Q925T6	Grip1	Glutamate receptor-interacting protein 1			14272.28	21154.21	**17713.24463**
O35516	Notch2	Neurogenic locus notch homolog protein 2					
P31649	Slc6a13	Sodium- and chloride-dependent GABA transporter 2					
O35074	Ptgis	Prostacyclin synthase					
O54782	Man2b2	Epididymis-specific alpha-mannosidase					
Q9Z126	Pf4	Platelet factor 4		2195.399			**2195.398682**
Q91WD0	Gpr108	Protein GPR108					
P49300	Clec10a	C-type lectin domain family 10 member A					
Q5SW46	Lpo	Lactoperoxidase					
Q9CYA6	Zcchc8	Zinc finger CCHC domain-containing protein 8					
Q9DCE5	Pak1ip1	p21-activated protein kinase-interacting protein 1					
P18155	Mthfd2	Bifunctional methylenetetrahydrofolate dehydrogenase/cyclohydrolase, mitochondrial				1293.672	**1293.671509**
P97290	Serping1	Plasma protease C1 inhibitor					
Q99PN3	Trim26	Tripartite motif-containing protein 26					
Q8BYU6	Tor1aip2	Torsin-1A-interacting protein 2					
O54749;O54750	Cyp2j5;Cyp2j6	Cytochrome P450 2J5;Cytochrome P450 2J6	12014.01953				**12014.01953**
P49138	Mapkapk2	MAP kinase-activated protein kinase 2					
P52875	Tmem165	Transmembrane protein 165					
P49935	Ctsh	Pro-cathepsin H					

P04186	Cfb	Complement factor B				35811.9	**35811.90234**
Q9D0E5	Efcab11	EF-hand calcium-binding domain-containing protein 11					
Q3UWW6	Gas2l3	GAS2-like protein 3					
P97401	Frzb	Secreted frizzled-related protein 3					
Q91VS8	Farp2	FERM, ARHGEF and pleckstrin domain-containing protein 2		3008.201			**3008.201416**
P28843	Dpp4	Dipeptidyl peptidase 4				66348.23	**66348.23438**
Q99JV5	Stard4	StAR-related lipid transfer protein 4					
O70423	Aoc3	Membrane primary amine oxidase			5785.188		**5785.1875**
Q8K449	Abca9	ATP-binding cassette sub-family A member 9					
C0HKG5;C0HKG6	Rnaset2a;Rnaset2b	Ribonuclease T2-A;Ribonuclease T2-B					
Q8K2C8	Gpat4	Glycerol-3-phosphate acyltransferase 4		12171.6			**12171.59863**
Q9WVC3	Cav2	Caveolin-2		409570.1	12955.23	5197.428	**142574.2415**
Q8C0D4	Arhgap12	Rho GTPase-activating protein 12			998.7192		**998.7192383**
Q9D6J1	Cers4	Ceramide synthase 4		3384.639			**3384.638916**
Q8CDJ8	Ston1	Stonin-1					
P97366	Evi5	Ecotropic viral integration site 5 protein					
Q91X91	Qprt	Nicotinate-nucleotide pyrophosphorylase [carboxylating]					
Q8BW10	Nob1	RNA-binding protein NOB1				2713.92	**2713.92041**
O88968	Tcn2	Transcobalamin-2					
Q03145	Epha2	Ephrin type-A receptor 2					
Q9ES46	Parvb	Beta-parvin					
Q9CQL4	Mrpl20	Large ribosomal subunit protein bL20m					
P21843	Mcpt3	Mast cell protease 3 (Fragment)					
O08800	Serpinb8	Serpin B8					
Q8BPB5	Efemp1	EGF-containing fibulin-like extracellular matrix protein 1				7376.881	**7376.881348**
Q60778	Nfkbib	NF-kappa-B inhibitor beta					
Q8BG30	Nelfa	Negative elongation factor A					
Q3UY34	Custos	Protein CUSTOS					
Q80TP3	Ubr5	E3 ubiquitin-protein ligase UBR5					
P06537	Nr3c1	Glucocorticoid receptor					
B2RXR6	Ankrd44	Serine/threonine-protein phosphatase 6 regulatory ankyrin repeat subunit B					
O70475	Ugdh	UDP-glucose 6-dehydrogenase					
Q9CWU2	Zdhhc13	Palmitoyltransferase ZDHHC13					
O08715	Akap1	A-kinase anchor protein 1, mitochondrial					
P11881	Itpr1	Inositol 1,4,5-trisphosphate receptor type 1					
Q99J31	Ophn1	Oligophrenin-1			29150.87	6842.329	**17996.60107**
Q99PM9	Uck2	Uridine-cytidine kinase 2					
Q9EQW7	Kif13a	Kinesin-like protein KIF13A					
P49222	Epb42	Protein 4.2				833.0895	**833.0894775**
D0QMC3;P0DOV2	Mndal;Ifi204	Myeloid cell nuclear differentiation antigen-like protein;Interferon-activable protein 204					
Q8BND5	Qsox1	Sulfhydryl oxidase 1					
Q99PP7	Trim33	E3 ubiquitin-protein ligase TRIM33					
Q9DBD0	Ica	Inhibitor of carbonic anhydrase					
P21956	Mfge8	Lactadherin	18554.69336				**18554.69336**
P58196	Plscr4	Phospholipid scramblase 4					
Q9ER69	Wtap	Pre-mRNA-splicing regulator WTAP					
Q9EPB4	Pycard	Apoptosis-associated speck-like protein containing a CARD					
Q80TT8	Cul9	Cullin-9					
P01796;P01797;P01799;P01801		Ig heavy chain V-III region A4;Ig heavy chain V-III region U61;Ig heavy chain V-III region ABE-47N;Ig heavy chain V-III region J606					
A2AQ07	Tubb1	Tubulin beta-1 chain	31278.93164	27355.65	40536.04	51730.11	**37725.18262**
Q8BGT7	Smndc1	Survival of motor neuron-related-splicing factor 30			2722.254	2880.543	**2801.398315**
Q60596	Xrcc1	DNA repair protein XRCC1	11015.26563				**11015.26563**
P18528		Ig heavy chain V region 6.96					
Q9CR25	Dph2	2-(3-amino-3-carboxypropyl)histidine synthase subunit 2					
Q80XC3	Usp6nl	USP6 N-terminal-like protein					
Q6PB93	Galnt2	Polypeptide N-acetylgalactosaminyltransferase 2					
Q8K4P0	Wdr33	pre-mRNA 3' end processing protein WDR33					
Q8R0W6	Ndfip1	NEDD4 family-interacting protein 1					
D3YU32	Tex13c1	Testis-expressed protein 13C-1					
Q8R409	Hexim1	Protein HEXIM1					
Q9D8T4	Tvp23b	Golgi apparatus membrane protein TVP23 homolog B				3049.63	**3049.629639**
Q3TEW6	Mpzl1	Myelin protein zero-like protein 1					
Q8R2T8	Gtf3c5	General transcription factor 3C polypeptide 5					
O35153	Bet1l	BET1-like protein					
P22935	Crabp2	Cellular retinoic acid-binding protein 2					

Q62009	Postn	Periostin				4940.304	**4940.304199**
P37889	Fbln2	Fibulin-2			8717.13		**8717.129883**
P0C7L0	Wipf3	WAS/WASL-interacting protein family member 3					
Q99MQ4	Aspn	Asporin			1630.946		**1630.945557**
P01644;P01645		Ig kappa chain V-V region HP R16.7;Ig kappa chain V-V region HP 93G7					
P28293	Ctsg	Cathepsin G			6649.782		**6649.781738**
Q8BG18	Necab1	N-terminal EF-hand calcium-binding protein 1					
Q9D2U5	Naa38	N-alpha-acetyltransferase 38, NatC auxiliary subunit					
Q8VE28	Nkd2	Protein naked cuticle homolog 2					
Q8K2I3	Fmo2	Dimethylaniline monooxygenase [N-oxide-forming] 2					
Q9CXI3	Moxd1	DBH-like monooxygenase protein 1					
P27641	Xrcc5	X-ray repair cross-complementing protein 5					
Q80WW9	Ddrgk1	DDRGK domain-containing protein 1					
Q9CQJ2	Pih1d1	PIH1 domain-containing protein 1	961.8953857			2340.953	**1651.424377**
Q64435	Ugt1a6	UDP-glucuronosyltransferase 1-6		4369.027			**4369.027344**
O89090	Sp1	Transcription factor Sp1					
Q00724	Rbp4	Retinol-binding protein 4					
Q9D8T2	Gsdmd	Gasdermin-D					
Q62074	Prkci	Protein kinase C iota type				4296.303	**4296.302734**
Q9DBF7	Cwc25	Pre-mRNA-splicing factor CWC25 homolog					
Q9CZR2	Naalad2	N-acetylated-alpha-linked acidic dipeptidase 2			987.5921	7187.536	**4087.564117**
Q62356	Fstl1	Follistatin-related protein 1					
Q9EPS3	Glce	D-glucuronyl C5-epimerase		2152.81			**2152.810303**
Q9R053	Scn11a	Sodium channel protein type 11 subunit alpha					
Q6NXN1	Szrd1	SUZ domain-containing protein 1					
Q3TUA9	Pomk	Protein O-mannose kinase			1819.515		**1819.514648**
Q6NXJ0	Wwc2	Protein WWC2					
Q9DBB4	Naa16	N-alpha-acetyltransferase 16, NatA auxiliary subunit					
P36371	Tap2	Antigen peptide transporter 2				5428.095	**5428.094727**
Q8C6U2	Slc66a3	Solute carrier family 66 member 3					
Q07646	Mest	Mesoderm-specific transcript protein					
P06800	Ptprc	Receptor-type tyrosine-protein phosphatase C					
Q8BW00	Ptrh1	Probable peptidyl-tRNA hydrolase					
Q8VCB1	Ndc1	Nucleoporin NDC1	1148.877686	2190.783	1465.51		**1601.723592**
Q8BX09	Rbbp5	Retinoblastoma-binding protein 5				1757.396	**1757.395996**
Q8BQ30	Ppp1r18	Phostensin					
Q9DBU0	Tm9sf1	Transmembrane 9 superfamily member 1					
Q3U3W5	Prmt9	Protein arginine N-methyltransferase 9		4456.499			**4456.499023**
Q1EG27	Myo3b	Myosin-IIIb					
Q91WT4	Dnajc17	DnaJ homolog subfamily C member 17					
Q8BG07	Pld4	5'-3' exonuclease PLD4					
Q9CTN4	Rhobtb3	Rho-related BTB domain-containing protein 3			5370.499		**5370.499023**
P52293	Kpna2	Importin subunit alpha-1					
Q9QY40	Plxnb3	Plexin-B3	4435.547363				**4435.547363**
Q640N1	Aebp1	Adipocyte enhancer-binding protein 1			24235.08		**24235.08398**
Q3UR32	P2rx3	P2X purinoceptor 3					
O35730;Q9CQJ4	Ring1;Rnf2	E3 ubiquitin-protein ligase RING1;E3 ubiquitin-protein ligase RING2					
P21958	Tap1	Antigen peptide transporter 1					
Q7TQ48	Srl	Sarcalumenin					
Q9D6N1	Ca13	Carbonic anhydrase 13					
Q8K2J0	Plcd3	1-phosphatidylinositol 4,5-bisphosphate phosphodiesterase delta-3					
P53808	Pctp	Phosphatidylcholine transfer protein					
Q68FE6	Ripor1	Rho family-interacting cell polarization regulator 1					
Q9EPM5	Sync	Syncoilin					
P58022	Loxl2	Lysyl oxidase homolog 2					
Q9CSH3	Dis3	Exosome complex exonuclease RRP44					
P28076	Psmb9	Proteasome subunit beta type-9					
Q6NVG7	Colgalt2	Procollagen galactosyltransferase 2					
Q9CQT5	Pomp	Proteasome maturation protein		11116.17			**11116.17188**
E9PYK3	Parp4	Protein mono-ADP-ribosyltransferase PARP4		1450.728		1.73494	**726.2312393**
Q8BQM4	Heatr3	HEAT repeat-containing protein 3					
P10854;Q64478;Q6452	H2bc14;H2bc9;Hist2h2bb;H	Histone H2B type 1-M;Histone H2B type 1-H;Histone H2B type 2-B;Histone H2B type 1-C/E/G					
Q91X88	Pomgnt1	Protein O-linked-mannose beta-1,2-N-acetylglucosaminyltransferase 1					
Q8VCT4	Ces1d	Carboxylesterase 1D					
Q8CIB6	Tmem230	Transmembrane protein 230					

Q8R422	Cd109	CD109 antigen					
Q5RKZ7	Mocs1	Molybdenum cofactor biosynthesis protein 1			20182.54		**20182.54297**
P47759	Nsg2	Neuronal vesicle trafficking-associated protein 2					
O08811	Ercc2	General transcription and DNA repair factor IIH helicase subunit XPD					
Q80XL7	Mppe1	Metallophosphoesterase 1					
Q6PIX5	Rhbdf1	Inactive rhomboid protein 1					
Q9D945	Llph	Protein LLP homolog					
Q8K4L3	Svil	Supervillin					
P01654		Ig kappa chain V-III region PC 2880/PC 1229					
P97801	Smn1	Survival motor neuron protein					
F6W8I0	Yjefn3	YjeF N-terminal domain-containing protein 3	5313.132324				**5313.132324**
Q9Z2B9	Rps6ka4	Ribosomal protein S6 kinase alpha-4					
Q99K41	Emilin1	EMILIN-1					
P97792	Cxadr	Coxsackievirus and adenovirus receptor homolog					
Q8K377	Lrrtm1	Leucine-rich repeat transmembrane neuronal protein 1					
D3KU66	Asmt	Acetylserotonin O-methyltransferase					
Q02780	Nfia	Nuclear factor 1 A-type					
Q9DD23	Lypd2	Ly6/PLAUR domain-containing protein 2					
Q9DB76	Emc9	ER membrane protein complex subunit 9					
Q91XC8	Dap	Death-associated protein 1					
O70433	Fhl2	Four and a half LIM domains protein 2					
Q2HXL6	Edem3	ER degradation-enhancing alpha-mannosidase-like protein 3					
Q64345	Ifit3	Interferon-induced protein with tetratricopeptide repeats 3	4130.275391		6267.188	1.112554	**3466.191978**
Q9CW46	Raver1	Ribonucleoprotein PTB-binding 1					
Q8BVD5	Mpp7	MAGUK p55 subfamily member 7					
Q61142	Spin1	Spindlin-1					
P10417	Bcl2	Apoptosis regulator Bcl-2					
P04945		Ig kappa chain V-VI region NQ2-6.1	2158.599121				**2158.599121**
P70665	Siae	Sialate O-acetylesterase					
P26262	Klkb1	Plasma kallikrein				65724.57	**65724.57031**
P06683	C9	Complement component C9					
Q8C170	Myo9a	Unconventional myosin-IXa				17594.34	**17594.3418**
Q8R2Q8	Bst2	Bone marrow stromal antigen 2					
Q91X78	Erlin1	Erlin-1					
Q9D0Y8	Mrpl52	Large ribosomal subunit protein mL52		653.9507			**653.9506836**
Q69ZN7	Myof	Myoferlin					
Q9QZK2	Bcar3	Breast cancer anti-estrogen resistance protein 3 homolog				1545.537	**1545.537476**
Q9DBG1	Cyp27a1	Sterol 26-hydroxylase, mitochondrial					
Q9JLC4	Sorcs1	VPS10 domain-containing receptor SorCS1					
P63271;Q9Z199	Supt4h1a;Supt4h1b	Transcription elongation factor SPT4-A;Transcription elongation factor SPT4-B					
Q8BRH0	Tmtc3	Protein O-mannosyl-transferase TMTC3					
Q3V0C5	Usp48	Ubiquitin carboxyl-terminal hydrolase 48					
P97386	Lig3	DNA ligase 3					
Q8BQ47	Cnpy4	Protein canopy homolog 4	31219.24219				**31219.24219**
Q8BGT0	Ostm1	Osteopetrosis-associated transmembrane protein 1					
P0DOV1;Q8CGE8	Mnda;Ifi205a	Interferon-activable protein 205-B;Interferon-activable protein 205-A					
Q9QXT5	Egfl7	Epidermal growth factor-like protein 7					
A6H8H5;Q03717	Kcnb2;Kcnb1	Potassium voltage-gated channel subfamily B member 2;Potassium voltage-gated channel subfamily B member 1					
Q9Z247	Fkbp9	Peptidyl-prolyl cis-trans isomerase FKBP9			55539.71		**55539.70703**
Q9CWU9	Nup37	Nucleoporin Nup37					
P01843		Ig lambda-1 chain C region					
P22227;Q00899;Q3TTC2	Zfp42;Yy1;Yy2	Zinc finger protein 42;Transcriptional repressor protein YY1;Transcription factor YY2					
Q9Z0F8	Adam17	Disintegrin and metalloproteinase domain-containing protein 17					
Q8BMZ5	Tsen34	tRNA-splicing endonuclease subunit Sen34				1360.316	**1360.315796**
P70444	Bid	BH3-interacting domain death agonist				17732.41	**17732.40625**
Q9JJR9	Nrip3	Nuclear receptor-interacting protein 3					
Q921I9	Exosc4	Exosome complex component RRP41					
Q9D9I4	Tbc1d20	TBC1 domain family member 20			7246.188		**7246.188477**
Q03347	Runx1	Runt-related transcription factor 1					
Q99PP9	Trim16	Tripartite motif-containing protein 16					
Q8CD26	Slc35e1	Solute carrier family 35 member E1					
Q9JJY4	Ddx20	Probable ATP-dependent RNA helicase DDX20					
P06330		Ig heavy chain V region AC38 205.12			21048.56		**21048.56055**
Q9CQG2	Mettl16	RNA N6-adenosine-methyltransferase METTL16					
Q9QY06	Myo9b	Unconventional myosin-IXb					

Q9WVL1	Ap4s1	AP-4 complex subunit sigma-1					
Q9JLV2	Trpc4ap	Short transient receptor potential channel 4-associated protein					
Q3U962	Col5a2	Collagen alpha-2(V) chain			1746.974		**1746.973511**
Q8VC98	Plekha4	Pleckstrin homology domain-containing family A member 4					
Q60953	Pml	Protein PML					
P28654	Dcn	Decorin		83525.15	45808.39	4708.347	**44680.62988**
Q8K114	Ints9	Integrator complex subunit 9					
Q2QI47	Ush2A	Usherin					
Q6PAV2	Herc4	Probable E3 ubiquitin-protein ligase HERC4		17259.44			**17259.44336**
Q8BK62	Olfml3	Olfactomedin-like protein 3					
P14483	H2-Ab1	H-2 class II histocompatibility antigen, A beta chain					
Q9CTG6	Atp13a2	Polyamine-transporting ATPase 13A2					
Q9EPK6	Sil1	Nucleotide exchange factor SIL1					
Q9JJH1	Rnase4	Ribonuclease 4					
O55226	Chad	Chondroadherin			7377.124		**7377.124023**
Q8BJ64	Chdh	Choline dehydrogenase, mitochondrial			4491.015		**4491.014648**
P08074	Cbr2	Carbonyl reductase [NADPH] 2			4210.799		**4210.799316**
Q9JI48	Plac8	Placenta-specific gene 8 protein			10011.29		**10011.28516**
Q9R061	Nubp2	Cytosolic Fe-S cluster assembly factor NUBP2					
P97287	Mcl1	Induced myeloid leukemia cell differentiation protein Mcl-1 homolog					
Q9JJA2	Cog8	Conserved oligomeric Golgi complex subunit 8			36972.6		**36972.60156**
P01901	H2-K1	H-2 class I histocompatibility antigen, K-B alpha chain					
Q9DBU3	Riok3	Serine/threonine-protein kinase RIO3					
P49182	Serpind1	Heparin cofactor 2					
P58058	Nadk	NAD kinase					
P21844	Cma1	Chymase					
Q8BH65	Dennd6a	Protein DENND6A		1239.914			**1239.914185**
P70403	Cux1	Protein CASP				1074.213	**1074.213135**
Q9CY97	Ssu72	RNA polymerase II subunit A C-terminal domain phosphatase SSU72	3818.291748				**3818.291748**
P86046	Kcnj13	Inward rectifier potassium channel 13					
Q99JY3	Gimap4	GTPase IMAP family member 4					
Q9CXJ1	Ears2	Probable glutamate--tRNA ligase, mitochondrial					
Q9ER04	Tmprss5	Transmembrane protease serine 5					
P47880	Igfbp6	Insulin-like growth factor-binding protein 6					
Q78HU7	Gypc	Glycophorin-C					
P01898	H2-Q10	H-2 class I histocompatibility antigen, Q10 alpha chain					
Q4VBE8	Wdr18	WD repeat-containing protein 18					
P06684	C5	Complement C5			227.2726		**227.272583**
P97402	Gcnt2	N-acetyllactosaminide beta-1,6-N-acetylglucosaminyl-transferase					
Q9D853	Eef1akmt2	EEF1A lysine methyltransferase 2					
P03953	Cfd	Complement factor D					
Q9BCZ4	Selenos	Selenoprotein S					
P28301	Lox	Protein-lysine 6-oxidase					
P19788	Mgp	Matrix Gla protein			6671.891		**6671.890625**
Q99KG3	Rbm10	RNA-binding protein 10					
Q8JZM0	Tfb1m	Dimethyladenosine transferase 1, mitochondrial					
Q9EQK7	Icmt	Protein-S-isoprenylcysteine O-methyltransferase					
Q06770	Serpina6	Corticosteroid-binding globulin					
Q9R0M4	Podxl	Podocalyxin					
Q9WVL2	Stat2	Signal transducer and activator of transcription 2				2842.877	**2842.876709**
Q8VDD9	Phip	PH-interacting protein					
Q99P91	Gpnmb	Transmembrane glycoprotein NMB					
Q61488	Dhh	Desert hedgehog protein					
Q8VEA4	Chchd4	Mitochondrial intermembrane space import and assembly protein 40					
Q61576	Fkbp10	Peptidyl-prolyl cis-trans isomerase FKBP10					
Q6P5E6	Gga2	ADP-ribosylation factor-binding protein GGA2					
P31428	Dpep1	Dipeptidase 1					
O70404	Vamp8	Vesicle-associated membrane protein 8					
Q8C4J7	Tbl3	Transducin beta-like protein 3					
Q7TSH8	Tmem94	Transmembrane protein 94					
Q08857	Cd36	Platelet glycoprotein 4					
Q9WVH9	Fbln5	Fibulin-5			6136.167		**6136.16748**
Q6S5L9	Shc4	SHC-transforming protein 4					
Q921G6	Lrch4	Leucine-rich repeat and calponin homology domain-containing protein 4		3794.263			**3794.262939**
P12242	Ucp1	Mitochondrial brown fat uncoupling protein 1					

P03987		Ig gamma-3 chain C region					
Q9CQG6	Tmem147	BOS complex subunit TMEM147					
Q8K4G1	Ltbp4	Latent-transforming growth factor beta-binding protein 4				26615.72	**26615.72461**
Q9JLQ2	Git2	ARF GTPase-activating protein GIT2					
Q91YI4	Arrb2	Beta-arrestin-2					
Q9WVJ5	Crybb1	Beta-crystallin B1					
Q8BTI7	Ankrd52	Serine/threonine-protein phosphatase 6 regulatory ankyrin repeat subunit C					
Q8BTW3	Exosc6	Exosome complex component MTR3					
Q6GU68	Islr	Immunoglobulin superfamily containing leucine-rich repeat protein			3282.279		**3282.278809**
Q8CGN5	Plin1	Perilipin-1					
P97426	Ear1	Eosinophil cationic protein 1					
Q8BH35	C8b	Complement component C8 beta chain					
Q61466	Smarcd1	SWI/SNF-related matrix-associated actin-dependent regulator of chromatin subfamily D member 1					
Q9CZH3	Psmg3	Proteasome assembly chaperone 3					
Q6ZPF4	Fmnl3	Formin-like protein 3					
P46656	Fdx1	Adrenodoxin, mitochondrial		9395.261			**9395.260742**
Q08024	Cbfb	Core-binding factor subunit beta			2305.335		**2305.334717**
Q6P1J0	Maneal	Glycoprotein endo-alpha-1,2-mannosidase-like protein					
Q7TSZ8	Nacc1	Nucleus accumbens-associated protein 1				4205.779	**4205.779297**
Q7TSF4	Lrrc75a	Leucine-rich repeat-containing protein 75A					
Q3U284	Tmem231	Transmembrane protein 231					
Q61559	Fcgrt	IgG receptor FcRn large subunit p51					
Q9CQS2	Nop10	H/ACA ribonucleoprotein complex subunit 3	1339.35083		2123.448		**1731.39917**
O70422	Gtf2h4	General transcription factor IIH subunit 4					
Q3KNJ2	Nhej1	Non-homologous end-joining factor 1					
Q8R1Z9	Rnf121	E3 ubiquitin ligase Rnf121					
P08905	Lyz2	Lysozyme C-2			14675.02	4.065659	**7339.540642**
Q61398	Pcolce	Procollagen C-endopeptidase enhancer 1					
Q505D9	Trim67	Tripartite motif-containing protein 67		736.4031			**736.4030762**
P50481	Lhx3	LIM/homeobox protein Lhx3					
Q8BPG6	Sumf2	Inactive C-alpha-formylglycine-generating enzyme 2					
P21845	Tpsb2	Tryptase beta-2					
Q9CQ45	Nenf	Neudesin					
Q91YR9	Ptgr1	Prostaglandin reductase 1					
P19973	Lsp1	Lymphocyte-specific protein 1					
Q80TN4	Dnajc16	DnaJ homolog subfamily C member 16					
Q3UDF0	Slc2a6	Solute carrier family 2, facilitated glucose transporter member 6					
Q9D8T0	Fam3a	Protein FAM3A					
Q9WUE4	Nprl2	GATOR1 complex protein NPRL2					
Q9D081	Alg14	UDP-N-acetylglucosamine transferase subunit ALG14 homolog					
P18531	Ighv3-6	Ig heavy chain V region 3-6					
Q3UV71	Tmtc1	Protein O-mannosyl-transferase TMTC1					
P70166	Cpeb1	Cytoplasmic polyadenylation element-binding protein 1					
A2AI05	Ndor1	NADPH-dependent diflavin oxidoreductase 1					
P03958	Ada	Adenosine deaminase					
P01633	Igkv6-17	Immunoglobulin kappa chain variable 6-17					
A2A761	Zfp69	Zinc finger protein 69					
O35638	Stag2	Cohesin subunit SA-2		813.7476			**813.7475586**
Q3TV70	Nr2c2ap	Nuclear receptor 2C2-associated protein					
Q91WD2	Trpv6	Transient receptor potential cation channel subfamily V member 6					
Q91VJ2	Cavin3	Caveolae-associated protein 3			13307.88		**13307.87695**
Q9CYA0	Creld2	Protein disulfide isomerase Creld2					
Q7TQ62	Podn	Podocan					
O89032	Sh3pxd2a	SH3 and PX domain-containing protein 2A		41753.02			**41753.02344**
Q9CQJ0	Them5	Acyl-coenzyme A thioesterase THEM5			6696.604		**6696.603516**
Q61329	Zfhx3	Zinc finger homeobox protein 3					
Q7TMR0	Prcp	Lysosomal Pro-X carboxypeptidase	6231.970215		6280.397		**6256.183838**
Q5FW52	Mlip	Muscular LMNA-interacting protein			3869.656		**3869.65625**
P01753		Ig heavy chain V region 186-1					
Q8BRH3	Arhgap19	Rho GTPase-activating protein 19					
Q6AW69	Cgnl1	Cingulin-like protein 1					
Q8VEL9	Rem2	GTP-binding protein REM 2					
O35486	Crygs	Gamma-crystallin S					
Q99J93	Ifitm2	Interferon-induced transmembrane protein 2					
Q60961	Laptm4a	Lysosomal-associated transmembrane protein 4A	2589.651123				**2589.651123**

Q61526	Erbb3	Receptor tyrosine-protein kinase erbB-3					
Q8R3G9	Tspan8	Tetraspanin-8			700.1925		**700.1925049**
P82343	Renbp	N-acylglucosamine 2-epimerase		4382.808			**4382.807617**
Q921C5	Bicd2	Protein bicaudal D homolog 2	3342.162598		7299.505		**5320.833984**
Q9WU42	Ncor2	Nuclear receptor corepressor 2				18275.16	**18275.16406**
Q91V81	Rbm42	RNA-binding protein 42				1.916646	**1.916646361**
Q3UV16	Itpripl2	Inositol 1,4,5-trisphosphate receptor-interacting protein-like 2					
Q9QXM0	Abhd2	Monoacylglycerol lipase ABHD2					
Q6TCG2	Paqr9	Membrane progesterone receptor epsilon					
P55002	Mfap2	Microfibrillar-associated protein 2				4.013364	**4.013364315**
Q9CRA0	Art4	Ecto-ADP-ribosyltransferase 4					
Q70FJ1	Akap9	A-kinase anchor protein 9					
Q62177	Sema3b	Semaphorin-3B					
Q3V0G7	Garnl3	GTPase-activating Rap/Ran-GAP domain-like protein 3					
Q920A7	Afg3l1	AFG3-like protein 1				1.328128	**1.328128338**
P52795	Efnb1	Ephrin-B1			8428.147	4476.352	**6452.249512**
Q9DBZ1	Ikbip	Inhibitor of nuclear factor kappa-B kinase-interacting protein					
Q00780	Col8a1	Collagen alpha-1(VIII) chain					
Q4PNJ2	Nkain2	Sodium/potassium-transporting ATPase subunit beta-1-interacting protein 2					
Q78IS1	Tmed3	Transmembrane emp24 domain-containing protein 3					
Q8R100	Calhm5	Calcium homeostasis modulator protein 5					
Q6DFX2	Antxr2	Anthrax toxin receptor 2					
O08734	Bak1	Bcl-2 homologous antagonist/killer					
Q9D7B7	Gpx8	Probable glutathione peroxidase 8					
Q3V3R4	Itga1	Integrin alpha-1					
P14434;P14438	H2-Aa	H-2 class II histocompatibility antigen, A-B alpha chain;H-2 class II histocompatibility antigen, A-U alpha chain (Fragment)					
Q80U58	Pum2	Pumilio homolog 2					
P13597	Icam1	Intercellular adhesion molecule 1					
E9Q394	Akap13	A-kinase anchor protein 13					
Q9JHA8	Vwa7	von Willebrand factor A domain-containing protein 7					
P97473	Tarbp2	RISC-loading complex subunit TARBP2					
P15089	Cpa3	Mast cell carboxypeptidase A					
Q9WVJ9	Efemp2	EGF-containing fibulin-like extracellular matrix protein 2					
Q9JKJ9	Cyp39a1	24-hydroxycholesterol 7-alpha-hydroxylase					
Q91YE7	Rbm5	RNA-binding protein 5					
Q8BTP0	Pigz	GPI mannosyltransferase 4					
Q78T81	Eeig1	Early estrogen-induced gene 1 protein			5188.91		**5188.910156**
Q61809	Lrrn1	Leucine-rich repeat neuronal protein 1					
Q9R0B6	Lamc3	Laminin subunit gamma-3			3533.288		**3533.28833**
Q9DBV4	Mxra8	Matrix remodeling-associated protein 8					
Q9QXP7	C1qtnf1	Complement C1q tumor necrosis factor-related protein 1					
Q8BVG5	Galnt14	Polypeptide N-acetylgalactosaminyltransferase 14					
Q8R3H7	Hs2st1	Heparan sulfate 2-O-sulfotransferase 1					
Q9D6Y4	Borcs8	BLOC-1-related complex subunit 8					
O35607	Bmpr2	Bone morphogenetic protein receptor type-2					
Q61164	Ctcf	Transcriptional repressor CTCF					
P97858	Slc35b1	Solute carrier family 35 member B1					
P62915	Gtf2b	Transcription initiation factor IIB					
Q3UGP8	Alg10b	Putative Dol-P-Glc:Glc(2)Man(9)GlcNAc(2)-PP-Dol alpha-1,2-glucosyltransferase		8021.459			**8021.458984**
Q9Z1Y4	Trip6	Thyroid receptor-interacting protein 6					
Q3UH93	Plxnd1	Plexin-D1					
Q9CQ43	Dut	Deoxyuridine 5'-triphosphate nucleotidohydrolase					
Q91ZN5	Slc35b2	Adenosine 3'-phospho 5'-phosphosulfate transporter 1					
Q8CI11	Gnl3	Guanine nucleotide-binding protein-like 3					
Q8BKT7	Thoc5	THO complex subunit 5 homolog					
Q8VHK1	Caskin2	Caskin-2	2049.400391	1180.918			**1615.159119**
Q6ZWQ0	Syne2	Nesprin-2					
Q91X84	Crtc3	CREB-regulated transcription coactivator 3			3939.252		**3939.252441**
Q9D270	Zdhhc21	Palmitoyltransferase ZDHHC21					
Q8BG16	Slc6a15	Sodium-dependent neutral amino acid transporter B(0)AT2					
Q9DBJ3	Baiap2l1	Brain-specific angiogenesis inhibitor 1-associated protein 2-like protein 1					
Q64449	Mrc2	C-type mannose receptor 2					
Q8BHD0	Rab39a	Ras-related protein Rab-39A					
P14901	Hmox1	Heme oxygenase 1		3385.058			**3385.05835**
Q8R088	Golph3l	Golgi phosphoprotein 3-like					

O88792	F11r	Junctional adhesion molecule A					
Q8R4Y4	Stab1	Stabilin-1					
Q9WVL6	Extl3	Exostosin-like 3					
Q9Z2X8	Keap1	Kelch-like ECH-associated protein 1					
Q8K2C7	Os9	Protein OS-9					
Q8R2G4	Art3	Ecto-ADP-ribosyltransferase 3	77408.14844		5847.514		**41627.8313**
Q8K1N4	Spats2	Spermatogenesis-associated serine-rich protein 2					
Q80W68	Kirrel1	Kin of IRRE-like protein 1					
Q8VCG4	C8g	Complement component C8 gamma chain					
P52633	Stat6	Signal transducer and transcription activator 6					
E9PXT9	Fam170b	Protein FAM170B					
Q9QZZ6	Dpt	Dermatopontin					
Q8BJ05	Zc3h14	Zinc finger CCCH domain-containing protein 14					
Q9DCA5	Brix1	Ribosome biogenesis protein BRX1 homolog					
Q0P678	Zc3h18	Zinc finger CCCH domain-containing protein 18					
Q80ZK9	Wdtc1	WD and tetratricopeptide repeats protein 1					
Q5FWI3	Cemip2	Cell surface hyaluronidase					
A6X8Z5	Arhgap31	Rho GTPase-activating protein 31					
P25318	Col8a2	Collagen alpha-2(VIII) chain					
O54998	Fkbp7	Peptidyl-prolyl cis-trans isomerase FKBP7					
Q9D2C6	Polr3h	DNA-directed RNA polymerase III subunit RPC8					
Q8K1E6	Alkbh3	Alpha-ketoglutarate-dependent dioxygenase alkB homolog 3			3014.757		**3014.75708**
Q9ERU3	Znf22	Zinc finger protein 22			5.311982		**5.311981678**
Q9DCK3	Tspan4	Tetraspanin-4					
Q9R207	Nbn	Nibrin					
Q8BL99	Dop1a	Protein dopey-1					
Q8C2S7	Amigo3	Amphoterin-induced protein 3					
Q8CGB3	Uaca	Uveal autoantigen with coiled-coil domains and ankyrin repeats					
Q9WTU0	Phf2	Lysine-specific demethylase PHF2					
P01642	Gm10881	Ig kappa chain V-V region L7 (Fragment)					
A4Q9F0	Ttll7	Tubulin polyglutamylase TTLL7					
Q80UN1	Kctd9	BTB/POZ domain-containing protein KCTD9			23310.27	18361.21	**20835.74023**
P11679	Krt8	Keratin, type II cytoskeletal 8			40552.29	30558.35	**35555.32031**
Q9R0E2	Plod1	Procollagen-lysine,2-oxoglutarate 5-dioxygenase 1					
Q8BHK3	Slc36a2	Proton-coupled amino acid transporter 2					
P01638		Ig kappa chain V-V region L6 (Fragment)					
Q76N33	Stambpl1	AMSH-like protease				1.286313	**1.286313415**
G3X982	Aox3	Aldehyde oxidase 3			68377.14		**68377.14063**
O35099	Map3k5	Mitogen-activated protein kinase kinase kinase 5	9624.342773			8051.823	**8838.083008**
Q8BH74	Nup107	Nuclear pore complex protein Nup107	3970.784912				**3970.784912**
Q5HZJ5	Entrep3	Protein ENTREP3					
P16879	Fes	Tyrosine-protein kinase Fes/Fps					
Q9Z2F2	Oasl2	2'-5'-oligoadenylate synthase-like protein 2					
Q8BXA1	Golim4	Golgi integral membrane protein 4					
Q8BHC9	Fut11	Alpha-(1,3)-fucosyltransferase 11					
Q9CYD3	Crtap	Cartilage-associated protein		5762.769			**5762.768555**
P97479	Myo7a	Unconventional myosin-VIIa					
Q8BJL1	Fbxo30	F-box only protein 30					
Q8K296	Mtmr3	Myotubularin-related protein 3					
Q9D2X5	Mau2	MAU2 chromatid cohesion factor homolog					
Q91W98	Slc15a4	Solute carrier family 15 member 4					
Q9EP71	Rai14	Ankycorbin					
Q5SZV5	Kiaa0319	Dyslexia-associated protein KIAA0319 homolog					
Q8BUV8	Gpr107	Protein GPR107					
O88551	Cldn1	Claudin-1					
O70579	Slc25a17	Peroxisomal membrane protein PMP34					
P0DW87	Zftraf1	Zinc finger TRAF-type-containing protein 1					
Q8BGV0	Nars2	Probable asparagine--tRNA ligase, mitochondrial					
Q9WU66	Sfrp5	Secreted frizzled-related protein 5					
B2RRE7	Otud4	OTU domain-containing protein 4					
Q8BG19	Tmtc4	Protein O-mannosyl-transferase TMTC4					
Q80V94	Ap4e1	AP-4 complex subunit epsilon-1		979.8239	3299.434	3583.071	**2620.776428**
Q921X9	Pdia5	Protein disulfide-isomerase A5					
Q99JH8	Kdelr1	ER lumen protein-retaining receptor 1				4181.153	**4181.152832**
Q9ERE9	Mapk8ip2	C-Jun-amino-terminal kinase-interacting protein 2				3826.185	**3826.185303**

Q8BXL7	Arfrp1	ADP-ribosylation factor-related protein 1				1731.318	**1731.318481**
Q3UQ28	Pxdn	Peroxidasin homolog					
Q922B9	Itprid2	Protein ITPRID2	12812.9668				**12812.9668**
Q99KW3	Triobp	TRIO and F-actin-binding protein		16533.83			**16533.83008**
Q8BMF8	Gldn	Gliomedin					
P30412	Ppic	Peptidyl-prolyl cis-trans isomerase C				4.595021	**4.595020771**
P97872	Fmo5	Flavin-containing monooxygenase 5					
Q3UHI0	Ccser2	Serine-rich coiled-coil domain-containing protein 2					
P01664;P01665;P01666;P01668		Ig kappa chain V-III region CBPC 101;Ig kappa chain V-III region PC 7043;Ig kappa chain V-III region PC 7183;Ig kappa chain V-III region PC 7210					
P60605	Ube2g2	Ubiquitin-conjugating enzyme E2 G2	2880.777832				**2880.777832**
Q5ND29	Rilp	Rab-interacting lysosomal protein					
P01635	Igkv12-41	Immunoglobulin kappa chain variable 12-41 (Fragment)					
Q9Z0J1	Reck	Reversion-inducing cysteine-rich protein with Kazal motifs					
Q4QQM5	Miga1	Mitoguardin 1					
Q8K4D3	Slc36a1	Proton-coupled amino acid transporter 1					
Q61107	Gbp3	Guanylate-binding protein 3	4391.999512				**4391.999512**
Q64282	Ifit1	Interferon-induced protein with tetratricopeptide repeats 1					
Q9ET38	Cldn19	Claudin-19					
Q3V209	Tmub2	Transmembrane and ubiquitin-like domain-containing protein 2					
Q9WV68	Decr2	Peroxisomal 2,4-dienoyl-CoA reductase [(3E)-enoyl-CoA-producing]					
P52840	Sult1a1	Sulfotransferase 1A1			8584.076		**8584.076172**
Q91XD2	Lims2	LIM and senescent cell antigen-like-containing domain protein 2					
Q61103	Dpf2	Zinc finger protein ubi-d4					
Q8CIF6	Sidt2	SID1 transmembrane family member 2					
Q5D525	Syce1l	Synaptonemal complex central element protein 1-like					
A0A0G2JDV3	Gbp6	Guanylate-binding protein 6					
Q9Z211	Pex11a	Peroxisomal membrane protein 11A					
P15655	Fgf2	Fibroblast growth factor 2					
Q9JM51	Ptges	Prostaglandin E synthase					
Q921R8	Slc41a3	Solute carrier family 41 member 3					
P42867	Dpagt1	UDP-N-acetylglucosamine--dolichyl-phosphate N-acetylglucosaminephosphotransferase					
Q9CXY1	Tmem175	Endosomal/lysosomal proton channel TMEM175					
Q3UFY8	Trmt10c	tRNA methyltransferase 10 homolog C					
O88329	Myo1a	Unconventional myosin-Ia	2.119855165				**2.119855165**
P04441	Cd74	H-2 class II histocompatibility antigen gamma chain					
P01630		Ig kappa chain V-II region 7S34.1					
Q9D9K3	Aven	Cell death regulator Aven					
P01674		Ig kappa chain V-III region PC 2154					
O88593	Pglyrp1	Peptidoglycan recognition protein 1					
Q3UU94	Mansc4	MANSC domain-containing protein 4					
P01844	Iglc2	Ig lambda-2 chain C region					
P18525		Ig heavy chain V region 5-84					
Q3U1C6	Tatdn3	Putative deoxyribonuclease TATDN3					
P01670;P01671;P01672		Ig kappa chain V-III region PC 6684;Ig kappa chain V-III region PC 7175;Ig kappa chain V-III region PC 7940					
P97425	Ear2	Eosinophil cationic protein 2					
Q9DBR0	Akap8	A-kinase anchor protein 8					
P01639	Igkv9-120	Immunoglobulin kappa chain variable 9-120					
Q8CFD4	Snx8	Sorting nexin-8					
Q62273	Slc26a2	Sulfate transporter					
Q8BPB0;Q921Y0	Mob1b;Mob1a	MOB kinase activator 1B;MOB kinase activator 1A					
P70662	Ldb1	LIM domain-binding protein 1					
Q9DC33	Hmg20a	High mobility group protein 20A					
O35654	Pold2	DNA polymerase delta subunit 2					
P04223	H2-K1	H-2 class I histocompatibility antigen, K-K alpha chain			13356.13		**13356.13086**
P29452	Casp1	Caspase-1					
Q2VPA6	Helq	Helicase POLQ-like					
O35701	Matn3	Matrilin-3					
Q5PSV9	Mdc1	Mediator of DNA damage checkpoint protein 1					
O08912	Galnt1	Polypeptide N-acetylgalactosaminyltransferase 1					
Q9QWV9	Ccnt1	Cyclin-T1					
P09542	Myl3	Myosin light chain 3					
Q91YJ3	Thyn1	Thymocyte nuclear protein 1					
Q91VB4	Hps3	BLOC-2 complex member HPS3					
P01728		Ig lambda-2 chain V region					
A2AHL1	Ano3	Anoctamin-3		1795.086			**1795.086304**

Q61781	Krt14	Keratin, type I cytoskeletal 14			5664.236		**5664.23584**
Q7TMD7	Dsg4	Desmoglein-4					
P01629		Ig kappa chain V-II region 2S1.3					
P19426	Nelfe	Negative elongation factor E					
Q7M6Z0	Rtn4rl2	Reticulon-4 receptor-like 2					
Q9Z1P7	Kank3	KN motif and ankyrin repeat domain-containing protein 3					
Q3TXX3	Zfyve27	Protrudin					
Q64387	Pnoc	Prepronociceptin	3474.286133			3636.99	**3555.637939**
P21661	Pcsk2	Neuroendocrine convertase 2	4532.221191		4017.189	5650.514	**4733.308268**
Q8VCE1	Dnajc28	DnaJ homolog subfamily C member 28	4585.711426			4141.731	**4363.721436**
Q2TA57	Asphd1	Aspartate beta-hydroxylase domain-containing protein 1					
Q0GUM3	Gm12250	Interferon-gamma-inducible GTPase 10					
P29351	Ptpn6	Tyrosine-protein phosphatase non-receptor type 6					
Q62230	Siglec1	Sialoadhesin					
P35991	Btk	Tyrosine-protein kinase BTK		1220.085			**1220.085327**
Q9Z0T9	Itgb6	Integrin beta-6					
Q3U2J5	Camkmt	Calmodulin-lysine N-methyltransferase		475.7078			**475.7077942**
Q8C5W0	Clmn	Calmin		3407.855			**3407.855469**
G3XA57	Rab11fip2	Rab11 family-interacting protein 2				8158.672	**8158.671875**
